# DNA methylation is associated with lung function in never smokers

**DOI:** 10.1186/s12931-019-1222-8

**Published:** 2019-12-02

**Authors:** Maaike de Vries, Ivana Nedeljkovic, Diana A. van der Plaat, Alexandra Zhernakova, Lies Lahousse, Guy G. Brusselle, BIOS Consortium, Najaf Amin, Cornelia M. van Duijn, Judith M. Vonk, H. Marike Boezen

**Affiliations:** 1 University of Groningen, University Medical Center Groningen, Department of Epidemiology, Hanzeplein 1, 9713 GZ Groningen, The Netherlands; 2 University of Groningen, University Medical Center Groningen, Groningen Research Institute for Asthma and COPD (GRIAC), Groningen, The Netherlands; 3 Department of Epidemiology, Erasmus Medical Center, Rotterdam, The Netherlands; 4 University of Groningen, University Medical Center Groningen, Department of Genetics, Groningen, The Netherlands; 5 Department of Bioanalysis, FFW, Ghent University, Ghent, Belgium; 6 Department of Respiratory Medicine, Ghent University Hospital, Ghent, Belgium; 7 Department of Respiratory Medicine, Erasmus Medical Center, Rotterdam, The Netherlands; 8 BIOS Consortium, https://www.bbmri.nl/acquisition-use-analyze/bios

**Keywords:** DNA methylation, Never smokers, FEV_1_/FVC, EWAS, COPD

## Abstract

**Background:**

Active smoking is the main risk factor for COPD. Here, epigenetic mechanisms may play a role, since cigarette smoking is associated with differential DNA methylation in whole blood. So far, it is unclear whether epigenetics also play a role in subjects with COPD who never smoked. Therefore, we aimed to identify differential DNA methylation associated with lung function in never smokers.

**Methods:**

We determined epigenome-wide DNA methylation levels of 396,243 CpG-sites (Illumina 450 K) in blood of never smokers in four independent cohorts, LifeLines COPD&C (*N* = 903), LifeLines DEEP (*N* = 166), Rotterdam Study (RS)-III (*N* = 150) and RS-BIOS (*N* = 206). We meta-analyzed the cohort-specific methylation results to identify differentially methylated CpG-sites with FEV_1_/FVC. Expression Quantitative Trait Methylation (eQTM) analysis was performed in the Biobank-based Integrative Omics Studies (BIOS).

**Results:**

A total of 36 CpG-sites were associated with FEV_1_/FVC in never smokers at *p*-value< 0.0001, but the meta-analysis did not reveal any epigenome-wide significant CpG-sites. Of interest, 35 of these 36 CpG-sites have not been associated with lung function before in studies including subjects irrespective of smoking history. Among the top hits were cg10012512, cg02885771, annotated to the gene LTV1 Ribosome Biogenesis factor *(LTV1),* and cg25105536, annotated to Kelch Like Family Member 32 (*KLHL32*). Moreover, a total of 11 eQTMS were identified.

**Conclusions:**

With the identification of 35 CpG-sites that are unique for never smokers, our study shows that DNA methylation is also associated with FEV_1_/FVC in subjects that never smoked and therefore not merely related to smoking.

## Background

Chronic Obstructive Pulmonary Disease (COPD) is a progressive inflammatory lung disease characterized by persistent airway obstruction that causes severe respiratory symptoms and poor quality of life [1]. Although smoking is generally considered the main environmental risk factor, estimations are that 25–45% of patients with COPD have never smoked [2]. Despite extensive research, the etiology of COPD remains incompletely understood. It is known that the development of this complex heterogeneous disease is influenced by both genetic and environmental factors, as well as their interactions [3–6]. As interface between the inherited genome and environmental exposures, an important role has been postulated for the epigenome [7]. The epigenome includes multiple epigenetic mechanisms that affect gene expression without modifying the DNA sequence. These epigenetic mechanisms are highly dynamic and respond to environmental exposures, ageing and diseases [8]. One such epigenetic mechanism is DNA methylation, which involves the binding of a methyl group to a cytosine base located adjacent to a guanine base. Methylation of these so called CpG-sites in regulatory regions of the DNA generally result in decreased expression of a particular gene [9].

So far, only a few studies have investigated the association between DNA methylation in peripheral blood and COPD or lung function using an epigenome-wide hypothesis free approach [10–17]. Although findings across the studies are not consistent, there is suggestive evidence that alterations in DNA methylation might play a role in the etiology of COPD. However, in previous studies, subjects were mainly included irrespective of smoking status, thus including current smokers, ex-smokers and never smokers. As a consequence, it is currently not known if there are differences in DNA methylation between healthy individuals and patients with COPD who have never smoked. Recently, we studied the association between epigenome-wide DNA methylation and COPD in both current smokers and never smokers [16]. Although we did not find any epigenome-wide significant association in current smokers nor in never smokers, the associations between DNA methylation and COPD were different between both groups. Hence, by further exploring the role of DNA methylation in a much larger set of never smokers together with a continuous measurement of lung function, we might be able to reveal important novel insights in the etiology of COPD. In this study, we aim to assess the association between DNA methylation and lung function in never smokers, meta-analyzing four independent population-based cohorts.

## Methods

### Study population

To study the association between epigenome-wide DNA methylation and lung function, defined as the ratio between the Forced Expiratory Volume in 1 s (FEV_1_) and Forced Vital Capacity (FVC), in never smokers, we performed a meta-analysis in four different cohorts. Two cohorts originated from the LifeLines population-based cohort study [18]: the LifeLines COPD & Controls DNA methylation study [16, 19] (LL COPD&C, *n* = 903) and the LifeLines DEEP study [20] (LLDEEP, *n* = 166). The two other cohorts originated from the population-based Rotterdam study (RS) [21]: The first visit of the third RS cohort (RS-III-1, *n* = 150) and a cohort selected for the Biobank-based Integrative Omics Studies (BIOS) project (RS-BIOS, *n* = 206). Both population-based cohort studies were approved by the local university medical hospital ethical committees and all participants signed written informed consent. In all cohorts, never smoking was defined based on self-reported never smoking history and 0 pack years included in the standardized questionnaires.

### Measurements

#### Lung function

Within the LifeLines population-based cohort study, pre-bronchodilator spirometry was performed with a Welch Allyn Version 1.6.0.489, PC-based Spiroperfect with CA Workstation software according to ATS/ERS guidelines. Technical quality and results were evaluated by well-trained assistants and difficult to interpret results were re-evaluated by a lung physician. Within the population-based Rotterdam study, pre-bronchodilator spirometry was performed during the research center visit using a SpiroPro portable spirometer (RS-III-1) or a Master Screen® PFT Pro (RS-BIOS) by trained paramedical staff according to the ERS/ATS Guidelines. Spirometry results were analyzed by two researchers and verified by a specialist in pulmonary medicine.

#### DNA methylation

In all four cohorts, DNA methylation levels in whole blood were determined with the Illumina Infinium Methylation 450 K array. Data was presented as beta values (ratio of methylated probe intensity and the overall intensity) ranging from 0 to 1. Quality control has been performed for all datasets separately as described before [19, 22]. After quality control, data was available on 396,243 CpG-sites in all four datasets.

### Statistical analysis

#### Epigenome-wide association study and meta-analysis

We performed an epigenome-wide association study (EWAS) on lung function defined as FEV_1_/FVC in all four cohorts separately using robust linear regression analysis in R. The analysis was adjusted for the potential confounders age and sex. To adjust for the cellular heterogeneity of the whole blood samples, we included proportional white blood cell counts of mononuclear cells, lymphocytes, neutrophils and eosinophils, obtained by standard laboratory techniques. For LL COPD&C, we adjusted for technical variation by performing a principal components analysis using the 220 control probes incorporated in the Illumina 450 k Chip. The 7 principal components that explained > 1% of the technical variation were included in the analysis. For LLDEEP, data on technical variance was not accessible. For the two RS cohorts, we included the position on the array and array number to adjust for technical variation. Regression estimates from all four individual EWA studies were combined by a weighted by the inverse of the variance random-effect meta-analysis using the effect estimates and standard errors in “rmeta” package in R. CpG-sites with a *p*-value below 1.26 × 10^^− 7^ (Bonferroni corrected *p*-value by number of CpG-sites 0.05/396243) were considered epigenome-wide significant. CpG-sites with a *p*-value below 0.0001 in the meta-analysis were defined as top associations in our study.

#### Expression quantitative trait methylation (eQTM) analysis

To assess whether top associations were also associated with gene expression levels, we used the never smokers included in the Biobank-based Integrative Omics Studies (BIOS). For all cohorts separately, reads were normalized to counts per million. To adjust for technical variation for gene expression and DNA methylation, principal component analysis was conducted on the residual normalized counts and beta-values excluding the potential confounders age and gender. Principal components that explained more than 5% of the technical variation in gene expression or DNA methylation were included in the analysis. Subsequently, robust linear regression analysis was performed on the CpG-sites and the genes within 1 MB around the CpG-sites. The analyses were adjusted for the potential confounders age, sex and technical variation by principal components as stated before. The individuals eQTM analysis were combined by a random-effect meta-analysis using the effect estimates and standard errors in RMeta. An eQTM was considered significant when the Bonferroni-adjusted *p*-value for the number of genes within 1 MB around the CpG-sites was below 0.05.

## Results

### Subject characteristics

An overview of the characteristics of the subjects included in the study is shown in Table 1. LL COPD&C was the largest cohort included in this meta-analysis. Notably, since this cohort is a non-random selection from the LifeLines cohort study with COPD (defined as FEV_1_/FVC < 0.70) as one of the selection criteria, the percentages of COPD cases should not be interpreted as prevalence.

**Table 1:** Subject characteristics of the subjects from the four different DNA methylation datasets

	LL COPD&C	LLDEEP	RS-III-1	RS-BIOS
Number of subjects, N (%)	903	166	150	206
Male, N (%)	508 (56.3)	71 (42.8)	74 (49.3)	80 (38.8)
Age (yrs), median (min-max)	46 (18–80)	42 (20–78)	63 (53–93)	68 (52–79)
Airway obstruction (FEV_1_/FVC< 70%), N (%)	316 (35.0)	15 (9.0)	13 (8.7)	19 (9.0)
- FEV_1_ (L), mean (SE)	3.5 (0.9)	3.6 (0.9)	3.2 (0.8)	2.7 (0.7)
- FEV_1_/FVC, mean (SE)	84.5 (8.2)	78.6 (6.2)	77.8 (5.9)	77.9 (5.9)

### Meta-analysis of the four epigenome-wide association studies

**Fig. 1:**
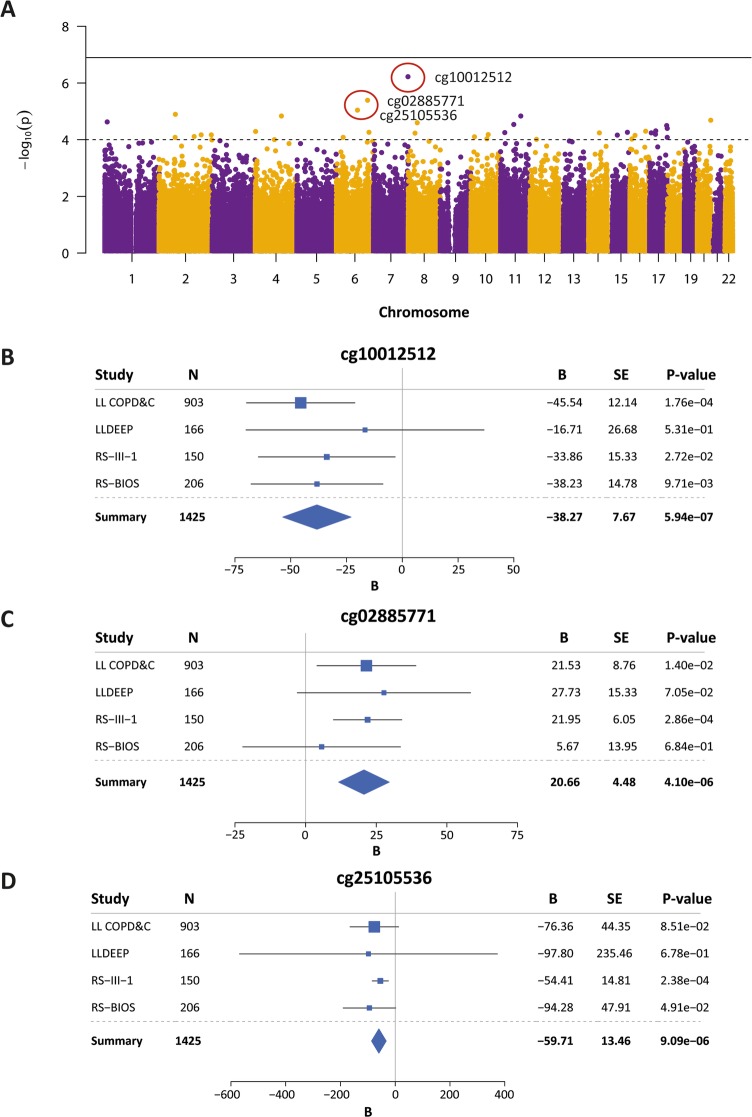
Manhattan and forest plots of the meta-analysis on four independent epigenome-wide association studies on FEV_1_/FVC in never smokers. **a** Manhattan plot in which every dot represents an individual CpG-site. Location on the X-axis indicated the chromosomal position and location on the Y-axis indicates the inversed log [10] *p*-value of the meta-analysis. Dotted horizontal line indicates *p*-value of 0.0001, horizontal fixed line indicates epigenome-wide significance (*p*-value < 0.05/396,243 = 1.26 × 10^^− 7^). **b**-**d** Forest plots showing the effect estimates and standard errors of the 4 independent EWA studies and meta-analysis for the top hits cg10012512 (**b**), cg028885771 (**c**) and cg25105536 (**d**)

The meta-analysis of the four different cohorts did not reveal CpG-sites that were epigenome wide significantly associated with FEV_1_/FVC. We identified 36 CpG-sites as our top associations (Table 2). The Manhattan plot of the meta-analysis is shown in Fig. 1a. Forest plots of the three most significant CpG-sites cg10012512, located in the intergenic region of chromosome 7q36.3 (*p*=5.94 × 10^^− 7^), cg02285771, annotated to LTV1 Ribosome Biogenesis Factor (*LTV1)* (*p*=4.10 × 10^^− 6^) and cg25105536, annotated to Kelch Like Family Member 32 (*KLHL32)* (*p*=9.09 × 10^^− 6^) are shown in Fig. 1b-d. An overview of all CpG-sites associated with FEV_1_/FVC at nominal *p*-value of 0.05 can be found in Additional file 1: Table S1.

**Table 2:** Results of the meta-analysis and individual EWA studies on FEV_1_/FCV in never smokers

		Meta-analysis			LL COPD&C			LLDEEP			RS-III-1			RS-BIOS		
		Beta	SE	*P*-value	Beta	SE	*P*-value	Beta	SE	*P*-value	Beta	SE	*P*-value	Beta	SE	*P*-value
cg10012512	*Intergenic*	−38.27	7.67	5.94E-07	−45.54	12.14	1.76E-04	−16.71	26.68	5.31E-01	−33.86	15.33	2.72E-02	−38.23	14.78	9.71E-03
cg02885771	*LTV1*	20.66	4.48	4.10E-06	21.53	8.76	1.40E-02	27.73	15.33	7.05E-02	21.95	6.05	2.86E-04	5.67	13.95	6.84E-01
cg25105536	*KLHL32*	−59.71	13.46	9.09E-06	−76.36	44.35	8.51E-02	−97.80	235.46	6.78E-01	−54.41	14.81	2.38E-04	−94.28	47.91	4.91E-02
cg20102034	*RTKN*	36.14	8.28	1.28E-05	42.57	15.29	5.35E-03	29.70	15.94	6.25E-02	40.85	14.65	5.29E-03	22.02	24.20	3.63E-01
cg03703840	*KIAA1731*	84.04	19.38	1.45E-05	100.48	42.84	1.90E-02	−43.70	187.80	8.16E-01	88.13	23.36	1.61E-04	33.87	62.55	5.88E-01
cg21614201	*SYNPO2*	−22.66	5.23	1.45E-05	−28.17	13.55	3.76E-02	−25.53	28.56	3.71E-01	−21.10	6.11	5.58E-04	−25.22	17.72	1.55E-01
cg07957088	*PRIC285*	35.48	8.33	2.06E-05	49.48	15.72	1.64E-03	31.33	16.68	6.03E-02	38.68	13.97	5.62E-03	−0.10	24.74	9.97E-01
cg05304461	*C1orf127*	−80.31	19.00	2.37E-05	−95.35	36.04	8.16E-03	152.12	153.04	3.20E-01	−82.63	25.66	1.28E-03	−68.52	47.73	1.51E-01
cg11749902	*Intergenic*	−22.32	5.30	2.55E-05	−26.22	7.75	7.17E-04	−16.37	12.44	1.88E-01	−12.69	14.61	3.85E-01	−24.69	11.32	2.91E-02
cg02207312	*PRPF19*	75.53	18.05	2.87E-05	79.32	53.44	1.38E-01	− 177.08	222.75	4.27E-01	77.18	20.22	1.35E-04	74.46	63.10	2.38E-01
cg19734370	*NPTX1*	12.65	3.04	3.19E-05	12.29	4.11	2.76E-03	12.09	6.95	8.21E-02	9.23	8.85	2.97E-01	17.64	8.07	2.88E-02
cg03077331	*FN3K*	14.19	3.45	3.99E-05	16.08	4.94	1.14E-03	9.62	8.41	2.52E-01	29.01	16.49	7.85E-02	11.51	6.31	6.84E-02
cg18387671	*ANKRD13B*	−88.73	21.86	4.92E-05	− 110.71	69.61	1.12E-01	4.44	272.02	9.87E-01	−87.37	24.33	3.30E-04	−83.43	73.78	2.58E-01
cg03224276	*ZFHX3*	37.55	9.26	5.00E-05	52.17	19.25	6.73E-03	16.06	44.59	7.19E-01	28.97	11.60	1.25E-02	71.59	31.14	2.15E-02
cg02137691	*FGFR3*	28.80	7.11	5.11E-05	13.24	13.60	3.30E-01	40.83	15.87	1.01E-02	35.10	10.64	9.74E-04	16.63	25.22	5.10E-01
cg25884324	*UNC45A*	−36.97	9.16	5.45E-05	−42.03	19.42	3.05E-02	−32.96	50.06	5.10E-01	−35.47	11.31	1.71E-03	−36.84	30.86	2.32E-01
cg27158523	*PPIL4*	−49.97	12.40	5.54E-05	−62.31	22.65	5.94E-03	− 241.34	161.10	1.34E-01	−37.48	14.71	1.09E-02	−83.47	40.23	3.80E-02
cg01157143	*NAV2*	−23.11	5.74	5.63E-05	−31.05	15.70	4.80E-02	−10.87	23.51	6.44E-01	−24.64	6.82	3.03E-04	−8.89	18.20	6.25E-01
cg07160694	*DCAF5*	77.84	19.34	5.69E-05	63.24	40.81	1.21E-01	54.41	155.03	7.26E-01	73.37	27.79	8.29E-03	98.91	36.83	7.24E-03
cg22127773	*KDM6B*	−48.39	12.03	5.75E-05	−58.63	19.17	2.22E-03	3.55	81.11	9.65E-01	−56.26	21.72	9.60E-03	−29.26	22.85	2.00E-01
cg20939319	*TEX15*	−14.90	3.71	5.84E-05	−17.12	8.37	4.07E-02	−26.90	17.30	1.20E-01	−13.61	4.55	2.80E-03	−13.49	12.02	2.62E-01
cg02206852	*PROCA1*	23.87	5.97	6.39E-05	28.18	16.23	8.24E-02	26.98	20.97	1.98E-01	22.38	7.02	1.45E-03	27.78	24.10	2.49E-01
cg17075019	*Intergenic*	35.53	8.90	6.56E-05	49.59	13.38	2.12E-04	26.62	17.55	1.29E-01	13.65	25.97	5.99E-01	28.14	20.81	1.76E-01
cg25556432	*Intergenic*	23.02	5.78	6.75E-05	25.96	8.69	2.82E-03	21.69	13.17	9.95E-02	32.14	17.96	7.36E-02	15.46	11.29	1.71E-01
cg22742965	*TMEFF2*	−17.79	4.47	6.76E-05	−24.96	11.10	2.45E-02	0.42	20.86	9.84E-01	−17.82	5.43	1.03E-03	−14.83	13.14	2.59E-01
cg16734845	*CTDSPL2*	−33.94	8.52	6.82E-05	−54.67	21.90	1.26E-02	−38.26	26.03	1.42E-01	−31.88	10.86	3.32E-03	−15.33	24.10	5.25E-01
cg09108394	*PRKCB*	−14.93	3.76	7.11E-05	−16.43	8.33	4.84E-02	−27.78	14.95	6.31E-02	−14.34	4.92	3.55E-03	−9.74	9.71	3.16E-01
cg10034572	*Intergenic*	−20.08	5.08	7.77E-05	−19.86	13.39	1.38E-01	−56.52	27.77	4.18E-02	−19.29	5.90	1.09E-03	−12.71	17.73	4.73E-01
cg20066227	*C1QL3*	32.20	8.16	7.92E-05	26.51	18.29	1.47E-01	24.42	30.70	4.26E-01	40.00	10.35	1.12E-04	3.19	24.73	8.97E-01
cg07148038	*TNXB*	44.32	11.26	8.23E-05	51.79	16.72	1.95E-03	41.06	24.11	8.85E-02	55.29	30.47	6.96E-02	22.61	25.67	3.78E-01
cg23396786	*SFXN5*	20.16	5.12	8.26E-05	22.48	7.68	3.43E-03	13.97	10.89	2.00E-01	45.93	18.48	1.30E-02	13.79	10.08	1.71E-01
cg06218079	*TBCD*	8.18	2.08	8.34E-05	5.68	3.00	5.79E-02	12.74	3.45	2.26E-04	3.33	8.96	7.10E-01	6.35	6.52	3.30E-01
cg06982745	*ADAMTS14*	−40.80	10.44	9.37E-05	−36.77	18.57	4.77E-02	13.29	44.30	7.64E-01	−48.83	14.67	8.71E-04	−42.55	30.04	1.57E-01
cg05946118	*Intergenic*	−20.27	5.19	9.38E-05	−17.24	6.98	1.35E-02	−23.39	14.23	1.00E-01	−25.24	13.56	6.28E-02	−23.41	12.66	6.46E-02
cg08065963	*Intergenic*	−16.72	4.28	9.56E-05	−18.12	5.84	1.93E-03	−9.56	11.07	3.88E-01	−29.63	11.66	1.10E-02	−8.68	10.18	3.94E-01
cg12064372	*Intergenic*	32.85	8.43	9.75E-05	48.15	18.52	9.33E-03	26.64	92.88	7.74E-01	31.50	10.10	1.81E-03	7.96	28.48	7.80E-01

Ranking of CpG-sites is based on the *P*-value of the meta-analysis

The direction of the effect of the 36 top CpG-sites did not change in a sensitivity analysis in the LL COPD&C cohort excluding the subjects that were exposed to environmental tobacco smoke (ETS)(*N*=659 subjects) (Additional file 2: Table S2).

### Expression quantitative trait methylation (eQTM) analysis

In total, 803 genes were located within 2 MB of the 36 CpG-sites. The expression of 11 genes was significantly associated with DNA methylation levels at the 9 different CpG-sites (Table 3). DNA methylation at cg25105536, annotated to *KLHL32*, was significantly associated with gene expression levels of *KLHL32*. DNA methylation levels at cg08065963, located in the intergenic region on chromosome 16 and not yet annotated to a gene, showed a significant association with gene expression levels of 4-Aminobutyrate Aminotransferase *(ABAT)*. For the other 7 CpG-sites, DNA methylation levels were associated with gene expression levels of one or two genes other than the previously annotated genes. An overview of the association between DNA methylation and gene expression levels of all genes can be found in Additional file 3: Table S3.

**Table 3:** Overview of the results of the meta-analysis of the eQTM analysis

CpG-site	Gene annotation CpG-site	Genes located within 1 MB (N)	Gene (expression)	Beta	SE	*p*-value	Adjusted *p*-value
cg02137691	*FGFR3*	31	*SLC26A1*	0.0156	0.0038	3.53E-05	0.0011
cg02206852	*PROCA1*	52	*NUFIP2*	0.0084	0.0022	1.06E-04	0.0055
cg02206852	*PROCA1*	52	*GIT1*	0.0080	0.0023	6.11E-04	0.0318
cg02885771	*LTV1*	11	*VDAC1P8*	0.0096	0.0033	3.51E-03	0.0386
cg07148038	*TNXB*	89	*ATP6V1G2*	0.0074	0.0021	3.79E-04	0.0337
cg07148038	*TNXB*	89	*STK19B*	0.0035	0.0010	3.77E-04	0.0335
cg08065963		12	*ABAT*	0.0127	0.0034	1.85E-04	0.0022
cg20939319	*TEX15*	10	*SARAF*	−0.0029	0.0010	3.36E-03	0.0336
cg22127773	*KDM6B*	80	*TMEM88*	0.0011	0.0003	1.82E-04	0.0146
cg23396786	*SFXN5*	18	*CYP26B1*	0.0024	0.0008	1.78E-03	0.0321
cg25105536	*KLHL32*	4	*KLHL32*	−0.0004	0.0002	5.52E-03	0.0221

## Discussion

This study is the first large general population-based EWA study on lung function in never smokers. So far, virtually all EWA studies on the origin of COPD included subjects with a history of cigarette smoking. As a consequence, these studies mainly addressed the origins of COPD in response to smoking. It is unclear if the results of these studies help to explain the etiology of COPD or rather explain the contribution of cigarette smoke towards the disease. Therefore, our study importantly contributes to the current understanding of COPD in never smokers.

We identified 36 CpG-sites that were significantly associated with FEV_1_/FVC at *p*-value below 0.0001. The top hit of our meta-analysis, cg10012512, is located in the intergenic region of chromosome 7q36.3. It is therefore not possible to speculate on the functional effect of differences in DNA methylation at this specific CpG-site and how these differences may affect FEV_1_/FVC. While associations found with an eQTM analysis may help to get more insight in the function of a CpG-site, our eQTM analysis did not reveal any nominal significant associations for cg10012512. However, this CpG-site was differentially methylated between never smokers and current smokers [23]. Presumably, this CpG-site does also respond to other inhaled deleterious substances, which in turn affects lung function. The second top hit, cg02885771 located on chromosome 6q24.2 is annotated *LTV1*. Previously, this CpG-site has been associated with asthma in airway epithelial cells [24] and *LTV1* was shown to be expressed in lung tissue in the Genotype Tissue Expression (GTEx) project. Although studies in yeast describe *LTV1* as a conserved 40S-associated biogenesis factor that functions in small subunit nuclear export, a specific role for *LTV1* in respiratory diseases is not known [25]. The third top hit, cg25105536, is annotated to *KLHL32* on chromosome 6q16.1 and we found a significant association between DNA methylation levels of cg25105536 and gene expression levels of *KLHL32*. The function of *KLHL32* is poorly understood, however, four genetic variants in the *KLHL32* gene have been associated with FEV_1_ and FEV_1_/FVC in African American subjects with COPD and a history of smoking [26]. Notwithstanding the fact that these associations were only identified in a specific group, it might suggest a role for *KLHL32* in the respiratory system. Next to *KLHL32*, we found that gene expression levels of 10 additional genes were significantly associated with DNA methylation levels at one of the 36 CpG-sites. cg08065963, which was not yet annotated to a gene, was significantly associated with 4-Aminobutyrate Aminotransferase (*ABAT*). Interestingly, a role for *ABAT* in COPD has not been described before. The remaining nine genes were other genes than the annotated genes of the particular CpG-sites. This suggest that the CpG-sites may also regulate distant genes within a region of 2 MB, which complicates the functional assessment of differences in DNA methylation even further.

To the best of our knowledge, there are eight studies in literature describing the association between DNA methylation and lung function (Table 4). Six of these studies included both subjects with and without a history of cigarette smoking and, except for the study by *Qui* et al., adjusted for smoking status in the statistical analysis. In addition, the recent study by *Imboden* et al. performed analyses with and without adjustment for smoking status and pack years. Altogether, these seven studies identified 462 unique CpG-sites. Interestingly, none of the 36 CpG-sites from our meta-analysis in never smokers were among these 462 previously identified CpG-sites (Table 5). Apparently these 36 CpG-sites are only associated with lung function level in never smokers. The fact that 17 CpG-sites (47%) were associated at nominal *p*-value < 0.05 with COPD (dichotomously defined as the ratio of FEV_1_/FVC below 70%) in our previously EWAS stratified for never smoking, further underscores this assumption [16]. There is, however, one exception, since cg22742965, annotated to Transmembrane Protein With EGF Like And Two Follistatin Like Domains 2 (*TMEFF2)*, was also significantly associated with COPD in smokers. Most likely, this CpG-site shows a general response to inhaled deleterious substances such as cigarette smoke and other yet unknown substances.

**Table 4:** Overview of studies reporting results of differential DNA methylation with lung function or COPD in whole blood

Study	Study population	Trait	Adjustment included in model	DNA methylation platform	Number of CpG-sites available for comparison
Epigenome-wide association study of lung function level and its change *Imboden* et al., 2019 [17]	Discovery-replication approach. Discovery included 3 cohorts (*N*=2043) and replication included 7 cohorts (Adult: *N*=3327, Childhood: *N*=420) - Smoking status: self-reported, subjects with and without smoking history; never smokers only	- FEV_1_ - FVC - FEV_1_/FVC Analyses were performed twice: with and without adjustment for smoking status and pack years	- Age - Age^2^ - Height - Height^2^ deviation - Sex - Sex Age, Age^2^, height, Height^2^ deviation - Education - BMI - Spirometer type - Study Center - Blood cell composition	Discovery: Illumina Infinium Human Methylation 450 K BeadChip and EPIC BeadChip Replication: various arrays for the discovery-identified CpG-sites only	Without smoking adjustment: 56^a^ With smoking adjustment: 12^a^ Never smokers: 8 (from discovery). None of the CpG sites were replicated^a^
No association between DNA methylation and COPD in never and current smokers *De Vries* et al., 2018 [16]	Non-random selection from LifeLines cohort (*N*=1561 subjects) - Smoking status: Stratified for smoking (658 smokers and 903 never smokers)	- COPD (defined as FEV_1_/FVC ≤ 0.7)	- Sex - Age - Pack years (in smoking stratified analysis) - Batch effects - Blood cell composition	Illumina Infinium Human Methylation450K BeadChip array - Number of included probes: 420,938	Smokers: 19492^b^ Never smokers: 19393^b^
Lung function discordance in monozygotic twins and ssociated differences in blood DNA methylation *Bolund* et al., 2017 [11]	Sub-population of twins from the Middle-Aged Danish Twin (MADT) study (*N*=169 twin pairs) - Smoking status: subjects with and without smoking history	Intra-pair difference in *z*-score calculated as “superior” minus “inferior” twin at baseline and during follow-up period for: - FEV_1_ - FVC - FEV_1_/FVC	- Sex - Age - BMI - Pack years - Smoking status at follow-up - Blood cell composition Intra-pair difference was calculated for all the variables	Illumina Infinium Human Methylation450K BeadChip array - Number of included probes: 453,014	37^a^
Epigenome-wide association study of chronic obstructive pulmonary disease and lung function in Koreans *Lee* et al., 2017 [12]	Sample of Korean COPD cohort (*N*=100 subjects) - Smoking status: subjects with and without smoking history	- COPD status (defined as FEV_1_/FVC < 0.7) - FEV_1_ - FVC - FEV_1_/FVC	- Sex - Age - Height - Smoking status - Pack years - Blood cell composition	Illumina Infinium Human Methylation450K BeadChip array - Number of included probes: 402,508	16^a^
Differential DNA methylation marks and gene comethylation of COPD in African-Americans with COPD exacerbations *Busch* et al., 2016 [13]	Sample of PA-SCOPE AA study population (*N*=362 subjects) - Smoking status: smokers > 20 pack years	- COPD (defined as FEV_1_/FVC ≤ 0.7 and FEV_1_ ≤ 80%)	- Sex - Age - Pack years - Batch number - Blood cell composition	Illumina Infinium Human Methylation27K BeadChip array - Number of included probes: 19,302	12^a^
The epigenetic clock is correlated with physical and cognitive fitness in the Lothian Birth Cohort *Marioni* et al., 2015 [15]	The Lothian Birth Cohort of 1936 (*N*=1091) - Smoking status: self-reported, subjects with and without smoking history	- FEV_1_	- Sex - Age - Height - Smoking status - Blood cell composition	Illumina Infinium Human Methylation450K BeadChip array - Number of included probes: 450,726	2^a^
Variable DNA methylation is associated with chronic obstructive pulmonary disease and lung function *Qiu* et al., 2012 [10]	Test-replication approach in 2 family-based cohorts (*N*=1085 and 369 subjects) - Smoking status: subjects with and without smoking history	- COPD status (FEV_1_/FVC ≤0.7 and FEV_1_ ≤70%) - FEV_1_/FVC - FEV_1_	- Random family effect	Illumina Infinium Human Methylation27K BeadChip array - Number of included probes: 26,485	349^a^
Epigenome-wide scans identify differentially methylated regions for age and age-related phenotypes in a healthy ageing population *Bell* et al., 2012 [14]	Sample of the TwinsUK cohort (*N*=172 female twin pairs) - Smoking status: unknown	- FEV_1_ - FVC	- Age - Batch effects	Illumina Infinium Human Methylation27K BeadChip array - Number of included probes: 24,641	1^a^

*COPD* Chronic Obstructive Pulmonary Disease, *FEV*_*1*_ Forced Expiratory Volume in 1 s, *FVC* Forced Expiratory Capacity

^a^CpG-sites obtained from the online available data

^b^CpG-sites selected at nominal *p*-value < 0.05 available from self-performed analyses

**Table 5:** Overview of CpG location, gene annotation, gene function and literature comparison of the top 36 CpG-sites of the meta analysis

CpG-site	CpG location	Gene annotation	Gene function	Previously associated with lung function
cg10012512	7:157224041	*Intergenic*	*NA*	Yes^a^
cg02885771	6:144163654	*LTV1*	Involved in ribosome biogenesis	No
cg25105536	6:97372436	*KLHL32*	Only described as protein coding gene	No
cg20102034	2:74653166	*RTKN*	Negative regulator of GTPase activity of Rho proteins	Yes^a^
cg03703840	11:93394809	*KIAA1731*	Mediating of centriole-to-centrosome conversion at late mitosis	No
cg21614201	4:119888794	*SYNPO2*	Only described as protein coding gene	No
cg07957088	20:62196387	*PRIC285*	Nuclear transcriptional co-activator for peroxisome proliferator activated receptor alpha	Yes^a^
cg05304461	1:11019377	*C1orf127*	Only described as protein coding gene	No
cg11749902	8:41093619	*Intergenic*	*NA*	Yes^a^
cg02207312	11:60674164	*PRPF19*	Involved in cell survival and DNA repair	No
cg19734370	17:78444348	*NPTX1*	Exclusively localized to the nervous system as binding protein for taipoxin	Yes^a^
cg03077331	17:80693076	*FN3K*	Catalyzes the phosphorylation of fructosamines	Yes^a^
cg18387671	17:27920246	*ANKRD13B*	Only described as protein coding gene	Yes^a^
cg03224276	16:72829831	*ZFHX3*	Regulates myogenic and neuronal differentiation	No
cg02137691	4:1805671	*FGFR3*	Involved in bone development and maintenance	No
cg25884324	15:91482502	*UNC45A*	Regulator of the progesterone receptor chaperoning pathway	No
cg27158523	6:149867355	*PPIL4*	Involved in protein folding, immunosuppression and infection of HIV-1 virions	Yes^a^
cg01157143	11:19478542	*NAV2*	Plays a role in cellular growth and migration	No
cg07160694	14:69619856	*DCAF5*	Only described as protein coding gene	No
cg22127773	17:7754785	*KDM6B*	Demethylation of di- or tri-methylated lysine 27 of histone H3	Yes^a^
cg20939319	8:30707701	*TEX15*	Involved in cell cycle processes of spermatocytes	No
cg02206852	17:27030540	*PROCA1*	Only described as protein coding gene	No
cg17075019	10:79541650	*Intergenic*	*NA*	Yes^a^
cg25556432	2:239628926	*Intergenic*	*NA*	Yes^a^
cg22742965	2:192891657	*TMEFF2*	Cellular context-dependent oncogene or tumor suppressor	Yes
cg16734845	15:44781962	*CTDSPL2*	Only described as protein coding gene	No
cg09108394	16:23850106	*PRKCB*	As kinase involved in diverse cellular signaling pathways	No
cg10034572	2:160921789	*Intergenic*	*NA*	No
cg20066227	10:16564552	*C1QL3*	Only described as protein coding gene	No
cg07148038	6:32061160	*TNXB*	Anti-adhesive protein involved in matrix maturation during wound healing	Yes^a^
cg23396786	2:73299151	*SFXN5*	Only described as protein coding gene	Yes^a^
cg06218079	17:80834228	*TBCD*	As co-factor D involved in the correct folding of beta-tubulin	No
cg06982745	10:72454006	*ADAMTS14*	The matured enzyme is involved in the formation of collagen fibers	No
cg05946118	16:8985638	*Intergenic*	*NA*	Yes^a^
cg08065963	16:8985593	*Intergenic*	*NA*	Yes^a^
cg12064372	12:30948792	*Intergenic*	*NA*	Yes^a^

^a^Only observed in study by *de Vries* et al. in never smokers; Gene function obtained by www.genecards.org

Assuming that the observed differential DNA methylation at the majority of the CpG-sites in our study occurs without exposure to smoking, the question arises why this differential DNA methylation is observed. One possible explanation may be that other factors within the environment such as air pollution and job-related exposures are responsible for the observed differences in DNA methylation. Recently, we studied the epigenome-wide association between DNA methylation and exposure to air pollution and job-related exposures in a selection of the LifeLines population cohort including both never and current smokers [19, 27]. While we did find significant associations, none of them were replicated in independent cohorts. Additional analyses in never smokers for this paper did not reveal novel associations between DNA methylation and environmental exposures (Additional file 4: Table S4 and Additional file 5: Figure S1). This might potentially be due to lack of power, since only a small percentage of the subjects that have never smoked in the LL COPD&C cohort have been exposed to environmental exposures. Moreover, exposure levels to air pollution in the LL COPD&C are relatively low compared to the average Dutch levels determined within the 2012 Dutch national health survey as described by Strak et al [28]. Next to environmental exposures, another explanation may be that a reduced lung function level precedes the differences in DNA methylation. However, with the cross-sectional design of this study, we cannot derive conclusions on the direction of the association and causality. Large longitudinal studies are required to investigate causality between DNA methylation and FEV_1_/FVC. Moreover, this will give the opportunity to investigate if low levels of FEV_1_ and decline in FEV_1_ over the years is associated with DNA methylation in never smokers.

## Conclusions

With this study we show that epigenetics indeed may be associated with FEV_1_/FVC in subjects who never smoked. Moreover, since 35 out of the 36 identified CpG-sites are unique for never smokers, our data suggest that factors other than smoking affect FEV_1_/FVC via DNA methylation.

## Supplementary information

**Additional file 1: Table S1.** Overview of all CpG-sites associated with FEV1/FVC at nominal *p*-value of 0.05.

**Additional file 2: Table S2.** Sensitivity analysis of the association of the top 36 CpG-sites with FEV_1_/FVC in 659 subjects that were not exposed to environmental tobacco smoke.

**Additional file 3: Table S3.** Overview of association between DNA methylation and gene expression.

**Additional file 4: Table S4.** Results of the association between 36 top CpG-sites identified from the meta-analysis and A: environmental exposures and B: air pollution measurements.

**Additional file 5: Figure S1:** Forest plots of the associations between DNA methylation and environmental exposures.

## Data Availability

The datasets and/or analyzed during the current study are available from the corresponding authors on reasonable request. Summary statistics of the meta-analysis and the four individual EWAS studies with nominal *p*-value of 0.05 have been made freely available as Additional file.

## Abbreviations

ATS: American Thoracic Society
BIOS: Biobank-based Integrative Omics Studies
COPD: Chronic Obstructive Pulmonary Disease
CpG: Cytosine-phosphate-Guanine
DNA: Deoxyribonucleic acid
eQTM: Expression Quantitative Trait Methylation
ERS: European Respiratory Society
ETS: Environmental tobacco smoke
EWAS: Epigenome-wide association study
FEV_1_: Forced expiratory volume in 1 s
FVC: Forced vital capacity
GTEx: Genotype tissue expression
