# Supplementary material for: DNA methylation is associated with lung function in never smokers
Source: Respir Res. 2019 Dec 2;20:268. doi: 10.1186/s12931-019-1222-8 (PMC6889726; doi:10.1186/s12931-019-1222-8)
Supplement: Supplementary file 1 — Additional file 1: Table S1. Overview of all CpG-sites associated with FEV1/FVC at nominal p-value of 0.05. [file 12931_2019_1222_MOESM1_ESM.pdf]

Online supplement table 1: Overview of al CpG-sites associated with FEV_1/FVC at nominal p-value of 0.05.

**Study**	**Meta**			**LL COPD&C**			**LLDEEP**			**RS-III-1**			**RS-BIOS**		
**Variable**	**Beta**	**SE**	**P-value**	**Beta**	**SE**	**P-value**	**Beta**	**SE**	**P-value**	**Beta**	**SE**	**P-value**	**Beta**	**SE**	**P-value**
cg10012512	-38.27	7.67	5.94E-07	-45.54	12.14	1.76E-04	-16.71	26.68	5.31E-01	-33.86	15.33	2.72E-02	-38.23	14.78	9.71E-03
cg02885771	20.66	4.48	4.10E-06	21.53	8.76	1.40E-02	27.73	15.33	7.05E-02	21.95	6.05	2.86E-04	5.67	13.95	6.84E-01
cg25105536	-59.71	13.46	9.09E-06	-76.36	44.35	8.51E-02	-97.80	235.46	6.78E-01	-54.41	14.81	2.38E-04	-94.28	47.91	4.91E-02
cg20102034	36.14	8.28	1.28E-05	42.57	15.29	5.35E-03	29.70	15.94	6.25E-02	40.85	14.65	5.29E-03	22.02	24.20	3.63E-01
cg03703840	84.04	19.38	1.45E-05	100.48	42.84	1.90E-02	-43.70	187.80	8.16E-01	88.13	23.36	1.61E-04	33.87	62.55	5.88E-01
cg21614201	-22.66	5.23	1.45E-05	-28.17	13.55	3.76E-02	-25.53	28.56	3.71E-01	-21.10	6.11	5.58E-04	-25.22	17.72	1.55E-01
cg07957088	35.48	8.33	2.06E-05	49.48	15.72	1.64E-03	31.33	16.68	6.03E-02	38.68	13.97	5.62E-03	-0.10	24.74	9.97E-01
cg05304461	-80.31	19.00	2.37E-05	-95.35	36.04	8.16E-03	152.12	153.04	3.20E-01	-82.63	25.66	1.28E-03	-68.52	47.73	1.51E-01
cg11749902	-22.32	5.30	2.55E-05	-26.22	7.75	7.17E-04	-16.37	12.44	1.88E-01	-12.69	14.61	3.85E-01	-24.69	11.32	2.91E-02
cg02207312	75.53	18.05	2.87E-05	79.32	53.44	1.38E-01	-177.08	222.75	4.27E-01	77.18	20.22	1.35E-04	74.46	63.10	2.38E-01
cg19734370	12.65	3.04	3.19E-05	12.29	4.11	2.76E-03	12.09	6.95	8.21E-02	9.23	8.85	2.97E-01	17.64	8.07	2.88E-02
cg03077331	14.19	3.45	3.99E-05	16.08	4.94	1.14E-03	9.62	8.41	2.52E-01	29.01	16.49	7.85E-02	11.51	6.31	6.84E-02
cg18387671	-88.73	21.86	4.92E-05	-110.71	69.61	1.12E-01	4.44	272.02	9.87E-01	-87.37	24.33	3.30E-04	-83.43	73.78	2.58E-01
cg03224276	37.55	9.26	5.00E-05	52.17	19.25	6.73E-03	16.06	44.59	7.19E-01	28.97	11.60	1.25E-02	71.59	31.14	2.15E-02
cg02137691	28.80	7.11	5.11E-05	13.24	13.60	3.30E-01	40.83	15.87	1.01E-02	35.10	10.64	9.74E-04	16.63	25.22	5.10E-01
cg25884324	-36.97	9.16	5.45E-05	-42.03	19.42	3.05E-02	-32.96	50.06	5.10E-01	-35.47	11.31	1.71E-03	-36.84	30.86	2.32E-01
cg27158523	-49.97	12.40	5.54E-05	-62.31	22.65	5.94E-03	-241.34	161.10	1.34E-01	-37.48	14.71	1.09E-02	-83.47	40.23	3.80E-02
cg01157143	-23.11	5.74	5.63E-05	-31.05	15.70	4.80E-02	-10.87	23.51	6.44E-01	-24.64	6.82	3.03E-04	-8.89	18.20	6.25E-01
cg07160694	77.84	19.34	5.69E-05	63.24	40.81	1.21E-01	54.41	155.03	7.26E-01	73.37	27.79	8.29E-03	98.91	36.83	7.24E-03
cg22127773	-48.39	12.03	5.75E-05	-58.63	19.17	2.22E-03	3.55	81.11	9.65E-01	-56.26	21.72	9.60E-03	-29.26	22.85	2.00E-01
cg20939319	-14.90	3.71	5.84E-05	-17.12	8.37	4.07E-02	-26.90	17.30	1.20E-01	-13.61	4.55	2.80E-03	-13.49	12.02	2.62E-01
cg02206852	23.87	5.97	6.39E-05	28.18	16.23	8.24E-02	26.98	20.97	1.98E-01	22.38	7.02	1.45E-03	27.78	24.10	2.49E-01
cg17075019	35.53	8.90	6.56E-05	49.59	13.38	2.12E-04	26.62	17.55	1.29E-01	13.65	25.97	5.99E-01	28.14	20.81	1.76E-01
cg25556432	23.02	5.78	6.75E-05	25.96	8.69	2.82E-03	21.69	13.17	9.95E-02	32.14	17.96	7.36E-02	15.46	11.29	1.71E-01
cg22742965	-17.79	4.47	6.76E-05	-24.96	11.10	2.45E-02	0.42	20.86	9.84E-01	-17.82	5.43	1.03E-03	-14.83	13.14	2.59E-01
cg16734845	-33.94	8.52	6.82E-05	-54.67	21.90	1.26E-02	-38.26	26.03	1.42E-01	-31.88	10.86	3.32E-03	-15.33	24.10	5.25E-01
cg09108394	-14.93	3.76	7.11E-05	-16.43	8.33	4.84E-02	-27.78	14.95	6.31E-02	-14.34	4.92	3.55E-03	-9.74	9.71	3.16E-01
cg10034572	-20.08	5.08	7.77E-05	-19.86	13.39	1.38E-01	-56.52	27.77	4.18E-02	-19.29	5.90	1.09E-03	-12.71	17.73	4.73E-01
cg20066227	32.20	8.16	7.92E-05	26.51	18.29	1.47E-01	24.42	30.70	4.26E-01	40.00	10.35	1.12E-04	3.19	24.73	8.97E-01
cg07148038	44.32	11.26	8.23E-05	51.79	16.72	1.95E-03	41.06	24.11	8.85E-02	55.29	30.47	6.96E-02	22.61	25.67	3.78E-01
cg23396786	20.16	5.12	8.26E-05	22.48	7.68	3.43E-03	13.97	10.89	2.00E-01	45.93	18.48	1.30E-02	13.79	10.08	1.71E-01

cg06218079	8.18	2.08	8.34E-05	5.68	3.00	5.79E-02	12.74	3.45	2.26E-04	3.33	8.96	7.10E-01	6.35	6.52	3.30E-01
cg06982745	-40.80	10.44	9.37E-05	-36.77	18.57	4.77E-02	13.29	44.30	7.64E-01	-48.83	14.67	8.71E-04	-42.55	30.04	1.57E-01
cg05946118	-20.27	5.19	9.38E-05	-17.24	6.98	1.35E-02	-23.39	14.23	1.00E-01	-25.24	13.56	6.28E-02	-23.41	12.66	6.46E-02
cg08065963	-16.72	4.28	9.56E-05	-18.12	5.84	1.93E-03	-9.56	11.07	3.88E-01	-29.63	11.66	1.10E-02	-8.68	10.18	3.94E-01
cg12064372	32.85	8.43	9.75E-05	48.15	18.52	9.33E-03	26.64	92.88	7.74E-01	31.50	10.10	1.81E-03	7.96	28.48	7.80E-01
cg24551420	49.03	12.60	1.00E-04	1.67	40.99	9.67E-01	1.22	170.35	9.94E-01	52.05	13.80	1.61E-04	83.07	49.36	9.24E-02
cg19390934	-18.69	4.81	1.01E-04	-17.37	7.46	1.98E-02	-14.50	11.51	2.08E-01	-22.01	11.40	5.35E-02	-21.68	9.99	3.00E-02
cg02737554	-16.15	4.17	1.08E-04	-28.44	8.38	6.88E-04	-11.94	14.77	4.19E-01	-11.47	5.71	4.45E-02	-14.63	11.24	1.93E-01
cg17920789	-15.88	4.11	1.12E-04	-29.31	10.27	4.34E-03	-19.69	19.18	3.04E-01	-14.20	4.97	4.28E-03	-5.19	12.39	6.75E-01
cg06960413	24.47	6.34	1.14E-04	39.41	11.95	9.74E-04	8.43	22.77	7.11E-01	21.51	8.88	1.55E-02	13.41	17.47	4.43E-01
cg01459673	40.37	10.47	1.15E-04	20.12	42.53	6.36E-01	70.56	114.17	5.37E-01	40.64	11.12	2.58E-04	56.42	49.09	2.50E-01
cg18485530	-14.40	3.74	1.20E-04	-5.85	9.14	5.22E-01	-9.65	14.13	4.95E-01	-17.92	4.58	9.03E-05	-8.07	12.28	5.11E-01
cg09547025	-8.70	2.26	1.23E-04	-4.65	4.26	2.75E-01	-9.82	5.22	6.00E-02	-9.46	3.51	7.06E-03	-14.06	6.70	3.59E-02
cg21647560	-19.98	5.20	1.23E-04	-20.13	7.93	1.11E-02	-24.45	11.76	3.75E-02	-18.59	15.86	2.41E-01	-16.99	10.09	9.22E-02
cg16672557	32.01	8.34	1.23E-04	56.78	18.52	2.18E-03	26.25	21.13	2.14E-01	25.85	11.07	1.95E-02	23.64	30.65	4.41E-01
cg07476963	-12.90	3.37	1.29E-04	-12.85	6.49	4.76E-02	-2.45	8.23	7.66E-01	-15.60	4.99	1.77E-03	-17.95	10.35	8.28E-02
cg05888917	37.22	9.74	1.32E-04	48.46	27.85	8.18E-02	-9.39	120.66	9.38E-01	34.42	10.88	1.56E-03	53.99	36.81	1.42E-01
cg13205384	17.86	4.68	1.35E-04	0.13	14.95	9.93E-01	83.76	70.42	2.34E-01	18.99	5.12	2.08E-04	25.73	18.64	1.67E-01
cg22069588	47.10	12.34	1.36E-04	43.08	14.77	3.54E-03	-22.55	64.47	7.27E-01	6.69	77.60	9.31E-01	73.69	25.20	3.46E-03
cg08445550	-17.98	4.71	1.36E-04	-21.51	7.08	2.39E-03	-12.81	10.52	2.23E-01	-13.63	12.10	2.60E-01	-18.62	10.40	7.35E-02
cg03870501	28.61	7.50	1.37E-04	40.25	16.17	1.28E-02	31.40	12.36	1.11E-02	22.42	13.27	9.10E-02	12.46	24.19	6.07E-01
cg03645088	-21.98	5.78	1.44E-04	-2.70	18.86	8.86E-01	-38.98	44.52	3.81E-01	-24.87	6.31	8.18E-05	-4.12	25.73	8.73E-01
cg07425474	34.07	8.97	1.45E-04	55.07	15.60	4.16E-04	22.98	17.34	1.85E-01	27.53	16.52	9.56E-02	15.08	27.33	5.81E-01
cg11362049	-11.37	3.00	1.47E-04	-15.17	5.25	3.83E-03	-7.29	6.96	2.95E-01	-8.15	4.80	8.97E-02	-19.21	9.52	4.35E-02
cg16253580	-13.88	3.67	1.53E-04	-23.13	8.68	7.72E-03	-11.63	13.39	3.85E-01	-12.83	4.59	5.14E-03	-6.38	11.14	5.67E-01
cg13245440	-27.00	7.13	1.54E-04	-27.86	13.70	4.20E-02	-35.96	29.32	2.20E-01	-27.22	9.52	4.24E-03	-18.76	21.72	3.88E-01
cg04997017	-23.32	6.17	1.57E-04	-40.59	16.36	1.31E-02	-35.82	29.47	2.24E-01	-18.02	7.11	1.13E-02	-39.18	24.84	1.15E-01
cg17095171	-28.65	7.61	1.67E-04	-41.33	19.57	3.47E-02	-23.52	26.45	3.74E-01	-28.60	9.16	1.79E-03	-9.38	27.64	7.34E-01
cg07020540	30.47	8.10	1.68E-04	28.43	13.58	3.63E-02	18.89	31.56	5.50E-01	27.57	11.51	1.66E-02	65.69	28.09	1.94E-02
cg04338250	29.59	7.87	1.70E-04	42.51	14.94	4.43E-03	25.30	19.32	1.90E-01	14.47	13.21	2.73E-01	41.94	17.52	1.67E-02
cg00019511	25.21	6.71	1.71E-04	37.46	17.70	3.43E-02	24.50	30.24	4.18E-01	23.06	7.80	3.13E-03	23.27	25.75	3.66E-01
cg01986263	-25.32	6.74	1.73E-04	-33.58	10.86	1.99E-03	-3.63	15.37	8.13E-01	-28.30	16.53	8.70E-02	-27.25	13.33	4.09E-02
cg07598082	-56.80	15.14	1.75E-04	-48.45	22.71	3.29E-02	11.15	52.80	8.33E-01	-64.47	28.60	2.42E-02	-93.38	34.26	6.42E-03
cg05331807	-24.55	6.55	1.76E-04	-23.54	10.13	2.02E-02	-14.35	17.44	4.10E-01	-35.52	16.59	3.23E-02	-25.08	12.23	4.04E-02
cg25302730	-25.28	6.74	1.78E-04	-4.00	18.92	8.33E-01	-6.12	32.57	8.51E-01	-29.21	7.64	1.31E-04	-34.48	30.07	2.52E-01

cg07280482	48.47	12.94	1.80E-04	68.31	40.67	9.30E-02	-24.12	142.69	8.66E-01	45.43	14.33	1.53E-03	62.57	47.06	1.84E-01
cg11677105	10.15	2.71	1.80E-04	6.88	3.81	7.13E-02	8.73	5.70	1.26E-01	17.07	9.43	7.02E-02	17.70	6.29	4.86E-03
cg07909265	60.75	16.24	1.83E-04	54.97	20.13	6.31E-03	1.69	84.68	9.84E-01	167.41	72.18	2.04E-02	62.80	31.75	4.79E-02
cg16526137	60.17	16.09	1.85E-04	52.25	53.55	3.29E-01	-118.99	236.36	6.15E-01	65.39	17.88	2.56E-04	32.08	52.09	5.38E-01
cg27554565	23.03	6.18	1.93E-04	5.24	23.76	8.25E-01	-33.30	86.28	7.00E-01	26.04	6.65	9.02E-05	5.88	24.31	8.09E-01
cg16016319	57.06	15.36	2.04E-04	53.97	20.95	9.99E-03	48.24	54.76	3.78E-01	59.32	45.46	1.92E-01	64.88	29.62	2.85E-02
cg11143953	11.35	3.07	2.13E-04	10.80	4.83	2.53E-02	9.93	8.70	2.54E-01	12.20	5.29	2.11E-02	12.20	8.29	1.41E-01
cg19983815	68.25	18.44	2.15E-04	123.19	53.47	2.12E-02	-55.35	153.43	7.18E-01	69.27	20.71	8.21E-04	-7.58	68.09	9.11E-01
cg06532856	-27.94	7.56	2.17E-04	-40.16	11.82	6.84E-04	-20.64	15.24	1.76E-01	-36.69	21.76	9.18E-02	-9.09	15.93	5.68E-01
cg12977942	-10.54	2.85	2.23E-04	-6.65	5.02	1.85E-01	-13.39	7.90	9.02E-02	-13.18	4.56	3.89E-03	-9.56	7.23	1.87E-01
cg16455376	-22.02	5.97	2.26E-04	-28.49	10.04	4.52E-03	-17.03	16.49	3.02E-01	-24.07	10.11	1.73E-02	-7.89	14.63	5.90E-01
cg01110525	40.61	11.02	2.28E-04	72.62	42.04	8.41E-02	-143.79	158.95	3.66E-01	40.00	11.77	6.76E-04	25.07	49.26	6.11E-01
cg21776674	29.40	7.98	2.29E-04	32.09	12.97	1.34E-02	35.05	15.37	2.26E-02	13.47	18.23	4.60E-01	32.56	19.91	1.02E-01
cg11914370	57.09	15.50	2.31E-04	67.46	19.35	4.91E-04	26.15	51.89	6.14E-01	40.20	61.22	5.11E-01	43.36	34.26	2.06E-01
cg00328058	20.03	5.45	2.35E-04	17.49	7.96	2.81E-02	29.10	11.95	1.49E-02	15.37	18.55	4.08E-01	18.81	11.16	9.19E-02
cg12392528	38.54	10.49	2.39E-04	55.74	28.55	5.09E-02	49.16	108.36	6.50E-01	35.99	11.94	2.59E-03	33.17	36.15	3.59E-01
cg04153882	-22.35	6.09	2.43E-04	-26.97	8.79	2.15E-03	-18.50	17.42	2.88E-01	-24.91	14.21	7.96E-02	-11.96	13.19	3.65E-01
cg14981432	12.23	3.33	2.44E-04	8.76	5.56	1.15E-01	18.00	5.21	5.51E-04	13.29	10.35	1.99E-01	2.63	9.33	7.78E-01
cg11486133	23.65	6.45	2.47E-04	30.75	9.14	7.69E-04	22.96	18.83	2.23E-01	22.39	21.55	2.99E-01	12.31	11.88	3.00E-01
cg03806693	-27.44	7.49	2.49E-04	-17.02	16.67	3.07E-01	-30.23	77.84	6.98E-01	-26.71	8.90	2.70E-03	-59.80	26.41	2.36E-02
cg27124276	26.66	7.28	2.51E-04	36.12	13.62	8.00E-03	23.06	19.45	2.36E-01	18.06	9.14	4.80E-02	63.22	27.16	1.99E-02
cg05012767	-56.88	15.54	2.51E-04	-38.06	58.87	5.18E-01	9.20	195.51	9.62E-01	-61.28	16.57	2.17E-04	-8.93	73.51	9.03E-01
cg07059167	44.03	12.05	2.59E-04	58.28	30.89	5.92E-02	12.86	134.16	9.24E-01	43.91	13.73	1.38E-03	17.57	45.92	7.02E-01
cg09276842	-31.65	8.67	2.61E-04	-46.51	14.52	1.36E-03	-48.13	62.23	4.39E-01	-35.45	18.49	5.52E-02	-15.72	13.63	2.49E-01
cg14896003	26.46	7.26	2.67E-04	39.74	16.25	1.45E-02	23.01	10.54	2.90E-02	19.35	15.11	2.00E-01	32.99	23.48	1.60E-01
cg01311051	19.27	5.29	2.69E-04	21.05	6.85	2.12E-03	28.17	14.64	5.43E-02	19.99	19.28	3.00E-01	7.77	11.89	5.13E-01
cg23998119	15.29	4.20	2.70E-04	22.01	6.61	8.74E-04	9.58	8.00	2.31E-01	12.20	11.42	2.85E-01	11.46	9.72	2.39E-01
cg00682125	-17.39	4.78	2.71E-04	-16.76	13.22	2.05E-01	-34.77	25.14	1.67E-01	-16.23	5.46	2.96E-03	-22.35	18.17	2.19E-01
cg06423533	-71.76	19.71	2.71E-04	-146.88	58.48	1.20E-02	-144.19	254.53	5.71E-01	-65.40	22.05	3.02E-03	-24.09	69.01	7.27E-01
cg16624124	-17.21	4.73	2.72E-04	-24.51	11.40	3.16E-02	-0.82	19.30	9.66E-01	-18.70	5.80	1.25E-03	-4.91	14.73	7.39E-01
cg24951100	33.23	9.13	2.72E-04	39.17	12.63	1.93E-03	19.29	30.49	5.27E-01	52.06	35.43	1.42E-01	23.59	16.09	1.43E-01
cg05265414	-18.58	5.11	2.73E-04	-16.78	11.75	1.53E-01	-19.57	11.45	8.75E-02	-20.95	7.41	4.70E-03	-11.46	13.77	4.05E-01
cg03454225	13.31	3.66	2.77E-04	20.61	7.38	5.21E-03	4.02	9.50	6.73E-01	10.69	5.24	4.12E-02	20.68	10.71	5.36E-02
cg02326806	18.33	5.05	2.82E-04	16.36	13.79	2.36E-01	49.43	38.03	1.94E-01	14.80	5.91	1.23E-02	37.46	14.60	1.03E-02
cg08066844	53.80	14.82	2.83E-04	73.14	39.32	6.29E-02	-22.35	164.36	8.92E-01	52.50	16.82	1.81E-03	38.67	54.57	4.78E-01

cg02265690	44.06	12.14	2.84E-04	51.62	21.19	1.48E-02	-19.03	61.62	7.58E-01	33.48	16.13	3.79E-02	83.58	32.09	9.19E-03
cg20399616	25.37	6.99	2.84E-04	20.49	24.88	4.10E-01	-13.81	106.37	8.97E-01	25.90	7.69	7.61E-04	26.62	23.12	2.50E-01
cg22330924	-24.33	6.71	2.86E-04	-22.70	10.00	2.32E-02	-21.27	15.44	1.68E-01	-36.38	16.94	3.18E-02	-21.52	14.82	1.46E-01
cg05658215	22.24	6.14	2.91E-04	40.16	17.36	2.07E-02	20.45	21.81	3.48E-01	17.10	7.26	1.84E-02	41.92	21.69	5.33E-02
cg03613525	24.31	6.71	2.94E-04	19.18	11.15	8.54E-02	-6.64	46.47	8.86E-01	26.86	9.08	3.08E-03	40.22	25.46	1.14E-01
cg06698399	-14.03	3.88	2.97E-04	-12.45	6.56	5.74E-02	-22.93	8.69	8.31E-03	-13.13	7.00	6.05E-02	-7.44	10.23	4.67E-01
cg22706424	-30.29	8.38	2.99E-04	-43.18	26.39	1.02E-01	-46.33	69.31	5.04E-01	-24.87	9.38	8.00E-03	-62.39	28.42	2.81E-02
cg18815779	55.05	15.24	3.03E-04	82.80	33.02	1.22E-02	-15.72	68.87	8.19E-01	53.92	19.29	5.19E-03	39.83	45.09	3.77E-01
cg03657837	-30.96	8.59	3.13E-04	-30.68	13.50	2.31E-02	-7.57	23.45	7.47E-01	-45.85	19.73	2.02E-02	-32.53	16.47	4.83E-02
cg21131424	30.28	8.41	3.17E-04	37.77	12.27	2.09E-03	29.35	22.72	1.96E-01	15.48	14.85	2.97E-01	48.85	31.11	1.16E-01
cg04214430	-32.37	9.00	3.22E-04	-48.35	13.59	3.73E-04	-10.51	30.08	7.27E-01	-35.25	14.92	1.81E-02	-11.38	17.66	5.19E-01
cg25433490	-63.70	17.71	3.23E-04	-72.08	46.67	1.22E-01	-113.95	142.89	4.25E-01	-49.84	19.19	9.39E-03	-151.61	56.79	7.59E-03
cg24879595	44.23	12.30	3.23E-04	40.03	30.00	1.82E-01	90.59	146.78	5.37E-01	46.53	14.29	1.13E-03	28.52	42.39	5.01E-01
cg12238015	38.13	10.60	3.23E-04	41.68	14.03	2.98E-03	33.07	33.74	3.27E-01	65.39	37.26	7.93E-02	23.18	21.23	2.75E-01
cg26933422	33.70	9.38	3.28E-04	59.63	30.42	5.00E-02	44.74	103.21	6.65E-01	28.54	10.16	4.96E-03	75.78	44.81	9.09E-02
cg07621104	-48.96	13.64	3.31E-04	-22.44	44.31	6.13E-01	-232.20	233.42	3.20E-01	-52.89	15.07	4.51E-04	-33.04	47.22	4.84E-01
cg00757731	23.87	6.65	3.33E-04	33.82	23.75	1.54E-01	28.03	52.48	5.93E-01	23.33	7.29	1.37E-03	18.39	24.82	4.59E-01
cg08663092	-68.94	19.24	3.39E-04	-80.85	52.17	1.21E-01	-247.37	265.24	3.51E-01	-74.71	21.78	6.02E-04	21.39	68.81	7.56E-01
cg06394621	-10.34	2.89	3.43E-04	-9.24	5.33	8.32E-02	-8.30	7.89	2.93E-01	-11.49	4.25	6.92E-03	-10.95	8.65	2.05E-01
cg23672176	-72.37	20.24	3.50E-04	-58.26	54.29	2.83E-01	34.21	266.97	8.98E-01	-77.76	23.04	7.38E-04	-53.39	70.02	4.46E-01
cg00890840	35.56	9.95	3.52E-04	42.40	37.72	2.61E-01	4.90	148.04	9.74E-01	35.60	10.61	7.91E-04	27.48	46.50	5.55E-01
cg27579953	22.51	6.30	3.54E-04	26.90	31.89	3.99E-01	104.02	78.82	1.87E-01	21.90	6.67	1.03E-03	20.18	25.06	4.21E-01
cg00175702	25.73	7.21	3.57E-04	29.83	11.64	1.04E-02	15.57	14.78	2.92E-01	29.95	13.71	2.90E-02	22.57	22.48	3.15E-01
cg13862566	28.48	7.98	3.59E-04	16.69	14.50	2.50E-01	70.41	37.48	6.03E-02	29.62	10.36	4.26E-03	45.32	32.91	1.68E-01
cg16580937	-13.55	3.80	3.64E-04	-7.14	9.79	4.66E-01	-3.09	15.90	8.46E-01	-13.38	4.63	3.87E-03	-27.62	11.01	1.21E-02
cg18679782	39.31	11.04	3.68E-04	32.32	28.75	2.61E-01	16.76	92.53	8.56E-01	46.07	12.86	3.39E-04	3.63	34.63	9.17E-01
cg13715901	34.46	9.68	3.71E-04	24.83	13.80	7.20E-02	14.95	24.05	5.34E-01	37.79	25.12	1.33E-01	60.50	18.34	9.70E-04
cg22631350	-25.71	7.23	3.74E-04	-11.77	21.39	5.82E-01	-13.37	26.49	6.14E-01	-27.81	8.28	7.81E-04	-43.88	32.38	1.75E-01
cg06368859	13.36	3.76	3.77E-04	14.39	7.26	4.74E-02	27.24	9.42	3.83E-03	8.33	4.66	7.42E-02	15.02	10.57	1.55E-01
cg25439798	-71.86	20.21	3.77E-04	-66.66	26.60	1.22E-02	86.65	106.79	4.17E-01	-103.06	50.08	3.96E-02	-87.91	42.69	3.95E-02
cg21908673	-20.84	5.86	3.78E-04	-24.23	12.87	5.96E-02	5.03	20.96	8.10E-01	-18.99	6.07	1.77E-03	-42.57	16.88	1.17E-02
cg17481912	18.44	5.19	3.80E-04	20.10	7.11	4.72E-03	29.26	15.45	5.83E-02	23.10	16.92	1.72E-01	8.70	10.16	3.92E-01
cg05996578	43.01	12.11	3.81E-04	45.24	43.81	3.02E-01	90.21	187.99	6.31E-01	46.03	13.11	4.48E-04	-0.71	46.67	9.88E-01
cg14503489	-18.12	5.10	3.83E-04	-27.02	7.34	2.32E-04	-14.42	12.88	2.63E-01	-8.62	12.47	4.89E-01	-7.33	11.54	5.25E-01
cg17088247	-67.27	18.94	3.83E-04	-31.24	53.50	5.59E-01	234.00	223.47	2.95E-01	-74.11	21.50	5.68E-04	-82.17	62.51	1.89E-01

cg16956686	10.11	2.85	3.93E-04	9.46	4.47	3.45E-02	13.31	6.42	3.83E-02	10.58	7.13	1.38E-01	8.25	5.87	1.60E-01
cg16612352	30.76	8.69	4.00E-04	33.20	14.30	2.03E-02	18.30	34.73	5.98E-01	31.48	13.44	1.92E-02	27.95	22.40	2.12E-01
cg04986968	21.65	6.12	4.05E-04	13.97	13.31	2.94E-01	49.35	25.37	5.17E-02	24.50	7.58	1.23E-03	-2.15	22.00	9.22E-01
cg08692903	18.71	5.29	4.08E-04	16.94	8.32	4.17E-02	17.87	10.44	8.71E-02	18.32	11.85	1.22E-01	26.04	14.23	6.74E-02
cg13932501	9.75	2.76	4.13E-04	9.63	4.05	1.76E-02	14.07	6.51	3.07E-02	10.23	5.86	8.08E-02	3.62	7.53	6.31E-01
cg20332725	46.19	13.08	4.14E-04	37.32	17.18	2.99E-02	23.91	41.86	5.68E-01	63.18	44.14	1.52E-01	70.95	26.98	8.55E-03
cg14791922	28.29	8.02	4.17E-04	40.50	17.90	2.37E-02	36.47	19.07	5.59E-02	26.73	11.22	1.72E-02	0.71	23.94	9.76E-01
cg24711070	-15.42	4.37	4.18E-04	-9.93	11.37	3.82E-01	-13.66	21.09	5.17E-01	-14.78	5.12	3.87E-03	-32.20	15.41	3.67E-02
cg07374490	34.46	9.77	4.21E-04	21.53	20.19	2.86E-01	95.82	47.42	4.33E-02	32.01	12.32	9.36E-03	55.29	31.84	8.25E-02
cg17126901	28.21	8.00	4.24E-04	8.08	22.74	7.22E-01	70.59	83.51	3.98E-01	32.32	9.01	3.33E-04	13.45	28.84	6.41E-01
cg03428375	25.95	7.36	4.24E-04	26.18	16.54	1.13E-01	35.88	20.03	7.33E-02	24.19	9.53	1.12E-02	21.15	27.78	4.47E-01
cg12352399	12.12	3.44	4.26E-04	6.99	6.12	2.53E-01	12.81	6.78	5.89E-02	13.40	6.30	3.33E-02	20.51	9.65	3.35E-02
cg06446408	23.90	6.79	4.30E-04	35.82	15.31	1.93E-02	28.92	26.60	2.77E-01	18.47	8.36	2.72E-02	35.27	24.06	1.43E-01
cg04922029	16.57	4.71	4.32E-04	18.50	6.08	2.37E-03	27.31	11.94	2.21E-02	15.71	11.83	1.84E-01	-1.84	12.48	8.83E-01
cg02060566	34.75	9.89	4.39E-04	40.11	13.36	2.69E-03	21.11	33.13	5.24E-01	30.94	36.05	3.91E-01	29.80	18.41	1.05E-01
cg25645687	36.56	10.40	4.39E-04	45.05	27.38	1.00E-01	12.89	46.64	7.82E-01	37.96	12.17	1.81E-03	22.43	37.86	5.54E-01
cg00391741	13.35	3.80	4.41E-04	13.10	5.31	1.37E-02	23.58	9.08	9.41E-03	3.12	9.23	7.35E-01	13.87	10.01	1.66E-01
cg19879075	34.17	9.73	4.43E-04	12.46	20.32	5.40E-01	45.41	81.63	5.78E-01	44.71	12.00	1.95E-04	13.02	30.82	6.73E-01
cg11829616	21.63	6.16	4.48E-04	30.32	16.36	6.39E-02	27.37	22.44	2.22E-01	20.54	7.24	4.57E-03	6.66	25.53	7.94E-01
cg13752828	41.62	11.86	4.49E-04	25.57	19.08	1.80E-01	18.14	36.64	6.20E-01	47.38	21.02	2.42E-02	77.54	27.20	4.36E-03
cg05315334	13.19	3.76	4.50E-04	12.62	5.13	1.38E-02	8.16	9.96	4.13E-01	23.17	9.14	1.13E-02	8.80	9.69	3.64E-01
cg16181903	-29.97	8.55	4.59E-04	-43.32	13.27	1.09E-03	-1.20	20.91	9.54E-01	-24.15	16.69	1.48E-01	-34.91	19.61	7.51E-02
cg13357518	24.34	6.96	4.66E-04	36.07	18.00	4.51E-02	15.16	30.29	6.17E-01	27.63	8.97	2.07E-03	7.85	15.68	6.17E-01
cg10480741	-66.03	18.88	4.71E-04	-56.83	24.97	2.29E-02	-64.51	56.30	2.52E-01	-54.91	50.99	2.82E-01	-104.95	44.67	1.88E-02
cg18434848	42.31	12.10	4.71E-04	16.52	29.14	5.71E-01	-10.06	83.77	9.04E-01	49.58	14.60	6.83E-04	46.80	34.95	1.81E-01
cg18689332	19.73	5.65	4.80E-04	18.90	9.74	5.23E-02	31.42	13.59	2.08E-02	19.09	13.48	1.57E-01	14.56	10.07	1.48E-01
cg26560222	25.78	7.38	4.81E-04	31.69	16.05	4.84E-02	31.77	25.16	2.07E-01	21.59	10.14	3.33E-02	28.43	17.81	1.10E-01
cg11305134	-18.77	5.38	4.82E-04	-17.85	16.45	2.78E-01	-16.62	22.81	4.66E-01	-18.37	6.12	2.69E-03	-26.95	21.08	2.01E-01
cg00773696	42.91	12.30	4.84E-04	92.17	37.05	1.29E-02	-20.87	155.29	8.93E-01	35.11	13.51	9.35E-03	69.12	52.48	1.88E-01
cg24669728	46.76	13.40	4.84E-04	10.08	29.63	7.34E-01	57.52	133.53	6.67E-01	53.11	16.05	9.39E-04	80.42	45.09	7.45E-02
cg02277487	53.02	15.20	4.88E-04	49.18	43.76	2.61E-01	-141.16	160.31	3.79E-01	56.82	17.25	9.85E-04	44.98	49.81	3.67E-01
cg16933440	58.22	16.70	4.90E-04	64.55	23.91	6.93E-03	48.98	82.95	5.55E-01	79.25	52.24	1.29E-01	45.05	27.48	1.01E-01
cg09520904	9.67	2.77	4.91E-04	5.50	3.86	1.54E-01	18.72	7.56	1.33E-02	11.82	6.88	8.55E-02	12.86	6.45	4.62E-02
cg19022839	-18.50	5.31	4.92E-04	-28.53	11.14	1.04E-02	-46.79	27.97	9.44E-02	-11.52	6.96	9.79E-02	-23.37	13.46	8.24E-02
cg09449104	-21.51	6.17	4.93E-04	-21.35	17.11	2.12E-01	12.29	30.52	6.87E-01	-22.47	7.12	1.60E-03	-30.23	22.13	1.72E-01

cg23252587	-23.26	6.68	4.94E-04	-27.58	18.05	1.27E-01	12.24	29.39	6.77E-01	-24.71	7.68	1.30E-03	-25.78	28.08	3.58E-01
cg27598761	16.52	4.74	4.96E-04	12.58	7.61	9.84E-02	21.65	9.04	1.66E-02	26.65	12.65	3.51E-02	9.83	10.73	3.60E-01
cg03094675	23.68	6.80	4.97E-04	31.42	12.01	8.88E-03	32.17	30.66	2.94E-01	23.00	9.98	2.12E-02	8.09	16.72	6.29E-01
cg06962867	-15.74	4.52	4.99E-04	-22.35	13.60	1.00E-01	7.69	21.51	7.21E-01	-14.80	5.12	3.82E-03	-31.65	17.73	7.43E-02
cg00269615	29.19	8.39	5.00E-04	36.88	17.51	3.52E-02	39.16	38.11	3.04E-01	21.39	10.89	4.95E-02	47.55	23.32	4.15E-02
cg05135252	-21.43	6.16	5.02E-04	-19.81	13.04	1.29E-01	-13.81	21.68	5.24E-01	-23.76	7.75	2.18E-03	-13.86	24.22	5.67E-01
cg13514042	25.96	7.46	5.05E-04	17.13	11.37	1.32E-01	25.27	14.56	8.26E-02	23.89	19.14	2.12E-01	53.80	18.99	4.61E-03
cg14653390	-25.83	7.43	5.06E-04	-32.71	13.03	1.21E-02	-35.22	16.81	3.61E-02	-14.53	14.93	3.30E-01	-20.36	15.42	1.87E-01
cg22721796	-14.64	4.21	5.06E-04	-15.27	6.12	1.26E-02	-9.37	9.78	3.38E-01	-7.63	14.46	5.98E-01	-19.62	8.31	1.82E-02
cg10677324	-26.58	7.65	5.10E-04	-35.02	21.28	9.98E-02	-7.43	37.81	8.44E-01	-29.22	8.72	8.00E-04	12.31	31.15	6.93E-01
cg20238308	-9.91	2.85	5.11E-04	-12.93	5.83	2.65E-02	-2.96	9.63	7.58E-01	-10.13	3.91	9.65E-03	-8.29	7.57	2.74E-01
cg24011511	-11.92	3.43	5.12E-04	-7.78	7.38	2.91E-01	-1.34	16.30	9.35E-01	-14.52	4.40	9.61E-04	-10.23	9.45	2.79E-01
cg21781009	26.32	7.58	5.13E-04	28.60	12.55	2.27E-02	31.09	32.11	3.33E-01	13.59	11.91	2.54E-01	49.55	18.13	6.27E-03
cg06639733	21.51	6.19	5.17E-04	19.33	9.63	4.46E-02	29.02	11.65	1.27E-02	14.07	15.36	3.60E-01	21.40	16.52	1.95E-01
cg14875327	-27.33	7.87	5.18E-04	-38.62	11.69	9.49E-04	-14.38	20.45	4.82E-01	-31.24	18.02	8.30E-02	-8.23	17.30	6.34E-01
cg07000713	-10.83	3.12	5.23E-04	-14.61	6.13	1.72E-02	-1.77	10.35	8.64E-01	-9.43	4.40	3.22E-02	-14.59	8.17	7.40E-02
cg15924971	-23.60	6.81	5.26E-04	-40.31	15.30	8.40E-03	-15.96	24.78	5.19E-01	-21.18	8.63	1.40E-02	-11.77	21.16	5.78E-01
cg02633409	24.74	7.14	5.29E-04	27.67	15.33	7.11E-02	31.74	9.71	1.08E-03	8.99	21.43	6.75E-01	4.50	19.65	8.19E-01
cg18786782	15.26	4.40	5.29E-04	11.25	10.91	3.03E-01	9.79	13.28	4.61E-01	15.84	5.55	4.32E-03	24.35	14.08	8.37E-02
cg08109850	41.48	11.97	5.30E-04	0.54	45.07	9.91E-01	-25.60	210.63	9.03E-01	45.50	12.75	3.60E-04	31.74	56.43	5.74E-01
cg09067459	19.50	5.63	5.30E-04	14.95	7.39	4.31E-02	40.50	14.62	5.62E-03	27.58	18.81	1.43E-01	12.97	13.20	3.26E-01
cg16373769	47.08	13.59	5.32E-04	42.24	25.23	9.41E-02	-82.92	97.80	3.97E-01	50.21	17.87	4.95E-03	65.77	40.56	1.05E-01
cg08613350	-50.52	14.59	5.34E-04	-43.14	19.17	2.44E-02	-97.24	35.37	5.98E-03	-10.30	35.01	7.69E-01	-61.66	29.29	3.53E-02
cg03428945	-10.11	2.92	5.34E-04	-9.36	5.09	6.59E-02	-8.71	6.58	1.85E-01	-11.33	5.30	3.26E-02	-10.97	7.05	1.20E-01
cg12302110	-11.42	3.30	5.36E-04	-13.61	6.00	2.32E-02	-6.58	7.41	3.74E-01	-13.45	5.37	1.23E-02	-7.59	9.45	4.22E-01
cg06232807	18.48	5.34	5.38E-04	26.14	8.03	1.13E-03	12.62	13.09	3.35E-01	19.03	14.69	1.95E-01	8.88	10.50	3.98E-01
cg21649713	-19.32	5.58	5.42E-04	-11.19	8.70	1.98E-01	-23.62	11.50	4.00E-02	-38.47	17.04	2.39E-02	-20.43	11.28	7.01E-02
cg23746347	21.29	6.16	5.45E-04	17.68	15.45	2.53E-01	33.35	37.40	3.72E-01	24.81	7.68	1.25E-03	9.61	14.83	5.17E-01
cg26639747	-23.47	6.79	5.50E-04	-31.95	11.00	3.68E-03	1.05	17.37	9.52E-01	-25.90	14.82	8.05E-02	-23.51	13.44	8.03E-02
cg06688910	19.49	5.64	5.55E-04	24.65	7.79	1.55E-03	5.04	13.61	7.11E-01	33.96	20.28	9.39E-02	13.49	11.91	2.57E-01
cg18736711	-24.55	7.11	5.55E-04	-20.69	17.26	2.31E-01	-5.25	20.06	7.93E-01	-25.00	9.07	5.84E-03	-55.85	23.74	1.86E-02
cg09390241	-9.29	2.69	5.61E-04	-11.27	3.72	2.49E-03	-4.97	6.02	4.09E-01	-20.61	9.96	3.85E-02	-4.40	5.98	4.61E-01
cg02858288	27.64	8.01	5.63E-04	32.17	16.59	5.24E-02	0.17	18.62	9.93E-01	37.14	11.80	1.65E-03	24.72	23.11	2.85E-01
cg19345662	-10.40	3.02	5.70E-04	-7.71	4.27	7.13E-02	-14.72	7.00	3.54E-02	-4.42	9.50	6.42E-01	-15.69	6.51	1.59E-02
cg04103645	-22.16	6.43	5.74E-04	-27.54	10.21	6.97E-03	-8.75	16.43	5.94E-01	-22.01	12.19	7.10E-02	-21.91	15.57	1.59E-01

cg03398461	21.05	6.11	5.74E-04	25.59	10.73	1.71E-02	32.40	16.50	4.96E-02	16.32	9.24	7.72E-02	11.46	19.34	5.54E-01
cg08969304	17.83	5.19	5.84E-04	17.84	9.06	4.90E-02	9.84	20.89	6.38E-01	19.58	8.29	1.81E-02	16.94	11.07	1.26E-01
cg22795586	23.11	6.72	5.88E-04	20.54	14.53	1.57E-01	32.77	26.06	2.09E-01	25.92	9.76	7.91E-03	17.28	13.59	2.04E-01
cg07593845	-29.28	8.53	5.94E-04	-27.16	16.78	1.06E-01	23.09	32.22	4.74E-01	-36.06	11.08	1.14E-03	-32.39	25.50	2.04E-01
cg10698097	-31.00	9.03	5.97E-04	-30.51	13.76	2.67E-02	-36.65	31.34	2.42E-01	-60.87	22.47	6.75E-03	-15.37	15.84	3.32E-01
cg07842862	21.07	6.14	5.98E-04	18.15	8.87	4.07E-02	16.03	17.65	3.64E-01	59.83	27.13	2.74E-02	21.13	10.39	4.20E-02
cg08172575	-23.53	6.86	6.06E-04	-30.44	18.77	1.05E-01	-31.27	27.38	2.53E-01	-22.29	8.02	5.46E-03	-16.51	25.56	5.18E-01
cg07895499	-21.96	6.41	6.11E-04	-37.29	15.62	1.70E-02	-17.12	27.23	5.29E-01	-17.30	7.80	2.66E-02	-30.12	20.09	1.34E-01
cg02367144	19.87	5.80	6.12E-04	16.34	8.68	5.97E-02	33.51	16.69	4.46E-02	25.26	18.18	1.65E-01	17.99	10.08	7.43E-02
cg04601957	31.50	9.20	6.14E-04	28.81	11.30	1.08E-02	16.53	40.77	6.85E-01	58.16	52.18	2.65E-01	38.21	18.17	3.55E-02
cg07794169	-18.86	5.51	6.16E-04	-21.61	10.16	3.35E-02	-0.71	18.37	9.69E-01	-19.10	8.23	2.04E-02	-23.08	13.38	8.46E-02
cg04358516	20.03	5.85	6.17E-04	19.69	15.80	2.13E-01	41.33	19.72	3.61E-02	18.93	7.07	7.37E-03	7.99	19.52	6.82E-01
cg24248713	26.26	7.67	6.18E-04	34.51	11.06	1.80E-03	7.22	33.18	8.28E-01	36.59	24.79	1.40E-01	15.60	12.61	2.16E-01
cg00910695	25.94	7.58	6.18E-04	37.41	24.48	1.26E-01	33.28	35.09	3.43E-01	24.39	9.39	9.36E-03	23.85	16.71	1.53E-01
cg01213231	18.28	5.34	6.19E-04	14.53	7.04	3.88E-02	27.56	12.73	3.04E-02	27.92	17.81	1.17E-01	16.12	13.43	2.30E-01
cg20112110	23.55	6.88	6.19E-04	17.17	15.04	2.54E-01	20.53	27.16	4.50E-01	23.90	8.77	6.41E-03	35.44	20.68	8.66E-02
cg12041056	47.33	13.83	6.21E-04	44.95	33.22	1.76E-01	84.88	96.27	3.78E-01	48.49	16.66	3.61E-03	37.41	40.47	3.55E-01
cg25307332	44.95	13.15	6.27E-04	63.97	17.97	3.70E-04	15.91	41.32	7.00E-01	62.01	30.57	4.25E-02	18.61	21.76	3.92E-01
cg09890980	-10.48	3.07	6.39E-04	-4.79	5.78	4.07E-01	-14.91	11.18	1.82E-01	-14.61	4.52	1.24E-03	-7.02	7.19	3.29E-01
cg00308159	34.19	10.02	6.43E-04	47.22	13.87	6.60E-04	9.70	25.68	7.06E-01	31.54	39.61	4.26E-01	23.09	19.57	2.38E-01
cg02110782	47.11	13.81	6.44E-04	26.17	44.26	5.54E-01	-193.88	177.60	2.75E-01	50.40	15.07	8.27E-04	59.83	57.44	2.98E-01
cg14583869	43.65	12.80	6.52E-04	50.30	50.21	3.16E-01	95.93	170.94	5.75E-01	45.39	13.65	8.86E-04	-1.53	57.27	9.79E-01
cg18259253	-17.55	5.15	6.55E-04	-24.92	12.05	3.87E-02	-22.54	17.44	1.96E-01	-15.97	6.71	1.73E-02	-11.55	13.73	4.00E-01
cg16953067	-73.78	21.67	6.62E-04	-49.91	45.40	2.72E-01	61.70	271.45	8.20E-01	-90.03	28.44	1.55E-03	-56.86	50.37	2.59E-01
cg07563882	33.12	9.73	6.65E-04	38.79	19.23	4.37E-02	41.41	21.41	5.31E-02	17.64	14.78	2.33E-01	67.25	30.18	2.59E-02
cg01869273	53.66	15.79	6.77E-04	66.53	21.13	1.64E-03	24.29	61.07	6.91E-01	9.29	53.35	8.62E-01	49.00	29.44	9.61E-02
cg20485058	-17.17	5.05	6.79E-04	-10.18	14.71	4.89E-01	3.47	17.77	8.45E-01	-22.12	5.91	1.83E-04	-1.25	19.05	9.48E-01
cg13618906	35.06	10.32	6.82E-04	2.38	23.91	9.21E-01	8.72	81.31	9.15E-01	45.78	10.19	7.08E-06	20.99	30.72	4.94E-01
cg18772573	19.00	5.60	6.92E-04	16.67	8.44	4.81E-02	22.53	10.65	3.43E-02	13.49	15.21	3.75E-01	24.42	14.60	9.45E-02
cg02341038	65.07	19.18	6.92E-04	110.28	53.46	3.91E-02	2.91	267.90	9.91E-01	63.77	21.93	3.64E-03	20.64	60.21	7.32E-01
cg11072779	26.87	7.92	6.92E-04	8.50	21.51	6.93E-01	-5.69	40.39	8.88E-01	29.71	9.06	1.04E-03	52.44	31.94	1.01E-01
cg09495977	26.81	7.91	7.00E-04	24.91	17.41	1.53E-01	13.44	24.84	5.88E-01	20.47	12.00	8.80E-02	44.27	15.58	4.49E-03
cg09209257	-14.15	4.18	7.04E-04	-16.59	6.08	6.36E-03	-3.73	10.03	7.10E-01	-19.53	10.49	6.27E-02	-13.16	9.44	1.63E-01
cg14370507	-25.02	7.39	7.11E-04	-39.26	13.14	2.81E-03	-57.24	36.99	1.22E-01	-19.51	8.84	2.74E-02	-3.78	22.51	8.67E-01
cg02835467	26.40	7.80	7.12E-04	30.50	10.79	4.71E-03	44.66	38.11	2.41E-01	48.20	28.40	8.96E-02	13.77	13.00	2.89E-01

cg01906801	23.50	6.94	7.13E-04	27.96	10.33	6.80E-03	17.05	13.27	1.99E-01	42.00	20.34	3.90E-02	8.30	17.48	6.35E-01
cg26751588	-12.97	3.83	7.13E-04	-19.43	5.86	9.07E-04	-9.97	8.84	2.59E-01	-8.09	10.97	4.61E-01	-6.84	7.48	3.61E-01
cg04456754	-15.33	4.53	7.15E-04	-25.79	9.28	5.44E-03	-4.26	11.60	7.13E-01	-14.40	6.97	3.89E-02	-13.11	10.47	2.11E-01
cg07162905	25.20	7.45	7.15E-04	18.59	19.18	3.33E-01	31.43	21.77	1.49E-01	26.15	9.31	4.94E-03	21.45	24.58	3.83E-01
cg14615833	14.78	4.37	7.16E-04	21.13	10.85	5.15E-02	14.09	14.27	3.24E-01	15.44	5.43	4.46E-03	0.34	14.10	9.80E-01
cg02026535	29.74	8.79	7.20E-04	30.22	21.80	1.66E-01	51.92	34.36	1.31E-01	28.33	10.50	6.98E-03	22.06	33.14	5.05E-01
cg02110858	10.32	3.05	7.21E-04	10.31	4.55	2.36E-02	14.98	5.71	8.68E-03	5.82	10.93	5.94E-01	5.09	7.06	4.71E-01
cg01808968	-21.78	6.45	7.28E-04	-24.06	13.53	7.54E-02	-23.61	16.69	1.57E-01	-17.54	9.76	7.22E-02	-27.41	14.88	6.55E-02
cg14835423	48.40	14.33	7.30E-04	-7.17	42.75	8.67E-01	-39.72	158.63	8.02E-01	55.98	15.86	4.16E-04	60.72	56.99	2.87E-01
cg26385062	19.22	5.69	7.31E-04	15.14	7.83	5.31E-02	31.04	12.33	1.18E-02	31.19	18.36	8.93E-02	9.93	14.13	4.82E-01
cg06747322	-38.74	11.47	7.32E-04	-78.01	39.29	4.71E-02	-105.56	156.37	5.00E-01	-32.84	12.58	9.07E-03	-54.09	41.00	1.87E-01
cg05269040	-15.96	4.73	7.35E-04	-14.92	6.63	2.44E-02	-29.15	12.30	1.78E-02	-7.85	13.17	5.51E-01	-14.22	10.21	1.64E-01
cg06632214	13.56	4.02	7.37E-04	9.00	9.95	3.66E-01	16.25	18.16	3.71E-01	15.62	4.72	9.25E-04	-0.67	16.16	9.67E-01
cg24846343	16.85	5.00	7.45E-04	22.38	8.82	1.12E-02	17.76	10.66	9.59E-02	20.45	9.82	3.73E-02	2.39	11.14	8.30E-01
cg10559742	12.51	3.71	7.46E-04	14.72	5.88	1.23E-02	12.07	8.98	1.79E-01	8.89	7.28	2.22E-01	13.32	8.97	1.37E-01
cg24172553	25.35	7.52	7.46E-04	10.78	23.86	6.52E-01	-30.55	48.37	5.28E-01	27.96	8.32	7.73E-04	36.44	30.78	2.37E-01
cg11434976	46.56	13.81	7.49E-04	44.08	16.43	7.30E-03	96.22	93.45	3.03E-01	87.72	62.89	1.63E-01	40.67	29.24	1.64E-01
cg04638766	-12.24	3.63	7.50E-04	-14.93	5.07	3.21E-03	-8.78	8.22	2.86E-01	-12.04	11.81	3.08E-01	-8.75	8.20	2.86E-01
cg10319399	26.88	7.98	7.51E-04	22.78	24.41	3.51E-01	35.06	85.20	6.81E-01	26.47	8.83	2.71E-03	37.22	30.60	2.24E-01
cg17833902	-25.78	7.65	7.52E-04	-39.61	18.28	3.03E-02	19.48	52.28	7.09E-01	-23.79	8.95	7.84E-03	-25.86	28.48	3.64E-01
cg24037166	35.65	10.58	7.53E-04	43.20	23.91	7.08E-02	46.40	68.83	5.00E-01	31.73	12.63	1.20E-02	48.68	37.75	1.97E-01
cg01400040	-11.51	3.42	7.58E-04	-12.61	7.57	9.61E-02	-10.61	17.26	5.39E-01	-10.00	4.28	1.93E-02	-18.08	9.95	6.91E-02
cg02462818	16.19	4.81	7.66E-04	16.26	6.64	1.44E-02	19.56	9.56	4.07E-02	17.07	28.31	5.47E-01	11.46	10.96	2.96E-01
cg02109605	27.91	8.29	7.66E-04	39.94	19.23	3.78E-02	33.36	19.65	8.96E-02	22.15	11.13	4.67E-02	27.78	29.18	3.41E-01
cg02589576	-13.77	4.10	7.72E-04	-17.86	5.75	1.90E-03	-6.30	9.24	4.95E-01	-4.98	14.89	7.38E-01	-14.03	8.72	1.07E-01
cg20759823	-25.43	7.56	7.73E-04	-15.65	16.58	3.45E-01	-40.06	33.91	2.37E-01	-28.83	9.54	2.52E-03	-18.19	22.42	4.17E-01
cg14687660	-18.23	5.42	7.74E-04	-12.48	7.63	1.02E-01	-32.39	12.91	1.21E-02	0.61	19.95	9.75E-01	-24.71	10.42	1.77E-02
cg03853987	23.17	6.90	7.83E-04	18.81	9.07	3.80E-02	38.53	18.84	4.08E-02	30.79	35.92	3.91E-01	23.91	13.80	8.32E-02
cg13528199	-74.40	22.16	7.87E-04	-61.43	35.31	8.19E-02	-39.53	199.57	8.43E-01	-41.45	48.25	3.90E-01	-107.01	35.81	2.81E-03
cg08572513	-10.06	3.00	7.88E-04	-10.45	4.27	1.44E-02	-0.75	6.74	9.12E-01	-15.88	9.25	8.63E-02	-15.11	6.61	2.23E-02
cg23327992	60.28	17.96	7.90E-04	36.27	40.41	3.69E-01	-42.79	197.77	8.29E-01	69.44	21.57	1.28E-03	52.72	56.55	3.51E-01
cg22439651	-11.13	3.32	7.90E-04	-14.03	7.29	5.43E-02	-12.68	12.47	3.09E-01	-7.89	4.36	7.05E-02	-19.13	8.71	2.81E-02
cg05434952	58.69	17.49	7.91E-04	41.30	46.74	3.77E-01	-108.34	191.79	5.72E-01	68.08	19.90	6.22E-04	15.23	62.27	8.07E-01
cg10325659	-40.56	12.09	7.93E-04	-37.41	16.67	2.48E-02	-39.96	51.69	4.39E-01	-45.68	25.08	6.85E-02	-43.23	27.95	1.22E-01
cg23689052	30.72	9.16	7.96E-04	29.49	15.58	5.84E-02	17.62	34.76	6.12E-01	32.49	14.77	2.78E-02	33.98	20.44	9.65E-02

cg10969919	-17.12	5.11	8.01E-04	-16.27	10.06	1.06E-01	-4.59	15.63	7.69E-01	-22.12	7.39	2.77E-03	-11.90	12.86	3.55E-01
cg09876101	91.87	27.42	8.06E-04	143.97	78.22	6.57E-02	322.09	336.79	3.39E-01	94.75	31.09	2.31E-03	-17.45	89.89	8.46E-01
cg25820728	-15.30	4.57	8.06E-04	-15.73	6.73	1.94E-02	-4.88	14.79	7.41E-01	-26.34	12.74	3.88E-02	-13.33	8.13	1.01E-01
cg08286785	29.69	8.87	8.12E-04	27.16	15.52	8.01E-02	27.19	16.79	1.05E-01	21.11	17.57	2.30E-01	56.10	23.66	1.77E-02
cg07423050	-12.07	3.60	8.12E-04	-14.88	5.45	6.39E-03	-18.86	8.95	3.50E-02	-3.86	9.93	6.97E-01	-7.44	6.94	2.84E-01
cg00341935	25.80	7.71	8.17E-04	25.78	19.30	1.82E-01	41.29	32.30	2.01E-01	24.51	9.09	7.00E-03	26.59	30.46	3.83E-01
cg22597040	27.74	8.30	8.24E-04	48.78	13.72	3.79E-04	20.90	19.85	2.92E-01	13.15	18.11	4.68E-01	20.33	12.68	1.09E-01
cg06845943	-70.82	21.18	8.26E-04	-78.70	51.07	1.23E-01	-410.50	197.76	3.79E-02	-62.06	21.34	3.64E-03	-83.25	66.42	2.10E-01
cg21635265	26.39	7.90	8.30E-04	40.10	15.12	8.00E-03	22.99	15.42	1.36E-01	16.81	13.59	2.16E-01	29.43	22.11	1.83E-01
cg04830825	23.45	7.02	8.35E-04	21.57	8.81	1.43E-02	13.04	21.48	5.44E-01	41.53	31.93	1.93E-01	30.26	15.32	4.81E-02
cg05290058	8.22	2.46	8.35E-04	6.06	4.08	1.37E-01	12.18	5.39	2.39E-02	11.41	5.53	3.90E-02	5.29	5.14	3.03E-01
cg04753786	35.65	10.67	8.35E-04	37.56	13.86	6.71E-03	21.92	34.24	5.22E-01	79.16	65.98	2.30E-01	32.34	20.04	1.07E-01
cg22949256	-13.02	3.90	8.39E-04	-20.82	8.68	1.65E-02	-1.16	12.80	9.28E-01	-11.15	5.13	2.98E-02	-17.77	10.90	1.03E-01
cg14992768	-14.61	4.37	8.40E-04	-21.08	11.15	5.86E-02	-13.30	18.20	4.65E-01	-12.50	5.26	1.75E-02	-20.10	14.03	1.52E-01
cg02072896	-55.01	16.48	8.43E-04	-65.29	19.21	6.78E-04	-2.22	40.46	9.56E-01	-93.04	32.74	4.48E-03	-31.88	32.70	3.30E-01
cg18817459	-11.71	3.51	8.43E-04	-7.15	5.99	2.33E-01	-8.99	9.19	3.28E-01	-17.95	5.63	1.42E-03	-7.90	10.01	4.30E-01
cg13134448	36.01	10.79	8.47E-04	33.43	13.27	1.17E-02	9.00	41.95	8.30E-01	-0.37	57.48	9.95E-01	56.13	22.16	1.13E-02
cg03660085	-11.57	3.47	8.49E-04	-19.25	6.75	4.33E-03	-5.59	10.11	5.81E-01	-8.40	5.34	1.16E-01	-11.63	7.83	1.37E-01
cg26572992	-19.73	5.92	8.54E-04	-22.67	8.84	1.03E-02	-12.33	14.63	3.99E-01	-22.46	14.34	1.17E-01	-17.09	12.68	1.78E-01
cg02198144	-16.38	4.91	8.58E-04	-23.44	7.71	2.35E-03	-5.13	14.54	7.24E-01	-22.65	11.77	5.44E-02	-7.60	8.90	3.94E-01
cg14723032	21.21	6.36	8.58E-04	19.62	11.72	9.40E-02	16.13	13.96	2.48E-01	22.50	10.41	3.07E-02	29.68	18.11	1.01E-01
cg06928741	11.77	3.53	8.59E-04	10.74	4.41	1.50E-02	13.86	8.64	1.09E-01	27.00	14.30	5.90E-02	7.06	9.74	4.68E-01
cg07593852	-14.00	4.20	8.64E-04	-14.83	8.81	9.21E-02	-0.25	14.72	9.87E-01	-14.30	5.53	9.72E-03	-20.63	12.46	9.76E-02
cg17039391	29.45	8.84	8.66E-04	4.40	32.47	8.92E-01	-63.61	147.27	6.66E-01	31.74	9.48	8.13E-04	33.30	38.69	3.89E-01
cg16814786	9.42	2.83	8.69E-04	8.92	3.64	1.44E-02	16.65	6.51	1.05E-02	4.80	11.21	6.68E-01	4.08	7.48	5.85E-01
cg09104284	-34.06	10.23	8.72E-04	-25.54	18.81	1.74E-01	-67.19	37.95	7.67E-02	-26.40	17.20	1.25E-01	-44.26	19.43	2.28E-02
cg22635088	-14.91	4.48	8.73E-04	-17.56	6.90	1.09E-02	-4.09	10.95	7.09E-01	-20.54	10.47	4.98E-02	-13.43	9.37	1.52E-01
cg03974423	73.87	22.19	8.74E-04	96.89	30.87	1.70E-03	47.40	72.05	5.11E-01	45.61	57.47	4.27E-01	52.26	45.39	2.50E-01
cg06691616	-14.33	4.31	8.75E-04	-18.46	10.60	8.15E-02	-40.59	17.07	1.74E-02	-11.99	5.26	2.28E-02	-7.48	12.50	5.50E-01
cg26658743	-8.47	2.54	8.76E-04	-7.70	4.52	8.82E-02	-7.56	5.85	1.97E-01	-10.90	4.12	8.09E-03	-3.85	7.62	6.13E-01
cg09315878	-10.07	3.03	8.80E-04	-10.08	4.05	1.28E-02	-7.71	6.78	2.56E-01	-11.24	10.50	2.84E-01	-12.37	7.59	1.03E-01
cg00856157	26.57	7.99	8.83E-04	22.24	10.91	4.15E-02	6.88	30.13	8.19E-01	29.08	18.37	1.13E-01	42.38	17.68	1.65E-02
cg07856521	26.39	7.94	8.88E-04	35.13	11.63	2.54E-03	28.76	21.34	1.78E-01	37.11	23.72	1.18E-01	6.67	14.91	6.55E-01
cg07479864	37.37	11.25	8.95E-04	36.83	34.80	2.90E-01	-16.80	148.17	9.10E-01	41.91	12.39	7.18E-04	-14.47	44.09	7.43E-01
cg04705952	22.52	6.78	8.96E-04	18.85	9.74	5.30E-02	26.79	14.23	5.98E-02	27.33	18.66	1.43E-01	23.64	17.13	1.68E-01

cg24048759	46.14	13.89	8.96E-04	30.84	38.60	4.24E-01	28.96	197.02	8.83E-01	48.33	15.55	1.88E-03	50.90	53.63	3.43E-01
cg04172115	29.05	8.75	8.97E-04	8.56	13.45	5.24E-01	36.31	15.83	2.18E-02	40.69	17.60	2.08E-02	43.14	19.38	2.60E-02
cg18404374	14.44	4.35	8.98E-04	17.98	6.28	4.22E-03	6.05	10.59	5.68E-01	24.54	13.77	7.47E-02	9.34	8.64	2.79E-01
cg23261171	-12.74	3.84	9.02E-04	-11.29	8.03	1.60E-01	-28.31	12.15	1.98E-02	-11.98	5.34	2.50E-02	-7.43	9.74	4.45E-01
cg14034270	20.00	6.03	9.08E-04	18.73	9.04	3.83E-02	18.73	13.11	1.53E-01	12.77	13.92	3.59E-01	34.05	15.26	2.57E-02
cg07995570	44.85	13.52	9.10E-04	44.92	19.88	2.38E-02	14.77	53.60	7.83E-01	55.73	28.78	5.28E-02	42.80	26.89	1.11E-01
cg08113895	-12.10	3.65	9.11E-04	-13.10	6.23	3.54E-02	-8.98	7.57	2.35E-01	-15.15	7.75	5.07E-02	-10.63	8.08	1.88E-01
cg21446692	-35.64	10.75	9.11E-04	-39.17	35.39	2.68E-01	-72.70	118.98	5.41E-01	-35.21	11.76	2.75E-03	-31.53	42.32	4.56E-01
cg04652932	34.49	10.41	9.20E-04	35.44	14.82	1.68E-02	33.10	22.13	1.35E-01	28.98	37.48	4.39E-01	35.75	22.80	1.17E-01
cg12061886	14.16	4.28	9.25E-04	9.44	5.92	1.11E-01	23.59	9.22	1.05E-02	3.83	13.53	7.77E-01	23.15	10.57	2.85E-02
cg26939365	19.77	5.97	9.27E-04	16.94	15.21	2.65E-01	33.29	25.87	1.98E-01	17.41	6.96	1.23E-02	45.27	25.03	7.06E-02
cg18596952	-29.43	8.89	9.28E-04	-13.28	33.24	6.89E-01	90.46	145.61	5.34E-01	-30.06	9.44	1.45E-03	-56.47	45.22	2.12E-01
cg10386914	46.46	14.03	9.32E-04	36.52	25.45	1.51E-01	-2.02	72.48	9.78E-01	62.79	20.13	1.81E-03	28.47	33.80	4.00E-01
cg00371368	24.22	7.32	9.42E-04	56.55	31.15	6.95E-02	31.76	110.66	7.74E-01	22.91	7.78	3.22E-03	12.00	31.66	7.05E-01
cg26074618	46.29	14.00	9.48E-04	48.44	17.99	7.09E-03	17.50	49.72	7.25E-01	46.58	48.36	3.35E-01	50.44	29.15	8.36E-02
cg19757631	-15.96	4.83	9.50E-04	-21.97	7.92	5.55E-03	0.81	11.93	9.46E-01	-15.60	10.91	1.53E-01	-18.13	9.32	5.16E-02
cg00625351	-21.16	6.41	9.57E-04	-32.30	12.00	7.11E-03	-6.02	18.02	7.38E-01	-15.40	10.04	1.25E-01	-27.09	15.01	7.12E-02
cg22900607	7.59	2.30	9.58E-04	6.78	2.83	1.66E-02	1.68	5.35	7.54E-01	14.60	9.27	1.15E-01	13.46	5.23	1.00E-02
cg16559727	27.60	8.37	9.70E-04	15.77	16.81	3.48E-01	3.83	42.76	9.29E-01	36.90	10.66	5.35E-04	8.22	26.79	7.59E-01
cg08376828	23.39	7.09	9.71E-04	28.61	9.82	3.58E-03	7.65	20.06	7.03E-01	-8.22	36.31	8.21E-01	24.80	12.61	4.92E-02
cg03216261	33.44	10.14	9.74E-04	34.05	35.19	3.33E-01	15.56	167.89	9.26E-01	35.80	10.89	1.01E-03	-10.27	47.03	8.27E-01
cg19761682	-59.63	18.08	9.76E-04	-63.49	25.70	1.35E-02	-7.91	85.32	9.26E-01	-34.02	40.23	3.98E-01	-81.29	35.60	2.24E-02
cg11809107	-12.45	3.78	9.79E-04	-14.44	6.10	1.80E-02	-14.18	9.12	1.20E-01	-8.49	7.02	2.27E-01	-13.01	9.57	1.74E-01
cg10582499	42.40	12.86	9.81E-04	41.52	16.96	1.44E-02	40.10	41.84	3.38E-01	8.93	43.65	8.38E-01	57.29	26.06	2.79E-02
cg09736194	15.66	4.75	9.84E-04	14.70	12.79	2.51E-01	-0.78	20.13	9.69E-01	17.76	5.49	1.23E-03	6.71	19.68	7.33E-01
cg20988098	-8.44	2.56	9.88E-04	-9.40	4.12	2.24E-02	-5.92	4.80	2.18E-01	-10.01	6.38	1.16E-01	-9.01	6.28	1.52E-01
cg09105442	-11.92	3.62	9.89E-04	-13.39	5.12	8.93E-03	0.43	8.78	9.61E-01	-22.11	9.82	2.43E-02	-11.99	7.11	9.18E-02
cg23994702	-27.72	8.42	9.94E-04	-50.53	23.61	3.23E-02	-173.36	125.80	1.68E-01	-23.37	9.40	1.29E-02	-26.78	32.90	4.16E-01
cg21033632	-11.09	3.37	1.01E-03	-15.24	7.85	5.22E-02	2.06	16.88	9.03E-01	-9.84	4.13	1.73E-02	-16.48	10.19	1.06E-01
cg10501828	47.55	14.47	1.01E-03	57.19	17.61	1.17E-03	42.71	65.21	5.13E-01	51.36	81.93	5.31E-01	21.48	29.22	4.62E-01
cg25739943	-23.71	7.21	1.01E-03	-23.01	19.24	2.32E-01	4.26	29.55	8.85E-01	-29.79	8.60	5.33E-04	2.38	23.24	9.19E-01
cg01186551	26.11	7.95	1.02E-03	-2.27	23.07	9.22E-01	22.19	38.13	5.61E-01	29.52	8.99	1.02E-03	41.65	33.57	2.15E-01
cg07568344	46.18	14.06	1.02E-03	37.33	19.69	5.79E-02	26.26	41.00	5.22E-01	86.82	40.51	3.21E-02	53.95	27.99	5.39E-02
cg05916684	11.86	3.61	1.02E-03	6.21	5.47	2.56E-01	19.68	6.73	3.45E-03	10.04	9.86	3.09E-01	15.08	9.60	1.16E-01
cg02403541	42.50	12.94	1.02E-03	10.96	34.46	7.50E-01	-71.48	145.55	6.23E-01	48.77	14.71	9.15E-04	49.01	46.74	2.94E-01

cg02773337	90.05	27.43	1.03E-03	91.76	36.62	1.22E-02	264.55	135.20	5.04E-02	80.96	63.55	2.03E-01	59.57	59.62	3.18E-01
cg21596825	-20.71	6.31	1.03E-03	-39.33	17.48	2.45E-02	-2.23	32.37	9.45E-01	-17.97	7.16	1.21E-02	-28.18	26.97	2.96E-01
cg09473613	-10.68	3.26	1.03E-03	-12.70	6.25	4.22E-02	-13.62	9.75	1.62E-01	-8.00	4.89	1.02E-01	-12.49	7.81	1.10E-01
cg00596687	55.15	16.81	1.04E-03	56.06	27.42	4.09E-02	64.82	30.17	3.17E-02	14.60	45.84	7.50E-01	66.90	39.72	9.21E-02
cg23351479	-17.91	5.46	1.04E-03	-27.64	13.27	3.73E-02	-13.21	35.93	7.13E-01	-16.14	6.40	1.17E-02	-14.84	19.22	4.40E-01
cg16345311	-8.99	2.74	1.04E-03	-12.67	4.95	1.04E-02	2.55	6.81	7.08E-01	-10.70	3.40	1.65E-03	-9.02	6.19	1.45E-01
cg13892474	33.83	10.32	1.04E-03	32.44	23.78	1.73E-01	-37.46	55.58	5.00E-01	35.79	12.63	4.60E-03	46.68	31.14	1.34E-01
cg15922305	18.44	5.63	1.05E-03	24.13	7.37	1.06E-03	19.53	13.90	1.60E-01	-4.98	19.45	7.98E-01	9.33	13.70	4.96E-01
cg19559114	38.02	11.61	1.06E-03	39.57	20.45	5.30E-02	25.54	23.14	2.70E-01	24.57	15.27	1.08E-01	82.11	26.36	1.84E-03
cg02798801	-12.92	3.95	1.06E-03	-14.16	5.49	9.91E-03	-7.53	8.42	3.71E-01	-3.80	18.87	8.40E-01	-17.21	8.41	4.07E-02
cg04299808	23.61	7.21	1.06E-03	27.53	11.00	1.23E-02	34.63	13.68	1.14E-02	1.37	19.36	9.44E-01	12.86	18.38	4.84E-01
cg18064219	-68.75	21.00	1.06E-03	-74.82	26.03	4.04E-03	-61.12	119.34	6.09E-01	-11.33	74.70	8.79E-01	-72.18	42.98	9.31E-02
cg24629941	20.57	6.29	1.07E-03	10.84	14.66	4.60E-01	31.06	20.18	1.24E-01	20.52	7.79	8.46E-03	32.47	24.21	1.80E-01
cg12042264	17.53	5.36	1.07E-03	16.73	7.34	2.26E-02	19.39	12.86	1.32E-01	45.03	22.23	4.28E-02	11.16	11.08	3.14E-01
cg18101249	56.15	17.17	1.08E-03	76.03	40.08	5.79E-02	13.74	199.95	9.45E-01	52.87	19.91	7.93E-03	42.45	67.10	5.27E-01
cg21160429	-16.41	5.02	1.08E-03	-23.30	9.89	1.85E-02	-4.53	10.88	6.77E-01	-16.43	8.33	4.84E-02	-20.94	12.33	8.95E-02
cg14518185	-19.18	5.87	1.08E-03	-17.70	17.57	3.14E-01	-20.65	34.38	5.48E-01	-19.95	6.61	2.53E-03	-12.35	22.08	5.76E-01
cg09773238	-20.00	6.12	1.08E-03	-27.00	15.92	8.98E-02	2.20	17.98	9.02E-01	-18.77	5.91	1.49E-03	-44.73	19.64	2.27E-02
cg25629930	29.45	9.01	1.09E-03	19.49	12.36	1.15E-01	29.24	34.19	3.92E-01	24.91	32.98	4.50E-01	46.91	15.84	3.07E-03
cg06733568	39.11	11.97	1.09E-03	39.71	16.39	1.54E-02	65.81	25.16	8.91E-03	-7.15	32.20	8.24E-01	40.11	27.16	1.40E-01
cg03180953	-63.03	19.29	1.09E-03	-66.29	25.56	9.49E-03	27.41	82.37	7.39E-01	-109.18	41.24	8.11E-03	-29.85	42.59	4.83E-01
cg22534724	-13.30	4.07	1.09E-03	-22.15	8.28	7.51E-03	-10.21	12.77	4.24E-01	-9.52	5.66	9.28E-02	-14.24	10.90	1.91E-01
cg11901043	17.22	5.28	1.10E-03	22.37	15.78	1.56E-01	-4.80	46.29	9.17E-01	17.00	5.86	3.73E-03	15.52	20.68	4.53E-01
cg03485840	-15.06	4.62	1.10E-03	-14.76	13.82	2.86E-01	-11.14	22.17	6.15E-01	-14.48	5.24	5.72E-03	-24.53	17.52	1.62E-01
cg15806103	-22.14	6.78	1.10E-03	-1.55	19.39	9.36E-01	-18.68	23.11	4.19E-01	-28.14	8.00	4.34E-04	-1.26	25.34	9.60E-01
cg00003965	12.01	3.68	1.10E-03	18.10	7.37	1.41E-02	13.28	10.37	2.00E-01	7.64	5.08	1.33E-01	18.31	11.72	1.18E-01
cg10187221	-37.48	11.49	1.11E-03	-66.27	39.00	8.93E-02	83.41	194.25	6.68E-01	-37.66	12.45	2.48E-03	1.73	48.13	9.71E-01
cg26727666	-15.10	4.63	1.11E-03	-22.20	12.60	7.81E-02	-39.38	18.61	3.43E-02	-11.16	5.45	4.08E-02	-19.73	16.20	2.23E-01
cg16878088	54.93	16.85	1.11E-03	51.80	19.85	9.06E-03	93.37	104.96	3.74E-01	118.72	69.19	8.62E-02	41.96	38.23	2.72E-01
cg07934552	13.06	4.01	1.12E-03	18.43	5.68	1.17E-03	11.30	10.42	2.78E-01	5.60	11.69	6.32E-01	6.56	8.24	4.26E-01
cg10909939	5.93	1.82	1.12E-03	7.46	2.38	1.75E-03	5.89	4.37	1.77E-01	-3.59	6.84	5.99E-01	4.71	4.37	2.80E-01
cg07260849	-11.56	3.55	1.12E-03	-17.30	6.51	7.86E-03	2.52	12.35	8.38E-01	-10.56	5.24	4.37E-02	-11.01	8.80	2.11E-01
cg04520704	-14.78	4.54	1.12E-03	-21.27	6.37	8.48E-04	-16.39	9.68	9.03E-02	3.75	15.07	8.04E-01	-7.97	8.96	3.74E-01
cg04362790	-20.73	6.37	1.13E-03	-27.57	9.93	5.49E-03	-2.02	12.71	8.74E-01	-35.45	15.15	1.93E-02	-19.10	10.19	6.07E-02
cg23877497	-21.29	6.54	1.13E-03	-26.17	13.87	5.91E-02	-15.82	19.96	4.28E-01	-24.83	9.01	5.87E-03	-4.94	17.21	7.74E-01

cg21502255	28.25	8.68	1.13E-03	51.94	25.68	4.31E-02	82.68	59.01	1.61E-01	24.74	10.51	1.86E-02	20.12	20.30	3.22E-01
cg20114113	-23.32	7.17	1.13E-03	-15.79	15.23	3.00E-01	-13.13	18.50	4.78E-01	-27.60	9.89	5.26E-03	-32.52	22.25	1.44E-01
cg26099018	-13.14	4.04	1.13E-03	-11.93	9.89	2.28E-01	-11.05	11.83	3.51E-01	-10.09	5.27	5.56E-02	-30.46	11.22	6.64E-03
cg01802898	-21.41	6.58	1.14E-03	-31.73	12.32	1.00E-02	4.47	18.09	8.05E-01	-20.58	10.13	4.22E-02	-26.52	16.39	1.06E-01
cg10778994	16.75	5.15	1.14E-03	17.84	6.38	5.16E-03	27.94	13.86	4.39E-02	21.17	21.32	3.21E-01	0.27	13.19	9.84E-01
cg11721803	42.03	12.92	1.14E-03	30.53	26.78	2.54E-01	-18.70	41.57	6.53E-01	55.74	17.78	1.72E-03	51.12	34.15	1.34E-01
cg03461298	-36.38	11.18	1.14E-03	-27.91	40.15	4.87E-01	-115.77	192.57	5.48E-01	-31.35	12.17	9.98E-03	-98.82	41.02	1.60E-02
cg06171787	35.06	10.78	1.14E-03	44.92	15.36	3.45E-03	15.18	30.93	6.24E-01	54.18	31.32	8.37E-02	17.51	20.82	4.01E-01
cg26943658	-72.98	22.43	1.14E-03	-113.01	67.50	9.41E-02	-94.04	218.52	6.67E-01	-73.25	25.44	3.99E-03	-25.17	70.43	7.21E-01
cg24357447	30.18	9.28	1.15E-03	44.25	15.03	3.25E-03	-0.33	28.22	9.91E-01	42.65	27.60	1.22E-01	21.45	14.72	1.45E-01
cg26470924	24.09	7.41	1.15E-03	17.67	17.62	3.16E-01	21.38	31.22	4.94E-01	24.48	8.86	5.72E-03	39.30	28.63	1.70E-01
cg12309653	27.44	8.44	1.15E-03	35.28	18.41	5.54E-02	12.88	36.22	7.22E-01	28.51	10.79	8.24E-03	15.23	23.96	5.25E-01
cg08467185	49.71	15.29	1.15E-03	40.36	37.13	2.77E-01	107.01	164.92	5.16E-01	50.48	17.64	4.21E-03	57.03	57.62	3.22E-01
cg17431952	-15.03	4.62	1.15E-03	-22.79	8.92	1.06E-02	-3.44	12.29	7.80E-01	-12.52	7.15	8.00E-02	-18.53	11.16	9.67E-02
cg12221580	-13.82	4.25	1.15E-03	-9.09	9.53	3.40E-01	-4.61	12.00	7.01E-01	-19.19	5.67	7.11E-04	-5.65	12.65	6.55E-01
cg16710517	-34.12	10.50	1.16E-03	-49.74	17.08	3.58E-03	-27.71	28.45	3.30E-01	-35.25	18.36	5.49E-02	-0.11	26.35	9.97E-01
cg01760983	16.93	5.21	1.16E-03	18.95	6.55	3.81E-03	22.52	22.13	3.09E-01	11.94	25.82	6.44E-01	11.81	10.01	2.38E-01
cg03049445	-16.48	5.07	1.16E-03	-20.46	10.02	4.12E-02	-2.97	13.53	8.26E-01	-14.68	7.28	4.38E-02	-31.36	14.79	3.40E-02
cg08995368	32.21	9.92	1.17E-03	27.64	36.26	4.46E-01	13.27	157.43	9.33E-01	35.27	10.53	8.09E-04	-36.08	54.09	5.05E-01
cg13549497	13.85	4.27	1.17E-03	17.86	6.71	7.81E-03	4.29	9.67	6.57E-01	11.05	8.61	1.99E-01	19.85	10.82	6.65E-02
cg26379707	45.91	14.14	1.17E-03	46.50	19.56	1.75E-02	78.75	31.69	1.29E-02	0.47	50.66	9.93E-01	29.38	31.61	3.53E-01
cg03623403	37.44	11.53	1.17E-03	26.19	34.54	4.48E-01	105.82	126.37	4.02E-01	40.89	12.83	1.44E-03	8.15	42.98	8.50E-01
cg04955333	17.19	5.30	1.17E-03	18.38	7.85	1.92E-02	18.25	10.62	8.57E-02	-2.82	16.72	8.66E-01	23.35	11.98	5.12E-02
cg25237894	-12.96	3.99	1.17E-03	-13.67	5.83	1.90E-02	-18.81	10.18	6.46E-02	3.09	15.20	8.39E-01	-12.55	7.20	8.13E-02
cg04367107	-12.79	3.94	1.17E-03	-13.12	7.59	8.39E-02	-8.14	12.44	5.13E-01	-13.84	5.44	1.09E-02	-11.12	12.16	3.60E-01
cg23689457	47.23	14.56	1.18E-03	12.48	45.40	7.83E-01	106.18	175.44	5.45E-01	49.77	16.14	2.05E-03	61.50	52.35	2.40E-01
cg15768620	20.33	6.26	1.18E-03	24.38	9.66	1.16E-02	22.84	14.48	1.15E-01	18.82	14.95	2.08E-01	11.50	13.46	3.93E-01
cg06029700	12.06	3.72	1.19E-03	16.10	5.53	3.60E-03	4.64	10.12	6.47E-01	17.51	10.11	8.32E-02	6.42	7.07	3.64E-01
cg26870570	-23.54	7.26	1.19E-03	-11.15	10.75	3.00E-01	-36.37	14.96	1.51E-02	-23.09	23.09	3.17E-01	-36.36	15.88	2.21E-02
cg12694224	13.93	4.30	1.19E-03	11.22	6.70	9.42E-02	21.16	10.45	4.29E-02	21.17	9.57	2.70E-02	6.75	9.19	4.63E-01
cg08309041	-40.63	12.54	1.19E-03	-45.35	17.87	1.12E-02	-33.81	43.81	4.40E-01	-33.05	27.62	2.31E-01	-39.71	26.77	1.38E-01
cg11629220	-24.49	7.56	1.20E-03	-32.14	11.68	5.94E-03	5.26	49.09	9.15E-01	-26.53	13.62	5.15E-02	-11.97	15.13	4.29E-01
cg12435677	28.71	8.86	1.20E-03	23.44	15.02	1.19E-01	31.45	16.68	5.93E-02	28.71	19.31	1.37E-01	35.39	22.26	1.12E-01
cg14799457	12.81	3.96	1.20E-03	11.90	7.14	9.55E-02	12.76	8.49	1.33E-01	4.98	8.37	5.52E-01	20.85	7.86	8.01E-03
cg15079315	13.05	4.03	1.20E-03	12.81	8.85	1.48E-01	20.28	13.85	1.43E-01	14.66	5.33	5.91E-03	2.09	10.96	8.49E-01

cg00687612	40.48	12.50	1.21E-03	31.81	19.29	9.91E-02	45.30	31.52	1.51E-01	45.53	24.93	6.77E-02	49.91	30.25	9.89E-02
cg13265869	26.76	8.27	1.21E-03	33.60	27.08	2.15E-01	0.95	87.73	9.91E-01	27.45	9.06	2.45E-03	11.85	32.20	7.13E-01
cg07886914	-14.06	4.34	1.21E-03	-21.94	10.07	2.93E-02	-25.19	17.26	1.45E-01	-11.61	5.52	3.54E-02	-9.07	11.98	4.49E-01
cg03835292	20.24	6.25	1.21E-03	24.34	11.28	3.10E-02	13.74	19.33	4.77E-01	22.16	9.33	1.75E-02	9.81	16.80	5.59E-01
cg10835308	49.59	15.33	1.22E-03	11.94	40.61	7.69E-01	265.32	160.26	9.78E-02	50.23	16.23	1.96E-03	82.95	52.86	1.17E-01
cg25962358	-10.06	3.11	1.22E-03	-10.58	4.89	3.06E-02	-11.59	6.88	9.17E-02	-18.53	8.17	2.33E-02	-2.95	6.28	6.39E-01
cg08918576	-13.74	4.25	1.23E-03	-10.17	15.62	5.15E-01	-14.63	30.27	6.29E-01	-13.92	4.62	2.59E-03	-15.33	17.32	3.76E-01
cg19756700	54.46	16.85	1.23E-03	47.12	45.57	3.01E-01	-101.58	237.12	6.68E-01	55.92	19.05	3.33E-03	63.10	61.09	3.02E-01
cg01910481	25.51	7.89	1.23E-03	11.29	18.28	5.37E-01	-19.96	39.20	6.11E-01	28.59	9.69	3.16E-03	47.92	23.88	4.48E-02
cg09641919	-55.14	17.06	1.23E-03	-67.51	21.34	1.56E-03	-30.40	75.38	6.87E-01	37.96	63.17	5.48E-01	-55.79	35.09	1.12E-01
cg24966902	-11.64	3.60	1.23E-03	-16.71	7.02	1.74E-02	-6.40	12.45	6.07E-01	-10.77	5.18	3.76E-02	-8.86	8.76	3.12E-01
cg19515530	32.57	10.08	1.24E-03	25.52	28.94	3.78E-01	47.63	78.64	5.45E-01	32.23	11.50	5.08E-03	41.82	32.89	2.04E-01
cg01700462	9.84	3.05	1.24E-03	9.66	3.97	1.50E-02	11.68	8.00	1.44E-01	8.47	8.32	3.09E-01	10.00	8.34	2.31E-01
cg19898108	23.27	7.20	1.24E-03	20.00	9.27	3.10E-02	32.00	21.36	1.34E-01	12.66	45.31	7.80E-01	28.13	14.22	4.78E-02
cg26944052	27.78	8.60	1.24E-03	12.59	16.40	4.43E-01	36.55	53.18	4.92E-01	29.43	10.91	6.97E-03	65.91	31.09	3.40E-02
cg04270347	47.98	14.86	1.25E-03	43.17	17.07	1.15E-02	52.26	100.30	6.02E-01	147.61	74.37	4.72E-02	45.61	35.01	1.93E-01
cg05894910	19.42	6.02	1.25E-03	22.52	7.73	3.58E-03	11.16	23.10	6.29E-01	9.21	29.64	7.56E-01	16.28	11.26	1.48E-01
cg11228717	17.35	5.38	1.25E-03	25.21	8.01	1.65E-03	15.16	11.59	1.91E-01	14.46	24.08	5.48E-01	7.07	10.08	4.83E-01
cg00585174	-30.26	9.38	1.25E-03	-43.21	16.47	8.72E-03	-21.70	45.51	6.34E-01	-24.09	13.69	7.85E-02	-24.57	23.10	2.88E-01
cg21376004	25.35	7.85	1.25E-03	22.17	11.43	5.25E-02	21.75	15.31	1.55E-01	29.99	22.27	1.78E-01	38.62	20.94	6.51E-02
cg22162597	12.39	3.84	1.25E-03	11.98	7.77	1.23E-01	5.69	8.22	4.89E-01	17.22	6.07	4.55E-03	9.70	10.37	3.49E-01
cg03175030	-29.71	9.21	1.25E-03	-30.88	11.60	7.76E-03	-6.03	25.72	8.15E-01	-11.88	31.70	7.08E-01	-53.76	23.17	2.03E-02
cg20699780	8.63	2.68	1.26E-03	7.08	3.49	4.24E-02	13.95	6.25	2.56E-02	14.96	11.12	1.79E-01	6.13	6.51	3.46E-01
cg17748822	17.66	5.48	1.26E-03	20.81	9.53	2.90E-02	8.14	13.88	5.58E-01	10.54	9.49	2.67E-01	33.15	12.85	9.90E-03
cg02566627	24.54	7.61	1.26E-03	25.35	10.06	1.17E-02	36.18	19.48	6.33E-02	42.63	25.45	9.40E-02	3.78	17.66	8.31E-01
cg06840743	8.13	2.52	1.26E-03	9.17	3.45	7.89E-03	12.62	6.54	5.37E-02	8.94	6.76	1.86E-01	0.63	5.99	9.17E-01
cg20719132	47.86	14.85	1.27E-03	41.82	40.24	2.99E-01	-26.10	101.49	7.97E-01	54.37	17.20	1.57E-03	22.70	47.61	6.34E-01
cg07777540	-27.11	8.41	1.27E-03	1.98	23.38	9.32E-01	-44.13	49.40	3.72E-01	-33.67	9.54	4.16E-04	1.40	33.23	9.66E-01
cg11715999	-64.46	20.01	1.28E-03	-80.28	27.35	3.33E-03	-124.40	68.53	6.95E-02	-2.31	56.07	9.67E-01	-41.99	39.85	2.92E-01
cg10105917	-17.67	5.49	1.28E-03	-8.30	12.69	5.13E-01	-2.90	25.56	9.10E-01	-23.50	6.79	5.41E-04	-5.74	16.19	7.23E-01
cg03765206	21.80	6.77	1.28E-03	17.38	18.31	3.42E-01	53.27	36.55	1.45E-01	19.90	7.86	1.14E-02	32.49	22.92	1.56E-01
cg13744822	56.44	17.53	1.28E-03	37.32	46.77	4.25E-01	-78.12	221.02	7.24E-01	59.83	19.96	2.73E-03	67.63	61.20	2.69E-01
cg18166990	-17.98	5.59	1.29E-03	-27.71	9.36	3.07E-03	-11.92	11.70	3.08E-01	-15.07	11.29	1.82E-01	-9.95	13.52	4.61E-01
cg26516489	-26.03	8.09	1.29E-03	-7.84	24.32	7.47E-01	-116.64	113.16	3.03E-01	-27.48	8.78	1.75E-03	-34.68	42.62	4.16E-01
cg05257772	-27.72	8.62	1.29E-03	-31.61	13.66	2.07E-02	-28.78	20.87	1.68E-01	-21.22	17.11	2.15E-01	-27.27	20.41	1.81E-01

cg05963618	38.68	12.02	1.30E-03	75.98	33.14	2.19E-02	62.32	68.04	3.60E-01	30.83	14.18	2.96E-02	38.62	35.03	2.70E-01
cg01015354	9.93	3.09	1.30E-03	10.92	6.42	8.89E-02	1.09	8.31	8.96E-01	13.45	4.39	2.19E-03	4.41	8.36	5.97E-01
cg21865845	16.89	5.25	1.31E-03	23.57	7.70	2.19E-03	6.16	13.88	6.57E-01	17.80	11.30	1.15E-01	6.72	12.59	5.93E-01
cg02538046	26.00	8.09	1.31E-03	37.94	17.00	2.56E-02	35.95	18.51	5.21E-02	15.53	11.55	1.79E-01	31.64	26.55	2.33E-01
cg27429080	-29.41	9.15	1.31E-03	7.71	27.42	7.78E-01	-10.60	85.29	9.01E-01	-35.56	10.13	4.45E-04	-18.32	37.33	6.23E-01
cg08783639	25.26	7.86	1.32E-03	34.89	18.06	5.35E-02	-30.42	38.15	4.25E-01	27.37	9.61	4.40E-03	16.40	25.06	5.13E-01
cg05706117	28.20	8.79	1.33E-03	31.00	16.64	6.25E-02	31.27	27.37	2.53E-01	28.55	13.45	3.38E-02	21.71	20.06	2.79E-01
cg25565138	31.51	9.82	1.33E-03	36.30	24.43	1.37E-01	51.84	115.17	6.53E-01	29.03	11.16	9.27E-03	48.85	41.01	2.34E-01
cg08679800	-15.72	4.90	1.34E-03	-19.93	7.45	7.44E-03	-19.92	11.20	7.52E-02	-8.99	11.50	4.34E-01	-8.46	11.12	4.47E-01
cg18774642	-10.08	3.14	1.34E-03	-9.88	4.41	2.50E-02	-13.33	7.13	6.16E-02	6.54	15.16	6.66E-01	-10.80	6.23	8.29E-02
cg21776667	-13.42	4.18	1.34E-03	-11.02	11.70	3.46E-01	-13.24	24.16	5.84E-01	-14.21	4.73	2.67E-03	-8.29	16.95	6.25E-01
cg15824705	34.56	10.78	1.34E-03	35.10	15.97	2.79E-02	70.75	35.72	4.76E-02	52.38	52.80	3.21E-01	24.16	16.79	1.50E-01
cg01452581	32.21	10.05	1.34E-03	32.56	19.28	9.12E-02	37.99	18.96	4.51E-02	14.11	18.79	4.53E-01	53.58	24.98	3.20E-02
cg26112846	17.56	5.48	1.35E-03	9.47	14.84	5.23E-01	33.11	55.91	5.54E-01	19.04	6.22	2.21E-03	15.12	19.47	4.37E-01
cg22115808	57.18	17.85	1.36E-03	115.61	46.85	1.36E-02	-69.82	203.79	7.32E-01	45.09	20.48	2.77E-02	76.20	60.23	2.06E-01
cg01802593	-15.44	4.82	1.36E-03	-8.83	13.25	5.05E-01	1.15	24.10	9.62E-01	-18.01	5.60	1.30E-03	-11.18	16.27	4.92E-01
cg22767408	33.46	10.44	1.36E-03	34.04	21.17	1.08E-01	-29.00	67.32	6.67E-01	32.81	12.99	1.15E-02	54.10	35.59	1.29E-01
cg23045594	16.18	5.05	1.36E-03	23.32	11.46	4.19E-02	7.55	17.87	6.73E-01	19.70	8.32	1.80E-02	10.60	8.46	2.10E-01
cg18290848	23.04	7.20	1.37E-03	35.50	17.37	4.10E-02	4.50	27.23	8.69E-01	24.30	9.89	1.40E-02	16.38	15.08	2.78E-01
cg04166042	18.16	5.67	1.38E-03	24.26	10.07	1.60E-02	2.90	12.53	8.17E-01	26.56	10.87	1.46E-02	12.80	12.54	3.08E-01
cg08969102	23.20	7.25	1.38E-03	17.32	11.78	1.42E-01	45.20	15.13	2.81E-03	19.99	15.94	2.10E-01	11.47	16.88	4.97E-01
cg14124066	-17.70	5.53	1.38E-03	-12.28	16.29	4.51E-01	5.80	23.03	8.01E-01	-18.38	6.39	4.05E-03	-36.39	19.75	6.54E-02
cg12046867	33.65	10.52	1.38E-03	42.80	32.54	1.88E-01	69.62	152.33	6.48E-01	33.32	11.51	3.80E-03	18.35	44.58	6.81E-01
cg27138951	14.93	4.67	1.38E-03	17.60	6.84	1.01E-02	15.65	11.46	1.72E-01	11.03	11.88	3.53E-01	11.39	10.08	2.59E-01
cg04875709	-56.52	17.67	1.38E-03	-92.80	52.91	7.95E-02	58.10	251.80	8.18E-01	-52.99	19.99	8.02E-03	-49.39	55.37	3.72E-01
cg16601904	14.89	4.65	1.38E-03	20.20	6.76	2.81E-03	3.87	9.60	6.87E-01	9.28	13.82	5.02E-01	18.86	11.04	8.76E-02
cg02309655	-19.25	6.02	1.39E-03	0.73	19.07	9.69E-01	-34.89	34.21	3.08E-01	-19.17	6.66	4.01E-03	-49.52	26.42	6.08E-02
cg23012591	-11.09	3.47	1.39E-03	-19.76	7.66	9.88E-03	-2.67	11.66	8.19E-01	-10.05	4.45	2.39E-02	-7.01	11.12	5.29E-01
cg01811109	-11.94	3.74	1.40E-03	-18.52	8.16	2.33E-02	3.28	15.36	8.31E-01	-10.63	4.78	2.60E-02	-14.67	10.82	1.75E-01
cg12045158	-19.91	6.23	1.40E-03	-15.78	16.51	3.39E-01	-8.98	37.60	8.11E-01	-20.74	7.34	4.74E-03	-22.64	18.88	2.30E-01
cg00137634	41.13	12.89	1.41E-03	42.01	14.43	3.60E-03	8.46	65.39	8.97E-01	-67.98	86.02	4.29E-01	62.56	34.32	6.84E-02
cg09765797	-19.46	6.10	1.41E-03	3.55	20.03	8.59E-01	-10.99	27.40	6.88E-01	-22.40	6.78	9.55E-04	-22.87	27.31	4.02E-01
cg21011827	16.75	5.25	1.41E-03	6.00	13.31	6.52E-01	20.56	54.75	7.07E-01	18.87	5.96	1.54E-03	16.63	21.55	4.40E-01
cg03672272	16.27	5.10	1.42E-03	14.61	7.16	4.13E-02	12.16	12.47	3.30E-01	31.48	15.21	3.85E-02	15.45	11.04	1.62E-01
cg11065575	-6.59	2.07	1.43E-03	-6.90	3.46	4.60E-02	-6.17	4.64	1.83E-01	-2.78	4.05	4.92E-01	-11.77	4.80	1.42E-02

cg12535280	15.15	4.75	1.43E-03	21.03	10.12	3.77E-02	5.90	13.20	6.55E-01	15.55	7.38	3.50E-02	14.00	9.78	1.53E-01
cg15791108	-10.13	3.18	1.43E-03	-10.38	4.28	1.52E-02	-6.84	6.93	3.24E-01	-15.43	12.50	2.17E-01	-11.30	7.60	1.37E-01
cg25584862	11.62	3.65	1.44E-03	5.47	5.35	3.07E-01	18.87	7.85	1.62E-02	16.32	12.56	1.94E-01	15.48	7.53	3.99E-02
cg14924671	-14.34	4.50	1.44E-03	-20.08	9.36	3.18E-02	-11.86	20.42	5.62E-01	-11.42	5.70	4.53E-02	-20.68	14.47	1.53E-01
cg22722777	-18.18	5.71	1.45E-03	-36.76	14.76	1.28E-02	-6.32	26.93	8.14E-01	-14.05	6.76	3.76E-02	-25.79	18.83	1.71E-01
cg04168494	-19.33	6.07	1.45E-03	-31.46	12.99	1.54E-02	-12.04	28.56	6.73E-01	-18.99	7.88	1.60E-02	-4.49	16.07	7.80E-01
cg02052217	16.29	5.12	1.46E-03	13.05	7.03	6.33E-02	22.67	10.92	3.78E-02	26.98	16.70	1.06E-01	11.86	12.96	3.60E-01
cg05388963	-24.63	7.74	1.46E-03	-27.46	10.37	8.10E-03	-0.56	20.26	9.78E-01	-41.03	23.63	8.25E-02	-25.58	17.78	1.50E-01
cg09974136	50.86	15.99	1.46E-03	22.79	41.18	5.80E-01	-52.06	188.48	7.82E-01	53.88	18.24	3.13E-03	86.70	58.80	1.40E-01
cg05591447	16.94	5.32	1.47E-03	15.17	8.87	8.72E-02	17.43	8.48	3.98E-02	46.31	24.57	5.94E-02	12.21	11.94	3.07E-01
cg05977671	-23.30	7.32	1.47E-03	-32.83	16.94	5.25E-02	3.47	34.42	9.20E-01	-20.49	8.97	2.24E-02	-36.11	22.98	1.16E-01
cg26903766	24.62	7.74	1.47E-03	34.85	12.39	4.89E-03	13.90	14.43	3.35E-01	33.09	21.10	1.17E-01	13.61	17.94	4.48E-01
cg22790835	-17.27	5.43	1.47E-03	-18.96	8.53	2.63E-02	-21.04	12.53	9.31E-02	-13.90	15.19	3.60E-01	-13.83	10.28	1.79E-01
cg00168634	35.62	11.21	1.48E-03	43.51	15.34	4.56E-03	43.14	34.04	2.05E-01	27.37	30.50	3.70E-01	18.09	23.73	4.46E-01
cg20704530	-12.26	3.86	1.48E-03	-17.17	7.14	1.61E-02	-11.32	8.81	1.99E-01	-15.67	6.74	2.00E-02	0.35	8.90	9.69E-01
cg26158950	62.25	19.59	1.48E-03	51.88	29.51	7.87E-02	7.88	58.03	8.92E-01	147.89	56.90	9.35E-03	62.69	28.89	3.00E-02
cg14006405	22.18	6.98	1.49E-03	16.54	13.69	2.27E-01	28.54	20.98	1.74E-01	21.45	9.54	2.46E-02	34.62	22.88	1.30E-01
cg14003265	-11.41	3.59	1.50E-03	-10.18	4.62	2.74E-02	-13.37	7.75	8.44E-02	-14.92	15.07	3.22E-01	-12.45	10.29	2.26E-01
cg07570835	15.66	4.93	1.50E-03	18.21	9.09	4.53E-02	17.31	11.78	1.42E-01	18.96	7.57	1.22E-02	-7.46	15.17	6.23E-01
cg14998460	-25.38	8.00	1.51E-03	-31.24	11.89	8.61E-03	2.78	21.70	8.98E-01	-39.39	18.10	2.95E-02	-18.16	17.21	2.91E-01
cg05232016	-22.60	7.12	1.51E-03	-13.95	12.04	2.47E-01	-29.38	15.35	5.56E-02	-30.44	14.21	3.22E-02	-20.41	16.65	2.20E-01
cg07500019	39.89	12.58	1.52E-03	30.72	15.74	5.09E-02	23.23	37.11	5.31E-01	110.33	49.68	2.64E-02	57.81	29.49	5.00E-02
cg25970726	-13.05	4.12	1.52E-03	-10.43	7.04	1.38E-01	-14.18	8.70	1.03E-01	-20.33	8.35	1.49E-02	-7.15	9.42	4.48E-01
cg15169988	16.79	5.30	1.53E-03	16.26	13.28	2.21E-01	21.48	11.40	5.95E-02	18.97	7.17	8.14E-03	-10.33	18.96	5.86E-01
cg09222269	-25.89	8.17	1.54E-03	-31.22	12.51	1.26E-02	-0.80	28.61	9.78E-01	-39.21	17.70	2.67E-02	-14.86	15.50	3.38E-01
cg02850933	-69.49	21.94	1.54E-03	-48.61	56.73	3.91E-01	-361.91	348.16	2.99E-01	-72.68	25.53	4.42E-03	-65.91	66.83	3.24E-01
cg11205072	29.22	9.23	1.55E-03	32.88	12.65	9.33E-03	12.71	43.39	7.70E-01	36.46	31.64	2.49E-01	23.82	15.90	1.34E-01
cg19758054	40.31	12.74	1.55E-03	36.93	29.91	2.17E-01	-14.11	75.23	8.51E-01	43.32	15.10	4.11E-03	40.63	45.51	3.72E-01
cg04604420	15.85	5.01	1.56E-03	6.37	11.78	5.88E-01	8.67	27.96	7.56E-01	18.30	5.88	1.85E-03	18.71	20.60	3.64E-01
cg10181414	7.31	2.31	1.56E-03	6.64	3.04	2.87E-02	3.67	6.00	5.41E-01	11.65	6.81	8.72E-02	9.98	5.81	8.57E-02
cg07134033	63.47	20.07	1.56E-03	11.23	40.77	7.83E-01	-80.38	176.50	6.49E-01	70.88	16.47	1.68E-05	116.77	50.09	1.97E-02
cg02770857	13.60	4.30	1.57E-03	12.45	6.00	3.79E-02	20.53	11.45	7.30E-02	17.72	10.57	9.35E-02	7.62	10.16	4.53E-01
cg05100540	-52.05	16.47	1.57E-03	-52.51	18.11	3.73E-03	-157.51	133.71	2.39E-01	55.50	106.04	6.01E-01	-56.59	45.05	2.09E-01
cg05507566	7.00	2.21	1.58E-03	6.51	3.47	6.07E-02	9.31	5.05	6.54E-02	6.47	3.88	9.49E-02	5.96	8.04	4.59E-01
cg21379885	-52.00	16.46	1.58E-03	-53.35	19.18	5.40E-03	-212.43	109.46	5.23E-02	-24.22	80.72	7.64E-01	-34.56	36.91	3.49E-01

cg19464419	-16.43	5.21	1.60E-03	-9.79	8.82	2.67E-01	-12.19	17.48	4.86E-01	-24.13	6.01	5.98E-05	1.09	15.97	9.46E-01
cg11377875	-12.33	3.91	1.60E-03	-16.30	10.07	1.05E-01	-5.00	12.34	6.85E-01	-13.07	4.80	6.44E-03	-8.23	13.35	5.38E-01
cg13926569	24.46	7.75	1.60E-03	20.69	12.25	9.13E-02	29.48	15.73	6.10E-02	10.74	17.41	5.37E-01	43.36	19.43	2.57E-02
cg05993778	-15.11	4.79	1.61E-03	-34.51	15.93	3.02E-02	11.07	32.14	7.30E-01	-14.48	5.31	6.35E-03	-5.99	17.86	7.37E-01
cg09949949	19.89	6.31	1.61E-03	24.45	17.30	1.57E-01	8.99	67.16	8.94E-01	17.90	7.08	1.14E-02	36.53	24.83	1.41E-01
cg01416724	21.71	6.88	1.61E-03	10.45	19.62	5.94E-01	25.00	37.21	5.02E-01	24.96	7.79	1.36E-03	1.36	27.63	9.61E-01
cg02235659	11.64	3.69	1.62E-03	13.90	5.70	1.47E-02	13.46	7.93	8.96E-02	13.55	8.10	9.46E-02	0.47	9.36	9.60E-01
cg03714573	62.79	19.92	1.62E-03	70.10	28.73	1.47E-02	-83.21	119.88	4.88E-01	86.17	53.33	1.06E-01	55.00	33.56	1.01E-01
cg17851046	-30.20	9.58	1.62E-03	-36.20	13.45	7.10E-03	-0.68	24.70	9.78E-01	-45.79	31.28	1.43E-01	-29.95	19.27	1.20E-01
cg16238854	-42.10	13.36	1.63E-03	-28.50	16.67	8.74E-02	-35.60	41.19	3.87E-01	-94.38	35.73	8.26E-03	-58.33	37.99	1.25E-01
cg17480278	20.80	6.60	1.63E-03	20.89	9.82	3.33E-02	15.00	16.23	3.56E-01	-3.89	23.70	8.69E-01	30.12	11.96	1.18E-02
cg19756929	26.19	8.31	1.63E-03	26.42	19.08	1.66E-01	18.43	81.52	8.21E-01	28.78	9.95	3.82E-03	8.78	26.11	7.37E-01
cg23183906	-65.00	20.63	1.63E-03	-79.46	70.97	2.63E-01	-273.30	248.58	2.72E-01	-62.33	22.78	6.21E-03	-59.72	69.60	3.91E-01
cg22891070	5.58	1.77	1.63E-03	7.17	2.50	4.08E-03	4.09	3.60	2.57E-01	0.04	6.08	9.94E-01	5.76	4.30	1.81E-01
cg23647884	-54.70	17.37	1.64E-03	-84.19	43.44	5.26E-02	-114.54	286.49	6.89E-01	-41.09	19.82	3.82E-02	-134.95	66.28	4.17E-02
cg18592195	-55.09	17.50	1.64E-03	-68.07	23.90	4.40E-03	13.59	91.38	8.82E-01	-103.16	68.01	1.29E-01	-34.01	29.12	2.43E-01
cg01555981	-25.50	8.10	1.64E-03	-5.78	21.01	7.83E-01	-4.36	40.62	9.14E-01	-32.71	9.41	5.12E-04	-3.66	30.28	9.04E-01
cg15976539	-17.97	5.71	1.64E-03	-33.86	14.68	2.10E-02	-6.24	20.46	7.60E-01	-17.39	6.82	1.08E-02	-2.75	21.40	8.98E-01
cg22368281	-16.30	5.18	1.64E-03	-19.18	7.21	7.81E-03	-9.71	12.04	4.20E-01	-20.61	18.28	2.60E-01	-13.48	11.05	2.22E-01
cg20170777	-14.16	4.50	1.64E-03	-12.76	7.17	7.52E-02	-19.19	10.39	6.46E-02	-12.44	10.37	2.30E-01	-13.87	9.37	1.39E-01
cg18366748	24.64	7.83	1.65E-03	24.47	11.73	3.70E-02	17.41	23.08	4.51E-01	48.59	24.81	5.02E-02	20.29	13.42	1.30E-01
cg04461802	15.02	4.77	1.65E-03	9.00	6.96	1.96E-01	22.11	11.59	5.63E-02	28.57	12.80	2.56E-02	13.88	10.17	1.72E-01
cg15160686	-19.37	6.16	1.65E-03	-15.07	9.08	9.70E-02	-13.26	14.57	3.63E-01	-34.87	15.06	2.05E-02	-21.82	13.94	1.18E-01
cg05559445	15.88	5.05	1.65E-03	16.18	7.27	2.60E-02	21.02	11.83	7.57E-02	15.59	12.42	2.09E-01	9.84	12.20	4.20E-01
cg04186606	70.45	22.40	1.66E-03	87.80	30.48	3.97E-03	118.65	50.80	1.95E-02	-8.28	53.64	8.77E-01	58.00	41.39	1.61E-01
cg14245135	24.32	7.73	1.66E-03	25.79	10.57	1.47E-02	32.84	21.12	1.20E-01	18.52	26.99	4.93E-01	18.48	15.50	2.33E-01
cg18931760	12.97	4.12	1.66E-03	10.63	9.66	2.71E-01	9.74	12.66	4.42E-01	16.61	5.20	1.42E-03	-5.16	14.26	7.17E-01
cg09964921	14.89	4.74	1.67E-03	9.81	8.66	2.58E-01	15.79	13.86	2.54E-01	16.57	6.59	1.20E-02	22.91	18.14	2.07E-01
cg01402347	-11.65	3.71	1.67E-03	-20.63	9.17	2.46E-02	-10.08	14.47	4.86E-01	-8.67	4.54	5.61E-02	-17.65	11.47	1.24E-01
cg22188068	59.90	19.06	1.67E-03	101.58	52.60	5.35E-02	-181.59	189.28	3.37E-01	56.95	21.52	8.12E-03	50.27	70.10	4.73E-01
cg08988364	-26.70	8.50	1.68E-03	-37.27	31.74	2.40E-01	-21.66	85.24	7.99E-01	-23.81	9.10	8.92E-03	-65.03	39.08	9.61E-02
cg12886676	-32.68	10.40	1.68E-03	-38.27	13.35	4.15E-03	-4.31	27.02	8.73E-01	-44.05	43.71	3.14E-01	-33.55	23.99	1.62E-01
cg02833116	-51.37	16.36	1.69E-03	-71.28	22.42	1.48E-03	-67.53	102.14	5.09E-01	-4.53	80.25	9.55E-01	-28.74	25.85	2.66E-01
cg21449646	-22.59	7.19	1.69E-03	-10.94	21.96	6.18E-01	10.81	43.86	8.05E-01	-20.16	10.18	4.77E-02	-31.76	11.89	7.54E-03
cg05500783	-15.10	4.81	1.70E-03	-20.76	8.94	2.03E-02	-8.41	18.52	6.50E-01	-9.82	5.15	5.64E-02	-34.56	14.23	1.51E-02

cg21304453	16.36	5.22	1.71E-03	16.92	9.09	6.27E-02	16.66	11.20	1.37E-01	17.60	12.09	1.45E-01	14.57	10.10	1.49E-01
cg25219318	-77.94	24.85	1.71E-03	-59.47	32.51	6.73E-02	-222.66	143.81	1.22E-01	-154.76	60.80	1.09E-02	-55.03	41.34	1.83E-01
cg12277524	-21.25	6.78	1.71E-03	-28.74	22.83	2.08E-01	-19.32	13.06	1.39E-01	-23.38	8.78	7.76E-03	8.42	31.08	7.87E-01
cg01843946	22.77	7.26	1.71E-03	20.96	12.80	1.02E-01	13.27	19.65	4.99E-01	21.80	12.52	8.16E-02	33.56	16.04	3.64E-02
cg15126544	-26.93	8.59	1.71E-03	-42.42	26.57	1.10E-01	-9.08	42.68	8.32E-01	-23.34	9.56	1.46E-02	-69.65	39.62	7.88E-02
cg08319074	26.89	8.58	1.72E-03	48.04	18.59	9.74E-03	21.93	21.04	2.97E-01	18.68	11.78	1.13E-01	34.21	28.38	2.28E-01
cg07842635	-10.58	3.37	1.72E-03	-13.20	7.63	8.34E-02	-16.63	14.50	2.51E-01	-6.80	4.26	1.10E-01	-23.04	9.64	1.68E-02
cg17756105	-12.01	3.83	1.72E-03	-9.64	6.13	1.16E-01	-12.10	7.80	1.21E-01	-15.91	9.18	8.30E-02	-13.16	8.70	1.30E-01
cg11334406	-17.36	5.54	1.72E-03	-27.69	9.67	4.18E-03	-10.43	12.78	4.14E-01	-7.38	10.22	4.70E-01	-21.81	12.71	8.62E-02
cg26075905	-62.14	19.83	1.72E-03	-71.15	30.55	1.99E-02	-25.19	54.97	6.47E-01	-70.49	47.68	1.39E-01	-60.57	37.76	1.09E-01
cg27122127	33.43	10.67	1.73E-03	13.49	31.04	6.64E-01	40.11	154.38	7.95E-01	36.26	11.81	2.13E-03	33.72	43.54	4.39E-01
cg03225444	11.41	3.64	1.73E-03	9.10	5.37	8.98E-02	4.84	8.30	5.60E-01	29.39	12.10	1.51E-02	13.44	6.52	3.93E-02
cg24128316	-12.09	3.86	1.75E-03	-12.63	6.17	4.07E-02	-11.93	9.09	1.89E-01	-11.95	7.34	1.04E-01	-11.15	9.97	2.64E-01
cg19289921	12.56	4.01	1.75E-03	12.46	6.06	3.98E-02	9.50	9.02	2.92E-01	29.63	17.15	8.40E-02	11.65	7.24	1.07E-01
cg09798202	-47.24	15.10	1.75E-03	-49.42	22.65	2.91E-02	12.97	97.25	8.94E-01	-78.24	31.62	1.33E-02	-25.55	27.40	3.51E-01
cg14427009	16.02	5.12	1.75E-03	23.41	9.10	1.01E-02	3.90	14.64	7.90E-01	18.74	8.92	3.57E-02	8.47	10.62	4.25E-01
cg13777473	-14.96	4.78	1.76E-03	-18.51	7.74	1.67E-02	2.39	13.33	8.58E-01	-19.33	8.52	2.33E-02	-12.09	11.49	2.93E-01
cg27382405	38.57	12.33	1.77E-03	69.20	36.58	5.85E-02	-18.67	134.92	8.90E-01	28.53	14.12	4.33E-02	79.20	36.43	2.97E-02
cg02348462	13.92	4.45	1.77E-03	10.65	5.90	7.09E-02	23.26	9.92	1.90E-02	26.48	15.14	8.03E-02	6.11	11.81	6.05E-01
cg04214966	11.92	3.82	1.77E-03	12.04	5.17	2.00E-02	6.90	9.52	4.69E-01	4.64	13.40	7.29E-01	18.15	8.23	2.75E-02
cg14267671	18.75	6.00	1.78E-03	25.03	8.47	3.13E-03	13.44	13.05	3.03E-01	20.88	18.60	2.61E-01	6.46	14.03	6.45E-01
cg05943554	-19.53	6.25	1.78E-03	-26.33	18.07	1.45E-01	-25.17	36.36	4.89E-01	-19.24	7.03	6.23E-03	-7.23	25.31	7.75E-01
cg10858828	77.29	24.74	1.78E-03	92.01	33.60	6.18E-03	-67.64	102.46	5.09E-01	88.59	72.55	2.22E-01	74.32	46.45	1.10E-01
cg23060513	9.60	3.07	1.79E-03	5.64	3.98	1.57E-01	16.14	5.89	6.16E-03	24.90	17.17	1.47E-01	10.19	9.13	2.64E-01
cg25384906	40.11	12.84	1.79E-03	32.15	17.24	6.23E-02	18.44	35.11	6.00E-01	63.89	42.72	1.35E-01	63.49	27.31	2.01E-02
cg16224951	39.00	12.49	1.79E-03	30.69	13.93	2.75E-02	111.08	61.56	7.12E-02	94.95	77.01	2.18E-01	56.48	34.86	1.05E-01
cg04737087	-13.80	4.42	1.80E-03	-13.38	5.99	2.54E-02	-7.20	11.25	5.22E-01	-4.62	15.03	7.58E-01	-23.39	9.58	1.46E-02
cg04724659	-9.69	3.10	1.80E-03	-8.27	5.59	1.39E-01	-6.46	9.89	5.14E-01	-14.67	4.70	1.80E-03	-0.64	7.85	9.35E-01
cg25332717	-23.88	7.65	1.80E-03	-41.63	13.53	2.09E-03	-12.05	16.12	4.55E-01	-13.89	14.00	3.21E-01	-23.70	19.37	2.21E-01
cg23665824	32.26	10.34	1.80E-03	20.31	26.69	4.47E-01	36.18	46.24	4.34E-01	34.05	12.50	6.46E-03	35.41	30.27	2.42E-01
cg01643690	-17.93	5.75	1.81E-03	-19.90	8.82	2.41E-02	-19.68	14.21	1.66E-01	-28.84	14.14	4.14E-02	-6.09	11.56	5.98E-01
cg11530213	-7.46	2.39	1.81E-03	-6.30	3.52	7.32E-02	-4.77	4.90	3.31E-01	-19.65	8.75	2.47E-02	-8.63	5.04	8.64E-02
cg22745355	96.18	30.83	1.81E-03	82.45	41.39	4.64E-02	312.62	165.29	5.86E-02	38.57	85.61	6.52E-01	123.12	58.18	3.43E-02
cg03865667	14.86	4.76	1.81E-03	19.69	7.46	8.28E-03	16.42	16.92	3.32E-01	13.53	8.37	1.06E-01	6.07	10.94	5.79E-01
cg16313807	-7.79	2.50	1.81E-03	-11.62	3.17	2.45E-04	-4.96	5.33	3.52E-01	-6.77	7.58	3.72E-01	-0.84	5.66	8.82E-01

cg09826364	6.57	2.11	1.81E-03	5.13	2.95	8.21E-02	5.90	5.19	2.56E-01	8.02	5.77	1.64E-01	9.96	4.80	3.80E-02
cg18182038	34.94	11.20	1.81E-03	41.44	14.15	3.40E-03	68.58	41.68	9.99E-02	-0.59	54.87	9.91E-01	15.56	21.99	4.79E-01
cg22545027	-56.40	18.08	1.81E-03	-47.01	22.51	3.68E-02	37.63	66.45	5.71E-01	-100.08	42.60	1.88E-02	-70.27	30.89	2.29E-02
cg01022939	21.87	7.01	1.82E-03	12.22	15.88	4.42E-01	-13.11	25.37	6.05E-01	27.14	9.03	2.64E-03	32.79	19.80	9.76E-02
cg10588705	29.22	9.37	1.82E-03	11.37	16.35	4.87E-01	38.22	18.28	3.66E-02	23.94	15.41	1.20E-01	60.29	23.26	9.54E-03
cg15357078	-52.71	16.90	1.82E-03	-77.72	23.40	8.94E-04	-17.72	71.20	8.03E-01	-31.09	38.16	4.15E-01	-22.41	35.57	5.29E-01
cg00893348	-71.86	23.05	1.82E-03	-86.87	30.22	4.05E-03	40.55	88.90	6.48E-01	-104.62	58.67	7.45E-02	-40.28	51.92	4.38E-01
cg08582957	22.85	7.33	1.83E-03	25.52	13.07	5.09E-02	31.51	14.43	2.89E-02	25.50	11.61	2.80E-02	-14.47	22.56	5.21E-01
cg15164472	-11.94	3.83	1.83E-03	-7.00	5.99	2.43E-01	-21.20	8.42	1.18E-02	-12.36	8.66	1.54E-01	-12.01	8.79	1.72E-01
cg19876649	-13.72	4.40	1.83E-03	-18.78	9.22	4.18E-02	-5.15	15.93	7.47E-01	-12.16	5.89	3.89E-02	-16.47	11.92	1.67E-01
cg15491134	-11.92	3.83	1.84E-03	-18.29	7.97	2.16E-02	-10.71	12.23	3.82E-01	-9.22	5.21	7.66E-02	-12.73	10.55	2.28E-01
cg02223028	21.36	6.86	1.84E-03	21.19	20.19	2.94E-01	41.54	31.75	1.91E-01	18.74	7.78	1.60E-02	39.49	27.70	1.54E-01
cg02913553	14.34	4.60	1.84E-03	14.34	6.60	2.98E-02	26.92	11.39	1.81E-02	3.04	13.14	8.17E-01	11.39	9.64	2.37E-01
cg22335802	15.78	5.07	1.84E-03	14.56	7.05	3.88E-02	9.83	10.35	3.42E-01	25.35	17.90	1.57E-01	23.72	12.54	5.86E-02
cg25620356	27.71	8.90	1.84E-03	39.70	11.81	7.71E-04	16.13	28.35	5.69E-01	31.31	33.46	3.49E-01	5.17	17.36	7.66E-01
cg02266878	-18.80	6.04	1.84E-03	-26.54	9.74	6.44E-03	-5.51	17.39	7.51E-01	-16.45	12.34	1.82E-01	-15.65	11.92	1.89E-01
cg09699787	11.44	3.67	1.84E-03	13.25	5.62	1.84E-02	12.41	8.47	1.43E-01	7.37	10.56	4.85E-01	9.68	7.13	1.75E-01
cg13854962	-24.36	7.83	1.86E-03	-37.04	12.25	2.50E-03	-11.60	17.19	5.00E-01	-19.45	21.77	3.72E-01	-16.95	15.49	2.74E-01
cg13534734	32.27	10.37	1.86E-03	26.59	14.85	7.33E-02	19.90	38.78	6.08E-01	46.59	52.11	3.71E-01	39.95	16.36	1.46E-02
cg20276402	10.22	3.28	1.86E-03	10.63	5.01	3.40E-02	5.59	5.75	3.30E-01	12.00	16.24	4.60E-01	16.39	7.27	2.42E-02
cg14602640	-37.23	11.97	1.86E-03	-29.73	52.18	5.69E-01	-97.54	215.48	6.51E-01	-35.74	12.57	4.45E-03	-78.81	61.80	2.02E-01
cg10969550	22.93	7.37	1.87E-03	53.14	20.18	8.46E-03	36.61	36.82	3.20E-01	18.06	8.49	3.34E-02	10.46	27.38	7.03E-01
cg08230332	30.27	9.73	1.87E-03	67.36	30.31	2.62E-02	12.61	71.37	8.60E-01	23.67	11.81	4.50E-02	35.25	21.82	1.06E-01
cg16462297	-18.26	5.87	1.87E-03	-18.63	16.28	2.53E-01	-21.92	18.79	2.43E-01	-16.68	7.07	1.83E-02	-26.52	20.40	1.94E-01
cg08250081	12.14	3.91	1.88E-03	9.43	5.65	9.50E-02	20.87	9.21	2.34E-02	8.74	9.21	3.43E-01	14.24	9.70	1.42E-01
cg11060907	40.05	12.88	1.88E-03	36.92	16.83	2.82E-02	19.71	48.31	6.83E-01	33.14	32.05	3.01E-01	64.36	30.28	3.36E-02
cg13546977	-33.97	10.93	1.88E-03	-51.02	19.69	9.57E-03	7.70	28.16	7.85E-01	-37.61	19.26	5.09E-02	-33.31	23.32	1.53E-01
cg14491820	20.30	6.53	1.88E-03	26.97	19.52	1.67E-01	23.21	42.61	5.86E-01	20.61	7.50	5.98E-03	10.35	20.09	6.06E-01
cg10136168	17.44	5.61	1.89E-03	15.94	7.72	3.89E-02	17.82	12.42	1.51E-01	24.74	16.86	1.42E-01	16.81	14.17	2.35E-01
cg09640425	4.55	1.46	1.89E-03	2.45	2.25	2.78E-01	6.88	2.87	1.66E-02	4.62	4.83	3.38E-01	5.79	3.09	6.11E-02
cg07472772	35.83	11.53	1.89E-03	38.51	26.74	1.50E-01	73.62	68.00	2.79E-01	34.28	13.98	1.42E-02	30.73	35.64	3.89E-01
cg07123811	54.23	17.45	1.89E-03	56.95	20.80	6.18E-03	19.71	93.15	8.32E-01	13.43	55.60	8.09E-01	74.69	43.34	8.48E-02
cg26482164	16.66	5.36	1.89E-03	22.00	7.92	5.48E-03	9.54	15.58	5.40E-01	21.05	10.34	4.17E-02	-1.42	13.67	9.17E-01
cg12513379	-9.97	3.21	1.89E-03	-8.14	5.04	1.06E-01	-9.24	6.72	1.69E-01	-10.90	7.81	1.63E-01	-13.79	7.22	5.63E-02
cg12134633	8.48	2.73	1.89E-03	8.36	4.22	4.79E-02	9.15	4.91	6.23E-02	12.58	9.96	2.06E-01	6.14	6.15	3.18E-01

cg19955318	39.95	12.86	1.90E-03	43.73	18.53	1.83E-02	27.70	39.29	4.81E-01	31.49	27.11	2.46E-01	47.46	29.81	1.11E-01
cg02944123	-13.31	4.29	1.90E-03	-16.06	8.99	7.41E-02	-18.51	16.76	2.69E-01	-14.98	5.65	8.08E-03	1.20	11.77	9.19E-01
cg08456993	-16.26	5.24	1.91E-03	-32.12	16.84	5.64E-02	0.03	21.41	9.99E-01	-14.93	5.98	1.26E-02	-22.22	18.86	2.39E-01
cg11173131	9.87	3.18	1.91E-03	10.89	4.10	7.96E-03	12.18	8.44	1.49E-01	3.69	10.10	7.15E-01	7.79	7.96	3.28E-01
cg25065716	-6.26	2.02	1.91E-03	-4.69	3.23	1.47E-01	-4.20	4.48	3.48E-01	-6.26	3.94	1.12E-01	-13.17	5.24	1.20E-02
cg25092247	25.33	8.16	1.92E-03	26.09	31.14	4.02E-01	6.91	125.40	9.56E-01	27.42	8.73	1.69E-03	-8.62	35.41	8.08E-01
cg01577604	20.68	6.66	1.92E-03	21.58	9.14	1.82E-02	23.49	14.80	1.13E-01	-8.04	20.83	6.99E-01	32.28	16.52	5.07E-02
cg19872335	30.62	9.87	1.92E-03	26.20	10.78	1.51E-02	74.10	49.58	1.35E-01	68.99	40.62	8.94E-02	26.12	39.44	5.08E-01
cg07002382	-18.27	5.89	1.92E-03	-27.78	17.11	1.04E-01	-31.05	58.42	5.95E-01	-16.59	6.53	1.11E-02	-20.18	24.38	4.08E-01
cg11053466	-18.08	5.83	1.93E-03	-23.76	9.23	1.00E-02	-7.56	13.58	5.78E-01	-24.76	14.89	9.63E-02	-12.94	11.36	2.54E-01
cg01996325	30.00	9.68	1.94E-03	19.67	22.70	3.86E-01	44.90	51.33	3.82E-01	29.41	11.64	1.15E-02	49.30	32.06	1.24E-01
cg14650577	31.48	10.15	1.94E-03	40.85	35.23	2.46E-01	17.83	142.63	9.01E-01	31.43	11.07	4.51E-03	21.89	38.35	5.68E-01
cg22971939	-20.24	6.53	1.94E-03	-25.48	13.95	6.78E-02	-5.91	17.57	7.37E-01	-24.67	9.60	1.02E-02	-13.48	15.37	3.80E-01
cg22051199	-20.32	6.56	1.94E-03	-27.76	11.63	1.70E-02	-17.02	24.95	4.95E-01	-10.94	9.26	2.37E-01	-43.08	19.55	2.76E-02
cg05595301	13.60	4.39	1.94E-03	13.12	7.00	6.10E-02	18.81	9.43	4.60E-02	9.10	9.85	3.55E-01	13.34	9.99	1.82E-01
cg27582986	-68.70	22.17	1.94E-03	-61.44	33.04	6.30E-02	129.93	128.48	3.12E-01	-100.68	43.35	2.02E-02	-71.87	43.58	9.91E-02
cg10806318	-20.26	6.54	1.94E-03	-31.16	9.71	1.33E-03	-4.98	17.63	7.78E-01	-12.31	16.65	4.60E-01	-13.95	12.94	2.81E-01
cg14402955	20.88	6.74	1.95E-03	24.83	12.82	5.28E-02	22.41	13.73	1.03E-01	18.05	10.56	8.74E-02	16.75	24.42	4.93E-01
cg08902940	-11.32	3.66	1.95E-03	-11.29	5.79	5.11E-02	-15.96	8.01	4.63E-02	-14.91	8.25	7.08E-02	-2.90	8.24	7.25E-01
cg26744490	-59.18	19.11	1.95E-03	-76.93	51.96	1.39E-01	-358.42	251.26	1.54E-01	-62.05	21.89	4.58E-03	5.96	61.33	9.23E-01
cg03041030	45.19	14.59	1.96E-03	52.17	19.26	6.75E-03	72.02	42.20	8.79E-02	-9.49	36.09	7.93E-01	57.23	38.61	1.38E-01
cg02838492	10.44	3.37	1.96E-03	7.95	4.72	9.19E-02	8.83	6.73	1.90E-01	6.89	13.25	6.03E-01	21.39	8.09	8.19E-03
cg10893908	-12.85	4.15	1.96E-03	-17.18	6.25	5.96E-03	-13.92	9.52	1.44E-01	-10.08	11.83	3.94E-01	-5.62	8.38	5.03E-01
cg25937790	-12.46	4.02	1.96E-03	-9.42	7.82	2.28E-01	-18.15	17.00	2.86E-01	-11.36	5.32	3.29E-02	-22.77	12.25	6.30E-02
cg03341469	-9.72	3.14	1.97E-03	-10.11	4.24	1.70E-02	-10.38	6.68	1.20E-01	-16.51	21.25	4.37E-01	-7.26	6.89	2.92E-01
cg11012980	46.63	15.07	1.97E-03	57.90	55.22	2.94E-01	76.12	244.91	7.56E-01	48.11	16.14	2.87E-03	2.10	67.17	9.75E-01
cg14213692	-12.58	4.06	1.97E-03	-7.55	5.97	2.06E-01	-8.05	9.47	3.95E-01	-16.58	13.05	2.04E-01	-23.41	8.03	3.57E-03
cg20195319	-19.30	6.23	1.97E-03	-16.64	18.58	3.70E-01	-6.64	34.47	8.47E-01	-20.54	7.01	3.38E-03	-15.03	24.76	5.44E-01
cg11149658	36.09	11.66	1.97E-03	45.79	17.21	7.79E-03	55.92	23.43	1.70E-02	11.50	35.19	7.44E-01	4.36	25.66	8.65E-01
cg14290450	-14.26	4.61	1.97E-03	-25.99	12.65	3.99E-02	-5.57	21.92	7.99E-01	-12.23	5.35	2.23E-02	-18.24	16.06	2.56E-01
cg00645339	-58.87	19.02	1.97E-03	3.73	47.47	9.37E-01	-217.16	257.27	3.99E-01	-75.92	22.25	6.45E-04	-27.13	59.26	6.47E-01
cg20826024	-20.34	6.57	1.98E-03	-7.48	14.66	6.10E-01	-21.55	20.99	3.05E-01	-22.78	8.36	6.46E-03	-31.89	22.80	1.62E-01
cg15516558	8.49	2.74	1.99E-03	7.07	3.59	4.88E-02	13.77	6.08	2.35E-02	17.15	8.96	5.55E-02	0.25	7.60	9.74E-01
cg15929797	11.14	3.60	1.99E-03	16.75	6.11	6.09E-03	-1.04	8.48	9.02E-01	14.74	5.86	1.20E-02	7.94	6.91	2.51E-01
cg00270974	-22.66	7.33	2.00E-03	-23.80	14.73	1.06E-01	0.32	37.85	9.93E-01	-27.80	9.36	2.97E-03	2.85	23.08	9.02E-01

cg05937687	-16.42	5.31	2.00E-03	-30.22	8.00	1.58E-04	-18.20	11.95	1.28E-01	-10.10	4.55	2.65E-02	-9.75	10.99	3.75E-01
cg13244315	-37.76	12.23	2.02E-03	-50.95	20.73	1.40E-02	49.90	83.00	5.48E-01	-35.32	19.62	7.18E-02	-30.56	24.88	2.19E-01
cg17907457	-16.89	5.47	2.02E-03	-12.47	12.04	3.00E-01	-16.84	17.70	3.41E-01	-15.09	6.89	2.85E-02	-47.88	21.24	2.41E-02
cg11010909	-22.94	7.43	2.02E-03	-29.08	10.69	6.51E-03	9.05	23.29	6.98E-01	-37.86	20.78	6.85E-02	-17.29	13.86	2.12E-01
cg11664652	20.46	6.63	2.03E-03	35.54	20.14	7.77E-02	32.91	24.15	1.73E-01	17.13	7.58	2.38E-02	19.92	29.19	4.95E-01
cg18608389	38.10	12.35	2.03E-03	12.92	47.30	7.85E-01	184.99	179.85	3.04E-01	42.62	13.41	1.48E-03	2.77	43.75	9.50E-01
cg10022248	-17.29	5.61	2.04E-03	-20.01	8.20	1.47E-02	-24.59	14.51	9.02E-02	-22.15	12.70	8.12E-02	0.25	12.92	9.85E-01
cg05148217	-61.74	20.02	2.04E-03	-54.94	28.07	5.03E-02	-87.24	73.97	2.38E-01	-104.73	51.81	4.33E-02	-43.80	38.59	2.56E-01
cg10506549	70.22	22.77	2.04E-03	86.49	70.09	2.17E-01	-92.63	307.76	7.63E-01	78.83	25.09	1.68E-03	-50.14	88.77	5.72E-01
cg25694447	38.04	12.34	2.04E-03	28.31	18.46	1.25E-01	36.86	31.58	2.43E-01	41.90	36.69	2.53E-01	52.25	22.99	2.30E-02
cg00238662	32.94	10.68	2.05E-03	24.67	20.38	2.26E-01	42.97	21.24	4.30E-02	41.13	19.55	3.54E-02	17.32	25.64	4.99E-01
cg27528222	-44.23	14.35	2.05E-03	-50.77	25.73	4.85E-02	-105.70	70.41	1.33E-01	-57.35	23.74	1.57E-02	-11.04	27.00	6.83E-01
cg09018441	37.06	12.02	2.05E-03	57.82	28.53	4.27E-02	-12.58	117.63	9.15E-01	34.64	14.02	1.35E-02	19.04	43.38	6.61E-01
cg02385474	-17.66	5.73	2.05E-03	-20.01	10.49	5.65E-02	-0.34	12.88	9.79E-01	-21.08	10.26	3.99E-02	-26.32	13.08	4.42E-02
cg11486935	8.40	2.72	2.06E-03	7.94	5.10	1.19E-01	11.94	6.24	5.56E-02	8.86	4.14	3.25E-02	0.19	9.04	9.83E-01
cg11470337	-23.06	7.48	2.06E-03	-26.91	13.09	3.98E-02	4.75	22.51	8.33E-01	-24.42	13.44	6.93E-02	-28.55	14.86	5.48E-02
cg02059950	-22.87	7.42	2.06E-03	-10.48	18.91	5.80E-01	21.20	31.30	4.98E-01	-29.46	8.70	7.08E-04	-18.47	26.90	4.92E-01
cg11204139	8.42	2.73	2.06E-03	6.37	3.49	6.84E-02	14.00	5.62	1.27E-02	3.40	12.18	7.80E-01	10.27	8.57	2.31E-01
cg15165676	16.21	5.26	2.06E-03	14.06	6.72	3.64E-02	10.48	12.75	4.11E-01	2.86	25.23	9.10E-01	32.77	12.63	9.49E-03
cg14486995	-16.61	5.39	2.07E-03	-19.65	16.76	2.41E-01	19.47	21.54	3.66E-01	-18.98	6.16	2.08E-03	-18.55	20.59	3.68E-01
cg27036456	-12.16	3.95	2.07E-03	-11.45	10.28	2.65E-01	-33.04	24.05	1.69E-01	-11.74	4.54	9.66E-03	-10.07	14.98	5.01E-01
cg12996171	24.80	8.05	2.07E-03	18.58	11.08	9.36E-02	44.29	16.50	7.28E-03	16.54	27.79	5.52E-01	20.40	20.83	3.27E-01
cg24364144	18.34	5.96	2.08E-03	30.93	14.84	3.71E-02	12.96	27.84	6.41E-01	16.29	7.15	2.27E-02	14.69	18.96	4.39E-01
cg08223717	17.16	5.57	2.08E-03	23.24	11.39	4.12E-02	13.80	20.03	4.91E-01	18.94	7.21	8.61E-03	-9.32	19.08	6.25E-01
cg01970336	-17.08	5.55	2.08E-03	-15.08	8.42	7.34E-02	-31.65	12.94	1.44E-02	-2.27	14.42	8.75E-01	-18.72	11.47	1.03E-01
cg05578937	-56.52	18.36	2.08E-03	-60.51	23.15	8.95E-03	-167.57	79.82	3.58E-02	-35.00	71.01	6.22E-01	-28.87	36.61	4.30E-01
cg24059197	54.86	17.82	2.09E-03	64.46	45.46	1.56E-01	-155.42	177.27	3.81E-01	56.09	20.55	6.35E-03	51.58	61.50	4.02E-01
cg09198866	24.07	7.82	2.10E-03	36.60	21.18	8.41E-02	8.13	28.65	7.77E-01	23.27	9.09	1.05E-02	25.60	35.71	4.73E-01
cg09637363	21.37	6.95	2.10E-03	14.01	11.51	2.23E-01	35.49	12.77	5.45E-03	17.74	16.93	2.95E-01	16.20	16.83	3.36E-01
cg07919901	-44.59	14.50	2.10E-03	-51.35	16.03	1.36E-03	-3.33	108.34	9.75E-01	-38.35	82.83	6.43E-01	-10.11	39.70	7.99E-01
cg23690350	15.65	5.09	2.10E-03	20.99	7.90	7.92E-03	22.73	14.71	1.22E-01	13.97	9.35	1.35E-01	0.52	12.37	9.67E-01
cg01070903	21.37	6.95	2.10E-03	18.85	11.44	9.95E-02	24.73	15.19	1.04E-01	11.73	14.89	4.31E-01	32.84	15.41	3.30E-02
cg00898920	-19.00	6.18	2.11E-03	-24.43	8.90	6.04E-03	-4.18	14.30	7.70E-01	-6.04	18.04	7.38E-01	-26.77	13.35	4.50E-02
cg01140247	-11.82	3.84	2.11E-03	-12.58	6.32	4.65E-02	-7.60	10.00	4.47E-01	-13.78	7.06	5.09E-02	-10.52	8.92	2.38E-01
cg16383871	30.00	9.76	2.11E-03	32.49	15.89	4.08E-02	23.04	18.74	2.19E-01	29.90	23.17	1.97E-01	35.51	23.37	1.29E-01

cg08802607	20.27	6.60	2.12E-03	32.99	7.87	2.77E-05	7.82	12.21	5.22E-01	12.55	15.91	4.30E-01	14.62	12.26	2.33E-01
cg14091677	22.40	7.29	2.12E-03	7.50	19.81	7.05E-01	55.80	29.73	6.05E-02	22.32	8.43	8.07E-03	23.76	31.08	4.44E-01
cg03275648	-18.31	5.96	2.13E-03	-13.11	15.16	3.87E-01	-10.97	18.20	5.47E-01	-21.13	7.46	4.61E-03	-16.23	18.93	3.91E-01
cg05102288	28.47	9.27	2.13E-03	45.06	21.23	3.38E-02	-55.24	78.31	4.81E-01	28.64	10.88	8.49E-03	-1.82	35.08	9.59E-01
cg19480470	-16.88	5.50	2.13E-03	-4.52	12.45	7.16E-01	2.51	30.87	9.35E-01	-19.27	6.58	3.39E-03	-34.78	20.00	8.21E-02
cg21731304	-10.93	3.56	2.14E-03	-22.83	9.09	1.20E-02	-13.45	17.61	4.45E-01	-8.88	4.22	3.53E-02	-5.90	11.67	6.13E-01
cg25439867	-14.63	4.77	2.14E-03	-16.62	9.07	6.68E-02	7.86	44.58	8.60E-01	-16.29	6.17	8.29E-03	-3.56	14.01	8.00E-01
cg16891104	18.62	6.06	2.14E-03	13.74	8.96	1.25E-01	19.58	14.00	1.62E-01	37.44	16.06	1.97E-02	15.65	13.20	2.36E-01
cg24150325	-11.65	3.79	2.14E-03	-11.89	9.70	2.20E-01	-14.71	14.44	3.08E-01	-11.68	4.55	1.03E-02	-8.39	13.07	5.21E-01
cg22994849	9.72	3.17	2.15E-03	18.83	7.18	8.70E-03	12.42	7.80	1.12E-01	5.99	4.22	1.56E-01	8.19	11.37	4.71E-01
cg18361093	-13.93	4.54	2.15E-03	-14.33	5.85	1.43E-02	-1.89	11.32	8.68E-01	-4.26	15.37	7.82E-01	-26.39	9.99	8.25E-03
cg10333311	11.70	3.81	2.15E-03	17.09	5.98	4.29E-03	5.72	8.36	4.94E-01	2.37	10.89	8.27E-01	12.44	7.42	9.36E-02
cg18861231	-17.21	5.61	2.15E-03	-17.21	10.59	1.04E-01	-5.88	19.68	7.65E-01	-16.75	7.81	3.19E-02	-26.72	16.06	9.62E-02
cg02328578	-16.61	5.41	2.15E-03	-36.08	12.96	5.35E-03	-8.53	23.29	7.14E-01	-11.97	6.54	6.73E-02	-19.03	18.32	2.99E-01
cg16496462	42.53	13.86	2.15E-03	52.47	20.95	1.23E-02	56.76	25.86	2.82E-02	-10.59	35.85	7.68E-01	38.53	39.15	3.25E-01
cg06545180	-57.19	18.64	2.15E-03	-81.48	34.45	1.80E-02	-225.43	275.72	4.14E-01	-53.84	23.80	2.37E-02	8.22	62.47	8.95E-01
cg16759888	50.77	16.55	2.16E-03	40.49	39.73	3.08E-01	-97.19	133.88	4.68E-01	51.22	19.09	7.31E-03	112.68	67.59	9.55E-02
cg20489345	-28.66	9.35	2.16E-03	-8.15	23.96	7.34E-01	-98.90	120.59	4.12E-01	-34.39	10.63	1.21E-03	-3.38	35.74	9.25E-01
cg07198369	-20.76	6.77	2.17E-03	-7.43	18.56	6.89E-01	-45.61	30.03	1.29E-01	-20.82	7.87	8.18E-03	-26.89	24.52	2.73E-01
cg17863042	-37.35	12.19	2.17E-03	-50.91	37.23	1.71E-01	-190.18	185.71	3.06E-01	-30.68	13.56	2.37E-02	-77.67	42.76	6.93E-02
cg12289926	24.64	8.04	2.18E-03	17.62	13.15	1.80E-01	11.43	16.97	5.01E-01	25.02	17.12	1.44E-01	50.56	17.53	3.92E-03
cg03809403	24.71	8.06	2.18E-03	31.43	19.18	1.01E-01	-2.48	22.85	9.14E-01	26.64	10.27	9.44E-03	37.05	28.27	1.90E-01
cg02865242	29.13	9.51	2.19E-03	29.39	18.09	1.04E-01	34.52	19.62	7.85E-02	26.38	15.70	9.30E-02	26.39	27.24	3.33E-01
cg18814808	-10.91	3.56	2.19E-03	-15.41	7.56	4.14E-02	-4.48	7.48	5.49E-01	-13.08	5.43	1.61E-02	-6.99	10.22	4.94E-01
cg18156471	-12.51	4.08	2.20E-03	-14.31	8.17	7.97E-02	-10.25	13.02	4.31E-01	-11.69	5.66	3.88E-02	-13.98	11.28	2.15E-01
cg20502536	-10.09	3.29	2.20E-03	-15.06	5.29	4.44E-03	-3.12	6.98	6.55E-01	-8.63	6.60	1.91E-01	-10.02	8.79	2.54E-01
cg06234765	-20.45	6.68	2.20E-03	-25.02	18.52	1.77E-01	2.69	38.86	9.45E-01	-22.53	7.64	3.21E-03	-1.08	23.99	9.64E-01
cg11224783	-11.73	3.83	2.21E-03	-18.14	8.27	2.83E-02	-5.58	13.96	6.89E-01	-10.62	5.05	3.56E-02	-9.68	10.45	3.54E-01
cg14426682	35.83	11.71	2.21E-03	56.69	23.88	1.76E-02	51.29	46.41	2.69E-01	24.16	15.05	1.09E-01	47.41	38.67	2.20E-01
cg26168400	-21.26	6.95	2.21E-03	-24.16	28.42	3.95E-01	-159.01	100.78	1.15E-01	-21.34	7.45	4.20E-03	-7.83	26.96	7.71E-01
cg16095155	-18.34	5.99	2.21E-03	-22.08	10.61	3.74E-02	-12.04	13.16	3.60E-01	-20.85	11.72	7.54E-02	-15.79	13.02	2.25E-01
cg08924203	-15.19	4.96	2.21E-03	-10.40	7.30	1.54E-01	-17.10	10.81	1.14E-01	-34.94	19.48	7.29E-02	-17.23	9.71	7.61E-02
cg18282099	-9.88	3.23	2.22E-03	-5.47	4.77	2.51E-01	-7.18	7.16	3.16E-01	-16.90	9.33	7.01E-02	-17.76	6.90	1.01E-02
cg14468055	-23.38	7.65	2.22E-03	-31.60	13.43	1.86E-02	3.29	18.19	8.57E-01	-30.68	14.79	3.80E-02	-23.80	15.87	1.34E-01
cg09391966	-77.09	25.22	2.24E-03	-92.95	33.20	5.12E-03	-59.74	109.86	5.87E-01	53.41	85.68	5.33E-01	-87.92	47.35	6.33E-02

cg02843349	28.16	9.21	2.24E-03	29.28	13.62	3.15E-02	25.16	24.14	2.97E-01	22.87	29.08	4.32E-01	29.68	16.94	7.97E-02
cg10452206	-73.68	24.11	2.25E-03	-66.25	31.93	3.80E-02	-89.83	120.70	4.57E-01	-19.73	70.11	7.78E-01	-110.42	46.27	1.70E-02
cg17910478	11.61	3.80	2.25E-03	6.25	4.89	2.01E-01	18.39	6.66	5.77E-03	19.33	8.34	2.05E-02	4.34	10.20	6.71E-01
cg00709652	-14.34	4.70	2.26E-03	-19.05	7.46	1.07E-02	-6.43	11.61	5.80E-01	-8.32	9.06	3.59E-01	-20.47	11.34	7.12E-02
cg19244640	36.11	11.83	2.27E-03	12.82	36.94	7.29E-01	-129.89	166.77	4.36E-01	42.32	12.99	1.13E-03	5.70	47.07	9.04E-01
cg09987231	22.33	7.32	2.30E-03	28.92	12.30	1.87E-02	27.10	13.65	4.71E-02	16.98	15.44	2.71E-01	3.41	20.11	8.65E-01
cg26475649	24.31	7.97	2.30E-03	22.67	10.26	2.71E-02	20.91	20.57	3.09E-01	46.20	38.46	2.30E-01	27.05	17.71	1.27E-01
cg17619082	-38.63	12.67	2.30E-03	-1.88	26.64	9.44E-01	-149.32	118.17	2.06E-01	-47.15	13.20	3.55E-04	-44.42	39.30	2.58E-01
cg18781032	-12.00	3.94	2.31E-03	-17.86	7.69	2.01E-02	7.93	12.56	5.28E-01	-12.61	5.28	1.68E-02	-13.01	10.14	1.99E-01
cg05280508	-23.00	7.55	2.31E-03	-23.41	21.78	2.83E-01	-40.13	38.25	2.94E-01	-21.98	8.58	1.04E-02	-24.10	29.24	4.10E-01
cg06800040	-44.98	14.76	2.32E-03	-68.60	22.95	2.80E-03	11.34	46.67	8.08E-01	-50.78	25.10	4.31E-02	-20.17	31.39	5.20E-01
cg00713275	27.76	9.12	2.32E-03	30.00	21.78	1.68E-01	37.60	23.57	1.11E-01	24.45	11.58	3.48E-02	31.09	38.47	4.19E-01
cg01688536	20.71	6.80	2.33E-03	-1.71	21.68	9.37E-01	16.55	46.48	7.22E-01	24.33	7.66	1.48E-03	14.47	22.56	5.21E-01
cg04011469	-10.87	3.57	2.33E-03	-18.95	8.02	1.82E-02	-17.57	15.17	2.47E-01	-7.65	4.48	8.78E-02	-11.54	10.71	2.81E-01
cg02295118	26.85	8.82	2.33E-03	-10.16	24.58	6.79E-01	10.55	81.42	8.97E-01	33.76	9.92	6.64E-04	19.39	33.57	5.63E-01
cg07464025	35.10	11.53	2.34E-03	2.77	22.77	9.03E-01	2.00	74.22	9.79E-01	45.10	13.56	8.85E-04	55.55	32.85	9.08E-02
cg13881452	28.90	9.49	2.34E-03	37.75	22.17	8.86E-02	17.75	21.93	4.18E-01	27.14	12.92	3.56E-02	44.74	31.81	1.60E-01
cg12661316	22.22	7.30	2.34E-03	22.03	8.13	6.76E-03	14.03	45.55	7.58E-01	44.45	53.85	4.09E-01	21.91	18.86	2.45E-01
cg19438750	-12.31	4.05	2.35E-03	-13.42	7.27	6.49E-02	-14.56	11.45	2.03E-01	-15.40	6.45	1.70E-02	-1.55	9.80	8.74E-01
cg26236177	19.38	6.37	2.35E-03	15.44	9.18	9.26E-02	10.42	17.97	5.62E-01	59.89	30.65	5.07E-02	23.03	10.78	3.26E-02
cg06799248	28.96	9.52	2.35E-03	16.06	20.43	4.32E-01	21.73	39.70	5.84E-01	27.45	11.84	2.04E-02	82.52	34.03	1.53E-02
cg05627029	-18.47	6.07	2.36E-03	-16.79	17.47	3.37E-01	-18.74	64.28	7.71E-01	-19.06	6.74	4.67E-03	-13.63	25.18	5.88E-01
cg09637367	26.68	8.77	2.36E-03	40.44	16.20	1.26E-02	27.51	32.15	3.92E-01	22.75	12.56	7.01E-02	11.60	23.06	6.15E-01
cg09390792	24.10	7.93	2.36E-03	22.13	17.12	1.96E-01	8.33	45.13	8.54E-01	25.51	9.68	8.42E-03	23.62	27.21	3.85E-01
cg09767602	21.91	7.21	2.37E-03	45.03	25.71	7.99E-02	16.29	24.38	5.04E-01	24.46	8.76	5.25E-03	2.46	18.21	8.93E-01
cg18857618	-12.85	4.23	2.38E-03	-24.35	11.29	3.10E-02	-17.97	18.74	3.37E-01	-8.99	5.08	7.69E-02	-19.67	12.39	1.12E-01
cg14520424	18.07	5.95	2.39E-03	29.37	23.69	2.15E-01	142.82	107.11	1.82E-01	15.88	6.38	1.28E-02	30.73	23.56	1.92E-01
cg18335796	15.63	5.15	2.39E-03	20.03	7.47	7.32E-03	26.06	10.80	1.59E-02	9.72	12.09	4.21E-01	1.26	10.88	9.08E-01
cg13860447	-14.27	4.70	2.39E-03	-12.75	10.04	2.04E-01	-13.53	21.45	5.28E-01	-12.48	5.95	3.58E-02	-27.77	14.20	5.05E-02
cg02821464	-17.83	5.87	2.39E-03	-23.02	9.78	1.86E-02	-10.62	12.59	3.99E-01	-26.15	13.50	5.27E-02	-9.78	12.18	4.22E-01
cg17692702	18.04	5.94	2.41E-03	18.20	13.32	1.72E-01	18.14	14.06	1.97E-01	14.05	8.35	9.23E-02	35.27	17.54	4.44E-02
cg11687406	59.51	19.61	2.41E-03	37.04	44.57	4.06E-01	-133.53	193.87	4.91E-01	66.24	23.19	4.29E-03	78.04	68.70	2.56E-01
cg20164479	-14.49	4.78	2.41E-03	-27.51	11.89	2.06E-02	3.97	21.74	8.55E-01	-12.76	5.86	2.94E-02	-13.99	13.45	2.98E-01
cg01667946	32.63	10.75	2.41E-03	58.65	27.03	3.00E-02	-2.30	75.18	9.76E-01	29.98	12.62	1.76E-02	17.12	34.80	6.23E-01
cg26441877	9.49	3.13	2.41E-03	8.03	4.61	8.15E-02	8.43	6.30	1.81E-01	15.86	8.85	7.31E-02	10.35	7.65	1.76E-01

cg26564040	19.24	6.34	2.42E-03	18.88	14.72	2.00E-01	7.86	71.40	9.12E-01	19.76	7.39	7.52E-03	16.07	23.91	5.02E-01
cg05746617	28.51	9.40	2.42E-03	32.08	16.69	5.45E-02	24.01	15.89	1.31E-01	32.52	22.88	1.55E-01	27.08	23.19	2.43E-01
cg10135753	20.06	6.62	2.43E-03	15.82	9.07	8.13E-02	35.35	15.22	2.02E-02	12.31	22.88	5.91E-01	20.16	14.97	1.78E-01
cg19869698	23.46	7.74	2.43E-03	22.84	12.24	6.21E-02	28.70	14.10	4.18E-02	30.48	19.31	1.14E-01	5.76	20.76	7.81E-01
cg16124562	14.12	4.66	2.43E-03	19.36	9.63	4.45E-02	6.71	16.38	6.82E-01	15.00	6.11	1.41E-02	3.31	14.34	8.18E-01
cg20744304	37.13	12.25	2.43E-03	23.02	25.70	3.70E-01	-97.78	174.05	5.74E-01	41.12	14.88	5.72E-03	50.01	40.63	2.18E-01
cg04920214	-18.56	6.12	2.43E-03	-28.78	17.33	9.68E-02	-20.31	37.96	5.93E-01	-17.72	6.91	1.03E-02	-8.15	24.29	7.37E-01
cg22267908	21.41	7.06	2.43E-03	18.94	16.02	2.37E-01	-29.16	60.50	6.30E-01	24.47	8.34	3.34E-03	7.72	25.78	7.65E-01
cg01991934	19.54	6.45	2.43E-03	36.40	15.74	2.08E-02	22.53	18.20	2.16E-01	12.92	8.48	1.28E-01	24.36	17.93	1.74E-01
cg22521310	32.20	10.62	2.43E-03	37.94	16.95	2.52E-02	-0.42	27.47	9.88E-01	24.83	25.90	3.38E-01	45.52	19.73	2.11E-02
cg16714055	31.92	10.53	2.44E-03	75.39	29.82	1.15E-02	45.17	65.81	4.92E-01	23.87	12.72	6.06E-02	30.45	25.99	2.41E-01
cg24952102	-23.35	7.70	2.44E-03	-27.39	14.35	5.62E-02	-17.18	20.39	4.00E-01	-24.05	11.55	3.73E-02	-18.55	21.88	3.97E-01
cg13139843	-11.43	3.77	2.44E-03	-13.39	7.80	8.57E-02	-6.15	15.39	6.89E-01	-11.22	5.01	2.51E-02	-11.29	10.15	2.66E-01
cg25264268	7.37	2.43	2.45E-03	7.71	3.19	1.58E-02	12.22	4.99	1.43E-02	2.52	10.74	8.15E-01	-1.09	6.75	8.72E-01
cg08650784	-12.82	4.23	2.45E-03	-17.35	6.38	6.51E-03	-7.34	9.28	4.29E-01	-0.37	10.86	9.73E-01	-18.03	9.48	5.71E-02
cg11725581	6.45	2.13	2.45E-03	5.18	2.97	8.17E-02	7.62	4.70	1.05E-01	8.08	4.64	8.16E-02	7.46	7.98	3.50E-01
cg01328473	12.20	4.03	2.45E-03	13.99	5.75	1.50E-02	19.28	9.22	3.64E-02	11.57	12.03	3.36E-01	1.72	8.88	8.46E-01
cg00453808	27.36	9.03	2.46E-03	39.77	15.92	1.25E-02	10.76	17.12	5.29E-01	14.09	18.47	4.45E-01	48.63	21.40	2.31E-02
cg04109092	11.41	3.77	2.46E-03	10.79	5.18	3.75E-02	14.69	8.02	6.68E-02	-7.20	13.23	5.86E-01	17.92	9.13	4.97E-02
cg12092716	-12.52	4.14	2.47E-03	-15.55	5.77	7.08E-03	-16.00	9.15	8.05E-02	-7.31	15.31	6.33E-01	-3.53	9.03	6.96E-01
cg08524015	37.98	12.55	2.48E-03	41.93	18.15	2.09E-02	42.09	34.27	2.19E-01	34.15	32.98	3.00E-01	30.21	25.44	2.35E-01
cg05062489	-13.69	4.52	2.48E-03	-17.32	7.44	1.99E-02	-11.08	9.90	2.63E-01	-11.40	10.86	2.94E-01	-12.07	9.08	1.84E-01
cg11616271	33.83	11.18	2.48E-03	36.71	27.51	1.82E-01	25.90	114.34	8.21E-01	37.64	12.94	3.62E-03	-7.34	39.79	8.54E-01
cg04354002	3.14	1.04	2.48E-03	3.13	1.43	2.80E-02	2.15	2.29	3.48E-01	1.35	3.48	6.99E-01	5.25	2.48	3.44E-02
cg22305209	15.46	5.11	2.48E-03	11.66	7.42	1.16E-01	18.52	11.39	1.04E-01	3.12	18.47	8.66E-01	24.07	10.27	1.90E-02
cg01522095	-22.16	7.32	2.48E-03	-8.77	18.39	6.33E-01	28.23	49.57	5.69E-01	-27.42	8.43	1.15E-03	-10.84	28.57	7.04E-01
cg19539664	-21.91	7.24	2.49E-03	-4.55	19.37	8.14E-01	-58.08	40.50	1.52E-01	-27.40	7.52	2.70E-04	-2.06	19.26	9.15E-01
cg10320997	-13.31	4.40	2.49E-03	-11.68	9.06	1.97E-01	-15.29	13.36	2.52E-01	-14.48	6.06	1.68E-02	-9.79	12.31	4.27E-01
cg19594666	9.71	3.21	2.49E-03	6.88	4.59	1.33E-01	16.98	6.84	1.30E-02	2.38	9.70	8.07E-01	12.96	7.57	8.69E-02
cg06934654	-18.50	6.12	2.50E-03	-19.26	10.58	6.88E-02	-7.22	23.86	7.62E-01	-17.43	10.81	1.07E-01	-21.50	11.58	6.34E-02
cg16190575	25.30	8.37	2.50E-03	6.68	17.46	7.02E-01	39.15	19.02	3.96E-02	22.68	12.58	7.13E-02	45.82	22.86	4.50E-02
cg04560456	18.72	6.19	2.50E-03	17.65	8.40	3.56E-02	31.14	17.44	7.42E-02	24.66	34.99	4.81E-01	14.80	11.33	1.91E-01
cg20644426	25.45	8.42	2.50E-03	44.36	22.50	4.86E-02	65.15	80.70	4.19E-01	23.87	9.61	1.30E-02	2.63	29.46	9.29E-01
cg01537471	22.77	7.53	2.51E-03	1.35	20.71	9.48E-01	30.93	51.53	5.48E-01	25.44	8.69	3.43E-03	29.64	24.41	2.25E-01
cg08900511	21.06	6.97	2.51E-03	12.61	10.83	2.44E-01	25.04	13.45	6.25E-02	32.44	16.22	4.54E-02	23.47	19.08	2.19E-01

cg08942191	-11.66	3.86	2.51E-03	-13.14	10.16	1.96E-01	-5.08	18.44	7.83E-01	-12.04	4.60	8.91E-03	-9.90	11.65	3.95E-01
cg16907934	11.12	3.68	2.52E-03	10.84	5.60	5.31E-02	19.19	10.28	6.20E-02	13.76	6.69	3.98E-02	-1.29	9.93	8.97E-01
cg23053624	-26.20	8.67	2.52E-03	-35.30	23.22	1.28E-01	-0.73	71.69	9.92E-01	-25.32	9.88	1.04E-02	-23.28	31.44	4.59E-01
cg16573701	48.70	16.12	2.52E-03	46.01	22.06	3.70E-02	107.89	42.73	1.16E-02	29.33	46.05	5.24E-01	25.75	35.97	4.74E-01
cg00167710	-52.23	17.29	2.52E-03	0.74	55.05	9.89E-01	-148.44	224.30	5.08E-01	-60.77	19.04	1.42E-03	-18.46	64.98	7.76E-01
cg08881067	-66.19	21.92	2.53E-03	-71.70	25.69	5.25E-03	35.59	111.67	7.50E-01	-126.44	94.15	1.79E-01	-47.45	51.78	3.60E-01
cg19774694	28.02	9.28	2.53E-03	23.08	11.28	4.07E-02	-10.12	30.52	7.40E-01	65.30	41.40	1.15E-01	40.74	15.06	6.82E-03
cg20768445	-14.49	4.80	2.53E-03	-20.93	12.94	1.06E-01	7.43	20.10	7.12E-01	-13.28	5.66	1.89E-02	-28.60	16.27	7.88E-02
cg05989533	-7.40	2.45	2.54E-03	-9.94	3.40	3.44E-03	-8.95	5.66	1.14E-01	-9.66	8.88	2.77E-01	0.44	5.15	9.32E-01
cg17928799	-41.37	13.70	2.54E-03	-48.51	25.23	5.45E-02	-10.51	67.69	8.77E-01	-36.11	18.82	5.51E-02	-55.91	37.46	1.36E-01
cg15543551	10.76	3.56	2.54E-03	16.65	5.44	2.22E-03	7.10	9.78	4.67E-01	9.86	8.25	2.32E-01	3.31	7.11	6.41E-01
cg21751540	14.36	4.76	2.54E-03	14.87	8.49	7.97E-02	14.90	10.45	1.54E-01	5.11	8.82	5.63E-01	27.23	10.98	1.31E-02
cg09395844	-17.70	5.87	2.55E-03	-30.19	10.56	4.23E-03	-11.39	13.58	4.02E-01	-11.00	10.89	3.13E-01	-14.27	12.66	2.60E-01
cg16258017	36.09	11.96	2.55E-03	56.73	41.51	1.72E-01	262.87	172.61	1.28E-01	35.57	13.05	6.43E-03	3.26	44.55	9.42E-01
cg24742092	18.36	6.09	2.56E-03	22.29	14.22	1.17E-01	27.88	23.60	2.37E-01	13.14	8.38	1.17E-01	24.67	12.91	5.59E-02
cg27470486	-16.17	5.36	2.56E-03	-15.13	8.28	6.76E-02	-9.69	11.50	4.00E-01	-14.17	11.66	2.24E-01	-30.97	13.71	2.39E-02
cg25979180	-20.52	6.80	2.56E-03	-23.98	14.59	1.00E-01	8.53	35.60	8.11E-01	-23.79	8.44	4.81E-03	-1.48	22.04	9.46E-01
cg20278382	29.57	9.81	2.56E-03	23.20	18.80	2.17E-01	6.23	59.54	9.17E-01	33.95	12.60	7.03E-03	26.56	31.82	4.04E-01
cg16872172	25.91	8.59	2.56E-03	36.93	27.74	1.83E-01	27.29	139.89	8.45E-01	24.51	9.36	8.87E-03	27.86	35.39	4.31E-01
cg17142743	20.65	6.85	2.56E-03	3.36	19.64	8.64E-01	31.91	64.81	6.22E-01	23.37	7.78	2.68E-03	19.25	22.43	3.91E-01
cg19896198	-42.28	14.02	2.56E-03	-23.98	17.25	1.65E-01	-67.68	42.86	1.14E-01	-113.18	58.36	5.25E-02	-54.71	25.29	3.05E-02
cg05938285	39.34	13.04	2.57E-03	40.97	15.93	1.01E-02	44.89	50.42	3.73E-01	-10.35	62.12	8.68E-01	42.66	27.90	1.26E-01
cg15052217	26.16	8.68	2.57E-03	13.49	20.50	5.10E-01	45.48	20.96	3.00E-02	27.34	11.66	1.91E-02	8.46	28.04	7.63E-01
cg16845880	-13.26	4.40	2.58E-03	-22.45	8.60	9.02E-03	-4.99	16.12	7.57E-01	-9.07	5.89	1.24E-01	-18.42	13.51	1.73E-01
cg03021192	51.11	16.97	2.59E-03	56.52	49.21	2.51E-01	-18.97	135.75	8.89E-01	57.31	19.16	2.78E-03	-3.20	59.54	9.57E-01
cg23843505	-14.57	4.84	2.60E-03	-15.63	6.81	2.17E-02	-23.17	10.65	2.95E-02	-11.40	17.30	5.10E-01	-4.74	10.56	6.53E-01
cg09896120	48.66	16.16	2.60E-03	43.44	19.02	2.24E-02	54.94	91.25	5.47E-01	53.98	55.27	3.29E-01	67.99	40.23	9.11E-02
cg04742977	14.27	4.74	2.61E-03	18.44	7.68	1.63E-02	6.07	9.49	5.22E-01	15.07	11.56	1.92E-01	15.83	10.54	1.33E-01
cg25475843	-15.91	5.29	2.61E-03	-15.36	11.81	1.93E-01	-15.98	20.71	4.40E-01	-14.27	6.49	2.78E-02	-32.89	19.90	9.83E-02
cg18151134	-14.59	4.85	2.61E-03	-11.14	13.05	3.93E-01	2.10	24.16	9.31E-01	-14.43	5.56	9.38E-03	-35.45	19.65	7.12E-02
cg11891735	41.21	13.69	2.61E-03	52.25	20.00	8.97E-03	46.21	28.66	1.07E-01	34.31	41.59	4.09E-01	12.57	31.05	6.85E-01
cg26338757	-16.22	5.39	2.62E-03	-14.94	7.23	3.88E-02	1.55	12.58	9.02E-01	-29.05	10.57	6.00E-03	-19.02	11.02	8.43E-02
cg08591668	9.66	3.21	2.62E-03	3.09	5.53	5.76E-01	17.85	7.62	1.92E-02	11.60	5.18	2.50E-02	9.86	10.15	3.32E-01
cg06520369	22.77	7.57	2.62E-03	41.81	23.87	7.99E-02	34.72	26.34	1.87E-01	18.21	8.61	3.43E-02	37.10	36.25	3.06E-01
cg12636369	16.40	5.45	2.62E-03	21.53	9.00	1.67E-02	12.88	13.44	3.38E-01	10.84	11.78	3.57E-01	15.97	10.81	1.40E-01

cg09412707	-13.63	4.53	2.63E-03	-17.28	11.15	1.21E-01	-13.31	12.69	2.94E-01	-11.85	5.97	4.70E-02	-17.15	12.52	1.71E-01
cg22449302	40.16	13.35	2.63E-03	9.80	41.29	8.12E-01	187.60	179.16	2.95E-01	44.38	14.83	2.77E-03	26.80	47.33	5.71E-01
cg18192917	29.40	9.78	2.65E-03	51.56	18.98	6.59E-03	24.68	19.18	1.98E-01	15.51	18.07	3.91E-01	26.14	23.01	2.56E-01
cg17125990	17.82	5.93	2.66E-03	20.19	8.22	1.41E-02	22.43	15.55	1.49E-01	20.57	14.17	1.47E-01	2.84	14.86	8.49E-01
cg26221971	-60.44	20.12	2.67E-03	-47.83	27.14	7.80E-02	-68.84	74.38	3.55E-01	-31.74	59.27	5.92E-01	-97.22	39.33	1.34E-02
cg14742341	14.39	4.79	2.67E-03	7.54	8.31	3.64E-01	21.37	10.92	5.02E-02	14.82	7.64	5.25E-02	23.79	16.78	1.56E-01
cg03342215	-56.29	18.74	2.67E-03	-65.25	28.54	2.22E-02	23.11	75.01	7.58E-01	-82.68	41.37	4.56E-02	-41.92	34.17	2.20E-01
cg00996315	-13.15	4.38	2.68E-03	-13.02	6.80	5.55E-02	-13.00	8.84	1.41E-01	-11.17	14.13	4.29E-01	-14.32	8.88	1.07E-01
cg09695261	-90.66	30.19	2.68E-03	-92.50	43.98	3.54E-02	190.37	250.86	4.48E-01	-112.09	52.53	3.28E-02	-69.55	70.43	3.23E-01
cg13587180	-5.72	1.90	2.68E-03	-5.80	2.79	3.75E-02	-5.52	3.79	1.45E-01	-2.83	7.54	7.07E-01	-6.63	4.10	1.06E-01
cg27519016	17.71	5.90	2.68E-03	24.57	12.34	4.65E-02	34.49	14.49	1.73E-02	10.53	8.30	2.05E-01	10.57	18.59	5.70E-01
cg10233133	34.41	11.46	2.68E-03	34.83	15.25	2.24E-02	39.69	39.68	3.17E-01	29.16	37.57	4.38E-01	33.69	22.55	1.35E-01
cg07592723	61.17	20.38	2.69E-03	75.03	25.19	2.90E-03	-52.89	134.56	6.94E-01	40.45	68.14	5.53E-01	41.43	42.21	3.26E-01
cg14582400	17.71	5.90	2.69E-03	16.09	8.02	4.48E-02	4.36	16.71	7.94E-01	11.24	22.62	6.19E-01	28.96	11.45	1.15E-02
cg04792024	28.13	9.37	2.69E-03	36.32	20.23	7.26E-02	23.55	17.13	1.69E-01	14.77	15.74	3.48E-01	61.35	25.90	1.78E-02
cg19659215	-9.83	3.28	2.69E-03	-4.98	6.01	4.07E-01	-10.80	9.51	2.56E-01	-12.05	5.07	1.74E-02	-12.23	8.04	1.28E-01
cg16423738	-10.63	3.54	2.69E-03	-15.03	7.75	5.26E-02	-12.71	10.32	2.18E-01	-10.31	4.76	3.03E-02	-2.36	10.29	8.19E-01
cg21735093	24.62	8.21	2.70E-03	42.64	27.04	1.15E-01	-0.77	111.44	9.94E-01	20.17	8.93	2.38E-02	63.56	34.23	6.33E-02
cg25450333	-35.63	11.88	2.70E-03	-29.23	20.07	1.45E-01	-46.89	17.39	7.00E-03	13.64	49.79	7.84E-01	-34.00	33.44	3.09E-01
cg16672562	5.06	1.69	2.70E-03	6.54	2.40	6.50E-03	3.18	3.43	3.54E-01	2.40	5.26	6.48E-01	5.03	4.17	2.27E-01
cg25339966	24.32	8.11	2.70E-03	43.93	13.14	8.25E-04	9.97	16.37	5.42E-01	17.16	12.71	1.77E-01	18.00	23.14	4.37E-01
cg23049079	23.59	7.87	2.71E-03	27.30	13.67	4.58E-02	55.35	23.20	1.70E-02	15.88	11.32	1.60E-01	7.25	29.60	8.06E-01
cg25120705	-28.45	9.49	2.71E-03	-34.55	15.27	2.37E-02	-6.66	23.71	7.79E-01	-29.80	21.31	1.62E-01	-31.87	18.78	8.97E-02
cg03724229	8.10	2.70	2.72E-03	8.18	4.27	5.55E-02	14.60	6.36	2.17E-02	5.16	4.75	2.77E-01	5.46	8.75	5.33E-01
cg15640062	54.22	18.09	2.72E-03	57.26	22.40	1.06E-02	97.49	88.83	2.72E-01	7.79	83.11	9.25E-01	48.13	35.53	1.76E-01
cg11831988	13.39	4.47	2.72E-03	13.51	7.79	8.28E-02	7.44	16.01	6.42E-01	10.26	6.53	1.16E-01	28.50	12.64	2.41E-02
cg20585530	21.63	7.22	2.73E-03	47.06	18.13	9.42E-03	30.81	20.65	1.36E-01	17.40	10.14	8.62E-02	9.28	14.32	5.17E-01
cg02281662	-76.31	25.47	2.74E-03	-75.72	31.75	1.71E-02	51.35	130.90	6.95E-01	-151.07	64.58	1.93E-02	-36.88	63.14	5.59E-01
cg00986119	24.56	8.20	2.74E-03	33.20	16.04	3.85E-02	16.85	17.80	3.44E-01	26.61	18.29	1.46E-01	21.38	14.36	1.37E-01
cg00590324	24.86	8.30	2.75E-03	46.63	21.18	2.77E-02	15.28	30.03	6.11E-01	19.68	12.98	1.30E-01	23.50	13.81	8.89E-02
cg26265070	38.14	12.74	2.75E-03	-9.57	41.93	8.19E-01	110.72	163.99	5.00E-01	42.05	13.89	2.48E-03	49.13	51.34	3.39E-01
cg13785473	27.00	9.02	2.75E-03	25.34	11.56	2.84E-02	33.12	21.57	1.25E-01	38.56	32.08	2.29E-01	19.92	24.32	4.13E-01
cg25412230	-15.78	5.27	2.77E-03	-17.10	10.42	1.01E-01	-29.51	17.52	9.20E-02	-15.09	7.28	3.82E-02	-6.33	14.67	6.66E-01
cg01046309	28.17	9.41	2.77E-03	11.34	43.32	7.94E-01	-134.40	176.48	4.46E-01	28.94	10.02	3.88E-03	36.71	36.19	3.10E-01
cg27199976	-14.11	4.72	2.77E-03	-11.26	6.60	8.81E-02	-18.14	11.32	1.09E-01	-7.18	17.36	6.79E-01	-19.37	9.59	4.34E-02

cg00170536	-27.38	9.15	2.77E-03	-9.49	29.22	7.45E-01	-3.11	125.30	9.80E-01	-31.09	10.03	1.93E-03	-8.43	36.32	8.16E-01
cg07207682	41.29	13.80	2.77E-03	32.04	26.10	2.20E-01	-111.96	97.00	2.48E-01	50.91	17.60	3.83E-03	38.69	47.23	4.13E-01
cg01423964	46.45	15.53	2.78E-03	24.35	43.03	5.71E-01	-100.99	152.88	5.09E-01	49.65	17.38	4.27E-03	76.77	62.96	2.23E-01
cg17181362	-10.66	3.56	2.78E-03	-10.14	5.44	6.21E-02	-7.45	9.42	4.29E-01	-14.67	8.07	6.90E-02	-10.24	7.41	1.67E-01
cg15285436	6.17	2.06	2.78E-03	5.85	2.84	3.94E-02	9.30	5.04	6.49E-02	5.98	4.43	1.77E-01	2.61	6.90	7.05E-01
cg06468978	18.20	6.09	2.78E-03	25.09	10.12	1.31E-02	9.44	11.45	4.10E-01	15.85	13.92	2.55E-01	20.81	15.00	1.65E-01
cg12637676	-10.86	3.63	2.78E-03	-15.94	6.49	1.40E-02	-4.90	8.28	5.53E-01	-6.78	6.88	3.25E-01	-14.08	7.82	7.19E-02
cg25764464	48.32	16.16	2.79E-03	60.29	27.02	2.57E-02	67.08	49.30	1.74E-01	34.41	26.67	1.97E-01	41.26	39.46	2.96E-01
ch.9.2503392R	-24.77	8.28	2.79E-03	-21.29	12.82	9.68E-02	-30.90	14.79	3.67E-02	-41.56	41.81	3.20E-01	-19.86	17.29	2.51E-01
cg10192877	28.89	9.67	2.81E-03	25.72	15.72	1.02E-01	32.83	20.35	1.07E-01	26.08	21.13	2.17E-01	33.70	22.39	1.32E-01
cg18231614	24.06	8.05	2.81E-03	43.30	15.61	5.53E-03	11.54	20.38	5.71E-01	19.73	13.11	1.32E-01	16.40	17.98	3.62E-01
cg24128943	15.52	5.19	2.81E-03	8.30	14.55	5.68E-01	16.74	24.30	4.91E-01	16.68	5.93	4.91E-03	15.02	21.26	4.80E-01
cg24498587	97.39	32.60	2.82E-03	104.11	46.50	2.52E-02	167.73	99.95	9.33E-02	63.47	92.44	4.92E-01	73.73	61.88	2.33E-01
cg03134157	14.00	4.69	2.82E-03	27.04	10.50	1.00E-02	14.40	27.54	6.01E-01	12.42	5.93	3.63E-02	2.94	12.24	8.10E-01
cg09425228	-16.76	5.61	2.83E-03	-19.26	17.68	2.76E-01	-5.61	37.07	8.80E-01	-15.29	6.20	1.37E-02	-37.55	23.30	1.07E-01
cg25142954	-26.39	8.84	2.83E-03	-26.63	10.98	1.53E-02	-19.64	21.39	3.58E-01	-17.09	35.71	6.32E-01	-39.45	25.53	1.22E-01
cg17841421	30.20	10.11	2.83E-03	64.24	24.64	9.13E-03	52.86	43.47	2.24E-01	20.10	12.14	9.78E-02	30.72	35.04	3.81E-01
cg05076914	13.82	4.63	2.83E-03	15.92	7.07	2.42E-02	10.39	13.27	4.34E-01	15.22	10.05	1.30E-01	10.51	9.50	2.69E-01
cg14633721	16.98	5.69	2.84E-03	16.39	8.93	6.64E-02	23.80	10.65	2.55E-02	13.39	15.36	3.83E-01	9.92	13.74	4.70E-01
cg20888326	23.11	7.74	2.84E-03	30.10	9.72	1.95E-03	-11.83	27.25	6.64E-01	21.69	42.66	6.11E-01	16.86	15.44	2.75E-01
cg10930226	33.17	11.12	2.84E-03	20.90	19.23	2.77E-01	27.86	39.76	4.84E-01	29.73	16.63	7.38E-02	76.15	29.61	1.01E-02
cg13249789	41.12	13.78	2.84E-03	92.65	39.72	1.97E-02	112.96	197.92	5.68E-01	29.54	13.11	2.42E-02	77.22	47.59	1.05E-01
cg14663965	8.20	2.75	2.85E-03	9.51	3.83	1.30E-02	8.00	6.71	2.33E-01	17.52	12.67	1.67E-01	4.20	5.30	4.28E-01
cg07047808	14.71	4.93	2.86E-03	15.40	9.53	1.06E-01	-24.66	23.79	3.00E-01	17.16	6.54	8.68E-03	15.60	14.18	2.71E-01
cg25137687	20.82	6.98	2.87E-03	34.14	22.67	1.32E-01	-69.04	99.95	4.90E-01	22.13	7.61	3.62E-03	-12.71	29.12	6.62E-01
cg14507868	20.24	6.79	2.88E-03	37.99	15.58	1.47E-02	12.55	19.42	5.18E-01	21.15	9.90	3.27E-02	7.08	14.56	6.26E-01
cg15774543	-26.29	8.82	2.88E-03	-24.67	33.05	4.55E-01	139.72	136.27	3.05E-01	-27.86	9.47	3.27E-03	-16.69	36.86	6.51E-01
cg10024730	20.48	6.87	2.88E-03	22.07	10.36	3.32E-02	16.88	13.27	2.03E-01	25.43	18.34	1.66E-01	17.65	17.67	3.18E-01
cg03773731	30.85	10.35	2.88E-03	38.19	16.62	2.16E-02	6.54	25.06	7.94E-01	11.32	30.93	7.14E-01	41.43	18.03	2.16E-02
cg26981593	25.34	8.50	2.89E-03	39.55	17.69	2.54E-02	18.10	21.76	4.06E-01	17.87	12.08	1.39E-01	37.88	24.44	1.21E-01
cg15047774	16.25	5.45	2.89E-03	23.28	11.03	3.48E-02	33.35	19.30	8.40E-02	7.16	8.19	3.83E-01	20.32	11.31	7.25E-02
cg19095568	11.80	3.96	2.89E-03	15.32	6.88	2.59E-02	0.96	21.52	9.64E-01	15.50	6.29	1.38E-02	2.29	8.12	7.78E-01
cg13912027	16.53	5.55	2.89E-03	20.08	7.61	8.34E-03	16.22	16.89	3.37E-01	30.10	19.75	1.28E-01	6.15	10.46	5.57E-01
cg04870460	-14.84	4.98	2.89E-03	-7.90	6.53	2.27E-01	-15.29	11.71	1.92E-01	-3.69	26.87	8.91E-01	-26.59	8.42	1.59E-03
cg22233667	-16.05	5.39	2.90E-03	-25.81	14.11	6.74E-02	-10.29	24.08	6.69E-01	-14.86	6.37	1.96E-02	-12.83	18.18	4.80E-01

cg11760395	6.29	2.11	2.90E-03	7.41	3.48	3.34E-02	9.83	5.03	5.05E-02	3.54	3.57	3.21E-01	5.58	6.48	3.89E-01
cg27317439	17.10	5.74	2.90E-03	2.20	9.53	8.17E-01	28.31	15.52	6.82E-02	22.66	7.72	3.35E-03	21.02	12.69	9.76E-02
cg12157045	-28.61	9.61	2.90E-03	7.63	24.33	7.54E-01	-46.24	123.83	7.09E-01	-35.47	10.85	1.08E-03	-31.74	41.35	4.43E-01
cg06733602	-13.08	4.39	2.91E-03	-13.35	6.52	4.07E-02	-3.00	11.11	7.87E-01	-31.24	13.72	2.28E-02	-11.66	8.20	1.55E-01
cg05407442	-69.08	23.21	2.91E-03	-72.95	29.57	1.36E-02	139.46	149.72	3.52E-01	-0.40	90.05	9.96E-01	-93.55	42.82	2.89E-02
cg05601722	30.25	10.16	2.91E-03	46.97	16.67	4.84E-03	27.80	17.45	1.11E-01	36.72	18.36	4.55E-02	-6.89	23.94	7.73E-01
cg23986470	-32.76	11.00	2.91E-03	-13.36	18.45	4.69E-01	-132.90	175.63	4.49E-01	-39.10	14.36	6.47E-03	-85.11	47.76	7.47E-02
cg09529783	-19.85	6.67	2.92E-03	-33.68	11.50	3.39E-03	-18.33	17.12	2.84E-01	-9.90	10.65	3.53E-01	-15.44	19.27	4.23E-01
cg23928910	-27.61	9.28	2.93E-03	-20.39	22.41	3.63E-01	18.50	120.78	8.78E-01	-32.13	10.75	2.79E-03	-3.55	33.34	9.15E-01
cg06217494	33.59	11.29	2.93E-03	36.97	17.78	3.75E-02	33.70	27.10	2.14E-01	38.50	28.04	1.70E-01	25.22	22.11	2.54E-01
cg06382167	42.38	14.25	2.94E-03	52.11	19.55	7.68E-03	40.05	41.57	3.35E-01	77.65	43.98	7.75E-02	7.38	28.75	7.98E-01
cg12044693	15.62	5.25	2.95E-03	17.06	6.54	9.06E-03	11.56	18.75	5.37E-01	10.44	18.45	5.72E-01	14.65	11.92	2.19E-01
cg03532926	14.41	4.85	2.95E-03	21.21	9.04	1.89E-02	8.94	10.87	4.11E-01	11.78	10.01	2.39E-01	13.51	9.18	1.41E-01
cg09055988	-17.38	5.85	2.95E-03	-17.79	13.76	1.96E-01	-4.78	22.64	8.33E-01	-20.48	7.26	4.78E-03	-5.39	18.13	7.66E-01
cg22055728	35.07	11.80	2.96E-03	14.77	25.32	5.60E-01	-8.10	97.10	9.33E-01	45.05	14.82	2.37E-03	25.58	32.19	4.27E-01
cg05207184	-50.68	17.05	2.96E-03	-107.55	70.74	1.28E-01	-259.71	224.29	2.47E-01	-51.41	18.41	5.24E-03	14.78	60.89	8.08E-01
cg03611151	14.95	5.03	2.97E-03	14.99	8.24	6.89E-02	23.74	10.10	1.87E-02	17.22	12.50	1.68E-01	3.12	10.80	7.73E-01
cg13217116	36.82	12.39	2.97E-03	39.59	38.51	3.04E-01	-123.04	134.22	3.59E-01	37.54	13.87	6.79E-03	42.37	41.47	3.07E-01
cg14014879	30.39	10.23	2.98E-03	22.86	20.32	2.61E-01	41.96	25.19	9.58E-02	27.48	15.59	7.79E-02	38.72	26.37	1.42E-01
cg24519084	29.05	9.78	2.98E-03	41.19	20.61	4.57E-02	20.16	29.33	4.92E-01	24.18	14.74	1.01E-01	30.84	20.71	1.37E-01
cg07470694	-19.47	6.56	2.98E-03	-21.72	8.19	7.95E-03	-19.13	22.82	4.02E-01	-39.83	20.04	4.68E-02	1.89	15.99	9.06E-01
cg07912402	115.39	38.86	2.98E-03	102.73	61.22	9.34E-02	370.89	195.62	5.80E-02	131.47	91.42	1.50E-01	94.47	63.30	1.36E-01
cg17462329	18.64	6.28	2.99E-03	16.04	10.08	1.11E-01	15.59	13.80	2.58E-01	31.70	12.24	9.61E-03	5.94	16.71	7.22E-01
cg22045977	-11.68	3.94	2.99E-03	-20.53	8.39	1.44E-02	-12.81	16.04	4.24E-01	-10.60	4.98	3.31E-02	2.64	12.87	8.38E-01
cg07053841	-7.89	2.66	2.99E-03	-10.96	4.78	2.18E-02	-6.15	7.08	3.85E-01	-6.96	4.13	9.17E-02	-5.52	7.20	4.44E-01
cg23349517	-16.89	5.69	2.99E-03	-21.37	8.43	1.13E-02	-0.98	14.66	9.47E-01	-30.82	15.52	4.70E-02	-11.05	11.17	3.22E-01
cg12664038	8.43	2.84	2.99E-03	8.95	3.89	2.14E-02	13.37	6.91	5.30E-02	16.05	12.85	2.12E-01	2.46	5.70	6.66E-01
cg09829319	28.20	9.50	3.00E-03	25.01	12.96	5.37E-02	5.20	30.44	8.64E-01	64.55	31.03	3.75E-02	30.22	18.24	9.75E-02
cg18817426	18.95	6.38	3.00E-03	18.34	14.33	2.01E-01	12.52	14.03	3.72E-01	22.17	10.29	3.11E-02	19.94	13.94	1.53E-01
cg10512951	-11.25	3.79	3.00E-03	-12.79	6.68	5.56E-02	-4.76	13.34	7.21E-01	-12.92	5.40	1.66E-02	-3.60	11.75	7.59E-01
cg19871235	-7.49	2.52	3.00E-03	-12.22	4.22	3.76E-03	-4.44	6.69	5.08E-01	-8.22	3.99	3.93E-02	0.08	5.75	9.88E-01
cg23976431	-14.47	4.88	3.01E-03	-20.82	6.60	1.61E-03	-17.36	10.45	9.67E-02	2.68	10.11	7.91E-01	-16.18	8.15	4.71E-02
cg08315133	-13.26	4.47	3.02E-03	-12.02	6.56	6.67E-02	-12.94	10.74	2.28E-01	-4.08	15.27	7.90E-01	-18.45	8.53	3.05E-02
cg02578087	-6.85	2.31	3.02E-03	-5.68	3.15	7.10E-02	0.30	6.04	9.61E-01	-10.40	6.52	1.10E-01	-12.26	4.87	1.18E-02
cg13738144	19.52	6.58	3.02E-03	23.80	10.50	2.34E-02	15.56	16.71	3.52E-01	24.06	14.45	9.60E-02	11.30	13.33	3.96E-01

cg02451691	25.30	8.53	3.03E-03	14.99	18.15	4.09E-01	15.79	33.82	6.41E-01	32.82	10.91	2.64E-03	8.83	26.46	7.39E-01
cg26162932	41.12	13.87	3.03E-03	42.98	16.31	8.40E-03	-48.79	72.82	5.03E-01	37.49	71.04	5.98E-01	51.27	30.83	9.64E-02
cg27425612	22.72	7.67	3.03E-03	-6.32	34.50	8.55E-01	-37.82	127.13	7.66E-01	26.53	8.09	1.04E-03	-12.72	34.39	7.12E-01
cg03889226	37.61	12.69	3.03E-03	26.96	15.77	8.74E-02	13.11	41.14	7.50E-01	79.86	49.70	1.08E-01	71.28	28.93	1.38E-02
cg14059126	-28.20	9.51	3.04E-03	-23.48	23.28	3.13E-01	-19.85	42.59	6.41E-01	-31.56	11.56	6.30E-03	-17.97	29.34	5.40E-01
cg27146050	12.02	4.06	3.04E-03	15.57	5.79	7.16E-03	6.18	8.91	4.88E-01	14.42	10.68	1.77E-01	6.47	10.21	5.27E-01
cg13080465	-37.93	12.80	3.04E-03	-15.64	21.80	4.73E-01	-66.16	43.09	1.25E-01	-35.96	30.26	2.35E-01	-52.21	20.54	1.10E-02
cg08650934	13.79	4.65	3.04E-03	9.77	8.19	2.33E-01	10.10	10.93	3.55E-01	15.14	7.57	4.54E-02	26.26	13.60	5.35E-02
cg04075973	19.19	6.47	3.04E-03	9.76	9.90	3.24E-01	5.94	26.71	8.24E-01	34.13	10.63	1.33E-03	16.10	13.61	2.37E-01
cg15790184	17.50	5.91	3.05E-03	18.60	10.33	7.19E-02	18.70	11.44	1.02E-01	8.57	11.10	4.40E-01	32.51	16.82	5.33E-02
cg01375869	-32.23	10.88	3.05E-03	-25.12	15.97	1.16E-01	-12.09	50.54	8.11E-01	-56.38	26.04	3.03E-02	-32.28	19.38	9.59E-02
cg01053259	18.90	6.38	3.05E-03	21.45	19.49	2.71E-01	12.90	29.85	6.66E-01	20.32	7.15	4.48E-03	-3.29	28.22	9.07E-01
cg02609473	60.19	20.32	3.06E-03	34.15	21.22	1.08E-01	38.45	49.79	4.40E-01	142.02	56.01	1.12E-02	79.24	31.16	1.10E-02
cg08800670	-17.78	6.00	3.06E-03	-20.30	8.86	2.20E-02	-23.03	14.47	1.11E-01	1.76	12.83	8.91E-01	-28.00	12.72	2.77E-02
cg08782899	-14.85	5.01	3.06E-03	-11.52	6.98	9.86E-02	-17.76	11.34	1.17E-01	-13.36	15.68	3.94E-01	-21.82	11.62	6.04E-02
cg06996175	11.98	4.05	3.07E-03	11.04	5.28	3.66E-02	4.70	9.87	6.34E-01	23.16	14.10	1.00E-01	17.21	10.02	8.59E-02
cg05534865	23.21	7.84	3.07E-03	17.91	16.27	2.71E-01	26.81	15.87	9.13E-02	15.08	12.78	2.38E-01	46.44	20.44	2.31E-02
cg24803687	-10.20	3.45	3.07E-03	-12.49	7.24	8.48E-02	-15.52	14.73	2.92E-01	-7.91	4.52	8.03E-02	-13.93	9.24	1.32E-01
cg07004443	44.58	15.06	3.08E-03	42.22	20.37	3.82E-02	75.07	43.97	8.78E-02	38.52	40.55	3.42E-01	37.24	33.87	2.72E-01
cg09744376	25.71	8.69	3.08E-03	40.15	17.48	2.16E-02	21.66	47.70	6.50E-01	19.91	10.96	6.93E-02	28.04	28.68	3.28E-01
cg07050525	32.69	11.04	3.08E-03	36.22	12.93	5.07E-03	20.79	43.18	6.30E-01	147.51	74.21	4.68E-02	17.13	21.12	4.17E-01
cg03021690	21.29	7.19	3.08E-03	29.65	21.05	1.59E-01	48.91	23.78	3.97E-02	17.89	8.45	3.43E-02	5.85	27.60	8.32E-01
cg05404787	26.43	8.93	3.08E-03	32.67	15.07	3.01E-02	31.58	17.20	6.63E-02	13.62	18.69	4.66E-01	22.09	23.03	3.38E-01
cg22937444	27.67	9.35	3.08E-03	42.52	17.93	1.77E-02	21.92	16.38	1.81E-01	24.70	18.83	1.90E-01	18.47	23.71	4.36E-01
cg24098643	-65.19	22.03	3.09E-03	-58.17	29.10	4.56E-02	-31.64	131.71	8.10E-01	-177.54	75.08	1.80E-02	-50.12	39.40	2.03E-01
cg27625897	23.04	7.79	3.09E-03	28.83	23.95	2.29E-01	47.99	34.63	1.66E-01	21.58	8.82	1.44E-02	11.64	30.72	7.05E-01
cg05213387	-33.48	11.32	3.09E-03	-19.58	19.02	3.03E-01	-34.81	51.32	4.98E-01	-54.53	26.85	4.23E-02	-36.15	17.47	3.85E-02
cg11648249	-39.89	13.49	3.10E-03	-44.98	20.41	2.76E-02	-25.18	23.76	2.89E-01	-91.20	43.09	3.43E-02	-22.39	35.64	5.30E-01
cg26000554	17.83	6.03	3.10E-03	46.43	28.66	1.05E-01	15.58	72.24	8.29E-01	13.89	6.71	3.84E-02	31.60	16.08	4.93E-02
cg14609668	22.82	7.72	3.10E-03	23.70	12.84	6.50E-02	32.56	14.30	2.28E-02	17.61	18.12	3.31E-01	9.55	18.91	6.13E-01
cg14449575	19.52	6.60	3.11E-03	9.30	9.18	3.11E-01	30.98	14.60	3.38E-02	18.57	22.35	4.06E-01	35.21	15.06	1.94E-02
cg16234907	18.58	6.28	3.11E-03	21.08	13.00	1.05E-01	23.96	22.59	2.89E-01	20.31	9.03	2.45E-02	9.61	13.88	4.89E-01
cg06749098	45.21	15.29	3.12E-03	74.76	41.92	7.46E-02	-24.20	162.10	8.81E-01	37.56	17.50	3.18E-02	72.03	49.84	1.48E-01
cg00529742	-13.09	4.43	3.12E-03	-10.04	9.99	3.15E-01	-15.77	16.47	3.38E-01	-11.59	5.61	3.86E-02	-25.64	13.55	5.84E-02
cg13457961	-21.50	7.27	3.12E-03	-50.67	27.16	6.21E-02	-66.83	105.30	5.26E-01	-19.63	7.74	1.12E-02	-5.31	36.04	8.83E-01

cg04496246	36.92	12.49	3.12E-03	38.33	20.54	6.21E-02	-52.16	123.59	6.73E-01	40.13	17.53	2.21E-02	25.86	37.29	4.88E-01
cg17529436	21.93	7.42	3.13E-03	25.39	17.86	1.55E-01	-1.94	36.52	9.58E-01	21.85	8.95	1.47E-02	26.49	23.67	2.63E-01
cg09884213	-12.66	4.28	3.13E-03	-12.40	10.78	2.50E-01	-0.77	19.53	9.68E-01	-12.79	5.17	1.34E-02	-17.47	13.00	1.79E-01
cg07880384	-11.19	3.79	3.13E-03	-12.02	5.26	2.23E-02	-7.40	8.30	3.73E-01	-22.71	13.01	8.09E-02	-7.91	8.73	3.65E-01
cg26129606	-11.84	4.01	3.13E-03	-15.73	8.80	7.40E-02	-23.10	12.14	5.70E-02	-10.46	5.29	4.79E-02	-0.47	12.12	9.69E-01
cg13583272	7.58	2.57	3.14E-03	8.39	4.27	4.92E-02	8.94	5.20	8.53E-02	2.11	5.14	6.82E-01	12.59	6.71	6.05E-02
cg27113015	-14.65	4.96	3.14E-03	-16.98	7.99	3.36E-02	-15.36	11.26	1.72E-01	-4.31	11.63	7.11E-01	-18.20	10.15	7.31E-02
cg21341592	20.18	6.83	3.14E-03	25.86	10.24	1.15E-02	16.28	14.21	2.52E-01	15.18	19.16	4.28E-01	15.12	15.43	3.27E-01
cg18512262	-10.01	3.39	3.15E-03	-13.25	7.04	6.00E-02	-10.53	14.79	4.76E-01	-8.97	4.33	3.85E-02	-8.65	10.49	4.10E-01
cg16657928	-38.25	12.95	3.15E-03	-41.38	14.20	3.56E-03	111.86	167.55	5.04E-01	-148.15	96.48	1.25E-01	-12.49	34.25	7.15E-01
cg10222579	16.67	5.65	3.15E-03	6.01	12.87	6.41E-01	32.12	24.38	1.88E-01	17.15	6.86	1.24E-02	28.55	20.51	1.64E-01
cg06154159	-15.12	5.12	3.15E-03	-13.74	9.57	1.51E-01	-22.13	11.30	5.03E-02	-15.68	9.26	9.03E-02	-9.13	11.37	4.22E-01
cg03344384	-8.68	2.94	3.15E-03	-10.76	4.22	1.08E-02	-6.98	7.54	3.55E-01	-2.92	8.42	7.29E-01	-8.50	6.00	1.57E-01
cg22806908	34.40	11.65	3.16E-03	34.36	21.50	1.10E-01	16.45	33.24	6.21E-01	43.50	17.58	1.33E-02	22.01	30.70	4.73E-01
cg08852033	-14.72	4.99	3.16E-03	-15.25	6.87	2.63E-02	-6.47	13.10	6.21E-01	-1.52	15.76	9.23E-01	-24.54	10.46	1.89E-02
cg21062347	-13.47	4.56	3.16E-03	-8.71	7.03	2.15E-01	-11.99	9.33	1.99E-01	-29.80	12.38	1.60E-02	-14.13	10.11	1.62E-01
cg08129093	-38.04	12.89	3.16E-03	-49.97	17.18	3.63E-03	3.01	52.59	9.54E-01	-32.71	37.29	3.80E-01	-24.02	25.37	3.44E-01
cg21759057	38.07	12.90	3.17E-03	52.82	32.11	1.00E-01	-42.74	114.87	7.10E-01	38.68	14.98	9.81E-03	16.47	44.44	7.11E-01
cg27425169	-14.80	5.01	3.17E-03	-19.54	9.40	3.77E-02	-27.24	13.36	4.15E-02	-12.25	8.13	1.32E-01	-3.79	11.39	7.39E-01
cg13189271	10.44	3.54	3.17E-03	13.53	5.39	1.20E-02	11.80	8.90	1.85E-01	9.27	8.26	2.61E-01	4.54	7.45	5.42E-01
cg15457934	-15.10	5.12	3.17E-03	-22.25	6.73	9.51E-04	-7.16	14.48	6.21E-01	-9.51	19.03	6.17E-01	-2.93	10.80	7.86E-01
cg01415562	11.83	4.01	3.18E-03	14.11	6.05	1.97E-02	14.82	8.68	8.77E-02	12.71	13.00	3.28E-01	4.98	7.99	5.34E-01
cg02210498	-11.00	3.73	3.18E-03	-9.29	6.72	1.67E-01	3.91	12.96	7.63E-01	-11.69	5.26	2.62E-02	-24.51	11.50	3.31E-02
cg07535740	30.34	10.29	3.19E-03	26.00	15.23	8.76E-02	17.62	21.05	4.03E-01	33.76	28.04	2.29E-01	57.11	24.94	2.20E-02
cg11878872	51.58	17.49	3.19E-03	69.16	43.41	1.11E-01	-74.74	164.57	6.50E-01	49.45	20.08	1.38E-02	54.42	67.34	4.19E-01
cg01396176	28.35	9.61	3.19E-03	50.11	13.63	2.36E-04	5.66	21.14	7.89E-01	18.21	17.03	2.85E-01	25.23	15.80	1.10E-01
cg24815934	-15.97	5.42	3.19E-03	-15.54	7.60	4.09E-02	-21.66	14.41	1.33E-01	-9.82	13.76	4.75E-01	-17.85	12.23	1.45E-01
cg01560871	16.31	5.53	3.19E-03	7.11	9.16	4.38E-01	30.31	12.20	1.29E-02	14.78	10.85	1.73E-01	21.40	13.40	1.10E-01
cg09155001	25.51	8.65	3.20E-03	21.88	13.78	1.13E-01	28.37	17.34	1.02E-01	13.15	19.99	5.11E-01	43.47	21.03	3.88E-02
cg01005350	19.08	6.47	3.20E-03	13.71	16.04	3.93E-01	6.19	20.26	7.60E-01	24.31	7.96	2.25E-03	1.87	23.83	9.38E-01
cg07282465	-17.85	6.05	3.20E-03	-25.97	12.46	3.71E-02	-15.04	26.27	5.67E-01	-16.36	7.69	3.35E-02	-8.59	20.03	6.68E-01
cg13454184	-50.10	17.00	3.20E-03	-55.53	19.96	5.40E-03	-91.66	111.24	4.10E-01	6.06	85.18	9.43E-01	-37.47	36.95	3.11E-01
cg13595308	-19.96	6.77	3.21E-03	-29.85	11.20	7.67E-03	-23.21	15.82	1.43E-01	-0.38	14.16	9.79E-01	-21.17	14.38	1.41E-01
cg25976393	-56.12	19.05	3.21E-03	-131.30	62.60	3.60E-02	-153.62	260.50	5.55E-01	-52.36	21.78	1.62E-02	-22.68	51.32	6.59E-01
cg09246203	-19.02	6.46	3.21E-03	-27.76	8.81	1.62E-03	6.35	17.66	7.19E-01	-27.00	12.97	3.74E-02	-12.99	10.13	1.99E-01

cg16426215	-15.30	5.19	3.21E-03	-21.49	7.84	6.13E-03	-11.78	11.33	2.98E-01	-5.68	15.87	7.21E-01	-11.43	10.50	2.77E-01
cg06964876	25.71	8.73	3.22E-03	22.64	15.89	1.54E-01	13.04	15.86	4.11E-01	25.18	18.53	1.74E-01	53.91	20.97	1.01E-02
cg14866567	8.83	3.00	3.22E-03	6.63	4.15	1.10E-01	10.89	7.58	1.51E-01	7.38	6.47	2.54E-01	19.32	9.12	3.41E-02
cg01806921	-18.20	6.18	3.23E-03	-25.27	9.33	6.75E-03	-6.87	13.89	6.21E-01	-14.30	17.35	4.10E-01	-16.64	12.69	1.90E-01
cg03405781	-59.61	20.24	3.23E-03	-66.52	29.03	2.19E-02	0.69	66.63	9.92E-01	-82.59	42.93	5.44E-02	-45.05	45.33	3.20E-01
cg02455397	20.16	6.84	3.23E-03	11.90	18.11	5.11E-01	1.31	94.30	9.89E-01	23.40	7.72	2.43E-03	0.69	26.76	9.79E-01
cg12980527	-36.12	12.27	3.23E-03	-33.78	37.78	3.71E-01	-111.39	140.93	4.29E-01	-32.52	13.42	1.54E-02	-88.62	54.23	1.02E-01
cg27385590	-6.81	2.31	3.25E-03	-8.67	3.33	9.12E-03	-5.19	5.10	3.09E-01	-8.41	7.48	2.61E-01	-3.46	4.98	4.88E-01
cg25972997	-9.57	3.25	3.25E-03	-15.54	6.01	9.74E-03	-5.03	8.37	5.48E-01	-10.58	5.02	3.52E-02	1.22	8.77	8.89E-01
cg04505972	-13.34	4.53	3.25E-03	-10.86	8.99	2.27E-01	-4.07	12.83	7.51E-01	-16.12	6.47	1.27E-02	-16.56	12.56	1.88E-01
cg13847437	-23.65	8.04	3.25E-03	-13.91	21.07	5.09E-01	-16.65	22.62	4.62E-01	-25.19	9.91	1.10E-02	-41.96	30.32	1.66E-01
cg07032245	26.36	8.96	3.26E-03	43.16	30.68	1.60E-01	-5.08	126.46	9.68E-01	28.52	10.11	4.80E-03	2.61	25.34	9.18E-01
cg08433504	5.79	1.97	3.26E-03	4.80	2.79	8.51E-02	4.67	3.64	2.00E-01	10.56	7.65	1.68E-01	9.31	5.20	7.32E-02
cg23760103	-29.01	9.86	3.26E-03	-6.92	17.26	6.88E-01	-25.30	56.17	6.52E-01	-39.12	13.42	3.56E-03	-47.21	30.77	1.25E-01
cg16870215	-15.05	5.12	3.27E-03	-17.64	8.29	3.33E-02	-5.40	12.32	6.61E-01	-14.77	10.20	1.48E-01	-18.91	11.60	1.03E-01
cg06867623	56.19	19.11	3.27E-03	50.11	26.35	5.72E-02	85.64	67.45	2.04E-01	107.03	64.21	9.55E-02	44.19	34.57	2.01E-01
cg20707279	-44.42	15.10	3.27E-03	-119.88	56.39	3.35E-02	-178.88	180.14	3.21E-01	-40.48	16.42	1.37E-02	-3.93	55.28	9.43E-01
cg02903822	-31.84	10.83	3.28E-03	-43.40	15.86	6.22E-03	26.90	32.93	4.14E-01	-31.85	17.71	7.21E-02	-38.66	17.23	2.49E-02
cg15447231	9.46	3.22	3.29E-03	10.56	4.67	2.37E-02	13.25	7.53	7.83E-02	6.30	7.39	3.94E-01	5.43	8.27	5.11E-01
cg11530293	33.83	11.51	3.29E-03	44.16	18.04	1.44E-02	-53.30	57.44	3.53E-01	39.91	15.65	1.08E-02	13.79	28.99	6.34E-01
cg24506394	-26.77	9.11	3.29E-03	-25.41	21.79	2.44E-01	0.57	37.45	9.88E-01	-32.78	11.20	3.43E-03	-6.60	28.08	8.14E-01
cg13048591	23.89	8.13	3.29E-03	30.85	14.68	3.56E-02	4.28	14.49	7.68E-01	48.33	20.24	1.69E-02	23.26	12.72	6.76E-02
cg03386791	-16.44	5.59	3.29E-03	-21.98	7.93	5.57E-03	-19.74	12.09	1.03E-01	-20.74	18.97	2.74E-01	2.58	12.45	8.36E-01
cg17719126	-36.85	12.54	3.29E-03	-50.76	23.12	2.81E-02	-18.88	90.53	8.35E-01	-29.32	18.63	1.15E-01	-35.40	25.95	1.72E-01
cg03613942	-10.09	3.43	3.29E-03	-12.00	6.35	5.88E-02	-0.70	11.54	9.52E-01	-12.54	5.04	1.28E-02	-4.53	8.72	6.04E-01
cg20673255	21.50	7.31	3.30E-03	34.84	17.18	4.25E-02	15.80	17.86	3.76E-01	21.42	9.82	2.92E-02	6.67	23.62	7.78E-01
cg07138603	29.67	10.10	3.30E-03	56.72	26.24	3.07E-02	-19.15	75.46	8.00E-01	28.69	11.68	1.40E-02	1.99	34.34	9.54E-01
cg06955484	16.96	5.77	3.30E-03	24.88	10.53	1.81E-02	17.64	14.66	2.29E-01	22.38	10.87	3.96E-02	1.69	11.25	8.80E-01
cg07456480	-10.92	3.72	3.30E-03	-7.41	7.59	3.29E-01	-9.83	8.45	2.45E-01	-10.43	5.72	6.83E-02	-19.50	9.72	4.49E-02
cg18648031	24.15	8.22	3.31E-03	31.63	11.97	8.22E-03	16.73	17.13	3.29E-01	19.98	23.08	3.87E-01	16.61	19.91	4.04E-01
cg17890764	14.56	4.96	3.31E-03	11.26	7.57	1.37E-01	23.93	10.48	2.24E-02	15.46	11.31	1.71E-01	9.05	12.58	4.72E-01
cg23408442	29.88	10.18	3.32E-03	57.01	22.12	9.96E-03	7.02	23.03	7.60E-01	23.07	12.45	6.40E-02	51.92	30.19	8.54E-02
cg10203943	-24.66	8.40	3.32E-03	-20.56	24.41	4.00E-01	-63.12	56.58	2.65E-01	-22.03	9.46	1.98E-02	-48.61	31.46	1.22E-01
cg05803296	14.66	4.99	3.32E-03	18.05	7.05	1.04E-02	12.41	13.18	3.47E-01	8.05	12.63	5.24E-01	12.92	11.22	2.50E-01
cg04826355	120.77	41.14	3.33E-03	150.32	67.48	2.59E-02	-54.92	245.60	8.23E-01	178.72	99.74	7.31E-02	83.79	62.71	1.82E-01

cg04983933	28.11	9.57	3.33E-03	11.04	12.39	3.73E-01	38.49	15.26	1.16E-02	18.58	20.47	3.64E-01	52.06	18.55	5.00E-03
cg16090347	44.25	15.07	3.33E-03	49.86	20.37	1.44E-02	12.42	59.84	8.36E-01	52.05	39.53	1.88E-01	35.26	30.52	2.48E-01
cg05844937	23.60	8.04	3.33E-03	31.94	11.05	3.85E-03	25.66	37.83	4.98E-01	-12.54	42.40	7.67E-01	15.37	12.88	2.33E-01
cg09222732	-24.65	8.40	3.34E-03	-25.49	14.08	7.02E-02	-8.26	20.34	6.85E-01	-45.20	20.24	2.55E-02	-21.18	15.30	1.66E-01
cg17132696	-13.87	4.73	3.36E-03	-16.27	12.13	1.80E-01	-21.14	20.93	3.13E-01	-10.87	5.64	5.38E-02	-28.37	15.40	6.55E-02
cg19906737	26.12	8.91	3.36E-03	29.53	19.91	1.38E-01	-207.60	208.60	3.20E-01	26.08	10.10	9.80E-03	14.87	63.11	8.14E-01
cg08661164	-12.60	4.30	3.36E-03	-14.93	11.33	1.88E-01	-4.51	17.19	7.93E-01	-13.30	5.04	8.37E-03	-7.64	16.39	6.41E-01
cg03190045	-20.35	6.94	3.37E-03	-15.18	15.46	3.26E-01	-12.43	37.36	7.39E-01	-22.33	8.29	7.09E-03	-19.19	27.48	4.85E-01
cg19802390	8.66	2.95	3.37E-03	7.79	4.26	6.76E-02	7.46	6.44	2.47E-01	4.66	8.39	5.79E-01	14.91	6.85	2.94E-02
cg26121053	-21.46	7.32	3.38E-03	-16.27	9.43	8.45E-02	-16.20	37.16	6.63E-01	-32.75	19.45	9.23E-02	-29.48	15.74	6.11E-02
cg07859753	-8.92	3.04	3.38E-03	-15.88	5.79	6.07E-03	-1.41	10.65	8.95E-01	-6.49	4.47	1.46E-01	-7.91	7.24	2.75E-01
cg21468416	-20.55	7.02	3.40E-03	-24.59	10.93	2.44E-02	-32.40	21.28	1.28E-01	-10.38	14.86	4.85E-01	-17.89	13.88	1.97E-01
cg20299572	-14.06	4.80	3.40E-03	-10.03	6.83	1.42E-01	-16.05	10.78	1.36E-01	-22.19	13.09	8.99E-02	-17.03	11.58	1.42E-01
cg25781595	30.64	10.46	3.41E-03	33.36	13.08	1.08E-02	-46.28	58.64	4.30E-01	43.00	46.28	3.53E-01	30.90	19.86	1.20E-01
cg13178198	-19.62	6.70	3.41E-03	-24.57	12.70	5.30E-02	-11.74	16.45	4.76E-01	-24.01	11.00	2.90E-02	-10.41	15.61	5.05E-01
cg11724493	24.86	8.49	3.41E-03	32.92	20.59	1.10E-01	15.04	61.08	8.05E-01	19.95	9.93	4.47E-02	55.05	30.04	6.68E-02
cg22618402	23.70	8.10	3.41E-03	47.03	19.71	1.70E-02	-4.29	47.67	9.28E-01	21.32	9.56	2.57E-02	7.05	27.70	7.99E-01
cg04499648	20.41	6.97	3.41E-03	34.62	11.77	3.26E-03	-1.91	16.47	9.08E-01	24.52	12.65	5.26E-02	15.43	11.32	1.73E-01
cg20722590	17.14	5.85	3.42E-03	15.96	9.17	8.17E-02	11.47	10.79	2.88E-01	8.61	20.14	6.69E-01	30.59	12.67	1.58E-02
cg22396632	-65.16	22.26	3.42E-03	-49.86	34.28	1.46E-01	-99.96	125.87	4.27E-01	-113.33	36.45	1.88E-03	-18.54	43.09	6.67E-01
cg16739342	31.89	10.89	3.42E-03	48.63	14.26	6.50E-04	42.59	33.41	2.02E-01	14.08	9.52	1.39E-01	43.76	25.29	8.36E-02
cg08292485	-6.28	2.15	3.43E-03	-5.55	3.51	1.14E-01	-5.90	4.40	1.80E-01	-11.28	4.78	1.83E-02	-2.83	4.98	5.69E-01
cg17184165	-15.45	5.28	3.43E-03	-12.89	9.76	1.87E-01	-11.82	12.27	3.36E-01	-15.89	8.71	6.81E-02	-23.63	13.43	7.85E-02
cg14742211	-15.29	5.22	3.43E-03	-19.18	7.77	1.36E-02	-18.94	12.22	1.21E-01	-2.65	12.78	8.36E-01	-13.71	11.72	2.42E-01
cg21289015	12.68	4.34	3.44E-03	24.74	16.83	1.41E-01	52.55	45.52	2.48E-01	10.43	4.67	2.56E-02	24.77	17.11	1.48E-01
cg02932355	15.24	5.21	3.44E-03	16.42	8.98	6.74E-02	-4.72	24.32	8.46E-01	14.47	7.23	4.55E-02	24.43	16.52	1.39E-01
cg25719102	28.57	9.76	3.44E-03	43.54	19.25	2.37E-02	32.51	20.08	1.05E-01	14.57	15.88	3.59E-01	32.52	27.26	2.33E-01
cg20409298	25.97	8.88	3.44E-03	22.15	15.15	1.44E-01	21.61	15.46	1.62E-01	15.80	20.74	4.46E-01	58.04	23.41	1.32E-02
cg18192919	23.15	7.91	3.44E-03	22.72	15.35	1.39E-01	-2.45	26.78	9.27E-01	34.90	14.33	1.49E-02	19.54	13.53	1.49E-01
cg15794873	10.28	3.51	3.44E-03	6.46	4.57	1.58E-01	5.30	8.59	5.37E-01	22.70	9.52	1.71E-02	15.48	7.45	3.77E-02
cg00423871	18.47	6.31	3.44E-03	11.59	15.12	4.44E-01	31.20	19.39	1.08E-01	20.87	7.88	8.06E-03	-3.48	22.71	8.78E-01
cg14554491	12.47	4.26	3.45E-03	13.51	5.61	1.61E-02	5.94	12.22	6.27E-01	33.65	20.36	9.83E-02	9.62	8.39	2.52E-01
cg04622775	54.42	18.61	3.45E-03	44.54	26.57	9.37E-02	105.82	61.02	8.29E-02	66.28	35.29	6.04E-02	31.12	49.95	5.33E-01
cg21180599	16.15	5.52	3.46E-03	23.54	8.51	5.69E-03	11.78	15.47	4.47E-01	28.34	18.37	1.23E-01	6.02	9.19	5.12E-01
cg06087619	14.00	4.79	3.47E-03	11.50	11.71	3.26E-01	4.05	16.66	8.08E-01	17.05	5.91	3.93E-03	5.90	15.67	7.07E-01

cg08663298	-12.90	4.41	3.47E-03	-22.48	11.83	5.73E-02	-8.58	18.33	6.40E-01	-11.65	5.28	2.75E-02	-10.92	13.66	4.24E-01
cg06934774	20.55	7.03	3.47E-03	17.83	9.61	6.35E-02	32.45	20.72	1.17E-01	-4.31	25.93	8.68E-01	27.52	13.40	4.00E-02
cg17140992	16.90	5.78	3.48E-03	27.39	9.97	5.99E-03	-2.77	17.85	8.77E-01	16.20	9.84	9.97E-02	11.14	12.52	3.74E-01
cg01655607	-35.47	12.14	3.48E-03	-17.23	31.53	5.85E-01	-53.71	123.45	6.64E-01	-42.13	13.77	2.21E-03	5.70	47.89	9.05E-01
cg23702848	13.70	4.69	3.48E-03	7.87	6.63	2.36E-01	22.81	10.03	2.30E-02	16.38	14.58	2.61E-01	17.39	11.14	1.19E-01
cg18617770	32.31	11.06	3.48E-03	11.65	16.80	4.88E-01	33.53	21.66	1.22E-01	32.89	19.29	8.81E-02	67.66	24.22	5.22E-03
cg01553182	-11.31	3.87	3.49E-03	-19.48	8.83	2.74E-02	6.94	15.19	6.48E-01	-11.13	4.74	1.89E-02	-7.77	14.06	5.81E-01
cg08903587	39.82	13.63	3.49E-03	14.45	45.36	7.50E-01	-48.19	179.18	7.88E-01	42.28	14.81	4.30E-03	52.43	57.31	3.60E-01
cg16639880	22.67	7.76	3.49E-03	8.24	22.60	7.15E-01	80.54	47.22	8.81E-02	23.99	8.66	5.62E-03	5.10	33.89	8.80E-01
cg16149919	10.81	3.70	3.49E-03	6.51	5.70	2.53E-01	8.35	7.26	2.50E-01	24.38	11.52	3.42E-02	15.66	7.97	4.94E-02
cg06831361	-23.54	8.06	3.51E-03	-17.44	13.52	1.97E-01	-25.43	18.82	1.77E-01	-38.02	19.89	5.60E-02	-21.65	14.80	1.43E-01
cg27226927	11.67	4.00	3.51E-03	12.49	6.02	3.79E-02	17.55	6.93	1.14E-02	9.01	13.94	5.18E-01	-2.87	10.51	7.85E-01
cg17775490	9.71	3.33	3.51E-03	10.23	4.49	2.27E-02	6.92	9.18	4.51E-01	6.39	10.39	5.38E-01	11.63	7.13	1.03E-01
cg14776894	-16.75	5.74	3.51E-03	-14.25	14.84	3.37E-01	-1.26	24.70	9.59E-01	-19.54	6.84	4.28E-03	-8.62	18.86	6.48E-01
cg14065881	17.95	6.15	3.51E-03	18.30	16.74	2.74E-01	5.90	18.41	7.48E-01	18.05	7.32	1.37E-02	43.55	28.03	1.20E-01
cg17126947	-9.36	3.21	3.52E-03	-13.55	7.53	7.19E-02	-10.53	10.01	2.93E-01	-9.10	4.12	2.73E-02	-2.82	9.66	7.70E-01
cg00736299	7.90	2.71	3.52E-03	6.69	3.35	4.59E-02	11.65	6.07	5.50E-02	14.23	12.07	2.38E-01	5.10	8.60	5.53E-01
cg21359335	-56.22	19.27	3.52E-03	-52.00	26.71	5.16E-02	2.94	64.25	9.63E-01	-50.03	52.44	3.40E-01	-88.99	38.17	1.97E-02
cg20983312	44.28	15.18	3.52E-03	1.34	47.68	9.78E-01	-156.91	182.66	3.90E-01	46.70	16.96	5.89E-03	86.27	50.39	8.69E-02
cg01164291	27.04	9.27	3.53E-03	20.28	16.56	2.21E-01	26.67	18.33	1.46E-01	30.83	18.98	1.04E-01	33.81	21.09	1.09E-01
cg27052709	-14.02	4.81	3.53E-03	-15.26	7.15	3.28E-02	1.95	11.31	8.63E-01	-24.80	12.36	4.48E-02	-17.22	10.32	9.52E-02
cg00532474	9.78	3.35	3.53E-03	11.61	4.97	1.94E-02	10.19	7.55	1.77E-01	8.69	9.62	3.66E-01	6.32	7.06	3.70E-01
cg07108118	-16.44	5.63	3.53E-03	-12.47	16.12	4.39E-01	5.63	34.23	8.69E-01	-15.77	6.40	1.38E-02	-37.17	20.29	6.69E-02
cg18740810	-13.87	4.75	3.53E-03	-18.12	9.42	5.44E-02	-4.05	14.55	7.81E-01	-17.58	5.91	2.92E-03	8.40	15.36	5.84E-01
cg16092370	-15.86	5.44	3.53E-03	-27.14	14.09	5.42E-02	11.40	24.56	6.43E-01	-16.10	6.33	1.10E-02	-7.77	21.59	7.19E-01
cg16361840	50.91	17.45	3.54E-03	-35.18	64.92	5.88E-01	113.28	289.41	6.95E-01	60.68	18.93	1.35E-03	19.63	64.18	7.60E-01
cg05620762	-17.34	5.95	3.54E-03	-13.97	11.07	2.07E-01	-20.40	27.96	4.66E-01	-20.26	7.90	1.03E-02	-9.07	18.87	6.31E-01
cg08418076	-18.19	6.24	3.54E-03	-16.98	15.52	2.74E-01	-5.02	34.18	8.83E-01	-16.50	7.43	2.63E-02	-36.56	19.77	6.45E-02
cg00924004	19.96	6.84	3.55E-03	32.84	23.02	1.54E-01	16.28	60.03	7.86E-01	16.53	7.44	2.64E-02	54.33	29.81	6.84E-02
cg07658508	7.37	2.53	3.55E-03	5.36	3.61	1.37E-01	11.24	4.63	1.53E-02	6.51	9.06	4.72E-01	6.63	6.92	3.38E-01
cg20980960	45.16	15.49	3.55E-03	61.92	23.95	9.72E-03	90.57	50.81	7.47E-02	21.90	38.33	5.68E-01	22.32	27.14	4.11E-01
cg18056695	13.84	4.75	3.55E-03	20.31	6.98	3.59E-03	10.76	13.64	4.30E-01	18.47	14.64	2.07E-01	3.82	8.53	6.54E-01
cg14193339	42.25	14.49	3.56E-03	30.23	32.26	3.49E-01	55.46	119.44	6.42E-01	54.00	12.18	9.31E-06	-31.61	47.30	5.04E-01
cg12815974	-59.02	20.25	3.56E-03	-47.50	70.99	5.03E-01	-105.57	264.78	6.90E-01	-68.06	22.73	2.76E-03	-4.48	58.65	9.39E-01
cg07030704	-8.42	2.89	3.56E-03	-11.83	5.33	2.66E-02	-9.57	7.81	2.20E-01	-5.93	4.70	2.07E-01	-7.29	6.60	2.69E-01

cg20462449	-34.88	11.97	3.56E-03	-26.78	16.30	1.00E-01	9.12	39.67	8.18E-01	-69.87	30.96	2.40E-02	-47.81	24.49	5.09E-02
cg10514770	10.04	3.44	3.56E-03	8.19	5.36	1.26E-01	9.45	7.39	2.01E-01	13.14	9.23	1.54E-01	12.03	7.18	9.38E-02
cg08607221	21.32	7.32	3.57E-03	17.68	22.95	4.41E-01	56.65	29.97	5.87E-02	14.46	10.28	1.59E-01	26.55	12.69	3.65E-02
cg19314777	-14.43	4.95	3.57E-03	-23.85	11.52	3.84E-02	-3.28	18.85	8.62E-01	-12.22	6.11	4.55E-02	-19.81	16.59	2.32E-01
cg11365360	39.62	13.60	3.57E-03	42.95	16.99	1.15E-02	23.93	40.62	5.56E-01	20.34	54.67	7.10E-01	44.03	31.60	1.64E-01
cg25315819	-10.63	3.65	3.57E-03	-15.53	8.06	5.39E-02	-12.31	18.68	5.10E-01	-10.37	4.55	2.26E-02	-2.68	10.80	8.04E-01
cg10550882	20.05	6.88	3.58E-03	16.54	7.13	2.05E-02	46.37	20.43	2.32E-02	32.97	14.47	2.27E-02	6.11	12.10	6.13E-01
cg01641908	11.63	3.99	3.58E-03	9.27	9.05	3.05E-01	23.76	12.28	5.29E-02	12.25	5.24	1.94E-02	1.66	11.61	8.86E-01
cg05406868	21.10	7.24	3.58E-03	25.05	10.98	2.25E-02	22.01	23.24	3.44E-01	6.74	21.25	7.51E-01	20.69	12.22	9.04E-02
cg23422763	-8.18	2.81	3.59E-03	-9.31	5.43	8.64E-02	-8.35	6.21	1.79E-01	-4.94	4.44	2.66E-01	-15.58	7.82	4.63E-02
cg09446896	16.66	5.72	3.60E-03	21.17	13.96	1.29E-01	31.11	17.29	7.19E-02	15.36	7.09	3.02E-02	-4.46	21.46	8.35E-01
cg14074351	31.28	10.74	3.60E-03	53.28	21.45	1.30E-02	23.11	14.03	9.95E-02	20.51	58.68	7.27E-01	28.41	29.94	3.43E-01
cg05816298	33.82	11.62	3.60E-03	40.58	27.07	1.34E-01	16.14	52.49	7.59E-01	33.27	14.36	2.05E-02	33.61	34.68	3.33E-01
cg06996273	-8.03	2.76	3.61E-03	-9.16	4.22	3.01E-02	-8.75	6.73	1.94E-01	-17.33	8.20	3.45E-02	-2.36	5.11	6.45E-01
cg00663068	-31.81	10.93	3.62E-03	-38.15	15.23	1.23E-02	6.49	57.54	9.10E-01	-42.79	22.40	5.61E-02	-10.49	23.80	6.59E-01
cg07255158	-56.96	19.57	3.62E-03	-71.93	26.29	6.22E-03	25.36	88.36	7.74E-01	-53.72	53.39	3.14E-01	-42.35	38.23	2.68E-01
cg09110388	18.58	6.39	3.62E-03	21.17	10.29	3.96E-02	9.53	11.95	4.25E-01	28.90	15.42	6.09E-02	17.41	16.08	2.79E-01
cg12738763	3.85	1.32	3.62E-03	3.76	1.89	4.68E-02	3.69	2.77	1.83E-01	-1.31	4.68	7.80E-01	6.27	2.94	3.27E-02
cg11993720	51.92	17.85	3.62E-03	83.76	59.61	1.60E-01	78.05	203.59	7.01E-01	52.93	19.76	7.40E-03	7.49	60.40	9.01E-01
cg19594360	35.08	12.06	3.63E-03	47.30	37.66	2.09E-01	-50.26	162.36	7.57E-01	32.78	13.15	1.26E-02	58.08	53.85	2.81E-01
cg03818193	-25.54	8.78	3.63E-03	-25.74	16.98	1.29E-01	-4.37	40.02	9.13E-01	-27.81	11.70	1.75E-02	-22.96	25.19	3.62E-01
cg07084019	-16.81	5.78	3.63E-03	-32.92	15.66	3.55E-02	-23.79	17.70	1.79E-01	-10.49	7.06	1.37E-01	-31.58	19.55	1.06E-01
cg16347858	49.21	16.92	3.64E-03	49.24	24.18	4.17E-02	18.70	56.51	7.41E-01	81.69	40.52	4.38E-02	37.25	34.10	2.75E-01
cg02807446	-16.34	5.62	3.64E-03	-8.10	11.45	4.79E-01	-0.78	16.31	9.62E-01	-20.37	7.73	8.44E-03	-31.67	16.82	5.97E-02
cg01869288	-18.56	6.38	3.64E-03	-29.60	8.95	9.38E-04	-5.23	14.62	7.20E-01	-6.30	13.07	6.30E-01	-20.29	15.54	1.92E-01
cg20192747	13.10	4.51	3.64E-03	15.49	6.76	2.20E-02	11.55	10.92	2.90E-01	21.37	13.28	1.08E-01	6.63	8.67	4.44E-01
cg27260772	13.12	4.51	3.65E-03	21.89	12.17	7.22E-02	7.77	33.16	8.15E-01	13.86	5.45	1.10E-02	2.97	11.31	7.93E-01
cg13674249	-11.39	3.92	3.65E-03	-2.84	8.97	7.51E-01	-17.12	13.47	2.04E-01	-11.50	4.97	2.08E-02	-21.66	12.12	7.38E-02
cg23881697	28.13	9.68	3.66E-03	31.43	18.65	9.19E-02	20.98	17.54	2.32E-01	22.44	20.31	2.69E-01	41.09	21.69	5.82E-02
cg08148697	28.68	9.87	3.66E-03	43.46	17.85	1.49E-02	6.77	18.40	7.13E-01	28.82	18.50	1.19E-01	42.97	28.24	1.28E-01
cg20960277	-30.77	10.59	3.66E-03	-25.66	14.61	7.91E-02	0.32	33.20	9.92E-01	-33.81	23.24	1.46E-01	-62.36	26.05	1.67E-02
cg00885918	22.62	7.79	3.66E-03	26.20	12.25	3.24E-02	17.15	15.84	2.79E-01	23.20	17.31	1.80E-01	21.05	19.96	2.92E-01
cg14438812	-14.93	5.14	3.67E-03	-15.21	8.56	7.55E-02	-15.87	11.23	1.58E-01	-13.62	11.33	2.30E-01	-14.79	10.83	1.72E-01
cg22204103	12.42	4.28	3.68E-03	12.59	6.01	3.62E-02	6.65	10.07	5.09E-01	21.62	11.13	5.20E-02	10.00	10.51	3.42E-01
cg13518195	51.78	17.83	3.68E-03	46.57	25.54	6.82E-02	20.39	69.07	7.68E-01	125.44	54.71	2.19E-02	42.39	30.59	1.66E-01

cg04237482	22.65	7.80	3.68E-03	25.64	13.93	6.56E-02	11.04	28.07	6.94E-01	23.66	11.31	3.64E-02	18.72	21.32	3.80E-01
cg07628073	-31.19	10.74	3.68E-03	-25.07	16.51	1.29E-01	-2.80	50.04	9.55E-01	-27.48	22.62	2.24E-01	-46.71	19.44	1.63E-02
cg13246426	15.72	5.41	3.68E-03	10.03	11.04	3.64E-01	9.27	11.24	4.09E-01	19.65	8.55	2.15E-02	25.86	15.20	8.89E-02
cg04594378	-17.74	6.11	3.69E-03	-20.74	9.42	2.76E-02	-12.52	14.34	3.83E-01	-6.83	13.87	6.23E-01	-26.60	13.55	4.96E-02
cg22402853	-26.11	9.00	3.70E-03	-77.11	39.28	4.96E-02	-115.95	147.43	4.32E-01	-23.48	9.54	1.38E-02	-13.60	38.89	7.26E-01
cg04257865	-12.75	4.39	3.71E-03	-16.55	11.45	1.48E-01	-4.82	12.84	7.08E-01	-13.57	5.45	1.28E-02	-10.84	14.91	4.67E-01
cg22985281	-19.25	6.63	3.71E-03	-34.96	14.08	1.30E-02	6.98	29.93	8.16E-01	-14.43	8.50	8.96E-02	-25.42	19.17	1.85E-01
cg17382990	-36.07	12.43	3.71E-03	-45.95	21.43	3.21E-02	25.53	56.31	6.50E-01	-48.95	24.84	4.88E-02	-26.34	20.58	2.01E-01
cg03287432	8.46	2.92	3.72E-03	10.51	4.70	2.53E-02	4.55	6.30	4.70E-01	10.16	6.26	1.05E-01	6.72	6.79	3.23E-01
cg21032150	13.69	4.72	3.72E-03	24.00	12.08	4.70E-02	-10.69	33.07	7.47E-01	13.19	5.39	1.44E-02	2.28	19.11	9.05E-01
cg13524563	41.62	14.35	3.72E-03	34.71	19.42	7.39E-02	-12.87	65.44	8.44E-01	33.37	38.58	3.87E-01	69.75	27.73	1.19E-02
cg02313495	-4.33	1.49	3.73E-03	-4.44	1.99	2.55E-02	-2.55	3.47	4.62E-01	-6.24	5.30	2.38E-01	-5.03	3.63	1.66E-01
cg21665905	-13.55	4.67	3.73E-03	-18.33	8.61	3.32E-02	-2.39	10.61	8.22E-01	-12.59	7.49	9.31E-02	-22.77	13.34	8.78E-02
cg22825220	-13.44	4.64	3.74E-03	-17.90	9.44	5.79E-02	-2.70	15.38	8.61E-01	-13.29	6.19	3.19E-02	-13.35	14.18	3.46E-01
cg19675599	-11.58	4.00	3.74E-03	-23.36	11.05	3.45E-02	0.65	24.32	9.79E-01	-9.75	4.65	3.60E-02	-13.04	12.47	2.96E-01
cg19193136	10.85	3.74	3.75E-03	9.54	4.89	5.08E-02	24.47	10.34	1.79E-02	-0.55	12.90	9.66E-01	10.54	8.41	2.10E-01
cg10242089	29.19	10.07	3.75E-03	34.66	23.69	1.43E-01	-5.93	89.76	9.47E-01	32.01	11.82	6.78E-03	-2.88	35.44	9.35E-01
cg17721897	-77.08	26.60	3.75E-03	-87.11	36.87	1.81E-02	141.09	136.71	3.02E-01	-102.91	58.11	7.66E-02	-66.89	55.18	2.25E-01
cg17559156	29.42	10.15	3.76E-03	24.44	20.56	2.34E-01	-2.99	48.23	9.51E-01	26.82	12.93	3.80E-02	74.26	32.97	2.43E-02
cg21477508	39.30	13.56	3.76E-03	41.86	18.24	2.18E-02	49.18	59.71	4.10E-01	46.76	57.72	4.18E-01	32.42	23.24	1.63E-01
cg18669966	41.47	14.32	3.77E-03	36.77	16.99	3.04E-02	13.61	74.86	8.56E-01	70.80	65.24	2.78E-01	55.85	31.62	7.74E-02
cg17383853	-11.57	3.99	3.77E-03	-14.41	6.25	2.11E-02	-10.62	9.87	2.82E-01	-9.47	8.59	2.70E-01	-8.97	8.69	3.02E-01
cg19224278	17.27	5.96	3.78E-03	13.21	8.93	1.39E-01	22.59	17.50	1.97E-01	34.71	17.79	5.10E-02	14.91	10.44	1.53E-01
cg04718853	21.99	7.59	3.78E-03	26.55	12.37	3.19E-02	33.32	12.74	8.94E-03	-4.33	17.52	8.05E-01	18.66	18.78	3.20E-01
cg05228379	-38.33	13.23	3.78E-03	14.72	41.02	7.20E-01	-123.84	213.94	5.63E-01	-43.06	14.59	3.16E-03	-57.15	50.34	2.56E-01
cg00310410	-8.18	2.83	3.78E-03	-6.46	5.12	2.07E-01	-6.57	6.71	3.27E-01	-9.48	4.85	5.07E-02	-10.24	6.68	1.25E-01
cg03939566	-24.78	8.56	3.79E-03	-10.89	22.24	6.24E-01	-40.02	53.08	4.51E-01	-26.59	9.83	6.86E-03	-29.09	32.77	3.75E-01
cg04360780	-56.42	19.49	3.80E-03	-135.17	53.77	1.19E-02	-10.58	248.78	9.66E-01	-40.04	23.49	8.83E-02	-63.41	46.73	1.75E-01
cg15422307	-15.41	5.32	3.80E-03	-21.22	10.70	4.73E-02	-12.49	14.38	3.85E-01	-13.44	7.91	8.93E-02	-14.51	13.21	2.72E-01
cg00751937	31.61	10.92	3.80E-03	34.98	34.18	3.06E-01	8.36	137.40	9.51E-01	29.91	11.86	1.16E-02	60.32	52.51	2.51E-01
cg25122252	38.43	13.28	3.80E-03	23.31	42.91	5.87E-01	-11.95	159.60	9.40E-01	42.48	14.67	3.79E-03	19.10	47.35	6.87E-01
cg25367999	-11.91	4.12	3.81E-03	-24.98	9.14	6.25E-03	-10.10	15.03	5.02E-01	-7.16	4.89	1.44E-01	-16.18	11.81	1.71E-01
cg23685965	13.83	4.78	3.81E-03	12.11	7.29	9.64E-02	15.70	12.88	2.23E-01	14.85	10.93	1.75E-01	15.01	9.73	1.23E-01
cg06629775	65.00	22.47	3.82E-03	57.37	26.70	3.17E-02	150.57	175.93	3.92E-01	162.50	89.25	6.86E-02	54.77	48.80	2.62E-01
cg27141863	-52.29	18.08	3.82E-03	-79.73	61.99	1.98E-01	27.31	243.55	9.11E-01	-50.21	19.71	1.09E-02	-50.17	69.12	4.68E-01

cg02962406	-29.79	10.30	3.82E-03	-25.66	10.00	1.03E-02	8.17	23.18	7.24E-01	-54.42	22.71	1.66E-02	-42.78	14.96	4.23E-03
cg03727673	-19.78	6.84	3.82E-03	-33.30	15.33	2.98E-02	-19.65	17.93	2.73E-01	-17.94	9.42	5.69E-02	-6.54	19.05	7.31E-01
cg21386611	23.33	8.07	3.83E-03	40.28	19.32	3.71E-02	-16.52	46.29	7.21E-01	21.65	9.56	2.36E-02	16.78	27.75	5.45E-01
cg07962812	-40.92	14.15	3.83E-03	-8.67	41.90	8.36E-01	-194.61	219.42	3.75E-01	-44.80	15.67	4.26E-03	-39.02	54.90	4.77E-01
cg18384106	15.94	5.51	3.84E-03	18.83	14.77	2.02E-01	9.56	20.11	6.34E-01	14.79	6.76	2.87E-02	22.88	15.86	1.49E-01
cg21179654	-14.64	5.06	3.84E-03	-24.03	7.90	2.33E-03	-7.46	11.11	5.02E-01	-1.81	11.11	8.71E-01	-16.61	10.15	1.02E-01
cg09939344	-37.30	12.90	3.84E-03	-46.96	20.27	2.05E-02	-3.97	37.53	9.16E-01	-42.22	27.05	1.19E-01	-32.91	25.87	2.03E-01
cg18793463	23.30	8.06	3.85E-03	33.53	22.51	1.36E-01	-25.71	34.69	4.58E-01	22.65	9.29	1.47E-02	51.49	31.73	1.05E-01
cg24348479	15.61	5.40	3.85E-03	19.96	10.23	5.10E-02	6.78	14.26	6.34E-01	11.05	7.96	1.65E-01	34.01	15.78	3.11E-02
cg23621912	-9.85	3.41	3.85E-03	-12.91	6.38	4.28E-02	4.30	9.68	6.57E-01	-12.84	5.01	1.04E-02	-5.91	9.51	5.34E-01
cg00374492	43.38	15.01	3.85E-03	31.44	18.44	8.82E-02	64.94	66.25	3.27E-01	55.20	61.23	3.67E-01	70.30	31.55	2.59E-02
cg26985142	-48.61	16.82	3.85E-03	-70.64	51.28	1.68E-01	167.97	220.56	4.46E-01	-46.17	18.64	1.32E-02	-60.71	62.53	3.32E-01
cg12762432	39.16	13.55	3.85E-03	57.61	48.90	2.39E-01	-32.24	184.17	8.61E-01	33.21	14.82	2.50E-02	87.52	47.47	6.52E-02
cg04436971	19.36	6.70	3.85E-03	23.23	10.55	2.76E-02	18.82	12.86	1.43E-01	2.51	16.93	8.82E-01	26.63	16.32	1.03E-01
cg23093116	12.74	4.41	3.85E-03	12.71	12.04	2.91E-01	9.50	21.89	6.64E-01	15.56	5.36	3.67E-03	0.74	11.47	9.49E-01
cg11240634	32.95	11.40	3.86E-03	33.21	14.73	2.42E-02	84.20	46.87	7.24E-02	39.35	28.27	1.64E-01	9.34	26.95	7.29E-01
cg07813851	-15.61	5.40	3.86E-03	-25.56	13.80	6.39E-02	-11.70	20.76	5.73E-01	-11.41	6.63	8.52E-02	-28.95	15.96	6.97E-02
cg00332212	34.93	12.09	3.87E-03	3.37	32.62	9.18E-01	59.43	97.99	5.44E-01	42.85	13.95	2.13E-03	14.15	39.04	7.17E-01
cg02477175	-54.04	18.71	3.88E-03	-45.26	42.50	2.87E-01	-227.09	228.93	3.21E-01	-63.43	22.71	5.23E-03	-5.75	53.89	9.15E-01
cg11621104	39.53	13.69	3.88E-03	46.97	18.50	1.11E-02	70.82	42.10	9.25E-02	20.73	49.86	6.78E-01	17.56	26.27	5.04E-01
cg24283621	30.40	10.53	3.89E-03	34.54	44.31	4.36E-01	14.22	66.23	8.30E-01	29.51	11.35	9.30E-03	46.98	44.15	2.87E-01
cg04704887	19.13	6.63	3.90E-03	25.98	19.31	1.78E-01	23.51	19.41	2.26E-01	16.72	7.98	3.63E-02	23.57	23.90	3.24E-01
cg12154110	22.29	7.72	3.90E-03	20.31	12.94	1.16E-01	23.79	13.85	8.59E-02	21.14	18.74	2.59E-01	24.93	19.11	1.92E-01
cg02459543	-15.28	5.29	3.90E-03	-18.13	11.68	1.21E-01	-1.56	21.85	9.43E-01	-13.81	6.62	3.69E-02	-27.19	17.02	1.10E-01
cg10946573	-13.46	4.66	3.90E-03	-19.82	7.08	5.09E-03	-5.26	10.55	6.18E-01	-18.07	5.04	3.38E-04	2.85	10.96	7.95E-01
cg11723801	10.84	3.75	3.91E-03	18.95	7.42	1.06E-02	3.19	10.52	7.62E-01	11.59	6.18	6.09E-02	5.25	7.55	4.86E-01
cg15001618	24.16	8.37	3.91E-03	31.73	16.28	5.13E-02	17.79	16.64	2.85E-01	24.23	14.03	8.41E-02	20.86	23.57	3.76E-01
cg03797537	17.49	6.06	3.91E-03	14.97	14.89	3.15E-01	42.02	21.36	4.92E-02	16.23	7.29	2.60E-02	6.49	24.30	7.89E-01
cg20792735	26.55	9.20	3.91E-03	48.31	27.55	7.95E-02	-14.31	63.40	8.21E-01	24.86	10.80	2.14E-02	24.16	24.48	3.24E-01
cg08751997	-17.72	6.14	3.92E-03	-5.72	11.85	6.29E-01	-36.75	28.54	1.98E-01	-23.90	8.22	3.62E-03	-8.88	17.29	6.08E-01
cg08293002	18.51	6.42	3.92E-03	7.94	14.90	5.94E-01	0.94	31.83	9.76E-01	18.94	7.45	1.10E-02	57.18	26.00	2.79E-02
cg09442613	-15.13	5.25	3.92E-03	-18.97	12.17	1.19E-01	-4.74	13.16	7.19E-01	-15.48	7.48	3.87E-02	-19.82	12.95	1.26E-01
cg10337290	-31.47	10.91	3.92E-03	-53.84	17.76	2.43E-03	-9.65	37.84	7.99E-01	-14.66	20.80	4.81E-01	-23.88	21.23	2.61E-01
cg03189871	-24.72	8.57	3.92E-03	-20.78	18.01	2.49E-01	-44.05	51.79	3.95E-01	-23.50	10.53	2.56E-02	-38.71	29.66	1.92E-01
cg14406134	-13.51	4.68	3.92E-03	-18.26	7.01	9.17E-03	-6.42	12.78	6.15E-01	-7.88	11.43	4.91E-01	-12.62	9.36	1.77E-01

cg06690831	-10.27	3.56	3.92E-03	-7.42	8.15	3.63E-01	-18.73	12.40	1.31E-01	-11.80	4.59	1.00E-02	-1.56	10.10	8.77E-01
cg04125765	-21.64	7.51	3.94E-03	-20.55	10.90	5.94E-02	-8.99	16.20	5.79E-01	-32.82	19.31	8.92E-02	-31.33	18.78	9.53E-02
cg12130725	-12.92	4.48	3.94E-03	-5.81	10.67	5.86E-01	-6.86	16.35	6.75E-01	-16.63	5.48	2.39E-03	-3.07	15.99	8.48E-01
cg05672327	20.08	6.97	3.94E-03	38.08	18.17	3.61E-02	10.27	19.47	5.98E-01	17.35	8.66	4.52E-02	24.95	24.91	3.17E-01
cg26637898	23.51	8.16	3.95E-03	31.73	20.94	1.30E-01	39.89	24.08	9.76E-02	15.42	10.03	1.24E-01	54.25	30.34	7.38E-02
cg01467419	26.81	9.30	3.95E-03	31.83	11.54	5.83E-03	23.18	31.40	4.60E-01	-21.07	30.73	4.93E-01	35.25	22.47	1.17E-01
cg16202736	-18.55	6.44	3.96E-03	1.41	20.43	9.45E-01	-5.25	44.20	9.05E-01	-20.85	7.12	3.39E-03	-24.92	26.12	3.40E-01
cg14392066	24.44	8.48	3.96E-03	14.34	17.25	4.06E-01	8.67	38.37	8.21E-01	32.33	10.89	2.98E-03	8.99	26.57	7.35E-01
cg19323958	-15.66	5.44	3.97E-03	-24.93	8.88	5.02E-03	-2.83	12.63	8.23E-01	-13.96	13.47	3.00E-01	-12.72	10.31	2.17E-01
cg18716979	24.67	8.56	3.97E-03	44.97	18.30	1.40E-02	12.02	25.77	6.41E-01	17.06	12.57	1.75E-01	26.99	18.83	1.52E-01
cg21762523	31.15	10.82	3.97E-03	20.08	25.19	4.25E-01	17.51	50.30	7.28E-01	26.11	14.22	6.65E-02	60.36	24.72	1.46E-02
cg00961326	23.56	8.18	3.98E-03	44.60	19.28	2.07E-02	22.53	22.74	3.22E-01	22.07	13.37	9.88E-02	13.77	14.56	3.44E-01
cg08735550	18.86	6.55	3.99E-03	20.98	9.85	3.31E-02	19.10	12.89	1.38E-01	-5.36	18.78	7.75E-01	29.84	15.55	5.50E-02
cg18871670	45.45	15.79	3.99E-03	53.60	21.70	1.35E-02	3.42	44.47	9.39E-01	118.32	72.22	1.01E-01	37.05	28.96	2.01E-01
cg08122151	-11.70	4.07	4.00E-03	-15.00	6.50	2.09E-02	-5.07	8.66	5.58E-01	-26.08	12.66	3.94E-02	-7.10	7.62	3.52E-01
cg07099161	22.32	7.76	4.01E-03	30.48	12.44	1.42E-02	16.15	19.84	4.16E-01	10.93	18.95	5.64E-01	21.20	14.38	1.40E-01
cg16099804	22.88	7.95	4.01E-03	44.94	21.97	4.08E-02	22.80	32.54	4.84E-01	19.64	11.17	7.88E-02	18.78	14.45	1.94E-01
cg01394339	-6.54	2.28	4.02E-03	-5.54	3.06	6.97E-02	-4.90	4.93	3.20E-01	-6.03	11.48	5.99E-01	-11.34	5.18	2.85E-02
cg07007165	21.72	7.55	4.03E-03	13.14	16.37	4.22E-01	16.13	19.08	3.98E-01	28.64	14.07	4.18E-02	23.76	12.89	6.53E-02
cg12603173	42.75	14.86	4.03E-03	49.04	18.25	7.20E-03	49.55	55.90	3.75E-01	53.66	46.12	2.45E-01	6.97	36.96	8.50E-01
cg00673202	-7.27	2.53	4.03E-03	-8.11	3.61	2.47E-02	-5.68	5.03	2.60E-01	8.57	12.23	4.83E-01	-10.39	5.46	5.70E-02
cg23463335	21.37	7.43	4.03E-03	29.14	19.39	1.33E-01	24.78	25.76	3.36E-01	17.58	8.81	4.60E-02	43.45	30.91	1.60E-01
cg16544169	20.24	7.04	4.03E-03	20.79	10.42	4.59E-02	19.56	18.50	2.90E-01	16.30	18.05	3.66E-01	22.05	14.18	1.20E-01
cg02034720	-54.41	18.92	4.04E-03	-51.86	24.58	3.48E-02	-55.98	67.54	4.07E-01	5.44	66.71	9.35E-01	-79.38	37.98	3.66E-02
cg18440893	33.80	11.76	4.04E-03	32.26	38.05	3.97E-01	182.45	170.76	2.85E-01	35.25	12.82	5.96E-03	3.39	48.68	9.44E-01
cg24795627	34.45	11.98	4.04E-03	33.41	31.22	2.85E-01	56.44	63.60	3.75E-01	27.64	14.93	6.42E-02	56.21	28.81	5.11E-02
cg22452837	-4.50	1.57	4.04E-03	-5.33	2.55	3.65E-02	-5.82	3.12	6.22E-02	-7.21	4.24	8.87E-02	-0.16	3.24	9.60E-01
cg00808243	24.39	8.48	4.05E-03	14.44	24.61	5.57E-01	34.07	54.13	5.29E-01	24.57	9.60	1.05E-02	34.97	30.82	2.56E-01
cg08901867	-14.45	5.03	4.05E-03	-7.13	10.72	5.06E-01	-11.98	13.46	3.74E-01	-14.93	7.25	3.96E-02	-25.21	12.56	4.47E-02
cg11826961	14.33	4.98	4.05E-03	15.82	6.77	1.94E-02	17.20	12.36	1.64E-01	3.76	13.99	7.88E-01	14.71	12.17	2.27E-01
cg13418632	-22.31	7.76	4.05E-03	-28.25	12.05	1.91E-02	2.73	19.11	8.86E-01	-33.01	17.55	6.00E-02	-20.43	16.40	2.13E-01
cg14877413	26.06	9.07	4.05E-03	17.97	16.42	2.74E-01	29.98	17.00	7.78E-02	17.63	17.52	3.14E-01	51.24	23.97	3.25E-02
cg20005760	-14.38	5.00	4.05E-03	-16.80	12.62	1.83E-01	-21.96	24.35	3.67E-01	-14.44	5.99	1.60E-02	-7.19	15.56	6.44E-01
cg10662943	-23.74	8.26	4.06E-03	-7.45	19.34	7.00E-01	-44.50	35.68	2.12E-01	-22.37	9.89	2.37E-02	-66.47	32.17	3.88E-02
cg13289884	-29.77	10.36	4.06E-03	-21.31	15.00	1.55E-01	-13.14	23.67	5.79E-01	-55.36	37.03	1.35E-01	-50.36	20.59	1.44E-02

cg11742746	-59.36	20.66	4.06E-03	-48.77	26.01	6.07E-02	-65.49	68.34	3.38E-01	-20.73	66.90	7.57E-01	-113.08	48.37	1.94E-02
cg22752049	160.85	55.98	4.06E-03	228.67	82.09	5.34E-03	264.07	294.94	3.71E-01	165.56	148.22	2.64E-01	60.00	93.80	5.22E-01
cg08353308	20.83	7.25	4.06E-03	9.07	17.15	5.97E-01	-1.80	38.93	9.63E-01	23.58	8.60	6.11E-03	33.03	26.26	2.09E-01
cg03373714	31.71	11.04	4.07E-03	27.24	14.84	6.64E-02	-11.64	40.12	7.72E-01	57.85	47.18	2.20E-01	45.41	19.64	2.07E-02
cg07515865	52.73	18.35	4.07E-03	-20.28	62.19	7.44E-01	-99.85	168.77	5.54E-01	62.42	20.17	1.97E-03	54.66	67.97	4.21E-01
cg11345703	-13.49	4.70	4.07E-03	-17.26	7.00	1.37E-02	-11.81	9.65	2.21E-01	-13.97	15.63	3.72E-01	-7.50	9.95	4.51E-01
cg24734586	20.83	7.25	4.07E-03	3.39	23.66	8.86E-01	-52.61	101.01	6.02E-01	22.32	8.05	5.54E-03	29.89	24.30	2.19E-01
cg10956762	-21.18	7.37	4.07E-03	-14.24	11.86	2.30E-01	-17.78	21.82	4.15E-01	-28.27	16.00	7.72E-02	-26.64	13.77	5.31E-02
cg05455279	-9.34	3.25	4.08E-03	-14.75	6.96	3.39E-02	-3.04	12.14	8.02E-01	-8.66	4.26	4.22E-02	-6.72	9.10	4.60E-01
cg23641635	13.34	4.64	4.08E-03	14.66	9.19	1.11E-01	18.35	16.22	2.58E-01	11.80	6.13	5.42E-02	14.85	15.58	3.41E-01
cg05165935	21.13	7.36	4.08E-03	24.16	20.92	2.48E-01	0.08	47.68	9.99E-01	23.05	8.36	5.83E-03	3.68	26.36	8.89E-01
cg04744409	10.82	3.77	4.08E-03	7.85	6.02	1.92E-01	13.54	8.88	1.27E-01	9.55	8.24	2.46E-01	15.09	8.04	6.05E-02
cg10100811	-11.87	4.13	4.08E-03	-7.97	7.76	3.04E-01	-0.68	10.07	9.46E-01	-15.85	6.37	1.28E-02	-22.16	11.57	5.55E-02
cg24664861	-59.09	20.58	4.08E-03	-72.56	25.48	4.40E-03	68.60	108.97	5.29E-01	-11.81	84.55	8.89E-01	-53.42	40.92	1.92E-01
cg15603354	-10.55	3.67	4.08E-03	-15.41	6.68	2.11E-02	-8.32	9.45	3.78E-01	-10.62	5.90	7.20E-02	-3.26	9.21	7.23E-01
cg06417385	-14.67	5.11	4.09E-03	-8.03	9.09	3.77E-01	-12.60	12.31	3.06E-01	-19.20	9.32	3.94E-02	-19.87	11.12	7.39E-02
cg24908166	-32.23	11.22	4.09E-03	-29.04	16.26	7.40E-02	-21.72	46.85	6.43E-01	-41.83	23.91	8.02E-02	-32.24	22.65	1.55E-01
cg02110836	-20.14	7.02	4.10E-03	-34.93	9.06	1.16E-04	-9.81	13.21	4.58E-01	-18.89	15.97	2.37E-01	-9.35	11.34	4.10E-01
cg01170591	-26.73	9.31	4.11E-03	-22.45	24.07	3.51E-01	-2.88	30.41	9.25E-01	-35.61	11.24	1.53E-03	19.67	35.33	5.78E-01
cg24851651	1.77	0.62	4.11E-03	1.37	0.89	1.26E-01	3.60	1.32	6.27E-03	1.51	1.61	3.48E-01	0.68	1.55	6.63E-01
cg02680909	-13.24	4.62	4.11E-03	-18.25	6.78	7.13E-03	-10.34	11.13	3.53E-01	2.12	13.51	8.75E-01	-13.14	9.26	1.56E-01
cg05217213	-11.87	4.14	4.12E-03	-13.96	9.81	1.55E-01	-18.50	10.91	9.00E-02	-12.39	5.58	2.64E-02	0.56	11.48	9.61E-01
cg02356111	46.02	16.04	4.12E-03	39.87	51.31	4.37E-01	-127.32	252.25	6.14E-01	50.42	17.49	3.93E-03	3.71	67.27	9.56E-01
cg00597366	-70.52	24.58	4.12E-03	-75.53	31.72	1.73E-02	-40.54	133.65	7.62E-01	11.42	75.52	8.80E-01	-96.28	48.22	4.59E-02
cg14151259	32.45	11.31	4.12E-03	27.41	16.04	8.74E-02	98.00	41.90	1.94E-02	17.16	41.67	6.80E-01	29.24	18.95	1.23E-01
cg27555803	22.06	7.69	4.12E-03	6.77	21.71	7.55E-01	-51.81	77.16	5.02E-01	25.30	8.53	3.02E-03	22.45	33.75	5.06E-01
cg07129879	-12.07	4.21	4.12E-03	-22.57	8.26	6.27E-03	-4.70	10.48	6.54E-01	-9.48	6.32	1.34E-01	-9.19	11.38	4.19E-01
cg24182581	-18.17	6.34	4.14E-03	-17.25	12.30	1.61E-01	-14.36	22.78	5.28E-01	-17.87	9.02	4.76E-02	-22.41	15.70	1.53E-01
cg20948923	-11.79	4.11	4.15E-03	-7.04	8.86	4.27E-01	-8.80	16.45	5.93E-01	-12.50	5.11	1.44E-02	-22.05	15.18	1.46E-01
cg08473553	34.85	12.16	4.15E-03	52.86	28.80	6.64E-02	40.20	104.77	7.01E-01	33.89	14.80	2.20E-02	15.08	33.30	6.51E-01
cg23530263	29.84	10.41	4.15E-03	21.52	32.55	5.09E-01	-211.83	152.78	1.66E-01	33.06	11.36	3.59E-03	15.84	45.54	7.28E-01
cg25130993	24.43	8.52	4.15E-03	-7.08	30.09	8.14E-01	-1.69	103.13	9.87E-01	28.66	9.42	2.35E-03	16.42	27.66	5.53E-01
cg11837402	43.01	15.01	4.16E-03	-16.53	45.79	7.18E-01	0.07	196.51	1.00E+00	48.52	16.47	3.21E-03	79.77	63.32	2.08E-01
cg03232390	-7.00	2.44	4.16E-03	-11.08	5.14	3.09E-02	-6.54	6.42	3.09E-01	-4.56	3.53	1.96E-01	-9.05	6.29	1.50E-01
cg03724990	20.84	7.27	4.16E-03	21.52	10.18	3.44E-02	37.12	22.85	1.04E-01	23.61	22.04	2.84E-01	12.60	13.76	3.60E-01

cg23154272	26.25	9.16	4.16E-03	27.92	16.40	8.86E-02	26.97	16.98	1.12E-01	26.02	20.69	2.08E-01	22.85	20.43	2.64E-01
cg19921170	-54.87	19.15	4.16E-03	-45.23	23.06	4.98E-02	28.65	107.13	7.89E-01	3.98	88.97	9.64E-01	-106.77	39.75	7.23E-03
cg04456666	45.62	15.92	4.17E-03	15.57	40.18	6.98E-01	-63.51	141.05	6.53E-01	50.73	18.48	6.05E-03	72.04	53.70	1.80E-01
cg16404364	17.31	6.04	4.18E-03	28.17	14.62	5.40E-02	40.54	21.28	5.68E-02	10.77	7.23	1.37E-01	34.12	27.00	2.06E-01
cg00834988	9.39	3.28	4.19E-03	10.61	4.53	1.92E-02	2.82	7.92	7.22E-01	10.30	7.99	1.97E-01	11.86	8.89	1.82E-01
cg14663951	21.15	7.39	4.19E-03	26.17	16.67	1.16E-01	47.41	23.76	4.60E-02	12.95	9.27	1.62E-01	44.31	27.43	1.06E-01
cg13092405	4.93	1.72	4.19E-03	6.00	2.27	8.32E-03	3.31	3.63	3.62E-01	-0.57	6.90	9.35E-01	5.58	4.61	2.25E-01
cg07742633	30.53	10.66	4.20E-03	18.07	23.29	4.38E-01	-72.02	78.76	3.61E-01	32.02	11.63	5.91E-03	68.32	35.58	5.48E-02
cg18736791	32.17	11.23	4.20E-03	27.06	14.44	6.09E-02	-25.82	48.95	5.98E-01	53.10	36.74	1.48E-01	49.01	22.54	2.97E-02
cg19550904	13.35	4.66	4.20E-03	14.26	7.43	5.50E-02	2.42	11.31	8.30E-01	14.24	9.77	1.45E-01	19.55	10.20	5.53E-02
cg00754584	-37.39	13.06	4.20E-03	-28.39	25.79	2.71E-01	-59.12	213.07	7.81E-01	-41.10	15.93	9.88E-03	-33.48	50.38	5.06E-01
cg18347630	15.02	5.25	4.20E-03	14.52	7.27	4.59E-02	32.14	13.28	1.55E-02	5.57	14.24	6.96E-01	9.02	12.13	4.57E-01
cg24199203	10.55	3.69	4.20E-03	9.62	5.71	9.22E-02	11.54	8.22	1.60E-01	15.54	7.01	2.67E-02	-0.63	11.30	9.56E-01
cg03844838	-51.83	18.11	4.21E-03	-55.27	28.09	4.91E-02	11.06	50.34	8.26E-01	-60.53	33.79	7.33E-02	-76.92	44.19	8.18E-02
cg13285077	-13.79	4.82	4.21E-03	-17.76	6.87	9.74E-03	-18.70	11.84	1.14E-01	-2.53	14.27	8.59E-01	-7.31	10.07	4.68E-01
cg16681239	18.01	6.29	4.21E-03	28.08	10.88	9.83E-03	7.18	17.35	6.79E-01	16.54	13.13	2.08E-01	12.72	11.41	2.65E-01
cg08472795	18.65	6.52	4.21E-03	21.11	9.99	3.46E-02	17.48	12.92	1.76E-01	23.64	17.44	1.75E-01	10.64	15.33	4.88E-01
cg09272702	-15.38	5.37	4.22E-03	-32.82	12.64	9.42E-03	-27.85	29.87	3.51E-01	-11.83	6.47	6.73E-02	-3.87	17.34	8.23E-01
cg06213047	-15.49	5.41	4.22E-03	-11.69	12.90	3.65E-01	-1.63	18.61	9.30E-01	-16.62	6.80	1.45E-02	-26.26	16.74	1.17E-01
cg21748223	20.40	7.13	4.22E-03	36.73	20.87	7.85E-02	41.44	64.80	5.22E-01	19.23	8.08	1.74E-02	6.94	23.41	7.67E-01
cg00698906	27.00	9.44	4.23E-03	26.83	11.81	2.31E-02	34.86	36.49	3.39E-01	1.64	72.93	9.82E-01	27.01	17.91	1.32E-01
cg03642472	-16.59	5.80	4.24E-03	-18.57	12.67	1.43E-01	-2.83	16.56	8.64E-01	-15.31	7.95	5.41E-02	-31.10	15.79	4.90E-02
cg09858767	-12.74	4.46	4.24E-03	-14.49	7.60	5.66E-02	-13.50	10.23	1.87E-01	-10.23	9.59	2.86E-01	-11.93	8.90	1.80E-01
cg03369250	27.99	9.79	4.24E-03	28.50	31.18	3.61E-01	35.81	120.03	7.65E-01	28.12	10.87	9.67E-03	25.54	33.89	4.51E-01
cg19506025	18.64	6.52	4.25E-03	24.08	13.54	7.54E-02	44.05	40.56	2.77E-01	13.38	8.05	9.65E-02	36.55	22.24	1.00E-01
cg05305278	-12.73	4.45	4.25E-03	-5.52	8.85	5.33E-01	-19.73	12.65	1.19E-01	-15.00	6.22	1.59E-02	-10.88	13.40	4.17E-01
cg05281206	-12.72	4.45	4.25E-03	-23.78	9.54	1.27E-02	-1.78	15.85	9.10E-01	-9.68	5.96	1.05E-01	-13.74	11.58	2.36E-01
cg07111719	22.46	7.86	4.26E-03	38.42	17.00	2.39E-02	33.20	19.24	8.44E-02	10.52	10.73	3.27E-01	36.96	27.24	1.75E-01
cg00747372	-11.19	3.91	4.26E-03	-14.90	9.53	1.18E-01	-21.93	18.92	2.46E-01	-10.40	4.70	2.68E-02	-5.42	12.79	6.72E-01
cg13981325	7.74	2.71	4.26E-03	8.94	3.70	1.57E-02	5.46	5.24	2.97E-01	3.99	10.28	6.98E-01	9.52	7.58	2.09E-01
cg18618431	21.70	7.59	4.26E-03	21.33	18.21	2.41E-01	-35.72	40.95	3.83E-01	25.12	9.07	5.62E-03	17.75	25.07	4.79E-01
cg10582608	-4.03	1.41	4.27E-03	-5.38	2.10	1.02E-02	-4.08	2.94	1.66E-01	-5.69	4.44	2.00E-01	-0.39	3.02	8.97E-01
cg27084736	-16.32	5.71	4.27E-03	-9.42	17.31	5.86E-01	-9.06	24.29	7.09E-01	-18.05	6.45	5.11E-03	-12.28	25.17	6.26E-01
cg13534999	-14.64	5.12	4.27E-03	-29.39	10.04	3.42E-03	-27.90	26.37	2.90E-01	-9.79	4.45	2.78E-02	-9.51	14.76	5.19E-01
cg13049624	-17.88	6.26	4.28E-03	-0.98	15.31	9.49E-01	-4.92	29.31	8.67E-01	-21.64	7.49	3.88E-03	-26.73	20.91	2.01E-01

cg05628049	12.15	4.25	4.28E-03	16.03	6.02	7.72E-03	12.10	10.89	2.66E-01	10.95	9.54	2.51E-01	0.80	11.02	9.42E-01
cg21274025	25.32	8.86	4.28E-03	32.76	17.67	6.38E-02	23.13	18.96	2.23E-01	25.72	14.52	7.66E-02	15.56	22.34	4.86E-01
cg04106076	-17.72	6.20	4.28E-03	-21.21	9.90	3.21E-02	-21.97	15.31	1.51E-01	-11.93	15.95	4.54E-01	-13.62	11.48	2.35E-01
cg14517743	17.80	6.23	4.29E-03	29.95	19.85	1.31E-01	-27.68	57.97	6.33E-01	19.11	6.97	6.13E-03	-1.11	20.68	9.57E-01
cg11988910	18.69	6.54	4.29E-03	26.65	10.78	1.34E-02	9.93	15.43	5.20E-01	18.88	11.68	1.06E-01	8.38	17.64	6.35E-01
cg20220316	41.60	14.57	4.29E-03	39.01	20.73	5.99E-02	-3.53	65.87	9.57E-01	41.33	42.02	3.25E-01	52.06	25.09	3.80E-02
cg18815120	5.38	1.88	4.30E-03	4.73	2.85	9.74E-02	6.07	4.97	2.22E-01	3.66	4.98	4.62E-01	6.93	3.58	5.29E-02
cg19016694	-11.04	3.87	4.30E-03	-11.21	5.17	3.00E-02	-17.40	11.01	1.14E-01	-6.94	9.38	4.59E-01	-9.80	10.12	3.33E-01
cg17741993	-7.39	2.59	4.30E-03	-8.13	3.45	1.84E-02	-5.80	5.39	2.81E-01	-13.56	10.51	1.97E-01	-4.46	6.81	5.12E-01
cg03519879	-17.91	6.27	4.30E-03	-20.27	9.80	3.86E-02	-20.88	14.75	1.57E-01	1.10	15.78	9.45E-01	-23.90	12.52	5.63E-02
cg04838709	-45.95	16.10	4.32E-03	-39.27	23.32	9.22E-02	-29.45	90.28	7.44E-01	-59.64	30.67	5.18E-02	-45.65	34.66	1.88E-01
cg13434842	13.22	4.63	4.32E-03	8.10	7.61	2.87E-01	13.06	10.18	2.00E-01	27.35	11.50	1.74E-02	11.82	9.07	1.93E-01
cg00213142	16.52	5.79	4.33E-03	8.94	9.31	3.37E-01	32.91	11.14	3.13E-03	8.68	12.13	4.74E-01	17.54	12.76	1.69E-01
cg20670923	11.93	4.18	4.33E-03	15.93	5.28	2.56E-03	11.39	11.16	3.07E-01	14.51	16.33	3.74E-01	-3.66	10.24	7.21E-01
cg25185525	29.31	10.27	4.33E-03	39.67	20.98	5.86E-02	27.41	21.23	1.97E-01	21.81	16.24	1.79E-01	37.01	28.99	2.02E-01
cg13201297	-16.34	5.73	4.33E-03	-24.10	8.41	4.18E-03	-9.10	11.86	4.43E-01	-10.22	17.76	5.65E-01	-9.97	12.85	4.37E-01
cg10503891	63.01	22.09	4.33E-03	38.32	30.36	2.07E-01	96.46	130.65	4.60E-01	84.90	49.37	8.55E-02	94.94	44.90	3.45E-02
cg24303306	-33.42	11.72	4.34E-03	-33.78	19.23	7.89E-02	-3.14	31.36	9.20E-01	-51.30	22.87	2.49E-02	-30.78	24.62	2.11E-01
cg19656847	21.79	7.64	4.34E-03	27.36	12.59	2.97E-02	8.01	21.99	7.16E-01	16.23	11.78	1.68E-01	43.51	25.46	8.75E-02
cg04802386	-10.58	3.71	4.34E-03	-8.41	7.40	2.56E-01	1.27	9.01	8.88E-01	-14.37	5.45	8.40E-03	-17.55	10.92	1.08E-01
cg24428758	26.02	9.12	4.34E-03	25.26	12.45	4.24E-02	8.09	34.69	8.16E-01	61.68	50.19	2.19E-01	27.33	15.20	7.21E-02
cg11358199	-11.21	3.93	4.35E-03	-11.56	5.00	2.07E-02	-13.60	9.74	1.63E-01	-0.35	15.45	9.82E-01	-11.83	10.01	2.37E-01
cg15600835	-27.33	9.58	4.35E-03	-20.29	27.37	4.59E-01	-93.87	116.21	4.19E-01	-30.22	10.62	4.45E-03	6.85	40.21	8.65E-01
cg06674117	-15.37	5.39	4.35E-03	-20.30	15.14	1.80E-01	-43.03	30.09	1.53E-01	-11.78	6.23	5.85E-02	-28.19	17.79	1.13E-01
cg07901571	-12.94	4.54	4.36E-03	-20.09	10.10	4.67E-02	3.93	14.00	7.79E-01	-11.61	5.99	5.24E-02	-22.18	13.21	9.32E-02
cg18142162	16.07	5.64	4.36E-03	14.24	7.69	6.41E-02	30.79	13.87	2.64E-02	16.34	20.50	4.26E-01	9.44	11.97	4.30E-01
cg04578317	13.99	4.91	4.37E-03	7.46	6.92	2.81E-01	25.81	10.62	1.51E-02	6.85	17.07	6.88E-01	20.78	10.97	5.82E-02
cg02624019	29.77	10.44	4.37E-03	24.37	21.70	2.62E-01	19.65	22.59	3.84E-01	26.00	16.04	1.05E-01	68.21	28.93	1.84E-02
cg07841500	19.36	6.79	4.38E-03	18.74	15.16	2.17E-01	11.64	21.45	5.88E-01	19.35	8.59	2.43E-02	31.70	25.03	2.05E-01
cg01287975	16.06	5.64	4.38E-03	16.38	7.95	3.95E-02	4.17	13.69	7.61E-01	48.19	22.09	2.91E-02	15.18	10.99	1.67E-01
cg27179533	25.11	8.81	4.39E-03	44.80	24.75	7.03E-02	87.41	49.50	7.74E-02	17.65	10.79	1.02E-01	28.04	21.16	1.85E-01
cg04341153	38.82	13.63	4.39E-03	61.59	48.06	2.00E-01	-159.20	234.13	4.97E-01	34.26	14.64	1.93E-02	94.92	61.11	1.20E-01
cg20134241	36.50	12.81	4.39E-03	47.30	16.58	4.33E-03	10.03	37.28	7.88E-01	-23.25	47.88	6.27E-01	40.96	27.74	1.40E-01
cg16590154	-24.13	8.47	4.39E-03	-7.13	23.46	7.61E-01	-17.13	29.69	5.64E-01	-30.37	9.96	2.30E-03	2.44	33.26	9.42E-01
cg02909320	-21.30	7.48	4.40E-03	-7.01	13.65	6.08E-01	-59.28	37.72	1.16E-01	-23.25	10.12	2.16E-02	-36.46	22.11	9.92E-02

cg15787807	11.03	3.87	4.40E-03	13.26	7.50	7.70E-02	12.95	11.38	2.55E-01	9.28	5.35	8.27E-02	12.14	12.73	3.40E-01
cg01995099	20.27	7.12	4.40E-03	18.05	10.07	7.31E-02	20.93	14.18	1.40E-01	22.58	24.42	3.55E-01	24.84	17.59	1.58E-01
cg22645350	36.74	12.90	4.40E-03	20.80	20.77	3.17E-01	-7.67	47.57	8.72E-01	61.22	21.22	3.91E-03	38.85	31.20	2.13E-01
cg19759172	13.29	4.67	4.41E-03	7.39	16.05	6.45E-01	1.09	19.97	9.57E-01	13.37	5.16	9.59E-03	38.95	22.54	8.39E-02
cg18490614	12.67	4.45	4.42E-03	15.64	6.82	2.18E-02	11.31	10.44	2.79E-01	10.89	10.12	2.82E-01	9.28	9.98	3.52E-01
cg15835542	13.58	4.77	4.42E-03	15.45	6.84	2.40E-02	7.98	17.33	6.45E-01	13.28	10.89	2.22E-01	11.86	9.62	2.18E-01
cg19243391	-18.36	6.45	4.42E-03	-23.68	10.92	3.01E-02	-4.82	12.91	7.09E-01	-10.23	14.19	4.71E-01	-34.73	14.59	1.73E-02
cg27235662	-12.32	4.33	4.42E-03	-14.98	6.32	1.79E-02	-16.13	9.44	8.75E-02	-6.90	11.91	5.62E-01	-5.29	9.93	5.94E-01
cg25455598	43.69	15.35	4.42E-03	42.44	49.11	3.88E-01	-270.36	189.48	1.54E-01	45.06	17.05	8.21E-03	56.29	52.62	2.85E-01
cg26543333	10.28	3.61	4.42E-03	10.25	5.48	6.16E-02	8.69	7.04	2.17E-01	19.98	8.64	2.07E-02	0.45	10.05	9.64E-01
cg13472192	17.51	6.15	4.43E-03	30.41	14.41	3.48E-02	27.49	20.01	1.69E-01	13.62	9.65	1.58E-01	12.09	10.93	2.68E-01
cg03580279	26.30	9.24	4.43E-03	43.63	20.00	2.92E-02	26.31	19.24	1.71E-01	12.93	14.26	3.65E-01	40.35	25.05	1.07E-01
cg16411152	17.18	6.04	4.43E-03	28.61	15.68	6.80E-02	12.93	23.59	5.84E-01	15.64	7.00	2.55E-02	10.84	28.99	7.08E-01
cg07022554	17.56	6.17	4.43E-03	16.86	13.48	2.11E-01	54.45	40.21	1.76E-01	14.69	7.47	4.92E-02	32.09	21.18	1.30E-01
cg04536588	-10.10	3.55	4.45E-03	-16.86	9.14	6.50E-02	-6.05	13.95	6.64E-01	-10.96	4.33	1.13E-02	1.92	10.63	8.57E-01
cg12493107	-24.47	8.60	4.45E-03	-26.98	14.42	6.14E-02	-33.05	20.64	1.09E-01	-14.67	16.20	3.65E-01	-26.47	19.79	1.81E-01
cg21005438	-20.73	7.29	4.45E-03	-20.37	13.51	1.32E-01	1.87	20.78	9.28E-01	-30.36	11.03	5.92E-03	-11.92	18.82	5.27E-01
cg23557906	-24.68	8.68	4.45E-03	-26.17	12.22	3.22E-02	-6.10	23.99	7.99E-01	-40.87	23.74	8.52E-02	-22.61	18.05	2.10E-01
cg15061025	24.45	8.60	4.45E-03	23.79	12.19	5.10E-02	49.34	23.50	3.58E-02	17.97	26.95	5.05E-01	15.68	16.62	3.45E-01
cg05004900	-31.32	11.01	4.46E-03	-48.56	28.81	9.19E-02	-177.51	161.19	2.71E-01	-30.97	12.29	1.17E-02	32.83	51.67	5.25E-01
cg03872934	-14.57	5.13	4.46E-03	-11.39	6.74	9.09E-02	-13.60	11.82	2.50E-01	-22.17	21.22	2.96E-01	-23.63	12.26	5.39E-02
cg00110846	-19.85	6.98	4.46E-03	-18.71	14.19	1.87E-01	-3.78	21.42	8.60E-01	-18.08	9.92	6.83E-02	-38.18	17.67	3.07E-02
cg26302103	-62.17	21.87	4.46E-03	-108.46	68.11	1.11E-01	-202.01	210.89	3.38E-01	-62.10	24.98	1.29E-02	-10.28	63.18	8.71E-01
cg13219625	37.92	13.34	4.47E-03	55.22	38.23	1.49E-01	-46.12	121.93	7.05E-01	36.83	14.87	1.32E-02	34.23	53.72	5.24E-01
cg07606115	15.13	5.32	4.48E-03	5.50	10.16	5.88E-01	4.30	13.78	7.55E-01	25.02	8.31	2.61E-03	16.34	13.05	2.11E-01
cg24128630	22.32	7.85	4.48E-03	7.40	15.34	6.29E-01	41.73	46.53	3.70E-01	27.90	10.07	5.59E-03	21.98	24.72	3.74E-01
cg00613385	-18.61	6.55	4.48E-03	-26.32	16.41	1.09E-01	-1.14	19.76	9.54E-01	-15.89	8.10	4.97E-02	-50.62	23.58	3.18E-02
cg08691479	-42.05	14.80	4.48E-03	-77.10	32.74	1.85E-02	-79.35	82.60	3.37E-01	-32.63	18.32	7.50E-02	-22.25	44.34	6.16E-01
cg23487226	-13.86	4.88	4.48E-03	-27.62	10.25	7.05E-03	-14.03	11.48	2.22E-01	-6.55	6.76	3.33E-01	-18.61	14.54	2.01E-01
cg07727425	17.73	6.24	4.48E-03	37.89	13.92	6.48E-03	17.88	17.12	2.96E-01	10.34	7.95	1.93E-01	23.64	23.88	3.22E-01
cg15481161	16.69	5.87	4.49E-03	33.42	16.22	3.94E-02	99.25	65.82	1.32E-01	13.40	6.56	4.10E-02	13.06	24.18	5.89E-01
cg08417857	-11.94	4.20	4.49E-03	-11.40	8.07	1.57E-01	-6.90	14.42	6.32E-01	-10.39	5.96	8.13E-02	-21.08	10.96	5.45E-02
cg17783244	13.79	4.85	4.49E-03	19.58	7.44	8.55E-03	14.62	9.64	1.29E-01	-3.51	12.53	7.79E-01	13.38	11.72	2.54E-01
cg06073141	-13.16	4.63	4.49E-03	-13.39	13.63	3.26E-01	1.12	19.93	9.55E-01	-13.33	5.28	1.16E-02	-22.97	18.54	2.16E-01
cg16473511	35.74	12.58	4.49E-03	23.57	22.66	2.98E-01	-104.60	147.49	4.78E-01	44.19	16.07	5.95E-03	29.98	46.95	5.23E-01

cg25960378	7.15	2.52	4.50E-03	7.89	3.68	3.19E-02	4.48	5.69	4.31E-01	1.60	6.77	8.14E-01	11.94	5.67	3.51E-02
cg21496913	20.61	7.25	4.50E-03	26.03	26.08	3.18E-01	-94.39	120.08	4.32E-01	20.73	7.77	7.67E-03	18.40	33.04	5.78E-01
cg09973663	-30.53	10.75	4.51E-03	-34.38	22.11	1.20E-01	7.25	47.96	8.80E-01	-29.01	13.88	3.67E-02	-47.20	31.84	1.38E-01
cg18996153	-28.26	9.95	4.51E-03	-25.40	15.19	9.46E-02	-32.49	31.49	3.02E-01	-32.33	17.31	6.18E-02	-24.46	26.56	3.57E-01
cg18034049	-15.57	5.48	4.51E-03	-25.44	15.61	1.03E-01	-5.93	20.53	7.73E-01	-16.01	6.47	1.34E-02	-5.96	18.53	7.48E-01
cg26846726	-41.14	14.48	4.51E-03	-45.73	18.36	1.28E-02	-79.50	45.16	7.84E-02	-26.11	43.97	5.53E-01	-10.01	35.53	7.78E-01
cg06038358	-49.30	17.36	4.51E-03	-47.25	23.24	4.20E-02	27.78	80.21	7.29E-01	-46.59	35.68	1.92E-01	-83.43	43.65	5.60E-02
cg08288703	-12.34	4.35	4.52E-03	-16.35	6.29	9.33E-03	-9.73	10.73	3.65E-01	-13.73	12.18	2.60E-01	-5.15	9.03	5.68E-01
cg23615572	-27.59	9.72	4.52E-03	-31.57	15.86	4.66E-02	2.83	26.39	9.15E-01	-29.09	18.96	1.25E-01	-37.50	20.43	6.64E-02
cg21745184	33.14	11.67	4.52E-03	28.84	14.47	4.63E-02	37.87	54.15	4.84E-01	98.03	44.86	2.89E-02	25.42	24.05	2.91E-01
cg16806210	22.86	8.05	4.52E-03	17.67	12.78	1.67E-01	18.86	14.47	1.92E-01	46.41	21.44	3.04E-02	22.72	20.65	2.71E-01
ch.8.1995451R	-28.07	9.89	4.53E-03	-27.54	15.93	8.39E-02	-36.46	17.91	4.18E-02	0.97	27.19	9.72E-01	-36.43	23.44	1.20E-01
cg10983752	-11.93	4.20	4.53E-03	-5.52	8.53	5.17E-01	-7.33	9.87	4.58E-01	-16.65	6.57	1.12E-02	-14.63	10.29	1.55E-01
cg16600934	19.74	6.95	4.53E-03	30.70	16.24	5.87E-02	17.33	14.59	2.35E-01	12.55	9.81	2.01E-01	44.56	23.62	5.92E-02
cg02227969	-36.03	12.70	4.54E-03	-38.89	15.91	1.45E-02	2.15	70.90	9.76E-01	-49.59	43.10	2.50E-01	-28.78	25.67	2.62E-01
cg24482850	11.81	4.16	4.54E-03	19.09	7.76	1.40E-02	15.70	9.81	1.09E-01	3.23	6.21	6.03E-01	17.03	10.21	9.53E-02
cg10973622	4.94	1.74	4.54E-03	5.97	2.99	4.56E-02	5.25	4.22	2.13E-01	6.22	2.97	3.65E-02	-0.81	4.56	8.59E-01
cg00613284	-14.31	5.04	4.54E-03	-11.71	9.01	1.93E-01	-23.60	13.96	9.10E-02	-16.81	7.94	3.43E-02	-5.11	12.89	6.92E-01
cg08289409	30.71	10.82	4.55E-03	43.63	30.43	1.52E-01	27.82	107.00	7.95E-01	30.94	12.17	1.10E-02	6.25	40.00	8.76E-01
cg26635366	-60.02	21.16	4.56E-03	-39.92	61.47	5.16E-01	-29.59	250.86	9.06E-01	-53.10	24.45	2.99E-02	-122.09	59.75	4.10E-02
cg00514684	33.92	11.96	4.56E-03	44.41	14.00	1.52E-03	-3.57	58.80	9.52E-01	-33.21	69.03	6.31E-01	13.46	26.77	6.15E-01
cg22862529	18.18	6.41	4.56E-03	36.06	17.20	3.61E-02	29.18	20.34	1.51E-01	15.99	7.77	3.95E-02	-7.61	22.56	7.36E-01
cg02902102	-9.64	3.40	4.57E-03	-12.66	5.09	1.28E-02	-11.65	6.95	9.37E-02	7.25	12.38	5.58E-01	-7.31	6.96	2.93E-01
cg03392965	-15.56	5.49	4.57E-03	-20.75	7.94	8.94E-03	-14.04	13.21	2.88E-01	-0.21	17.85	9.91E-01	-12.54	10.85	2.48E-01
cg12879386	-25.17	8.88	4.57E-03	3.48	30.28	9.09E-01	-87.99	78.62	2.63E-01	-28.36	10.13	5.11E-03	-19.25	24.31	4.28E-01
cg19430577	31.19	11.00	4.58E-03	64.15	32.52	4.85E-02	87.49	91.37	3.38E-01	28.65	13.01	2.76E-02	13.40	27.85	6.30E-01
cg00907842	7.83	2.76	4.59E-03	7.04	3.56	4.77E-02	13.22	7.06	6.13E-02	12.20	10.10	2.27E-01	3.84	6.70	5.67E-01
cg06943925	28.79	10.16	4.59E-03	56.51	18.80	2.65E-03	-7.58	29.92	8.00E-01	24.46	8.90	5.97E-03	28.25	28.43	3.20E-01
cg15257719	-11.32	3.99	4.59E-03	-22.98	9.62	1.70E-02	-10.91	19.61	5.78E-01	-10.14	4.90	3.84E-02	-1.38	11.46	9.04E-01
cg08259958	37.50	13.23	4.60E-03	21.79	25.58	3.94E-01	94.68	144.64	5.13E-01	41.37	17.00	1.50E-02	49.12	38.44	2.01E-01
cg09303778	-15.91	5.62	4.60E-03	-33.91	14.84	2.23E-02	-12.18	32.92	7.11E-01	-14.22	6.38	2.59E-02	5.74	24.29	8.13E-01
cg14975061	-14.46	5.10	4.60E-03	-17.18	10.51	1.02E-01	2.53	14.43	8.61E-01	-15.56	7.34	3.39E-02	-20.61	12.97	1.12E-01
cg02841875	25.66	9.06	4.60E-03	35.32	14.16	1.26E-02	28.99	22.56	1.99E-01	13.91	16.41	3.97E-01	18.42	25.57	4.71E-01
cg27329548	54.33	19.18	4.61E-03	57.74	55.71	3.00E-01	-227.41	270.60	4.01E-01	61.85	21.11	3.39E-03	-46.76	84.64	5.81E-01
cg09757644	-12.41	4.38	4.61E-03	-19.72	6.63	2.91E-03	-2.41	10.05	8.10E-01	-5.94	11.79	6.15E-01	-10.69	9.03	2.37E-01

cg13608733	15.82	5.58	4.61E-03	25.03	10.44	1.66E-02	11.98	14.49	4.08E-01	14.49	10.20	1.55E-01	9.56	10.84	3.78E-01
cg26419728	35.19	12.42	4.62E-03	37.98	24.76	1.25E-01	79.09	40.23	4.93E-02	23.72	18.69	2.04E-01	35.99	27.00	1.83E-01
cg11338420	39.43	13.92	4.62E-03	48.33	17.83	6.73E-03	18.39	17.74	3.00E-01	19.06	18.88	3.13E-01	85.05	25.16	7.23E-04
cg01055518	-8.38	2.96	4.62E-03	-9.16	5.27	8.25E-02	-12.07	7.27	9.67E-02	-4.68	4.91	3.40E-01	-11.48	7.48	1.25E-01
cg13011388	19.92	7.03	4.62E-03	20.06	16.15	2.14E-01	-1.92	19.57	9.22E-01	27.12	11.82	2.17E-02	20.67	12.30	9.30E-02
cg08506260	69.02	24.37	4.63E-03	59.22	59.59	3.20E-01	-300.62	307.55	3.28E-01	65.99	28.85	2.21E-02	123.37	72.62	8.93E-02
cg14024243	-16.37	5.78	4.63E-03	-32.22	15.33	3.56E-02	-17.23	12.88	1.81E-01	-13.00	7.64	8.86E-02	-10.39	20.06	6.04E-01
cg22014834	-24.76	8.74	4.63E-03	-17.23	22.30	4.40E-01	-10.62	47.62	8.24E-01	-28.99	10.16	4.30E-03	-3.67	32.70	9.11E-01
cg00135416	21.71	7.67	4.63E-03	19.06	13.20	1.49E-01	3.40	13.72	8.04E-01	30.43	13.36	2.27E-02	41.61	18.21	2.23E-02
cg11429111	-10.99	3.88	4.63E-03	-15.84	5.57	4.48E-03	-1.29	8.91	8.85E-01	-6.52	9.69	5.01E-01	-12.26	9.58	2.00E-01
cg20025824	26.58	9.39	4.63E-03	37.71	18.92	4.62E-02	32.46	19.59	9.76E-02	21.06	14.31	1.41E-01	8.31	30.64	7.86E-01
cg03342032	-12.32	4.35	4.63E-03	-12.27	10.54	2.44E-01	-13.80	17.79	4.38E-01	-10.31	5.31	5.23E-02	-25.10	13.82	6.94E-02
cg23250374	38.62	13.64	4.64E-03	27.57	32.27	3.93E-01	0.45	126.84	9.97E-01	47.28	15.90	2.95E-03	-14.90	50.19	7.67E-01
cg11081833	14.91	5.27	4.64E-03	14.85	8.38	7.65E-02	21.68	11.17	5.22E-02	12.74	12.45	3.06E-01	9.56	11.67	4.13E-01
cg01724150	16.99	6.00	4.64E-03	20.11	8.00	1.19E-02	18.24	16.32	2.64E-01	45.29	29.26	1.22E-01	4.98	11.79	6.73E-01
cg10768875	-16.36	5.78	4.64E-03	-29.34	14.28	3.99E-02	-38.57	24.82	1.20E-01	-11.90	6.94	8.66E-02	-13.69	19.36	4.80E-01
cg07950220	23.09	8.16	4.64E-03	16.74	20.07	4.04E-01	-30.87	38.01	4.17E-01	29.11	9.89	3.24E-03	17.90	24.84	4.71E-01
cg11265516	-53.97	19.07	4.64E-03	-58.59	28.58	4.04E-02	10.24	64.74	8.74E-01	-104.58	42.43	1.37E-02	-28.80	36.93	4.36E-01
cg03265037	23.14	8.18	4.65E-03	24.04	19.21	2.11E-01	25.13	20.52	2.21E-01	21.11	10.99	5.48E-02	29.25	25.05	2.43E-01
cg26332258	-67.51	23.85	4.65E-03	-64.80	49.67	1.92E-01	-127.49	253.38	6.15E-01	-72.38	29.75	1.50E-02	-41.75	69.45	5.48E-01
cg09219813	30.21	10.67	4.65E-03	36.87	12.34	2.81E-03	-30.41	46.94	5.17E-01	70.93	65.94	2.82E-01	13.42	25.61	6.00E-01
cg21541833	14.98	5.29	4.66E-03	21.49	14.63	1.42E-01	82.02	59.36	1.67E-01	15.02	6.24	1.61E-02	4.95	14.11	7.26E-01
cg13931707	-11.34	4.01	4.66E-03	-9.41	8.17	2.50E-01	-21.09	14.87	1.56E-01	-9.33	5.20	7.30E-02	-21.55	13.14	1.01E-01
cg07685977	-16.45	5.81	4.67E-03	-16.86	10.27	1.01E-01	-13.10	12.54	2.96E-01	-14.63	11.12	1.88E-01	-22.11	13.30	9.65E-02
cg23520688	-33.55	11.86	4.67E-03	-36.40	17.59	3.85E-02	-4.81	26.71	8.57E-01	-47.40	30.31	1.18E-01	-45.12	26.86	9.31E-02
cg06912939	24.64	8.71	4.68E-03	20.95	15.70	1.82E-01	34.38	16.34	3.55E-02	11.09	17.93	5.36E-01	33.79	21.02	1.08E-01
cg08330404	30.09	10.64	4.68E-03	30.38	19.72	1.24E-01	19.36	31.55	5.40E-01	35.29	16.83	3.59E-02	25.28	24.08	2.94E-01
cg04031465	25.70	9.09	4.68E-03	17.86	20.49	3.84E-01	69.95	44.69	1.18E-01	24.08	11.12	3.03E-02	34.10	29.61	2.50E-01
cg14430698	-12.85	4.54	4.69E-03	-13.67	9.64	1.56E-01	-34.68	19.26	7.17E-02	-10.45	5.79	7.09E-02	-13.64	13.99	3.30E-01
cg13700094	-17.62	6.23	4.69E-03	-37.00	18.24	4.25E-02	-28.15	25.58	2.71E-01	-13.90	7.24	5.49E-02	-16.08	21.68	4.58E-01
cg11359720	7.22	2.55	4.69E-03	8.71	3.48	1.23E-02	2.57	5.97	6.67E-01	4.75	9.48	6.16E-01	8.30	5.61	1.39E-01
cg02588889	-7.93	2.81	4.69E-03	-10.23	3.93	9.21E-03	-2.53	6.41	6.93E-01	-9.94	10.83	3.58E-01	-6.76	5.84	2.47E-01
cg23901918	9.14	3.23	4.70E-03	16.11	4.58	4.34E-04	5.47	5.95	3.58E-01	7.14	6.92	3.02E-01	2.90	6.58	6.60E-01
cg11148581	-46.97	16.61	4.70E-03	-70.65	25.97	6.53E-03	29.51	91.51	7.47E-01	-41.14	25.00	9.98E-02	-7.43	48.74	8.79E-01
cg07396827	-18.21	6.44	4.70E-03	-8.51	15.70	5.88E-01	-15.47	25.32	5.41E-01	-18.97	7.90	1.63E-02	-31.19	20.28	1.24E-01

cg25250717	15.33	5.43	4.71E-03	17.75	15.56	2.54E-01	24.07	8.28	3.65E-03	8.14	8.59	3.44E-01	-7.94	24.13	7.42E-01
cg11361201	10.59	3.75	4.72E-03	14.25	5.05	4.78E-03	11.99	9.38	2.01E-01	11.03	10.80	3.07E-01	-2.97	9.13	7.45E-01
cg20674174	-17.01	6.02	4.72E-03	-19.31	16.39	2.39E-01	-32.92	33.25	3.22E-01	-13.26	6.95	5.64E-02	-41.30	21.05	4.98E-02
cg18646864	21.32	7.55	4.72E-03	20.70	11.38	6.89E-02	29.71	15.92	6.21E-02	23.61	17.50	1.77E-01	7.71	19.51	6.93E-01
cg01272393	-16.89	5.98	4.73E-03	-2.00	19.53	9.19E-01	-25.70	25.04	3.05E-01	-18.92	6.69	4.70E-03	-2.70	26.51	9.19E-01
cg01831771	-27.93	9.89	4.73E-03	-29.74	15.93	6.19E-02	10.61	45.29	8.15E-01	-27.98	17.61	1.12E-01	-32.36	19.68	1.00E-01
cg27342720	-9.88	3.50	4.73E-03	-17.39	7.62	2.24E-02	-16.10	12.39	1.94E-01	-8.41	4.56	6.48E-02	0.24	10.10	9.81E-01
cg09301222	18.12	6.41	4.73E-03	0.77	21.35	9.71E-01	26.00	57.08	6.49E-01	20.54	6.96	3.16E-03	5.61	29.55	8.50E-01
cg19942977	-12.71	4.50	4.74E-03	-2.08	12.43	8.67E-01	2.76	22.30	9.02E-01	-16.40	5.23	1.69E-03	-4.43	15.24	7.71E-01
cg02127689	-17.58	6.23	4.74E-03	-17.95	9.43	5.69E-02	-16.80	13.71	2.20E-01	-10.29	15.54	5.08E-01	-23.50	14.01	9.35E-02
cg13790810	-16.99	6.02	4.75E-03	-19.23	12.40	1.21E-01	-28.28	15.50	6.81E-02	-9.86	9.10	2.79E-01	-21.96	14.29	1.24E-01
cg14339007	34.99	12.39	4.75E-03	37.78	17.46	3.05E-02	22.82	26.18	3.84E-01	82.75	43.92	5.95E-02	22.12	28.21	4.33E-01
cg05401965	22.33	7.91	4.75E-03	28.73	12.43	2.08E-02	0.31	21.15	9.88E-01	34.74	22.07	1.15E-01	18.94	13.83	1.71E-01
cg03366574	-13.71	4.85	4.75E-03	-18.60	6.84	6.58E-03	-8.27	10.40	4.27E-01	1.94	16.45	9.06E-01	-14.17	11.09	2.02E-01
cg10692932	-46.13	16.34	4.76E-03	-38.33	32.45	2.38E-01	-432.79	249.84	8.32E-02	-42.63	19.86	3.19E-02	-87.11	63.76	1.72E-01
cg18730437	22.57	7.99	4.76E-03	11.04	15.03	4.63E-01	6.15	38.00	8.71E-01	28.99	10.68	6.67E-03	26.07	23.79	2.73E-01
cg06609646	-16.30	5.78	4.76E-03	-22.19	15.18	1.44E-01	-52.21	27.50	5.76E-02	-12.81	6.73	5.72E-02	-18.10	21.01	3.89E-01
cg19255333	-38.11	13.50	4.76E-03	-24.10	19.45	2.15E-01	18.90	52.42	7.18E-01	-52.85	27.97	5.88E-02	-66.24	27.02	1.42E-02
cg10800369	-7.68	2.72	4.77E-03	-13.44	4.39	2.22E-03	-4.73	6.31	4.54E-01	-5.12	5.24	3.29E-01	-1.63	6.80	8.10E-01
cg05682094	64.23	22.76	4.77E-03	75.95	28.16	7.01E-03	-129.99	138.83	3.49E-01	76.39	88.08	3.86E-01	51.42	45.21	2.55E-01
cg11588903	11.02	3.91	4.77E-03	12.32	5.98	3.93E-02	18.52	8.53	2.99E-02	6.82	8.09	3.99E-01	2.25	10.79	8.35E-01
cg19201019	37.25	13.20	4.78E-03	45.23	15.68	3.92E-03	41.69	18.53	2.45E-02	8.41	12.71	5.08E-01	72.71	25.66	4.60E-03
cg02300377	33.12	11.74	4.78E-03	27.83	17.21	1.06E-01	34.62	31.75	2.76E-01	53.01	28.55	6.34E-02	28.30	24.55	2.49E-01
cg27070808	17.89	6.34	4.78E-03	23.99	19.68	2.23E-01	30.33	27.68	2.73E-01	16.11	7.10	2.32E-02	21.00	29.91	4.83E-01
cg15344504	-10.60	3.76	4.79E-03	-7.07	7.40	3.39E-01	-8.41	9.44	3.73E-01	-12.12	5.46	2.64E-02	-15.58	11.38	1.71E-01
cg00436254	24.92	8.83	4.79E-03	30.13	15.31	4.90E-02	14.06	15.98	3.79E-01	24.23	21.23	2.54E-01	33.99	20.37	9.52E-02
cg02030452	-17.13	6.07	4.79E-03	-4.55	17.45	7.94E-01	-31.58	29.35	2.82E-01	-17.63	6.82	9.76E-03	-28.56	28.88	3.23E-01
cg03604930	-16.88	5.98	4.79E-03	-19.41	10.00	5.23E-02	-14.71	12.54	2.41E-01	-6.38	13.97	6.48E-01	-23.40	12.44	5.99E-02
cg22532843	32.93	11.67	4.79E-03	30.64	15.81	5.26E-02	-2.43	52.40	9.63E-01	78.63	65.20	2.28E-01	37.06	19.12	5.26E-02
cg03107393	17.69	6.27	4.79E-03	12.74	8.34	1.27E-01	21.07	16.44	2.00E-01	33.70	24.81	1.74E-01	23.36	13.20	7.69E-02
cg23030159	10.23	3.63	4.79E-03	6.92	5.35	1.96E-01	18.19	7.23	1.19E-02	14.81	11.27	1.89E-01	5.08	8.40	5.45E-01
cg22426397	-33.39	11.84	4.80E-03	-34.29	15.30	2.50E-02	-48.15	31.49	1.26E-01	-19.49	37.70	6.05E-01	-25.60	29.53	3.86E-01
cg14370653	-13.45	4.77	4.81E-03	-27.51	16.18	8.90E-02	-25.07	24.67	3.10E-01	-12.87	5.24	1.41E-02	11.27	21.87	6.06E-01
cg20387341	29.10	10.32	4.81E-03	39.99	16.73	1.68E-02	31.21	36.89	3.98E-01	31.20	20.33	1.25E-01	11.97	19.39	5.37E-01
cg03006588	25.49	9.04	4.81E-03	22.78	15.97	1.54E-01	30.84	18.16	8.94E-02	19.07	15.63	2.23E-01	42.92	29.01	1.39E-01

cg16002660	-13.72	4.87	4.81E-03	-16.81	10.91	1.23E-01	-30.48	16.75	6.88E-02	-7.92	6.52	2.24E-01	-21.39	12.24	8.05E-02
cg21157904	12.45	4.42	4.82E-03	11.58	5.49	3.49E-02	26.77	11.98	2.54E-02	3.12	19.23	8.71E-01	7.02	10.93	5.21E-01
cg21151163	-11.92	4.23	4.82E-03	-7.02	6.66	2.91E-01	-10.95	8.54	1.99E-01	-22.27	6.48	5.96E-04	-4.30	8.54	6.15E-01
cg13849253	19.78	7.02	4.82E-03	32.04	12.57	1.08E-02	27.19	13.93	5.09E-02	22.77	12.34	6.49E-02	2.17	10.97	8.43E-01
cg05539265	-13.00	4.61	4.82E-03	-27.08	11.12	1.49E-02	-18.38	17.84	3.03E-01	-8.93	5.75	1.20E-01	-11.59	13.38	3.86E-01
cg02658668	-19.32	6.86	4.82E-03	-29.44	13.16	2.52E-02	17.46	30.16	5.63E-01	-15.07	7.31	3.91E-02	-39.51	21.07	6.07E-02
cg11554728	18.35	6.51	4.82E-03	22.55	11.27	4.54E-02	21.80	11.98	6.88E-02	15.78	14.09	2.63E-01	6.48	16.39	6.93E-01
cg26165853	-34.73	12.32	4.83E-03	-40.11	16.68	1.62E-02	-37.61	46.71	4.21E-01	-99.65	66.92	1.36E-01	-19.49	20.81	3.49E-01
cg07565028	23.46	8.32	4.83E-03	14.52	21.29	4.95E-01	20.30	33.61	5.46E-01	28.98	9.86	3.28E-03	-9.22	30.87	7.65E-01
cg20060632	14.03	4.98	4.83E-03	30.21	11.04	6.19E-03	12.62	13.94	3.65E-01	10.21	6.39	1.10E-01	1.08	20.06	9.57E-01
cg00886554	34.45	12.23	4.84E-03	40.86	18.21	2.49E-02	27.96	29.03	3.35E-01	74.89	47.72	1.17E-01	20.10	22.11	3.63E-01
cg22904406	11.86	4.21	4.85E-03	10.43	5.52	5.87E-02	15.54	9.53	1.03E-01	15.28	14.87	3.04E-01	10.76	11.16	3.35E-01
cg10253371	20.77	7.37	4.85E-03	26.97	11.41	1.81E-02	4.50	19.30	8.16E-01	53.97	25.84	3.68E-02	13.20	11.84	2.65E-01
cg08519233	-29.78	10.57	4.85E-03	-39.24	17.89	2.83E-02	-56.58	26.61	3.35E-02	-13.22	20.19	5.13E-01	-16.09	22.59	4.76E-01
cg20730198	14.45	5.13	4.85E-03	15.02	7.25	3.84E-02	-0.65	17.68	9.70E-01	13.74	24.15	5.69E-01	17.19	8.43	4.13E-02
cg27038472	23.18	8.23	4.85E-03	25.65	21.26	2.28E-01	24.56	20.98	2.42E-01	16.74	10.03	9.52E-02	73.70	31.18	1.81E-02
cg12746717	31.14	11.06	4.85E-03	40.80	31.77	1.99E-01	67.78	85.76	4.29E-01	27.72	12.43	2.57E-02	44.13	41.41	2.87E-01
cg01676795	7.38	2.62	4.86E-03	7.13	3.60	4.75E-02	10.33	6.56	1.16E-01	11.09	6.06	6.71E-02	-1.00	7.46	8.93E-01
cg08833010	-19.30	6.85	4.87E-03	-19.53	10.76	6.97E-02	-26.14	24.57	2.87E-01	-26.41	11.31	1.95E-02	2.43	17.77	8.91E-01
cg06456738	10.25	3.64	4.87E-03	9.72	4.92	4.79E-02	19.94	7.97	1.23E-02	16.78	16.65	3.14E-01	1.17	7.65	8.79E-01
cg11822964	13.69	4.86	4.87E-03	12.25	7.43	9.93E-02	19.46	9.10	3.25E-02	7.81	13.01	5.48E-01	12.26	12.70	3.34E-01
cg17115737	12.83	4.56	4.88E-03	19.91	6.01	9.20E-04	14.51	9.18	1.14E-01	1.00	9.27	9.14E-01	5.09	12.52	6.85E-01
cg16478536	9.45	3.36	4.88E-03	6.62	5.04	1.89E-01	18.29	7.09	9.93E-03	9.01	10.28	3.81E-01	6.44	7.08	3.63E-01
cg12494529	-15.64	5.56	4.90E-03	-26.04	8.05	1.22E-03	-2.70	11.81	8.19E-01	-5.49	12.52	6.61E-01	-17.47	9.86	7.65E-02
cg19719807	13.09	4.65	4.91E-03	17.24	6.83	1.16E-02	20.87	11.13	6.08E-02	9.33	12.02	4.37E-01	0.19	10.12	9.85E-01
cg00774088	-32.19	11.44	4.91E-03	-36.97	12.89	4.15E-03	-87.84	51.85	9.03E-02	-39.11	26.87	1.46E-01	2.56	25.08	9.19E-01
cg10599362	-61.83	21.98	4.91E-03	-101.69	51.45	4.81E-02	-166.49	259.32	5.21E-01	-53.59	25.93	3.88E-02	-38.97	72.51	5.91E-01
cg18108716	37.48	13.33	4.91E-03	25.77	30.51	3.98E-01	97.84	120.11	4.15E-01	41.50	16.29	1.08E-02	28.06	37.36	4.53E-01
cg03681150	36.28	12.90	4.92E-03	43.22	18.88	2.21E-02	13.73	28.87	6.34E-01	52.39	37.06	1.57E-01	33.05	28.00	2.38E-01
cg01359532	-11.73	4.17	4.92E-03	-12.67	7.14	7.60E-02	-12.55	8.89	1.58E-01	-4.67	8.78	5.95E-01	-16.85	9.04	6.22E-02
cg01697354	40.38	14.36	4.92E-03	35.43	16.45	3.12E-02	69.95	70.74	3.23E-01	67.69	62.68	2.80E-01	48.11	37.77	2.03E-01
cg17018021	26.75	9.51	4.92E-03	16.17	15.65	3.02E-01	20.01	21.98	3.63E-01	40.04	22.04	6.93E-02	37.25	18.76	4.71E-02
cg26160945	8.57	3.05	4.92E-03	11.31	4.30	8.54E-03	3.28	7.82	6.75E-01	12.55	6.66	5.94E-02	-1.75	8.25	8.32E-01
cg10541813	28.56	10.16	4.92E-03	24.62	11.52	3.26E-02	50.95	80.91	5.29E-01	25.64	55.45	6.44E-01	44.71	24.37	6.66E-02
cg00843019	-15.65	5.57	4.93E-03	-37.16	16.68	2.59E-02	-30.27	27.11	2.64E-01	-12.99	6.50	4.55E-02	-6.19	16.63	7.10E-01

cg08309809	29.24	10.40	4.93E-03	28.22	27.44	3.04E-01	31.45	87.77	7.20E-01	31.11	12.16	1.05E-02	17.83	31.35	5.69E-01
cg10949706	20.25	7.20	4.93E-03	19.21	11.43	9.29E-02	1.29	18.30	9.44E-01	26.81	20.18	1.84E-01	28.06	12.71	2.72E-02
cg12630522	-37.33	13.28	4.95E-03	-32.94	41.52	4.28E-01	-117.45	182.69	5.20E-01	-44.24	14.82	2.83E-03	24.67	44.49	5.79E-01
cg13145289	23.70	8.44	4.96E-03	32.08	13.88	2.08E-02	26.50	16.05	9.88E-02	9.94	19.64	6.13E-01	15.89	20.45	4.37E-01
cg09247392	-21.83	7.77	4.96E-03	-37.20	12.27	2.42E-03	-21.73	15.02	1.48E-01	-0.99	20.33	9.61E-01	-10.35	15.59	5.07E-01
cg14506751	-66.68	23.73	4.96E-03	-69.66	70.38	3.22E-01	-469.67	337.03	1.63E-01	-59.41	26.80	2.66E-02	-101.49	76.25	1.83E-01
cg25573386	-13.14	4.68	4.96E-03	-16.05	7.82	4.02E-02	-15.95	10.50	1.29E-01	-6.40	9.30	4.91E-01	-13.68	10.68	2.00E-01
cg00049892	-12.64	4.50	4.96E-03	-14.41	11.08	1.94E-01	-19.68	21.47	3.59E-01	-13.03	5.52	1.81E-02	-5.79	12.73	6.49E-01
cg03658294	59.39	21.14	4.97E-03	49.12	27.11	7.00E-02	25.68	122.94	8.35E-01	127.96	56.20	2.28E-02	48.24	45.00	2.84E-01
cg21994576	27.17	9.67	4.97E-03	41.58	19.41	3.22E-02	-5.93	40.54	8.84E-01	26.09	13.12	4.67E-02	19.83	24.84	4.25E-01
cg00011350	32.95	11.73	4.97E-03	28.28	16.98	9.58E-02	66.35	24.54	6.84E-03	23.77	47.94	6.20E-01	12.22	24.24	6.14E-01
cg03640151	12.48	4.44	4.97E-03	7.35	6.48	2.57E-01	10.81	8.53	2.05E-01	25.30	13.15	5.43E-02	22.18	11.69	5.78E-02
cg24212047	-19.00	6.77	4.99E-03	-23.13	15.62	1.39E-01	-11.82	14.16	4.04E-01	-22.05	9.84	2.50E-02	-13.84	20.31	4.96E-01
cg09501469	21.21	7.55	4.99E-03	11.90	15.07	4.30E-01	1.02	27.53	9.71E-01	22.31	9.88	2.39E-02	57.60	25.40	2.33E-02
cg26331991	33.93	12.08	4.99E-03	44.65	15.64	4.31E-03	56.49	23.31	1.54E-02	1.84	31.67	9.54E-01	7.86	24.46	7.48E-01
cg05281796	9.86	3.51	5.00E-03	7.23	5.59	1.96E-01	10.73	6.00	7.38E-02	26.58	15.24	8.11E-02	9.15	7.66	2.33E-01
cg16217297	19.79	7.05	5.00E-03	2.09	14.95	8.89E-01	8.32	30.48	7.85E-01	28.85	8.87	1.14E-03	6.98	23.29	7.64E-01
cg27263151	-14.71	5.24	5.00E-03	-21.46	13.84	1.21E-01	-35.09	28.38	2.16E-01	-13.28	6.18	3.15E-02	-8.54	16.37	6.02E-01
cg21447620	24.52	8.74	5.00E-03	25.30	14.87	8.89E-02	14.91	16.50	3.66E-01	15.02	16.38	3.59E-01	61.67	23.77	9.48E-03
cg17469039	22.19	7.91	5.01E-03	9.52	15.49	5.39E-01	22.90	15.95	1.51E-01	28.82	12.96	2.62E-02	27.62	22.69	2.23E-01
cg23184070	16.84	6.00	5.01E-03	16.73	8.43	4.72E-02	8.55	15.92	5.91E-01	17.14	20.55	4.04E-01	21.35	11.62	6.61E-02
cg25101396	11.29	4.02	5.01E-03	10.15	5.23	5.22E-02	18.31	9.02	4.25E-02	13.65	19.03	4.73E-01	6.27	9.94	5.28E-01
cg24482314	-14.29	5.09	5.01E-03	-29.58	13.90	3.33E-02	5.43	29.10	8.52E-01	-10.43	5.94	7.93E-02	-27.86	15.97	8.10E-02
cg09295573	11.28	4.02	5.02E-03	11.32	4.36	9.42E-03	6.33	25.14	8.01E-01	23.20	13.39	8.33E-02	-16.56	21.34	4.38E-01
cg01663469	29.07	10.36	5.02E-03	29.64	15.32	5.30E-02	3.58	29.03	9.02E-01	47.54	29.64	1.09E-01	31.55	19.13	9.90E-02
cg08249608	36.29	12.93	5.02E-03	29.51	16.59	7.53E-02	88.33	40.59	2.95E-02	29.87	45.36	5.10E-01	33.20	28.24	2.40E-01
cg19416088	43.03	15.34	5.02E-03	46.63	35.88	1.94E-01	47.67	188.95	8.01E-01	45.46	18.44	1.37E-02	23.11	44.41	6.03E-01
cg01693650	41.88	14.93	5.03E-03	51.19	20.85	1.41E-02	5.43	51.28	9.16E-01	43.92	46.08	3.41E-01	35.50	27.38	1.95E-01
cg06880365	22.09	7.87	5.03E-03	18.97	10.79	7.87E-02	-7.89	27.05	7.71E-01	27.46	15.47	7.59E-02	44.78	22.39	4.55E-02
cg04208237	-45.05	16.06	5.04E-03	-62.03	24.55	1.15E-02	-10.30	39.92	7.96E-01	-34.34	40.62	3.98E-01	-45.19	31.89	1.56E-01
cg09355820	-20.61	7.35	5.04E-03	-26.60	11.62	2.21E-02	-19.21	37.28	6.06E-01	-13.01	11.23	2.47E-01	-27.48	20.16	1.73E-01
cg02602413	-19.21	6.85	5.04E-03	-22.90	11.20	4.08E-02	-10.34	15.09	4.93E-01	-28.09	15.19	6.44E-02	-12.93	14.72	3.80E-01
cg17749468	-55.32	19.72	5.04E-03	-90.36	59.28	1.27E-01	-315.94	265.98	2.35E-01	-45.82	21.67	3.45E-02	-101.63	83.85	2.26E-01
cg12729822	10.06	3.59	5.04E-03	10.83	4.86	2.56E-02	8.38	8.03	2.97E-01	9.31	8.99	3.00E-01	10.38	11.58	3.70E-01
cg05012825	31.96	11.39	5.04E-03	26.32	41.76	5.29E-01	-83.73	164.21	6.10E-01	34.33	12.59	6.39E-03	22.41	35.80	5.31E-01

cg24636477	22.12	7.89	5.04E-03	31.03	25.13	2.17E-01	20.20	40.89	6.21E-01	29.80	9.51	1.72E-03	-2.31	16.14	8.86E-01
cg03970086	21.68	7.73	5.05E-03	27.41	15.93	8.53E-02	24.75	15.80	1.17E-01	19.07	11.78	1.05E-01	11.45	25.19	6.50E-01
cg04538990	-11.79	4.21	5.05E-03	-18.10	8.37	3.05E-02	-4.68	13.83	7.35E-01	-10.56	5.74	6.58E-02	-9.48	12.26	4.39E-01
cg00755836	41.20	14.70	5.05E-03	43.48	18.43	1.84E-02	41.12	94.38	6.63E-01	88.34	62.93	1.60E-01	27.15	27.50	3.23E-01
cg20059682	-19.36	6.90	5.05E-03	-22.99	12.51	6.60E-02	12.53	27.33	6.47E-01	-23.75	9.69	1.42E-02	-8.81	19.61	6.53E-01
cg00560747	-13.01	4.64	5.06E-03	-15.94	6.28	1.11E-02	-4.18	12.31	7.34E-01	-14.21	14.50	3.27E-01	-10.75	10.15	2.90E-01
cg07135303	22.67	8.08	5.06E-03	24.90	12.63	4.87E-02	23.40	15.34	1.27E-01	15.90	21.57	4.61E-01	21.69	19.50	2.66E-01
cg01024455	19.58	6.98	5.06E-03	20.56	10.57	5.17E-02	17.68	19.07	3.54E-01	24.16	16.89	1.53E-01	15.87	13.74	2.48E-01
cg22316093	6.37	2.27	5.06E-03	7.55	3.21	1.88E-02	1.36	4.87	7.79E-01	8.77	8.81	3.20E-01	7.98	4.92	1.04E-01
cg00578039	11.95	4.26	5.07E-03	10.00	5.46	6.69E-02	21.17	11.38	6.28E-02	24.80	12.73	5.14E-02	0.67	11.52	9.54E-01
cg04012896	34.04	12.15	5.07E-03	16.63	19.87	4.03E-01	48.65	36.51	1.83E-01	25.11	9.89	1.11E-02	80.54	27.07	2.93E-03
cg21225501	-60.00	21.41	5.07E-03	-51.98	32.83	1.13E-01	161.24	141.97	2.56E-01	-88.71	49.72	7.44E-02	-68.51	35.37	5.27E-02
cg21671806	20.37	7.27	5.08E-03	21.68	13.85	1.17E-01	28.73	18.64	1.23E-01	21.20	11.49	6.49E-02	8.93	17.54	6.11E-01
cg08977791	28.54	10.19	5.08E-03	13.69	25.98	5.98E-01	26.12	53.93	6.28E-01	27.75	11.89	1.95E-02	67.35	36.94	6.83E-02
cg21127735	-17.93	6.40	5.08E-03	-15.52	8.60	7.12E-02	-27.12	17.27	1.16E-01	-23.38	25.70	3.63E-01	-16.86	12.87	1.90E-01
cg02873098	8.71	3.11	5.08E-03	6.09	4.24	1.51E-01	17.96	7.13	1.18E-02	12.62	7.46	9.09E-02	1.82	7.79	8.15E-01
cg23697467	27.58	9.85	5.09E-03	34.98	20.70	9.11E-02	31.46	21.76	1.48E-01	27.22	14.37	5.82E-02	4.48	31.25	8.86E-01
cg17679470	-16.00	5.71	5.09E-03	-20.42	10.67	5.58E-02	-16.63	12.34	1.78E-01	-14.80	10.85	1.73E-01	-11.20	12.12	3.55E-01
cg05961802	36.08	12.88	5.10E-03	43.34	25.26	8.63E-02	13.41	57.09	8.14E-01	26.42	17.13	1.23E-01	74.33	36.63	4.25E-02
cg03654841	-9.05	3.23	5.10E-03	-8.82	5.93	1.37E-01	-15.62	10.97	1.54E-01	-8.12	4.53	7.31E-02	-8.76	9.84	3.74E-01
cg11391618	-16.26	5.81	5.11E-03	-30.86	12.19	1.14E-02	-1.22	32.00	9.70E-01	-16.26	7.75	3.60E-02	-0.56	13.71	9.67E-01
cg20666967	-40.88	14.60	5.11E-03	-31.43	20.97	1.34E-01	-7.74	47.55	8.71E-01	-28.92	44.27	5.14E-01	-69.74	26.14	7.62E-03
cg21007852	13.38	4.78	5.11E-03	14.10	6.47	2.93E-02	13.81	12.09	2.53E-01	18.46	14.25	1.95E-01	7.84	11.09	4.80E-01
cg12316887	-14.54	5.19	5.11E-03	-24.48	11.12	2.76E-02	-33.83	19.15	7.73E-02	-7.87	5.67	1.65E-01	-20.03	14.18	1.58E-01
cg16281600	-12.20	4.36	5.12E-03	-12.53	6.93	7.06E-02	-16.40	10.42	1.15E-01	-12.16	10.00	2.24E-01	-8.62	8.91	3.33E-01
cg26966246	30.52	10.90	5.12E-03	67.88	39.49	8.56E-02	-107.21	141.51	4.49E-01	29.08	11.71	1.30E-02	15.17	48.30	7.54E-01
cg03502812	15.03	5.37	5.13E-03	6.00	9.27	5.17E-01	19.74	9.97	4.77E-02	15.49	12.98	2.33E-01	22.76	11.89	5.55E-02
cg09814128	-32.76	11.70	5.13E-03	-30.33	25.69	2.38E-01	-85.61	49.15	8.15E-02	-34.95	14.97	1.96E-02	-1.99	33.15	9.52E-01
cg18786593	15.11	5.40	5.14E-03	3.25	12.04	7.87E-01	47.33	29.48	1.08E-01	14.17	6.54	3.03E-02	28.72	14.54	4.83E-02
cg22169206	27.84	9.95	5.14E-03	34.20	12.16	4.90E-03	-22.83	39.27	5.61E-01	19.73	43.13	6.47E-01	25.14	21.60	2.44E-01
cg16494108	10.30	3.68	5.15E-03	5.10	6.37	4.23E-01	14.22	8.05	7.75E-02	13.17	6.47	4.18E-02	10.17	10.06	3.12E-01
cg22758189	23.96	8.57	5.15E-03	19.93	17.42	2.52E-01	2.63	20.48	8.98E-01	36.24	12.58	3.95E-03	15.64	24.80	5.28E-01
cg20272619	32.43	11.59	5.16E-03	38.89	16.41	1.78E-02	6.78	34.74	8.45E-01	41.37	37.93	2.75E-01	28.36	21.31	1.83E-01
cg23878264	-6.57	2.35	5.16E-03	-7.12	4.46	1.10E-01	-7.81	6.76	2.48E-01	-8.11	3.51	2.09E-02	-0.10	6.00	9.86E-01
cg05605980	16.66	5.96	5.16E-03	18.09	12.36	1.43E-01	4.15	21.36	8.46E-01	18.35	7.68	1.69E-02	12.41	19.93	5.34E-01

cg08099797	-10.06	3.60	5.16E-03	-9.46	5.12	6.48E-02	-6.69	8.42	4.27E-01	-12.42	9.91	2.10E-01	-13.17	8.19	1.08E-01
cg19500851	27.93	9.99	5.16E-03	22.76	17.80	2.01E-01	28.35	20.12	1.59E-01	20.22	18.59	2.77E-01	52.94	25.79	4.01E-02
cg16374656	6.84	2.45	5.17E-03	6.42	3.52	6.83E-02	8.09	6.88	2.40E-01	11.19	4.84	2.06E-02	-1.02	6.62	8.77E-01
cg08379897	19.65	7.03	5.17E-03	20.51	10.35	4.76E-02	50.02	19.27	9.45E-03	10.42	19.86	6.00E-01	11.28	10.73	2.93E-01
cg24930634	46.53	16.64	5.17E-03	98.93	58.91	9.31E-02	-114.05	250.12	6.48E-01	43.19	18.00	1.64E-02	36.50	67.17	5.87E-01
cg04699199	-14.75	5.28	5.18E-03	-18.07	11.75	1.24E-01	-4.29	12.94	7.41E-01	-12.96	7.56	8.67E-02	-28.04	13.81	4.23E-02
cg13671109	23.58	8.43	5.18E-03	13.57	29.50	6.46E-01	-61.17	136.29	6.54E-01	26.41	9.06	3.56E-03	-3.70	38.46	9.23E-01
cg21548155	18.83	6.74	5.19E-03	30.03	12.73	1.83E-02	17.40	15.92	2.74E-01	12.40	12.32	3.14E-01	14.85	13.69	2.78E-01
cg19664013	8.48	3.03	5.19E-03	8.46	4.08	3.82E-02	6.18	7.27	3.95E-01	16.02	10.60	1.31E-01	7.40	6.93	2.86E-01
cg08423507	-52.79	18.89	5.20E-03	-55.03	23.03	1.69E-02	86.83	80.01	2.78E-01	-64.95	44.82	1.47E-01	-69.01	33.58	3.99E-02
cg03755955	20.73	7.42	5.20E-03	17.48	17.71	3.24E-01	31.04	17.91	8.31E-02	17.44	10.03	8.19E-02	26.36	22.80	2.48E-01
cg23055159	33.43	11.96	5.21E-03	20.25	18.55	2.75E-01	-40.49	72.79	5.78E-01	53.97	17.89	2.55E-03	19.52	34.55	5.72E-01
cg18762422	-14.85	5.32	5.22E-03	-21.63	5.92	2.57E-04	-2.02	23.34	9.31E-01	-3.15	8.58	7.14E-01	-19.88	13.49	1.41E-01
cg05229965	29.51	10.57	5.23E-03	26.99	13.27	4.21E-02	4.04	40.18	9.20E-01	80.15	39.75	4.37E-02	28.52	22.19	1.99E-01
cg05446010	-56.92	20.38	5.23E-03	-68.85	67.48	3.08E-01	-497.29	303.80	1.02E-01	-56.09	22.37	1.22E-02	-24.82	74.82	7.40E-01
cg05663418	-40.95	14.67	5.24E-03	-70.64	36.34	5.19E-02	-191.19	187.90	3.09E-01	-33.63	17.14	4.97E-02	-36.97	46.70	4.28E-01
cg20686446	-45.32	16.23	5.24E-03	-44.03	21.54	4.09E-02	-2.52	68.19	9.71E-01	-47.01	49.95	3.47E-01	-56.38	31.26	7.13E-02
cg02552255	13.59	4.87	5.24E-03	14.42	6.89	3.63E-02	23.29	10.83	3.14E-02	4.04	15.92	7.99E-01	6.35	10.73	5.54E-01
cg02774439	31.72	11.36	5.24E-03	34.21	16.66	4.00E-02	42.08	31.85	1.86E-01	37.92	29.27	1.95E-01	18.45	22.40	4.10E-01
cg25620760	-7.34	2.63	5.24E-03	-8.31	4.19	4.74E-02	-12.73	5.95	3.24E-02	-6.33	5.15	2.19E-01	0.46	6.80	9.46E-01
cg08997628	33.87	12.13	5.24E-03	29.64	35.34	4.02E-01	110.42	98.21	2.61E-01	36.29	13.76	8.33E-03	5.15	40.73	8.99E-01
cg11328253	17.33	6.21	5.24E-03	18.84	10.49	7.25E-02	11.53	11.78	3.28E-01	23.07	16.53	1.63E-01	18.49	12.91	1.52E-01
cg03290730	-30.43	10.90	5.25E-03	-36.25	21.98	9.91E-02	21.33	50.08	6.70E-01	-31.33	14.04	2.56E-02	-35.01	33.85	3.01E-01
cg19979738	-21.54	7.72	5.25E-03	-22.29	10.01	2.59E-02	-0.35	37.74	9.93E-01	-10.99	21.34	6.07E-01	-29.32	15.98	6.66E-02
cg01734786	29.70	10.64	5.26E-03	26.96	14.73	6.73E-02	39.84	30.67	1.94E-01	43.28	34.37	2.08E-01	25.52	20.79	2.20E-01
cg20064897	10.33	3.70	5.26E-03	14.21	9.07	1.17E-01	21.48	13.42	1.09E-01	6.84	4.48	1.27E-01	22.41	13.62	1.00E-01
cg10982851	-24.99	8.95	5.26E-03	-27.75	16.55	9.36E-02	-36.87	47.80	4.40E-01	-30.05	12.03	1.25E-02	9.13	26.05	7.26E-01
cg20835725	49.07	17.58	5.26E-03	-15.76	53.32	7.68E-01	220.68	211.80	2.97E-01	54.43	19.65	5.61E-03	67.86	60.79	2.64E-01
cg01583034	40.27	14.43	5.26E-03	59.53	39.47	1.32E-01	162.41	230.76	4.82E-01	42.10	16.39	1.02E-02	-11.25	48.99	8.18E-01
cg01348293	15.13	5.42	5.27E-03	8.78	8.00	2.73E-01	22.03	11.07	4.66E-02	19.65	21.99	3.71E-01	19.24	11.06	8.20E-02
cg02703198	52.63	18.86	5.27E-03	85.58	50.58	9.06E-02	-211.44	218.26	3.33E-01	45.99	21.28	3.07E-02	90.90	72.31	2.09E-01
cg27298170	-18.69	6.70	5.27E-03	-15.14	18.84	4.21E-01	-27.96	35.24	4.27E-01	-18.19	7.63	1.71E-02	-26.41	26.13	3.12E-01
cg22153345	68.62	24.60	5.28E-03	96.81	35.99	7.15E-03	131.72	124.93	2.92E-01	43.91	58.42	4.52E-01	33.17	43.69	4.48E-01
cg05141578	18.95	6.80	5.28E-03	22.34	10.68	3.64E-02	16.29	14.48	2.61E-01	15.09	14.74	3.06E-01	19.19	16.87	2.55E-01
cg18195384	41.00	14.70	5.29E-03	47.79	17.36	5.91E-03	9.77	77.67	9.00E-01	56.31	79.51	4.79E-01	20.92	31.87	5.11E-01

cg01981048	-9.39	3.37	5.29E-03	-9.24	5.02	6.56E-02	-13.15	6.96	5.87E-02	-4.93	10.58	6.41E-01	-7.70	7.27	2.90E-01
cg17339927	16.74	6.00	5.29E-03	12.57	8.65	1.46E-01	23.99	12.83	6.16E-02	19.03	20.19	3.46E-01	17.79	13.07	1.73E-01
cg11803184	21.32	7.65	5.31E-03	20.91	18.90	2.69E-01	30.18	21.55	1.61E-01	16.62	9.61	8.38E-02	46.66	27.67	9.17E-02
cg14560703	-7.81	2.80	5.31E-03	-6.30	4.40	1.52E-01	-8.84	5.89	1.34E-01	-9.05	5.92	1.26E-01	-8.50	7.36	2.48E-01
cg19418458	3.29	1.18	5.31E-03	2.05	1.68	2.22E-01	4.20	2.62	1.08E-01	2.70	4.02	5.03E-01	5.45	2.52	3.05E-02
cg01191660	-11.44	4.10	5.32E-03	-14.97	7.92	5.86E-02	-33.08	16.27	4.20E-02	-6.62	5.12	1.96E-01	-18.11	13.99	1.95E-01
cg22075362	-15.14	5.43	5.32E-03	-22.76	8.51	7.48E-03	-10.56	11.24	3.47E-01	-11.04	12.63	3.82E-01	-7.79	13.02	5.49E-01
cg26145873	-11.06	3.97	5.32E-03	-12.57	8.63	1.45E-01	-19.66	12.53	1.17E-01	-9.31	5.23	7.50E-02	-9.46	11.80	4.23E-01
cg04838847	-21.86	7.85	5.33E-03	-23.16	9.67	1.66E-02	-5.52	34.70	8.74E-01	-39.11	21.79	7.26E-02	-7.82	19.59	6.90E-01
cg22773522	-10.03	3.60	5.33E-03	-19.21	9.66	4.69E-02	-9.52	16.11	5.55E-01	-10.38	4.22	1.39E-02	7.99	12.49	5.22E-01
cg24531009	-21.59	7.75	5.33E-03	-29.87	13.73	2.97E-02	-23.20	15.68	1.39E-01	-21.70	16.81	1.97E-01	-8.03	16.35	6.23E-01
cg10141261	-9.69	3.48	5.33E-03	-11.91	6.47	6.58E-02	-18.32	11.84	1.22E-01	-8.05	4.89	1.00E-01	-5.08	10.01	6.12E-01
cg03002735	-16.16	5.80	5.35E-03	-17.46	8.57	4.16E-02	-12.85	13.54	3.43E-01	-16.35	14.43	2.57E-01	-16.06	13.09	2.20E-01
cg01571283	-22.08	7.93	5.35E-03	-13.33	19.47	4.93E-01	-24.92	45.90	5.87E-01	-21.87	9.31	1.89E-02	-41.28	28.12	1.42E-01
cg04278905	21.14	7.59	5.35E-03	23.13	12.11	5.61E-02	21.69	15.39	1.59E-01	20.12	15.35	1.90E-01	15.54	21.99	4.80E-01
cg07220782	15.06	5.41	5.36E-03	10.73	11.41	3.47E-01	20.82	12.51	9.59E-02	13.83	10.40	1.83E-01	15.76	9.59	1.00E-01
cg01550716	43.97	15.79	5.36E-03	52.77	22.72	2.02E-02	2.88	61.87	9.63E-01	45.57	42.10	2.79E-01	38.19	28.30	1.77E-01
cg20738733	21.54	7.74	5.37E-03	21.73	20.77	2.95E-01	21.56	21.75	3.21E-01	21.47	9.58	2.50E-02	21.73	26.97	4.21E-01
cg03899643	-13.23	4.75	5.38E-03	-17.17	7.24	1.77E-02	-18.47	11.19	9.89E-02	1.00	11.20	9.29E-01	-12.85	10.40	2.17E-01
cg01729401	18.41	6.61	5.38E-03	25.56	12.24	3.68E-02	-8.11	22.07	7.13E-01	21.64	9.40	2.14E-02	7.85	18.84	6.77E-01
cg27311041	16.50	5.93	5.38E-03	16.57	15.39	2.82E-01	30.69	26.66	2.50E-01	15.88	6.84	2.02E-02	11.67	26.24	6.57E-01
cg02696169	15.56	5.59	5.38E-03	13.28	6.37	3.71E-02	14.99	7.84	5.60E-02	38.74	12.49	1.92E-03	3.95	9.57	6.80E-01
cg03007522	18.79	6.75	5.38E-03	44.45	21.35	3.74E-02	13.21	32.73	6.86E-01	19.69	8.26	1.72E-02	3.37	15.49	8.28E-01
cg23274923	23.94	8.60	5.39E-03	45.87	20.80	2.74E-02	18.53	22.33	4.07E-01	18.48	11.35	1.03E-01	25.73	26.49	3.32E-01
cg25970266	42.61	15.31	5.39E-03	33.39	42.77	4.35E-01	0.61	192.95	9.97E-01	40.26	17.08	1.84E-02	96.94	61.81	1.17E-01
cg18676237	-47.98	17.24	5.40E-03	-42.69	20.17	3.43E-02	58.07	99.52	5.60E-01	-129.31	79.90	1.06E-01	-64.94	39.32	9.87E-02
cg15724773	32.08	11.53	5.40E-03	26.65	33.11	4.21E-01	60.95	238.27	7.98E-01	27.78	12.67	2.83E-02	119.13	52.80	2.41E-02
cg26540302	-22.15	7.96	5.40E-03	-13.61	21.45	5.26E-01	-69.03	42.50	1.04E-01	-24.44	9.15	7.59E-03	9.08	30.02	7.62E-01
cg01907761	-11.49	4.13	5.41E-03	-22.13	7.80	4.57E-03	-0.54	9.68	9.55E-01	-9.15	5.86	1.18E-01	-12.45	9.99	2.13E-01
cg06266861	-24.14	8.68	5.41E-03	-22.02	19.12	2.49E-01	-88.27	50.20	7.86E-02	-23.34	10.43	2.53E-02	-11.27	32.38	7.28E-01
cg01783362	31.89	11.47	5.41E-03	8.12	40.66	8.42E-01	-41.01	139.59	7.69E-01	37.11	12.37	2.69E-03	-7.10	49.35	8.86E-01
cg21538834	16.88	6.07	5.42E-03	10.47	9.07	2.48E-01	28.86	11.44	1.16E-02	19.64	19.42	3.12E-01	12.37	14.57	3.96E-01
cg05098381	15.71	5.65	5.42E-03	23.46	9.89	1.77E-02	19.54	13.14	1.37E-01	12.01	9.16	1.90E-01	-1.18	17.16	9.45E-01
cg06633013	-22.16	7.97	5.43E-03	-20.52	9.91	3.83E-02	2.16	29.34	9.41E-01	-32.74	27.99	2.42E-01	-32.25	17.91	7.18E-02
cg26683252	-16.65	5.99	5.43E-03	-20.52	8.96	2.21E-02	3.95	19.58	8.40E-01	-26.76	16.75	1.10E-01	-13.37	10.40	1.98E-01

cg06488678	-13.82	4.97	5.44E-03	-21.45	13.70	1.17E-01	-29.93	28.26	2.89E-01	-11.51	5.73	4.47E-02	-16.50	16.97	3.31E-01
cg27132732	-12.56	4.52	5.44E-03	-19.75	8.06	1.42E-02	-8.23	11.05	4.56E-01	-6.84	7.68	3.73E-01	-15.15	10.90	1.65E-01
cg08118354	-13.41	4.82	5.44E-03	-15.85	7.31	3.02E-02	-10.63	11.31	3.47E-01	-11.00	13.80	4.25E-01	-12.39	9.44	1.89E-01
cg06411987	-19.31	6.95	5.45E-03	-21.60	17.82	2.26E-01	37.46	38.06	3.25E-01	-22.61	8.11	5.32E-03	-8.55	24.40	7.26E-01
cg13511885	11.72	4.22	5.45E-03	8.96	5.95	1.32E-01	22.25	10.44	3.30E-02	5.40	10.00	5.89E-01	16.83	10.69	1.16E-01
cg24623244	32.43	11.67	5.45E-03	39.33	15.90	1.34E-02	14.62	27.72	5.98E-01	6.00	45.02	8.94E-01	38.05	25.05	1.29E-01
cg01550799	-14.84	5.34	5.46E-03	-27.20	13.12	3.82E-02	-16.42	19.25	3.94E-01	-11.55	6.61	8.05E-02	-14.69	16.57	3.75E-01
cg11715092	15.59	5.61	5.46E-03	15.63	7.22	3.05E-02	21.84	13.13	9.63E-02	2.44	25.95	9.25E-01	12.29	13.70	3.70E-01
cg04657044	12.84	4.62	5.47E-03	16.21	6.43	1.17E-02	18.58	11.01	9.16E-02	20.31	15.11	1.79E-01	-1.31	9.40	8.89E-01
cg16312496	-12.75	4.59	5.47E-03	-12.13	12.07	3.15E-01	-8.22	16.37	6.16E-01	-11.53	5.55	3.77E-02	-26.64	15.11	7.79E-02
cg27021553	10.37	3.73	5.48E-03	6.28	4.91	2.01E-01	22.91	9.17	1.24E-02	7.59	13.09	5.62E-01	13.25	8.91	1.37E-01
cg26551975	20.06	7.22	5.48E-03	19.87	23.91	4.06E-01	23.63	64.63	7.15E-01	20.32	8.05	1.16E-02	17.46	23.89	4.65E-01
cg24722112	41.93	15.10	5.50E-03	50.11	38.79	1.96E-01	-153.88	156.25	3.25E-01	39.31	17.48	2.46E-02	69.32	49.56	1.62E-01
cg09618998	9.33	3.36	5.50E-03	7.97	4.22	5.89E-02	13.71	8.61	1.11E-01	10.95	13.55	4.19E-01	9.93	8.61	2.48E-01
cg08783300	27.90	10.05	5.50E-03	24.09	13.87	8.25E-02	37.72	29.59	2.02E-01	74.29	38.82	5.57E-02	20.23	18.56	2.76E-01
cg13992678	14.44	5.20	5.50E-03	13.52	8.13	9.65E-02	30.31	11.87	1.06E-02	13.22	10.11	1.91E-01	0.02	12.86	9.99E-01
cg24625894	23.12	8.33	5.51E-03	27.23	17.34	1.16E-01	29.40	16.76	7.95E-02	13.54	13.23	3.06E-01	33.44	23.47	1.54E-01
cg19899237	-38.82	13.99	5.51E-03	-12.09	40.91	7.68E-01	-241.93	191.92	2.07E-01	-40.74	15.65	9.22E-03	-45.32	49.84	3.63E-01
cg09743950	10.52	3.79	5.51E-03	11.28	7.00	1.07E-01	4.21	7.72	5.86E-01	12.72	6.77	6.01E-02	14.58	9.76	1.35E-01
cg05370838	18.09	6.52	5.51E-03	37.81	18.56	4.16E-02	5.95	23.54	8.01E-01	15.74	7.55	3.69E-02	22.63	28.06	4.20E-01
cg03939371	24.18	8.71	5.52E-03	27.84	14.51	5.50E-02	6.07	18.20	7.39E-01	31.59	16.31	5.28E-02	29.95	24.70	2.25E-01
cg03783925	17.92	6.46	5.52E-03	14.82	10.74	1.68E-01	18.01	14.44	2.12E-01	26.01	13.40	5.23E-02	14.17	14.21	3.19E-01
cg13759418	22.35	8.05	5.52E-03	27.02	14.08	5.49E-02	18.87	17.35	2.77E-01	12.13	14.32	3.97E-01	39.77	21.47	6.40E-02
cg19678564	42.04	15.15	5.52E-03	46.93	21.73	3.08E-02	0.74	35.50	9.83E-01	78.92	42.01	6.03E-02	43.76	33.75	1.95E-01
cg09302474	9.30	3.35	5.53E-03	7.92	4.39	7.12E-02	20.44	8.01	1.07E-02	0.49	9.96	9.61E-01	8.15	9.39	3.86E-01
cg02491234	19.24	6.94	5.53E-03	22.41	10.49	3.26E-02	25.84	13.43	5.43E-02	18.26	18.23	3.17E-01	-0.63	17.84	9.72E-01
cg02722672	8.19	2.95	5.53E-03	7.97	3.93	4.26E-02	14.01	6.33	2.69E-02	3.65	10.55	7.30E-01	2.59	7.88	7.42E-01
cg05089897	6.50	2.34	5.53E-03	5.40	3.68	1.42E-01	12.89	5.67	2.31E-02	5.98	4.03	1.38E-01	1.01	8.07	9.00E-01
cg02133456	-11.01	3.97	5.53E-03	-7.25	7.73	3.48E-01	-6.69	10.85	5.37E-01	-11.86	5.69	3.69E-02	-20.96	11.67	7.25E-02
cg25653204	21.84	7.87	5.54E-03	28.80	10.36	5.44E-03	-25.54	31.89	4.23E-01	28.97	37.19	4.36E-01	17.26	13.99	2.17E-01
cg06377222	20.76	7.48	5.54E-03	20.41	11.38	7.29E-02	22.45	16.37	1.70E-01	11.73	22.14	5.96E-01	24.15	15.14	1.11E-01
cg23121203	-21.99	7.93	5.54E-03	-16.64	18.92	3.79E-01	10.83	43.99	8.06E-01	-26.33	9.22	4.30E-03	0.86	34.62	9.80E-01
cg09731694	17.21	6.21	5.55E-03	18.28	10.83	9.14E-02	23.03	14.37	1.09E-01	25.02	11.96	3.64E-02	0.82	13.35	9.51E-01
cg05589784	6.73	2.43	5.55E-03	10.94	4.22	9.53E-03	7.69	4.57	9.29E-02	2.01	6.80	7.68E-01	2.66	4.75	5.75E-01
cg23818062	16.06	5.79	5.55E-03	25.14	15.67	1.09E-01	0.19	28.20	9.95E-01	14.82	6.63	2.55E-02	22.43	23.85	3.47E-01

cg11579222	-12.40	4.47	5.55E-03	-23.52	11.84	4.69E-02	3.57	14.64	8.08E-01	-12.22	5.38	2.32E-02	-12.82	16.47	4.36E-01
cg16742925	-37.26	13.44	5.55E-03	-65.89	36.99	7.49E-02	135.90	200.00	4.97E-01	-35.38	14.97	1.81E-02	-11.89	55.68	8.31E-01
cg18892473	42.35	15.28	5.56E-03	22.78	44.07	6.05E-01	81.13	207.86	6.96E-01	51.30	16.82	2.28E-03	-64.17	68.85	3.51E-01
cg16327883	12.69	4.58	5.56E-03	10.31	8.66	2.33E-01	2.95	28.96	9.19E-01	12.03	5.95	4.32E-02	25.04	14.15	7.69E-02
cg18316500	28.07	10.12	5.57E-03	42.21	14.52	3.64E-03	22.78	22.33	3.08E-01	-10.95	32.08	7.33E-01	18.91	22.18	3.94E-01
cg21794222	-10.71	3.86	5.57E-03	-11.59	5.01	2.07E-02	-16.51	11.02	1.34E-01	-9.85	12.66	4.37E-01	-4.59	8.90	6.06E-01
cg18574274	8.55	3.09	5.57E-03	7.79	7.58	3.04E-01	-1.73	10.94	8.74E-01	10.79	3.90	5.70E-03	5.05	8.59	5.56E-01
cg27126872	7.42	2.68	5.57E-03	5.85	3.94	1.37E-01	10.77	5.69	5.82E-02	-1.11	7.67	8.85E-01	12.72	6.09	3.68E-02
cg05452406	16.78	6.05	5.57E-03	27.87	16.03	8.22E-02	20.29	21.97	3.56E-01	13.00	8.51	1.27E-01	17.01	11.53	1.40E-01
cg01100912	19.01	6.86	5.58E-03	25.79	21.53	2.31E-01	25.11	30.22	4.06E-01	16.76	8.55	5.00E-02	21.21	15.20	1.63E-01
cg02930951	20.79	7.50	5.58E-03	17.93	12.45	1.50E-01	31.22	14.55	3.19E-02	17.41	15.98	2.76E-01	14.24	19.33	4.61E-01
cg24878005	30.44	10.98	5.58E-03	32.49	19.55	9.66E-02	10.04	24.27	6.79E-01	38.17	18.36	3.76E-02	36.71	31.47	2.43E-01
cg11702455	18.44	6.65	5.58E-03	29.54	15.18	5.16E-02	28.85	32.85	3.80E-01	12.13	8.01	1.30E-01	41.98	24.07	8.12E-02
cg23415916	-20.98	7.57	5.59E-03	-0.39	19.99	9.84E-01	-39.26	26.61	1.40E-01	-22.62	9.02	1.22E-02	-25.35	28.22	3.69E-01
cg06221470	20.13	7.27	5.60E-03	15.92	11.81	1.78E-01	-21.52	47.18	6.48E-01	24.08	10.12	1.74E-02	26.71	25.24	2.90E-01
cg24866764	-9.33	3.37	5.60E-03	-7.63	7.17	2.87E-01	-1.35	10.53	8.98E-01	-9.49	4.47	3.37E-02	-19.51	10.23	5.65E-02
cg09692822	24.02	8.67	5.61E-03	43.14	17.65	1.45E-02	25.17	16.58	1.29E-01	8.70	14.51	5.49E-01	28.23	24.21	2.43E-01
cg10092198	-12.57	4.54	5.61E-03	-17.05	7.58	2.45E-02	-7.96	10.62	4.54E-01	-7.54	8.67	3.85E-01	-15.94	10.57	1.31E-01
cg24497686	-16.00	5.78	5.61E-03	-20.88	14.90	1.61E-01	7.18	35.50	8.40E-01	-14.27	6.87	3.79E-02	-25.38	16.88	1.33E-01
cg17288560	9.21	3.33	5.62E-03	9.49	4.52	3.57E-02	10.77	7.10	1.30E-01	4.21	12.73	7.41E-01	8.32	8.06	3.02E-01
cg22750001	-11.49	4.15	5.63E-03	-12.32	9.25	1.83E-01	-25.50	18.20	1.61E-01	-9.36	5.21	7.25E-02	-15.51	12.36	2.09E-01
cg08799778	18.20	6.57	5.63E-03	14.02	16.41	3.93E-01	16.73	20.03	4.03E-01	19.52	8.04	1.52E-02	17.33	26.05	5.06E-01
cg08135278	-36.72	13.26	5.63E-03	-18.16	42.78	6.71E-01	-140.18	136.13	3.03E-01	-38.64	14.37	7.18E-03	-17.33	64.02	7.87E-01
cg17289597	14.01	5.06	5.63E-03	8.49	7.01	2.26E-01	28.00	9.99	5.08E-03	19.90	15.42	1.97E-01	6.41	11.35	5.72E-01
cg07142797	25.92	9.36	5.63E-03	12.64	20.84	5.44E-01	5.92	36.37	8.71E-01	33.12	12.13	6.35E-03	23.89	25.34	3.46E-01
cg10086141	-43.28	15.63	5.63E-03	-42.07	19.60	3.18E-02	-59.35	51.25	2.47E-01	-76.34	68.88	2.68E-01	-32.20	33.41	3.35E-01
cg02861178	-25.82	9.33	5.64E-03	-27.02	13.04	3.83E-02	-43.99	27.62	1.11E-01	7.76	36.41	8.31E-01	-24.24	16.77	1.48E-01
cg16701559	-20.83	7.52	5.64E-03	-29.00	13.95	3.75E-02	3.87	24.97	8.77E-01	-23.93	10.50	2.27E-02	-4.33	23.24	8.52E-01
cg21541120	30.58	11.05	5.65E-03	8.22	28.51	7.73E-01	-109.61	118.24	3.54E-01	37.70	12.73	3.06E-03	21.72	37.37	5.61E-01
cg18067096	-14.58	5.27	5.65E-03	-20.21	12.77	1.14E-01	-18.62	18.18	3.06E-01	-12.78	6.60	5.28E-02	-13.19	15.98	4.09E-01
cg14060020	17.09	6.17	5.65E-03	26.25	9.13	4.05E-03	10.04	11.30	3.74E-01	9.65	17.53	5.82E-01	7.40	17.81	6.78E-01
cg19056833	30.39	10.98	5.65E-03	18.48	20.55	3.69E-01	-31.29	54.94	5.69E-01	42.97	14.80	3.68E-03	21.81	31.25	4.85E-01
cg27175024	-7.09	2.56	5.65E-03	-7.31	3.86	5.86E-02	-4.32	5.58	4.38E-01	-5.16	8.16	5.27E-01	-9.80	5.12	5.55E-02
cg04436383	-12.46	4.50	5.66E-03	-21.47	9.44	2.30E-02	-4.99	15.00	7.39E-01	-11.46	6.21	6.49E-02	-7.03	11.41	5.38E-01
cg20750319	-10.14	3.66	5.66E-03	-11.65	5.55	3.58E-02	-16.28	8.96	6.93E-02	-4.83	8.04	5.48E-01	-7.06	8.42	4.01E-01

cg13723693	40.10	14.49	5.66E-03	37.27	40.54	3.58E-01	-143.41	198.81	4.71E-01	42.41	16.18	8.76E-03	32.14	57.22	5.74E-01
cg07754138	36.44	13.17	5.66E-03	5.31	34.52	8.78E-01	-100.08	162.93	5.39E-01	39.21	14.82	8.16E-03	91.60	54.40	9.22E-02
cg25197500	5.20	1.88	5.66E-03	5.69	2.54	2.53E-02	5.43	4.45	2.22E-01	5.91	4.22	1.61E-01	-0.67	6.83	9.22E-01
cg11841394	25.31	9.15	5.66E-03	20.72	12.82	1.06E-01	9.46	26.55	7.22E-01	35.68	30.06	2.35E-01	36.93	17.29	3.27E-02
cg14204266	-35.51	12.84	5.67E-03	-41.94	20.70	4.27E-02	-3.86	61.20	9.50E-01	-17.39	24.24	4.73E-01	-49.28	23.81	3.84E-02
cg17427615	18.47	6.68	5.67E-03	20.75	9.86	3.54E-02	17.66	12.91	1.71E-01	39.79	21.04	5.86E-02	1.31	16.04	9.35E-01
cg03128060	7.65	2.76	5.67E-03	3.50	2.81	2.12E-01	10.95	4.59	1.69E-02	18.15	7.83	2.05E-02	6.98	5.35	1.92E-01
cg04120329	-9.03	3.26	5.67E-03	-5.75	4.67	2.17E-01	-9.38	7.00	1.80E-01	-14.96	11.53	1.94E-01	-13.97	7.07	4.83E-02
cg23585979	37.31	13.49	5.67E-03	43.78	20.12	2.95E-02	-1.86	34.10	9.57E-01	89.28	42.23	3.45E-02	30.37	24.64	2.18E-01
cg25669309	-32.13	11.62	5.67E-03	-21.02	38.84	5.88E-01	-150.35	117.90	2.02E-01	-35.19	12.70	5.60E-03	9.66	45.59	8.32E-01
cg17629322	-14.05	5.08	5.67E-03	-26.67	13.06	4.11E-02	-5.86	22.20	7.92E-01	-14.57	6.10	1.69E-02	3.86	15.84	8.07E-01
cg19856845	13.72	4.96	5.68E-03	23.25	8.09	4.03E-03	13.15	13.33	3.24E-01	9.75	9.58	3.08E-01	2.44	10.66	8.19E-01
cg03725447	19.62	7.09	5.68E-03	16.38	16.57	3.23E-01	7.03	36.38	8.47E-01	22.80	8.69	8.67E-03	10.22	21.22	6.30E-01
cg21191176	-21.95	7.94	5.69E-03	-19.66	18.01	2.75E-01	-37.31	41.43	3.68E-01	-17.65	9.62	6.66E-02	-53.63	26.67	4.43E-02
cg14936968	25.30	9.15	5.69E-03	46.37	24.45	5.79E-02	17.54	45.31	6.99E-01	24.68	11.59	3.32E-02	13.80	20.66	5.04E-01
cg27363829	11.77	4.26	5.69E-03	21.55	9.95	3.03E-02	-0.45	12.85	9.72E-01	14.36	6.39	2.46E-02	5.71	8.30	4.91E-01
cg07165459	-41.49	15.01	5.70E-03	-40.48	21.17	5.59E-02	-64.67	49.20	1.89E-01	-40.66	51.38	4.29E-01	-36.54	26.57	1.69E-01
cg18269183	27.23	9.85	5.71E-03	39.39	13.86	4.48E-03	30.49	39.68	4.42E-01	39.66	30.40	1.92E-01	3.91	17.20	8.20E-01
cg22633936	-21.03	7.61	5.71E-03	-44.44	19.46	2.24E-02	-21.20	40.27	5.99E-01	-15.80	8.87	7.49E-02	-24.54	27.64	3.75E-01
cg24554151	6.79	2.45	5.71E-03	8.16	4.31	5.81E-02	10.67	5.17	3.90E-02	2.63	4.39	5.50E-01	6.60	6.61	3.18E-01
cg02532096	16.43	5.95	5.72E-03	26.70	10.63	1.20E-02	-3.30	16.21	8.39E-01	16.13	11.15	1.48E-01	14.68	11.48	2.01E-01
cg16643706	-10.88	3.94	5.72E-03	-22.69	9.76	2.01E-02	4.60	20.61	8.23E-01	-9.22	4.62	4.58E-02	-8.76	14.43	5.44E-01
cg01583131	19.61	7.10	5.72E-03	30.32	10.62	4.29E-03	14.04	15.63	3.69E-01	27.66	12.64	2.87E-02	0.22	13.17	9.87E-01
cg05059438	-22.73	8.22	5.73E-03	-33.21	13.98	1.75E-02	-11.77	15.20	4.39E-01	-19.95	18.40	2.78E-01	-23.58	20.48	2.50E-01
cg06315149	18.06	6.54	5.73E-03	2.79	16.61	8.67E-01	12.91	21.99	5.57E-01	20.79	8.07	9.96E-03	28.38	20.69	1.70E-01
cg19052164	-12.48	4.52	5.73E-03	-21.24	7.25	3.41E-03	-2.59	10.19	8.00E-01	-6.33	10.06	5.29E-01	-11.49	9.77	2.40E-01
cg14866032	-5.50	1.99	5.73E-03	-5.24	2.97	7.79E-02	-5.94	3.77	1.15E-01	-3.51	7.60	6.44E-01	-6.11	4.39	1.64E-01
cg18795786	-12.10	4.38	5.74E-03	-23.24	7.61	2.25E-03	-17.63	11.62	1.29E-01	-7.18	4.31	9.56E-02	-5.07	10.73	6.37E-01
cg25093234	-55.51	20.10	5.74E-03	-44.03	27.05	1.04E-01	-112.30	92.42	2.24E-01	-49.80	40.86	2.23E-01	-87.24	50.42	8.36E-02
cg23277061	20.71	7.50	5.74E-03	19.00	11.75	1.06E-01	22.37	14.08	1.12E-01	30.46	19.67	1.22E-01	13.44	18.53	4.68E-01
cg14101604	16.38	5.93	5.74E-03	4.15	15.45	7.88E-01	-22.91	54.64	6.75E-01	20.71	6.73	2.08E-03	-0.77	23.46	9.74E-01
cg13906860	-6.34	2.29	5.74E-03	-8.42	3.37	1.25E-02	-9.41	5.40	8.12E-02	-0.87	6.25	8.89E-01	-2.79	4.88	5.68E-01
cg19296354	15.36	5.56	5.75E-03	25.05	8.87	4.75E-03	11.17	10.51	2.88E-01	8.60	12.65	4.97E-01	5.43	15.20	7.21E-01
cg25983305	-42.26	15.30	5.75E-03	-16.02	39.19	6.83E-01	32.16	224.15	8.86E-01	-47.26	17.70	7.60E-03	-48.66	49.40	3.25E-01
cg11016847	16.66	6.03	5.75E-03	16.91	10.25	9.88E-02	19.72	21.32	3.55E-01	20.33	8.65	1.87E-02	-7.63	20.41	7.08E-01

cg14675869	-16.26	5.89	5.75E-03	-12.76	11.90	2.83E-01	-32.10	29.36	2.74E-01	-15.06	7.67	4.96E-02	-23.61	16.60	1.55E-01
cg24948990	40.43	14.64	5.76E-03	49.09	45.36	2.79E-01	46.47	52.91	3.80E-01	42.51	17.22	1.36E-02	10.73	47.08	8.20E-01
cg05967403	14.55	5.27	5.76E-03	21.48	8.02	7.42E-03	10.35	13.34	4.38E-01	5.76	13.98	6.80E-01	10.53	10.12	2.98E-01
cg02882448	26.99	9.77	5.76E-03	44.32	34.68	2.01E-01	-0.13	147.68	9.99E-01	27.49	10.57	9.32E-03	-0.50	39.40	9.90E-01
cg07089235	-21.37	7.74	5.77E-03	5.62	29.37	8.48E-01	25.80	92.39	7.80E-01	-22.87	8.31	5.95E-03	-37.39	32.53	2.50E-01
cg16874442	32.93	11.93	5.77E-03	49.53	16.54	2.76E-03	41.69	31.83	1.90E-01	41.28	25.21	1.02E-01	2.73	19.36	8.88E-01
cg02137984	-14.83	5.37	5.77E-03	-23.56	16.72	1.59E-01	-26.52	34.64	4.44E-01	-11.64	6.15	5.85E-02	-25.98	16.09	1.06E-01
cg16910293	-21.32	7.72	5.77E-03	-19.64	21.14	3.53E-01	16.91	43.73	6.99E-01	-25.44	8.89	4.24E-03	-0.52	27.15	9.85E-01
cg09273112	5.55	2.01	5.78E-03	3.76	2.67	1.60E-01	11.66	4.35	7.38E-03	6.11	3.91	1.18E-01	-0.76	7.06	9.14E-01
cg06085579	-10.78	3.90	5.78E-03	-12.84	5.55	2.07E-02	-10.91	7.56	1.49E-01	-10.18	12.43	4.13E-01	-3.61	10.46	7.30E-01
cg12768770	-29.72	10.77	5.78E-03	-26.20	12.17	3.14E-02	28.02	41.56	5.00E-01	-20.61	54.90	7.07E-01	-49.77	18.07	5.87E-03
cg07875121	13.06	4.73	5.79E-03	12.98	6.54	4.73E-02	18.52	11.52	1.08E-01	15.23	12.13	2.09E-01	5.29	12.01	6.60E-01
cg11312408	15.99	5.79	5.79E-03	19.86	9.24	3.17E-02	18.40	14.98	2.19E-01	9.81	12.12	4.19E-01	13.93	12.11	2.50E-01
cg00538604	-68.41	24.79	5.79E-03	-55.47	33.31	9.59E-02	38.75	139.60	7.81E-01	-120.37	48.48	1.30E-02	-48.54	63.35	4.44E-01
cg10358816	36.25	13.14	5.79E-03	21.63	39.65	5.85E-01	-17.60	124.20	8.87E-01	39.19	14.51	6.90E-03	32.79	54.12	5.45E-01
cg05502283	24.53	8.89	5.79E-03	37.39	19.08	5.00E-02	21.09	16.02	1.88E-01	21.47	15.17	1.57E-01	19.34	24.52	4.30E-01
cg16729495	47.60	17.25	5.79E-03	-14.33	39.13	7.14E-01	50.23	115.99	6.65E-01	61.91	18.61	8.79E-04	61.40	50.59	2.25E-01
cg03163184	-10.59	3.84	5.79E-03	-15.59	5.67	6.01E-03	-8.78	9.59	3.60E-01	4.21	11.74	7.20E-01	-9.08	7.31	2.14E-01
cg25007511	-12.10	4.39	5.79E-03	-22.29	7.92	4.90E-03	-20.74	19.60	2.90E-01	-6.12	5.00	2.20E-01	-14.67	13.26	2.69E-01
cg24045357	-10.26	3.72	5.79E-03	-9.83	5.08	5.29E-02	-16.13	8.18	4.85E-02	9.23	13.42	4.92E-01	-13.10	8.76	1.35E-01
cg24137216	41.92	15.20	5.80E-03	40.59	20.86	5.16E-02	11.55	53.64	8.30E-01	3.09	43.71	9.44E-01	71.17	29.35	1.53E-02
cg13659951	26.20	9.50	5.80E-03	18.56	27.90	5.06E-01	-22.80	65.88	7.29E-01	27.63	10.70	9.83E-03	36.47	34.50	2.90E-01
cg17473398	38.69	14.03	5.81E-03	74.68	21.64	5.58E-04	21.06	24.20	3.84E-01	18.57	18.25	3.09E-01	44.60	29.32	1.28E-01
cg18547371	-54.25	19.67	5.82E-03	-120.77	69.70	8.31E-02	-159.64	251.47	5.26E-01	-44.42	21.46	3.85E-02	-85.70	72.42	2.37E-01
cg24196566	-14.40	5.22	5.82E-03	-21.31	12.84	9.71E-02	-2.99	19.72	8.79E-01	-15.00	6.33	1.78E-02	-5.52	18.00	7.59E-01
cg00879667	23.24	8.43	5.82E-03	22.41	13.74	1.03E-01	34.96	15.87	2.76E-02	15.26	17.57	3.85E-01	12.88	25.25	6.10E-01
cg03152720	19.38	7.03	5.82E-03	39.24	20.33	5.36E-02	33.62	30.11	2.64E-01	13.96	8.05	8.28E-02	35.15	28.05	2.10E-01
cg13729043	11.73	4.25	5.83E-03	9.78	9.03	2.79E-01	18.07	20.39	3.75E-01	9.51	5.40	7.83E-02	25.12	12.57	4.57E-02
cg19290902	-10.54	3.82	5.84E-03	-15.85	7.23	2.84E-02	-3.68	11.47	7.49E-01	-9.81	5.59	7.92E-02	-7.85	10.15	4.39E-01
cg20098710	15.65	5.68	5.84E-03	9.79	9.26	2.90E-01	24.03	11.36	3.44E-02	8.01	13.97	5.66E-01	22.20	12.41	7.35E-02
cg08430516	32.24	11.69	5.84E-03	3.17	30.29	9.17E-01	-44.74	108.36	6.80E-01	36.52	13.45	6.61E-03	56.23	40.58	1.66E-01
cg07915528	-56.11	20.36	5.85E-03	-54.71	23.89	2.20E-02	-129.22	42.75	2.51E-03	-15.81	21.29	4.58E-01	-64.56	30.84	3.63E-02
cg19110758	-16.28	5.91	5.85E-03	-20.67	8.80	1.88E-02	-20.67	13.89	1.37E-01	-9.78	17.44	5.75E-01	-8.28	11.73	4.80E-01
cg17557230	24.57	8.92	5.87E-03	14.92	22.19	5.01E-01	50.07	68.14	4.62E-01	29.85	10.43	4.22E-03	-5.72	29.71	8.47E-01
cg14862385	-21.36	7.75	5.87E-03	-13.71	22.80	5.48E-01	-65.14	57.57	2.58E-01	-22.80	8.59	7.92E-03	-0.10	34.30	9.98E-01

cg14412794	-18.18	6.60	5.87E-03	-22.69	11.48	4.81E-02	-32.02	22.76	1.60E-01	-14.56	10.71	1.74E-01	-11.97	14.53	4.10E-01
cg13137476	21.20	7.70	5.87E-03	12.04	19.97	5.47E-01	-24.96	77.61	7.48E-01	25.52	8.78	3.65E-03	0.72	28.38	9.80E-01
cg19323865	-21.43	7.78	5.87E-03	3.00	20.53	8.84E-01	-18.98	30.75	5.37E-01	-25.51	9.04	4.78E-03	-34.02	34.28	3.21E-01
cg14855367	-10.91	3.96	5.88E-03	-14.33	8.54	9.34E-02	-11.79	9.95	2.36E-01	-10.32	5.69	6.95E-02	-6.74	10.51	5.21E-01
cg23124486	20.53	7.45	5.88E-03	16.61	16.64	3.18E-01	-33.21	37.57	3.77E-01	22.73	9.07	1.22E-02	37.16	25.55	1.46E-01
cg08231348	20.68	7.51	5.89E-03	33.62	33.22	3.11E-01	76.52	101.19	4.50E-01	21.74	8.24	8.38E-03	4.50	22.21	8.40E-01
cg14763104	-13.01	4.72	5.89E-03	-19.37	6.83	4.58E-03	-14.80	10.51	1.59E-01	-5.93	12.10	6.24E-01	0.87	11.54	9.40E-01
cg17369513	-19.95	7.24	5.89E-03	-36.23	17.57	3.92E-02	-12.60	29.94	6.74E-01	-17.51	8.86	4.83E-02	-13.12	22.49	5.60E-01
cg02481934	-15.31	5.56	5.89E-03	-22.48	9.55	1.85E-02	-19.27	14.12	1.72E-01	-14.17	10.21	1.65E-01	-2.39	12.14	8.44E-01
cg09105479	38.63	14.03	5.90E-03	-5.68	58.01	9.22E-01	-89.15	246.90	7.18E-01	43.90	14.81	3.04E-03	-3.06	69.02	9.65E-01
cg16442712	-13.81	5.02	5.90E-03	-16.14	15.15	2.87E-01	-16.24	22.76	4.75E-01	-12.24	5.72	3.24E-02	-25.38	18.65	1.74E-01
cg00363811	-21.69	7.88	5.90E-03	-16.82	26.93	5.32E-01	-66.99	89.75	4.55E-01	-20.84	8.52	1.44E-02	-37.19	34.76	2.85E-01
cg15516314	-14.91	5.42	5.91E-03	-18.77	14.71	2.02E-01	16.61	20.62	4.21E-01	-15.61	6.40	1.46E-02	-29.49	19.32	1.27E-01
cg09351715	53.88	19.57	5.91E-03	71.72	27.74	9.73E-03	19.15	53.45	7.20E-01	9.29	56.54	8.70E-01	58.39	39.30	1.37E-01
cg26019112	19.08	6.93	5.92E-03	17.90	12.82	1.63E-01	-0.43	20.26	9.83E-01	20.79	10.20	4.14E-02	33.35	19.34	8.45E-02
cg23963071	-9.61	3.49	5.92E-03	-9.34	4.58	4.16E-02	-18.93	8.05	1.87E-02	-4.76	12.34	7.00E-01	-1.64	8.97	8.55E-01
cg00295206	37.16	13.50	5.92E-03	25.24	17.29	1.44E-01	27.64	43.20	5.22E-01	66.89	66.73	3.16E-01	64.87	26.91	1.59E-02
cg00241663	23.37	8.49	5.92E-03	24.61	11.82	3.73E-02	7.91	22.25	7.22E-01	34.32	29.47	2.44E-01	26.12	16.81	1.20E-01
cg14394327	-20.01	7.27	5.92E-03	-39.21	20.82	5.96E-02	6.44	48.86	8.95E-01	-18.10	8.13	2.59E-02	-15.86	31.03	6.09E-01
cg04546061	21.28	7.73	5.92E-03	18.25	15.08	2.26E-01	2.80	31.73	9.30E-01	28.46	10.39	6.15E-03	4.42	21.98	8.41E-01
cg16121685	-40.61	14.76	5.93E-03	-46.19	27.84	9.71E-02	-23.18	58.40	6.91E-01	-45.21	21.52	3.57E-02	-26.45	34.32	4.41E-01
cg16231923	-10.29	3.74	5.93E-03	-5.70	4.83	2.38E-01	-6.13	6.52	3.47E-01	-11.77	5.87	4.48E-02	-25.92	9.14	4.57E-03
cg19388016	39.45	14.34	5.94E-03	40.90	26.79	1.27E-01	31.29	133.67	8.15E-01	36.51	18.32	4.63E-02	56.04	47.97	2.43E-01
cg13552867	21.46	7.80	5.94E-03	20.58	15.63	1.88E-01	0.53	38.71	9.89E-01	25.35	10.08	1.19E-02	10.19	23.35	6.62E-01
cg04611649	-16.37	5.95	5.94E-03	-9.06	12.41	4.65E-01	-49.63	22.10	2.48E-02	-13.42	6.28	3.26E-02	-30.43	21.05	1.48E-01
cg05648303	17.22	6.26	5.94E-03	23.24	7.24	1.32E-03	-1.89	14.83	8.99E-01	31.53	15.75	4.54E-02	8.81	12.04	4.64E-01
cg12977625	28.30	10.29	5.95E-03	35.34	29.60	2.33E-01	-14.75	71.40	8.36E-01	26.22	11.68	2.48E-02	48.48	35.89	1.77E-01
cg21297992	37.54	13.65	5.95E-03	31.71	16.19	5.02E-02	24.42	18.41	1.85E-01	16.69	19.39	3.89E-01	83.50	21.43	9.80E-05
cg02828023	18.45	6.71	5.95E-03	38.41	15.76	1.48E-02	18.44	20.24	3.62E-01	15.88	9.86	1.07E-01	8.59	13.52	5.25E-01
cg26998274	18.85	6.85	5.95E-03	35.11	16.76	3.62E-02	41.33	40.98	3.13E-01	17.12	8.20	3.69E-02	-1.23	20.99	9.53E-01
cg24650915	22.07	8.02	5.95E-03	30.96	19.37	1.10E-01	-2.31	25.01	9.26E-01	18.69	9.21	4.23E-02	58.62	26.99	2.98E-02
cg15865243	-7.61	2.77	5.96E-03	-9.43	3.93	1.63E-02	-5.63	5.89	3.39E-01	5.22	9.63	5.88E-01	-10.55	6.18	8.76E-02
cg08502490	-57.67	20.97	5.96E-03	-53.01	25.40	3.69E-02	-185.31	142.48	1.93E-01	-27.83	104.09	7.89E-01	-64.03	41.45	1.22E-01
cg16288089	15.00	5.45	5.96E-03	25.58	16.87	1.29E-01	10.12	29.39	7.30E-01	17.37	6.63	8.79E-03	1.20	12.70	9.25E-01
cg21144338	18.33	6.67	5.96E-03	35.88	19.52	6.61E-02	-8.29	23.57	7.25E-01	17.34	7.74	2.51E-02	31.64	26.85	2.39E-01

cg10133935	6.84	2.49	5.96E-03	5.70	3.04	6.10E-02	16.06	6.70	1.65E-02	3.36	7.96	6.73E-01	5.09	8.06	5.27E-01
cg13761284	26.80	9.75	5.96E-03	52.04	19.04	6.28E-03	6.49	21.40	7.62E-01	18.31	15.59	2.40E-01	33.08	25.53	1.95E-01
cg02360514	11.67	4.25	5.96E-03	12.91	6.99	6.49E-02	19.78	8.71	2.32E-02	4.09	10.04	6.84E-01	6.92	9.14	4.49E-01
cg15010390	-28.62	10.41	5.97E-03	-25.31	26.42	3.38E-01	-42.80	44.68	3.38E-01	-27.69	28.58	3.33E-01	-28.42	12.83	2.68E-02
cg12030690	29.00	10.55	5.97E-03	44.75	17.11	8.91E-03	22.67	33.06	4.93E-01	35.17	21.11	9.57E-02	3.35	20.37	8.69E-01
cg14026106	-10.15	3.69	5.97E-03	-16.81	8.22	4.07E-02	-3.95	10.61	7.09E-01	-10.00	4.93	4.27E-02	-5.79	10.78	5.91E-01
cg06521562	15.88	5.78	5.98E-03	22.19	8.51	9.11E-03	16.39	10.94	1.34E-01	18.39	18.53	3.21E-01	-4.34	14.32	7.62E-01
cg02857074	-9.01	3.28	5.98E-03	-14.16	6.12	2.06E-02	-2.98	10.17	7.70E-01	-6.61	4.75	1.64E-01	-11.28	9.05	2.13E-01
cg15083678	14.52	5.28	5.98E-03	23.21	16.21	1.52E-01	41.78	20.71	4.37E-02	10.41	5.98	8.16E-02	25.49	24.27	2.94E-01
cg03314100	-23.91	8.70	5.98E-03	-10.66	16.66	5.22E-01	-14.48	34.19	6.72E-01	-31.37	11.65	7.06E-03	-24.43	26.82	3.62E-01
cg16732469	41.20	14.99	5.98E-03	22.73	42.26	5.91E-01	-20.74	150.48	8.90E-01	42.53	16.75	1.11E-02	70.71	59.58	2.35E-01
cg10680411	21.78	7.92	5.98E-03	14.09	9.72	1.47E-01	46.90	26.20	7.34E-02	71.69	39.70	7.10E-02	22.70	14.35	1.14E-01
cg16676676	-74.07	26.95	5.98E-03	-80.79	40.61	4.67E-02	-160.86	154.12	2.97E-01	-62.62	47.44	1.87E-01	-64.79	59.30	2.75E-01
cg20865806	32.25	11.73	5.99E-03	26.63	27.19	3.27E-01	126.05	118.16	2.86E-01	28.62	13.86	3.89E-02	63.64	39.80	1.10E-01
cg04217515	7.79	2.83	5.99E-03	6.51	3.68	7.75E-02	12.43	5.80	3.22E-02	11.36	12.15	3.50E-01	3.09	8.36	7.12E-01
cg25842633	14.14	5.15	5.99E-03	10.32	8.30	2.14E-01	14.87	24.28	5.40E-01	21.10	6.02	4.61E-04	-5.29	14.06	7.07E-01
cg20689661	25.82	9.40	6.00E-03	13.88	16.57	4.02E-01	24.43	39.03	5.31E-01	34.73	13.39	9.48E-03	22.18	26.29	3.99E-01
cg22578425	-12.14	4.42	6.00E-03	-6.03	9.93	5.44E-01	-2.60	14.70	8.60E-01	-14.87	5.64	8.42E-03	-16.14	14.03	2.50E-01
cg12805764	51.42	18.72	6.01E-03	33.43	38.04	3.79E-01	-82.85	163.08	6.11E-01	66.70	23.74	4.96E-03	24.04	53.33	6.52E-01
cg20227886	30.87	11.24	6.02E-03	33.74	21.87	1.23E-01	-32.53	59.14	5.82E-01	34.01	15.11	2.44E-02	29.51	29.36	3.15E-01
cg00405190	6.62	2.41	6.02E-03	7.09	3.12	2.30E-02	9.03	5.77	1.17E-01	0.48	7.48	9.48E-01	6.12	6.88	3.73E-01
cg00921219	-11.90	4.33	6.03E-03	-13.82	7.89	7.99E-02	5.26	20.26	7.95E-01	-13.18	6.03	2.87E-02	-8.53	11.74	4.67E-01
cg25995212	-16.10	5.86	6.03E-03	-4.76	17.32	7.83E-01	-35.92	23.30	1.23E-01	-14.09	6.68	3.48E-02	-47.20	25.89	6.84E-02
cg05121093	18.11	6.60	6.03E-03	31.62	16.05	4.89E-02	27.32	42.09	5.16E-01	17.87	7.82	2.22E-02	-6.57	21.44	7.59E-01
cg09175843	26.20	9.54	6.04E-03	21.14	24.41	3.86E-01	-2.63	85.59	9.75E-01	29.69	10.80	5.97E-03	-3.41	41.13	9.34E-01
cg02485642	-20.36	7.42	6.04E-03	-11.90	11.11	2.84E-01	-9.31	37.22	8.02E-01	-38.42	14.30	7.23E-03	-17.72	14.94	2.35E-01
cg11593111	27.97	10.19	6.04E-03	29.74	27.44	2.78E-01	98.72	135.44	4.66E-01	30.64	11.54	7.95E-03	-7.10	36.55	8.46E-01
cg13353942	-12.91	4.70	6.04E-03	-10.22	7.29	1.61E-01	-16.18	9.82	9.94E-02	-13.98	12.26	2.54E-01	-13.94	10.33	1.77E-01
cg15347348	-21.58	7.86	6.05E-03	-31.99	12.18	8.66E-03	-33.64	15.21	2.70E-02	-5.71	18.30	7.55E-01	-4.29	15.76	7.86E-01
cg26279253	18.28	6.66	6.05E-03	24.65	14.66	9.26E-02	5.67	24.95	8.20E-01	19.62	8.29	1.79E-02	1.57	24.01	9.48E-01
cg20519373	-9.40	3.42	6.06E-03	-12.98	5.00	9.39E-03	-6.15	6.98	3.78E-01	-17.47	13.66	2.01E-01	-3.20	7.19	6.57E-01
cg03285219	18.74	6.83	6.07E-03	20.32	14.48	1.60E-01	-14.22	36.66	6.98E-01	15.12	7.90	5.57E-02	48.95	20.93	1.93E-02
cg05590982	-14.64	5.34	6.08E-03	-14.41	14.34	3.15E-01	-3.60	18.18	8.43E-01	-13.20	6.52	4.29E-02	-32.99	16.39	4.42E-02
cg00550498	19.24	7.01	6.08E-03	38.11	17.19	2.66E-02	-12.59	38.22	7.42E-01	14.20	8.37	8.97E-02	34.31	22.45	1.26E-01
cg02330121	27.40	9.99	6.09E-03	15.66	21.48	4.66E-01	7.28	31.32	8.16E-01	31.00	13.22	1.90E-02	50.08	29.92	9.41E-02

cg17436506	35.52	12.95	6.09E-03	21.94	19.20	2.53E-01	67.51	29.56	2.24E-02	38.75	27.90	1.65E-01	30.74	34.86	3.78E-01
cg00269033	-28.22	10.29	6.09E-03	-23.19	21.08	2.71E-01	-30.93	47.67	5.16E-01	-34.00	13.36	1.09E-02	-9.00	29.39	7.59E-01
cg26442107	-89.27	32.55	6.10E-03	-90.66	52.95	8.69E-02	-132.43	225.07	5.56E-01	-61.73	53.01	2.44E-01	-129.17	68.73	6.02E-02
cg22014112	-10.95	3.99	6.11E-03	-14.94	5.41	5.77E-03	-18.28	14.43	2.05E-01	-15.92	14.61	2.76E-01	-0.75	7.23	9.17E-01
cg00395579	-21.82	7.96	6.11E-03	-18.69	19.39	3.35E-01	-69.86	34.01	3.99E-02	-22.70	7.00	1.18E-03	7.56	25.56	7.67E-01
cg16973406	16.17	5.90	6.12E-03	18.88	10.07	6.09E-02	23.46	12.85	6.79E-02	24.02	12.90	6.26E-02	-1.13	12.10	9.26E-01
cg12716367	-13.92	5.08	6.12E-03	-21.35	7.73	5.71E-03	-6.47	13.78	6.38E-01	-11.96	14.82	4.20E-01	-7.66	9.05	3.97E-01
cg24874254	-8.19	2.99	6.12E-03	-8.01	3.84	3.71E-02	-13.88	5.96	1.99E-02	8.25	10.63	4.38E-01	-8.42	6.23	1.76E-01
cg15209604	-16.71	6.10	6.13E-03	-23.76	9.05	8.70E-03	-10.05	13.54	4.58E-01	-3.04	17.06	8.59E-01	-16.27	13.12	2.15E-01
cg04445871	16.14	5.89	6.13E-03	25.90	9.97	9.41E-03	7.85	16.43	6.33E-01	9.18	11.53	4.26E-01	14.15	11.50	2.19E-01
cg13468400	25.16	9.18	6.13E-03	16.44	20.38	4.20E-01	39.60	19.88	4.64E-02	21.21	13.07	1.05E-01	32.23	30.53	2.91E-01
cg24102622	39.36	14.36	6.14E-03	49.81	20.56	1.54E-02	18.22	47.23	7.00E-01	40.42	41.17	3.26E-01	28.36	26.32	2.81E-01
cg19302831	53.75	19.62	6.14E-03	76.99	26.46	3.62E-03	7.84	63.27	9.01E-01	78.42	34.57	2.33E-02	5.03	36.59	8.91E-01
cg12072529	24.50	8.94	6.15E-03	22.07	17.39	2.04E-01	37.42	23.11	1.05E-01	15.63	12.68	2.18E-01	59.47	29.97	4.73E-02
cg06098215	7.83	2.86	6.15E-03	8.70	3.92	2.63E-02	6.46	6.80	3.42E-01	15.43	8.20	5.98E-02	1.08	6.93	8.76E-01
cg13480465	-12.83	4.68	6.16E-03	-20.82	7.70	6.84E-03	3.51	10.99	7.49E-01	-15.14	6.94	2.91E-02	-9.41	10.78	3.83E-01
cg11284411	6.80	2.48	6.16E-03	6.52	3.65	7.44E-02	5.16	7.76	5.06E-01	7.88	5.51	1.53E-01	7.17	5.16	1.65E-01
cg15110127	-25.89	9.45	6.16E-03	-31.31	10.19	2.13E-03	59.98	75.66	4.28E-01	28.97	61.70	6.39E-01	-5.85	29.72	8.44E-01
cg04352288	-13.73	5.01	6.16E-03	-14.02	7.09	4.79E-02	-12.13	15.83	4.44E-01	-17.63	12.66	1.64E-01	-11.26	10.17	2.68E-01
cg19316579	-6.64	2.43	6.17E-03	-12.01	4.13	3.67E-03	-5.31	5.80	3.60E-01	-2.90	4.29	5.00E-01	-4.05	6.03	5.01E-01
cg25092206	-52.20	19.06	6.17E-03	-84.26	44.16	5.64E-02	-171.13	257.95	5.07E-01	-38.92	22.44	8.29E-02	-86.34	64.71	1.82E-01
cg03334076	18.77	6.85	6.18E-03	28.25	16.01	7.76E-02	13.33	22.86	5.60E-01	18.49	8.54	3.04E-02	5.85	23.80	8.06E-01
cg07092157	-14.61	5.33	6.18E-03	-10.88	12.99	4.02E-01	13.93	22.51	5.36E-01	-18.48	6.45	4.16E-03	-9.98	17.72	5.73E-01
cg24234141	18.49	6.75	6.18E-03	10.01	13.98	4.74E-01	16.12	33.34	6.29E-01	22.28	8.31	7.36E-03	12.03	26.31	6.48E-01
cg11235498	17.33	6.33	6.18E-03	22.85	13.65	9.42E-02	5.00	17.24	7.72E-01	17.10	11.45	1.35E-01	18.92	10.78	7.93E-02
cg24570346	24.20	8.84	6.18E-03	32.07	17.65	6.91E-02	45.67	41.15	2.67E-01	18.78	11.59	1.05E-01	25.74	25.36	3.10E-01
cg05898482	-15.58	5.69	6.19E-03	-13.47	11.56	2.44E-01	-16.21	28.96	5.76E-01	-17.24	7.55	2.24E-02	-12.59	14.68	3.91E-01
cg26305174	9.59	3.50	6.20E-03	8.64	4.81	7.23E-02	13.08	7.59	8.48E-02	15.49	10.20	1.29E-01	2.81	9.42	7.66E-01
cg06128688	20.28	7.41	6.20E-03	14.96	14.01	2.86E-01	91.73	44.36	3.86E-02	15.52	7.87	4.87E-02	30.96	16.28	5.73E-02
cg13571700	-18.46	6.74	6.20E-03	-11.31	13.63	4.07E-01	-2.65	26.17	9.19E-01	-20.06	9.11	2.77E-02	-32.04	17.94	7.40E-02
cg24902842	16.52	6.04	6.20E-03	21.43	8.35	1.03E-02	19.00	13.38	1.56E-01	-8.87	19.23	6.45E-01	13.32	14.42	3.56E-01
cg00986053	20.68	7.55	6.20E-03	19.39	16.36	2.36E-01	14.21	30.49	6.41E-01	21.87	9.51	2.15E-02	19.82	24.59	4.20E-01
cg23936587	23.12	8.45	6.21E-03	31.40	23.99	1.91E-01	-30.29	50.90	5.52E-01	22.52	9.64	1.96E-02	34.24	29.62	2.48E-01
cg02295678	38.20	13.96	6.21E-03	-3.59	46.01	9.38E-01	-124.75	155.98	4.24E-01	43.42	15.27	4.48E-03	50.45	54.85	3.58E-01
cg26596651	22.13	8.09	6.21E-03	5.01	18.85	7.91E-01	8.71	33.52	7.95E-01	29.55	9.85	2.69E-03	9.22	28.00	7.42E-01

cg22222261	22.93	8.38	6.22E-03	14.30	18.69	4.44E-01	27.28	46.69	5.59E-01	22.38	10.05	2.60E-02	50.60	31.35	1.07E-01
cg19515518	-16.37	5.98	6.22E-03	-25.72	16.13	1.11E-01	-31.95	38.88	4.11E-01	-14.72	7.05	3.67E-02	-12.40	17.47	4.78E-01
cg06774875	53.82	19.67	6.22E-03	50.60	25.48	4.71E-02	88.71	76.42	2.46E-01	75.72	60.68	2.12E-01	42.22	40.78	3.01E-01
cg00114012	19.52	7.14	6.23E-03	18.68	11.75	1.12E-01	13.24	14.82	3.72E-01	23.19	14.40	1.07E-01	25.19	18.21	1.67E-01
cg25573488	-46.03	16.83	6.23E-03	-26.79	38.39	4.85E-01	161.51	183.61	3.79E-01	-57.03	19.87	4.10E-03	-16.22	58.72	7.82E-01
cg06754047	-23.61	8.63	6.23E-03	-23.64	9.07	9.11E-03	-4.28	116.29	9.71E-01	-28.77	90.95	7.52E-01	-24.00	30.68	4.34E-01
cg13508904	-14.14	5.17	6.23E-03	0.95	16.70	9.55E-01	3.50	29.39	9.05E-01	-14.38	5.77	1.27E-02	-39.67	19.46	4.15E-02
cg27354094	-17.17	6.28	6.23E-03	-27.74	11.11	1.26E-02	-20.09	13.38	1.33E-01	-5.99	11.97	6.17E-01	-12.12	14.55	4.05E-01
cg17513182	15.63	5.71	6.23E-03	9.69	10.78	3.68E-01	17.94	40.77	6.60E-01	17.56	7.17	1.43E-02	21.83	22.56	3.33E-01
cg26860113	40.23	14.71	6.23E-03	53.07	45.53	2.44E-01	-37.46	123.60	7.62E-01	33.73	16.37	3.94E-02	107.63	53.96	4.61E-02
cg19601653	-72.11	26.36	6.23E-03	-71.00	36.43	5.13E-02	149.94	146.76	3.07E-01	-83.82	67.85	2.17E-01	-92.51	48.69	5.75E-02
cg14507014	-12.40	4.53	6.24E-03	-19.51	6.74	3.78E-03	-14.37	9.84	1.44E-01	-0.52	7.71	9.47E-01	-15.04	9.75	1.23E-01
cg16710042	14.65	5.36	6.24E-03	16.00	6.74	1.77E-02	2.68	16.56	8.71E-01	22.24	26.17	3.96E-01	15.02	11.35	1.86E-01
cg16792183	-41.52	15.18	6.25E-03	-49.96	19.11	8.93E-03	34.17	76.16	6.54E-01	-35.54	46.22	4.42E-01	-33.94	32.32	2.94E-01
cg13660701	40.62	14.86	6.25E-03	30.60	20.15	1.29E-01	39.13	43.21	3.65E-01	-1.02	76.77	9.89E-01	64.47	27.07	1.73E-02
cg04267526	14.01	5.12	6.26E-03	23.43	8.30	4.75E-03	0.55	14.29	9.69E-01	13.79	9.81	1.60E-01	5.74	10.98	6.01E-01
cg04575202	10.39	3.80	6.27E-03	16.85	6.07	5.50E-03	11.57	8.37	1.67E-01	-0.32	10.16	9.75E-01	5.50	7.43	4.59E-01
cg04452195	5.77	2.11	6.28E-03	5.26	3.02	8.11E-02	12.22	4.95	1.36E-02	2.76	4.38	5.29E-01	3.44	6.86	6.16E-01
cg18348736	22.13	8.10	6.28E-03	28.36	9.58	3.07E-03	-22.14	37.31	5.53E-01	30.11	46.73	5.19E-01	9.60	17.76	5.89E-01
cg23263911	-48.61	17.79	6.28E-03	-50.86	21.73	1.93E-02	48.34	86.10	5.74E-01	-52.27	49.90	2.95E-01	-62.14	44.45	1.62E-01
cg01112965	40.86	14.95	6.28E-03	42.76	18.16	1.85E-02	52.30	71.19	4.63E-01	64.08	58.53	2.74E-01	25.30	32.43	4.35E-01
cg00916179	-20.82	7.62	6.30E-03	-19.03	12.97	1.42E-01	-11.71	14.99	4.35E-01	-7.91	18.67	6.72E-01	-43.06	15.89	6.72E-03
cg09322899	-26.96	9.87	6.30E-03	-5.14	21.51	8.11E-01	-23.71	51.88	6.48E-01	-34.10	12.22	5.27E-03	-27.48	30.96	3.75E-01
cg22905292	-19.50	7.14	6.30E-03	-46.73	18.94	1.36E-02	1.63	37.89	9.66E-01	-15.00	8.24	6.86E-02	-23.15	26.67	3.85E-01
cg02288341	50.53	18.50	6.30E-03	50.39	24.59	4.04E-02	92.23	78.05	2.37E-01	71.84	54.42	1.87E-01	32.51	36.13	3.68E-01
cg10884975	-27.71	10.15	6.30E-03	-17.17	13.95	2.18E-01	-69.63	28.68	1.52E-02	-16.25	29.15	5.77E-01	-35.35	21.39	9.84E-02
cg01350996	12.83	4.70	6.31E-03	13.39	7.78	8.54E-02	15.02	8.90	9.15E-02	14.17	9.96	1.55E-01	4.59	12.81	7.20E-01
cg27312338	12.36	4.53	6.31E-03	12.97	7.36	7.82E-02	20.90	8.98	2.00E-02	0.16	14.49	9.91E-01	7.91	8.71	3.64E-01
cg19695404	20.11	7.36	6.31E-03	8.37	14.60	5.67E-01	-4.45	38.44	9.08E-01	21.87	9.76	2.50E-02	40.78	19.71	3.85E-02
cg05230834	19.15	7.01	6.31E-03	22.18	10.30	3.13E-02	19.20	17.14	2.63E-01	-2.81	26.29	9.15E-01	19.66	12.84	1.26E-01
cg04939496	10.27	3.76	6.32E-03	18.34	7.54	1.50E-02	10.11	9.79	3.01E-01	6.81	5.20	1.90E-01	8.07	13.15	5.39E-01
cg10632093	14.28	5.23	6.32E-03	10.67	10.71	3.19E-01	6.70	13.65	6.24E-01	16.68	7.78	3.20E-02	19.72	12.96	1.28E-01
cg06685950	16.17	5.92	6.32E-03	10.08	13.31	4.49E-01	-0.57	22.12	9.79E-01	18.90	7.31	9.67E-03	24.36	21.79	2.64E-01
cg16792312	-26.33	9.64	6.32E-03	-16.93	16.46	3.03E-01	-15.27	23.68	5.19E-01	-34.86	20.15	8.37E-02	-38.19	18.84	4.27E-02
cg02731042	30.80	11.28	6.32E-03	12.16	39.39	7.58E-01	85.05	141.31	5.47E-01	32.03	12.31	9.26E-03	32.97	42.25	4.35E-01

cg07121640	-39.49	14.46	6.33E-03	-14.26	32.31	6.59E-01	-220.36	169.13	1.93E-01	-43.07	17.60	1.44E-02	-50.73	42.27	2.30E-01
cg09717927	11.98	4.39	6.33E-03	23.04	5.54	3.17E-05	10.91	6.54	9.52E-02	8.95	6.34	1.58E-01	3.37	6.41	5.99E-01
cg22054793	-13.25	4.85	6.33E-03	-20.76	10.83	5.51E-02	-12.09	19.73	5.40E-01	-14.10	6.11	2.10E-02	5.18	14.83	7.27E-01
cg05997801	23.82	8.73	6.33E-03	39.54	17.75	2.59E-02	22.88	15.70	1.45E-01	22.09	15.99	1.67E-01	4.08	22.42	8.56E-01
cg14479709	-14.97	5.48	6.33E-03	-6.49	9.64	5.01E-01	-4.98	13.58	7.14E-01	-20.45	9.38	2.91E-02	-29.51	13.24	2.58E-02
cg24777950	7.20	2.64	6.33E-03	6.27	3.45	6.93E-02	11.03	6.74	1.02E-01	12.20	6.82	7.36E-02	0.25	7.82	9.75E-01
cg00987918	-12.89	4.72	6.34E-03	-15.11	7.15	3.47E-02	-18.82	11.09	8.99E-02	-4.61	12.28	7.08E-01	-9.43	9.73	3.32E-01
cg11702351	-19.14	7.01	6.34E-03	-26.98	13.15	4.02E-02	-28.22	20.18	1.62E-01	-16.67	10.22	1.03E-01	-1.76	19.86	9.29E-01
cg02901861	27.50	10.08	6.34E-03	45.26	23.33	5.24E-02	14.73	71.58	8.37E-01	23.02	12.11	5.72E-02	27.99	31.68	3.77E-01
cg01248080	28.67	10.50	6.34E-03	70.81	34.86	4.22E-02	-37.52	125.23	7.65E-01	26.84	11.57	2.04E-02	4.97	37.60	8.95E-01
cg15848350	18.38	6.73	6.34E-03	21.22	9.61	2.73E-02	32.57	16.11	4.32E-02	22.90	18.22	2.09E-01	-2.88	14.67	8.44E-01
cg23597562	23.60	8.65	6.34E-03	22.39	18.13	2.17E-01	4.64	33.94	8.91E-01	25.19	11.27	2.54E-02	28.38	25.06	2.57E-01
cg23243463	19.61	7.18	6.35E-03	19.70	10.08	5.06E-02	24.09	27.48	3.81E-01	36.98	29.46	2.09E-01	15.79	11.91	1.85E-01
cg24971846	-22.09	8.09	6.35E-03	-23.17	11.71	4.79E-02	-43.54	29.89	1.45E-01	-31.37	17.87	7.91E-02	-5.72	16.38	7.27E-01
cg12079362	32.37	11.86	6.35E-03	38.35	14.42	7.82E-03	-20.44	51.36	6.91E-01	-6.27	66.60	9.25E-01	32.35	24.30	1.83E-01
cg23679333	-21.35	7.82	6.35E-03	-17.62	19.95	3.77E-01	-6.38	57.14	9.11E-01	-18.99	9.22	3.94E-02	-45.24	23.91	5.85E-02
cg16715162	29.01	10.63	6.36E-03	34.28	39.14	3.81E-01	111.41	150.42	4.59E-01	27.94	11.39	1.41E-02	31.62	47.62	5.07E-01
cg03459809	-13.23	4.85	6.36E-03	-11.31	9.05	2.11E-01	-4.67	12.20	7.02E-01	-16.73	8.11	3.90E-02	-16.53	10.92	1.30E-01
cg24932241	-21.25	7.79	6.36E-03	-25.33	10.93	2.05E-02	-28.13	20.91	1.78E-01	-13.64	22.25	5.40E-01	-12.19	16.20	4.52E-01
cg01899540	15.09	5.53	6.36E-03	20.93	17.36	2.28E-01	35.11	25.87	1.75E-01	14.00	6.18	2.35E-02	2.93	24.29	9.04E-01
cg12529301	45.98	16.86	6.38E-03	51.02	53.62	3.41E-01	169.01	202.11	4.03E-01	45.70	18.66	1.43E-02	31.64	60.11	5.99E-01
cg03728296	11.51	4.22	6.38E-03	10.93	7.20	1.29E-01	13.52	8.78	1.23E-01	3.65	9.28	6.94E-01	17.69	9.01	4.97E-02
cg15812469	16.95	6.22	6.38E-03	13.39	7.68	8.13E-02	7.57	19.10	6.92E-01	25.51	22.29	2.52E-01	33.41	15.46	3.07E-02
cg24556026	12.32	4.52	6.39E-03	13.60	6.77	4.46E-02	18.97	8.64	2.81E-02	7.06	13.49	6.01E-01	1.73	10.96	8.75E-01
cg04995978	-42.80	15.70	6.39E-03	-41.84	25.44	1.00E-01	24.64	47.62	6.05E-01	-59.00	27.26	3.04E-02	-55.77	37.08	1.33E-01
cg05848579	-18.05	6.62	6.39E-03	-14.70	9.24	1.11E-01	3.02	21.60	8.89E-01	-29.19	16.26	7.26E-02	-26.22	13.90	5.93E-02
cg04718050	-12.96	4.75	6.39E-03	-9.70	6.69	1.47E-01	-22.90	13.57	9.16E-02	-8.27	10.64	4.37E-01	-20.77	11.40	6.84E-02
cg04373948	-9.04	3.31	6.40E-03	-13.54	6.39	3.42E-02	-5.60	11.93	6.39E-01	-8.61	4.54	5.80E-02	-3.12	9.52	7.43E-01
cg23197628	-25.20	9.24	6.40E-03	-29.84	13.50	2.71E-02	-4.00	26.24	8.79E-01	-10.74	25.39	6.72E-01	-33.87	17.64	5.48E-02
cg07781399	20.84	7.64	6.40E-03	20.14	10.45	5.40E-02	5.18	19.94	7.95E-01	19.31	30.46	5.26E-01	31.68	15.12	3.62E-02
cg05617732	-15.85	5.82	6.40E-03	-13.68	11.34	2.28E-01	-4.54	15.36	7.68E-01	-19.62	8.81	2.60E-02	-19.34	14.62	1.86E-01
cg16977270	-12.10	4.44	6.41E-03	-11.58	9.09	2.03E-01	-7.75	8.17	3.43E-01	-19.96	13.49	1.39E-01	-13.65	7.41	6.56E-02
cg15386434	-6.39	2.35	6.41E-03	-7.93	3.74	3.39E-02	-0.59	4.48	8.95E-01	-6.41	5.82	2.71E-01	-12.24	5.71	3.20E-02
cg10974128	-9.79	3.59	6.41E-03	-14.79	5.32	5.47E-03	-9.76	9.57	3.08E-01	-6.57	8.87	4.59E-01	-2.53	7.32	7.29E-01
cg14250984	17.93	6.58	6.41E-03	18.92	9.65	5.00E-02	29.75	13.83	3.15E-02	17.02	16.62	3.06E-01	-1.57	16.79	9.26E-01

cg16874494	-64.47	23.65	6.42E-03	-41.37	33.60	2.18E-01	43.13	112.37	7.01E-01	-92.65	46.62	4.69E-02	-108.60	52.50	3.86E-02
cg09889987	21.94	8.05	6.42E-03	42.27	21.80	5.25E-02	-18.14	41.15	6.59E-01	19.88	9.32	3.28E-02	25.76	28.68	3.69E-01
cg09166022	22.79	8.36	6.42E-03	43.68	20.68	3.47E-02	69.87	85.16	4.12E-01	17.35	9.59	7.05E-02	26.72	32.28	4.08E-01
cg13137533	-24.59	9.02	6.42E-03	-35.43	19.72	7.24E-02	-50.95	30.76	9.76E-02	-23.83	10.07	1.80E-02	19.78	30.22	5.13E-01
cg16686396	9.81	3.60	6.42E-03	13.64	5.06	7.01E-03	8.85	7.25	2.22E-01	2.41	14.89	8.71E-01	3.08	8.29	7.10E-01
cg23066159	-9.20	3.38	6.42E-03	-11.79	5.98	4.85E-02	0.20	8.47	9.81E-01	-8.05	6.15	1.90E-01	-13.81	7.19	5.50E-02
cg07979652	12.06	4.42	6.43E-03	6.65	8.04	4.08E-01	7.68	11.73	5.13E-01	17.72	7.02	1.16E-02	12.10	11.13	2.77E-01
cg06046705	27.57	10.12	6.43E-03	28.20	12.39	2.29E-02	38.87	32.20	2.27E-01	36.77	41.05	3.70E-01	15.55	24.24	5.21E-01
cg15541401	23.59	8.66	6.44E-03	38.00	18.67	4.18E-02	27.26	17.66	1.23E-01	16.89	13.13	1.98E-01	13.86	26.16	5.96E-01
cg12892303	17.09	6.27	6.44E-03	26.05	13.54	5.43E-02	8.78	12.98	4.99E-01	17.96	10.79	9.60E-02	15.81	13.59	2.45E-01
cg18516150	-10.13	3.72	6.44E-03	-18.63	8.98	3.81E-02	-6.70	14.42	6.42E-01	-7.70	4.63	9.63E-02	-13.06	10.89	2.30E-01
cg06993955	14.37	5.27	6.44E-03	19.58	7.79	1.19E-02	4.71	11.61	6.85E-01	17.93	12.31	1.45E-01	7.42	13.56	5.84E-01
cg26596307	-17.03	6.25	6.44E-03	-22.26	9.16	1.50E-02	-6.41	13.40	6.32E-01	-11.68	17.26	4.99E-01	-20.12	14.52	1.66E-01
cg06978288	15.60	5.73	6.44E-03	15.36	8.36	6.61E-02	23.10	12.73	6.95E-02	6.07	14.40	6.74E-01	16.20	13.89	2.43E-01
cg00837860	26.97	9.90	6.44E-03	24.46	13.31	6.62E-02	57.52	40.87	1.59E-01	-3.38	42.40	9.37E-01	30.71	17.13	7.30E-02
cg16429063	23.88	8.77	6.45E-03	15.65	17.06	3.59E-01	35.35	19.17	6.51E-02	25.85	13.60	5.73E-02	14.46	26.30	5.82E-01
cg00852783	17.66	6.48	6.46E-03	21.98	8.48	9.54E-03	37.22	36.27	3.05E-01	-6.00	30.22	8.43E-01	11.55	11.16	3.01E-01
cg20455001	-12.52	4.60	6.46E-03	-24.68	9.65	1.05E-02	3.53	19.84	8.59E-01	-9.01	5.86	1.24E-01	-14.97	14.21	2.92E-01
cg04436634	-10.06	3.69	6.46E-03	-15.45	9.04	8.72E-02	-1.03	17.44	9.53E-01	-8.24	4.51	6.76E-02	-16.28	10.81	1.32E-01
cg09875921	18.36	6.74	6.46E-03	5.36	20.16	7.90E-01	20.28	26.79	4.49E-01	20.03	7.73	9.62E-03	19.36	26.41	4.63E-01
cg00746693	20.39	7.49	6.46E-03	18.68	19.00	3.26E-01	-51.80	79.02	5.12E-01	22.68	8.69	9.03E-03	11.90	24.53	6.28E-01
cg06745695	21.07	7.74	6.46E-03	41.13	18.90	2.95E-02	21.73	79.01	7.83E-01	16.49	8.90	6.40E-02	22.42	29.78	4.52E-01
cg23854488	9.05	3.32	6.47E-03	9.18	5.95	1.23E-01	13.16	7.55	8.13E-02	14.89	8.28	7.23E-02	3.71	5.76	5.19E-01
cg07486732	-13.84	5.08	6.47E-03	-13.30	9.06	1.42E-01	-13.91	15.27	3.62E-01	-4.68	10.46	6.55E-01	-20.70	8.73	1.78E-02
cg05211662	18.20	6.69	6.47E-03	28.51	12.10	1.84E-02	16.85	19.70	3.92E-01	23.70	11.17	3.39E-02	-1.04	13.23	9.38E-01
cg01838965	-9.93	3.65	6.48E-03	-12.43	7.31	8.89E-02	-6.72	12.41	5.88E-01	-9.93	4.96	4.52E-02	-7.12	10.40	4.94E-01
cg26064870	-12.93	4.75	6.48E-03	0.07	14.62	9.96E-01	-2.82	19.18	8.83E-01	-15.17	5.44	5.33E-03	-16.95	17.72	3.39E-01
cg27009439	-11.26	4.14	6.49E-03	-8.37	8.56	3.28E-01	-1.97	15.66	9.00E-01	-12.20	5.39	2.37E-02	-18.39	12.59	1.44E-01
cg02466113	-53.45	19.64	6.49E-03	-65.17	22.53	3.82E-03	-99.38	50.42	4.87E-02	-65.19	64.53	3.12E-01	2.50	36.89	9.46E-01
cg23405575	16.32	5.99	6.49E-03	38.99	18.33	3.34E-02	33.56	35.26	3.41E-01	14.44	6.92	3.69E-02	3.03	17.76	8.64E-01
cg09479341	17.29	6.35	6.50E-03	20.24	13.80	1.42E-01	17.37	17.78	3.29E-01	13.27	8.45	1.16E-01	34.46	20.59	9.43E-02
cg25582295	-22.06	8.11	6.50E-03	-7.47	38.28	8.45E-01	49.99	172.37	7.72E-01	-20.66	8.50	1.51E-02	-70.14	38.93	7.16E-02
cg21755257	57.36	21.08	6.50E-03	58.53	61.01	3.37E-01	10.76	281.70	9.70E-01	68.39	24.07	4.49E-03	-19.75	64.10	7.58E-01
cg18898336	23.19	8.52	6.50E-03	12.29	18.49	5.06E-01	-6.34	21.75	7.71E-01	34.06	10.19	8.27E-04	27.69	22.13	2.11E-01
cg07626482	-16.77	6.16	6.51E-03	-7.97	10.79	4.60E-01	-20.99	17.02	2.18E-01	-17.90	10.32	8.27E-02	-27.08	14.30	5.83E-02

cg07895203	-21.59	7.94	6.52E-03	-35.58	17.37	4.05E-02	-4.29	28.13	8.79E-01	-14.38	10.17	1.57E-01	-49.49	24.81	4.61E-02
cg03466717	6.04	2.22	6.52E-03	6.51	3.04	3.20E-02	4.38	5.30	4.08E-01	6.44	7.11	3.65E-01	6.03	5.06	2.33E-01
cg13287772	27.74	10.20	6.52E-03	39.50	21.03	6.03E-02	19.02	22.02	3.88E-01	18.99	16.19	2.41E-01	44.55	26.03	8.70E-02
cg07715277	28.42	10.45	6.53E-03	54.86	22.49	1.47E-02	18.72	50.29	7.10E-01	23.80	13.34	7.42E-02	9.10	29.32	7.56E-01
cg23259381	-31.77	11.68	6.53E-03	17.15	33.43	6.08E-01	-104.54	126.46	4.08E-01	-39.42	13.14	2.71E-03	-23.12	41.45	5.77E-01
cg26841647	11.52	4.24	6.53E-03	13.33	9.03	1.40E-01	8.45	11.64	4.68E-01	13.92	5.80	1.64E-02	0.41	12.53	9.74E-01
cg24055703	-15.07	5.54	6.53E-03	-14.63	9.16	1.10E-01	-18.98	13.40	1.57E-01	-22.54	12.16	6.39E-02	-7.03	10.96	5.21E-01
cg01062651	17.58	6.46	6.53E-03	22.25	17.36	2.00E-01	30.43	19.20	1.13E-01	14.82	7.80	5.75E-02	14.13	25.83	5.84E-01
cg10608256	27.35	10.06	6.53E-03	10.48	32.94	7.50E-01	42.07	131.20	7.48E-01	31.22	10.95	4.36E-03	-3.46	41.91	9.34E-01
cg25308562	-8.78	3.23	6.54E-03	-7.00	5.99	2.43E-01	-20.19	8.77	2.13E-02	-7.01	4.98	1.59E-01	-6.91	8.20	3.99E-01
cg24964368	10.69	3.93	6.54E-03	8.68	5.27	9.94E-02	20.32	12.17	9.50E-02	14.51	10.71	1.75E-01	8.72	8.69	3.16E-01
cg04475027	16.97	6.24	6.54E-03	32.50	16.97	5.56E-02	-1.02	26.65	9.69E-01	16.33	8.08	4.34E-02	13.58	13.50	3.14E-01
cg07096247	-16.24	5.97	6.54E-03	-39.66	18.53	3.24E-02	-16.09	27.63	5.60E-01	-11.58	6.79	8.81E-02	-31.75	21.66	1.43E-01
cg07800892	-32.30	11.88	6.54E-03	-22.44	21.85	3.05E-01	-34.65	54.69	5.26E-01	-42.37	15.93	7.83E-03	-4.84	37.26	8.97E-01
cg00021030	24.02	8.83	6.54E-03	19.00	16.55	2.51E-01	20.34	17.90	2.56E-01	19.49	16.11	2.26E-01	45.66	21.39	3.28E-02
cg01389409	10.60	3.90	6.55E-03	10.69	5.10	3.62E-02	18.09	9.64	6.07E-02	-0.55	12.91	9.66E-01	9.00	9.68	3.53E-01
cg09602350	-14.83	5.45	6.55E-03	-16.21	11.04	1.42E-01	-25.66	13.39	5.54E-02	-13.44	8.55	1.16E-01	-6.30	12.72	6.20E-01
cg14571714	16.71	6.15	6.55E-03	12.74	15.60	4.14E-01	-1.13	21.00	9.57E-01	22.10	7.53	3.35E-03	1.29	20.11	9.49E-01
cg02124498	-14.91	5.49	6.56E-03	-12.76	13.97	3.61E-01	-42.31	29.64	1.53E-01	-11.28	6.51	8.34E-02	-34.20	17.15	4.62E-02
cg17894069	20.50	7.54	6.56E-03	28.46	11.33	1.20E-02	20.17	16.79	2.30E-01	16.51	15.78	2.95E-01	0.36	21.16	9.87E-01
ch.9.26162534R	-40.44	14.88	6.56E-03	-45.74	16.64	5.98E-03	-22.19	77.50	7.75E-01	20.43	83.60	8.07E-01	-28.07	40.97	4.93E-01
cg18249991	53.10	19.54	6.57E-03	54.48	44.96	2.26E-01	-263.66	248.93	2.90E-01	51.99	23.07	2.42E-02	81.39	65.90	2.17E-01
cg07329769	-50.50	18.58	6.58E-03	-43.45	23.38	6.31E-02	-85.96	64.19	1.81E-01	-50.89	64.88	4.33E-01	-57.66	41.32	1.63E-01
cg02251799	33.00	12.15	6.58E-03	43.41	19.50	2.60E-02	96.83	68.60	1.58E-01	31.16	18.94	9.99E-02	1.81	29.52	9.51E-01
cg19876838	24.77	9.12	6.59E-03	34.47	18.65	6.47E-02	17.29	18.72	3.55E-01	28.11	14.03	4.52E-02	5.60	28.60	8.45E-01
cg04745004	-9.71	3.57	6.59E-03	-1.00	8.54	9.07E-01	-21.49	13.24	1.05E-01	-11.51	4.45	9.67E-03	-5.06	10.97	6.45E-01
cg03470736	-67.86	24.98	6.60E-03	-83.98	68.71	2.22E-01	124.27	183.08	4.97E-01	-76.60	29.40	9.18E-03	-29.72	69.95	6.71E-01
cg18429196	15.04	5.54	6.60E-03	21.59	8.29	9.17E-03	18.87	13.32	1.57E-01	9.88	12.73	4.37E-01	1.40	12.65	9.12E-01
cg23648082	-15.06	5.54	6.60E-03	-25.73	8.19	1.67E-03	-4.87	10.64	6.47E-01	-16.37	16.88	3.32E-01	-6.60	11.23	5.57E-01
cg19325793	-8.28	3.05	6.60E-03	-12.63	5.35	1.84E-02	-10.51	8.29	2.05E-01	-7.11	4.88	1.46E-01	0.02	7.84	9.98E-01
cg11122009	7.69	2.83	6.61E-03	8.65	3.92	2.74E-02	8.36	5.85	1.53E-01	16.63	10.74	1.22E-01	0.39	6.77	9.54E-01
cg09875213	-14.82	5.46	6.61E-03	-24.39	8.18	2.86E-03	-8.17	12.86	5.25E-01	-10.80	13.20	4.13E-01	-3.17	12.07	7.93E-01
cg09968630	19.02	7.00	6.61E-03	21.25	17.32	2.20E-01	126.26	73.18	8.45E-02	14.74	7.56	5.13E-02	35.66	21.17	9.21E-02
cg24310460	-40.23	14.81	6.61E-03	-29.83	31.12	3.38E-01	-131.90	228.69	5.64E-01	-48.53	17.79	6.39E-03	9.29	53.61	8.62E-01
cg18762620	37.10	13.66	6.61E-03	30.86	38.34	4.21E-01	166.43	191.37	3.84E-01	37.02	15.22	1.50E-02	40.22	54.56	4.61E-01

cg19189176	-14.65	5.39	6.62E-03	-18.38	9.63	5.63E-02	-2.23	9.96	8.23E-01	-15.99	14.67	2.76E-01	-23.53	10.63	2.68E-02
cg10775230	21.92	8.07	6.63E-03	22.09	11.05	4.57E-02	46.53	17.78	8.88E-03	9.34	23.10	6.86E-01	4.99	17.50	7.76E-01
cg26993848	13.71	5.05	6.63E-03	15.79	7.62	3.84E-02	17.77	11.61	1.26E-01	18.57	12.85	1.48E-01	2.54	10.82	8.14E-01
cg14102186	-10.47	3.86	6.63E-03	-22.57	10.91	3.85E-02	-17.63	25.69	4.93E-01	-9.30	4.43	3.59E-02	-2.18	12.51	8.62E-01
cg22304662	18.81	6.93	6.64E-03	14.46	11.40	2.05E-01	27.00	13.03	3.82E-02	12.73	15.09	3.99E-01	22.94	18.68	2.20E-01
cg02758272	-30.28	11.16	6.64E-03	-26.11	17.20	1.29E-01	-40.09	31.61	2.05E-01	-27.45	23.75	2.48E-01	-35.23	23.04	1.26E-01
cg02106116	-18.42	6.78	6.64E-03	-30.10	12.51	1.61E-02	-5.09	16.19	7.53E-01	-11.36	8.07	1.59E-01	-39.24	18.43	3.33E-02
cg15002294	21.87	8.06	6.64E-03	25.44	10.45	1.49E-02	3.84	21.25	8.57E-01	21.88	33.12	5.09E-01	24.20	17.90	1.76E-01
cg09690103	-8.87	3.27	6.64E-03	-13.09	6.30	3.77E-02	-5.01	8.61	5.61E-01	-8.06	4.85	9.64E-02	-7.25	8.92	4.17E-01
cg15269394	10.75	3.96	6.64E-03	12.53	6.23	4.42E-02	20.63	8.94	2.10E-02	5.78	7.63	4.49E-01	0.50	11.06	9.64E-01
cg23598352	8.28	3.05	6.65E-03	9.42	5.83	1.06E-01	15.39	9.30	9.80E-02	6.66	4.12	1.06E-01	5.66	11.59	6.25E-01
cg08596817	20.96	7.72	6.66E-03	25.72	10.05	1.05E-02	27.82	38.95	4.75E-01	48.57	36.60	1.85E-01	7.72	13.54	5.69E-01
cg13311890	12.81	4.72	6.66E-03	17.55	7.62	2.13E-02	15.46	9.56	1.06E-01	8.13	11.07	4.63E-01	4.32	10.81	6.90E-01
cg24491328	24.70	9.10	6.66E-03	27.82	23.55	2.38E-01	70.81	52.79	1.80E-01	22.72	10.49	3.04E-02	19.64	34.77	5.72E-01
cg08657020	71.78	26.45	6.66E-03	77.04	31.80	1.54E-02	-71.43	150.54	6.35E-01	85.32	91.73	3.52E-01	70.00	60.08	2.44E-01
cg09980058	13.70	5.05	6.66E-03	21.77	10.43	3.68E-02	2.29	14.31	8.73E-01	17.47	8.69	4.44E-02	7.94	9.16	3.86E-01
cg08221272	22.77	8.39	6.66E-03	18.96	21.38	3.75E-01	9.72	46.99	8.36E-01	20.93	9.90	3.46E-02	46.95	27.08	8.29E-02
cg03918530	18.08	6.67	6.67E-03	24.69	11.53	3.22E-02	19.39	13.76	1.59E-01	15.35	13.45	2.54E-01	8.13	15.49	5.99E-01
cg02467646	-29.73	10.96	6.67E-03	1.41	26.69	9.58E-01	-1.13	94.76	9.90E-01	-36.66	13.14	5.28E-03	-36.33	31.26	2.45E-01
cg10047041	-11.45	4.22	6.68E-03	-15.07	8.17	6.52E-02	-6.88	10.18	4.99E-01	-10.26	6.60	1.20E-01	-13.48	10.81	2.13E-01
cg11746651	-21.20	7.82	6.68E-03	-32.47	10.07	1.26E-03	-0.26	13.16	9.84E-01	-13.25	15.22	3.84E-01	-31.68	11.83	7.39E-03
cg19559621	25.09	9.25	6.68E-03	12.86	27.21	6.37E-01	29.59	124.21	8.12E-01	25.20	10.13	1.29E-02	53.51	43.39	2.18E-01
cg12652953	20.43	7.53	6.68E-03	13.44	14.31	3.48E-01	34.36	19.67	8.07E-02	22.12	10.93	4.31E-02	11.52	23.58	6.25E-01
cg10055431	-19.23	7.09	6.68E-03	-0.92	21.29	9.65E-01	-42.14	28.36	1.37E-01	-19.69	8.10	1.51E-02	-23.13	28.59	4.19E-01
cg11152384	-16.54	6.10	6.68E-03	-18.25	7.80	1.94E-02	-15.97	14.46	2.70E-01	-3.05	31.89	9.24E-01	-13.99	14.59	3.38E-01
cg04361551	27.78	10.24	6.69E-03	20.41	21.27	3.37E-01	23.46	54.32	6.66E-01	25.98	13.51	5.44E-02	46.29	25.86	7.34E-02
cg01978105	13.55	5.00	6.69E-03	8.51	10.10	3.99E-01	33.37	40.51	4.10E-01	13.81	6.25	2.71E-02	21.21	15.77	1.79E-01
cg24074448	-16.93	6.24	6.69E-03	-19.44	9.07	3.21E-02	2.89	14.40	8.41E-01	-17.02	16.13	2.91E-01	-30.30	14.38	3.51E-02
cg22118017	17.37	6.41	6.69E-03	16.84	11.49	1.43E-01	10.45	16.20	5.19E-01	22.14	9.83	2.43E-02	10.18	19.49	6.01E-01
cg06850852	-66.59	24.56	6.70E-03	-85.96	68.63	2.10E-01	-193.90	247.51	4.33E-01	-50.64	28.47	7.53E-02	-135.38	71.44	5.81E-02
cg00303335	15.78	5.82	6.70E-03	20.02	8.92	2.48E-02	1.86	14.38	8.97E-01	21.88	11.28	5.23E-02	7.78	15.38	6.13E-01
cg04085713	-14.08	5.19	6.70E-03	-1.73	12.47	8.90E-01	-14.24	13.32	2.85E-01	-16.16	6.71	1.61E-02	-25.68	18.89	1.74E-01
cg23235277	32.70	12.06	6.70E-03	55.65	17.81	1.78E-03	26.62	22.92	2.45E-01	3.11	21.99	8.88E-01	37.48	28.59	1.90E-01
cg04882584	-22.68	8.36	6.70E-03	-41.07	14.16	3.71E-03	-9.63	26.29	7.14E-01	-13.74	15.13	3.64E-01	-12.96	16.93	4.44E-01
cg07658771	8.78	3.24	6.70E-03	11.37	4.71	1.59E-02	6.69	6.61	3.11E-01	4.54	13.18	7.31E-01	6.74	6.79	3.21E-01

cg07983888	-25.73	9.49	6.71E-03	-20.32	17.56	2.47E-01	-19.22	18.43	2.97E-01	-38.32	17.48	2.84E-02	-23.03	24.68	3.51E-01
cg11329853	-10.39	3.83	6.71E-03	-8.62	10.16	3.96E-01	-34.41	16.31	3.49E-02	-9.93	4.51	2.76E-02	-1.13	13.53	9.34E-01
cg20425384	-42.81	15.79	6.71E-03	-14.31	26.02	5.82E-01	-137.76	291.26	6.36E-01	-69.71	23.18	2.64E-03	-29.04	38.91	4.55E-01
cg10370850	-26.19	9.66	6.72E-03	-28.44	14.81	5.48E-02	-12.76	21.00	5.43E-01	-51.43	22.09	1.99E-02	-9.00	23.35	7.00E-01
cg07985589	-11.33	4.18	6.72E-03	-16.63	7.95	3.66E-02	-3.15	12.50	8.01E-01	-14.95	5.84	1.04E-02	3.00	10.50	7.75E-01
cg18986218	19.27	7.11	6.72E-03	25.96	11.07	1.90E-02	22.38	20.33	2.71E-01	43.52	28.65	1.29E-01	7.78	11.20	4.87E-01
cg22468894	19.69	7.26	6.73E-03	21.36	14.02	1.28E-01	8.47	15.45	5.83E-01	27.71	11.17	1.31E-02	4.15	24.51	8.65E-01
cg15721134	6.51	2.40	6.73E-03	4.87	3.72	1.90E-01	6.18	5.26	2.40E-01	10.59	4.68	2.35E-02	3.57	7.17	6.18E-01
cg25536373	15.33	5.66	6.73E-03	20.04	8.71	2.13E-02	4.98	12.42	6.89E-01	28.57	16.32	8.00E-02	9.61	11.32	3.96E-01
cg13981078	22.18	8.19	6.74E-03	6.02	19.36	7.56E-01	45.40	35.06	1.95E-01	24.91	9.89	1.17E-02	19.10	28.77	5.07E-01
cg12760869	16.49	6.09	6.74E-03	12.55	11.01	2.54E-01	10.50	16.12	5.15E-01	15.97	11.14	1.52E-01	25.25	12.10	3.69E-02
cg06850687	10.91	4.03	6.75E-03	6.30	5.81	2.78E-01	22.03	9.61	2.19E-02	15.16	6.47	1.92E-02	-0.14	11.03	9.90E-01
cg03352346	25.90	9.56	6.75E-03	11.92	16.85	4.79E-01	31.90	18.85	9.05E-02	38.56	18.96	4.20E-02	24.36	23.45	2.99E-01
cg00240178	-9.10	3.36	6.75E-03	-16.31	8.62	5.87E-02	-22.08	13.67	1.06E-01	-6.83	4.11	9.67E-02	-6.09	9.65	5.28E-01
cg02571055	-8.98	3.32	6.75E-03	-11.11	4.73	1.89E-02	-8.16	9.45	3.88E-01	-6.23	10.24	5.43E-01	-6.65	6.25	2.87E-01
cg20548765	-81.95	30.25	6.75E-03	-84.41	37.79	2.55E-02	-182.45	145.02	2.08E-01	2.57	99.60	9.79E-01	-90.21	64.00	1.59E-01
cg24516259	-17.73	6.55	6.76E-03	-29.71	10.06	3.15E-03	-20.43	21.72	3.47E-01	-11.11	13.52	4.11E-01	-2.76	13.05	8.33E-01
cg00371702	-11.31	4.17	6.76E-03	-16.83	6.43	8.88E-03	-4.92	10.15	6.28E-01	-4.75	11.58	6.82E-01	-9.90	7.89	2.10E-01
cg21626573	24.46	9.03	6.76E-03	37.98	17.96	3.44E-02	11.40	15.98	4.76E-01	16.93	18.04	3.48E-01	39.36	21.46	6.67E-02
cg07901253	18.40	6.79	6.77E-03	16.31	9.13	7.42E-02	23.84	19.05	2.11E-01	6.99	18.20	7.01E-01	29.80	16.01	6.26E-02
cg24415688	-12.24	4.52	6.77E-03	-11.79	6.60	7.40E-02	-19.04	9.69	4.94E-02	-9.71	16.62	5.59E-01	-7.72	9.25	4.04E-01
cg15420692	33.70	12.45	6.78E-03	78.74	32.09	1.41E-02	-66.00	95.55	4.90E-01	28.35	10.45	6.66E-03	28.47	38.60	4.61E-01
cg16877606	50.74	18.74	6.78E-03	93.43	50.39	6.37E-02	8.00	245.49	9.74E-01	44.82	21.12	3.38E-02	36.09	71.79	6.15E-01
cg21202522	21.56	7.96	6.79E-03	22.29	11.84	5.97E-02	20.91	15.24	1.70E-01	48.33	22.51	3.18E-02	-1.87	20.59	9.28E-01
cg01912040	9.55	3.53	6.79E-03	15.26	6.37	1.66E-02	10.60	8.17	1.94E-01	4.03	5.44	4.59E-01	13.96	12.04	2.46E-01
cg02624460	16.14	5.96	6.79E-03	20.31	12.28	9.81E-02	8.59	17.03	6.14E-01	17.04	8.30	3.99E-02	11.95	16.85	4.78E-01
cg14311485	-28.87	10.66	6.79E-03	-20.10	17.81	2.59E-01	-42.85	46.25	3.54E-01	-29.70	16.37	6.96E-02	-41.37	26.34	1.16E-01
cg24180219	-21.75	8.04	6.79E-03	-18.37	13.59	1.76E-01	-24.52	15.32	1.10E-01	-18.80	19.12	3.25E-01	-26.53	18.05	1.42E-01
cg23417096	21.42	7.91	6.79E-03	16.40	12.95	2.05E-01	32.16	16.44	5.04E-02	23.69	15.26	1.21E-01	11.71	22.27	5.99E-01
cg04076387	9.61	3.55	6.80E-03	4.30	5.25	4.12E-01	10.95	7.85	1.63E-01	24.50	11.59	3.45E-02	12.69	7.18	7.71E-02
cg15900034	-11.92	4.40	6.80E-03	-13.92	6.29	2.68E-02	-11.05	11.42	3.33E-01	-6.21	10.65	5.60E-01	-12.56	10.11	2.14E-01
cg01229873	-46.07	17.02	6.80E-03	-57.94	19.30	2.68E-03	64.94	125.14	6.04E-01	-50.33	103.55	6.27E-01	-4.74	40.51	9.07E-01
cg08159743	23.59	8.72	6.81E-03	38.69	18.23	3.38E-02	13.09	44.96	7.71E-01	8.95	13.30	5.01E-01	34.20	15.80	3.04E-02
cg02895319	31.47	11.63	6.81E-03	32.17	14.54	2.70E-02	51.41	33.92	1.30E-01	65.04	43.93	1.39E-01	1.68	27.99	9.52E-01
cg20087303	-31.37	11.59	6.81E-03	-35.00	26.71	1.90E-01	-116.95	91.70	2.02E-01	-25.45	13.73	6.38E-02	-57.70	40.34	1.53E-01

cg22707397	-6.88	2.54	6.81E-03	-6.05	3.82	1.13E-01	-4.64	7.55	5.39E-01	-14.66	6.20	1.79E-02	-4.37	4.85	3.67E-01
cg14939082	11.65	4.31	6.82E-03	15.45	6.19	1.26E-02	10.76	11.09	3.32E-01	12.22	10.35	2.38E-01	2.28	9.81	8.16E-01
cg13691209	-13.97	5.16	6.82E-03	-10.96	8.08	1.75E-01	-14.05	11.62	2.27E-01	-17.26	11.56	1.35E-01	-16.82	11.70	1.50E-01
cg12735838	13.91	5.14	6.82E-03	15.62	7.33	3.30E-02	26.37	11.92	2.70E-02	0.59	13.98	9.66E-01	6.63	11.89	5.77E-01
cg18703515	-15.20	5.62	6.82E-03	-20.69	11.42	6.99E-02	-12.68	14.63	3.86E-01	-12.00	8.21	1.44E-01	-19.07	14.96	2.02E-01
cg15949830	19.16	7.08	6.82E-03	25.59	16.27	1.16E-01	17.80	37.99	6.39E-01	18.85	8.59	2.83E-02	9.21	22.77	6.86E-01
cg02126603	40.22	14.87	6.83E-03	6.23	31.76	8.45E-01	-9.79	55.55	8.60E-01	58.09	19.40	2.75E-03	44.60	42.65	2.96E-01
cg23204612	-22.58	8.35	6.83E-03	-5.56	20.50	7.86E-01	15.98	38.36	6.77E-01	-27.65	9.90	5.24E-03	-36.20	30.17	2.30E-01
cg18912107	-11.53	4.26	6.84E-03	-12.44	6.34	4.98E-02	-12.24	10.04	2.23E-01	-2.53	8.72	7.71E-01	-24.05	11.88	4.29E-02
cg00495443	10.05	3.72	6.84E-03	14.07	5.71	1.37E-02	8.03	7.33	2.73E-01	-0.18	10.71	9.87E-01	10.27	8.33	2.18E-01
cg02651400	28.24	10.44	6.84E-03	28.54	27.90	3.06E-01	-18.75	47.67	6.94E-01	27.26	13.37	4.16E-02	42.09	23.20	6.96E-02
cg07462279	15.96	5.90	6.85E-03	20.70	10.48	4.82E-02	15.98	14.16	2.59E-01	17.40	9.26	6.02E-02	-4.28	18.35	8.15E-01
cg01053766	-43.84	16.22	6.86E-03	-26.04	27.49	3.43E-01	-92.99	51.76	7.24E-02	-35.61	25.62	1.65E-01	-74.33	41.42	7.28E-02
cg07184807	-7.63	2.82	6.86E-03	-7.72	4.56	9.04E-02	-4.49	6.28	4.74E-01	-12.60	5.71	2.73E-02	-4.02	6.84	5.57E-01
cg22790142	-48.05	17.77	6.86E-03	-24.08	46.91	6.08E-01	68.91	232.80	7.67E-01	-45.04	19.95	2.40E-02	-162.50	74.49	2.91E-02
cg07853743	36.49	13.50	6.86E-03	42.55	27.05	1.16E-01	-37.05	91.34	6.85E-01	36.57	16.99	3.14E-02	36.98	43.11	3.91E-01
cg15720043	23.03	8.52	6.86E-03	29.45	10.78	6.30E-03	0.73	38.80	9.85E-01	8.41	35.77	8.14E-01	15.25	16.37	3.51E-01
cg01933251	34.47	12.75	6.87E-03	28.80	41.66	4.89E-01	-69.03	135.15	6.10E-01	41.03	14.00	3.38E-03	-24.73	49.11	6.15E-01
cg00971827	-12.51	4.63	6.87E-03	-15.95	12.13	1.88E-01	-35.50	21.07	9.20E-02	-9.89	5.45	6.95E-02	-15.88	15.97	3.20E-01
cg00955307	12.46	4.61	6.88E-03	14.59	6.65	2.81E-02	19.17	9.56	4.51E-02	7.52	14.19	5.96E-01	1.10	10.81	9.19E-01
cg17805199	16.52	6.11	6.88E-03	12.72	8.68	1.43E-01	27.54	12.22	2.43E-02	28.92	19.58	1.40E-01	3.25	15.42	8.33E-01
cg20487152	-15.15	5.60	6.88E-03	-15.08	15.23	3.22E-01	-23.43	19.10	2.20E-01	-14.06	6.84	3.99E-02	-15.39	17.08	3.68E-01
cg11940663	34.91	12.92	6.88E-03	-12.76	43.43	7.69E-01	44.27	131.36	7.36E-01	41.03	14.19	3.83E-03	21.96	47.74	6.46E-01
cg15469655	-15.01	5.56	6.88E-03	-5.89	14.08	6.76E-01	1.48	17.97	9.35E-01	-21.90	7.02	1.81E-03	-4.29	15.87	7.87E-01
cg26230245	-57.42	21.25	6.89E-03	-47.00	27.62	8.89E-02	-115.06	60.57	5.75E-02	-6.32	64.39	9.22E-01	-83.70	50.59	9.80E-02
cg23680049	62.23	23.03	6.89E-03	84.81	64.14	1.86E-01	-96.01	247.48	6.98E-01	60.54	26.53	2.25E-02	59.84	69.77	3.91E-01
cg17053591	24.44	9.04	6.89E-03	21.50	18.58	2.47E-01	-0.67	37.93	9.86E-01	30.89	11.61	7.80E-03	6.43	28.69	8.23E-01
cg08292483	13.80	5.11	6.89E-03	4.74	10.76	6.60E-01	25.18	10.23	1.39E-02	15.93	8.05	4.79E-02	0.39	14.55	9.79E-01
cg13373343	-13.81	5.11	6.89E-03	-8.52	11.95	4.76E-01	-8.80	31.02	7.77E-01	-14.35	6.12	1.91E-02	-21.60	16.75	1.97E-01
cg03562868	8.08	2.99	6.90E-03	6.39	5.36	2.34E-01	-0.65	8.02	9.36E-01	10.40	4.50	2.10E-02	14.54	9.00	1.06E-01
cg15506894	10.98	4.06	6.90E-03	9.44	8.09	2.43E-01	12.10	9.10	1.84E-01	9.88	6.17	1.09E-01	16.66	12.04	1.67E-01
cg10054197	18.95	7.01	6.90E-03	14.88	22.24	5.03E-01	-99.07	112.60	3.79E-01	17.98	7.69	1.93E-02	44.77	27.60	1.05E-01
cg09747891	-10.95	4.05	6.90E-03	-9.62	10.90	3.78E-01	-19.94	16.59	2.30E-01	-11.19	4.73	1.81E-02	-3.29	15.39	8.31E-01
cg14531564	-8.60	3.18	6.90E-03	-9.92	3.96	1.23E-02	-5.19	6.90	4.52E-01	-7.15	12.52	5.68E-01	-8.20	11.46	4.74E-01
cg16199098	18.47	6.84	6.90E-03	6.65	19.37	7.31E-01	65.06	53.68	2.25E-01	18.79	7.64	1.40E-02	26.32	28.19	3.50E-01

cg00646546	22.83	8.45	6.90E-03	39.20	20.15	5.18E-02	26.53	22.31	2.34E-01	19.78	10.97	7.15E-02	4.57	28.54	8.73E-01
cg02663193	24.20	8.96	6.91E-03	30.30	17.02	7.51E-02	20.30	18.98	2.85E-01	22.18	14.79	1.34E-01	23.58	24.51	3.36E-01
cg22524657	21.04	7.79	6.91E-03	16.54	25.49	5.17E-01	-27.85	87.82	7.51E-01	24.16	8.57	4.81E-03	-3.34	28.85	9.08E-01
cg12236164	24.04	8.90	6.91E-03	34.50	13.30	9.48E-03	31.09	23.84	1.92E-01	28.33	23.80	2.34E-01	1.07	17.04	9.50E-01
cg24029050	-16.91	6.26	6.91E-03	-29.07	16.20	7.27E-02	10.36	19.99	6.04E-01	-18.09	7.75	1.96E-02	-17.81	19.79	3.68E-01
cg20994801	19.75	7.31	6.91E-03	18.37	10.22	7.23E-02	29.99	16.82	7.45E-02	-2.47	31.51	9.37E-01	19.61	14.76	1.84E-01
cg02358434	21.57	7.99	6.92E-03	34.80	13.23	8.51E-03	5.37	14.67	7.15E-01	31.75	21.71	1.44E-01	14.70	17.69	4.06E-01
cg21578322	22.99	8.51	6.92E-03	35.92	23.30	1.23E-01	-84.82	87.38	3.32E-01	21.77	9.63	2.38E-02	26.31	31.02	3.96E-01
cg01610488	26.14	9.68	6.92E-03	35.18	17.01	3.87E-02	25.86	28.53	3.65E-01	26.35	20.70	2.03E-01	17.56	16.55	2.88E-01
cg20027296	19.07	7.06	6.92E-03	15.00	9.37	1.09E-01	17.11	16.37	2.96E-01	45.75	15.88	3.97E-03	9.17	12.19	4.52E-01
cg12314527	13.61	5.04	6.93E-03	15.82	9.06	8.07E-02	-7.39	16.03	6.45E-01	18.72	7.65	1.44E-02	8.32	12.70	5.12E-01
cg25274468	-16.77	6.21	6.93E-03	-26.51	10.53	1.18E-02	1.10	15.65	9.44E-01	-14.73	11.39	1.96E-01	-16.92	13.99	2.27E-01
cg21307043	-24.27	8.99	6.93E-03	-23.56	23.91	3.24E-01	-53.09	50.45	2.93E-01	-22.63	10.27	2.76E-02	-31.47	36.33	3.86E-01
cg08968034	-13.80	5.11	6.94E-03	-10.25	8.11	2.06E-01	-19.23	12.09	1.12E-01	-10.38	11.58	3.70E-01	-18.67	10.70	8.11E-02
cg13332193	-16.78	6.22	6.94E-03	-15.20	8.81	8.43E-02	-10.52	14.99	4.83E-01	-35.32	16.07	2.79E-02	-11.73	14.66	4.24E-01
cg16700673	-41.22	15.27	6.95E-03	-57.68	45.86	2.08E-01	195.38	189.54	3.03E-01	-44.54	17.08	9.10E-03	-5.75	53.03	9.14E-01
cg02469487	61.03	22.61	6.95E-03	57.97	53.29	2.77E-01	-166.53	266.95	5.33E-01	59.16	26.26	2.43E-02	110.74	84.39	1.89E-01
cg17019285	49.38	18.29	6.95E-03	84.19	30.26	5.40E-03	-117.56	157.52	4.55E-01	35.11	24.48	1.52E-01	17.90	64.91	7.83E-01
cg04508233	41.22	15.27	6.96E-03	56.59	38.91	1.46E-01	-43.11	189.46	8.20E-01	36.67	17.43	3.53E-02	64.72	57.23	2.58E-01
cg08058988	-15.99	5.93	6.96E-03	-36.99	17.21	3.16E-02	6.66	35.54	8.51E-01	-14.56	6.84	3.33E-02	-8.39	18.46	6.49E-01
cg20294155	27.82	10.31	6.97E-03	14.21	17.27	4.11E-01	28.15	42.55	5.08E-01	35.02	15.62	2.50E-02	39.15	26.68	1.42E-01
cg21899743	-48.11	17.83	6.97E-03	-62.46	24.92	1.22E-02	-9.17	95.66	9.24E-01	-45.24	43.46	2.98E-01	-28.73	33.41	3.90E-01
cg11313780	14.87	5.51	6.98E-03	11.43	7.08	1.06E-01	33.49	11.47	3.51E-03	8.60	12.46	4.90E-01	7.60	13.36	5.69E-01
cg01807748	-17.04	6.31	6.98E-03	-6.54	16.39	6.90E-01	0.90	28.38	9.75E-01	-21.16	7.36	4.07E-03	-8.31	24.43	7.34E-01
cg09003373	19.83	7.35	6.99E-03	22.22	11.44	5.20E-02	19.61	14.85	1.87E-01	17.90	15.66	2.53E-01	15.64	21.11	4.59E-01
cg24098938	35.04	12.99	6.99E-03	6.02	33.04	8.55E-01	2.91	145.12	9.84E-01	43.48	15.01	3.78E-03	17.23	43.71	6.93E-01
cg11521212	-10.87	4.03	7.00E-03	-15.69	7.19	2.91E-02	-16.19	10.42	1.20E-01	-7.55	6.68	2.58E-01	-4.45	9.72	6.47E-01
cg01312837	18.45	6.84	7.00E-03	17.03	9.20	6.42E-02	38.83	14.07	5.79E-03	5.04	14.33	7.25E-01	7.94	22.24	7.21E-01
cg06874403	13.33	4.94	7.00E-03	6.41	6.80	3.46E-01	18.41	10.97	9.33E-02	22.85	18.51	2.17E-01	23.18	11.13	3.72E-02
cg05028089	39.47	14.64	7.01E-03	39.65	17.90	2.68E-02	-12.89	69.78	8.53E-01	50.89	69.60	4.65E-01	46.40	29.70	1.18E-01
cg24363955	-13.35	4.95	7.01E-03	-13.89	14.43	3.36E-01	-5.24	20.67	8.00E-01	-12.55	5.72	2.83E-02	-26.73	18.04	1.39E-01
cg05470497	26.61	9.87	7.01E-03	42.12	16.61	1.12E-02	20.67	17.92	2.49E-01	11.73	22.38	6.00E-01	21.38	25.57	4.03E-01
cg05607502	9.32	3.46	7.02E-03	6.55	4.08	1.09E-01	17.82	7.10	1.21E-02	17.97	8.91	4.38E-02	2.99	5.98	6.17E-01
cg04249066	39.48	14.65	7.03E-03	58.61	34.32	8.77E-02	-26.96	104.14	7.96E-01	34.60	18.12	5.62E-02	46.50	38.47	2.27E-01
cg08659357	28.25	10.48	7.03E-03	45.79	41.87	2.74E-01	-46.16	119.95	7.00E-01	29.94	11.29	8.02E-03	-0.68	39.97	9.86E-01

cg23194719	-38.77	14.38	7.03E-03	-42.75	20.14	3.37E-02	-34.93	37.54	3.52E-01	19.17	48.86	6.95E-01	-52.61	28.40	6.40E-02
cg01310330	19.33	7.17	7.03E-03	18.52	10.87	8.85E-02	27.56	14.25	5.31E-02	0.18	19.91	9.93E-01	23.44	16.80	1.63E-01
cg02895051	23.61	8.76	7.03E-03	13.36	19.57	4.95E-01	32.52	20.57	1.14E-01	19.97	12.41	1.07E-01	42.33	25.27	9.39E-02
cg00754253	7.28	2.70	7.03E-03	8.60	3.70	2.01E-02	14.25	7.18	4.72E-02	4.54	6.55	4.88E-01	-0.58	6.84	9.32E-01
cg09789768	-10.10	3.75	7.03E-03	-9.39	5.39	8.14E-02	-7.61	9.13	4.04E-01	-17.63	10.15	8.24E-02	-8.86	8.15	2.77E-01
cg21818274	-11.91	4.42	7.04E-03	-14.95	7.72	5.27E-02	-2.44	9.91	8.06E-01	-20.00	9.35	3.25E-02	-8.24	8.85	3.52E-01
cg25709978	24.43	9.07	7.04E-03	9.84	17.41	5.72E-01	32.71	18.75	8.11E-02	24.12	16.66	1.48E-01	35.04	20.32	8.46E-02
cg26257869	24.48	9.08	7.04E-03	36.87	17.47	3.48E-02	13.07	19.30	4.98E-01	16.14	14.72	2.73E-01	42.88	25.42	9.17E-02
cg11577689	37.22	13.81	7.04E-03	62.07	39.68	1.18E-01	-62.70	193.07	7.45E-01	39.47	15.58	1.13E-02	-11.34	46.57	8.08E-01
cg09503045	32.76	12.16	7.04E-03	23.34	15.80	1.39E-01	14.23	35.86	6.92E-01	44.26	38.61	2.52E-01	66.65	27.62	1.58E-02
cg03292648	26.80	9.94	7.04E-03	29.84	19.64	1.29E-01	-5.94	40.59	8.84E-01	25.99	13.21	4.91E-02	40.83	29.07	1.60E-01
cg07728793	17.43	6.47	7.05E-03	10.83	12.83	3.99E-01	26.72	34.80	4.43E-01	16.58	8.00	3.83E-02	50.82	27.00	5.98E-02
cg21229079	20.31	7.54	7.05E-03	46.25	23.61	5.01E-02	16.57	36.37	6.49E-01	16.52	8.39	4.91E-02	32.00	34.10	3.48E-01
cg15894948	23.07	8.57	7.07E-03	16.13	14.92	2.80E-01	-1.29	34.23	9.70E-01	31.29	12.12	9.85E-03	20.41	25.98	4.32E-01
cg06216620	24.57	9.12	7.07E-03	-1.47	30.40	9.62E-01	33.91	136.37	8.04E-01	29.02	9.94	3.51E-03	1.82	36.26	9.60E-01
cg08252499	30.50	11.32	7.07E-03	20.58	36.11	5.69E-01	25.39	176.23	8.85E-01	28.73	12.40	2.05E-02	69.71	45.07	1.22E-01
cg00121884	20.38	7.57	7.07E-03	32.53	19.01	8.70E-02	30.54	25.01	2.22E-01	15.02	9.20	1.03E-01	30.68	27.73	2.69E-01
cg24843389	54.37	20.18	7.07E-03	59.36	49.08	2.26E-01	119.77	196.62	5.42E-01	65.23	24.30	7.28E-03	-14.72	55.85	7.92E-01
cg09388991	9.35	3.47	7.07E-03	4.67	5.13	3.62E-01	11.80	6.32	6.19E-02	18.18	5.97	2.31E-03	3.07	6.69	6.46E-01
cg22787604	-17.37	6.45	7.07E-03	-20.56	18.48	2.66E-01	-18.81	20.53	3.60E-01	-18.23	7.64	1.71E-02	-0.41	24.85	9.87E-01
cg03779078	27.29	10.13	7.08E-03	31.19	19.11	1.03E-01	36.41	35.54	3.06E-01	16.17	8.88	6.87E-02	70.37	28.18	1.25E-02
cg10700634	-32.42	12.04	7.08E-03	-29.31	14.77	4.73E-02	10.84	32.18	7.36E-01	-45.16	26.36	8.67E-02	-65.66	30.37	3.06E-02
cg15889106	13.95	5.18	7.08E-03	21.08	7.64	5.82E-03	13.81	13.57	3.09E-01	0.77	22.92	9.73E-01	6.45	8.83	4.65E-01
cg17535702	-15.97	5.93	7.09E-03	-17.52	11.22	1.18E-01	-28.82	13.00	2.66E-02	-11.25	10.36	2.77E-01	-7.51	13.78	5.86E-01
cg20715365	26.53	9.85	7.09E-03	25.87	22.03	2.40E-01	-31.49	69.18	6.49E-01	29.59	11.93	1.32E-02	18.61	31.42	5.54E-01
cg19795722	-15.57	5.78	7.10E-03	-13.55	16.60	4.14E-01	-24.86	28.62	3.85E-01	-15.22	6.58	2.08E-02	-17.76	22.68	4.34E-01
cg27532955	10.15	3.77	7.10E-03	11.31	9.10	2.14E-01	6.73	12.18	5.81E-01	10.97	4.76	2.13E-02	6.52	11.64	5.75E-01
cg20487230	23.47	8.72	7.10E-03	34.24	19.43	7.80E-02	34.60	39.00	3.75E-01	17.05	10.82	1.15E-01	37.85	27.57	1.70E-01
cg12549345	-17.44	6.48	7.11E-03	-17.42	9.91	7.87E-02	-18.06	14.38	2.09E-01	-16.52	15.51	2.87E-01	-17.68	14.69	2.29E-01
cg02840367	19.19	7.13	7.12E-03	20.30	14.22	1.54E-01	-13.06	24.94	6.00E-01	17.81	8.11	2.82E-02	44.13	19.70	2.51E-02
cg13683301	-15.61	5.80	7.12E-03	-16.80	16.15	2.98E-01	-3.38	26.60	8.99E-01	-17.84	6.64	7.19E-03	5.65	23.64	8.11E-01
cg03119028	10.37	3.85	7.12E-03	21.03	8.71	1.57E-02	7.04	13.44	6.01E-01	8.81	4.89	7.15E-02	1.92	12.22	8.75E-01
cg07549297	-9.46	3.52	7.12E-03	-17.33	7.87	2.77E-02	8.84	14.81	5.50E-01	-7.60	4.33	7.95E-02	-15.39	10.49	1.42E-01
cg00945666	12.22	4.54	7.13E-03	13.86	6.87	4.37E-02	19.14	14.64	1.91E-01	13.49	15.36	3.80E-01	8.29	7.37	2.61E-01
cg27494615	28.62	10.64	7.13E-03	11.15	31.85	7.26E-01	75.95	104.72	4.68E-01	30.35	11.95	1.11E-02	29.68	36.32	4.14E-01

cg14488605	-29.47	10.95	7.13E-03	-33.35	14.58	2.22E-02	0.59	29.31	9.84E-01	-34.38	36.97	3.52E-01	-37.04	23.99	1.23E-01
cg03700308	39.97	14.85	7.13E-03	35.49	17.53	4.29E-02	-12.95	68.07	8.49E-01	57.11	70.26	4.16E-01	66.15	34.11	5.24E-02
cg07212778	20.00	7.43	7.13E-03	21.09	17.40	2.26E-01	17.53	25.35	4.89E-01	19.83	10.10	4.96E-02	20.56	17.07	2.29E-01
cg27388962	-11.84	4.40	7.14E-03	-21.10	6.38	9.47E-04	-13.86	11.18	2.15E-01	-4.17	5.33	4.34E-01	-10.82	9.24	2.42E-01
cg03164043	37.70	14.01	7.14E-03	64.17	39.79	1.07E-01	-200.24	177.05	2.58E-01	36.82	15.65	1.86E-02	21.68	53.83	6.87E-01
cg20114732	18.90	7.03	7.14E-03	30.32	12.97	1.94E-02	24.12	30.31	4.26E-01	6.89	8.05	3.92E-01	29.98	12.82	1.93E-02
cg23352003	23.88	8.88	7.15E-03	21.65	11.64	6.28E-02	44.09	42.02	2.94E-01	25.30	33.91	4.56E-01	24.86	16.09	1.22E-01
cg23221052	-2.52	0.94	7.16E-03	-3.49	1.29	6.74E-03	-1.33	2.00	5.05E-01	-4.48	3.54	2.06E-01	-0.38	2.19	8.64E-01
cg00229423	26.40	9.82	7.16E-03	39.00	13.43	3.68E-03	19.12	29.69	5.20E-01	28.20	31.81	3.75E-01	3.01	19.21	8.76E-01
cg18822036	16.71	6.21	7.16E-03	14.06	13.90	3.12E-01	25.04	13.93	7.22E-02	10.52	8.91	2.38E-01	33.04	18.32	7.13E-02
cg05887636	-53.51	19.90	7.16E-03	-69.66	25.72	6.76E-03	-6.88	62.99	9.13E-01	-24.57	73.71	7.39E-01	-40.81	41.59	3.26E-01
cg13995427	13.68	5.09	7.16E-03	14.93	16.95	3.79E-01	42.57	51.56	4.09E-01	13.53	5.54	1.46E-02	9.02	21.15	6.70E-01
cg22713187	-21.11	7.85	7.17E-03	-2.12	21.88	9.23E-01	-65.51	34.23	5.57E-02	-22.25	8.97	1.32E-02	-6.76	34.16	8.43E-01
cg16501323	10.78	4.01	7.17E-03	7.41	5.32	1.64E-01	4.29	10.14	6.72E-01	17.14	13.45	2.02E-01	23.29	9.24	1.17E-02
cg13840174	-14.07	5.23	7.18E-03	-19.24	7.85	1.42E-02	-4.88	12.77	7.02E-01	-9.70	11.62	4.04E-01	-14.81	12.19	2.25E-01
cg24522654	-10.02	3.73	7.18E-03	-12.90	5.30	1.50E-02	-3.92	9.59	6.83E-01	-3.40	10.88	7.55E-01	-11.19	7.66	1.44E-01
cg12980632	-21.39	7.96	7.18E-03	-31.35	16.71	6.07E-02	-1.30	22.53	9.54E-01	-16.79	9.25	6.96E-02	-58.70	27.59	3.34E-02
cg13712012	-10.70	3.98	7.19E-03	-10.97	8.57	2.01E-01	-16.01	13.04	2.20E-01	-9.14	5.41	9.09E-02	-12.65	10.29	2.19E-01
cg17879133	-14.89	5.54	7.19E-03	-23.50	9.34	1.18E-02	-13.91	21.13	5.10E-01	-6.53	5.12	2.02E-01	-25.73	11.22	2.19E-02
cg23648516	-14.81	5.51	7.19E-03	-24.93	11.82	3.49E-02	-5.56	26.58	8.34E-01	-11.17	7.08	1.15E-01	-17.79	15.03	2.37E-01
cg11675492	11.21	4.17	7.20E-03	10.54	6.67	1.14E-01	6.45	7.84	4.11E-01	11.85	12.23	3.33E-01	18.54	9.12	4.20E-02
cg02518338	13.47	5.01	7.20E-03	16.94	8.89	5.69E-02	16.07	10.31	1.19E-01	12.81	9.91	1.96E-01	5.35	11.48	6.41E-01
cg23394462	-19.38	7.21	7.21E-03	-19.16	14.20	1.77E-01	-20.34	34.53	5.56E-01	-13.02	9.39	1.66E-01	-53.89	21.84	1.36E-02
cg08931647	-16.95	6.31	7.21E-03	-15.76	9.68	1.04E-01	-6.81	18.14	7.07E-01	-32.30	12.51	9.82E-03	-6.12	14.07	6.64E-01
cg08117745	16.95	6.31	7.22E-03	8.35	14.65	5.69E-01	20.38	25.57	4.25E-01	18.09	7.94	2.28E-02	22.39	18.00	2.14E-01
cg18741586	20.28	7.55	7.22E-03	23.71	12.99	6.79E-02	17.05	11.53	1.39E-01	30.19	23.40	1.97E-01	14.06	20.95	5.02E-01
cg07267294	-11.22	4.18	7.22E-03	-22.72	7.53	2.54E-03	-8.82	13.90	5.26E-01	-6.47	5.39	2.30E-01	-7.08	10.22	4.89E-01
cg25812095	-13.00	4.84	7.22E-03	-18.15	7.22	1.19E-02	-8.95	12.22	4.64E-01	-2.81	11.45	8.06E-01	-13.65	10.44	1.91E-01
cg21915647	-29.08	10.83	7.23E-03	-14.71	33.93	6.65E-01	-232.69	159.15	1.44E-01	-27.34	11.84	2.10E-02	-63.21	44.97	1.60E-01
cg24504014	6.96	2.59	7.23E-03	5.58	3.48	1.09E-01	14.31	7.16	4.56E-02	8.52	5.87	1.47E-01	2.75	7.50	7.14E-01
cg24801210	-52.06	19.38	7.23E-03	-70.17	51.51	1.73E-01	-98.84	187.15	5.97E-01	-42.03	22.49	6.16E-02	-93.86	59.85	1.17E-01
cg26188685	-7.21	2.68	7.23E-03	-5.00	2.81	7.52E-02	-14.68	4.24	5.42E-04	-1.07	6.31	8.65E-01	-6.51	4.94	1.87E-01
cg20603611	27.37	10.19	7.23E-03	14.10	15.28	3.56E-01	56.44	30.26	6.22E-02	33.48	37.73	3.75E-01	33.25	16.79	4.76E-02
cg21646598	13.75	5.12	7.24E-03	7.36	18.42	6.90E-01	42.98	75.06	5.67E-01	15.27	5.59	6.28E-03	2.04	18.29	9.11E-01
cg03115444	17.59	6.55	7.24E-03	10.80	15.72	4.92E-01	-3.27	31.15	9.16E-01	23.34	7.75	2.61E-03	-11.39	24.90	6.47E-01

cg27561875	27.49	10.23	7.24E-03	6.35	21.62	7.69E-01	27.94	23.04	2.25E-01	35.49	14.96	1.77E-02	35.65	30.78	2.47E-01
cg17176016	19.98	7.44	7.24E-03	20.97	10.21	4.00E-02	5.92	19.34	7.59E-01	37.63	31.51	2.32E-01	22.12	14.44	1.26E-01
cg25339112	-14.10	5.25	7.24E-03	-18.75	8.03	1.95E-02	-6.21	10.48	5.54E-01	-6.20	15.85	6.96E-01	-18.13	11.41	1.12E-01
cg11399640	-10.24	3.81	7.24E-03	-16.51	7.37	2.51E-02	-4.59	8.57	5.93E-01	-11.12	6.24	7.49E-02	-4.74	9.48	6.17E-01
cg01056478	24.31	9.05	7.25E-03	31.85	10.91	3.51E-03	37.57	48.60	4.40E-01	3.95	39.81	9.21E-01	3.91	19.06	8.38E-01
cg20605045	24.51	9.13	7.25E-03	18.83	27.26	4.90E-01	-54.91	117.48	6.40E-01	27.76	10.04	5.68E-03	-4.14	38.96	9.15E-01
cg16523850	-4.80	1.79	7.25E-03	-7.52	2.88	9.11E-03	-0.71	3.48	8.39E-01	-6.22	4.04	1.24E-01	-3.24	4.52	4.74E-01
cg06845268	-16.19	6.03	7.25E-03	-29.71	16.65	7.44E-02	18.29	27.11	5.00E-01	-16.62	6.88	1.57E-02	-8.34	26.66	7.54E-01
cg09157127	-10.41	3.88	7.26E-03	-22.35	9.27	1.59E-02	3.35	16.99	8.43E-01	-8.16	4.88	9.41E-02	-10.74	10.34	2.99E-01
cg10210728	28.19	10.50	7.26E-03	26.18	24.43	2.84E-01	25.93	69.37	7.09E-01	29.43	13.07	2.43E-02	25.58	27.40	3.51E-01
cg03231447	-40.18	14.97	7.26E-03	-43.79	21.54	4.21E-02	37.41	65.75	5.69E-01	-63.73	31.35	4.21E-02	-27.18	30.70	3.76E-01
cg13460556	7.34	2.73	7.26E-03	6.45	3.79	8.92E-02	9.07	5.94	1.27E-01	12.46	4.68	7.68E-03	-3.13	7.09	6.59E-01
cg27173322	51.54	19.20	7.26E-03	96.49	54.05	7.42E-02	-142.25	262.51	5.88E-01	48.45	21.66	2.53E-02	24.99	66.53	7.07E-01
cg09744448	-18.76	6.99	7.27E-03	-23.52	10.67	2.75E-02	-3.06	16.79	8.55E-01	-28.60	19.27	1.38E-01	-16.45	13.55	2.25E-01
cg12519676	-6.12	2.28	7.27E-03	-7.18	3.42	3.58E-02	-0.81	4.46	8.56E-01	-12.45	7.88	1.14E-01	-7.98	4.98	1.09E-01
cg09103234	16.83	6.27	7.27E-03	-0.91	11.45	9.36E-01	17.25	11.19	1.23E-01	24.01	8.20	3.41E-03	30.65	19.53	1.17E-01
cg15829619	-51.53	19.20	7.28E-03	-53.15	25.24	3.52E-02	-56.40	71.36	4.29E-01	-6.62	66.89	9.21E-01	-60.58	37.21	1.03E-01
cg26434278	44.35	16.53	7.28E-03	33.52	55.76	5.48E-01	-87.84	212.70	6.80E-01	45.52	17.96	1.13E-02	57.05	67.68	3.99E-01
cg02648589	8.20	3.06	7.30E-03	11.12	4.08	6.38E-03	7.89	7.56	2.97E-01	8.45	8.84	3.39E-01	-2.24	7.76	7.72E-01
cg27339840	-48.32	18.01	7.30E-03	-66.48	22.71	3.41E-03	-54.15	76.93	4.81E-01	-45.60	67.85	5.02E-01	-1.27	36.35	9.72E-01
cg13338302	-42.91	15.99	7.30E-03	-42.67	39.67	2.82E-01	8.65	253.83	9.73E-01	-51.57	18.63	5.64E-03	20.58	51.43	6.89E-01
cg26927427	11.62	4.33	7.31E-03	11.12	6.15	7.04E-02	22.50	8.67	9.45E-03	-6.42	15.25	6.74E-01	7.85	8.55	3.59E-01
cg08349093	-13.61	5.07	7.32E-03	-14.88	9.47	1.16E-01	-6.83	13.10	6.02E-01	-11.05	9.23	2.31E-01	-19.06	9.94	5.50E-02
cg24254196	-9.96	3.71	7.32E-03	-10.72	7.01	1.26E-01	2.47	11.51	8.30E-01	-11.20	5.04	2.64E-02	-15.64	13.80	2.57E-01
cg07196426	8.95	3.34	7.32E-03	7.50	4.37	8.57E-02	7.31	8.55	3.93E-01	9.97	9.84	3.11E-01	15.48	8.64	7.32E-02
cg00923919	-35.90	13.39	7.33E-03	-54.04	22.22	1.50E-02	7.64	119.24	9.49E-01	-29.29	20.99	1.63E-01	-20.51	28.72	4.75E-01
cg14354895	18.15	6.77	7.33E-03	18.73	9.25	4.28E-02	13.95	27.97	6.18E-01	30.87	24.33	2.05E-01	14.95	11.82	2.06E-01
cg20872692	-20.01	7.46	7.33E-03	-1.91	19.15	9.21E-01	-18.63	34.69	5.91E-01	-23.58	8.76	7.10E-03	-22.90	27.04	3.97E-01
cg19510663	22.62	8.44	7.34E-03	16.69	16.64	3.16E-01	4.50	17.65	7.99E-01	36.46	13.09	5.34E-03	21.70	26.84	4.19E-01
cg11797656	24.79	9.25	7.34E-03	26.36	19.84	1.84E-01	39.75	18.39	3.07E-02	24.16	14.64	9.90E-02	-4.78	25.56	8.52E-01
cg11122493	59.41	22.16	7.35E-03	-14.40	52.63	7.84E-01	239.37	188.10	2.03E-01	65.01	17.61	2.22E-04	101.24	60.73	9.55E-02
cg23616097	10.64	3.97	7.35E-03	13.98	7.40	5.90E-02	12.92	12.38	2.96E-01	8.80	5.43	1.05E-01	7.86	14.44	5.86E-01
cg25240964	-9.28	3.46	7.35E-03	-18.74	8.67	3.06E-02	-10.44	13.56	4.41E-01	-5.84	4.19	1.63E-01	-17.70	11.47	1.23E-01
cg24514412	38.06	14.20	7.36E-03	45.46	21.15	3.16E-02	18.20	45.35	6.88E-01	66.61	38.55	8.40E-02	21.37	25.29	3.98E-01
cg06254729	26.27	9.80	7.36E-03	6.38	19.74	7.47E-01	23.38	51.23	6.48E-01	34.76	12.63	5.92E-03	25.37	28.94	3.81E-01

cg23716696	27.17	10.14	7.36E-03	-9.53	30.78	7.57E-01	28.00	161.10	8.62E-01	31.54	11.14	4.64E-03	33.25	41.62	4.24E-01
cg10913077	-12.32	4.60	7.36E-03	-18.75	7.07	8.01E-03	-10.52	10.70	3.26E-01	-1.71	11.72	8.84E-01	-9.16	9.39	3.29E-01
cg06900494	-11.93	4.45	7.36E-03	-13.07	6.30	3.81E-02	-20.39	9.68	3.52E-02	12.33	18.12	4.96E-01	-8.04	9.30	3.87E-01
cg03883256	-11.84	4.42	7.36E-03	-13.74	6.85	4.50E-02	-5.84	10.24	5.69E-01	-17.32	10.46	9.78E-02	-8.89	9.43	3.46E-01
cg24033042	14.71	5.49	7.37E-03	16.67	8.02	3.78E-02	25.45	13.45	5.84E-02	13.10	16.65	4.31E-01	4.85	10.84	6.55E-01
cg05150697	28.25	10.54	7.37E-03	23.16	19.58	2.37E-01	34.68	19.57	7.63E-02	23.29	19.96	2.43E-01	35.30	28.09	2.09E-01
cg16564824	38.46	14.35	7.38E-03	49.56	21.59	2.17E-02	11.05	40.28	7.84E-01	16.14	39.22	6.81E-01	43.72	26.34	9.69E-02
cg03882967	-9.77	3.65	7.38E-03	-12.84	8.14	1.15E-01	-13.90	13.34	2.97E-01	-6.93	4.64	1.36E-01	-17.40	11.09	1.17E-01
cg26172504	8.92	3.33	7.38E-03	5.05	5.01	3.13E-01	18.98	7.76	1.45E-02	11.63	7.65	1.29E-01	5.38	7.74	4.87E-01
cg00083596	-10.85	4.05	7.38E-03	-24.38	11.27	3.05E-02	-5.89	22.67	7.95E-01	-9.13	4.66	5.00E-02	-7.37	14.09	6.01E-01
cg25815482	7.68	2.87	7.38E-03	1.98	6.24	7.51E-01	0.63	7.58	9.34E-01	11.96	4.05	3.16E-03	8.17	7.53	2.78E-01
cg06297554	18.19	6.79	7.38E-03	33.02	17.48	5.89E-02	-6.65	21.33	7.55E-01	17.82	8.24	3.06E-02	25.77	25.73	3.17E-01
cg13382694	14.92	5.57	7.39E-03	11.37	7.35	1.22E-01	21.22	13.75	1.23E-01	18.51	21.05	3.79E-01	18.85	12.73	1.39E-01
cg18464274	10.29	3.84	7.39E-03	11.37	5.19	2.83E-02	6.31	9.38	5.01E-01	13.53	10.90	2.14E-01	8.23	9.61	3.92E-01
cg16151494	4.89	1.83	7.40E-03	6.01	2.76	2.93E-02	2.02	4.00	6.13E-01	6.90	4.33	1.11E-01	3.46	4.37	4.28E-01
cg17560929	15.60	5.82	7.40E-03	12.83	8.61	1.36E-01	28.07	11.27	1.28E-02	13.58	15.58	3.84E-01	2.49	15.77	8.74E-01
cg05393578	17.72	6.62	7.41E-03	17.08	10.14	9.23E-02	22.67	14.19	1.10E-01	25.76	14.72	8.02E-02	2.05	16.82	9.03E-01
cg15229747	16.13	6.02	7.41E-03	18.85	7.82	1.60E-02	11.33	18.51	5.40E-01	7.35	31.79	8.17E-01	13.16	11.70	2.61E-01
cg15095913	22.64	8.45	7.41E-03	31.73	16.75	5.83E-02	22.79	15.35	1.37E-01	20.16	15.09	1.82E-01	10.27	23.64	6.64E-01
cg20441701	13.70	5.12	7.41E-03	11.97	5.75	3.75E-02	31.80	9.79	1.16E-03	4.96	9.72	6.10E-01	8.64	8.67	3.19E-01
cg16497219	-52.90	19.76	7.41E-03	-66.34	26.50	1.23E-02	-61.74	115.69	5.94E-01	-48.78	42.11	2.47E-01	-17.94	44.73	6.88E-01
cg20312179	-9.42	3.52	7.41E-03	-11.26	7.07	1.11E-01	1.65	13.18	9.00E-01	-8.81	4.85	6.94E-02	-13.61	8.91	1.27E-01
cg02055351	26.10	9.75	7.43E-03	26.63	17.56	1.29E-01	20.13	19.79	3.09E-01	23.18	17.88	1.95E-01	40.25	25.01	1.08E-01
cg03813443	24.19	9.04	7.43E-03	39.89	27.05	1.40E-01	25.85	15.51	9.56E-02	19.24	13.21	1.45E-01	24.26	31.78	4.45E-01
cg09012544	36.95	13.80	7.43E-03	33.90	19.29	7.89E-02	-6.36	68.30	9.26E-01	14.90	41.54	7.20E-01	54.08	23.79	2.30E-02
cg17946266	-12.35	4.61	7.43E-03	-27.37	10.70	1.06E-02	-8.40	20.31	6.79E-01	-7.94	5.62	1.58E-01	-16.66	15.46	2.81E-01
cg09302724	25.28	9.44	7.43E-03	22.31	16.28	1.70E-01	28.51	20.89	1.72E-01	18.23	16.17	2.60E-01	48.50	27.49	7.77E-02
cg02289168	23.67	8.84	7.43E-03	30.62	18.47	9.73E-02	20.92	18.13	2.49E-01	30.98	14.03	2.72E-02	-4.78	24.07	8.43E-01
cg27372015	21.54	8.05	7.44E-03	10.45	18.47	5.71E-01	48.21	26.11	6.49E-02	19.47	9.98	5.10E-02	35.48	31.49	2.60E-01
cg16592713	25.43	9.50	7.44E-03	47.83	34.63	1.67E-01	-53.01	110.98	6.33E-01	24.50	10.22	1.65E-02	19.69	41.35	6.34E-01
cg17769836	-18.17	6.79	7.44E-03	-17.50	10.69	1.02E-01	-16.10	14.04	2.51E-01	-16.64	17.94	3.54E-01	-22.63	14.50	1.19E-01
cg06461306	19.42	7.26	7.45E-03	15.80	9.46	9.50E-02	30.95	17.19	7.17E-02	-18.32	34.50	5.95E-01	28.62	16.68	8.62E-02
cg08961621	-19.71	7.37	7.45E-03	-15.61	14.89	2.94E-01	-17.66	16.48	2.84E-01	-24.24	11.39	3.34E-02	-16.24	19.88	4.14E-01
cg15162330	33.52	12.53	7.46E-03	45.85	17.56	9.05E-03	21.64	44.60	6.28E-01	55.62	43.45	2.01E-01	11.75	21.82	5.90E-01
cg04824189	-13.53	5.06	7.46E-03	-26.70	10.60	1.18E-02	4.48	16.94	7.92E-01	-12.94	7.04	6.62E-02	-7.03	12.34	5.69E-01

cg10616216	-20.70	7.74	7.46E-03	-0.46	17.25	9.79E-01	-4.74	42.09	9.10E-01	-29.60	9.46	1.76E-03	-6.88	24.90	7.82E-01
cg26535834	-15.16	5.67	7.46E-03	2.66	14.08	8.50E-01	-22.88	28.81	4.27E-01	-19.85	6.60	2.65E-03	-1.48	22.59	9.48E-01
cg15033552	-14.38	5.38	7.47E-03	-22.79	13.99	1.03E-01	-21.68	22.81	3.42E-01	-10.99	6.45	8.84E-02	-21.41	16.88	2.05E-01
cg23401624	50.03	18.70	7.48E-03	33.35	51.81	5.20E-01	-28.62	174.54	8.70E-01	46.04	21.03	2.86E-02	142.69	72.12	4.79E-02
cg07731025	-36.72	13.73	7.48E-03	-54.36	48.95	2.67E-01	-75.41	198.29	7.04E-01	-36.74	14.85	1.33E-02	-10.77	55.36	8.46E-01
cg03407631	16.49	6.17	7.50E-03	13.81	15.22	3.64E-01	-16.93	20.77	4.15E-01	22.08	5.92	1.90E-04	14.05	15.70	3.71E-01
cg09781307	-12.62	4.72	7.50E-03	-13.94	7.58	6.59E-02	-14.74	9.94	1.38E-01	-6.46	10.91	5.54E-01	-13.44	10.56	2.03E-01
cg03270036	21.64	8.09	7.50E-03	15.87	18.92	4.02E-01	20.16	18.59	2.78E-01	21.96	11.00	4.58E-02	35.33	27.72	2.02E-01
cg02079034	17.66	6.60	7.51E-03	39.92	16.51	1.56E-02	39.28	35.57	2.70E-01	12.96	7.81	9.69E-02	7.15	22.01	7.45E-01
cg23923495	21.62	8.09	7.52E-03	28.95	10.83	7.53E-03	24.17	24.30	3.20E-01	14.39	35.05	6.81E-01	7.32	15.33	6.33E-01
cg17554636	-19.61	7.33	7.52E-03	-16.90	8.18	3.88E-02	-77.27	45.65	9.05E-02	-36.11	28.90	2.11E-01	-16.01	22.56	4.78E-01
cg11424828	-2.38	0.89	7.52E-03	-3.36	1.27	8.42E-03	-1.26	2.07	5.43E-01	-0.16	2.75	9.54E-01	-2.21	1.89	2.42E-01
cg24018321	-14.97	5.60	7.52E-03	-20.51	8.74	1.89E-02	-8.55	11.50	4.57E-01	-0.73	17.19	9.66E-01	-18.08	11.31	1.10E-01
cg24055349	33.15	12.40	7.52E-03	31.56	34.52	3.61E-01	-24.79	134.96	8.54E-01	32.91	14.07	1.93E-02	43.61	42.57	3.06E-01
cg06800231	-8.11	3.03	7.53E-03	-7.15	4.51	1.13E-01	-14.27	6.44	2.67E-02	-2.46	11.35	8.28E-01	-6.02	6.03	3.18E-01
cg15496388	13.42	5.02	7.53E-03	15.69	6.78	2.06E-02	21.46	12.83	9.43E-02	16.75	14.73	2.56E-01	-2.42	11.81	8.38E-01
cg27158490	-61.44	22.99	7.54E-03	-96.01	39.48	1.50E-02	-418.81	250.01	9.39E-02	-45.08	24.22	6.28E-02	-35.77	62.99	5.70E-01
cg04909834	-27.80	10.41	7.54E-03	-36.60	14.49	1.16E-02	7.42	24.44	7.61E-01	-26.22	28.13	3.51E-01	-40.21	25.50	1.15E-01
cg07970724	-12.12	4.54	7.54E-03	-21.39	8.15	8.64E-03	2.02	21.68	9.26E-01	-8.04	6.03	1.83E-01	-12.83	16.04	4.24E-01
cg16290301	-36.29	13.58	7.55E-03	-27.84	17.61	1.14E-01	-79.62	39.86	4.58E-02	-34.56	49.17	4.82E-01	-36.89	29.45	2.10E-01
cg16678522	7.72	2.89	7.56E-03	7.04	4.07	8.38E-02	13.00	7.25	7.31E-02	12.06	6.31	5.61E-02	-3.22	8.05	6.90E-01
cg06644998	-12.87	4.82	7.56E-03	-12.28	9.33	1.88E-01	-8.79	19.77	6.56E-01	-14.78	6.69	2.71E-02	-9.04	12.23	4.60E-01
cg26271970	-20.34	7.62	7.56E-03	-29.22	10.40	4.96E-03	4.80	16.15	7.66E-01	-33.33	15.77	3.45E-02	-16.53	12.71	1.93E-01
cg21239001	6.12	2.29	7.57E-03	4.92	3.38	1.46E-01	14.01	5.57	1.20E-02	3.51	4.34	4.19E-01	5.55	7.51	4.60E-01
cg06932535	-11.20	4.19	7.57E-03	-23.82	10.28	2.05E-02	-6.47	18.86	7.32E-01	-10.77	5.08	3.40E-02	4.00	13.01	7.59E-01
cg19851563	4.76	1.78	7.57E-03	4.11	2.26	6.90E-02	0.99	4.04	8.06E-01	14.26	7.27	4.99E-02	7.47	4.04	6.45E-02
cg24009118	19.79	7.41	7.57E-03	18.17	11.60	1.17E-01	22.65	15.27	1.38E-01	33.20	17.23	5.40E-02	5.22	17.92	7.71E-01
cg17985656	27.81	10.42	7.57E-03	23.98	16.83	1.54E-01	6.32	33.03	8.48E-01	44.58	21.08	3.44E-02	26.03	19.92	1.91E-01
cg13220109	84.83	31.77	7.57E-03	59.65	82.82	4.71E-01	-126.85	309.96	6.82E-01	102.88	37.01	5.44E-03	15.05	97.72	8.78E-01
cg10407136	-51.98	19.46	7.57E-03	-54.73	24.33	2.45E-02	-41.68	102.85	6.85E-01	-37.72	78.98	6.33E-01	-49.99	37.93	1.88E-01
cg17126555	15.04	5.63	7.58E-03	18.86	9.20	4.02E-02	8.07	11.37	4.78E-01	10.14	10.58	3.38E-01	32.38	18.18	7.49E-02
cg13556511	17.10	6.40	7.58E-03	5.91	24.18	8.07E-01	81.89	64.21	2.02E-01	18.79	7.04	7.61E-03	3.48	21.11	8.69E-01
cg08460464	-12.85	4.81	7.58E-03	-22.30	11.47	5.19E-02	-8.95	17.00	5.99E-01	-8.66	6.04	1.52E-01	-24.67	14.49	8.87E-02
cg13379408	15.56	5.83	7.58E-03	15.29	11.23	1.73E-01	-10.50	25.16	6.76E-01	19.11	7.93	1.60E-02	12.31	15.68	4.33E-01
cg23205454	-14.27	5.34	7.58E-03	-21.52	8.48	1.12E-02	-7.57	11.73	5.19E-01	-15.49	11.85	1.91E-01	-5.23	12.18	6.68E-01

cg18813527	-13.65	5.11	7.60E-03	-22.96	10.22	2.47E-02	-16.17	12.22	1.86E-01	-3.24	8.12	6.90E-01	-21.16	12.08	7.97E-02
cg21556527	-17.81	6.67	7.60E-03	-31.37	12.92	1.52E-02	-8.91	16.51	5.90E-01	-9.88	6.19	1.11E-01	-35.58	16.37	2.97E-02
cg01306688	12.86	4.82	7.61E-03	9.48	7.32	1.96E-01	13.62	9.10	1.35E-01	-0.43	14.72	9.77E-01	27.74	11.36	1.46E-02
cg05337391	37.64	14.10	7.61E-03	41.09	15.72	8.93E-03	-39.28	100.91	6.97E-01	-28.99	83.99	7.30E-01	41.69	36.74	2.57E-01
cg08653904	21.99	8.24	7.61E-03	35.04	22.92	1.26E-01	-0.39	45.35	9.93E-01	19.53	9.37	3.72E-02	36.63	32.25	2.56E-01
cg26373134	18.26	6.84	7.61E-03	39.23	20.26	5.28E-02	-1.85	29.04	9.49E-01	16.24	7.88	3.94E-02	21.33	24.51	3.84E-01
cg13738327	-14.26	5.34	7.61E-03	-13.24	6.87	5.39E-02	-7.30	21.02	7.28E-01	-29.43	13.61	3.06E-02	-7.03	12.73	5.81E-01
cg27242132	8.05	3.02	7.62E-03	10.17	4.98	4.11E-02	4.06	6.14	5.09E-01	11.19	7.18	1.19E-01	6.33	6.50	3.30E-01
cg02408850	19.95	7.48	7.62E-03	22.60	10.95	3.91E-02	17.90	15.23	2.40E-01	31.04	19.29	1.08E-01	3.12	19.77	8.75E-01
cg17661135	-12.22	4.58	7.62E-03	-17.04	8.16	3.67E-02	-21.29	12.20	8.10E-02	-8.45	7.36	2.51E-01	-3.68	11.59	7.51E-01
cg03260991	15.17	5.68	7.62E-03	11.61	16.33	4.77E-01	1.90	19.39	9.22E-01	15.38	6.62	2.02E-02	40.79	24.17	9.15E-02
cg06883279	-9.91	3.71	7.62E-03	-17.16	7.06	1.51E-02	-12.36	16.35	4.50E-01	-6.81	5.04	1.76E-01	-6.42	10.33	5.34E-01
cg14679444	-17.27	6.47	7.62E-03	-23.32	10.91	3.25E-02	1.76	23.93	9.41E-01	-24.54	13.96	7.87E-02	-10.87	10.79	3.14E-01
cg04903759	6.84	2.56	7.62E-03	5.67	3.49	1.05E-01	9.82	6.94	1.57E-01	12.34	5.85	3.49E-02	0.58	7.01	9.34E-01
cg17209280	-14.78	5.54	7.63E-03	-12.75	14.86	3.91E-01	-0.71	24.89	9.77E-01	-14.51	6.49	2.54E-02	-28.82	19.16	1.32E-01
cg05690069	28.66	10.74	7.63E-03	48.07	19.84	1.54E-02	-6.59	41.38	8.73E-01	21.39	15.40	1.65E-01	30.14	27.49	2.73E-01
cg04467093	12.84	4.81	7.64E-03	10.44	7.29	1.52E-01	26.82	11.95	2.48E-02	5.21	10.96	6.35E-01	14.06	10.54	1.82E-01
cg27600265	12.71	4.77	7.65E-03	11.16	7.12	1.17E-01	16.31	9.85	9.78E-02	27.58	17.99	1.25E-01	7.92	9.57	4.08E-01
cg23019936	7.62	2.86	7.65E-03	14.07	5.54	1.12E-02	4.63	7.97	5.61E-01	7.04	4.22	9.48E-02	0.35	7.48	9.63E-01
cg01287514	20.27	7.60	7.65E-03	17.82	9.16	5.17E-02	-7.92	30.27	7.93E-01	74.48	47.18	1.14E-01	29.45	16.09	6.71E-02
cg21626886	54.98	20.62	7.65E-03	30.65	57.52	5.94E-01	180.96	232.29	4.36E-01	67.84	24.26	5.17E-03	4.48	54.80	9.35E-01
cg10476260	-28.87	10.82	7.65E-03	-43.05	37.96	2.57E-01	27.14	145.58	8.52E-01	-24.31	11.85	4.02E-02	-66.67	38.64	8.45E-02
cg04508739	13.70	5.14	7.65E-03	16.97	7.43	2.24E-02	8.22	11.59	4.78E-01	9.23	17.31	5.94E-01	13.32	10.54	2.06E-01
cg25197880	26.44	9.91	7.65E-03	25.59	19.55	1.90E-01	37.34	43.44	3.90E-01	25.48	12.86	4.75E-02	28.70	31.91	3.68E-01
cg02904062	-40.25	15.09	7.66E-03	-34.31	20.04	8.68E-02	5.27	35.87	8.83E-01	-41.77	25.29	9.86E-02	-85.51	31.14	6.03E-03
cg22633819	27.75	10.41	7.66E-03	28.83	17.54	1.00E-01	35.81	19.61	6.78E-02	11.23	20.68	5.87E-01	41.26	30.94	1.82E-01
cg08528000	-20.56	7.71	7.67E-03	-14.39	19.86	4.69E-01	7.63	36.72	8.35E-01	-27.57	8.91	1.97E-03	14.44	26.71	5.89E-01
cg08364970	-36.06	13.52	7.67E-03	-45.70	19.26	1.76E-02	-18.48	92.48	8.42E-01	-51.52	25.00	3.94E-02	10.05	30.77	7.44E-01
cg03765543	27.82	10.44	7.67E-03	41.67	19.05	2.87E-02	6.44	36.34	8.59E-01	35.66	11.46	1.85E-03	-9.79	24.98	6.95E-01
cg24125269	-21.24	7.97	7.68E-03	-44.66	18.92	1.82E-02	3.01	19.89	8.80E-01	-21.17	10.50	4.39E-02	-19.13	24.86	4.42E-01
cg00700455	-18.88	7.08	7.68E-03	-11.41	16.51	4.90E-01	-42.05	21.55	5.11E-02	-16.85	9.27	6.91E-02	-19.35	20.08	3.35E-01
cg07845011	21.83	8.19	7.68E-03	26.47	18.01	1.42E-01	-0.18	19.85	9.93E-01	22.72	11.12	4.10E-02	50.64	28.94	8.01E-02
cg17526887	-20.21	7.58	7.68E-03	-23.60	12.41	5.72E-02	5.99	23.83	8.02E-01	-12.97	19.75	5.11E-01	-26.67	12.32	3.04E-02
cg16567087	40.68	15.26	7.68E-03	39.88	18.87	3.46E-02	18.37	94.16	8.45E-01	-20.44	88.03	8.16E-01	50.86	28.36	7.29E-02
cg00955547	-10.70	4.01	7.68E-03	-13.79	7.61	7.00E-02	-8.65	10.19	3.96E-01	-8.85	6.22	1.55E-01	-12.21	10.35	2.38E-01

cg27160284	13.54	5.08	7.69E-03	12.37	7.39	9.41E-02	22.32	10.51	3.37E-02	16.39	15.12	2.78E-01	3.48	11.95	7.71E-01
cg15382538	18.86	7.08	7.69E-03	27.34	17.68	1.22E-01	3.65	19.72	8.53E-01	18.34	11.60	1.14E-01	21.21	12.16	8.11E-02
cg02084485	-13.25	4.97	7.69E-03	-16.44	14.44	2.55E-01	-4.90	22.84	8.30E-01	-14.45	5.91	1.45E-02	-6.69	13.99	6.33E-01
cg13266951	46.67	17.51	7.69E-03	59.50	24.03	1.33E-02	20.41	53.38	7.02E-01	24.21	47.43	6.10E-01	42.59	36.89	2.48E-01
cg19273253	49.99	18.76	7.69E-03	50.67	28.78	7.83E-02	113.32	49.33	2.16E-02	41.72	55.60	4.53E-01	23.15	33.32	4.87E-01
cg04800681	13.58	5.09	7.70E-03	18.10	7.35	1.37E-02	12.85	14.56	3.77E-01	5.85	12.23	6.32E-01	10.24	10.79	3.43E-01
cg11251728	-9.56	3.59	7.70E-03	-18.42	7.35	1.22E-02	-5.67	9.81	5.63E-01	-7.95	5.44	1.44E-01	-4.93	8.18	5.47E-01
cg01030121	23.53	8.83	7.71E-03	38.97	14.77	8.32E-03	17.64	25.05	4.81E-01	28.27	21.86	1.96E-01	7.87	14.82	5.96E-01
cg04774364	-13.89	5.21	7.71E-03	-22.43	11.21	4.55E-02	-8.43	16.16	6.02E-01	-10.92	6.73	1.04E-01	-20.21	18.50	2.75E-01
cg07632771	-14.26	5.35	7.72E-03	-20.88	7.95	8.60E-03	-11.93	12.58	3.43E-01	-10.38	16.27	5.23E-01	-5.85	10.56	5.80E-01
cg02824817	-16.22	6.09	7.72E-03	-32.48	15.60	3.74E-02	-16.56	26.66	5.35E-01	-10.08	6.18	1.03E-01	-36.98	19.27	5.50E-02
cg14169886	-13.93	5.23	7.72E-03	-18.41	8.85	3.74E-02	-11.67	11.36	3.04E-01	-7.06	10.86	5.16E-01	-16.35	11.48	1.54E-01
cg13600314	-11.72	4.40	7.72E-03	-22.58	11.29	4.54E-02	-24.25	19.56	2.15E-01	-6.65	5.32	2.12E-01	-22.03	13.00	9.03E-02
cg13826452	12.62	4.74	7.72E-03	13.44	7.19	6.15E-02	9.07	10.95	4.07E-01	17.23	11.27	1.26E-01	10.12	10.57	3.38E-01
cg02823066	-12.70	4.77	7.73E-03	-10.32	7.93	1.93E-01	-3.32	10.18	7.45E-01	-19.43	9.75	4.63E-02	-19.99	11.24	7.53E-02
cg23021796	18.04	6.77	7.73E-03	25.71	9.06	4.56E-03	4.42	19.53	8.21E-01	6.59	28.93	8.20E-01	10.48	13.12	4.24E-01
cg01125010	13.37	5.02	7.73E-03	16.00	7.15	2.52E-02	25.15	11.37	2.70E-02	0.81	14.25	9.54E-01	2.56	11.58	8.25E-01
cg18449135	-13.09	4.91	7.74E-03	-17.82	14.48	2.19E-01	-28.95	19.95	1.47E-01	-8.99	5.71	1.15E-01	-31.54	17.06	6.45E-02
cg03906033	17.36	6.52	7.74E-03	27.49	20.84	1.87E-01	69.00	66.80	3.02E-01	16.91	7.17	1.83E-02	0.36	25.48	9.89E-01
cg27273675	14.12	5.30	7.75E-03	15.15	8.53	7.57E-02	7.35	13.10	5.75E-01	19.08	9.95	5.53E-02	9.94	13.04	4.46E-01
cg14312063	7.84	2.94	7.75E-03	6.65	5.21	2.02E-01	8.14	6.07	1.80E-01	4.08	5.16	4.29E-01	20.56	8.48	1.54E-02
cg19834045	-11.82	4.44	7.75E-03	-20.17	8.68	2.01E-02	-3.18	12.27	7.95E-01	-9.14	6.70	1.72E-01	-12.59	10.86	2.47E-01
cg12633355	18.12	6.81	7.76E-03	24.83	16.32	1.28E-01	30.02	35.47	3.97E-01	13.78	8.00	8.50E-02	41.69	26.63	1.17E-01
cg12503292	-34.29	12.88	7.76E-03	-32.46	14.33	2.35E-02	-145.03	87.37	9.69E-02	18.96	63.83	7.66E-01	-43.84	35.74	2.20E-01
cg00530015	-10.84	4.07	7.77E-03	-12.98	8.08	1.08E-01	-15.36	14.14	2.77E-01	-10.47	5.76	6.92E-02	-6.35	10.08	5.29E-01
cg16686960	-17.65	6.63	7.77E-03	-24.96	11.06	2.40E-02	-8.62	14.96	5.64E-01	-28.52	16.54	8.46E-02	-8.47	12.45	4.96E-01
cg19691505	-12.62	4.74	7.77E-03	-13.37	8.52	1.17E-01	-0.16	11.53	9.89E-01	-16.94	8.09	3.62E-02	-14.85	11.26	1.87E-01
cg10676472	-21.62	8.12	7.77E-03	-39.81	18.95	3.56E-02	18.42	34.69	5.95E-01	-18.43	9.89	6.25E-02	-32.99	27.42	2.29E-01
cg06281297	19.38	7.28	7.77E-03	18.53	11.01	9.24E-02	-1.41	21.78	9.48E-01	40.93	21.27	5.43E-02	19.89	12.60	1.15E-01
cg26332560	21.68	8.15	7.78E-03	27.36	13.38	4.09E-02	15.46	21.93	4.81E-01	16.52	16.74	3.24E-01	21.58	16.14	1.81E-01
cg13670911	36.07	13.55	7.78E-03	-14.07	45.61	7.58E-01	-70.73	165.65	6.69E-01	43.10	14.78	3.55E-03	24.19	53.59	6.52E-01
cg25983553	27.21	10.23	7.79E-03	35.08	13.81	1.11E-02	24.19	27.67	3.82E-01	14.35	33.58	6.69E-01	15.03	21.68	4.88E-01
cg16759218	-18.02	6.77	7.79E-03	-26.25	14.88	7.78E-02	23.91	27.31	3.81E-01	-21.47	7.52	4.29E-03	-6.42	20.13	7.50E-01
cg09246897	-9.58	3.60	7.79E-03	-7.93	7.84	3.12E-01	-5.18	12.74	6.84E-01	-8.14	4.65	8.01E-02	-23.51	10.77	2.91E-02
cg09848096	18.94	7.12	7.80E-03	30.28	20.20	1.34E-01	-39.35	35.95	2.74E-01	20.29	8.36	1.52E-02	18.00	21.29	3.98E-01

cg20428133	20.27	7.62	7.80E-03	9.91	15.34	5.18E-01	28.33	14.13	4.50E-02	18.24	13.20	1.67E-01	27.18	21.20	2.00E-01
cg02341197	9.08	3.41	7.80E-03	7.06	4.77	1.39E-01	16.65	7.27	2.19E-02	7.12	10.97	5.16E-01	6.48	8.28	4.34E-01
cg00561891	-75.25	28.29	7.81E-03	-109.39	35.77	2.23E-03	35.49	102.35	7.29E-01	-105.41	46.05	2.21E-02	-16.30	50.79	7.48E-01
cg09807315	26.39	9.92	7.81E-03	43.40	18.45	1.86E-02	17.43	18.63	3.50E-01	14.70	20.29	4.69E-01	28.63	22.89	2.11E-01
cg08836353	13.21	4.97	7.81E-03	22.21	7.48	2.97E-03	9.20	10.90	3.98E-01	-2.33	15.55	8.81E-01	6.98	9.94	4.83E-01
cg27565473	-10.31	3.87	7.81E-03	-8.71	5.94	1.42E-01	-17.20	8.11	3.39E-02	-4.84	12.27	6.93E-01	-8.87	7.81	2.56E-01
cg03962691	20.64	7.76	7.82E-03	34.41	9.32	2.24E-04	19.13	16.08	2.34E-01	23.11	14.80	1.18E-01	2.80	11.31	8.04E-01
cg23925201	-16.31	6.13	7.82E-03	-19.54	9.88	4.79E-02	-10.07	14.31	4.82E-01	-15.13	13.89	2.76E-01	-16.86	12.62	1.81E-01
cg14777244	21.28	8.00	7.82E-03	7.20	13.08	5.82E-01	32.88	14.19	2.05E-02	33.27	19.90	9.46E-02	18.85	20.90	3.67E-01
cg01655355	44.44	16.71	7.83E-03	44.55	20.50	2.98E-02	-34.14	75.46	6.51E-01	13.17	36.20	7.16E-01	82.82	31.07	7.69E-03
cg15295732	-6.95	2.61	7.83E-03	-2.47	3.87	5.23E-01	-10.22	5.96	8.65E-02	-8.64	6.79	2.03E-01	-12.69	5.80	2.87E-02
cg06770532	-17.32	6.51	7.83E-03	-25.18	9.16	5.98E-03	-22.51	12.88	8.04E-02	-21.10	14.52	1.46E-01	2.69	12.24	8.26E-01
cg09547219	-46.39	17.44	7.83E-03	-45.46	21.73	3.64E-02	-139.36	101.94	1.72E-01	-25.12	93.05	7.87E-01	-41.65	32.33	1.98E-01
cg18986154	-21.97	8.26	7.84E-03	-11.19	26.19	6.69E-01	-42.21	122.35	7.30E-01	-22.39	8.99	1.28E-02	-34.14	36.41	3.48E-01
cg03115470	17.79	6.69	7.84E-03	16.61	10.32	1.07E-01	11.42	12.53	3.62E-01	10.28	17.18	5.50E-01	41.98	17.71	1.78E-02
cg24100636	17.47	6.57	7.84E-03	28.95	12.03	1.61E-02	-2.16	17.07	8.99E-01	25.56	11.89	3.15E-02	9.06	11.22	4.19E-01
cg19176696	38.20	14.37	7.84E-03	27.26	19.96	1.72E-01	31.96	19.31	9.78E-02	17.76	19.04	3.51E-01	92.71	26.74	5.27E-04
cg04449108	-33.54	12.62	7.84E-03	-19.12	23.47	4.15E-01	-81.68	61.98	1.88E-01	-39.84	17.17	2.04E-02	-24.12	34.97	4.90E-01
cg09815911	43.78	16.47	7.85E-03	27.82	31.95	3.84E-01	-52.39	210.93	8.04E-01	55.18	20.95	8.43E-03	23.65	49.56	6.33E-01
cg05389024	-12.62	4.75	7.85E-03	-7.38	10.26	4.72E-01	-2.98	22.72	8.96E-01	-14.38	5.80	1.31E-02	-17.63	17.69	3.19E-01
cg04966888	26.83	10.09	7.85E-03	19.83	14.96	1.85E-01	40.24	23.00	8.01E-02	20.45	27.47	4.57E-01	33.53	21.63	1.21E-01
cg23940467	-40.57	15.26	7.85E-03	-9.37	49.87	8.51E-01	-204.10	195.81	2.97E-01	-41.37	16.69	1.32E-02	-60.12	60.12	3.17E-01
cg18726079	-14.21	5.35	7.85E-03	-21.83	13.42	1.04E-01	-8.16	23.90	7.33E-01	-11.11	6.41	8.30E-02	-27.23	17.27	1.15E-01
cg25287198	9.96	3.75	7.86E-03	6.11	4.67	1.91E-01	17.02	6.67	1.07E-02	22.69	11.86	5.58E-02	3.74	7.54	6.20E-01
cg26545143	17.20	6.47	7.86E-03	28.25	14.58	5.26E-02	31.37	33.18	3.44E-01	10.14	8.87	2.53E-01	21.65	13.40	1.06E-01
cg14685948	-18.25	6.87	7.87E-03	-18.45	9.81	5.98E-02	-20.28	16.56	2.21E-01	-5.86	18.29	7.49E-01	-24.84	15.48	1.09E-01
cg26249100	15.73	5.92	7.87E-03	26.28	14.94	7.85E-02	9.12	18.47	6.22E-01	13.04	7.15	6.80E-02	31.49	25.32	2.14E-01
cg06747432	23.67	8.91	7.87E-03	50.87	35.60	1.53E-01	27.57	150.83	8.55E-01	23.35	9.47	1.37E-02	-5.55	40.32	8.91E-01
cg05392265	15.99	6.02	7.87E-03	16.00	9.78	1.02E-01	13.94	13.72	3.10E-01	18.42	12.75	1.48E-01	15.25	13.25	2.50E-01
cg13644715	20.09	7.56	7.89E-03	28.31	12.05	1.88E-02	5.49	14.28	7.01E-01	15.82	17.00	3.52E-01	33.39	21.16	1.15E-01
cg17853642	12.28	4.62	7.89E-03	18.62	6.97	7.57E-03	5.46	10.67	6.09E-01	12.31	10.77	2.53E-01	4.27	10.64	6.88E-01
cg03431155	17.44	6.56	7.89E-03	13.94	17.29	4.20E-01	27.95	22.67	2.18E-01	16.70	7.75	3.12E-02	20.07	27.80	4.70E-01
cg02541210	30.91	11.63	7.89E-03	52.69	17.91	3.25E-03	36.37	19.48	6.19E-02	24.01	19.24	2.12E-01	-10.36	27.83	7.10E-01
cg09645699	19.87	7.48	7.89E-03	31.48	20.41	1.23E-01	10.25	21.89	6.40E-01	16.90	9.04	6.16E-02	44.40	29.33	1.30E-01
cg14948822	-15.00	5.64	7.89E-03	-18.10	14.13	2.00E-01	-10.52	25.79	6.83E-01	-15.29	6.69	2.24E-02	-8.96	19.76	6.50E-01

cg22572476	-5.52	2.08	7.89E-03	-5.78	2.59	2.59E-02	-4.66	4.31	2.80E-01	-13.34	5.17	9.88E-03	-1.01	3.86	7.94E-01
cg01145396	20.41	7.68	7.90E-03	19.67	12.95	1.29E-01	42.32	16.43	1.00E-02	6.25	14.07	6.57E-01	18.09	21.22	3.94E-01
cg22860172	16.21	6.10	7.90E-03	19.50	8.71	2.52E-02	14.16	12.52	2.58E-01	28.51	19.57	1.45E-01	2.88	14.61	8.44E-01
cg13713821	-10.35	3.90	7.90E-03	-13.01	8.40	1.21E-01	-6.40	10.50	5.42E-01	-12.01	5.80	3.84E-02	-6.38	8.81	4.69E-01
cg15714041	-56.37	21.22	7.90E-03	-50.75	24.67	3.97E-02	-71.42	106.52	5.03E-01	0.31	69.71	9.97E-01	-125.48	59.42	3.47E-02
cg05872923	8.07	3.04	7.91E-03	7.86	3.84	4.08E-02	7.33	7.23	3.11E-01	0.34	14.75	9.81E-01	11.88	7.71	1.23E-01
cg02282774	22.73	8.56	7.91E-03	20.77	10.62	5.06E-02	54.50	29.61	6.56E-02	5.33	35.59	8.81E-01	20.95	18.71	2.63E-01
cg13477182	-35.95	13.54	7.91E-03	-25.53	38.02	5.02E-01	38.01	202.77	8.51E-01	-40.97	15.08	6.60E-03	1.97	53.90	9.71E-01
cg16437021	10.43	3.93	7.92E-03	12.08	5.82	3.79E-02	14.47	8.87	1.03E-01	9.46	9.09	2.98E-01	2.00	9.75	8.38E-01
cg07529715	-8.94	3.37	7.92E-03	-11.51	5.33	3.09E-02	-2.32	8.45	7.83E-01	0.38	9.13	9.67E-01	-13.17	6.09	3.04E-02
cg18259342	8.15	3.07	7.92E-03	8.26	3.96	3.70E-02	1.52	6.93	8.27E-01	15.21	15.96	3.41E-01	14.13	7.57	6.22E-02
cg20300500	-15.16	5.71	7.92E-03	-17.93	8.51	3.52E-02	-7.89	13.02	5.44E-01	-24.19	16.19	1.35E-01	-11.01	11.81	3.51E-01
cg01411921	17.54	6.61	7.93E-03	21.74	8.15	7.61E-03	28.89	23.31	2.15E-01	-11.38	38.93	7.70E-01	5.35	13.70	6.96E-01
cg23610453	20.45	7.70	7.93E-03	37.65	15.03	1.22E-02	7.47	18.90	6.93E-01	11.81	13.35	3.76E-01	22.59	15.76	1.52E-01
cg27441225	18.02	6.79	7.93E-03	-0.36	17.74	9.84E-01	18.29	61.40	7.66E-01	24.09	7.72	1.81E-03	-11.02	25.88	6.70E-01
cg08084339	-8.80	3.32	7.93E-03	-6.55	6.09	2.82E-01	-3.99	9.75	6.83E-01	-7.70	4.61	9.48E-02	-25.23	10.13	1.28E-02
cg23636324	31.10	11.72	7.94E-03	32.71	36.83	3.74E-01	68.13	112.12	5.43E-01	29.85	13.12	2.29E-02	35.88	38.93	3.57E-01
cg12537405	4.29	1.62	7.94E-03	3.34	2.10	1.11E-01	5.40	3.32	1.04E-01	-3.12	5.66	5.82E-01	8.00	3.32	1.58E-02
cg10300188	9.59	3.61	7.94E-03	6.52	5.16	2.06E-01	16.39	8.91	6.59E-02	16.05	8.98	7.38E-02	6.01	8.46	4.78E-01
cg02081019	22.78	8.58	7.95E-03	59.03	26.99	2.88E-02	21.18	74.25	7.75E-01	18.52	9.58	5.34E-02	20.13	29.71	4.98E-01
cg09221932	44.55	16.79	7.95E-03	55.63	21.41	9.38E-03	-54.20	77.83	4.86E-01	48.01	56.88	3.99E-01	34.58	33.46	3.01E-01
cg07207286	25.92	9.77	7.95E-03	-1.96	27.29	9.43E-01	-18.63	95.91	8.46E-01	27.13	11.03	1.39E-02	65.74	35.08	6.09E-02
cg12551957	-12.46	4.70	7.96E-03	-15.64	6.57	1.73E-02	-19.10	11.51	9.72E-02	-10.95	14.14	4.39E-01	-0.39	10.19	9.69E-01
cg07959068	19.12	7.20	7.96E-03	3.03	19.20	8.75E-01	17.94	25.40	4.80E-01	22.11	8.43	8.69E-03	22.69	32.90	4.90E-01
cg18949666	8.87	3.34	7.97E-03	9.39	4.58	4.02E-02	13.08	7.26	7.17E-02	10.70	10.59	3.12E-01	0.15	8.50	9.86E-01
cg15302350	-12.69	4.78	7.97E-03	-19.89	7.55	8.45E-03	-9.14	12.74	4.73E-01	-12.69	12.79	3.21E-01	-5.19	8.48	5.40E-01
cg20649017	18.19	6.86	7.97E-03	25.82	13.45	5.49E-02	24.63	18.91	1.93E-01	12.14	11.10	2.74E-01	15.90	14.38	2.69E-01
cg24921186	13.91	5.24	7.97E-03	2.79	12.26	8.20E-01	-4.42	32.01	8.90E-01	16.70	6.11	6.30E-03	22.36	22.25	3.15E-01
cg00030423	32.42	12.22	7.97E-03	29.30	46.49	5.28E-01	56.13	203.62	7.83E-01	33.22	13.07	1.11E-02	21.88	52.87	6.79E-01
cg13355704	15.97	6.02	7.98E-03	16.49	8.01	3.97E-02	31.75	15.59	4.17E-02	7.70	21.99	7.26E-01	6.41	13.10	6.25E-01
cg17621580	22.75	8.58	7.98E-03	25.20	12.73	4.78E-02	18.31	18.90	3.33E-01	20.52	20.32	3.12E-01	23.99	21.28	2.60E-01
cg27157575	23.25	8.76	7.98E-03	9.61	15.72	5.41E-01	5.60	20.56	7.85E-01	40.76	15.51	8.59E-03	33.09	20.16	1.01E-01
cg09832551	37.79	14.25	7.98E-03	36.77	40.85	3.68E-01	79.47	197.46	6.87E-01	44.62	16.22	5.94E-03	-14.96	44.70	7.38E-01
cg01196744	-31.65	11.93	7.98E-03	-47.41	20.50	2.07E-02	-1.84	30.97	9.53E-01	-31.67	23.78	1.83E-01	-28.13	23.35	2.28E-01
cg09597638	7.92	2.99	7.98E-03	7.98	3.74	3.27E-02	7.77	7.10	2.74E-01	11.58	10.35	2.63E-01	4.80	9.41	6.10E-01

cg27341636	-5.33	2.01	7.99E-03	-5.33	3.32	1.09E-01	-4.49	4.60	3.29E-01	-7.25	3.77	5.43E-02	-2.91	5.06	5.65E-01
cg08635931	18.77	7.08	7.99E-03	16.53	8.59	5.42E-02	60.12	43.63	1.68E-01	38.39	29.71	1.96E-01	15.93	14.52	2.73E-01
cg20621765	-12.20	4.60	7.99E-03	-15.43	10.06	1.25E-01	-29.48	18.48	1.11E-01	-9.80	5.83	9.24E-02	-9.84	14.19	4.88E-01
cg00449728	-10.73	4.05	7.99E-03	-16.12	10.94	1.41E-01	-31.25	19.55	1.10E-01	-8.38	4.70	7.48E-02	-12.33	14.33	3.89E-01
cg27280396	15.42	5.81	8.00E-03	15.26	7.80	5.05E-02	14.23	28.20	6.14E-01	20.01	13.66	1.43E-01	12.30	12.36	3.20E-01
cg05046721	-9.48	3.57	8.00E-03	-12.07	8.93	1.77E-01	-10.57	13.14	4.21E-01	-9.33	4.47	3.69E-02	-6.33	10.08	5.30E-01
cg01313977	24.12	9.09	8.01E-03	36.20	16.99	3.31E-02	6.76	27.19	8.04E-01	34.09	15.16	2.45E-02	2.97	18.50	8.72E-01
cg13363640	15.39	5.80	8.01E-03	14.77	12.04	2.20E-01	-9.97	26.01	7.01E-01	17.95	7.66	1.91E-02	14.93	15.28	3.28E-01
cg15843825	-14.71	5.55	8.02E-03	-21.74	8.11	7.32E-03	-8.71	14.25	5.41E-01	7.08	15.36	6.45E-01	-16.56	11.11	1.36E-01
cg04599403	21.75	8.20	8.02E-03	33.63	12.72	8.18E-03	23.96	20.72	2.48E-01	36.48	25.68	1.55E-01	3.22	13.51	8.12E-01
cg08750510	-6.38	2.41	8.02E-03	-6.37	4.18	1.28E-01	-10.03	6.09	9.99E-02	-4.05	3.97	3.07E-01	-8.45	6.36	1.84E-01
cg13331691	-24.77	9.35	8.03E-03	-9.22	16.59	5.78E-01	-5.96	34.91	8.64E-01	-36.66	13.54	6.79E-03	-29.41	25.46	2.48E-01
cg00228891	-11.75	4.43	8.03E-03	-16.45	6.65	1.34E-02	-0.98	9.56	9.19E-01	-22.11	11.35	5.14E-02	-5.27	9.77	5.90E-01
cg23692680	20.32	7.66	8.03E-03	29.78	19.42	1.25E-01	37.72	41.97	3.69E-01	14.75	8.86	9.60E-02	54.36	30.73	7.68E-02
cg18382341	-67.07	25.30	8.04E-03	-22.53	54.60	6.80E-01	-247.57	206.51	2.31E-01	-73.11	31.93	2.20E-02	-88.62	67.11	1.87E-01
cg25806932	-9.03	3.41	8.04E-03	-14.61	6.13	1.72E-02	-12.80	9.21	1.65E-01	-7.04	5.39	1.91E-01	0.31	8.66	9.72E-01
cg23277389	-22.32	8.42	8.04E-03	-23.75	13.04	6.86E-02	-32.30	20.73	1.19E-01	-24.43	17.41	1.61E-01	-7.46	19.65	7.04E-01
cg15717558	32.91	12.42	8.04E-03	29.03	13.38	3.00E-02	55.08	77.29	4.76E-01	132.48	91.31	1.47E-01	42.72	40.42	2.91E-01
cg08457158	-14.31	5.40	8.05E-03	-19.90	7.33	6.61E-03	-16.92	14.99	2.59E-01	-7.07	15.53	6.49E-01	-2.18	11.90	8.54E-01
cg21584710	18.80	7.09	8.05E-03	32.09	11.35	4.69E-03	11.18	13.78	4.17E-01	-1.13	17.71	9.49E-01	18.92	16.55	2.53E-01
cg02144516	14.39	5.43	8.05E-03	17.67	7.63	2.06E-02	20.18	11.60	8.18E-02	19.33	16.88	2.52E-01	-5.69	13.12	6.64E-01
cg06217267	15.24	5.75	8.05E-03	27.94	13.76	4.23E-02	13.12	11.14	2.39E-01	15.41	8.15	5.87E-02	-13.13	23.21	5.72E-01
cg01381934	8.25	3.11	8.05E-03	7.93	4.14	5.54E-02	12.25	6.74	6.89E-02	20.62	12.22	9.15E-02	-1.26	7.91	8.74E-01
cg12267786	-32.71	12.34	8.05E-03	-36.13	17.23	3.60E-02	10.94	40.58	7.87E-01	-20.09	27.39	4.63E-01	-58.03	28.22	3.97E-02
cg03044471	18.52	6.99	8.05E-03	15.24	9.22	9.83E-02	41.72	15.85	8.48E-03	3.77	27.75	8.92E-01	8.42	17.09	6.22E-01
cg07117402	18.73	7.07	8.05E-03	11.21	20.75	5.89E-01	40.86	23.94	8.78E-02	15.28	8.34	6.71E-02	36.65	25.12	1.45E-01
cg12107247	-12.33	4.65	8.05E-03	-21.20	10.66	4.67E-02	-3.58	12.81	7.80E-01	-13.97	6.57	3.34E-02	-4.57	11.13	6.81E-01
cg07477602	7.68	2.90	8.05E-03	8.48	3.90	2.95E-02	10.45	7.76	1.78E-01	9.12	8.24	2.68E-01	2.19	6.78	7.47E-01
cg14456034	-30.75	11.61	8.06E-03	-35.40	20.31	8.14E-02	-14.70	45.03	7.44E-01	-29.07	16.93	8.59E-02	-33.23	31.34	2.89E-01
cg20544552	-6.24	2.36	8.06E-03	-6.11	3.35	6.81E-02	-9.93	5.16	5.42E-02	4.63	12.30	7.07E-01	-5.07	4.62	2.72E-01
cg14074486	16.38	6.18	8.06E-03	17.42	9.40	6.37E-02	25.96	13.82	6.03E-02	5.08	13.54	7.08E-01	16.29	15.52	2.94E-01
cg21892295	8.02	3.03	8.06E-03	11.61	4.12	4.86E-03	2.57	7.36	7.27E-01	10.26	7.62	1.78E-01	-2.27	8.31	7.85E-01
cg15082992	-8.85	3.34	8.07E-03	-7.09	5.91	2.31E-01	-11.00	6.70	1.01E-01	-8.21	7.10	2.47E-01	-9.66	7.28	1.84E-01
cg08698477	-19.72	7.44	8.07E-03	-22.14	15.91	1.64E-01	-32.95	19.49	9.10E-02	-19.14	10.26	6.21E-02	0.02	22.54	9.99E-01
cg20837456	10.68	4.03	8.07E-03	17.93	10.80	9.68E-02	11.43	25.23	6.51E-01	11.35	4.64	1.45E-02	-8.26	14.18	5.60E-01

cg01477861	-7.73	2.92	8.07E-03	-7.70	4.10	6.00E-02	-11.54	6.00	5.45E-02	2.44	10.75	8.20E-01	-6.96	6.82	3.08E-01
cg10301588	20.52	7.75	8.08E-03	25.74	14.93	8.48E-02	13.30	12.88	3.02E-01	13.85	16.07	3.89E-01	40.58	20.91	5.23E-02
cg10111335	-11.02	4.16	8.08E-03	-15.02	6.54	2.16E-02	-10.71	10.74	3.19E-01	-1.48	8.60	8.64E-01	-14.12	9.05	1.19E-01
cg22541001	-9.35	3.53	8.08E-03	-10.89	4.48	1.51E-02	0.74	6.86	9.14E-01	-19.97	9.11	2.84E-02	-10.00	6.89	1.47E-01
cg18843856	-12.17	4.60	8.09E-03	-25.44	9.90	1.02E-02	-7.56	17.04	6.57E-01	-8.13	5.97	1.73E-01	-11.07	13.34	4.07E-01
cg00693401	23.68	8.94	8.09E-03	18.05	23.81	4.48E-01	6.25	126.10	9.60E-01	25.67	10.01	1.04E-02	11.16	37.72	7.67E-01
cg11465939	12.56	4.74	8.09E-03	11.62	6.66	8.12E-02	13.89	10.71	1.95E-01	33.49	17.05	4.95E-02	6.18	10.13	5.42E-01
cg26328954	-13.08	4.94	8.09E-03	-11.01	7.65	1.50E-01	-18.70	11.12	9.25E-02	-11.95	10.90	2.73E-01	-13.00	11.64	2.64E-01
cg08034505	-12.71	4.80	8.10E-03	-8.40	10.04	4.02E-01	-1.99	15.18	8.95E-01	-14.66	6.67	2.79E-02	-19.48	12.25	1.12E-01
cg26875852	-6.60	2.49	8.11E-03	-6.15	4.31	1.54E-01	-11.74	5.30	2.67E-02	-1.51	5.03	7.65E-01	-7.90	5.57	1.56E-01
cg07628705	13.09	4.94	8.11E-03	12.84	7.73	9.70E-02	20.59	11.62	7.64E-02	20.12	10.66	5.91E-02	-1.08	11.19	9.23E-01
cg00811382	15.93	6.02	8.11E-03	9.88	11.80	4.02E-01	34.11	29.02	2.40E-01	15.14	8.30	6.82E-02	22.92	14.50	1.14E-01
cg15907944	10.07	3.80	8.11E-03	14.51	5.61	9.73E-03	8.26	8.27	3.18E-01	6.52	14.32	6.49E-01	4.63	7.48	5.36E-01
cg05511148	-12.58	4.75	8.12E-03	-17.12	9.99	8.66E-02	-12.59	19.36	5.15E-01	-13.80	6.18	2.56E-02	1.77	13.62	8.97E-01
cg12668216	-20.98	7.93	8.12E-03	-9.47	15.19	5.33E-01	-50.14	40.44	2.15E-01	-25.49	10.06	1.13E-02	-9.49	30.33	7.54E-01
cg00924143	15.70	5.93	8.12E-03	31.15	10.77	3.83E-03	8.50	14.73	5.64E-01	11.83	12.44	3.42E-01	7.33	10.57	4.88E-01
cg06296773	18.51	6.99	8.13E-03	41.25	19.52	3.46E-02	29.34	28.13	2.97E-01	13.62	8.00	8.87E-02	21.76	32.62	5.05E-01
cg09453252	26.45	9.99	8.13E-03	3.82	19.13	8.42E-01	13.65	43.30	7.53E-01	33.46	13.12	1.08E-02	56.23	32.66	8.52E-02
cg14252617	20.43	7.72	8.13E-03	2.53	16.59	8.79E-01	13.67	19.57	4.85E-01	25.12	10.73	1.92E-02	43.09	23.24	6.37E-02
cg00063291	-15.57	5.88	8.13E-03	-7.31	12.98	5.73E-01	0.68	17.53	9.69E-01	-18.80	7.61	1.34E-02	-34.74	20.39	8.84E-02
cg21125190	-12.64	4.78	8.14E-03	-18.10	10.02	7.10E-02	-26.09	23.01	2.57E-01	-12.27	6.21	4.83E-02	-1.07	12.86	9.34E-01
cg08399230	21.59	8.16	8.14E-03	11.56	16.07	4.72E-01	7.49	18.71	6.89E-01	38.09	13.06	3.54E-03	14.35	20.29	4.79E-01
cg12090001	37.43	14.14	8.14E-03	36.61	18.09	4.30E-02	36.21	81.34	6.56E-01	5.26	48.53	9.14E-01	49.37	27.04	6.79E-02
cg08466256	-9.15	3.46	8.14E-03	-11.09	6.94	1.10E-01	-2.82	8.44	7.38E-01	-9.56	5.46	8.00E-02	-11.47	8.11	1.58E-01
cg26573518	-37.28	14.09	8.15E-03	-67.13	17.58	1.34E-04	-21.88	30.95	4.80E-01	-13.26	16.24	4.14E-01	-42.42	23.89	7.57E-02
cg09776094	40.81	15.42	8.15E-03	44.29	21.12	3.60E-02	81.26	29.73	6.27E-03	15.78	36.30	6.64E-01	5.76	33.11	8.62E-01
cg05356848	-18.29	6.91	8.16E-03	-3.25	19.36	8.67E-01	45.90	57.39	4.24E-01	-20.86	8.97	2.00E-02	-23.33	13.48	8.35E-02
cg14913512	-8.83	3.34	8.16E-03	-14.51	5.74	1.15E-02	-7.57	7.86	3.36E-01	-6.17	6.12	3.13E-01	-3.91	7.81	6.17E-01
cg04478875	-11.45	4.33	8.16E-03	-13.41	11.57	2.47E-01	-4.49	17.73	8.00E-01	-11.67	5.23	2.57E-02	-11.31	12.67	3.72E-01
cg26856578	-9.96	3.77	8.17E-03	-15.64	7.92	4.83E-02	0.23	10.97	9.83E-01	-10.04	5.08	4.82E-02	-8.80	11.57	4.47E-01
cg14101357	11.37	4.30	8.17E-03	9.90	8.81	2.61E-01	4.60	19.85	8.17E-01	11.66	5.35	2.94E-02	18.28	16.26	2.61E-01
cg00300039	-33.04	12.49	8.17E-03	-87.86	42.45	3.85E-02	-250.26	212.56	2.39E-01	-26.83	13.63	4.90E-02	-29.01	47.18	5.39E-01
cg18617679	43.29	16.37	8.17E-03	45.22	19.93	2.33E-02	-64.65	95.30	4.98E-01	28.38	64.14	6.58E-01	55.66	34.06	1.02E-01
cg13436164	13.44	5.08	8.17E-03	11.00	7.79	1.58E-01	15.79	10.52	1.33E-01	8.21	14.94	5.83E-01	18.26	10.68	8.74E-02
cg09387970	10.26	3.88	8.17E-03	4.78	5.32	3.68E-01	5.83	7.18	4.17E-01	22.84	8.81	9.51E-03	14.36	7.22	4.66E-02

cg12497870	21.27	8.04	8.17E-03	22.92	13.47	8.89E-02	16.01	17.51	3.60E-01	12.76	10.45	2.22E-01	62.38	24.55	1.10E-02
cg25370869	22.08	8.35	8.17E-03	11.54	17.85	5.18E-01	11.69	17.82	5.12E-01	34.54	12.43	5.45E-03	12.67	25.07	6.13E-01
cg19714865	26.22	9.92	8.18E-03	28.98	21.69	1.82E-01	13.56	28.59	6.35E-01	25.22	13.24	5.67E-02	39.98	29.95	1.82E-01
cg06943309	-10.24	3.87	8.18E-03	-12.64	9.30	1.74E-01	-2.51	13.24	8.50E-01	-7.93	4.89	1.05E-01	-25.05	11.45	2.87E-02
cg11232245	18.23	6.89	8.18E-03	29.71	16.59	7.34E-02	29.82	31.19	3.39E-01	17.71	8.36	3.41E-02	-4.04	21.95	8.54E-01
cg03288922	-5.37	2.03	8.18E-03	-5.05	3.07	1.00E-01	-4.72	4.27	2.69E-01	-1.56	5.55	7.79E-01	-9.25	4.49	3.93E-02
cg02665037	15.00	5.67	8.19E-03	18.82	6.69	4.89E-03	23.55	12.13	5.22E-02	20.36	10.39	5.00E-02	-3.20	10.15	7.53E-01
cg14682005	12.92	4.89	8.19E-03	14.22	6.62	3.16E-02	9.48	11.82	4.23E-01	24.82	15.98	1.20E-01	6.44	11.21	5.66E-01
cg19495308	5.57	2.11	8.20E-03	6.36	2.97	3.24E-02	7.17	4.05	7.63E-02	-0.32	5.84	9.56E-01	4.90	6.79	4.70E-01
cg21550504	23.32	8.82	8.20E-03	18.03	15.63	2.49E-01	22.90	16.00	1.52E-01	21.38	18.79	2.55E-01	37.64	22.28	9.12E-02
cg06339090	-11.65	4.41	8.21E-03	-16.68	11.47	1.46E-01	-1.65	16.52	9.20E-01	-10.61	5.35	4.74E-02	-18.22	13.75	1.85E-01
cg17265994	12.07	4.57	8.21E-03	12.50	6.48	5.37E-02	10.62	9.16	2.46E-01	10.59	18.31	5.63E-01	13.30	10.40	2.01E-01
cg00808305	52.07	19.70	8.21E-03	45.66	22.38	4.14E-02	2.46	74.98	9.74E-01	137.75	52.54	8.74E-03	35.09	35.87	3.28E-01
cg08731067	16.96	6.41	8.21E-03	-5.23	16.83	7.56E-01	21.30	15.16	1.60E-01	20.85	8.24	1.14E-02	18.17	24.31	4.55E-01
cg22709144	-12.35	4.67	8.22E-03	-21.70	11.65	6.25E-02	-3.60	20.69	8.62E-01	-9.84	5.66	8.18E-02	-18.48	14.34	1.98E-01
cg21648012	-24.66	9.33	8.22E-03	-48.78	15.40	1.54E-03	-8.50	25.88	7.43E-01	-20.78	10.77	5.37E-02	-4.57	22.26	8.37E-01
cg23326197	-13.50	5.11	8.22E-03	-17.18	7.49	2.18E-02	-4.21	11.00	7.02E-01	-6.26	12.90	6.27E-01	-22.25	12.67	7.91E-02
cg09480647	20.25	7.66	8.22E-03	15.23	13.62	2.63E-01	14.85	13.45	2.69E-01	17.28	18.09	3.39E-01	41.85	18.09	2.07E-02
cg09347134	5.55	2.10	8.22E-03	3.23	2.79	2.47E-01	11.96	4.94	1.55E-02	10.32	7.87	1.90E-01	4.54	4.93	3.58E-01
cg13085338	-39.91	15.10	8.22E-03	-113.69	57.89	4.95E-02	-227.05	242.08	3.48E-01	-35.45	16.44	3.10E-02	-16.21	52.09	7.56E-01
cg11019743	-18.78	7.11	8.23E-03	-14.85	21.36	4.87E-01	7.39	19.67	7.07E-01	-25.91	8.53	2.37E-03	-1.53	28.06	9.56E-01
cg07103673	-8.89	3.37	8.23E-03	-2.58	7.13	7.17E-01	-4.79	10.16	6.38E-01	-9.00	4.23	3.35E-02	-22.93	9.49	1.56E-02
cg16847428	7.10	2.69	8.23E-03	10.16	3.65	5.36E-03	4.32	6.21	4.86E-01	6.19	6.88	3.69E-01	-1.41	7.85	8.58E-01
cg00039489	-9.82	3.72	8.23E-03	-18.54	8.45	2.82E-02	-4.95	21.28	8.16E-01	-9.11	4.56	4.58E-02	-0.34	11.07	9.76E-01
cg11859087	18.97	7.18	8.23E-03	17.30	11.96	1.48E-01	14.74	13.36	2.70E-01	11.79	17.98	5.12E-01	34.57	16.44	3.55E-02
cg20023783	12.33	4.67	8.24E-03	16.12	6.69	1.60E-02	11.46	9.53	2.29E-01	19.91	14.97	1.84E-01	-1.06	11.10	9.24E-01
cg26464796	7.63	2.89	8.24E-03	10.48	5.02	3.67E-02	13.87	6.86	4.32E-02	5.84	4.35	1.80E-01	-4.35	8.93	6.26E-01
cg08682544	32.80	12.41	8.24E-03	29.06	30.03	3.33E-01	152.31	140.56	2.79E-01	26.54	14.39	6.52E-02	89.21	44.63	4.56E-02
cg04939302	-8.73	3.30	8.24E-03	-13.39	7.49	7.38E-02	-4.19	9.05	6.43E-01	-9.72	4.49	3.05E-02	-2.33	9.15	7.99E-01
cg18886274	-10.62	4.02	8.25E-03	-16.92	9.27	6.79E-02	-9.48	15.59	5.43E-01	-7.01	4.98	1.60E-01	-23.67	13.05	6.97E-02
cg03073218	-9.97	3.77	8.26E-03	-18.61	8.34	2.56E-02	-10.32	11.52	3.71E-01	-7.57	5.00	1.30E-01	-6.21	10.99	5.72E-01
cg00387323	-56.66	21.45	8.26E-03	-36.60	27.38	1.81E-01	-165.97	117.62	1.58E-01	-69.74	101.49	4.92E-01	-82.91	38.64	3.19E-02
cg24108508	10.81	4.09	8.26E-03	6.90	7.49	3.57E-01	10.42	11.11	3.48E-01	12.61	5.94	3.37E-02	14.90	13.63	2.75E-01
cg04050867	20.37	7.71	8.26E-03	30.64	17.70	8.33E-02	13.26	21.21	5.32E-01	18.91	12.75	1.38E-01	18.83	13.80	1.72E-01
cg18376497	9.67	3.66	8.27E-03	8.75	4.75	6.55E-02	8.75	8.45	3.00E-01	26.06	17.79	1.43E-01	9.81	8.73	2.61E-01

cg15107554	-21.92	8.30	8.27E-03	-30.85	20.84	1.39E-01	-3.23	33.91	9.24E-01	-16.42	9.93	9.83E-02	-64.43	28.76	2.51E-02
cg05158854	-9.88	3.74	8.27E-03	-14.70	7.16	4.00E-02	0.12	13.04	9.93E-01	-6.17	5.15	2.31E-01	-22.20	10.89	4.14E-02
cg03893446	27.20	10.30	8.27E-03	29.33	15.38	5.66E-02	38.87	28.28	1.69E-01	25.03	20.66	2.26E-01	15.68	24.96	5.30E-01
cg01385708	20.45	7.74	8.28E-03	30.05	12.90	1.99E-02	15.19	14.79	3.04E-01	9.50	16.11	5.55E-01	24.30	21.14	2.50E-01
cg01913577	-11.46	4.34	8.28E-03	-18.34	8.03	2.24E-02	-9.43	10.08	3.50E-01	-6.91	7.10	3.30E-01	-11.95	11.25	2.88E-01
cg26703534	17.49	6.62	8.28E-03	16.70	9.55	8.04E-02	8.15	12.76	5.23E-01	39.28	21.81	7.17E-02	23.06	16.65	1.66E-01
cg23934927	26.68	10.10	8.28E-03	23.21	25.34	3.60E-01	23.10	67.02	7.30E-01	29.59	12.15	1.49E-02	15.78	28.40	5.79E-01
cg05629350	24.20	9.16	8.28E-03	28.47	24.71	2.49E-01	1.96	37.93	9.59E-01	24.82	10.82	2.17E-02	27.22	31.19	3.83E-01
cg09729012	7.52	2.85	8.28E-03	10.76	4.11	8.95E-03	5.04	6.12	4.10E-01	14.36	9.87	1.46E-01	0.35	6.07	9.54E-01
cg11953775	-17.36	6.58	8.28E-03	-21.61	9.41	2.17E-02	-9.76	14.85	5.11E-01	-25.29	17.50	1.49E-01	-7.62	15.75	6.29E-01
cg18431324	25.83	9.78	8.28E-03	12.45	32.12	6.98E-01	71.76	118.27	5.44E-01	25.39	10.61	1.67E-02	51.94	43.84	2.36E-01
cg04657062	26.46	10.02	8.29E-03	48.74	18.52	8.48E-03	11.90	22.27	5.93E-01	16.87	17.12	3.24E-01	24.65	24.90	3.22E-01
cg00349061	-7.84	2.97	8.29E-03	-10.86	4.24	1.05E-02	-5.24	6.83	4.43E-01	-8.42	8.28	3.09E-01	-2.30	6.78	7.34E-01
cg06820719	-15.82	5.99	8.29E-03	-20.91	10.51	4.67E-02	-1.97	26.53	9.41E-01	-13.82	8.27	9.48E-02	-16.89	19.05	3.75E-01
cg21286117	23.67	8.97	8.29E-03	46.48	19.39	1.65E-02	11.84	16.19	4.65E-01	13.34	14.78	3.67E-01	43.80	25.41	8.48E-02
cg01414882	13.70	5.19	8.30E-03	17.42	7.31	1.71E-02	23.59	11.99	4.91E-02	-4.56	15.15	7.63E-01	5.38	11.89	6.51E-01
cg05564266	-8.74	3.31	8.30E-03	-8.18	7.45	2.73E-01	-3.56	10.09	7.24E-01	-10.49	4.34	1.57E-02	-5.69	9.87	5.64E-01
cg18711066	-35.34	13.39	8.31E-03	-38.06	15.78	1.59E-02	-59.59	47.73	2.12E-01	-7.81	78.04	9.20E-01	-17.58	32.30	5.86E-01
cg25732045	39.74	15.06	8.31E-03	55.91	37.35	1.34E-01	31.87	160.96	8.43E-01	31.57	17.27	6.75E-02	93.51	57.71	1.05E-01
cg20216752	13.22	5.01	8.31E-03	15.05	6.49	2.03E-02	20.42	11.56	7.73E-02	5.19	17.74	7.70E-01	-0.07	13.61	9.96E-01
cg02353723	28.89	10.95	8.31E-03	11.43	20.61	5.79E-01	48.00	43.32	2.68E-01	41.34	14.91	5.55E-03	2.73	32.28	9.33E-01
cg26305881	20.57	7.79	8.32E-03	15.57	15.67	3.20E-01	49.68	51.61	3.36E-01	20.85	9.75	3.25E-02	24.81	25.78	3.36E-01
cg05860220	-10.77	4.08	8.32E-03	-25.69	10.59	1.53E-02	-1.06	18.08	9.53E-01	-8.13	4.83	9.22E-02	-12.72	13.93	3.61E-01
cg11643245	-21.62	8.19	8.32E-03	-31.82	16.11	4.83E-02	-26.22	46.01	5.69E-01	-11.48	10.76	2.86E-01	-45.59	22.78	4.54E-02
cg24140687	23.31	8.83	8.33E-03	34.10	13.57	1.20E-02	15.18	24.81	5.41E-01	26.72	24.83	2.82E-01	11.00	15.55	4.79E-01
cg05103803	25.57	9.69	8.33E-03	18.33	16.54	2.68E-01	45.20	17.95	1.18E-02	14.98	19.27	4.37E-01	20.60	28.93	4.76E-01
cg09476306	-16.20	6.14	8.33E-03	-13.59	15.39	3.77E-01	-0.14	29.95	9.96E-01	-15.25	7.23	3.49E-02	-39.47	22.18	7.52E-02
cg17526424	29.90	11.33	8.33E-03	18.93	16.34	2.47E-01	22.29	22.01	3.11E-01	55.48	47.69	2.45E-01	59.55	25.51	1.96E-02
cg15084390	16.56	6.28	8.33E-03	31.16	16.87	6.48E-02	3.11	56.29	9.56E-01	16.00	7.14	2.49E-02	-2.27	22.87	9.21E-01
cg09674469	15.95	6.04	8.34E-03	33.49	15.73	3.33E-02	12.93	24.98	6.05E-01	14.29	7.10	4.40E-02	-1.88	23.17	9.35E-01
cg20652404	11.18	4.24	8.34E-03	13.74	5.76	1.70E-02	6.99	9.74	4.73E-01	18.00	11.82	1.28E-01	0.68	11.31	9.52E-01
cg14975347	-24.64	9.34	8.34E-03	-19.39	15.12	2.00E-01	-60.87	40.71	1.35E-01	-33.33	13.78	1.55E-02	12.10	28.67	6.73E-01
cg15826426	-13.27	5.03	8.34E-03	-24.50	9.48	9.73E-03	-14.44	20.57	4.83E-01	-11.60	7.11	1.03E-01	1.95	12.68	8.78E-01
cg24277024	23.32	8.84	8.34E-03	27.38	15.82	8.35E-02	36.62	22.82	1.09E-01	22.61	15.14	1.35E-01	7.95	19.94	6.90E-01
cg03973866	-78.45	29.74	8.34E-03	-77.57	41.11	5.92E-02	-183.04	149.37	2.20E-01	-153.85	68.90	2.56E-02	-11.93	57.43	8.35E-01

cg09777883	11.59	4.39	8.34E-03	15.05	6.01	1.24E-02	17.38	10.10	8.53E-02	5.53	12.72	6.64E-01	-2.41	11.03	8.27E-01
cg12452608	-19.09	7.24	8.35E-03	-20.13	20.63	3.29E-01	-19.88	44.04	6.52E-01	-19.52	8.30	1.87E-02	-13.76	24.31	5.71E-01
cg21548116	29.53	11.19	8.35E-03	1.54	28.00	9.56E-01	23.49	128.25	8.55E-01	34.17	12.77	7.44E-03	44.42	44.39	3.17E-01
cg06483978	12.97	4.92	8.36E-03	12.73	7.21	7.72E-02	13.89	9.12	1.28E-01	20.91	15.29	1.71E-01	5.99	13.12	6.48E-01
cg20475550	-17.19	6.52	8.36E-03	-23.07	10.43	2.69E-02	0.06	15.50	9.97E-01	-17.34	14.77	2.40E-01	-20.25	13.39	1.30E-01
cg22687244	19.67	7.46	8.36E-03	24.08	9.75	1.35E-02	13.76	23.72	5.62E-01	27.07	21.50	2.08E-01	4.92	16.85	7.70E-01
cg10296516	6.15	2.33	8.37E-03	4.90	3.89	2.07E-01	7.01	4.76	1.41E-01	7.40	4.10	7.11E-02	4.03	8.57	6.38E-01
cg21040133	21.83	8.28	8.38E-03	31.11	21.93	1.56E-01	4.38	24.26	8.57E-01	23.93	10.14	1.82E-02	12.41	30.61	6.85E-01
cg02480685	-17.23	6.54	8.39E-03	-35.33	17.20	4.00E-02	-4.96	36.55	8.92E-01	-12.07	7.50	1.08E-01	-43.60	25.75	9.04E-02
cg24403305	35.80	13.58	8.39E-03	45.87	20.72	2.68E-02	56.95	21.23	7.30E-03	6.57	14.34	6.47E-01	50.81	26.89	5.88E-02
cg07460524	-18.41	6.98	8.39E-03	-26.27	17.75	1.39E-01	3.03	44.48	9.46E-01	-17.74	8.30	3.25E-02	-16.48	20.86	4.29E-01
cg13879483	11.66	4.42	8.39E-03	20.90	10.19	4.02E-02	21.77	21.86	3.19E-01	7.10	5.67	2.11E-01	15.41	10.95	1.59E-01
cg01557549	21.15	8.03	8.39E-03	19.69	12.42	1.13E-01	13.69	17.06	4.22E-01	28.95	16.76	8.40E-02	24.77	22.14	2.63E-01
cg13273340	-11.38	4.32	8.40E-03	-14.49	5.89	1.39E-02	7.38	12.52	5.56E-01	-14.92	11.18	1.82E-01	-11.52	9.75	2.38E-01
cg12906963	-9.56	3.63	8.41E-03	-16.08	5.72	4.93E-03	-11.59	8.22	1.58E-01	-0.09	6.59	9.89E-01	-9.34	7.22	1.96E-01
cg27049654	14.19	5.39	8.41E-03	13.49	14.78	3.62E-01	10.71	20.87	6.08E-01	14.92	6.29	1.77E-02	11.16	20.78	5.91E-01
cg10126181	12.55	4.76	8.41E-03	19.40	6.85	4.64E-03	1.08	12.05	9.29E-01	14.47	14.77	3.27E-01	5.87	9.40	5.33E-01
cg27023953	-28.24	10.72	8.41E-03	-29.28	12.90	2.31E-02	-34.42	46.00	4.54E-01	-8.90	62.48	8.87E-01	-26.06	22.55	2.48E-01
cg14017655	23.34	8.86	8.41E-03	34.52	33.65	3.05E-01	61.90	108.12	5.67E-01	20.22	9.56	3.44E-02	48.19	34.51	1.63E-01
cg15819225	-16.96	6.43	8.42E-03	-20.12	9.18	2.84E-02	-9.71	15.27	5.25E-01	-23.19	20.56	2.59E-01	-13.18	13.34	3.23E-01
cg27264049	23.54	8.93	8.42E-03	56.64	23.92	1.79E-02	54.63	43.66	2.11E-01	15.22	10.21	1.36E-01	22.52	23.50	3.38E-01
cg06190046	9.03	3.43	8.42E-03	7.85	4.81	1.03E-01	9.71	7.34	1.86E-01	22.89	11.43	4.52E-02	4.72	8.00	5.55E-01
cg14695814	-17.93	6.81	8.42E-03	-48.99	22.60	3.02E-02	-2.40	26.28	9.27E-01	-16.54	7.62	3.00E-02	-3.13	32.22	9.23E-01
cg09037288	23.34	8.86	8.43E-03	29.86	15.03	4.69E-02	30.56	15.10	4.29E-02	-0.07	20.70	9.97E-01	19.61	25.06	4.34E-01
cg12286159	-12.01	4.56	8.43E-03	-14.75	9.83	1.33E-01	-6.82	14.59	6.40E-01	-12.63	5.82	3.01E-02	-5.78	16.67	7.29E-01
cg00807235	-18.15	6.89	8.44E-03	-12.09	7.44	1.04E-01	-4.72	15.02	7.53E-01	-45.10	17.35	9.34E-03	-23.70	11.88	4.60E-02
cg23750361	19.63	7.45	8.44E-03	36.15	18.96	5.65E-02	22.73	22.20	3.06E-01	13.44	9.41	1.53E-01	28.85	22.90	2.08E-01
cg25728685	10.82	4.11	8.44E-03	12.93	7.79	9.70E-02	11.98	10.13	2.37E-01	11.32	7.08	1.10E-01	6.53	8.73	4.54E-01
cg10872844	-15.14	5.75	8.44E-03	-2.07	14.46	8.86E-01	-41.28	26.12	1.14E-01	-16.59	6.76	1.41E-02	-11.54	21.58	5.93E-01
cg19224164	15.44	5.86	8.45E-03	15.43	7.94	5.22E-02	13.78	20.61	5.04E-01	36.43	23.02	1.14E-01	11.50	10.52	2.75E-01
cg16107470	-10.47	3.98	8.45E-03	-20.45	7.19	4.44E-03	-2.00	9.56	8.35E-01	-6.85	6.20	2.70E-01	-9.78	10.83	3.67E-01
cg07660635	-14.67	5.57	8.45E-03	-22.82	12.51	6.81E-02	-16.61	16.44	3.12E-01	-12.26	7.46	1.00E-01	-10.85	15.50	4.84E-01
cg03915174	-18.09	6.87	8.45E-03	-26.10	19.43	1.79E-01	-37.85	29.67	2.02E-01	-16.12	7.89	4.11E-02	-9.27	27.22	7.34E-01
cg11553183	-26.07	9.90	8.45E-03	-27.79	15.40	7.11E-02	-12.69	21.91	5.62E-01	-44.93	24.06	6.18E-02	-20.58	21.45	3.37E-01
cg13316404	-15.48	5.88	8.45E-03	-14.20	8.32	8.81E-02	-25.81	14.23	6.98E-02	-6.74	15.18	6.57E-01	-16.52	13.82	2.32E-01

cg24049468	12.17	4.62	8.45E-03	13.44	6.59	4.16E-02	10.64	10.13	2.93E-01	33.39	16.64	4.47E-02	3.48	9.77	7.22E-01
cg00745399	14.73	5.59	8.46E-03	24.12	10.07	1.66E-02	12.30	36.79	7.38E-01	14.33	7.59	5.89E-02	-6.26	15.82	6.92E-01
cg25461208	-50.94	19.35	8.46E-03	-16.17	55.07	7.69E-01	-91.82	205.25	6.55E-01	-54.14	23.04	1.88E-02	-61.21	48.00	2.02E-01
cg01271812	11.74	4.46	8.46E-03	17.73	6.65	7.72E-03	11.24	11.48	3.28E-01	11.78	9.73	2.26E-01	-2.05	10.24	8.42E-01
cg06228737	30.67	11.65	8.47E-03	33.58	16.87	4.65E-02	21.56	29.56	4.66E-01	32.02	36.46	3.80E-01	30.24	22.60	1.81E-01
cg20436086	-15.11	5.74	8.48E-03	-28.58	10.31	5.56E-03	-22.54	14.93	1.31E-01	-5.05	7.92	5.23E-01	-12.63	11.12	2.56E-01
cg01507753	-11.55	4.39	8.48E-03	-23.31	7.17	1.15E-03	-7.97	9.13	3.82E-01	-6.18	6.79	3.62E-01	-6.74	8.71	4.38E-01
cg06355570	12.52	4.76	8.48E-03	17.87	8.40	3.33E-02	11.54	9.39	2.19E-01	12.36	9.28	1.83E-01	3.61	11.92	7.62E-01
cg06177599	-10.36	3.94	8.49E-03	-12.74	5.71	2.56E-02	5.26	10.15	6.04E-01	-10.59	9.76	2.78E-01	-15.95	8.57	6.27E-02
cg03045635	9.66	3.67	8.49E-03	16.74	6.73	1.28E-02	5.70	8.91	5.23E-01	12.47	7.67	1.04E-01	2.83	6.65	6.70E-01
cg16792800	13.72	5.21	8.49E-03	22.90	8.75	8.87E-03	6.11	14.67	6.77E-01	13.25	11.86	2.64E-01	6.94	9.13	4.47E-01
cg18464080	43.77	16.63	8.50E-03	41.07	20.48	4.49E-02	19.11	76.98	8.04E-01	150.71	73.14	3.93E-02	33.02	33.83	3.29E-01
cg22803222	-10.55	4.01	8.51E-03	-13.32	7.53	7.70E-02	-6.98	11.13	5.30E-01	-11.56	5.80	4.62E-02	-3.16	12.12	7.94E-01
cg02796621	25.25	9.60	8.51E-03	27.36	18.67	1.43E-01	16.91	18.26	3.55E-01	27.10	17.28	1.17E-01	33.02	24.66	1.81E-01
cg13494428	14.43	5.48	8.51E-03	16.53	6.43	1.01E-02	24.92	8.83	4.78E-03	-7.52	16.08	6.40E-01	5.86	11.66	6.15E-01
cg15311814	17.11	6.50	8.52E-03	5.17	12.35	6.76E-01	30.09	18.75	1.09E-01	20.71	11.10	6.21E-02	19.09	12.78	1.35E-01
cg22152446	6.97	2.65	8.52E-03	5.98	3.78	1.13E-01	12.56	5.99	3.61E-02	6.53	5.88	2.67E-01	2.25	8.05	7.80E-01
cg01907184	-17.36	6.60	8.52E-03	-25.95	17.00	1.27E-01	-1.81	23.67	9.39E-01	-19.62	7.99	1.40E-02	0.91	22.11	9.67E-01
cg10841540	-15.14	5.75	8.52E-03	-13.18	12.51	2.92E-01	-1.56	32.97	9.62E-01	-15.69	6.97	2.44E-02	-20.99	20.76	3.12E-01
cg21400170	13.40	5.09	8.52E-03	11.66	6.17	5.89E-02	27.23	8.62	1.57E-03	-1.44	14.15	9.19E-01	8.46	9.13	3.54E-01
cg16909084	13.28	5.05	8.53E-03	18.45	8.11	2.29E-02	11.94	14.07	3.96E-01	12.64	10.06	2.09E-01	6.09	10.46	5.61E-01
cg06844526	-10.93	4.16	8.53E-03	-9.59	8.71	2.71E-01	-3.90	15.92	8.07E-01	-10.94	5.58	4.98E-02	-16.17	10.77	1.33E-01
cg09175338	-10.87	4.13	8.53E-03	-15.69	7.92	4.76E-02	-9.45	12.29	4.42E-01	-11.15	6.14	6.94E-02	-2.94	10.31	7.76E-01
cg09941507	29.55	11.23	8.53E-03	15.79	39.43	6.89E-01	-73.19	154.36	6.35E-01	35.22	12.07	3.53E-03	-38.43	51.38	4.54E-01
cg26910651	-43.31	16.47	8.54E-03	-88.73	53.31	9.60E-02	-9.52	172.46	9.56E-01	-44.27	18.19	1.49E-02	19.99	59.71	7.38E-01
cg08348900	19.28	7.33	8.55E-03	13.94	10.12	1.68E-01	-0.34	18.33	9.85E-01	37.97	40.15	3.44E-01	34.29	12.34	5.44E-03
cg13778336	9.63	3.66	8.55E-03	3.09	5.34	5.63E-01	14.18	8.42	9.22E-02	15.73	13.38	2.40E-01	16.30	7.12	2.21E-02
cg02520804	5.70	2.17	8.56E-03	5.27	3.16	9.49E-02	7.37	4.57	1.07E-01	5.43	4.71	2.49E-01	4.40	7.18	5.40E-01
cg02354107	-20.93	7.96	8.56E-03	-70.60	37.31	5.85E-02	-32.70	165.32	8.43E-01	-19.28	8.30	2.02E-02	2.44	43.62	9.55E-01
cg06655539	22.74	8.65	8.57E-03	3.18	18.83	8.66E-01	25.27	19.36	1.92E-01	21.63	11.47	5.93E-02	60.27	26.14	2.11E-02
cg01663970	11.41	4.34	8.57E-03	10.57	7.04	1.33E-01	23.08	9.52	1.53E-02	11.49	5.90	5.15E-02	-6.92	12.93	5.92E-01
cg12809787	-28.61	10.88	8.57E-03	-56.84	38.36	1.38E-01	193.15	160.04	2.27E-01	-25.80	11.70	2.75E-02	-52.26	48.66	2.83E-01
cg01132484	-7.86	2.99	8.58E-03	-7.39	5.01	1.40E-01	-5.34	6.92	4.41E-01	-5.62	5.49	3.06E-01	-15.97	7.47	3.25E-02
cg06103243	22.26	8.47	8.58E-03	30.73	11.51	7.58E-03	7.30	22.75	7.49E-01	10.47	35.92	7.71E-01	15.22	16.49	3.56E-01
cg15274202	-11.76	4.48	8.58E-03	-23.18	9.77	1.77E-02	-5.76	19.08	7.63E-01	-9.36	5.61	9.51E-02	-6.33	14.28	6.58E-01

cg07297964	8.44	3.21	8.59E-03	6.12	4.48	1.72E-01	12.62	8.36	1.31E-01	12.75	7.11	7.29E-02	6.17	8.74	4.80E-01
cg00251358	-9.37	3.57	8.59E-03	-4.25	6.50	5.14E-01	-13.04	14.09	3.55E-01	-11.48	5.09	2.40E-02	-11.29	9.43	2.31E-01
cg15952370	-8.96	3.41	8.60E-03	-18.23	6.78	7.18E-03	-6.02	11.96	6.15E-01	-5.86	4.84	2.26E-01	-5.62	8.29	4.98E-01
cg10188823	15.87	6.04	8.60E-03	16.24	16.20	3.16E-01	25.13	24.27	3.00E-01	15.87	8.11	5.03E-02	13.31	12.22	2.76E-01
cg12758039	-21.33	8.12	8.60E-03	-34.62	15.41	2.47E-02	-3.16	19.36	8.70E-01	-19.94	13.53	1.41E-01	-21.39	18.81	2.55E-01
cg18643958	-34.08	12.97	8.60E-03	-23.20	22.34	2.99E-01	-132.06	162.82	4.17E-01	-39.43	16.96	2.01E-02	-32.92	48.57	4.98E-01
cg11755796	20.06	7.63	8.60E-03	31.94	14.93	3.24E-02	9.90	22.63	6.62E-01	19.83	11.65	8.88E-02	10.60	17.25	5.39E-01
cg04639905	-6.79	2.58	8.60E-03	-6.91	5.10	1.75E-01	3.76	7.37	6.10E-01	-8.32	3.79	2.82E-02	-10.32	6.54	1.14E-01
cg19096358	11.72	4.46	8.61E-03	8.42	6.24	1.78E-01	19.51	8.24	1.79E-02	24.92	20.78	2.31E-01	3.71	11.51	7.47E-01
cg14398353	20.01	7.62	8.61E-03	29.93	16.48	6.94E-02	-10.33	32.53	7.51E-01	19.81	9.43	3.58E-02	15.95	26.92	5.54E-01
cg05776630	-7.23	2.75	8.62E-03	-8.55	4.02	3.33E-02	-6.83	6.40	2.86E-01	-8.05	7.08	2.56E-01	-3.79	6.22	5.43E-01
cg21574204	38.33	14.59	8.62E-03	37.62	46.31	4.17E-01	-307.64	208.89	1.41E-01	41.64	16.10	9.70E-03	25.39	53.44	6.35E-01
cg26414000	11.91	4.53	8.63E-03	13.88	8.70	1.11E-01	7.35	11.50	5.23E-01	15.36	6.69	2.16E-02	-0.54	13.44	9.68E-01
cg01392017	-15.55	5.92	8.63E-03	-6.14	14.83	6.79E-01	-18.54	14.80	2.10E-01	-18.86	7.55	1.25E-02	-0.27	23.00	9.91E-01
cg23263937	11.50	4.38	8.63E-03	18.58	8.14	2.25E-02	10.39	9.42	2.70E-01	6.26	8.96	4.85E-01	9.31	8.64	2.81E-01
cg05452618	24.16	9.20	8.63E-03	28.86	17.22	9.38E-02	14.17	17.27	4.12E-01	24.58	18.95	1.95E-01	31.33	20.82	1.32E-01
cg11874272	-11.14	4.24	8.63E-03	-12.65	9.04	1.62E-01	-8.06	19.82	6.84E-01	-7.87	5.35	1.42E-01	-28.79	13.04	2.73E-02
cg00113951	42.44	16.16	8.63E-03	63.94	21.46	2.89E-03	-2.49	44.67	9.56E-01	31.46	51.06	5.38E-01	16.68	35.94	6.43E-01
cg17890778	18.70	7.12	8.63E-03	6.29	10.99	5.67E-01	43.61	15.87	5.98E-03	18.65	11.63	1.09E-01	16.89	13.57	2.13E-01
cg07158816	-10.09	3.84	8.63E-03	-17.25	9.65	7.39E-02	-8.07	13.53	5.51E-01	-6.42	4.86	1.87E-01	-19.67	10.37	5.77E-02
cg08211028	14.27	5.43	8.64E-03	23.44	9.33	1.20E-02	4.13	15.74	7.93E-01	18.90	13.26	1.54E-01	7.12	8.88	4.23E-01
cg23125492	21.78	8.29	8.64E-03	18.08	12.37	1.44E-01	-0.76	16.48	9.63E-01	41.88	14.44	3.72E-03	24.87	15.20	1.02E-01
cg07064066	-32.82	12.50	8.64E-03	-55.34	20.73	7.61E-03	-52.57	27.13	5.26E-02	-11.89	13.10	3.64E-01	-34.84	33.85	3.03E-01
cg18094781	22.86	8.71	8.64E-03	40.62	16.76	1.53E-02	22.72	22.03	3.03E-01	26.58	14.53	6.74E-02	-1.05	17.27	9.52E-01
cg16514113	8.25	3.14	8.64E-03	11.72	4.66	1.20E-02	0.39	8.60	9.64E-01	7.79	8.63	3.67E-01	6.60	5.94	2.67E-01
cg08815340	11.33	4.31	8.64E-03	17.58	6.55	7.27E-03	9.67	10.13	3.40E-01	12.59	12.64	3.19E-01	1.80	8.32	8.29E-01
cg22619824	11.85	4.51	8.64E-03	15.11	5.78	8.99E-03	-0.30	8.14	9.70E-01	23.27	10.86	3.21E-02	11.42	9.98	2.53E-01
cg15889793	-15.57	5.93	8.65E-03	-0.94	17.67	9.57E-01	-23.00	26.15	3.79E-01	-17.30	6.69	9.67E-03	-13.57	26.57	6.10E-01
cg00703902	31.05	11.82	8.65E-03	15.68	20.18	4.37E-01	13.86	33.84	6.82E-01	50.82	28.56	7.52E-02	42.02	19.62	3.22E-02
cg06002476	-16.76	6.38	8.65E-03	-14.82	21.36	4.88E-01	-8.49	29.79	7.76E-01	-17.21	7.04	1.46E-02	-21.02	30.61	4.92E-01
cg12009606	19.63	7.48	8.65E-03	24.49	17.13	1.53E-01	-27.87	102.01	7.85E-01	19.84	8.75	2.33E-02	8.42	27.63	7.60E-01
cg11202380	13.89	5.29	8.65E-03	7.42	11.35	5.13E-01	28.82	14.76	5.08E-02	13.54	6.94	5.09E-02	9.65	19.72	6.25E-01
cg18674618	26.34	10.03	8.65E-03	24.27	18.97	2.01E-01	30.64	21.87	1.61E-01	14.52	16.23	3.71E-01	59.12	28.06	3.51E-02
cg18930267	19.15	7.29	8.65E-03	32.03	12.51	1.05E-02	4.30	13.65	7.53E-01	15.82	18.00	3.79E-01	21.09	15.90	1.85E-01
cg26862691	-38.61	14.70	8.65E-03	-43.19	26.44	1.02E-01	-24.08	80.19	7.64E-01	-46.17	20.01	2.10E-02	4.40	43.05	9.18E-01

cg25901559	-11.46	4.37	8.66E-03	-10.70	6.43	9.61E-02	-13.49	9.74	1.66E-01	-7.21	11.75	5.39E-01	-14.12	9.76	1.48E-01
cg11481451	-11.76	4.48	8.66E-03	-25.84	9.78	8.25E-03	-16.04	18.72	3.92E-01	-7.05	5.86	2.29E-01	-8.74	11.58	4.50E-01
cg20038647	22.71	8.65	8.66E-03	-5.50	23.82	8.17E-01	6.74	51.96	8.97E-01	26.74	9.95	7.19E-03	35.89	29.77	2.28E-01
cg10868668	-13.33	5.08	8.67E-03	-3.43	12.38	7.82E-01	-10.45	13.69	4.45E-01	-15.94	6.70	1.73E-02	-18.03	14.71	2.20E-01
cg05463782	21.44	8.17	8.67E-03	29.65	20.26	1.43E-01	75.46	81.28	3.53E-01	20.06	9.43	3.34E-02	10.47	29.56	7.23E-01
cg15450349	15.68	5.97	8.68E-03	13.13	8.09	1.04E-01	16.81	12.20	1.68E-01	10.34	24.49	6.73E-01	24.91	15.16	1.00E-01
cg01300495	-16.11	6.14	8.68E-03	-11.82	13.58	3.84E-01	12.29	27.88	6.59E-01	-20.14	7.68	8.76E-03	-13.18	18.61	4.79E-01
cg26129417	21.61	8.24	8.68E-03	31.31	15.00	3.69E-02	-7.92	24.72	7.49E-01	31.86	17.39	6.68E-02	16.26	13.66	2.34E-01
cg02084214	13.58	5.17	8.69E-03	14.38	9.60	1.34E-01	8.62	17.07	6.13E-01	14.04	7.16	4.97E-02	13.37	16.82	4.27E-01
cg09149541	-36.33	13.85	8.69E-03	-23.79	20.38	2.43E-01	-30.11	54.07	5.78E-01	-77.09	42.99	7.29E-02	-41.67	22.79	6.75E-02
cg02030275	10.90	4.15	8.69E-03	11.54	5.98	5.35E-02	7.01	9.24	4.48E-01	2.59	10.98	8.14E-01	20.53	10.00	4.00E-02
cg06967105	-15.27	5.82	8.69E-03	-21.15	8.30	1.09E-02	-5.20	12.21	6.70E-01	-14.77	18.98	4.36E-01	-12.33	13.43	3.59E-01
cg21859455	23.35	8.90	8.69E-03	17.94	15.17	2.37E-01	17.32	16.92	3.06E-01	25.50	20.50	2.14E-01	39.77	20.38	5.10E-02
cg24425807	-12.61	4.80	8.70E-03	-19.91	6.50	2.18E-03	-3.83	11.81	7.46E-01	-7.28	13.97	6.02E-01	-1.33	11.66	9.09E-01
cg07473175	50.74	19.34	8.70E-03	39.96	24.53	1.03E-01	16.95	73.55	8.18E-01	141.25	77.81	6.95E-02	64.67	38.88	9.62E-02
cg14199144	-17.74	6.76	8.70E-03	-26.08	13.97	6.19E-02	-8.38	25.36	7.41E-01	-14.37	8.62	9.55E-02	-27.59	23.97	2.50E-01
cg17409891	19.16	7.30	8.71E-03	23.64	19.03	2.14E-01	1.04	68.50	9.88E-01	21.33	8.34	1.05E-02	-9.54	26.85	7.22E-01
cg00011239	11.43	4.36	8.71E-03	14.03	7.80	7.22E-02	16.70	11.58	1.49E-01	10.66	6.44	9.81E-02	-2.08	14.62	8.87E-01
cg16711011	-41.43	15.79	8.71E-03	-34.39	17.75	5.26E-02	31.27	44.98	4.87E-01	-65.98	27.37	1.59E-02	-62.67	28.44	2.75E-02
cg09293391	-11.50	4.39	8.71E-03	-14.27	6.97	4.08E-02	-8.17	8.76	3.51E-01	1.06	12.22	9.31E-01	-17.54	9.24	5.76E-02
cg18034859	22.81	8.69	8.71E-03	10.40	15.77	5.10E-01	47.33	19.68	1.62E-02	26.07	13.60	5.52E-02	-2.23	28.44	9.38E-01
cg26613648	21.31	8.13	8.71E-03	24.54	18.23	1.78E-01	-4.25	41.71	9.19E-01	21.54	9.94	3.03E-02	23.18	26.30	3.78E-01
cg20316440	41.67	15.89	8.72E-03	33.02	21.70	1.28E-01	11.27	50.04	8.22E-01	69.11	43.51	1.12E-01	59.26	33.14	7.38E-02
cg01228271	-9.10	3.47	8.73E-03	-10.95	6.84	1.10E-01	-11.22	11.26	3.19E-01	-6.29	4.94	2.03E-01	-13.67	8.82	1.21E-01
cg01094114	-9.09	3.46	8.74E-03	-7.96	5.94	1.80E-01	-14.63	12.78	2.52E-01	-8.08	5.08	1.12E-01	-12.72	9.96	2.02E-01
cg10127921	-10.02	3.82	8.74E-03	-16.33	9.07	7.18E-02	-10.22	15.38	5.07E-01	-6.67	4.73	1.59E-01	-19.70	11.59	8.90E-02
cg07398594	26.55	10.13	8.74E-03	13.55	33.89	6.89E-01	186.16	137.06	1.74E-01	30.45	11.12	6.18E-03	-12.00	36.65	7.43E-01
cg15822795	31.50	12.01	8.74E-03	25.88	15.98	1.05E-01	5.85	33.76	8.62E-01	64.57	37.12	8.20E-02	46.02	26.62	8.38E-02
cg15484532	16.46	6.28	8.74E-03	26.08	12.20	3.26E-02	6.52	15.78	6.80E-01	15.19	11.12	1.72E-01	14.27	12.35	2.48E-01
cg01872840	16.85	6.43	8.74E-03	5.11	16.99	7.63E-01	7.40	30.31	8.07E-01	16.68	7.40	2.42E-02	55.11	26.62	3.84E-02
cg17894889	26.98	10.29	8.75E-03	3.77	38.50	9.22E-01	-113.14	158.44	4.75E-01	30.77	10.99	5.10E-03	4.10	47.32	9.31E-01
cg07891473	19.37	7.39	8.75E-03	18.77	11.32	9.75E-02	11.40	19.79	5.64E-01	32.98	22.14	1.36E-01	18.92	12.99	1.45E-01
cg00910168	10.41	3.97	8.76E-03	15.23	8.45	7.13E-02	6.19	9.68	5.23E-01	12.90	5.79	2.59E-02	-0.47	10.61	9.65E-01
cg22623223	23.17	8.84	8.76E-03	20.40	19.79	3.03E-01	16.65	27.81	5.49E-01	17.64	11.96	1.40E-01	50.79	22.58	2.45E-02
cg01368227	-14.16	5.40	8.76E-03	-5.89	11.08	5.95E-01	-38.12	22.05	8.38E-02	-12.18	6.99	8.13E-02	-30.69	16.77	6.72E-02

cg03315649	15.63	5.96	8.76E-03	13.25	8.92	1.38E-01	27.61	12.11	2.27E-02	7.78	15.39	6.13E-01	11.54	14.88	4.38E-01
cg09205083	17.16	6.55	8.78E-03	12.81	10.26	2.12E-01	13.03	11.76	2.68E-01	41.07	17.75	2.07E-02	15.77	17.06	3.55E-01
cg00699934	-43.37	16.55	8.78E-03	-40.16	21.11	5.71E-02	10.44	48.52	8.30E-01	-53.58	56.51	3.43E-01	-83.49	38.65	3.08E-02
cg01859669	14.24	5.43	8.78E-03	7.09	6.48	2.73E-01	26.14	10.72	1.47E-02	7.00	17.10	6.82E-01	25.94	13.60	5.65E-02
cg23188819	24.05	9.18	8.78E-03	11.76	19.69	5.50E-01	144.54	89.13	1.05E-01	24.90	11.23	2.66E-02	32.01	28.46	2.61E-01
cg06158065	19.99	7.63	8.78E-03	22.84	9.66	1.81E-02	20.47	27.75	4.61E-01	42.24	41.95	3.14E-01	10.47	14.74	4.78E-01
cg06803614	-16.01	6.11	8.79E-03	-20.98	11.92	7.83E-02	-22.16	14.59	1.29E-01	-6.06	9.46	5.22E-01	-28.24	16.07	7.90E-02
cg00451388	-13.59	5.19	8.79E-03	-16.72	7.84	3.30E-02	-8.82	11.94	4.60E-01	-13.79	13.94	3.22E-01	-11.47	10.68	2.83E-01
cg05330678	-28.44	10.85	8.80E-03	-25.66	17.27	1.37E-01	-11.21	53.00	8.33E-01	-19.92	16.79	2.36E-01	-65.46	28.48	2.15E-02
cg19779116	-34.09	13.01	8.80E-03	-48.38	39.62	2.22E-01	-75.19	176.62	6.70E-01	-37.73	14.45	9.02E-03	27.97	47.17	5.53E-01
cg17905462	-9.31	3.56	8.81E-03	-17.54	8.49	3.89E-02	-13.27	13.57	3.28E-01	-5.77	4.44	1.94E-01	-14.12	10.47	1.77E-01
cg04986304	13.49	5.15	8.81E-03	-4.64	13.46	7.30E-01	16.48	16.37	3.14E-01	16.09	6.18	9.24E-03	22.60	20.93	2.80E-01
cg05656180	20.29	7.75	8.81E-03	16.29	12.43	1.90E-01	35.91	20.03	7.29E-02	48.02	22.65	3.40E-02	8.61	13.19	5.14E-01
cg10437224	-12.57	4.80	8.81E-03	-19.04	8.62	2.72E-02	2.84	10.19	7.81E-01	-13.90	8.86	1.17E-01	-17.56	10.50	9.45E-02
cg12011522	-10.97	4.19	8.81E-03	-13.16	6.62	4.68E-02	-16.67	9.85	9.06E-02	-0.63	8.79	9.43E-01	-13.23	9.54	1.66E-01
cg06748153	37.46	14.30	8.81E-03	23.18	36.12	5.21E-01	74.87	162.82	6.46E-01	39.10	16.47	1.76E-02	46.31	50.15	3.56E-01
cg10406295	22.11	8.44	8.81E-03	27.07	12.04	2.46E-02	-3.49	28.46	9.03E-01	41.14	30.75	1.81E-01	17.42	14.38	2.26E-01
cg11014740	-18.66	7.13	8.81E-03	-23.87	10.89	2.83E-02	-14.63	16.92	3.87E-01	-12.44	15.15	4.11E-01	-17.85	17.14	2.98E-01
cg05172425	88.65	33.85	8.81E-03	43.86	48.48	3.66E-01	129.68	220.60	5.57E-01	99.66	77.64	1.99E-01	151.38	61.88	1.44E-02
cg17291423	7.32	2.79	8.82E-03	8.93	3.74	1.70E-02	11.65	6.73	8.34E-02	6.33	8.52	4.58E-01	-2.17	6.94	7.54E-01
cg21909206	18.03	6.88	8.82E-03	15.55	19.66	4.29E-01	11.71	25.39	6.45E-01	15.72	7.98	4.88E-02	59.73	28.20	3.41E-02
cg18019825	-33.25	12.70	8.83E-03	-40.33	18.25	2.72E-02	26.28	39.03	5.01E-01	-51.58	33.97	1.29E-01	-34.41	24.40	1.58E-01
cg09651708	-12.85	4.91	8.83E-03	-12.66	12.90	3.26E-01	1.81	20.88	9.31E-01	-12.27	5.79	3.40E-02	-28.39	17.25	9.99E-02
cg18718102	-19.93	7.61	8.84E-03	-18.53	10.89	8.87E-02	-23.54	19.23	2.21E-01	-6.82	20.45	7.39E-01	-28.92	16.40	7.78E-02
cg05030518	-20.04	7.65	8.84E-03	-12.58	19.23	5.13E-01	-35.35	48.40	4.65E-01	-20.66	9.07	2.27E-02	-23.46	23.73	3.23E-01
cg03587978	-10.61	4.05	8.84E-03	-9.12	6.70	1.74E-01	-11.38	7.97	1.54E-01	-11.28	9.12	2.16E-01	-11.82	9.61	2.19E-01
cg22096687	11.68	4.46	8.84E-03	17.56	6.39	5.97E-03	18.73	10.08	6.31E-02	6.59	8.65	4.46E-01	-1.22	9.90	9.02E-01
cg18200741	12.02	4.59	8.84E-03	14.11	6.36	2.65E-02	15.91	11.94	1.83E-01	14.03	12.53	2.63E-01	2.18	10.36	8.33E-01
cg13642142	15.66	5.98	8.84E-03	14.60	9.03	1.06E-01	18.67	11.75	1.12E-01	23.38	14.87	1.16E-01	4.51	16.00	7.78E-01
cg00101715	12.44	4.75	8.84E-03	10.85	6.31	8.56E-02	20.82	10.68	5.13E-02	19.67	19.91	3.23E-01	5.89	11.24	6.01E-01
cg25637226	26.48	10.12	8.85E-03	30.51	17.25	7.70E-02	21.71	20.85	2.98E-01	23.96	19.11	2.10E-01	29.67	27.00	2.72E-01
cg02761866	7.02	2.68	8.85E-03	10.21	4.85	3.53E-02	6.86	6.44	2.87E-01	7.28	4.14	7.87E-02	-3.49	8.48	6.81E-01
cg21202452	25.16	9.61	8.86E-03	21.66	15.22	1.55E-01	32.62	27.78	2.40E-01	45.09	26.74	9.17E-02	19.27	16.20	2.34E-01
cg07140366	-12.94	4.95	8.86E-03	-12.11	10.37	2.43E-01	-14.80	18.65	4.28E-01	-12.93	6.48	4.60E-02	-13.51	14.29	3.45E-01
cg11852671	27.49	10.50	8.86E-03	24.62	16.00	1.24E-01	10.07	35.15	7.74E-01	30.44	21.49	1.57E-01	36.14	21.39	9.11E-02

cg18351711	-19.26	7.36	8.87E-03	-29.86	13.28	2.46E-02	-9.93	16.52	5.48E-01	-17.69	12.91	1.71E-01	-14.01	17.87	4.33E-01
cg09412728	6.45	2.46	8.88E-03	6.43	3.72	8.39E-02	6.60	3.72	7.58E-02	-7.52	14.46	6.03E-01	10.14	8.07	2.09E-01
cg08061360	-91.69	35.04	8.88E-03	-98.33	50.77	5.28E-02	16.03	197.24	9.35E-01	-50.18	64.08	4.34E-01	-157.26	79.77	4.87E-02
cg18156192	-14.48	5.53	8.89E-03	-16.94	8.58	4.82E-02	1.06	13.95	9.39E-01	-24.89	12.64	4.88E-02	-12.02	11.43	2.93E-01
cg25185518	25.42	9.72	8.89E-03	15.80	13.76	2.51E-01	84.01	136.91	5.39E-01	34.96	14.89	1.89E-02	31.69	36.56	3.86E-01
cg01203828	21.55	8.24	8.90E-03	36.72	15.39	1.71E-02	26.28	17.52	1.34E-01	4.38	13.21	7.40E-01	34.03	25.62	1.84E-01
cg23274680	34.96	13.37	8.91E-03	40.21	26.11	1.24E-01	-25.29	69.58	7.16E-01	37.29	17.26	3.08E-02	29.55	41.94	4.81E-01
cg26638341	22.43	8.57	8.91E-03	36.96	18.86	5.00E-02	28.19	19.88	1.56E-01	11.20	12.33	3.64E-01	33.33	24.37	1.71E-01
cg19477793	10.51	4.02	8.91E-03	15.49	7.30	3.38E-02	8.05	8.75	3.58E-01	5.82	6.61	3.79E-01	16.75	11.72	1.53E-01
cg07394347	42.54	16.27	8.92E-03	55.91	19.63	4.40E-03	73.02	80.21	3.63E-01	13.18	72.25	8.55E-01	2.16	34.56	9.50E-01
cg17686885	15.25	5.83	8.94E-03	15.31	8.29	6.48E-02	7.48	14.42	6.04E-01	15.39	25.05	5.39E-01	19.53	10.88	7.26E-02
cg01363198	-10.18	3.89	8.94E-03	-11.46	5.28	2.99E-02	-12.29	9.13	1.78E-01	-16.83	13.30	2.06E-01	-1.41	8.97	8.76E-01
cg04552921	-15.15	5.79	8.94E-03	-16.07	11.85	1.75E-01	-12.14	23.19	6.01E-01	-13.94	7.65	6.82E-02	-20.48	16.45	2.13E-01
cg19711800	18.83	7.20	8.94E-03	24.97	17.49	1.53E-01	35.37	20.65	8.67E-02	16.20	8.99	7.16E-02	-1.68	27.96	9.52E-01
cg26486441	-12.74	4.87	8.94E-03	-12.00	6.96	8.44E-02	-18.75	11.43	1.01E-01	-22.54	14.79	1.27E-01	-4.53	10.43	6.64E-01
cg24693376	-9.83	3.76	8.95E-03	-18.28	7.40	1.35E-02	-5.23	9.05	5.63E-01	-8.61	5.82	1.39E-01	-4.01	9.68	6.79E-01
cg24593832	-11.39	4.36	8.95E-03	-15.30	10.12	1.30E-01	-14.54	13.13	2.68E-01	-9.53	5.71	9.50E-02	-11.43	12.43	3.58E-01
cg18656860	37.70	14.42	8.95E-03	35.14	31.62	2.66E-01	-88.39	175.01	6.14E-01	35.49	17.12	3.82E-02	76.78	52.42	1.43E-01
cg13253847	10.47	4.01	8.95E-03	11.49	5.44	3.49E-02	14.88	9.10	1.02E-01	7.79	10.56	4.61E-01	2.04	11.53	8.59E-01
cg03725444	13.41	5.13	8.95E-03	18.34	8.86	3.84E-02	11.25	8.49	1.85E-01	23.62	16.57	1.54E-01	4.35	11.38	7.02E-01
cg09085842	-33.34	12.76	8.95E-03	0.07	40.72	9.99E-01	105.20	237.97	6.58E-01	-37.55	13.80	6.50E-03	-35.06	60.45	5.62E-01
cg07766263	15.75	6.03	8.96E-03	27.02	9.34	3.83E-03	16.14	15.29	2.91E-01	1.41	14.15	9.20E-01	7.06	12.12	5.60E-01
cg04839835	10.74	4.11	8.97E-03	15.16	6.98	2.99E-02	10.71	10.71	3.17E-01	11.65	6.61	7.80E-02	-5.07	11.93	6.71E-01
cg09258878	36.74	14.06	8.97E-03	50.66	17.89	4.63E-03	27.84	44.03	5.27E-01	87.01	69.78	2.12E-01	-1.22	27.89	9.65E-01
cg11233547	12.73	4.87	8.98E-03	7.74	10.62	4.66E-01	6.60	12.25	5.90E-01	18.91	6.64	4.39E-03	-1.35	15.96	9.33E-01
cg05671968	-15.60	5.97	8.98E-03	-19.98	12.44	1.08E-01	-22.14	17.41	2.03E-01	-17.09	8.54	4.54E-02	-0.28	14.75	9.85E-01
cg16935061	-8.13	3.11	8.98E-03	-5.38	4.65	2.48E-01	-15.79	7.33	3.12E-02	-6.76	8.60	4.32E-01	-8.26	6.33	1.92E-01
cg17050724	24.34	9.32	8.98E-03	7.75	22.29	7.28E-01	7.91	67.77	9.07E-01	29.15	10.87	7.32E-03	19.82	34.87	5.70E-01
cg03127349	-10.98	4.20	8.98E-03	-9.84	5.97	9.95E-02	-8.68	9.69	3.70E-01	-12.38	11.89	2.98E-01	-15.26	9.59	1.12E-01
cg21556309	49.02	18.76	8.99E-03	54.25	44.85	2.26E-01	-143.90	146.71	3.27E-01	54.15	22.19	1.47E-02	33.85	61.29	5.81E-01
cg19708340	-16.25	6.22	8.99E-03	-23.50	15.74	1.35E-01	-19.23	25.15	4.45E-01	-11.64	7.51	1.21E-01	-35.55	20.09	7.68E-02
cg05655915	5.55	2.13	9.00E-03	4.48	2.85	1.16E-01	11.40	4.88	1.96E-02	7.38	5.40	1.72E-01	-1.95	6.52	7.65E-01
cg15816511	57.02	21.83	9.00E-03	40.69	27.25	1.35E-01	67.73	111.45	5.43E-01	43.94	78.04	5.73E-01	102.88	44.40	2.05E-02
cg23196129	18.74	7.17	9.01E-03	25.61	11.16	2.18E-02	11.56	16.87	4.93E-01	23.63	15.41	1.25E-01	5.00	16.48	7.62E-01
cg22058906	15.02	5.75	9.02E-03	10.95	8.07	1.75E-01	18.63	12.09	1.23E-01	21.17	18.33	2.48E-01	18.92	14.10	1.80E-01

cg16103159	-11.29	4.32	9.02E-03	-14.61	6.74	3.01E-02	-11.18	9.10	2.19E-01	-0.48	11.48	9.66E-01	-12.16	9.22	1.87E-01
cg04290346	19.77	7.57	9.03E-03	18.58	17.50	2.89E-01	8.28	24.50	7.35E-01	27.39	11.08	1.34E-02	10.81	15.16	4.76E-01
cg07137983	16.45	6.30	9.04E-03	20.31	11.81	8.55E-02	-3.28	21.29	8.78E-01	16.85	8.64	5.12E-02	20.69	20.27	3.07E-01
cg06362078	24.86	9.52	9.05E-03	4.09	22.10	8.53E-01	3.84	38.10	9.20E-01	26.54	12.01	2.71E-02	58.38	27.19	3.18E-02
cg23287229	-27.91	10.69	9.05E-03	-28.88	15.88	6.89E-02	-4.98	25.73	8.47E-01	-50.41	26.89	6.09E-02	-27.75	23.04	2.28E-01
cg16805291	6.62	2.54	9.05E-03	5.00	3.58	1.62E-01	14.43	6.35	2.31E-02	7.75	5.37	1.49E-01	0.67	7.46	9.29E-01
cg13839957	-8.94	3.42	9.06E-03	-8.90	4.64	5.51E-02	-5.92	7.90	4.54E-01	-9.35	13.19	4.78E-01	-11.75	7.67	1.25E-01
cg07562835	19.01	7.28	9.06E-03	28.38	10.08	4.86E-03	0.81	16.63	9.61E-01	21.34	22.44	3.42E-01	9.88	17.13	5.64E-01
cg02280348	-16.39	6.28	9.06E-03	5.26	16.22	7.46E-01	-4.10	41.08	9.20E-01	-21.43	7.21	2.97E-03	-12.22	23.99	6.11E-01
cg03541934	15.70	6.02	9.07E-03	-2.15	18.41	9.07E-01	-7.79	32.17	8.09E-01	17.65	6.69	8.35E-03	38.50	26.83	1.51E-01
cg03744954	-33.74	12.93	9.07E-03	-35.21	21.87	1.07E-01	43.46	104.59	6.78E-01	-26.65	18.34	1.46E-01	-64.10	34.80	6.54E-02
cg05940703	-28.79	11.03	9.08E-03	-13.45	18.17	4.59E-01	-36.82	40.41	3.62E-01	-7.18	30.78	8.16E-01	-47.11	16.87	5.22E-03
cg03074946	12.07	4.63	9.08E-03	14.15	6.49	2.93E-02	14.83	9.90	1.34E-01	19.73	13.04	1.30E-01	-5.60	12.01	6.41E-01
cg21912060	12.55	4.81	9.09E-03	16.15	7.07	2.22E-02	9.97	12.97	4.42E-01	17.94	20.43	3.80E-01	7.85	8.22	3.39E-01
cg26246387	48.50	18.59	9.09E-03	23.92	61.43	6.97E-01	282.42	220.37	2.00E-01	55.47	20.66	7.24E-03	-7.11	61.63	9.08E-01
cg19440720	12.12	4.64	9.09E-03	7.33	9.52	4.41E-01	-1.02	16.19	9.50E-01	18.34	6.06	2.48E-03	-3.38	15.27	8.25E-01
cg02037013	-12.69	4.87	9.09E-03	-14.27	12.30	2.46E-01	-13.44	13.96	3.36E-01	-9.53	6.37	1.35E-01	-23.69	13.12	7.09E-02
cg19243130	25.02	9.59	9.09E-03	28.16	17.30	1.04E-01	-5.47	42.27	8.97E-01	27.15	13.54	4.50E-02	21.73	25.69	3.98E-01
cg06603828	-47.23	18.11	9.09E-03	-44.25	21.04	3.55E-02	-12.67	88.10	8.86E-01	-197.21	103.71	5.72E-02	-42.37	41.88	3.12E-01
cg26228241	-52.62	20.17	9.10E-03	-55.71	59.17	3.46E-01	-116.27	313.17	7.10E-01	-44.50	22.75	5.04E-02	-114.71	66.19	8.31E-02
cg23018242	-42.72	16.38	9.10E-03	-60.55	31.43	5.40E-02	16.58	94.68	8.61E-01	-30.51	23.10	1.87E-01	-58.40	37.02	1.15E-01
cg02019072	37.80	14.49	9.10E-03	23.40	20.70	2.58E-01	46.05	49.93	3.56E-01	67.52	44.30	1.28E-01	47.78	25.67	6.27E-02
cg24145020	-12.60	4.83	9.10E-03	-19.90	12.34	1.07E-01	-28.76	23.87	2.28E-01	-11.78	5.74	4.01E-02	-0.27	15.49	9.86E-01
cg10964330	-12.68	4.86	9.10E-03	-10.92	12.76	3.92E-01	-18.83	28.02	5.02E-01	-14.70	5.69	9.81E-03	2.03	15.73	8.97E-01
cg04651548	13.45	5.16	9.11E-03	13.25	8.37	1.14E-01	5.21	10.78	6.29E-01	15.72	10.43	1.32E-01	23.08	13.48	8.68E-02
cg07598199	-11.97	4.59	9.12E-03	-17.24	9.60	7.26E-02	-7.17	16.96	6.72E-01	-9.22	6.36	1.47E-01	-15.26	10.92	1.62E-01
cg00971364	41.18	15.79	9.12E-03	51.20	23.83	3.17E-02	76.22	67.97	2.62E-01	121.42	66.72	6.88E-02	17.26	23.53	4.63E-01
cg10229594	19.93	7.64	9.12E-03	18.80	10.49	7.32E-02	-16.35	36.87	6.57E-01	42.80	21.27	4.42E-02	17.27	14.02	2.18E-01
cg04450052	-9.57	3.67	9.12E-03	-10.21	5.19	4.90E-02	-10.44	9.91	2.92E-01	-5.28	8.27	5.23E-01	-12.00	9.01	1.83E-01
cg27420415	-11.06	4.24	9.13E-03	-17.85	10.22	8.07E-02	-2.40	18.76	8.98E-01	-9.60	5.16	6.31E-02	-13.57	13.26	3.06E-01
cg09857324	15.41	5.91	9.13E-03	17.02	9.41	7.04E-02	13.16	11.78	2.64E-01	10.10	14.34	4.81E-01	19.95	13.80	1.48E-01
cg22103219	-10.93	4.19	9.13E-03	-14.54	5.95	1.45E-02	-13.00	11.07	2.40E-01	-5.82	10.73	5.87E-01	-4.59	9.21	6.18E-01
cg01793445	-19.38	7.43	9.14E-03	-32.61	13.37	1.47E-02	-27.43	18.10	1.30E-01	-5.70	15.06	7.05E-01	-11.78	14.09	4.03E-01
cg25358938	19.76	7.58	9.14E-03	5.73	22.31	7.97E-01	103.44	102.93	3.15E-01	20.05	8.32	1.60E-02	38.42	34.10	2.60E-01
cg25141766	21.04	8.07	9.14E-03	15.95	10.96	1.46E-01	37.61	14.22	8.16E-03	30.26	26.29	2.50E-01	-6.34	22.67	7.80E-01

cg19596804	-9.78	3.75	9.14E-03	-21.13	10.14	3.71E-02	-7.08	14.05	6.14E-01	-9.34	4.46	3.64E-02	2.47	12.77	8.47E-01
cg03733196	32.87	12.61	9.14E-03	74.85	45.10	9.70E-02	-3.91	215.83	9.86E-01	30.41	13.65	2.59E-02	16.67	49.28	7.35E-01
cg13126530	16.38	6.28	9.14E-03	14.87	21.17	4.82E-01	28.99	32.19	3.68E-01	18.98	7.38	1.02E-02	1.43	16.28	9.30E-01
cg13421412	10.77	4.13	9.14E-03	16.45	6.97	1.83E-02	12.95	9.06	1.53E-01	8.60	9.88	3.84E-01	2.97	8.02	7.11E-01
cg07989501	31.33	12.02	9.15E-03	27.95	19.49	1.52E-01	-26.24	79.76	7.42E-01	37.74	17.56	3.16E-02	28.11	33.58	4.02E-01
cg03339609	-15.56	5.97	9.15E-03	-12.04	8.27	1.45E-01	-12.82	15.23	4.00E-01	-19.04	17.14	2.67E-01	-24.57	13.24	6.34E-02
cg24020826	-17.49	6.71	9.16E-03	-36.96	12.60	3.36E-03	-3.63	17.04	8.31E-01	-13.28	8.83	1.32E-01	-11.64	15.95	4.65E-01
cg22795239	-19.02	7.30	9.17E-03	-14.93	13.98	2.86E-01	-26.14	21.57	2.26E-01	-18.26	10.27	7.55E-02	-25.38	22.23	2.54E-01
cg25181170	6.90	2.65	9.17E-03	9.49	4.08	2.00E-02	4.74	6.19	4.44E-01	8.62	5.04	8.71E-02	-2.87	7.65	7.07E-01
cg19587237	12.91	4.96	9.18E-03	14.70	6.99	3.55E-02	15.00	10.80	1.65E-01	26.80	15.79	8.98E-02	-1.48	11.44	8.97E-01
cg23168603	-18.48	7.09	9.19E-03	-16.68	21.24	4.32E-01	30.04	33.98	3.77E-01	-19.43	8.04	1.56E-02	-42.20	27.55	1.26E-01
cg27640302	17.61	6.76	9.19E-03	21.21	9.97	3.35E-02	23.84	14.08	9.05E-02	20.75	19.05	2.76E-01	-1.31	15.76	9.34E-01
cg09581137	-9.93	3.81	9.19E-03	-8.23	5.79	1.55E-01	-8.72	7.58	2.50E-01	-18.46	11.29	1.02E-01	-10.26	8.51	2.28E-01
cg03238901	24.83	9.53	9.20E-03	17.13	18.38	3.51E-01	-19.10	51.23	7.09E-01	31.09	12.36	1.19E-02	23.47	29.89	4.32E-01
cg05144928	-6.22	2.39	9.20E-03	-6.02	3.40	7.61E-02	-6.86	4.50	1.27E-01	-10.31	10.06	3.05E-01	-4.35	5.85	4.57E-01
cg09610286	27.29	10.48	9.20E-03	45.74	22.56	4.26E-02	103.41	59.34	8.14E-02	20.91	13.35	1.17E-01	9.57	28.33	7.35E-01
cg02968844	24.73	9.49	9.21E-03	35.03	17.93	5.08E-02	27.57	17.28	1.11E-01	6.47	18.09	7.21E-01	33.73	25.16	1.80E-01
cg11071231	25.50	9.79	9.21E-03	36.73	27.09	1.75E-01	-8.33	57.22	8.84E-01	23.94	11.78	4.20E-02	29.53	25.38	2.45E-01
cg10514207	-11.66	4.48	9.21E-03	-13.83	9.00	1.24E-01	4.49	18.89	8.12E-01	-9.64	6.09	1.14E-01	-20.95	11.30	6.39E-02
cg20167471	24.45	9.39	9.21E-03	25.68	18.89	1.74E-01	31.41	18.89	9.64E-02	22.92	16.28	1.59E-01	15.71	22.54	4.86E-01
cg27298262	20.94	8.04	9.21E-03	18.46	16.11	2.52E-01	16.87	16.59	3.09E-01	17.46	12.57	1.65E-01	49.03	24.61	4.64E-02
cg22619549	-19.66	7.55	9.21E-03	-27.23	9.71	5.03E-03	-28.87	18.95	1.28E-01	-19.35	24.42	4.28E-01	3.73	15.33	8.08E-01
cg11176095	13.21	5.07	9.22E-03	13.74	7.42	6.40E-02	24.74	12.71	5.15E-02	16.69	14.11	2.37E-01	2.79	10.28	7.86E-01
cg17254229	-14.36	5.52	9.22E-03	-16.46	7.84	3.57E-02	-5.83	13.40	6.63E-01	-20.73	14.13	1.42E-01	-11.28	12.90	3.82E-01
cg26105283	-17.66	6.78	9.22E-03	-35.09	12.05	3.60E-03	1.04	14.30	9.42E-01	-15.83	9.27	8.78E-02	-16.96	13.62	2.13E-01
cg10111314	-32.80	12.60	9.22E-03	-0.72	34.32	9.83E-01	-125.62	142.22	3.77E-01	-34.32	14.29	1.64E-02	-62.71	44.32	1.57E-01
cg24579887	13.11	5.04	9.22E-03	20.20	7.40	6.34E-03	22.00	13.01	9.08E-02	5.98	13.67	6.62E-01	2.31	8.77	7.92E-01
cg19363699	-68.28	26.22	9.22E-03	-126.76	69.80	6.94E-02	-439.09	263.43	9.55E-02	-57.27	30.88	6.36E-02	-37.02	73.42	6.14E-01
cg09051513	22.09	8.49	9.23E-03	21.93	18.99	2.48E-01	0.49	21.32	9.82E-01	20.82	9.28	2.49E-02	62.74	26.63	1.85E-02
cg24674703	-9.81	3.77	9.24E-03	-12.22	5.28	2.07E-02	-3.75	9.99	7.07E-01	-9.80	9.02	2.77E-01	-7.74	9.04	3.92E-01
cg03916864	-57.25	22.00	9.25E-03	-81.06	63.68	2.03E-01	-142.98	280.86	6.11E-01	-54.14	25.41	3.31E-02	-48.98	62.09	4.30E-01
cg17125472	8.82	3.39	9.25E-03	6.51	4.32	1.32E-01	20.69	8.34	1.31E-02	3.53	11.56	7.60E-01	8.20	9.27	3.77E-01
cg06576119	59.66	22.93	9.26E-03	78.91	53.48	1.40E-01	-128.64	202.29	5.25E-01	57.51	27.33	3.54E-02	63.63	72.55	3.80E-01
cg26070383	23.96	9.21	9.26E-03	33.62	16.95	4.73E-02	11.53	17.11	5.00E-01	28.22	18.55	1.28E-01	22.15	22.38	3.22E-01
cg15543281	45.20	17.37	9.26E-03	79.84	44.24	7.11E-02	168.21	223.50	4.52E-01	33.86	19.74	8.63E-02	86.78	68.08	2.02E-01

cg03906115	7.69	2.96	9.27E-03	8.27	4.08	4.25E-02	14.59	7.29	4.55E-02	7.36	8.85	4.05E-01	0.62	6.65	9.25E-01
cg23294388	-38.31	14.72	9.27E-03	-43.53	17.23	1.15E-02	-7.14	69.33	9.18E-01	-54.47	78.04	4.85E-01	-22.59	33.84	5.04E-01
cg09176893	-28.95	11.13	9.27E-03	-23.85	17.71	1.78E-01	0.29	46.59	9.95E-01	-27.21	24.96	2.76E-01	-40.48	18.82	3.15E-02
cg06705138	19.29	7.41	9.27E-03	28.48	15.02	5.80E-02	-0.35	35.56	9.92E-01	17.54	9.41	6.22E-02	16.07	24.47	5.11E-01
cg10181822	28.76	11.05	9.27E-03	53.21	34.77	1.26E-01	-82.05	158.54	6.05E-01	28.87	12.04	1.65E-02	-10.81	48.82	8.25E-01
cg12616683	34.37	13.21	9.27E-03	35.65	16.92	3.52E-02	60.85	57.07	2.86E-01	32.73	45.44	4.71E-01	26.23	26.30	3.19E-01
cg13552869	-12.50	4.80	9.27E-03	-5.89	5.38	2.74E-01	-16.97	8.15	3.73E-02	-27.41	9.23	2.99E-03	-6.10	7.88	4.39E-01
cg15538809	18.09	6.95	9.28E-03	18.89	8.90	3.38E-02	14.00	19.56	4.74E-01	42.55	26.69	1.11E-01	9.74	15.75	5.36E-01
cg02576296	-22.37	8.60	9.28E-03	-18.25	19.04	3.38E-01	-12.94	22.74	5.69E-01	-29.86	11.51	9.48E-03	-1.37	27.92	9.61E-01
cg13115617	-110.05	42.30	9.28E-03	-123.03	72.41	8.93E-02	-86.23	179.82	6.32E-01	-59.96	28.73	3.69E-02	-240.94	83.07	3.73E-03
cg06132069	16.07	6.18	9.29E-03	24.34	8.80	5.69E-03	5.48	17.49	7.54E-01	4.29	21.18	8.40E-01	10.19	11.32	3.68E-01
cg12099279	-18.92	7.28	9.29E-03	-29.82	16.12	6.44E-02	-45.61	35.06	1.93E-01	-12.79	9.29	1.69E-01	-21.69	19.40	2.64E-01
cg10019392	-26.97	10.37	9.29E-03	-30.27	13.42	2.41E-02	-16.22	43.45	7.09E-01	-54.19	34.09	1.12E-01	-11.71	20.57	5.69E-01
cg27070729	21.03	8.08	9.29E-03	23.60	13.67	8.42E-02	16.50	36.62	6.52E-01	21.60	11.63	6.33E-02	12.95	23.47	5.81E-01
cg21792134	5.26	2.02	9.30E-03	5.48	2.93	6.18E-02	7.58	5.87	1.97E-01	5.96	3.73	1.11E-01	0.10	6.00	9.87E-01
cg18785679	-15.16	5.83	9.30E-03	-19.08	9.34	4.12E-02	-4.36	11.61	7.07E-01	-9.76	14.14	4.90E-01	-26.41	13.43	4.92E-02
cg23770553	21.66	8.33	9.30E-03	48.19	22.09	2.91E-02	12.86	45.39	7.77E-01	15.72	9.63	1.03E-01	34.31	30.16	2.55E-01
cg02322376	-43.15	16.59	9.31E-03	-49.87	20.71	1.60E-02	-62.34	74.42	4.02E-01	6.50	52.16	9.01E-01	-41.98	36.44	2.49E-01
cg00381179	40.55	15.59	9.31E-03	38.22	19.31	4.78E-02	-29.12	59.70	6.26E-01	38.64	75.47	6.09E-01	67.36	32.03	3.55E-02
cg01585985	32.44	12.47	9.31E-03	15.66	32.70	6.32E-01	27.81	159.45	8.62E-01	40.22	14.12	4.39E-03	-20.56	47.82	6.67E-01
cg01449064	-47.89	18.42	9.31E-03	-65.02	24.32	7.51E-03	-64.32	70.79	3.64E-01	-24.31	70.43	7.30E-01	-15.78	34.19	6.44E-01
cg20098659	5.36	2.06	9.32E-03	2.80	3.40	4.10E-01	11.22	4.43	1.14E-02	4.92	3.55	1.66E-01	3.10	7.27	6.70E-01
cg23218979	39.22	15.09	9.32E-03	43.00	20.02	3.17E-02	64.45	61.52	2.95E-01	58.30	49.12	2.35E-01	19.58	28.62	4.94E-01
cg14633757	17.55	6.75	9.33E-03	24.78	16.15	1.25E-01	-18.77	55.20	7.34E-01	16.92	8.08	3.62E-02	15.04	20.16	4.56E-01
cg26681183	10.30	3.96	9.33E-03	15.20	6.02	1.15E-02	12.41	7.51	9.84E-02	0.57	13.74	9.67E-01	1.03	8.75	9.07E-01
cg15081720	-15.62	6.01	9.33E-03	-28.98	13.43	3.10E-02	-20.73	17.42	2.34E-01	-10.78	7.92	1.74E-01	-10.92	18.47	5.55E-01
cg13686589	32.38	12.46	9.33E-03	57.12	40.59	1.59E-01	-180.59	174.47	3.01E-01	29.33	13.61	3.11E-02	53.43	49.86	2.84E-01
cg03594311	-12.94	4.98	9.34E-03	-12.79	7.03	6.90E-02	-13.78	11.55	2.33E-01	-24.43	21.97	2.66E-01	-10.39	9.74	2.86E-01
cg07064595	25.64	9.86	9.34E-03	37.87	17.42	2.97E-02	33.00	31.25	2.91E-01	11.85	14.92	4.27E-01	35.27	26.14	1.77E-01
cg02570171	-37.02	14.24	9.35E-03	-63.74	26.03	1.44E-02	84.06	77.63	2.79E-01	-34.21	13.93	1.40E-02	-30.42	36.34	4.03E-01
cg14041976	-11.09	4.27	9.35E-03	-15.12	10.34	1.44E-01	-20.02	22.64	3.77E-01	-8.34	5.12	1.04E-01	-20.07	13.44	1.35E-01
cg19564093	-12.36	4.76	9.35E-03	-9.23	8.75	2.91E-01	-5.89	14.71	6.89E-01	-15.12	7.11	3.34E-02	-14.78	12.19	2.26E-01
cg09465746	15.45	5.94	9.35E-03	22.31	17.02	1.90E-01	88.76	62.40	1.55E-01	14.98	6.83	2.83E-02	5.15	17.82	7.72E-01
cg23655651	9.29	3.57	9.35E-03	9.74	5.56	7.97E-02	9.56	7.56	2.06E-01	14.38	7.56	5.72E-02	-0.59	9.54	9.50E-01
cg02214188	6.97	2.68	9.35E-03	7.50	3.43	2.86E-02	0.52	6.22	9.33E-01	24.81	15.67	1.13E-01	8.99	6.45	1.63E-01

cg20904489	12.90	4.96	9.35E-03	3.94	8.16	6.29E-01	28.65	12.34	2.03E-02	15.64	11.39	1.70E-01	13.79	9.40	1.43E-01
cg02417684	14.38	5.53	9.36E-03	7.43	16.56	6.54E-01	27.94	54.10	6.06E-01	14.94	6.12	1.46E-02	17.47	22.81	4.44E-01
cg00292312	-11.08	4.27	9.36E-03	-13.99	6.02	2.02E-02	-16.36	8.31	4.90E-02	-16.17	9.65	9.38E-02	2.13	8.01	7.91E-01
cg22872195	-13.40	5.16	9.37E-03	-19.14	11.38	9.25E-02	-16.86	13.65	2.17E-01	-13.04	6.84	5.67E-02	4.11	17.80	8.17E-01
cg12670943	17.69	6.81	9.38E-03	23.81	9.94	1.66E-02	29.13	13.50	3.09E-02	5.28	15.78	7.38E-01	-2.86	17.10	8.67E-01
cg25225073	30.88	11.89	9.38E-03	41.57	15.22	6.31E-03	9.27	42.24	8.26E-01	6.63	39.49	8.67E-01	19.03	25.32	4.52E-01
cg14780540	22.16	8.53	9.38E-03	12.67	26.17	6.28E-01	25.52	90.16	7.77E-01	22.44	9.39	1.68E-02	34.91	35.13	3.20E-01
cg22357164	-9.77	3.76	9.38E-03	-11.20	4.94	2.34E-02	4.32	9.92	6.63E-01	-12.18	12.75	3.40E-01	-15.00	8.64	8.24E-02
cg13965321	-30.97	11.92	9.38E-03	-30.42	29.95	3.10E-01	-14.96	112.83	8.95E-01	-33.91	13.82	1.42E-02	-8.74	40.63	8.30E-01
ch.8.49616578R	-70.44	27.12	9.38E-03	-58.01	27.54	3.52E-02	112.50	172.31	5.14E-01	-189.82	87.19	2.95E-02	-80.54	59.05	1.73E-01
cg14384093	-15.16	5.84	9.39E-03	-13.34	15.75	3.97E-01	-31.19	27.38	2.55E-01	-12.36	6.80	6.91E-02	-34.79	20.54	9.03E-02
cg15019432	24.73	9.52	9.39E-03	36.27	15.65	2.05E-02	22.65	18.07	2.10E-01	8.92	20.91	6.70E-01	21.87	25.02	3.82E-01
cg26954951	-12.84	4.94	9.39E-03	-15.69	6.33	1.32E-02	-17.53	14.77	2.35E-01	1.46	14.67	9.21E-01	-8.95	12.19	4.63E-01
cg27555092	-11.90	4.58	9.40E-03	-23.40	10.78	2.99E-02	-12.07	15.30	4.30E-01	-7.77	5.89	1.87E-01	-15.10	12.95	2.44E-01
cg04782776	21.00	8.08	9.40E-03	13.36	16.74	4.25E-01	2.64	40.23	9.48E-01	31.27	9.68	1.23E-03	-6.13	21.40	7.75E-01
cg19542907	35.03	13.49	9.41E-03	75.89	49.88	1.28E-01	193.29	191.66	3.13E-01	33.34	14.53	2.17E-02	-3.60	55.02	9.48E-01
cg05048806	18.38	7.08	9.41E-03	27.70	16.95	1.02E-01	10.53	26.24	6.88E-01	17.39	8.60	4.32E-02	13.31	25.77	6.05E-01
cg03109660	-10.66	4.10	9.41E-03	-9.46	5.61	9.18E-02	-7.46	9.34	4.24E-01	-13.53	13.90	3.30E-01	-16.11	9.55	9.16E-02
cg13543106	-11.25	4.33	9.42E-03	-5.64	9.52	5.54E-01	-0.53	14.17	9.70E-01	-14.31	5.57	1.03E-02	-14.63	14.07	2.98E-01
cg14613832	54.65	21.05	9.42E-03	54.72	27.35	4.54E-02	27.07	77.05	7.25E-01	82.93	62.31	1.83E-01	49.14	44.97	2.75E-01
cg16874347	-16.35	6.30	9.43E-03	-15.88	10.37	1.26E-01	-23.54	13.80	8.81E-02	-11.74	14.89	4.30E-01	-14.28	12.74	2.62E-01
cg06401643	-15.48	5.96	9.44E-03	-10.80	10.76	3.15E-01	8.20	32.31	8.00E-01	-20.86	7.78	7.36E-03	-2.69	22.30	9.04E-01
cg07106911	27.61	10.64	9.44E-03	12.18	17.94	4.97E-01	-22.00	61.10	7.19E-01	39.32	15.11	9.24E-03	36.70	30.37	2.27E-01
cg02184918	19.70	7.59	9.45E-03	19.38	11.81	1.01E-01	32.94	16.72	4.89E-02	1.76	15.72	9.11E-01	30.47	19.75	1.23E-01
cg07297068	15.35	5.92	9.46E-03	21.52	8.88	1.54E-02	4.98	11.99	6.78E-01	6.91	15.83	6.63E-01	20.94	14.20	1.40E-01
cg26397662	10.19	3.93	9.46E-03	-1.55	7.73	8.41E-01	9.05	10.35	3.82E-01	15.00	4.39	6.29E-04	11.98	10.74	2.65E-01
cg16175725	24.43	9.41	9.46E-03	35.58	17.48	4.18E-02	16.89	17.51	3.35E-01	21.37	17.43	2.20E-01	23.15	26.12	3.75E-01
cg06534061	59.89	23.08	9.47E-03	63.20	29.44	3.18E-02	108.61	119.50	3.63E-01	39.53	74.50	5.96E-01	52.34	45.97	2.55E-01
cg01412450	16.15	6.23	9.47E-03	32.21	12.05	7.52E-03	22.46	55.99	6.88E-01	8.95	6.15	1.45E-01	25.78	19.57	1.88E-01
cg00791321	21.42	8.26	9.48E-03	20.59	12.40	9.69E-02	29.80	16.52	7.12E-02	28.02	20.65	1.75E-01	2.51	21.52	9.07E-01
cg15646626	17.18	6.62	9.48E-03	13.75	20.14	4.95E-01	2.20	36.17	9.52E-01	17.66	7.38	1.67E-02	26.51	28.95	3.60E-01
cg27443867	19.51	7.52	9.49E-03	7.67	27.84	7.83E-01	34.82	96.88	7.19E-01	21.08	8.07	8.97E-03	8.02	33.02	8.08E-01
cg04558907	-19.74	7.61	9.49E-03	-11.06	20.16	5.83E-01	28.39	41.28	4.92E-01	-22.18	8.72	1.10E-02	-36.15	30.61	2.38E-01
cg12982090	17.49	6.74	9.49E-03	20.04	12.82	1.18E-01	27.00	13.71	4.89E-02	8.73	12.32	4.79E-01	15.41	15.82	3.30E-01
cg08947915	6.97	2.69	9.49E-03	4.67	3.67	2.03E-01	10.81	5.54	5.13E-02	6.26	9.55	5.12E-01	9.50	6.91	1.69E-01

cg09522479	11.45	4.42	9.49E-03	18.27	5.49	8.87E-04	19.52	7.97	1.43E-02	4.95	6.26	4.29E-01	1.96	7.78	8.02E-01
cg03291835	7.42	2.86	9.50E-03	3.19	4.48	4.76E-01	7.55	6.93	2.76E-01	14.24	5.47	9.25E-03	6.30	7.40	3.95E-01
cg13099330	17.08	6.58	9.50E-03	20.35	9.79	3.76E-02	24.56	18.22	1.78E-01	17.63	15.92	2.68E-01	6.69	13.28	6.15E-01
cg04880412	11.85	4.57	9.50E-03	15.52	6.31	1.39E-02	2.12	10.08	8.33E-01	-0.45	14.64	9.75E-01	19.19	10.98	8.06E-02
cg10112781	-15.19	5.86	9.50E-03	-10.00	13.24	4.50E-01	-34.87	27.16	1.99E-01	-16.27	7.36	2.71E-02	-10.50	16.58	5.26E-01
cg21123160	10.80	4.17	9.51E-03	14.45	6.59	2.84E-02	7.21	9.43	4.45E-01	11.70	11.50	3.09E-01	7.62	7.96	3.38E-01
cg10435849	20.58	7.94	9.51E-03	9.48	26.47	7.20E-01	26.53	38.65	4.93E-01	17.89	9.23	5.25E-02	41.93	22.17	5.86E-02
cg22532194	23.20	8.95	9.51E-03	34.01	17.49	5.19E-02	30.28	22.39	1.76E-01	7.22	14.04	6.07E-01	37.82	21.51	7.87E-02
cg03653504	-18.46	7.12	9.52E-03	-12.15	16.65	4.66E-01	-13.43	26.63	6.14E-01	-23.46	8.87	8.16E-03	-1.54	22.42	9.45E-01
cg26053358	21.23	8.19	9.52E-03	24.47	18.01	1.74E-01	28.03	17.48	1.09E-01	13.40	11.75	2.54E-01	39.75	27.51	1.49E-01
cg06672737	9.89	3.81	9.52E-03	11.45	5.69	4.41E-02	17.75	10.22	8.25E-02	1.83	8.42	8.28E-01	9.18	8.39	2.74E-01
cg23189410	10.21	3.94	9.52E-03	12.42	6.81	6.83E-02	11.08	9.07	2.22E-01	7.35	8.11	3.65E-01	9.27	8.02	2.47E-01
cg00544674	-9.84	3.79	9.52E-03	-6.51	7.35	3.75E-01	-4.97	14.20	7.26E-01	-10.88	5.16	3.49E-02	-15.37	10.91	1.59E-01
cg05506209	45.69	17.62	9.53E-03	61.77	23.12	7.54E-03	74.36	68.77	2.80E-01	24.73	49.13	6.15E-01	7.69	37.19	8.36E-01
cg13796319	35.13	13.55	9.53E-03	36.10	44.84	4.21E-01	165.22	164.08	3.14E-01	32.03	14.79	3.03E-02	61.40	54.43	2.59E-01
cg04830316	-12.00	4.63	9.53E-03	-12.97	7.08	6.71E-02	-14.66	10.77	1.73E-01	-6.09	11.78	6.05E-01	-12.01	9.56	2.09E-01
cg02494664	36.96	14.26	9.53E-03	44.78	16.23	5.81E-03	39.89	70.93	5.74E-01	-58.07	78.66	4.60E-01	17.47	36.17	6.29E-01
cg04808813	72.94	28.14	9.53E-03	70.53	37.62	6.08E-02	97.10	123.91	4.33E-01	88.55	67.04	1.87E-01	60.50	60.98	3.21E-01
cg12104133	21.07	8.13	9.53E-03	13.40	14.19	3.45E-01	34.78	16.02	2.99E-02	3.09	18.71	8.69E-01	31.58	17.10	6.48E-02
cg11653966	-7.77	3.00	9.54E-03	-6.98	4.14	9.19E-02	-7.99	6.99	2.53E-01	-8.00	13.30	5.48E-01	-9.28	6.11	1.29E-01
cg02614660	14.63	5.65	9.54E-03	11.88	8.30	1.52E-01	23.14	12.53	6.47E-02	15.49	17.67	3.81E-01	12.31	11.72	2.94E-01
cg08555772	-42.92	16.56	9.54E-03	-38.99	21.59	7.09E-02	-70.50	45.71	1.23E-01	-52.30	57.88	3.66E-01	-32.47	37.13	3.82E-01
cg18035537	-52.33	20.19	9.54E-03	-1.48	65.37	9.82E-01	-289.08	235.52	2.20E-01	-62.90	19.26	1.09E-03	29.71	75.24	6.93E-01
cg10522770	11.96	4.62	9.55E-03	14.50	6.31	2.16E-02	8.68	11.36	4.45E-01	27.10	14.83	6.75E-02	0.74	10.23	9.42E-01
cg22353823	5.18	2.00	9.55E-03	7.82	2.92	7.35E-03	5.49	4.23	1.95E-01	2.96	5.74	6.07E-01	-0.40	4.64	9.31E-01
cg02877433	25.95	10.01	9.55E-03	39.49	17.66	2.54E-02	10.33	21.33	6.28E-01	20.79	18.04	2.49E-01	30.44	25.82	2.38E-01
cg11689732	31.62	12.20	9.55E-03	31.78	19.23	9.85E-02	60.21	28.38	3.39E-02	34.48	25.83	1.82E-01	0.06	28.01	9.98E-01
cg11223003	36.55	14.10	9.56E-03	46.56	33.24	1.61E-01	-204.76	137.27	1.36E-01	36.90	14.13	9.00E-03	42.19	42.02	3.15E-01
cg10994263	-11.77	4.54	9.56E-03	-9.73	9.64	3.13E-01	4.59	14.83	7.57E-01	-17.64	6.02	3.40E-03	-0.07	13.34	9.96E-01
cg11612647	-13.31	5.14	9.56E-03	-11.95	7.33	1.03E-01	-15.08	14.03	2.82E-01	-4.15	12.08	7.31E-01	-24.08	11.66	3.89E-02
cg26926973	-74.61	28.79	9.56E-03	-63.54	36.82	8.45E-02	8.13	213.92	9.70E-01	-171.91	69.69	1.36E-02	-32.86	64.44	6.10E-01
cg21017887	11.48	4.43	9.57E-03	11.07	11.84	3.50E-01	22.56	26.50	3.95E-01	10.17	5.10	4.60E-02	21.14	16.04	1.87E-01
cg06801028	7.59	2.93	9.57E-03	6.47	3.82	9.00E-02	10.55	6.79	1.21E-01	11.25	10.93	3.03E-01	6.60	7.50	3.79E-01
cg17112108	-15.96	6.16	9.57E-03	-27.19	11.46	1.76E-02	4.13	14.80	7.80E-01	-19.68	13.02	1.31E-01	-14.04	11.00	2.02E-01
cg02512540	-23.34	9.01	9.58E-03	-1.13	19.76	9.55E-01	12.96	46.06	7.78E-01	-33.22	11.15	2.88E-03	-18.87	28.42	5.07E-01

cg24259363	-13.39	5.17	9.58E-03	-18.58	7.78	1.69E-02	-21.19	12.47	8.93E-02	-4.13	12.46	7.40E-01	-3.93	11.13	7.24E-01
cg04850731	23.84	9.20	9.58E-03	49.64	20.36	1.48E-02	34.75	50.57	4.92E-01	12.96	11.49	2.59E-01	34.92	26.40	1.86E-01
cg10278025	-10.94	4.22	9.58E-03	-14.83	6.57	2.40E-02	1.75	8.95	8.45E-01	-15.71	10.31	1.28E-01	-13.07	9.53	1.70E-01
cg07314148	25.18	9.72	9.58E-03	27.67	11.82	1.92E-02	3.08	39.96	9.39E-01	50.66	58.02	3.83E-01	20.56	19.98	3.04E-01
cg04454086	14.37	5.55	9.59E-03	22.37	10.65	3.57E-02	6.76	10.76	5.30E-01	25.92	9.53	6.50E-03	4.60	8.28	5.78E-01
cg19701577	22.21	8.58	9.59E-03	28.06	19.65	1.53E-01	-17.67	48.30	7.15E-01	24.25	10.33	1.88E-02	7.92	28.85	7.84E-01
cg01033356	7.12	2.75	9.59E-03	9.11	3.82	1.71E-02	1.94	6.28	7.58E-01	-2.12	9.13	8.17E-01	11.13	6.15	7.05E-02
cg10316857	-13.93	5.38	9.60E-03	-21.99	11.23	5.01E-02	-1.42	17.81	9.36E-01	-11.18	7.35	1.28E-01	-19.25	14.19	1.75E-01
cg00255135	28.08	10.84	9.60E-03	34.36	14.32	1.64E-02	32.54	41.19	4.30E-01	13.15	32.34	6.84E-01	18.98	21.91	3.86E-01
cg12158993	24.04	9.28	9.60E-03	24.69	13.19	6.13E-02	20.06	26.80	4.54E-01	30.27	25.40	2.33E-01	21.35	18.51	2.49E-01
cg24194730	21.39	8.26	9.61E-03	32.75	12.41	8.33E-03	1.53	17.70	9.31E-01	9.78	17.49	5.76E-01	37.68	24.25	1.20E-01
cg04468281	13.72	5.30	9.63E-03	13.39	8.46	1.14E-01	11.80	12.66	3.51E-01	20.28	9.73	3.71E-02	2.81	14.41	8.45E-01
cg12691229	13.20	5.10	9.63E-03	17.37	7.17	1.54E-02	19.45	12.47	1.19E-01	-0.60	20.79	9.77E-01	4.47	9.88	6.51E-01
cg06483699	29.74	11.49	9.63E-03	68.92	35.08	4.95E-02	27.34	161.27	8.65E-01	26.94	12.52	3.15E-02	-10.02	53.52	8.52E-01
cg17693604	11.32	4.37	9.64E-03	9.20	6.12	1.32E-01	19.31	8.58	2.44E-02	24.78	16.87	1.42E-01	-0.41	10.87	9.70E-01
cg11550126	45.70	17.65	9.64E-03	62.06	21.75	4.33E-03	-22.97	99.29	8.17E-01	39.88	72.88	5.84E-01	12.76	35.24	7.17E-01
cg22537081	-56.99	22.02	9.65E-03	-47.98	26.27	6.78E-02	-173.57	167.78	3.01E-01	-102.13	75.58	1.77E-01	-59.51	49.82	2.32E-01
cg26019498	18.42	7.12	9.65E-03	34.69	16.61	3.67E-02	18.61	32.52	5.67E-01	15.40	8.64	7.46E-02	7.84	23.76	7.42E-01
cg22110213	-22.49	8.69	9.65E-03	-30.32	23.20	1.91E-01	19.28	34.85	5.80E-01	-27.17	10.19	7.67E-03	4.58	32.78	8.89E-01
cg00892737	24.44	9.45	9.66E-03	31.38	21.15	1.38E-01	24.39	25.34	3.36E-01	15.88	12.67	2.10E-01	56.48	29.05	5.19E-02
cg10332321	19.59	7.57	9.66E-03	25.82	14.15	6.79E-02	-0.77	24.29	9.75E-01	15.45	10.65	1.47E-01	40.04	22.67	7.74E-02
cg26027526	-6.93	2.68	9.66E-03	-5.78	4.39	1.88E-01	-0.75	5.47	8.91E-01	-10.76	5.22	3.91E-02	-14.06	7.56	6.29E-02
cg06139239	-11.54	4.46	9.66E-03	-18.47	9.57	5.37E-02	-11.24	14.12	4.26E-01	-9.49	6.08	1.19E-01	-8.99	11.66	4.41E-01
cg24271863	23.19	8.96	9.66E-03	24.13	13.59	7.59E-02	18.22	23.51	4.38E-01	35.26	20.77	8.96E-02	14.93	18.53	4.20E-01
cg23817981	26.12	10.09	9.67E-03	37.47	14.42	9.38E-03	35.30	32.91	2.84E-01	49.65	53.02	3.49E-01	6.97	16.38	6.71E-01
cg19531536	-9.81	3.79	9.67E-03	-8.90	5.55	1.09E-01	-14.42	8.88	1.04E-01	-1.21	9.47	8.99E-01	-14.84	8.67	8.70E-02
cg09940032	14.24	5.51	9.68E-03	19.04	9.87	5.38E-02	15.58	14.05	2.68E-01	13.88	9.30	1.35E-01	5.75	12.80	6.53E-01
cg00747890	26.86	10.38	9.68E-03	32.67	13.88	1.86E-02	15.30	27.05	5.72E-01	8.65	31.38	7.83E-01	29.31	24.23	2.26E-01
cg15174823	37.00	14.31	9.69E-03	29.14	19.24	1.30E-01	13.50	84.29	8.73E-01	4.68	45.05	9.17E-01	63.10	25.39	1.30E-02
cg18498035	27.57	10.66	9.69E-03	38.07	38.05	3.17E-01	86.37	138.00	5.31E-01	23.38	11.42	4.07E-02	82.73	50.33	1.00E-01
cg26121591	12.72	4.92	9.70E-03	20.58	15.94	1.97E-01	57.70	45.38	2.04E-01	11.87	5.52	3.15E-02	6.67	15.55	6.68E-01
cg22960952	-10.79	4.17	9.70E-03	-15.34	6.99	2.82E-02	-0.89	10.15	9.30E-01	-12.59	7.99	1.15E-01	-8.63	9.28	3.53E-01
cg21704448	-11.91	4.60	9.70E-03	-11.29	11.34	3.20E-01	-23.40	18.25	2.00E-01	-10.82	5.53	5.03E-02	-13.47	16.46	4.13E-01
cg00806680	-18.98	7.34	9.71E-03	-6.87	19.00	7.18E-01	-45.70	28.00	1.03E-01	-19.24	8.71	2.73E-02	-16.10	27.29	5.55E-01
cg25113589	20.39	7.89	9.72E-03	44.07	21.90	4.42E-02	42.89	37.41	2.52E-01	14.60	9.05	1.06E-01	25.39	30.72	4.08E-01

cg06358171	7.15	2.76	9.72E-03	9.99	4.20	1.73E-02	10.59	6.20	8.78E-02	3.13	7.12	6.60E-01	1.12	5.93	8.50E-01
cg23015361	-8.98	3.47	9.72E-03	-11.65	5.97	5.12E-02	-4.05	10.28	6.93E-01	-7.65	5.23	1.44E-01	-11.30	10.61	2.87E-01
cg16681349	17.58	6.80	9.72E-03	24.11	14.48	9.59E-02	0.97	28.63	9.73E-01	15.00	8.29	7.05E-02	41.96	30.21	1.65E-01
cg20932516	-16.00	6.19	9.73E-03	-23.22	18.50	2.10E-01	-54.20	28.71	5.91E-02	-14.62	7.10	3.94E-02	2.92	21.80	8.94E-01
cg10797557	54.13	20.94	9.73E-03	58.70	26.15	2.48E-02	3.07	91.51	9.73E-01	121.16	72.83	9.62E-02	28.22	44.26	5.24E-01
cg25417766	10.92	4.22	9.73E-03	16.01	5.74	5.31E-03	2.87	10.04	7.75E-01	17.59	16.18	2.77E-01	2.58	9.13	7.78E-01
cg05694021	10.20	3.95	9.74E-03	11.78	5.59	3.53E-02	9.12	8.15	2.63E-01	21.58	14.66	1.41E-01	3.27	8.92	7.14E-01
cg07802350	17.51	6.77	9.74E-03	3.31	24.03	8.90E-01	-35.25	69.72	6.13E-01	20.36	7.32	5.43E-03	2.80	28.82	9.23E-01
cg16592453	21.90	8.47	9.74E-03	16.49	17.21	3.38E-01	55.84	64.19	3.84E-01	23.73	10.33	2.16E-02	14.34	32.62	6.60E-01
cg07452306	15.48	5.99	9.74E-03	21.47	9.17	1.92E-02	18.63	13.22	1.59E-01	18.19	14.11	1.97E-01	-4.07	13.78	7.68E-01
cg19356179	21.54	8.33	9.74E-03	35.52	21.08	9.20E-02	41.50	29.48	1.59E-01	13.08	8.37	1.18E-01	58.96	32.41	6.89E-02
cg22559669	-37.07	14.34	9.75E-03	-88.05	33.59	8.77E-03	-15.86	195.65	9.35E-01	-26.18	16.61	1.15E-01	-21.18	55.57	7.03E-01
cg02995055	-22.79	8.82	9.75E-03	-30.63	12.13	1.16E-02	-10.48	22.21	6.37E-01	5.37	37.25	8.85E-01	-20.40	17.36	2.40E-01
cg17133056	38.09	14.74	9.75E-03	35.18	21.41	1.00E-01	33.77	32.41	2.98E-01	55.87	37.65	1.38E-01	35.36	36.14	3.28E-01
cg10114372	63.25	24.47	9.75E-03	79.42	49.09	1.06E-01	20.78	200.89	9.18E-01	71.82	31.61	2.31E-02	1.08	66.06	9.87E-01
cg01757116	-9.39	3.63	9.75E-03	-3.83	7.90	6.28E-01	-14.84	12.14	2.22E-01	-9.41	4.78	4.93E-02	-14.95	10.41	1.51E-01
cg06317622	-52.68	20.38	9.76E-03	-50.66	27.10	6.16E-02	-39.66	61.76	5.21E-01	-12.79	74.12	8.63E-01	-75.03	40.79	6.59E-02
cg19360907	12.03	4.66	9.76E-03	15.40	6.19	1.29E-02	-1.37	15.73	9.31E-01	7.58	15.06	6.15E-01	10.81	9.28	2.44E-01
cg04972065	9.91	3.84	9.76E-03	12.86	5.68	2.37E-02	16.12	8.23	5.02E-02	-4.04	10.99	7.13E-01	5.09	8.47	5.48E-01
cg16388178	14.65	5.67	9.77E-03	16.08	8.58	6.09E-02	10.24	11.28	3.64E-01	14.73	18.96	4.37E-01	16.82	12.05	1.63E-01
cg15440661	9.26	3.58	9.77E-03	9.27	4.76	5.17E-02	12.02	7.58	1.13E-01	-0.21	11.47	9.85E-01	11.94	10.67	2.63E-01
cg09440340	-19.55	7.57	9.77E-03	-26.40	11.46	2.12E-02	-7.87	16.47	6.33E-01	-6.65	18.82	7.24E-01	-27.70	17.29	1.09E-01
cg07620167	9.04	3.50	9.78E-03	4.52	4.88	3.55E-01	16.80	6.56	1.04E-02	-0.38	11.80	9.74E-01	13.21	7.75	8.84E-02
cg19731276	19.10	7.39	9.78E-03	6.76	23.65	7.75E-01	20.99	29.11	4.71E-01	19.11	8.27	2.09E-02	46.31	37.28	2.14E-01
cg08588414	21.01	8.13	9.78E-03	18.80	14.85	2.06E-01	15.10	38.69	6.96E-01	18.02	10.95	9.99E-02	45.69	25.18	6.96E-02
cg11624728	-8.69	3.36	9.78E-03	-5.47	5.70	3.37E-01	-6.93	7.82	3.76E-01	-11.33	6.52	8.22E-02	-12.43	7.52	9.85E-02
cg12419195	27.41	10.61	9.78E-03	28.89	35.55	4.16E-01	-60.14	132.02	6.49E-01	27.87	11.47	1.51E-02	28.23	47.91	5.56E-01
cg12146709	15.79	6.11	9.79E-03	10.30	9.09	2.57E-01	25.45	11.07	2.15E-02	33.12	22.18	1.35E-01	5.15	14.94	7.30E-01
cg27299712	-16.24	6.29	9.79E-03	-12.09	9.55	2.05E-01	-24.99	15.24	1.01E-01	-20.33	13.87	1.43E-01	-13.46	14.39	3.50E-01
cg22613905	-50.01	19.36	9.79E-03	-70.54	29.29	1.60E-02	40.08	99.97	6.89E-01	-63.79	37.61	8.98E-02	-14.52	37.93	7.02E-01
cg04400131	-13.02	5.04	9.79E-03	-16.95	11.18	1.29E-01	2.34	16.29	8.86E-01	-11.17	6.68	9.46E-02	-26.06	13.87	6.02E-02
cg26269038	18.30	7.08	9.79E-03	10.62	15.20	4.85E-01	37.29	13.72	6.57E-03	6.69	11.95	5.76E-01	21.19	15.17	1.63E-01
cg14216672	-67.52	26.14	9.80E-03	-41.20	36.81	2.63E-01	-5.92	109.55	9.57E-01	-112.98	53.71	3.54E-02	-97.28	58.18	9.45E-02
cg16786297	-13.58	5.26	9.80E-03	-7.51	13.67	5.83E-01	-28.88	32.22	3.70E-01	-10.41	6.28	9.71E-02	-35.69	14.99	1.73E-02
cg20130213	19.23	7.45	9.80E-03	25.09	11.27	2.59E-02	24.71	18.91	1.91E-01	10.35	32.74	7.52E-01	10.95	12.47	3.80E-01

cg14585694	44.96	17.41	9.80E-03	73.56	45.61	1.07E-01	-147.13	150.81	3.29E-01	38.30	20.23	5.83E-02	78.23	55.02	1.55E-01
cg15252509	-9.05	3.50	9.81E-03	-12.82	6.38	4.47E-02	-9.29	10.27	3.66E-01	-5.39	4.99	2.80E-01	-16.15	11.68	1.67E-01
cg16581097	51.67	20.01	9.81E-03	42.03	27.65	1.28E-01	10.17	58.62	8.62E-01	72.46	50.01	1.47E-01	84.47	44.76	5.91E-02
cg24231854	9.47	3.67	9.81E-03	13.87	5.25	8.29E-03	11.16	8.10	1.69E-01	0.51	12.92	9.68E-01	1.70	7.68	8.24E-01
cg16598600	14.30	5.54	9.82E-03	15.18	9.33	1.04E-01	7.42	11.42	5.16E-01	24.41	13.32	6.69E-02	12.48	11.30	2.69E-01
cg14353162	54.40	21.07	9.82E-03	50.09	27.03	6.38E-02	125.70	79.50	1.14E-01	74.12	68.73	2.81E-01	35.81	44.11	4.17E-01
cg03038233	19.03	7.37	9.82E-03	11.62	13.49	3.89E-01	-3.92	30.66	8.98E-01	26.77	10.28	9.24E-03	15.66	20.43	4.43E-01
cg13372927	28.36	10.99	9.83E-03	19.47	17.31	2.61E-01	-67.05	68.17	3.25E-01	39.72	16.03	1.32E-02	35.50	34.52	3.04E-01
cg18758339	-20.75	8.04	9.83E-03	-28.04	20.94	1.80E-01	-21.99	41.61	5.97E-01	-21.69	9.27	1.93E-02	7.59	31.61	8.10E-01
cg12506005	29.93	11.59	9.83E-03	53.28	26.07	4.10E-02	16.61	53.45	7.56E-01	19.35	14.67	1.87E-01	50.00	32.08	1.19E-01
cg18280830	13.99	5.42	9.83E-03	17.83	8.55	3.71E-02	-0.71	12.94	9.56E-01	13.10	13.05	3.16E-01	18.71	10.81	8.34E-02
cg09105802	28.02	10.85	9.83E-03	35.90	25.86	1.65E-01	12.43	48.77	7.99E-01	27.37	13.39	4.09E-02	26.39	31.64	4.04E-01
cg23982890	16.80	6.51	9.83E-03	12.81	12.43	3.03E-01	9.69	12.01	4.20E-01	17.20	11.39	1.31E-01	45.55	19.98	2.26E-02
cg07812877	26.50	10.27	9.83E-03	41.91	21.82	5.48E-02	3.82	54.88	9.45E-01	22.88	13.01	7.87E-02	23.50	29.47	4.25E-01
cg07855639	-15.48	6.00	9.83E-03	-10.76	8.64	2.13E-01	-10.32	17.06	5.45E-01	-37.06	16.88	2.81E-02	-16.16	11.55	1.62E-01
cg20894145	21.45	8.31	9.83E-03	27.71	16.33	8.97E-02	46.31	19.63	1.83E-02	8.50	12.48	4.96E-01	18.62	24.11	4.40E-01
cg04115307	-8.30	3.21	9.84E-03	-5.49	4.44	2.16E-01	-8.85	7.26	2.23E-01	-9.32	9.72	3.38E-01	-15.65	7.79	4.44E-02
cg11611320	-13.52	5.24	9.84E-03	-22.24	11.31	4.92E-02	5.55	14.91	7.10E-01	-16.49	6.14	7.21E-03	1.92	18.09	9.15E-01
cg17831467	53.95	20.90	9.84E-03	62.52	53.08	2.39E-01	-182.82	212.68	3.90E-01	57.19	24.05	1.74E-02	35.30	73.97	6.33E-01
cg17242490	22.57	8.74	9.84E-03	30.30	22.79	1.84E-01	41.16	29.48	1.63E-01	16.17	10.61	1.27E-01	41.14	29.99	1.70E-01
cg04453677	28.26	10.95	9.84E-03	42.76	33.16	1.97E-01	1.70	107.80	9.87E-01	28.65	12.25	1.93E-02	8.34	38.43	8.28E-01
cg09577907	-15.15	5.87	9.84E-03	-19.67	10.95	7.25E-02	-1.86	21.58	9.31E-01	-18.54	8.61	3.14E-02	-4.33	14.07	7.58E-01
cg02147791	52.00	20.15	9.84E-03	42.70	25.39	9.26E-02	-49.46	108.78	6.49E-01	72.42	80.21	3.67E-01	81.48	38.55	3.46E-02
cg24739407	-7.42	2.88	9.85E-03	-10.79	4.93	2.88E-02	-9.89	6.45	1.25E-01	-4.65	5.31	3.81E-01	-2.55	7.02	7.17E-01
cg02526522	-28.17	10.91	9.85E-03	-24.31	14.06	8.38E-02	18.94	44.80	6.72E-01	-30.16	54.32	5.79E-01	-45.12	20.01	2.41E-02
cg06534800	23.77	9.21	9.86E-03	25.44	17.23	1.40E-01	29.61	17.04	8.22E-02	14.54	16.63	3.82E-01	29.40	27.13	2.78E-01
cg03828603	21.98	8.52	9.86E-03	21.42	10.44	4.03E-02	34.46	29.52	2.43E-01	79.02	59.75	1.86E-01	14.09	17.71	4.26E-01
cg01381171	23.30	9.03	9.87E-03	42.49	27.93	1.28E-01	-4.67	140.07	9.73E-01	20.33	9.77	3.75E-02	40.58	46.67	3.85E-01
cg00832703	-19.54	7.57	9.87E-03	-26.33	21.11	2.12E-01	-58.39	39.32	1.38E-01	-13.41	8.72	1.24E-01	-48.80	26.93	7.00E-02
cg01031251	10.76	4.17	9.87E-03	11.10	5.65	4.96E-02	19.34	9.65	4.50E-02	6.48	11.53	5.74E-01	1.89	11.19	8.66E-01
cg17085123	-10.79	4.18	9.88E-03	-8.91	5.61	1.13E-01	-8.97	10.88	4.10E-01	0.04	17.02	9.98E-01	-19.12	8.60	2.62E-02
cg17133833	-16.28	6.31	9.88E-03	-29.59	13.44	2.76E-02	-17.48	26.90	5.16E-01	-12.49	8.09	1.23E-01	-10.28	18.53	5.79E-01
cg15591578	20.88	8.09	9.89E-03	21.04	17.61	2.32E-01	-7.55	56.11	8.93E-01	22.16	9.71	2.25E-02	16.35	29.96	5.85E-01
cg08483980	21.80	8.45	9.89E-03	33.53	14.76	2.30E-02	8.92	15.60	5.67E-01	24.99	16.91	1.39E-01	15.08	23.57	5.22E-01
cg21911861	45.37	17.59	9.90E-03	55.14	25.60	3.13E-02	3.83	108.59	9.72E-01	25.52	11.98	3.31E-02	110.03	42.14	9.02E-03

cg14864276	-81.11	31.45	9.91E-03	-58.16	33.87	8.60E-02	58.36	128.78	6.50E-01	-160.31	49.94	1.33E-03	-65.48	53.61	2.22E-01
cg22952828	-43.33	16.80	9.91E-03	-36.74	20.68	7.57E-02	-114.60	70.25	1.03E-01	-46.18	79.69	5.62E-01	-43.93	34.41	2.02E-01
cg26694616	31.30	12.14	9.91E-03	41.22	38.41	2.83E-01	-138.59	191.68	4.70E-01	33.76	13.39	1.17E-02	0.14	44.37	9.98E-01
cg13721644	24.84	9.63	9.92E-03	27.27	16.90	1.07E-01	11.44	18.06	5.26E-01	22.01	19.33	2.55E-01	51.06	25.55	4.56E-02
cg19908362	-32.87	12.75	9.92E-03	-31.71	17.97	7.76E-02	-9.77	27.65	7.24E-01	-63.44	44.05	1.50E-01	-47.55	28.49	9.51E-02
cg18805457	6.29	2.44	9.92E-03	5.81	3.67	1.13E-01	7.66	6.43	2.33E-01	9.76	4.61	3.41E-02	-0.86	6.65	8.97E-01
cg19244655	10.96	4.25	9.92E-03	5.66	6.45	3.81E-01	25.87	13.80	6.09E-02	11.73	8.38	1.61E-01	14.19	9.20	1.23E-01
cg17187764	-11.23	4.35	9.92E-03	-15.95	10.22	1.19E-01	-2.78	9.66	7.73E-01	-12.62	5.68	2.64E-02	-12.54	25.60	6.24E-01
cg08132815	5.43	2.11	9.93E-03	4.61	2.76	9.48E-02	8.98	4.70	5.59E-02	2.62	5.59	6.39E-01	7.65	7.75	3.23E-01
cg00522588	38.56	14.96	9.93E-03	61.89	25.98	1.72E-02	81.08	35.74	2.33E-02	18.99	10.25	6.38E-02	30.06	36.98	4.16E-01
cg00461578	-12.03	4.67	9.93E-03	-12.57	12.47	3.13E-01	17.97	20.63	3.84E-01	-13.73	5.45	1.17E-02	-14.82	17.00	3.83E-01
cg15906260	-11.41	4.43	9.93E-03	-20.90	7.40	4.71E-03	-1.80	10.78	8.67E-01	-6.86	9.17	4.55E-01	-8.42	9.03	3.51E-01
cg13501225	-25.56	9.92	9.93E-03	-30.47	11.77	9.60E-03	-0.58	45.69	9.90E-01	-56.31	36.53	1.23E-01	1.53	24.13	9.50E-01
cg26307701	-9.68	3.76	9.93E-03	-18.08	10.28	7.86E-02	-3.26	12.36	7.92E-01	-8.21	4.50	6.85E-02	-15.95	13.34	2.32E-01
cg15904664	10.48	4.07	9.94E-03	6.82	5.85	2.43E-01	8.99	9.01	3.18E-01	28.08	12.15	2.09E-02	11.01	9.08	2.25E-01
cg00972246	19.65	7.62	9.94E-03	17.76	12.15	1.44E-01	25.52	15.69	1.04E-01	20.32	17.34	2.41E-01	15.27	18.11	3.99E-01
cg27180153	15.19	5.90	9.95E-03	12.95	8.34	1.20E-01	-4.53	14.35	7.52E-01	23.04	14.48	1.11E-01	26.35	11.10	1.75E-02
cg18581663	-10.56	4.10	9.95E-03	-22.86	10.33	2.69E-02	-2.97	17.65	8.66E-01	-7.79	4.95	1.15E-01	-14.22	12.80	2.67E-01
cg17832364	-17.01	6.60	9.95E-03	-31.40	18.80	9.50E-02	2.04	30.33	9.46E-01	-17.87	7.48	1.68E-02	13.85	29.53	6.39E-01
cg15992399	22.56	8.75	9.96E-03	41.26	21.62	5.63E-02	21.58	29.16	4.59E-01	20.86	10.75	5.24E-02	0.26	30.42	9.93E-01
cg20504025	29.07	11.28	9.96E-03	55.70	31.94	8.12E-02	155.80	114.81	1.75E-01	28.93	11.69	1.33E-02	-5.21	31.20	8.67E-01
cg20924425	22.68	8.80	9.96E-03	28.81	19.45	1.39E-01	49.47	25.71	5.44E-02	13.37	11.34	2.38E-01	38.73	31.97	2.26E-01
cg00436663	32.63	12.66	9.96E-03	27.38	15.38	7.51E-02	28.12	41.54	4.99E-01	81.36	62.89	1.96E-01	43.26	29.14	1.38E-01
cg20414505	21.40	8.31	9.96E-03	29.62	18.18	1.03E-01	56.71	24.55	2.09E-02	12.17	8.90	1.71E-01	24.37	27.00	3.67E-01
cg24398318	25.14	9.76	9.96E-03	42.06	29.88	1.59E-01	21.36	116.41	8.54E-01	26.22	10.84	1.56E-02	-9.30	35.21	7.92E-01
cg26406292	-11.83	4.59	9.97E-03	-17.04	6.47	8.48E-03	-12.44	11.41	2.75E-01	-13.04	13.88	3.48E-01	0.76	9.64	9.37E-01
cg13509934	-18.65	7.24	9.97E-03	-29.81	13.19	2.38E-02	-2.13	22.25	9.24E-01	-8.56	12.65	4.99E-01	-24.97	14.02	7.49E-02
cg03509039	41.66	16.17	9.97E-03	34.21	26.71	2.00E-01	49.90	52.00	3.37E-01	43.39	24.10	7.17E-02	54.86	54.82	3.17E-01
cg14009440	14.86	5.77	9.98E-03	15.94	8.40	5.77E-02	23.73	12.43	5.63E-02	16.16	16.16	3.17E-01	0.94	13.40	9.44E-01
cg02986801	22.87	8.88	9.98E-03	33.96	14.78	2.16E-02	19.35	15.88	2.23E-01	32.70	22.70	1.50E-01	-2.37	21.26	9.11E-01
cg23188121	-44.09	17.11	9.99E-03	-34.85	52.16	5.04E-01	-123.47	166.47	4.58E-01	-37.45	19.23	5.15E-02	-104.79	57.27	6.73E-02
cg00891025	20.79	8.07	9.99E-03	24.37	13.06	6.20E-02	17.80	14.11	2.07E-01	6.86	19.38	7.23E-01	37.93	23.50	1.07E-01
cg24928053	-20.12	7.81	9.99E-03	-37.68	15.05	1.23E-02	-16.75	16.88	3.21E-01	-20.63	9.95	3.81E-02	11.49	22.08	6.03E-01
cg21379004	21.49	8.34	9.99E-03	24.66	16.97	1.46E-01	11.70	55.36	8.33E-01	15.71	10.50	1.35E-01	51.14	25.77	4.73E-02
cg18952026	23.23	9.02	9.99E-03	25.30	13.17	5.47E-02	46.71	24.73	5.89E-02	10.85	26.44	6.82E-01	13.81	16.98	4.16E-01

cg01586116	11.52	4.47	9.99E-03	16.70	7.15	1.95E-02	12.99	10.66	2.23E-01	20.27	21.54	3.47E-01	4.69	7.16	5.13E-01
cg01081303	-11.00	4.27	9.99E-03	-9.83	11.56	3.95E-01	-22.10	25.02	3.77E-01	-12.51	4.91	1.09E-02	5.66	15.26	7.10E-01
cg19690494	20.30	7.88	1.00E-02	26.72	18.68	1.53E-01	3.14	28.06	9.11E-01	20.27	9.63	3.53E-02	23.51	29.15	4.20E-01
cg23396732	-15.27	5.93	1.00E-02	-6.10	15.42	6.93E-01	-26.39	30.06	3.80E-01	-16.34	7.01	1.97E-02	-16.88	18.97	3.74E-01
cg02414175	32.52	12.63	1.00E-02	43.15	16.27	8.01E-03	69.15	32.66	3.43E-02	-3.70	34.44	9.14E-01	14.89	21.04	4.79E-01
cg01012244	33.99	13.20	1.00E-02	29.47	18.25	1.06E-01	23.25	30.53	4.46E-01	30.30	43.64	4.88E-01	57.60	29.57	5.14E-02
cg14898260	34.74	13.49	1.00E-02	43.14	31.75	1.74E-01	-36.17	61.71	5.58E-01	37.08	16.43	2.41E-02	37.73	43.04	3.81E-01
cg13649658	23.19	9.00	1.00E-02	11.87	17.13	4.88E-01	16.77	19.64	3.93E-01	30.71	14.52	3.44E-02	35.54	25.13	1.57E-01
cg06401414	-7.80	3.03	1.00E-02	-9.68	4.16	1.99E-02	-2.17	7.12	7.61E-01	-10.17	9.37	2.78E-01	-6.59	7.09	3.52E-01
cg03453781	-10.50	4.08	1.00E-02	-12.39	8.78	1.58E-01	-2.89	16.54	8.61E-01	-9.76	5.28	6.45E-02	-14.35	11.40	2.08E-01
cg17722129	-6.05	2.35	1.00E-02	-9.67	4.60	3.57E-02	-6.16	7.26	3.96E-01	-6.22	3.23	5.44E-02	3.64	7.15	6.10E-01
cg24001070	46.48	18.05	1.00E-02	39.99	22.05	6.97E-02	-42.89	100.54	6.70E-01	139.94	77.29	7.02E-02	55.27	36.61	1.31E-01
cg16112844	27.04	10.50	1.00E-02	27.86	31.78	3.81E-01	17.04	139.38	9.03E-01	28.91	11.65	1.31E-02	5.89	38.86	8.80E-01
cg08746138	-46.18	17.93	1.00E-02	-30.99	23.38	1.85E-01	-106.56	50.15	3.36E-02	-77.90	66.54	2.42E-01	-41.04	39.03	2.93E-01
cg10978355	10.68	4.15	1.00E-02	12.58	5.86	3.17E-02	14.63	9.29	1.15E-01	4.35	11.59	7.07E-01	5.23	10.03	6.02E-01
cg02116856	-13.94	5.42	1.00E-02	-20.50	12.33	9.63E-02	-19.94	22.06	3.66E-01	-8.49	7.19	2.38E-01	-22.05	12.76	8.39E-02
cg11306628	-26.11	10.14	1.00E-02	-28.52	14.84	5.45E-02	-31.43	31.97	3.26E-01	-10.91	30.69	7.22E-01	-26.11	17.86	1.44E-01
cg05544807	14.40	5.59	1.00E-02	22.65	8.22	5.87E-03	9.20	11.35	4.17E-01	13.09	18.38	4.76E-01	2.34	12.44	8.51E-01
cg24182329	-22.23	8.64	1.00E-02	-30.76	15.91	5.31E-02	-14.54	21.36	4.96E-01	-17.25	13.69	2.08E-01	-27.34	22.81	2.31E-01
cg14173185	41.14	15.98	1.00E-02	32.60	20.59	1.13E-01	68.19	50.74	1.79E-01	-6.02	55.46	9.14E-01	70.77	34.44	3.99E-02
cg05090972	47.52	18.46	1.01E-02	46.81	22.07	3.39E-02	60.78	81.92	4.58E-01	-21.64	82.50	7.93E-01	64.04	41.39	1.22E-01
cg27256309	22.96	8.92	1.01E-02	36.65	17.12	3.23E-02	0.91	18.09	9.60E-01	31.01	15.25	4.20E-02	15.21	23.55	5.18E-01
cg21528927	9.06	3.52	1.01E-02	6.74	5.60	2.29E-01	1.97	8.05	8.07E-01	12.24	6.36	5.44E-02	21.10	10.72	4.90E-02
cg00014333	-11.59	4.50	1.01E-02	-20.32	7.59	7.39E-03	-2.40	10.04	8.11E-01	-11.95	11.32	2.91E-01	-7.13	8.39	3.95E-01
cg05057352	13.26	5.15	1.01E-02	9.65	11.66	4.08E-01	-0.20	15.00	9.89E-01	19.46	6.27	1.93E-03	-6.98	18.42	7.05E-01
cg03944501	-21.59	8.39	1.01E-02	-41.90	25.04	9.43E-02	-23.32	45.15	6.06E-01	-21.75	9.44	2.13E-02	17.17	33.29	6.06E-01
cg02703843	42.97	16.70	1.01E-02	67.04	23.38	4.13E-03	9.17	63.70	8.86E-01	17.95	58.69	7.60E-01	19.63	28.65	4.93E-01
cg09007620	20.72	8.05	1.01E-02	13.74	10.19	1.77E-01	24.74	24.87	3.20E-01	17.41	53.95	7.47E-01	36.84	16.15	2.25E-02
cg09006749	32.67	12.70	1.01E-02	36.95	17.76	3.75E-02	-29.79	43.38	4.92E-01	21.64	33.76	5.21E-01	50.70	24.81	4.10E-02
cg01217601	-11.81	4.59	1.01E-02	-18.02	8.28	2.94E-02	-5.76	11.99	6.31E-01	-11.27	6.91	1.03E-01	-4.25	14.25	7.66E-01
cg26840272	-28.02	10.89	1.01E-02	-44.37	23.54	5.94E-02	38.49	50.69	4.48E-01	-30.59	13.73	2.58E-02	-9.42	32.82	7.74E-01
cg10452522	-24.45	9.50	1.01E-02	-32.58	11.45	4.45E-03	-1.40	29.68	9.62E-01	-64.31	55.38	2.46E-01	0.10	22.42	9.97E-01
cg22702833	49.51	19.24	1.01E-02	56.27	27.32	3.94E-02	77.77	87.99	3.77E-01	22.96	53.06	6.65E-01	45.76	33.78	1.76E-01
cg20806172	-20.64	8.02	1.01E-02	-26.53	12.73	3.72E-02	-7.34	20.87	7.25E-01	-6.06	18.07	7.37E-01	-30.32	15.79	5.49E-02
cg26515805	22.16	8.62	1.01E-02	26.39	13.68	5.36E-02	-5.67	37.20	8.79E-01	25.82	27.10	3.41E-01	20.91	12.86	1.04E-01

cg18202741	-12.73	4.95	1.01E-02	-16.92	7.63	2.65E-02	-9.81	11.84	4.08E-01	-14.61	11.13	1.89E-01	-4.83	10.90	6.58E-01
cg08171937	-51.97	20.21	1.01E-02	-58.47	24.58	1.74E-02	-20.42	80.74	8.00E-01	-83.19	80.93	3.04E-01	-30.05	45.30	5.07E-01
cg25565850	17.83	6.94	1.01E-02	15.27	8.38	6.86E-02	17.81	22.28	4.24E-01	37.76	28.08	1.79E-01	21.26	17.46	2.23E-01
cg03872584	-9.80	3.81	1.01E-02	-15.01	6.16	1.48E-02	-10.01	8.44	2.36E-01	0.90	9.13	9.22E-01	-9.08	7.79	2.44E-01
cg23870903	10.07	3.92	1.01E-02	12.19	10.18	2.31E-01	19.85	15.87	2.11E-01	10.39	4.59	2.35E-02	-8.12	15.60	6.03E-01
cg16994041	5.45	2.12	1.01E-02	5.51	3.32	9.67E-02	8.04	5.81	1.66E-01	4.79	4.05	2.38E-01	4.44	4.94	3.68E-01
cg07377341	17.18	6.68	1.01E-02	19.86	12.99	1.26E-01	15.53	52.51	7.67E-01	16.66	8.40	4.71E-02	13.02	22.75	5.67E-01
cg24408436	-11.29	4.39	1.01E-02	-13.62	5.97	2.24E-02	3.03	10.92	7.81E-01	-9.05	14.46	5.31E-01	-17.52	9.74	7.21E-02
cg04618277	-19.72	7.67	1.01E-02	-9.05	14.66	5.37E-01	-10.93	20.07	5.86E-01	-34.79	12.16	4.23E-03	-9.92	17.96	5.81E-01
cg04724556	-24.84	9.66	1.01E-02	-19.66	19.13	3.04E-01	21.79	46.69	6.41E-01	-34.76	12.73	6.32E-03	-5.85	27.21	8.30E-01
cg26693681	-8.10	3.15	1.01E-02	-3.36	5.53	5.43E-01	-4.05	6.83	5.53E-01	-15.20	4.89	1.87E-03	-5.57	7.79	4.75E-01
cg08712631	13.57	5.28	1.02E-02	17.80	13.33	1.82E-01	34.47	18.43	6.14E-02	11.66	6.42	6.96E-02	0.79	18.15	9.65E-01
cg04184278	17.06	6.64	1.02E-02	25.21	15.23	9.80E-02	10.16	25.07	6.85E-01	15.46	8.16	5.83E-02	16.95	23.57	4.72E-01
cg12162353	8.41	3.27	1.02E-02	9.97	4.49	2.65E-02	3.70	6.09	5.43E-01	-1.22	12.51	9.22E-01	19.03	9.77	5.14E-02
cg27468773	13.11	5.10	1.02E-02	5.83	8.24	4.79E-01	22.89	8.35	6.10E-03	13.89	35.97	7.00E-01	9.14	10.80	3.97E-01
cg10840007	32.18	12.52	1.02E-02	28.43	27.17	2.95E-01	-2.74	40.22	9.46E-01	37.71	16.16	1.96E-02	41.71	41.76	3.18E-01
cg16423505	23.42	9.11	1.02E-02	58.44	42.28	1.67E-01	-117.41	144.66	4.17E-01	24.33	9.66	1.18E-02	-8.28	37.43	8.25E-01
cg00476955	11.55	4.49	1.02E-02	15.28	6.66	2.17E-02	12.27	11.00	2.64E-01	17.74	10.58	9.38E-02	-3.38	10.13	7.38E-01
cg14434062	12.84	5.00	1.02E-02	19.24	7.69	1.23E-02	15.69	12.39	2.05E-01	10.69	10.85	3.24E-01	-0.54	11.09	9.61E-01
cg23174201	-15.99	6.22	1.02E-02	-21.64	10.46	3.86E-02	-10.58	14.58	4.68E-01	-0.80	14.64	9.56E-01	-22.09	11.69	5.89E-02
cg02577046	23.60	9.19	1.02E-02	42.02	20.12	3.68E-02	55.09	46.70	2.38E-01	21.35	11.57	6.49E-02	-6.23	26.30	8.13E-01
cg02618355	-28.23	10.99	1.02E-02	-24.59	19.39	2.05E-01	-59.10	42.45	1.64E-01	-31.55	17.02	6.39E-02	-16.51	24.91	5.08E-01
cg23413506	12.23	4.76	1.02E-02	8.38	5.53	1.30E-01	4.60	8.72	5.98E-01	32.49	12.46	9.10E-03	15.69	8.77	7.37E-02
cg26654358	-28.98	11.28	1.02E-02	-39.73	15.35	9.67E-03	-9.63	35.54	7.86E-01	-46.44	32.81	1.57E-01	-4.40	22.99	8.48E-01
cg10297426	16.84	6.56	1.02E-02	29.08	12.79	2.30E-02	5.17	18.37	7.78E-01	14.06	9.30	1.30E-01	13.73	19.57	4.83E-01
cg11681959	35.78	13.93	1.02E-02	52.38	18.67	5.02E-03	43.03	37.52	2.51E-01	10.90	55.85	8.45E-01	0.00	28.26	1.00E+00
cg18750049	31.62	12.31	1.02E-02	31.13	16.83	6.44E-02	32.96	48.30	4.95E-01	-11.04	65.87	8.67E-01	36.18	20.38	7.59E-02
cg12949616	31.97	12.45	1.02E-02	33.33	17.05	5.07E-02	51.71	35.73	1.48E-01	16.15	32.99	6.24E-01	27.70	27.62	3.16E-01
cg26457809	7.39	2.88	1.02E-02	4.37	4.30	3.10E-01	11.22	6.94	1.06E-01	14.06	7.75	6.97E-02	6.46	5.84	2.69E-01
cg02213045	-13.00	5.06	1.02E-02	-21.16	15.02	1.59E-01	-5.31	33.49	8.74E-01	-12.56	5.68	2.70E-02	-7.15	19.28	7.11E-01
cg21596313	-45.67	17.79	1.02E-02	-54.99	24.41	2.43E-02	-37.87	40.66	3.52E-01	13.19	63.35	8.35E-01	-51.64	39.90	1.96E-01
cg06594404	12.37	4.82	1.02E-02	19.62	7.91	1.32E-02	8.24	10.82	4.46E-01	12.34	10.70	2.49E-01	4.20	10.08	6.77E-01
cg24728698	-7.77	3.02	1.02E-02	-11.16	5.35	3.72E-02	-2.87	8.18	7.26E-01	-6.68	4.73	1.58E-01	-7.97	8.19	3.31E-01
cg25622036	26.76	10.42	1.03E-02	19.10	14.64	1.92E-01	56.18	34.10	9.94E-02	88.47	47.46	6.23E-02	21.51	17.58	2.21E-01
cg08922201	-14.06	5.48	1.03E-02	-8.91	7.70	2.48E-01	-16.32	13.98	2.43E-01	-39.16	17.88	2.85E-02	-13.67	11.02	2.15E-01

cg12946514	23.88	9.30	1.03E-02	30.95	12.31	1.20E-02	8.34	29.07	7.74E-01	-37.80	42.82	3.77E-01	25.55	17.59	1.46E-01
cg10187879	22.15	8.63	1.03E-02	23.43	12.62	6.34E-02	35.03	27.24	1.98E-01	9.12	29.62	7.58E-01	19.90	14.65	1.74E-01
cg12409874	21.02	8.19	1.03E-02	28.12	14.04	4.51E-02	3.92	35.78	9.13E-01	18.83	11.55	1.03E-01	16.99	25.37	5.03E-01
cg27408184	-18.31	7.13	1.03E-02	-25.91	11.97	3.05E-02	-16.49	15.33	2.82E-01	-14.76	17.59	4.02E-01	-11.80	13.88	3.95E-01
cg00151914	18.22	7.10	1.03E-02	29.17	12.17	1.65E-02	16.91	21.54	4.32E-01	15.64	20.07	4.36E-01	10.57	10.88	3.31E-01
cg25649641	15.27	5.95	1.03E-02	30.92	13.18	1.90E-02	4.80	20.98	8.19E-01	11.25	10.56	2.87E-01	12.58	9.41	1.81E-01
cg24059623	-10.31	4.02	1.03E-02	-18.87	7.79	1.54E-02	-4.19	9.87	6.71E-01	-4.49	5.66	4.28E-01	-17.67	9.07	5.13E-02
cg26361892	35.06	13.66	1.03E-02	-19.42	42.21	6.45E-01	20.61	171.27	9.04E-01	42.70	15.27	5.18E-03	31.53	45.70	4.90E-01
cg11079989	33.97	13.24	1.03E-02	25.65	15.23	9.22E-02	149.16	100.43	1.38E-01	93.82	66.34	1.57E-01	44.15	30.57	1.49E-01
cg09186979	19.98	7.79	1.03E-02	34.94	23.02	1.29E-01	5.90	29.64	8.42E-01	17.75	8.97	4.77E-02	35.14	31.16	2.59E-01
cg08708790	-14.63	5.70	1.03E-02	-19.74	9.15	3.09E-02	-19.09	13.82	1.67E-01	-6.36	11.95	5.94E-01	-10.61	12.33	3.90E-01
cg18480946	-11.05	4.30	1.03E-02	-14.61	7.60	5.45E-02	-2.58	13.69	8.51E-01	-9.54	6.36	1.34E-01	-14.20	12.30	2.48E-01
cg23564243	12.61	4.91	1.03E-02	19.72	7.38	7.58E-03	14.45	13.22	2.74E-01	3.28	9.87	7.39E-01	6.24	11.89	6.00E-01
cg00406796	-28.42	11.07	1.03E-02	-17.90	31.18	5.66E-01	-22.50	112.45	8.41E-01	-30.61	12.30	1.28E-02	-21.07	47.90	6.60E-01
cg26386846	27.95	10.89	1.03E-02	49.87	30.24	9.91E-02	8.53	144.86	9.53E-01	23.01	12.11	5.74E-02	50.64	46.16	2.73E-01
cg02798066	36.72	14.31	1.03E-02	29.95	43.33	4.90E-01	-119.71	120.21	3.19E-01	43.54	16.14	6.97E-03	10.10	47.62	8.32E-01
cg03224364	41.77	16.28	1.03E-02	49.96	48.07	2.99E-01	57.19	208.27	7.84E-01	45.05	18.02	1.24E-02	-17.27	64.95	7.90E-01
cg10025432	-16.53	6.44	1.03E-02	-21.58	10.54	4.06E-02	-18.24	14.42	2.06E-01	-3.18	14.17	8.23E-01	-18.97	13.74	1.68E-01
cg14767442	19.52	7.61	1.03E-02	24.01	14.45	9.67E-02	5.01	25.74	8.46E-01	18.20	11.84	1.24E-01	22.05	16.10	1.71E-01
cg14950321	-10.98	4.28	1.03E-02	-8.47	5.90	1.51E-01	-12.06	10.46	2.49E-01	-22.41	12.45	7.18E-02	-9.86	9.86	3.18E-01
cg01684429	22.85	8.91	1.03E-02	40.07	18.44	2.98E-02	5.38	28.80	8.52E-01	22.04	12.11	6.88E-02	8.23	24.68	7.39E-01
cg13989999	-10.96	4.27	1.03E-02	-11.18	5.81	5.43E-02	-16.23	9.74	9.56E-02	-3.47	12.28	7.78E-01	-9.38	11.17	4.01E-01
cg19308436	-14.96	5.83	1.03E-02	-3.99	16.87	8.13E-01	11.65	32.23	7.18E-01	-18.06	6.57	6.00E-03	-10.65	23.69	6.53E-01
cg03143365	-13.32	5.19	1.03E-02	-16.58	7.08	1.92E-02	-7.13	12.20	5.59E-01	13.15	33.16	6.92E-01	-13.36	10.24	1.92E-01
cg03571604	27.53	10.73	1.03E-02	52.93	19.76	7.41E-03	-8.58	80.92	9.16E-01	19.93	13.96	1.54E-01	3.15	34.47	9.27E-01
cg04275506	-9.10	3.55	1.03E-02	-4.45	6.81	5.14E-01	-9.80	9.85	3.20E-01	-11.55	5.64	4.07E-02	-10.11	7.87	1.99E-01
cg09745087	39.63	15.45	1.03E-02	37.79	22.00	8.59E-02	97.04	63.87	1.29E-01	4.55	45.23	9.20E-01	44.59	26.83	9.65E-02
cg19718450	-6.54	2.55	1.03E-02	-9.41	3.77	1.24E-02	-4.09	5.27	4.38E-01	2.11	10.48	8.40E-01	-5.61	5.12	2.73E-01
cg24966702	18.87	7.36	1.03E-02	40.02	20.44	5.03E-02	9.08	25.59	7.23E-01	13.61	8.96	1.29E-01	33.14	21.83	1.29E-01
cg14281092	-23.26	9.07	1.03E-02	-13.82	25.22	5.84E-01	-28.87	37.90	4.46E-01	-28.37	10.56	7.20E-03	14.82	33.03	6.54E-01
cg12386808	-11.62	4.53	1.03E-02	-22.79	14.50	1.16E-01	-28.73	29.30	3.27E-01	-10.01	5.11	5.00E-02	-9.12	15.02	5.44E-01
cg16786808	5.38	2.10	1.03E-02	2.88	3.15	3.62E-01	9.44	3.63	9.30E-03	2.03	8.79	8.18E-01	5.02	5.12	3.27E-01
cg04479713	11.39	4.44	1.03E-02	13.40	6.78	4.80E-02	8.48	8.24	3.03E-01	11.57	13.60	3.95E-01	11.18	10.67	2.95E-01
cg20007894	-9.39	3.66	1.03E-02	-12.57	8.03	1.17E-01	-0.86	12.83	9.46E-01	-8.77	4.71	6.27E-02	-13.24	11.23	2.38E-01
cg25949513	29.86	11.64	1.03E-02	71.73	31.16	2.13E-02	47.22	150.49	7.54E-01	19.03	9.98	5.64E-02	51.90	32.68	1.12E-01

cg08584627	30.87	12.04	1.03E-02	20.79	17.69	2.40E-01	33.71	29.45	2.52E-01	46.79	34.16	1.71E-01	39.87	24.28	1.01E-01
cg21852721	-49.97	19.49	1.03E-02	-47.84	26.06	6.64E-02	-56.41	60.86	3.54E-01	-69.63	53.74	1.95E-01	-40.04	42.84	3.50E-01
cg25297790	-11.53	4.50	1.03E-02	-4.30	11.95	7.19E-01	9.11	25.47	7.20E-01	-14.94	5.23	4.26E-03	-1.72	15.21	9.10E-01
cg11031737	-16.42	6.40	1.03E-02	-24.40	12.78	5.62E-02	-0.11	17.56	9.95E-01	-15.85	9.04	7.97E-02	-20.31	18.89	2.82E-01
cg13840239	-11.28	4.40	1.03E-02	-26.72	10.33	9.73E-03	-5.12	13.52	7.05E-01	-7.57	5.79	1.91E-01	-11.27	11.99	3.47E-01
cg15957004	31.63	12.33	1.03E-02	43.98	16.64	8.21E-03	33.37	22.45	1.37E-01	-41.57	43.50	3.39E-01	30.89	24.63	2.10E-01
cg07159958	11.73	4.57	1.03E-02	9.02	7.82	2.49E-01	14.38	10.67	1.78E-01	14.22	9.54	1.36E-01	11.19	9.26	2.27E-01
cg24647031	14.98	5.84	1.03E-02	36.13	15.96	2.36E-02	22.61	34.82	5.16E-01	12.52	6.76	6.39E-02	1.57	19.47	9.36E-01
cg02453013	5.95	2.32	1.04E-02	4.13	2.91	1.55E-01	9.86	4.18	1.85E-02	-0.97	6.02	8.72E-01	11.63	6.03	5.36E-02
cg17055274	-43.21	16.85	1.04E-02	-52.92	25.54	3.83E-02	-58.15	49.00	2.35E-01	-3.69	29.11	8.99E-01	-85.35	41.19	3.82E-02
cg25515997	19.36	7.55	1.04E-02	4.56	18.99	8.10E-01	-16.88	39.60	6.70E-01	25.82	8.95	3.91E-03	9.25	24.78	7.09E-01
cg25284089	19.67	7.67	1.04E-02	19.81	16.90	2.41E-01	19.93	15.98	2.12E-01	20.03	11.16	7.27E-02	16.85	25.60	5.11E-01
cg12926529	16.13	6.29	1.04E-02	10.96	16.86	5.16E-01	4.73	20.80	8.20E-01	17.53	7.49	1.92E-02	28.62	25.19	2.56E-01
cg27494470	-12.12	4.73	1.04E-02	-10.96	8.01	1.71E-01	-8.23	11.56	4.76E-01	-16.14	8.91	7.00E-02	-11.75	10.53	2.64E-01
cg02498422	25.94	10.12	1.04E-02	38.15	20.33	6.05E-02	-4.23	20.00	8.32E-01	33.07	15.32	3.09E-02	41.58	31.40	1.86E-01
cg08089041	-24.62	9.60	1.04E-02	-19.66	13.54	1.46E-01	-33.22	32.48	3.06E-01	-37.58	31.87	2.38E-01	-26.39	17.02	1.21E-01
cg03964111	-6.23	2.43	1.04E-02	-3.64	3.27	2.65E-01	-11.32	6.11	6.41E-02	-13.27	8.90	1.36E-01	-6.69	5.24	2.02E-01
cg19758881	-12.92	5.04	1.04E-02	-7.06	7.18	3.25E-01	-17.13	12.33	1.65E-01	-19.02	16.80	2.58E-01	-19.46	10.08	5.35E-02
cg10105681	11.28	4.40	1.04E-02	3.99	9.47	6.73E-01	18.09	13.26	1.72E-01	18.49	7.17	9.88E-03	4.89	8.07	5.44E-01
cg01534145	25.93	10.12	1.04E-02	17.80	14.18	2.10E-01	27.48	28.39	3.33E-01	72.74	37.82	5.44E-02	27.94	18.69	1.35E-01
cg03683132	14.66	5.72	1.04E-02	0.86	13.99	9.51E-01	4.59	24.30	8.50E-01	16.15	6.94	2.00E-02	33.32	18.18	6.68E-02
cg18033335	23.78	9.28	1.04E-02	3.15	18.40	8.64E-01	31.10	17.01	6.75E-02	25.91	17.34	1.35E-01	39.01	23.07	9.09E-02
cg12054981	-5.63	2.20	1.04E-02	-7.92	3.23	1.43E-02	-1.54	4.26	7.17E-01	-4.48	6.80	5.10E-01	-6.50	5.35	2.24E-01
cg24115221	28.68	11.19	1.04E-02	33.39	15.57	3.20E-02	12.66	39.85	7.51E-01	53.86	53.83	3.17E-01	22.44	18.63	2.28E-01
cg15278374	-8.05	3.14	1.04E-02	-7.42	4.61	1.07E-01	-4.99	7.97	5.31E-01	-11.75	9.19	2.01E-01	-9.35	6.14	1.28E-01
cg06954520	-8.91	3.48	1.04E-02	-10.13	6.51	1.19E-01	-4.84	11.01	6.60E-01	-6.80	5.05	1.79E-01	-16.39	9.25	7.64E-02
cg11801110	-13.85	5.41	1.04E-02	-19.82	8.31	1.70E-02	-10.78	11.51	3.49E-01	-8.64	16.52	6.01E-01	-8.65	10.83	4.24E-01
cg23301492	-43.43	16.95	1.04E-02	-38.92	40.56	3.37E-01	-375.26	203.99	6.58E-02	-43.19	19.98	3.07E-02	-29.98	54.01	5.79E-01
cg10192196	-21.51	8.40	1.04E-02	-25.27	12.08	3.64E-02	-37.23	23.62	1.15E-01	-17.49	21.63	4.19E-01	-8.16	17.16	6.34E-01
cg18352935	-11.58	4.52	1.04E-02	-10.68	9.25	2.48E-01	-6.61	13.35	6.20E-01	-10.28	6.36	1.06E-01	-21.64	11.97	7.06E-02
cg16487292	-37.54	14.65	1.04E-02	-26.39	21.72	2.24E-01	-7.74	36.53	8.32E-01	-60.96	30.24	4.38E-02	-66.80	37.93	7.82E-02
cg18906520	18.23	7.12	1.04E-02	20.34	11.33	7.25E-02	23.36	13.62	8.63E-02	23.61	17.21	1.70E-01	-1.29	17.70	9.42E-01
cg02372856	16.84	6.57	1.04E-02	23.27	17.14	1.75E-01	12.21	25.19	6.28E-01	15.09	7.70	4.99E-02	28.56	27.96	3.07E-01
cg23641722	-10.84	4.23	1.04E-02	-15.87	9.44	9.27E-02	-33.51	21.08	1.12E-01	-6.60	5.17	2.02E-01	-21.19	14.22	1.36E-01
cg09750084	8.13	3.18	1.04E-02	8.54	4.37	5.08E-02	16.00	7.96	4.44E-02	-0.98	6.52	8.80E-01	12.05	7.67	1.16E-01

cg14136506	-28.99	11.32	1.04E-02	-9.50	31.00	7.59E-01	-80.17	146.37	5.84E-01	-32.18	12.68	1.12E-02	-25.12	44.54	5.73E-01
cg04807470	16.99	6.64	1.04E-02	10.43	18.76	5.78E-01	-3.15	76.04	9.67E-01	19.25	7.81	1.37E-02	12.46	17.43	4.75E-01
cg01942423	10.07	3.93	1.04E-02	10.04	8.89	2.59E-01	1.38	16.52	9.34E-01	11.32	4.74	1.70E-02	4.13	15.90	7.95E-01
cg27424148	21.54	8.41	1.04E-02	22.05	11.17	4.84E-02	48.49	31.89	1.28E-01	-38.58	51.09	4.50E-01	19.95	14.50	1.69E-01
cg04962027	17.55	6.85	1.04E-02	5.34	11.31	6.37E-01	-1.35	35.17	9.69E-01	30.03	9.91	2.45E-03	13.74	16.78	4.13E-01
cg13000789	-16.52	6.45	1.04E-02	-25.69	17.30	1.38E-01	-62.81	33.25	5.89E-02	-12.57	7.44	9.13E-02	-15.83	24.02	5.10E-01
cg02969343	-73.46	28.69	1.04E-02	-110.64	37.54	3.21E-03	100.11	188.01	5.94E-01	-31.80	69.84	6.49E-01	-26.04	60.57	6.67E-01
cg15330117	26.72	10.43	1.04E-02	70.87	34.39	3.93E-02	-1.06	47.45	9.82E-01	24.61	12.12	4.23E-02	16.91	30.35	5.77E-01
cg23756272	9.18	3.59	1.05E-02	11.27	5.11	2.74E-02	9.47	7.75	2.22E-01	14.27	9.51	1.33E-01	-2.81	9.21	7.61E-01
cg07356555	16.35	6.38	1.05E-02	9.38	9.34	3.15E-01	12.79	12.71	3.14E-01	24.42	18.42	1.85E-01	36.27	15.96	2.31E-02
cg07268734	-13.26	5.18	1.05E-02	-17.72	10.68	9.69E-02	5.21	15.11	7.30E-01	-13.06	7.03	6.32E-02	-25.03	16.03	1.18E-01
cg19035004	34.33	13.41	1.05E-02	6.76	34.66	8.45E-01	142.80	194.84	4.64E-01	34.75	15.23	2.25E-02	80.74	50.37	1.09E-01
cg23122650	-8.24	3.22	1.05E-02	-10.46	6.28	9.58E-02	-12.99	9.61	1.76E-01	-4.97	4.69	2.89E-01	-10.92	8.16	1.81E-01
cg26986911	27.93	10.91	1.05E-02	29.01	37.21	4.36E-01	-44.95	130.10	7.30E-01	23.75	11.98	4.74E-02	78.82	39.44	4.57E-02
cg27331316	-15.59	6.09	1.05E-02	-12.80	12.18	2.93E-01	-14.94	15.93	3.48E-01	-16.89	9.40	7.25E-02	-16.93	14.19	2.33E-01
cg03368787	33.58	13.12	1.05E-02	49.98	19.21	9.26E-03	8.68	29.20	7.66E-01	-2.60	43.04	9.52E-01	36.68	26.86	1.72E-01
cg02637031	-16.22	6.34	1.05E-02	-22.29	10.11	2.75E-02	-22.73	14.69	1.22E-01	-0.87	12.75	9.46E-01	-17.36	15.18	2.53E-01
cg04391356	28.51	11.14	1.05E-02	31.66	25.01	2.06E-01	-82.32	118.61	4.88E-01	24.89	13.10	5.74E-02	71.27	42.28	9.18E-02
cg27177158	18.62	7.28	1.05E-02	23.95	10.51	2.28E-02	23.93	27.86	3.90E-01	31.17	32.59	3.39E-01	9.85	11.46	3.91E-01
cg07237926	-10.15	3.96	1.05E-02	-8.53	5.21	1.02E-01	-14.50	9.93	1.44E-01	-14.49	12.25	2.37E-01	-8.80	9.99	3.78E-01
cg21961970	20.30	7.93	1.05E-02	32.41	19.15	9.06E-02	40.47	37.13	2.76E-01	15.41	9.34	9.90E-02	28.94	32.01	3.66E-01
cg15223579	20.71	8.09	1.05E-02	15.58	10.15	1.25E-01	45.68	23.48	5.17E-02	-18.35	31.65	5.62E-01	28.67	14.16	4.30E-02
cg10960055	-4.39	1.71	1.05E-02	-5.96	2.43	1.43E-02	-4.80	4.27	2.60E-01	-6.07	4.79	2.05E-01	0.57	3.70	8.77E-01
cg03962019	-21.20	8.28	1.05E-02	-24.70	14.77	9.44E-02	-26.18	15.49	9.10E-02	-8.28	18.42	6.53E-01	-21.63	18.66	2.46E-01
cg01054502	-60.54	23.66	1.05E-02	-17.11	64.57	7.91E-01	-538.91	290.11	6.32E-02	-60.19	22.16	6.60E-03	-77.93	59.60	1.91E-01
cg12727680	-15.95	6.23	1.05E-02	-21.00	13.77	1.27E-01	-16.91	25.96	5.15E-01	-9.99	7.82	2.02E-01	-42.23	19.47	3.01E-02
cg09100654	12.67	4.95	1.05E-02	20.96	8.66	1.55E-02	18.65	12.50	1.36E-01	7.76	9.86	4.32E-01	3.52	9.65	7.15E-01
cg21060429	15.52	6.07	1.05E-02	7.34	10.75	4.95E-01	24.63	18.24	1.77E-01	17.31	8.64	4.51E-02	24.64	21.68	2.56E-01
cg23166590	10.53	4.11	1.05E-02	16.81	8.85	5.77E-02	8.83	11.72	4.51E-01	8.50	5.36	1.13E-01	11.24	15.49	4.68E-01
cg27139077	-36.30	14.19	1.05E-02	-37.17	34.76	2.85E-01	-69.70	182.70	7.03E-01	-31.16	16.33	5.64E-02	-84.75	52.56	1.07E-01
cg05662684	50.04	19.56	1.05E-02	71.07	26.52	7.36E-03	9.49	69.54	8.91E-01	43.44	43.22	3.15E-01	10.09	47.12	8.30E-01
cg04681879	21.45	8.38	1.05E-02	25.73	21.01	2.21E-01	13.25	22.61	5.58E-01	17.85	10.67	9.42E-02	52.79	28.72	6.61E-02
cg03138405	41.31	16.15	1.05E-02	38.23	19.42	4.91E-02	31.49	72.55	6.64E-01	94.82	59.38	1.10E-01	34.11	37.50	3.63E-01
cg04572085	19.31	7.55	1.05E-02	29.14	10.76	6.77E-03	26.62	17.33	1.25E-01	-2.29	23.86	9.24E-01	2.21	15.62	8.88E-01
cg19734030	-35.80	13.99	1.05E-02	-19.30	24.35	4.28E-01	42.37	255.80	8.68E-01	-48.57	18.11	7.31E-03	-7.84	53.13	8.83E-01

cg15454110	-12.01	4.69	1.05E-02	-17.51	7.80	2.48E-02	-7.84	11.49	4.95E-01	-10.78	8.94	2.28E-01	-7.12	10.60	5.02E-01
cg17168836	-14.16	5.54	1.05E-02	-12.10	7.64	1.13E-01	-22.46	14.19	1.13E-01	-12.95	16.52	4.33E-01	-13.93	12.06	2.48E-01
cg00397422	14.90	5.82	1.05E-02	25.90	12.40	3.68E-02	10.91	15.68	4.87E-01	13.71	7.90	8.27E-02	2.42	18.52	8.96E-01
cg26312493	-55.02	21.51	1.05E-02	-62.17	34.72	7.34E-02	-27.12	135.09	8.41E-01	-53.45	37.61	1.55E-01	-49.24	41.87	2.40E-01
cg23409168	-12.57	4.91	1.05E-02	2.22	13.20	8.66E-01	-33.36	22.44	1.37E-01	-12.44	5.76	3.09E-02	-25.81	16.69	1.22E-01
cg15056687	-74.01	28.93	1.05E-02	-76.05	80.06	3.42E-01	-474.26	369.85	2.00E-01	-66.28	33.40	4.72E-02	-101.28	86.11	2.40E-01
cg23415995	8.05	3.15	1.05E-02	5.47	4.22	1.94E-01	12.64	8.02	1.15E-01	12.08	9.45	2.01E-01	9.61	7.45	1.97E-01
cg20195957	15.34	6.00	1.05E-02	38.35	14.60	8.60E-03	10.92	38.91	7.79E-01	10.93	6.83	1.10E-01	7.46	25.24	7.67E-01
cg06511389	-45.53	17.80	1.05E-02	-89.14	49.57	7.21E-02	74.22	267.66	7.82E-01	-43.84	20.41	3.17E-02	-9.52	54.75	8.62E-01
cg24090068	-38.72	15.14	1.05E-02	-0.43	44.01	9.92E-01	-144.15	162.55	3.75E-01	-41.52	16.90	1.40E-02	-58.19	57.10	3.08E-01
cg13425637	34.80	13.61	1.05E-02	32.18	56.73	5.71E-01	-244.24	205.48	2.35E-01	34.56	14.50	1.71E-02	62.47	56.90	2.72E-01
cg07671221	19.00	7.43	1.05E-02	19.04	10.06	5.84E-02	3.01	16.99	8.59E-01	66.84	38.51	8.26E-02	24.52	15.60	1.16E-01
cg01963134	18.98	7.42	1.05E-02	27.03	26.18	3.02E-01	4.73	55.22	9.32E-01	21.40	8.30	9.93E-03	-3.77	23.21	8.71E-01
cg16340152	-20.39	7.97	1.06E-02	-13.02	16.64	4.34E-01	-39.71	42.50	3.50E-01	-23.56	9.87	1.69E-02	-7.48	27.87	7.89E-01
cg06747288	-14.96	5.85	1.06E-02	-14.46	8.38	8.44E-02	-13.22	14.31	3.56E-01	-16.79	14.13	2.35E-01	-16.24	14.05	2.48E-01
cg27586249	20.60	8.06	1.06E-02	24.30	12.41	5.03E-02	10.35	17.65	5.58E-01	8.23	9.91	4.06E-01	43.99	14.69	2.75E-03
cg25539045	14.00	5.48	1.06E-02	22.76	12.16	6.13E-02	16.15	21.09	4.44E-01	13.05	7.20	6.99E-02	4.92	14.12	7.28E-01
cg20507228	8.05	3.15	1.06E-02	9.30	4.25	2.87E-02	12.35	7.18	8.53E-02	7.33	9.75	4.52E-01	-1.26	8.01	8.75E-01
cg01881265	25.10	9.82	1.06E-02	14.32	16.95	3.98E-01	57.10	19.13	2.84E-03	15.00	11.64	1.97E-01	27.32	26.90	3.10E-01
cg09142313	14.98	5.86	1.06E-02	28.92	14.95	5.31E-02	5.98	17.93	7.39E-01	13.71	8.65	1.13E-01	12.84	11.05	2.45E-01
cg24060890	-9.09	3.56	1.06E-02	-11.43	7.34	1.20E-01	3.33	10.97	7.62E-01	-12.25	4.81	1.09E-02	-0.52	10.60	9.61E-01
cg03289833	-13.71	5.37	1.06E-02	-17.65	15.84	2.65E-01	-13.17	28.21	6.41E-01	-15.31	6.02	1.10E-02	17.41	22.96	4.48E-01
cg00019877	-17.79	6.96	1.06E-02	-22.49	17.46	1.98E-01	13.82	21.18	5.14E-01	-18.27	7.12	1.03E-02	-38.46	20.57	6.16E-02
cg04217177	4.67	1.83	1.06E-02	1.97	3.07	5.21E-01	3.95	3.95	3.18E-01	7.55	3.40	2.66E-02	6.62	4.82	1.69E-01
cg12172441	11.68	4.57	1.06E-02	6.66	6.76	3.24E-01	6.42	15.99	6.88E-01	24.89	11.12	2.52E-02	13.37	8.46	1.14E-01
cg05791881	-21.81	8.54	1.06E-02	-16.09	16.76	3.37E-01	17.54	44.28	6.92E-01	-22.12	10.86	4.17E-02	-53.76	29.06	6.43E-02
cg04516896	21.27	8.32	1.06E-02	16.21	18.60	3.83E-01	22.87	21.67	2.91E-01	16.45	11.34	1.47E-01	50.96	24.71	3.92E-02
cg01955025	14.16	5.54	1.06E-02	13.38	7.66	8.05E-02	23.17	11.83	5.02E-02	-5.97	18.39	7.45E-01	15.75	13.62	2.47E-01
cg00749323	20.57	8.05	1.06E-02	27.60	18.16	1.29E-01	-2.47	45.73	9.57E-01	16.79	9.54	7.83E-02	54.51	32.92	9.77E-02
cg11170318	8.79	3.44	1.06E-02	9.02	4.83	6.16E-02	4.77	7.59	5.30E-01	11.24	11.53	3.30E-01	11.27	7.74	1.45E-01
cg08520423	19.29	7.55	1.06E-02	28.89	10.37	5.33E-03	17.71	21.70	4.15E-01	14.17	30.78	6.45E-01	3.38	14.06	8.10E-01
cg05657090	24.66	9.65	1.06E-02	31.37	17.80	7.80E-02	13.51	23.67	5.68E-01	11.28	16.61	4.97E-01	46.44	21.47	3.06E-02
cg03550727	-14.63	5.73	1.06E-02	-24.85	9.78	1.11E-02	-8.01	13.35	5.49E-01	-12.33	12.04	3.06E-01	-7.48	11.53	5.16E-01
cg14158712	-76.79	30.05	1.06E-02	-134.93	46.34	3.59E-03	97.88	234.62	6.77E-01	-41.23	17.55	1.88E-02	-108.83	54.09	4.42E-02
cg11856822	18.27	7.15	1.06E-02	37.91	19.98	5.78E-02	12.83	19.60	5.13E-01	17.46	9.63	6.97E-02	11.09	16.51	5.02E-01

cg07812715	12.36	4.84	1.06E-02	12.79	7.15	7.37E-02	16.78	10.60	1.13E-01	3.31	12.30	7.88E-01	13.91	11.42	2.23E-01
cg07998213	11.79	4.61	1.06E-02	13.28	7.78	8.79E-02	15.39	10.27	1.34E-01	19.12	11.22	8.83E-02	2.79	8.77	7.51E-01
cg13488395	-28.91	11.32	1.06E-02	-43.00	44.58	3.35E-01	24.58	183.06	8.93E-01	-30.56	12.11	1.16E-02	8.04	47.02	8.64E-01
cg11024341	39.48	15.46	1.06E-02	29.48	19.91	1.39E-01	53.24	63.39	4.01E-01	50.10	44.94	2.65E-01	57.48	32.98	8.14E-02
cg05553829	-8.49	3.32	1.06E-02	-9.22	4.81	5.50E-02	-5.81	7.91	4.62E-01	-13.31	8.43	1.14E-01	-5.18	7.63	4.97E-01
cg27104809	20.31	7.95	1.06E-02	29.58	22.56	1.90E-01	-1.80	74.62	9.81E-01	18.20	8.96	4.22E-02	30.32	28.75	2.92E-01
cg25241559	-22.88	8.96	1.06E-02	-26.05	11.47	2.31E-02	-4.63	37.69	9.02E-01	-51.81	24.45	3.41E-02	-0.79	19.29	9.67E-01
cg02535674	13.86	5.43	1.06E-02	12.29	8.72	1.59E-01	-13.68	38.02	7.19E-01	17.97	7.59	1.78E-02	2.26	19.15	9.06E-01
cg16028336	-25.38	9.94	1.06E-02	-5.60	21.01	7.90E-01	8.14	47.80	8.65E-01	-37.28	12.60	3.09E-03	-11.59	29.75	6.97E-01
cg20626964	24.28	9.51	1.06E-02	27.00	14.37	6.02E-02	12.89	20.61	5.32E-01	30.80	27.61	2.65E-01	26.26	19.78	1.84E-01
cg25179685	-12.96	5.07	1.07E-02	-20.49	9.48	3.06E-02	-21.36	13.17	1.05E-01	-6.18	9.63	5.21E-01	-7.65	9.46	4.19E-01
cg17056789	16.79	6.58	1.07E-02	12.68	11.84	2.84E-01	23.42	16.04	1.44E-01	9.16	10.82	3.97E-01	36.12	16.76	3.11E-02
cg16517394	25.01	9.79	1.07E-02	29.25	12.12	1.58E-02	-0.55	31.76	9.86E-01	2.90	37.33	9.38E-01	31.51	22.91	1.69E-01
cg17772649	8.48	3.32	1.07E-02	6.61	4.24	1.19E-01	14.41	7.84	6.60E-02	20.64	10.37	4.66E-02	0.13	8.71	9.88E-01
cg26682517	-20.13	7.88	1.07E-02	-36.85	18.19	4.27E-02	-4.97	18.69	7.90E-01	-12.67	9.91	2.01E-01	-41.59	19.30	3.12E-02
cg09622504	-17.92	7.02	1.07E-02	-27.56	15.12	6.84E-02	-25.69	81.34	7.52E-01	-18.43	8.32	2.67E-02	20.07	27.36	4.63E-01
cg13775575	46.72	18.30	1.07E-02	-29.18	60.57	6.30E-01	-66.98	214.34	7.55E-01	53.19	20.42	9.19E-03	72.74	58.32	2.12E-01
cg26562691	-9.66	3.78	1.07E-02	-11.07	6.13	7.11E-02	-13.26	9.30	1.54E-01	-6.31	7.45	3.97E-01	-8.28	8.53	3.32E-01
cg19602920	-27.84	10.90	1.07E-02	-11.04	29.83	7.11E-01	-120.47	124.76	3.34E-01	-29.10	12.34	1.83E-02	-34.94	39.10	3.71E-01
cg21853806	23.41	9.17	1.07E-02	38.49	16.47	1.95E-02	15.42	17.04	3.65E-01	17.07	17.87	3.39E-01	18.38	24.73	4.57E-01
cg25149253	-13.48	5.28	1.07E-02	-7.15	9.98	4.73E-01	-29.04	17.51	9.73E-02	-16.23	8.07	4.43E-02	-9.40	11.78	4.25E-01
cg13765278	-7.30	2.86	1.07E-02	-8.31	4.05	4.02E-02	-2.77	6.47	6.69E-01	-15.86	10.84	1.44E-01	-6.40	5.88	2.76E-01
cg13519191	-10.18	3.99	1.07E-02	-18.53	7.66	1.55E-02	-13.74	12.09	2.56E-01	-5.02	5.94	3.98E-01	-8.23	9.67	3.95E-01
cg13719287	-11.13	4.36	1.07E-02	-15.37	6.29	1.46E-02	-4.77	10.66	6.54E-01	3.19	14.05	8.20E-01	-12.72	8.62	1.40E-01
cg20704159	8.36	3.27	1.07E-02	10.18	4.31	1.81E-02	-0.72	7.92	9.28E-01	14.30	13.76	2.99E-01	9.20	7.43	2.16E-01
cg03155200	6.09	2.39	1.07E-02	5.46	3.18	8.61E-02	7.97	4.77	9.48E-02	3.08	9.28	7.40E-01	6.78	6.86	3.23E-01
cg03561416	22.12	8.67	1.07E-02	26.41	10.34	1.06E-02	36.11	12.03	2.68E-03	21.82	18.78	2.45E-01	-9.05	17.54	6.06E-01
cg18148375	-15.45	6.05	1.07E-02	-24.22	9.32	9.38E-03	5.44	15.63	7.28E-01	-17.21	13.91	2.16E-01	-11.70	12.38	3.45E-01
cg16632924	-22.95	8.99	1.07E-02	-12.36	19.58	5.28E-01	-29.90	48.86	5.41E-01	-28.39	10.94	9.46E-03	-1.98	31.72	9.50E-01
cg17826763	15.20	5.96	1.07E-02	14.49	12.79	2.57E-01	25.54	20.24	2.07E-01	16.09	7.84	4.01E-02	4.64	17.29	7.89E-01
cg00434535	-7.50	2.94	1.07E-02	-5.40	5.66	3.40E-01	-8.03	8.64	3.53E-01	-9.43	4.11	2.18E-02	-2.88	9.10	7.52E-01
cg13247398	10.78	4.22	1.07E-02	11.34	6.31	7.21E-02	12.69	8.63	1.41E-01	3.29	12.24	7.88E-01	11.70	9.61	2.24E-01
cg19255818	-19.13	7.50	1.07E-02	-15.73	16.74	3.47E-01	-8.76	21.96	6.90E-01	-20.93	9.71	3.11E-02	-28.45	25.42	2.63E-01
cg11157076	-19.37	7.59	1.07E-02	-32.04	20.04	1.10E-01	-70.13	48.74	1.50E-01	-16.83	8.76	5.47E-02	-5.29	26.59	8.42E-01
cg05170353	-35.28	13.83	1.07E-02	-49.15	28.44	8.39E-02	-8.52	58.62	8.84E-01	-38.31	18.80	4.16E-02	-14.74	33.82	6.63E-01

cg00050336	-13.26	5.20	1.07E-02	-18.54	7.64	1.53E-02	-7.45	12.52	5.52E-01	-9.80	12.13	4.19E-01	-8.82	12.20	4.70E-01
cg22089878	28.45	11.15	1.07E-02	26.09	13.91	6.06E-02	-22.68	54.84	6.79E-01	0.00	55.17	1.00E+00	45.92	21.28	3.10E-02
cg07362341	24.83	9.73	1.07E-02	22.64	12.91	7.95E-02	41.69	32.87	2.05E-01	55.81	32.64	8.73E-02	13.10	19.27	4.96E-01
cg20779239	25.52	10.01	1.07E-02	26.00	16.90	1.24E-01	20.02	30.69	5.14E-01	43.28	17.17	1.17E-02	-2.08	22.19	9.25E-01
cg13897468	15.11	5.92	1.07E-02	29.72	14.00	3.38E-02	18.67	20.38	3.59E-01	8.60	7.25	2.36E-01	35.90	22.56	1.12E-01
cg21526019	9.83	3.86	1.08E-02	12.38	5.20	1.74E-02	14.96	9.84	1.29E-01	6.79	9.54	4.77E-01	-2.68	10.50	7.98E-01
cg27388703	-43.38	17.01	1.08E-02	-40.44	20.51	4.86E-02	-170.07	77.56	2.83E-02	12.14	74.76	8.71E-01	-39.03	26.12	1.35E-01
cg17617843	14.19	5.56	1.08E-02	16.12	8.09	4.63E-02	11.55	13.12	3.79E-01	37.63	18.56	4.26E-02	4.31	10.96	6.94E-01
cg12336777	12.65	4.96	1.08E-02	17.79	8.41	3.44E-02	14.58	15.50	3.47E-01	8.41	8.37	3.15E-01	10.15	11.13	3.62E-01
cg26010756	21.92	8.59	1.08E-02	17.85	16.23	2.71E-01	-13.59	23.43	5.62E-01	25.18	8.98	5.04E-03	45.25	20.54	2.76E-02
cg19356854	-21.64	8.48	1.08E-02	-2.15	30.85	9.44E-01	113.28	139.62	4.17E-01	-24.36	9.01	6.88E-03	-8.96	45.42	8.44E-01
cg04549418	23.89	9.37	1.08E-02	32.26	15.11	3.27E-02	25.59	17.16	1.36E-01	11.02	22.55	6.25E-01	13.51	24.59	5.83E-01
cg21295838	15.11	5.93	1.08E-02	19.88	9.41	3.46E-02	1.56	15.92	9.22E-01	23.84	11.43	3.69E-02	3.05	13.40	8.20E-01
cg20220678	-9.84	3.86	1.08E-02	-8.76	6.81	1.99E-01	-0.65	9.11	9.43E-01	-11.14	6.17	7.10E-02	-23.37	11.65	4.49E-02
cg21552081	18.40	7.22	1.08E-02	11.11	11.08	3.16E-01	31.25	15.57	4.48E-02	24.20	16.17	1.34E-01	13.33	17.92	4.57E-01
cg15769388	-11.41	4.48	1.08E-02	-16.44	10.42	1.15E-01	-9.89	15.34	5.19E-01	-10.05	5.80	8.34E-02	-11.52	12.15	3.43E-01
cg15961904	-13.95	5.47	1.08E-02	-37.19	14.40	9.81E-03	-6.96	13.82	6.15E-01	-10.22	6.72	1.29E-01	-13.44	14.16	3.43E-01
cg08699181	39.10	15.34	1.08E-02	42.51	19.35	2.80E-02	34.68	46.49	4.56E-01	117.21	59.71	4.97E-02	4.49	34.55	8.97E-01
cg10027639	-9.10	3.57	1.08E-02	-16.02	7.25	2.70E-02	-0.87	9.58	9.28E-01	-10.13	5.17	5.02E-02	-1.88	9.44	8.42E-01
cg09361748	-13.89	5.45	1.08E-02	-23.22	8.24	4.84E-03	0.84	13.11	9.49E-01	-8.39	14.17	5.54E-01	-10.91	11.08	3.25E-01
cg03317517	16.14	6.33	1.08E-02	29.19	9.98	3.47E-03	12.65	23.91	5.97E-01	9.77	20.01	6.25E-01	5.93	9.69	5.41E-01
cg13578229	-40.14	15.75	1.08E-02	-60.80	30.39	4.54E-02	-57.93	196.10	7.68E-01	-25.98	19.85	1.91E-01	-74.31	51.03	1.45E-01
cg13531120	-10.63	4.17	1.08E-02	-15.56	6.46	1.59E-02	-6.50	9.93	5.13E-01	-16.13	13.27	2.24E-01	-4.52	7.54	5.48E-01
cg24115922	24.79	9.73	1.08E-02	40.97	20.41	4.47E-02	112.44	104.50	2.82E-01	14.20	9.72	1.44E-01	49.63	27.10	6.71E-02
cg00025795	24.05	9.44	1.08E-02	43.63	18.70	1.97E-02	12.25	18.86	5.16E-01	12.12	15.72	4.41E-01	40.78	25.68	1.12E-01
cg03391055	37.06	14.54	1.08E-02	57.48	34.70	9.76E-02	-48.11	82.57	5.60E-01	37.20	17.44	3.30E-02	26.44	46.42	5.69E-01
cg27212428	-11.72	4.60	1.08E-02	-8.19	10.68	4.43E-01	-20.38	14.23	1.52E-01	-11.43	5.91	5.30E-02	-11.02	14.29	4.41E-01
cg13235761	15.58	6.11	1.08E-02	12.57	8.26	1.28E-01	26.21	13.88	5.90E-02	29.17	20.29	1.51E-01	5.72	14.95	7.02E-01
cg24292761	10.27	4.03	1.08E-02	10.07	6.41	1.16E-01	18.62	8.88	3.59E-02	13.29	10.08	1.88E-01	1.37	8.24	8.67E-01
cg09968702	-9.75	3.83	1.08E-02	-7.20	5.53	1.93E-01	-1.83	8.59	8.31E-01	-15.83	15.34	3.02E-01	-19.05	7.51	1.12E-02
cg02441149	-10.28	4.04	1.08E-02	-15.75	9.05	8.18E-02	-14.04	17.81	4.30E-01	-8.17	5.06	1.07E-01	-10.84	11.92	3.63E-01
cg03308056	20.24	7.94	1.08E-02	15.10	19.06	4.28E-01	32.12	33.68	3.40E-01	25.32	9.53	7.87E-03	-23.63	28.97	4.15E-01
cg02618229	16.23	6.37	1.08E-02	11.21	12.12	3.55E-01	13.75	29.69	6.43E-01	19.16	8.64	2.65E-02	15.55	17.44	3.73E-01
cg01519765	13.07	5.13	1.08E-02	15.72	9.07	8.30E-02	4.06	11.97	7.34E-01	15.87	10.77	1.41E-01	13.72	9.89	1.66E-01
cg08139512	-16.23	6.37	1.08E-02	-24.69	9.78	1.15E-02	-15.52	13.64	2.55E-01	-1.72	17.89	9.23E-01	-9.29	13.29	4.85E-01

cg23679332	19.92	7.82	1.08E-02	18.38	19.96	3.57E-01	33.62	37.67	3.72E-01	18.86	9.17	3.98E-02	25.25	28.13	3.69E-01
cg13757263	-8.33	3.27	1.08E-02	-10.17	4.85	3.59E-02	-4.21	7.78	5.88E-01	-6.70	7.32	3.60E-01	-9.56	7.93	2.28E-01
cg26010734	12.38	4.86	1.08E-02	24.50	14.10	8.24E-02	87.51	62.87	1.64E-01	9.16	5.47	9.43E-02	19.86	16.47	2.28E-01
cg05872758	-11.62	4.56	1.08E-02	-12.05	6.93	8.21E-02	-9.14	9.92	3.57E-01	-2.70	12.04	8.23E-01	-19.25	9.91	5.19E-02
cg17977273	8.34	3.27	1.09E-02	9.66	4.88	4.81E-02	8.27	7.18	2.49E-01	5.95	8.41	4.79E-01	7.21	7.49	3.35E-01
cg27338109	22.05	8.66	1.09E-02	36.58	19.52	6.09E-02	13.20	16.64	4.27E-01	16.86	12.85	1.89E-01	46.07	30.83	1.35E-01
cg10147462	11.28	4.43	1.09E-02	17.12	10.57	1.05E-01	31.80	13.98	2.29E-02	7.10	4.83	1.42E-01	8.49	13.63	5.33E-01
cg02879776	-10.79	4.24	1.09E-02	-16.31	7.91	3.92E-02	-1.92	12.93	8.82E-01	-9.09	6.45	1.58E-01	-11.41	10.18	2.62E-01
cg09202851	14.02	5.50	1.09E-02	5.32	8.27	5.20E-01	18.01	10.86	9.73E-02	13.78	17.88	4.41E-01	27.91	12.14	2.15E-02
cg21195162	-9.56	3.75	1.09E-02	-14.95	6.25	1.68E-02	0.52	12.11	9.65E-01	-10.18	5.55	6.68E-02	4.82	12.69	7.04E-01
cg10065153	11.72	4.60	1.09E-02	10.26	6.78	1.30E-01	3.47	9.53	7.15E-01	24.43	10.34	1.81E-02	12.48	13.99	3.72E-01
cg20197622	-11.31	4.44	1.09E-02	-4.46	10.24	6.63E-01	-18.75	16.39	2.53E-01	-12.34	5.56	2.64E-02	-12.17	14.06	3.87E-01
cg16475951	28.16	11.06	1.09E-02	22.78	21.93	2.99E-01	88.56	59.42	1.36E-01	20.62	15.04	1.70E-01	47.84	26.78	7.40E-02
cg08785724	-7.73	3.03	1.09E-02	-4.04	4.46	3.65E-01	-13.48	5.70	1.81E-02	-4.02	10.32	6.97E-01	-10.12	7.42	1.73E-01
cg26398467	17.63	6.92	1.09E-02	9.02	14.34	5.29E-01	-13.59	44.02	7.57E-01	22.09	9.72	2.30E-02	19.82	14.28	1.65E-01
cg07039378	-8.20	3.22	1.09E-02	-6.93	4.43	1.18E-01	-13.75	6.49	3.42E-02	3.12	12.55	8.04E-01	-8.54	8.06	2.89E-01
cg00157012	10.41	4.09	1.09E-02	9.62	5.67	9.00E-02	15.17	9.96	1.28E-01	16.32	11.57	1.58E-01	4.37	9.45	6.44E-01
cg16388791	-17.14	6.73	1.09E-02	-33.76	19.63	8.55E-02	-15.18	69.67	8.28E-01	-15.93	7.56	3.52E-02	-5.00	23.79	8.33E-01
cg01930679	-51.48	20.22	1.09E-02	-32.20	27.86	2.48E-01	-11.07	77.62	8.87E-01	-100.88	44.76	2.42E-02	-65.47	45.05	1.46E-01
cg04275239	-13.27	5.21	1.09E-02	-10.54	11.96	3.78E-01	5.28	12.30	6.68E-01	-19.22	6.46	2.93E-03	-17.13	14.97	2.53E-01
cg03446863	35.56	13.97	1.09E-02	34.85	16.61	3.59E-02	135.57	87.91	1.23E-01	45.47	44.24	3.04E-01	17.66	34.04	6.04E-01
cg11891925	-13.28	5.22	1.09E-02	-16.55	7.65	3.04E-02	-6.78	11.48	5.55E-01	-7.52	14.58	6.06E-01	-16.05	11.65	1.68E-01
cg03496220	11.19	4.39	1.09E-02	5.98	5.14	2.44E-01	25.72	8.79	3.44E-03	8.35	9.78	3.93E-01	10.19	9.38	2.77E-01
cg17064754	-12.79	5.02	1.09E-02	-11.12	6.88	1.06E-01	-12.07	11.96	3.13E-01	-21.38	19.25	2.67E-01	-14.72	10.65	1.67E-01
cg01432432	19.49	7.66	1.09E-02	24.74	14.33	8.43E-02	-17.79	35.30	6.14E-01	24.55	10.72	2.20E-02	4.70	19.29	8.07E-01
cg01142475	-6.03	2.37	1.09E-02	-4.74	4.31	2.71E-01	-0.87	5.20	8.67E-01	-10.98	4.53	1.53E-02	-6.51	5.09	2.01E-01
cg24725560	35.33	13.88	1.09E-02	46.78	17.28	6.77E-03	38.30	70.21	5.85E-01	23.35	33.20	4.82E-01	-3.19	37.04	9.31E-01
cg22682213	-25.70	10.10	1.09E-02	-26.02	26.19	3.21E-01	-74.20	93.70	4.28E-01	-24.51	11.57	3.41E-02	-29.41	36.12	4.16E-01
cg17822269	-17.65	6.94	1.09E-02	-20.28	18.29	2.68E-01	-43.67	34.31	2.03E-01	-19.03	8.01	1.75E-02	20.13	27.11	4.58E-01
cg19124316	-49.68	19.52	1.09E-02	-43.97	19.07	2.11E-02	8.81	58.63	8.81E-01	7.80	78.38	9.21E-01	-97.00	33.54	3.83E-03
cg25341032	-11.36	4.46	1.09E-02	-21.21	9.68	2.84E-02	-7.46	12.19	5.41E-01	-10.01	5.90	8.97E-02	-1.49	15.67	9.24E-01
cg19026356	31.63	12.43	1.09E-02	19.35	25.53	4.48E-01	-17.25	96.20	8.58E-01	32.83	15.39	3.29E-02	63.11	40.63	1.20E-01
cg23440144	19.65	7.72	1.09E-02	30.25	20.20	1.34E-01	36.19	24.03	1.32E-01	16.85	9.40	7.29E-02	1.41	28.24	9.60E-01
cg11784623	29.11	11.44	1.09E-02	33.89	17.97	5.92E-02	46.86	34.78	1.78E-01	29.30	22.70	1.97E-01	12.29	23.74	6.05E-01
cg16963683	-57.58	22.63	1.09E-02	-84.10	30.59	5.98E-03	-54.23	87.22	5.34E-01	3.42	62.92	9.57E-01	-32.63	44.70	4.65E-01

cg27473406	-10.64	4.18	1.09E-02	-4.97	6.35	4.33E-01	-16.71	8.96	6.22E-02	-5.98	14.20	6.73E-01	-16.58	8.20	4.31E-02
cg00969446	-11.82	4.65	1.09E-02	-15.49	6.39	1.54E-02	1.11	10.75	9.18E-01	-6.94	15.60	6.56E-01	-16.45	10.48	1.17E-01
cg06051581	-15.07	5.92	1.10E-02	-25.57	14.47	7.72E-02	-10.59	28.46	7.10E-01	-13.68	7.03	5.19E-02	-7.86	20.96	7.08E-01
cg07036137	27.53	10.82	1.10E-02	48.99	16.89	3.71E-03	4.01	27.16	8.83E-01	29.39	29.18	3.14E-01	11.58	18.94	5.41E-01
cg01150105	-8.11	3.19	1.10E-02	-13.26	6.36	3.71E-02	-5.38	10.58	6.11E-01	-4.87	4.35	2.63E-01	-13.87	9.17	1.30E-01
cg05609780	-15.88	6.24	1.10E-02	-34.92	14.40	1.53E-02	0.15	33.80	9.96E-01	-10.41	7.76	1.80E-01	-19.75	17.23	2.52E-01
cg19477921	-14.96	5.88	1.10E-02	-20.36	11.64	8.01E-02	-3.11	11.54	7.87E-01	-12.67	11.72	2.80E-01	-24.71	12.18	4.25E-02
cg15619125	21.02	8.26	1.10E-02	6.07	16.27	7.09E-01	21.30	14.16	1.32E-01	32.42	16.02	4.30E-02	26.41	22.47	2.40E-01
cg08645402	20.34	8.00	1.10E-02	30.31	14.27	3.37E-02	12.78	15.65	4.14E-01	16.20	16.28	3.20E-01	19.49	18.68	2.97E-01
cg09978353	11.71	4.60	1.10E-02	5.71	9.04	5.28E-01	9.75	10.90	3.71E-01	15.50	6.95	2.57E-02	13.65	13.08	2.97E-01
cg26614154	21.24	8.35	1.10E-02	31.79	17.50	6.93E-02	-3.34	19.93	8.67E-01	22.28	13.22	9.18E-02	28.84	18.81	1.25E-01
cg12172659	17.84	7.02	1.10E-02	37.42	20.01	6.14E-02	47.24	28.82	1.01E-01	11.12	8.08	1.69E-01	32.29	27.80	2.45E-01
cg08961893	21.11	8.30	1.10E-02	30.95	15.00	3.91E-02	5.82	15.92	7.15E-01	17.07	17.74	3.36E-01	31.16	18.45	9.13E-02
cg10720210	15.58	6.13	1.10E-02	-2.00	19.19	9.17E-01	35.72	30.89	2.48E-01	16.12	6.77	1.72E-02	29.82	31.10	3.38E-01
cg27203437	28.82	11.33	1.10E-02	23.76	33.26	4.75E-01	58.21	142.94	6.84E-01	30.17	12.60	1.66E-02	18.70	43.27	6.66E-01
cg20498746	12.54	4.93	1.10E-02	10.60	7.12	1.36E-01	8.97	13.43	5.04E-01	13.21	21.28	5.35E-01	16.68	8.56	5.14E-02
cg03270995	-47.88	18.83	1.10E-02	-76.90	30.56	1.19E-02	7.36	50.12	8.83E-01	-52.73	35.61	1.39E-01	-24.95	42.18	5.54E-01
cg12981595	-5.43	2.14	1.10E-02	-5.53	3.57	1.21E-01	3.22	5.87	5.83E-01	-7.13	3.41	3.62E-02	-9.17	6.26	1.43E-01
cg07300384	-14.42	5.67	1.10E-02	-17.97	16.14	2.66E-01	31.03	27.06	2.51E-01	-16.76	6.10	5.97E-03	-9.99	23.21	6.67E-01
cg09374627	-9.21	3.62	1.10E-02	-17.87	5.54	1.27E-03	-7.32	6.49	2.60E-01	-4.81	6.91	4.87E-01	-2.63	7.46	7.25E-01
cg01023593	-17.59	6.92	1.10E-02	-28.33	9.12	1.90E-03	-1.98	12.48	8.74E-01	-25.20	8.74	3.93E-03	-0.61	15.83	9.69E-01
cg16558330	-19.19	7.55	1.10E-02	-40.91	18.91	3.05E-02	-33.76	36.15	3.50E-01	-14.43	8.91	1.05E-01	-10.61	26.80	6.92E-01
cg07343527	46.18	18.17	1.10E-02	55.42	37.46	1.39E-01	5.37	185.90	9.77E-01	57.25	22.67	1.16E-02	-32.87	54.18	5.44E-01
cg04863679	-8.81	3.47	1.10E-02	-9.34	6.22	1.33E-01	-13.27	12.86	3.02E-01	-10.77	5.09	3.43E-02	0.31	8.85	9.72E-01
cg19436415	23.14	9.11	1.11E-02	35.62	12.35	3.91E-03	31.21	15.20	4.00E-02	17.12	30.83	5.79E-01	-1.21	15.67	9.38E-01
cg20092122	-25.94	10.21	1.11E-02	-30.90	15.16	4.15E-02	-6.99	29.66	8.14E-01	-18.62	27.64	5.01E-01	-29.34	18.90	1.21E-01
cg10096929	8.16	3.21	1.11E-02	10.49	4.46	1.88E-02	10.86	6.53	9.59E-02	6.32	11.38	5.79E-01	-2.48	8.00	7.56E-01
cg27631114	35.08	13.81	1.11E-02	33.09	16.67	4.72E-02	24.91	57.19	6.63E-01	102.15	60.44	9.10E-02	27.51	30.60	3.69E-01
cg06699669	-44.31	17.44	1.11E-02	-60.25	23.00	8.81E-03	12.89	61.97	8.35E-01	-10.63	41.66	7.99E-01	-51.79	42.25	2.20E-01
cg06817516	20.63	8.12	1.11E-02	36.63	18.56	4.85E-02	9.22	20.66	6.55E-01	17.93	13.50	1.84E-01	19.52	15.01	1.93E-01
cg13433366	26.45	10.41	1.11E-02	21.84	13.91	1.16E-01	49.00	30.58	1.09E-01	39.94	29.48	1.76E-01	17.84	23.33	4.44E-01
cg24064506	22.37	8.81	1.11E-02	18.72	15.26	2.20E-01	17.22	17.07	3.13E-01	26.34	18.28	1.50E-01	32.26	21.45	1.33E-01
cg24437859	-8.01	3.15	1.11E-02	-8.23	3.91	3.54E-02	-8.57	7.43	2.48E-01	-2.07	12.43	8.68E-01	-9.25	9.63	3.37E-01
cg16303825	35.99	14.17	1.11E-02	-10.76	40.80	7.92E-01	-24.28	103.38	8.14E-01	42.05	15.88	8.10E-03	66.17	55.73	2.35E-01
cg18577829	9.72	3.82	1.11E-02	10.37	7.56	1.70E-01	14.08	13.27	2.89E-01	9.29	4.89	5.76E-02	4.35	17.10	7.99E-01

cg00129556	20.96	8.25	1.11E-02	24.39	10.68	2.24E-02	30.27	25.45	2.34E-01	-28.27	39.13	4.70E-01	17.66	16.39	2.81E-01
cg04132917	-16.49	6.49	1.11E-02	-17.16	19.27	3.73E-01	82.92	67.92	2.22E-01	-16.60	7.30	2.30E-02	-25.25	22.15	2.54E-01
cg12730323	19.16	7.54	1.11E-02	31.94	11.65	6.13E-03	16.16	20.63	4.33E-01	19.53	19.81	3.24E-01	2.60	13.71	8.50E-01
cg21322149	-55.05	21.68	1.11E-02	-58.41	27.74	3.52E-02	-129.21	57.08	2.36E-02	11.71	63.51	8.54E-01	-28.40	50.82	5.76E-01
cg26795848	10.42	4.10	1.11E-02	5.79	8.40	4.91E-01	7.30	10.01	4.66E-01	13.58	6.25	2.98E-02	12.03	10.14	2.36E-01
cg01217876	15.02	5.92	1.11E-02	19.47	8.48	2.17E-02	10.37	11.48	3.66E-01	22.77	17.29	1.88E-01	1.01	16.34	9.51E-01
cg13844402	19.88	7.83	1.11E-02	31.83	15.49	3.98E-02	36.92	26.23	1.59E-01	19.96	12.12	9.96E-02	0.55	16.05	9.73E-01
cg01642653	22.43	8.83	1.11E-02	18.18	12.76	1.54E-01	56.63	30.89	6.68E-02	54.21	34.22	1.13E-01	14.70	14.46	3.10E-01
cg12229666	53.70	21.15	1.11E-02	67.13	25.69	8.97E-03	-68.23	98.48	4.88E-01	2.41	57.55	9.67E-01	78.07	56.26	1.65E-01
cg08751451	-4.45	1.75	1.11E-02	-2.88	2.87	3.16E-01	-3.46	4.39	4.31E-01	-3.80	3.06	2.14E-01	-11.37	4.70	1.57E-02
cg04844175	-16.58	6.53	1.11E-02	-19.96	15.43	1.96E-01	-17.76	38.44	6.44E-01	-14.07	7.63	6.52E-02	-36.73	26.73	1.69E-01
cg21961270	30.27	11.92	1.11E-02	40.26	18.47	2.93E-02	56.72	32.17	7.79E-02	20.32	38.99	6.02E-01	10.81	20.08	5.90E-01
cg09308580	9.61	3.79	1.11E-02	11.89	5.02	1.79E-02	10.90	9.95	2.73E-01	3.50	9.87	7.23E-01	5.47	10.11	5.88E-01
cg16072661	4.99	1.97	1.11E-02	6.49	2.93	2.68E-02	8.86	4.42	4.51E-02	0.01	3.74	9.99E-01	4.15	7.08	5.58E-01
cg12796428	51.73	20.38	1.11E-02	96.44	55.29	8.11E-02	-132.13	209.14	5.28E-01	49.67	23.74	3.65E-02	28.05	59.21	6.36E-01
cg03340408	14.12	5.56	1.11E-02	11.68	7.58	1.23E-01	23.91	11.90	4.45E-02	8.42	20.03	6.74E-01	11.80	13.64	3.87E-01
cg03332271	18.22	7.18	1.11E-02	20.48	12.64	1.05E-01	6.10	13.25	6.45E-01	15.92	15.98	3.19E-01	36.33	16.82	3.08E-02
cg04651603	16.47	6.49	1.11E-02	12.44	9.29	1.81E-01	15.27	16.96	3.68E-01	41.08	20.87	4.90E-02	15.58	12.49	2.12E-01
cg00291981	38.07	15.00	1.11E-02	71.84	45.04	1.11E-01	9.62	206.10	9.63E-01	40.74	16.96	1.63E-02	-17.75	46.99	7.06E-01
cg04102905	-10.29	4.05	1.11E-02	-14.80	7.94	6.24E-02	-17.46	11.11	1.16E-01	-4.14	6.02	4.91E-01	-14.55	10.34	1.60E-01
cg02465295	-37.70	14.86	1.12E-02	-8.12	43.41	8.52E-01	16.54	191.78	9.31E-01	-45.46	16.53	5.96E-03	-2.03	56.41	9.71E-01
cg21321815	34.22	13.49	1.12E-02	35.82	19.45	6.55E-02	21.72	38.99	5.78E-01	20.03	34.52	5.62E-01	45.94	27.14	9.05E-02
cg08886154	-14.26	5.62	1.12E-02	-20.77	9.19	2.37E-02	-13.62	12.13	2.61E-01	-6.16	12.35	6.18E-01	-11.21	12.42	3.67E-01
cg17673897	12.75	5.03	1.12E-02	15.83	7.15	2.69E-02	23.16	14.04	9.89E-02	-0.86	18.40	9.63E-01	6.70	9.13	4.63E-01
cg06086316	-29.39	11.58	1.12E-02	-25.94	13.84	6.09E-02	-63.15	37.35	9.09E-02	-8.30	52.10	8.73E-01	-30.77	29.49	2.97E-01
cg02495518	-6.05	2.39	1.12E-02	-6.68	3.90	8.69E-02	1.72	5.55	7.56E-01	-8.05	4.56	7.73E-02	-9.96	5.83	8.74E-02
cg20848343	25.67	10.11	1.12E-02	18.76	14.34	1.91E-01	55.84	22.25	1.21E-02	-4.13	30.72	8.93E-01	27.75	22.75	2.22E-01
cg14200569	10.31	4.06	1.12E-02	12.68	9.36	1.76E-01	10.44	23.53	6.57E-01	9.12	4.99	6.75E-02	13.27	11.88	2.64E-01
cg18090577	-15.01	5.92	1.12E-02	-21.14	11.03	5.52E-02	-16.20	14.26	2.56E-01	-14.13	8.53	9.77E-02	11.16	24.38	6.47E-01
cg02493604	20.55	8.10	1.12E-02	24.69	10.85	2.29E-02	4.24	22.63	8.51E-01	27.93	26.86	2.98E-01	16.57	17.09	3.32E-01
cg12126901	-42.50	16.75	1.12E-02	-26.43	21.06	2.10E-01	-44.58	80.10	5.78E-01	1.74	85.67	9.84E-01	-83.65	31.33	7.58E-03
cg01591881	17.48	6.89	1.12E-02	7.22	7.84	3.57E-01	31.31	13.40	1.94E-02	31.27	13.89	2.44E-02	9.93	16.56	5.49E-01
cg22077197	7.47	2.94	1.12E-02	4.16	4.31	3.35E-01	11.26	5.55	4.24E-02	11.78	10.45	2.60E-01	8.22	7.06	2.44E-01
cg02523640	12.47	4.92	1.12E-02	14.96	12.29	2.23E-01	16.82	14.94	2.60E-01	13.96	6.83	4.10E-02	4.81	10.63	6.51E-01
cg26290716	8.34	3.29	1.12E-02	7.20	4.17	8.40E-02	13.94	7.94	7.91E-02	10.14	9.84	3.03E-01	3.57	10.61	7.37E-01

cg00218447	35.87	14.14	1.12E-02	38.59	21.13	6.78E-02	39.31	73.92	5.95E-01	25.07	46.33	5.88E-01	35.07	21.75	1.07E-01
cg21468929	13.33	5.25	1.12E-02	15.17	7.13	3.32E-02	27.53	17.17	1.09E-01	8.43	19.83	6.71E-01	6.53	9.71	5.01E-01
cg20895785	18.00	7.10	1.12E-02	25.63	14.47	7.65E-02	7.23	29.94	8.09E-01	15.94	9.41	9.02E-02	17.59	19.35	3.63E-01
cg23849826	5.52	2.18	1.12E-02	4.36	3.77	2.47E-01	1.84	4.35	6.73E-01	8.78	4.11	3.29E-02	8.38	5.86	1.53E-01
cg07952877	28.70	11.31	1.12E-02	56.72	19.89	4.35E-03	17.96	20.44	3.79E-01	13.38	8.85	1.30E-01	48.76	25.32	5.41E-02
cg12919119	13.07	5.15	1.12E-02	12.48	6.76	6.49E-02	24.60	13.28	6.40E-02	19.30	16.24	2.35E-01	1.08	12.54	9.31E-01
cg18651821	31.35	12.36	1.12E-02	22.11	15.74	1.60E-01	34.95	35.93	3.31E-01	70.94	40.24	7.79E-02	40.31	29.87	1.77E-01
cg19514721	10.92	4.30	1.12E-02	8.50	6.11	1.64E-01	14.45	10.41	1.65E-01	10.36	10.90	3.42E-01	14.78	10.24	1.49E-01
cg10064491	-15.40	6.07	1.12E-02	-9.89	11.46	3.88E-01	-20.62	25.81	4.24E-01	-21.78	7.99	6.41E-03	12.52	20.59	5.43E-01
cg22573731	-10.14	4.00	1.12E-02	-16.47	7.45	2.72E-02	-13.81	10.06	1.70E-01	-3.24	4.89	5.08E-01	-18.70	10.25	6.80E-02
cg13894719	-12.02	4.74	1.12E-02	-23.26	9.68	1.63E-02	-5.92	21.45	7.83E-01	-6.12	6.02	3.09E-01	-23.58	14.59	1.06E-01
cg11598054	-13.16	5.19	1.12E-02	-19.60	7.94	1.35E-02	-0.06	11.29	9.96E-01	-23.91	13.44	7.53E-02	-6.34	10.84	5.58E-01
cg11816229	18.92	7.46	1.12E-02	10.24	15.54	5.10E-01	25.36	18.77	1.77E-01	18.34	10.24	7.33E-02	34.92	26.23	1.83E-01
cg12743419	-15.17	5.98	1.12E-02	-16.63	11.45	1.46E-01	-15.17	11.96	2.05E-01	-10.67	10.63	3.15E-01	-21.52	14.92	1.49E-01
cg21834463	6.90	2.72	1.12E-02	4.88	3.57	1.71E-01	14.10	7.32	5.40E-02	10.52	7.34	1.52E-01	4.68	7.19	5.15E-01
cg03770912	31.21	12.31	1.12E-02	95.55	43.80	2.91E-02	-66.82	186.21	7.20E-01	25.51	13.82	6.50E-02	30.17	34.94	3.88E-01
cg02317785	-19.37	7.64	1.12E-02	-1.31	29.10	9.64E-01	-109.29	102.35	2.86E-01	-20.55	8.16	1.18E-02	-13.42	34.61	6.98E-01
cg17580045	26.38	10.40	1.12E-02	15.67	13.28	2.38E-01	27.71	44.75	5.36E-01	16.09	46.69	7.30E-01	51.17	19.56	8.91E-03
cg23547181	23.39	9.23	1.12E-02	4.61	18.27	8.01E-01	38.97	19.51	4.58E-02	24.09	14.32	9.26E-02	32.91	28.26	2.44E-01
cg02160684	5.21	2.06	1.12E-02	4.75	2.85	9.61E-02	2.12	4.88	6.65E-01	7.19	4.42	1.03E-01	9.29	6.94	1.80E-01
cg02918054	18.28	7.21	1.12E-02	39.98	15.58	1.03E-02	9.37	16.54	5.71E-01	17.33	13.63	2.03E-01	9.77	12.83	4.47E-01
cg20622311	-43.14	17.01	1.12E-02	-36.61	22.07	9.72E-02	-63.78	48.96	1.93E-01	10.85	45.20	8.10E-01	-88.56	38.49	2.14E-02
cg03215160	-10.25	4.04	1.12E-02	-11.32	8.62	1.89E-01	-19.97	19.57	3.08E-01	-9.24	5.16	7.35E-02	-10.06	11.52	3.83E-01
cg27534828	15.69	6.19	1.12E-02	16.06	9.50	9.07E-02	20.90	14.78	1.57E-01	2.86	14.63	8.45E-01	21.25	13.17	1.07E-01
cg16289848	-10.36	4.09	1.12E-02	-18.93	11.19	9.08E-02	-18.28	27.98	5.14E-01	-9.03	4.72	5.56E-02	-7.10	13.29	5.93E-01
cg05657709	9.01	3.56	1.12E-02	5.31	4.74	2.62E-01	17.03	8.38	4.23E-02	4.45	10.89	6.83E-01	16.45	9.14	7.19E-02
cg08798184	-39.44	15.56	1.12E-02	2.26	44.94	9.60E-01	-127.55	228.14	5.76E-01	-43.15	17.40	1.31E-02	-60.85	56.51	2.82E-01
cg19755069	-39.32	15.51	1.12E-02	-14.14	15.22	3.53E-01	-10.84	34.83	7.56E-01	-71.07	22.77	1.80E-03	-57.32	20.04	4.23E-03
cg16653173	41.20	16.25	1.12E-02	88.23	44.48	4.73E-02	26.04	94.82	7.84E-01	35.53	18.73	5.79E-02	22.69	55.87	6.85E-01
cg21135682	30.49	12.03	1.12E-02	34.31	34.24	3.16E-01	13.53	133.33	9.19E-01	34.72	13.50	1.01E-02	-19.14	44.08	6.64E-01
cg20884780	19.21	7.58	1.12E-02	19.09	22.57	3.97E-01	9.50	39.92	8.12E-01	17.59	8.59	4.07E-02	41.38	27.99	1.39E-01
cg15996406	16.79	6.62	1.12E-02	23.17	12.37	6.10E-02	-15.08	19.11	4.30E-01	21.46	6.82	1.66E-03	10.48	20.68	6.12E-01
cg05759948	-14.63	5.77	1.12E-02	-21.99	8.70	1.15E-02	-7.86	13.10	5.48E-01	-14.05	13.97	3.15E-01	-5.28	13.05	6.86E-01
cg20522398	-15.05	5.94	1.13E-02	-14.63	13.45	2.77E-01	-19.50	29.18	5.04E-01	-13.19	7.27	6.96E-02	-26.89	19.14	1.60E-01
cg24318598	16.73	6.60	1.13E-02	20.82	17.87	2.44E-01	21.59	29.44	4.63E-01	14.23	7.62	6.18E-02	33.82	26.33	1.99E-01

cg15596932	-54.61	21.54	1.13E-02	-79.45	23.69	7.97E-04	43.08	64.16	5.02E-01	-84.91	37.03	2.19E-02	-30.24	31.11	3.31E-01
cg23530330	-18.85	7.44	1.13E-02	-7.04	17.88	6.94E-01	-41.02	22.88	7.30E-02	-18.33	9.60	5.61E-02	-18.94	21.40	3.76E-01
cg01717524	-13.36	5.27	1.13E-02	-17.71	7.69	2.14E-02	-3.31	13.42	8.05E-01	-12.74	15.43	4.09E-01	-11.76	10.35	2.56E-01
cg10078511	6.37	2.51	1.13E-02	5.92	3.60	1.00E-01	3.79	4.87	4.36E-01	8.83	9.06	3.30E-01	10.58	6.10	8.29E-02
cg10938788	-10.50	4.14	1.13E-02	-11.29	8.55	1.86E-01	-1.38	7.80	8.60E-01	-16.51	6.88	1.64E-02	-12.18	11.94	3.08E-01
cg09046427	11.30	4.46	1.13E-02	17.27	7.67	2.44E-02	3.99	9.60	6.78E-01	2.19	9.08	8.09E-01	19.90	9.86	4.35E-02
cg02086790	13.60	5.37	1.13E-02	14.09	7.49	6.00E-02	12.23	16.10	4.48E-01	10.53	18.67	5.73E-01	14.12	9.91	1.54E-01
cg01043250	-11.01	4.34	1.13E-02	-11.26	8.85	2.03E-01	3.02	12.00	8.01E-01	-13.22	5.89	2.49E-02	-17.82	14.93	2.33E-01
cg24478733	-11.60	4.58	1.13E-02	-14.25	8.87	1.08E-01	3.63	10.91	7.39E-01	-11.68	7.23	1.06E-01	-22.53	10.84	3.76E-02
cg06718003	-6.82	2.69	1.13E-02	-4.64	4.64	3.17E-01	-11.56	8.51	1.74E-01	-10.46	4.54	2.11E-02	-1.97	5.87	7.38E-01
cg23813681	-6.63	2.62	1.13E-02	-7.99	4.66	8.63E-02	-4.04	6.08	5.07E-01	-10.28	4.53	2.32E-02	0.44	6.45	9.45E-01
cg18147147	13.52	5.34	1.13E-02	17.86	7.83	2.26E-02	12.03	15.63	4.41E-01	9.53	11.35	4.01E-01	8.68	11.98	4.69E-01
cg27427051	30.05	11.86	1.13E-02	36.23	34.15	2.89E-01	96.40	141.22	4.95E-01	32.81	13.23	1.31E-02	-20.05	45.32	6.58E-01
cg01550445	8.54	3.37	1.13E-02	6.59	4.44	1.37E-01	15.83	7.72	4.04E-02	2.57	10.69	8.10E-01	11.02	9.25	2.34E-01
cg19410046	-13.15	5.19	1.13E-02	-19.64	11.90	9.90E-02	-4.90	16.52	7.67E-01	-10.33	6.82	1.30E-01	-22.51	14.37	1.17E-01
cg11957400	6.29	2.49	1.13E-02	7.50	3.78	4.74E-02	7.27	4.63	1.17E-01	8.73	6.20	1.60E-01	-3.62	7.17	6.13E-01
cg17440939	43.05	17.00	1.13E-02	45.65	20.58	2.66E-02	71.32	71.76	3.20E-01	-25.88	114.29	8.21E-01	35.38	34.70	3.08E-01
cg27642416	-9.26	3.66	1.13E-02	-9.33	7.66	2.23E-01	-6.39	12.36	6.05E-01	-8.21	4.99	9.97E-02	-14.67	9.52	1.23E-01
cg07570498	13.27	5.24	1.13E-02	16.98	10.20	9.60E-02	9.87	12.36	4.25E-01	15.12	7.73	5.05E-02	0.75	16.78	9.64E-01
cg26401512	-29.35	11.59	1.13E-02	-16.68	20.30	4.11E-01	7.34	51.42	8.86E-01	-46.71	16.43	4.48E-03	-8.37	32.64	7.98E-01
cg25981875	24.58	9.71	1.13E-02	42.23	15.99	8.26E-03	4.44	19.74	8.22E-01	14.16	20.30	4.85E-01	29.22	24.20	2.27E-01
cg20912169	23.31	9.21	1.13E-02	48.02	31.71	1.30E-01	-128.40	107.18	2.31E-01	22.47	10.05	2.53E-02	19.51	35.07	5.78E-01
cg18456459	12.64	4.99	1.13E-02	10.37	6.76	1.25E-01	15.39	11.50	1.81E-01	29.49	22.80	1.96E-01	12.22	10.66	2.51E-01
cg06215569	30.29	11.96	1.13E-02	24.17	15.94	1.29E-01	23.58	62.68	7.07E-01	29.56	40.86	4.69E-01	42.26	21.35	4.77E-02
cg14302214	-16.86	6.66	1.13E-02	-20.60	9.56	3.12E-02	-10.63	16.57	5.21E-01	-17.23	21.78	4.29E-01	-13.60	13.06	2.98E-01
cg25009141	12.19	4.81	1.14E-02	11.21	6.63	9.06E-02	20.69	11.21	6.49E-02	20.95	13.31	1.15E-01	-1.86	12.17	8.79E-01
cg25692621	-14.93	5.90	1.14E-02	2.82	15.15	8.52E-01	-7.70	27.97	7.83E-01	-19.47	6.92	4.88E-03	-11.17	21.24	5.99E-01
cg15560801	-15.17	5.99	1.14E-02	-21.43	8.14	8.48E-03	-4.96	12.58	6.94E-01	-31.70	16.88	6.05E-02	-2.29	12.59	8.56E-01
cg01512084	17.57	6.94	1.14E-02	12.31	15.61	4.31E-01	11.64	18.44	5.28E-01	21.34	9.21	2.05E-02	14.79	22.72	5.15E-01
cg27559224	-10.30	4.07	1.14E-02	-16.43	8.45	5.18E-02	4.02	9.76	6.80E-01	-11.23	6.00	6.13E-02	-14.94	11.03	1.76E-01
cg21607076	15.16	5.99	1.14E-02	16.56	14.65	2.58E-01	29.35	56.54	6.04E-01	14.36	6.88	3.69E-02	18.43	23.54	4.34E-01
cg22175873	9.09	3.59	1.14E-02	7.14	5.56	1.99E-01	13.05	8.92	1.43E-01	20.16	9.28	2.98E-02	3.60	6.89	6.01E-01
cg19018709	15.06	5.95	1.14E-02	18.57	8.24	2.42E-02	21.35	13.07	1.02E-01	15.14	18.39	4.10E-01	-3.75	14.57	7.97E-01
cg26197530	-17.29	6.83	1.14E-02	-3.86	13.28	7.71E-01	-27.40	26.12	2.94E-01	-21.41	9.38	2.25E-02	-22.19	18.42	2.28E-01
cg12713445	15.32	6.05	1.14E-02	20.74	12.35	9.33E-02	-3.52	13.82	7.99E-01	17.06	8.67	4.92E-02	33.64	21.33	1.15E-01

cg05581297	-12.68	5.01	1.14E-02	-11.38	12.29	3.54E-01	-12.81	12.22	2.95E-01	-10.83	6.55	9.83E-02	-28.36	17.56	1.06E-01
cg12632264	21.28	8.41	1.14E-02	41.19	15.99	1.00E-02	15.41	16.13	3.39E-01	17.51	15.22	2.50E-01	2.53	21.91	9.08E-01
cg26392367	-12.05	4.76	1.14E-02	-8.76	6.65	1.88E-01	-6.36	12.00	5.96E-01	-24.95	13.61	6.68E-02	-16.89	10.44	1.06E-01
cg27547442	-21.25	8.39	1.14E-02	-63.67	28.54	2.57E-02	-34.68	107.41	7.47E-01	-18.32	9.06	4.31E-02	4.45	38.27	9.07E-01
cg06146977	19.72	7.79	1.14E-02	23.86	15.80	1.31E-01	43.41	30.84	1.59E-01	11.11	10.21	2.77E-01	42.31	23.43	7.10E-02
cg08460635	44.70	17.66	1.14E-02	53.25	25.90	3.97E-02	41.65	40.96	3.09E-01	83.93	57.04	1.41E-01	16.35	35.11	6.41E-01
cg16113298	15.63	6.17	1.14E-02	-7.98	21.62	7.12E-01	135.85	107.92	2.08E-01	16.60	6.73	1.36E-02	25.39	22.91	2.68E-01
cg07906632	20.07	7.93	1.14E-02	18.31	9.94	6.55E-02	15.85	26.49	5.50E-01	38.23	27.65	1.67E-01	20.10	18.12	2.67E-01
cg01151699	41.04	16.22	1.14E-02	40.58	19.83	4.07E-02	71.87	91.71	4.33E-01	82.29	75.06	2.73E-01	30.84	32.23	3.39E-01
cg15256491	-14.25	5.63	1.14E-02	-12.33	8.21	1.33E-01	-9.10	14.62	5.34E-01	-17.93	15.06	2.34E-01	-19.05	11.47	9.67E-02
cg01285652	-18.63	7.36	1.14E-02	-31.06	14.33	3.02E-02	-7.14	14.73	6.28E-01	-14.32	14.57	3.26E-01	-21.61	15.31	1.58E-01
cg08862366	-9.33	3.69	1.14E-02	-11.53	6.98	9.84E-02	-4.08	9.76	6.76E-01	-9.23	5.82	1.13E-01	-10.33	8.77	2.39E-01
cg11644817	41.67	16.47	1.14E-02	35.32	20.44	8.40E-02	84.53	49.25	8.61E-02	10.51	56.95	8.54E-01	54.16	41.78	1.95E-01
cg03835709	-13.79	5.45	1.14E-02	-14.20	10.89	1.92E-01	-6.92	20.21	7.32E-01	-12.57	7.20	8.07E-02	-24.41	16.95	1.50E-01
cg27405732	-8.70	3.44	1.14E-02	-6.41	4.91	1.92E-01	-10.94	7.07	1.22E-01	-21.32	10.35	3.94E-02	-3.79	8.55	6.58E-01
cg13947137	19.17	7.58	1.14E-02	24.75	11.92	3.79E-02	6.32	17.00	7.10E-01	11.34	20.38	5.78E-01	24.50	14.88	9.97E-02
cg04664328	-14.72	5.82	1.14E-02	-29.66	9.97	2.94E-03	-2.25	14.13	8.74E-01	-9.97	7.32	1.73E-01	-13.48	18.51	4.66E-01
cg12459932	-9.19	3.63	1.14E-02	-10.95	4.88	2.49E-02	-12.26	9.34	1.89E-01	3.90	11.15	7.26E-01	-8.95	8.38	2.85E-01
cg16209310	-12.88	5.09	1.14E-02	-13.48	14.71	3.60E-01	-25.57	23.18	2.70E-01	-12.98	5.93	2.85E-02	-4.81	16.57	7.71E-01
cg25829961	-25.96	10.26	1.14E-02	-21.34	14.58	1.43E-01	-20.89	21.28	3.26E-01	-44.43	32.21	1.68E-01	-35.34	24.85	1.55E-01
cg01138972	-30.06	11.88	1.14E-02	-31.24	20.51	1.28E-01	-153.45	97.07	1.14E-01	36.55	51.98	4.82E-01	-31.96	12.33	9.57E-03
cg14647190	-14.40	5.69	1.14E-02	-21.05	18.00	2.42E-01	-0.48	30.03	9.87E-01	-13.12	6.30	3.74E-02	-32.91	26.04	2.06E-01
cg21162003	19.49	7.70	1.14E-02	35.81	11.06	1.21E-03	5.54	14.81	7.08E-01	12.59	14.84	3.96E-01	10.80	18.88	5.67E-01
cg10338518	5.90	2.33	1.14E-02	6.44	3.13	3.94E-02	7.58	6.33	2.31E-01	9.97	5.77	8.41E-02	-2.35	6.13	7.01E-01
cg14952312	6.84	2.70	1.14E-02	10.29	4.04	1.09E-02	6.09	6.55	3.53E-01	5.65	6.69	3.99E-01	1.28	5.76	8.25E-01
cg10498524	27.02	10.68	1.14E-02	33.73	29.97	2.60E-01	85.67	118.23	4.69E-01	28.45	12.24	2.01E-02	3.40	33.35	9.19E-01
cg08581018	-14.36	5.68	1.14E-02	-14.23	7.64	6.23E-02	4.36	13.90	7.54E-01	-31.56	15.34	3.97E-02	-17.99	11.90	1.31E-01
cg00201457	-12.18	4.82	1.14E-02	-19.10	7.48	1.07E-02	-13.81	11.66	2.36E-01	-1.40	10.79	8.97E-01	-7.58	10.37	4.64E-01
cg23531468	-14.60	5.77	1.14E-02	-16.52	11.89	1.65E-01	-4.27	13.17	7.46E-01	-15.95	9.30	8.63E-02	-20.01	13.36	1.34E-01
cg15597136	108.15	42.76	1.14E-02	147.80	62.33	1.77E-02	132.70	127.42	2.98E-01	36.30	122.12	7.66E-01	65.27	78.86	4.08E-01
cg16618417	-74.85	29.60	1.14E-02	-53.53	27.79	5.41E-02	90.33	116.89	4.40E-01	-138.12	63.69	3.01E-02	-110.45	47.89	2.11E-02
cg24686918	-9.56	3.78	1.14E-02	-13.57	7.60	7.43E-02	-15.10	11.72	1.98E-01	-7.08	5.26	1.79E-01	-7.39	10.37	4.76E-01
cg16370556	16.01	6.33	1.15E-02	13.92	8.75	1.11E-01	21.05	15.87	1.85E-01	40.96	30.75	1.83E-01	13.21	12.08	2.74E-01
cg11287531	26.63	10.53	1.15E-02	9.66	25.24	7.02E-01	-15.63	95.08	8.69E-01	31.31	12.24	1.06E-02	26.85	38.84	4.89E-01
cg16260977	-40.46	16.00	1.15E-02	-45.48	25.09	6.99E-02	-49.25	59.45	4.07E-01	-21.69	28.11	4.40E-01	-57.75	36.07	1.09E-01

cg15442737	9.80	3.88	1.15E-02	13.98	5.81	1.62E-02	9.17	8.92	3.04E-01	10.74	9.51	2.59E-01	0.33	8.67	9.70E-01
cg23847017	9.02	3.57	1.15E-02	7.71	4.84	1.11E-01	13.33	8.19	1.04E-01	15.22	12.47	2.22E-01	5.71	8.27	4.90E-01
cg07566700	12.06	4.77	1.15E-02	10.49	6.45	1.04E-01	12.66	11.51	2.72E-01	14.37	14.69	3.28E-01	15.02	11.40	1.87E-01
cg17396404	18.71	7.40	1.15E-02	10.30	10.14	3.10E-01	25.75	16.25	1.13E-01	34.37	27.65	2.14E-01	28.75	17.04	9.16E-02
cg18030409	22.19	8.78	1.15E-02	25.01	12.11	3.89E-02	31.86	19.60	1.04E-01	28.83	44.28	5.15E-01	6.49	18.13	7.20E-01
cg02395441	-32.76	12.96	1.15E-02	-78.03	44.68	8.07E-02	-15.79	170.06	9.26E-01	-26.74	13.97	5.55E-02	-62.53	58.37	2.84E-01
cg01146232	15.79	6.25	1.15E-02	14.92	8.99	9.71E-02	11.77	16.42	4.74E-01	22.29	22.75	3.27E-01	17.51	11.46	1.26E-01
cg27591502	5.89	2.33	1.15E-02	5.00	3.33	1.34E-01	8.53	6.57	1.94E-01	10.49	4.64	2.39E-02	-1.93	6.33	7.60E-01
cg06963709	5.02	1.99	1.15E-02	6.58	2.75	1.67E-02	7.80	4.44	7.89E-02	2.29	5.66	6.85E-01	-1.72	5.07	7.35E-01
cg25433648	24.35	9.63	1.15E-02	38.61	18.14	3.33E-02	33.07	17.82	6.34E-02	9.05	17.88	6.13E-01	8.63	26.21	7.42E-01
cg14480507	13.64	5.40	1.15E-02	14.97	7.70	5.19E-02	8.65	12.16	4.77E-01	26.86	18.02	1.36E-01	9.79	11.46	3.93E-01
cg24175899	29.36	11.62	1.15E-02	33.20	14.16	1.91E-02	15.04	31.09	6.28E-01	83.87	73.00	2.51E-01	17.28	28.83	5.49E-01
cg04947907	9.90	3.92	1.15E-02	11.89	5.63	3.47E-02	9.58	10.19	3.47E-01	12.85	12.64	3.09E-01	5.48	7.50	4.65E-01
cg06927337	17.01	6.73	1.15E-02	25.75	12.70	4.27E-02	8.32	14.01	5.53E-01	19.99	11.02	6.98E-02	3.52	19.83	8.59E-01
cg02227879	5.63	2.23	1.15E-02	5.94	2.91	4.11E-02	9.59	5.01	5.57E-02	2.71	6.37	6.71E-01	-0.88	7.29	9.04E-01
cg05168491	18.31	7.25	1.15E-02	29.09	11.45	1.11E-02	19.00	17.07	2.65E-01	21.22	21.26	3.18E-01	2.55	13.17	8.47E-01
cg02978184	-15.42	6.10	1.15E-02	17.79	28.32	5.30E-01	-16.56	72.01	8.18E-01	-17.93	6.55	6.20E-03	-7.23	21.84	7.40E-01
cg04125586	-36.50	14.44	1.15E-02	-46.18	21.59	3.25E-02	-16.55	46.25	7.20E-01	-44.32	30.73	1.49E-01	-18.93	29.85	5.26E-01
cg17063452	12.09	4.78	1.15E-02	19.12	11.17	8.69E-02	33.43	17.53	5.65E-02	9.41	5.85	1.08E-01	-2.74	17.60	8.76E-01
cg02808411	19.06	7.55	1.15E-02	31.68	24.20	1.90E-01	-5.87	30.39	8.47E-01	19.64	8.54	2.15E-02	16.77	30.59	5.84E-01
cg07613792	20.69	8.19	1.15E-02	33.60	14.10	1.72E-02	-6.12	24.42	8.02E-01	31.12	17.55	7.62E-02	9.83	14.20	4.89E-01
cg16389924	26.07	10.32	1.15E-02	14.86	14.21	2.96E-01	31.25	25.20	2.15E-01	14.84	34.79	6.70E-01	53.90	22.16	1.50E-02
cg12063947	-47.00	18.60	1.15E-02	-52.94	24.12	2.82E-02	85.70	82.28	2.98E-01	-72.58	38.35	5.84E-02	-37.06	39.72	3.51E-01
cg15691822	28.26	11.19	1.15E-02	51.43	20.98	1.42E-02	30.05	47.07	5.23E-01	14.19	15.45	3.58E-01	33.39	30.48	2.73E-01
cg09326832	8.48	3.36	1.15E-02	10.82	4.34	1.27E-02	6.35	9.14	4.88E-01	1.15	10.85	9.15E-01	6.10	8.08	4.50E-01
cg06187843	39.92	15.80	1.15E-02	45.56	48.19	3.44E-01	-92.87	189.21	6.24E-01	38.82	17.59	2.73E-02	55.47	56.53	3.26E-01
cg07801181	13.98	5.54	1.15E-02	22.60	8.86	1.08E-02	7.97	11.95	5.05E-01	9.69	9.33	2.99E-01	0.87	26.83	9.74E-01
cg16041611	-18.44	7.30	1.15E-02	-20.07	13.07	1.25E-01	-6.59	22.65	7.71E-01	-26.32	11.40	2.10E-02	-4.04	17.49	8.18E-01
cg16372625	57.96	22.95	1.15E-02	57.71	27.56	3.62E-02	3.63	190.79	9.85E-01	65.67	78.54	4.03E-01	59.42	50.47	2.39E-01
cg08982864	-16.32	6.46	1.15E-02	-20.31	9.95	4.12E-02	-7.78	15.70	6.20E-01	-14.01	15.87	3.77E-01	-16.91	13.09	1.96E-01
cg09102329	-11.09	4.39	1.15E-02	-23.07	9.07	1.10E-02	-7.91	14.41	5.83E-01	-5.34	5.40	3.22E-01	-17.24	11.60	1.37E-01
cg23698950	-8.69	3.44	1.15E-02	-7.59	6.14	2.16E-01	-15.46	14.13	2.74E-01	-8.25	4.82	8.66E-02	-10.20	10.15	3.15E-01
cg14655316	34.55	13.68	1.15E-02	46.31	21.05	2.78E-02	28.02	47.28	5.53E-01	61.62	41.58	1.38E-01	15.50	22.02	4.82E-01
cg16705929	-8.57	3.39	1.15E-02	-8.50	4.67	6.90E-02	-9.35	8.21	2.55E-01	-1.70	8.43	8.40E-01	-15.75	9.04	8.14E-02
cg01191259	6.05	2.39	1.15E-02	6.36	3.27	5.22E-02	4.92	5.37	3.59E-01	16.23	8.71	6.26E-02	2.33	5.49	6.72E-01

cg05292310	-12.38	4.90	1.16E-02	-11.41	10.47	2.76E-01	-7.85	16.03	6.25E-01	-10.43	6.63	1.16E-01	-24.29	13.00	6.17E-02
cg11018723	-45.98	18.21	1.16E-02	-30.53	41.61	4.63E-01	-420.70	284.28	1.39E-01	-47.69	21.44	2.61E-02	-48.23	63.03	4.44E-01
cg02792086	36.70	14.53	1.16E-02	15.19	30.63	6.20E-01	-80.21	136.97	5.58E-01	46.37	17.77	9.06E-03	33.45	47.26	4.79E-01
cg07267845	-13.69	5.42	1.16E-02	-20.66	8.63	1.67E-02	-0.15	14.91	9.92E-01	-11.61	9.99	2.45E-01	-11.77	12.81	3.58E-01
cg14999001	17.21	6.82	1.16E-02	8.55	19.45	6.60E-01	5.61	25.49	8.26E-01	23.13	8.45	6.18E-03	4.52	17.36	7.94E-01
cg06429887	18.85	7.46	1.16E-02	33.60	18.04	6.25E-02	36.72	20.60	7.46E-02	12.04	9.59	2.09E-01	10.64	24.65	6.66E-01
cg23304647	8.67	3.43	1.16E-02	9.18	4.79	5.50E-02	9.00	8.26	2.76E-01	9.74	8.58	2.56E-01	5.43	8.78	5.36E-01
cg22776451	-10.52	4.17	1.16E-02	-24.86	10.30	1.58E-02	-13.51	14.06	3.37E-01	-5.82	5.20	2.64E-01	-14.34	12.72	2.60E-01
cg14192130	-9.23	3.66	1.16E-02	-8.94	4.65	5.46E-02	-7.16	9.77	4.63E-01	-13.09	12.04	2.77E-01	-10.03	9.48	2.90E-01
ch.5.236818R	-30.68	12.15	1.16E-02	-32.21	16.40	4.95E-02	-27.61	31.96	3.88E-01	-19.49	41.47	6.38E-01	-33.23	25.85	1.99E-01
cg15074329	-15.17	6.01	1.16E-02	-7.17	11.24	5.24E-01	-1.35	16.16	9.34E-01	-22.98	10.23	2.47E-02	-21.65	12.48	8.29E-02
cg00791024	20.98	8.31	1.16E-02	46.09	20.20	2.25E-02	18.97	43.48	6.63E-01	15.92	10.13	1.16E-01	14.61	23.80	5.39E-01
cg06642397	-12.59	4.99	1.16E-02	-7.06	12.37	5.68E-01	-14.19	20.89	4.97E-01	-13.10	5.95	2.77E-02	-18.45	17.90	3.02E-01
cg05852824	-13.22	5.24	1.16E-02	-9.37	7.91	2.37E-01	-1.03	12.71	9.36E-01	-22.72	12.04	5.92E-02	-22.91	11.63	4.89E-02
cg07325692	-15.03	5.95	1.16E-02	-18.08	11.58	1.18E-01	1.37	20.08	9.46E-01	-16.29	8.08	4.37E-02	-14.60	18.46	4.29E-01
cg16245698	7.12	2.82	1.16E-02	8.72	4.81	6.97E-02	13.29	5.89	2.41E-02	7.00	4.22	9.67E-02	-2.90	6.57	6.59E-01
cg08321129	-30.82	12.21	1.16E-02	-37.54	12.38	2.43E-03	36.48	39.47	3.55E-01	-25.57	50.34	6.12E-01	-40.68	24.44	9.60E-02
cg14171824	-9.54	3.78	1.16E-02	-9.49	5.07	6.13E-02	-15.27	8.57	7.48E-02	7.01	12.87	5.86E-01	-11.58	9.31	2.14E-01
cg03286391	-29.39	11.65	1.16E-02	-20.68	15.72	1.88E-01	23.15	54.17	6.69E-01	-63.51	31.84	4.61E-02	-39.18	22.37	7.99E-02
cg06865119	-15.66	6.21	1.16E-02	-8.72	19.73	6.58E-01	-11.22	22.30	6.15E-01	-17.57	7.16	1.41E-02	-10.10	23.10	6.62E-01
cg25650360	-8.77	3.47	1.16E-02	-16.32	7.77	3.56E-02	-20.64	13.89	1.37E-01	-5.64	4.40	2.00E-01	-6.11	10.35	5.55E-01
cg16116203	-12.59	4.99	1.16E-02	-19.59	7.32	7.44E-03	-10.47	10.38	3.13E-01	-8.14	18.66	6.63E-01	-2.10	10.33	8.39E-01
cg25010998	-70.44	27.91	1.16E-02	-85.72	33.11	9.64E-03	-243.87	183.02	1.83E-01	18.32	103.71	8.60E-01	-26.79	63.41	6.73E-01
cg24530471	-14.12	5.60	1.16E-02	-16.42	12.61	1.93E-01	2.60	22.17	9.07E-01	-17.17	6.93	1.33E-02	1.45	18.86	9.39E-01
cg24590633	-44.31	17.56	1.16E-02	-74.13	33.51	2.70E-02	-193.94	109.81	7.74E-02	-32.96	17.26	5.61E-02	-14.35	48.05	7.65E-01
cg08619893	-11.87	4.71	1.16E-02	-18.23	7.94	2.16E-02	-1.59	11.62	8.91E-01	-9.69	9.39	3.02E-01	-11.88	9.73	2.22E-01
cg15717071	37.58	14.89	1.16E-02	27.91	22.18	2.08E-01	40.09	44.33	3.66E-01	54.52	50.76	2.83E-01	45.06	25.16	7.34E-02
cg24009995	38.67	15.33	1.16E-02	42.70	19.43	2.80E-02	44.49	51.57	3.88E-01	82.27	53.10	1.21E-01	6.41	33.76	8.49E-01
cg19823494	-16.76	6.64	1.16E-02	-26.35	11.61	2.32E-02	0.10	14.08	9.94E-01	-11.31	12.51	3.66E-01	-29.59	16.23	6.83E-02
cg03717367	7.67	3.04	1.16E-02	8.80	4.20	3.63E-02	14.25	7.03	4.26E-02	0.12	7.58	9.88E-01	2.95	8.48	7.28E-01
cg01414687	13.98	5.54	1.16E-02	12.21	14.15	3.88E-01	51.37	27.79	6.45E-02	12.10	6.74	7.25E-02	14.42	15.33	3.47E-01
cg20801110	12.34	4.89	1.16E-02	9.33	7.25	1.98E-01	23.15	9.29	1.27E-02	12.62	16.70	4.50E-01	3.28	11.46	7.75E-01
cg27578811	11.98	4.75	1.16E-02	7.53	8.62	3.82E-01	17.19	10.42	9.90E-02	18.78	11.12	9.11E-02	8.79	8.56	3.05E-01
cg21888330	-16.66	6.61	1.16E-02	-17.73	17.72	3.17E-01	-1.15	24.10	9.62E-01	-15.17	7.76	5.05E-02	-51.41	26.85	5.55E-02
cg03948781	6.30	2.50	1.16E-02	6.24	3.54	7.76E-02	8.62	5.31	1.04E-01	-2.14	7.44	7.74E-01	9.13	6.13	1.36E-01

cg16216305	-36.63	14.52	1.16E-02	-19.10	47.51	6.88E-01	6.04	185.48	9.74E-01	-32.47	15.94	4.17E-02	-111.91	54.49	4.00E-02
cg09925620	23.45	9.30	1.17E-02	23.90	12.49	5.57E-02	47.77	20.38	1.91E-02	11.41	32.30	7.24E-01	-4.34	23.59	8.54E-01
cg02169333	44.37	17.59	1.17E-02	-1.01	53.32	9.85E-01	35.44	243.21	8.84E-01	53.55	19.35	5.66E-03	0.98	71.81	9.89E-01
cg14838566	-13.53	5.36	1.17E-02	-6.41	10.02	5.22E-01	5.50	20.50	7.89E-01	-20.21	7.67	8.39E-03	-13.96	13.56	3.03E-01
cg02770054	21.35	8.47	1.17E-02	13.59	11.23	2.26E-01	56.07	34.71	1.06E-01	36.88	37.11	3.20E-01	26.14	14.95	8.04E-02
cg11866943	12.33	4.89	1.17E-02	12.10	6.98	8.31E-02	19.72	10.24	5.41E-02	1.95	15.22	8.98E-01	9.53	11.57	4.10E-01
cg27291710	-11.22	4.45	1.17E-02	-9.27	6.27	1.39E-01	-23.23	10.01	2.03E-02	4.02	14.59	7.83E-01	-11.36	9.82	2.47E-01
cg05388330	21.64	8.58	1.17E-02	15.09	15.24	3.22E-01	38.85	21.01	6.45E-02	25.76	13.67	5.96E-02	1.92	24.53	9.38E-01
cg27230009	11.31	4.48	1.17E-02	9.59	5.98	1.09E-01	20.28	10.94	6.38E-02	22.40	14.32	1.18E-01	1.81	10.82	8.67E-01
cg00445566	41.19	16.33	1.17E-02	26.06	20.17	1.96E-01	130.17	83.83	1.20E-01	42.63	87.90	6.28E-01	65.09	31.33	3.77E-02
cg04567588	34.10	13.52	1.17E-02	64.49	32.70	4.86E-02	-116.60	123.11	3.44E-01	34.24	14.48	1.80E-02	-5.48	46.37	9.06E-01
cg20878925	16.98	6.73	1.17E-02	19.31	16.01	2.28E-01	11.47	36.56	7.54E-01	13.33	8.02	9.64E-02	45.16	23.30	5.27E-02
cg24235828	-39.94	15.84	1.17E-02	-37.19	22.45	9.76E-02	-5.03	46.97	9.15E-01	-60.85	35.34	8.52E-02	-46.03	36.56	2.08E-01
cg00086493	-12.14	4.81	1.17E-02	-12.17	6.74	7.09E-02	-17.78	10.62	9.41E-02	-4.83	18.60	7.95E-01	-8.99	10.33	3.84E-01
cg23153707	21.73	8.62	1.17E-02	31.51	12.00	8.65E-03	36.37	31.51	2.48E-01	8.96	37.50	8.11E-01	6.41	14.44	6.57E-01
cg08595592	-8.64	3.42	1.17E-02	-8.76	7.46	2.40E-01	3.73	15.27	8.07E-01	-8.23	4.41	6.21E-02	-14.84	9.29	1.10E-01
cg19627145	19.52	7.74	1.17E-02	11.85	16.48	4.72E-01	18.90	18.08	2.96E-01	28.49	11.15	1.06E-02	-2.76	23.03	9.05E-01
cg05896714	-50.65	20.09	1.17E-02	-61.82	28.62	3.08E-02	55.71	72.13	4.40E-01	-73.04	48.89	1.35E-01	-46.69	39.34	2.35E-01
cg05122803	-9.48	3.76	1.17E-02	-13.25	7.91	9.38E-02	-12.05	14.74	4.14E-01	-6.61	4.96	1.82E-01	-14.16	10.26	1.68E-01
cg10838789	30.30	12.02	1.17E-02	26.10	15.39	8.99E-02	73.52	58.20	2.07E-01	42.14	57.72	4.65E-01	30.99	21.80	1.55E-01
cg00433284	62.53	24.80	1.17E-02	22.78	26.64	3.93E-01	91.60	75.77	2.27E-01	155.04	86.37	7.27E-02	86.50	33.07	8.90E-03
cg17535314	-10.40	4.13	1.17E-02	-12.29	7.17	8.63E-02	-11.81	10.10	2.42E-01	-5.02	7.57	5.07E-01	-14.00	9.12	1.25E-01
cg22534681	-24.72	9.81	1.17E-02	-19.44	33.25	5.59E-01	-235.89	165.25	1.53E-01	-23.45	10.61	2.71E-02	-39.22	41.75	3.48E-01
cg20373747	23.93	9.49	1.17E-02	37.76	22.29	9.02E-02	22.82	38.94	5.58E-01	17.86	11.56	1.22E-01	43.56	32.70	1.83E-01
cg14724918	-45.25	17.95	1.17E-02	-51.67	22.90	2.41E-02	-88.12	63.39	1.64E-01	59.10	72.74	4.16E-01	-41.07	36.32	2.58E-01
cg25741837	12.09	4.80	1.17E-02	11.26	6.62	8.89E-02	17.46	10.27	8.91E-02	6.92	16.12	6.68E-01	10.45	11.70	3.72E-01
cg02167201	-17.40	6.90	1.17E-02	-19.60	10.11	5.25E-02	-0.50	15.13	9.74E-01	-21.44	20.38	2.93E-01	-27.01	15.03	7.23E-02
cg11531021	22.71	9.01	1.17E-02	28.85	34.47	4.03E-01	-83.80	118.23	4.78E-01	26.45	9.74	6.63E-03	-19.75	33.88	5.60E-01
cg04460548	60.76	24.11	1.17E-02	47.31	34.47	1.70E-01	144.04	87.50	9.97E-02	31.10	53.41	5.60E-01	87.97	50.12	7.92E-02
cg06627801	-13.57	5.38	1.17E-02	-14.48	7.41	5.07E-02	-5.47	11.68	6.39E-01	-23.77	22.67	2.94E-01	-16.83	11.94	1.59E-01
cg17156432	-11.12	4.41	1.17E-02	-8.36	6.58	2.04E-01	-22.99	9.60	1.66E-02	-0.57	13.64	9.67E-01	-10.42	9.13	2.53E-01
cg17907520	-20.56	8.16	1.17E-02	-12.95	10.29	2.08E-01	-33.23	17.76	6.13E-02	-3.82	16.21	8.14E-01	-41.81	16.42	1.09E-02
cg08591998	23.20	9.21	1.17E-02	7.26	18.52	6.95E-01	40.49	18.22	2.62E-02	13.74	15.27	3.68E-01	45.26	25.13	7.17E-02
cg18057618	-18.24	7.24	1.17E-02	-11.62	11.39	3.08E-01	-45.45	18.58	1.45E-02	-8.75	14.10	5.35E-01	-23.40	16.40	1.54E-01
cg16145799	19.06	7.56	1.17E-02	7.99	20.87	7.02E-01	31.69	31.23	3.10E-01	20.99	8.71	1.60E-02	5.98	31.85	8.51E-01

cg05266437	34.50	13.69	1.17E-02	26.12	17.24	1.30E-01	51.64	20.57	1.21E-02	8.10	12.94	5.31E-01	70.37	23.75	3.05E-03
cg17297628	-19.33	7.67	1.18E-02	-11.97	19.48	5.39E-01	-4.03	23.71	8.65E-01	-22.38	9.45	1.79E-02	-28.38	26.86	2.91E-01
cg11168293	-12.16	4.83	1.18E-02	-15.17	7.51	4.34E-02	-4.79	12.18	6.94E-01	-20.47	10.71	5.60E-02	-4.35	10.13	6.68E-01
cg07309864	-15.12	6.00	1.18E-02	-22.76	12.37	6.58E-02	-16.17	14.66	2.70E-01	-7.62	9.51	4.23E-01	-20.20	13.44	1.33E-01
cg05357527	66.69	26.47	1.18E-02	38.11	39.41	3.33E-01	171.35	100.23	8.73E-02	68.99	70.65	3.29E-01	82.24	45.48	7.06E-02
cg22864337	43.47	17.25	1.18E-02	54.12	35.72	1.30E-01	77.99	64.29	2.25E-01	21.74	12.90	9.19E-02	90.21	36.56	1.36E-02
cg01290568	7.86	3.12	1.18E-02	5.44	4.09	1.84E-01	15.02	6.52	2.13E-02	8.96	12.32	4.67E-01	5.44	8.74	5.34E-01
cg10357841	30.46	12.09	1.18E-02	-5.30	40.71	8.96E-01	-7.91	119.25	9.47E-01	33.65	13.27	1.12E-02	43.00	45.26	3.42E-01
cg02714994	-7.30	2.90	1.18E-02	-8.48	4.22	4.43E-02	-8.25	6.08	1.75E-01	-5.91	9.85	5.49E-01	-4.25	6.28	4.98E-01
cg05277146	-10.23	4.06	1.18E-02	-8.79	10.70	4.11E-01	-6.53	20.06	7.45E-01	-9.87	4.74	3.71E-02	-18.16	14.46	2.09E-01
cg00901598	-5.59	2.22	1.18E-02	-4.67	3.63	1.98E-01	-6.51	4.92	1.86E-01	-6.66	4.23	1.15E-01	-4.66	5.81	4.23E-01
cg24023588	-28.72	11.40	1.18E-02	-19.13	28.98	5.09E-01	-82.73	116.95	4.79E-01	-34.83	13.08	7.77E-03	19.40	41.31	6.39E-01
cg06956444	-15.22	6.04	1.18E-02	-19.75	12.15	1.04E-01	-1.27	14.80	9.32E-01	-14.67	9.84	1.36E-01	-21.96	13.22	9.67E-02
cg27465569	-12.84	5.10	1.18E-02	2.32	12.70	8.55E-01	1.21	15.75	9.39E-01	-18.34	6.10	2.64E-03	-16.09	16.24	3.22E-01
cg08656816	-12.01	4.77	1.18E-02	-15.10	7.30	3.87E-02	-9.12	11.24	4.17E-01	-4.83	11.44	6.73E-01	-14.06	10.17	1.67E-01
cg02102684	12.46	4.95	1.18E-02	12.53	7.86	1.11E-01	6.40	9.93	5.20E-01	11.03	13.37	4.10E-01	20.06	10.55	5.73E-02
cg13559225	-47.52	18.87	1.18E-02	-55.38	46.28	2.31E-01	-160.95	196.13	4.12E-01	-43.95	22.15	4.72E-02	-49.92	60.10	4.06E-01
cg25924746	9.92	3.94	1.18E-02	14.22	7.29	5.10E-02	6.73	10.18	5.09E-01	9.81	5.89	9.56E-02	3.31	11.90	7.81E-01
cg12027420	29.65	11.77	1.18E-02	38.50	31.96	2.28E-01	-24.43	120.05	8.39E-01	26.94	13.74	5.00E-02	40.49	33.88	2.32E-01
cg01515896	38.40	15.25	1.18E-02	49.01	18.89	9.49E-03	12.74	52.11	8.07E-01	4.30	53.77	9.36E-01	27.61	35.71	4.40E-01
cg02815374	13.75	5.46	1.18E-02	11.51	6.81	9.08E-02	28.98	15.79	6.64E-02	3.38	20.97	8.72E-01	15.64	13.27	2.39E-01
cg17062109	17.23	6.84	1.18E-02	46.88	19.98	1.89E-02	51.58	74.86	4.91E-01	14.60	7.25	4.39E-02	2.70	19.83	8.92E-01
cg25018403	-13.57	5.39	1.18E-02	-20.17	6.75	2.82E-03	-6.17	11.45	5.90E-01	-26.74	16.95	1.15E-01	-0.93	10.48	9.29E-01
cg14323117	-8.10	3.22	1.18E-02	-1.34	6.47	8.36E-01	-8.32	11.41	4.66E-01	-10.77	4.36	1.35E-02	-9.69	8.99	2.81E-01
cg17420028	6.55	2.60	1.18E-02	6.73	3.78	7.51E-02	18.12	8.17	2.64E-02	4.97	4.61	2.81E-01	-0.56	7.99	9.44E-01
cg03653601	35.96	14.28	1.18E-02	25.61	18.11	1.57E-01	80.93	75.87	2.86E-01	97.05	63.39	1.26E-01	41.92	26.44	1.13E-01
cg21674704	-16.19	6.43	1.18E-02	-16.98	10.36	1.01E-01	-0.80	18.57	9.66E-01	-16.51	13.22	2.12E-01	-21.87	12.66	8.41E-02
cg22785629	21.91	8.70	1.18E-02	20.55	14.59	1.59E-01	13.60	16.78	4.18E-01	39.49	18.75	3.52E-02	15.23	21.78	4.85E-01
cg09825309	10.52	4.18	1.18E-02	12.78	6.40	4.60E-02	14.70	8.66	8.95E-02	-3.83	10.58	7.17E-01	12.17	9.72	2.11E-01
cg17331738	26.03	10.34	1.18E-02	14.99	37.70	6.91E-01	-9.49	74.79	8.99E-01	31.18	11.33	5.91E-03	-12.47	38.47	7.46E-01
cg05962028	-7.40	2.94	1.18E-02	-5.68	4.21	1.77E-01	-14.43	6.44	2.51E-02	2.01	11.08	8.56E-01	-7.57	6.09	2.14E-01
cg07558275	14.19	5.64	1.18E-02	5.79	8.72	5.07E-01	29.76	16.95	7.90E-02	26.16	11.73	2.58E-02	10.14	11.50	3.78E-01
cg01382137	23.87	9.49	1.18E-02	29.10	13.77	3.46E-02	7.06	28.90	8.07E-01	33.91	21.94	1.22E-01	12.85	19.75	5.15E-01
cg05258834	-19.84	7.88	1.18E-02	-19.53	14.33	1.73E-01	-9.14	15.58	5.58E-01	-23.21	14.87	1.19E-01	-31.54	19.66	1.09E-01
cg03832697	19.15	7.61	1.18E-02	25.17	15.89	1.13E-01	34.21	46.83	4.65E-01	14.37	11.07	1.94E-01	20.92	14.60	1.52E-01

cg06987348	-8.69	3.45	1.19E-02	-0.60	7.56	9.36E-01	-17.97	10.83	9.72E-02	-8.51	4.55	6.18E-02	-16.21	10.23	1.13E-01
cg27059970	-16.70	6.64	1.19E-02	-21.09	9.35	2.41E-02	-16.57	15.80	2.94E-01	-13.29	16.70	4.26E-01	-6.54	16.47	6.91E-01
cg08258867	30.35	12.06	1.19E-02	66.55	18.54	3.33E-04	17.03	17.68	3.36E-01	16.88	16.19	2.97E-01	22.57	21.45	2.93E-01
cg06382546	-7.56	3.01	1.19E-02	-6.67	6.99	3.40E-01	-4.96	7.47	5.06E-01	-7.48	4.05	6.45E-02	-13.89	9.48	1.43E-01
cg03102494	-4.31	1.71	1.19E-02	-7.22	2.60	5.45E-03	-0.61	3.32	8.55E-01	-4.55	5.16	3.78E-01	-2.68	3.93	4.94E-01
cg24363146	-34.25	13.61	1.19E-02	-16.24	48.82	7.39E-01	-124.68	208.32	5.50E-01	-34.20	14.61	1.93E-02	-55.59	60.96	3.62E-01
cg05366561	-6.47	2.57	1.19E-02	-5.46	3.79	1.49E-01	-9.12	6.04	1.31E-01	-1.45	7.67	8.50E-01	-8.68	5.18	9.40E-02
cg01489177	15.37	6.11	1.19E-02	11.85	9.45	2.10E-01	24.98	11.31	2.72E-02	18.47	18.18	3.10E-01	5.88	14.52	6.86E-01
cg19030682	-7.68	3.05	1.19E-02	-7.05	4.33	1.03E-01	-4.74	8.29	5.68E-01	-13.00	8.26	1.16E-01	-7.60	6.35	2.31E-01
cg14016089	15.03	5.97	1.19E-02	30.96	16.85	6.63E-02	-2.71	51.28	9.58E-01	14.37	6.76	3.37E-02	-0.41	21.09	9.85E-01
cg14938738	20.69	8.22	1.19E-02	15.58	11.03	1.58E-01	8.15	24.66	7.41E-01	25.24	23.65	2.86E-01	38.06	17.86	3.30E-02
cg21702971	-26.90	10.69	1.19E-02	-38.60	13.72	4.92E-03	4.47	18.91	8.13E-01	-48.70	21.74	2.51E-02	-23.54	16.90	1.64E-01
cg00429402	26.89	10.69	1.19E-02	5.64	25.76	8.27E-01	5.69	36.51	8.76E-01	30.88	13.53	2.24E-02	52.29	31.18	9.36E-02
cg13050258	-17.54	6.97	1.19E-02	-1.81	19.35	9.25E-01	-2.47	37.01	9.47E-01	-19.41	7.94	1.45E-02	-35.52	27.76	2.01E-01
cg19785918	9.09	3.61	1.19E-02	9.82	5.33	6.57E-02	13.84	7.35	5.96E-02	3.73	14.12	7.92E-01	4.25	7.48	5.70E-01
cg15547624	31.24	12.42	1.19E-02	11.62	30.09	6.99E-01	-171.46	187.79	3.61E-01	37.91	14.21	7.64E-03	17.23	49.81	7.29E-01
cg19809165	-40.82	16.23	1.19E-02	-23.69	24.11	3.26E-01	-76.48	41.57	6.58E-02	-2.27	41.76	9.57E-01	-71.71	31.75	2.39E-02
cg12296269	-15.95	6.34	1.19E-02	-16.18	10.67	1.29E-01	-12.39	13.34	3.53E-01	-25.72	16.47	1.18E-01	-13.29	12.14	2.74E-01
cg01800521	17.00	6.76	1.19E-02	31.49	11.48	6.09E-03	21.09	15.65	1.78E-01	9.98	16.68	5.49E-01	4.10	11.29	7.17E-01
cg23689615	10.59	4.21	1.19E-02	12.79	5.58	2.18E-02	18.38	11.26	1.02E-01	-2.05	13.40	8.78E-01	4.85	9.63	6.15E-01
cg15035278	-23.27	9.25	1.19E-02	-25.46	10.45	1.49E-02	-22.35	32.63	4.93E-01	-41.64	50.01	4.05E-01	-0.95	29.02	9.74E-01
cg07202012	-14.85	5.91	1.19E-02	-12.54	10.49	2.32E-01	-7.24	17.50	6.79E-01	-18.87	9.02	3.65E-02	-14.01	15.77	3.74E-01
cg13662628	-20.73	8.24	1.19E-02	-19.12	16.76	2.54E-01	7.24	20.47	7.24E-01	-26.35	13.29	4.74E-02	-33.82	17.94	5.94E-02
cg23498925	-16.70	6.64	1.19E-02	-20.14	14.22	1.57E-01	-11.62	17.34	5.03E-01	-19.76	9.12	3.03E-02	-1.23	20.49	9.52E-01
cg12888712	-11.59	4.61	1.19E-02	-24.32	8.76	5.48E-03	-6.45	15.00	6.67E-01	-7.86	6.45	2.23E-01	-3.00	12.62	8.12E-01
cg03707634	-14.87	5.91	1.19E-02	-13.05	10.53	2.15E-01	-14.19	14.82	3.38E-01	-16.02	8.92	7.26E-02	-16.97	20.19	4.01E-01
cg05753675	-59.73	23.75	1.19E-02	-62.37	65.34	3.40E-01	-77.51	223.17	7.28E-01	-54.00	28.10	5.47E-02	-84.65	63.01	1.79E-01
cg00297584	40.71	16.19	1.19E-02	34.28	23.05	1.37E-01	42.26	49.99	3.98E-01	30.64	40.58	4.50E-01	59.70	32.87	6.93E-02
cg20749005	27.10	10.78	1.19E-02	38.11	17.39	2.84E-02	29.83	51.83	5.65E-01	7.62	16.75	6.49E-01	50.47	27.03	6.19E-02
cg25471916	39.58	15.74	1.19E-02	47.33	19.30	1.42E-02	1.17	55.37	9.83E-01	38.53	50.91	4.49E-01	27.26	39.54	4.91E-01
cg00168118	19.46	7.74	1.19E-02	14.86	13.67	2.77E-01	16.34	15.76	3.00E-01	23.61	15.08	1.17E-01	25.97	18.53	1.61E-01
cg09185797	-15.91	6.33	1.19E-02	-2.62	16.09	8.71E-01	-3.27	29.38	9.11E-01	-18.60	7.42	1.22E-02	-25.37	23.56	2.82E-01
cg06706894	13.70	5.45	1.19E-02	19.96	14.18	1.59E-01	29.78	42.12	4.80E-01	11.22	6.16	6.85E-02	27.97	23.78	2.40E-01
cg20385229	-11.14	4.43	1.19E-02	-15.39	8.10	5.75E-02	-0.79	11.08	9.43E-01	-10.51	7.54	1.63E-01	-14.26	10.05	1.56E-01
cg25482146	10.17	4.05	1.19E-02	10.45	5.64	6.39E-02	8.46	9.06	3.50E-01	19.44	13.34	1.45E-01	6.79	9.22	4.61E-01

cg11008718	-37.58	14.95	1.19E-02	-9.71	38.27	8.00E-01	84.69	195.40	6.65E-01	-46.66	16.97	5.97E-03	-6.07	58.25	9.17E-01
cg05839748	21.25	8.45	1.19E-02	22.89	16.85	1.74E-01	20.93	20.36	3.04E-01	19.29	12.01	1.08E-01	28.82	29.73	3.32E-01
cg12195984	33.01	13.13	1.19E-02	12.16	37.57	7.46E-01	-90.03	172.50	6.02E-01	30.73	14.71	3.68E-02	100.29	47.79	3.58E-02
cg06524192	-15.79	6.28	1.19E-02	-1.66	16.38	9.19E-01	6.41	32.63	8.44E-01	-21.36	7.27	3.32E-03	1.94	23.60	9.35E-01
cg13017471	15.43	6.14	1.19E-02	23.98	11.43	3.60E-02	14.84	14.45	3.04E-01	12.16	9.34	1.93E-01	5.94	19.51	7.61E-01
cg22874802	16.52	6.57	1.19E-02	17.39	10.81	1.08E-01	15.09	13.56	2.66E-01	20.46	19.22	2.87E-01	14.93	12.46	2.31E-01
cg02582218	-9.19	3.66	1.20E-02	-10.81	9.08	2.34E-01	-15.04	16.10	3.50E-01	-9.26	4.41	3.56E-02	-2.90	11.74	8.05E-01
cg05519606	-10.99	4.37	1.20E-02	-15.40	10.73	1.51E-01	-0.18	18.58	9.92E-01	-7.75	4.95	1.17E-01	-30.51	13.26	2.14E-02
cg00186529	6.92	2.75	1.20E-02	2.30	4.00	5.65E-01	11.92	6.23	5.56E-02	11.25	8.34	1.78E-01	10.20	5.83	7.98E-02
cg04205943	21.11	8.40	1.20E-02	32.18	21.80	1.40E-01	36.80	30.88	2.33E-01	20.13	11.27	7.40E-02	10.93	17.82	5.40E-01
cg06839631	14.91	5.93	1.20E-02	28.91	12.36	1.93E-02	5.51	17.78	7.57E-01	11.47	10.19	2.60E-01	11.74	10.50	2.64E-01
cg27300829	11.10	4.42	1.20E-02	18.17	5.78	1.66E-03	5.14	8.82	5.60E-01	16.50	8.90	6.36E-02	0.83	7.83	9.16E-01
cg25354617	27.88	11.09	1.20E-02	29.34	15.47	5.79E-02	32.28	22.89	1.58E-01	31.05	33.58	3.55E-01	12.88	29.44	6.62E-01
cg09986774	18.08	7.19	1.20E-02	13.68	16.20	3.98E-01	58.36	58.07	3.15E-01	18.02	8.59	3.59E-02	21.42	24.55	3.83E-01
cg22489931	-8.19	3.26	1.20E-02	-8.64	4.48	5.38E-02	-10.44	8.24	2.05E-01	-6.47	8.75	4.59E-01	-6.17	7.74	4.26E-01
cg24595580	7.42	2.95	1.20E-02	7.19	3.87	6.31E-02	13.28	7.55	7.85E-02	10.37	8.01	1.95E-01	-1.60	8.21	8.45E-01
cg01135648	7.56	3.01	1.20E-02	8.10	3.92	3.90E-02	8.29	9.08	3.61E-01	7.26	7.57	3.37E-01	5.12	7.91	5.17E-01
cg26663490	9.97	3.97	1.20E-02	9.22	5.34	8.44E-02	10.31	8.59	2.30E-01	13.74	12.89	2.87E-01	9.84	10.58	3.53E-01
cg24342013	10.96	4.36	1.20E-02	6.11	5.99	3.08E-01	11.05	8.26	1.81E-01	21.04	19.25	2.75E-01	25.46	11.67	2.92E-02
cg16423812	31.40	12.50	1.20E-02	24.45	44.42	5.82E-01	170.00	210.61	4.20E-01	32.25	13.45	1.65E-02	18.83	54.12	7.28E-01
cg11999580	-10.49	4.18	1.20E-02	-20.94	8.85	1.81E-02	-7.78	13.78	5.72E-01	-5.18	5.45	3.42E-01	-20.89	13.23	1.14E-01
cg25713684	-34.55	13.76	1.20E-02	-43.32	16.79	9.88E-03	3.17	58.20	9.57E-01	40.87	54.62	4.54E-01	-39.36	30.04	1.90E-01
cg06788514	-15.53	6.18	1.20E-02	-17.40	8.42	3.88E-02	-20.28	13.71	1.39E-01	-9.48	12.66	4.54E-01	12.23	44.91	7.85E-01
cg24754507	22.98	9.15	1.20E-02	41.01	20.30	4.34E-02	38.67	32.32	2.32E-01	18.92	11.62	1.03E-01	-1.91	29.45	9.48E-01
cg20735050	-15.24	6.07	1.20E-02	-29.67	17.39	8.81E-02	-17.98	24.27	4.59E-01	-15.93	7.63	3.67E-02	-2.32	14.19	8.70E-01
cg06742778	-17.05	6.79	1.20E-02	-28.84	11.08	9.26E-03	-8.02	15.25	5.99E-01	-6.57	15.18	6.65E-01	-14.68	14.25	3.03E-01
cg26039482	-23.23	9.25	1.20E-02	-13.29	15.19	3.82E-01	-43.47	20.50	3.39E-02	-12.55	11.05	2.56E-01	-54.24	24.75	2.84E-02
cg08964929	12.06	4.80	1.20E-02	10.19	10.66	3.39E-01	12.58	15.79	4.26E-01	16.50	6.26	8.34E-03	-7.76	14.14	5.83E-01
cg08747676	-10.92	4.35	1.20E-02	-6.95	6.25	2.66E-01	-16.83	10.76	1.18E-01	-19.65	13.00	1.31E-01	-10.82	8.84	2.21E-01
cg23733744	27.32	10.88	1.20E-02	60.42	30.97	5.11E-02	32.05	106.49	7.63E-01	24.23	12.26	4.80E-02	5.70	38.77	8.83E-01
cg26157756	34.17	13.61	1.20E-02	50.51	17.80	4.55E-03	-41.52	59.90	4.88E-01	55.15	48.97	2.60E-01	18.28	21.07	3.86E-01
cg10784258	-9.65	3.84	1.20E-02	-15.47	9.94	1.20E-01	1.85	19.71	9.25E-01	-8.71	4.58	5.72E-02	-11.77	11.67	3.13E-01
cg12833765	-10.73	4.27	1.20E-02	-13.13	7.15	6.64E-02	-8.28	10.13	4.14E-01	-13.22	8.24	1.09E-01	-5.19	9.63	5.90E-01
cg04315434	33.62	13.39	1.20E-02	13.41	47.36	7.77E-01	-14.22	201.70	9.44E-01	32.97	14.45	2.25E-02	75.36	55.99	1.78E-01
cg20984924	-19.33	7.70	1.20E-02	-23.18	18.42	2.08E-01	-4.34	23.11	8.51E-01	-23.05	9.96	2.07E-02	-8.81	22.49	6.95E-01

cg09556823	22.31	8.89	1.20E-02	31.55	15.84	4.64E-02	16.05	35.90	6.55E-01	15.84	12.41	2.02E-01	29.51	26.66	2.68E-01
cg24376214	-9.26	3.69	1.20E-02	-12.71	6.73	5.88E-02	-5.27	14.60	7.18E-01	-5.62	5.18	2.79E-01	-17.44	10.24	8.86E-02
cg00469800	11.50	4.58	1.20E-02	9.60	5.59	8.61E-02	27.11	11.21	1.56E-02	17.88	15.36	2.45E-01	1.65	9.92	8.68E-01
cg02920992	-17.31	6.89	1.21E-02	-10.04	13.80	4.67E-01	-47.57	30.59	1.20E-01	-17.69	8.92	4.73E-02	-17.83	21.64	4.10E-01
cg09039367	17.56	6.99	1.21E-02	30.34	16.94	7.32E-02	4.19	31.57	8.94E-01	12.22	8.38	1.45E-01	43.94	24.23	6.97E-02
cg13527203	-16.19	6.45	1.21E-02	-19.63	8.69	2.38E-02	-14.39	25.56	5.73E-01	-24.84	22.06	2.60E-01	-7.78	11.77	5.09E-01
cg15701332	-16.30	6.49	1.21E-02	-5.10	17.00	7.64E-01	17.04	30.27	5.73E-01	-22.25	7.57	3.30E-03	0.27	24.06	9.91E-01
cg21387147	18.88	7.52	1.21E-02	34.98	19.23	6.89E-02	19.87	25.71	4.40E-01	12.21	9.10	1.79E-01	44.51	26.88	9.77E-02
cg14207147	11.31	4.51	1.21E-02	13.84	6.36	2.95E-02	-3.15	10.97	7.74E-01	20.32	12.27	9.77E-02	11.11	10.25	2.78E-01
cg23561531	-13.01	5.18	1.21E-02	-17.33	7.74	2.52E-02	-8.20	12.63	5.16E-01	-11.11	13.78	4.20E-01	-9.45	10.53	3.69E-01
cg08559424	-17.05	6.79	1.21E-02	-24.46	10.99	2.61E-02	-23.41	14.31	1.02E-01	-11.38	15.58	4.65E-01	-1.39	15.06	9.27E-01
cg18905161	-11.90	4.74	1.21E-02	-13.70	7.31	6.09E-02	-10.02	9.59	2.96E-01	1.58	13.17	9.04E-01	-18.94	10.46	7.00E-02
cg11199137	-35.40	14.10	1.21E-02	-33.30	52.87	5.29E-01	-193.01	228.89	3.99E-01	-29.61	15.26	5.23E-02	-99.05	53.05	6.19E-02
cg24536782	-2.70	1.08	1.21E-02	-3.30	1.61	3.98E-02	-3.93	1.92	4.12E-02	-0.74	3.87	8.49E-01	0.45	2.70	8.69E-01
cg22483610	14.45	5.76	1.21E-02	22.41	8.79	1.08E-02	16.02	16.52	3.32E-01	14.47	13.35	2.79E-01	0.76	11.21	9.46E-01
cg19447630	-28.49	11.35	1.21E-02	-32.67	15.55	3.57E-02	8.93	23.99	7.10E-01	-44.62	31.13	1.52E-01	-45.05	22.52	4.54E-02
cg04797742	12.46	4.96	1.21E-02	4.71	7.57	5.34E-01	10.10	10.25	3.25E-01	18.65	16.76	2.66E-01	25.60	9.86	9.43E-03
cg14990262	-30.79	12.27	1.21E-02	-62.18	45.58	1.73E-01	-159.89	144.16	2.67E-01	-23.25	13.27	7.98E-02	-79.82	47.78	9.48E-02
cg12612091	18.75	7.47	1.21E-02	18.83	20.01	3.47E-01	21.59	37.19	5.62E-01	18.92	8.62	2.82E-02	15.09	28.50	5.96E-01
cg21870884	14.46	5.76	1.21E-02	17.59	9.13	5.42E-02	7.98	16.58	6.30E-01	18.39	10.75	8.71E-02	6.26	13.08	6.32E-01
cg08324862	-8.94	3.56	1.21E-02	-14.62	5.52	8.05E-03	-3.59	8.65	6.78E-01	-10.79	6.09	7.64E-02	2.44	8.71	7.79E-01
cg11592786	-34.09	13.58	1.21E-02	-41.36	23.53	7.88E-02	-20.62	47.95	6.67E-01	-48.46	26.91	7.18E-02	-19.00	23.59	4.21E-01
cg18460375	-15.19	6.05	1.21E-02	-27.59	15.62	7.74E-02	-6.40	19.98	7.49E-01	-11.58	7.39	1.17E-01	-30.53	20.33	1.33E-01
cg10608476	-8.24	3.28	1.21E-02	-7.93	4.73	9.36E-02	-11.30	7.84	1.50E-01	-5.02	10.95	6.47E-01	-7.86	6.53	2.29E-01
cg07566790	-17.59	7.01	1.21E-02	-8.70	18.96	6.46E-01	-49.56	24.13	4.00E-02	-17.35	8.21	3.46E-02	8.97	31.69	7.77E-01
cg21142977	-51.81	20.65	1.21E-02	-50.95	24.75	3.95E-02	-150.97	87.16	8.33E-02	-5.36	84.78	9.50E-01	-40.09	47.57	3.99E-01
cg11022060	-9.50	3.79	1.21E-02	-15.09	6.00	1.19E-02	-0.95	6.47	8.83E-01	-14.81	5.24	4.70E-03	-2.73	8.28	7.42E-01
cg01641455	25.66	10.23	1.21E-02	18.08	13.01	1.65E-01	38.74	40.20	3.35E-01	108.27	58.81	6.56E-02	30.32	19.09	1.12E-01
cg02628360	15.02	5.99	1.21E-02	23.14	11.12	3.74E-02	22.78	17.42	1.91E-01	8.17	9.42	3.86E-01	12.34	13.81	3.71E-01
cg12133393	35.30	14.07	1.21E-02	18.66	35.15	5.96E-01	-72.13	113.29	5.24E-01	41.39	16.37	1.15E-02	33.29	48.10	4.89E-01
cg19818826	11.74	4.68	1.21E-02	12.92	6.47	4.59E-02	16.34	20.21	4.19E-01	15.02	12.75	2.39E-01	7.22	8.72	4.07E-01
cg24336646	43.93	17.51	1.21E-02	45.04	36.12	2.13E-01	-125.77	103.41	2.24E-01	45.81	18.86	1.52E-02	86.35	61.93	1.63E-01
cg03775240	97.56	38.89	1.21E-02	68.14	57.89	2.39E-01	321.90	178.75	7.17E-02	148.69	90.60	1.01E-01	76.21	69.08	2.70E-01
cg04943741	6.83	2.72	1.21E-02	6.00	3.35	7.30E-02	14.28	7.60	6.04E-02	10.52	9.36	2.61E-01	1.09	7.69	8.87E-01
cg18596947	-24.47	9.75	1.21E-02	-40.38	13.79	3.41E-03	5.12	25.60	8.41E-01	-26.16	20.14	1.94E-01	-8.95	20.85	6.68E-01

cg23855715	-10.80	4.31	1.21E-02	-12.57	9.63	1.92E-01	-21.90	13.52	1.05E-01	-7.65	5.70	1.80E-01	-13.30	12.03	2.69E-01
cg26382678	-10.87	4.33	1.21E-02	-10.53	6.33	9.64E-02	-3.18	8.43	7.06E-01	-18.18	14.91	2.23E-01	-19.48	10.13	5.46E-02
cg25250650	-11.97	4.77	1.21E-02	-17.83	6.96	1.04E-02	-4.49	11.28	6.91E-01	-5.31	12.95	6.82E-01	-9.59	10.31	3.52E-01
cg00405677	26.02	10.37	1.21E-02	40.58	16.38	1.33E-02	62.25	34.13	6.81E-02	18.51	15.82	2.42E-01	3.08	20.04	8.78E-01
cg17465304	10.15	4.05	1.21E-02	5.57	6.10	3.61E-01	6.88	7.31	3.46E-01	8.76	6.52	1.79E-01	26.59	9.37	4.56E-03
cg10172250	34.28	13.67	1.22E-02	15.12	26.26	5.65E-01	67.37	69.06	3.29E-01	43.70	18.45	1.79E-02	25.26	36.43	4.88E-01
cg09773473	16.67	6.65	1.22E-02	25.80	12.24	3.50E-02	35.37	21.45	9.91E-02	12.28	9.14	1.79E-01	-10.40	23.51	6.58E-01
cg02567879	13.16	5.25	1.22E-02	12.90	8.27	1.19E-01	5.00	10.02	6.17E-01	24.84	13.03	5.67E-02	16.00	13.13	2.23E-01
cg04023641	10.68	4.26	1.22E-02	8.91	6.25	1.53E-01	13.88	8.92	1.19E-01	27.71	12.91	3.18E-02	1.75	9.59	8.55E-01
cg14134497	13.46	5.37	1.22E-02	20.28	9.23	2.80E-02	20.52	15.90	1.97E-01	12.22	9.92	2.18E-01	2.68	10.63	8.01E-01
cg08039560	-8.40	3.35	1.22E-02	-8.79	4.35	4.36E-02	-4.84	8.06	5.48E-01	-10.84	11.30	3.37E-01	-9.56	8.73	2.73E-01
cg12972233	12.74	5.08	1.22E-02	14.71	7.69	5.58E-02	8.88	14.10	5.29E-01	15.23	9.61	1.13E-01	5.90	12.93	6.48E-01
cg16295461	18.97	7.57	1.22E-02	10.67	12.37	3.89E-01	-8.94	21.18	6.73E-01	30.06	9.25	1.16E-03	23.95	18.36	1.92E-01
cg19649018	-17.49	6.98	1.22E-02	-27.91	9.94	4.99E-03	-1.42	15.95	9.29E-01	-1.01	20.11	9.60E-01	-17.11	15.79	2.78E-01
cg16553297	-8.69	3.47	1.22E-02	-12.89	6.32	4.14E-02	-4.39	10.85	6.86E-01	-6.37	5.26	2.26E-01	-9.81	8.59	2.53E-01
cg19350115	-9.64	3.84	1.22E-02	-11.67	8.01	1.45E-01	7.14	17.06	6.75E-01	-12.02	4.93	1.48E-02	0.00	11.56	1.00E+00
cg07683636	11.95	4.77	1.22E-02	10.46	6.56	1.11E-01	20.74	12.13	8.75E-02	13.53	12.12	2.64E-01	6.97	11.81	5.55E-01
ch.21.39866785R	-51.75	20.65	1.22E-02	-60.48	26.45	2.22E-02	-57.30	121.49	6.37E-01	-9.09	89.35	9.19E-01	-41.40	37.16	2.65E-01
cg12841566	17.28	6.89	1.22E-02	31.93	21.10	1.30E-01	16.00	24.40	5.12E-01	16.09	7.92	4.22E-02	7.33	29.02	8.01E-01
cg04189187	-10.02	4.00	1.22E-02	-13.57	5.99	2.35E-02	-6.28	14.71	6.70E-01	-11.31	9.90	2.53E-01	-5.25	7.10	4.60E-01
cg05735765	21.50	8.58	1.22E-02	17.08	10.78	1.13E-01	21.11	36.37	5.62E-01	76.38	45.99	9.68E-02	24.77	16.29	1.28E-01
cg22032364	-8.53	3.40	1.22E-02	-13.25	7.23	6.67E-02	-3.90	12.09	7.47E-01	-7.09	4.59	1.22E-01	-9.24	8.80	2.94E-01
cg04442328	19.16	7.64	1.22E-02	37.47	16.45	2.27E-02	-25.42	38.11	5.05E-01	17.00	9.60	7.66E-02	11.98	23.10	6.04E-01
cg06601071	-7.88	3.14	1.22E-02	-8.96	4.73	5.83E-02	-5.77	6.81	3.97E-01	-7.66	7.03	2.76E-01	-7.98	8.23	3.32E-01
cg22398226	-9.07	3.62	1.22E-02	-11.25	8.08	1.64E-01	1.66	13.63	9.03E-01	-8.91	4.66	5.58E-02	-12.37	10.20	2.25E-01
cg14241609	-77.25	30.82	1.22E-02	-61.80	42.17	1.43E-01	-126.90	128.12	3.22E-01	-76.35	69.24	2.70E-01	-103.76	67.30	1.23E-01
cg00821413	24.78	9.89	1.22E-02	29.57	19.46	1.29E-01	19.27	22.94	4.01E-01	27.79	14.92	6.25E-02	11.65	28.89	6.87E-01
cg26667946	10.35	4.13	1.22E-02	10.26	5.73	7.32E-02	21.29	13.91	1.26E-01	28.60	19.29	1.38E-01	5.28	7.03	4.53E-01
cg14045814	21.17	8.45	1.22E-02	33.19	14.56	2.26E-02	19.43	16.28	2.33E-01	4.06	17.19	8.13E-01	24.86	21.67	2.51E-01
cg05764847	41.85	16.70	1.22E-02	58.22	21.84	7.67E-03	27.53	57.78	6.34E-01	-4.87	44.07	9.12E-01	32.97	38.49	3.92E-01
cg17798563	12.31	4.91	1.22E-02	7.65	7.98	3.38E-01	16.64	9.52	8.04E-02	29.30	17.33	9.09E-02	9.56	9.38	3.08E-01
cg00580652	-11.13	4.44	1.22E-02	-3.32	5.65	5.57E-01	-23.10	9.56	1.57E-02	-9.82	8.94	2.72E-01	-16.46	8.38	4.95E-02
cg02996796	17.97	7.17	1.22E-02	19.30	14.70	1.89E-01	30.87	38.61	4.24E-01	16.00	8.88	7.17E-02	24.97	26.15	3.40E-01
cg10708955	10.78	4.30	1.22E-02	11.84	5.45	2.98E-02	17.36	11.98	1.47E-01	11.23	16.91	5.07E-01	2.38	10.05	8.13E-01
cg06791380	27.78	11.09	1.22E-02	26.35	18.49	1.54E-01	62.03	26.35	1.86E-02	13.27	6.67	4.68E-02	51.54	27.94	6.51E-02

cg24882875	19.56	7.81	1.22E-02	5.52	13.06	6.72E-01	14.08	23.41	5.48E-01	26.91	13.23	4.19E-02	36.33	18.25	4.65E-02
cg25528199	32.19	12.85	1.22E-02	30.22	12.32	1.42E-02	95.09	59.86	1.12E-01	-15.27	43.69	7.27E-01	79.40	52.82	1.33E-01
cg13320014	51.13	20.41	1.22E-02	48.72	27.51	7.65E-02	-51.28	88.18	5.61E-01	49.73	65.86	4.50E-01	74.26	37.25	4.62E-02
cg24197477	9.50	3.79	1.22E-02	10.12	5.59	7.00E-02	6.58	8.88	4.59E-01	17.11	7.77	2.75E-02	-3.71	10.99	7.36E-01
cg17993073	28.87	11.52	1.22E-02	24.12	18.44	1.91E-01	18.03	33.81	5.94E-01	28.83	33.12	3.84E-01	37.27	18.89	4.85E-02
cg03045721	-51.33	20.49	1.22E-02	14.20	52.85	7.88E-01	-78.77	265.02	7.66E-01	-67.62	24.09	5.00E-03	-33.90	59.03	5.66E-01
cg24296036	22.64	9.04	1.22E-02	23.21	19.29	2.29E-01	23.16	19.90	2.44E-01	11.13	14.26	4.35E-01	47.98	21.72	2.72E-02
cg27159979	-12.15	4.85	1.22E-02	-18.39	7.03	8.90E-03	-9.18	11.55	4.27E-01	-3.94	12.30	7.49E-01	-6.07	11.04	5.82E-01
cg09182678	-18.62	7.43	1.22E-02	-12.06	10.91	2.69E-01	-23.41	21.02	2.65E-01	-6.63	27.01	8.06E-01	-28.59	12.83	2.58E-02
cg13545212	16.49	6.58	1.22E-02	21.30	14.36	1.38E-01	20.92	28.19	4.58E-01	17.28	8.40	3.95E-02	2.06	18.97	9.13E-01
cg01156834	15.04	6.01	1.22E-02	10.61	7.43	1.53E-01	31.81	12.62	1.17E-02	24.23	11.49	3.50E-02	2.74	9.59	7.75E-01
cg02612026	39.56	15.79	1.22E-02	36.21	20.15	7.23E-02	52.45	73.05	4.73E-01	96.98	76.89	2.07E-01	36.29	28.98	2.11E-01
cg22784836	-12.08	4.82	1.22E-02	-20.42	7.21	4.63E-03	-4.98	9.75	6.10E-01	-1.78	16.67	9.15E-01	-7.06	10.18	4.88E-01
cg22289815	17.60	7.03	1.22E-02	21.55	10.54	4.08E-02	-0.09	16.60	9.96E-01	21.08	13.00	1.05E-01	22.42	24.34	3.57E-01
cg21992932	6.52	2.60	1.23E-02	7.40	3.56	3.75E-02	7.08	5.76	2.18E-01	17.06	13.00	1.89E-01	1.94	5.55	7.27E-01
cg04763519	9.51	3.80	1.23E-02	10.07	5.61	7.25E-02	7.32	7.61	3.36E-01	5.03	12.92	6.97E-01	12.81	8.38	1.27E-01
cg23313445	-16.98	6.78	1.23E-02	-25.16	18.69	1.78E-01	10.52	29.20	7.19E-01	-14.48	7.95	6.87E-02	-42.35	22.91	6.45E-02
cg19513282	29.02	11.59	1.23E-02	33.99	16.42	3.85E-02	20.68	23.54	3.80E-01	-6.52	37.40	8.62E-01	47.16	28.68	1.00E-01
cg02711190	26.70	10.66	1.23E-02	17.61	33.84	6.03E-01	-92.86	123.97	4.54E-01	32.94	11.79	5.21E-03	-17.31	38.84	6.56E-01
cg24941342	6.38	2.55	1.23E-02	4.18	3.18	1.89E-01	10.28	6.79	1.30E-01	16.51	7.16	2.11E-02	2.94	6.63	6.58E-01
cg00128702	18.78	7.50	1.23E-02	21.65	14.70	1.41E-01	15.01	22.07	4.96E-01	28.31	11.78	1.63E-02	-0.35	16.05	9.82E-01
cg23325364	-16.20	6.47	1.23E-02	-27.13	8.67	1.76E-03	0.38	16.04	9.81E-01	-3.35	17.65	8.49E-01	-12.65	12.37	3.07E-01
cg07726139	50.46	20.16	1.23E-02	21.65	15.61	1.65E-01	74.35	39.45	5.95E-02	131.22	47.48	5.72E-03	38.48	26.16	1.41E-01
cg16867023	26.39	10.54	1.23E-02	31.74	18.32	8.32E-02	-31.57	37.44	3.99E-01	36.33	14.83	1.43E-02	14.37	27.28	5.98E-01
cg02718078	-14.29	5.71	1.23E-02	-15.14	14.70	3.03E-01	-51.56	31.22	9.87E-02	-11.88	6.61	7.24E-02	-20.19	21.46	3.47E-01
cg12516621	-35.83	14.32	1.23E-02	-68.90	46.99	1.43E-01	-190.90	191.77	3.20E-01	-27.54	15.79	8.12E-02	-72.01	50.74	1.56E-01
cg05370193	20.72	8.28	1.23E-02	36.31	13.41	6.76E-03	18.48	15.93	2.46E-01	1.06	18.33	9.54E-01	11.53	21.80	5.97E-01
cg12020794	24.26	9.70	1.23E-02	25.60	16.48	1.20E-01	26.35	17.93	1.42E-01	26.93	19.72	1.72E-01	9.96	28.00	7.22E-01
cg15169189	22.95	9.17	1.23E-02	26.40	12.54	3.52E-02	-0.71	40.20	9.86E-01	11.07	45.71	8.09E-01	22.59	15.02	1.33E-01
cg21376908	20.59	8.23	1.23E-02	26.16	15.84	9.87E-02	23.92	17.83	1.80E-01	25.16	20.39	2.17E-01	12.25	13.82	3.75E-01
cg13754437	19.99	7.99	1.23E-02	31.92	22.54	1.57E-01	1.89	34.13	9.56E-01	19.10	9.18	3.74E-02	22.71	32.11	4.80E-01
cg24468070	10.92	4.36	1.23E-02	20.15	13.16	1.26E-01	-7.47	15.23	6.24E-01	14.88	6.25	1.73E-02	6.45	7.71	4.03E-01
cg07491809	-59.25	23.68	1.23E-02	-42.55	32.44	1.90E-01	-70.07	90.04	4.36E-01	-109.36	35.81	2.26E-03	-2.05	50.05	9.67E-01
cg24248367	-14.14	5.65	1.23E-02	-21.69	14.22	1.27E-01	-29.32	25.91	2.58E-01	-12.96	6.68	5.24E-02	-0.52	20.21	9.80E-01
cg00796963	7.98	3.19	1.24E-02	9.59	4.12	1.99E-02	7.62	8.02	3.42E-01	7.96	11.21	4.78E-01	2.37	7.95	7.66E-01

cg10829227	19.68	7.86	1.24E-02	9.04	13.34	4.98E-01	25.22	13.25	5.71E-02	12.26	20.07	5.41E-01	39.33	20.53	5.54E-02
cg12021671	13.73	5.49	1.24E-02	16.35	7.93	3.93E-02	7.11	12.26	5.62E-01	7.36	12.88	5.67E-01	22.65	14.75	1.25E-01
cg25988204	9.66	3.86	1.24E-02	6.81	5.41	2.08E-01	15.83	9.06	8.06E-02	8.91	14.42	5.37E-01	11.30	7.94	1.55E-01
cg14067541	-16.91	6.76	1.24E-02	-23.38	15.17	1.23E-01	6.17	23.81	7.95E-01	-16.41	8.46	5.24E-02	-27.64	23.46	2.39E-01
cg13492337	16.05	6.42	1.24E-02	8.68	19.71	6.60E-01	52.30	28.51	6.66E-02	13.59	7.20	5.90E-02	34.45	29.03	2.35E-01
cg05754688	-42.17	16.86	1.24E-02	-36.76	48.54	4.49E-01	-15.75	235.35	9.47E-01	-41.24	18.99	2.99E-02	-59.86	57.45	2.97E-01
cg26220061	27.86	11.14	1.24E-02	32.98	30.65	2.82E-01	12.95	102.93	9.00E-01	26.90	12.62	3.31E-02	31.00	39.99	4.38E-01
cg14948290	31.37	12.54	1.24E-02	20.83	17.45	2.33E-01	5.95	49.78	9.05E-01	63.64	66.17	3.36E-01	46.76	20.25	2.09E-02
cg08236767	11.48	4.59	1.24E-02	8.76	17.26	6.12E-01	25.38	31.73	4.24E-01	11.25	5.15	2.89E-02	12.14	13.52	3.69E-01
cg06756385	17.07	6.83	1.24E-02	8.94	9.17	3.30E-01	20.89	13.30	1.16E-01	43.74	17.54	1.26E-02	10.92	15.96	4.94E-01
cg19860160	12.79	5.12	1.24E-02	7.64	11.67	5.13E-01	7.40	16.42	6.52E-01	14.38	6.32	2.30E-02	21.23	21.55	3.24E-01
cg09643313	26.43	10.57	1.24E-02	26.36	15.60	9.10E-02	29.88	29.89	3.17E-01	64.33	30.07	3.24E-02	9.05	19.53	6.43E-01
cg27240008	45.29	18.11	1.24E-02	71.45	24.77	3.92E-03	12.71	59.21	8.30E-01	48.42	56.22	3.89E-01	3.26	34.99	9.26E-01
cg00459816	15.00	6.00	1.24E-02	28.98	12.27	1.82E-02	17.34	23.81	4.66E-01	9.35	7.42	2.08E-01	19.51	28.52	4.94E-01
cg18098089	18.86	7.54	1.24E-02	12.70	14.45	3.80E-01	29.93	18.74	1.10E-01	18.47	11.01	9.35E-02	19.58	24.33	4.21E-01
cg23717686	-31.73	12.69	1.24E-02	-33.40	39.26	3.95E-01	-235.15	150.57	1.18E-01	-30.69	13.97	2.80E-02	-19.66	50.49	6.97E-01
cg18337963	-38.42	15.37	1.24E-02	-44.50	19.75	2.42E-02	-46.51	66.84	4.87E-01	-42.86	54.60	4.32E-01	-21.40	30.02	4.76E-01
cg07770866	-23.06	9.22	1.24E-02	-20.71	13.37	1.21E-01	-28.21	21.17	1.83E-01	-39.48	28.83	1.71E-01	-16.41	19.16	3.92E-01
cg14180479	25.65	10.26	1.24E-02	0.86	21.23	9.68E-01	25.29	22.56	2.62E-01	30.05	15.59	5.39E-02	56.90	28.83	4.85E-02
cg19747911	-15.42	6.17	1.24E-02	-24.55	12.38	4.74E-02	-2.29	25.84	9.29E-01	-11.71	7.82	1.34E-01	-26.30	22.88	2.50E-01
cg17961932	22.99	9.20	1.24E-02	38.41	21.60	7.54E-02	35.86	34.06	2.92E-01	16.90	11.34	1.36E-01	26.16	31.05	4.00E-01
cg16549809	16.76	6.70	1.24E-02	17.42	10.01	8.20E-02	19.47	14.04	1.65E-01	12.53	17.29	4.69E-01	15.14	16.09	3.47E-01
cg07232642	-8.87	3.55	1.24E-02	-6.65	5.30	2.09E-01	-13.11	8.26	1.13E-01	-13.18	9.57	1.68E-01	-7.22	7.40	3.30E-01
cg08454563	-11.97	4.79	1.24E-02	-14.27	8.25	8.36E-02	-3.29	11.77	7.80E-01	-15.60	8.74	7.43E-02	-9.79	10.78	3.64E-01
cg08834573	-12.23	4.89	1.24E-02	-21.17	7.64	5.61E-03	-2.53	11.20	8.21E-01	-3.64	10.83	7.37E-01	-11.92	11.07	2.81E-01
cg05872706	27.24	10.90	1.24E-02	18.86	13.75	1.70E-01	9.86	40.32	8.07E-01	55.62	36.61	1.29E-01	46.39	23.79	5.12E-02
cg10696445	17.02	6.81	1.24E-02	26.61	10.45	1.09E-02	10.67	13.44	4.27E-01	-4.28	18.39	8.16E-01	19.64	15.98	2.19E-01
cg23493585	31.42	12.57	1.24E-02	26.42	15.56	8.95E-02	-18.92	38.95	6.27E-01	53.97	34.36	1.16E-01	58.77	28.60	3.99E-02
cg02185464	-12.62	5.05	1.24E-02	-13.36	6.96	5.48E-02	-11.77	22.34	5.98E-01	-26.21	13.83	5.80E-02	-5.14	9.39	5.84E-01
cg20294962	-18.80	7.52	1.25E-02	-18.29	12.66	1.49E-01	-3.59	13.12	7.84E-01	-16.88	13.49	2.11E-01	-42.54	15.71	6.78E-03
cg17156255	-47.92	19.18	1.25E-02	-133.88	60.24	2.62E-02	-105.28	258.98	6.84E-01	-42.79	21.36	4.51E-02	8.32	65.02	8.98E-01
cg19591881	30.34	12.14	1.25E-02	31.94	17.55	6.87E-02	40.64	29.98	1.75E-01	56.99	40.80	1.62E-01	12.43	23.43	5.96E-01
cg10833037	18.02	7.21	1.25E-02	26.72	11.44	1.96E-02	7.65	21.78	7.25E-01	5.76	16.81	7.32E-01	17.84	12.97	1.69E-01
cg23107161	34.31	13.73	1.25E-02	37.86	39.51	3.38E-01	-52.29	167.54	7.55E-01	31.07	15.24	4.15E-02	80.05	55.68	1.50E-01
cg19488158	-10.86	4.35	1.25E-02	-1.60	9.97	8.72E-01	-10.04	12.93	4.38E-01	-14.55	5.94	1.42E-02	-10.06	10.81	3.52E-01

cg00190412	19.75	7.91	1.25E-02	26.40	18.27	1.49E-01	23.29	25.03	3.52E-01	10.31	7.34	1.60E-01	46.23	18.59	1.29E-02
cg00623826	29.07	11.64	1.25E-02	46.41	24.38	5.70E-02	30.43	47.50	5.22E-01	28.13	15.16	6.34E-02	0.80	33.17	9.81E-01
cg08760493	7.79	3.12	1.25E-02	8.37	4.10	4.12E-02	14.06	8.73	1.07E-01	8.31	8.51	3.29E-01	0.22	7.83	9.78E-01
cg02448597	39.89	15.97	1.25E-02	34.88	19.38	7.19E-02	87.66	85.41	3.05E-01	-4.60	81.34	9.55E-01	53.88	32.13	9.36E-02
cg25975369	17.92	7.17	1.25E-02	27.25	16.02	8.90E-02	22.40	18.22	2.19E-01	14.89	9.60	1.21E-01	7.78	24.37	7.49E-01
cg16249340	17.90	7.17	1.25E-02	12.54	20.11	5.33E-01	36.75	37.33	3.25E-01	16.09	8.18	4.92E-02	38.21	27.50	1.65E-01
cg05465059	29.60	11.85	1.25E-02	36.95	15.36	1.61E-02	50.73	19.45	9.09E-03	32.93	16.00	3.95E-02	-15.78	23.68	5.05E-01
cg07044006	21.75	8.71	1.25E-02	12.52	17.06	4.63E-01	27.63	16.56	9.51E-02	13.68	14.95	3.60E-01	50.28	24.81	4.27E-02
cg17163527	13.30	5.33	1.25E-02	19.16	9.14	3.60E-02	18.22	11.98	1.28E-01	7.22	12.40	5.61E-01	6.69	10.10	5.08E-01
cg24986572	-49.59	19.86	1.25E-02	-57.33	25.35	2.37E-02	11.66	59.09	8.44E-01	-35.91	85.39	6.74E-01	-62.82	42.41	1.38E-01
cg06884401	9.67	3.87	1.25E-02	11.96	5.53	3.04E-02	7.43	9.49	4.33E-01	12.39	9.47	1.91E-01	2.76	9.27	7.66E-01
cg10135854	-21.60	8.65	1.25E-02	-32.31	16.07	4.44E-02	33.90	40.41	4.01E-01	-17.71	11.41	1.21E-01	-40.06	28.74	1.63E-01
cg22773899	21.96	8.79	1.25E-02	22.87	17.44	1.90E-01	25.91	23.59	2.72E-01	16.17	12.66	2.02E-01	38.12	24.92	1.26E-01
cg09454925	19.34	7.74	1.25E-02	33.14	10.27	1.25E-03	4.17	16.31	7.98E-01	25.35	20.42	2.14E-01	6.80	13.31	6.09E-01
cg11128212	-13.89	5.56	1.25E-02	-1.57	13.12	9.05E-01	-10.51	18.26	5.65E-01	-20.70	7.25	4.30E-03	-3.21	14.93	8.30E-01
cg19131103	34.09	13.65	1.25E-02	23.94	38.02	5.29E-01	210.05	156.18	1.79E-01	38.18	15.37	1.30E-02	-9.84	50.05	8.44E-01
cg13406605	-11.46	4.59	1.25E-02	-11.98	7.34	1.03E-01	-4.92	10.97	6.54E-01	-29.56	12.81	2.10E-02	-6.94	8.29	4.02E-01
cg03661817	7.85	3.14	1.25E-02	7.50	4.24	7.68E-02	17.50	6.71	9.15E-03	-1.98	8.79	8.22E-01	5.72	5.64	3.10E-01
cg05157961	21.00	8.41	1.25E-02	9.59	20.93	6.47E-01	37.54	19.21	5.07E-02	14.85	11.43	1.94E-01	39.91	25.87	1.23E-01
cg18372136	13.49	5.40	1.25E-02	14.93	12.74	2.41E-01	7.22	18.32	6.93E-01	15.29	6.56	1.97E-02	-3.68	23.06	8.73E-01
cg26014466	13.17	5.28	1.25E-02	25.23	16.70	1.31E-01	14.75	22.66	5.15E-01	10.07	5.94	8.99E-02	34.13	22.36	1.27E-01
cg02981663	16.29	6.52	1.25E-02	21.81	20.04	2.77E-01	-32.43	42.31	4.43E-01	16.09	7.22	2.58E-02	29.86	28.09	2.88E-01
cg13481969	8.12	3.25	1.25E-02	15.08	8.98	9.30E-02	13.91	15.85	3.80E-01	10.07	5.11	4.88E-02	3.50	5.01	4.85E-01
cg20849025	-11.07	4.44	1.25E-02	-10.79	10.67	3.12E-01	-22.79	21.03	2.79E-01	-11.01	5.40	4.17E-02	-7.16	13.43	5.94E-01
cg00065957	-33.57	13.45	1.25E-02	-28.21	17.41	1.05E-01	-33.65	36.87	3.61E-01	-39.78	40.89	3.31E-01	-49.04	33.38	1.42E-01
cg19358349	25.99	10.41	1.25E-02	27.27	12.80	3.31E-02	4.39	44.98	9.22E-01	78.26	71.06	2.71E-01	22.89	20.29	2.59E-01
cg03785696	25.45	10.20	1.26E-02	59.12	31.17	5.79E-02	-52.84	69.93	4.50E-01	26.21	11.70	2.51E-02	3.12	30.41	9.18E-01
cg07924575	10.69	4.28	1.26E-02	6.73	5.75	2.42E-01	21.96	11.26	5.13E-02	15.94	10.92	1.44E-01	9.07	11.18	4.17E-01
cg13743091	-10.11	4.05	1.26E-02	-11.44	10.95	2.96E-01	-7.76	16.67	6.41E-01	-10.03	4.80	3.65E-02	-10.26	13.41	4.44E-01
cg24831725	18.96	7.60	1.26E-02	31.33	25.09	2.12E-01	63.89	78.83	4.18E-01	16.31	8.32	5.00E-02	29.25	29.83	3.27E-01
cg02205739	43.10	17.26	1.26E-02	49.05	21.78	2.43E-02	8.64	58.69	8.83E-01	-12.06	69.50	8.62E-01	54.94	36.55	1.33E-01
cg13670601	14.86	5.95	1.26E-02	-0.74	22.31	9.73E-01	-1.71	66.14	9.79E-01	17.70	6.41	5.72E-03	-6.43	24.98	7.97E-01
cg13463639	14.99	6.00	1.26E-02	11.54	9.52	2.25E-01	22.05	11.62	5.77E-02	1.55	17.00	9.28E-01	20.51	13.09	1.17E-01
cg04391232	21.30	8.54	1.26E-02	33.49	14.90	2.46E-02	8.87	16.80	5.98E-01	28.89	16.21	7.46E-02	0.02	23.14	9.99E-01
cg27535648	-24.92	9.98	1.26E-02	-26.46	14.78	7.34E-02	-46.58	31.03	1.33E-01	-17.58	21.24	4.08E-01	-18.87	21.32	3.76E-01

cg03363633	14.40	5.77	1.26E-02	14.54	7.53	5.37E-02	2.04	13.14	8.76E-01	8.14	31.25	7.94E-01	27.86	13.35	3.69E-02
cg26247737	22.72	9.10	1.26E-02	24.21	12.51	5.31E-02	-17.43	29.23	5.51E-01	43.91	25.52	8.53E-02	24.38	18.34	1.84E-01
cg18158419	12.98	5.20	1.26E-02	13.15	7.63	8.49E-02	23.94	13.04	6.64E-02	16.54	11.18	1.39E-01	-3.26	13.02	8.02E-01
cg25186332	18.67	7.48	1.26E-02	-8.28	19.30	6.68E-01	23.74	20.83	2.55E-01	22.46	9.32	1.60E-02	31.18	27.10	2.50E-01
cg23497678	9.85	3.95	1.26E-02	10.21	7.51	1.74E-01	-2.30	17.83	8.98E-01	9.58	5.19	6.51E-02	16.69	12.73	1.90E-01
cg04100434	-26.05	10.44	1.26E-02	-48.11	34.67	1.65E-01	16.98	115.20	8.83E-01	-23.27	11.35	4.02E-02	-38.96	44.78	3.84E-01
cg10005475	9.90	3.97	1.26E-02	13.64	5.96	2.21E-02	9.28	7.62	2.23E-01	0.38	11.86	9.75E-01	7.45	9.51	4.33E-01
cg04871582	33.45	13.41	1.26E-02	34.55	18.82	6.64E-02	42.27	30.12	1.61E-01	6.10	53.40	9.09E-01	30.96	27.90	2.67E-01
cg19725343	26.25	10.52	1.26E-02	20.33	31.50	5.19E-01	-39.23	85.41	6.46E-01	28.75	11.71	1.41E-02	20.71	41.04	6.14E-01
cg19471856	-13.76	5.52	1.26E-02	-20.50	13.11	1.18E-01	-4.64	22.23	8.35E-01	-16.37	6.69	1.45E-02	15.45	19.23	4.22E-01
cg03341377	-8.80	3.53	1.26E-02	-7.21	4.75	1.29E-01	-18.17	8.29	2.83E-02	-5.56	12.23	6.49E-01	-5.79	8.27	4.83E-01
cg18599361	9.36	3.75	1.26E-02	7.47	7.20	3.00E-01	12.55	13.43	3.50E-01	10.78	5.27	4.07E-02	6.17	9.93	5.34E-01
cg27230711	29.78	11.94	1.26E-02	4.31	35.79	9.04E-01	-1.13	98.37	9.91E-01	37.99	13.30	4.29E-03	-19.14	45.79	6.76E-01
cg03564228	-15.72	6.30	1.26E-02	-3.97	14.71	7.87E-01	-58.44	27.19	3.16E-02	-14.55	6.64	2.85E-02	-20.99	19.04	2.70E-01
cg21212076	-6.76	2.71	1.26E-02	-8.15	4.24	5.46E-02	-3.91	5.98	5.13E-01	-3.76	5.63	5.04E-01	-11.31	6.88	1.00E-01
cg27301230	6.93	2.78	1.26E-02	9.41	4.32	2.93E-02	6.85	6.65	3.03E-01	6.01	4.97	2.27E-01	-0.43	8.88	9.62E-01
cg10711778	11.15	4.47	1.26E-02	13.31	6.84	5.16E-02	12.35	9.98	2.16E-01	11.81	15.91	4.58E-01	6.99	8.27	3.98E-01
cg26647617	19.73	7.91	1.26E-02	24.66	13.62	7.02E-02	3.30	24.77	8.94E-01	31.68	13.72	2.09E-02	2.32	16.57	8.88E-01
cg15100501	-21.57	8.65	1.27E-02	-38.18	23.90	1.10E-01	-16.63	36.26	6.46E-01	-18.68	9.96	6.06E-02	-26.57	36.19	4.63E-01
cg20862119	-18.29	7.33	1.27E-02	0.05	22.87	9.98E-01	-15.46	38.99	6.92E-01	-21.50	8.21	8.85E-03	-9.30	29.00	7.48E-01
cg10828561	-12.49	5.01	1.27E-02	-21.17	7.29	3.68E-03	-9.29	11.79	4.31E-01	3.57	14.32	8.03E-01	-6.88	9.90	4.87E-01
cg02478762	-21.73	8.71	1.27E-02	-15.21	16.80	3.65E-01	19.31	43.21	6.55E-01	-24.48	11.29	3.01E-02	-40.62	28.37	1.52E-01
cg01261351	13.27	5.32	1.27E-02	24.20	9.32	9.41E-03	5.15	12.82	6.88E-01	13.38	11.73	2.54E-01	5.86	9.79	5.50E-01
cg00390769	-35.85	14.38	1.27E-02	-82.22	54.24	1.30E-01	-319.63	192.40	9.67E-02	-30.21	14.05	3.15E-02	-35.73	51.29	4.86E-01
cg13244522	-13.53	5.43	1.27E-02	-12.05	12.92	3.51E-01	-8.46	29.57	7.75E-01	-14.82	6.46	2.17E-02	-7.76	18.88	6.81E-01
cg04600253	20.86	8.37	1.27E-02	34.55	21.69	1.11E-01	13.30	22.42	5.53E-01	17.17	10.55	1.04E-01	37.00	29.08	2.03E-01
cg00840257	28.74	11.53	1.27E-02	27.22	21.15	1.98E-01	-15.32	51.63	7.67E-01	36.36	16.76	3.01E-02	23.43	27.19	3.89E-01
cg17103638	14.54	5.84	1.27E-02	22.17	10.26	3.08E-02	32.71	20.87	1.17E-01	11.64	8.67	1.80E-01	-3.09	15.29	8.40E-01
cg23190164	8.42	3.38	1.27E-02	10.97	5.21	3.52E-02	-3.91	9.62	6.85E-01	10.85	5.64	5.44E-02	4.04	10.88	7.10E-01
cg00071012	11.15	4.47	1.27E-02	15.35	8.98	8.74E-02	-3.40	10.40	7.44E-01	11.09	6.28	7.76E-02	23.10	11.96	5.34E-02
cg03005087	-11.73	4.71	1.27E-02	-18.30	6.74	6.57E-03	5.03	12.35	6.84E-01	-4.37	18.04	8.09E-01	-10.81	8.62	2.10E-01
cg19096849	-12.11	4.86	1.27E-02	-9.51	9.48	3.16E-01	-10.31	13.15	4.33E-01	-12.54	7.24	8.32E-02	-17.02	12.54	1.75E-01
cg00527307	-13.71	5.50	1.27E-02	-2.63	13.22	8.42E-01	21.36	30.46	4.83E-01	-16.48	6.45	1.06E-02	-29.17	21.16	1.68E-01
cg03625260	15.63	6.27	1.27E-02	20.80	21.02	3.23E-01	5.06	24.27	8.35E-01	19.67	7.98	1.37E-02	5.71	13.18	6.65E-01
cg26527263	-9.96	4.00	1.27E-02	-10.65	5.75	6.42E-02	-6.64	9.32	4.76E-01	-9.90	11.48	3.89E-01	-11.34	8.70	1.92E-01

cg25208479	15.58	6.25	1.27E-02	15.51	24.32	5.24E-01	96.05	89.09	2.81E-01	14.17	6.71	3.47E-02	29.43	25.44	2.47E-01
cg21851282	11.18	4.49	1.27E-02	4.83	10.55	6.47E-01	-1.05	12.56	9.33E-01	15.45	5.85	8.24E-03	13.18	14.10	3.50E-01
cg19309009	17.09	6.86	1.27E-02	32.79	17.26	5.75E-02	9.72	25.61	7.04E-01	10.59	8.55	2.15E-01	34.73	19.28	7.16E-02
cg16936094	-8.63	3.46	1.27E-02	-15.31	8.60	7.52E-02	-24.12	17.74	1.74E-01	-7.24	4.16	8.17E-02	-1.96	10.60	8.53E-01
cg03267779	39.23	15.75	1.27E-02	51.29	42.20	2.24E-01	-48.98	136.90	7.20E-01	43.77	18.10	1.56E-02	-4.75	52.55	9.28E-01
cg07919131	-18.24	7.32	1.27E-02	-8.86	11.56	4.43E-01	-31.99	20.88	1.25E-01	-17.30	14.83	2.44E-01	-28.17	15.21	6.40E-02
cg26251865	-13.35	5.36	1.27E-02	-23.07	8.29	5.39E-03	-6.89	12.54	5.82E-01	0.88	12.60	9.44E-01	-11.96	11.48	2.98E-01
cg19008320	16.20	6.51	1.28E-02	20.47	10.68	5.52E-02	13.44	13.39	3.15E-01	7.16	14.04	6.10E-01	21.85	15.42	1.57E-01
cg09324669	11.89	4.77	1.28E-02	17.29	6.74	1.03E-02	12.22	16.17	4.50E-01	12.05	18.01	5.04E-01	3.83	8.17	6.39E-01
cg01977519	-12.35	4.96	1.28E-02	-14.69	6.71	2.86E-02	-11.56	12.62	3.60E-01	-15.87	10.82	1.42E-01	8.74	16.52	5.97E-01
cg23018092	16.90	6.78	1.28E-02	27.32	15.19	7.22E-02	29.55	20.79	1.55E-01	11.13	10.76	3.01E-01	13.07	12.45	2.94E-01
cg20209754	-45.96	18.45	1.28E-02	-47.41	28.06	9.10E-02	-19.75	57.40	7.31E-01	-68.51	45.69	1.34E-01	-40.64	33.64	2.27E-01
cg22692964	-27.27	10.95	1.28E-02	-27.03	14.92	7.01E-02	6.98	29.65	8.14E-01	-2.59	52.59	9.61E-01	-48.08	20.62	1.97E-02
cg12458913	17.10	6.87	1.28E-02	28.47	10.60	7.22E-03	11.60	17.10	4.97E-01	16.58	21.35	4.37E-01	4.95	12.23	6.86E-01
cg07186154	14.60	5.86	1.28E-02	19.33	12.84	1.32E-01	3.27	17.81	8.54E-01	15.39	8.40	6.70E-02	13.87	13.25	2.95E-01
cg21620968	20.69	8.31	1.28E-02	33.52	23.03	1.46E-01	1.85	37.91	9.61E-01	16.99	9.60	7.67E-02	48.47	30.90	1.17E-01
cg19198993	17.43	7.00	1.28E-02	29.90	13.78	3.00E-02	6.65	15.72	6.72E-01	16.70	10.68	1.18E-01	10.70	20.70	6.05E-01
cg18369866	21.27	8.54	1.28E-02	27.30	12.25	2.58E-02	-4.36	26.81	8.71E-01	50.52	32.73	1.23E-01	14.52	14.57	3.19E-01
cg03962214	-59.52	23.90	1.28E-02	-60.78	31.43	5.31E-02	-10.65	136.05	9.38E-01	-53.50	60.73	3.78E-01	-66.78	49.24	1.75E-01
cg12212206	25.80	10.36	1.28E-02	60.73	32.99	6.57E-02	-62.79	121.24	6.05E-01	19.65	11.31	8.23E-02	68.57	44.13	1.20E-01
cg06328831	11.56	4.64	1.28E-02	15.55	6.81	2.25E-02	11.43	9.60	2.34E-01	18.86	16.26	2.46E-01	0.59	9.90	9.53E-01
cg03508504	16.07	6.46	1.28E-02	23.35	9.61	1.52E-02	5.75	15.39	7.08E-01	-8.72	19.44	6.54E-01	20.91	12.59	9.68E-02
cg24387472	18.31	7.35	1.28E-02	41.41	24.26	8.79E-02	25.47	26.74	3.41E-01	15.79	8.62	6.71E-02	10.40	22.72	6.47E-01
cg12220663	-10.22	4.11	1.28E-02	-16.65	9.34	7.47E-02	-6.94	13.91	6.18E-01	-7.83	5.31	1.40E-01	-14.12	11.78	2.31E-01
cg03827772	21.66	8.70	1.28E-02	20.87	16.86	2.16E-01	13.09	18.59	4.81E-01	26.47	13.69	5.32E-02	22.98	26.15	3.80E-01
cg13577149	-12.53	5.03	1.28E-02	-18.76	10.09	6.29E-02	-0.87	17.20	9.59E-01	-11.27	6.80	9.72E-02	-13.71	14.73	3.52E-01
cg03560424	13.21	5.31	1.28E-02	6.54	7.75	3.99E-01	24.16	10.34	1.94E-02	24.19	17.15	1.58E-01	8.53	12.83	5.06E-01
cg27319536	6.81	2.74	1.28E-02	9.00	4.07	2.70E-02	-1.54	7.00	8.26E-01	9.86	5.97	9.84E-02	4.89	6.36	4.41E-01
cg04094067	24.21	9.73	1.28E-02	30.23	21.01	1.50E-01	21.80	20.52	2.88E-01	13.24	14.33	3.56E-01	67.12	30.70	2.88E-02
cg14545570	12.81	5.15	1.28E-02	4.58	10.43	6.61E-01	-1.82	15.30	9.05E-01	19.75	7.10	5.37E-03	12.88	15.02	3.91E-01
cg17199247	16.51	6.63	1.28E-02	33.15	20.82	1.11E-01	11.59	32.93	7.25E-01	13.63	7.66	7.54E-02	22.62	20.07	2.60E-01
cg07814707	10.52	4.23	1.28E-02	12.55	6.17	4.19E-02	11.90	10.08	2.38E-01	12.87	14.78	3.84E-01	5.44	8.09	5.02E-01
cg20333727	21.73	8.73	1.28E-02	14.27	17.00	4.01E-01	16.99	16.14	2.93E-01	17.50	16.20	2.80E-01	51.64	22.30	2.06E-02
cg03308839	5.50	2.21	1.28E-02	4.82	2.93	1.00E-01	10.11	5.55	6.88E-02	8.80	5.37	1.01E-01	-3.12	6.83	6.48E-01
cg20070090	7.33	2.94	1.28E-02	6.32	3.81	9.72E-02	14.02	6.82	3.98E-02	8.95	10.77	4.06E-01	1.94	7.78	8.03E-01

cg10634403	-8.97	3.60	1.28E-02	-17.49	8.23	3.36E-02	-18.66	15.34	2.24E-01	-6.30	4.57	1.68E-01	-5.11	9.96	6.08E-01
cg12317815	-6.56	2.64	1.28E-02	-10.20	4.28	1.71E-02	-1.74	5.52	7.52E-01	-9.22	5.54	9.59E-02	-1.17	6.52	8.57E-01
cg04011707	22.40	9.00	1.28E-02	22.22	16.63	1.82E-01	23.24	14.55	1.10E-01	9.51	20.27	6.39E-01	40.24	25.23	1.11E-01
cg06599586	-19.45	7.81	1.28E-02	-22.45	11.52	5.13E-02	-4.90	16.94	7.72E-01	-34.85	21.49	1.05E-01	-17.79	17.70	3.15E-01
cg19578305	-15.23	6.12	1.28E-02	-20.73	10.22	4.26E-02	-1.11	16.04	9.45E-01	-11.60	9.56	2.25E-01	-33.65	20.93	1.08E-01
cg03130533	36.99	14.87	1.28E-02	-8.09	44.83	8.57E-01	-62.72	192.63	7.45E-01	38.98	16.43	1.76E-02	97.32	58.30	9.51E-02
cg23547017	26.97	10.84	1.28E-02	49.64	28.83	8.51E-02	26.72	42.03	5.25E-01	24.13	14.33	9.21E-02	19.86	23.11	3.90E-01
cg11569431	20.86	8.38	1.28E-02	34.08	16.60	4.01E-02	9.45	15.21	5.35E-01	9.49	15.40	5.38E-01	44.72	22.01	4.22E-02
cg24116317	12.50	5.02	1.28E-02	6.37	11.15	5.68E-01	36.30	21.75	9.51E-02	13.42	6.25	3.18E-02	6.16	16.04	7.01E-01
cg21190228	6.38	2.56	1.28E-02	5.28	3.26	1.05E-01	13.94	6.70	3.76E-02	4.66	8.57	5.87E-01	4.50	6.76	5.05E-01
cg08145373	-8.20	3.30	1.28E-02	-8.97	4.34	3.89E-02	-14.00	8.31	9.22E-02	2.86	9.13	7.54E-01	-8.87	8.94	3.21E-01
cg18026197	5.43	2.18	1.28E-02	3.89	3.11	2.10E-01	1.77	4.55	6.98E-01	12.06	7.67	1.16E-01	10.88	4.94	2.75E-02
cg11285834	10.96	4.41	1.28E-02	10.27	5.93	8.31E-02	10.87	9.65	2.60E-01	22.78	16.85	1.77E-01	8.57	10.66	4.22E-01
cg04099652	23.93	9.62	1.29E-02	47.39	21.42	2.69E-02	-20.93	46.70	6.54E-01	24.06	12.13	4.73E-02	0.99	27.02	9.71E-01
cg13782932	-22.94	9.22	1.29E-02	-24.67	11.36	2.99E-02	-23.96	31.75	4.50E-01	19.50	59.87	7.45E-01	-21.99	19.09	2.49E-01
cg18283361	-17.84	7.17	1.29E-02	-24.28	12.26	4.77E-02	-5.26	15.16	7.28E-01	-14.34	16.74	3.92E-01	-22.86	14.33	1.11E-01
cg15806588	-11.48	4.62	1.29E-02	-21.82	7.90	5.73E-03	-1.75	11.69	8.81E-01	-7.22	8.46	3.93E-01	-7.85	10.21	4.42E-01
cg12832355	17.28	6.94	1.29E-02	30.10	17.93	9.32E-02	-15.22	69.85	8.28E-01	15.98	7.82	4.09E-02	5.77	30.88	8.52E-01
cg05477024	21.12	8.49	1.29E-02	24.41	12.09	4.36E-02	8.06	20.58	6.95E-01	22.52	27.83	4.18E-01	23.05	17.19	1.80E-01
cg01431993	13.78	5.54	1.29E-02	18.21	16.94	2.83E-01	-15.09	45.09	7.38E-01	16.07	6.16	9.07E-03	-13.62	21.08	5.18E-01
cg01943813	-18.77	7.54	1.29E-02	-16.97	9.29	6.78E-02	-21.14	18.81	2.61E-01	-40.86	37.21	2.72E-01	-18.00	20.26	3.74E-01
cg22275125	-11.17	4.49	1.29E-02	-14.04	6.87	4.10E-02	-4.24	9.56	6.57E-01	-26.21	12.87	4.17E-02	-4.53	9.37	6.29E-01
cg24748448	-18.96	7.62	1.29E-02	-17.69	11.43	1.22E-01	-26.60	18.44	1.49E-01	-13.42	16.82	4.25E-01	-21.18	18.03	2.40E-01
cg04111344	22.44	9.02	1.29E-02	21.34	17.37	2.19E-01	83.36	38.65	3.10E-02	15.06	11.36	1.85E-01	33.92	27.09	2.11E-01
cg01381170	18.30	7.36	1.29E-02	18.55	19.47	3.41E-01	21.24	30.31	4.83E-01	15.24	8.54	7.44E-02	55.65	31.27	7.52E-02
cg01032570	-33.26	13.37	1.29E-02	-35.02	16.19	3.05E-02	-71.02	70.48	3.14E-01	-16.94	69.66	8.08E-01	-25.23	27.05	3.51E-01
cg07867367	7.37	2.96	1.29E-02	1.40	5.77	8.08E-01	14.74	7.54	5.07E-02	9.02	4.29	3.54E-02	3.99	9.19	6.65E-01
cg01715248	7.68	3.09	1.29E-02	6.82	4.15	9.99E-02	4.15	8.15	6.11E-01	16.79	8.60	5.08E-02	6.56	7.43	3.78E-01
cg19215677	26.98	10.85	1.29E-02	45.52	32.68	1.64E-01	37.27	155.69	8.11E-01	24.08	11.86	4.23E-02	34.04	49.69	4.93E-01
cg07536847	17.62	7.09	1.29E-02	21.75	14.51	1.34E-01	-21.54	30.57	4.81E-01	21.12	9.80	3.12E-02	13.80	16.47	4.02E-01
cg02771673	-11.05	4.45	1.29E-02	-13.94	7.31	5.64E-02	-10.60	11.04	3.37E-01	-1.39	8.37	8.68E-01	-20.34	10.31	4.84E-02
cg23371476	9.21	3.70	1.29E-02	6.50	6.32	3.04E-01	15.28	7.81	5.03E-02	5.07	6.46	4.33E-01	18.21	11.55	1.15E-01
cg09712135	17.87	7.19	1.29E-02	25.39	9.33	6.50E-03	45.66	36.95	2.16E-01	-10.81	32.06	7.36E-01	5.30	12.64	6.75E-01
cg18084215	23.73	9.54	1.29E-02	32.50	21.28	1.27E-01	-24.55	49.08	6.17E-01	17.87	11.74	1.28E-01	61.34	29.58	3.81E-02
cg00828556	6.03	2.42	1.29E-02	5.13	3.27	1.16E-01	10.56	4.78	2.71E-02	-2.06	8.61	8.11E-01	5.71	7.19	4.27E-01

cg20592707	-9.26	3.72	1.29E-02	-15.92	7.07	2.43E-02	-4.82	10.81	6.56E-01	-4.19	5.44	4.41E-01	-17.00	10.12	9.29E-02
cg14708940	-10.55	4.24	1.29E-02	-13.75	6.50	3.46E-02	-7.28	8.33	3.82E-01	-1.01	12.36	9.35E-01	-13.66	9.57	1.53E-01
cg15687973	10.96	4.41	1.29E-02	9.13	6.54	1.63E-01	12.83	11.52	2.65E-01	6.10	9.58	5.24E-01	19.43	10.19	5.64E-02
cg10806146	8.71	3.50	1.29E-02	7.37	5.11	1.49E-01	9.08	6.78	1.80E-01	17.64	10.88	1.05E-01	6.20	8.77	4.80E-01
cg23749482	-6.70	2.69	1.29E-02	-7.29	3.75	5.21E-02	-11.87	6.56	7.04E-02	-8.73	9.48	3.57E-01	-0.98	5.56	8.60E-01
cg11405655	-11.96	4.81	1.29E-02	-4.14	7.48	5.80E-01	-12.39	12.89	3.37E-01	-21.20	9.55	2.65E-02	-16.28	10.96	1.38E-01
cg07017869	19.03	7.66	1.29E-02	11.74	20.33	5.64E-01	52.12	40.15	1.94E-01	21.38	8.84	1.56E-02	-7.76	28.49	7.85E-01
cg22324022	29.36	11.82	1.29E-02	41.35	17.42	1.76E-02	40.57	39.60	3.06E-01	36.59	30.17	2.25E-01	3.79	21.65	8.61E-01
cg02672332	-18.18	7.32	1.29E-02	-33.89	9.71	4.83E-04	1.95	15.50	9.00E-01	-16.81	12.05	1.63E-01	-13.04	11.90	2.73E-01
cg22747516	-10.28	4.14	1.30E-02	-11.17	5.00	2.56E-02	-22.57	8.26	6.27E-03	-5.94	7.53	4.30E-01	0.76	9.31	9.35E-01
cg15146906	16.66	6.70	1.30E-02	25.88	14.65	7.74E-02	67.91	48.11	1.58E-01	14.62	8.36	8.03E-02	4.08	18.70	8.27E-01
cg10832005	29.03	11.68	1.30E-02	32.41	16.80	5.38E-02	23.51	31.63	4.57E-01	35.06	26.14	1.80E-01	17.47	27.50	5.25E-01
cg13582960	36.40	14.65	1.30E-02	-10.26	41.37	8.04E-01	-117.72	167.40	4.82E-01	47.67	16.40	3.64E-03	7.72	55.87	8.90E-01
cg25932290	7.74	3.12	1.30E-02	7.60	4.01	5.84E-02	16.66	7.68	3.01E-02	3.19	10.50	7.61E-01	0.98	8.20	9.05E-01
cg18655025	-13.96	5.62	1.30E-02	-18.78	9.87	5.72E-02	-11.96	12.65	3.44E-01	-8.77	11.76	4.56E-01	-14.04	11.23	2.11E-01
cg04681472	16.85	6.78	1.30E-02	-5.73	18.66	7.59E-01	6.35	41.65	8.79E-01	18.66	7.71	1.55E-02	44.05	25.94	8.95E-02
cg02365862	25.35	10.20	1.30E-02	33.77	32.07	2.92E-01	19.27	90.20	8.31E-01	19.45	11.36	8.68E-02	75.93	36.34	3.66E-02
cg15615022	12.39	4.98	1.30E-02	17.18	7.83	2.82E-02	4.42	8.81	6.16E-01	6.61	16.22	6.84E-01	18.81	11.75	1.10E-01
cg01953069	17.40	7.00	1.30E-02	22.99	16.35	1.60E-01	-7.52	18.60	6.86E-01	21.70	8.95	1.53E-02	15.36	28.15	5.85E-01
cg13283199	-5.18	2.09	1.30E-02	-4.24	2.98	1.55E-01	-9.17	4.48	4.05E-02	1.51	9.87	8.78E-01	-4.76	4.18	2.55E-01
cg03804083	-11.92	4.80	1.30E-02	-10.97	11.22	3.28E-01	0.53	14.78	9.71E-01	-13.16	6.22	3.44E-02	-18.26	14.00	1.92E-01
cg15474755	-12.62	5.08	1.30E-02	-12.51	11.60	2.81E-01	-10.48	22.75	6.45E-01	-14.48	6.36	2.28E-02	-3.82	14.64	7.94E-01
cg02333517	13.80	5.55	1.30E-02	17.38	9.13	5.70E-02	9.08	11.43	4.27E-01	19.51	15.32	2.03E-01	10.13	10.84	3.50E-01
cg05279387	-15.55	6.26	1.30E-02	-9.56	11.05	3.87E-01	-3.49	12.80	7.85E-01	-25.57	12.65	4.32E-02	-27.57	14.16	5.15E-02
cg23279559	33.79	13.60	1.30E-02	35.21	15.76	2.54E-02	-43.70	56.26	4.37E-01	48.82	49.75	3.26E-01	52.98	38.93	1.74E-01
cg14603539	-15.51	6.24	1.30E-02	-20.86	18.01	2.47E-01	13.94	26.59	6.00E-01	-15.80	7.17	2.76E-02	-27.07	24.27	2.65E-01
cg15509286	-10.01	4.03	1.30E-02	-19.80	8.13	1.49E-02	-6.00	9.82	5.41E-01	-10.72	6.45	9.62E-02	0.30	9.14	9.74E-01
cg21380024	-8.62	3.47	1.30E-02	-7.61	4.62	9.93E-02	-9.05	8.57	2.91E-01	-6.16	10.90	5.72E-01	-12.95	8.38	1.22E-01
cg15112006	18.52	7.45	1.30E-02	23.87	11.68	4.10E-02	6.64	14.49	6.47E-01	22.91	16.26	1.59E-01	18.83	21.70	3.86E-01
cg00933859	-10.76	4.33	1.30E-02	-25.40	10.15	1.23E-02	-18.37	15.91	2.48E-01	-6.35	4.99	2.04E-01	-8.23	13.32	5.36E-01
cg03122532	-9.95	4.00	1.30E-02	-12.47	6.89	7.05E-02	-6.59	9.01	4.64E-01	-9.54	7.46	2.01E-01	-9.54	9.52	3.16E-01
cg21620139	6.61	2.66	1.30E-02	7.63	3.67	3.76E-02	1.50	6.81	8.25E-01	17.71	8.46	3.62E-02	2.77	5.65	6.24E-01
cg25355291	25.49	10.26	1.30E-02	42.56	19.58	2.98E-02	5.88	23.90	8.06E-01	26.17	15.86	9.88E-02	14.39	29.34	6.24E-01
cg03897452	7.68	3.09	1.30E-02	6.95	4.45	1.18E-01	1.12	8.00	8.89E-01	16.83	9.31	7.06E-02	8.95	6.11	1.43E-01
cg25424279	12.95	5.21	1.30E-02	13.14	7.67	8.67E-02	13.66	10.43	1.90E-01	9.21	14.90	5.36E-01	14.10	12.79	2.70E-01

cg25390413	11.28	4.54	1.30E-02	16.02	6.78	1.81E-02	14.56	9.62	1.30E-01	-2.85	14.61	8.45E-01	4.84	9.43	6.07E-01
cg06271720	-9.67	3.89	1.30E-02	-6.68	6.84	3.29E-01	-8.46	14.13	5.49E-01	-8.93	5.76	1.21E-01	-19.36	10.25	5.90E-02
cg27541604	-9.97	4.01	1.30E-02	-12.86	6.29	4.08E-02	-7.11	9.57	4.57E-01	-6.81	8.79	4.38E-01	-9.88	8.79	2.61E-01
cg00386101	12.95	5.21	1.30E-02	8.69	8.21	2.90E-01	21.68	9.35	2.04E-02	11.16	14.11	4.29E-01	7.92	13.49	5.57E-01
cg00007324	-63.09	25.40	1.30E-02	-47.38	29.87	1.13E-01	-152.62	166.41	3.59E-01	-159.98	82.18	5.16E-02	-63.21	63.88	3.22E-01
cg01572333	-14.48	5.83	1.30E-02	-23.89	9.27	9.98E-03	5.95	15.44	7.00E-01	-13.54	11.87	2.54E-01	-11.85	12.41	3.40E-01
cg23400056	22.44	9.04	1.30E-02	9.80	20.09	6.26E-01	65.17	50.93	2.01E-01	24.66	10.88	2.34E-02	18.33	32.71	5.75E-01
cg19274698	48.41	19.49	1.30E-02	54.39	24.64	2.73E-02	30.90	128.21	8.10E-01	-5.73	92.14	9.50E-01	45.42	35.21	1.97E-01
cg21538208	7.29	2.93	1.30E-02	6.82	5.05	1.77E-01	3.11	6.58	6.36E-01	8.87	6.12	1.47E-01	9.96	6.07	1.01E-01
cg22210627	13.19	5.31	1.30E-02	18.90	8.01	1.83E-02	12.17	14.78	4.10E-01	16.77	22.53	4.57E-01	6.33	8.66	4.65E-01
cg02248749	-10.83	4.36	1.30E-02	-22.06	11.59	5.69E-02	-26.78	17.81	1.33E-01	-8.25	5.14	1.09E-01	-2.07	15.52	8.94E-01
cg01128642	25.15	10.13	1.30E-02	63.08	38.12	9.79E-02	63.50	92.30	4.91E-01	20.28	11.15	6.89E-02	34.65	33.35	2.99E-01
cg26407316	-12.60	5.07	1.30E-02	-9.00	8.62	2.96E-01	6.10	18.72	7.44E-01	-18.39	7.49	1.41E-02	-12.30	14.63	4.01E-01
cg03196720	8.07	3.25	1.30E-02	8.62	4.86	7.58E-02	11.75	7.50	1.17E-01	10.30	10.25	3.15E-01	3.66	6.33	5.63E-01
cg09566995	7.22	2.91	1.30E-02	5.23	4.04	1.96E-01	13.91	6.85	4.23E-02	11.89	8.45	1.59E-01	3.27	6.75	6.28E-01
cg03847279	15.09	6.08	1.30E-02	20.84	11.95	8.11E-02	-2.79	19.62	8.87E-01	17.73	10.08	7.86E-02	12.49	11.45	2.75E-01
cg15517098	22.15	8.92	1.30E-02	17.45	16.81	2.99E-01	24.60	17.26	1.54E-01	15.99	15.35	2.98E-01	46.50	26.53	7.96E-02
cg06585141	-24.83	10.00	1.30E-02	-39.54	15.40	1.02E-02	-11.93	23.68	6.14E-01	-15.76	22.11	4.76E-01	-14.32	22.66	5.27E-01
cg02299465	20.40	8.22	1.30E-02	31.92	13.13	1.51E-02	23.81	15.42	1.23E-01	8.48	17.47	6.27E-01	-7.16	25.57	7.79E-01
cg05377041	37.93	15.28	1.30E-02	24.81	17.02	1.45E-01	56.01	25.45	2.78E-02	-0.86	28.07	9.76E-01	75.12	26.00	3.86E-03
cg26123532	-10.75	4.33	1.30E-02	-12.64	10.89	2.46E-01	-34.22	23.60	1.47E-01	-9.09	5.05	7.18E-02	-12.49	16.00	4.35E-01
cg16793781	27.44	11.05	1.30E-02	21.76	16.64	1.91E-01	23.62	44.70	5.97E-01	49.57	18.92	8.79E-03	-3.32	27.93	9.05E-01
cg13972124	24.87	10.02	1.31E-02	43.23	15.03	4.02E-03	27.50	31.70	3.86E-01	15.01	31.23	6.31E-01	5.19	16.38	7.51E-01
cg12809655	-16.67	6.72	1.31E-02	-18.08	13.10	1.68E-01	-15.41	18.29	4.00E-01	-10.12	5.84	8.32E-02	-48.53	18.96	1.05E-02
cg19583655	10.82	4.36	1.31E-02	12.94	6.64	5.14E-02	7.80	9.58	4.16E-01	8.30	12.56	5.09E-01	10.87	8.85	2.19E-01
cg08521859	22.62	9.11	1.31E-02	33.28	18.15	6.68E-02	7.04	16.03	6.60E-01	19.54	17.00	2.50E-01	46.28	24.63	6.02E-02
cg20063640	-14.05	5.66	1.31E-02	-4.05	7.84	6.06E-01	-25.52	11.23	2.31E-02	-8.05	7.96	3.12E-01	-26.23	10.15	9.74E-03
cg26888836	43.99	17.72	1.31E-02	56.57	65.43	3.87E-01	-319.47	264.86	2.28E-01	45.04	19.02	1.79E-02	40.03	76.32	6.00E-01
cg18973735	-12.10	4.87	1.31E-02	0.71	15.03	9.62E-01	-38.33	23.28	9.97E-02	-10.26	5.61	6.75E-02	-25.95	14.53	7.41E-02
cg20627646	-15.07	6.07	1.31E-02	-7.58	18.56	6.83E-01	-6.64	28.23	8.14E-01	-17.36	6.87	1.15E-02	-5.91	23.90	8.05E-01
cg16251357	18.56	7.48	1.31E-02	14.88	11.33	1.89E-01	23.14	19.02	2.24E-01	37.64	30.46	2.17E-01	17.82	12.65	1.59E-01
cg20525486	-15.83	6.38	1.31E-02	-15.74	8.73	7.15E-02	-10.52	15.77	5.05E-01	12.34	24.54	6.15E-01	-27.87	13.17	3.43E-02
cg14427527	20.94	8.44	1.31E-02	9.47	14.34	5.09E-01	31.49	15.91	4.78E-02	20.91	18.34	2.54E-01	27.23	21.05	1.96E-01
cg22162821	18.57	7.48	1.31E-02	16.88	17.21	3.27E-01	28.68	29.10	3.24E-01	12.66	9.33	1.75E-01	52.49	23.47	2.53E-02
cg07679219	3.72	1.50	1.31E-02	3.89	1.93	4.42E-02	4.27	3.43	2.13E-01	1.61	6.11	7.92E-01	3.19	3.92	4.16E-01

cg10298815	-10.79	4.35	1.31E-02	-8.15	5.64	1.48E-01	-18.10	9.51	5.69E-02	9.32	14.96	5.33E-01	-17.74	8.91	4.64E-02
cg13694867	20.99	8.46	1.31E-02	27.87	18.37	1.29E-01	-16.79	40.60	6.79E-01	25.31	10.90	2.02E-02	3.98	22.40	8.59E-01
cg03390187	-22.49	9.06	1.31E-02	-11.51	21.13	5.86E-01	-48.03	26.44	6.92E-02	-21.17	11.93	7.58E-02	-20.64	26.08	4.29E-01
cg06540578	13.45	5.42	1.31E-02	4.49	7.03	5.23E-01	14.40	8.23	8.00E-02	7.76	8.37	3.54E-01	29.15	8.48	5.82E-04
cg18249414	20.08	8.09	1.31E-02	-8.16	23.52	7.29E-01	14.39	29.32	6.24E-01	23.16	9.56	1.54E-02	37.80	27.23	1.65E-01
cg07043025	-23.19	9.35	1.31E-02	-8.46	21.89	6.99E-01	-73.19	46.13	1.13E-01	-20.90	11.56	7.07E-02	-40.51	26.64	1.28E-01
cg05473387	17.70	7.14	1.31E-02	15.03	11.78	2.02E-01	15.01	25.79	5.61E-01	22.28	10.50	3.39E-02	7.95	23.15	7.31E-01
cg15397374	16.36	6.60	1.31E-02	20.39	10.95	6.25E-02	-2.33	18.62	9.00E-01	35.76	15.77	2.34E-02	9.40	10.98	3.92E-01
cg07344172	19.32	7.79	1.31E-02	5.12	19.40	7.92E-01	59.98	29.00	3.86E-02	20.86	8.63	1.56E-02	-0.72	26.19	9.78E-01
cg02700472	-17.57	7.08	1.31E-02	-24.26	11.87	4.10E-02	-8.03	36.60	8.26E-01	-13.96	12.47	2.63E-01	-14.53	13.28	2.74E-01
cg14414316	-12.43	5.01	1.31E-02	-12.69	8.76	1.48E-01	-9.90	9.94	3.19E-01	-0.80	16.01	9.60E-01	-17.69	8.83	4.52E-02
cg24705960	15.51	6.25	1.31E-02	33.48	13.14	1.08E-02	7.96	21.86	7.16E-01	8.14	9.05	3.68E-01	15.81	13.51	2.42E-01
cg12372461	-8.11	3.27	1.31E-02	-12.43	4.41	4.83E-03	-1.43	8.70	8.70E-01	-8.93	10.98	4.16E-01	-1.29	6.97	8.53E-01
cg10532048	44.66	18.01	1.31E-02	92.23	49.74	6.37E-02	-151.38	204.98	4.60E-01	37.11	20.37	6.84E-02	59.45	63.76	3.51E-01
cg19370166	17.43	7.03	1.31E-02	45.68	29.07	1.16E-01	75.60	103.67	4.66E-01	17.00	7.45	2.24E-02	-15.59	32.59	6.33E-01
cg07904926	25.04	10.10	1.31E-02	31.16	32.35	3.35E-01	-81.85	130.23	5.30E-01	22.72	10.97	3.83E-02	65.94	45.47	1.47E-01
cg07521668	-34.79	14.03	1.31E-02	-30.48	21.18	1.50E-01	-8.32	53.22	8.76E-01	-55.28	27.22	4.23E-02	-27.25	29.48	3.55E-01
cg15965815	-14.23	5.74	1.31E-02	-22.93	17.77	1.97E-01	-41.84	38.40	2.76E-01	-13.23	6.30	3.57E-02	1.34	27.07	9.61E-01
cg24618910	27.35	11.03	1.31E-02	66.59	37.20	7.35E-02	-113.95	163.64	4.86E-01	26.25	12.00	2.87E-02	-2.42	43.94	9.56E-01
cg14945696	25.06	10.10	1.31E-02	34.61	21.36	1.05E-01	-10.27	48.74	8.33E-01	18.70	12.94	1.48E-01	51.41	28.75	7.37E-02
cg15249629	39.11	15.77	1.31E-02	-9.63	46.24	8.35E-01	-0.26	62.70	9.97E-01	49.20	18.39	7.45E-03	47.81	54.14	3.77E-01
cg19276540	-41.07	16.56	1.31E-02	-34.69	25.00	1.65E-01	-22.29	49.52	6.53E-01	-74.05	30.96	1.67E-02	-13.23	41.01	7.47E-01
cg25953310	23.24	9.37	1.31E-02	31.07	15.31	4.24E-02	28.14	18.05	1.19E-01	3.75	20.44	8.55E-01	22.18	24.59	3.67E-01
cg15736553	22.46	9.06	1.31E-02	21.11	13.70	1.23E-01	28.42	18.13	1.17E-01	37.97	25.39	1.35E-01	7.03	20.99	7.38E-01
cg19600538	-10.79	4.35	1.32E-02	-16.51	10.19	1.05E-01	-13.55	16.87	4.22E-01	-9.49	5.37	7.69E-02	-6.80	14.17	6.31E-01
cg04779720	24.28	9.79	1.32E-02	45.93	32.71	1.60E-01	-13.69	108.73	9.00E-01	24.94	10.95	2.28E-02	3.28	30.52	9.14E-01
cg18090064	17.88	7.21	1.32E-02	21.34	10.89	5.00E-02	25.68	35.91	4.75E-01	8.73	11.32	4.41E-01	34.16	21.23	1.08E-01
cg04890237	22.85	9.21	1.32E-02	11.57	16.09	4.72E-01	40.73	17.73	2.16E-02	22.80	18.89	2.27E-01	16.00	22.74	4.82E-01
cg05666828	-20.96	8.45	1.32E-02	-13.32	24.01	5.79E-01	-112.82	144.52	4.35E-01	-17.43	9.52	6.71E-02	-61.48	29.13	3.48E-02
cg10547761	-12.35	4.98	1.32E-02	-15.54	9.72	1.10E-01	-15.63	15.48	3.13E-01	-5.59	5.90	3.44E-01	-30.11	12.80	1.87E-02
cg03880814	19.35	7.81	1.32E-02	33.12	15.88	3.71E-02	17.16	24.02	4.75E-01	8.63	10.59	4.15E-01	44.37	23.61	6.02E-02
cg24952224	17.28	6.97	1.32E-02	15.66	8.61	6.90E-02	20.93	20.68	3.12E-01	33.75	60.45	5.77E-01	19.23	14.92	1.97E-01
cg24683623	11.38	4.59	1.32E-02	15.93	9.49	9.35E-02	5.48	9.49	5.64E-01	9.93	7.17	1.66E-01	18.91	13.16	1.51E-01
cg24996491	-9.96	4.02	1.32E-02	-10.96	8.34	1.89E-01	-12.20	14.19	3.90E-01	-10.15	5.60	6.97E-02	-7.01	9.69	4.70E-01
cg00627241	17.52	7.07	1.32E-02	44.18	25.86	8.75E-02	-61.16	97.87	5.32E-01	15.04	7.60	4.79E-02	27.48	29.83	3.57E-01

cg10858746	27.29	11.01	1.32E-02	22.92	24.51	3.50E-01	-33.61	61.67	5.86E-01	26.89	14.33	6.05E-02	44.65	26.24	8.88E-02
cg16046227	23.01	9.28	1.32E-02	34.31	17.26	4.68E-02	9.37	18.46	6.12E-01	18.08	16.74	2.80E-01	34.35	23.97	1.52E-01
cg27166718	19.00	7.67	1.32E-02	20.18	9.58	3.51E-02	-4.07	27.23	8.81E-01	59.72	36.12	9.83E-02	15.76	15.83	3.19E-01
cg07415373	9.55	3.85	1.32E-02	15.11	5.34	4.69E-03	3.11	8.04	6.99E-01	-2.89	12.51	8.17E-01	8.05	9.77	4.10E-01
cg12195820	-28.95	11.68	1.32E-02	-22.49	21.29	2.91E-01	14.49	46.40	7.55E-01	-37.43	17.79	3.54E-02	-34.06	25.83	1.87E-01
cg03358506	20.90	8.43	1.32E-02	37.62	22.39	9.29E-02	31.08	37.40	4.06E-01	16.11	9.80	1.00E-01	30.56	32.47	3.47E-01
cg14637649	-9.32	3.76	1.32E-02	-10.50	5.44	5.35E-02	-9.94	9.09	2.74E-01	-8.61	10.31	4.04E-01	-6.67	8.07	4.09E-01
cg22718169	-29.11	11.75	1.32E-02	-43.50	19.17	2.33E-02	-10.95	46.28	8.13E-01	-21.03	23.75	3.76E-01	-21.96	20.92	2.94E-01
cg18421360	22.60	9.12	1.32E-02	20.20	22.81	3.76E-01	-19.76	55.42	7.21E-01	25.52	10.79	1.80E-02	16.96	29.08	5.60E-01
cg07624008	-16.12	6.51	1.32E-02	-10.92	12.72	3.91E-01	-11.59	14.66	4.29E-01	-19.56	10.42	6.06E-02	-22.21	16.73	1.84E-01
cg25910261	6.08	2.45	1.32E-02	4.42	3.52	2.09E-01	9.21	6.16	1.35E-01	11.73	6.60	7.54E-02	3.90	5.28	4.60E-01
cg19177465	54.65	22.06	1.32E-02	57.10	27.04	3.47E-02	-76.21	106.81	4.76E-01	43.97	83.95	6.00E-01	75.70	46.73	1.05E-01
cg23711554	12.14	4.90	1.32E-02	16.98	9.02	5.97E-02	9.75	17.75	5.83E-01	10.56	6.83	1.22E-01	8.32	14.55	5.68E-01
cg01765249	26.37	10.64	1.32E-02	17.92	36.56	6.24E-01	195.25	190.09	3.04E-01	27.47	11.61	1.80E-02	16.05	39.82	6.87E-01
cg15395971	-10.82	4.37	1.32E-02	-16.79	10.13	9.75E-02	-29.72	20.02	1.38E-01	-6.75	5.34	2.06E-01	-18.18	14.02	1.95E-01
cg18588504	-29.71	11.99	1.32E-02	-33.59	47.42	4.79E-01	-92.95	237.74	6.96E-01	-29.13	12.71	2.19E-02	-32.18	57.67	5.77E-01
cg18778433	15.03	6.07	1.32E-02	22.33	14.59	1.26E-01	-0.15	19.58	9.94E-01	19.18	9.34	4.00E-02	9.99	10.92	3.60E-01
cg04724406	-16.58	6.69	1.32E-02	-11.72	9.92	2.38E-01	-1.67	17.03	9.22E-01	-22.22	15.60	1.54E-01	-33.42	14.73	2.33E-02
cg02316156	16.71	6.74	1.32E-02	18.20	10.65	8.74E-02	9.61	13.23	4.67E-01	17.75	18.27	3.31E-01	22.13	14.97	1.39E-01
cg06071395	24.24	9.78	1.32E-02	35.40	24.42	1.47E-01	78.72	108.93	4.70E-01	23.45	11.46	4.08E-02	8.15	30.50	7.89E-01
cg07368443	15.15	6.11	1.32E-02	16.38	9.10	7.19E-02	16.96	15.20	2.65E-01	15.04	13.94	2.80E-01	10.88	13.88	4.33E-01
cg09213964	17.55	7.08	1.33E-02	18.77	9.76	5.46E-02	26.78	19.32	1.66E-01	-4.44	28.56	8.77E-01	15.63	13.44	2.45E-01
cg03707183	8.12	3.28	1.33E-02	4.79	5.11	3.48E-01	15.64	8.00	5.07E-02	13.26	7.20	6.55E-02	3.63	7.09	6.09E-01
cg11863609	-27.80	11.22	1.33E-02	-4.84	25.17	8.47E-01	-53.46	52.14	3.05E-01	-34.61	14.02	1.36E-02	-19.16	33.21	5.64E-01
cg04561791	-11.29	4.56	1.33E-02	-12.08	6.20	5.15E-02	-14.31	10.99	1.93E-01	-15.18	13.00	2.43E-01	-2.66	11.23	8.12E-01
cg21068339	35.41	14.30	1.33E-02	35.39	17.68	4.54E-02	13.06	56.23	8.16E-01	61.50	47.96	2.00E-01	30.94	32.55	3.42E-01
cg05625559	12.07	4.87	1.33E-02	14.64	10.11	1.48E-01	32.83	22.68	1.48E-01	10.00	6.72	1.37E-01	9.67	11.03	3.81E-01
cg22645209	21.08	8.51	1.33E-02	40.43	14.67	5.86E-03	21.29	26.79	4.27E-01	5.55	10.75	6.05E-01	25.60	14.62	8.00E-02
cg13265740	9.02	3.64	1.33E-02	10.02	5.11	4.97E-02	10.54	11.58	3.63E-01	5.74	13.22	6.64E-01	7.72	6.48	2.33E-01
cg00223900	-28.45	11.49	1.33E-02	-20.02	33.32	5.48E-01	-71.73	106.76	5.02E-01	-24.49	12.79	5.55E-02	-88.08	46.11	5.61E-02
cg27264484	-43.23	17.46	1.33E-02	-48.50	25.77	5.98E-02	-73.04	50.48	1.48E-01	-26.81	39.34	4.96E-01	-30.96	36.85	4.01E-01
cg05757530	-14.05	5.67	1.33E-02	-18.50	8.83	3.62E-02	-13.87	13.28	2.96E-01	-14.82	14.69	3.13E-01	-6.55	11.21	5.59E-01
cg16172043	24.06	9.72	1.33E-02	18.88	19.13	3.24E-01	2.29	20.58	9.11E-01	30.22	15.03	4.43E-02	59.75	30.54	5.04E-02
cg27284840	-46.82	18.91	1.33E-02	-36.51	34.59	2.91E-01	-300.80	214.66	1.61E-01	-41.69	25.21	9.82E-02	-77.51	52.35	1.39E-01
cg09696747	-10.68	4.31	1.33E-02	-8.76	9.93	3.78E-01	1.60	19.62	9.35E-01	-14.41	5.36	7.15E-03	2.05	12.73	8.72E-01

cg26787787	-9.10	3.68	1.33E-02	-8.46	6.29	1.78E-01	-7.21	10.62	4.97E-01	-11.91	5.99	4.67E-02	-5.31	9.13	5.61E-01
cg13557752	-16.18	6.53	1.33E-02	-28.76	9.54	2.58E-03	-5.85	12.82	6.48E-01	0.43	18.08	9.81E-01	-13.80	13.06	2.91E-01
cg08998657	-9.70	3.92	1.33E-02	-19.87	9.72	4.09E-02	0.02	19.93	9.99E-01	-6.92	4.68	1.39E-01	-16.54	12.51	1.86E-01
cg08926365	-20.03	8.09	1.33E-02	-8.06	18.88	6.69E-01	9.63	48.89	8.44E-01	-25.89	9.67	7.45E-03	-7.87	27.13	7.72E-01
cg20502694	-58.45	23.61	1.33E-02	-59.59	31.09	5.52E-02	-48.23	133.53	7.18E-01	-43.79	51.35	3.94E-01	-73.72	55.56	1.85E-01
cg20070618	-13.52	5.46	1.33E-02	-27.61	11.18	1.35E-02	-8.31	20.24	6.81E-01	-6.31	7.33	3.89E-01	-21.22	14.98	1.57E-01
cg18494902	-9.50	3.84	1.33E-02	-10.75	5.78	6.29E-02	1.04	8.47	9.02E-01	-12.13	8.44	1.50E-01	-16.72	9.99	9.42E-02
cg14879760	14.47	5.85	1.33E-02	8.68	7.79	2.65E-01	15.81	14.46	2.74E-01	23.51	27.88	3.99E-01	26.02	12.21	3.31E-02
cg03374965	-44.51	17.98	1.33E-02	-38.60	22.99	9.31E-02	-22.98	60.17	7.03E-01	-28.47	53.62	5.95E-01	-83.93	41.65	4.39E-02
cg13234732	31.98	12.92	1.33E-02	51.23	20.83	1.39E-02	-1.20	32.67	9.71E-01	22.36	22.66	3.24E-01	38.75	35.32	2.72E-01
cg21871394	-20.62	8.33	1.33E-02	-34.61	20.74	9.52E-02	26.23	55.80	6.38E-01	-20.79	9.94	3.64E-02	-8.85	24.72	7.21E-01
cg03803086	10.34	4.18	1.33E-02	6.25	5.87	2.87E-01	19.59	8.90	2.77E-02	3.91	15.20	7.97E-01	13.02	9.42	1.67E-01
cg04554033	23.48	9.49	1.33E-02	54.88	31.76	8.41E-02	-9.50	92.32	9.18E-01	18.16	10.52	8.43E-02	45.29	32.30	1.61E-01
cg05544007	31.90	12.89	1.33E-02	47.47	24.41	5.18E-02	-13.16	58.20	8.21E-01	32.57	18.29	7.50E-02	17.87	30.74	5.61E-01
cg12353636	5.66	2.29	1.33E-02	2.64	3.67	4.72E-01	-0.98	5.94	8.68E-01	9.18	3.29	5.27E-03	9.00	5.51	1.02E-01
cg23808213	10.92	4.41	1.33E-02	3.04	4.53	5.03E-01	18.40	7.01	8.71E-03	19.66	9.85	4.60E-02	10.43	8.41	2.15E-01
cg21614420	-35.16	14.21	1.33E-02	-20.24	38.27	5.97E-01	-89.60	130.27	4.92E-01	-39.76	16.02	1.31E-02	-0.59	56.19	9.92E-01
cg17338133	14.15	5.72	1.33E-02	11.86	7.17	9.79E-02	22.23	17.15	1.95E-01	33.33	17.57	5.79E-02	4.08	14.98	7.85E-01
cg12211856	-25.83	10.44	1.33E-02	-29.71	16.91	7.89E-02	6.19	27.61	8.23E-01	-33.56	30.53	2.72E-01	-31.93	17.42	6.68E-02
cg26489057	-10.85	4.39	1.34E-02	-12.49	6.10	4.06E-02	-12.37	9.72	2.03E-01	-1.36	21.49	9.50E-01	-7.66	9.01	3.95E-01
cg19358493	19.36	7.82	1.34E-02	22.38	11.38	4.93E-02	-9.62	21.84	6.60E-01	41.32	22.44	6.56E-02	18.00	14.84	2.25E-01
cg20749283	23.26	9.40	1.34E-02	48.60	25.09	5.27E-02	-12.95	64.28	8.40E-01	19.67	10.73	6.68E-02	22.74	35.11	5.17E-01
cg15648958	31.19	12.61	1.34E-02	30.20	18.27	9.84E-02	55.77	25.83	3.08E-02	-30.31	42.63	4.77E-01	31.17	28.32	2.71E-01
cg04928670	11.59	4.68	1.34E-02	9.15	7.26	2.07E-01	6.15	9.41	5.13E-01	27.93	11.38	1.41E-02	9.13	11.48	4.26E-01
cg04102427	20.48	8.28	1.34E-02	28.64	14.71	5.15E-02	11.83	38.59	7.59E-01	13.13	11.51	2.54E-01	33.93	23.90	1.56E-01
cg18120376	26.85	10.85	1.34E-02	35.70	23.11	1.22E-01	33.73	57.69	5.59E-01	21.29	13.61	1.18E-01	39.30	33.06	2.34E-01
cg22882539	15.16	6.13	1.34E-02	17.81	23.97	4.57E-01	97.77	79.37	2.18E-01	14.13	6.52	3.02E-02	20.89	29.27	4.75E-01
cg03300883	15.32	6.19	1.34E-02	-2.79	19.33	8.85E-01	25.88	27.52	3.47E-01	18.07	6.97	9.49E-03	0.19	26.18	9.94E-01
cg23216724	6.02	2.43	1.34E-02	3.63	3.87	3.48E-01	11.33	4.60	1.37E-02	5.80	5.12	2.57E-01	0.96	7.77	9.02E-01
cg13071386	15.23	6.16	1.34E-02	32.65	11.88	6.00E-03	5.97	13.67	6.62E-01	8.99	14.18	5.26E-01	10.48	10.52	3.19E-01
cg07135032	11.64	4.70	1.34E-02	20.49	9.22	2.62E-02	8.48	11.97	4.79E-01	8.99	8.81	3.08E-01	8.09	8.59	3.47E-01
cg23312086	16.83	6.81	1.34E-02	5.90	10.20	5.63E-01	35.92	13.30	6.91E-03	21.45	11.70	6.68E-02	7.75	14.11	5.83E-01
cg21302951	14.06	5.68	1.34E-02	14.16	8.21	8.44E-02	27.11	16.34	9.71E-02	33.14	18.25	6.94E-02	2.55	10.34	8.05E-01
cg07217630	43.41	17.56	1.34E-02	73.49	35.24	3.70E-02	-31.76	158.02	8.41E-01	43.69	22.04	4.75E-02	-20.35	54.11	7.07E-01
cg23804585	21.72	8.78	1.34E-02	40.68	16.92	1.62E-02	7.10	31.92	8.24E-01	19.32	11.88	1.04E-01	-3.26	26.77	9.03E-01

cg24166628	22.68	9.17	1.34E-02	38.19	18.75	4.17E-02	13.70	17.04	4.21E-01	14.03	18.37	4.45E-01	27.41	19.49	1.60E-01
cg17952467	15.57	6.30	1.34E-02	23.02	16.25	1.56E-01	33.50	29.20	2.51E-01	17.06	7.37	2.06E-02	-15.90	20.35	4.35E-01
cg00978117	26.31	10.64	1.34E-02	19.75	20.02	3.24E-01	19.89	54.10	7.13E-01	26.04	14.42	7.09E-02	43.14	29.07	1.38E-01
cg11490987	34.07	13.78	1.34E-02	23.15	31.51	4.63E-01	-2.46	109.46	9.82E-01	46.77	16.60	4.83E-03	-24.53	42.75	5.66E-01
cg26968544	-9.24	3.74	1.34E-02	-13.89	7.15	5.20E-02	-15.38	12.32	2.12E-01	-6.29	5.32	2.38E-01	-6.54	9.89	5.08E-01
cg23510527	-15.54	6.29	1.34E-02	-27.65	22.32	2.15E-01	22.72	38.65	5.57E-01	-13.89	6.92	4.49E-02	-35.79	23.76	1.32E-01
cg14746387	7.68	3.11	1.34E-02	6.83	4.05	9.19E-02	8.72	7.23	2.28E-01	16.75	11.04	1.29E-01	4.90	8.05	5.42E-01
cg02153855	8.75	3.54	1.34E-02	4.33	4.79	3.66E-01	14.83	7.17	3.85E-02	20.26	14.90	1.74E-01	10.57	9.03	2.42E-01
cg07326149	-11.55	4.67	1.34E-02	-22.18	11.91	6.25E-02	-1.38	16.31	9.33E-01	-9.68	5.70	8.97E-02	-16.42	15.32	2.84E-01
cg00528902	18.39	7.44	1.34E-02	17.89	8.72	4.01E-02	25.17	35.14	4.74E-01	-36.41	50.70	4.73E-01	24.36	16.38	1.37E-01
cg03345037	17.69	7.15	1.34E-02	16.21	9.40	8.47E-02	38.46	25.23	1.27E-01	40.64	47.74	3.95E-01	13.50	12.69	2.87E-01
cg01371233	18.94	7.66	1.34E-02	30.90	14.28	3.05E-02	31.59	33.28	3.42E-01	12.38	10.16	2.23E-01	14.77	25.54	5.63E-01
cg14073497	27.07	10.95	1.34E-02	40.36	30.45	1.85E-01	-44.34	156.15	7.76E-01	21.44	12.29	8.10E-02	70.66	41.02	8.50E-02
cg19017293	12.09	4.89	1.34E-02	14.22	6.72	3.45E-02	6.39	9.70	5.10E-01	28.81	19.79	1.45E-01	7.60	12.43	5.41E-01
cg25800638	7.61	3.08	1.34E-02	6.94	4.36	1.11E-01	9.71	5.61	8.38E-02	20.29	12.34	1.00E-01	-0.29	8.32	9.72E-01
cg18637901	17.28	6.99	1.35E-02	15.30	10.01	1.26E-01	14.00	14.26	3.26E-01	20.16	23.22	3.85E-01	25.53	16.44	1.20E-01
cg21554569	-15.05	6.09	1.35E-02	-12.26	13.12	3.50E-01	8.27	16.69	6.20E-01	-20.17	8.20	1.39E-02	-23.99	19.38	2.16E-01
cg25945642	6.39	2.58	1.35E-02	7.86	3.52	2.56E-02	4.83	5.84	4.09E-01	11.75	8.30	1.57E-01	0.40	6.30	9.49E-01
cg23405198	19.83	8.02	1.35E-02	19.82	21.07	3.47E-01	-17.87	29.27	5.42E-01	25.55	9.57	7.61E-03	4.52	28.77	8.75E-01
cg07038400	9.63	3.90	1.35E-02	16.67	6.37	8.94E-03	2.07	7.63	7.86E-01	2.77	8.78	7.52E-01	13.75	9.49	1.48E-01
cg15520279	17.04	6.89	1.35E-02	23.78	13.47	7.75E-02	21.37	18.07	2.37E-01	11.02	17.14	5.20E-01	13.74	10.51	1.91E-01
cg15830591	8.17	3.31	1.35E-02	3.52	7.02	6.16E-01	19.30	7.85	1.40E-02	5.40	4.98	2.78E-01	9.90	8.24	2.30E-01
cg14354168	6.14	2.49	1.35E-02	6.16	3.57	8.50E-02	7.68	5.45	1.59E-01	6.22	7.97	4.35E-01	4.56	5.41	3.99E-01
cg14223966	4.74	1.92	1.35E-02	4.32	3.15	1.69E-01	6.06	4.21	1.50E-01	9.56	4.27	2.50E-02	-0.26	4.10	9.50E-01
cg07478501	22.71	9.19	1.35E-02	33.80	16.70	4.30E-02	12.08	16.64	4.68E-01	16.43	18.97	3.86E-01	31.32	23.15	1.76E-01
cg21841024	25.41	10.28	1.35E-02	34.93	20.23	8.42E-02	39.89	19.95	4.55E-02	2.85	16.06	8.59E-01	44.32	27.88	1.12E-01
cg17101285	18.17	7.36	1.35E-02	19.27	11.36	8.96E-02	18.33	14.15	1.95E-01	31.71	19.46	1.03E-01	3.62	17.97	8.40E-01
cg04101116	15.18	6.15	1.35E-02	9.64	19.93	6.29E-01	13.37	36.05	7.11E-01	15.17	6.76	2.49E-02	26.97	27.40	3.25E-01
cg03228555	-8.89	3.60	1.35E-02	-11.43	8.74	1.91E-01	-10.60	18.89	5.75E-01	-7.27	4.35	9.47E-02	-14.52	10.85	1.81E-01
cg18836485	-12.76	5.16	1.35E-02	-14.02	14.29	3.27E-01	-29.61	27.01	2.73E-01	-13.77	5.94	2.03E-02	7.50	18.67	6.88E-01
cg19804488	13.98	5.66	1.35E-02	6.87	8.00	3.91E-01	23.50	12.50	6.02E-02	25.28	16.41	1.24E-01	15.55	13.52	2.50E-01
cg01165177	18.87	7.64	1.35E-02	0.66	19.46	9.73E-01	46.09	19.43	1.77E-02	16.32	9.68	9.16E-02	21.63	29.24	4.60E-01
cg10404399	16.28	6.59	1.35E-02	33.74	16.03	3.53E-02	28.36	27.20	2.97E-01	10.81	7.72	1.61E-01	24.01	31.81	4.50E-01
cg02132946	-11.19	4.53	1.35E-02	-13.57	6.61	4.01E-02	-15.91	10.34	1.24E-01	-3.66	13.79	7.91E-01	-5.94	9.44	5.29E-01
cg08245249	-13.68	5.54	1.35E-02	-17.27	11.23	1.24E-01	3.44	13.09	7.93E-01	-14.54	8.07	7.16E-02	-30.30	16.91	7.31E-02

cg02175033	6.56	2.66	1.35E-02	7.03	3.32	3.43E-02	10.52	6.35	9.78E-02	5.18	10.66	6.27E-01	-0.76	7.56	9.20E-01
cg03119532	-18.58	7.52	1.35E-02	-13.12	15.61	4.01E-01	-4.46	49.12	9.28E-01	-21.28	9.48	2.47E-02	-17.70	22.24	4.26E-01
cg04625338	-9.53	3.86	1.35E-02	-12.67	5.70	2.63E-02	-0.45	11.45	9.68E-01	-13.39	10.54	2.04E-01	-6.39	7.09	3.68E-01
cg11850861	-12.45	5.04	1.35E-02	-15.32	7.18	3.28E-02	-15.72	12.14	1.95E-01	9.02	15.27	5.55E-01	-14.04	10.60	1.85E-01
cg06162030	11.97	4.85	1.35E-02	14.61	6.22	1.88E-02	21.49	13.46	1.10E-01	5.77	13.12	6.60E-01	-3.77	13.63	7.82E-01
cg02867514	-12.34	4.99	1.35E-02	-12.54	8.05	1.19E-01	-10.05	13.08	4.43E-01	-7.63	9.88	4.40E-01	-19.16	10.81	7.63E-02
cg01159623	27.37	11.08	1.35E-02	38.84	20.24	5.50E-02	-9.14	23.88	7.02E-01	25.69	17.41	1.40E-01	47.25	21.39	2.72E-02
cg02613601	-17.09	6.92	1.35E-02	-16.94	11.48	1.40E-01	-1.08	14.11	9.39E-01	-26.47	17.11	1.22E-01	-27.21	14.32	5.74E-02
cg12724221	11.05	4.48	1.35E-02	13.66	6.22	2.81E-02	10.33	10.72	3.35E-01	11.10	11.17	3.20E-01	2.73	11.65	8.15E-01
cg13216331	6.35	2.57	1.35E-02	6.89	3.63	5.78E-02	6.06	6.71	3.66E-01	6.16	5.25	2.40E-01	4.71	7.69	5.40E-01
cg16582156	-61.46	24.88	1.35E-02	-44.81	62.56	4.74E-01	-534.60	274.20	5.12E-02	-61.88	26.69	2.04E-02	-45.30	77.02	5.56E-01
cg24613798	-20.21	8.18	1.35E-02	-35.63	16.98	3.58E-02	-22.63	18.88	2.31E-01	-7.89	12.38	5.24E-01	-29.71	21.70	1.71E-01
cg05176211	17.70	7.17	1.35E-02	18.72	11.09	9.14E-02	20.39	16.01	2.03E-01	0.78	19.12	9.67E-01	23.57	14.60	1.07E-01
cg24786875	7.00	2.84	1.35E-02	6.18	3.76	1.00E-01	13.73	7.65	7.27E-02	6.78	7.23	3.49E-01	3.99	7.62	6.01E-01
cg10001720	4.57	1.85	1.35E-02	6.58	2.33	4.71E-03	0.08	4.15	9.85E-01	-1.02	8.77	9.07E-01	3.55	5.23	4.98E-01
cg15275019	-29.68	12.02	1.35E-02	-44.06	36.54	2.28E-01	52.28	132.11	6.92E-01	-25.88	13.39	5.33E-02	-57.70	43.05	1.80E-01
cg18246262	23.44	9.49	1.35E-02	49.22	21.16	2.00E-02	-4.99	49.61	9.20E-01	16.47	11.72	1.60E-01	27.57	29.28	3.46E-01
cg07716287	16.94	6.86	1.35E-02	22.44	15.36	1.44E-01	16.75	18.39	3.62E-01	18.23	9.02	4.33E-02	-4.89	23.78	8.37E-01
cg16113793	-34.37	13.92	1.35E-02	-35.98	18.53	5.21E-02	-32.46	38.04	3.93E-01	5.78	44.78	8.97E-01	-50.09	30.74	1.03E-01
cg04510564	-13.60	5.51	1.35E-02	-10.80	13.47	4.23E-01	-14.84	27.47	5.89E-01	-11.95	6.57	6.89E-02	-31.09	18.35	9.03E-02
cg23120848	-43.17	17.48	1.35E-02	-51.59	20.36	1.13E-02	-68.15	80.11	3.95E-01	20.44	71.38	7.75E-01	-20.00	44.42	6.53E-01
cg09767598	23.68	9.59	1.36E-02	40.98	22.65	7.04E-02	29.19	53.73	5.87E-01	16.47	12.06	1.72E-01	31.95	24.31	1.89E-01
cg01553209	39.09	15.83	1.36E-02	44.33	21.57	3.99E-02	-28.49	84.62	7.36E-01	115.63	61.40	5.97E-02	23.68	26.39	3.70E-01
cg17869514	19.66	7.96	1.36E-02	24.42	10.86	2.46E-02	41.17	20.28	4.24E-02	23.29	17.47	1.82E-01	-0.76	13.83	9.56E-01
cg26751372	20.43	8.28	1.36E-02	16.81	21.58	4.36E-01	-5.83	43.40	8.93E-01	23.27	9.53	1.46E-02	9.95	33.10	7.64E-01
cg14566857	-41.36	16.76	1.36E-02	-35.07	46.62	4.52E-01	-240.95	186.45	1.96E-01	-38.08	19.24	4.78E-02	-57.63	51.92	2.67E-01
cg19210349	-20.83	8.44	1.36E-02	7.58	21.94	7.30E-01	-47.32	42.33	2.64E-01	-21.94	9.99	2.81E-02	-44.61	26.80	9.60E-02
cg00718409	-11.57	4.69	1.36E-02	-10.78	9.42	2.52E-01	3.95	21.00	8.51E-01	-12.80	6.30	4.24E-02	-13.55	12.15	2.65E-01
cg20942219	25.75	10.43	1.36E-02	23.73	20.45	2.46E-01	36.58	23.73	1.23E-01	12.61	16.15	4.35E-01	56.15	29.05	5.33E-02
cg00551910	8.12	3.29	1.36E-02	7.08	4.76	1.36E-01	8.45	7.84	2.81E-01	15.36	8.00	5.50E-02	3.68	7.84	6.39E-01
cg13474450	7.33	2.97	1.36E-02	7.30	3.77	5.29E-02	9.37	7.60	2.17E-01	7.20	9.22	4.34E-01	5.04	8.44	5.50E-01
cg08517286	16.71	6.77	1.36E-02	50.48	26.75	5.92E-02	-31.98	48.65	5.11E-01	14.82	8.08	6.66E-02	17.22	14.63	2.39E-01
cg05171937	5.35	2.17	1.36E-02	6.16	2.90	3.34E-02	8.67	6.34	1.71E-01	6.63	4.97	1.83E-01	-2.78	5.93	6.39E-01
cg00109764	11.91	4.83	1.36E-02	7.38	6.93	2.87E-01	27.85	11.45	1.50E-02	5.67	11.66	6.27E-01	14.50	11.81	2.20E-01
cg25666403	-36.58	14.82	1.36E-02	-40.01	34.22	2.42E-01	-141.72	169.30	4.03E-01	-43.83	18.10	1.54E-02	10.54	40.49	7.95E-01

cg22314482	15.80	6.40	1.36E-02	5.19	15.54	7.38E-01	23.01	58.59	6.95E-01	19.11	7.43	1.01E-02	6.01	23.30	7.97E-01
cg02566775	6.95	2.82	1.36E-02	6.02	3.68	1.01E-01	12.90	7.21	7.36E-02	11.61	7.66	1.30E-01	-0.97	7.94	9.03E-01
cg06659580	39.16	15.87	1.36E-02	-27.64	52.77	6.00E-01	-182.92	225.22	4.17E-01	49.51	17.62	4.96E-03	25.78	51.87	6.19E-01
cg18622538	-9.96	4.03	1.36E-02	-8.87	4.71	5.93E-02	-8.76	8.72	3.15E-01	-29.17	11.12	8.72E-03	-3.85	6.99	5.82E-01
cg09588360	-39.95	16.19	1.36E-02	-41.22	19.21	3.19E-02	16.00	60.26	7.91E-01	-50.02	56.40	3.75E-01	-57.05	44.08	1.96E-01
cg04766371	24.08	9.76	1.36E-02	18.08	18.05	3.16E-01	67.15	29.84	2.44E-02	26.00	32.19	4.19E-01	18.13	13.69	1.85E-01
cg06100570	-13.03	5.28	1.36E-02	-2.67	17.28	8.77E-01	-18.71	31.79	5.56E-01	-14.67	5.88	1.26E-02	-6.05	19.46	7.56E-01
cg27528660	19.13	7.75	1.36E-02	37.36	22.48	9.65E-02	27.45	39.78	4.90E-01	16.90	9.39	7.18E-02	13.13	19.28	4.96E-01
cg05471296	35.49	14.38	1.36E-02	21.13	20.96	3.13E-01	-1.18	58.87	9.84E-01	69.90	44.04	1.12E-01	50.04	23.88	3.61E-02
cg01821022	6.33	2.57	1.36E-02	6.65	3.64	6.74E-02	7.72	6.04	2.02E-01	1.74	5.85	7.66E-01	10.04	7.16	1.61E-01
cg18865075	-13.34	5.41	1.36E-02	-10.03	10.22	3.27E-01	6.39	16.98	7.06E-01	-16.38	7.90	3.80E-02	-23.31	13.95	9.47E-02
cg09282201	-17.47	7.08	1.36E-02	-5.70	19.30	7.68E-01	-10.94	32.19	7.34E-01	-18.13	8.14	2.60E-02	-40.81	28.85	1.57E-01
cg20847228	-39.23	15.90	1.36E-02	-46.75	23.56	4.73E-02	71.58	73.72	3.32E-01	-41.06	27.84	1.40E-01	-45.81	38.33	2.32E-01
cg27567922	-13.38	5.42	1.36E-02	-22.79	13.60	9.38E-02	-1.21	17.35	9.45E-01	-9.70	7.26	1.82E-01	-22.84	12.61	7.01E-02
cg15627078	27.55	11.17	1.36E-02	17.98	35.71	6.15E-01	9.49	109.84	9.31E-01	25.64	12.32	3.75E-02	65.84	42.11	1.18E-01
cg26165081	7.94	3.22	1.36E-02	8.90	4.83	6.56E-02	6.64	8.14	4.15E-01	11.06	6.06	6.80E-02	-1.44	9.41	8.78E-01
cg17999280	18.25	7.40	1.36E-02	16.53	11.64	1.55E-01	20.07	14.64	1.70E-01	18.17	15.68	2.46E-01	20.31	21.54	3.46E-01
cg01825245	42.07	17.06	1.36E-02	-9.00	32.94	7.85E-01	20.15	108.55	8.53E-01	56.54	17.51	1.24E-03	66.39	42.01	1.14E-01
cg17698584	35.01	14.19	1.36E-02	39.43	20.42	5.35E-02	-1.86	33.95	9.56E-01	42.94	42.84	3.16E-01	49.79	29.44	9.08E-02
cg19427746	4.96	2.01	1.36E-02	2.68	2.70	3.21E-01	7.07	4.35	1.04E-01	3.44	7.07	6.27E-01	11.13	5.17	3.12E-02
cg23308107	41.02	16.63	1.36E-02	31.72	38.59	4.11E-01	56.77	87.15	5.15E-01	42.97	20.49	3.60E-02	39.92	48.18	4.07E-01
cg09983885	8.43	3.42	1.36E-02	3.82	4.80	4.26E-01	17.20	8.19	3.56E-02	16.19	9.86	1.01E-01	7.82	7.68	3.08E-01
cg25915307	16.49	6.69	1.36E-02	14.88	11.01	1.77E-01	22.08	17.43	2.05E-01	75.85	41.45	6.72E-02	12.62	9.88	2.01E-01
cg00831222	5.62	2.28	1.37E-02	2.65	3.32	4.24E-01	10.54	5.04	3.66E-02	2.98	7.90	7.06E-01	8.14	4.63	7.87E-02
cg14516287	-8.99	3.65	1.37E-02	-8.44	6.21	1.74E-01	-7.18	8.22	3.82E-01	-13.76	7.21	5.63E-02	-5.67	8.10	4.84E-01
cg18096987	9.94	4.03	1.37E-02	12.34	6.03	4.05E-02	10.22	9.99	3.06E-01	11.56	9.38	2.18E-01	3.02	8.90	7.35E-01
cg25707057	-69.28	28.09	1.37E-02	-75.57	36.50	3.84E-02	-119.03	108.35	2.72E-01	26.07	94.69	7.83E-01	-74.52	55.93	1.83E-01
cg05246315	21.07	8.54	1.37E-02	22.57	15.54	1.47E-01	24.97	15.91	1.16E-01	28.14	17.58	1.09E-01	2.29	20.55	9.11E-01
cg08703808	-15.21	6.17	1.37E-02	-27.59	11.17	1.36E-02	-13.11	14.90	3.79E-01	-8.08	11.65	4.88E-01	-9.42	12.49	4.51E-01
cg21888118	16.84	6.83	1.37E-02	33.00	19.53	9.10E-02	37.60	30.45	2.17E-01	12.16	7.77	1.18E-01	27.79	29.21	3.42E-01
cg07780095	11.70	4.74	1.37E-02	24.54	13.34	6.59E-02	12.14	14.16	3.91E-01	15.77	7.11	2.66E-02	0.67	8.44	9.36E-01
cg07602008	-25.66	10.41	1.37E-02	-4.52	24.72	8.55E-01	20.04	45.44	6.59E-01	-37.12	12.53	3.05E-03	-3.77	36.67	9.18E-01
cg07436562	18.18	7.37	1.37E-02	17.74	11.78	1.32E-01	28.97	12.62	2.17E-02	7.31	21.45	7.33E-01	3.24	19.11	8.66E-01
cg08799173	18.41	7.46	1.37E-02	-9.61	24.26	6.92E-01	23.77	39.76	5.50E-01	18.49	9.12	4.25E-02	30.47	16.71	6.82E-02
cg06500134	24.26	9.84	1.37E-02	41.67	13.42	1.90E-03	0.58	22.64	9.80E-01	10.30	27.92	7.12E-01	15.42	19.30	4.24E-01

cg11631160	18.55	7.52	1.37E-02	17.25	10.66	1.06E-01	35.50	31.64	2.62E-01	32.28	33.50	3.35E-01	16.01	11.96	1.81E-01
cg02433905	32.85	13.32	1.37E-02	36.20	16.12	2.47E-02	67.82	59.49	2.54E-01	-22.65	72.86	7.56E-01	23.46	27.59	3.95E-01
cg13992382	-14.71	5.96	1.37E-02	-28.51	14.42	4.80E-02	-2.54	34.50	9.41E-01	-10.44	7.25	1.50E-01	-21.97	17.07	1.98E-01
cg00809021	-18.81	7.63	1.37E-02	-19.89	12.97	1.25E-01	-26.02	14.47	7.20E-02	0.86	21.07	9.68E-01	-19.64	15.44	2.03E-01
cg02717503	17.93	7.27	1.37E-02	16.92	15.30	2.69E-01	-30.69	31.55	3.31E-01	25.05	9.87	1.11E-02	12.29	16.99	4.69E-01
cg07163991	-74.34	30.16	1.37E-02	-87.24	43.46	4.47E-02	82.01	189.02	6.64E-01	-78.58	60.70	1.95E-01	-61.04	60.76	3.15E-01
cg15103426	-53.59	21.74	1.37E-02	-49.08	27.77	7.72E-02	-109.88	110.76	3.21E-01	-2.02	96.22	9.83E-01	-64.44	39.85	1.06E-01
cg00456086	16.38	6.65	1.37E-02	25.04	11.41	2.82E-02	-6.64	28.87	8.18E-01	12.89	11.66	2.69E-01	14.31	12.50	2.52E-01
cg24625984	-9.05	3.67	1.37E-02	-10.10	5.76	7.96E-02	-5.92	9.05	5.13E-01	-4.35	7.46	5.60E-01	-15.61	8.49	6.60E-02
cg23636568	8.31	3.37	1.37E-02	1.87	5.14	7.17E-01	14.08	8.03	7.97E-02	12.93	7.34	7.81E-02	12.54	7.87	1.11E-01
cg26098816	-9.01	3.65	1.37E-02	-13.23	8.23	1.08E-01	-15.95	15.08	2.90E-01	-4.78	4.56	2.94E-01	-23.66	11.51	3.98E-02
cg08239297	17.70	7.18	1.37E-02	11.48	11.24	3.07E-01	18.97	13.46	1.59E-01	20.96	17.25	2.24E-01	29.79	19.65	1.30E-01
cg16818443	-39.28	15.94	1.37E-02	-37.03	42.15	3.80E-01	298.32	235.06	2.04E-01	-45.74	17.95	1.09E-02	10.54	62.73	8.67E-01
cg15543534	-17.14	6.95	1.37E-02	-19.02	15.26	2.12E-01	-7.41	32.85	8.22E-01	-20.76	8.58	1.55E-02	8.78	23.17	7.05E-01
cg05769505	-12.62	5.12	1.37E-02	-24.50	11.30	3.01E-02	-5.29	18.64	7.77E-01	-8.42	6.63	2.04E-01	-17.77	14.70	2.27E-01
cg22755816	-25.51	10.35	1.37E-02	-2.45	40.72	9.52E-01	-135.07	120.68	2.63E-01	-29.33	11.34	9.69E-03	0.93	33.64	9.78E-01
cg20557688	-8.47	3.44	1.37E-02	-16.21	7.70	3.53E-02	0.57	9.61	9.53E-01	-8.68	4.67	6.33E-02	-4.69	9.49	6.21E-01
cg23139584	9.71	3.94	1.37E-02	6.23	5.08	2.20E-01	13.10	11.11	2.38E-01	31.49	18.47	8.83E-02	12.68	8.27	1.25E-01
cg09317398	-13.47	5.47	1.37E-02	-15.79	7.90	4.56E-02	-23.99	12.25	5.03E-02	-5.34	16.26	7.43E-01	-2.51	11.98	8.34E-01
cg15091068	48.11	19.53	1.37E-02	38.97	24.41	1.10E-01	52.42	60.16	3.84E-01	67.93	63.05	2.81E-01	70.07	48.98	1.53E-01
cg14159026	17.65	7.16	1.38E-02	39.97	27.25	1.42E-01	-24.85	69.67	7.21E-01	17.01	7.78	2.88E-02	10.07	26.58	7.05E-01
cg03928429	-36.15	14.67	1.38E-02	-18.97	20.67	3.59E-01	-69.03	48.74	1.57E-01	-55.85	32.66	8.73E-02	-44.53	32.54	1.71E-01
cg12126038	9.96	4.04	1.38E-02	9.55	5.45	7.95E-02	12.46	9.48	1.89E-01	11.10	14.00	4.28E-01	8.20	9.44	3.85E-01
cg11190890	22.25	9.03	1.38E-02	34.30	17.22	4.64E-02	11.26	17.71	5.25E-01	11.30	15.48	4.66E-01	48.54	25.61	5.80E-02
cg18182358	32.43	13.16	1.38E-02	34.32	17.17	4.56E-02	101.30	69.62	1.46E-01	76.09	62.87	2.26E-01	15.90	22.83	4.86E-01
cg10175688	-22.75	9.24	1.38E-02	-10.73	38.28	7.79E-01	130.06	147.96	3.79E-01	-26.65	9.86	6.87E-03	12.55	37.64	7.39E-01
cg14499387	10.96	4.45	1.38E-02	14.07	7.80	7.11E-02	14.51	10.81	1.79E-01	9.84	6.88	1.53E-01	-2.25	15.08	8.81E-01
cg09163998	22.32	9.06	1.38E-02	31.70	12.24	9.59E-03	5.21	31.23	8.68E-01	-43.18	64.15	5.01E-01	15.43	15.37	3.15E-01
cg01959730	13.45	5.46	1.38E-02	18.91	12.46	1.29E-01	4.80	19.49	8.05E-01	14.50	7.88	6.60E-02	9.96	10.93	3.62E-01
cg12644264	31.22	12.68	1.38E-02	31.53	16.84	6.12E-02	44.01	73.06	5.47E-01	21.40	38.24	5.76E-01	33.00	23.41	1.59E-01
cg05716350	8.28	3.36	1.38E-02	2.28	5.84	6.97E-01	8.79	7.44	2.38E-01	14.05	5.79	1.51E-02	7.78	9.43	4.10E-01
cg07184698	52.34	21.25	1.38E-02	60.27	49.37	2.22E-01	-170.20	251.07	4.98E-01	55.59	24.98	2.61E-02	25.74	73.45	7.26E-01
cg02954056	14.62	5.94	1.38E-02	25.29	9.94	1.09E-02	8.22	18.07	6.49E-01	15.18	9.99	1.29E-01	-3.51	13.89	8.00E-01
cg00029246	7.27	2.95	1.38E-02	10.10	4.22	1.66E-02	5.50	6.27	3.80E-01	11.73	8.91	1.88E-01	-1.09	7.02	8.76E-01
cg06885284	16.23	6.59	1.38E-02	19.96	14.04	1.55E-01	12.25	21.60	5.71E-01	15.63	8.73	7.35E-02	15.29	19.26	4.27E-01

cg07593390	7.87	3.20	1.38E-02	5.02	4.96	3.11E-01	8.45	7.59	2.65E-01	11.85	5.89	4.43E-02	7.12	9.57	4.57E-01
cg05474761	8.70	3.53	1.38E-02	5.10	4.63	2.70E-01	14.47	7.99	7.03E-02	17.02	13.41	2.04E-01	11.35	9.10	2.12E-01
cg25127011	17.67	7.17	1.38E-02	20.35	17.12	2.34E-01	18.89	22.64	4.04E-01	13.43	9.06	1.38E-01	39.03	23.09	9.09E-02
cg04861793	-19.00	7.71	1.38E-02	-19.07	21.92	3.84E-01	-26.31	31.30	4.01E-01	-17.28	8.94	5.34E-02	-30.57	28.89	2.90E-01
cg14819242	-15.71	6.38	1.38E-02	-24.07	13.48	7.40E-02	-6.33	15.62	6.85E-01	-11.92	9.16	1.93E-01	-28.20	18.20	1.21E-01
cg25503565	27.51	11.17	1.38E-02	31.68	17.94	7.74E-02	-14.12	39.45	7.20E-01	32.24	17.93	7.22E-02	26.72	29.52	3.65E-01
cg14585415	17.54	7.13	1.38E-02	13.47	11.78	2.53E-01	25.75	12.72	4.29E-02	-2.96	19.70	8.80E-01	26.00	16.40	1.13E-01
cg08754088	-10.38	4.22	1.38E-02	-14.14	6.91	4.06E-02	-1.68	10.27	8.70E-01	-14.87	8.23	7.07E-02	-4.70	9.52	6.22E-01
cg01392273	23.56	9.57	1.38E-02	34.13	17.39	4.97E-02	12.99	17.77	4.65E-01	26.84	18.86	1.55E-01	16.99	24.75	4.92E-01
cg13253303	18.14	7.37	1.38E-02	28.58	11.98	1.70E-02	-26.56	25.05	2.89E-01	18.60	7.65	1.50E-02	20.96	16.16	1.95E-01
cg20327105	17.04	6.92	1.38E-02	13.88	8.84	1.16E-01	25.99	20.65	2.08E-01	-8.48	39.85	8.32E-01	23.99	14.00	8.67E-02
cg18484309	-8.35	3.39	1.38E-02	-8.59	5.35	1.08E-01	-3.52	7.36	6.32E-01	-8.17	7.53	2.78E-01	-13.62	7.94	8.61E-02
cg01198491	-18.32	7.44	1.38E-02	-15.77	13.82	2.54E-01	-24.66	20.91	2.38E-01	-10.51	5.04	3.72E-02	-48.54	18.18	7.58E-03
cg01049678	16.94	6.88	1.38E-02	18.27	9.03	4.29E-02	31.54	21.17	1.36E-01	20.56	20.64	3.19E-01	3.44	15.33	8.23E-01
cg19201068	-20.78	8.44	1.38E-02	-29.75	15.71	5.82E-02	-16.12	20.49	4.31E-01	-13.35	14.88	3.70E-01	-23.46	18.03	1.93E-01
cg24338411	-12.69	5.16	1.38E-02	-17.94	7.34	1.45E-02	-3.93	12.63	7.56E-01	-15.38	14.10	2.75E-01	-5.46	11.37	6.31E-01
cg05986044	32.03	13.01	1.39E-02	25.42	19.11	1.83E-01	-26.09	100.82	7.96E-01	33.35	23.24	1.51E-01	49.57	28.67	8.38E-02
cg22149355	11.78	4.79	1.39E-02	11.48	9.38	2.21E-01	1.51	11.24	8.93E-01	9.32	8.57	2.77E-01	22.84	9.66	1.81E-02
cg16734549	10.15	4.12	1.39E-02	13.01	5.54	1.88E-02	14.92	9.87	1.30E-01	-5.99	11.24	5.94E-01	8.31	11.21	4.59E-01
cg07655479	37.36	15.18	1.39E-02	45.70	19.07	1.66E-02	-17.56	79.21	8.25E-01	80.95	46.77	8.35E-02	2.30	32.06	9.43E-01
cg14240790	-14.17	5.76	1.39E-02	-17.11	9.12	6.08E-02	-16.87	14.72	2.52E-01	-14.65	12.31	2.34E-01	-6.83	12.01	5.70E-01
cg01747263	27.33	11.10	1.39E-02	23.96	13.34	7.24E-02	10.38	44.26	8.15E-01	82.78	73.76	2.62E-01	37.03	23.63	1.17E-01
cg01478155	12.61	5.12	1.39E-02	15.87	6.91	2.17E-02	19.00	12.05	1.15E-01	9.26	15.48	5.50E-01	-3.53	12.81	7.83E-01
cg21210630	19.25	7.83	1.39E-02	25.69	13.56	5.81E-02	19.83	16.04	2.16E-01	15.84	15.13	2.95E-01	10.79	19.49	5.80E-01
cg24788034	-25.83	10.50	1.39E-02	-32.45	14.73	2.76E-02	-40.43	39.36	3.04E-01	-36.93	24.90	1.38E-01	0.36	21.27	9.87E-01
cg17977795	35.48	14.42	1.39E-02	51.57	38.61	1.82E-01	-81.72	147.60	5.80E-01	32.85	16.43	4.55E-02	46.74	50.98	3.59E-01
cg24542360	-18.65	7.58	1.39E-02	1.46	14.02	9.17E-01	-50.04	38.78	1.97E-01	-20.40	9.89	3.91E-02	-29.97	13.32	2.44E-02
cg23990124	-9.38	3.81	1.39E-02	-12.91	7.40	8.10E-02	5.99	12.19	6.23E-01	-10.38	5.11	4.22E-02	-9.52	13.53	4.82E-01
cg21661111	-12.26	4.98	1.39E-02	-23.56	8.84	7.69E-03	-12.84	13.98	3.58E-01	-3.27	8.36	6.96E-01	-9.91	11.15	3.74E-01
cg05140624	-7.62	3.10	1.39E-02	-7.83	4.67	9.35E-02	-5.44	6.77	4.21E-01	-4.65	8.31	5.76E-01	-11.31	6.74	9.36E-02
cg26798452	18.64	7.58	1.39E-02	19.45	11.14	8.08E-02	18.01	16.76	2.83E-01	46.40	18.01	9.98E-03	5.53	11.02	6.16E-01
cg14065841	40.85	16.61	1.39E-02	28.64	21.85	1.90E-01	-42.86	76.18	5.74E-01	63.30	69.43	3.62E-01	71.52	29.46	1.52E-02
cg14148981	-11.74	4.77	1.39E-02	-11.53	8.24	1.62E-01	-8.04	15.02	5.92E-01	-8.24	7.15	2.49E-01	-29.00	13.99	3.81E-02
cg04638014	8.21	3.34	1.39E-02	5.03	4.88	3.03E-01	15.17	7.59	4.55E-02	9.15	8.47	2.80E-01	8.17	7.78	2.94E-01
cg23695880	-10.75	4.37	1.39E-02	-13.38	6.49	3.92E-02	0.12	9.20	9.90E-01	-13.65	14.16	3.35E-01	-15.10	9.21	1.01E-01

cg19379845	-20.43	8.31	1.39E-02	-15.72	24.31	5.18E-01	-61.60	30.62	4.43E-02	-19.52	9.67	4.36E-02	4.52	30.79	8.83E-01
cg18759209	20.41	8.30	1.39E-02	32.64	18.94	8.49E-02	1.48	24.41	9.52E-01	18.51	11.97	1.22E-01	23.95	18.01	1.84E-01
cg14208041	-7.62	3.10	1.39E-02	-9.32	5.86	1.12E-01	-1.26	9.80	8.98E-01	-5.51	4.46	2.17E-01	-16.02	8.29	5.33E-02
cg04240660	24.07	9.79	1.39E-02	38.82	17.53	2.68E-02	22.78	18.44	2.17E-01	9.84	8.21	2.31E-01	56.76	26.42	3.17E-02
cg05856556	-14.53	5.91	1.39E-02	-23.79	9.18	9.56E-03	-15.00	13.16	2.54E-01	-6.92	12.55	5.81E-01	-0.71	14.65	9.61E-01
cg14579177	-37.47	15.24	1.39E-02	-49.11	18.83	9.12E-03	-50.38	82.31	5.41E-01	-10.40	57.39	8.56E-01	-11.90	31.06	7.02E-01
cg21625817	-10.11	4.11	1.39E-02	-10.61	9.21	2.49E-01	-27.73	22.69	2.22E-01	-8.42	5.22	1.07E-01	-12.57	10.66	2.38E-01
cg08838842	11.57	4.71	1.39E-02	9.75	6.34	1.24E-01	23.29	8.64	7.02E-03	-1.24	8.54	8.85E-01	15.23	8.01	5.74E-02
cg21095280	-9.95	4.05	1.39E-02	-12.20	5.92	3.94E-02	-15.57	9.14	8.83E-02	-4.14	12.05	7.31E-01	-3.27	8.55	7.02E-01
cg19380303	-14.33	5.83	1.39E-02	-11.54	8.87	1.93E-01	-14.75	15.03	3.26E-01	-19.68	14.73	1.82E-01	-15.51	11.41	1.74E-01
cg00978203	20.33	8.27	1.39E-02	8.82	16.58	5.95E-01	26.12	18.80	1.65E-01	15.24	13.51	2.59E-01	40.21	19.30	3.72E-02
cg13567156	-33.24	13.52	1.39E-02	-43.05	23.47	6.66E-02	41.01	74.14	5.80E-01	-30.96	25.88	2.32E-01	-32.78	22.46	1.44E-01
cg05808356	18.69	7.60	1.39E-02	9.60	20.30	6.36E-01	-1.45	36.32	9.68E-01	24.32	8.93	6.48E-03	-2.31	25.18	9.27E-01
cg02656594	6.83	2.78	1.39E-02	5.08	3.76	1.76E-01	10.40	6.64	1.17E-01	10.99	8.46	1.94E-01	6.16	6.77	3.63E-01
cg26144909	-6.48	2.63	1.40E-02	-3.21	3.24	3.21E-01	-2.25	5.66	6.91E-01	-13.34	6.93	5.43E-02	-11.53	4.62	1.26E-02
cg15476528	29.34	11.93	1.40E-02	35.47	42.93	4.09E-01	-17.73	114.39	8.77E-01	29.18	13.27	2.79E-02	30.92	37.12	4.05E-01
cg12636835	-8.20	3.34	1.40E-02	-6.54	4.69	1.63E-01	-12.48	7.84	1.12E-01	-6.18	9.01	4.93E-01	-10.13	7.94	2.02E-01
cg18489675	-10.53	4.28	1.40E-02	-13.77	5.85	1.85E-02	-0.69	11.88	9.54E-01	1.48	12.11	9.03E-01	-15.55	9.40	9.82E-02
cg03358154	-10.93	4.45	1.40E-02	-15.52	6.10	1.10E-02	-1.84	12.18	8.80E-01	-2.69	13.34	8.40E-01	-9.55	9.36	3.08E-01
cg20235651	-26.54	10.80	1.40E-02	-21.48	12.14	7.67E-02	25.19	76.37	7.42E-01	-77.29	76.82	3.14E-01	-50.41	26.27	5.50E-02
cg22994198	6.77	2.75	1.40E-02	3.37	4.49	4.53E-01	5.29	5.17	3.06E-01	16.49	7.50	2.78E-02	8.68	6.09	1.55E-01
cg13023677	9.33	3.80	1.40E-02	11.00	5.11	3.13E-02	14.02	9.03	1.20E-01	14.56	13.56	2.83E-01	-1.93	8.67	8.24E-01
cg19828146	-13.45	5.47	1.40E-02	-12.96	11.18	2.46E-01	5.26	16.71	7.53E-01	-20.97	7.67	6.28E-03	-1.66	14.41	9.08E-01
cg01275932	12.78	5.20	1.40E-02	10.97	7.26	1.31E-01	17.36	10.51	9.87E-02	31.82	17.96	7.63E-02	1.50	13.04	9.09E-01
cg09789650	-13.59	5.53	1.40E-02	-16.94	8.13	3.73E-02	-17.83	12.35	1.49E-01	-10.85	18.25	5.52E-01	-4.85	11.16	6.64E-01
cg27597084	-29.19	11.88	1.40E-02	-34.29	32.60	2.93E-01	5.92	43.02	8.91E-01	-33.02	14.03	1.86E-02	-19.24	43.36	6.57E-01
cg02200939	32.29	13.14	1.40E-02	39.92	17.41	2.19E-02	-68.20	85.91	4.27E-01	45.46	40.95	2.67E-01	21.28	23.82	3.72E-01
ch.6.1361432F	-19.19	7.81	1.40E-02	-24.34	13.66	7.47E-02	-15.76	14.23	2.68E-01	-20.00	17.20	2.45E-01	-14.26	19.17	4.57E-01
cg24998879	-20.55	8.36	1.40E-02	-9.09	19.56	6.42E-01	-23.27	26.25	3.75E-01	-20.08	10.50	5.58E-02	-46.77	29.39	1.12E-01
cg09340205	-12.76	5.19	1.40E-02	-19.32	14.76	1.90E-01	37.77	32.85	2.50E-01	-12.96	5.89	2.78E-02	-16.78	19.12	3.80E-01
cg20383624	-38.72	15.76	1.40E-02	-32.32	19.92	1.05E-01	-36.13	50.19	4.72E-01	-87.40	32.34	6.89E-03	-6.57	31.77	8.36E-01
cg06528306	24.42	9.94	1.40E-02	40.99	14.77	5.53E-03	27.11	38.00	4.76E-01	2.98	32.03	9.26E-01	9.74	16.06	5.44E-01
cg13414916	54.25	22.08	1.40E-02	52.94	26.73	4.77E-02	-31.21	113.58	7.83E-01	53.54	102.27	6.01E-01	72.05	45.69	1.15E-01
cg09355803	32.67	13.30	1.40E-02	19.35	18.24	2.89E-01	23.73	44.56	5.94E-01	68.00	45.46	1.35E-01	49.22	24.54	4.49E-02
cg10647547	-11.82	4.81	1.40E-02	-18.59	12.09	1.24E-01	-36.13	24.04	1.33E-01	-6.87	5.51	2.12E-01	-26.17	15.01	8.13E-02

cg09685182	17.54	7.14	1.40E-02	25.69	16.61	1.22E-01	6.25	16.82	7.10E-01	30.17	12.38	1.48E-02	5.43	12.97	6.76E-01
cg09430445	-14.17	5.77	1.40E-02	-17.71	10.26	8.44E-02	-18.36	12.92	1.55E-01	-8.63	10.87	4.27E-01	-12.23	12.78	3.39E-01
cg09774917	13.62	5.55	1.40E-02	22.39	9.96	2.45E-02	16.51	17.50	3.45E-01	12.66	9.35	1.76E-01	2.38	11.38	8.35E-01
cg00050133	-14.21	5.79	1.40E-02	-18.29	11.37	1.08E-01	-6.71	14.60	6.46E-01	-13.45	8.32	1.06E-01	-19.10	18.22	2.94E-01
cg27497299	21.98	8.95	1.40E-02	31.61	20.28	1.19E-01	10.57	20.38	6.04E-01	21.50	12.56	8.69E-02	27.36	27.59	3.21E-01
cg18963365	7.23	2.94	1.40E-02	6.00	3.40	7.77E-02	14.49	4.95	3.44E-03	9.50	8.94	2.88E-01	0.15	5.46	9.78E-01
cg25356611	8.46	3.45	1.40E-02	8.05	5.20	1.22E-01	8.71	7.06	2.17E-01	2.91	9.10	7.49E-01	13.62	8.15	9.47E-02
cg15963499	-9.35	3.81	1.40E-02	-19.70	8.54	2.11E-02	0.21	16.93	9.90E-01	-5.65	4.78	2.38E-01	-15.90	11.10	1.52E-01
cg07547746	15.61	6.35	1.40E-02	24.94	12.69	4.93E-02	7.04	19.17	7.13E-01	13.01	8.68	1.34E-01	15.52	19.75	4.32E-01
cg14058010	28.67	11.67	1.40E-02	31.38	15.06	3.72E-02	18.53	29.60	5.31E-01	5.77	48.54	9.05E-01	35.53	27.08	1.89E-01
cg20356241	78.23	31.85	1.40E-02	77.15	44.77	8.49E-02	168.96	164.41	3.04E-01	120.04	70.70	8.95E-02	33.46	63.27	5.97E-01
cg20610452	8.22	3.35	1.41E-02	9.70	5.59	8.28E-02	12.06	6.89	8.02E-02	6.87	8.39	4.13E-01	3.28	6.73	6.26E-01
cg12467960	46.27	18.84	1.41E-02	49.23	27.29	7.12E-02	-3.70	56.94	9.48E-01	101.13	53.05	5.66E-02	36.35	35.14	3.01E-01
cg15392844	-6.77	2.75	1.41E-02	-4.02	3.92	3.05E-01	-14.06	7.02	4.53E-02	-13.40	8.09	9.75E-02	-4.49	5.67	4.28E-01
cg13974192	10.21	4.16	1.41E-02	15.83	8.66	6.75E-02	11.43	9.88	2.47E-01	7.52	7.58	3.21E-01	7.79	7.70	3.12E-01
cg27521563	-30.52	12.43	1.41E-02	-20.10	39.25	6.09E-01	-79.26	148.76	5.94E-01	-32.69	13.77	1.76E-02	-16.78	44.57	7.07E-01
cg03163459	8.78	3.58	1.41E-02	6.95	4.92	1.58E-01	11.84	7.79	1.28E-01	0.42	14.67	9.77E-01	12.83	7.96	1.07E-01
cg25540028	-10.04	4.09	1.41E-02	-8.34	6.66	2.10E-01	4.34	12.87	7.36E-01	-12.53	6.24	4.46E-02	-21.13	13.46	1.16E-01
cg09954820	-10.20	4.16	1.41E-02	-16.19	10.99	1.41E-01	-11.38	17.46	5.15E-01	-6.80	5.02	1.75E-01	-22.61	12.31	6.61E-02
cg23924137	13.95	5.68	1.41E-02	13.17	12.99	3.11E-01	3.18	23.61	8.93E-01	16.02	6.94	2.11E-02	6.45	20.01	7.47E-01
cg07979236	5.91	2.41	1.41E-02	5.68	3.08	6.47E-02	12.43	5.59	2.61E-02	5.54	8.74	5.26E-01	-2.29	6.73	7.34E-01
cg21897880	19.50	7.94	1.41E-02	16.64	15.15	2.72E-01	28.94	15.15	5.60E-02	8.12	18.54	6.61E-01	20.56	15.39	1.82E-01
cg07001734	23.50	9.57	1.41E-02	30.45	16.12	5.89E-02	-29.19	39.36	4.58E-01	23.34	13.98	9.51E-02	29.68	27.68	2.84E-01
cg26266876	8.97	3.65	1.41E-02	11.17	5.12	2.92E-02	12.39	10.41	2.34E-01	9.51	8.36	2.55E-01	-0.31	8.68	9.72E-01
cg23990435	22.56	9.19	1.41E-02	22.26	20.14	2.69E-01	19.51	19.03	3.05E-01	19.18	13.97	1.70E-01	40.31	25.88	1.19E-01
cg05381746	18.64	7.59	1.41E-02	25.04	27.28	3.59E-01	0.16	47.65	9.97E-01	20.23	8.50	1.73E-02	5.59	24.09	8.16E-01
cg11303605	20.57	8.38	1.41E-02	1.23	20.41	9.52E-01	13.38	35.98	7.10E-01	26.32	10.21	9.95E-03	18.47	26.06	4.79E-01
cg16719228	14.35	5.85	1.41E-02	9.31	17.93	6.04E-01	57.80	60.41	3.39E-01	16.14	6.60	1.44E-02	1.43	18.62	9.39E-01
cg04439516	75.67	30.82	1.41E-02	81.96	41.26	4.70E-02	35.68	169.40	8.33E-01	38.10	17.61	3.05E-02	176.57	60.61	3.58E-03
cg18151410	21.04	8.57	1.41E-02	44.20	21.05	3.57E-02	14.59	35.67	6.83E-01	16.09	10.24	1.16E-01	21.03	31.02	4.98E-01
cg19692710	-13.59	5.54	1.41E-02	-12.35	15.36	4.21E-01	10.85	18.88	5.65E-01	-16.83	6.50	9.58E-03	-12.03	22.99	6.01E-01
cg02103647	-11.78	4.80	1.41E-02	-19.21	11.26	8.81E-02	6.17	20.89	7.68E-01	-11.33	5.83	5.18E-02	-10.65	16.22	5.12E-01
cg12636435	7.31	2.98	1.41E-02	4.69	3.05	1.24E-01	14.34	5.15	5.32E-03	13.63	9.58	1.55E-01	1.00	6.72	8.82E-01
cg15567853	-13.00	5.30	1.41E-02	-9.97	12.67	4.31E-01	16.34	20.69	4.30E-01	-14.35	6.50	2.73E-02	-28.99	16.95	8.72E-02
cg01256227	-7.25	2.95	1.41E-02	-6.43	4.09	1.16E-01	-7.81	7.33	2.87E-01	-7.39	9.95	4.57E-01	-8.69	6.19	1.60E-01

cg24013351	-14.31	5.83	1.41E-02	-23.53	16.56	1.55E-01	7.91	21.12	7.08E-01	-14.88	6.85	2.99E-02	-16.15	21.24	4.47E-01
cg02608831	-10.42	4.24	1.41E-02	-14.34	8.53	9.27E-02	-7.25	9.36	4.39E-01	-13.68	7.15	5.60E-02	-2.89	9.61	7.63E-01
cg07849409	-30.83	12.56	1.41E-02	-32.63	13.58	1.63E-02	-4.38	82.51	9.58E-01	-43.24	72.69	5.52E-01	-16.70	41.46	6.87E-01
cg14807793	15.92	6.49	1.41E-02	20.29	9.84	3.91E-02	8.61	14.21	5.45E-01	17.46	18.06	3.34E-01	13.41	13.60	3.24E-01
cg16253997	-23.81	9.70	1.41E-02	-21.63	18.94	2.53E-01	-72.61	54.98	1.87E-01	-22.17	12.92	8.61E-02	-23.63	25.71	3.58E-01
cg17124844	-17.47	7.12	1.41E-02	-25.52	15.53	1.00E-01	-12.05	19.43	5.35E-01	-15.63	10.09	1.21E-01	-17.19	17.93	3.38E-01
cg26539468	8.47	3.45	1.41E-02	8.80	4.94	7.45E-02	14.04	8.17	8.59E-02	5.86	8.35	4.82E-01	4.07	8.59	6.35E-01
cg03297569	-31.86	12.98	1.41E-02	-34.61	17.87	5.29E-02	-207.16	255.90	4.18E-01	-45.66	20.77	2.79E-02	19.57	35.05	5.77E-01
cg05576959	9.63	3.92	1.41E-02	11.80	5.46	3.07E-02	14.64	8.69	9.19E-02	2.89	11.95	8.09E-01	1.39	9.46	8.83E-01
cg14361895	-17.42	7.10	1.41E-02	-25.50	15.03	8.97E-02	-5.80	31.57	8.54E-01	-15.57	8.92	8.08E-02	-16.89	23.26	4.68E-01
cg13866527	22.80	9.29	1.41E-02	28.01	10.59	8.18E-03	1.64	74.22	9.82E-01	30.29	39.70	4.46E-01	-2.71	23.20	9.07E-01
cg10448529	24.79	10.10	1.41E-02	25.18	16.18	1.20E-01	-28.57	48.89	5.59E-01	28.59	16.23	7.81E-02	28.41	23.80	2.33E-01
cg11700230	11.80	4.81	1.41E-02	9.87	7.12	1.66E-01	15.15	10.00	1.30E-01	1.47	16.34	9.28E-01	16.21	10.10	1.08E-01
cg17828223	-44.02	17.94	1.41E-02	-104.87	68.58	1.26E-01	-73.89	283.53	7.94E-01	-38.17	19.33	4.83E-02	-55.47	69.79	4.27E-01
cg22760295	41.83	17.05	1.41E-02	-8.17	52.65	8.77E-01	111.56	203.23	5.83E-01	41.10	19.14	3.18E-02	97.80	55.27	7.68E-02
cg19940482	-11.19	4.56	1.41E-02	-18.24	6.78	7.19E-03	-4.99	10.94	6.48E-01	-1.56	13.31	9.07E-01	-7.39	8.98	4.11E-01
cg00386338	-53.20	21.68	1.41E-02	-43.52	29.34	1.38E-01	10.90	82.53	8.95E-01	-3.55	76.86	9.63E-01	-97.93	39.24	1.26E-02
cg10233401	-17.07	6.96	1.41E-02	-21.18	19.39	2.75E-01	-16.91	24.78	4.95E-01	-17.51	8.22	3.32E-02	-6.24	25.08	8.03E-01
cg24494102	-8.55	3.49	1.41E-02	-13.59	6.53	3.74E-02	-6.79	11.00	5.37E-01	-7.91	5.19	1.28E-01	-2.65	8.61	7.58E-01
cg20052509	27.39	11.16	1.42E-02	36.64	15.98	2.18E-02	5.29	25.33	8.35E-01	-3.15	30.14	9.17E-01	49.41	26.30	6.03E-02
cg26843469	-29.91	12.19	1.42E-02	-31.05	28.98	2.84E-01	-25.95	107.89	8.10E-01	-31.13	14.72	3.44E-02	-21.94	34.66	5.27E-01
cg01223697	20.86	8.50	1.42E-02	25.29	21.82	2.46E-01	34.38	28.85	2.33E-01	19.29	10.13	5.70E-02	7.94	35.65	8.24E-01
cg21654404	18.47	7.53	1.42E-02	29.34	15.21	5.37E-02	-5.48	24.63	8.24E-01	14.60	10.21	1.53E-01	32.67	21.92	1.36E-01
cg15049964	-41.44	16.89	1.42E-02	-32.46	23.34	1.64E-01	-38.92	47.43	4.12E-01	-52.20	43.55	2.31E-01	-58.60	37.90	1.22E-01
cg01089858	-14.31	5.83	1.42E-02	-17.14	9.35	6.68E-02	-25.15	13.67	6.57E-02	-16.38	12.91	2.04E-01	1.26	12.30	9.18E-01
cg13812377	23.40	9.54	1.42E-02	-7.44	31.59	8.14E-01	22.77	130.57	8.62E-01	27.50	10.42	8.29E-03	13.79	37.57	7.14E-01
cg09661503	-37.14	15.14	1.42E-02	-39.03	18.58	3.57E-02	-86.16	31.94	6.98E-03	-12.11	6.63	6.76E-02	-48.53	25.45	5.66E-02
cg18898992	16.71	6.81	1.42E-02	23.54	11.11	3.41E-02	29.10	14.81	4.94E-02	-1.00	12.58	9.36E-01	16.73	18.07	3.55E-01
cg07466320	21.38	8.72	1.42E-02	22.63	14.56	1.20E-01	19.19	16.50	2.45E-01	27.59	20.05	1.69E-01	15.57	20.95	4.57E-01
cg19126615	12.18	4.97	1.42E-02	13.46	6.32	3.32E-02	22.72	9.81	2.05E-02	-8.05	14.32	5.74E-01	7.79	11.62	5.03E-01
cg27410136	16.43	6.70	1.42E-02	17.59	11.95	1.41E-01	20.79	14.15	1.42E-01	12.31	11.54	2.86E-01	16.83	19.05	3.77E-01
cg24316515	13.87	5.66	1.42E-02	11.14	9.20	2.26E-01	0.66	20.28	9.74E-01	20.82	10.03	3.78E-02	13.21	11.92	2.68E-01
cg14784617	-42.30	17.25	1.42E-02	-28.60	21.98	1.93E-01	-76.13	50.06	1.28E-01	-20.26	82.15	8.05E-01	-66.68	36.68	6.91E-02
cg06050193	-18.45	7.53	1.42E-02	-13.65	19.76	4.90E-01	-52.26	48.91	2.85E-01	-15.13	8.58	7.77E-02	-58.24	30.32	5.47E-02
cg00852675	-12.24	4.99	1.42E-02	-10.30	6.46	1.11E-01	-8.35	20.03	6.77E-01	-22.81	12.80	7.48E-02	-11.13	11.49	3.33E-01

cg04222027	55.71	22.72	1.42E-02	46.38	29.05	1.10E-01	-73.52	177.15	6.78E-01	76.16	83.46	3.61E-01	76.91	41.64	6.48E-02
cg27234067	24.52	10.00	1.42E-02	40.73	21.27	5.55E-02	24.45	24.82	3.25E-01	28.98	15.01	5.35E-02	-7.60	24.05	7.52E-01
cg01714172	21.04	8.58	1.42E-02	23.81	16.87	1.58E-01	12.26	16.60	4.60E-01	21.60	14.41	1.34E-01	33.02	24.84	1.84E-01
cg04229487	-18.71	7.63	1.42E-02	-27.18	13.67	4.68E-02	-33.69	18.36	6.65E-02	-12.66	12.69	3.18E-01	1.09	19.48	9.55E-01
cg22380921	12.67	5.17	1.42E-02	20.30	8.38	1.54E-02	3.17	11.97	7.91E-01	18.91	14.27	1.85E-01	6.21	9.42	5.10E-01
cg09769104	-12.52	5.11	1.42E-02	-12.04	12.19	3.23E-01	-26.96	24.96	2.80E-01	-11.04	6.15	7.26E-02	-17.97	16.79	2.84E-01
cg24274090	-9.24	3.77	1.42E-02	-18.40	7.65	1.62E-02	-8.60	10.07	3.93E-01	-5.49	5.48	3.17E-01	-6.74	9.93	4.97E-01
cg26688762	8.09	3.30	1.42E-02	5.05	4.98	3.10E-01	14.62	6.81	3.18E-02	16.69	8.43	4.78E-02	2.03	6.33	7.49E-01
cg01804452	36.31	14.81	1.42E-02	52.21	20.52	1.09E-02	1.25	31.07	9.68E-01	48.80	47.26	3.02E-01	26.19	37.85	4.89E-01
cg01407748	17.56	7.16	1.42E-02	16.47	14.37	2.52E-01	13.39	26.63	6.15E-01	12.14	9.81	2.16E-01	41.31	18.77	2.77E-02
cg09234698	13.30	5.43	1.42E-02	19.01	14.48	1.89E-01	4.87	21.33	8.19E-01	13.36	6.39	3.66E-02	9.31	20.07	6.43E-01
cg12037129	21.62	8.82	1.42E-02	36.89	18.24	4.31E-02	3.92	36.66	9.15E-01	12.52	11.57	2.79E-01	43.24	24.76	8.07E-02
cg15769920	29.82	12.17	1.42E-02	40.16	14.17	4.59E-03	-32.13	49.31	5.15E-01	-13.39	69.16	8.46E-01	15.10	29.42	6.08E-01
cg26495839	16.60	6.77	1.43E-02	27.75	10.43	7.79E-03	12.99	13.01	3.18E-01	-7.54	26.72	7.78E-01	7.65	13.75	5.78E-01
cg03196627	45.66	18.63	1.43E-02	35.68	23.60	1.31E-01	148.73	77.29	5.43E-02	21.64	56.56	7.02E-01	59.13	40.64	1.46E-01
cg19954205	-26.72	10.90	1.43E-02	-43.50	19.14	2.30E-02	-26.48	39.39	5.01E-01	-10.42	16.46	5.26E-01	-37.44	27.23	1.69E-01
cg25815682	9.00	3.67	1.43E-02	6.14	7.27	3.99E-01	3.49	8.14	6.68E-01	12.20	5.63	3.02E-02	13.28	10.82	2.20E-01
cg22031392	26.84	10.95	1.43E-02	7.06	28.56	8.05E-01	-34.66	121.12	7.75E-01	30.23	12.53	1.58E-02	36.98	38.59	3.38E-01
cg11919525	-10.83	4.42	1.43E-02	-8.93	16.47	5.88E-01	-4.83	7.54	5.21E-01	48.81	43.11	2.58E-01	-14.91	4.75	1.70E-03
cg16624013	-34.78	14.19	1.43E-02	-34.17	19.00	7.21E-02	3.90	38.26	9.19E-01	-22.50	54.28	6.78E-01	-62.37	29.23	3.29E-02
cg14828484	-52.62	21.48	1.43E-02	-89.72	57.04	1.16E-01	-385.84	250.07	1.23E-01	-42.24	24.80	8.86E-02	-53.32	67.55	4.30E-01
cg04501217	20.87	8.52	1.43E-02	30.36	15.31	4.73E-02	18.78	16.90	2.66E-01	23.41	15.16	1.23E-01	-5.65	24.49	8.18E-01
cg08367465	21.75	8.88	1.43E-02	19.23	13.16	1.44E-01	17.19	16.85	3.08E-01	55.38	28.47	5.17E-02	16.71	21.50	4.37E-01
cg19628343	19.97	8.15	1.43E-02	26.46	20.67	2.01E-01	0.02	26.10	9.99E-01	20.29	10.00	4.25E-02	28.64	28.26	3.11E-01
cg12169512	47.47	19.37	1.43E-02	25.50	46.12	5.80E-01	-45.69	183.31	8.03E-01	51.09	22.91	2.57E-02	71.34	62.09	2.51E-01
cg00256046	5.12	2.09	1.43E-02	2.78	3.99	4.86E-01	2.85	5.16	5.80E-01	6.95	3.24	3.18E-02	6.83	5.48	2.12E-01
cg07565956	11.58	4.73	1.43E-02	12.42	6.05	4.01E-02	10.98	12.12	3.65E-01	26.96	14.58	6.45E-02	-3.80	12.99	7.70E-01
cg09876519	13.45	5.49	1.43E-02	17.86	9.00	4.72E-02	8.24	13.99	5.56E-01	24.28	12.51	5.22E-02	3.04	10.36	7.69E-01
cg12898512	17.39	7.10	1.43E-02	23.97	18.58	1.97E-01	12.95	29.11	6.56E-01	16.20	8.39	5.35E-02	19.39	25.32	4.44E-01
cg25465322	11.51	4.70	1.43E-02	15.86	7.53	3.53E-02	14.96	9.62	1.20E-01	16.97	12.37	1.70E-01	-2.98	9.85	7.62E-01
cg05269784	6.62	2.70	1.43E-02	5.72	3.69	1.21E-01	6.88	6.55	2.93E-01	10.01	9.50	2.92E-01	7.44	5.90	2.07E-01
cg16301857	42.35	17.29	1.43E-02	46.40	19.71	1.85E-02	-34.13	99.54	7.32E-01	59.29	67.73	3.81E-01	28.15	47.00	5.49E-01
cg02267488	-18.33	7.48	1.43E-02	-9.99	14.37	4.87E-01	-14.93	18.22	4.13E-01	-23.65	11.77	4.45E-02	-22.69	18.92	2.30E-01
cg22309826	-45.84	18.71	1.43E-02	-34.44	23.89	1.49E-01	-49.86	58.21	3.92E-01	0.70	60.31	9.91E-01	-104.90	43.26	1.53E-02
cg01985106	-11.98	4.89	1.43E-02	-22.25	10.55	3.49E-02	-1.82	12.73	8.87E-01	-11.73	6.67	7.86E-02	-6.23	15.54	6.89E-01

cg18569661	14.32	5.85	1.43E-02	11.87	8.27	1.51E-01	27.54	12.32	2.54E-02	15.09	20.04	4.52E-01	4.77	13.40	7.22E-01
cg08933505	21.48	8.77	1.43E-02	38.85	24.62	1.15E-01	1.25	43.24	9.77E-01	20.32	10.57	5.46E-02	17.50	23.14	4.50E-01
cg18317135	11.74	4.79	1.43E-02	10.50	6.80	1.23E-01	17.32	8.47	4.09E-02	18.46	21.36	3.87E-01	0.41	13.13	9.75E-01
cg01392351	-14.28	5.83	1.43E-02	-19.84	15.48	2.00E-01	1.54	27.14	9.55E-01	-13.10	6.79	5.37E-02	-24.95	21.21	2.39E-01
cg08246659	19.44	7.94	1.43E-02	22.48	17.99	2.11E-01	-38.01	37.61	3.12E-01	23.30	9.77	1.71E-02	13.73	24.91	5.82E-01
cg25016308	-15.12	6.18	1.43E-02	-15.47	18.30	3.98E-01	6.46	24.95	7.96E-01	-16.38	7.01	1.94E-02	-21.38	27.94	4.44E-01
cg24889694	-15.28	6.24	1.43E-02	-19.54	17.94	2.76E-01	9.95	35.49	7.79E-01	-16.77	7.01	1.67E-02	1.42	26.60	9.58E-01
cg06421238	-71.49	29.19	1.43E-02	-64.57	38.61	9.44E-02	2.25	126.04	9.86E-01	-118.42	71.14	9.60E-02	-71.53	64.28	2.66E-01
cg05559643	-10.06	4.11	1.43E-02	-6.63	8.40	4.30E-01	-7.52	14.82	6.12E-01	-12.35	5.57	2.67E-02	-8.45	10.95	4.40E-01
cg17658266	-9.71	3.97	1.43E-02	-15.66	9.79	1.10E-01	9.66	14.23	4.98E-01	-10.89	4.94	2.76E-02	-7.69	11.70	5.11E-01
cg25704407	-54.33	22.19	1.43E-02	-56.42	29.43	5.52E-02	47.35	92.76	6.10E-01	-16.24	59.33	7.84E-01	-96.78	45.82	3.46E-02
cg20360947	18.43	7.53	1.43E-02	-0.38	24.07	9.87E-01	29.40	29.23	3.15E-01	20.14	8.49	1.77E-02	13.84	33.54	6.80E-01
cg15761609	23.57	9.62	1.43E-02	23.25	12.48	6.24E-02	47.22	34.27	1.68E-01	6.02	39.98	8.80E-01	21.10	18.58	2.56E-01
cg14597281	-25.69	10.49	1.43E-02	-22.65	16.34	1.66E-01	-9.75	29.21	7.39E-01	-43.26	20.45	3.44E-02	-18.97	23.73	4.24E-01
cg11943190	-17.71	7.23	1.43E-02	-12.29	14.41	3.94E-01	-1.00	31.98	9.75E-01	-25.12	9.27	6.71E-03	8.49	24.46	7.29E-01
cg04290563	16.91	6.91	1.43E-02	33.64	13.83	1.50E-02	-11.84	38.64	7.59E-01	13.14	8.82	1.36E-01	7.92	21.31	7.10E-01
cg07807481	20.62	8.42	1.44E-02	34.23	14.33	1.69E-02	14.86	15.91	3.50E-01	-2.41	18.28	8.95E-01	31.69	20.91	1.30E-01
cg22029824	-49.95	20.40	1.44E-02	-73.16	59.85	2.22E-01	212.41	254.66	4.04E-01	-45.09	23.43	5.43E-02	-72.30	59.08	2.21E-01
cg17445007	-31.07	12.69	1.44E-02	-41.32	36.98	2.64E-01	-42.33	130.73	7.46E-01	-35.56	14.13	1.19E-02	43.22	49.24	3.80E-01
cg21512817	24.15	9.87	1.44E-02	38.38	20.72	6.39E-02	23.09	37.48	5.38E-01	17.67	12.76	1.66E-01	30.96	30.31	3.07E-01
cg04745336	-13.18	5.38	1.44E-02	-19.39	10.05	5.37E-02	5.24	15.89	7.41E-01	-18.27	7.40	1.36E-02	0.32	13.70	9.81E-01
cg21609706	-10.30	4.21	1.44E-02	-17.34	9.71	7.41E-02	-16.51	20.52	4.21E-01	-5.60	5.25	2.86E-01	-21.45	11.74	6.78E-02
cg13523125	7.75	3.17	1.44E-02	8.09	4.41	6.63E-02	11.45	7.36	1.20E-01	8.74	8.38	2.97E-01	1.33	8.02	8.69E-01
cg13252730	-8.82	3.60	1.44E-02	-6.10	5.85	2.97E-01	-10.45	7.68	1.74E-01	-9.88	7.39	1.82E-01	-11.38	8.90	2.01E-01
cg22677065	29.68	12.13	1.44E-02	31.67	25.79	2.20E-01	35.91	42.28	3.96E-01	24.05	15.96	1.32E-01	48.94	35.07	1.63E-01
cg13732465	15.42	6.30	1.44E-02	9.20	8.81	2.97E-01	24.96	13.18	5.83E-02	3.60	19.64	8.55E-01	29.49	15.87	6.31E-02
cg25549230	-43.51	17.77	1.44E-02	-44.78	20.72	3.07E-02	-129.54	97.84	1.86E-01	6.14	82.09	9.40E-01	-35.66	41.38	3.89E-01
cg24810917	-10.71	4.38	1.44E-02	-14.06	6.29	2.53E-02	-10.34	10.16	3.09E-01	4.86	13.50	7.19E-01	-11.08	9.23	2.30E-01
cg24090202	22.39	9.15	1.44E-02	9.22	14.21	5.17E-01	11.86	38.05	7.55E-01	69.24	51.93	1.82E-01	31.66	12.98	1.47E-02
cg23900696	-18.53	7.57	1.44E-02	-23.15	18.00	1.98E-01	-10.80	25.37	6.70E-01	-16.43	9.51	8.41E-02	-30.49	23.89	2.02E-01
cg10707565	18.02	7.36	1.44E-02	-1.81	17.29	9.17E-01	5.80	24.52	8.13E-01	26.05	9.15	4.41E-03	11.76	25.91	6.50E-01
cg11739225	47.49	19.40	1.44E-02	46.40	52.45	3.76E-01	-216.60	211.52	3.06E-01	53.94	22.00	1.42E-02	12.99	70.04	8.53E-01
cg10798815	14.26	5.83	1.44E-02	18.71	7.62	1.41E-02	12.50	22.46	5.78E-01	27.28	21.36	2.02E-01	1.64	11.15	8.83E-01
cg22971216	-8.54	3.49	1.44E-02	-9.96	4.30	2.05E-02	-16.72	9.66	8.35E-02	3.83	22.95	8.67E-01	0.63	8.07	9.38E-01
cg06912964	19.61	8.01	1.44E-02	26.30	12.44	3.45E-02	14.47	15.65	3.55E-01	0.73	19.27	9.70E-01	31.83	20.68	1.24E-01

cg02645368	-9.63	3.94	1.44E-02	-7.84	5.48	1.53E-01	-15.76	10.38	1.29E-01	-11.73	9.16	2.00E-01	-7.42	9.94	4.55E-01
cg24278165	-19.22	7.85	1.44E-02	-18.94	12.59	1.32E-01	6.12	39.39	8.77E-01	-19.51	15.97	2.22E-01	-22.40	13.70	1.02E-01
cg13998945	-14.02	5.73	1.44E-02	-18.04	11.08	1.04E-01	4.80	17.27	7.81E-01	-20.60	8.73	1.83E-02	-4.42	13.10	7.36E-01
cg03410903	41.41	16.92	1.44E-02	5.41	50.14	9.14E-01	-154.20	185.32	4.05E-01	46.40	18.81	1.36E-02	66.26	64.79	3.06E-01
cg08690664	20.39	8.33	1.44E-02	24.24	16.81	1.49E-01	24.44	22.14	2.70E-01	15.87	11.46	1.66E-01	30.75	28.77	2.85E-01
cg24366913	60.92	24.90	1.44E-02	69.73	31.87	2.87E-02	-17.59	95.14	8.53E-01	15.51	104.76	8.82E-01	70.63	48.42	1.45E-01
cg10987840	-13.09	5.35	1.44E-02	-26.17	10.74	1.48E-02	-14.60	18.06	4.19E-01	-7.79	7.11	2.73E-01	-9.20	17.09	5.91E-01
cg02687418	-5.41	2.21	1.44E-02	-5.49	3.24	9.00E-02	-10.11	4.82	3.61E-02	0.55	8.79	9.50E-01	-2.92	4.33	5.00E-01
cg05781826	-45.48	18.59	1.44E-02	-57.90	22.49	1.00E-02	-17.27	96.86	8.59E-01	-58.51	56.56	3.01E-01	6.00	44.82	8.94E-01
cg05885688	-11.20	4.58	1.44E-02	-14.22	9.69	1.42E-01	-31.82	18.60	8.70E-02	-6.66	5.97	2.65E-01	-16.93	12.74	1.84E-01
cg08017233	-17.66	7.22	1.44E-02	-35.18	18.34	5.51E-02	24.26	37.80	5.21E-01	-16.48	8.48	5.21E-02	-13.80	24.84	5.79E-01
cg25094081	-21.16	8.65	1.44E-02	-18.16	19.25	3.46E-01	17.55	49.04	7.20E-01	-23.69	10.41	2.29E-02	-22.04	31.26	4.81E-01
cg18704047	-11.10	4.54	1.44E-02	-22.93	8.73	8.59E-03	-7.29	11.78	5.36E-01	-4.58	6.97	5.11E-01	-11.92	11.43	2.97E-01
cg03323092	-47.86	19.56	1.44E-02	-126.84	58.08	2.90E-02	-241.02	239.83	3.15E-01	-36.87	22.23	9.73E-02	-31.41	60.23	6.02E-01
cg00247361	19.76	8.08	1.44E-02	26.33	15.67	9.29E-02	29.87	24.65	2.26E-01	13.91	11.59	2.30E-01	19.84	21.48	3.56E-01
cg14839558	-16.10	6.58	1.44E-02	-13.00	9.11	1.53E-01	-0.43	18.43	9.81E-01	-38.30	15.01	1.07E-02	-12.61	14.73	3.92E-01
cg16996788	12.42	5.08	1.44E-02	12.12	5.10	1.74E-02	2.82	6.84	6.80E-01	31.96	10.15	1.64E-03	9.49	8.54	2.66E-01
cg08601673	10.79	4.41	1.44E-02	11.43	6.33	7.11E-02	16.06	9.97	1.07E-01	17.43	14.52	2.30E-01	2.17	9.27	8.15E-01
cg00882281	-6.63	2.71	1.44E-02	-6.58	5.28	2.13E-01	2.72	11.52	8.14E-01	-9.34	3.80	1.40E-02	-1.74	6.51	7.89E-01
cg03680932	-24.64	10.07	1.45E-02	-31.35	15.07	3.75E-02	-37.26	54.05	4.91E-01	-23.12	16.41	1.59E-01	-4.39	26.77	8.70E-01
cg08632659	-36.18	14.79	1.45E-02	-31.28	36.96	3.97E-01	-16.35	192.94	9.32E-01	-37.75	16.77	2.44E-02	-30.54	62.59	6.26E-01
cg04834436	14.52	5.94	1.45E-02	20.54	9.35	2.81E-02	10.50	15.25	4.91E-01	15.37	13.97	2.71E-01	7.08	11.53	5.39E-01
cg25218152	6.10	2.49	1.45E-02	1.66	3.34	6.19E-01	11.06	5.30	3.68E-02	10.02	4.55	2.79E-02	5.37	7.72	4.87E-01
cg25967745	-38.03	15.55	1.45E-02	-56.98	24.73	2.13E-02	15.35	59.11	7.95E-01	-37.36	27.54	1.75E-01	-21.48	33.43	5.21E-01
cg01089498	13.99	5.72	1.45E-02	21.11	13.90	1.29E-01	20.37	17.64	2.48E-01	13.70	7.98	8.61E-02	5.83	12.43	6.39E-01
cg14592284	21.00	8.59	1.45E-02	-5.70	20.98	7.86E-01	11.24	58.45	8.48E-01	25.23	10.02	1.18E-02	41.51	30.97	1.80E-01
cg06994016	-5.73	2.34	1.45E-02	-6.32	3.30	5.54E-02	-3.22	4.47	4.71E-01	-4.27	9.24	6.44E-01	-8.85	5.93	1.36E-01
cg00099976	17.36	7.10	1.45E-02	24.49	12.24	4.53E-02	4.19	16.42	7.99E-01	17.87	14.75	2.26E-01	17.14	14.36	2.33E-01
cg09423651	-14.39	5.89	1.45E-02	-12.37	16.68	4.58E-01	12.60	28.26	6.56E-01	-16.90	6.80	1.30E-02	-8.93	20.39	6.61E-01
cg09389824	-10.51	4.30	1.45E-02	-9.12	6.69	1.73E-01	-11.80	9.55	2.16E-01	-6.95	10.89	5.23E-01	-14.29	8.98	1.12E-01
cg00555905	11.76	4.81	1.45E-02	16.98	8.82	5.42E-02	16.16	12.28	1.88E-01	9.11	8.60	2.89E-01	5.85	9.88	5.54E-01
cg23082067	-45.65	18.67	1.45E-02	-48.43	23.86	4.24E-02	-22.79	54.43	6.75E-01	-17.27	72.36	8.11E-01	-59.78	41.38	1.49E-01
cg15705062	103.62	42.38	1.45E-02	105.80	58.29	6.95E-02	354.29	237.96	1.37E-01	14.82	115.59	8.98E-01	112.91	76.72	1.41E-01
cg04089901	6.75	2.76	1.45E-02	5.41	3.60	1.33E-01	13.92	6.55	3.36E-02	10.87	8.78	2.16E-01	0.21	7.44	9.78E-01
cg16732970	54.61	22.34	1.45E-02	53.60	50.67	2.90E-01	133.68	191.58	4.85E-01	65.52	26.88	1.48E-02	-28.44	70.19	6.85E-01

cg01597373	-10.39	4.25	1.45E-02	-6.19	5.91	2.95E-01	-9.35	11.70	4.24E-01	-13.45	14.13	3.41E-01	-18.18	8.32	2.90E-02
cg04738700	22.56	9.23	1.45E-02	34.72	17.26	4.42E-02	6.70	17.50	7.02E-01	24.66	16.74	1.41E-01	24.81	25.41	3.29E-01
cg01659184	-14.76	6.04	1.45E-02	-15.52	10.99	1.58E-01	-15.58	12.16	2.00E-01	-6.52	11.65	5.76E-01	-24.46	14.10	8.27E-02
cg02454536	8.54	3.49	1.45E-02	7.85	5.59	1.61E-01	15.24	6.85	2.61E-02	0.67	7.50	9.29E-01	10.34	9.60	2.81E-01
cg16900796	-9.44	3.86	1.45E-02	-12.11	7.10	8.80E-02	-0.62	11.93	9.59E-01	-8.92	5.97	1.35E-01	-11.40	9.09	2.10E-01
cg26193015	11.50	4.70	1.45E-02	6.07	9.15	5.07E-01	27.71	10.39	7.64E-03	7.79	5.73	1.74E-01	17.95	25.80	4.87E-01
cg13692446	13.10	5.36	1.45E-02	23.61	15.05	1.17E-01	33.56	25.24	1.84E-01	8.79	6.41	1.70E-01	18.99	14.93	2.03E-01
cg04162153	-11.02	4.51	1.45E-02	-5.65	9.57	5.55E-01	-12.45	10.38	2.31E-01	-12.48	6.67	6.14E-02	-13.00	12.40	2.94E-01
cg05402319	-12.29	5.03	1.45E-02	-15.46	7.46	3.82E-02	8.70	13.46	5.18E-01	-16.24	12.60	1.97E-01	-15.76	10.12	1.19E-01
cg13273396	15.18	6.21	1.45E-02	10.99	8.72	2.07E-01	44.10	28.03	1.16E-01	21.79	26.00	4.02E-01	16.02	9.98	1.09E-01
cg12084925	25.66	10.50	1.45E-02	25.05	19.86	2.07E-01	33.33	22.75	1.43E-01	30.39	17.07	7.51E-02	0.59	29.15	9.84E-01
cg16151082	18.46	7.55	1.45E-02	30.24	14.94	4.29E-02	-2.67	17.30	8.77E-01	23.73	12.05	4.89E-02	11.90	18.86	5.28E-01
cg19269511	17.67	7.23	1.45E-02	14.91	11.46	1.93E-01	10.99	18.78	5.58E-01	12.72	16.75	4.47E-01	28.94	13.98	3.85E-02
cg09123307	20.49	8.38	1.45E-02	24.75	22.99	2.82E-01	10.39	55.25	8.51E-01	20.93	9.58	2.90E-02	11.95	29.92	6.90E-01
cg16206324	32.87	13.45	1.45E-02	30.35	19.80	1.25E-01	105.62	44.19	1.68E-02	16.93	11.57	1.43E-01	49.22	31.31	1.16E-01
cg06767191	17.96	7.35	1.45E-02	39.98	19.74	4.28E-02	32.02	28.48	2.61E-01	15.25	8.56	7.48E-02	-16.63	30.63	5.87E-01
cg02051616	12.84	5.25	1.45E-02	18.13	8.88	4.12E-02	11.05	14.85	4.57E-01	12.04	9.97	2.27E-01	7.15	10.58	4.99E-01
cg05483021	-14.71	6.02	1.45E-02	-15.32	8.01	5.58E-02	-8.19	15.87	6.06E-01	4.85	28.79	8.66E-01	-20.58	12.11	8.91E-02
cg17755321	-12.24	5.01	1.45E-02	-11.00	14.76	4.56E-01	-46.35	33.14	1.62E-01	-10.58	5.77	6.67E-02	-18.02	15.28	2.38E-01
cg02084087	22.15	9.06	1.45E-02	23.00	11.92	5.36E-02	46.60	31.61	1.40E-01	19.09	62.68	7.61E-01	14.49	16.06	3.67E-01
cg27272853	-52.92	21.65	1.45E-02	-66.03	29.43	2.48E-02	70.39	92.14	4.45E-01	-62.08	53.05	2.42E-01	-45.25	44.52	3.09E-01
cg24803517	8.90	3.64	1.45E-02	3.86	6.83	5.71E-01	10.98	8.32	1.87E-01	14.91	5.97	1.25E-02	0.98	9.33	9.16E-01
cg04202511	7.54	3.09	1.45E-02	6.07	3.94	1.24E-01	17.78	7.98	2.58E-02	5.42	9.23	5.57E-01	4.42	8.74	6.13E-01
cg21044433	18.91	7.74	1.46E-02	15.76	12.04	1.90E-01	31.80	15.78	4.39E-02	11.38	17.64	5.19E-01	16.65	19.74	3.99E-01
cg04554505	-8.44	3.45	1.46E-02	-16.10	7.04	2.21E-02	-5.76	11.35	6.12E-01	-7.60	4.65	1.02E-01	1.46	10.20	8.86E-01
cg22628500	39.36	16.11	1.46E-02	43.16	19.40	2.61E-02	9.66	75.68	8.98E-01	37.44	52.13	4.73E-01	32.96	39.12	4.00E-01
cg03208426	24.13	9.88	1.46E-02	35.92	16.28	2.74E-02	26.67	33.06	4.20E-01	18.81	17.95	2.95E-01	11.81	20.17	5.58E-01
cg21088438	6.09	2.49	1.46E-02	3.74	3.36	2.65E-01	9.78	6.39	1.26E-01	14.46	6.42	2.42E-02	2.44	6.51	7.08E-01
cg18207676	23.41	9.58	1.46E-02	29.61	22.36	1.85E-01	28.54	23.55	2.26E-01	20.63	13.03	1.13E-01	19.00	28.93	5.11E-01
cg05954830	5.26	2.15	1.46E-02	5.69	3.13	6.85E-02	10.10	5.63	7.28E-02	4.93	4.09	2.28E-01	-2.77	6.73	6.80E-01
cg10342963	6.00	2.46	1.46E-02	8.00	3.52	2.31E-02	6.86	4.19	1.02E-01	4.91	11.30	6.64E-01	-4.00	7.05	5.70E-01
cg23390619	9.58	3.92	1.46E-02	10.15	5.48	6.41E-02	15.96	9.07	7.85E-02	5.97	10.99	5.87E-01	3.69	9.41	6.95E-01
cg00415971	-12.01	4.91	1.46E-02	-13.26	6.93	5.58E-02	-11.61	10.88	2.86E-01	-14.09	15.40	3.60E-01	-8.03	11.23	4.75E-01
cg19990316	-9.85	4.03	1.46E-02	-9.11	6.26	1.45E-01	-6.07	8.84	4.92E-01	-14.35	10.92	1.89E-01	-11.83	8.22	1.50E-01
cg14927855	3.54	1.45	1.46E-02	5.27	1.96	7.32E-03	3.55	3.64	3.30E-01	2.68	3.94	4.97E-01	-1.51	3.57	6.73E-01

cg24942226	19.15	7.84	1.46E-02	23.15	14.79	1.18E-01	-16.54	31.96	6.05E-01	19.75	10.57	6.16E-02	25.56	23.82	2.83E-01
cg10374564	-11.71	4.79	1.46E-02	-21.44	12.12	7.68E-02	-34.57	32.09	2.81E-01	-10.26	5.53	6.36E-02	1.84	18.20	9.19E-01
cg00502254	12.02	4.92	1.46E-02	14.04	6.46	2.98E-02	6.29	9.94	5.27E-01	32.24	14.68	2.81E-02	1.07	11.55	9.26E-01
cg24850711	-7.39	3.03	1.46E-02	-3.92	5.37	4.66E-01	-7.11	8.23	3.87E-01	-10.48	5.15	4.18E-02	-7.74	6.73	2.50E-01
cg07080358	19.61	8.03	1.46E-02	10.36	29.16	7.22E-01	-17.40	73.61	8.13E-01	19.39	8.86	2.86E-02	34.19	26.69	2.00E-01
cg00151607	40.98	16.78	1.46E-02	-2.32	46.34	9.60E-01	-248.34	218.02	2.55E-01	48.13	19.15	1.20E-02	61.00	54.48	2.63E-01
cg20326117	20.24	8.29	1.46E-02	44.20	24.08	6.64E-02	-12.06	43.46	7.81E-01	16.88	9.33	7.05E-02	37.75	34.99	2.81E-01
cg24137448	-9.74	3.99	1.46E-02	-11.43	6.21	6.55E-02	-15.12	11.24	1.79E-01	-10.01	7.78	1.98E-01	-2.42	8.99	7.88E-01
cg14275880	14.41	5.90	1.46E-02	7.55	10.49	4.72E-01	12.03	15.37	4.34E-01	15.85	9.41	9.19E-02	28.23	15.68	7.18E-02
cg24359323	15.83	6.48	1.46E-02	-10.49	30.09	7.27E-01	67.31	76.70	3.80E-01	18.77	6.91	6.60E-03	-10.14	25.04	6.85E-01
cg12727398	14.20	5.82	1.46E-02	19.20	8.35	2.15E-02	-2.92	14.38	8.39E-01	22.69	26.63	3.94E-01	14.10	10.55	1.82E-01
cg00308914	14.15	5.79	1.46E-02	17.37	7.49	2.04E-02	14.60	15.89	3.58E-01	8.57	20.53	6.76E-01	5.99	13.32	6.53E-01
cg10426076	5.74	2.35	1.46E-02	5.00	3.43	1.45E-01	7.33	5.75	2.02E-01	8.35	4.86	8.58E-02	1.64	6.55	8.03E-01
cg11114873	28.65	11.73	1.46E-02	38.36	25.55	1.33E-01	-15.20	45.51	7.38E-01	27.56	15.95	8.39E-02	36.68	27.58	1.84E-01
cg17314700	8.86	3.63	1.46E-02	10.27	4.93	3.72E-02	-1.25	8.32	8.80E-01	11.57	13.57	3.94E-01	13.79	8.20	9.25E-02
cg16830966	70.45	28.86	1.46E-02	0.13	66.30	9.98E-01	-269.77	281.36	3.38E-01	94.77	31.53	2.65E-03	70.21	73.99	3.43E-01
cg15736994	9.60	3.93	1.46E-02	7.72	6.02	2.00E-01	11.63	7.70	1.31E-01	14.36	11.56	2.14E-01	8.22	8.89	3.55E-01
cg06943667	32.46	13.30	1.46E-02	30.21	16.59	6.87E-02	30.25	50.53	5.49E-01	74.24	42.42	8.01E-02	19.28	30.48	5.27E-01
cg08408453	9.83	4.03	1.47E-02	8.15	5.77	1.58E-01	4.01	8.99	6.56E-01	2.79	19.44	8.86E-01	18.34	7.76	1.81E-02
cg11767757	-8.18	3.35	1.47E-02	-10.98	4.48	1.43E-02	-4.03	8.69	6.43E-01	-10.82	9.59	2.59E-01	-0.67	8.14	9.34E-01
cg13601496	29.55	12.11	1.47E-02	28.29	19.37	1.44E-01	6.82	33.83	8.40E-01	63.44	28.37	2.53E-02	20.27	22.14	3.60E-01
cg08026618	34.37	14.08	1.47E-02	55.43	22.08	1.21E-02	43.56	48.48	3.69E-01	43.22	39.75	2.77E-01	7.11	22.74	7.54E-01
cg22902505	7.00	2.87	1.47E-02	6.70	4.05	9.86E-02	2.84	6.56	6.65E-01	14.35	7.49	5.52E-02	6.17	7.13	3.86E-01
cg15393051	-41.07	16.83	1.47E-02	-30.83	20.52	1.33E-01	-105.15	51.35	4.06E-02	-32.43	88.19	7.13E-01	-42.82	39.30	2.76E-01
cg15434398	-13.73	5.62	1.47E-02	0.39	13.36	9.77E-01	-14.48	23.37	5.36E-01	-17.43	6.93	1.19E-02	-13.92	17.28	4.20E-01
cg26036479	-39.83	16.32	1.47E-02	-28.27	50.41	5.75E-01	-155.20	185.24	4.02E-01	-39.93	18.45	3.04E-02	-42.07	50.40	4.04E-01
cg06914817	17.40	7.13	1.47E-02	36.80	13.92	8.18E-03	9.25	16.61	5.78E-01	11.77	10.53	2.64E-01	6.71	23.22	7.73E-01
cg12687125	12.20	5.00	1.47E-02	8.18	11.21	4.65E-01	-1.26	20.85	9.52E-01	12.34	6.27	4.89E-02	26.12	15.30	8.78E-02
cg11857033	23.52	9.64	1.47E-02	32.42	11.29	4.08E-03	43.26	25.99	9.60E-02	29.01	19.86	1.44E-01	-2.13	15.45	8.91E-01
cg10053879	20.68	8.47	1.47E-02	24.73	13.22	6.13E-02	23.59	16.57	1.54E-01	25.69	19.12	1.79E-01	-5.42	23.43	8.17E-01
cg24399830	20.01	8.20	1.47E-02	15.23	10.59	1.51E-01	65.38	28.99	2.41E-02	28.15	40.71	4.89E-01	16.10	15.50	2.99E-01
cg23495748	15.83	6.49	1.47E-02	39.32	17.72	2.65E-02	22.06	28.22	4.34E-01	10.35	8.03	1.98E-01	16.44	16.17	3.10E-01
cg16537196	-34.49	14.13	1.47E-02	-33.03	17.43	5.81E-02	-2.78	67.78	9.67E-01	-48.52	42.91	2.58E-01	-38.76	32.36	2.31E-01
cg00210271	13.17	5.40	1.47E-02	9.25	14.14	5.13E-01	42.19	38.07	2.68E-01	13.51	6.92	5.08E-02	12.20	11.36	2.83E-01
cg11747820	-12.97	5.32	1.47E-02	-22.84	11.66	5.02E-02	-33.75	27.66	2.22E-01	-9.37	6.50	1.49E-01	-8.26	18.19	6.50E-01

cg18555433	-13.64	5.59	1.47E-02	-20.72	7.89	8.60E-03	-5.05	13.27	7.03E-01	-7.02	15.30	6.46E-01	-7.46	12.94	5.64E-01
cg16284168	-34.36	14.09	1.47E-02	-42.51	17.56	1.55E-02	-16.15	69.33	8.16E-01	-22.66	73.58	7.58E-01	-19.79	26.67	4.58E-01
cg07906855	11.14	4.57	1.47E-02	4.90	6.92	4.79E-01	20.04	8.74	2.18E-02	21.75	11.17	5.15E-02	4.24	9.24	6.46E-01
cg09506081	4.61	1.89	1.47E-02	4.14	2.61	1.13E-01	2.52	4.61	5.85E-01	10.75	5.62	5.55E-02	4.11	4.29	3.38E-01
cg27440715	31.39	12.87	1.47E-02	51.12	16.67	2.16E-03	-6.33	29.14	8.28E-01	44.02	35.48	2.15E-01	18.33	22.54	4.16E-01
cg06738602	13.90	5.70	1.47E-02	19.53	8.59	2.29E-02	21.92	14.87	1.40E-01	-0.24	17.16	9.89E-01	6.96	10.35	5.01E-01
cg21163717	-9.00	3.69	1.47E-02	-13.24	4.82	6.00E-03	-6.48	8.50	4.45E-01	-0.12	10.27	9.91E-01	0.03	11.94	9.98E-01
cg05476182	19.67	8.06	1.47E-02	10.92	13.83	4.30E-01	35.24	14.63	1.60E-02	21.99	18.28	2.29E-01	5.99	20.08	7.65E-01
cg17191178	31.81	13.04	1.47E-02	30.84	16.44	6.07E-02	63.70	52.20	2.22E-01	28.17	70.24	6.88E-01	27.23	24.93	2.75E-01
cg05180985	30.39	12.46	1.47E-02	45.86	20.32	2.40E-02	34.31	33.15	3.01E-01	10.11	32.65	7.57E-01	20.25	21.47	3.46E-01
cg08855671	23.48	9.63	1.47E-02	26.62	17.13	1.20E-01	23.40	18.99	2.18E-01	13.81	17.71	4.36E-01	37.82	26.53	1.54E-01
cg08058544	17.21	7.05	1.47E-02	25.07	31.15	4.21E-01	50.14	97.64	6.08E-01	16.23	7.49	3.02E-02	22.24	29.57	4.52E-01
cg12556960	30.70	12.59	1.47E-02	72.31	27.60	8.80E-03	12.32	85.95	8.86E-01	24.75	11.80	3.60E-02	-4.04	40.33	9.20E-01
cg26572165	-32.18	13.20	1.47E-02	-35.11	19.00	6.46E-02	-69.67	36.14	5.39E-02	11.90	37.39	7.50E-01	-28.64	25.89	2.69E-01
cg09818588	11.65	4.78	1.47E-02	11.22	7.84	1.53E-01	1.93	11.20	8.63E-01	11.43	9.16	2.12E-01	23.10	11.44	4.35E-02
cg07640645	19.30	7.91	1.47E-02	37.67	19.68	5.56E-02	-18.71	97.76	8.48E-01	15.06	9.01	9.47E-02	28.25	32.10	3.79E-01
cg04861263	-23.57	9.67	1.47E-02	-29.25	24.55	2.34E-01	-45.65	107.51	6.71E-01	-22.50	11.19	4.44E-02	-20.70	31.99	5.18E-01
cg13883851	11.94	4.90	1.47E-02	14.05	6.70	3.60E-02	5.88	11.25	6.01E-01	6.87	17.74	6.99E-01	13.94	10.92	2.01E-01
cg10790791	20.01	8.21	1.47E-02	29.92	10.76	5.42E-03	14.29	25.06	5.68E-01	14.02	25.44	5.82E-01	-1.84	18.02	9.19E-01
cg08179353	-8.59	3.52	1.47E-02	-10.12	5.82	8.22E-02	-10.50	10.01	2.94E-01	-7.97	5.83	1.71E-01	-4.65	9.26	6.16E-01
cg10935723	-5.86	2.40	1.48E-02	-6.93	4.34	1.10E-01	-5.14	5.68	3.65E-01	-2.78	3.81	4.65E-01	-14.77	7.08	3.69E-02
cg05699739	6.87	2.82	1.48E-02	5.22	3.97	1.89E-01	8.32	8.83	3.46E-01	12.69	5.74	2.69E-02	2.16	7.23	7.65E-01
cg14769021	-14.58	5.98	1.48E-02	-20.80	8.89	1.94E-02	9.57	15.33	5.33E-01	-22.09	12.87	8.61E-02	-13.12	10.72	2.21E-01
cg19863595	-7.88	3.23	1.48E-02	-7.20	4.36	9.89E-02	-9.74	6.75	1.49E-01	0.22	10.75	9.84E-01	-12.98	8.89	1.44E-01
cg13283635	8.55	3.51	1.48E-02	5.80	4.51	1.99E-01	8.14	7.98	3.08E-01	15.39	13.08	2.39E-01	18.07	9.68	6.19E-02
cg20234007	11.77	4.83	1.48E-02	7.85	7.22	2.77E-01	14.48	10.95	1.86E-01	16.71	12.11	1.68E-01	13.98	10.81	1.96E-01
cg03627957	-28.04	11.50	1.48E-02	-42.95	16.76	1.04E-02	-40.12	46.31	3.86E-01	9.86	30.88	7.49E-01	-20.41	20.06	3.09E-01
cg13971140	-32.77	13.44	1.48E-02	-37.78	17.10	2.72E-02	-29.79	59.91	6.19E-01	-49.70	41.13	2.27E-01	-11.67	28.33	6.80E-01
cg11617821	-8.86	3.64	1.48E-02	-14.41	7.39	5.11E-02	-5.15	11.53	6.55E-01	-7.07	5.05	1.62E-01	-8.54	9.70	3.79E-01
cg08736883	-7.93	3.25	1.48E-02	-5.54	5.26	2.93E-01	-6.96	6.64	2.95E-01	-6.74	7.34	3.58E-01	-15.55	7.64	4.18E-02
cg16102240	-8.87	3.64	1.48E-02	-7.55	4.87	1.21E-01	-9.03	9.11	3.22E-01	1.71	13.19	8.97E-01	-16.30	8.04	4.26E-02
cg26090062	-5.37	2.20	1.48E-02	-5.98	3.19	6.07E-02	-8.84	4.99	7.63E-02	-9.56	6.84	1.62E-01	0.91	4.67	8.45E-01
cg23779604	-3.87	1.59	1.48E-02	-4.24	1.96	3.06E-02	-1.92	2.94	5.13E-01	2.77	5.79	6.32E-01	-8.44	3.64	2.06E-02
cg18175779	-19.46	7.98	1.48E-02	-19.74	13.21	1.35E-01	-15.09	21.11	4.75E-01	-16.61	13.98	2.35E-01	-28.18	19.59	1.50E-01
cg22587600	5.83	2.39	1.48E-02	5.47	3.89	1.59E-01	6.54	5.13	2.03E-01	4.98	4.73	2.93E-01	7.16	6.20	2.48E-01

cg22875527	-16.22	6.65	1.48E-02	-24.56	13.44	6.77E-02	-6.04	14.93	6.86E-01	-16.00	10.17	1.16E-01	-16.79	18.60	3.67E-01
cg21188154	25.31	10.39	1.48E-02	19.20	14.10	1.73E-01	28.11	29.07	3.34E-01	79.40	33.49	1.77E-02	15.77	21.36	4.60E-01
cg17680611	-8.60	3.53	1.48E-02	-7.94	4.98	1.11E-01	-12.04	7.96	1.30E-01	-8.09	9.57	3.98E-01	-6.96	8.68	4.23E-01
cg15422147	-18.77	7.70	1.48E-02	-11.95	15.05	4.27E-01	-29.32	17.08	8.60E-02	-23.14	13.44	8.51E-02	-10.08	16.92	5.51E-01
cg08594589	17.26	7.08	1.48E-02	24.02	10.82	2.64E-02	13.34	13.25	3.14E-01	16.28	19.45	4.03E-01	6.48	18.12	7.21E-01
cg16877339	-21.81	8.95	1.48E-02	-12.20	13.27	3.58E-01	-31.65	23.15	1.71E-01	-28.21	27.66	3.08E-01	-29.51	16.60	7.56E-02
cg00192468	-22.32	9.16	1.48E-02	-25.17	19.55	1.98E-01	-32.58	39.73	4.12E-01	-18.55	11.63	1.11E-01	-33.18	27.94	2.35E-01
cg02886806	-14.45	5.93	1.48E-02	-17.36	13.85	2.10E-01	-4.29	22.74	8.50E-01	-17.48	7.38	1.78E-02	3.04	18.47	8.69E-01
cg22176895	20.59	8.45	1.48E-02	25.49	13.24	5.41E-02	15.20	23.26	5.13E-01	15.99	23.77	5.01E-01	18.48	14.62	2.06E-01
cg01768328	20.20	8.29	1.48E-02	27.67	20.09	1.68E-01	12.55	21.83	5.65E-01	17.50	10.64	9.99E-02	38.85	29.58	1.89E-01
cg09590852	15.49	6.36	1.48E-02	15.71	9.15	8.60E-02	25.22	10.95	2.12E-02	5.49	30.34	8.56E-01	-6.17	17.22	7.20E-01
cg13574150	-11.74	4.82	1.48E-02	-7.73	11.49	5.01E-01	9.57	19.47	6.23E-01	-13.59	5.88	2.09E-02	-20.15	15.94	2.06E-01
cg22896991	16.53	6.79	1.48E-02	33.01	16.34	4.34E-02	94.75	82.03	2.48E-01	12.74	7.80	1.02E-01	8.61	26.71	7.47E-01
cg25492364	23.64	9.70	1.48E-02	19.51	14.20	1.70E-01	51.26	22.34	2.18E-02	9.93	30.73	7.47E-01	15.81	19.60	4.20E-01
cg01648999	24.89	10.22	1.48E-02	18.14	9.53	5.68E-02	59.20	17.36	6.49E-04	27.79	21.42	1.95E-01	7.53	12.01	5.31E-01
cg27257926	15.96	6.55	1.48E-02	25.82	11.99	3.13E-02	3.65	17.04	8.30E-01	9.55	9.91	3.35E-01	30.31	19.18	1.14E-01
cg15808604	12.91	5.30	1.49E-02	22.32	11.18	4.58E-02	3.92	23.57	8.68E-01	9.24	6.59	1.61E-01	22.02	18.97	2.46E-01
cg07693569	-61.95	25.43	1.49E-02	-42.59	37.17	2.52E-01	46.30	111.27	6.77E-01	-111.43	49.97	2.58E-02	-70.59	54.16	1.92E-01
cg15357118	13.16	5.40	1.49E-02	16.76	8.34	4.45E-02	17.14	12.60	1.74E-01	20.91	13.08	1.10E-01	-2.65	11.39	8.16E-01
cg00908117	-17.82	7.32	1.49E-02	-43.83	20.47	3.23E-02	4.85	41.76	9.08E-01	-15.91	8.36	5.69E-02	-2.33	26.76	9.31E-01
cg00958955	28.16	11.56	1.49E-02	12.05	21.98	5.84E-01	22.43	46.72	6.31E-01	43.76	16.20	6.92E-03	7.67	29.57	7.95E-01
cg05620095	16.09	6.60	1.49E-02	14.42	14.44	3.18E-01	-2.55	19.49	8.96E-01	18.80	8.80	3.27E-02	24.59	19.66	2.11E-01
cg15065877	-8.81	3.62	1.49E-02	-13.22	9.30	1.55E-01	7.94	16.80	6.36E-01	-9.75	4.35	2.49E-02	-3.92	10.85	7.18E-01
cg17405031	8.55	3.51	1.49E-02	7.36	7.36	3.18E-01	17.61	12.92	1.73E-01	11.22	4.72	1.75E-02	-4.33	9.21	6.38E-01
cg02565196	14.25	5.85	1.49E-02	11.84	8.50	1.64E-01	11.74	12.23	3.37E-01	27.17	13.54	4.48E-02	7.98	17.54	6.49E-01
cg09577317	-11.72	4.81	1.49E-02	-8.17	13.03	5.31E-01	-6.90	18.86	7.14E-01	-11.75	5.67	3.83E-02	-21.71	17.22	2.08E-01
cg27524460	-21.27	8.74	1.49E-02	-14.46	12.37	2.42E-01	-20.89	21.01	3.20E-01	-8.72	14.78	5.55E-01	-49.86	17.50	4.38E-03
cg17895511	15.03	6.17	1.49E-02	17.94	15.79	2.56E-01	48.47	26.83	7.08E-02	14.24	7.25	4.94E-02	-8.69	23.42	7.11E-01
cg03718677	-27.81	11.42	1.49E-02	-31.25	24.54	2.03E-01	-60.18	45.15	1.83E-01	-28.70	14.56	4.88E-02	4.42	35.33	9.00E-01
cg04663057	13.38	5.50	1.49E-02	8.68	8.94	3.32E-01	27.01	14.59	6.41E-02	21.92	9.90	2.67E-02	0.30	11.85	9.80E-01
cg03963919	-7.76	3.19	1.49E-02	-12.18	5.39	2.40E-02	1.89	8.94	8.32E-01	-6.22	4.96	2.11E-01	-10.68	9.56	2.64E-01
cg16341159	17.95	7.37	1.49E-02	22.67	10.16	2.57E-02	10.57	21.98	6.31E-01	-5.17	27.70	8.52E-01	17.89	13.68	1.91E-01
cg24874350	14.85	6.10	1.49E-02	19.73	9.54	3.87E-02	17.79	15.22	2.43E-01	10.69	17.33	5.37E-01	8.49	11.01	4.40E-01
cg12130328	25.72	10.56	1.49E-02	41.25	15.63	8.30E-03	-3.87	32.42	9.05E-01	15.12	31.24	6.28E-01	17.21	18.59	3.55E-01
cg17164016	-7.80	3.20	1.49E-02	-9.25	6.57	1.59E-01	7.33	13.74	5.94E-01	-8.89	4.19	3.39E-02	-6.52	9.13	4.75E-01

cg22533317	10.46	4.30	1.49E-02	8.86	5.78	1.25E-01	11.46	9.80	2.42E-01	3.05	16.40	8.53E-01	16.92	9.96	8.94E-02
cg03595538	39.49	16.22	1.49E-02	32.41	44.02	4.62E-01	71.68	143.91	6.18E-01	47.85	18.76	1.08E-02	-15.35	50.32	7.60E-01
cg03895857	7.34	3.01	1.49E-02	8.22	4.99	9.96E-02	6.76	5.79	2.43E-01	6.60	6.75	3.29E-01	7.24	7.44	3.30E-01
cg15202134	35.41	14.55	1.49E-02	60.84	18.15	8.01E-04	75.60	34.07	2.65E-02	19.97	9.49	3.53E-02	5.08	24.45	8.36E-01
cg12705352	28.10	11.54	1.49E-02	32.09	14.48	2.67E-02	-40.76	102.21	6.90E-01	-2.30	41.04	9.55E-01	30.85	22.13	1.63E-01
cg26014373	14.62	6.01	1.49E-02	16.18	17.87	3.65E-01	34.06	33.95	3.16E-01	13.56	6.73	4.38E-02	15.71	25.00	5.30E-01
cg07145683	39.87	16.38	1.49E-02	30.61	16.44	6.26E-02	107.34	39.65	6.78E-03	2.82	38.70	9.42E-01	40.77	28.78	1.57E-01
cg16152136	-12.87	5.29	1.49E-02	-10.57	12.04	3.80E-01	-20.73	25.29	4.12E-01	-14.72	6.52	2.39E-02	-2.22	16.29	8.91E-01
cg16168509	-10.53	4.33	1.50E-02	-13.97	9.75	1.52E-01	14.43	20.72	4.86E-01	-9.46	5.32	7.55E-02	-21.88	13.78	1.12E-01
cg08965655	-14.59	6.00	1.50E-02	-17.30	9.33	6.38E-02	-11.93	13.55	3.79E-01	-5.83	18.34	7.50E-01	-15.78	11.24	1.60E-01
cg21871883	15.54	6.39	1.50E-02	15.20	7.47	4.18E-02	28.70	24.93	2.50E-01	26.77	31.82	4.00E-01	8.99	15.84	5.70E-01
cg18513123	-42.10	17.30	1.50E-02	29.59	52.90	5.76E-01	-112.71	240.64	6.40E-01	-53.40	19.64	6.54E-03	-28.96	51.82	5.76E-01
cg16067628	12.16	5.00	1.50E-02	9.62	7.17	1.80E-01	23.45	10.41	2.42E-02	-4.46	15.78	7.77E-01	13.77	11.65	2.37E-01
cg22012156	-13.57	5.58	1.50E-02	-19.48	11.54	9.12E-02	-8.55	15.16	5.73E-01	-13.36	8.21	1.04E-01	-10.00	13.54	4.60E-01
cg25657752	24.75	10.17	1.50E-02	46.29	21.66	3.26E-02	15.79	25.05	5.29E-01	21.54	14.95	1.50E-01	12.93	26.15	6.21E-01
cg11672390	16.63	6.84	1.50E-02	21.44	14.19	1.31E-01	24.69	15.51	1.11E-01	11.57	9.88	2.42E-01	13.91	22.18	5.30E-01
cg25547580	15.16	6.23	1.50E-02	37.18	11.96	1.87E-03	14.62	14.00	2.96E-01	10.55	6.65	1.12E-01	6.11	9.39	5.16E-01
cg24701309	22.12	9.09	1.50E-02	28.46	17.84	1.11E-01	13.64	18.96	4.72E-01	19.02	15.00	2.05E-01	32.21	24.05	1.81E-01
cg27576485	6.22	2.55	1.50E-02	5.58	3.97	1.60E-01	7.68	4.20	6.77E-02	4.79	9.67	6.21E-01	5.00	6.66	4.52E-01
cg25663741	27.28	11.21	1.50E-02	5.99	17.19	7.28E-01	27.53	48.42	5.70E-01	31.95	13.63	1.91E-02	69.38	31.45	2.74E-02
cg13874040	26.36	10.84	1.50E-02	54.86	21.57	1.10E-02	32.74	31.01	2.91E-01	12.44	9.53	1.92E-01	39.67	30.30	1.90E-01
cg16178907	-9.43	3.88	1.50E-02	-20.23	11.04	6.68E-02	-32.57	20.44	1.11E-01	-7.45	4.52	9.91E-02	-2.64	12.03	8.26E-01
cg24926042	-15.22	6.26	1.50E-02	-21.65	9.52	2.29E-02	-26.64	14.68	6.97E-02	3.05	18.15	8.66E-01	-5.20	12.09	6.67E-01
cg06696028	26.78	11.01	1.50E-02	42.92	23.28	6.52E-02	31.84	27.85	2.53E-01	12.81	8.67	1.40E-01	63.28	29.47	3.18E-02
cg25520040	15.36	6.32	1.50E-02	18.66	11.69	1.10E-01	6.24	19.15	7.45E-01	16.18	9.52	8.93E-02	13.30	15.83	4.01E-01
cg03457984	17.44	7.17	1.50E-02	-0.79	18.45	9.66E-01	20.82	24.48	3.95E-01	22.22	8.67	1.04E-02	7.28	25.41	7.75E-01
cg19981409	-7.20	2.96	1.50E-02	-8.68	4.19	3.81E-02	-6.79	7.60	3.72E-01	-2.54	7.09	7.20E-01	-7.94	7.07	2.62E-01
cg24047665	11.99	4.93	1.50E-02	6.61	7.06	3.49E-01	18.23	9.44	5.36E-02	15.33	15.42	3.20E-01	16.15	13.25	2.23E-01
cg09508448	21.49	8.84	1.50E-02	17.35	16.19	2.84E-01	30.27	16.95	7.42E-02	18.04	16.01	2.60E-01	20.66	24.86	4.06E-01
cg17250812	-13.46	5.53	1.50E-02	-7.60	11.55	5.11E-01	-16.63	19.24	3.88E-01	-18.42	7.25	1.11E-02	3.76	17.08	8.26E-01
cg03575041	14.29	5.88	1.50E-02	22.57	9.35	1.57E-02	6.99	11.88	5.56E-01	8.19	13.44	5.42E-01	12.39	14.30	3.86E-01
cg01818648	32.72	13.45	1.50E-02	16.85	48.11	7.26E-01	62.43	190.67	7.43E-01	36.13	14.52	1.28E-02	1.50	55.41	9.78E-01
cg12173558	18.67	7.68	1.50E-02	15.20	11.07	1.70E-01	18.57	22.90	4.17E-01	22.75	16.04	1.56E-01	22.89	18.21	2.09E-01
cg25881344	33.39	13.73	1.50E-02	28.46	21.13	1.78E-01	-25.39	66.27	7.02E-01	35.76	20.38	7.93E-02	77.02	48.27	1.11E-01
cg06458469	-33.17	13.64	1.50E-02	-39.07	36.45	2.84E-01	-70.77	72.02	3.26E-01	-36.01	15.89	2.35E-02	15.58	46.11	7.35E-01

cg03857664	-11.40	4.69	1.50E-02	-18.38	8.82	3.72E-02	-9.89	12.03	4.11E-01	-5.00	7.79	5.21E-01	-14.20	10.37	1.71E-01
cg15260951	-15.19	6.25	1.50E-02	-15.91	9.33	8.83E-02	-11.97	23.16	6.05E-01	-35.13	14.75	1.72E-02	-2.98	11.41	7.94E-01
cg19478558	-37.94	15.60	1.50E-02	-31.84	22.60	1.59E-01	-7.71	45.30	8.65E-01	-27.42	31.42	3.83E-01	-95.29	39.21	1.51E-02
cg00581103	25.04	10.30	1.50E-02	21.82	16.27	1.80E-01	3.95	21.60	8.55E-01	24.89	23.83	2.96E-01	57.88	23.88	1.54E-02
cg27049886	50.19	20.64	1.50E-02	58.14	24.55	1.79E-02	-105.39	118.45	3.74E-01	87.07	89.54	3.31E-01	36.58	45.05	4.17E-01
cg01729739	27.16	11.17	1.50E-02	50.97	23.85	3.26E-02	-0.71	22.63	9.75E-01	23.26	17.60	1.86E-01	47.02	28.32	9.68E-02
cg15197050	-12.22	5.03	1.50E-02	-21.47	12.80	9.33E-02	-30.52	28.51	2.84E-01	-11.32	5.85	5.29E-02	5.21	18.17	7.74E-01
cg09611599	4.08	1.68	1.50E-02	2.23	2.38	3.50E-01	4.67	3.42	1.72E-01	4.77	6.35	4.53E-01	7.84	3.81	3.97E-02
cg13916117	-4.87	2.00	1.50E-02	-5.80	2.95	4.95E-02	-2.28	4.46	6.09E-01	-7.78	6.04	1.97E-01	-3.87	4.18	3.54E-01
cg01541846	-14.94	6.14	1.50E-02	-28.15	16.19	8.20E-02	-20.72	21.09	3.26E-01	-9.83	7.36	1.82E-01	-30.36	22.43	1.76E-01
cg03874868	38.94	16.02	1.51E-02	37.47	25.18	1.37E-01	70.83	46.09	1.24E-01	9.77	33.63	7.71E-01	52.49	32.17	1.03E-01
cg01979171	39.23	16.14	1.51E-02	29.14	22.26	1.90E-01	71.51	43.91	1.03E-01	17.43	12.99	1.80E-01	90.35	32.98	6.15E-03
cg13024709	24.40	10.04	1.51E-02	62.34	33.42	6.22E-02	-10.76	71.29	8.80E-01	19.32	11.39	8.99E-02	35.03	29.70	2.38E-01
cg26417874	24.19	9.95	1.51E-02	19.81	18.54	2.85E-01	14.14	23.93	5.55E-01	14.55	17.09	3.94E-01	55.53	22.25	1.26E-02
cg00169897	38.81	15.96	1.51E-02	43.31	21.91	4.81E-02	39.12	72.71	5.91E-01	80.73	54.45	1.38E-01	20.87	27.57	4.49E-01
cg05616589	-9.66	3.97	1.51E-02	-10.74	5.77	6.24E-02	-6.11	9.74	5.30E-01	-4.11	11.88	7.29E-01	-12.48	8.00	1.19E-01
cg19192590	-9.69	3.99	1.51E-02	-11.22	5.43	3.89E-02	-11.48	8.77	1.91E-01	12.60	17.58	4.74E-01	-9.48	8.86	2.84E-01
cg19704102	5.60	2.30	1.51E-02	2.40	3.76	5.23E-01	8.16	4.97	1.01E-01	9.11	4.16	2.86E-02	1.52	7.16	8.32E-01
cg03270710	14.54	5.98	1.51E-02	16.99	10.86	1.18E-01	27.24	13.61	4.53E-02	0.01	11.05	9.99E-01	19.53	13.02	1.33E-01
cg12461930	13.98	5.75	1.51E-02	27.14	10.88	1.26E-02	12.60	10.75	2.41E-01	-0.69	12.35	9.55E-01	13.54	12.34	2.73E-01
cg08791813	26.76	11.01	1.51E-02	9.26	20.98	6.59E-01	-9.01	38.85	8.17E-01	35.19	15.63	2.44E-02	50.38	28.57	7.78E-02
cg22767348	-13.37	5.50	1.51E-02	-18.28	11.03	9.76E-02	-33.01	20.82	1.13E-01	-9.22	7.15	1.97E-01	-11.82	18.37	5.20E-01
cg02140579	6.20	2.55	1.51E-02	9.59	3.58	7.42E-03	1.92	5.85	7.43E-01	4.67	7.09	5.10E-01	2.10	6.14	7.32E-01
cg09347306	-22.77	9.37	1.51E-02	-40.53	17.32	1.93E-02	14.11	38.31	7.13E-01	-25.42	15.83	1.08E-01	-9.57	17.19	5.78E-01
cg12619940	21.80	8.97	1.51E-02	13.35	13.98	3.40E-01	21.67	22.60	3.38E-01	29.90	17.09	8.02E-02	30.00	22.78	1.88E-01
cg13372766	-12.44	5.12	1.51E-02	-5.00	10.97	6.49E-01	-9.41	12.30	4.44E-01	-23.20	9.40	1.36E-02	-9.09	9.17	3.22E-01
cg17419815	5.31	2.18	1.51E-02	3.69	3.02	2.22E-01	10.26	5.49	6.17E-02	6.66	4.91	1.75E-01	3.59	6.23	5.65E-01
cg19061112	-15.59	6.41	1.51E-02	-17.66	11.54	1.26E-01	-0.01	13.75	9.99E-01	-16.19	11.05	1.43E-01	-34.20	17.35	4.87E-02
cg02969164	-10.22	4.20	1.51E-02	-11.18	10.31	2.78E-01	-24.84	18.52	1.80E-01	-7.10	5.09	1.63E-01	-22.50	13.36	9.22E-02
cg11311332	-6.37	2.62	1.51E-02	-6.19	4.77	1.94E-01	-13.96	7.10	4.91E-02	-2.19	4.27	6.08E-01	-9.58	6.11	1.17E-01
cg19324627	22.52	9.27	1.51E-02	37.70	17.76	3.38E-02	12.65	16.57	4.45E-01	10.54	18.21	5.63E-01	35.77	23.50	1.28E-01
cg17861150	31.20	12.84	1.51E-02	35.26	21.43	9.99E-02	-33.06	60.16	5.83E-01	26.68	18.87	1.57E-01	58.15	35.32	9.96E-02
cg27211576	15.39	6.34	1.51E-02	14.51	9.58	1.30E-01	19.05	10.91	8.09E-02	-3.96	21.11	8.51E-01	22.04	17.23	2.01E-01
cg11916758	16.08	6.62	1.51E-02	12.84	13.43	3.39E-01	-8.03	42.81	8.51E-01	20.58	8.31	1.33E-02	1.01	21.07	9.62E-01
cg24921319	15.34	6.31	1.51E-02	15.00	8.92	9.27E-02	24.10	13.57	7.58E-02	8.98	16.84	5.94E-01	9.48	16.73	5.71E-01

cg13210763	-7.54	3.11	1.51E-02	-7.01	4.42	1.13E-01	-14.88	6.93	3.18E-02	-1.45	8.98	8.72E-01	-4.97	7.21	4.91E-01
cg09032310	29.99	12.34	1.51E-02	52.28	30.16	8.30E-02	27.67	71.58	6.99E-01	24.46	15.51	1.15E-01	28.99	30.02	3.34E-01
cg04534765	13.06	5.38	1.51E-02	25.83	12.88	4.50E-02	16.84	13.51	2.12E-01	12.89	9.06	1.55E-01	4.29	9.58	6.54E-01
cg02820413	20.21	8.32	1.51E-02	22.42	13.96	1.08E-01	11.76	19.53	5.47E-01	19.24	14.75	1.92E-01	27.55	21.88	2.08E-01
cg14665965	9.74	4.01	1.51E-02	10.12	8.54	2.36E-01	1.50	12.45	9.04E-01	14.73	5.31	5.51E-03	-4.41	10.75	6.82E-01
cg20159095	-19.65	8.09	1.51E-02	-30.19	14.88	4.24E-02	-14.47	19.78	4.65E-01	-11.89	13.11	3.65E-01	-24.16	20.46	2.38E-01
cg13046164	37.85	15.58	1.52E-02	90.47	48.91	6.44E-02	-44.51	196.05	8.20E-01	29.84	17.17	8.23E-02	63.70	59.49	2.84E-01
cg18308184	9.11	3.75	1.52E-02	9.89	5.32	6.29E-02	6.22	10.37	5.49E-01	18.93	12.89	1.42E-01	6.18	7.01	3.78E-01
cg16016716	18.78	7.73	1.52E-02	13.15	12.29	2.85E-01	32.73	13.97	1.91E-02	13.02	18.20	4.74E-01	10.19	22.53	6.51E-01
cg21177426	-16.68	6.87	1.52E-02	-25.40	12.20	3.74E-02	-13.11	14.77	3.75E-01	-9.46	12.54	4.51E-01	-17.78	16.85	2.91E-01
cg01286631	-23.67	9.75	1.52E-02	17.77	30.48	5.60E-01	-74.74	143.47	6.02E-01	-27.22	10.68	1.08E-02	-41.01	39.66	3.01E-01
cg13387677	-65.59	27.01	1.52E-02	-49.99	70.99	4.81E-01	64.45	184.05	7.26E-01	-65.55	32.09	4.11E-02	-106.20	76.34	1.64E-01
cg00133595	23.20	9.56	1.52E-02	25.51	44.87	5.70E-01	76.22	116.32	5.12E-01	22.43	10.12	2.67E-02	27.22	40.08	4.97E-01
cg05062179	37.67	15.51	1.52E-02	44.62	23.92	6.21E-02	7.52	40.79	8.54E-01	43.34	30.08	1.50E-01	37.26	37.77	3.24E-01
cg25188165	-37.41	15.41	1.52E-02	-11.46	24.00	6.33E-01	-14.35	72.67	8.43E-01	-74.79	31.46	1.74E-02	-46.50	27.97	9.64E-02
cg26115276	14.93	6.15	1.52E-02	13.97	7.44	6.06E-02	-10.13	22.36	6.51E-01	34.21	42.85	4.25E-01	24.67	13.07	5.91E-02
cg01757312	16.76	6.90	1.52E-02	28.01	11.24	1.27E-02	22.91	19.19	2.33E-01	18.01	16.11	2.63E-01	-0.26	12.41	9.83E-01
cg14774844	-33.13	13.64	1.52E-02	-51.23	40.77	2.09E-01	244.04	192.64	2.05E-01	-31.45	15.03	3.64E-02	-45.82	56.31	4.16E-01
cg10779492	11.08	4.57	1.52E-02	13.36	4.98	7.26E-03	13.78	8.23	9.39E-02	20.70	8.72	1.76E-02	-2.71	7.50	7.18E-01
cg08374472	20.59	8.48	1.52E-02	18.79	13.52	1.65E-01	26.44	15.94	9.70E-02	5.84	21.49	7.86E-01	28.64	20.73	1.67E-01
cg03943218	17.25	7.11	1.52E-02	36.69	15.25	1.61E-02	15.03	28.77	6.01E-01	16.56	10.41	1.12E-01	2.53	14.05	8.57E-01
cg20309339	-12.34	5.08	1.52E-02	-8.79	10.02	3.81E-01	-11.44	17.15	5.05E-01	-11.99	7.03	8.82E-02	-21.21	13.96	1.29E-01
cg07308381	-13.49	5.56	1.52E-02	-15.45	8.73	7.69E-02	-2.45	13.36	8.54E-01	-16.05	11.67	1.69E-01	-16.28	12.59	1.96E-01
cg11291773	-14.93	6.15	1.52E-02	-15.72	14.09	2.64E-01	-6.73	16.16	6.77E-01	-12.13	8.97	1.77E-01	-26.96	13.92	5.27E-02
cg19222784	-11.22	4.62	1.52E-02	-20.35	7.26	5.07E-03	-7.31	11.00	5.07E-01	-3.10	9.31	7.39E-01	-5.36	11.17	6.32E-01
cg06898279	5.03	2.07	1.52E-02	4.91	2.87	8.66E-02	7.91	4.78	9.83E-02	8.03	6.11	1.88E-01	0.31	4.97	9.51E-01
cg05788138	-7.56	3.11	1.52E-02	-10.09	5.51	6.71E-02	-0.74	8.92	9.33E-01	-7.28	4.87	1.36E-01	-8.46	8.01	2.91E-01
cg04351169	53.37	21.99	1.52E-02	50.83	42.82	2.35E-01	18.80	89.88	8.34E-01	35.53	15.25	1.98E-02	143.79	52.51	6.17E-03
cg00170213	24.66	10.16	1.52E-02	21.84	14.39	1.29E-01	19.58	27.12	4.70E-01	62.31	36.35	8.65E-02	21.74	19.08	2.54E-01
cg00405554	27.87	11.48	1.52E-02	23.25	16.24	1.52E-01	36.72	25.67	1.53E-01	72.40	40.74	7.56E-02	14.29	24.42	5.58E-01
cg15920472	-6.87	2.83	1.52E-02	-6.21	5.05	2.18E-01	-9.27	7.49	2.16E-01	-7.69	4.53	8.97E-02	-3.89	7.23	5.91E-01
cg08486269	10.14	4.18	1.52E-02	14.58	5.71	1.06E-02	10.78	10.37	2.98E-01	11.17	14.90	4.54E-01	-1.40	8.86	8.75E-01
cg12010544	-31.58	13.01	1.52E-02	-28.64	16.74	8.72E-02	44.13	56.79	4.37E-01	-42.93	36.66	2.42E-01	-51.38	27.87	6.52E-02
cg01714553	-10.42	4.29	1.52E-02	-12.12	6.80	7.47E-02	-17.31	8.99	5.41E-02	-15.16	11.92	2.03E-01	1.32	8.68	8.79E-01
cg19785810	-28.76	11.85	1.52E-02	-13.29	17.03	4.35E-01	-22.91	42.10	5.86E-01	-42.13	24.88	9.04E-02	-52.17	25.86	4.36E-02

cg24573310	-31.76	13.09	1.52E-02	-10.56	43.54	8.08E-01	-173.30	196.07	3.77E-01	-31.45	14.22	2.69E-02	-58.50	54.42	2.82E-01
cg15130481	19.88	8.19	1.52E-02	31.52	17.10	6.52E-02	4.85	19.16	8.00E-01	18.17	11.85	1.25E-01	28.01	24.72	2.57E-01
cg00578885	17.12	7.05	1.52E-02	-0.42	19.01	9.82E-01	33.69	27.30	2.17E-01	16.29	8.29	4.94E-02	43.52	26.27	9.76E-02
cg23460578	6.93	2.86	1.52E-02	6.76	4.09	9.83E-02	5.27	7.29	4.70E-01	14.66	9.00	1.03E-01	5.23	5.63	3.53E-01
cg12892506	15.51	6.39	1.52E-02	33.53	16.78	4.57E-02	8.32	25.86	7.48E-01	12.83	8.10	1.13E-01	12.57	15.48	4.17E-01
cg20388823	15.14	6.24	1.52E-02	12.05	8.72	1.67E-01	23.79	22.58	2.92E-01	42.95	27.67	1.21E-01	13.78	10.38	1.84E-01
cg01928104	21.35	8.80	1.52E-02	18.02	17.97	3.16E-01	-12.70	38.17	7.39E-01	22.86	11.14	4.02E-02	41.08	30.34	1.76E-01
cg00815832	-5.63	2.32	1.52E-02	-3.96	3.27	2.26E-01	-10.64	4.80	2.67E-02	-4.81	9.30	6.05E-01	-4.24	5.16	4.11E-01
cg00964997	15.68	6.46	1.52E-02	17.04	10.07	9.05E-02	12.35	17.59	4.83E-01	32.57	16.44	4.76E-02	6.57	11.82	5.78E-01
cg08741280	19.61	8.08	1.52E-02	17.18	22.54	4.46E-01	21.47	27.02	4.27E-01	15.47	9.68	1.10E-01	55.07	27.65	4.64E-02
cg00076538	20.55	8.47	1.52E-02	15.56	21.43	4.68E-01	33.52	36.76	3.62E-01	19.47	10.60	6.61E-02	25.73	21.76	2.37E-01
cg13466180	6.81	2.81	1.53E-02	5.92	3.65	1.05E-01	11.44	6.04	5.81E-02	2.61	11.07	8.14E-01	5.25	7.88	5.05E-01
cg23783747	-51.53	21.24	1.53E-02	-9.85	59.18	8.68E-01	-288.83	200.43	1.50E-01	-59.02	24.45	1.58E-02	-23.48	65.49	7.20E-01
cg20462888	9.00	3.71	1.53E-02	8.38	7.23	2.46E-01	-4.45	10.01	6.56E-01	11.40	5.76	4.77E-02	14.51	8.64	9.32E-02
cg00639289	32.72	13.48	1.53E-02	41.20	17.94	2.17E-02	8.85	55.21	8.73E-01	47.61	32.94	1.48E-01	4.51	29.57	8.79E-01
cg09596131	-10.47	4.31	1.53E-02	-14.65	5.95	1.38E-02	-1.03	11.82	9.31E-01	-2.93	11.96	8.06E-01	-10.66	9.39	2.56E-01
cg03014634	16.17	6.67	1.53E-02	21.42	10.16	3.50E-02	21.66	13.83	1.17E-01	2.20	19.87	9.12E-01	7.43	14.08	5.98E-01
cg20710519	8.37	3.45	1.53E-02	9.15	6.21	1.40E-01	15.38	7.29	3.48E-02	9.25	7.00	1.86E-01	-0.65	7.27	9.29E-01
cg19998644	15.89	6.55	1.53E-02	5.02	8.09	5.35E-01	19.34	10.68	7.01E-02	9.29	16.41	5.71E-01	32.90	11.47	4.14E-03
cg14813603	19.28	7.95	1.53E-02	18.21	11.37	1.09E-01	32.22	29.12	2.69E-01	52.59	29.26	7.23E-02	11.30	13.19	3.91E-01
cg14109388	-10.26	4.23	1.53E-02	-18.77	7.84	1.67E-02	-0.07	10.72	9.95E-01	-5.77	6.96	4.07E-01	-14.48	9.88	1.43E-01
cg23856090	14.67	6.05	1.53E-02	15.93	18.85	3.98E-01	40.91	45.83	3.72E-01	15.57	6.63	1.88E-02	-13.41	27.71	6.28E-01
cg07179669	24.64	10.16	1.53E-02	19.47	12.84	1.29E-01	19.63	44.92	6.62E-01	35.12	25.45	1.68E-01	35.82	25.15	1.54E-01
cg23216612	-7.87	3.24	1.53E-02	-8.03	5.14	1.18E-01	-9.11	5.59	1.03E-01	-2.12	10.32	8.37E-01	-8.37	7.94	2.92E-01
cg23517548	50.64	20.88	1.53E-02	31.75	20.93	1.29E-01	48.77	76.39	5.23E-01	-29.19	95.19	7.59E-01	97.92	32.91	2.92E-03
cg15921911	7.79	3.21	1.53E-02	6.18	4.29	1.50E-01	16.38	7.59	3.09E-02	7.40	8.23	3.69E-01	2.46	9.70	8.00E-01
cg11144056	38.65	15.93	1.53E-02	-0.18	52.95	9.97E-01	-119.93	180.88	5.07E-01	42.63	17.56	1.52E-02	57.51	57.07	3.14E-01
cg03832040	23.80	9.81	1.53E-02	26.87	15.63	8.56E-02	37.21	17.37	3.22E-02	-6.62	19.52	7.34E-01	37.03	23.56	1.16E-01
cg01628737	27.83	11.47	1.53E-02	48.66	24.79	4.96E-02	14.29	24.83	5.65E-01	15.77	11.44	1.68E-01	69.75	31.37	2.62E-02
cg06272678	-48.82	20.13	1.53E-02	-34.30	59.46	5.64E-01	-161.41	200.51	4.21E-01	-56.50	22.31	1.13E-02	45.02	81.46	5.80E-01
cg07834408	97.46	40.19	1.53E-02	85.55	58.08	1.41E-01	200.69	176.51	2.56E-01	33.23	86.70	7.01E-01	153.06	79.66	5.47E-02
cg17479280	-7.99	3.29	1.53E-02	-7.49	4.02	6.27E-02	-11.64	8.56	1.74E-01	-4.32	12.34	7.26E-01	-8.49	9.91	3.92E-01
cg06834503	-51.62	21.29	1.53E-02	-76.09	26.49	4.07E-03	15.28	119.11	8.98E-01	-39.12	107.91	7.17E-01	-5.09	39.98	8.99E-01
cg13987226	44.45	18.33	1.53E-02	37.49	25.48	1.41E-01	79.48	51.31	1.21E-01	23.60	44.61	5.97E-01	58.67	42.48	1.67E-01
cg23416788	39.61	16.34	1.53E-02	13.04	17.72	4.62E-01	29.40	28.01	2.94E-01	94.19	53.57	7.87E-02	65.72	22.03	2.85E-03

cg24612707	-8.64	3.56	1.53E-02	-11.70	4.90	1.70E-02	-2.96	8.06	7.14E-01	-7.51	14.06	5.93E-01	-6.61	7.75	3.94E-01
cg21292067	10.97	4.52	1.53E-02	11.73	5.90	4.69E-02	17.64	10.38	8.95E-02	16.99	16.00	2.88E-01	-4.35	11.94	7.16E-01
cg14131289	-15.84	6.53	1.53E-02	-16.14	11.33	1.55E-01	-21.54	17.53	2.19E-01	-7.97	10.02	4.26E-01	-39.59	20.32	5.14E-02
cg08822625	6.80	2.80	1.53E-02	8.05	4.08	4.85E-02	9.09	6.19	1.42E-01	1.64	6.54	8.01E-01	5.99	7.54	4.27E-01
cg00022145	18.69	7.71	1.53E-02	21.19	15.64	1.75E-01	29.65	17.59	9.19E-02	14.24	11.57	2.18E-01	12.62	22.18	5.69E-01
cg10287970	7.51	3.10	1.53E-02	7.31	3.95	6.47E-02	10.38	8.70	2.33E-01	11.28	8.38	1.79E-01	1.38	8.86	8.76E-01
cg24783510	-50.01	20.63	1.53E-02	-56.18	25.39	2.69E-02	-65.77	70.79	3.53E-01	70.85	97.11	4.66E-01	-50.19	45.01	2.65E-01
cg13573513	6.72	2.77	1.53E-02	7.20	4.31	9.48E-02	11.10	6.97	1.11E-01	6.92	4.73	1.44E-01	-4.51	9.47	6.34E-01
cg25921544	-14.48	5.97	1.53E-02	-19.34	10.09	5.52E-02	-13.08	13.53	3.34E-01	-5.03	12.58	6.89E-01	-17.55	12.48	1.60E-01
cg11912330	19.83	8.18	1.53E-02	-6.43	25.88	8.04E-01	-22.31	78.98	7.78E-01	23.37	9.71	1.61E-02	22.97	19.27	2.33E-01
cg04510639	13.45	5.55	1.53E-02	11.33	10.09	2.61E-01	-4.95	18.01	7.84E-01	16.87	8.28	4.17E-02	19.00	14.15	1.79E-01
cg12432303	-13.42	5.54	1.54E-02	-6.93	12.46	5.78E-01	-27.48	23.75	2.47E-01	-15.59	6.83	2.24E-02	-3.43	18.35	8.52E-01
cg26067203	26.52	10.94	1.54E-02	30.15	32.39	3.52E-01	49.10	56.97	3.89E-01	26.67	13.31	4.51E-02	18.73	26.29	4.76E-01
cg06727060	18.80	7.76	1.54E-02	8.90	13.56	5.11E-01	32.54	21.56	1.31E-01	23.07	12.69	6.90E-02	18.01	18.84	3.39E-01
cg19162768	16.64	6.87	1.54E-02	25.74	8.45	2.32E-03	4.03	17.90	8.22E-01	46.41	36.37	2.02E-01	5.53	10.68	6.04E-01
cg17513325	-24.21	9.99	1.54E-02	-30.68	13.99	2.83E-02	-15.95	41.36	7.00E-01	-23.91	25.61	3.50E-01	-14.32	18.89	4.48E-01
cg00414013	11.39	4.70	1.54E-02	4.55	12.63	7.19E-01	2.28	13.13	8.62E-01	16.23	5.94	6.29E-03	2.89	14.32	8.40E-01
cg06674793	36.57	15.09	1.54E-02	43.64	17.64	1.34E-02	0.20	79.01	9.98E-01	84.11	64.52	1.92E-01	0.17	35.86	9.96E-01
cg11728821	-9.15	3.78	1.54E-02	-6.45	6.52	3.23E-01	-4.17	10.05	6.78E-01	-12.69	6.36	4.59E-02	-11.24	9.12	2.18E-01
cg12832204	-9.78	4.04	1.54E-02	-16.03	6.08	8.34E-03	-14.01	8.44	9.69E-02	-0.32	5.99	9.58E-01	-11.45	9.10	2.08E-01
cg22904401	-20.09	8.29	1.54E-02	-9.39	10.66	3.78E-01	-25.79	15.19	8.95E-02	-42.04	13.74	2.22E-03	-6.08	15.07	6.86E-01
cg06174296	23.16	9.56	1.54E-02	28.62	17.39	9.98E-02	-3.39	22.08	8.78E-01	29.65	15.76	6.00E-02	29.68	25.25	2.40E-01
cg09036154	-47.21	19.48	1.54E-02	-78.08	42.24	6.45E-02	-81.97	209.21	6.95E-01	-19.77	22.46	3.79E-01	-91.80	40.23	2.25E-02
cg03272932	-12.63	5.21	1.54E-02	-12.94	8.88	1.45E-01	-4.46	11.00	6.85E-01	-12.73	10.32	2.18E-01	-22.26	12.41	7.28E-02
cg10253022	-27.22	11.23	1.54E-02	1.12	35.99	9.75E-01	-72.64	154.20	6.38E-01	-27.74	12.28	2.38E-02	-62.04	45.93	1.77E-01
cg17222755	-19.42	8.02	1.54E-02	-24.39	13.66	7.42E-02	-12.52	19.96	5.30E-01	-13.45	15.28	3.79E-01	-24.18	17.12	1.58E-01
cg12565585	25.37	10.47	1.54E-02	49.49	26.09	5.78E-02	21.15	35.69	5.53E-01	22.10	14.03	1.15E-01	16.68	23.66	4.81E-01
cg06705017	6.87	2.84	1.54E-02	7.27	3.92	6.35E-02	7.49	6.47	2.47E-01	6.30	7.19	3.81E-01	5.03	7.94	5.27E-01
cg06191068	12.44	5.13	1.54E-02	18.38	7.56	1.51E-02	13.92	13.43	3.00E-01	14.41	12.14	2.35E-01	-3.04	11.11	7.84E-01
cg12902405	-12.37	5.11	1.54E-02	-11.02	10.11	2.75E-01	-12.40	19.48	5.24E-01	-11.87	6.78	7.98E-02	-18.13	15.51	2.42E-01
cg17816908	19.77	8.16	1.54E-02	30.38	14.20	3.24E-02	4.32	23.02	8.51E-01	18.35	14.45	2.04E-01	14.84	17.22	3.89E-01
cg22931362	-39.20	16.18	1.54E-02	-34.41	30.53	2.60E-01	14.79	165.57	9.29E-01	-40.76	20.54	4.72E-02	-49.22	54.30	3.65E-01
cg00406022	7.11	2.94	1.54E-02	8.68	4.06	3.27E-02	3.55	7.53	6.37E-01	14.95	9.33	1.09E-01	2.48	6.15	6.86E-01
cg08482979	8.14	3.36	1.54E-02	8.15	4.33	5.99E-02	15.48	8.72	7.58E-02	12.02	9.56	2.09E-01	-4.32	9.43	6.47E-01
cg15712777	9.63	3.98	1.54E-02	14.51	5.71	1.10E-02	7.52	10.10	4.56E-01	11.90	10.54	2.59E-01	-1.23	8.52	8.85E-01

cg22960907	40.85	16.86	1.54E-02	28.83	21.54	1.81E-01	94.86	59.02	1.08E-01	91.36	67.78	1.78E-01	40.13	34.18	2.40E-01
cg25159668	-11.46	4.73	1.54E-02	-16.01	11.29	1.56E-01	-2.13	23.34	9.27E-01	-10.02	5.61	7.41E-02	-19.66	17.47	2.60E-01
cg03323067	9.81	4.05	1.54E-02	8.95	5.88	1.28E-01	14.09	8.18	8.50E-02	5.86	19.14	7.60E-01	7.84	8.34	3.47E-01
cg18427787	13.52	5.58	1.54E-02	24.29	10.72	2.35E-02	16.37	20.99	4.36E-01	8.61	7.67	2.61E-01	9.49	15.61	5.43E-01
cg15825104	-26.46	10.92	1.54E-02	-32.05	24.14	1.84E-01	29.89	53.60	5.77E-01	-23.57	13.59	8.29E-02	-54.81	33.27	9.95E-02
cg13500388	11.08	4.57	1.54E-02	15.32	7.00	2.86E-02	15.23	10.91	1.63E-01	0.90	9.80	9.27E-01	9.28	10.81	3.91E-01
cg27291304	10.28	4.24	1.54E-02	13.51	6.16	2.82E-02	16.35	9.28	7.81E-02	-4.92	15.93	7.58E-01	3.23	8.58	7.07E-01
cg06937491	-49.72	20.53	1.54E-02	-49.49	26.13	5.83E-02	203.99	130.63	1.18E-01	-53.73	31.11	8.41E-02	-66.25	30.87	3.19E-02
cg07085664	-18.24	7.53	1.54E-02	-10.40	14.45	4.72E-01	-34.63	37.96	3.62E-01	-17.88	10.05	7.52E-02	-31.51	21.12	1.36E-01
cg13682095	24.76	10.22	1.54E-02	8.36	15.37	5.86E-01	147.90	155.68	3.42E-01	37.60	14.46	9.29E-03	30.46	44.47	4.93E-01
cg25609480	21.32	8.80	1.54E-02	18.78	18.05	2.98E-01	7.26	32.64	8.24E-01	27.87	11.38	1.43E-02	-4.04	29.25	8.90E-01
cg05850338	6.08	2.51	1.55E-02	4.29	3.94	2.77E-01	10.51	5.18	4.25E-02	6.50	4.85	1.80E-01	1.40	8.30	8.66E-01
cg19159842	17.90	7.39	1.55E-02	24.21	10.90	2.63E-02	14.89	14.85	3.16E-01	1.98	20.31	9.22E-01	17.61	18.48	3.41E-01
cg15745921	36.36	15.01	1.55E-02	57.72	23.45	1.38E-02	14.37	41.58	7.30E-01	12.22	35.44	7.30E-01	30.80	28.37	2.78E-01
cg04982401	19.03	7.86	1.55E-02	27.80	25.31	2.72E-01	13.22	69.03	8.48E-01	18.36	9.07	4.28E-02	17.13	21.09	4.17E-01
cg13175532	16.44	6.79	1.55E-02	21.48	10.59	4.24E-02	-3.10	20.06	8.77E-01	12.94	18.37	4.81E-01	18.34	11.69	1.17E-01
cg08657851	-24.88	10.27	1.55E-02	-39.08	17.93	2.93E-02	18.72	27.35	4.94E-01	-27.44	17.62	1.19E-01	-28.25	19.70	1.52E-01
cg09971646	6.19	2.56	1.55E-02	6.76	3.66	6.46E-02	-1.30	6.93	8.51E-01	5.31	6.01	3.77E-01	10.86	5.81	6.16E-02
cg08084454	22.90	9.46	1.55E-02	36.74	29.74	2.17E-01	49.68	162.34	7.60E-01	21.54	10.43	3.89E-02	17.83	34.95	6.10E-01
cg13046832	19.55	8.08	1.55E-02	36.57	19.71	6.36E-02	27.79	34.96	4.27E-01	12.53	10.95	2.52E-01	21.81	16.69	1.91E-01
cg03456220	21.56	8.90	1.55E-02	42.00	18.13	2.05E-02	13.13	19.73	5.06E-01	13.41	13.10	3.06E-01	27.45	29.16	3.47E-01
cg23847069	-7.69	3.18	1.55E-02	-7.05	4.10	8.52E-02	-10.98	7.75	1.57E-01	1.45	11.56	9.00E-01	-11.12	8.09	1.69E-01
cg14720706	28.65	11.83	1.55E-02	25.60	15.03	8.85E-02	41.25	37.81	2.75E-01	28.96	42.40	4.95E-01	31.73	26.19	2.26E-01
cg19182008	-7.90	3.26	1.55E-02	-4.75	6.29	4.50E-01	-7.29	10.10	4.70E-01	-7.56	4.60	1.00E-01	-16.70	9.30	7.27E-02
cg04139007	-15.78	6.52	1.55E-02	-24.00	9.31	9.91E-03	-8.99	13.63	5.09E-01	-15.03	22.27	5.00E-01	-3.39	14.75	8.18E-01
cg04018448	16.02	6.62	1.55E-02	17.89	15.90	2.60E-01	-16.96	66.92	8.00E-01	12.87	7.89	1.03E-01	35.26	19.55	7.12E-02
cg22602019	44.92	18.55	1.55E-02	34.65	22.31	1.20E-01	99.30	65.38	1.29E-01	50.13	77.89	5.20E-01	59.07	44.85	1.88E-01
cg17820025	21.63	8.94	1.55E-02	35.59	15.33	2.03E-02	17.75	15.64	2.57E-01	18.71	21.75	3.90E-01	3.59	21.98	8.70E-01
cg22170528	19.67	8.12	1.55E-02	15.39	21.25	4.69E-01	-10.77	27.24	6.93E-01	25.81	9.73	8.02E-03	5.82	31.10	8.52E-01
cg17226042	7.34	3.03	1.55E-02	3.24	4.41	4.62E-01	12.91	5.76	2.50E-02	14.05	7.34	5.55E-02	1.63	8.00	8.39E-01
cg24149898	-37.59	15.53	1.55E-02	-52.95	48.51	2.75E-01	-11.17	227.15	9.61E-01	-39.46	17.22	2.20E-02	-0.40	54.94	9.94E-01
cg03202804	21.27	8.79	1.55E-02	23.62	13.11	7.16E-02	21.96	35.46	5.36E-01	-5.22	30.52	8.64E-01	23.96	13.78	8.21E-02
cg21631443	-19.30	7.97	1.55E-02	-28.26	13.57	3.73E-02	-20.23	17.50	2.48E-01	-12.29	15.68	4.33E-01	-11.49	18.37	5.32E-01
cg16380285	24.16	9.98	1.55E-02	26.92	21.26	2.06E-01	33.94	20.69	1.01E-01	19.55	15.71	2.13E-01	16.97	26.38	5.20E-01
cg20944315	-10.34	4.27	1.55E-02	-15.30	5.59	6.20E-03	-2.06	10.86	8.50E-01	5.54	12.30	6.52E-01	-12.80	9.58	1.82E-01

cg11100481	11.26	4.65	1.55E-02	6.26	3.95	1.13E-01	20.84	6.80	2.17E-03	22.73	12.09	6.01E-02	2.81	8.05	7.27E-01
cg01889899	-18.25	7.54	1.55E-02	-28.87	14.06	4.00E-02	1.11	17.31	9.49E-01	-18.88	12.95	1.45E-01	-20.42	17.59	2.46E-01
cg08739188	18.67	7.72	1.55E-02	16.35	11.76	1.65E-01	31.04	21.12	1.42E-01	21.70	16.00	1.75E-01	12.02	17.11	4.82E-01
cg13356175	10.05	4.15	1.55E-02	12.73	6.04	3.50E-02	4.99	8.61	5.62E-01	-0.69	16.65	9.67E-01	12.50	8.60	1.46E-01
cg08955941	9.37	3.87	1.55E-02	7.23	5.57	1.94E-01	13.90	8.66	1.09E-01	21.59	9.60	2.46E-02	0.71	8.18	9.31E-01
cg17896129	16.89	6.98	1.55E-02	17.45	21.61	4.19E-01	16.23	70.45	8.18E-01	18.06	7.76	2.00E-02	3.88	25.22	8.78E-01
cg08098256	-8.37	3.46	1.55E-02	-4.62	6.15	4.53E-01	-9.29	10.00	3.53E-01	-6.15	3.99	1.23E-01	-24.13	9.32	9.64E-03
cg23244192	-40.93	16.92	1.55E-02	-36.46	35.55	3.05E-01	-167.06	152.13	2.72E-01	-46.72	20.63	2.35E-02	9.18	56.82	8.72E-01
cg16442298	-15.79	6.53	1.56E-02	-23.83	9.40	1.12E-02	-4.83	15.96	7.62E-01	-18.78	17.82	2.92E-01	-4.47	14.03	7.50E-01
cg22495921	23.21	9.60	1.56E-02	31.72	18.61	8.83E-02	31.31	18.10	8.36E-02	12.49	16.38	4.46E-01	15.42	28.91	5.94E-01
cg10971346	-38.44	15.89	1.56E-02	-39.85	19.61	4.22E-02	-47.99	63.47	4.50E-01	-25.15	38.67	5.15E-01	-44.78	47.42	3.45E-01
cg13584784	-16.73	6.92	1.56E-02	-22.19	11.52	5.40E-02	-4.26	24.48	8.62E-01	-16.72	11.86	1.59E-01	-12.32	14.77	4.04E-01
cg17738213	10.74	4.44	1.56E-02	11.17	6.15	6.96E-02	4.29	9.88	6.64E-01	2.93	19.14	8.78E-01	17.46	9.39	6.30E-02
cg27003776	-71.99	29.76	1.56E-02	-81.57	41.42	4.89E-02	58.10	144.72	6.88E-01	-59.99	60.46	3.21E-01	-89.43	66.71	1.80E-01
cg00870242	-11.44	4.73	1.56E-02	-18.07	6.81	7.97E-03	-9.98	12.11	4.10E-01	5.60	12.36	6.50E-01	-9.24	10.10	3.60E-01
cg03337886	18.43	7.62	1.56E-02	19.18	13.92	1.68E-01	17.73	14.80	2.31E-01	8.59	13.91	5.37E-01	39.92	20.70	5.37E-02
cg16283183	6.74	2.79	1.56E-02	6.52	3.45	5.87E-02	11.10	6.73	9.90E-02	10.01	11.56	3.86E-01	-0.02	8.14	9.98E-01
cg17787502	-13.85	5.72	1.56E-02	-17.88	13.02	1.70E-01	-23.76	18.18	1.91E-01	-14.41	7.60	5.80E-02	0.96	15.26	9.50E-01
cg11828741	-10.55	4.36	1.56E-02	-19.41	10.66	6.87E-02	-30.92	18.82	1.00E-01	-5.57	4.82	2.48E-01	-16.58	11.84	1.61E-01
cg04682135	-11.61	4.80	1.56E-02	-9.94	6.61	1.32E-01	-6.26	12.54	6.18E-01	-8.76	12.29	4.76E-01	-23.77	11.55	3.95E-02
cg17795158	15.98	6.61	1.56E-02	21.69	10.94	4.75E-02	5.83	16.56	7.25E-01	14.68	19.20	4.45E-01	15.11	11.05	1.71E-01
cg23642826	15.29	6.32	1.56E-02	15.51	9.00	8.47E-02	20.85	13.82	1.32E-01	36.68	19.56	6.08E-02	-2.93	14.42	8.39E-01
cg08486875	11.90	4.92	1.56E-02	14.86	7.51	4.78E-02	29.02	12.71	2.24E-02	5.08	9.59	5.96E-01	3.28	10.09	7.45E-01
cg15166089	17.46	7.22	1.56E-02	17.46	11.49	1.29E-01	16.93	13.78	2.19E-01	22.41	17.21	1.93E-01	12.80	18.33	4.85E-01
cg04310063	-9.91	4.10	1.56E-02	-5.64	11.36	6.19E-01	-21.73	21.04	3.02E-01	-10.26	4.65	2.75E-02	-7.01	17.19	6.83E-01
cg13380511	-35.01	14.48	1.56E-02	-98.62	55.60	7.61E-02	-148.43	196.25	4.49E-01	-29.19	15.56	6.06E-02	-36.87	58.64	5.29E-01
cg02861733	11.09	4.59	1.56E-02	17.92	10.80	9.70E-02	-10.92	28.04	6.97E-01	10.28	5.36	5.49E-02	10.23	18.79	5.86E-01
cg15742700	-11.15	4.61	1.56E-02	-11.75	7.72	1.28E-01	-17.44	14.31	2.23E-01	-10.78	7.55	1.54E-01	-6.79	11.27	5.47E-01
cg21446000	-8.95	3.70	1.56E-02	-9.04	7.17	2.07E-01	-2.39	9.43	8.00E-01	-10.32	5.55	6.28E-02	-11.79	10.13	2.45E-01
cg11418595	15.63	6.46	1.56E-02	15.90	7.52	3.45E-02	1.47	13.84	9.16E-01	45.93	18.32	1.22E-02	12.22	10.67	2.52E-01
cg19223622	37.37	15.46	1.56E-02	59.56	38.83	1.25E-01	-195.24	176.80	2.69E-01	31.64	17.93	7.76E-02	65.31	51.40	2.04E-01
cg01203331	30.81	12.74	1.56E-02	31.22	20.63	1.30E-01	35.65	25.32	1.59E-01	2.45	21.63	9.10E-01	67.96	27.78	1.44E-02
cg07269819	16.34	6.76	1.56E-02	16.22	15.88	3.07E-01	10.81	16.43	5.11E-01	18.40	8.80	3.64E-02	11.99	27.79	6.66E-01
cg05642546	6.32	2.61	1.56E-02	6.60	3.42	5.35E-02	13.05	7.01	6.27E-02	7.16	7.34	3.29E-01	-1.74	6.76	7.97E-01
cg24933919	5.54	2.29	1.56E-02	7.14	3.41	3.63E-02	7.33	5.38	1.72E-01	4.12	4.52	3.62E-01	-0.63	6.87	9.27E-01

cg09472600	9.04	3.74	1.56E-02	10.56	5.28	4.57E-02	14.20	8.60	9.89E-02	12.45	10.91	2.54E-01	-2.01	8.50	8.13E-01
cg25216662	17.16	7.10	1.56E-02	5.18	19.87	7.94E-01	7.89	26.17	7.63E-01	20.28	8.24	1.39E-02	15.37	29.61	6.04E-01
cg09886755	27.22	11.26	1.56E-02	29.60	12.81	2.08E-02	44.64	54.04	4.09E-01	68.10	69.45	3.27E-01	3.85	28.41	8.92E-01
cg16922763	-7.14	2.95	1.56E-02	-5.83	4.23	1.68E-01	-8.59	6.34	1.76E-01	1.12	8.54	8.96E-01	-14.55	7.02	3.83E-02
cg00557947	16.70	6.91	1.56E-02	9.97	23.80	6.75E-01	15.30	54.90	7.80E-01	19.92	7.95	1.22E-02	4.01	18.11	8.25E-01
cg19216994	-16.26	6.73	1.56E-02	-27.81	11.26	1.35E-02	-4.24	13.29	7.49E-01	-0.99	15.55	9.49E-01	-23.84	13.31	7.32E-02
cg02825373	24.51	10.14	1.56E-02	33.81	15.60	3.02E-02	-6.32	29.22	8.29E-01	30.41	32.78	3.54E-01	22.35	16.88	1.86E-01
cg14713139	12.48	5.16	1.57E-02	13.69	7.93	8.42E-02	11.77	12.97	3.64E-01	19.07	11.39	9.39E-02	4.18	11.21	7.09E-01
cg18563812	-9.92	4.11	1.57E-02	-6.88	6.73	3.07E-01	-7.90	28.79	7.84E-01	-14.88	6.46	2.12E-02	-5.85	9.09	5.20E-01
cg12379046	17.11	7.08	1.57E-02	35.54	20.43	8.19E-02	39.20	33.81	2.46E-01	12.25	8.02	1.26E-01	27.82	29.79	3.50E-01
cg10896616	16.59	6.86	1.57E-02	11.19	13.44	4.05E-01	-12.95	64.75	8.41E-01	17.51	8.51	3.97E-02	31.17	24.54	2.04E-01
ch.3.151587F	-50.71	20.98	1.57E-02	-63.48	28.76	2.73E-02	-21.80	71.91	7.62E-01	-69.09	70.04	3.24E-01	-30.29	38.78	4.35E-01
cg25252977	6.84	2.83	1.57E-02	8.97	3.85	1.99E-02	7.88	6.12	1.98E-01	-0.34	7.42	9.64E-01	3.61	8.94	6.86E-01
cg10508127	32.97	13.64	1.57E-02	34.24	18.39	6.26E-02	70.10	48.46	1.48E-01	58.65	35.59	9.94E-02	-0.23	28.86	9.94E-01
cg26986928	-10.70	4.43	1.57E-02	-14.08	6.67	3.46E-02	-8.67	12.64	4.93E-01	-5.43	8.90	5.42E-01	-11.02	10.18	2.79E-01
cg02866639	6.50	2.69	1.57E-02	2.36	3.35	4.80E-01	3.98	5.58	4.75E-01	11.71	6.01	5.12E-02	12.47	5.17	1.58E-02
cg05627561	18.22	7.54	1.57E-02	31.02	19.89	1.19E-01	21.74	24.36	3.72E-01	19.31	9.11	3.41E-02	-20.56	27.47	4.54E-01
cg02507501	-13.92	5.76	1.57E-02	-25.20	8.80	4.20E-03	3.58	12.34	7.72E-01	-12.02	8.00	1.33E-01	-16.59	15.08	2.71E-01
cg21012061	14.25	5.90	1.57E-02	12.60	8.52	1.39E-01	18.70	13.20	1.57E-01	7.67	14.10	5.86E-01	21.43	15.41	1.64E-01
cg13341470	-18.80	7.78	1.57E-02	-25.16	13.43	6.10E-02	-12.67	15.37	4.10E-01	-19.03	16.81	2.58E-01	-15.64	17.69	3.77E-01
cg16708495	16.53	6.84	1.57E-02	0.21	19.08	9.91E-01	13.76	18.09	4.47E-01	18.25	8.36	2.90E-02	39.50	28.29	1.63E-01
cg09568061	-58.31	24.13	1.57E-02	-55.40	27.69	4.54E-02	-131.46	102.18	1.98E-01	55.39	77.49	4.75E-01	-92.25	46.32	4.64E-02
cg25080501	-10.87	4.50	1.57E-02	-10.56	9.57	2.70E-01	-14.34	19.54	4.63E-01	-12.60	5.69	2.68E-02	1.11	14.26	9.38E-01
cg15348679	-12.69	5.25	1.57E-02	-12.81	7.61	9.22E-02	-12.33	11.32	2.76E-01	-24.24	15.66	1.22E-01	-6.15	11.87	6.04E-01
cg24029640	-13.55	5.61	1.57E-02	-18.06	9.24	5.07E-02	-10.11	17.08	5.54E-01	-7.47	8.71	3.91E-01	-24.85	16.97	1.43E-01
cg10604550	13.93	5.77	1.57E-02	18.30	8.36	2.86E-02	3.24	13.15	8.05E-01	11.34	13.98	4.17E-01	16.52	14.34	2.49E-01
cg08927145	-6.66	2.76	1.57E-02	-6.09	3.89	1.17E-01	-10.71	5.82	6.57E-02	6.98	13.11	5.94E-01	-6.59	5.78	2.54E-01
cg12112556	25.94	10.74	1.57E-02	10.41	18.62	5.76E-01	31.17	24.78	2.08E-01	40.59	18.73	3.02E-02	21.76	27.66	4.32E-01
cg18276112	6.07	2.51	1.57E-02	5.17	3.23	1.10E-01	14.47	6.09	1.75E-02	6.37	8.71	4.65E-01	-0.34	6.68	9.60E-01
cg02338538	23.38	9.68	1.57E-02	34.83	21.24	1.01E-01	6.71	52.44	8.98E-01	20.78	12.04	8.44E-02	22.17	28.82	4.42E-01
cg14865393	39.91	16.52	1.57E-02	55.92	23.18	1.58E-02	57.76	55.80	3.01E-01	9.07	59.40	8.79E-01	17.53	28.90	5.44E-01
cg16711522	-6.46	2.68	1.57E-02	-4.47	3.68	2.25E-01	-10.60	5.10	3.76E-02	0.49	11.87	9.67E-01	-8.31	7.01	2.36E-01
cg17049032	40.20	16.64	1.57E-02	13.85	44.09	7.53E-01	-38.60	182.94	8.33E-01	51.65	19.33	7.53E-03	2.23	50.74	9.65E-01
cg00549706	22.07	9.14	1.57E-02	19.34	15.90	2.24E-01	31.55	17.72	7.50E-02	17.81	17.90	3.20E-01	18.55	24.18	4.43E-01
cg26549552	-62.39	25.83	1.57E-02	-42.78	48.17	3.75E-01	-273.18	296.21	3.56E-01	-75.56	35.02	3.10E-02	-42.95	64.40	5.05E-01

cg03547606	31.30	12.96	1.57E-02	32.68	17.06	5.54E-02	-34.85	55.09	5.27E-01	53.85	57.30	3.47E-01	36.70	23.04	1.11E-01
cg19670885	-14.61	6.05	1.57E-02	-30.28	14.85	4.15E-02	-33.45	32.70	3.06E-01	-10.64	7.09	1.33E-01	-9.68	22.73	6.70E-01
cg23815646	-7.61	3.15	1.57E-02	-9.23	4.52	4.13E-02	-6.22	6.85	3.64E-01	15.14	14.00	2.79E-01	-10.23	6.27	1.03E-01
cg04362887	7.74	3.21	1.57E-02	8.29	4.14	4.53E-02	13.77	7.60	7.00E-02	-0.13	10.33	9.90E-01	2.66	9.01	7.68E-01
cg16421621	18.64	7.72	1.57E-02	31.05	19.05	1.03E-01	35.74	27.85	1.99E-01	14.70	9.29	1.14E-01	9.45	29.39	7.48E-01
cg27581644	-23.57	9.76	1.57E-02	-0.74	21.95	9.73E-01	-13.45	53.42	8.01E-01	-32.03	11.96	7.40E-03	-15.96	30.50	6.01E-01
cg02860602	19.88	8.23	1.57E-02	15.10	13.15	2.51E-01	24.06	21.54	2.64E-01	29.58	19.99	1.39E-01	18.56	15.22	2.23E-01
cg27357134	-10.81	4.48	1.57E-02	-18.74	11.47	1.02E-01	0.02	15.45	9.99E-01	-11.96	5.45	2.81E-02	1.37	15.04	9.28E-01
cg04003047	-18.45	7.64	1.57E-02	-15.53	16.45	3.45E-01	110.97	184.57	5.48E-01	-30.66	24.22	2.06E-01	-17.91	9.24	5.26E-02
cg04789529	8.47	3.51	1.57E-02	9.40	4.60	4.10E-02	12.94	9.87	1.90E-01	7.85	8.56	3.59E-01	0.42	9.93	9.67E-01
cg20164068	-22.67	9.39	1.58E-02	-23.44	12.34	5.74E-02	8.48	29.12	7.71E-01	-39.99	28.59	1.62E-01	-27.11	20.54	1.87E-01
cg05132283	33.51	13.88	1.58E-02	35.35	26.05	1.75E-01	121.13	109.01	2.66E-01	29.29	18.51	1.14E-01	36.59	37.35	3.27E-01
cg23844013	-13.82	5.72	1.58E-02	-21.90	13.96	1.17E-01	-13.68	23.11	5.54E-01	-11.98	6.93	8.37E-02	-12.71	19.30	5.10E-01
cg13406908	28.91	11.97	1.58E-02	52.26	19.77	8.21E-03	-98.71	88.77	2.66E-01	24.47	10.40	1.87E-02	17.03	32.08	5.95E-01
cg19183842	9.69	4.01	1.58E-02	8.91	5.40	9.88E-02	5.09	10.37	6.24E-01	20.80	14.95	1.64E-01	11.07	8.43	1.89E-01
cg05108031	30.84	12.77	1.58E-02	30.22	18.76	1.07E-01	35.17	51.71	4.96E-01	63.10	41.19	1.26E-01	22.72	20.74	2.73E-01
cg15664905	12.47	5.17	1.58E-02	16.25	7.58	3.21E-02	17.85	11.38	1.17E-01	8.54	12.39	4.91E-01	-1.52	13.10	9.08E-01
cg04857172	-18.42	7.63	1.58E-02	-30.74	13.27	2.05E-02	-25.68	16.32	1.16E-01	4.65	16.54	7.79E-01	-15.54	15.19	3.06E-01
cg01775612	11.20	4.64	1.58E-02	10.01	7.57	1.86E-01	13.70	9.13	1.33E-01	15.60	10.89	1.52E-01	5.78	10.80	5.92E-01
cg24534258	-20.28	8.40	1.58E-02	-20.79	12.16	8.73E-02	-36.52	22.15	9.92E-02	-5.64	7.69	4.63E-01	-38.34	14.60	8.66E-03
cg26673264	14.71	6.09	1.58E-02	4.97	13.90	7.21E-01	10.14	35.92	7.78E-01	15.94	7.29	2.88E-02	28.80	21.39	1.78E-01
cg02643667	-22.03	9.12	1.58E-02	-17.97	24.15	4.57E-01	8.29	37.52	8.25E-01	-27.46	10.71	1.04E-02	-0.47	33.78	9.89E-01
cg27474175	9.11	3.78	1.58E-02	9.25	6.84	1.76E-01	1.46	7.63	8.49E-01	10.95	6.40	8.70E-02	20.90	11.85	7.77E-02
cg03445404	34.53	14.30	1.58E-02	38.59	18.32	3.52E-02	4.48	53.84	9.34E-01	9.29	40.66	8.19E-01	48.65	32.31	1.32E-01
cg13439730	15.71	6.51	1.58E-02	15.26	15.02	3.10E-01	-7.47	16.86	6.58E-01	23.19	7.91	3.37E-03	2.08	23.44	9.29E-01
cg09152120	12.90	5.34	1.58E-02	19.40	13.20	1.42E-01	14.30	13.27	2.81E-01	10.36	6.96	1.37E-01	15.30	18.37	4.05E-01
cg05458764	20.12	8.34	1.58E-02	16.17	16.90	3.39E-01	39.14	17.64	2.65E-02	15.06	13.35	2.59E-01	10.99	21.99	6.17E-01
cg07103895	19.23	7.97	1.58E-02	20.45	19.23	2.88E-01	-13.08	21.17	5.37E-01	27.00	10.45	9.78E-03	17.87	24.58	4.67E-01
cg21203569	7.89	3.27	1.58E-02	6.35	4.79	1.85E-01	12.15	7.38	9.95E-02	15.42	8.72	7.69E-02	1.94	7.36	7.92E-01
cg14069333	-9.96	4.13	1.58E-02	-5.47	11.89	6.45E-01	-1.18	19.55	9.52E-01	-10.22	4.75	3.15E-02	-19.09	14.52	1.89E-01
cg13265003	8.23	3.41	1.58E-02	6.72	4.32	1.20E-01	19.61	8.85	2.68E-02	7.98	10.70	4.56E-01	2.60	9.49	7.84E-01
cg20336981	20.07	8.31	1.58E-02	35.66	12.62	4.70E-03	-9.14	21.18	6.66E-01	16.54	18.87	3.81E-01	18.06	12.92	1.62E-01
cg06842409	-5.64	2.34	1.58E-02	-5.88	3.24	6.94E-02	-7.65	5.30	1.48E-01	-4.60	9.12	6.14E-01	-3.59	5.00	4.73E-01
cg00480161	31.89	13.21	1.58E-02	27.68	17.27	1.09E-01	66.26	44.47	1.36E-01	9.48	36.80	7.97E-01	43.58	29.70	1.42E-01
cg12259256	-33.84	14.02	1.58E-02	-35.59	15.56	2.22E-02	-59.43	71.19	4.04E-01	-84.00	78.87	2.87E-01	0.10	40.84	9.98E-01

cg02169201	23.02	9.54	1.58E-02	29.09	17.76	1.01E-01	19.17	18.77	3.07E-01	12.54	16.90	4.58E-01	42.16	25.98	1.05E-01
cg00572843	14.74	6.11	1.58E-02	17.06	8.04	3.38E-02	21.55	16.08	1.80E-01	-18.17	22.03	4.09E-01	15.78	13.60	2.46E-01
cg07108754	12.48	5.17	1.58E-02	15.46	8.08	5.57E-02	23.54	14.89	1.14E-01	14.49	7.37	4.93E-02	-8.68	13.06	5.07E-01
cg05966983	36.08	14.95	1.58E-02	32.16	42.53	4.50E-01	-18.58	169.06	9.13E-01	40.53	16.88	1.63E-02	5.43	51.52	9.16E-01
cg22158462	-11.81	4.89	1.58E-02	-26.68	12.06	2.70E-02	3.04	18.57	8.70E-01	-10.62	6.06	7.97E-02	-6.18	14.48	6.70E-01
cg13628022	-9.60	3.98	1.58E-02	-8.84	5.55	1.11E-01	-12.28	7.93	1.21E-01	-32.01	17.74	7.12E-02	-1.95	9.25	8.33E-01
cg01076861	-9.68	4.01	1.58E-02	-15.70	7.08	2.66E-02	-10.53	16.79	5.30E-01	-7.07	5.69	2.14E-01	-4.21	11.36	7.11E-01
cg23001000	54.42	22.55	1.58E-02	66.57	48.82	1.73E-01	6.62	215.75	9.76E-01	54.39	27.62	4.89E-02	35.51	68.33	6.03E-01
cg09073106	-36.56	15.15	1.58E-02	-9.52	52.06	8.55E-01	-81.42	206.68	6.94E-01	-42.95	16.53	9.38E-03	10.87	57.26	8.49E-01
cg04080448	7.84	3.25	1.58E-02	12.03	4.95	1.50E-02	9.77	7.76	2.08E-01	5.82	8.21	4.78E-01	0.10	6.67	9.89E-01
cg22544571	-16.82	6.97	1.58E-02	-34.73	8.45	3.95E-05	-13.33	11.08	2.29E-01	-7.79	7.75	3.15E-01	-9.11	11.74	4.38E-01
cg13978856	-13.58	5.63	1.58E-02	-20.86	10.02	3.74E-02	-8.06	13.65	5.55E-01	-10.11	10.24	3.23E-01	-12.12	12.24	3.22E-01
cg09874127	-43.11	17.87	1.58E-02	-30.89	21.66	1.54E-01	-79.92	67.79	2.38E-01	11.20	80.64	8.90E-01	-85.12	39.90	3.29E-02
cg06099244	-15.53	6.44	1.58E-02	-18.04	12.33	1.43E-01	-5.40	18.01	7.65E-01	-19.97	9.18	2.96E-02	-0.98	19.56	9.60E-01
cg04451353	11.35	4.70	1.58E-02	15.21	10.11	1.33E-01	26.02	12.33	3.49E-02	7.04	6.37	2.69E-01	4.69	15.43	7.61E-01
cg23662331	-10.61	4.40	1.59E-02	-4.87	7.11	4.93E-01	-19.80	9.87	4.49E-02	-12.89	10.19	2.06E-01	-10.37	9.11	2.55E-01
cg22299454	15.44	6.40	1.59E-02	36.82	21.16	8.19E-02	16.69	65.26	7.98E-01	15.18	7.13	3.31E-02	-3.65	21.09	8.63E-01
cg17216737	16.58	6.87	1.59E-02	11.49	10.16	2.58E-01	27.22	13.45	4.29E-02	20.48	20.08	3.08E-01	11.05	16.96	5.15E-01
cg07586193	17.15	7.11	1.59E-02	7.28	16.19	6.53E-01	-20.70	70.23	7.68E-01	21.15	8.31	1.09E-02	7.43	27.84	7.90E-01
cg13764106	23.62	9.79	1.59E-02	-9.35	28.33	7.41E-01	18.39	92.82	8.43E-01	31.83	11.05	3.96E-03	-5.67	33.83	8.67E-01
cg17471779	-29.64	12.29	1.59E-02	-30.13	15.50	5.19E-02	-38.03	30.64	2.15E-01	-57.72	53.41	2.80E-01	-9.75	30.93	7.53E-01
cg26815291	18.08	7.49	1.59E-02	16.82	23.46	4.73E-01	3.25	74.52	9.65E-01	19.38	8.26	1.90E-02	5.93	29.35	8.40E-01
cg23648239	5.34	2.21	1.59E-02	3.62	3.10	2.43E-01	11.54	5.85	4.86E-02	7.70	4.62	9.57E-02	0.62	6.48	9.23E-01
cg25649765	-9.14	3.79	1.59E-02	-13.37	5.98	2.54E-02	-2.19	8.50	7.96E-01	-4.67	8.44	5.80E-01	-12.09	8.51	1.55E-01
cg00681365	-12.01	4.98	1.59E-02	-14.01	13.42	2.96E-01	3.68	23.99	8.78E-01	-11.95	5.87	4.19E-02	-16.47	15.73	2.95E-01
cg14207539	-15.06	6.25	1.59E-02	-10.41	16.73	5.34E-01	-0.27	27.97	9.92E-01	-16.16	7.18	2.45E-02	-25.18	26.61	3.44E-01
cg10514113	17.12	7.10	1.59E-02	19.13	16.19	2.37E-01	36.82	25.40	1.47E-01	18.45	7.96	2.04E-02	-27.07	28.10	3.35E-01
cg19570574	28.50	11.82	1.59E-02	36.30	16.39	2.68E-02	-0.78	34.71	9.82E-01	31.42	27.46	2.53E-01	21.80	27.90	4.35E-01
cg22968936	20.16	8.36	1.59E-02	42.89	23.64	6.96E-02	9.59	65.83	8.84E-01	22.94	9.77	1.89E-02	-9.73	21.17	6.46E-01
cg25764120	-10.23	4.24	1.59E-02	-10.82	9.08	2.33E-01	-8.15	16.95	6.31E-01	-9.76	5.56	7.91E-02	-12.28	11.51	2.86E-01
cg11367973	12.34	5.12	1.59E-02	8.55	6.81	2.09E-01	30.25	10.87	5.38E-03	6.06	10.74	5.73E-01	8.63	11.71	4.61E-01
cg18351858	14.39	5.97	1.59E-02	12.98	8.15	1.11E-01	19.48	19.60	3.20E-01	20.49	15.85	1.96E-01	11.86	12.45	3.41E-01
cg01101873	9.76	4.05	1.59E-02	9.81	4.75	3.90E-02	18.38	8.67	3.41E-02	14.42	9.00	1.09E-01	-4.23	9.00	6.39E-01
cg13506158	-15.88	6.58	1.59E-02	-19.60	13.15	1.36E-01	-16.41	18.28	3.69E-01	-15.24	9.52	1.09E-01	-10.92	17.52	5.33E-01
cg03258665	11.31	4.69	1.59E-02	12.62	6.28	4.43E-02	5.56	10.32	5.90E-01	2.32	16.65	8.89E-01	18.88	11.93	1.13E-01

cg17680767	-13.56	5.62	1.59E-02	-9.00	8.01	2.61E-01	-15.48	12.06	1.99E-01	-0.64	21.92	9.77E-01	-25.56	11.90	3.17E-02
cg20334115	8.13	3.37	1.59E-02	7.96	4.36	6.83E-02	11.43	9.41	2.24E-01	3.66	9.77	7.08E-01	9.46	8.53	2.67E-01
cg19985056	4.30	1.78	1.59E-02	6.41	3.22	4.65E-02	6.14	5.44	2.59E-01	3.41	2.78	2.20E-01	1.54	4.29	7.19E-01
cg11074070	-14.71	6.10	1.59E-02	-5.12	16.59	7.57E-01	-12.44	23.98	6.04E-01	-14.78	7.09	3.70E-02	-38.19	25.10	1.28E-01
cg06418871	8.05	3.34	1.59E-02	14.52	6.95	3.67E-02	9.73	6.63	1.42E-01	3.95	6.76	5.59E-01	4.67	6.42	4.67E-01
cg17828057	-6.28	2.60	1.59E-02	-7.73	3.52	2.80E-02	0.73	5.96	9.03E-01	-8.52	7.16	2.34E-01	-8.16	7.24	2.60E-01
cg12208258	16.64	6.90	1.59E-02	-28.24	28.37	3.19E-01	24.52	44.16	5.79E-01	20.68	6.86	2.56E-03	6.62	22.66	7.70E-01
cg22280682	22.87	9.49	1.59E-02	14.50	19.36	4.54E-01	28.15	18.70	1.32E-01	21.13	15.48	1.72E-01	33.19	26.69	2.14E-01
cg00618626	32.38	13.43	1.59E-02	40.93	21.16	5.30E-02	53.93	28.82	6.13E-02	8.06	31.09	7.95E-01	13.78	30.59	6.52E-01
cg24916020	32.16	13.34	1.59E-02	36.61	18.47	4.75E-02	23.47	34.00	4.90E-01	-16.39	41.93	6.96E-01	49.79	28.26	7.81E-02
cg17231999	14.18	5.89	1.59E-02	21.92	17.45	2.09E-01	11.54	31.66	7.15E-01	10.81	7.10	1.28E-01	23.48	14.52	1.06E-01
cg01172345	-15.89	6.59	1.59E-02	-29.04	11.92	1.49E-02	-15.24	16.84	3.65E-01	-14.58	7.95	6.69E-02	16.81	22.38	4.53E-01
cg15479752	18.88	7.83	1.59E-02	49.58	20.63	1.62E-02	8.22	25.10	7.43E-01	13.63	9.45	1.49E-01	21.90	29.31	4.55E-01
cg06048662	-29.37	12.19	1.59E-02	-34.14	18.59	6.63E-02	-23.88	23.88	3.17E-01	-34.39	27.06	2.04E-01	-14.04	37.30	7.07E-01
cg09637172	-9.43	3.91	1.59E-02	-9.04	5.80	1.19E-01	-11.27	11.54	3.29E-01	-11.98	10.06	2.33E-01	-7.92	7.39	2.84E-01
cg16182757	9.73	4.04	1.60E-02	9.87	5.40	6.78E-02	12.62	11.48	2.72E-01	14.00	10.00	1.61E-01	2.42	10.29	8.14E-01
cg10313368	-9.40	3.90	1.60E-02	-7.28	7.77	3.48E-01	2.75	13.25	8.36E-01	-12.00	5.35	2.49E-02	-10.99	10.83	3.10E-01
cg14042799	10.33	4.29	1.60E-02	14.54	6.00	1.53E-02	-0.23	9.71	9.81E-01	8.59	15.14	5.71E-01	10.55	9.26	2.55E-01
cg07546360	18.02	7.48	1.60E-02	25.72	20.94	2.19E-01	33.82	19.35	8.06E-02	11.85	9.30	2.03E-01	26.30	26.84	3.27E-01
cg15393903	-10.52	4.37	1.60E-02	-15.00	10.81	1.65E-01	-9.99	12.11	4.09E-01	-8.17	5.48	1.36E-01	-21.85	16.14	1.76E-01
cg21109666	-2.89	1.20	1.60E-02	-1.74	1.73	3.15E-01	-5.78	2.33	1.32E-02	-3.30	3.22	3.05E-01	-0.59	3.48	8.65E-01
cg25943061	-24.39	10.12	1.60E-02	-36.92	24.09	1.25E-01	-133.63	163.72	4.14E-01	-19.93	11.65	8.70E-02	-35.74	39.80	3.69E-01
cg27012396	20.18	8.37	1.60E-02	18.30	16.79	2.76E-01	32.35	19.37	9.49E-02	12.27	12.63	3.31E-01	33.57	23.71	1.57E-01
cg11390468	36.46	15.13	1.60E-02	45.35	19.05	1.73E-02	3.12	81.44	9.69E-01	-22.00	52.46	6.75E-01	38.10	30.17	2.07E-01
cg14296561	6.52	2.71	1.60E-02	4.27	3.56	2.31E-01	11.79	6.49	6.91E-02	12.49	8.13	1.25E-01	4.49	7.25	5.36E-01
cg19902593	-11.15	4.63	1.60E-02	-19.91	9.80	4.22E-02	-21.16	13.16	1.08E-01	-6.16	6.50	3.43E-01	-6.65	12.11	5.83E-01
cg26177311	-12.54	5.20	1.60E-02	-17.27	12.99	1.84E-01	-1.92	24.07	9.36E-01	-11.71	6.31	6.32E-02	-15.24	15.59	3.28E-01
cg21376774	15.37	6.38	1.60E-02	20.46	10.98	6.24E-02	12.37	14.94	4.08E-01	22.38	13.36	9.40E-02	4.39	12.71	7.30E-01
cg00561854	-10.72	4.45	1.60E-02	-6.36	14.71	6.66E-01	-4.50	27.98	8.72E-01	-12.75	4.89	9.17E-03	8.95	18.60	6.30E-01
cg24641352	11.44	4.75	1.60E-02	9.70	7.70	2.08E-01	10.56	11.47	3.57E-01	6.57	10.03	5.12E-01	19.87	10.00	4.70E-02
cg27573344	-10.09	4.19	1.60E-02	-12.23	7.22	9.02E-02	-2.32	9.26	8.02E-01	-4.87	8.68	5.75E-01	-19.33	8.82	2.83E-02
cg10758022	-12.79	5.31	1.60E-02	3.75	13.78	7.85E-01	-1.51	26.48	9.54E-01	-16.58	6.23	7.74E-03	-14.64	18.33	4.25E-01
cg24707200	-15.91	6.60	1.60E-02	-11.17	9.85	2.56E-01	-24.59	15.39	1.10E-01	-15.00	15.44	3.31E-01	-19.71	15.43	2.01E-01
cg09555414	-64.55	26.79	1.60E-02	-65.00	36.12	7.19E-02	61.47	165.82	7.11E-01	-162.10	75.82	3.25E-02	-33.96	49.02	4.89E-01
cg15431934	18.16	7.54	1.60E-02	15.00	9.85	1.28E-01	29.88	17.15	8.16E-02	5.87	34.05	8.63E-01	19.27	18.15	2.88E-01

cg09175325	19.00	7.89	1.60E-02	9.40	11.92	4.30E-01	24.38	15.71	1.21E-01	26.71	18.54	1.50E-01	30.24	21.93	1.68E-01
cg22782986	31.11	12.91	1.60E-02	27.13	17.09	1.13E-01	76.26	63.93	2.33E-01	44.86	41.62	2.81E-01	28.04	23.88	2.40E-01
cg14310703	17.51	7.27	1.60E-02	12.94	15.60	4.07E-01	43.76	30.78	1.55E-01	14.92	9.43	1.14E-01	25.45	19.86	2.00E-01
cg00824880	29.30	12.16	1.60E-02	24.87	27.82	3.71E-01	-40.26	114.08	7.24E-01	39.93	14.90	7.36E-03	-12.24	33.59	7.15E-01
cg02725362	19.87	8.25	1.60E-02	35.17	17.23	4.13E-02	4.56	16.06	7.77E-01	14.88	13.27	2.62E-01	40.22	23.72	8.99E-02
cg22851252	49.90	20.71	1.60E-02	39.22	26.23	1.35E-01	20.04	91.79	8.27E-01	58.50	63.53	3.57E-01	83.02	44.24	6.06E-02
cg18258482	-7.52	3.12	1.60E-02	-7.29	6.24	2.43E-01	-1.78	10.96	8.71E-01	-9.57	4.18	2.19E-02	-1.81	9.46	8.49E-01
cg20949198	-23.97	9.95	1.60E-02	-27.21	19.95	1.73E-01	3.22	33.48	9.23E-01	-25.35	13.36	5.78E-02	-31.74	30.32	2.95E-01
cg15896696	9.03	3.75	1.60E-02	10.53	5.57	5.90E-02	8.10	7.57	2.85E-01	12.49	10.82	2.48E-01	4.31	8.79	6.24E-01
cg07419921	-10.43	4.33	1.60E-02	-10.97	10.07	2.76E-01	-10.29	20.54	6.16E-01	-9.86	5.36	6.61E-02	-12.80	12.56	3.08E-01
cg03231471	-11.92	4.95	1.60E-02	-17.91	8.01	2.54E-02	-1.10	11.67	9.25E-01	-7.74	10.53	4.62E-01	-14.59	10.60	1.69E-01
cg27420946	-16.49	6.85	1.60E-02	-23.34	22.42	2.98E-01	-16.49	34.01	6.28E-01	-15.42	7.62	4.29E-02	-20.38	28.38	4.73E-01
cg24706697	40.07	16.64	1.60E-02	57.70	39.84	1.47E-01	-80.79	189.28	6.70E-01	38.13	19.57	5.14E-02	32.35	53.94	5.49E-01
cg16122628	-9.08	3.77	1.60E-02	-9.72	7.06	1.69E-01	2.79	10.04	7.81E-01	-12.68	5.43	1.96E-02	-6.48	12.44	6.03E-01
cg11367260	-11.25	4.67	1.60E-02	-9.48	7.16	1.85E-01	-3.98	12.28	7.46E-01	-10.15	10.16	3.18E-01	-20.60	10.01	3.95E-02
cg00899855	12.34	5.12	1.60E-02	14.74	9.13	1.06E-01	21.03	12.29	8.70E-02	8.98	8.07	2.66E-01	3.90	15.59	8.03E-01
cg14372570	18.66	7.75	1.60E-02	25.16	20.33	2.16E-01	33.67	38.22	3.78E-01	15.36	8.97	8.67E-02	32.05	29.92	2.84E-01
cg23705108	-15.16	6.29	1.60E-02	-15.68	12.64	2.15E-01	-44.45	28.22	1.15E-01	-15.64	8.42	6.31E-02	-2.15	16.64	8.97E-01
cg06846458	19.14	7.95	1.60E-02	41.94	20.43	4.01E-02	32.34	27.13	2.33E-01	10.58	8.41	2.08E-01	44.04	30.73	1.52E-01
cg25569396	31.16	12.94	1.60E-02	36.20	15.30	1.80E-02	19.87	67.30	7.68E-01	45.32	51.68	3.81E-01	9.13	30.09	7.61E-01
cg19637591	-10.63	4.42	1.60E-02	-21.80	8.19	7.80E-03	-1.56	8.66	8.57E-01	-10.43	6.93	1.32E-01	-4.42	12.66	7.27E-01
cg09185231	-14.57	6.05	1.60E-02	-11.26	18.68	5.47E-01	-15.60	11.22	1.64E-01	-11.61	8.08	1.51E-01	-53.65	28.97	6.40E-02
cg25520872	18.79	7.80	1.60E-02	25.59	20.06	2.02E-01	43.24	30.61	1.58E-01	7.59	10.05	4.50E-01	35.79	16.41	2.92E-02
cg14784398	-20.88	8.67	1.60E-02	-31.96	29.05	2.71E-01	-63.17	70.81	3.72E-01	-22.04	9.47	2.00E-02	24.13	36.16	5.05E-01
cg16549684	-12.20	5.07	1.61E-02	-12.39	10.45	2.36E-01	-3.44	23.36	8.83E-01	-11.92	6.61	7.15E-02	-16.31	14.05	2.45E-01
cg25657700	8.62	3.58	1.61E-02	9.43	5.37	7.88E-02	7.81	7.73	3.13E-01	12.00	10.80	2.67E-01	6.20	7.46	4.06E-01
cg20682895	25.48	10.58	1.61E-02	9.78	20.81	6.38E-01	-4.23	50.42	9.33E-01	35.21	14.06	1.23E-02	24.38	29.26	4.05E-01
cg23355040	22.63	9.40	1.61E-02	20.05	15.17	1.86E-01	42.58	35.27	2.27E-01	12.88	7.69	9.40E-02	63.22	25.58	1.35E-02
cg07471190	24.18	10.05	1.61E-02	21.10	20.91	3.13E-01	42.42	24.79	8.71E-02	15.82	14.08	2.61E-01	44.78	32.42	1.67E-01
cg10672416	-13.20	5.48	1.61E-02	-6.15	10.25	5.49E-01	-0.72	13.25	9.57E-01	-21.56	8.83	1.46E-02	-19.15	13.86	1.67E-01
cg09161138	20.45	8.49	1.61E-02	22.09	13.80	1.09E-01	20.58	17.53	2.40E-01	22.55	16.10	1.61E-01	8.96	25.85	7.29E-01
cg19044706	-22.72	9.44	1.61E-02	-30.30	17.04	7.53E-02	16.67	38.01	6.61E-01	-36.73	18.01	4.14E-02	-12.23	15.79	4.39E-01
cg10636896	-11.28	4.69	1.61E-02	-5.12	9.30	5.82E-01	-16.15	18.33	3.78E-01	-12.21	6.35	5.44E-02	-16.76	12.74	1.88E-01
cg11268280	9.26	3.85	1.61E-02	12.25	5.15	1.75E-02	14.73	9.08	1.05E-01	7.06	13.70	6.06E-01	-4.18	8.97	6.42E-01
cg00947878	7.94	3.30	1.61E-02	6.68	3.74	7.45E-02	20.24	7.68	8.40E-03	-0.41	7.77	9.58E-01	7.63	6.08	2.09E-01

ch.22.728807R	15.03	6.24	1.61E-02	14.05	8.50	9.83E-02	7.39	13.24	5.77E-01	73.21	37.39	5.02E-02	17.94	13.65	1.89E-01
cg00007987	-24.89	10.34	1.61E-02	-22.18	19.73	2.61E-01	-2.98	55.62	9.57E-01	-26.15	14.72	7.56E-02	-29.31	23.26	2.08E-01
cg00145118	10.48	4.35	1.61E-02	13.32	5.85	2.28E-02	17.50	11.23	1.19E-01	-4.44	10.97	6.86E-01	8.45	11.68	4.69E-01
cg26087735	19.84	8.24	1.61E-02	27.94	15.11	6.44E-02	10.64	14.61	4.66E-01	20.59	17.13	2.29E-01	22.13	21.14	2.95E-01
cg02675010	-24.40	10.14	1.61E-02	-59.66	34.55	8.42E-02	63.39	117.72	5.90E-01	-19.25	10.98	7.96E-02	-61.34	43.52	1.59E-01
cg07292140	-11.81	4.91	1.61E-02	-13.21	12.66	2.97E-01	9.61	16.43	5.59E-01	-16.06	6.03	7.73E-03	-0.45	15.68	9.77E-01
cg24915751	43.16	17.93	1.61E-02	42.27	20.06	3.51E-02	25.67	125.47	8.38E-01	81.26	61.72	1.88E-01	20.75	57.88	7.20E-01
cg15077558	-24.37	10.12	1.61E-02	2.06	25.15	9.35E-01	-65.14	79.35	4.12E-01	-27.81	11.93	1.98E-02	-35.56	31.69	2.62E-01
cg17969789	15.77	6.55	1.61E-02	13.08	10.80	2.26E-01	14.53	13.05	2.65E-01	12.31	15.82	4.36E-01	24.90	14.37	8.32E-02
cg09189538	8.67	3.60	1.61E-02	7.86	8.57	3.59E-01	13.91	13.07	2.87E-01	6.91	4.47	1.22E-01	17.99	11.62	1.21E-01
cg04742719	9.85	4.09	1.61E-02	8.70	5.21	9.50E-02	15.59	10.51	1.38E-01	10.60	12.01	3.78E-01	7.71	12.11	5.24E-01
cg20400383	30.15	12.53	1.61E-02	73.02	37.46	5.13E-02	55.27	166.01	7.39E-01	25.93	14.07	6.53E-02	12.38	41.92	7.68E-01
cg06369833	33.72	14.01	1.61E-02	50.50	39.72	2.04E-01	-144.50	165.41	3.82E-01	36.17	15.58	2.02E-02	-13.26	57.46	8.17E-01
cg07523098	-31.79	13.21	1.61E-02	-35.41	16.29	2.97E-02	-47.93	40.75	2.39E-01	-42.73	48.92	3.82E-01	-2.17	32.55	9.47E-01
cg11007406	23.67	9.84	1.61E-02	41.34	16.13	1.04E-02	41.17	31.37	1.89E-01	9.66	20.47	6.37E-01	6.73	18.00	7.09E-01
cg04048411	24.91	10.36	1.61E-02	32.17	17.73	6.96E-02	-75.94	55.71	1.73E-01	26.60	11.13	1.69E-02	28.30	27.48	3.03E-01
cg23800778	17.54	7.29	1.61E-02	20.81	14.17	1.42E-01	0.65	12.90	9.60E-01	26.35	12.82	3.99E-02	35.57	23.91	1.37E-01
cg15100231	26.76	11.12	1.61E-02	19.48	16.16	2.28E-01	45.01	25.65	7.94E-02	-5.80	35.52	8.70E-01	40.12	22.70	7.71E-02
cg01016857	-10.13	4.21	1.61E-02	-14.44	5.74	1.19E-02	-7.44	10.35	4.72E-01	-12.31	12.92	3.41E-01	1.00	9.68	9.18E-01
cg15487600	26.68	11.09	1.61E-02	9.16	22.63	6.86E-01	23.89	58.27	6.82E-01	31.60	14.26	2.67E-02	37.92	32.19	2.39E-01
cg14917589	22.57	9.38	1.61E-02	32.11	19.84	1.06E-01	30.24	21.29	1.55E-01	7.54	13.62	5.80E-01	55.28	28.64	5.35E-02
cg20459332	43.60	18.13	1.61E-02	44.53	23.80	6.14E-02	-29.86	63.56	6.39E-01	43.52	66.09	5.10E-01	64.24	35.30	6.88E-02
cg19197915	-18.32	7.62	1.62E-02	-22.13	9.68	2.22E-02	-12.99	25.47	6.10E-01	-30.96	10.54	3.31E-03	3.54	13.54	7.94E-01
cg26511321	18.04	7.50	1.62E-02	20.88	11.75	7.55E-02	27.56	19.05	1.48E-01	33.11	23.43	1.58E-01	5.57	12.95	6.67E-01
cg19818278	50.64	21.06	1.62E-02	65.68	25.45	9.86E-03	-56.65	102.41	5.80E-01	-4.20	110.41	9.70E-01	34.78	43.26	4.21E-01
cg22315928	-11.62	4.83	1.62E-02	-12.45	12.24	3.09E-01	10.41	16.90	5.38E-01	-13.07	6.05	3.09E-02	-17.59	13.63	1.97E-01
cg20250250	-9.81	4.08	1.62E-02	-13.98	7.42	5.94E-02	-28.65	12.93	2.67E-02	-5.98	5.20	2.50E-01	-3.72	9.22	6.87E-01
cg23971517	-19.04	7.92	1.62E-02	-38.63	17.94	3.13E-02	0.57	18.01	9.75E-01	-19.34	11.24	8.53E-02	-17.49	23.25	4.52E-01
cg03169059	7.15	2.97	1.62E-02	7.20	3.83	6.04E-02	13.11	7.32	7.34E-02	9.09	9.01	3.13E-01	-2.55	8.36	7.60E-01
cg02747822	16.53	6.87	1.62E-02	25.68	9.91	9.55E-03	20.31	14.46	1.60E-01	-1.64	16.85	9.23E-01	-1.11	19.33	9.54E-01
cg19418190	-15.80	6.57	1.62E-02	-22.62	14.32	1.14E-01	-18.53	36.37	6.10E-01	-16.44	8.14	4.34E-02	2.66	20.27	8.95E-01
cg20919730	-7.86	3.27	1.62E-02	-14.25	5.53	9.97E-03	-2.17	9.44	8.18E-01	-2.66	5.19	6.08E-01	-11.74	8.95	1.90E-01
cg26398044	-10.38	4.32	1.62E-02	-19.35	7.81	1.33E-02	1.87	11.52	8.71E-01	-6.13	7.36	4.05E-01	-12.49	9.41	1.84E-01
cg13042252	43.39	18.04	1.62E-02	44.05	23.15	5.70E-02	20.97	70.66	7.67E-01	122.55	53.73	2.25E-02	11.54	36.15	7.50E-01
cg23696808	24.66	10.26	1.62E-02	26.17	13.40	5.09E-02	31.07	34.16	3.63E-01	-24.99	49.74	6.15E-01	26.98	19.32	1.63E-01

cg02440396	-7.11	2.96	1.62E-02	-11.52	5.52	3.71E-02	-3.31	8.73	7.04E-01	-5.53	4.29	1.97E-01	-6.51	8.42	4.40E-01
cg14381448	11.68	4.86	1.62E-02	13.08	7.52	8.18E-02	20.22	10.04	4.40E-02	-1.28	11.38	9.11E-01	10.34	11.93	3.86E-01
cg04747180	5.67	2.36	1.62E-02	4.00	2.98	1.80E-01	12.12	5.66	3.21E-02	11.14	7.00	1.11E-01	-0.52	6.86	9.40E-01
cg18005693	11.05	4.60	1.62E-02	22.34	12.01	6.30E-02	20.86	37.17	5.75E-01	6.99	5.52	2.06E-01	18.01	12.04	1.35E-01
cg01549929	12.23	5.09	1.62E-02	-2.59	13.36	8.46E-01	19.72	16.02	2.18E-01	14.91	6.14	1.52E-02	5.75	19.55	7.69E-01
cg24109273	-14.03	5.83	1.62E-02	-20.74	10.92	5.75E-02	24.48	52.28	6.40E-01	-15.18	8.23	6.52E-02	-3.93	13.05	7.63E-01
cg12868320	20.23	8.41	1.62E-02	2.74	15.56	8.60E-01	29.80	16.00	6.25E-02	35.93	15.43	1.99E-02	6.67	21.02	7.51E-01
cg02586852	-13.87	5.77	1.62E-02	-14.38	11.83	2.24E-01	-4.24	22.43	8.50E-01	-12.23	7.24	9.11E-02	-39.08	23.25	9.28E-02
cg11822932	7.10	2.95	1.62E-02	5.75	3.78	1.28E-01	11.69	7.13	1.01E-01	10.53	11.05	3.41E-01	5.70	7.74	4.62E-01
cg17774797	39.14	16.28	1.62E-02	44.74	20.12	2.62E-02	-4.47	61.16	9.42E-01	-27.37	76.95	7.22E-01	49.56	33.95	1.44E-01
cg15801433	-11.42	4.75	1.62E-02	-15.08	7.28	3.85E-02	-5.96	10.87	5.84E-01	-10.72	12.87	4.05E-01	-9.75	9.55	3.08E-01
cg03175049	24.96	10.38	1.62E-02	37.21	18.92	4.92E-02	21.06	18.35	2.51E-01	5.82	16.49	7.24E-01	61.94	29.68	3.69E-02
cg23275972	-19.27	8.02	1.62E-02	-25.48	12.36	3.92E-02	0.93	20.54	9.64E-01	-23.90	17.74	1.78E-01	-17.11	17.00	3.14E-01
cg11263507	18.65	7.76	1.62E-02	25.07	22.28	2.60E-01	77.47	91.54	3.97E-01	16.93	8.62	4.95E-02	21.87	31.46	4.87E-01
cg20038187	-10.07	4.19	1.62E-02	-7.96	7.51	2.89E-01	-10.23	9.48	2.81E-01	-9.12	7.49	2.23E-01	-15.20	9.86	1.23E-01
cg22967008	-10.20	4.24	1.62E-02	-6.15	5.97	3.02E-01	-23.98	8.96	7.43E-03	-10.26	13.02	4.31E-01	-5.23	8.79	5.52E-01
cg02062326	11.06	4.60	1.62E-02	25.84	12.89	4.51E-02	8.03	27.53	7.71E-01	10.47	5.20	4.40E-02	-10.80	18.62	5.62E-01
cg09012125	10.66	4.43	1.62E-02	10.27	7.36	1.63E-01	17.76	9.98	7.53E-02	15.63	8.63	7.01E-02	-3.99	10.58	7.06E-01
cg02423817	-7.14	2.97	1.62E-02	-8.09	3.60	2.48E-02	-4.12	6.98	5.55E-01	-3.25	12.07	7.88E-01	-8.85	10.53	4.01E-01
cg25631489	-10.68	4.44	1.63E-02	-14.33	6.30	2.28E-02	-5.93	10.51	5.73E-01	-2.30	17.12	8.93E-01	-9.09	8.78	3.01E-01
cg10625578	36.50	15.19	1.63E-02	55.03	44.36	2.15E-01	177.22	169.87	2.97E-01	38.09	17.01	2.51E-02	-22.58	54.63	6.79E-01
cg26603050	17.74	7.38	1.63E-02	15.54	10.08	1.23E-01	25.41	18.38	1.67E-01	46.54	18.95	1.40E-02	6.03	10.38	5.61E-01
cg26560794	25.76	10.72	1.63E-02	40.78	40.79	3.17E-01	15.52	160.00	9.23E-01	25.11	11.59	3.02E-02	19.52	40.27	6.28E-01
cg09134593	20.25	8.43	1.63E-02	27.46	12.62	2.95E-02	21.27	22.74	3.50E-01	31.33	24.41	1.99E-01	4.52	15.46	7.70E-01
cg00867901	-10.43	4.34	1.63E-02	-13.27	5.99	2.67E-02	-3.71	11.90	7.56E-01	-15.37	12.28	2.11E-01	-4.82	9.32	6.05E-01
cg19112564	-39.62	16.49	1.63E-02	-46.36	50.80	3.61E-01	34.36	237.42	8.85E-01	-39.54	18.53	3.29E-02	-36.68	52.71	4.86E-01
cg11364468	19.45	8.10	1.63E-02	23.66	16.98	1.63E-01	-6.74	33.94	8.43E-01	22.66	11.21	4.33E-02	13.58	18.35	4.59E-01
cg04738237	-12.18	5.07	1.63E-02	-14.88	7.38	4.38E-02	-8.65	11.99	4.71E-01	-5.51	13.11	6.74E-01	-13.98	11.37	2.19E-01
cg17579667	11.97	4.98	1.63E-02	19.31	12.08	1.10E-01	9.82	20.24	6.28E-01	16.63	6.88	1.56E-02	-2.66	10.10	7.92E-01
cg26023087	27.13	11.30	1.63E-02	29.82	16.12	6.44E-02	20.49	32.47	5.28E-01	46.74	30.77	1.29E-01	14.66	22.44	5.14E-01
cg05578398	21.69	9.03	1.63E-02	44.87	31.97	1.61E-01	39.65	120.10	7.41E-01	19.36	9.72	4.63E-02	22.88	40.10	5.68E-01
cg03316237	18.55	7.73	1.63E-02	22.65	10.85	3.69E-02	1.18	18.95	9.50E-01	45.49	21.56	3.48E-02	6.93	16.28	6.70E-01
cg07219667	31.27	13.02	1.63E-02	36.19	35.99	3.15E-01	-27.39	66.46	6.80E-01	32.97	16.17	4.15E-02	34.06	30.48	2.64E-01
cg22521151	19.87	8.27	1.63E-02	20.10	18.28	2.72E-01	13.68	17.15	4.25E-01	17.06	12.85	1.84E-01	37.10	21.49	8.43E-02
cg05649906	-13.20	5.49	1.63E-02	-28.28	15.06	6.04E-02	-22.63	20.17	2.62E-01	-8.50	6.49	1.91E-01	-21.70	19.82	2.74E-01

cg19765175	-13.55	5.64	1.63E-02	-13.86	8.66	1.10E-01	-16.22	15.54	2.97E-01	-11.42	12.94	3.77E-01	-13.23	11.20	2.37E-01
cg02571198	-18.87	7.86	1.63E-02	-9.30	21.99	6.72E-01	-6.96	28.41	8.06E-01	-19.42	9.22	3.51E-02	-44.37	30.11	1.41E-01
cg02858413	39.33	16.38	1.63E-02	20.26	47.97	6.73E-01	-107.57	159.06	4.99E-01	45.70	18.48	1.34E-02	25.34	55.41	6.47E-01
cg00029282	36.38	15.15	1.63E-02	31.76	23.43	1.75E-01	38.41	30.41	2.07E-01	67.84	40.17	9.13E-02	20.45	34.65	5.55E-01
cg23465213	11.88	4.95	1.63E-02	7.20	11.39	5.27E-01	1.49	11.33	8.96E-01	13.85	6.93	4.55E-02	28.84	14.93	5.35E-02
cg18196900	12.88	5.37	1.63E-02	16.19	9.75	9.68E-02	24.79	14.51	8.76E-02	13.10	8.07	1.04E-01	-10.21	15.62	5.13E-01
cg11452221	22.19	9.24	1.63E-02	25.35	14.42	7.87E-02	9.17	25.76	7.22E-01	31.89	22.12	1.49E-01	17.59	17.28	3.09E-01
cg04791477	32.52	13.54	1.64E-02	49.06	20.36	1.60E-02	11.27	53.60	8.33E-01	21.85	31.12	4.83E-01	19.58	24.54	4.25E-01
cg19775718	-11.36	4.73	1.64E-02	-12.93	8.82	1.43E-01	-8.07	12.70	5.25E-01	-17.05	6.51	8.85E-03	7.94	12.63	5.30E-01
cg08287940	5.97	2.49	1.64E-02	6.06	3.31	6.71E-02	9.88	5.96	9.74E-02	4.99	6.85	4.66E-01	1.31	6.94	8.50E-01
cg26289824	-59.31	24.70	1.64E-02	-56.99	30.96	6.57E-02	-254.07	122.91	3.87E-02	-0.35	90.56	9.97E-01	-51.24	49.54	3.01E-01
cg07826522	25.54	10.64	1.64E-02	22.42	19.74	2.56E-01	31.38	23.01	1.73E-01	14.48	18.21	4.27E-01	47.72	27.05	7.77E-02
cg01515574	-7.49	3.12	1.64E-02	-11.02	5.25	3.59E-02	-7.81	7.80	3.16E-01	-8.23	5.86	1.61E-01	-0.10	6.91	9.89E-01
cg06030535	6.07	2.53	1.64E-02	4.07	3.38	2.29E-01	11.68	5.78	4.33E-02	13.38	8.68	1.23E-01	2.58	6.22	6.79E-01
cg13095737	12.99	5.41	1.64E-02	5.31	7.70	4.91E-01	15.17	9.97	1.28E-01	3.74	15.36	8.08E-01	27.78	10.35	7.29E-03
cg16548154	16.11	6.71	1.64E-02	21.88	9.50	2.13E-02	22.10	13.75	1.08E-01	6.46	20.30	7.50E-01	-5.09	17.15	7.67E-01
cg17037355	12.57	5.24	1.64E-02	6.32	5.90	2.84E-01	5.50	9.86	5.77E-01	18.89	14.65	1.97E-01	26.00	8.76	2.99E-03
cg19786983	-11.09	4.62	1.64E-02	-17.93	7.27	1.37E-02	-16.00	11.58	1.67E-01	1.48	9.67	8.78E-01	-7.86	10.09	4.36E-01
cg02611466	17.06	7.11	1.64E-02	12.61	20.39	5.36E-01	34.42	26.05	1.86E-01	16.67	8.27	4.37E-02	9.89	27.96	7.23E-01
cg02279537	23.64	9.85	1.64E-02	38.71	16.66	2.01E-02	-19.83	22.25	3.73E-01	23.55	7.50	1.70E-03	38.49	17.08	2.43E-02
cg21030400	-8.00	3.33	1.64E-02	-5.99	4.41	1.74E-01	-12.69	7.34	8.37E-02	-7.83	11.70	5.03E-01	-9.31	8.81	2.91E-01
cg08410996	19.21	8.00	1.64E-02	6.01	10.02	5.49E-01	43.35	16.86	1.01E-02	16.88	20.08	4.01E-01	23.37	13.33	7.94E-02
cg08626876	-6.68	2.78	1.64E-02	-5.97	5.00	2.32E-01	-1.80	8.44	8.31E-01	-11.21	4.29	8.89E-03	0.58	6.95	9.33E-01
cg11186365	20.47	8.53	1.64E-02	19.36	17.66	2.73E-01	-11.61	44.79	7.96E-01	27.73	10.75	9.90E-03	-10.67	26.83	6.91E-01
cg11863717	11.59	4.83	1.64E-02	23.47	13.56	8.36E-02	40.89	43.51	3.47E-01	9.94	5.55	7.33E-02	5.65	15.00	7.07E-01
cg26362078	-9.72	4.05	1.64E-02	-8.60	6.97	2.17E-01	-3.05	10.57	7.73E-01	-14.71	6.59	2.57E-02	-5.93	10.90	5.86E-01
cg07773434	9.23	3.85	1.64E-02	11.16	5.45	4.05E-02	9.69	8.55	2.57E-01	6.40	10.09	5.26E-01	5.04	9.83	6.08E-01
cg25120290	16.16	6.74	1.64E-02	16.38	14.16	2.48E-01	10.75	42.01	7.98E-01	16.50	8.81	6.12E-02	15.52	16.64	3.51E-01
cg08202895	14.45	6.02	1.64E-02	26.66	13.96	5.61E-02	4.61	16.88	7.85E-01	16.41	8.03	4.09E-02	-2.79	17.16	8.71E-01
cg13644645	17.79	7.42	1.64E-02	22.54	15.56	1.48E-01	3.80	35.84	9.16E-01	18.98	9.18	3.87E-02	1.65	26.65	9.51E-01
cg17926114	51.55	21.48	1.64E-02	58.25	30.10	5.30E-02	19.25	61.92	7.56E-01	102.56	65.54	1.18E-01	32.49	41.91	4.38E-01
cg25509121	-16.85	7.03	1.64E-02	-22.53	18.00	2.11E-01	-27.93	82.43	7.35E-01	-14.56	8.15	7.41E-02	-24.67	22.49	2.73E-01
cg01115274	15.61	6.51	1.64E-02	23.25	14.83	1.17E-01	3.74	23.45	8.73E-01	13.71	8.45	1.05E-01	19.70	17.50	2.60E-01
cg00789671	-17.54	7.31	1.64E-02	-24.34	10.33	1.85E-02	-5.77	20.96	7.83E-01	13.37	22.56	5.54E-01	-22.20	14.01	1.13E-01
cg19302555	-35.28	14.71	1.65E-02	-29.01	18.07	1.08E-01	19.10	51.50	7.11E-01	-77.26	29.67	9.22E-03	-25.61	32.55	4.31E-01

cg09447808	17.69	7.38	1.65E-02	25.09	10.77	1.99E-02	13.96	27.16	6.07E-01	2.77	23.55	9.06E-01	12.89	12.30	2.95E-01
cg11222836	29.05	12.11	1.65E-02	34.01	20.74	1.01E-01	25.89	30.61	3.98E-01	31.17	20.47	1.28E-01	16.33	30.98	5.98E-01
cg03736826	-10.64	4.43	1.65E-02	-7.61	6.83	2.66E-01	-12.08	8.78	1.69E-01	-18.88	13.41	1.59E-01	-10.67	9.57	2.65E-01
cg03227037	12.22	5.10	1.65E-02	12.42	6.97	7.47E-02	23.86	9.74	1.43E-02	5.98	19.74	7.62E-01	-2.50	11.65	8.30E-01
cg11399100	24.17	10.08	1.65E-02	24.63	14.99	1.00E-01	13.65	30.03	6.49E-01	36.00	24.46	1.41E-01	20.29	19.54	2.99E-01
cg20984614	-15.12	6.30	1.65E-02	-24.94	16.49	1.30E-01	5.63	35.21	8.73E-01	-12.31	7.25	8.95E-02	-36.13	24.77	1.45E-01
cg15751117	-14.71	6.13	1.65E-02	-15.72	11.41	1.68E-01	-6.86	16.69	6.81E-01	-13.91	9.57	1.46E-01	-21.30	15.07	1.58E-01
cg15074033	18.95	7.90	1.65E-02	40.56	19.94	4.20E-02	26.47	29.21	3.65E-01	22.92	11.83	5.26E-02	1.29	13.90	9.26E-01
cg18301598	-10.38	4.33	1.65E-02	-23.40	8.29	4.75E-03	-9.38	16.68	5.74E-01	-6.74	4.68	1.50E-01	-3.50	11.28	7.57E-01
cg14509581	26.13	10.90	1.65E-02	46.61	26.13	7.45E-02	49.97	121.95	6.82E-01	27.97	12.86	2.96E-02	-24.51	34.44	4.77E-01
cg07447870	-8.29	3.45	1.65E-02	-13.33	4.83	5.81E-03	-2.49	7.73	7.48E-01	-11.71	12.58	3.52E-01	-0.44	7.47	9.53E-01
cg22846516	-57.91	24.15	1.65E-02	-33.95	34.97	3.32E-01	13.78	147.05	9.25E-01	-127.69	50.83	1.20E-02	-49.07	46.43	2.91E-01
cg04387418	-27.62	11.52	1.65E-02	-8.41	37.53	8.23E-01	-75.76	172.09	6.60E-01	-31.84	12.51	1.09E-02	9.26	49.65	8.52E-01
cg03533561	-16.57	6.91	1.65E-02	-36.27	14.11	1.01E-02	-2.52	14.80	8.65E-01	-11.65	11.99	3.31E-01	-16.42	14.82	2.68E-01
cg18244487	7.76	3.24	1.65E-02	6.35	3.94	1.07E-01	18.29	8.28	2.72E-02	7.01	12.12	5.63E-01	1.84	10.13	8.56E-01
cg17397836	-13.11	5.47	1.65E-02	-17.02	7.75	2.80E-02	-12.26	12.85	3.40E-01	10.95	15.66	4.84E-01	-18.87	12.27	1.24E-01
cg06940309	-37.93	15.82	1.65E-02	-85.66	54.60	1.17E-01	-49.75	176.77	7.78E-01	-40.51	17.38	1.98E-02	39.92	55.87	4.75E-01
cg27371984	22.21	9.26	1.65E-02	21.26	21.63	3.26E-01	27.94	58.59	6.33E-01	24.04	11.93	4.38E-02	16.51	21.33	4.39E-01
cg02767093	-15.89	6.63	1.65E-02	-20.40	10.10	4.33E-02	1.14	19.43	9.53E-01	-5.74	10.46	5.83E-01	-32.58	13.33	1.45E-02
cg00971332	18.68	7.79	1.65E-02	31.70	19.92	1.12E-01	21.75	24.00	3.65E-01	14.03	9.51	1.40E-01	29.92	29.19	3.05E-01
cg07513435	-25.09	10.47	1.65E-02	4.46	22.31	8.42E-01	7.54	61.86	9.03E-01	-35.89	13.50	7.85E-03	-31.43	27.01	2.45E-01
cg15735129	-8.58	3.58	1.65E-02	-18.79	8.65	2.99E-02	0.20	13.32	9.88E-01	-7.51	4.43	9.05E-02	-4.65	10.96	6.71E-01
cg24598712	11.06	4.61	1.65E-02	10.38	6.14	9.06E-02	21.08	11.09	5.74E-02	15.38	13.54	2.56E-01	-1.63	12.08	8.93E-01
cg15470261	-56.13	23.41	1.65E-02	-82.49	31.39	8.59E-03	51.90	159.55	7.45E-01	-9.18	54.40	8.66E-01	-40.74	48.11	3.97E-01
cg07756647	36.76	15.34	1.65E-02	44.32	18.69	1.77E-02	64.39	31.99	4.41E-02	-13.30	28.70	6.43E-01	49.85	28.33	7.85E-02
cg08472449	8.20	3.42	1.65E-02	9.11	4.73	5.43E-02	7.59	6.91	2.72E-01	-7.23	12.22	5.54E-01	14.01	8.74	1.09E-01
cg03715846	10.18	4.25	1.65E-02	13.96	6.21	2.46E-02	6.79	12.44	5.85E-01	7.14	9.68	4.61E-01	6.67	9.00	4.59E-01
cg16411445	-15.60	6.51	1.65E-02	-19.12	8.57	2.56E-02	17.58	25.22	4.86E-01	-33.19	20.02	9.72E-02	-8.89	12.99	4.94E-01
cg00117005	67.57	28.19	1.65E-02	22.38	38.64	5.63E-01	-17.10	123.42	8.90E-01	124.02	45.49	6.40E-03	87.80	53.12	9.84E-02
cg16179674	8.90	3.71	1.65E-02	5.25	4.99	2.92E-01	20.68	9.24	2.52E-02	15.49	9.75	1.12E-01	2.91	9.91	7.69E-01
cg26913155	-15.50	6.47	1.65E-02	-19.83	10.73	6.45E-02	-21.80	14.88	1.43E-01	-0.69	13.95	9.61E-01	-17.30	13.40	1.97E-01
cg06739256	-11.37	4.75	1.65E-02	-27.55	11.42	1.59E-02	-12.69	20.48	5.35E-01	-7.04	5.78	2.23E-01	-12.01	15.10	4.26E-01
cg21740264	-32.09	13.39	1.65E-02	-10.12	33.81	7.65E-01	-89.88	176.33	6.10E-01	-34.25	15.09	2.33E-02	-60.18	59.64	3.13E-01
cg17010118	18.80	7.84	1.65E-02	44.03	21.00	3.60E-02	24.05	22.65	2.88E-01	11.73	9.57	2.20E-01	27.45	29.82	3.57E-01
cg10531637	-10.62	4.43	1.65E-02	-11.40	5.75	4.75E-02	-13.81	10.31	1.80E-01	-4.26	17.52	8.08E-01	-6.54	11.16	5.58E-01

cg19638477	26.84	11.20	1.65E-02	37.29	18.16	4.00E-02	19.50	34.20	5.69E-01	32.05	21.74	1.40E-01	8.35	22.53	7.11E-01
cg22570970	7.91	3.30	1.65E-02	10.28	4.61	2.56E-02	8.45	7.48	2.58E-01	6.30	9.90	5.24E-01	1.58	7.76	8.39E-01
cg14196303	51.58	21.52	1.65E-02	47.45	26.34	7.17E-02	191.82	101.69	5.93E-02	-36.24	102.61	7.24E-01	52.94	43.58	2.24E-01
cg09934326	-46.96	19.59	1.66E-02	-70.26	26.89	8.98E-03	-30.86	65.47	6.37E-01	37.12	75.61	6.23E-01	-30.03	35.07	3.92E-01
cg17705041	10.93	4.56	1.66E-02	14.70	6.23	1.83E-02	11.43	12.26	3.51E-01	13.07	10.26	2.03E-01	-8.65	12.74	4.97E-01
cg14809932	-14.57	6.08	1.66E-02	-28.19	12.78	2.74E-02	-13.51	21.68	5.33E-01	-7.69	8.07	3.40E-01	-21.72	17.06	2.03E-01
cg06350432	98.80	41.23	1.66E-02	93.55	56.01	9.49E-02	48.01	303.43	8.74E-01	87.24	74.91	2.44E-01	152.13	111.56	1.73E-01
cg14959580	-7.13	2.98	1.66E-02	-3.91	4.36	3.71E-01	-6.20	5.49	2.58E-01	-17.53	14.02	2.11E-01	-13.80	6.73	4.03E-02
cg09772075	-8.43	3.52	1.66E-02	-6.93	5.16	1.79E-01	-19.10	8.71	2.83E-02	1.72	9.29	8.53E-01	-10.25	7.38	1.65E-01
cg16926734	12.90	5.38	1.66E-02	1.62	11.48	8.88E-01	3.48	15.87	8.27E-01	17.07	6.89	1.32E-02	32.00	23.29	1.69E-01
cg18241780	-27.73	11.57	1.66E-02	-32.12	14.93	3.14E-02	-9.58	36.73	7.94E-01	58.22	55.16	2.91E-01	-39.06	22.53	8.30E-02
cg14704968	-9.69	4.04	1.66E-02	-12.36	9.46	1.91E-01	-4.73	18.00	7.93E-01	-10.51	5.00	3.55E-02	-2.76	12.07	8.19E-01
cg12614318	11.34	4.73	1.66E-02	8.96	6.97	1.98E-01	7.78	12.54	5.35E-01	32.61	12.95	1.18E-02	7.22	8.27	3.83E-01
cg18753341	73.12	30.52	1.66E-02	83.64	41.65	4.46E-02	195.22	187.49	2.98E-01	-6.53	92.64	9.44E-01	72.38	53.31	1.75E-01
cg01824931	-14.18	5.92	1.66E-02	-11.49	10.31	2.65E-01	-22.70	13.17	8.49E-02	-15.70	12.36	2.04E-01	-9.23	12.09	4.45E-01
cg00960700	13.74	5.73	1.66E-02	13.05	20.66	5.27E-01	33.25	25.75	1.97E-01	14.43	6.94	3.76E-02	6.44	13.14	6.24E-01
cg11130441	15.29	6.39	1.66E-02	17.10	7.88	3.01E-02	21.07	17.61	2.31E-01	-12.08	26.04	6.43E-01	13.34	16.36	4.15E-01
cg23916044	15.09	6.30	1.66E-02	13.62	8.75	1.20E-01	15.40	13.46	2.53E-01	6.33	19.32	7.43E-01	25.52	15.94	1.09E-01
cg03559064	-12.69	5.30	1.66E-02	5.03	14.56	7.30E-01	-18.26	30.58	5.50E-01	-13.51	6.13	2.77E-02	-29.76	17.49	8.88E-02
cg19049152	-11.96	4.99	1.66E-02	-9.87	6.80	1.47E-01	-4.83	11.43	6.72E-01	-33.10	21.88	1.30E-01	-18.37	10.72	8.67E-02
cg11348599	-12.73	5.32	1.66E-02	-13.01	15.96	4.15E-01	-8.61	29.63	7.71E-01	-13.89	5.95	1.95E-02	1.61	22.22	9.42E-01
cg15453345	32.53	13.58	1.66E-02	26.54	15.21	8.10E-02	40.51	75.05	5.89E-01	-26.79	80.68	7.40E-01	76.29	36.10	3.46E-02
cg09684160	-9.66	4.03	1.66E-02	-10.09	5.44	6.33E-02	-9.67	10.04	3.35E-01	-11.90	10.46	2.56E-01	-5.54	10.79	6.07E-01
cg07743231	9.27	3.87	1.66E-02	12.00	6.76	7.58E-02	11.15	7.69	1.47E-01	2.18	8.01	7.85E-01	10.75	8.97	2.31E-01
cg05233899	-6.60	2.76	1.66E-02	-2.94	4.31	4.95E-01	-6.35	5.60	2.57E-01	-11.41	6.98	1.02E-01	-10.79	6.28	8.56E-02
cg26768918	41.77	17.44	1.66E-02	32.95	21.54	1.26E-01	12.40	93.07	8.94E-01	74.69	46.12	1.05E-01	54.44	42.79	2.03E-01
cg25512903	-39.00	16.29	1.66E-02	-49.22	24.64	4.58E-02	-30.12	46.79	5.20E-01	-5.77	38.38	8.80E-01	-48.92	31.83	1.24E-01
cg03366858	18.58	7.76	1.66E-02	14.50	9.22	1.16E-01	74.86	37.58	4.63E-02	20.22	35.19	5.65E-01	20.66	17.34	2.34E-01
cg07702750	23.10	9.65	1.66E-02	19.70	26.97	4.65E-01	18.90	57.90	7.44E-01	19.74	11.34	8.16E-02	47.97	27.85	8.50E-02
cg26192857	50.94	21.28	1.67E-02	60.28	26.69	2.39E-02	46.12	77.12	5.50E-01	75.37	63.57	2.36E-01	3.92	50.62	9.38E-01
cg08977702	-11.30	4.72	1.67E-02	-32.83	14.16	2.05E-02	-0.51	30.74	9.87E-01	-8.42	5.31	1.13E-01	-13.13	17.19	4.45E-01
cg24059694	-32.43	13.54	1.67E-02	-59.50	23.11	1.00E-02	42.04	47.40	3.75E-01	-26.86	10.94	1.41E-02	-44.57	30.34	1.42E-01
cg21205978	-15.38	6.42	1.67E-02	-10.53	8.23	2.01E-01	-34.87	15.60	2.54E-02	5.69	28.43	8.41E-01	-19.64	15.57	2.07E-01
cg20248516	14.72	6.15	1.67E-02	21.36	9.95	3.18E-02	15.22	13.87	2.73E-01	21.41	14.84	1.49E-01	-0.41	12.29	9.73E-01
cg04405075	-15.96	6.67	1.67E-02	-14.93	11.71	2.02E-01	3.34	14.96	8.24E-01	-28.91	14.88	5.21E-02	-21.63	12.68	8.80E-02

cg11576513	23.70	9.90	1.67E-02	9.16	15.60	5.57E-01	-0.36	46.99	9.94E-01	32.87	15.10	2.95E-02	47.83	28.17	8.95E-02
cg27509823	5.12	2.14	1.67E-02	2.20	3.69	5.51E-01	2.49	4.51	5.81E-01	8.20	3.61	2.31E-02	10.53	7.13	1.39E-01
cg12329318	-13.07	5.46	1.67E-02	-17.39	14.99	2.46E-01	-22.29	25.54	3.83E-01	-13.58	6.33	3.18E-02	4.81	19.69	8.07E-01
cg12886494	-12.76	5.33	1.67E-02	-14.83	10.86	1.72E-01	-8.27	13.74	5.47E-01	-11.49	7.77	1.39E-01	-18.37	14.34	2.00E-01
cg08144588	-4.56	1.91	1.67E-02	-5.32	2.68	4.72E-02	-1.78	4.41	6.87E-01	-8.89	8.04	2.69E-01	-4.14	3.80	2.76E-01
cg18975659	-13.38	5.59	1.67E-02	-17.64	11.33	1.19E-01	-5.04	14.53	7.29E-01	-13.02	9.42	1.67E-01	-14.64	11.05	1.85E-01
cg23216122	20.26	8.46	1.67E-02	14.64	18.02	4.16E-01	24.46	17.20	1.55E-01	16.95	12.90	1.89E-01	35.72	25.92	1.68E-01
cg19035993	21.18	8.85	1.67E-02	22.50	23.03	3.29E-01	27.41	34.64	4.29E-01	14.46	10.61	1.73E-01	65.71	29.27	2.47E-02
cg20724476	-8.45	3.53	1.67E-02	-14.72	5.36	6.00E-03	-5.18	7.82	5.08E-01	-5.45	9.84	5.79E-01	-1.29	7.30	8.59E-01
cg05952666	27.62	11.54	1.67E-02	30.60	23.84	1.99E-01	-41.67	133.40	7.55E-01	24.40	14.17	8.50E-02	48.19	37.45	1.98E-01
cg20618826	-14.97	6.26	1.67E-02	-18.90	8.91	3.39E-02	-15.38	14.52	2.89E-01	-8.84	16.23	5.86E-01	-8.60	15.07	5.68E-01
cg10992219	13.13	5.49	1.67E-02	17.70	10.79	1.01E-01	6.93	14.37	6.29E-01	16.28	8.23	4.78E-02	2.01	14.12	8.87E-01
cg14785449	-35.31	14.75	1.67E-02	-88.95	51.16	8.21E-02	-139.12	175.89	4.29E-01	-24.13	16.35	1.40E-01	-76.43	47.80	1.10E-01
cg12894142	15.73	6.57	1.67E-02	26.03	12.56	3.83E-02	-7.92	21.19	7.09E-01	17.41	9.20	5.83E-02	3.94	19.06	8.36E-01
cg25178798	-20.61	8.61	1.67E-02	-63.96	30.75	3.76E-02	45.69	131.08	7.27E-01	-16.89	9.36	7.11E-02	-21.11	32.40	5.15E-01
cg26895571	24.33	10.17	1.67E-02	28.55	19.84	1.50E-01	23.32	22.36	2.97E-01	15.51	16.93	3.60E-01	37.80	24.69	1.26E-01
cg11050601	35.55	14.86	1.67E-02	10.38	51.91	8.41E-01	48.34	43.50	2.66E-01	37.14	17.19	3.07E-02	24.14	63.93	7.06E-01
cg02382400	11.00	4.60	1.67E-02	4.50	7.16	5.30E-01	10.21	8.36	2.22E-01	14.76	14.91	3.22E-01	24.53	10.56	2.02E-02
cg00653790	10.12	4.23	1.67E-02	3.63	4.66	4.36E-01	8.50	7.19	2.38E-01	24.53	8.03	2.26E-03	9.66	6.81	1.56E-01
cg14505299	-19.16	8.01	1.67E-02	-36.11	12.52	3.94E-03	-8.96	17.75	6.14E-01	-4.47	12.02	7.10E-01	-25.77	16.00	1.07E-01
cg23752963	44.77	18.71	1.67E-02	50.91	25.72	4.78E-02	-3.15	76.58	9.67E-01	-2.01	46.08	9.65E-01	74.50	37.70	4.81E-02
cg03742214	42.37	17.70	1.67E-02	33.97	67.38	6.14E-01	-128.17	261.78	6.24E-01	51.31	19.04	7.03E-03	-61.61	71.48	3.89E-01
cg05821464	-14.42	6.02	1.67E-02	-24.79	9.50	9.07E-03	-6.59	12.57	6.00E-01	-13.29	16.45	4.19E-01	-4.92	12.45	6.93E-01
cg25516823	22.77	9.51	1.67E-02	41.62	18.06	2.12E-02	-1.70	37.42	9.64E-01	24.84	10.39	1.68E-02	-13.71	27.06	6.12E-01
cg09219521	26.91	11.24	1.67E-02	37.20	16.20	2.17E-02	5.46	36.99	8.83E-01	47.36	26.85	7.78E-02	0.71	22.46	9.75E-01
cg10831684	-43.79	18.30	1.67E-02	-31.51	29.98	2.93E-01	-28.28	97.36	7.71E-01	-78.35	31.50	1.29E-02	-18.09	36.27	6.18E-01
cg08167110	-9.22	3.85	1.67E-02	-14.94	5.78	9.74E-03	-12.60	9.91	2.04E-01	-0.15	9.44	9.87E-01	-2.73	7.91	7.30E-01
cg07537734	20.85	8.72	1.67E-02	27.60	10.96	1.18E-02	-3.17	25.25	9.00E-01	53.66	28.31	5.81E-02	8.47	15.06	5.74E-01
cg03086003	-9.57	4.00	1.67E-02	-15.96	7.48	3.29E-02	-7.27	8.73	4.05E-01	-5.72	6.82	4.01E-01	-9.47	10.01	3.44E-01
cg06191390	14.18	5.92	1.67E-02	23.00	9.94	2.06E-02	13.76	15.40	3.72E-01	17.32	9.45	6.69E-02	-5.96	13.12	6.50E-01
cg04708313	17.71	7.40	1.67E-02	18.82	15.40	2.22E-01	-8.78	19.85	6.58E-01	20.48	10.72	5.60E-02	31.44	18.91	9.63E-02
cg12117227	-13.95	5.83	1.67E-02	-26.08	9.24	4.78E-03	-10.89	13.45	4.18E-01	-1.60	11.33	8.88E-01	-9.11	13.57	5.02E-01
cg24586685	-9.30	3.89	1.67E-02	-19.41	6.68	3.67E-03	-5.03	10.26	6.24E-01	-4.31	5.12	4.01E-01	-8.35	10.12	4.09E-01
cg27053785	-87.18	36.44	1.67E-02	-77.36	48.64	1.12E-01	188.95	217.74	3.86E-01	-178.88	82.92	3.10E-02	-66.65	78.10	3.93E-01
cg14741685	-32.70	13.67	1.67E-02	-5.36	39.83	8.93E-01	-212.40	134.88	1.15E-01	-35.54	15.38	2.09E-02	-22.24	47.58	6.40E-01

cg18924229	4.20	1.76	1.67E-02	6.13	3.01	4.12E-02	1.58	4.46	7.22E-01	3.20	2.77	2.47E-01	5.68	5.54	3.06E-01
cg00479463	-7.53	3.15	1.67E-02	-9.36	4.26	2.79E-02	-3.36	7.12	6.37E-01	-11.02	10.46	2.92E-01	-4.53	7.73	5.58E-01
cg17580416	-20.09	8.40	1.67E-02	-27.31	14.57	6.08E-02	-6.40	16.64	7.01E-01	-5.36	18.53	7.73E-01	-39.96	18.45	3.03E-02
cg13930557	14.72	6.15	1.68E-02	17.74	9.61	6.50E-02	16.93	10.90	1.20E-01	6.69	18.24	7.14E-01	8.21	15.51	5.97E-01
cg02387368	11.83	4.95	1.68E-02	11.66	6.96	9.40E-02	19.07	10.56	7.10E-02	6.17	15.46	6.90E-01	6.52	11.88	5.83E-01
cg09596674	9.57	4.00	1.68E-02	14.50	5.87	1.35E-02	5.63	9.78	5.65E-01	9.65	10.76	3.70E-01	2.41	8.36	7.73E-01
cg20799547	-37.44	15.65	1.68E-02	-21.59	37.72	5.67E-01	-175.91	194.33	3.65E-01	-35.38	18.09	5.05E-02	-83.71	57.98	1.49E-01
cg08852641	-8.53	3.56	1.68E-02	-8.75	4.79	6.77E-02	0.10	6.21	9.87E-01	-15.96	10.84	1.41E-01	-16.30	7.54	3.06E-02
cg21325475	-16.15	6.75	1.68E-02	-31.91	15.93	4.52E-02	-1.29	19.76	9.48E-01	-17.53	8.86	4.80E-02	-0.86	19.18	9.64E-01
cg14758525	-9.75	4.07	1.68E-02	-4.77	5.79	4.09E-01	-17.65	8.13	2.99E-02	-5.19	14.35	7.18E-01	-14.67	9.82	1.35E-01
cg14063129	21.42	8.95	1.68E-02	23.95	18.39	1.93E-01	10.49	20.43	6.08E-01	23.28	12.81	6.92E-02	28.56	31.21	3.60E-01
cg24543696	18.40	7.69	1.68E-02	17.19	15.05	2.53E-01	29.14	20.24	1.50E-01	15.36	11.93	1.98E-01	18.58	18.20	3.07E-01
cg23366861	22.02	9.21	1.68E-02	52.27	19.80	8.29E-03	0.50	78.31	9.95E-01	14.06	10.98	2.00E-01	13.06	32.80	6.91E-01
cg23893802	54.44	22.76	1.68E-02	67.20	51.56	1.92E-01	71.49	231.59	7.58E-01	43.05	27.09	1.12E-01	114.49	76.02	1.32E-01
cg20934892	22.09	9.24	1.68E-02	37.59	19.30	5.14E-02	-16.22	41.48	6.96E-01	17.87	11.74	1.28E-01	31.46	28.90	2.76E-01
cg02875130	-45.99	19.23	1.68E-02	14.09	64.73	8.28E-01	-178.46	257.45	4.88E-01	-44.78	21.12	3.40E-02	-117.76	69.07	8.82E-02
cg26895804	12.38	5.18	1.68E-02	16.70	7.01	1.73E-02	15.70	13.10	2.31E-01	13.41	19.37	4.89E-01	-0.54	10.85	9.60E-01
cg11754095	35.55	14.87	1.68E-02	45.63	18.75	1.49E-02	51.19	83.18	5.38E-01	-2.52	75.76	9.73E-01	17.69	27.11	5.14E-01
cg02231590	-7.82	3.27	1.68E-02	-7.69	4.40	8.07E-02	-4.98	7.20	4.89E-01	-20.10	10.42	5.37E-02	-3.98	8.63	6.45E-01
cg27060391	-13.09	5.47	1.68E-02	-19.02	9.23	3.93E-02	3.18	11.65	7.85E-01	-17.62	11.47	1.24E-01	-15.46	12.25	2.07E-01
cg15059176	-14.11	5.90	1.68E-02	-12.47	9.19	1.75E-01	-5.78	13.82	6.76E-01	-24.27	13.74	7.73E-02	-15.55	12.54	2.15E-01
cg01269620	17.49	7.31	1.68E-02	26.00	13.18	4.85E-02	27.89	20.50	1.74E-01	5.76	13.16	6.62E-01	16.23	14.46	2.62E-01
cg16674264	-12.92	5.40	1.68E-02	-17.81	9.62	6.42E-02	-14.65	12.98	2.59E-01	-6.90	9.03	4.45E-01	-15.01	13.83	2.78E-01
cg16746455	35.19	14.72	1.68E-02	38.59	18.23	3.42E-02	19.99	59.26	7.36E-01	67.62	67.38	3.16E-01	23.34	30.13	4.39E-01
cg12623852	4.93	2.06	1.68E-02	7.73	3.04	1.11E-02	2.86	4.25	5.02E-01	-2.49	6.24	6.90E-01	4.97	4.63	2.83E-01
cg14593314	37.38	15.63	1.68E-02	35.25	37.39	3.46E-01	17.43	180.38	9.23E-01	35.03	18.12	5.31E-02	68.55	57.91	2.37E-01
cg12030370	14.57	6.09	1.68E-02	14.80	11.19	1.86E-01	-0.35	21.37	9.87E-01	19.52	8.43	2.06E-02	0.19	19.27	9.92E-01
cg07537152	-11.35	4.75	1.68E-02	-18.56	10.20	6.86E-02	-31.41	20.00	1.16E-01	-8.51	6.08	1.61E-01	-3.11	13.85	8.22E-01
cg06515732	12.42	5.19	1.68E-02	20.46	13.19	1.21E-01	29.01	31.09	3.51E-01	11.20	6.00	6.20E-02	0.67	19.95	9.73E-01
cg24428099	22.59	9.45	1.68E-02	15.00	16.95	3.76E-01	22.96	17.00	1.77E-01	40.68	19.36	3.56E-02	8.09	25.03	7.47E-01
cg05192402	-10.49	4.39	1.68E-02	-20.39	9.63	3.43E-02	-5.93	13.01	6.48E-01	-5.72	5.87	3.30E-01	-19.85	12.65	1.17E-01
cg23185774	-18.29	7.65	1.68E-02	-21.80	12.06	7.08E-02	-15.17	15.83	3.38E-01	-18.14	19.08	3.42E-01	-15.06	16.96	3.75E-01
cg20464719	6.05	2.53	1.68E-02	7.46	3.75	4.67E-02	8.35	4.70	7.59E-02	3.82	8.31	6.46E-01	-0.73	6.28	9.08E-01
cg26121561	18.56	7.76	1.68E-02	19.38	9.36	3.84E-02	-8.35	26.04	7.49E-01	42.10	44.54	3.45E-01	24.34	17.70	1.69E-01
cg21497236	25.75	10.77	1.68E-02	41.36	19.34	3.24E-02	-0.94	22.42	9.66E-01	23.27	19.71	2.38E-01	38.55	26.89	1.52E-01

cg12911208	11.25	4.71	1.68E-02	24.06	9.97	1.58E-02	13.49	10.37	1.93E-01	5.33	6.76	4.30E-01	6.05	16.04	7.06E-01
cg15920131	-89.53	37.45	1.68E-02	-115.09	53.26	3.07E-02	-34.61	220.56	8.75E-01	-137.80	70.67	5.12E-02	36.20	84.65	6.69E-01
cg23627022	-25.39	10.62	1.68E-02	-0.91	22.99	9.68E-01	-27.18	48.80	5.78E-01	-38.55	12.69	2.38E-03	3.81	31.11	9.02E-01
cg00171565	-30.69	12.84	1.68E-02	-21.62	15.83	1.72E-01	-96.16	43.68	2.77E-02	-43.32	47.33	3.60E-01	-27.30	30.09	3.64E-01
cg08419450	-10.06	4.21	1.68E-02	-15.66	9.35	9.41E-02	-9.68	15.49	5.32E-01	-5.08	4.98	3.08E-01	-25.69	11.66	2.76E-02
cg10293297	16.88	7.06	1.68E-02	31.33	16.92	6.40E-02	-36.75	41.52	3.76E-01	14.11	8.18	8.47E-02	38.35	31.15	2.18E-01
cg01835635	16.76	7.01	1.68E-02	20.14	17.09	2.39E-01	17.14	21.84	4.33E-01	13.59	8.69	1.18E-01	35.37	25.10	1.59E-01
cg27360685	11.74	4.91	1.68E-02	11.38	6.74	9.13E-02	11.48	13.96	4.11E-01	39.58	21.32	6.34E-02	7.45	9.11	4.14E-01
cg18054172	20.70	8.66	1.68E-02	30.02	14.83	4.30E-02	37.80	21.52	7.90E-02	-2.19	15.45	8.87E-01	24.67	16.27	1.29E-01
cg06951245	11.44	4.78	1.68E-02	15.15	7.98	5.75E-02	24.05	11.85	4.25E-02	3.66	12.14	7.63E-01	4.65	8.43	5.82E-01
cg02644494	12.29	5.14	1.69E-02	12.40	7.81	1.12E-01	12.29	12.59	3.29E-01	0.27	17.54	9.88E-01	15.46	9.20	9.28E-02
cg16199460	-7.58	3.17	1.69E-02	-8.34	5.15	1.05E-01	-8.72	6.71	1.94E-01	-10.35	7.31	1.57E-01	-2.47	6.95	7.23E-01
cg11973479	35.82	14.99	1.69E-02	43.75	18.29	1.68E-02	7.58	72.49	9.17E-01	-26.79	49.46	5.88E-01	44.24	34.04	1.94E-01
cg11539857	-11.87	4.97	1.69E-02	-12.59	7.10	7.64E-02	-13.93	15.48	3.68E-01	-8.19	13.03	5.30E-01	-11.76	9.69	2.25E-01
cg19001703	-34.26	14.34	1.69E-02	-28.03	17.10	1.01E-01	-52.50	47.65	2.71E-01	-55.20	71.83	4.42E-01	-45.57	35.08	1.94E-01
cg03166779	33.92	14.20	1.69E-02	12.50	19.30	5.17E-01	50.10	53.31	3.47E-01	48.27	33.69	1.52E-01	71.42	30.94	2.10E-02
cg13285004	18.43	7.71	1.69E-02	25.36	14.58	8.20E-02	17.38	15.80	2.72E-01	3.31	13.95	8.12E-01	35.08	18.37	5.62E-02
cg10758278	-15.14	6.34	1.69E-02	-22.05	12.26	7.20E-02	-7.80	22.82	7.33E-01	-12.68	8.60	1.40E-01	-15.60	18.81	4.07E-01
cg23528247	6.14	2.57	1.69E-02	5.64	3.60	1.17E-01	7.98	5.88	1.75E-01	9.23	6.51	1.56E-01	2.05	6.85	7.65E-01
cg22241045	-17.21	7.20	1.69E-02	-11.96	19.09	5.31E-01	-32.05	26.83	2.32E-01	-17.10	8.51	4.45E-02	-13.68	27.50	6.19E-01
cg24475171	15.19	6.36	1.69E-02	13.87	7.65	6.99E-02	41.09	24.19	8.94E-02	11.75	24.58	6.33E-01	11.48	15.27	4.52E-01
cg23555120	12.95	5.42	1.69E-02	17.77	8.36	3.35E-02	0.74	13.53	9.56E-01	17.91	17.05	2.94E-01	11.16	9.61	2.45E-01
cg11130955	-8.51	3.56	1.69E-02	-13.85	8.56	1.06E-01	-1.96	18.79	9.17E-01	-8.10	4.31	6.00E-02	-4.69	10.91	6.67E-01
cg06821199	6.55	2.74	1.69E-02	3.85	4.60	4.03E-01	12.15	5.64	3.11E-02	2.46	6.79	7.17E-01	7.75	5.52	1.60E-01
cg27589744	-46.39	19.42	1.69E-02	-56.92	23.91	1.73E-02	13.38	99.47	8.93E-01	3.41	45.31	9.40E-01	-84.09	56.36	1.36E-01
cg07226718	-9.28	3.89	1.69E-02	-10.95	5.29	3.86E-02	-1.14	8.42	8.93E-01	-9.00	13.68	5.11E-01	-14.42	9.50	1.29E-01
cg02996181	11.82	4.95	1.69E-02	7.90	6.94	2.55E-01	25.87	12.71	4.18E-02	9.36	11.32	4.08E-01	14.08	12.84	2.73E-01
cg25090051	12.74	5.33	1.69E-02	14.82	8.02	6.46E-02	8.42	12.50	5.00E-01	13.69	14.14	3.33E-01	11.59	11.05	2.94E-01
cg00299737	-26.55	11.12	1.69E-02	-19.49	15.55	2.10E-01	-0.05	47.43	9.99E-01	-59.62	29.86	4.59E-02	-28.20	20.45	1.68E-01
cg05365960	-40.62	17.01	1.69E-02	-8.29	41.47	8.42E-01	-31.82	202.97	8.75E-01	-49.48	19.74	1.22E-02	-27.62	59.09	6.40E-01
cg20263853	-20.71	8.67	1.69E-02	-29.56	14.69	4.43E-02	-12.19	17.25	4.80E-01	-10.77	18.16	5.53E-01	-28.58	20.98	1.73E-01
cg04001071	11.77	4.93	1.69E-02	8.96	10.55	3.95E-01	1.70	13.53	9.00E-01	16.44	6.57	1.24E-02	4.09	16.60	8.06E-01
cg20972917	30.95	12.96	1.69E-02	28.06	19.69	1.54E-01	26.76	41.65	5.21E-01	64.95	32.08	4.29E-02	18.28	23.38	4.34E-01
cg22588850	-15.84	6.63	1.69E-02	-20.48	10.99	6.24E-02	-13.56	14.64	3.54E-01	-6.10	16.39	7.10E-01	-17.22	12.82	1.79E-01
cg27004521	16.89	7.07	1.69E-02	-2.58	17.97	8.86E-01	12.59	26.13	6.30E-01	20.52	8.63	1.74E-02	25.79	22.36	2.49E-01

cg16875629	21.00	8.79	1.69E-02	13.78	17.06	4.19E-01	57.83	29.69	5.14E-02	13.94	14.97	3.52E-01	24.74	16.01	1.22E-01
cg13308964	-6.46	2.71	1.69E-02	-1.69	4.09	6.79E-01	-12.43	5.66	2.81E-02	-9.93	7.21	1.68E-01	-7.68	6.16	2.13E-01
cg23296369	-13.37	5.60	1.69E-02	-29.53	16.54	7.42E-02	-3.49	22.64	8.78E-01	-11.46	6.41	7.37E-02	-16.85	22.67	4.57E-01
cg23296652	15.22	6.37	1.69E-02	-8.61	16.97	6.12E-01	26.28	11.33	2.04E-02	13.02	8.81	1.39E-01	23.79	18.13	1.89E-01
cg17018767	-12.56	5.26	1.69E-02	-20.20	7.58	7.70E-03	3.22	10.95	7.69E-01	-17.94	14.27	2.09E-01	-10.59	10.18	2.98E-01
cg01148731	-6.67	2.79	1.69E-02	-9.53	4.01	1.75E-02	2.03	6.37	7.50E-01	-5.41	10.03	5.89E-01	-8.22	5.64	1.44E-01
cg27428551	5.04	2.11	1.69E-02	6.06	3.22	6.00E-02	6.64	5.03	1.87E-01	2.77	3.73	4.57E-01	5.10	7.66	5.06E-01
cg08821431	8.65	3.62	1.69E-02	8.34	5.69	1.43E-01	5.62	7.06	4.26E-01	17.26	13.56	2.03E-01	9.84	7.10	1.66E-01
cg01595870	-9.97	4.18	1.69E-02	-10.16	6.76	1.33E-01	-7.09	8.96	4.29E-01	-1.87	10.58	8.60E-01	-17.35	8.42	3.93E-02
cg18123385	-15.74	6.59	1.70E-02	-19.01	9.53	4.61E-02	-12.95	15.81	4.13E-01	-6.88	18.89	7.16E-01	-15.73	13.86	2.57E-01
cg27338466	-16.44	6.88	1.70E-02	-17.13	12.29	1.63E-01	-13.08	19.83	5.10E-01	-11.98	10.29	2.44E-01	-34.88	20.02	8.15E-02
cg17584288	19.17	8.03	1.70E-02	18.74	14.20	1.87E-01	24.93	25.36	3.25E-01	10.80	12.37	3.83E-01	38.62	20.15	5.54E-02
cg14709901	-12.83	5.37	1.70E-02	-6.59	8.28	4.26E-01	-14.18	12.21	2.46E-01	-10.44	12.69	4.11E-01	-26.40	11.84	2.58E-02
cg16421616	7.51	3.14	1.70E-02	-0.69	6.49	9.16E-01	16.11	9.21	8.02E-02	7.74	4.27	6.95E-02	14.36	9.28	1.22E-01
cg20022223	-18.87	7.90	1.70E-02	-16.04	10.62	1.31E-01	-0.19	20.45	9.93E-01	-21.16	27.30	4.38E-01	-38.45	17.14	2.48E-02
cg13563094	21.00	8.80	1.70E-02	17.30	16.80	3.03E-01	34.71	15.40	2.43E-02	8.44	18.05	6.40E-01	18.09	21.83	4.07E-01
cg05225549	24.16	10.12	1.70E-02	21.62	13.31	1.04E-01	25.40	27.71	3.59E-01	78.76	39.07	4.38E-02	13.50	21.49	5.30E-01
cg20709275	-10.59	4.44	1.70E-02	-15.32	11.72	1.91E-01	-44.83	21.92	4.09E-02	-8.65	5.30	1.03E-01	-4.36	13.05	7.38E-01
cg10012878	24.70	10.35	1.70E-02	38.75	20.38	5.72E-02	20.45	20.27	3.13E-01	23.15	17.66	1.90E-01	10.38	27.84	7.09E-01
cg10609068	37.93	15.89	1.70E-02	0.58	35.68	9.87E-01	-41.16	131.45	7.54E-01	50.39	19.04	8.13E-03	36.71	52.82	4.87E-01
cg06363968	-12.37	5.18	1.70E-02	-21.06	7.10	3.01E-03	-6.03	16.75	7.19E-01	-9.69	13.12	4.60E-01	-0.75	9.62	9.38E-01
cg27181005	16.93	7.09	1.70E-02	25.09	19.22	1.92E-01	-2.64	34.97	9.40E-01	12.69	5.67	2.52E-02	54.91	24.63	2.58E-02
cg03387092	-30.57	12.80	1.70E-02	-40.08	18.10	2.68E-02	-15.48	54.62	7.77E-01	-34.11	26.40	1.96E-01	-7.78	27.99	7.81E-01
cg08934208	-16.71	7.00	1.70E-02	-26.17	16.88	1.21E-01	-23.01	39.31	5.58E-01	-16.42	8.11	4.30E-02	14.23	30.66	6.43E-01
cg17161130	10.38	4.35	1.70E-02	15.42	9.30	9.73E-02	27.27	13.04	3.65E-02	6.00	5.95	3.13E-01	5.68	11.78	6.30E-01
cg23591152	25.13	10.53	1.70E-02	39.68	19.20	3.88E-02	21.75	22.39	3.31E-01	19.24	18.22	2.91E-01	13.65	27.72	6.22E-01
cg02450742	-24.96	10.46	1.70E-02	23.79	44.58	5.94E-01	20.98	197.19	9.15E-01	-28.35	11.03	1.02E-02	-19.63	50.05	6.95E-01
cg25007705	8.37	3.51	1.70E-02	8.77	4.66	6.01E-02	13.45	8.48	1.13E-01	3.81	9.93	7.01E-01	4.58	9.44	6.28E-01
cg26546504	-41.59	17.42	1.70E-02	-55.45	33.15	9.44E-02	-81.74	273.05	7.65E-01	-39.94	21.77	6.65E-02	-4.39	62.02	9.44E-01
cg20558112	11.02	4.62	1.70E-02	14.83	7.10	3.68E-02	10.80	10.28	2.93E-01	7.62	10.65	4.74E-01	6.08	10.63	5.68E-01
cg23001918	-9.23	3.87	1.70E-02	-11.89	5.63	3.46E-02	-4.71	9.66	6.26E-01	-8.22	10.90	4.50E-01	-7.56	7.87	3.37E-01
cg00751288	49.91	20.91	1.70E-02	59.20	27.45	3.10E-02	74.46	77.71	3.38E-01	36.66	66.68	5.82E-01	26.30	41.94	5.31E-01
cg14916175	8.43	3.53	1.70E-02	12.64	5.19	1.48E-02	11.96	7.60	1.16E-01	-0.23	11.93	9.84E-01	0.03	7.33	9.97E-01
cg16647921	9.12	3.82	1.70E-02	15.93	9.84	1.05E-01	12.60	15.46	4.15E-01	8.01	4.99	1.08E-01	6.20	8.54	4.68E-01
cg25048344	17.64	7.39	1.70E-02	45.90	17.06	7.14E-03	18.31	24.71	4.59E-01	10.26	8.48	2.26E-01	13.38	13.28	3.14E-01

cg09918512	17.03	7.14	1.70E-02	14.81	21.26	4.86E-01	32.87	63.60	6.05E-01	15.72	7.82	4.43E-02	44.79	35.20	2.03E-01
cg14833621	13.98	5.86	1.70E-02	15.69	11.17	1.60E-01	-2.47	21.10	9.07E-01	14.74	7.85	6.03E-02	18.10	19.47	3.52E-01
cg17507897	-11.63	4.87	1.70E-02	-9.03	9.62	3.48E-01	-11.32	12.23	3.55E-01	-9.92	7.54	1.88E-01	-20.14	11.91	9.08E-02
cg03564793	27.40	11.48	1.70E-02	23.17	13.45	8.50E-02	40.36	19.65	4.00E-02	64.92	25.50	1.09E-02	3.39	15.07	8.22E-01
cg07027513	26.10	10.94	1.70E-02	32.76	15.69	3.68E-02	18.07	32.76	5.81E-01	72.63	39.46	6.57E-02	7.93	19.16	6.79E-01
cg10473842	15.79	6.62	1.70E-02	4.26	11.45	7.10E-01	24.78	13.39	6.42E-02	23.07	13.12	7.88E-02	14.61	16.15	3.66E-01
cg15636365	-54.15	22.70	1.70E-02	-23.17	59.46	6.97E-01	-479.01	265.35	7.10E-02	-52.96	26.09	4.24E-02	-79.71	75.51	2.91E-01
cg19648552	-13.47	5.65	1.70E-02	-16.12	8.82	6.76E-02	-9.66	11.89	4.16E-01	-22.78	13.53	9.22E-02	-3.76	12.94	7.71E-01
cg07152177	-41.49	17.39	1.70E-02	-34.08	47.90	4.77E-01	-42.17	174.74	8.09E-01	-53.35	19.84	7.17E-03	48.79	57.91	4.00E-01
cg12492885	9.02	3.78	1.70E-02	6.10	6.20	3.25E-01	12.57	7.80	1.07E-01	10.51	9.39	2.63E-01	9.08	7.87	2.49E-01
cg25198847	5.58	2.34	1.70E-02	6.62	3.14	3.51E-02	8.76	5.64	1.20E-01	-1.07	6.17	8.62E-01	4.30	6.49	5.08E-01
cg19559587	-14.66	6.15	1.70E-02	-23.36	9.96	1.90E-02	5.71	15.20	7.07E-01	-17.04	12.66	1.79E-01	-12.22	13.11	3.51E-01
cg24536827	-9.35	3.92	1.71E-02	-2.89	9.11	7.51E-01	-17.61	17.65	3.18E-01	-7.74	4.87	1.12E-01	-24.92	11.42	2.90E-02
cg13255542	6.16	2.58	1.71E-02	5.18	3.50	1.39E-01	13.13	6.91	5.74E-02	9.86	5.21	5.82E-02	-1.61	6.09	7.92E-01
cg02700498	-26.93	11.29	1.71E-02	-20.04	14.10	1.55E-01	-45.96	41.39	2.67E-01	-38.41	58.90	5.14E-01	-37.34	22.67	9.96E-02
cg14908653	-6.02	2.53	1.71E-02	-6.80	4.28	1.12E-01	3.21	6.30	6.11E-01	-7.15	4.64	1.23E-01	-10.52	5.72	6.58E-02
cg22865058	25.61	10.74	1.71E-02	10.01	33.39	7.64E-01	87.74	80.53	2.76E-01	24.32	12.15	4.53E-02	41.09	34.30	2.31E-01
cg00946032	40.44	16.95	1.71E-02	48.78	21.58	2.38E-02	62.00	94.24	5.11E-01	-4.32	52.23	9.34E-01	35.82	34.21	2.95E-01
cg21099123	31.51	13.21	1.71E-02	21.36	17.63	2.26E-01	22.10	49.88	6.58E-01	28.45	44.65	5.24E-01	55.09	24.92	2.71E-02
cg05189242	17.90	7.50	1.71E-02	25.92	14.09	6.57E-02	1.06	21.72	9.61E-01	27.45	15.42	7.50E-02	10.87	12.50	3.85E-01
cg01435791	11.48	4.81	1.71E-02	3.72	7.69	6.28E-01	14.82	9.09	1.03E-01	12.22	14.80	4.09E-01	20.66	10.23	4.35E-02
cg06434997	-28.90	12.12	1.71E-02	-20.41	40.57	6.15E-01	210.37	140.30	1.34E-01	-31.13	13.00	1.67E-02	-37.27	41.43	3.68E-01
cg26992245	-8.43	3.54	1.71E-02	-10.97	7.19	1.27E-01	-13.65	14.79	3.56E-01	-5.83	4.75	2.20E-01	-12.06	9.24	1.92E-01
cg00971613	14.69	6.16	1.71E-02	26.70	12.54	3.32E-02	-1.00	19.83	9.60E-01	14.15	8.28	8.75E-02	4.67	18.71	8.03E-01
cg05472468	26.53	11.13	1.71E-02	34.02	23.49	1.47E-01	55.15	45.80	2.29E-01	20.02	15.91	2.08E-01	25.74	23.33	2.70E-01
cg24256786	-9.53	3.99	1.71E-02	-20.83	11.13	6.13E-02	-3.73	18.95	8.44E-01	-8.27	4.64	7.49E-02	-6.44	13.62	6.36E-01
cg21068013	-12.72	5.33	1.71E-02	-20.03	7.70	9.33E-03	-2.20	10.76	8.38E-01	-3.84	17.28	8.24E-01	-12.30	12.56	3.28E-01
cg25436126	-16.56	6.94	1.71E-02	-15.47	18.52	4.04E-01	-2.30	40.28	9.54E-01	-16.07	8.08	4.68E-02	-26.65	22.86	2.44E-01
cg24718866	22.17	9.30	1.71E-02	46.91	24.40	5.45E-02	-58.50	111.83	6.01E-01	15.29	10.63	1.50E-01	48.89	32.22	1.29E-01
cg17834768	9.20	3.86	1.71E-02	6.35	5.26	2.27E-01	15.06	9.42	1.10E-01	20.62	14.56	1.57E-01	8.08	8.17	3.22E-01
cg14524956	-12.84	5.39	1.71E-02	-6.11	8.31	4.62E-01	-11.50	13.17	3.83E-01	-19.65	12.82	1.25E-01	-20.65	11.07	6.21E-02
cg05223210	-13.36	5.60	1.71E-02	-20.55	8.35	1.39E-02	-8.50	13.59	5.32E-01	-6.29	15.81	6.91E-01	-7.38	11.10	5.06E-01
cg21846461	-20.00	8.39	1.71E-02	-28.61	16.28	7.88E-02	-16.35	20.86	4.33E-01	-16.00	13.25	2.27E-01	-19.46	20.21	3.36E-01
cg13266327	7.70	3.23	1.71E-02	5.77	4.53	2.02E-01	14.40	8.30	8.28E-02	10.32	11.03	3.50E-01	6.67	6.39	2.97E-01
cg13328209	-22.12	9.28	1.71E-02	-17.43	14.48	2.29E-01	-8.90	30.46	7.70E-01	-33.79	19.33	8.06E-02	-23.87	17.98	1.84E-01

cg19634332	-69.36	29.09	1.71E-02	-26.42	74.14	7.22E-01	-22.39	257.50	9.31E-01	-80.00	34.74	2.13E-02	-67.49	80.12	4.00E-01
cg08563994	36.98	15.51	1.71E-02	-2.47	28.45	9.31E-01	17.19	82.04	8.34E-01	49.59	17.58	4.78E-03	83.50	55.20	1.30E-01
cg16303923	-10.88	4.57	1.71E-02	-13.43	9.20	1.44E-01	2.44	17.22	8.87E-01	-7.65	6.25	2.21E-01	-24.50	11.81	3.80E-02
cg14124310	-8.63	3.62	1.71E-02	-13.08	5.38	1.50E-02	0.82	9.08	9.28E-01	-9.40	10.50	3.71E-01	-6.39	6.99	3.61E-01
cg24457897	42.53	17.84	1.71E-02	41.94	21.12	4.70E-02	141.95	98.26	1.49E-01	60.00	77.34	4.38E-01	23.61	39.88	5.54E-01
cg00449821	15.07	6.32	1.71E-02	19.04	20.03	3.42E-01	-32.09	93.95	7.33E-01	16.38	6.91	1.78E-02	-6.34	25.88	8.06E-01
cg09575189	-21.13	8.87	1.71E-02	-28.17	15.01	6.05E-02	-11.08	24.16	6.46E-01	-37.70	16.73	2.42E-02	1.73	17.71	9.22E-01
cg23704195	-6.18	2.59	1.71E-02	-7.18	3.68	5.07E-02	-2.77	5.29	6.01E-01	4.53	12.97	7.27E-01	-9.54	5.49	8.26E-02
cg09404516	9.37	3.93	1.71E-02	13.40	5.49	1.46E-02	16.75	10.52	1.12E-01	-0.33	8.92	9.70E-01	1.43	10.08	8.87E-01
cg10314052	-21.92	9.20	1.71E-02	-25.20	12.80	4.90E-02	26.17	41.37	5.27E-01	-13.77	23.41	5.56E-01	-28.85	17.37	9.68E-02
cg27175475	48.17	20.21	1.71E-02	48.51	24.20	4.50E-02	-94.82	133.51	4.78E-01	54.31	100.50	5.89E-01	59.86	41.33	1.48E-01
cg27108362	25.83	10.84	1.71E-02	17.41	32.39	5.91E-01	-78.88	90.20	3.82E-01	25.03	12.21	4.04E-02	61.93	37.04	9.45E-02
cg02146933	-13.41	5.63	1.72E-02	-15.21	14.29	2.87E-01	-14.28	28.79	6.20E-01	-11.86	6.62	7.32E-02	-22.98	19.36	2.35E-01
cg27357181	19.79	8.30	1.72E-02	-11.27	23.12	6.26E-01	26.48	78.68	7.36E-01	22.51	9.31	1.56E-02	47.79	33.00	1.48E-01
cg18327586	9.43	3.96	1.72E-02	4.94	5.31	3.52E-01	15.75	8.42	6.13E-02	18.17	14.23	2.02E-01	12.27	10.32	2.34E-01
cg00778084	-8.55	3.59	1.72E-02	-4.75	7.08	5.03E-01	-5.25	11.70	6.54E-01	-12.22	5.08	1.61E-02	-4.91	9.28	5.97E-01
cg07423198	23.85	10.01	1.72E-02	21.65	19.51	2.67E-01	11.40	110.97	9.18E-01	25.83	12.38	3.70E-02	15.75	36.41	6.65E-01
cg14320054	27.96	11.73	1.72E-02	55.59	31.89	8.13E-02	45.25	70.64	5.22E-01	21.03	14.33	1.42E-01	30.54	28.76	2.88E-01
cg00414835	13.05	5.47	1.72E-02	3.45	7.11	6.28E-01	23.48	12.08	5.20E-02	29.23	16.91	8.38E-02	15.36	9.27	9.75E-02
cg22996783	-12.37	5.19	1.72E-02	-18.54	8.21	2.39E-02	3.74	12.26	7.60E-01	-9.62	13.88	4.88E-01	-15.24	9.79	1.20E-01
cg25969983	23.04	9.67	1.72E-02	2.72	22.74	9.05E-01	20.87	24.52	3.95E-01	34.57	10.56	1.06E-03	-14.36	32.14	6.55E-01
cg22307058	6.29	2.64	1.72E-02	-2.00	9.89	8.40E-01	5.56	4.10	1.75E-01	-5.30	19.85	7.90E-01	8.51	3.75	2.32E-02
cg08962305	-9.98	4.19	1.72E-02	-21.51	10.46	3.97E-02	-10.07	14.43	4.85E-01	-5.97	5.34	2.63E-01	-14.37	11.21	2.00E-01
cg24751773	6.32	2.65	1.72E-02	7.65	3.85	4.68E-02	5.37	5.70	3.46E-01	12.00	7.57	1.13E-01	0.27	6.16	9.65E-01
cg19106237	-10.77	4.52	1.72E-02	-8.39	12.67	5.08E-01	-16.56	29.73	5.77E-01	-11.72	5.14	2.27E-02	-3.49	16.22	8.30E-01
cg15297650	13.35	5.60	1.72E-02	14.44	8.52	9.01E-02	17.48	13.53	1.97E-01	16.23	11.91	1.73E-01	2.95	13.41	8.26E-01
cg13762691	10.67	4.48	1.72E-02	4.44	4.98	3.73E-01	17.25	8.81	5.04E-02	4.64	15.04	7.58E-01	21.57	9.18	1.88E-02
cg22792367	11.69	4.91	1.72E-02	5.57	7.10	4.32E-01	22.62	9.56	1.80E-02	4.73	18.57	7.99E-01	14.51	11.29	1.99E-01
cg15211499	-24.42	10.25	1.72E-02	-50.70	31.11	1.03E-01	13.81	107.71	8.98E-01	-22.75	11.42	4.64E-02	-9.32	37.10	8.02E-01
cg14074285	26.14	10.97	1.72E-02	28.62	15.44	6.38E-02	21.41	35.80	5.50E-01	-20.82	40.47	6.07E-01	34.19	19.16	7.43E-02
cg03220633	20.11	8.44	1.72E-02	30.99	13.02	1.73E-02	38.78	30.70	2.07E-01	22.32	29.05	4.42E-01	5.42	13.03	6.77E-01
cg12827262	17.38	7.30	1.72E-02	13.06	10.43	2.10E-01	13.48	20.15	5.03E-01	55.48	33.86	1.01E-01	19.96	12.64	1.14E-01
cg19180827	9.28	3.90	1.72E-02	6.07	6.39	3.42E-01	21.24	8.95	1.77E-02	4.63	6.90	5.02E-01	12.78	11.29	2.58E-01
cg26049379	-25.24	10.60	1.72E-02	-36.52	16.93	3.10E-02	-20.40	26.86	4.48E-01	-11.09	21.56	6.07E-01	-24.11	23.07	2.96E-01
cg24094550	20.70	8.69	1.72E-02	25.59	15.79	1.05E-01	19.59	21.34	3.58E-01	15.55	17.03	3.61E-01	20.85	16.70	2.12E-01

cg21484315	-13.16	5.53	1.72E-02	-21.42	8.18	8.83E-03	-10.60	12.54	3.98E-01	-5.00	15.96	7.54E-01	-3.17	11.53	7.84E-01
ch.2.85209317F	29.36	12.33	1.72E-02	27.08	15.63	8.32E-02	27.76	46.52	5.51E-01	14.79	61.37	8.10E-01	37.29	23.84	1.18E-01
cg19481355	-10.13	4.25	1.72E-02	-13.80	7.24	5.67E-02	-1.94	10.54	8.54E-01	-11.85	6.85	8.38E-02	-4.58	13.02	7.25E-01
cg11150074	19.40	8.15	1.72E-02	10.50	16.95	5.36E-01	51.80	35.64	1.46E-01	18.81	11.13	9.11E-02	23.17	19.12	2.26E-01
cg14423703	-27.06	11.36	1.72E-02	-4.41	33.63	8.96E-01	-182.21	161.60	2.60E-01	-25.31	12.60	4.46E-02	-74.57	43.51	8.66E-02
cg04349727	-8.94	3.75	1.72E-02	-18.43	7.09	9.37E-03	-9.60	9.13	2.93E-01	-4.65	5.93	4.33E-01	-1.95	9.67	8.40E-01
cg07820742	25.15	10.56	1.72E-02	22.01	17.16	2.00E-01	30.41	28.00	2.77E-01	31.66	32.26	3.26E-01	24.47	17.32	1.58E-01
cg10602135	-21.05	8.84	1.72E-02	-16.53	16.40	3.14E-01	-5.58	42.03	8.94E-01	-25.64	12.01	3.27E-02	-17.09	25.17	4.97E-01
cg10322510	43.64	18.32	1.72E-02	67.19	24.20	5.49E-03	-5.71	59.60	9.24E-01	21.72	56.41	7.00E-01	14.82	38.49	7.00E-01
cg21170304	-15.41	6.47	1.72E-02	-7.19	17.58	6.82E-01	-14.00	21.84	5.22E-01	-16.26	7.74	3.58E-02	-23.69	23.14	3.06E-01
cg06840973	21.12	8.87	1.72E-02	6.39	30.10	8.32E-01	-50.29	65.18	4.40E-01	24.83	9.70	1.04E-02	12.48	36.54	7.33E-01
cg01987516	7.15	3.00	1.72E-02	3.27	5.40	5.45E-01	11.76	7.28	1.06E-01	9.42	5.04	6.17E-02	4.80	7.35	5.14E-01
cg24679890	9.70	4.07	1.72E-02	9.83	6.18	1.12E-01	7.19	8.57	4.01E-01	17.05	9.86	8.39E-02	5.33	9.87	5.89E-01
cg05944661	-12.80	5.38	1.72E-02	-18.00	8.43	3.27E-02	-17.59	12.78	1.69E-01	-9.15	14.71	5.34E-01	-4.04	10.11	6.89E-01
cg06523224	14.11	5.92	1.72E-02	-4.13	17.23	8.11E-01	3.28	36.95	9.29E-01	17.41	6.79	1.03E-02	13.26	19.20	4.90E-01
cg00431447	26.13	10.97	1.72E-02	1.88	20.04	9.25E-01	59.07	39.13	1.31E-01	26.71	15.45	8.40E-02	52.08	27.11	5.48E-02
cg05223441	-18.82	7.90	1.73E-02	-16.82	13.64	2.18E-01	-3.79	17.42	8.28E-01	-32.05	16.77	5.59E-02	-22.30	16.25	1.70E-01
cg10616694	-11.21	4.71	1.73E-02	-18.49	9.12	4.27E-02	-1.32	15.32	9.31E-01	-10.61	6.49	1.02E-01	-5.07	14.05	7.18E-01
cg20541120	18.77	7.88	1.73E-02	21.91	14.83	1.40E-01	-21.79	35.41	5.38E-01	19.52	10.29	5.79E-02	27.25	27.67	3.25E-01
cg03131556	11.01	4.62	1.73E-02	11.15	5.49	4.22E-02	15.87	12.80	2.15E-01	24.21	16.67	1.46E-01	-10.16	16.09	5.28E-01
cg13823136	33.63	14.12	1.73E-02	22.87	18.82	2.24E-01	-70.24	99.21	4.79E-01	52.07	39.04	1.82E-01	53.74	26.42	4.19E-02
cg14143435	-42.77	17.96	1.73E-02	-43.62	33.18	1.89E-01	-121.58	198.76	5.41E-01	-48.47	23.30	3.75E-02	-1.88	55.51	9.73E-01
cg20780546	-13.17	5.53	1.73E-02	-6.58	13.08	6.15E-01	9.40	15.71	5.49E-01	-17.98	7.09	1.13E-02	-21.20	13.97	1.29E-01
cg15007228	17.70	7.44	1.73E-02	15.99	10.79	1.38E-01	23.99	16.94	1.57E-01	14.73	20.36	4.69E-01	17.71	16.68	2.88E-01
cg20725907	8.03	3.37	1.73E-02	-1.03	8.25	9.01E-01	8.36	8.07	3.00E-01	8.49	4.46	5.72E-02	21.75	11.44	5.72E-02
cg22803410	20.15	8.46	1.73E-02	18.62	14.66	2.04E-01	20.80	15.47	1.79E-01	20.44	18.50	2.69E-01	21.75	21.26	3.06E-01
cg00176638	-7.34	3.08	1.73E-02	-9.08	5.77	1.16E-01	-1.67	9.15	8.55E-01	-7.21	4.41	1.02E-01	-9.17	9.15	3.16E-01
cg23008352	-13.52	5.68	1.73E-02	-4.79	10.67	6.54E-01	-11.85	15.32	4.39E-01	-12.82	9.70	1.86E-01	-25.98	11.68	2.61E-02
cg23476065	-10.51	4.41	1.73E-02	-10.47	5.96	7.89E-02	-13.18	10.13	1.93E-01	-0.79	17.81	9.64E-01	-11.05	9.86	2.62E-01
cg01751605	38.66	16.24	1.73E-02	36.66	18.98	5.34E-02	-29.45	48.54	5.44E-01	75.91	27.84	6.41E-03	29.34	32.75	3.70E-01
cg18655958	-39.81	16.72	1.73E-02	-58.23	28.67	4.22E-02	-193.12	188.81	3.06E-01	-27.94	22.76	2.20E-01	-30.32	50.01	5.44E-01
cg09763373	7.28	3.06	1.73E-02	5.62	3.60	1.19E-01	13.12	8.10	1.05E-01	20.77	12.59	9.89E-02	1.74	10.93	8.74E-01
cg26227957	7.17	3.01	1.73E-02	4.89	4.08	2.31E-01	12.72	6.80	6.13E-02	13.90	7.82	7.55E-02	-0.54	9.10	9.52E-01
cg12213062	14.78	6.21	1.73E-02	14.62	15.32	3.40E-01	27.75	18.56	1.35E-01	12.52	8.74	1.52E-01	13.45	13.25	3.10E-01
cg09463882	28.95	12.16	1.73E-02	42.67	32.94	1.95E-01	-1.83	87.05	9.83E-01	28.13	13.93	4.35E-02	21.07	42.48	6.20E-01

cg14063809	37.10	15.59	1.73E-02	45.98	18.61	1.35E-02	63.06	79.96	4.30E-01	5.71	66.94	9.32E-01	10.36	34.34	7.63E-01
cg09099500	-12.89	5.41	1.73E-02	-24.97	16.47	1.29E-01	-24.46	27.68	3.77E-01	-9.64	6.09	1.13E-01	-25.89	21.58	2.30E-01
cg22242614	-11.32	4.76	1.73E-02	-3.90	12.05	7.46E-01	-12.07	17.98	5.02E-01	-12.42	6.00	3.86E-02	-14.17	12.44	2.55E-01
cg07962303	10.20	4.29	1.73E-02	11.49	6.63	8.30E-02	13.55	12.41	2.75E-01	11.35	8.09	1.60E-01	3.24	10.06	7.47E-01
cg22978515	9.67	4.06	1.73E-02	2.25	6.08	7.11E-01	12.91	6.62	5.13E-02	8.24	15.62	5.98E-01	20.96	9.49	2.72E-02
cg10359230	11.12	4.67	1.73E-02	9.64	5.93	1.04E-01	11.87	13.87	3.92E-01	4.80	25.03	8.48E-01	15.68	9.73	1.07E-01
cg07584558	-30.63	12.87	1.73E-02	-28.76	17.36	9.76E-02	-20.62	38.79	5.95E-01	-6.62	34.03	8.46E-01	-58.82	28.98	4.24E-02
cg01587190	-15.31	6.44	1.73E-02	-8.92	14.27	5.32E-01	12.92	56.07	8.18E-01	-16.34	7.87	3.78E-02	-23.86	18.99	2.09E-01
cg20902146	-24.25	10.19	1.73E-02	-3.49	27.72	9.00E-01	18.93	109.83	8.63E-01	-27.70	11.45	1.56E-02	-31.14	40.05	4.37E-01
cg16231500	39.76	16.71	1.73E-02	42.18	26.06	1.06E-01	26.56	44.04	5.46E-01	60.17	50.75	2.36E-01	35.87	28.81	2.13E-01
cg21427902	-11.73	4.93	1.73E-02	-24.31	9.04	7.18E-03	-1.26	12.67	9.21E-01	-6.48	5.78	2.62E-01	-17.25	11.92	1.48E-01
cg03082523	-12.05	5.06	1.73E-02	-12.95	7.05	6.64E-02	-16.95	12.90	1.89E-01	-1.72	14.54	9.06E-01	-12.21	11.08	2.70E-01
cg20897046	-35.55	14.94	1.74E-02	-31.11	20.79	1.35E-01	17.60	50.62	7.28E-01	-26.89	46.27	5.61E-01	-62.37	27.65	2.41E-02
cg24033067	-32.96	13.85	1.74E-02	-38.85	16.62	1.94E-02	40.13	58.67	4.94E-01	-78.63	51.13	1.24E-01	-13.83	33.00	6.75E-01
cg16840547	8.22	3.46	1.74E-02	1.73	5.57	7.56E-01	12.93	7.26	7.51E-02	14.81	6.60	2.49E-02	5.03	10.25	6.24E-01
cg23704703	35.32	14.85	1.74E-02	42.85	27.45	1.18E-01	46.11	57.25	4.21E-01	29.58	21.10	1.61E-01	34.69	38.96	3.73E-01
cg12184994	-25.57	10.75	1.74E-02	-22.95	13.82	9.68E-02	-23.92	40.00	5.50E-01	-31.92	26.77	2.33E-01	-29.81	26.75	2.65E-01
cg23385395	5.82	2.44	1.74E-02	4.91	3.11	1.15E-01	4.59	5.84	4.32E-01	11.35	10.15	2.64E-01	8.83	6.31	1.61E-01
cg12624721	-24.88	10.46	1.74E-02	-39.44	14.47	6.42E-03	16.17	25.13	5.20E-01	-29.94	19.53	1.25E-01	-23.66	18.88	2.10E-01
cg01586959	-13.43	5.64	1.74E-02	-14.36	7.17	4.52E-02	-37.30	17.89	3.70E-02	5.17	27.63	8.52E-01	-4.32	11.54	7.08E-01
cg00082833	-64.35	27.05	1.74E-02	-67.90	33.24	4.11E-02	160.66	161.08	3.19E-01	-49.39	70.34	4.83E-01	-102.89	67.37	1.27E-01
cg09060469	-13.76	5.79	1.74E-02	-31.47	19.23	1.02E-01	8.60	27.80	7.57E-01	-11.99	6.55	6.71E-02	-22.62	19.85	2.54E-01
cg11518846	21.60	9.08	1.74E-02	32.22	24.31	1.85E-01	17.80	46.19	7.00E-01	22.20	11.33	5.02E-02	12.04	21.40	5.74E-01
cg22284311	-14.13	5.94	1.74E-02	-16.03	13.11	2.21E-01	-34.51	15.86	2.96E-02	-6.30	8.04	4.33E-01	-23.54	18.03	1.92E-01
cg23198568	39.76	16.72	1.74E-02	32.33	22.43	1.49E-01	50.95	46.14	2.70E-01	53.69	53.11	3.12E-01	45.71	36.11	2.06E-01
cg10409560	-9.91	4.17	1.74E-02	-3.31	11.71	7.77E-01	-10.62	23.00	6.44E-01	-9.93	4.77	3.73E-02	-20.30	15.03	1.77E-01
cg16216212	-14.38	6.05	1.74E-02	-19.61	10.43	6.02E-02	10.35	14.34	4.71E-01	-19.95	6.71	2.95E-03	-13.38	15.55	3.90E-01
cg02996364	20.69	8.70	1.74E-02	26.87	15.90	9.11E-02	-2.88	19.14	8.81E-01	25.88	13.99	6.43E-02	30.08	26.52	2.57E-01
cg07549278	19.53	8.21	1.74E-02	13.46	20.17	5.05E-01	25.07	27.81	3.67E-01	14.51	11.09	1.91E-01	35.93	18.39	5.08E-02
cg11674130	17.16	7.21	1.74E-02	19.52	9.63	4.26E-02	4.71	22.90	8.37E-01	13.08	30.47	6.68E-01	17.64	13.56	1.93E-01
cg05305099	20.58	8.66	1.74E-02	17.70	18.20	3.31E-01	17.70	21.05	4.00E-01	20.29	12.43	1.03E-01	31.31	25.02	2.11E-01
cg24869815	11.31	4.76	1.74E-02	10.72	6.44	9.63E-02	14.48	12.84	2.59E-01	17.24	10.74	1.08E-01	0.85	13.63	9.50E-01
cg19294125	36.06	15.16	1.74E-02	21.43	25.50	4.01E-01	55.47	98.63	5.74E-01	50.92	20.74	1.41E-02	-0.58	51.04	9.91E-01
cg22588275	-15.98	6.72	1.74E-02	-4.05	17.27	8.15E-01	-13.86	34.71	6.90E-01	-16.51	8.09	4.13E-02	-28.50	19.31	1.40E-01
cg09684112	10.37	4.36	1.74E-02	15.47	7.60	4.17E-02	11.15	9.88	2.59E-01	7.33	8.99	4.15E-01	5.71	8.90	5.21E-01

cg12503190	36.23	15.24	1.74E-02	36.70	33.24	2.70E-01	-91.97	130.81	4.82E-01	42.83	18.80	2.28E-02	13.76	43.98	7.54E-01
cg03903296	-13.17	5.54	1.74E-02	-12.74	8.80	1.47E-01	-9.10	12.99	4.84E-01	-15.21	12.57	2.26E-01	-15.43	11.62	1.84E-01
cg02969426	6.41	2.69	1.74E-02	5.44	4.00	1.74E-01	8.88	7.48	2.36E-01	7.85	4.85	1.06E-01	3.38	8.19	6.80E-01
cg16248201	38.27	16.10	1.74E-02	-21.76	47.39	6.46E-01	-29.97	182.67	8.70E-01	43.60	17.92	1.50E-02	83.39	60.83	1.70E-01
cg13479215	-10.97	4.61	1.74E-02	-19.91	9.87	4.38E-02	-16.08	15.12	2.88E-01	-7.69	6.11	2.08E-01	-6.24	13.46	6.43E-01
cg03997139	10.35	4.35	1.74E-02	11.61	6.65	8.06E-02	22.15	11.66	5.75E-02	1.40	15.50	9.28E-01	6.15	7.32	4.01E-01
cg18297491	26.63	11.20	1.74E-02	29.41	12.38	1.75E-02	102.34	75.85	1.77E-01	27.04	53.11	6.11E-01	-7.81	33.12	8.14E-01
cg02747254	9.35	3.93	1.74E-02	9.24	5.85	1.14E-01	9.47	7.28	1.93E-01	13.08	12.69	3.02E-01	7.20	9.85	4.65E-01
cg21439539	-38.86	16.35	1.74E-02	8.27	36.06	8.19E-01	-15.26	181.96	9.33E-01	-52.11	19.16	6.55E-03	-42.86	67.37	5.25E-01
cg10087172	27.12	11.41	1.74E-02	20.31	32.69	5.34E-01	49.25	125.13	6.94E-01	29.27	12.77	2.19E-02	12.23	42.58	7.74E-01
cg18463001	-31.88	13.41	1.74E-02	-40.80	17.99	2.33E-02	28.21	70.77	6.90E-01	18.91	80.17	8.14E-01	-28.26	21.74	1.94E-01
cg16345171	-10.94	4.60	1.74E-02	-19.73	6.66	3.07E-03	-7.35	9.77	4.52E-01	4.11	11.31	7.16E-01	-10.26	7.37	1.64E-01
cg06355129	6.48	2.73	1.74E-02	3.59	4.13	3.85E-01	8.54	5.30	1.07E-01	16.64	8.56	5.20E-02	4.89	6.12	4.24E-01
cg14678774	13.97	5.88	1.74E-02	18.98	9.88	5.47E-02	6.19	15.41	6.88E-01	10.60	11.33	3.50E-01	15.09	12.19	2.16E-01
cg26226511	25.54	10.74	1.74E-02	34.05	15.27	2.57E-02	32.22	30.87	2.97E-01	-2.71	29.44	9.27E-01	20.51	21.47	3.39E-01
cg13581582	15.88	6.68	1.75E-02	12.95	12.64	3.06E-01	-26.30	32.45	4.18E-01	17.06	9.55	7.39E-02	26.66	15.40	8.34E-02
cg18633600	14.88	6.26	1.75E-02	20.66	10.14	4.15E-02	29.71	15.85	6.08E-02	3.86	12.95	7.66E-01	6.39	13.09	6.25E-01
cg14029856	-10.30	4.33	1.75E-02	-9.58	7.54	2.04E-01	-0.35	9.07	9.69E-01	-13.91	8.18	8.89E-02	-19.63	10.82	6.97E-02
cg21611775	-32.69	13.75	1.75E-02	-33.50	19.42	8.45E-02	37.55	52.02	4.70E-01	-50.61	29.05	8.15E-02	-35.05	30.41	2.49E-01
cg01947138	-37.57	15.81	1.75E-02	-35.95	20.71	8.27E-02	-19.20	42.32	6.50E-01	-34.00	52.40	5.16E-01	-58.06	36.54	1.12E-01
cg09929879	32.40	13.63	1.75E-02	27.37	21.15	1.96E-01	2.20	39.01	9.55E-01	29.83	27.27	2.74E-01	62.62	29.57	3.42E-02
cg13269166	-33.36	14.03	1.75E-02	-35.68	18.35	5.19E-02	-69.14	38.03	6.91E-02	-26.67	39.59	5.00E-01	1.80	35.84	9.60E-01
cg01267797	8.69	3.65	1.75E-02	8.36	4.55	6.59E-02	18.44	9.58	5.42E-02	3.97	15.82	8.02E-01	2.50	9.30	7.88E-01
cg04522866	16.16	6.80	1.75E-02	15.97	9.58	9.56E-02	16.65	15.13	2.71E-01	19.05	18.07	2.92E-01	13.46	17.37	4.38E-01
cg08687751	23.80	10.01	1.75E-02	37.33	20.71	7.15E-02	-3.99	25.16	8.74E-01	19.20	11.64	9.90E-02	63.05	31.60	4.60E-02
cg22379207	-36.43	15.33	1.75E-02	-39.47	17.06	2.07E-02	-189.77	123.87	1.26E-01	4.57	76.34	9.52E-01	-13.53	41.38	7.44E-01
cg01617280	-9.11	3.83	1.75E-02	-14.37	7.57	5.77E-02	-14.43	13.74	2.94E-01	-5.81	5.21	2.65E-01	-9.29	10.85	3.92E-01
cg18034719	16.73	7.04	1.75E-02	24.17	12.40	5.13E-02	9.74	13.64	4.75E-01	16.38	13.32	2.19E-01	13.44	19.37	4.88E-01
cg24054668	13.82	5.82	1.75E-02	11.20	16.01	4.84E-01	18.06	39.48	6.47E-01	16.00	6.58	1.51E-02	-8.10	22.64	7.20E-01
cg07054895	12.83	5.40	1.75E-02	18.69	11.74	1.12E-01	15.46	21.72	4.77E-01	7.60	5.58	1.73E-01	40.36	18.80	3.18E-02
cg06721806	-14.42	6.07	1.75E-02	-2.47	9.67	7.98E-01	-22.34	12.26	6.83E-02	-19.41	14.45	1.79E-01	-24.66	14.14	8.11E-02
cg11243995	-10.86	4.57	1.75E-02	-11.96	9.49	2.08E-01	-4.95	12.67	6.96E-01	-12.98	6.40	4.26E-02	-6.47	12.77	6.12E-01
cg10880928	8.86	3.73	1.75E-02	6.87	4.89	1.60E-01	10.45	6.79	1.24E-01	20.41	7.62	7.38E-03	0.13	7.12	9.85E-01
cg18739577	-21.95	9.24	1.75E-02	4.89	33.42	8.84E-01	-163.86	160.85	3.08E-01	-25.88	9.82	8.38E-03	31.77	49.11	5.18E-01
cg24217936	42.87	18.04	1.75E-02	81.69	55.18	1.39E-01	99.31	236.50	6.75E-01	43.13	20.07	3.16E-02	-16.02	63.93	8.02E-01

cg05203213	-12.96	5.45	1.75E-02	-11.02	7.79	1.57E-01	-18.37	14.32	2.00E-01	-2.83	17.39	8.71E-01	-17.34	10.58	1.01E-01
cg13470069	-7.48	3.15	1.75E-02	-4.50	4.97	3.66E-01	-5.30	6.90	4.42E-01	-14.89	7.48	4.66E-02	-9.03	6.79	1.83E-01
cg22313574	10.01	4.21	1.75E-02	14.03	5.97	1.89E-02	12.36	10.37	2.33E-01	8.25	13.76	5.49E-01	0.90	8.54	9.16E-01
cg09136878	6.49	2.73	1.75E-02	5.94	3.77	1.15E-01	7.74	5.38	1.50E-01	11.27	9.86	2.53E-01	3.64	7.32	6.19E-01
cg07186962	8.25	3.47	1.75E-02	9.80	4.47	2.84E-02	8.96	8.74	3.05E-01	13.48	10.65	2.06E-01	-3.80	9.52	6.90E-01
cg08116724	22.37	9.41	1.75E-02	25.12	14.54	8.41E-02	47.91	30.08	1.11E-01	25.77	22.36	2.49E-01	8.44	17.02	6.20E-01
cg00312009	28.91	12.17	1.75E-02	48.19	26.44	6.84E-02	47.45	59.80	4.27E-01	18.52	15.24	2.24E-01	44.95	36.68	2.20E-01
cg02792780	10.55	4.44	1.75E-02	12.95	5.82	2.62E-02	17.28	12.44	1.65E-01	3.68	10.82	7.34E-01	1.64	12.66	8.97E-01
cg13376199	-12.81	5.39	1.75E-02	-19.42	7.83	1.31E-02	-16.07	13.39	2.30E-01	6.87	13.95	6.22E-01	-9.42	11.66	4.19E-01
cg13462129	17.59	7.40	1.75E-02	24.10	11.02	2.87E-02	-1.33	20.44	9.48E-01	40.66	29.79	1.72E-01	12.29	12.41	3.22E-01
cg14186245	14.59	6.14	1.75E-02	16.91	10.17	9.63E-02	19.11	13.93	1.70E-01	5.63	12.64	6.56E-01	16.51	13.58	2.24E-01
cg12353549	-19.21	8.09	1.75E-02	-9.44	22.45	6.74E-01	10.75	92.64	9.08E-01	-20.94	9.07	2.10E-02	-21.01	31.05	4.99E-01
cg19844325	35.00	14.73	1.75E-02	9.29	42.74	8.28E-01	50.72	106.40	6.34E-01	34.01	16.57	4.01E-02	84.98	55.33	1.25E-01
cg17595347	20.94	8.81	1.75E-02	-6.69	23.96	7.80E-01	56.51	155.07	7.16E-01	27.05	9.71	5.34E-03	-16.80	45.58	7.12E-01
cg10778780	-13.10	5.52	1.75E-02	-23.50	11.31	3.77E-02	-0.96	12.72	9.40E-01	-10.78	8.22	1.90E-01	-20.02	15.66	2.01E-01
cg05710512	19.47	8.19	1.75E-02	8.98	18.45	6.27E-01	38.68	36.90	2.94E-01	21.92	10.03	2.89E-02	13.46	27.88	6.29E-01
cg10372920	10.97	4.62	1.75E-02	4.02	4.57	3.79E-01	4.73	6.72	4.82E-01	25.36	7.73	1.03E-03	14.02	6.24	2.47E-02
cg07320646	22.43	9.44	1.75E-02	0.29	31.80	9.93E-01	-63.88	129.99	6.23E-01	26.72	10.29	9.42E-03	3.79	37.25	9.19E-01
cg12082609	11.20	4.72	1.75E-02	14.81	6.14	1.60E-02	9.61	10.87	3.77E-01	18.46	9.48	5.16E-02	-6.19	10.99	5.73E-01
cg12854656	16.22	6.83	1.75E-02	32.23	20.39	1.14E-01	17.32	25.86	5.03E-01	14.46	7.78	6.29E-02	5.23	31.55	8.68E-01
cg02079551	-13.85	5.83	1.75E-02	-26.27	16.33	1.08E-01	9.30	27.96	7.40E-01	-12.54	6.77	6.39E-02	-18.37	19.77	3.53E-01
cg25096368	15.57	6.56	1.75E-02	9.70	10.16	3.40E-01	21.56	12.27	7.89E-02	22.42	20.82	2.82E-01	15.86	14.69	2.81E-01
cg01013054	11.83	4.98	1.75E-02	18.86	7.95	1.77E-02	14.45	9.10	1.12E-01	-6.71	14.34	6.40E-01	4.87	11.50	6.72E-01
cg00365963	23.79	10.02	1.75E-02	48.69	20.36	1.68E-02	7.65	23.02	7.40E-01	15.35	14.67	2.95E-01	33.22	31.28	2.88E-01
cg01721204	29.92	12.60	1.75E-02	3.59	43.88	9.35E-01	131.14	130.86	3.16E-01	27.76	13.91	4.60E-02	63.77	42.31	1.32E-01
cg17230002	12.67	5.33	1.75E-02	10.62	6.03	7.79E-02	24.54	11.07	2.66E-02	22.41	11.83	5.83E-02	-2.54	10.82	8.14E-01
cg20916500	41.12	17.31	1.75E-02	43.84	23.15	5.82E-02	44.45	55.09	4.20E-01	11.52	61.34	8.51E-01	43.04	33.80	2.03E-01
cg00966763	-10.05	4.23	1.75E-02	-11.95	6.69	7.39E-02	-11.14	10.09	2.70E-01	-7.73	9.64	4.23E-01	-7.87	8.81	3.72E-01
cg18440692	-18.18	7.66	1.75E-02	-25.59	9.49	6.99E-03	-11.11	26.30	6.73E-01	33.15	44.86	4.60E-01	-6.55	15.81	6.78E-01
cg12160586	12.05	5.08	1.76E-02	15.31	7.08	3.07E-02	20.16	14.45	1.63E-01	3.07	15.79	8.46E-01	5.35	9.95	5.91E-01
cg27238887	18.65	7.85	1.76E-02	31.88	14.36	2.65E-02	2.25	13.83	8.71E-01	16.75	16.22	3.02E-01	31.05	20.71	1.34E-01
cg00867406	-20.20	8.51	1.76E-02	-27.98	11.98	1.95E-02	-4.67	38.05	9.02E-01	-25.43	17.05	1.36E-01	2.44	19.19	8.99E-01
cg14912723	22.65	9.54	1.76E-02	11.67	26.97	6.65E-01	54.47	141.94	7.01E-01	27.03	10.63	1.10E-02	-12.34	37.20	7.40E-01
cg22490000	-39.44	16.61	1.76E-02	-30.79	21.48	1.52E-01	-82.63	53.78	1.24E-01	-19.38	43.12	6.53E-01	-64.83	41.71	1.20E-01
cg09387486	29.18	12.29	1.76E-02	29.61	15.09	4.97E-02	1.84	70.62	9.79E-01	20.65	46.46	6.57E-01	34.02	25.29	1.79E-01

cg23163783	41.83	17.62	1.76E-02	50.23	20.77	1.56E-02	-47.63	88.88	5.92E-01	14.38	88.63	8.71E-01	34.72	39.20	3.76E-01
cg17072805	16.73	7.05	1.76E-02	28.29	11.39	1.30E-02	-5.26	18.12	7.71E-01	23.72	11.94	4.70E-02	6.38	12.99	6.23E-01
cg20516176	21.81	9.18	1.76E-02	13.92	21.91	5.25E-01	19.01	50.92	7.09E-01	20.69	10.99	5.98E-02	45.85	30.00	1.26E-01
cg26151477	22.76	9.59	1.76E-02	39.00	26.86	1.47E-01	15.50	46.66	7.40E-01	18.53	11.03	9.29E-02	41.97	35.07	2.31E-01
cg12010173	-7.38	3.11	1.76E-02	-23.09	9.58	1.60E-02	-6.25	5.21	2.30E-01	-6.02	4.65	1.96E-01	-2.93	7.80	7.07E-01
cg17725129	-9.27	3.91	1.76E-02	-1.16	7.10	8.70E-01	-12.22	14.71	4.06E-01	-16.60	6.17	7.12E-03	-6.23	8.21	4.48E-01
cg12408189	-26.24	11.05	1.76E-02	-38.23	22.33	8.69E-02	-91.20	100.27	3.63E-01	-23.05	14.09	1.02E-01	-12.44	30.94	6.88E-01
cg01100068	30.57	12.88	1.76E-02	34.03	19.05	7.41E-02	14.79	36.37	6.84E-01	26.95	33.28	4.18E-01	34.09	24.87	1.70E-01
cg08742502	-6.19	2.61	1.76E-02	-4.84	3.59	1.77E-01	-12.06	5.76	3.62E-02	4.76	9.69	6.23E-01	-7.73	5.91	1.91E-01
cg16647364	-54.92	23.13	1.76E-02	-60.41	32.07	5.96E-02	32.72	153.52	8.31E-01	-94.42	51.99	6.93E-02	-21.38	45.45	6.38E-01
cg21618733	-49.10	20.68	1.76E-02	-60.80	25.81	1.85E-02	-24.63	73.55	7.38E-01	40.43	84.88	6.34E-01	-47.88	44.16	2.78E-01
cg14884902	-13.73	5.79	1.76E-02	-6.86	15.41	6.56E-01	-16.53	17.35	3.41E-01	-12.66	7.21	7.91E-02	-26.68	17.93	1.37E-01
cg08300570	5.89	2.48	1.76E-02	5.71	3.29	8.28E-02	8.00	6.03	1.84E-01	1.30	6.73	8.46E-01	8.89	7.03	2.06E-01
cg09232068	-55.15	23.23	1.76E-02	-49.62	28.82	8.51E-02	1.06	110.85	9.92E-01	5.30	100.92	9.58E-01	-91.74	46.17	4.69E-02
cg22258863	17.90	7.54	1.76E-02	15.60	20.71	4.51E-01	84.22	54.99	1.26E-01	19.21	8.98	3.25E-02	4.93	19.89	8.04E-01
cg13209113	-24.24	10.21	1.76E-02	-32.44	14.26	2.29E-02	12.52	19.74	5.26E-01	-26.46	14.17	6.17E-02	-41.68	17.27	1.58E-02
cg06716568	-37.44	15.78	1.76E-02	-50.10	20.38	1.40E-02	6.31	43.57	8.85E-01	-22.03	62.49	7.24E-01	-33.24	34.78	3.39E-01
cg26925644	21.29	8.97	1.76E-02	17.12	15.37	2.65E-01	28.29	18.14	1.19E-01	13.49	16.98	4.27E-01	35.16	24.35	1.49E-01
cg00534655	-17.36	7.32	1.76E-02	-20.27	12.47	1.04E-01	-15.27	19.04	4.23E-01	-12.34	13.38	3.57E-01	-21.25	15.99	1.84E-01
cg13990958	16.98	7.16	1.76E-02	10.45	15.28	4.94E-01	11.04	17.45	5.27E-01	20.11	10.34	5.18E-02	24.02	19.60	2.20E-01
cg01521220	7.85	3.31	1.76E-02	8.83	4.36	4.28E-02	9.65	7.97	2.26E-01	9.09	8.78	3.00E-01	-1.70	9.97	8.65E-01
cg21089667	18.81	7.93	1.76E-02	48.43	23.43	3.87E-02	42.51	28.05	1.30E-01	12.50	9.13	1.71E-01	8.69	34.55	8.01E-01
cg09664442	24.37	10.27	1.76E-02	-4.59	32.49	8.88E-01	16.59	78.91	8.33E-01	27.90	11.49	1.51E-02	26.83	35.51	4.50E-01
cg04117688	-70.08	29.53	1.76E-02	-100.18	39.95	1.22E-02	126.42	148.37	3.94E-01	-90.57	55.70	1.04E-01	-18.31	59.67	7.59E-01
cg11813009	11.94	5.03	1.76E-02	9.02	7.88	2.53E-01	5.42	17.14	7.52E-01	12.62	8.04	1.17E-01	24.92	14.86	9.36E-02
cg09514055	-32.11	13.53	1.76E-02	-45.30	23.06	4.94E-02	-12.11	70.70	8.64E-01	-31.10	19.25	1.06E-01	-5.59	38.28	8.84E-01
cg07641160	23.04	9.71	1.77E-02	22.81	31.77	4.73E-01	48.11	90.08	5.93E-01	22.85	10.76	3.38E-02	21.61	34.03	5.25E-01
cg18483549	-9.59	4.04	1.77E-02	-5.72	9.98	5.67E-01	-3.21	19.88	8.72E-01	-12.67	4.88	9.34E-03	1.79	12.35	8.85E-01
cg08716684	-11.33	4.78	1.77E-02	-18.30	8.20	2.56E-02	-14.49	11.84	2.21E-01	-4.78	8.82	5.88E-01	-6.66	10.55	5.28E-01
cg12342334	16.10	6.78	1.77E-02	12.61	23.15	5.86E-01	-13.91	95.92	8.85E-01	15.18	7.38	3.97E-02	35.57	27.00	1.88E-01
cg24899558	-5.84	2.46	1.77E-02	-4.83	4.30	2.62E-01	-0.57	5.72	9.21E-01	-8.35	4.21	4.72E-02	-8.96	6.47	1.66E-01
cg13826459	-10.97	4.62	1.77E-02	-12.62	7.30	8.40E-02	-1.60	10.88	8.83E-01	-21.69	14.14	1.25E-01	-10.61	8.29	2.01E-01
cg08697297	-13.93	5.87	1.77E-02	-16.45	11.23	1.43E-01	-6.61	14.57	6.50E-01	-19.00	9.21	3.92E-02	-4.10	14.73	7.81E-01
cg19296671	9.24	3.89	1.77E-02	10.39	6.07	8.68E-02	8.72	8.33	2.95E-01	1.99	9.15	8.28E-01	14.25	8.95	1.11E-01
cg22629065	-7.30	3.08	1.77E-02	-10.72	5.42	4.77E-02	0.59	9.17	9.48E-01	-7.76	4.76	1.03E-01	-4.53	8.06	5.74E-01

cg26657920	-14.17	5.97	1.77E-02	-16.00	14.48	2.69E-01	-0.80	23.62	9.73E-01	-13.52	7.38	6.67E-02	-22.91	17.98	2.03E-01
cg01211396	6.53	2.75	1.77E-02	5.38	3.31	1.05E-01	9.28	6.80	1.72E-01	12.24	10.56	2.47E-01	6.01	9.92	5.44E-01
cg11747499	15.95	6.72	1.77E-02	22.53	10.53	3.25E-02	19.76	21.27	3.53E-01	8.52	22.17	7.01E-01	10.02	10.62	3.45E-01
cg00510437	11.50	4.85	1.77E-02	13.15	6.72	5.03E-02	19.72	16.24	2.25E-01	17.58	15.61	2.60E-01	4.09	8.95	6.48E-01
cg01626681	17.96	7.57	1.77E-02	17.12	15.76	2.77E-01	0.32	26.99	9.90E-01	25.33	12.34	4.01E-02	14.17	13.51	2.94E-01
cg18117780	12.24	5.16	1.77E-02	13.97	7.60	6.60E-02	14.18	10.90	1.93E-01	20.86	16.28	2.00E-01	2.46	11.15	8.25E-01
cg06303171	-12.11	5.11	1.77E-02	-17.77	10.51	9.09E-02	-22.79	21.21	2.83E-01	-11.18	6.49	8.53E-02	3.54	17.22	8.37E-01
cg11689625	13.94	5.87	1.77E-02	17.25	7.28	1.79E-02	21.04	20.39	3.02E-01	0.95	27.63	9.73E-01	4.18	12.49	7.38E-01
cg18305324	12.42	5.23	1.77E-02	14.88	7.36	4.31E-02	22.28	14.28	1.19E-01	5.45	15.31	7.22E-01	5.16	10.63	6.27E-01
cg05908691	-15.45	6.51	1.77E-02	-16.68	10.12	9.94E-02	-1.64	17.27	9.25E-01	-26.21	14.05	6.22E-02	-11.71	13.62	3.90E-01
cg19923326	12.67	5.34	1.77E-02	20.37	8.75	1.99E-02	1.37	9.23	8.82E-01	20.42	16.70	2.21E-01	13.30	12.23	2.77E-01
cg14570531	23.25	9.80	1.77E-02	27.94	21.73	1.98E-01	37.77	22.14	8.81E-02	19.08	14.62	1.92E-01	10.51	25.21	6.77E-01
cg14018454	-13.61	5.74	1.77E-02	4.56	15.29	7.66E-01	-21.33	22.83	3.50E-01	-16.31	6.70	1.50E-02	-15.10	22.87	5.09E-01
cg02459271	-78.52	33.11	1.77E-02	-112.77	46.58	1.55E-02	-3.94	258.74	9.88E-01	-86.15	62.96	1.71E-01	11.56	73.68	8.75E-01
cg19421218	-7.45	3.14	1.77E-02	-7.89	5.81	1.75E-01	-12.96	10.17	2.03E-01	-7.08	4.48	1.15E-01	-3.59	8.97	6.89E-01
cg07510510	-10.30	4.34	1.77E-02	-12.54	8.41	1.36E-01	0.42	10.91	9.69E-01	-12.20	6.70	6.84E-02	-12.29	11.09	2.68E-01
cg01477985	11.70	4.93	1.77E-02	11.25	7.87	1.53E-01	-10.63	15.09	4.81E-01	18.22	8.33	2.88E-02	13.51	12.72	2.88E-01
cg15589689	17.33	7.31	1.77E-02	32.38	19.15	9.08E-02	17.68	26.75	5.09E-01	12.14	8.75	1.65E-01	34.46	25.54	1.77E-01
cg25372085	11.39	4.80	1.77E-02	15.62	5.73	6.45E-03	2.74	16.95	8.71E-01	11.51	25.44	6.51E-01	-1.07	11.23	9.24E-01
cg23166357	-34.71	14.64	1.77E-02	-11.14	57.34	8.46E-01	-41.02	256.13	8.73E-01	-37.97	15.60	1.49E-02	-8.39	64.52	8.97E-01
cg10586087	-14.19	5.99	1.77E-02	-7.99	16.76	6.34E-01	-9.25	34.61	7.89E-01	-16.17	6.79	1.72E-02	-4.95	23.56	8.34E-01
cg14028400	12.52	5.28	1.77E-02	7.02	5.55	2.05E-01	8.22	7.67	2.83E-01	38.87	13.14	3.10E-03	11.62	8.34	1.64E-01
cg10170269	12.92	5.45	1.77E-02	9.43	8.80	2.84E-01	14.45	12.39	2.44E-01	19.83	9.90	4.53E-02	4.21	15.69	7.88E-01
cg27228074	-18.20	7.68	1.77E-02	-28.82	19.85	1.47E-01	142.29	110.14	1.96E-01	-17.29	8.99	5.44E-02	-16.97	22.54	4.52E-01
cg11651007	24.29	10.24	1.77E-02	19.78	11.23	7.81E-02	-1.96	15.90	9.02E-01	46.79	19.21	1.48E-02	41.51	17.82	1.99E-02
cg10741399	-27.14	11.45	1.78E-02	-84.02	46.78	7.25E-02	-185.05	165.71	2.64E-01	-23.32	12.22	5.64E-02	-13.20	47.70	7.82E-01
cg03805566	12.43	5.24	1.78E-02	13.38	15.75	3.95E-01	-2.74	38.01	9.43E-01	14.97	5.82	1.01E-02	-20.54	21.97	3.50E-01
cg19133958	21.27	8.97	1.78E-02	23.21	13.43	8.39E-02	43.01	22.98	6.13E-02	38.92	26.80	1.46E-01	-0.01	16.67	9.99E-01
cg24781737	11.08	4.68	1.78E-02	11.43	6.90	9.76E-02	19.40	9.69	4.54E-02	2.27	11.89	8.48E-01	6.33	11.94	5.96E-01
cg03528246	12.92	5.45	1.78E-02	-3.65	13.12	7.81E-01	2.50	53.16	9.62E-01	14.98	6.56	2.24E-02	25.25	15.38	1.01E-01
cg09229620	8.34	3.52	1.78E-02	12.37	5.92	3.65E-02	5.70	7.04	4.19E-01	5.74	6.56	3.81E-01	8.16	10.66	4.44E-01
cg10696085	19.33	8.16	1.78E-02	18.87	25.69	4.63E-01	-79.37	113.57	4.85E-01	18.50	9.20	4.44E-02	30.53	24.77	2.18E-01
cg15964468	8.66	3.65	1.78E-02	9.57	5.52	8.27E-02	14.39	8.00	7.20E-02	4.66	10.04	6.42E-01	3.82	7.77	6.23E-01
cg22999603	15.70	6.62	1.78E-02	25.39	9.77	9.38E-03	17.33	15.38	2.60E-01	15.57	16.81	3.54E-01	-4.05	13.68	7.67E-01
cg03984866	21.06	8.89	1.78E-02	22.70	17.56	1.96E-01	15.20	19.38	4.33E-01	26.41	13.37	4.83E-02	4.17	29.34	8.87E-01

cg01995660	36.90	15.57	1.78E-02	42.05	19.88	3.44E-02	8.62	60.16	8.86E-01	32.60	49.60	5.11E-01	33.11	33.12	3.17E-01
cg16520049	38.02	16.04	1.78E-02	52.57	24.14	2.94E-02	33.53	36.11	3.53E-01	-11.44	38.23	7.65E-01	55.16	37.31	1.39E-01
cg11388802	-18.81	7.93	1.78E-02	-15.34	15.75	3.30E-01	-32.07	17.21	6.25E-02	-10.64	12.46	3.93E-01	-29.50	22.15	1.83E-01
cg16993754	8.04	3.39	1.78E-02	9.08	4.81	5.91E-02	5.02	7.73	5.16E-01	8.44	10.40	4.17E-01	8.14	7.50	2.78E-01
cg06432487	26.56	11.21	1.78E-02	19.31	22.95	4.00E-01	14.78	48.76	7.62E-01	26.47	14.59	6.96E-02	46.80	32.53	1.50E-01
cg03470073	33.55	14.16	1.78E-02	38.90	20.37	5.62E-02	10.02	43.52	8.18E-01	50.18	36.62	1.71E-01	23.69	27.67	3.92E-01
cg00745693	22.60	9.54	1.78E-02	21.30	19.84	2.83E-01	33.61	21.39	1.16E-01	16.01	14.76	2.78E-01	28.25	24.41	2.47E-01
cg08823975	30.99	13.08	1.78E-02	51.20	21.38	1.67E-02	53.12	54.25	3.27E-01	6.62	20.16	7.43E-01	40.57	34.09	2.34E-01
cg24834590	-12.97	5.47	1.78E-02	-17.59	15.57	2.59E-01	-6.34	27.29	8.16E-01	-12.32	6.22	4.74E-02	-16.13	22.09	4.65E-01
cg07178825	12.24	5.17	1.78E-02	9.58	8.23	2.45E-01	11.31	14.25	4.28E-01	11.21	10.03	2.64E-01	19.15	11.29	8.99E-02
cg15754109	27.39	11.56	1.78E-02	27.26	13.21	3.91E-02	88.83	133.18	5.05E-01	66.83	40.87	1.02E-01	3.42	30.19	9.10E-01
cg15965134	14.37	6.06	1.78E-02	15.62	9.52	1.01E-01	13.62	14.01	3.31E-01	16.22	16.00	3.10E-01	11.96	11.82	3.12E-01
cg05023013	18.61	7.86	1.78E-02	12.64	12.87	3.26E-01	35.87	15.24	1.86E-02	7.89	16.35	6.29E-01	19.45	21.69	3.70E-01
cg19287501	-17.22	7.27	1.78E-02	-26.17	13.30	4.91E-02	-15.28	15.04	3.10E-01	-5.98	13.54	6.58E-01	-22.85	17.13	1.82E-01
cg24696715	27.66	11.67	1.78E-02	41.61	18.82	2.70E-02	2.08	21.82	9.24E-01	14.98	16.28	3.57E-01	57.86	23.31	1.31E-02
cg24436715	20.80	8.78	1.78E-02	27.62	21.59	2.01E-01	24.70	40.80	5.45E-01	19.63	11.61	9.07E-02	17.82	18.87	3.45E-01
cg26781726	15.70	6.63	1.78E-02	21.32	9.64	2.70E-02	15.97	17.49	3.61E-01	20.91	13.04	1.09E-01	-12.27	17.56	4.85E-01
cg20664445	21.29	8.99	1.78E-02	10.16	9.49	2.85E-01	45.51	18.50	1.39E-02	44.69	30.51	1.43E-01	16.78	18.69	3.69E-01
cg11654904	-20.43	8.62	1.78E-02	-13.47	12.94	2.98E-01	-18.44	19.76	3.51E-01	-32.07	18.99	9.13E-02	-27.14	21.61	2.09E-01
cg22822599	-13.91	5.87	1.78E-02	-14.10	12.88	2.74E-01	-11.50	17.79	5.18E-01	-12.34	7.94	1.20E-01	-21.86	15.90	1.69E-01
cg12592365	-9.69	4.09	1.78E-02	-13.49	10.49	1.99E-01	-22.07	24.37	3.65E-01	-9.16	4.84	5.83E-02	-4.48	12.60	7.22E-01
cg19428602	25.08	10.59	1.78E-02	28.40	21.09	1.78E-01	32.93	21.81	1.31E-01	7.81	15.49	6.14E-01	65.94	30.66	3.15E-02
cg04926767	17.23	7.28	1.78E-02	9.52	11.83	4.21E-01	20.93	13.54	1.22E-01	41.70	18.21	2.20E-02	5.37	17.48	7.59E-01
cg20629387	27.27	11.51	1.78E-02	56.56	23.20	1.48E-02	66.23	43.01	1.24E-01	15.74	9.07	8.27E-02	16.63	30.81	5.89E-01
cg12737228	-7.42	3.13	1.79E-02	-10.76	4.32	1.29E-02	-3.91	7.12	5.83E-01	2.00	14.11	8.87E-01	-4.81	6.52	4.60E-01
cg00744866	8.59	3.63	1.79E-02	13.88	5.25	8.24E-03	4.48	8.40	5.94E-01	2.14	11.67	8.54E-01	3.89	7.39	5.99E-01
cg09207077	-13.79	5.82	1.79E-02	-23.72	8.59	5.75E-03	-3.48	13.71	7.99E-01	-12.13	13.92	3.83E-01	-0.75	13.53	9.56E-01
cg22236890	-9.28	3.92	1.79E-02	-14.40	5.66	1.09E-02	-1.68	8.55	8.44E-01	7.42	16.06	6.44E-01	-9.82	7.82	2.09E-01
cg03486157	21.49	9.07	1.79E-02	2.79	21.99	8.99E-01	107.17	72.93	1.42E-01	22.89	10.60	3.09E-02	31.63	31.67	3.18E-01
cg11594498	24.05	10.15	1.79E-02	40.15	23.59	8.87E-02	4.86	43.17	9.10E-01	21.90	12.70	8.47E-02	19.50	29.32	5.06E-01
cg26919780	-10.24	4.32	1.79E-02	-14.69	8.38	7.95E-02	-5.07	10.96	6.44E-01	-7.04	6.47	2.76E-01	-18.15	11.90	1.27E-01
cg09719031	16.95	7.16	1.79E-02	21.55	18.16	2.36E-01	49.00	31.19	1.16E-01	16.72	8.42	4.70E-02	-15.51	27.31	5.70E-01
cg19066682	-13.60	5.74	1.79E-02	-20.61	8.55	1.59E-02	-6.31	12.32	6.09E-01	-12.36	18.35	5.01E-01	-7.35	11.87	5.36E-01
cg23260254	-13.28	5.61	1.79E-02	-14.43	8.14	7.63E-02	-10.75	12.90	4.05E-01	-11.50	18.78	5.40E-01	-13.64	11.26	2.26E-01
cg13055685	16.65	7.03	1.79E-02	14.46	14.69	3.25E-01	16.49	16.29	3.11E-01	24.79	9.53	9.30E-03	-16.26	21.33	4.46E-01

cg14743291	26.13	11.03	1.79E-02	36.75	13.75	7.55E-03	-11.25	53.93	8.35E-01	30.53	60.08	6.11E-01	6.86	20.82	7.42E-01
cg22571135	-9.13	3.86	1.79E-02	-9.44	5.56	8.95E-02	-7.24	7.52	3.36E-01	2.34	9.19	7.99E-01	-19.82	8.10	1.44E-02
cg21925465	-12.21	5.15	1.79E-02	-7.42	11.90	5.33E-01	-4.74	13.32	7.22E-01	-13.84	6.98	4.74E-02	-21.77	15.04	1.48E-01
cg03760518	12.51	5.28	1.79E-02	7.06	7.72	3.60E-01	13.08	13.86	3.45E-01	9.43	12.49	4.50E-01	27.04	11.59	1.96E-02
cg14565764	-10.30	4.35	1.79E-02	-3.29	8.38	6.94E-01	-20.18	14.64	1.68E-01	-13.72	6.27	2.85E-02	-6.36	10.87	5.59E-01
cg00735923	18.35	7.75	1.79E-02	22.98	10.15	2.36E-02	4.18	33.44	9.00E-01	39.68	41.03	3.34E-01	10.12	13.54	4.55E-01
cg08302300	21.22	8.96	1.79E-02	32.42	16.11	4.42E-02	10.61	19.64	5.89E-01	19.76	16.49	2.31E-01	16.81	20.68	4.16E-01
cg00073650	18.87	7.97	1.79E-02	19.19	12.74	1.32E-01	9.45	15.82	5.50E-01	9.07	17.92	6.13E-01	45.72	20.12	2.31E-02
cg23995169	13.70	5.79	1.79E-02	7.76	9.20	3.99E-01	30.20	17.14	7.81E-02	4.99	12.36	6.86E-01	22.46	11.10	4.31E-02
cg09414156	-32.41	13.69	1.79E-02	-25.75	43.14	5.51E-01	16.39	198.50	9.34E-01	-32.82	14.86	2.72E-02	-44.07	63.23	4.86E-01
cg19363546	21.09	8.91	1.79E-02	19.68	11.88	9.77E-02	26.37	22.12	2.33E-01	-22.79	38.83	5.57E-01	31.13	18.85	9.86E-02
cg10673839	20.29	8.57	1.79E-02	42.44	21.61	4.96E-02	73.10	91.65	4.25E-01	14.99	10.08	1.37E-01	19.21	25.63	4.53E-01
cg13845094	18.00	7.60	1.79E-02	33.74	13.20	1.06E-02	-0.01	18.50	1.00E+00	16.16	14.61	2.69E-01	10.67	15.90	5.02E-01
cg00793648	17.90	7.56	1.79E-02	32.24	18.01	7.33E-02	-22.54	38.92	5.63E-01	16.26	8.94	6.90E-02	20.26	28.35	4.75E-01
cg17217195	13.71	5.79	1.79E-02	12.43	8.61	1.49E-01	16.12	11.20	1.50E-01	36.59	21.57	8.99E-02	5.49	12.67	6.65E-01
cg10050157	42.32	17.87	1.79E-02	61.16	28.58	3.23E-02	48.33	65.34	4.59E-01	13.10	39.48	7.40E-01	36.74	31.16	2.38E-01
cg17823004	20.52	8.67	1.79E-02	23.78	16.55	1.51E-01	20.24	27.41	4.60E-01	24.24	12.48	5.20E-02	1.94	22.89	9.32E-01
cg06750524	-12.10	5.11	1.79E-02	-14.04	6.92	4.24E-02	-9.80	11.36	3.88E-01	9.64	22.09	6.62E-01	-14.97	11.48	1.92E-01
cg10097651	14.29	6.04	1.79E-02	17.92	7.86	2.26E-02	9.48	17.24	5.82E-01	27.69	24.84	2.65E-01	4.05	12.63	7.49E-01
cg08190450	18.02	7.61	1.79E-02	19.23	10.65	7.10E-02	19.31	25.57	4.50E-01	32.50	26.54	2.21E-01	11.98	13.49	3.75E-01
cg08979895	9.56	4.04	1.79E-02	8.31	5.85	1.55E-01	6.28	8.12	4.39E-01	17.07	18.43	3.54E-01	14.11	8.45	9.47E-02
cg06057715	-22.81	9.64	1.79E-02	-26.93	18.63	1.48E-01	-8.23	26.53	7.56E-01	-25.38	13.82	6.62E-02	-19.02	28.54	5.05E-01
cg12102702	15.92	6.73	1.79E-02	30.22	13.40	2.42E-02	3.57	18.91	8.50E-01	9.52	6.80	1.61E-01	38.89	21.62	7.20E-02
cg05553511	17.37	7.34	1.79E-02	19.74	12.25	1.07E-01	1.71	15.00	9.09E-01	15.71	15.52	3.11E-01	35.62	17.35	4.00E-02
cg21431338	10.96	4.63	1.79E-02	6.85	10.09	4.97E-01	21.32	24.44	3.83E-01	12.35	5.66	2.90E-02	5.70	15.95	7.21E-01
cg07084627	9.88	4.18	1.79E-02	14.47	6.19	1.93E-02	9.95	11.18	3.74E-01	-0.95	9.95	9.24E-01	9.04	8.73	3.00E-01
cg22276612	-36.16	15.28	1.79E-02	-33.05	21.01	1.16E-01	-19.10	88.85	8.30E-01	-61.31	32.77	6.14E-02	-21.36	32.26	5.08E-01
cg03621504	8.43	3.56	1.79E-02	7.76	4.62	9.31E-02	9.56	8.15	2.41E-01	19.39	13.38	1.47E-01	4.29	9.41	6.48E-01
cg25948690	-20.60	8.70	1.80E-02	-9.55	12.90	4.59E-01	-4.96	18.25	7.86E-01	-42.66	21.31	4.53E-02	-35.17	15.66	2.47E-02
cg15648729	-13.63	5.76	1.80E-02	-5.79	11.89	6.26E-01	-17.70	33.95	6.02E-01	-18.87	7.08	7.75E-03	9.32	20.93	6.56E-01
cg19698789	-17.30	7.31	1.80E-02	-26.87	15.34	7.99E-02	10.00	17.58	5.70E-01	-23.30	10.09	2.09E-02	-15.02	18.58	4.19E-01
cg01747665	18.27	7.72	1.80E-02	16.34	12.97	2.08E-01	24.78	14.22	8.13E-02	10.75	17.26	5.34E-01	20.01	19.84	3.13E-01
cg02659794	-24.56	10.38	1.80E-02	-16.57	32.24	6.07E-01	30.31	142.06	8.31E-01	-31.50	11.79	7.56E-03	12.00	30.42	6.93E-01
cg16292768	8.02	3.39	1.80E-02	7.66	4.44	8.41E-02	10.17	8.40	2.26E-01	10.39	10.32	3.14E-01	5.28	8.85	5.50E-01
cg08562099	7.90	3.34	1.80E-02	7.52	4.42	8.90E-02	8.08	7.77	2.98E-01	3.43	12.40	7.82E-01	10.82	8.03	1.78E-01

cg10094886	-15.80	6.68	1.80E-02	-22.84	20.19	2.58E-01	-36.00	34.94	3.03E-01	-13.97	7.43	6.00E-02	-15.09	31.05	6.27E-01
cg09355528	-11.36	4.80	1.80E-02	-16.94	7.78	2.96E-02	-2.53	9.03	7.79E-01	-9.69	14.03	4.90E-01	-13.91	10.22	1.73E-01
cg00261832	-13.64	5.76	1.80E-02	-30.71	16.28	5.92E-02	0.82	28.25	9.77E-01	-12.56	6.57	5.59E-02	-2.43	22.97	9.16E-01
cg26613367	-9.09	3.84	1.80E-02	-14.17	8.63	1.01E-01	0.80	16.17	9.61E-01	-8.82	4.97	7.59E-02	-7.12	9.97	4.75E-01
cg16273961	24.88	10.52	1.80E-02	34.00	13.80	1.37E-02	16.29	39.42	6.79E-01	30.44	55.40	5.83E-01	9.20	18.83	6.25E-01
cg23547348	14.53	6.14	1.80E-02	9.92	12.76	4.37E-01	25.84	49.95	6.05E-01	15.91	7.70	3.88E-02	14.76	18.04	4.13E-01
cg02194396	7.25	3.06	1.80E-02	10.72	4.10	8.89E-03	0.44	6.91	9.49E-01	10.02	11.74	3.94E-01	2.76	7.29	7.05E-01
cg10321339	-37.93	16.03	1.80E-02	-27.98	24.69	2.57E-01	-85.74	42.62	4.43E-02	-22.13	32.29	4.93E-01	-44.93	36.79	2.22E-01
cg14847095	20.01	8.46	1.80E-02	11.97	15.27	4.33E-01	22.19	34.64	5.22E-01	14.54	6.82	3.29E-02	59.34	22.22	7.57E-03
cg08113562	19.22	8.13	1.80E-02	10.07	11.61	3.86E-01	12.95	28.35	6.48E-01	8.09	24.48	7.41E-01	38.79	14.41	7.10E-03
cg27578869	17.49	7.39	1.80E-02	36.78	18.97	5.25E-02	30.24	23.22	1.93E-01	10.19	9.01	2.58E-01	26.70	27.13	3.25E-01
cg15017604	7.68	3.25	1.80E-02	9.57	6.52	1.42E-01	11.95	12.55	3.41E-01	5.41	4.25	2.03E-01	13.28	10.18	1.92E-01
cg14952266	-13.49	5.70	1.80E-02	-17.68	12.23	1.48E-01	-1.53	12.16	9.00E-01	-18.07	8.54	3.43E-02	-10.71	16.73	5.22E-01
cg13766830	-30.12	12.73	1.80E-02	-12.38	48.35	7.98E-01	-98.24	169.49	5.62E-01	-30.22	13.61	2.64E-02	-45.28	56.91	4.26E-01
cg15466977	13.16	5.57	1.80E-02	16.99	13.86	2.20E-01	11.68	28.50	6.82E-01	13.09	6.37	4.00E-02	-0.04	28.51	9.99E-01
cg12757676	18.39	7.78	1.80E-02	19.58	19.77	3.22E-01	20.00	19.93	3.16E-01	13.90	9.81	1.56E-01	55.15	30.51	7.06E-02
cg00435526	35.46	14.99	1.80E-02	39.06	18.23	3.21E-02	18.64	63.91	7.71E-01	-45.26	67.23	5.01E-01	46.90	32.06	1.43E-01
cg15636887	-8.56	3.62	1.80E-02	-9.23	6.05	1.27E-01	-7.26	9.79	4.58E-01	-10.76	6.40	9.24E-02	-4.43	8.38	5.97E-01
cg21779954	-14.71	6.22	1.80E-02	-26.99	13.26	4.18E-02	-3.19	29.83	9.15E-01	-13.44	7.91	8.92E-02	-2.72	18.08	8.80E-01
cg06519449	28.85	12.20	1.80E-02	-5.65	35.41	8.73E-01	58.94	180.51	7.44E-01	35.80	13.50	8.01E-03	0.57	49.41	9.91E-01
cg15820809	33.49	14.16	1.80E-02	48.83	38.73	2.07E-01	62.24	159.25	6.96E-01	33.06	16.03	3.91E-02	8.58	50.62	8.65E-01
cg10119679	25.14	10.63	1.80E-02	26.00	13.41	5.25E-02	48.74	34.27	1.55E-01	26.03	65.15	6.90E-01	13.74	21.34	5.20E-01
cg08570691	-10.70	4.52	1.80E-02	-16.59	6.61	1.21E-02	-9.84	13.60	4.69E-01	-9.58	12.10	4.28E-01	-1.81	8.52	8.32E-01
cg01219549	-32.40	13.70	1.80E-02	-49.22	20.41	1.59E-02	-6.84	55.16	9.01E-01	-10.30	29.93	7.31E-01	-27.48	25.96	2.90E-01
cg12513880	16.34	6.91	1.80E-02	17.65	17.46	3.12E-01	0.67	22.93	9.77E-01	21.73	9.34	2.00E-02	7.89	15.25	6.05E-01
cg25868998	24.06	10.17	1.80E-02	66.93	34.60	5.31E-02	-5.64	112.14	9.60E-01	19.23	11.10	8.33E-02	33.00	39.63	4.05E-01
cg05565809	19.47	8.23	1.80E-02	13.63	13.79	3.23E-01	26.22	17.29	1.29E-01	10.69	16.53	5.18E-01	35.63	20.03	7.53E-02
cg08357990	12.89	5.45	1.81E-02	14.78	7.43	4.68E-02	14.40	13.52	2.87E-01	18.91	19.35	3.28E-01	5.00	11.60	6.67E-01
cg20283670	16.99	7.18	1.81E-02	16.92	10.33	1.02E-01	14.15	16.11	3.80E-01	37.47	23.28	1.08E-01	10.91	15.23	4.74E-01
cg12947757	18.05	7.63	1.81E-02	26.46	13.84	5.58E-02	14.71	11.75	2.10E-01	30.46	19.19	1.12E-01	-9.05	22.51	6.88E-01
cg00514713	-9.76	4.13	1.81E-02	-12.36	6.19	4.56E-02	-8.95	8.48	2.91E-01	-16.30	15.25	2.85E-01	-3.83	8.36	6.46E-01
cg26792080	44.57	18.85	1.81E-02	43.25	20.04	3.10E-02	62.23	62.12	3.16E-01	-30.15	46.19	5.14E-01	76.36	28.22	6.81E-03
cg18600211	18.94	8.01	1.81E-02	33.77	20.30	9.63E-02	15.12	27.23	5.79E-01	17.98	9.63	6.18E-02	-1.14	31.29	9.71E-01
cg01626493	-12.39	5.24	1.81E-02	-8.19	9.91	4.09E-01	-12.34	12.69	3.31E-01	-12.73	8.65	1.41E-01	-18.23	12.29	1.38E-01
cg12821632	-34.72	14.69	1.81E-02	-62.51	46.22	1.76E-01	2.51	170.01	9.88E-01	-29.45	16.35	7.18E-02	-55.01	50.35	2.75E-01

cg01949002	-16.89	7.15	1.81E-02	-12.14	13.52	3.69E-01	-20.93	15.22	1.69E-01	-9.49	12.27	4.39E-01	-35.23	17.81	4.80E-02
cg14878852	-16.10	6.81	1.81E-02	-27.70	13.09	3.44E-02	-0.26	17.17	9.88E-01	-15.16	10.40	1.45E-01	-14.42	17.99	4.23E-01
cg18150280	-11.07	4.68	1.81E-02	-15.00	5.74	9.03E-03	1.15	11.88	9.23E-01	3.58	23.88	8.81E-01	-10.03	12.46	4.21E-01
cg09584188	-6.89	2.91	1.81E-02	-7.35	4.36	9.16E-02	-1.76	5.74	7.59E-01	-1.63	9.17	8.59E-01	-15.39	6.62	2.01E-02
cg10873086	-19.11	8.09	1.81E-02	-16.32	14.22	2.51E-01	-51.80	20.01	9.63E-03	-16.92	7.97	3.37E-02	4.87	22.21	8.27E-01
cg10401017	7.57	3.20	1.81E-02	6.65	4.47	1.36E-01	17.45	6.23	5.12E-03	3.88	5.14	4.51E-01	0.21	9.81	9.83E-01
cg27341472	15.42	6.52	1.81E-02	12.97	11.18	2.46E-01	1.36	23.89	9.55E-01	19.96	9.87	4.31E-02	14.76	16.99	3.85E-01
cg11510931	13.36	5.65	1.81E-02	16.68	8.97	6.30E-02	21.10	12.68	9.61E-02	6.76	10.59	5.23E-01	5.18	16.39	7.52E-01
cg21407899	15.52	6.57	1.81E-02	14.87	9.10	1.02E-01	26.41	14.83	7.49E-02	-16.26	36.18	6.53E-01	12.53	13.14	3.40E-01
cg01397057	31.38	13.28	1.81E-02	31.28	16.90	6.41E-02	102.39	85.69	2.32E-01	71.05	52.85	1.79E-01	17.35	24.43	4.78E-01
cg16934969	-9.32	3.94	1.81E-02	-9.05	7.03	1.98E-01	-4.18	9.63	6.64E-01	-8.88	6.59	1.78E-01	-16.17	9.84	1.00E-01
cg01889070	33.37	14.12	1.81E-02	37.15	18.03	3.94E-02	39.14	45.81	3.93E-01	32.06	49.83	5.20E-01	20.31	30.68	5.08E-01
cg17406383	11.74	4.97	1.81E-02	16.71	7.83	3.29E-02	16.78	12.58	1.82E-01	9.42	9.17	3.04E-01	-2.50	12.93	8.47E-01
cg21545605	14.51	6.14	1.81E-02	8.75	9.05	3.34E-01	31.57	12.78	1.35E-02	16.57	18.64	3.74E-01	6.98	13.71	6.11E-01
cg21769006	13.45	5.69	1.81E-02	17.41	8.07	3.11E-02	11.86	12.17	3.30E-01	13.93	14.46	3.35E-01	0.40	15.81	9.80E-01
cg25875953	17.46	7.39	1.81E-02	18.41	19.32	3.41E-01	21.48	18.99	2.58E-01	12.29	9.44	1.93E-01	44.32	24.61	7.17E-02
cg04680037	47.94	20.28	1.81E-02	46.08	26.42	8.11E-02	27.71	71.41	6.98E-01	23.85	71.20	7.38E-01	66.78	40.70	1.01E-01
cg04441405	14.28	6.04	1.81E-02	4.29	8.83	6.27E-01	33.87	16.18	3.63E-02	28.33	27.02	2.94E-01	16.97	9.60	7.72E-02
cg05306107	-12.90	5.46	1.81E-02	-14.80	13.47	2.72E-01	-7.93	16.83	6.37E-01	-12.04	6.85	7.89E-02	-20.94	17.74	2.38E-01
cg18511798	-22.00	9.31	1.81E-02	-29.28	12.07	1.53E-02	-9.65	26.13	7.12E-01	-6.93	32.40	8.31E-01	-14.24	21.06	4.99E-01
cg07118196	25.99	11.00	1.81E-02	45.08	17.50	9.99E-03	11.86	19.14	5.35E-01	10.43	13.12	4.27E-01	57.73	28.13	4.01E-02
cg11230343	-18.87	7.98	1.81E-02	-15.00	17.57	3.93E-01	-13.98	88.74	8.75E-01	-14.97	9.73	1.24E-01	-49.76	23.87	3.71E-02
cg21241411	12.85	5.44	1.81E-02	10.76	8.37	1.99E-01	13.48	10.46	1.98E-01	5.74	12.45	6.44E-01	30.53	15.90	5.49E-02
cg03919694	20.75	8.78	1.81E-02	70.28	33.73	3.72E-02	-2.70	43.90	9.51E-01	17.30	10.03	8.46E-02	22.50	24.74	3.63E-01
cg11482099	31.64	13.39	1.81E-02	28.66	18.41	1.19E-01	15.85	41.96	7.06E-01	42.62	36.49	2.43E-01	38.94	27.69	1.60E-01
cg15485556	30.53	12.92	1.81E-02	14.72	32.70	6.53E-01	18.91	94.10	8.41E-01	35.18	15.11	1.99E-02	22.99	42.23	5.86E-01
cg07999953	-12.52	5.30	1.81E-02	-27.47	11.96	2.16E-02	5.93	17.88	7.40E-01	-10.37	6.87	1.31E-01	-12.23	15.29	4.24E-01
cg01310120	20.18	8.54	1.81E-02	22.21	10.98	4.32E-02	32.88	18.81	8.05E-02	36.12	18.19	4.70E-02	-4.34	15.36	7.77E-01
cg19193956	18.46	7.81	1.81E-02	19.66	10.72	6.65E-02	33.57	24.88	1.77E-01	41.93	32.91	2.03E-01	7.44	13.95	5.94E-01
cg20361600	33.44	14.15	1.82E-02	31.63	36.23	3.83E-01	-161.32	155.75	3.00E-01	32.42	16.32	4.70E-02	63.79	47.93	1.83E-01
cg01692482	-9.15	3.87	1.82E-02	-13.88	5.63	1.37E-02	-8.67	9.47	3.60E-01	-8.46	11.83	4.74E-01	-0.90	7.71	9.07E-01
cg17284384	29.42	12.46	1.82E-02	56.33	38.60	1.44E-01	72.84	217.52	7.38E-01	26.55	13.58	5.06E-02	19.17	54.96	7.27E-01
cg14425843	17.41	7.37	1.82E-02	7.51	16.32	6.45E-01	-13.99	29.86	6.39E-01	21.59	9.19	1.88E-02	31.10	24.43	2.03E-01
cg13582001	-17.74	7.51	1.82E-02	-7.68	14.08	5.86E-01	-22.85	25.35	3.67E-01	-21.64	11.44	5.86E-02	-21.50	16.96	2.05E-01
cg10042132	-14.78	6.26	1.82E-02	-22.57	17.73	2.03E-01	-69.73	38.86	7.27E-02	-11.64	6.98	9.56E-02	-17.32	28.80	5.48E-01

cg10032387	20.47	8.66	1.82E-02	26.45	12.91	4.05E-02	22.51	21.51	2.95E-01	-5.20	31.97	8.71E-01	16.83	15.47	2.77E-01
cg22501342	14.67	6.21	1.82E-02	16.97	8.70	5.12E-02	14.89	16.38	3.63E-01	-15.57	20.15	4.40E-01	21.34	12.39	8.51E-02
cg16104450	11.00	4.66	1.82E-02	12.41	6.42	5.35E-02	25.22	10.88	2.04E-02	1.84	11.37	8.71E-01	1.89	10.60	8.59E-01
cg10996201	-34.91	14.78	1.82E-02	-25.21	53.36	6.37E-01	-128.94	181.35	4.77E-01	-35.03	16.21	3.07E-02	-35.06	50.51	4.88E-01
cg08364561	11.52	4.88	1.82E-02	-1.88	13.01	8.85E-01	11.37	20.53	5.80E-01	12.60	6.35	4.72E-02	17.39	10.56	9.98E-02
cg25408314	-28.13	11.91	1.82E-02	-18.56	16.00	2.46E-01	-14.08	32.18	6.62E-01	-8.71	66.63	8.96E-01	-56.46	22.63	1.26E-02
cg19949550	-14.25	6.03	1.82E-02	-22.97	14.72	1.18E-01	-36.73	18.20	4.36E-02	-7.35	7.57	3.32E-01	-19.29	20.38	3.44E-01
cg06074896	14.70	6.22	1.82E-02	10.90	8.56	2.03E-01	26.72	15.29	8.06E-02	36.51	20.60	7.63E-02	5.50	13.44	6.83E-01
cg08127369	15.27	6.47	1.82E-02	11.33	15.64	4.69E-01	2.40	26.27	9.27E-01	17.46	7.71	2.35E-02	13.90	25.55	5.87E-01
cg12761282	-10.11	4.28	1.82E-02	-20.06	11.39	7.81E-02	-14.83	24.09	5.38E-01	-8.14	5.01	1.04E-01	-8.89	13.77	5.19E-01
cg21767402	-43.75	18.53	1.82E-02	-47.71	24.79	5.43E-02	-7.51	49.17	8.79E-01	-33.41	63.90	6.01E-01	-61.44	39.94	1.24E-01
cg04170948	-11.82	5.00	1.82E-02	-20.31	10.36	4.99E-02	-16.85	15.79	2.86E-01	-4.65	6.94	5.03E-01	-20.23	13.06	1.21E-01
cg15582789	12.47	5.28	1.82E-02	21.18	7.13	2.97E-03	5.31	13.55	6.95E-01	10.84	18.08	5.49E-01	1.03	9.83	9.16E-01
cg24898236	-15.75	6.67	1.82E-02	-6.80	12.60	5.89E-01	-8.05	17.21	6.40E-01	-22.68	11.83	5.52E-02	-21.51	13.27	1.05E-01
cg08096684	46.26	19.59	1.82E-02	29.46	25.45	2.47E-01	187.06	116.79	1.09E-01	41.22	50.59	4.15E-01	75.68	40.90	6.43E-02
cg13903917	-74.96	31.74	1.82E-02	-80.75	42.10	5.51E-02	220.56	185.20	2.34E-01	-112.94	81.06	1.64E-01	-73.20	63.66	2.50E-01
cg12967902	-18.96	8.03	1.82E-02	-20.53	11.49	7.39E-02	3.53	20.04	8.60E-01	-41.04	22.05	6.28E-02	-18.59	17.20	2.80E-01
cg08922547	-11.73	4.97	1.82E-02	-23.57	13.34	7.73E-02	-2.76	14.68	8.51E-01	-12.42	6.18	4.45E-02	-1.26	15.60	9.36E-01
cg23860321	24.79	10.50	1.82E-02	23.07	11.78	5.03E-02	82.26	58.98	1.63E-01	3.67	74.81	9.61E-01	24.52	26.66	3.58E-01
cg27175851	-11.54	4.89	1.82E-02	-4.87	11.49	6.72E-01	10.45	25.04	6.77E-01	-13.80	5.79	1.71E-02	-17.90	18.79	3.41E-01
cg06685968	34.22	14.49	1.82E-02	16.45	19.90	4.08E-01	7.76	102.04	9.39E-01	84.96	53.06	1.09E-01	50.70	23.68	3.23E-02
cg11009590	-33.53	14.20	1.82E-02	-32.05	17.17	6.19E-02	-49.04	57.91	3.97E-01	-119.87	67.89	7.75E-02	-16.07	30.87	6.03E-01
cg10521567	-23.71	10.04	1.82E-02	-51.95	20.72	1.22E-02	12.00	41.60	7.73E-01	-23.02	8.18	4.90E-03	6.30	29.95	8.33E-01
cg03711675	-14.80	6.27	1.82E-02	-5.64	12.50	6.52E-01	-4.30	31.47	8.91E-01	-22.99	8.32	5.73E-03	-1.18	16.65	9.44E-01
cg15304928	44.44	18.82	1.82E-02	28.71	23.85	2.29E-01	-3.24	94.85	9.73E-01	120.80	96.21	2.09E-01	73.68	34.40	3.22E-02
cg22897130	29.05	12.30	1.82E-02	20.44	20.04	3.08E-01	0.49	38.17	9.90E-01	28.14	21.42	1.89E-01	63.50	28.30	2.49E-02
cg11258381	-9.50	4.02	1.82E-02	-7.58	5.82	1.93E-01	-16.26	9.16	7.60E-02	-11.77	13.02	3.66E-01	-6.92	8.30	4.04E-01
cg10620680	8.99	3.81	1.82E-02	10.67	5.12	3.71E-02	11.18	8.92	2.10E-01	8.93	16.89	5.97E-01	2.78	8.24	7.36E-01
cg00874032	11.21	4.75	1.83E-02	11.16	6.66	9.38E-02	17.57	9.67	6.91E-02	0.97	13.79	9.44E-01	8.97	13.13	4.94E-01
cg05874270	-8.47	3.59	1.83E-02	-8.00	5.10	1.17E-01	-4.15	9.87	6.74E-01	-17.81	9.34	5.66E-02	-5.92	7.58	4.35E-01
cg01062116	-19.49	8.26	1.83E-02	-33.05	12.73	9.44E-03	8.30	17.82	6.41E-01	-19.18	14.85	1.97E-01	-23.20	15.74	1.41E-01
cg14287724	-8.75	3.71	1.83E-02	-8.18	5.59	1.44E-01	-11.64	6.84	8.88E-02	-5.96	9.31	5.22E-01	-7.35	11.32	5.16E-01
cg23429847	-60.24	25.52	1.83E-02	-9.62	64.90	8.82E-01	-434.52	245.22	7.64E-02	-57.80	26.07	2.66E-02	-93.08	63.25	1.41E-01
cg21840806	-13.13	5.56	1.83E-02	-7.97	13.48	5.55E-01	4.05	32.13	9.00E-01	-14.94	6.67	2.50E-02	-14.41	17.28	4.04E-01
cg03858663	-9.66	4.09	1.83E-02	-20.38	10.20	4.57E-02	-6.26	13.26	6.37E-01	-8.49	5.06	9.34E-02	-2.55	13.64	8.52E-01

cg15523958	15.50	6.57	1.83E-02	22.60	9.16	1.36E-02	-1.41	19.56	9.42E-01	43.59	28.49	1.26E-01	5.99	11.31	5.97E-01
cg16848524	12.65	5.36	1.83E-02	-1.29	13.01	9.21E-01	22.84	15.23	1.34E-01	12.66	8.55	1.39E-01	16.14	9.57	9.16E-02
cg21702497	-32.64	13.83	1.83E-02	-23.14	14.72	1.16E-01	-1.11	26.42	9.67E-01	-76.50	31.37	1.47E-02	-50.77	26.69	5.71E-02
cg15660353	15.22	6.45	1.83E-02	15.82	10.40	1.28E-01	8.11	12.98	5.32E-01	20.78	14.01	1.38E-01	17.40	16.27	2.85E-01
cg17344770	5.64	2.39	1.83E-02	5.81	3.03	5.52E-02	10.14	6.58	1.23E-01	8.27	7.58	2.75E-01	-0.99	6.29	8.75E-01
cg11832395	-9.60	4.07	1.83E-02	-11.13	6.17	7.10E-02	-8.08	9.50	3.95E-01	-14.92	10.42	1.52E-01	-4.36	8.50	6.08E-01
cg09507697	20.79	8.81	1.83E-02	29.57	20.16	1.42E-01	22.06	35.61	5.36E-01	15.49	10.89	1.55E-01	39.31	28.92	1.74E-01
cg01426475	-13.36	5.66	1.83E-02	-26.83	11.41	1.88E-02	-0.52	16.41	9.75E-01	-7.57	8.20	3.56E-01	-19.56	14.25	1.70E-01
cg23349767	25.39	10.76	1.83E-02	57.17	21.02	6.53E-03	27.19	25.78	2.92E-01	11.28	11.79	3.38E-01	24.69	28.56	3.87E-01
cg10063575	-12.34	5.23	1.83E-02	-18.64	9.99	6.20E-02	-8.37	14.24	5.57E-01	-7.32	8.03	3.62E-01	-17.92	12.78	1.61E-01
cg05205152	45.92	19.46	1.83E-02	17.45	52.60	7.40E-01	63.09	202.21	7.55E-01	57.45	22.23	9.76E-03	-12.33	65.80	8.51E-01
cg15188623	7.48	3.17	1.83E-02	6.46	4.14	1.19E-01	11.28	6.55	8.51E-02	10.54	13.97	4.51E-01	3.99	8.84	6.52E-01
cg00456326	9.60	4.07	1.83E-02	3.87	5.14	4.51E-01	6.88	7.13	3.34E-01	21.98	14.07	1.18E-01	19.08	7.70	1.32E-02
cg03102516	-12.59	5.34	1.83E-02	-3.83	8.84	6.65E-01	-23.36	18.04	1.95E-01	-19.60	6.66	3.26E-03	2.35	15.70	8.81E-01
cg10460003	-12.97	5.50	1.83E-02	-9.24	8.67	2.86E-01	-20.43	32.59	5.31E-01	-13.36	8.62	1.21E-01	-19.98	13.68	1.44E-01
cg13385203	15.22	6.45	1.83E-02	12.43	9.46	1.89E-01	15.32	13.45	2.55E-01	23.81	16.74	1.55E-01	15.18	16.27	3.51E-01
cg18990313	10.79	4.57	1.83E-02	11.67	7.34	1.12E-01	9.91	12.47	4.27E-01	13.32	8.74	1.27E-01	6.28	10.13	5.35E-01
cg14377720	-7.15	3.03	1.83E-02	-6.69	5.49	2.23E-01	-11.29	8.32	1.75E-01	-7.03	4.77	1.40E-01	-4.87	7.61	5.22E-01
cg23748172	-62.18	26.36	1.83E-02	-76.54	38.69	4.79E-02	63.28	106.53	5.53E-01	-38.51	45.93	4.02E-01	-122.80	69.14	7.57E-02
cg04446777	21.46	9.10	1.83E-02	28.04	28.37	3.23E-01	-7.99	94.04	9.32E-01	24.58	9.96	1.36E-02	-35.04	39.48	3.75E-01
cg09659197	14.36	6.09	1.83E-02	15.43	16.01	3.35E-01	-14.33	25.93	5.81E-01	15.09	7.14	3.45E-02	26.67	22.57	2.37E-01
cg07182965	39.36	16.68	1.83E-02	65.05	48.25	1.78E-01	-297.07	259.00	2.51E-01	38.03	18.42	3.89E-02	28.87	70.74	6.83E-01
cg08690200	-42.74	18.12	1.83E-02	-22.60	41.14	5.83E-01	-82.05	74.55	2.71E-01	-53.07	22.53	1.85E-02	7.98	57.15	8.89E-01
cg07285760	-15.60	6.61	1.83E-02	-2.47	11.50	8.30E-01	-11.02	19.61	5.74E-01	-25.09	9.60	8.94E-03	-20.04	23.30	3.90E-01
cg27604145	7.37	3.12	1.83E-02	8.07	4.53	7.48E-02	13.83	7.07	5.05E-02	7.98	9.51	4.01E-01	-0.14	6.64	9.83E-01
cg16015924	20.55	8.71	1.83E-02	48.12	21.34	2.41E-02	24.38	59.46	6.82E-01	12.97	10.20	2.04E-01	30.90	30.32	3.08E-01
cg17053085	-20.54	8.71	1.83E-02	-38.57	15.30	1.17E-02	-17.26	23.47	4.62E-01	-8.64	14.81	5.59E-01	-13.94	19.84	4.82E-01
cg23415212	19.24	8.16	1.83E-02	28.48	15.09	5.91E-02	-5.97	51.80	9.08E-01	13.84	11.07	2.11E-01	25.34	21.79	2.45E-01
cg27085741	16.84	7.14	1.83E-02	49.66	31.21	1.12E-01	-39.95	107.27	7.10E-01	13.88	7.73	7.26E-02	28.71	23.84	2.29E-01
cg07576632	7.24	3.07	1.83E-02	3.82	5.47	4.85E-01	3.48	9.55	7.16E-01	9.42	4.57	3.93E-02	10.98	8.52	1.97E-01
cg06903384	27.51	11.66	1.83E-02	24.04	15.78	1.28E-01	38.95	51.36	4.48E-01	44.31	43.93	3.13E-01	27.87	20.25	1.69E-01
cg02343823	7.26	3.08	1.84E-02	10.20	4.69	2.98E-02	7.14	6.96	3.05E-01	10.12	8.06	2.09E-01	0.02	6.43	9.98E-01
cg22328396	8.29	3.52	1.84E-02	7.27	5.40	1.78E-01	17.69	11.33	1.19E-01	8.29	6.62	2.10E-01	5.91	7.92	4.56E-01
cg25045127	-48.96	20.76	1.84E-02	-18.16	68.78	7.92E-01	98.18	238.17	6.80E-01	-61.89	22.90	6.88E-03	35.41	73.70	6.31E-01
cg02592473	19.10	8.10	1.84E-02	27.91	21.44	1.93E-01	7.45	27.20	7.84E-01	16.75	9.74	8.56E-02	36.92	28.94	2.02E-01

cg13774342	-9.88	4.19	1.84E-02	-6.51	11.33	5.65E-01	-8.39	23.46	7.20E-01	-7.76	4.93	1.15E-01	-28.91	12.78	2.37E-02
cg11967675	-27.06	11.48	1.84E-02	1.04	33.90	9.75E-01	-141.78	176.30	4.21E-01	-29.44	12.84	2.19E-02	-37.20	39.97	3.52E-01
cg15717719	-37.84	16.05	1.84E-02	-16.29	15.22	2.84E-01	-87.52	39.85	2.81E-02	-24.01	30.33	4.29E-01	-66.48	30.04	2.69E-02
cg11658419	8.46	3.59	1.84E-02	15.21	5.10	2.86E-03	3.13	8.66	7.18E-01	5.54	8.50	5.15E-01	1.30	7.39	8.61E-01
cg03935956	14.11	5.99	1.84E-02	16.39	8.87	6.45E-02	21.06	12.81	1.00E-01	0.61	14.72	9.67E-01	12.11	14.93	4.17E-01
cg23627948	-3.68	1.56	1.84E-02	-4.40	2.11	3.67E-02	-1.04	3.38	7.58E-01	1.96	6.02	7.45E-01	-6.86	3.77	6.88E-02
cg20385216	9.86	4.18	1.84E-02	10.42	5.97	8.09E-02	13.25	10.82	2.21E-01	18.46	11.36	1.04E-01	1.18	8.83	8.94E-01
cg15363887	21.09	8.95	1.84E-02	39.29	16.94	2.04E-02	-1.14	28.05	9.68E-01	19.30	12.83	1.32E-01	6.49	24.54	7.91E-01
cg06479755	-62.59	26.55	1.84E-02	-50.19	70.18	4.74E-01	-301.49	303.71	3.21E-01	-75.71	31.44	1.60E-02	6.31	71.87	9.30E-01
cg07061426	-17.08	7.25	1.84E-02	-14.61	15.90	3.58E-01	1.30	38.46	9.73E-01	-20.41	8.57	1.72E-02	11.94	35.48	7.37E-01
cg07337598	15.62	6.63	1.84E-02	20.45	11.85	8.43E-02	11.15	15.45	4.70E-01	10.95	10.26	2.86E-01	30.17	22.55	1.81E-01
cg15986030	-10.61	4.50	1.84E-02	-6.41	7.14	3.70E-01	-16.25	9.72	9.46E-02	-1.65	11.24	8.83E-01	-18.93	9.43	4.46E-02
cg16217510	-9.68	4.10	1.84E-02	-13.10	5.63	1.99E-02	-8.51	10.76	4.29E-01	-6.26	14.83	6.73E-01	-4.01	8.28	6.28E-01
cg20745153	20.48	8.69	1.84E-02	34.41	17.17	4.51E-02	11.83	16.14	4.64E-01	13.31	15.98	4.05E-01	27.18	21.83	2.13E-01
cg26872028	-48.52	20.58	1.84E-02	-49.71	24.39	4.16E-02	19.77	100.67	8.44E-01	-70.08	87.67	4.24E-01	-52.84	47.11	2.62E-01
cg10477073	44.79	19.00	1.84E-02	37.66	27.76	1.75E-01	77.08	81.10	3.42E-01	43.99	47.91	3.59E-01	50.08	33.61	1.36E-01
cg05376185	13.82	5.86	1.84E-02	22.13	9.60	2.12E-02	12.35	13.53	3.61E-01	15.39	11.99	1.99E-01	-2.13	13.08	8.71E-01
cg00495658	-10.99	4.66	1.84E-02	-11.85	6.96	8.87E-02	-14.61	12.59	2.46E-01	-4.14	10.91	7.04E-01	-12.57	9.68	1.94E-01
cg02054108	5.60	2.38	1.84E-02	4.07	3.59	2.57E-01	12.12	6.13	4.80E-02	6.96	4.21	9.82E-02	-2.47	7.85	7.53E-01
cg00369640	24.99	10.60	1.84E-02	16.86	14.67	2.50E-01	36.24	25.67	1.58E-01	40.17	39.01	3.03E-01	30.19	21.98	1.70E-01
cg20849109	8.58	3.64	1.84E-02	9.69	5.04	5.46E-02	12.18	8.76	1.65E-01	12.86	11.69	2.72E-01	0.87	7.96	9.13E-01
cg14891209	17.41	7.39	1.84E-02	4.06	30.74	8.95E-01	42.57	57.40	4.58E-01	20.93	8.07	9.50E-03	-12.08	24.89	6.27E-01
cg09330596	11.74	4.98	1.84E-02	12.09	7.56	1.10E-01	17.74	11.00	1.07E-01	13.65	10.48	1.93E-01	-1.68	13.56	9.01E-01
cg08608532	-41.31	17.53	1.84E-02	-37.21	43.21	3.89E-01	-332.99	210.35	1.13E-01	-50.37	23.09	2.92E-02	-15.27	34.88	6.62E-01
cg23210049	24.86	10.55	1.84E-02	54.12	26.14	3.84E-02	-31.57	73.91	6.69E-01	22.35	12.45	7.26E-02	6.49	33.63	8.47E-01
cg14415885	16.94	7.19	1.84E-02	-1.25	24.91	9.60E-01	11.28	91.63	9.02E-01	17.40	7.77	2.52E-02	37.75	30.53	2.16E-01
cg26197690	-34.23	14.52	1.84E-02	-49.14	45.17	2.77E-01	131.24	148.00	3.75E-01	-30.50	16.20	5.97E-02	-71.06	50.46	1.59E-01
cg02199239	42.50	18.03	1.84E-02	29.23	48.49	5.47E-01	-28.47	188.98	8.80E-01	34.19	20.70	9.86E-02	136.50	58.97	2.06E-02
cg16625671	-10.78	4.58	1.84E-02	-22.00	12.43	7.68E-02	1.94	11.39	8.64E-01	-11.29	5.86	5.41E-02	-13.21	14.96	3.77E-01
cg05592406	-40.27	17.09	1.85E-02	-29.31	24.72	2.36E-01	-33.47	48.01	4.86E-01	-51.21	39.57	1.96E-01	-59.68	37.38	1.10E-01
cg21002528	6.76	2.87	1.85E-02	6.29	3.69	8.86E-02	12.86	7.35	8.00E-02	7.98	8.64	3.56E-01	0.91	7.87	9.08E-01
cg24008801	-35.67	15.14	1.85E-02	-44.83	18.32	1.44E-02	-47.26	71.06	5.06E-01	-36.30	57.96	5.31E-01	-2.20	33.52	9.48E-01
cg19513582	5.75	2.44	1.85E-02	4.04	3.40	2.34E-01	11.94	5.97	4.56E-02	7.42	5.56	1.82E-01	1.94	6.91	7.79E-01
cg05986680	-47.23	20.04	1.85E-02	-43.33	35.27	2.19E-01	-116.32	269.71	6.66E-01	-57.28	25.88	2.69E-02	24.55	74.84	7.43E-01
cg17314888	11.13	4.72	1.85E-02	5.69	6.37	3.71E-01	14.39	9.40	1.26E-01	27.46	10.89	1.17E-02	4.76	10.21	6.41E-01

cg05758489	38.10	16.17	1.85E-02	49.11	19.09	1.01E-02	-1.52	68.95	9.82E-01	29.36	69.89	6.74E-01	7.89	38.79	8.39E-01
cg10510775	13.90	5.90	1.85E-02	18.22	8.16	2.55E-02	17.81	15.35	2.46E-01	17.94	17.67	3.10E-01	-1.14	12.61	9.28E-01
cg16717546	-11.88	5.04	1.85E-02	-9.66	10.30	3.48E-01	-9.28	18.97	6.25E-01	-10.04	6.53	1.24E-01	-31.15	16.44	5.82E-02
cg08261158	48.47	20.58	1.85E-02	-7.29	56.73	8.98E-01	-108.30	231.31	6.40E-01	58.45	23.38	1.24E-02	58.37	70.05	4.05E-01
cg01067137	11.60	4.92	1.85E-02	12.60	6.73	6.12E-02	14.47	10.13	1.53E-01	21.63	17.74	2.23E-01	-1.48	12.63	9.07E-01
cg10536276	8.98	3.81	1.85E-02	10.01	5.09	4.91E-02	3.02	8.68	7.28E-01	14.53	12.51	2.46E-01	9.35	9.76	3.38E-01
cg20411384	-8.73	3.71	1.85E-02	-16.46	7.36	2.54E-02	0.24	13.89	9.86E-01	-6.00	5.11	2.41E-01	-9.54	9.60	3.20E-01
cg07083513	17.10	7.26	1.85E-02	14.27	10.43	1.71E-01	21.43	14.53	1.40E-01	-21.37	27.71	4.41E-01	31.93	16.33	5.06E-02
cg19120782	22.11	9.39	1.85E-02	25.24	16.84	1.34E-01	12.01	18.66	5.20E-01	29.53	17.47	9.10E-02	18.33	24.47	4.54E-01
cg03229158	14.94	6.34	1.85E-02	22.23	9.71	2.20E-02	31.81	21.07	1.31E-01	12.05	12.31	3.28E-01	-2.95	13.63	8.28E-01
cg16393207	11.28	4.79	1.85E-02	15.24	7.76	4.97E-02	11.57	10.02	2.48E-01	12.83	10.14	2.06E-01	-0.18	11.72	9.88E-01
cg06591466	17.45	7.41	1.85E-02	31.60	15.78	4.52E-02	4.73	23.17	8.38E-01	24.54	13.45	6.80E-02	6.82	12.13	5.74E-01
cg04136018	21.16	8.98	1.85E-02	21.16	20.64	3.05E-01	-19.13	98.00	8.45E-01	25.67	10.52	1.47E-02	-19.66	33.42	5.56E-01
cg10627428	9.96	4.23	1.85E-02	10.56	4.72	2.52E-02	25.12	10.06	1.25E-02	7.37	9.32	4.29E-01	0.04	8.15	9.96E-01
cg27619481	17.24	7.32	1.85E-02	34.88	19.34	7.14E-02	19.91	13.87	1.51E-01	8.13	10.22	4.26E-01	38.76	28.67	1.76E-01
cg12909188	-16.60	7.05	1.85E-02	-31.40	11.43	6.01E-03	-30.30	20.53	1.40E-01	-6.46	5.62	2.50E-01	-15.68	14.30	2.73E-01
cg24098927	36.38	15.45	1.85E-02	36.98	21.54	8.59E-02	12.88	56.13	8.19E-01	113.52	58.17	5.10E-02	24.68	26.53	3.52E-01
cg08849574	21.49	9.12	1.85E-02	33.34	13.74	1.52E-02	18.63	26.94	4.89E-01	25.39	29.47	3.89E-01	6.36	15.46	6.81E-01
cg08572278	-15.29	6.49	1.85E-02	-21.09	12.06	8.03E-02	-40.05	21.22	5.91E-02	-6.64	5.45	2.24E-01	-25.20	15.80	1.11E-01
cg07303923	20.53	8.72	1.85E-02	-2.20	24.52	9.29E-01	8.11	35.73	8.21E-01	25.54	10.05	1.10E-02	17.80	35.30	6.14E-01
cg02276314	9.40	3.99	1.86E-02	11.14	5.44	4.04E-02	16.50	9.79	9.19E-02	-3.96	10.33	7.02E-01	8.54	10.47	4.15E-01
cg15628518	6.58	2.80	1.86E-02	6.61	3.53	6.13E-02	9.90	5.81	8.83E-02	4.78	11.98	6.90E-01	-1.34	9.51	8.88E-01
cg25436766	-12.76	5.42	1.86E-02	-14.86	6.75	2.78E-02	-23.63	15.10	1.18E-01	5.81	20.69	7.79E-01	-3.44	13.62	8.00E-01
cg10024478	19.56	8.31	1.86E-02	-1.71	15.50	9.12E-01	36.87	17.19	3.20E-02	21.75	15.79	1.68E-01	26.12	17.73	1.41E-01
cg14514987	-15.19	6.45	1.86E-02	-31.80	15.71	4.29E-02	-16.42	30.32	5.88E-01	-13.76	7.82	7.84E-02	2.72	19.86	8.91E-01
cg18345406	-12.86	5.46	1.86E-02	-17.06	9.22	6.42E-02	-12.67	19.45	5.15E-01	-4.95	5.88	4.00E-01	-29.77	12.46	1.69E-02
cg24360651	35.30	14.99	1.86E-02	26.25	24.04	2.75E-01	88.29	48.00	6.59E-02	31.24	31.74	3.25E-01	32.74	27.84	2.40E-01
cg10490227	-17.56	7.46	1.86E-02	-5.77	13.03	6.58E-01	-18.78	23.42	4.23E-01	-28.15	13.85	4.22E-02	-19.96	14.08	1.56E-01
cg10933959	5.96	2.53	1.86E-02	4.73	3.98	2.34E-01	11.40	6.13	6.28E-02	7.29	4.49	1.05E-01	-1.97	7.71	7.98E-01
cg19214024	12.40	5.27	1.86E-02	7.15	7.14	3.16E-01	13.10	9.32	1.60E-01	-2.79	18.22	8.78E-01	28.48	11.05	9.92E-03
cg08051602	17.28	7.34	1.86E-02	4.73	16.02	7.68E-01	-6.24	56.68	9.12E-01	17.79	8.90	4.56E-02	46.39	24.16	5.48E-02
cg22481632	25.24	10.72	1.86E-02	47.67	22.19	3.17E-02	23.85	39.78	5.49E-01	25.97	15.96	1.04E-01	2.67	21.79	9.02E-01
cg13715758	-61.40	26.08	1.86E-02	-54.91	32.46	9.07E-02	20.96	109.48	8.48E-01	-26.84	89.00	7.63E-01	-117.31	56.70	3.85E-02
cg01255591	19.38	8.23	1.86E-02	2.47	10.07	8.06E-01	22.87	13.24	8.40E-02	39.71	14.13	4.96E-03	20.29	14.96	1.75E-01
cg03427299	14.06	5.97	1.86E-02	25.41	11.05	2.15E-02	3.82	17.38	8.26E-01	8.49	11.06	4.42E-01	12.44	10.94	2.56E-01

cg05667097	11.24	4.77	1.86E-02	12.16	6.94	7.98E-02	20.19	14.00	1.49E-01	10.76	9.71	2.68E-01	3.19	11.61	7.84E-01
cg13607230	7.21	3.06	1.86E-02	8.34	4.56	6.76E-02	5.44	7.85	4.88E-01	10.39	6.25	9.66E-02	0.83	7.73	9.15E-01
cg16126900	-11.97	5.09	1.86E-02	-13.85	10.78	1.99E-01	-9.97	17.30	5.64E-01	-13.50	6.65	4.22E-02	-1.12	15.71	9.43E-01
cg11580026	-7.04	2.99	1.86E-02	-5.28	4.88	2.79E-01	-8.63	6.79	2.04E-01	-12.95	6.38	4.22E-02	-2.52	6.52	6.99E-01
cg16625218	-9.87	4.19	1.86E-02	-12.66	5.98	3.43E-02	-2.15	9.88	8.28E-01	-6.02	14.67	6.82E-01	-11.24	8.45	1.84E-01
cg21460503	-24.75	10.52	1.86E-02	-48.54	25.25	5.45E-02	34.43	84.58	6.84E-01	-19.25	12.31	1.18E-01	-34.73	37.04	3.48E-01
cg24404878	-28.87	12.27	1.86E-02	-49.11	22.92	3.21E-02	-31.16	66.90	6.41E-01	-24.40	17.89	1.73E-01	-10.89	26.80	6.85E-01
cg16234986	-12.44	5.29	1.86E-02	-21.08	13.32	1.14E-01	-6.68	16.91	6.93E-01	-10.87	6.40	8.94E-02	-16.82	21.15	4.26E-01
cg16535667	9.35	3.97	1.86E-02	11.65	7.33	1.12E-01	11.55	9.49	2.24E-01	8.53	6.37	1.81E-01	4.14	10.50	6.93E-01
cg25005739	13.06	5.55	1.86E-02	15.48	7.40	3.65E-02	16.22	16.53	3.26E-01	8.78	15.61	5.74E-01	7.14	12.45	5.67E-01
cg20996561	6.80	2.89	1.86E-02	4.44	3.95	2.61E-01	10.53	6.38	9.87E-02	12.51	10.60	2.38E-01	7.22	6.74	2.84E-01
cg06916709	-21.61	9.18	1.86E-02	-21.28	12.13	7.95E-02	-29.62	42.90	4.90E-01	-39.26	23.78	9.88E-02	-9.53	19.05	6.17E-01
cg16814808	18.66	7.93	1.86E-02	44.06	19.66	2.50E-02	13.81	47.27	7.70E-01	12.36	9.97	2.15E-01	18.55	18.82	3.24E-01
cg18935231	-17.26	7.34	1.86E-02	-21.93	13.33	9.99E-02	-11.11	15.07	4.61E-01	-17.92	15.05	2.34E-01	-16.75	15.55	2.81E-01
cg21491107	8.43	3.58	1.86E-02	8.53	4.89	8.12E-02	12.77	8.67	1.41E-01	11.21	8.90	2.08E-01	-1.07	9.87	9.14E-01
cg24826252	-13.08	5.56	1.86E-02	-8.43	12.85	5.12E-01	-3.33	26.65	9.01E-01	-13.52	6.61	4.06E-02	-29.07	22.45	1.95E-01
cg26965151	44.86	19.06	1.86E-02	46.73	24.98	6.14E-02	29.20	76.73	7.04E-01	21.38	12.21	8.00E-02	112.32	38.86	3.85E-03
cg12151545	14.17	6.02	1.86E-02	15.40	8.23	6.11E-02	11.93	13.67	3.83E-01	11.35	18.01	5.29E-01	14.75	15.15	3.30E-01
cg27009208	36.95	15.70	1.86E-02	32.69	48.90	5.04E-01	42.68	46.02	3.54E-01	36.38	18.57	5.01E-02	39.70	61.53	5.19E-01
cg20673721	7.51	3.19	1.86E-02	7.28	3.64	4.57E-02	14.45	6.45	2.51E-02	11.56	8.94	1.96E-01	-3.30	7.33	6.52E-01
cg13798679	-9.29	3.95	1.86E-02	-3.51	4.93	4.77E-01	-17.85	9.45	5.91E-02	-7.30	11.01	5.07E-01	-17.97	8.32	3.08E-02
cg04963535	33.94	14.42	1.86E-02	46.50	43.44	2.84E-01	88.58	167.90	5.98E-01	29.95	15.95	6.04E-02	56.78	56.81	3.18E-01
cg21461196	-31.03	13.19	1.86E-02	-27.22	15.19	7.31E-02	-24.25	50.17	6.29E-01	-37.87	47.60	4.26E-01	-59.06	41.63	1.56E-01
cg26850677	-20.29	8.62	1.86E-02	-13.50	16.51	4.14E-01	-48.23	34.07	1.57E-01	-28.01	11.90	1.86E-02	8.64	23.20	7.10E-01
cg02053092	18.74	7.97	1.86E-02	36.74	17.83	3.94E-02	2.23	41.20	9.57E-01	15.20	9.92	1.26E-01	12.91	23.17	5.77E-01
cg03227456	-58.79	24.99	1.86E-02	-59.07	31.97	6.46E-02	-25.59	110.82	8.17E-01	-30.50	78.52	6.98E-01	-77.32	51.38	1.32E-01
cg06472439	17.92	7.62	1.87E-02	12.48	10.53	2.36E-01	5.29	21.28	8.04E-01	43.46	32.53	1.82E-01	28.33	14.04	4.37E-02
cg01120307	19.60	8.33	1.87E-02	34.85	21.38	1.03E-01	33.59	22.63	1.38E-01	14.80	10.49	1.58E-01	5.07	29.19	8.62E-01
cg00514895	20.47	8.70	1.87E-02	16.83	24.72	4.96E-01	14.60	38.22	7.02E-01	24.01	10.44	2.15E-02	7.28	24.21	7.64E-01
cg08336300	9.89	4.20	1.87E-02	16.69	5.32	1.70E-03	9.00	10.54	3.93E-01	-2.44	11.82	8.37E-01	3.52	7.73	6.49E-01
cg19262968	32.15	13.67	1.87E-02	36.10	23.08	1.18E-01	-7.81	118.42	9.47E-01	31.26	18.66	9.39E-02	28.38	43.36	5.13E-01
cg03293206	-9.35	3.98	1.87E-02	-4.97	5.21	3.40E-01	-8.79	8.91	3.24E-01	-19.39	17.33	2.63E-01	-22.22	9.76	2.29E-02
cg04513185	-26.38	11.22	1.87E-02	-16.93	18.27	3.54E-01	-29.47	32.94	3.71E-01	-44.45	21.69	4.04E-02	-19.57	22.92	3.93E-01
cg18108753	-12.22	5.20	1.87E-02	-19.54	6.71	3.59E-03	2.80	10.35	7.87E-01	-19.44	11.51	9.12E-02	-7.99	9.46	3.98E-01
cg02666265	25.36	10.78	1.87E-02	19.31	20.41	3.44E-01	47.57	21.21	2.49E-02	7.94	20.15	6.93E-01	30.73	25.73	2.32E-01

cg10554801	-30.32	12.89	1.87E-02	-43.58	21.98	4.73E-02	-16.77	43.33	6.99E-01	-22.83	23.33	3.28E-01	-26.19	25.19	2.98E-01
cg04349506	23.82	10.13	1.87E-02	41.48	20.58	4.38E-02	13.02	34.81	7.08E-01	12.53	13.85	3.66E-01	43.10	27.22	1.13E-01
cg22729726	10.83	4.60	1.87E-02	14.54	6.89	3.50E-02	10.34	11.04	3.49E-01	17.42	11.52	1.30E-01	-1.09	9.81	9.12E-01
cg11239019	-34.04	14.48	1.87E-02	13.73	42.26	7.45E-01	3.80	164.36	9.82E-01	-43.07	16.11	7.50E-03	-13.31	55.81	8.11E-01
cg19377421	-14.49	6.16	1.87E-02	-25.48	10.97	2.02E-02	1.13	13.45	9.33E-01	-10.68	13.23	4.20E-01	-16.95	12.13	1.62E-01
cg10947001	-13.20	5.62	1.87E-02	-20.82	13.60	1.26E-01	-2.66	26.52	9.20E-01	-10.85	6.84	1.13E-01	-20.09	16.87	2.34E-01
cg08273957	11.69	4.97	1.87E-02	15.11	7.37	4.04E-02	9.63	14.52	5.07E-01	20.56	12.35	9.59E-02	1.33	9.65	8.90E-01
cg18518722	18.81	8.00	1.87E-02	5.12	15.40	7.39E-01	30.86	13.22	1.95E-02	16.72	18.43	3.64E-01	16.92	19.09	3.76E-01
cg00634714	12.23	5.20	1.87E-02	9.22	6.49	1.55E-01	22.36	13.88	1.07E-01	16.45	25.71	5.22E-01	14.15	12.42	2.54E-01
cg14353506	-10.92	4.65	1.87E-02	-14.20	13.13	2.79E-01	-4.89	17.79	7.83E-01	-9.76	5.43	7.23E-02	-22.24	16.95	1.90E-01
cg16489809	5.47	2.33	1.87E-02	5.72	3.57	1.10E-01	10.14	5.44	6.20E-02	4.00	4.28	3.50E-01	0.05	7.46	9.94E-01
cg13682912	-12.04	5.12	1.87E-02	-19.91	11.85	9.29E-02	-11.82	15.38	4.42E-01	-9.75	6.62	1.41E-01	-11.35	15.96	4.77E-01
cg13178361	5.89	2.51	1.87E-02	5.14	3.23	1.12E-01	15.26	7.75	4.91E-02	5.56	6.56	3.97E-01	2.69	6.47	6.78E-01
cg04424379	-37.05	15.76	1.87E-02	-39.59	49.03	4.19E-01	-84.21	235.59	7.21E-01	-32.81	17.46	6.02E-02	-75.70	56.74	1.82E-01
cg04421348	-13.08	5.57	1.87E-02	-17.40	7.74	2.46E-02	-4.03	14.26	7.78E-01	-9.07	13.36	4.97E-01	-12.11	14.04	3.88E-01
cg17167924	37.27	15.85	1.87E-02	-5.37	36.68	8.84E-01	-18.17	100.94	8.57E-01	45.21	19.22	1.87E-02	73.53	48.16	1.27E-01
cg11408933	16.67	7.09	1.87E-02	19.07	17.02	2.63E-01	12.16	17.43	4.85E-01	14.30	10.98	1.93E-01	22.09	14.37	1.24E-01
cg06041067	17.99	7.65	1.87E-02	13.84	9.93	1.64E-01	30.60	25.14	2.24E-01	34.52	31.81	2.78E-01	19.31	15.12	2.02E-01
cg02148270	15.16	6.45	1.87E-02	22.23	15.57	1.53E-01	9.68	20.53	6.37E-01	15.51	7.89	4.95E-02	0.74	25.70	9.77E-01
cg23409370	-30.22	12.86	1.87E-02	-33.85	15.31	2.70E-02	-58.46	77.96	4.53E-01	-56.64	74.63	4.48E-01	-12.92	26.37	6.24E-01
cg03531396	14.95	6.36	1.87E-02	6.46	20.62	7.54E-01	65.35	56.29	2.46E-01	15.57	7.07	2.77E-02	10.87	22.03	6.22E-01
cg16661744	15.68	6.67	1.88E-02	14.94	8.98	9.62E-02	14.96	17.03	3.80E-01	12.29	25.42	6.29E-01	19.00	14.03	1.76E-01
cg11521079	-7.42	3.16	1.88E-02	-9.39	4.28	2.83E-02	-7.04	7.48	3.47E-01	-8.51	10.71	4.27E-01	-1.70	7.20	8.14E-01
cg04124987	33.24	14.14	1.88E-02	28.61	17.00	9.24E-02	-38.42	65.64	5.58E-01	73.18	74.25	3.24E-01	55.83	29.81	6.11E-02
cg26960370	22.47	9.56	1.88E-02	23.11	16.83	1.70E-01	24.94	17.29	1.49E-01	17.81	21.05	3.98E-01	22.48	23.52	3.39E-01
cg27656486	-47.06	20.02	1.88E-02	-51.90	56.08	3.55E-01	-183.66	178.45	3.03E-01	-48.99	22.97	3.29E-02	-8.98	63.33	8.87E-01
cg04310833	18.67	7.94	1.88E-02	7.87	18.82	6.76E-01	35.17	32.60	2.81E-01	18.09	9.56	5.84E-02	37.54	29.71	2.06E-01
cg11592677	22.30	9.49	1.88E-02	26.20	15.64	9.39E-02	23.14	18.16	2.03E-01	12.81	20.10	5.24E-01	25.60	25.72	3.19E-01
cg24650394	26.40	11.23	1.88E-02	6.37	19.89	7.49E-01	-35.66	53.65	5.06E-01	37.43	12.83	3.54E-03	42.35	30.21	1.61E-01
cg20593611	17.63	7.50	1.88E-02	28.79	24.80	2.46E-01	10.87	37.85	7.74E-01	22.75	8.95	1.10E-02	-8.54	18.39	6.42E-01
cg12059507	25.29	10.76	1.88E-02	25.58	18.92	1.76E-01	-54.60	61.73	3.76E-01	24.90	15.35	1.05E-01	41.73	27.43	1.28E-01
cg19413417	22.16	9.43	1.88E-02	20.74	14.10	1.41E-01	40.06	24.57	1.03E-01	14.71	28.03	6.00E-01	18.20	17.44	2.97E-01
cg02856338	13.97	5.94	1.88E-02	18.75	18.12	3.01E-01	-49.23	61.95	4.27E-01	11.09	7.27	1.27E-01	23.22	12.82	7.02E-02
cg13433729	-8.83	3.76	1.88E-02	-13.56	6.64	4.11E-02	-7.58	9.28	4.14E-01	-5.21	6.03	3.87E-01	-9.59	10.54	3.63E-01
cg13442333	21.32	9.07	1.88E-02	27.42	18.49	1.38E-01	16.96	21.20	4.24E-01	11.57	13.45	3.90E-01	52.37	26.06	4.44E-02

cg10296205	7.09	3.02	1.88E-02	5.70	3.99	1.52E-01	5.40	8.01	5.00E-01	6.64	9.25	4.73E-01	13.15	7.14	6.54E-02
cg10558494	22.43	9.55	1.88E-02	19.07	14.23	1.80E-01	19.31	28.72	5.01E-01	26.69	31.38	3.95E-01	26.65	16.20	1.00E-01
cg25116216	25.67	10.92	1.88E-02	11.07	39.29	7.78E-01	29.78	158.08	8.51E-01	26.10	11.79	2.68E-02	38.20	44.94	3.95E-01
cg12351749	21.76	9.26	1.88E-02	18.95	16.54	2.52E-01	9.83	16.94	5.62E-01	23.16	17.85	1.94E-01	56.18	26.93	3.70E-02
cg15612063	44.01	18.73	1.88E-02	35.70	25.45	1.61E-01	38.03	106.86	7.22E-01	79.21	74.74	2.89E-01	50.80	31.02	1.01E-01
cg26342533	-19.14	8.15	1.88E-02	-21.59	22.54	3.38E-01	1.25	33.43	9.70E-01	-20.24	9.51	3.33E-02	-20.23	29.45	4.92E-01
cg11855409	-11.36	4.84	1.88E-02	-16.01	9.21	8.22E-02	-14.15	12.48	2.57E-01	-5.25	7.39	4.77E-01	-17.69	12.68	1.63E-01
cg10792660	-12.14	5.17	1.88E-02	-16.11	11.12	1.47E-01	1.58	12.46	8.99E-01	-13.91	7.20	5.34E-02	-18.23	16.61	2.72E-01
cg02569129	19.65	8.36	1.88E-02	19.57	12.75	1.25E-01	6.14	21.58	7.76E-01	42.78	22.03	5.22E-02	15.03	15.95	3.46E-01
cg25386426	13.64	5.81	1.88E-02	18.83	14.83	2.04E-01	4.60	15.76	7.70E-01	12.44	7.32	8.94E-02	27.95	20.19	1.66E-01
cg12802921	27.66	11.77	1.88E-02	34.50	35.63	3.33E-01	7.00	177.27	9.68E-01	30.88	13.09	1.83E-02	-14.44	42.27	7.33E-01
cg18754335	16.85	7.17	1.88E-02	20.06	12.43	1.06E-01	23.13	20.28	2.54E-01	15.11	10.52	1.51E-01	3.30	25.82	8.98E-01
cg22146642	14.77	6.29	1.88E-02	21.01	12.60	9.55E-02	2.92	16.08	8.56E-01	13.96	9.01	1.21E-01	20.64	18.87	2.74E-01
cg12499749	21.52	9.16	1.88E-02	-1.87	29.46	9.49E-01	-66.92	137.09	6.25E-01	24.19	9.91	1.46E-02	30.07	43.83	4.93E-01
cg03594801	9.15	3.89	1.88E-02	7.50	6.00	2.11E-01	12.95	7.97	1.04E-01	-0.96	8.43	9.09E-01	20.10	9.32	3.11E-02
cg15092231	18.41	7.84	1.88E-02	17.69	12.39	1.53E-01	14.47	14.13	3.06E-01	13.99	22.00	5.25E-01	30.80	19.25	1.10E-01
cg10459187	-15.40	6.56	1.88E-02	-17.18	9.17	6.11E-02	-10.05	15.12	5.06E-01	1.01	19.65	9.59E-01	-25.53	15.04	8.96E-02
cg11052958	29.98	12.76	1.88E-02	13.60	11.95	2.55E-01	26.55	20.93	2.05E-01	13.07	42.34	7.58E-01	61.80	18.19	6.79E-04
cg02371464	18.88	8.04	1.88E-02	32.68	12.80	1.07E-02	40.19	26.34	1.27E-01	15.45	11.97	1.97E-01	0.37	14.00	9.79E-01
cg00917156	15.86	6.75	1.88E-02	17.74	8.17	2.98E-02	30.54	9.84	1.90E-03	-6.20	13.23	6.39E-01	13.68	11.33	2.27E-01
cg16661143	20.09	8.55	1.88E-02	17.62	14.39	2.21E-01	34.14	17.28	4.82E-02	7.99	16.46	6.27E-01	25.32	23.51	2.82E-01
cg26720452	19.86	8.46	1.88E-02	16.69	11.63	1.51E-01	17.90	19.10	3.49E-01	45.95	32.80	1.61E-01	21.43	18.50	2.47E-01
cg05888433	-9.30	3.96	1.88E-02	-0.19	8.50	9.82E-01	-3.35	23.54	8.87E-01	-13.06	4.94	8.14E-03	-6.82	11.87	5.66E-01
cg05002837	-15.83	6.74	1.88E-02	-37.31	18.52	4.39E-02	-11.95	24.10	6.20E-01	-13.01	8.03	1.05E-01	-9.25	23.16	6.90E-01
cg07618900	-8.62	3.67	1.88E-02	-11.07	6.97	1.13E-01	-11.59	12.45	3.52E-01	-5.39	5.22	3.02E-01	-13.24	9.73	1.74E-01
cg18754857	-8.24	3.51	1.88E-02	-10.48	6.95	1.32E-01	4.55	10.15	6.54E-01	-12.59	5.18	1.51E-02	-2.06	8.58	8.10E-01
cg25961579	-55.53	23.64	1.88E-02	-40.06	31.41	2.02E-01	45.93	104.94	6.62E-01	-69.91	74.96	3.51E-01	-99.59	44.43	2.50E-02
cg25096566	14.92	6.35	1.88E-02	4.57	19.80	8.17E-01	22.27	28.66	4.37E-01	18.44	7.80	1.80E-02	6.05	14.83	6.83E-01
cg10999000	-12.40	5.28	1.88E-02	-17.69	14.62	2.26E-01	-17.59	36.01	6.25E-01	-11.44	6.07	5.94E-02	-11.54	17.43	5.08E-01
cg20289911	-15.14	6.45	1.88E-02	-14.51	9.04	1.08E-01	-17.62	14.98	2.40E-01	-9.56	19.54	6.25E-01	-17.51	14.51	2.27E-01
cg20817941	12.30	5.24	1.88E-02	19.07	8.27	2.12E-02	12.94	10.52	2.18E-01	5.14	16.74	7.59E-01	3.73	10.39	7.19E-01
cg24411314	-16.46	7.01	1.88E-02	0.97	17.23	9.55E-01	-47.07	50.82	3.54E-01	-21.49	8.12	8.12E-03	4.24	26.49	8.73E-01
cg03770703	-9.09	3.87	1.89E-02	-0.14	9.64	9.89E-01	-17.03	16.53	3.03E-01	-9.87	4.65	3.38E-02	-14.13	12.77	2.69E-01
cg08354053	-13.17	5.61	1.89E-02	-19.35	8.50	2.28E-02	4.01	12.13	7.41E-01	-16.87	16.15	2.96E-01	-15.47	11.67	1.85E-01
cg07215511	103.69	44.15	1.89E-02	143.09	65.65	2.93E-02	246.94	232.52	2.88E-01	169.61	116.38	1.45E-01	15.34	72.82	8.33E-01

cg07473985	-23.80	10.13	1.89E-02	-32.62	15.61	3.67E-02	1.94	20.17	9.23E-01	-15.31	16.21	3.45E-01	-50.61	21.27	1.73E-02
ch.7.313144R	34.46	14.67	1.89E-02	46.44	19.78	1.89E-02	-20.11	64.91	7.57E-01	57.18	76.83	4.57E-01	21.65	24.39	3.75E-01
cg01206472	-39.60	16.86	1.89E-02	-25.10	22.01	2.54E-01	-100.00	67.94	1.41E-01	2.91	58.63	9.60E-01	-70.54	32.55	3.02E-02
cg08073142	40.10	17.08	1.89E-02	22.78	56.99	6.89E-01	-144.28	199.19	4.69E-01	41.19	18.71	2.77E-02	68.81	64.61	2.87E-01
cg17102069	-32.40	13.80	1.89E-02	-66.81	45.89	1.45E-01	-7.39	184.78	9.68E-01	-26.17	15.05	8.21E-02	-67.97	54.71	2.14E-01
cg04919425	-15.55	6.62	1.89E-02	-19.30	13.90	1.65E-01	-10.73	25.00	6.68E-01	-17.75	8.47	3.61E-02	4.68	21.83	8.30E-01
cg02358112	-30.55	13.01	1.89E-02	-3.57	39.88	9.29E-01	22.25	155.07	8.86E-01	-39.49	14.35	5.93E-03	33.02	51.16	5.19E-01
cg26514961	5.72	2.44	1.89E-02	3.92	3.53	2.68E-01	9.77	5.64	8.35E-02	7.55	4.91	1.25E-01	1.95	8.10	8.09E-01
cg05768427	9.67	4.12	1.89E-02	23.62	9.41	1.21E-02	6.30	10.17	5.36E-01	8.52	7.18	2.36E-01	4.15	7.32	5.71E-01
cg04760574	25.55	10.88	1.89E-02	20.38	11.68	8.11E-02	58.35	76.52	4.46E-01	89.95	40.13	2.50E-02	16.07	27.66	5.61E-01
cg23138119	-13.48	5.74	1.89E-02	-4.46	9.71	6.46E-01	-13.93	13.71	3.10E-01	-23.38	9.30	1.19E-02	-5.97	18.82	7.51E-01
cg00259388	-11.89	5.06	1.89E-02	-24.05	12.98	6.39E-02	-18.44	20.96	3.79E-01	-9.32	6.12	1.28E-01	-7.35	15.61	6.38E-01
cg04951476	-9.79	4.17	1.89E-02	-15.17	9.10	9.54E-02	-11.89	14.22	4.03E-01	-7.03	5.62	2.11E-01	-11.08	10.61	2.96E-01
cg03671123	21.95	9.35	1.89E-02	16.22	22.76	4.76E-01	90.84	48.52	6.12E-02	20.51	11.29	6.93E-02	16.35	28.37	5.64E-01
cg15924831	22.76	9.69	1.89E-02	15.39	15.96	3.35E-01	19.62	18.58	2.91E-01	19.69	19.94	3.23E-01	57.84	27.71	3.68E-02
cg25144207	8.84	3.77	1.89E-02	11.99	5.34	2.48E-02	-3.18	9.73	7.44E-01	11.62	9.04	1.99E-01	7.47	8.89	4.01E-01
cg25024001	66.98	28.54	1.89E-02	71.19	38.49	6.44E-02	171.59	162.97	2.92E-01	108.56	88.32	2.19E-01	35.68	50.84	4.83E-01
cg21279603	11.05	4.71	1.89E-02	13.85	6.51	3.34E-02	10.87	9.52	2.53E-01	1.56	17.60	9.30E-01	6.46	11.75	5.83E-01
cg15174564	12.29	5.24	1.89E-02	7.15	8.71	4.11E-01	9.85	9.70	3.10E-01	25.92	11.58	2.52E-02	10.78	13.90	4.38E-01
cg22599005	15.31	6.52	1.89E-02	9.77	9.85	3.21E-01	21.12	12.10	8.10E-02	2.03	19.67	9.18E-01	29.02	16.27	7.45E-02
cg15136597	38.00	16.19	1.89E-02	60.12	36.43	9.89E-02	-34.63	173.03	8.41E-01	33.70	19.44	8.31E-02	30.55	51.22	5.51E-01
cg01641509	-21.48	9.15	1.89E-02	-48.72	22.85	3.30E-02	-30.03	29.63	3.11E-01	-13.38	11.27	2.35E-01	-23.25	31.36	4.58E-01
cg14508237	12.75	5.43	1.89E-02	23.26	10.18	2.23E-02	-0.61	13.14	9.63E-01	12.03	8.39	1.51E-01	9.45	15.42	5.40E-01
cg22666015	5.58	2.38	1.90E-02	3.76	3.14	2.32E-01	10.84	5.65	5.49E-02	10.31	6.86	1.33E-01	2.06	6.61	7.55E-01
cg14265823	14.01	5.97	1.90E-02	20.00	10.94	6.76E-02	5.38	13.80	6.97E-01	22.25	11.95	6.26E-02	5.63	11.58	6.27E-01
cg27068650	16.55	7.05	1.90E-02	29.19	20.39	1.52E-01	12.83	24.88	6.06E-01	15.87	8.22	5.35E-02	5.31	28.01	8.50E-01
cg02152417	9.18	3.91	1.90E-02	2.62	7.75	7.36E-01	18.89	7.22	8.88E-03	4.80	7.17	5.03E-01	10.01	10.01	3.17E-01
cg06003958	-13.14	5.60	1.90E-02	-32.38	13.98	2.05E-02	8.13	25.29	7.48E-01	-9.10	4.91	6.38E-02	-20.31	14.52	1.62E-01
cg25535435	-15.16	6.46	1.90E-02	-22.13	20.46	2.80E-01	-24.52	29.85	4.12E-01	-14.74	7.24	4.16E-02	-0.90	27.26	9.74E-01
cg14508405	-11.03	4.70	1.90E-02	-0.55	9.03	9.51E-01	-23.24	13.46	8.43E-02	-13.16	6.66	4.80E-02	-13.70	14.30	3.38E-01
cg10432569	-10.50	4.48	1.90E-02	-12.11	8.92	1.75E-01	-8.96	13.82	5.17E-01	-7.98	6.50	2.20E-01	-16.14	10.88	1.38E-01
cg14970666	23.89	10.18	1.90E-02	40.56	18.28	2.65E-02	30.32	25.32	2.31E-01	21.71	20.63	2.93E-01	3.91	19.10	8.38E-01
cg27215108	-46.72	19.92	1.90E-02	-60.50	25.93	1.96E-02	30.27	60.29	6.16E-01	5.99	70.48	9.32E-01	-66.99	42.36	1.14E-01
cg09419983	22.14	9.44	1.90E-02	52.38	26.67	4.95E-02	-1.39	21.16	9.47E-01	23.43	12.78	6.69E-02	23.61	26.08	3.65E-01
cg17603321	39.36	16.78	1.90E-02	44.80	19.02	1.85E-02	63.29	130.16	6.27E-01	84.40	94.61	3.72E-01	4.52	40.26	9.11E-01

cg14965694	7.43	3.17	1.90E-02	11.70	4.71	1.29E-02	8.40	7.52	2.64E-01	9.46	11.28	4.02E-01	-0.34	5.86	9.54E-01
cg13482010	-11.29	4.81	1.90E-02	-13.64	9.38	1.46E-01	6.29	15.69	6.88E-01	-14.55	6.77	3.17E-02	-6.87	12.99	5.97E-01
cg27016863	-44.64	19.03	1.90E-02	-32.65	22.50	1.47E-01	-14.96	162.62	9.27E-01	-25.53	68.58	7.10E-01	-98.57	43.21	2.26E-02
cg01203365	27.22	11.60	1.90E-02	19.21	21.75	3.77E-01	42.58	42.82	3.20E-01	23.08	16.43	1.60E-01	49.69	30.66	1.05E-01
cg01695225	20.97	8.94	1.90E-02	24.48	20.58	2.34E-01	26.65	22.27	2.31E-01	22.04	13.89	1.13E-01	12.40	18.40	5.00E-01
cg13587564	25.75	10.98	1.90E-02	41.50	13.11	1.55E-03	3.93	19.93	8.44E-01	9.39	18.65	6.15E-01	50.56	30.55	9.79E-02
cg02102872	-11.17	4.76	1.90E-02	-16.69	8.34	4.53E-02	-5.41	12.27	6.60E-01	-6.19	8.06	4.42E-01	-15.83	11.43	1.66E-01
cg09777775	14.54	6.20	1.90E-02	44.18	21.04	3.58E-02	18.86	24.37	4.39E-01	10.93	7.09	1.23E-01	13.39	21.28	5.29E-01
cg14236758	8.68	3.70	1.90E-02	5.26	4.50	2.42E-01	16.40	9.81	9.46E-02	24.55	14.45	8.92E-02	10.27	10.99	3.50E-01
cg08687163	17.50	7.46	1.90E-02	18.36	12.03	1.27E-01	16.47	13.24	2.13E-01	7.50	20.80	7.19E-01	25.05	18.12	1.67E-01
cg25967505	30.40	12.96	1.90E-02	1.22	38.54	9.75E-01	110.41	144.48	4.45E-01	32.40	14.78	2.84E-02	40.49	39.06	3.00E-01
cg16904686	-10.07	4.29	1.90E-02	-4.42	6.94	5.24E-01	-12.21	7.85	1.20E-01	-27.38	13.39	4.09E-02	-8.85	9.23	3.38E-01
cg13485874	20.10	8.57	1.90E-02	18.94	16.10	2.39E-01	2.64	35.22	9.40E-01	19.58	11.52	8.93E-02	35.82	26.49	1.76E-01
cg27049194	-50.25	21.43	1.90E-02	-34.95	29.25	2.32E-01	-18.21	69.98	7.95E-01	-106.61	51.63	3.89E-02	-57.88	48.20	2.30E-01
cg24168061	-12.84	5.47	1.90E-02	-7.60	13.47	5.73E-01	-3.86	20.14	8.48E-01	-15.15	6.70	2.38E-02	-12.69	17.82	4.76E-01
cg00879497	-23.73	10.12	1.90E-02	-30.20	14.69	3.97E-02	-35.29	23.31	1.30E-01	-14.50	26.87	5.89E-01	-3.52	22.91	8.78E-01
cg24731441	18.54	7.91	1.90E-02	22.89	11.16	4.03E-02	12.01	19.42	5.36E-01	11.19	18.60	5.47E-01	20.06	20.28	3.23E-01
cg17139085	7.43	3.17	1.90E-02	7.72	4.59	9.27E-02	14.74	6.56	2.47E-02	-2.70	8.95	7.63E-01	3.97	7.76	6.09E-01
cg20792833	-15.97	6.81	1.90E-02	-3.96	9.48	6.76E-01	-29.96	18.08	9.76E-02	-13.52	11.57	2.43E-01	-31.98	13.57	1.84E-02
cg12975230	13.01	5.55	1.90E-02	50.38	26.39	5.63E-02	13.53	33.67	6.88E-01	9.45	6.24	1.30E-01	21.35	14.94	1.53E-01
cg22373181	22.81	9.73	1.90E-02	27.69	33.46	4.08E-01	141.25	144.31	3.28E-01	22.29	10.59	3.54E-02	15.30	37.36	6.82E-01
cg09413013	10.66	4.55	1.90E-02	8.51	6.96	2.21E-01	17.74	9.04	4.96E-02	3.40	12.92	7.93E-01	10.80	10.27	2.93E-01
cg26687072	22.23	9.48	1.90E-02	23.74	20.08	2.37E-01	26.15	20.74	2.07E-01	14.68	13.94	2.92E-01	44.37	29.18	1.28E-01
cg21904726	-38.18	16.28	1.90E-02	-57.18	21.38	7.47E-03	-31.93	44.56	4.74E-01	-17.84	63.20	7.78E-01	1.98	34.70	9.54E-01
cg17759564	14.39	6.13	1.90E-02	20.05	16.37	2.21E-01	3.43	20.35	8.66E-01	15.64	7.29	3.18E-02	2.82	25.10	9.11E-01
cg08182446	14.27	6.09	1.90E-02	24.85	14.33	8.29E-02	19.22	24.83	4.39E-01	12.03	7.83	1.24E-01	8.80	15.46	5.69E-01
cg13476133	18.65	7.95	1.90E-02	10.11	18.55	5.86E-01	48.55	41.16	2.38E-01	24.79	10.34	1.66E-02	1.71	18.37	9.26E-01
cg18308535	21.02	8.96	1.90E-02	9.14	17.96	6.11E-01	20.72	18.27	2.57E-01	30.05	14.97	4.48E-02	19.66	22.99	3.93E-01
cg17844831	8.00	3.41	1.90E-02	7.42	4.55	1.03E-01	10.69	8.10	1.87E-01	7.27	10.46	4.87E-01	7.48	8.70	3.89E-01
cg12275370	13.89	5.92	1.90E-02	11.18	8.17	1.71E-01	24.96	13.09	5.66E-02	15.04	18.86	4.25E-01	8.30	14.35	5.63E-01
cg19938567	-13.08	5.58	1.91E-02	-17.79	7.48	1.74E-02	-15.35	14.22	2.80E-01	13.81	24.93	5.80E-01	-6.32	11.41	5.79E-01
cg07912416	14.70	6.27	1.91E-02	18.57	10.15	6.74E-02	9.46	11.65	4.17E-01	14.89	14.68	3.10E-01	14.74	16.39	3.68E-01
cg08219438	20.96	8.94	1.91E-02	41.91	34.03	2.18E-01	44.92	142.14	7.52E-01	20.86	9.60	2.98E-02	-3.14	36.41	9.31E-01
cg22558265	16.40	7.00	1.91E-02	6.92	13.11	5.97E-01	40.56	27.13	1.35E-01	12.15	9.94	2.22E-01	37.34	17.88	3.68E-02
cg25643229	-16.25	6.93	1.91E-02	-15.40	10.83	1.55E-01	-25.87	16.15	1.09E-01	-21.59	14.14	1.27E-01	0.06	17.01	9.97E-01

cg07530947	8.35	3.56	1.91E-02	5.67	4.98	2.54E-01	19.81	8.76	2.38E-02	9.23	9.26	3.19E-01	4.62	8.54	5.89E-01
cg13838599	-10.22	4.36	1.91E-02	-13.51	9.55	1.57E-01	-25.84	19.85	1.93E-01	-11.72	6.97	9.27E-02	-4.47	7.35	5.43E-01
cg13652372	-18.60	7.93	1.91E-02	-1.36	19.01	9.43E-01	-32.70	46.57	4.83E-01	-20.72	9.34	2.65E-02	-32.93	29.08	2.57E-01
cg23594208	19.26	8.22	1.91E-02	12.13	18.98	5.23E-01	42.60	20.63	3.89E-02	21.28	11.23	5.82E-02	-9.71	23.83	6.84E-01
cg15492003	59.81	25.51	1.91E-02	35.60	31.70	2.61E-01	99.29	107.83	3.57E-01	106.39	79.44	1.80E-01	104.67	58.06	7.14E-02
cg24207176	25.37	10.82	1.91E-02	33.23	16.61	4.54E-02	6.03	28.10	8.30E-01	52.28	28.19	6.36E-02	9.51	20.46	6.42E-01
cg20064008	16.85	7.19	1.91E-02	22.68	17.25	1.88E-01	52.23	33.76	1.22E-01	12.08	8.67	1.64E-01	23.87	23.44	3.09E-01
cg25050951	15.30	6.53	1.91E-02	27.25	13.48	4.32E-02	2.97	16.99	8.61E-01	11.97	10.50	2.54E-01	16.62	13.58	2.21E-01
cg06631603	27.22	11.61	1.91E-02	35.96	35.37	3.09E-01	-155.62	171.44	3.64E-01	26.05	12.77	4.14E-02	41.61	47.26	3.79E-01
cg15831875	21.21	9.05	1.91E-02	13.74	12.58	2.75E-01	29.73	20.58	1.48E-01	46.05	27.52	9.42E-02	18.63	21.29	3.82E-01
cg10568066	2.75	1.17	1.91E-02	1.69	1.62	2.97E-01	4.14	2.31	7.26E-02	3.73	4.23	3.78E-01	3.69	3.18	2.46E-01
cg00394823	30.32	12.94	1.91E-02	24.08	16.60	1.47E-01	27.30	35.34	4.40E-01	62.94	46.22	1.73E-01	39.38	30.45	1.96E-01
cg18323018	18.55	7.91	1.91E-02	14.43	17.64	4.13E-01	17.85	16.97	2.93E-01	17.98	14.87	2.27E-01	22.39	14.50	1.23E-01
cg11913072	-23.90	10.20	1.91E-02	-30.54	35.50	3.90E-01	-167.86	135.79	2.16E-01	-22.66	10.97	3.88E-02	-17.75	46.83	7.05E-01
cg25250431	15.02	6.41	1.91E-02	15.06	15.91	3.44E-01	15.78	27.89	5.72E-01	13.78	7.53	6.74E-02	28.98	25.91	2.63E-01
cg22888055	11.23	4.79	1.91E-02	15.26	8.55	7.44E-02	13.58	10.01	1.75E-01	14.58	10.06	1.47E-01	0.08	9.99	9.94E-01
cg05080940	7.56	3.23	1.91E-02	8.05	5.10	1.15E-01	13.48	6.14	2.82E-02	-1.68	7.33	8.19E-01	7.26	8.94	4.17E-01
cg12191293	18.99	8.10	1.91E-02	27.34	11.78	2.03E-02	22.48	19.54	2.50E-01	32.68	30.74	2.88E-01	-0.29	15.17	9.85E-01
cg23718633	25.33	10.81	1.91E-02	62.08	18.52	8.04E-04	0.62	26.29	9.81E-01	18.63	10.99	9.01E-02	17.91	13.71	1.91E-01
cg21341487	6.58	2.81	1.91E-02	5.81	3.71	1.17E-01	14.38	7.12	4.35E-02	3.45	7.85	6.60E-01	3.99	7.42	5.90E-01
cg13912849	21.89	9.34	1.91E-02	18.19	21.02	3.87E-01	-15.11	43.49	7.28E-01	23.95	11.46	3.66E-02	33.60	30.90	2.77E-01
cg00832923	18.62	7.95	1.91E-02	-4.92	17.07	7.73E-01	22.53	26.40	3.93E-01	23.16	10.32	2.49E-02	39.33	25.18	1.18E-01
cg26107076	-20.03	8.55	1.91E-02	-62.53	29.36	3.32E-02	-78.78	159.97	6.22E-01	-16.71	9.12	6.68E-02	5.87	47.11	9.01E-01
cg26370409	41.84	17.86	1.91E-02	57.79	21.44	7.04E-03	-95.84	96.68	3.22E-01	31.25	54.14	5.64E-01	15.61	41.23	7.05E-01
cg07087018	-24.08	10.28	1.91E-02	-20.79	25.99	4.24E-01	-35.49	130.86	7.86E-01	-21.43	11.94	7.26E-02	-48.94	33.07	1.39E-01
cg15856599	26.03	11.11	1.91E-02	31.54	16.45	5.53E-02	20.07	29.86	5.02E-01	34.30	29.59	2.46E-01	15.25	21.61	4.80E-01
cg12872357	7.21	3.08	1.91E-02	4.70	5.19	3.65E-01	10.16	6.86	1.39E-01	8.22	5.19	1.13E-01	6.49	9.91	5.12E-01
cg21390363	-10.40	4.44	1.91E-02	-11.56	6.43	7.21E-02	-15.36	10.74	1.53E-01	-10.40	11.84	3.80E-01	-3.79	9.64	6.94E-01
cg08452095	-15.78	6.73	1.91E-02	-21.83	18.70	2.43E-01	5.66	26.01	8.28E-01	-15.08	7.84	5.46E-02	-33.44	26.19	2.02E-01
cg16777106	-10.43	4.45	1.91E-02	-2.20	12.48	8.60E-01	-9.36	18.82	6.19E-01	-12.39	5.13	1.58E-02	-4.73	17.58	7.88E-01
cg14690220	37.99	16.22	1.91E-02	47.13	46.91	3.15E-01	-231.76	160.97	1.50E-01	41.34	18.27	2.37E-02	25.96	56.30	6.45E-01
cg00632379	-8.94	3.82	1.91E-02	-11.89	9.98	2.34E-01	8.07	20.90	6.99E-01	-9.92	4.46	2.60E-02	-2.24	12.97	8.63E-01
cg25437886	4.62	1.97	1.91E-02	4.71	2.82	9.46E-02	10.07	4.81	3.62E-02	1.75	3.98	6.60E-01	1.96	6.34	7.57E-01
cg23165899	20.95	8.94	1.91E-02	35.52	12.88	5.83E-03	-0.15	17.78	9.93E-01	41.14	29.47	1.63E-01	12.69	14.41	3.78E-01
cg03733433	-52.70	22.50	1.91E-02	-38.84	54.23	4.74E-01	-116.90	240.15	6.26E-01	-59.60	26.25	2.32E-02	-14.52	77.23	8.51E-01

cg08668316	13.89	5.93	1.92E-02	22.46	11.32	4.72E-02	7.53	13.31	5.72E-01	5.92	12.89	6.46E-01	15.79	10.56	1.35E-01
cg08608240	-24.85	10.61	1.92E-02	-30.67	13.71	2.53E-02	28.87	45.34	5.24E-01	-14.47	27.47	5.98E-01	-29.91	23.87	2.10E-01
cg12550344	-9.47	4.04	1.92E-02	-8.33	6.58	2.06E-01	-16.98	8.36	4.22E-02	-8.81	9.45	3.52E-01	-3.61	8.92	6.86E-01
cg06909503	-13.03	5.56	1.92E-02	-31.29	16.10	5.20E-02	11.63	29.83	6.97E-01	-11.36	6.33	7.26E-02	-12.59	20.57	5.40E-01
cg25537119	-11.50	4.91	1.92E-02	-25.70	9.00	4.31E-03	-13.88	14.92	3.52E-01	-7.16	4.78	1.34E-01	-2.60	12.29	8.33E-01
cg10610428	-11.27	4.81	1.92E-02	-14.95	7.45	4.49E-02	-5.64	11.12	6.12E-01	-10.29	11.78	3.82E-01	-9.90	10.06	3.25E-01
cg07535477	17.18	7.33	1.92E-02	26.48	13.34	4.71E-02	-0.17	56.26	9.98E-01	13.07	9.45	1.67E-01	16.64	26.22	5.26E-01
cg23671699	8.06	3.44	1.92E-02	11.27	4.60	1.42E-02	1.01	8.33	9.04E-01	5.28	12.34	6.69E-01	6.08	7.88	4.40E-01
cg18531779	12.43	5.31	1.92E-02	-0.04	14.49	9.98E-01	11.81	14.34	4.10E-01	16.35	6.46	1.14E-02	-4.02	22.81	8.60E-01
cg23224128	20.74	8.86	1.92E-02	-2.67	17.54	8.79E-01	31.28	17.89	8.03E-02	20.98	14.95	1.61E-01	41.92	22.48	6.22E-02
cg17476951	8.56	3.65	1.92E-02	8.68	4.82	7.21E-02	14.87	9.95	1.35E-01	8.73	9.39	3.53E-01	1.80	9.78	8.54E-01
cg05327187	11.87	5.07	1.92E-02	13.08	7.23	7.03E-02	6.48	11.27	5.65E-01	10.58	19.23	5.82E-01	14.35	10.44	1.69E-01
cg02343602	-16.11	6.88	1.92E-02	-28.54	21.19	1.78E-01	-15.90	30.18	5.98E-01	-14.19	7.82	6.95E-02	-19.02	26.53	4.73E-01
cg01781974	28.20	12.04	1.92E-02	54.64	26.92	4.24E-02	-64.90	77.55	4.03E-01	24.70	14.83	9.59E-02	21.87	35.26	5.35E-01
cg07255925	19.36	8.27	1.92E-02	41.84	17.59	1.74E-02	27.80	39.15	4.78E-01	14.58	10.38	1.60E-01	-3.75	26.13	8.86E-01
cg26672614	44.22	18.88	1.92E-02	33.12	62.70	5.97E-01	-237.01	244.31	3.32E-01	53.47	20.63	9.54E-03	-33.40	73.90	6.51E-01
cg17138539	12.77	5.45	1.92E-02	17.15	14.36	2.33E-01	35.88	22.19	1.06E-01	8.70	6.35	1.71E-01	29.55	22.61	1.91E-01
cg13670531	-17.59	7.51	1.92E-02	-22.76	14.39	1.14E-01	-0.97	36.58	9.79E-01	-16.04	9.92	1.06E-01	-19.19	22.41	3.92E-01
cg26418770	-9.88	4.22	1.92E-02	-11.14	6.79	1.01E-01	-6.04	11.90	6.12E-01	-17.70	7.77	2.27E-02	2.06	9.60	8.30E-01
cg13581015	5.43	2.32	1.92E-02	4.32	3.18	1.75E-01	6.98	5.09	1.70E-01	9.05	6.08	1.36E-01	3.19	6.81	6.39E-01
cg01981354	23.65	10.10	1.92E-02	22.32	13.41	9.60E-02	51.56	41.52	2.14E-01	20.18	32.82	5.39E-01	21.61	19.14	2.59E-01
cg23994714	-15.83	6.76	1.92E-02	-13.95	9.86	1.57E-01	-16.65	16.25	3.05E-01	-18.83	19.43	3.33E-01	-17.46	13.96	2.11E-01
cg27627006	-23.85	10.19	1.92E-02	-19.18	13.94	1.69E-01	-36.26	24.61	1.41E-01	-13.41	34.13	6.94E-01	-30.20	22.51	1.80E-01
cg12046882	17.77	7.59	1.92E-02	18.45	13.52	1.72E-01	-2.05	22.72	9.28E-01	23.11	11.65	4.72E-02	15.92	19.68	4.19E-01
cg13778140	18.79	8.02	1.92E-02	31.09	15.54	4.55E-02	19.33	28.34	4.95E-01	16.92	10.83	1.18E-01	-3.34	24.90	8.93E-01
cg10315347	28.84	12.32	1.92E-02	72.46	29.97	1.56E-02	16.46	114.31	8.86E-01	22.10	14.65	1.32E-01	6.94	36.74	8.50E-01
cg17070935	12.67	5.41	1.92E-02	10.12	10.41	3.31E-01	11.79	18.96	5.34E-01	13.62	7.29	6.19E-02	15.15	17.36	3.83E-01
cg09368485	8.36	3.57	1.92E-02	10.96	4.62	1.76E-02	8.72	9.00	3.32E-01	1.01	12.35	9.35E-01	2.13	8.93	8.11E-01
cg16285854	-16.96	7.24	1.92E-02	-9.83	17.75	5.80E-01	-19.04	29.57	5.20E-01	-15.44	8.63	7.34E-02	-48.18	27.72	8.22E-02
cg21115391	-8.12	3.47	1.92E-02	-10.69	6.10	7.97E-02	-8.61	10.25	4.01E-01	-8.57	5.36	1.10E-01	-0.65	9.14	9.44E-01
cg01704924	-18.05	7.71	1.92E-02	-6.95	12.41	5.75E-01	-21.11	19.59	2.81E-01	-25.22	15.82	1.11E-01	-27.55	16.38	9.26E-02
cg05161682	-14.64	6.25	1.92E-02	-26.56	8.50	1.77E-03	-4.79	13.73	7.27E-01	-7.20	18.74	7.01E-01	-5.01	11.41	6.60E-01
cg04368664	17.72	7.57	1.92E-02	30.06	14.39	3.68E-02	9.86	13.05	4.50E-01	0.23	17.33	9.89E-01	30.78	17.08	7.15E-02
cg05155840	19.99	8.54	1.92E-02	16.74	22.43	4.55E-01	19.38	52.52	7.12E-01	20.25	10.18	4.67E-02	22.43	24.15	3.53E-01
cg06822067	-15.77	6.74	1.92E-02	-24.42	13.45	6.94E-02	-13.19	15.69	4.01E-01	-5.51	11.95	6.45E-01	-22.15	13.58	1.03E-01

cg10625737	-10.50	4.49	1.92E-02	-11.51	10.91	2.91E-01	4.97	25.18	8.43E-01	-10.47	5.43	5.37E-02	-13.40	13.16	3.08E-01
cg22665655	26.84	11.47	1.93E-02	91.80	39.50	2.01E-02	-17.14	168.62	9.19E-01	21.64	11.16	5.24E-02	13.65	49.24	7.82E-01
cg00489394	11.66	4.98	1.93E-02	7.89	6.24	2.07E-01	22.70	16.86	1.78E-01	-6.47	21.33	7.62E-01	22.60	10.58	3.27E-02
cg10898067	-12.51	5.35	1.93E-02	-17.75	9.51	6.20E-02	-4.16	11.28	7.12E-01	-16.72	9.08	6.58E-02	-1.62	15.89	9.19E-01
cg04592201	18.69	7.99	1.93E-02	4.62	21.13	8.27E-01	68.87	31.85	3.06E-02	16.56	9.22	7.26E-02	24.73	30.82	4.22E-01
cg25642825	-28.40	12.13	1.93E-02	-40.34	33.68	2.31E-01	-109.88	152.42	4.71E-01	-20.66	13.64	1.30E-01	-84.32	45.08	6.14E-02
cg19004465	-26.93	11.51	1.93E-02	-34.61	14.56	1.75E-02	-56.20	51.26	2.73E-01	-44.70	37.42	2.32E-01	7.56	23.98	7.52E-01
cg20740029	16.58	7.09	1.93E-02	19.09	10.72	7.48E-02	32.34	17.81	6.94E-02	17.12	21.05	4.16E-01	4.04	13.13	7.58E-01
cg08040428	25.61	10.94	1.93E-02	45.45	15.87	4.19E-03	8.05	36.47	8.25E-01	7.42	25.62	7.72E-01	7.63	21.81	7.26E-01
cg02935024	-17.39	7.43	1.93E-02	-29.45	14.09	3.66E-02	-17.48	16.04	2.76E-01	-5.04	13.07	7.00E-01	-20.72	17.30	2.31E-01
cg10500121	22.16	9.47	1.93E-02	39.34	22.00	7.37E-02	-55.36	56.18	3.24E-01	24.24	11.77	3.94E-02	5.38	25.40	8.32E-01
cg16102971	-14.04	6.00	1.93E-02	-21.95	9.29	1.81E-02	-3.25	14.93	8.28E-01	-19.60	16.06	2.22E-01	-5.76	11.30	6.10E-01
cg23552637	19.02	8.13	1.93E-02	18.53	19.13	3.33E-01	29.90	19.15	1.18E-01	9.44	11.00	3.91E-01	54.76	26.53	3.90E-02
cg17387903	-9.26	3.96	1.93E-02	-8.34	5.93	1.60E-01	-11.82	9.07	1.93E-01	-6.72	9.13	4.62E-01	-11.56	9.42	2.20E-01
cg08167066	23.34	9.98	1.93E-02	-1.90	27.85	9.46E-01	-93.68	94.36	3.21E-01	31.16	10.20	2.26E-03	10.49	29.21	7.19E-01
cg12769732	-12.73	5.44	1.93E-02	-24.24	9.87	1.41E-02	-7.03	15.75	6.56E-01	-11.01	8.53	1.97E-01	-0.30	13.19	9.82E-01
cg11630989	21.40	9.15	1.93E-02	3.63	13.62	7.89E-01	22.85	11.05	3.87E-02	19.37	11.16	8.27E-02	72.22	27.36	8.29E-03
cg00613752	14.53	6.21	1.93E-02	13.94	10.58	1.88E-01	29.02	15.05	5.37E-02	11.88	15.96	4.57E-01	8.95	10.76	4.06E-01
cg08832054	18.59	7.94	1.93E-02	31.09	15.23	4.13E-02	15.18	16.45	3.56E-01	19.30	13.02	1.38E-01	-4.86	22.71	8.30E-01
cg26230651	10.84	4.63	1.93E-02	3.96	7.58	6.01E-01	18.13	9.28	5.09E-02	13.61	10.71	2.04E-01	12.10	10.63	2.55E-01
cg18931108	48.10	20.56	1.93E-02	69.21	27.54	1.20E-02	13.39	65.77	8.39E-01	53.64	51.12	2.94E-01	-2.39	47.99	9.60E-01
cg00106499	-36.07	15.42	1.93E-02	-53.69	56.71	3.44E-01	-137.49	206.20	5.05E-01	-27.99	16.75	9.47E-02	-104.17	57.02	6.77E-02
cg12658374	10.99	4.70	1.93E-02	3.03	6.08	6.18E-01	17.19	9.70	7.62E-02	20.31	13.18	1.23E-01	19.98	11.30	7.69E-02
cg00870514	9.18	3.92	1.93E-02	2.58	5.96	6.65E-01	17.22	8.21	3.60E-02	12.96	10.40	2.13E-01	11.65	8.86	1.88E-01
cg10230257	-19.31	8.25	1.93E-02	-7.35	16.47	6.55E-01	-11.98	23.88	6.16E-01	-15.88	6.59	1.61E-02	-55.69	20.36	6.23E-03
cg08311656	14.94	6.39	1.93E-02	26.29	12.29	3.24E-02	17.80	17.20	3.01E-01	7.71	9.59	4.22E-01	13.25	16.57	4.24E-01
cg23635130	14.77	6.31	1.93E-02	11.07	9.45	2.41E-01	20.39	11.46	7.52E-02	4.76	24.54	8.46E-01	18.11	14.75	2.19E-01
cg15712559	15.21	6.50	1.93E-02	30.39	13.30	2.23E-02	9.94	16.34	5.43E-01	15.35	10.78	1.55E-01	3.31	13.29	8.03E-01
cg24367568	-53.00	22.66	1.93E-02	-57.39	29.60	5.25E-02	-49.18	83.52	5.56E-01	13.48	59.88	8.22E-01	-89.56	50.98	7.90E-02
cg24533864	-18.40	7.87	1.93E-02	-39.76	16.50	1.60E-02	-16.02	19.57	4.13E-01	-5.95	11.97	6.19E-01	-23.49	18.59	2.06E-01
cg07027305	8.73	3.73	1.93E-02	7.52	5.24	1.51E-01	12.84	7.71	9.58E-02	23.30	12.71	6.68E-02	-0.58	8.99	9.49E-01
cg03254137	9.68	4.14	1.93E-02	13.19	5.33	1.33E-02	13.13	12.35	2.88E-01	1.88	13.48	8.89E-01	0.39	9.49	9.68E-01
cg02049682	13.52	5.78	1.93E-02	14.81	8.32	7.50E-02	22.41	11.61	5.36E-02	6.29	16.62	7.05E-01	0.35	15.03	9.82E-01
cg19314992	-9.36	4.00	1.93E-02	-4.19	10.27	6.83E-01	-4.06	13.13	7.57E-01	-11.33	4.93	2.16E-02	-9.16	12.86	4.76E-01
cg25927687	24.39	10.43	1.93E-02	1.43	21.30	9.47E-01	51.46	25.06	4.00E-02	22.08	15.05	1.42E-01	42.48	31.94	1.84E-01

cg24467291	21.85	9.34	1.93E-02	26.04	15.44	9.18E-02	30.08	29.80	3.13E-01	1.80	27.06	9.47E-01	21.98	14.48	1.29E-01
cg21875336	38.45	16.44	1.94E-02	45.10	46.12	3.28E-01	70.52	156.81	6.53E-01	37.52	18.68	4.46E-02	32.94	55.56	5.53E-01
cg02835003	24.81	10.61	1.94E-02	35.82	21.39	9.41E-02	-2.53	46.95	9.57E-01	19.55	14.17	1.68E-01	36.31	28.12	1.97E-01
cg18817318	-9.40	4.02	1.94E-02	-2.29	4.91	6.40E-01	-14.12	8.63	1.02E-01	-20.08	12.84	1.18E-01	-14.64	7.08	3.86E-02
cg12523691	17.08	7.30	1.94E-02	9.94	17.52	5.71E-01	2.98	34.47	9.31E-01	20.20	8.66	1.96E-02	12.05	27.70	6.64E-01
cg05135158	20.87	8.93	1.94E-02	27.20	17.87	1.28E-01	15.74	15.37	3.06E-01	13.67	18.02	4.48E-01	32.30	21.77	1.38E-01
cg06731611	22.85	9.77	1.94E-02	38.70	22.32	8.29E-02	11.30	46.52	8.08E-01	18.81	12.00	1.17E-01	24.36	30.77	4.29E-01
cg15207826	17.15	7.34	1.94E-02	14.20	10.26	1.66E-01	27.44	18.52	1.39E-01	26.93	29.77	3.66E-01	14.58	14.08	3.00E-01
cg09964575	-36.84	15.76	1.94E-02	-35.00	19.17	6.79E-02	-9.88	65.25	8.80E-01	27.87	80.97	7.31E-01	-59.93	32.98	6.92E-02
cg26207357	22.05	9.43	1.94E-02	3.08	25.43	9.04E-01	15.09	25.25	5.50E-01	27.47	13.97	4.92E-02	26.19	18.24	1.51E-01
cg04619859	31.45	13.45	1.94E-02	56.49	42.72	1.86E-01	-182.54	191.53	3.41E-01	29.07	14.87	5.05E-02	38.27	48.51	4.30E-01
cg19010223	7.18	3.07	1.94E-02	4.44	4.86	3.61E-01	5.71	5.90	3.33E-01	6.81	6.59	3.01E-01	21.04	9.13	2.12E-02
cg17516106	52.51	22.46	1.94E-02	57.75	29.13	4.75E-02	65.81	55.34	2.34E-01	-16.45	47.18	7.27E-01	107.06	51.13	3.63E-02
cg02973171	12.92	5.53	1.94E-02	15.19	11.48	1.86E-01	3.71	14.45	7.97E-01	12.75	8.80	1.47E-01	16.84	11.60	1.47E-01
cg04503340	-15.34	6.56	1.94E-02	-21.93	12.90	8.91E-02	-4.03	15.81	7.99E-01	-19.63	9.33	3.54E-02	9.87	24.05	6.82E-01
cg15382497	-20.04	8.57	1.94E-02	-27.74	31.73	3.82E-01	-100.01	158.67	5.29E-01	-21.43	9.16	1.93E-02	21.20	38.80	5.85E-01
cg14851114	-13.93	5.96	1.94E-02	-20.70	14.64	1.57E-01	-5.48	30.38	8.57E-01	-16.64	7.77	3.23E-02	-2.44	13.07	8.52E-01
cg17685004	-7.56	3.23	1.94E-02	-10.10	5.09	4.72E-02	-13.39	11.33	2.37E-01	-1.43	5.25	7.85E-01	-13.72	8.81	1.20E-01
cg02293228	18.33	7.84	1.94E-02	13.26	9.17	1.48E-01	17.89	33.97	5.98E-01	45.66	50.62	3.67E-01	34.29	17.88	5.51E-02
cg22607760	12.63	5.40	1.94E-02	19.20	9.38	4.06E-02	-3.73	17.92	8.35E-01	10.77	7.92	1.74E-01	14.15	16.15	3.81E-01
cg23858605	20.70	8.85	1.94E-02	23.75	18.10	1.89E-01	31.28	18.85	9.69E-02	12.07	13.29	3.64E-01	28.57	28.50	3.16E-01
cg08321684	14.35	6.14	1.94E-02	29.30	12.71	2.11E-02	21.56	14.31	1.32E-01	5.22	8.67	5.47E-01	11.47	21.66	5.96E-01
cg11751213	9.13	3.90	1.94E-02	10.22	5.03	4.21E-02	13.59	10.27	1.86E-01	14.40	14.38	3.17E-01	-0.33	9.22	9.71E-01
cg07546878	-13.70	5.86	1.94E-02	-25.21	15.05	9.39E-02	20.19	36.71	5.82E-01	-12.84	6.83	6.04E-02	-10.85	19.73	5.83E-01
cg14699728	11.07	4.73	1.94E-02	22.18	7.81	4.49E-03	6.79	9.76	4.87E-01	14.45	6.91	3.65E-02	1.29	6.65	8.46E-01
cg09848445	-47.59	20.36	1.94E-02	-52.06	31.18	9.50E-02	205.71	158.57	1.95E-01	-46.56	39.49	2.38E-01	-56.32	37.72	1.35E-01
cg07448997	-9.65	4.13	1.94E-02	-11.35	8.55	1.85E-01	-7.94	15.74	6.14E-01	-9.58	5.52	8.26E-02	-7.91	11.04	4.74E-01
cg20787665	24.96	10.68	1.94E-02	43.27	21.21	4.14E-02	32.99	21.45	1.24E-01	10.90	17.81	5.41E-01	13.68	28.63	6.33E-01
cg20126656	-10.41	4.46	1.94E-02	-11.09	8.51	1.93E-01	-25.93	12.88	4.42E-02	-10.51	7.58	1.66E-01	-2.48	8.72	7.76E-01
cg04638679	22.19	9.49	1.94E-02	39.47	13.85	4.37E-03	10.24	26.68	7.01E-01	-3.91	29.79	8.96E-01	10.19	16.77	5.43E-01
cg10563131	19.64	8.40	1.94E-02	36.26	17.50	3.83E-02	3.37	17.08	8.43E-01	19.72	12.93	1.27E-01	20.35	25.96	4.33E-01
cg25795774	-9.82	4.20	1.94E-02	-11.61	9.96	2.44E-01	-41.77	22.58	6.43E-02	-7.92	5.06	1.18E-01	-8.65	13.42	5.19E-01
cg18242288	6.22	2.66	1.94E-02	7.47	3.47	3.14E-02	6.21	7.37	4.00E-01	-0.49	6.64	9.42E-01	9.02	7.62	2.36E-01
cg19083779	14.10	6.03	1.94E-02	17.37	19.52	3.73E-01	89.55	80.88	2.68E-01	9.87	6.94	1.55E-01	31.38	15.97	4.94E-02
cg17086139	-13.15	5.63	1.94E-02	-3.40	12.86	7.91E-01	-6.29	14.94	6.74E-01	-16.56	7.36	2.45E-02	-23.35	19.51	2.31E-01

cg20721135	13.20	5.65	1.94E-02	12.00	7.72	1.20E-01	14.49	14.77	3.27E-01	37.52	18.95	4.77E-02	5.77	11.80	6.25E-01
cg22378614	24.44	10.46	1.94E-02	13.32	23.54	5.72E-01	72.28	70.75	3.07E-01	24.81	12.46	4.64E-02	36.19	37.95	3.40E-01
cg19604323	-10.45	4.47	1.94E-02	-5.85	13.07	6.55E-01	9.87	19.15	6.06E-01	-13.41	5.13	8.97E-03	-1.74	17.01	9.18E-01
cg14603406	-27.90	11.94	1.94E-02	-13.84	18.57	4.56E-01	-71.96	32.13	2.51E-02	-20.21	23.62	3.92E-01	-36.63	27.16	1.77E-01
cg13546173	-32.73	14.01	1.94E-02	-50.87	17.22	3.13E-03	9.89	33.32	7.67E-01	-34.92	61.16	5.68E-01	-13.35	32.13	6.78E-01
cg04599652	44.24	18.93	1.94E-02	27.97	48.82	5.67E-01	-302.78	258.73	2.42E-01	52.59	21.42	1.41E-02	9.26	75.24	9.02E-01
cg23729777	-10.07	4.31	1.94E-02	-12.89	6.65	5.26E-02	-7.84	8.98	3.83E-01	-1.25	11.87	9.16E-01	-12.29	9.20	1.82E-01
cg10431340	-10.57	4.52	1.95E-02	-24.79	9.38	8.19E-03	-10.21	21.31	6.32E-01	-7.27	5.11	1.55E-01	-1.21	13.70	9.29E-01
cg15839219	16.27	6.96	1.95E-02	29.10	9.57	2.36E-03	1.21	15.87	9.39E-01	23.68	18.13	1.91E-01	6.57	10.33	5.24E-01
cg06546469	-9.39	4.02	1.95E-02	-7.99	5.74	1.64E-01	-11.29	9.79	2.49E-01	-10.77	11.17	3.35E-01	-10.25	8.70	2.39E-01
cg23092777	20.77	8.89	1.95E-02	21.47	9.38	2.21E-02	-54.54	101.12	5.90E-01	24.11	56.73	6.71E-01	18.95	33.83	5.75E-01
cg00517202	-6.68	2.86	1.95E-02	-2.63	4.58	5.65E-01	-4.99	7.22	4.90E-01	-8.83	5.21	8.97E-02	-14.52	7.33	4.76E-02
cg13694662	12.75	5.46	1.95E-02	11.99	10.02	2.31E-01	27.33	15.77	8.30E-02	4.47	8.91	6.16E-01	20.41	11.98	8.84E-02
cg02974456	23.43	10.03	1.95E-02	36.59	23.56	1.20E-01	21.04	19.68	2.85E-01	11.29	12.85	3.80E-01	69.15	31.86	3.00E-02
cg17514199	-15.85	6.78	1.95E-02	-18.37	11.32	1.05E-01	-25.88	22.58	2.52E-01	-22.58	5.13	1.08E-05	6.28	12.30	6.10E-01
cg26839871	28.66	12.27	1.95E-02	48.20	22.94	3.57E-02	-7.25	44.05	8.69E-01	31.45	17.94	7.96E-02	4.40	29.83	8.83E-01
cg10012079	38.22	16.36	1.95E-02	37.15	21.75	8.77E-02	23.24	38.80	5.49E-01	92.19	35.07	8.56E-03	6.74	30.89	8.27E-01
cg06588782	-9.79	4.19	1.95E-02	-6.13	9.84	5.34E-01	-5.74	15.27	7.07E-01	-9.83	5.25	6.14E-02	-18.70	12.83	1.45E-01
cg23638252	18.94	8.10	1.95E-02	26.87	13.02	3.91E-02	13.24	24.15	5.84E-01	26.23	19.64	1.82E-01	7.80	14.11	5.81E-01
cg13344886	-13.34	5.71	1.95E-02	-20.46	10.07	4.22E-02	-4.27	14.38	7.67E-01	-16.72	12.03	1.64E-01	-7.86	10.50	4.54E-01
cg21258596	-21.06	9.02	1.95E-02	-19.75	13.53	1.44E-01	-22.25	24.22	3.58E-01	-29.83	22.95	1.94E-01	-17.50	17.58	3.20E-01
cg22841258	-19.39	8.30	1.95E-02	9.53	26.06	7.15E-01	48.48	100.34	6.29E-01	-21.39	9.33	2.19E-02	-37.49	26.17	1.52E-01
cg15101392	11.79	5.05	1.95E-02	2.67	7.72	7.29E-01	31.56	12.81	1.38E-02	13.87	5.48	1.14E-02	5.95	11.81	6.14E-01
cg22041712	13.89	5.94	1.95E-02	22.93	15.07	1.28E-01	38.47	20.10	5.56E-02	9.47	7.21	1.89E-01	6.68	21.39	7.55E-01
cg18689454	6.98	2.99	1.95E-02	5.26	3.82	1.69E-01	3.76	7.90	6.35E-01	14.23	10.35	1.69E-01	12.59	7.42	8.97E-02
cg14068146	104.01	44.52	1.95E-02	58.01	51.72	2.62E-01	397.39	161.51	1.39E-02	59.65	88.48	5.00E-01	120.57	55.89	3.10E-02
cg00462560	18.10	7.75	1.95E-02	29.72	21.16	1.60E-01	25.48	24.02	2.89E-01	15.83	9.34	9.02E-02	7.79	28.44	7.84E-01
cg00261690	5.75	2.46	1.95E-02	4.47	2.85	1.17E-01	12.84	5.81	2.72E-02	9.86	7.05	1.62E-01	-1.83	6.93	7.91E-01
cg02164225	34.58	14.81	1.95E-02	40.48	15.73	1.01E-02	5.35	28.27	8.50E-01	96.77	33.36	3.73E-03	17.86	15.65	2.54E-01
cg19879330	18.70	8.01	1.95E-02	5.65	15.49	7.15E-01	-4.62	31.15	8.82E-01	22.65	10.70	3.43E-02	45.10	24.50	6.56E-02
cg14523602	34.96	14.97	1.95E-02	32.88	21.40	1.24E-01	50.19	54.71	3.59E-01	38.93	41.94	3.53E-01	32.94	26.95	2.22E-01
cg20999167	-12.77	5.47	1.95E-02	-7.78	7.08	2.72E-01	-22.28	13.78	1.06E-01	-9.88	17.32	5.68E-01	-24.89	14.31	8.19E-02
cg21115727	16.24	6.95	1.95E-02	23.71	21.89	2.79E-01	-11.48	27.07	6.72E-01	19.50	7.86	1.31E-02	-12.89	30.81	6.76E-01
cg00621646	-8.48	3.63	1.95E-02	-11.37	8.82	1.97E-01	-1.76	14.57	9.04E-01	-8.55	4.48	5.64E-02	-7.40	10.84	4.95E-01
cg09597192	17.94	7.68	1.95E-02	-0.56	19.46	9.77E-01	50.85	23.86	3.31E-02	18.52	9.74	5.73E-02	10.48	22.24	6.37E-01

cg23142799	11.53	4.94	1.95E-02	19.33	8.33	2.03E-02	21.54	13.26	1.04E-01	2.17	9.41	8.18E-01	4.95	10.18	6.27E-01
cg08769634	-14.04	6.01	1.95E-02	-21.53	13.65	1.15E-01	-19.39	30.99	5.32E-01	-10.06	7.37	1.72E-01	-23.71	18.72	2.05E-01
cg10104336	-11.74	5.03	1.95E-02	-4.47	9.54	6.39E-01	-10.01	10.92	3.59E-01	-12.50	8.60	1.46E-01	-24.32	12.24	4.69E-02
cg12963656	7.09	3.04	1.95E-02	8.88	4.03	2.75E-02	6.26	7.38	3.96E-01	2.39	11.98	8.42E-01	4.20	6.81	5.37E-01
cg05208812	40.43	17.31	1.95E-02	51.44	14.88	5.44E-04	-7.64	47.66	8.73E-01	161.99	76.66	3.46E-02	24.87	20.37	2.22E-01
cg05982686	-13.67	5.85	1.95E-02	-15.02	7.47	4.42E-02	-18.43	16.85	2.74E-01	-25.98	20.29	2.00E-01	-0.29	13.73	9.83E-01
cg24637364	14.01	6.00	1.95E-02	12.78	13.98	3.61E-01	10.14	19.36	6.01E-01	12.58	9.33	1.78E-01	17.92	10.85	9.86E-02
cg12957265	-9.36	4.01	1.95E-02	-6.37	4.90	1.94E-01	-1.58	9.97	8.74E-01	-10.18	9.62	2.90E-01	-23.06	8.87	9.36E-03
cg24765446	-9.53	4.08	1.95E-02	-9.13	9.78	3.51E-01	-30.25	19.85	1.27E-01	-9.30	4.88	5.69E-02	-1.97	13.93	8.87E-01
cg16420199	14.97	6.41	1.96E-02	42.27	18.93	2.55E-02	8.37	25.64	7.44E-01	13.48	8.53	1.14E-01	7.68	12.65	5.44E-01
cg09048186	24.95	10.69	1.96E-02	32.05	13.42	1.69E-02	6.27	55.23	9.10E-01	50.15	35.39	1.56E-01	-0.73	21.93	9.73E-01
cg16179507	10.17	4.36	1.96E-02	6.77	6.22	2.76E-01	9.25	10.17	3.63E-01	0.59	16.87	9.72E-01	19.74	8.56	2.12E-02
cg14292368	-8.62	3.69	1.96E-02	-11.67	5.60	3.72E-02	-1.67	8.28	8.40E-01	-8.40	11.47	4.64E-01	-8.93	7.21	2.16E-01
cg23941527	-9.03	3.87	1.96E-02	-13.47	7.66	7.86E-02	1.21	10.16	9.06E-01	-8.33	5.63	1.39E-01	-14.43	10.86	1.84E-01
cg23511285	6.67	2.86	1.96E-02	5.86	3.47	9.17E-02	12.43	7.33	9.01E-02	3.77	10.08	7.09E-01	5.65	9.43	5.49E-01
cg17537073	3.20	1.37	1.96E-02	4.93	2.31	3.27E-02	4.93	2.88	8.67E-02	1.37	3.49	6.93E-01	0.49	2.66	8.55E-01
cg19987768	15.28	6.55	1.96E-02	15.69	9.08	8.39E-02	8.24	16.82	6.24E-01	28.42	23.06	2.18E-01	14.46	13.15	2.71E-01
cg08522428	-34.85	14.93	1.96E-02	-23.35	43.74	5.93E-01	-56.18	153.39	7.14E-01	-34.92	16.67	3.62E-02	-50.08	55.88	3.70E-01
cg14134703	18.18	7.79	1.96E-02	29.08	20.91	1.64E-01	36.87	25.15	1.43E-01	11.15	9.33	2.32E-01	41.81	29.89	1.62E-01
cg14433070	12.22	5.24	1.96E-02	11.38	13.75	4.08E-01	2.03	17.65	9.08E-01	12.94	6.36	4.18E-02	18.18	17.55	3.00E-01
cg04738877	-13.00	5.57	1.96E-02	-4.66	9.92	6.39E-01	-15.23	13.89	2.73E-01	-15.29	9.79	1.18E-01	-20.58	12.41	9.74E-02
cg27366891	-51.82	22.20	1.96E-02	-37.01	27.71	1.82E-01	-58.25	116.28	6.16E-01	-118.76	81.01	1.43E-01	-69.07	44.74	1.23E-01
cg20985486	31.48	13.49	1.96E-02	9.35	46.13	8.39E-01	-208.03	206.08	3.13E-01	34.29	14.60	1.89E-02	40.70	56.62	4.72E-01
cg05718255	-9.33	4.00	1.96E-02	-8.65	5.25	9.94E-02	-17.77	9.66	6.58E-02	-1.07	14.51	9.41E-01	-6.88	9.63	4.75E-01
cg23438868	-15.11	6.48	1.96E-02	-28.62	13.21	3.02E-02	-16.43	33.41	6.23E-01	-9.91	8.26	2.30E-01	-14.20	19.81	4.74E-01
cg02375585	-12.88	5.52	1.96E-02	-13.20	10.73	2.19E-01	-14.07	12.25	2.51E-01	-11.63	9.11	2.02E-01	-13.68	13.54	3.12E-01
cg18110104	-12.17	5.21	1.96E-02	-30.52	14.74	3.84E-02	0.65	27.69	9.81E-01	-10.26	6.03	8.88E-02	-7.64	17.22	6.57E-01
cg03117556	-17.01	7.29	1.96E-02	-23.20	14.18	1.02E-01	-52.52	23.23	2.37E-02	-11.31	7.51	1.32E-01	-2.59	23.24	9.11E-01
cg11859489	-12.97	5.56	1.96E-02	-42.64	18.64	2.21E-02	-3.78	23.58	8.73E-01	-10.42	6.16	9.07E-02	-11.68	27.20	6.68E-01
cg17515760	-15.10	6.47	1.96E-02	-24.26	11.31	3.19E-02	-14.61	16.16	3.66E-01	-6.14	5.33	2.50E-01	-34.80	16.54	3.54E-02
cg17347253	7.93	3.40	1.96E-02	2.19	5.64	6.98E-01	14.78	7.27	4.21E-02	10.91	6.42	8.93E-02	6.12	9.10	5.01E-01
cg00681462	14.64	6.27	1.96E-02	15.55	12.88	2.27E-01	22.38	23.83	3.48E-01	12.42	9.64	1.98E-01	15.34	12.08	2.04E-01
cg01594662	-5.78	2.48	1.96E-02	-4.08	4.25	3.37E-01	-1.99	5.78	7.31E-01	-10.02	4.29	1.96E-02	-4.83	6.55	4.60E-01
cg02362506	31.58	13.54	1.96E-02	21.14	16.08	1.89E-01	47.22	108.70	6.64E-01	45.27	74.44	5.43E-01	59.12	27.44	3.12E-02
cg23958948	-46.98	20.14	1.96E-02	-37.38	26.97	1.66E-01	-122.40	60.36	4.26E-02	-5.68	73.51	9.38E-01	-47.21	39.76	2.35E-01

cg07790727	-18.35	7.87	1.96E-02	-37.38	18.59	4.44E-02	-13.37	29.77	6.53E-01	-19.65	8.68	2.35E-02	15.03	23.11	5.15E-01
cg10926083	-18.26	7.82	1.96E-02	-15.68	14.60	2.83E-01	-5.42	18.95	7.75E-01	-17.69	13.31	1.84E-01	-34.15	17.64	5.28E-02
cg15472784	21.40	9.17	1.96E-02	-14.69	36.85	6.90E-01	-3.36	114.88	9.77E-01	24.85	9.97	1.27E-02	15.25	31.32	6.26E-01
cg24577160	11.77	5.04	1.96E-02	7.86	9.18	3.92E-01	24.82	11.65	3.32E-02	13.40	6.97	5.45E-02	-9.83	16.44	5.50E-01
cg08324090	4.78	2.05	1.96E-02	7.03	3.12	2.42E-02	8.61	4.85	7.58E-02	1.18	3.67	7.47E-01	-2.05	7.35	7.81E-01
cg21186966	-12.31	5.28	1.96E-02	-11.86	8.39	1.57E-01	-2.35	12.28	8.48E-01	-29.85	13.89	3.17E-02	-10.44	10.05	2.98E-01
cg09440493	-21.38	9.16	1.96E-02	-29.01	20.66	1.60E-01	-53.43	48.09	2.67E-01	-12.96	11.19	2.47E-01	-51.97	29.39	7.70E-02
cg18462898	5.96	2.55	1.97E-02	5.17	3.12	9.72E-02	6.17	6.59	3.49E-01	10.01	9.93	3.13E-01	7.92	7.57	2.95E-01
cg00676925	14.28	6.12	1.97E-02	23.28	14.63	1.12E-01	23.93	19.27	2.14E-01	11.58	7.48	1.22E-01	0.74	26.09	9.77E-01
cg01518225	4.66	2.00	1.97E-02	3.34	2.83	2.38E-01	8.83	4.43	4.62E-02	-1.57	6.70	8.15E-01	6.44	4.39	1.42E-01
cg12493761	-10.23	4.39	1.97E-02	-7.34	10.57	4.87E-01	-3.32	11.30	7.69E-01	-12.20	5.78	3.49E-02	-14.26	13.76	3.00E-01
cg07877987	-15.85	6.80	1.97E-02	-17.39	10.35	9.29E-02	-5.07	15.53	7.44E-01	-38.32	18.54	3.88E-02	-9.20	13.79	5.05E-01
cg14085203	-41.09	17.61	1.97E-02	-35.69	22.45	1.12E-01	-33.34	49.96	5.05E-01	-46.63	56.97	4.13E-01	-63.95	43.46	1.41E-01
cg05582310	-8.49	3.64	1.97E-02	-15.47	7.58	4.14E-02	-6.09	10.85	5.74E-01	-5.48	4.98	2.72E-01	-10.60	10.30	3.03E-01
cg05155595	8.16	3.50	1.97E-02	8.33	6.20	1.79E-01	7.12	9.48	4.53E-01	12.14	5.34	2.30E-02	-5.64	10.21	5.80E-01
cg20972416	-46.10	19.77	1.97E-02	-42.84	63.88	5.02E-01	-3.97	272.67	9.88E-01	-47.39	21.93	3.07E-02	-40.13	67.26	5.51E-01
cg24365417	-9.29	3.98	1.97E-02	-10.18	7.89	1.97E-01	-11.39	8.72	1.92E-01	-8.27	5.91	1.61E-01	-6.81	13.91	6.25E-01
cg26843807	-6.80	2.91	1.97E-02	-12.91	4.82	7.35E-03	-0.16	6.28	9.80E-01	-3.62	5.17	4.84E-01	-8.34	7.32	2.55E-01
cg14666113	15.92	6.83	1.97E-02	20.89	24.76	3.99E-01	20.28	37.37	5.87E-01	17.54	7.95	2.74E-02	4.75	17.44	7.85E-01
cg01599904	-40.80	17.49	1.97E-02	-39.25	22.79	8.51E-02	-58.84	57.99	3.10E-01	-139.39	78.83	7.70E-02	-20.18	33.61	5.48E-01
cg10062617	8.82	3.78	1.97E-02	8.54	6.85	2.12E-01	12.38	17.04	4.68E-01	7.61	5.05	1.32E-01	15.61	12.88	2.26E-01
cg15598442	-65.74	28.19	1.97E-02	-118.59	61.30	5.31E-02	222.58	270.89	4.11E-01	-52.38	35.27	1.38E-01	-69.20	75.61	3.60E-01
cg00637687	13.75	5.90	1.97E-02	13.62	8.51	1.09E-01	21.80	14.87	1.43E-01	12.93	12.07	2.84E-01	5.62	16.74	7.37E-01
cg11075878	18.98	8.14	1.97E-02	16.59	19.62	3.98E-01	44.88	70.30	5.23E-01	18.95	9.52	4.65E-02	20.05	28.21	4.77E-01
cg27640064	9.98	4.28	1.97E-02	5.01	5.96	4.00E-01	23.33	10.20	2.21E-02	9.73	12.15	4.23E-01	11.35	10.01	2.57E-01
cg10234841	-35.02	15.02	1.97E-02	-91.76	53.98	8.92E-02	-216.05	241.28	3.71E-01	-31.40	16.21	5.28E-02	-2.28	60.94	9.70E-01
cg02708704	-21.55	9.24	1.97E-02	-29.55	16.21	6.84E-02	-8.43	39.32	8.30E-01	-13.75	13.00	2.90E-01	-39.76	27.38	1.47E-01
cg27177415	-30.54	13.10	1.97E-02	-17.81	25.03	4.77E-01	3.42	88.99	9.69E-01	-41.53	22.59	6.60E-02	-31.97	21.59	1.39E-01
cg19779379	24.50	10.51	1.97E-02	26.44	20.25	1.92E-01	16.47	20.90	4.31E-01	10.41	16.66	5.32E-01	63.52	25.30	1.20E-02
cg27151677	13.02	5.58	1.97E-02	22.50	8.09	5.40E-03	-0.78	10.22	9.39E-01	15.04	16.81	3.71E-01	11.16	11.93	3.50E-01
cg14735741	-10.70	4.59	1.97E-02	-16.96	13.41	2.06E-01	8.84	25.33	7.27E-01	-10.93	5.16	3.42E-02	-6.08	18.69	7.45E-01
cg00476608	6.55	2.81	1.97E-02	6.25	4.53	1.68E-01	15.51	7.01	2.69E-02	4.38	4.77	3.58E-01	1.29	8.52	8.80E-01
cg03446502	19.17	8.22	1.97E-02	6.40	19.91	7.48E-01	40.67	18.63	2.90E-02	12.58	11.35	2.67E-01	32.54	24.93	1.92E-01
cg20266104	22.83	9.79	1.97E-02	23.60	11.77	4.49E-02	23.08	42.80	5.90E-01	32.55	39.77	4.13E-01	16.99	22.21	4.44E-01
cg10919177	16.57	7.11	1.97E-02	8.16	10.64	4.43E-01	21.28	17.16	2.15E-01	31.39	17.32	6.99E-02	18.67	15.38	2.25E-01

cg26389330	8.58	3.68	1.97E-02	10.45	5.19	4.40E-02	6.38	8.76	4.67E-01	13.12	9.02	1.46E-01	0.12	9.34	9.90E-01
cg15175194	-27.17	11.66	1.98E-02	6.86	28.50	8.10E-01	75.59	98.37	4.42E-01	-35.11	13.81	1.10E-02	-41.08	35.66	2.49E-01
cg11953015	-10.54	4.52	1.98E-02	-21.43	9.25	2.05E-02	-4.33	12.96	7.38E-01	-9.35	6.13	1.27E-01	2.04	14.63	8.89E-01
cg10113414	-10.29	4.42	1.98E-02	-15.46	7.33	3.49E-02	-2.55	8.96	7.76E-01	0.89	11.83	9.40E-01	-16.44	8.75	6.03E-02
cg19269530	-11.31	4.85	1.98E-02	-15.32	7.15	3.22E-02	10.26	13.44	4.45E-01	-13.58	13.45	3.13E-01	-13.73	9.19	1.35E-01
cg22256309	-12.97	5.56	1.98E-02	-21.20	12.38	8.66E-02	-7.73	13.86	5.77E-01	-15.94	8.07	4.82E-02	0.87	13.84	9.50E-01
cg24713499	21.79	9.35	1.98E-02	63.49	35.68	7.52E-02	-13.35	116.73	9.09E-01	18.37	10.06	6.79E-02	26.84	37.58	4.75E-01
cg14020129	-25.35	10.88	1.98E-02	-27.50	39.54	4.87E-01	24.91	163.03	8.79E-01	-29.74	11.73	1.13E-02	35.76	44.12	4.18E-01
cg00231405	-40.72	17.47	1.98E-02	-32.28	23.12	1.63E-01	-40.68	56.36	4.70E-01	16.61	53.87	7.58E-01	-88.47	36.64	1.58E-02
cg23005797	-18.30	7.85	1.98E-02	-13.39	13.47	3.20E-01	-2.96	40.93	9.42E-01	-28.04	17.51	1.09E-01	-18.95	12.08	1.17E-01
cg15591386	-9.02	3.87	1.98E-02	-5.41	8.04	5.01E-01	-1.85	10.95	8.66E-01	-11.80	5.28	2.53E-02	-11.27	11.93	3.45E-01
cg08587864	-10.12	4.34	1.98E-02	-13.27	6.08	2.90E-02	-12.34	10.99	2.62E-01	-1.00	11.82	9.33E-01	-6.48	9.77	5.08E-01
cg10326833	17.23	7.39	1.98E-02	1.22	17.13	9.43E-01	21.60	16.48	1.90E-01	21.19	10.11	3.62E-02	17.07	26.42	5.18E-01
cg26428889	33.64	14.44	1.98E-02	42.61	17.27	1.36E-02	50.77	52.20	3.31E-01	-8.90	75.73	9.06E-01	1.60	33.28	9.62E-01
cg25223055	20.86	8.95	1.98E-02	27.71	15.60	7.58E-02	11.48	15.58	4.61E-01	22.08	20.19	2.74E-01	25.07	23.60	2.88E-01
cg23175215	-35.00	15.02	1.98E-02	-62.85	39.57	1.12E-01	-154.28	227.05	4.97E-01	-35.12	16.89	3.75E-02	42.03	61.30	4.93E-01
cg21495385	-22.64	9.72	1.98E-02	-40.18	20.99	5.57E-02	1.18	42.69	9.78E-01	-27.39	10.77	1.10E-02	13.10	24.86	5.98E-01
cg00661970	14.87	6.38	1.98E-02	18.80	17.78	2.90E-01	35.51	39.54	3.69E-01	13.46	7.68	7.96E-02	14.42	16.25	3.75E-01
cg11749010	-6.84	2.93	1.98E-02	-10.71	5.49	5.13E-02	-5.13	5.23	3.27E-01	1.61	6.87	8.14E-01	-11.32	6.29	7.20E-02
cg25537434	16.88	7.24	1.98E-02	29.57	12.89	2.18E-02	15.58	16.96	3.58E-01	9.75	14.43	4.99E-01	8.98	14.49	5.35E-01
cg03002136	27.03	11.60	1.98E-02	37.79	13.83	6.27E-03	-44.65	64.09	4.86E-01	27.25	54.67	6.18E-01	3.04	24.82	9.02E-01
cg21876925	10.98	4.71	1.98E-02	18.08	5.90	2.17E-03	9.38	10.85	3.87E-01	4.03	16.15	8.03E-01	-0.99	9.34	9.16E-01
cg00374672	9.76	4.19	1.98E-02	13.51	5.76	1.91E-02	15.13	10.48	1.49E-01	1.23	10.28	9.05E-01	0.05	10.94	9.96E-01
cg20939320	-10.43	4.48	1.98E-02	0.21	10.45	9.84E-01	-9.58	14.08	4.96E-01	-14.85	5.84	1.10E-02	-6.03	12.56	6.31E-01
cg07751039	22.48	9.65	1.98E-02	45.39	23.54	5.38E-02	19.79	29.12	4.97E-01	19.83	11.94	9.69E-02	-3.75	36.62	9.18E-01
cg07956247	-19.10	8.20	1.98E-02	-16.95	12.51	1.75E-01	-9.00	20.35	6.58E-01	-46.88	19.90	1.85E-02	-10.09	16.80	5.48E-01
cg23341612	12.19	5.23	1.98E-02	13.01	12.36	2.93E-01	-6.04	17.52	7.30E-01	13.02	6.48	4.44E-02	23.85	18.52	1.98E-01
cg10040748	12.81	5.50	1.98E-02	13.24	8.11	1.03E-01	10.95	13.42	4.15E-01	19.80	15.34	1.97E-01	9.60	11.13	3.88E-01
cg03218303	32.73	14.05	1.98E-02	48.06	46.63	3.03E-01	204.47	175.13	2.43E-01	33.84	15.27	2.67E-02	-28.37	59.23	6.32E-01
cg19324978	-33.94	14.57	1.98E-02	-22.29	20.01	2.65E-01	-45.75	83.91	5.86E-01	-49.29	35.01	1.59E-01	-45.82	28.23	1.05E-01
cg11350278	-20.46	8.78	1.98E-02	-15.74	18.91	4.05E-01	7.76	50.63	8.78E-01	-23.72	10.59	2.51E-02	-14.84	33.85	6.61E-01
cg25720825	-13.16	5.65	1.98E-02	-21.13	7.98	8.07E-03	-1.03	11.40	9.28E-01	-22.03	10.51	3.60E-02	-2.32	10.85	8.30E-01
cg19267163	-9.70	4.16	1.98E-02	-5.59	10.40	5.91E-01	-5.38	17.79	7.62E-01	-8.87	5.00	7.62E-02	-25.40	13.63	6.24E-02
cg07042532	-12.53	5.38	1.98E-02	-19.73	15.20	1.94E-01	-5.63	18.60	7.62E-01	-11.84	6.30	6.03E-02	-15.39	21.49	4.74E-01
cg16640008	-16.83	7.23	1.98E-02	-16.07	11.52	1.63E-01	-25.93	21.75	2.33E-01	-13.77	15.22	3.65E-01	-16.79	13.90	2.27E-01

cg00268744	21.13	9.07	1.98E-02	15.70	15.26	3.03E-01	6.97	18.59	7.08E-01	29.50	19.04	1.21E-01	39.77	21.28	6.16E-02
cg20614854	-12.03	5.16	1.98E-02	-15.28	7.90	5.31E-02	-10.75	11.10	3.33E-01	-3.28	16.01	8.38E-01	-11.23	10.29	2.75E-01
cg13136716	11.76	5.05	1.98E-02	5.18	9.47	5.85E-01	5.18	16.50	7.53E-01	14.87	7.09	3.60E-02	19.67	14.88	1.86E-01
cg12819931	10.69	4.59	1.98E-02	1.45	10.54	8.91E-01	-9.24	21.54	6.68E-01	13.79	5.42	1.10E-02	19.41	20.59	3.46E-01
cg18025409	-7.12	3.06	1.99E-02	-6.45	4.15	1.20E-01	-10.45	6.03	8.28E-02	-12.98	16.65	4.36E-01	-2.96	7.51	6.93E-01
cg09703136	-9.08	3.90	1.99E-02	-16.14	9.05	7.43E-02	-10.26	12.86	4.25E-01	-5.81	5.01	2.46E-01	-13.71	11.31	2.25E-01
cg08166588	9.48	4.07	1.99E-02	6.63	5.76	2.50E-01	11.64	6.86	8.96E-02	19.50	7.31	7.66E-03	-2.64	9.19	7.74E-01
cg06620993	9.29	3.99	1.99E-02	13.54	5.76	1.87E-02	10.01	9.51	2.93E-01	-3.65	11.58	7.52E-01	6.49	8.39	4.39E-01
cg26051060	-52.21	22.42	1.99E-02	-50.72	27.36	6.38E-02	-211.26	95.08	2.63E-02	-46.43	66.61	4.86E-01	-26.69	40.17	5.06E-01
cg16336436	-16.88	7.25	1.99E-02	-1.42	16.10	9.30E-01	6.09	32.22	8.50E-01	-23.86	8.93	7.56E-03	-13.47	24.34	5.80E-01
cg07687029	18.85	8.09	1.99E-02	21.16	15.20	1.64E-01	33.51	32.80	3.07E-01	13.83	11.09	2.12E-01	27.95	23.04	2.25E-01
cg07007382	4.47	1.92	1.99E-02	4.15	2.51	9.86E-02	4.43	4.11	2.80E-01	-1.06	9.42	9.10E-01	7.14	4.83	1.39E-01
cg10233454	7.24	3.11	1.99E-02	5.20	3.98	1.92E-01	17.12	7.96	3.16E-02	6.44	9.17	4.83E-01	5.87	8.87	5.08E-01
cg02636234	42.30	18.16	1.99E-02	47.88	24.20	4.79E-02	0.89	100.80	9.93E-01	23.12	42.37	5.85E-01	50.12	38.69	1.95E-01
cg27477419	26.14	11.23	1.99E-02	46.13	13.43	5.94E-04	24.92	26.81	3.53E-01	29.96	16.44	6.84E-02	-1.12	16.14	9.45E-01
cg13641920	11.42	4.90	1.99E-02	21.06	7.71	6.28E-03	7.00	12.28	5.69E-01	4.34	13.71	7.52E-01	3.97	8.84	6.53E-01
cg15945754	-15.07	6.47	1.99E-02	-21.19	10.58	4.52E-02	-16.07	27.63	5.61E-01	-12.24	10.92	2.62E-01	-8.93	13.79	5.17E-01
cg11435872	11.59	4.98	1.99E-02	3.38	7.71	6.61E-01	19.87	9.16	3.01E-02	11.86	15.78	4.52E-01	16.59	11.44	1.47E-01
cg17450307	19.34	8.30	1.99E-02	45.91	27.79	9.86E-02	143.90	111.72	1.98E-01	16.38	8.99	6.83E-02	8.83	36.72	8.10E-01
cg13378781	-43.73	18.78	1.99E-02	-38.13	28.82	1.86E-01	26.76	75.22	7.22E-01	-84.01	39.06	3.15E-02	-34.72	35.36	3.26E-01
cg13441891	9.89	4.25	1.99E-02	13.56	5.53	1.42E-02	18.23	9.41	5.26E-02	5.94	8.52	4.86E-01	-4.68	10.23	6.47E-01
cg03238899	-12.58	5.40	1.99E-02	-11.60	12.03	3.35E-01	-29.33	20.19	1.46E-01	-12.78	6.76	5.86E-02	0.29	18.22	9.87E-01
cg16677191	10.61	4.56	1.99E-02	9.95	5.37	6.39E-02	16.77	13.56	2.16E-01	15.35	20.01	4.43E-01	6.57	13.45	6.25E-01
cg13944838	-2.10	0.90	1.99E-02	-2.78	1.21	2.14E-02	-0.56	2.05	7.84E-01	-6.10	3.48	8.00E-02	-0.21	2.10	9.20E-01
cg21739009	26.12	11.22	1.99E-02	30.28	12.69	1.71E-02	33.11	63.20	6.00E-01	-88.16	77.59	2.56E-01	19.67	27.51	4.75E-01
cg24401310	13.67	5.87	1.99E-02	0.56	18.95	9.76E-01	26.66	46.03	5.63E-01	14.42	6.52	2.69E-02	19.34	21.23	3.62E-01
cg13042250	19.17	8.24	1.99E-02	35.55	20.94	8.95E-02	9.04	36.11	8.02E-01	19.42	9.73	4.59E-02	-9.23	29.72	7.56E-01
cg06198451	-25.12	10.79	1.99E-02	-47.45	55.19	3.90E-01	-168.56	222.81	4.49E-01	-20.82	11.24	6.39E-02	-98.06	55.39	7.67E-02
cg00368356	18.42	7.91	1.99E-02	18.07	18.98	3.41E-01	8.41	18.73	6.54E-01	18.06	10.38	8.18E-02	49.42	30.70	1.07E-01
cg16478012	-58.86	25.28	1.99E-02	-90.89	72.42	2.09E-01	-315.33	304.74	3.01E-01	-46.71	29.39	1.12E-01	-84.22	69.83	2.28E-01
cg26829451	16.92	7.27	1.99E-02	44.08	26.47	9.58E-02	66.87	88.28	4.49E-01	14.22	7.85	7.01E-02	15.76	29.53	5.94E-01
cg08697251	-8.27	3.55	1.99E-02	-12.85	5.39	1.72E-02	-4.01	8.37	6.32E-01	0.10	10.53	9.92E-01	-7.29	6.81	2.85E-01
cg21566771	10.20	4.38	1.99E-02	3.33	6.03	5.81E-01	20.27	9.67	3.60E-02	15.71	13.32	2.38E-01	16.26	11.01	1.40E-01
cg12693436	-15.24	6.55	1.99E-02	-17.22	10.10	8.82E-02	-23.76	14.96	1.12E-01	0.32	16.15	9.84E-01	-15.65	13.83	2.58E-01
cg22369786	-11.33	4.87	1.99E-02	-9.97	6.42	1.21E-01	-10.29	20.51	6.16E-01	-33.16	15.60	3.36E-02	-6.62	9.35	4.79E-01

cg17522907	16.30	7.00	1.99E-02	46.25	19.79	1.94E-02	13.58	86.99	8.76E-01	11.41	7.83	1.45E-01	18.88	26.68	4.79E-01
cg11428189	-24.90	10.70	1.99E-02	-34.54	15.31	2.41E-02	2.90	26.54	9.13E-01	-17.27	31.31	5.81E-01	-27.94	22.19	2.08E-01
cg08239899	-19.45	8.36	1.99E-02	-37.71	14.02	7.16E-03	-7.53	15.79	6.34E-01	-21.91	14.11	1.21E-01	2.30	20.55	9.11E-01
cg15879316	36.24	15.57	1.99E-02	31.72	19.60	1.05E-01	82.67	63.33	1.92E-01	47.65	45.34	2.93E-01	29.41	35.69	4.10E-01
cg25247558	-6.07	2.61	1.99E-02	-6.06	3.51	8.44E-02	-8.25	6.22	1.85E-01	-2.81	8.41	7.38E-01	-5.74	6.24	3.58E-01
cg19169976	13.42	5.77	1.99E-02	9.32	9.89	3.46E-01	7.63	23.11	7.41E-01	17.51	8.32	3.52E-02	11.61	16.86	4.91E-01
cg19701531	15.34	6.59	1.99E-02	18.79	10.82	8.25E-02	14.58	13.37	2.76E-01	0.14	14.25	9.92E-01	27.88	15.89	7.93E-02
cg14997413	12.32	5.29	2.00E-02	20.37	9.92	4.00E-02	0.12	12.65	9.93E-01	15.47	10.14	1.27E-01	8.52	10.22	4.05E-01
cg03642503	-16.25	6.98	2.00E-02	-24.82	11.62	3.27E-02	0.50	13.80	9.71E-01	-15.03	17.30	3.85E-01	-22.61	14.90	1.29E-01
cg01782066	-7.86	3.38	2.00E-02	-11.36	5.28	3.14E-02	1.13	7.43	8.79E-01	-13.37	9.40	1.55E-01	-6.74	6.70	3.15E-01
cg10801328	9.86	4.24	2.00E-02	11.62	8.32	1.63E-01	4.25	10.11	6.74E-01	15.60	7.30	3.25E-02	3.69	8.87	6.78E-01
cg15823954	20.06	8.62	2.00E-02	6.66	19.62	7.34E-01	31.58	34.15	3.55E-01	17.68	10.74	9.98E-02	54.17	27.36	4.77E-02
cg19942213	-14.34	6.16	2.00E-02	-25.03	13.51	6.39E-02	-23.47	30.39	4.40E-01	-10.67	7.70	1.66E-01	-12.08	18.53	5.14E-01
cg15208267	-12.58	5.41	2.00E-02	-26.74	10.10	8.13E-03	-17.74	16.98	2.96E-01	-8.70	6.54	1.83E-01	0.52	13.00	9.68E-01
cg13393433	10.48	4.51	2.00E-02	12.30	6.40	5.44E-02	12.56	11.89	2.91E-01	15.79	14.93	2.90E-01	4.22	8.69	6.27E-01
cg14158558	-22.35	9.61	2.00E-02	-51.56	15.22	7.07E-04	0.64	21.61	9.76E-01	-17.47	7.15	1.46E-02	-15.06	18.29	4.10E-01
cg09936933	16.95	7.29	2.00E-02	17.41	10.00	8.18E-02	17.03	16.38	2.98E-01	25.04	22.84	2.73E-01	10.59	17.68	5.49E-01
cg06285439	-13.29	5.71	2.00E-02	-17.13	11.08	1.22E-01	-0.50	15.27	9.74E-01	-13.65	8.69	1.16E-01	-17.11	14.21	2.29E-01
cg00925229	-39.24	16.87	2.00E-02	-48.64	23.37	3.74E-02	-80.93	53.89	1.33E-01	-0.11	46.94	9.98E-01	-23.65	33.61	4.82E-01
cg26140802	-7.96	3.42	2.00E-02	-11.23	4.80	1.94E-02	-10.09	8.43	2.31E-01	-7.42	10.71	4.88E-01	0.69	7.20	9.24E-01
cg01377119	-37.80	16.25	2.00E-02	-35.43	18.82	5.98E-02	-56.66	53.73	2.92E-01	-32.41	75.42	6.67E-01	-40.29	47.49	3.96E-01
cg06304097	12.11	5.20	2.00E-02	41.87	18.84	2.62E-02	6.31	17.75	7.22E-01	11.73	6.96	9.21E-02	6.51	9.85	5.09E-01
cg20736997	37.82	16.26	2.00E-02	43.31	22.62	5.55E-02	80.76	102.26	4.30E-01	98.37	58.20	9.10E-02	15.07	26.37	5.68E-01
cg22517552	29.87	12.84	2.00E-02	49.48	37.20	1.83E-01	-63.87	133.10	6.31E-01	26.12	14.68	7.51E-02	43.13	39.42	2.74E-01
cg26446674	-13.24	5.69	2.00E-02	-20.22	13.12	1.23E-01	-12.75	30.70	6.78E-01	-10.79	6.82	1.13E-01	-18.25	19.99	3.61E-01
cg23010538	8.92	3.84	2.00E-02	9.64	14.00	4.91E-01	21.53	36.93	5.60E-01	10.45	4.21	1.32E-02	-7.97	13.08	5.42E-01
cg01819405	22.39	9.63	2.00E-02	16.49	20.42	4.19E-01	27.89	20.80	1.80E-01	18.24	14.53	2.09E-01	38.12	27.28	1.62E-01
cg01054402	9.75	4.19	2.00E-02	9.97	5.23	5.68E-02	17.21	10.56	1.03E-01	-12.27	17.30	4.78E-01	9.61	11.16	3.89E-01
cg12210293	33.52	14.41	2.00E-02	29.68	20.18	1.41E-01	39.89	47.28	3.99E-01	57.41	59.63	3.36E-01	33.45	24.77	1.77E-01
cg11549953	60.10	25.84	2.00E-02	21.38	62.11	7.31E-01	-74.68	270.19	7.82E-01	88.07	31.15	4.70E-03	-26.87	71.66	7.08E-01
cg01919011	-31.86	13.70	2.00E-02	-46.73	22.71	3.96E-02	79.58	82.02	3.32E-01	-37.13	22.14	9.35E-02	-12.72	28.84	6.59E-01
cg03667862	18.32	7.88	2.00E-02	13.90	19.83	4.83E-01	97.62	103.76	3.47E-01	17.56	8.91	4.89E-02	33.31	33.35	3.18E-01
cg16165575	-14.21	6.11	2.00E-02	-17.04	8.04	3.40E-02	-10.00	13.37	4.54E-01	-34.76	34.50	3.14E-01	-6.50	14.33	6.50E-01
cg04540882	13.78	5.92	2.00E-02	14.95	8.03	6.28E-02	12.62	12.71	3.21E-01	13.98	19.59	4.75E-01	11.05	15.45	4.74E-01
cg00435834	-8.56	3.68	2.00E-02	-9.71	8.08	2.30E-01	-8.92	12.45	4.73E-01	-9.46	4.75	4.66E-02	-0.84	11.41	9.41E-01

cg13169863	-17.17	7.38	2.00E-02	-32.61	12.00	6.57E-03	-37.85	33.47	2.58E-01	-8.01	5.78	1.66E-01	-18.74	19.85	3.45E-01
cg12659494	30.55	13.14	2.00E-02	41.60	19.00	2.85E-02	-25.45	38.12	5.04E-01	34.12	34.44	3.22E-01	33.84	25.88	1.91E-01
cg19825277	-9.87	4.24	2.00E-02	-22.30	11.69	5.65E-02	-17.91	21.74	4.10E-01	-7.36	4.91	1.33E-01	-8.99	14.75	5.42E-01
cg13563869	-12.97	5.58	2.01E-02	-14.26	12.12	2.39E-01	4.42	14.63	7.62E-01	-12.66	7.84	1.06E-01	-30.46	15.03	4.27E-02
cg03142837	19.61	8.43	2.01E-02	2.66	18.53	8.86E-01	32.01	43.17	4.58E-01	20.42	10.52	5.24E-02	41.91	25.10	9.50E-02
cg03885664	-7.10	3.05	2.01E-02	-8.22	4.25	5.33E-02	-2.59	7.33	7.24E-01	-9.63	8.59	2.63E-01	-6.48	7.10	3.61E-01
cg17871621	6.51	2.80	2.01E-02	8.86	4.24	3.65E-02	0.41	6.09	9.46E-01	14.73	9.13	1.06E-01	4.53	5.52	4.11E-01
cg06174407	9.68	4.16	2.01E-02	8.77	5.69	1.23E-01	18.01	10.23	7.84E-02	8.42	11.32	4.57E-01	5.29	10.29	6.07E-01
cg20960405	7.24	3.11	2.01E-02	2.30	6.11	7.07E-01	4.42	6.01	4.62E-01	11.99	5.59	3.21E-02	10.73	7.73	1.65E-01
cg18563680	21.00	9.03	2.01E-02	30.76	25.68	2.31E-01	1.83	89.48	9.84E-01	18.98	10.10	6.02E-02	29.91	34.94	3.92E-01
cg06493963	-9.72	4.18	2.01E-02	-18.93	9.32	4.22E-02	-6.85	19.33	7.23E-01	-7.78	5.25	1.39E-01	-5.57	12.15	6.46E-01
cg11033835	10.68	4.59	2.01E-02	18.40	18.74	3.26E-01	26.26	37.19	4.80E-01	10.84	5.08	3.27E-02	2.85	14.05	8.39E-01
cg09290175	15.35	6.60	2.01E-02	37.24	19.85	6.06E-02	20.19	33.56	5.47E-01	12.03	7.36	1.02E-01	16.75	30.77	5.86E-01
cg18492799	16.11	6.93	2.01E-02	1.10	16.79	9.48E-01	-9.88	28.90	7.32E-01	20.97	8.20	1.05E-02	26.17	28.92	3.65E-01
cg09780964	9.12	3.92	2.01E-02	9.21	4.97	6.39E-02	16.23	12.27	1.86E-01	4.19	11.91	7.25E-01	7.64	9.63	4.28E-01
cg02471183	-13.12	5.64	2.01E-02	-12.37	7.87	1.16E-01	-22.94	12.83	7.37E-02	-7.63	17.04	6.54E-01	-8.12	13.18	5.38E-01
cg22029709	61.60	26.50	2.01E-02	41.89	33.24	2.08E-01	32.02	139.42	8.18E-01	43.23	81.14	5.94E-01	131.71	56.27	1.92E-02
cg20108328	-56.75	24.41	2.01E-02	-51.68	32.23	1.09E-01	11.92	121.19	9.22E-01	-72.78	59.53	2.21E-01	-70.53	52.36	1.78E-01
cg14445814	13.33	5.73	2.01E-02	17.16	10.45	1.01E-01	-9.56	31.04	7.58E-01	10.29	8.57	2.30E-01	17.85	12.29	1.46E-01
cg25634175	-9.75	4.19	2.01E-02	-11.71	6.93	9.13E-02	-2.86	9.48	7.63E-01	-6.35	8.01	4.28E-01	-19.26	10.34	6.25E-02
cg01807313	-17.09	7.35	2.01E-02	-31.36	14.56	3.13E-02	-8.31	20.14	6.80E-01	-9.59	10.30	3.52E-01	-30.38	23.02	1.87E-01
cg02349373	-6.53	2.81	2.01E-02	-5.20	3.98	1.92E-01	-7.35	6.25	2.39E-01	6.41	11.11	5.64E-01	-12.11	5.77	3.60E-02
cg22571654	8.07	3.47	2.01E-02	5.28	5.03	2.93E-01	18.17	7.03	9.74E-03	7.68	10.30	4.56E-01	1.48	8.54	8.62E-01
cg03517998	-8.29	3.57	2.01E-02	-11.11	6.69	9.69E-02	2.77	10.49	7.91E-01	-8.70	5.08	8.67E-02	-10.90	10.92	3.18E-01
cg20611294	-38.05	16.37	2.01E-02	30.90	63.23	6.25E-01	-188.85	241.55	4.34E-01	-41.71	17.56	1.75E-02	-50.83	67.39	4.51E-01
cg16491046	-15.99	6.88	2.01E-02	-28.21	19.52	1.48E-01	38.83	96.43	6.87E-01	-14.11	7.71	6.74E-02	-19.49	25.17	4.39E-01
cg06706454	46.93	20.19	2.01E-02	84.39	53.15	1.12E-01	-20.00	210.07	9.24E-01	49.63	23.11	3.17E-02	-35.96	70.23	6.09E-01
cg01307115	14.94	6.43	2.01E-02	18.18	11.03	9.93E-02	9.63	13.24	4.67E-01	8.27	11.81	4.84E-01	31.54	17.95	7.89E-02
cg11147278	8.11	3.49	2.01E-02	6.97	4.69	1.37E-01	14.85	7.91	6.04E-02	12.92	13.88	3.52E-01	2.86	8.09	7.24E-01
cg15113009	-12.98	5.59	2.01E-02	-16.48	11.55	1.53E-01	-3.11	13.83	8.22E-01	-12.58	8.40	1.34E-01	-18.99	13.94	1.73E-01
cg02704217	11.91	5.13	2.01E-02	15.83	5.97	7.95E-03	3.45	9.89	7.27E-01	-11.20	15.56	4.72E-01	19.32	6.90	5.10E-03
cg13277493	7.43	3.20	2.01E-02	4.27	4.84	3.78E-01	3.95	7.16	5.81E-01	10.46	9.64	2.78E-01	14.28	6.34	2.44E-02
cg07015591	9.14	3.93	2.01E-02	2.65	8.22	7.47E-01	27.84	12.45	2.54E-02	9.23	5.29	8.11E-02	5.53	11.44	6.29E-01
cg17930365	-9.95	4.28	2.01E-02	-2.17	12.71	8.64E-01	-13.73	24.56	5.76E-01	-10.76	4.87	2.71E-02	-11.65	14.92	4.35E-01
cg20306180	-22.11	9.52	2.02E-02	-45.70	17.13	7.64E-03	-17.70	31.87	5.79E-01	8.79	68.92	8.99E-01	-11.31	12.46	3.64E-01

cg07385490	17.77	7.65	2.02E-02	3.15	12.48	8.01E-01	21.76	32.72	5.06E-01	17.87	7.83	2.25E-02	48.41	20.21	1.66E-02
cg01947224	26.17	11.26	2.02E-02	15.90	14.08	2.59E-01	72.07	45.89	1.16E-01	53.64	34.12	1.16E-01	30.46	25.82	2.38E-01
cg04415798	17.31	7.45	2.02E-02	19.23	18.21	2.91E-01	57.81	30.62	5.91E-02	19.27	9.24	3.70E-02	-1.19	16.07	9.41E-01
cg04036182	7.21	3.10	2.02E-02	6.87	4.50	1.27E-01	10.63	7.70	1.67E-01	10.50	7.51	1.62E-01	2.23	7.09	7.53E-01
cg08623644	12.15	5.23	2.02E-02	18.54	8.47	2.86E-02	9.33	11.11	4.01E-01	8.15	12.10	5.00E-01	7.09	11.42	5.35E-01
cg19925025	20.20	8.70	2.02E-02	-1.37	17.55	9.38E-01	27.14	18.35	1.39E-01	23.13	13.90	9.61E-02	39.00	23.40	9.56E-02
cg27224823	-14.17	6.10	2.02E-02	-12.22	9.47	1.97E-01	-13.86	11.42	2.25E-01	-5.11	19.75	7.96E-01	-22.76	13.48	9.12E-02
cg10930308	3.51	1.51	2.02E-02	2.51	2.24	2.61E-01	5.33	2.99	7.46E-02	2.46	4.41	5.77E-01	4.17	3.66	2.54E-01
cg01079599	-13.46	5.80	2.02E-02	-2.21	14.42	8.78E-01	-13.78	19.22	4.73E-01	-12.97	7.15	6.96E-02	-37.11	19.37	5.53E-02
cg03851398	22.28	9.59	2.02E-02	-11.77	25.52	6.45E-01	-16.61	112.84	8.83E-01	29.60	10.73	5.79E-03	7.71	41.94	8.54E-01
cg09768821	-15.84	6.82	2.02E-02	-17.73	15.57	2.55E-01	40.30	35.73	2.59E-01	-18.42	8.37	2.78E-02	-15.60	20.77	4.53E-01
cg22592490	25.18	10.84	2.02E-02	8.63	26.31	7.43E-01	4.79	54.07	9.29E-01	32.21	13.19	1.46E-02	15.36	32.06	6.32E-01
cg05602799	-14.80	6.37	2.02E-02	-32.92	16.78	4.98E-02	-43.40	32.57	1.83E-01	-10.56	7.40	1.54E-01	-7.42	23.00	7.47E-01
cg25276694	11.84	5.10	2.02E-02	10.44	12.75	4.13E-01	10.68	14.25	4.54E-01	14.39	7.28	4.82E-02	7.90	10.83	4.66E-01
cg09509553	-18.45	7.94	2.02E-02	-8.47	16.60	6.10E-01	-28.75	41.35	4.87E-01	-22.29	9.93	2.47E-02	-12.56	25.93	6.28E-01
cg15412772	5.99	2.58	2.02E-02	5.27	3.13	9.17E-02	13.05	6.62	4.86E-02	-3.11	9.84	7.51E-01	6.41	8.19	4.34E-01
cg03294491	11.34	4.88	2.02E-02	16.64	6.26	7.91E-03	6.81	15.11	6.52E-01	-0.91	22.16	9.67E-01	2.37	9.97	8.12E-01
cg20143982	26.46	11.39	2.02E-02	21.57	15.50	1.64E-01	50.88	34.37	1.39E-01	59.45	36.70	1.05E-01	13.75	22.65	5.44E-01
cg05928448	14.10	6.07	2.02E-02	11.66	7.18	1.04E-01	44.55	23.89	6.22E-02	21.13	24.61	3.91E-01	10.04	15.22	5.10E-01
cg19679543	-11.00	4.73	2.02E-02	-10.09	10.06	3.16E-01	-25.90	23.48	2.70E-01	-9.66	6.08	1.12E-01	-14.06	13.05	2.81E-01
cg18420965	13.01	5.60	2.02E-02	22.03	9.75	2.39E-02	10.72	16.17	5.07E-01	19.58	12.04	1.04E-01	0.63	9.71	9.48E-01
cg15111475	33.41	14.39	2.02E-02	47.26	32.95	1.51E-01	-103.92	112.66	3.56E-01	37.50	17.98	3.70E-02	13.60	36.87	7.12E-01
cg23413809	25.25	10.87	2.02E-02	24.86	17.98	1.67E-01	45.56	40.86	2.65E-01	18.42	17.02	2.79E-01	34.87	27.61	2.07E-01
cg25711078	-38.24	16.47	2.02E-02	-23.48	20.05	2.42E-01	-80.52	75.58	2.87E-01	-90.67	62.13	1.44E-01	-58.76	36.13	1.04E-01
cg12745325	12.15	5.23	2.02E-02	12.39	7.85	1.14E-01	16.41	11.35	1.48E-01	12.70	12.61	3.14E-01	5.66	12.68	6.55E-01
cg13963210	-9.13	3.93	2.02E-02	-12.75	8.31	1.25E-01	-11.76	17.66	5.05E-01	-8.59	5.00	8.57E-02	-3.49	12.00	7.71E-01
cg03931660	33.46	14.41	2.02E-02	22.99	18.76	2.20E-01	42.80	59.22	4.70E-01	15.40	38.38	6.88E-01	72.47	31.48	2.13E-02
cg17749520	24.12	10.39	2.02E-02	32.57	20.19	1.07E-01	-0.17	23.24	9.94E-01	33.17	16.71	4.71E-02	18.18	26.92	5.00E-01
cg08658787	12.94	5.57	2.02E-02	21.62	6.30	5.97E-04	-1.95	9.98	8.45E-01	14.76	15.76	3.49E-01	11.24	9.86	2.54E-01
cg13500819	-9.08	3.91	2.02E-02	-9.04	5.12	7.76E-02	-16.64	10.41	1.10E-01	5.15	11.58	6.57E-01	-12.64	9.70	1.93E-01
cg08911237	21.25	9.15	2.03E-02	37.93	19.99	5.78E-02	12.57	33.78	7.10E-01	22.14	11.67	5.78E-02	-12.19	28.69	6.71E-01
cg19305111	-37.11	15.98	2.03E-02	-18.34	62.01	7.67E-01	10.44	159.13	9.48E-01	-38.72	17.35	2.56E-02	-41.99	58.64	4.74E-01
cg12252039	-19.02	8.19	2.03E-02	-53.28	23.43	2.30E-02	-9.33	33.60	7.81E-01	-12.76	8.93	1.53E-01	-38.16	32.64	2.42E-01
cg01582066	-7.34	3.16	2.03E-02	-5.59	4.18	1.82E-01	-14.21	7.80	6.83E-02	-3.68	8.70	6.72E-01	-10.06	8.71	2.48E-01
cg13052625	-12.75	5.49	2.03E-02	-10.28	8.62	2.33E-01	-8.24	13.13	5.31E-01	-7.75	13.32	5.61E-01	-23.37	11.01	3.37E-02

cg23453466	-12.42	5.35	2.03E-02	-10.21	15.31	5.05E-01	-31.19	23.20	1.79E-01	-13.22	6.25	3.45E-02	1.74	17.53	9.21E-01
cg19946742	-14.72	6.34	2.03E-02	-26.26	10.74	1.44E-02	-0.23	13.00	9.86E-01	-17.18	13.21	1.93E-01	-8.47	14.85	5.68E-01
cg03104569	-8.27	3.56	2.03E-02	-5.29	4.82	2.73E-01	-4.91	8.29	5.54E-01	-24.45	9.60	1.09E-02	-7.42	6.35	2.43E-01
cg04389426	42.31	18.23	2.03E-02	52.92	25.30	3.65E-02	55.67	126.05	6.59E-01	44.96	57.28	4.32E-01	25.44	30.42	4.03E-01
cg16727774	-13.86	5.97	2.03E-02	-12.58	8.72	1.49E-01	1.23	17.11	9.43E-01	-32.11	13.10	1.42E-02	-7.14	13.30	5.91E-01
cg05543593	-12.33	5.31	2.03E-02	-14.04	10.13	1.66E-01	-12.60	13.12	3.37E-01	-12.41	8.35	1.38E-01	-8.85	13.45	5.10E-01
cg16258337	-15.64	6.74	2.03E-02	-17.26	8.85	5.11E-02	-32.87	21.43	1.25E-01	-14.74	40.95	7.19E-01	-6.74	12.41	5.87E-01
cg05459190	8.57	3.69	2.03E-02	6.09	5.06	2.29E-01	10.07	8.68	2.46E-01	6.21	10.37	5.49E-01	16.97	9.22	6.58E-02
cg27185128	27.22	11.73	2.03E-02	61.75	33.68	6.67E-02	61.91	137.32	6.52E-01	22.41	13.01	8.50E-02	18.21	48.30	7.06E-01
cg00920395	-16.78	7.23	2.03E-02	-42.11	19.68	3.24E-02	-9.64	27.26	7.24E-01	-13.38	8.76	1.27E-01	-11.50	21.42	5.91E-01
cg27062243	-11.98	5.16	2.03E-02	-18.93	8.84	3.22E-02	-1.13	11.96	9.24E-01	-10.98	9.92	2.69E-01	-11.59	11.50	3.13E-01
cg12668403	-12.70	5.47	2.03E-02	-21.62	13.88	1.19E-01	-23.31	19.35	2.28E-01	-12.27	6.71	6.75E-02	6.82	17.31	6.94E-01
cg01156629	-47.68	20.54	2.03E-02	-40.75	28.25	1.49E-01	-60.60	59.54	3.09E-01	-16.02	53.02	7.63E-01	-81.73	45.71	7.38E-02
cg21756587	17.44	7.51	2.03E-02	4.32	15.73	7.84E-01	25.29	19.95	2.05E-01	15.80	10.64	1.38E-01	38.01	20.75	6.69E-02
cg23791600	-10.60	4.57	2.03E-02	-14.89	8.34	7.44E-02	-20.47	11.25	6.88E-02	-1.71	6.38	7.89E-01	-17.33	11.01	1.16E-01
cg23878404	-10.09	4.35	2.03E-02	-8.38	7.57	2.68E-01	1.93	11.45	8.66E-01	-9.10	7.11	2.01E-01	-23.83	9.88	1.59E-02
cg22282764	-14.37	6.19	2.03E-02	-15.33	13.74	2.65E-01	-6.95	31.86	8.27E-01	-11.96	7.57	1.14E-01	-33.48	20.75	1.07E-01
cg07664990	17.71	7.63	2.03E-02	25.12	10.56	1.74E-02	19.55	24.81	4.31E-01	10.36	30.00	7.30E-01	6.50	13.54	6.31E-01
cg25786780	15.74	6.78	2.03E-02	13.07	10.57	2.17E-01	6.08	14.03	6.64E-01	20.04	15.90	2.08E-01	30.65	16.32	6.04E-02
cg10954944	10.52	4.54	2.03E-02	17.57	6.77	9.45E-03	6.84	8.19	4.04E-01	3.67	12.56	7.70E-01	0.55	13.41	9.68E-01
cg14983236	7.42	3.20	2.03E-02	10.68	5.48	5.12E-02	5.92	9.01	5.11E-01	3.54	4.77	4.58E-01	17.32	11.09	1.19E-01
cg20011940	-14.81	6.38	2.03E-02	-10.37	15.06	4.91E-01	10.51	18.87	5.78E-01	-18.94	8.15	2.01E-02	-27.31	20.99	1.93E-01
cg04036272	17.23	7.43	2.03E-02	28.67	25.57	2.62E-01	-9.27	87.92	9.16E-01	16.02	8.03	4.61E-02	22.09	32.15	4.92E-01
cg17191715	-13.32	5.74	2.03E-02	4.37	17.82	8.06E-01	-7.71	27.50	7.79E-01	-15.43	6.39	1.58E-02	-21.44	26.56	4.19E-01
cg10991752	-9.64	4.16	2.03E-02	-17.23	11.22	1.25E-01	-11.42	15.32	4.56E-01	-6.36	5.01	2.04E-01	-20.18	12.98	1.20E-01
cg16430510	24.27	10.46	2.03E-02	-8.59	43.58	8.44E-01	54.87	158.18	7.29E-01	25.15	11.04	2.27E-02	47.98	51.81	3.54E-01
cg21828432	-9.57	4.12	2.03E-02	-16.01	11.96	1.81E-01	-17.76	21.22	4.03E-01	-7.01	4.80	1.44E-01	-17.39	12.79	1.74E-01
cg26734950	22.73	9.80	2.03E-02	36.89	22.69	1.04E-01	8.29	25.16	7.42E-01	13.61	10.52	1.96E-01	59.96	26.20	2.21E-02
cg21622977	-10.15	4.38	2.03E-02	-3.31	8.64	7.01E-01	-8.62	12.20	4.80E-01	-15.69	6.24	1.19E-02	-3.82	12.55	7.61E-01
cg00146645	16.02	6.90	2.03E-02	15.62	8.96	8.14E-02	29.25	21.84	1.80E-01	2.89	36.03	9.36E-01	13.77	13.28	3.00E-01
cg19202014	14.04	6.05	2.03E-02	9.89	8.41	2.39E-01	12.88	15.24	3.98E-01	31.47	15.48	4.20E-02	12.09	14.63	4.08E-01
cg04041447	21.50	9.27	2.04E-02	27.66	12.32	2.48E-02	28.59	35.36	4.19E-01	58.04	30.56	5.76E-02	4.13	13.92	7.67E-01
cg07677446	-11.67	5.03	2.04E-02	-18.20	7.64	1.73E-02	-2.16	11.24	8.48E-01	-15.49	13.75	2.60E-01	-5.50	10.43	5.98E-01
cg20974659	10.43	4.50	2.04E-02	3.87	6.66	5.61E-01	12.33	8.61	1.52E-01	16.57	15.19	2.75E-01	20.97	10.48	4.55E-02
cg00969823	-19.74	8.51	2.04E-02	-14.85	12.06	2.18E-01	-21.07	19.57	2.81E-01	-11.32	22.87	6.21E-01	-38.95	20.38	5.60E-02

cg00970979	20.75	8.94	2.04E-02	11.44	13.46	3.95E-01	33.20	18.03	6.56E-02	11.23	21.66	6.04E-01	39.53	23.73	9.58E-02
cg23044488	-45.36	19.55	2.04E-02	-12.53	68.26	8.54E-01	-166.61	252.52	5.09E-01	-47.31	21.38	2.69E-02	-49.79	71.37	4.85E-01
cg16951533	-15.36	6.62	2.04E-02	-6.38	16.04	6.91E-01	12.65	32.69	6.99E-01	-20.76	7.84	8.08E-03	0.44	24.32	9.86E-01
cg27201871	-28.02	12.08	2.04E-02	-33.46	18.96	7.76E-02	10.08	89.52	9.10E-01	-33.22	18.51	7.26E-02	-3.12	31.19	9.20E-01
cg24469742	-12.02	5.18	2.04E-02	-20.96	8.76	1.67E-02	-9.85	14.76	5.05E-01	-9.04	8.86	3.08E-01	-2.02	12.09	8.67E-01
cg26259627	39.17	16.89	2.04E-02	6.79	48.53	8.89E-01	-48.75	177.26	7.83E-01	44.29	18.82	1.86E-02	48.31	66.50	4.68E-01
cg04617948	16.37	7.06	2.04E-02	31.24	9.91	1.61E-03	4.56	17.33	7.92E-01	12.88	13.67	3.46E-01	4.60	12.62	7.16E-01
cg24422797	13.54	5.84	2.04E-02	4.06	8.91	6.49E-01	18.18	10.30	7.76E-02	22.33	19.44	2.51E-01	24.84	14.66	9.03E-02
cg14096767	-10.17	4.38	2.04E-02	-16.57	11.07	1.34E-01	7.85	17.74	6.58E-01	-9.58	5.36	7.40E-02	-14.49	13.03	2.66E-01
cg10269476	17.93	7.73	2.04E-02	45.72	18.45	1.32E-02	34.57	41.83	4.09E-01	12.11	10.51	2.50E-01	9.58	14.21	5.00E-01
cg02531439	-9.98	4.30	2.04E-02	-11.57	8.04	1.50E-01	-7.68	15.49	6.20E-01	-5.01	6.06	4.09E-01	-26.87	11.85	2.34E-02
cg02622866	27.53	11.88	2.04E-02	47.85	46.90	3.08E-01	-104.48	187.79	5.78E-01	26.44	12.71	3.75E-02	30.66	49.22	5.33E-01
cg22675447	5.93	2.56	2.04E-02	5.22	3.61	1.48E-01	7.70	5.82	1.86E-01	6.34	5.77	2.72E-01	5.35	7.81	4.93E-01
cg23075260	18.93	8.16	2.04E-02	4.04	15.64	7.96E-01	27.92	15.28	6.78E-02	9.16	15.98	5.66E-01	41.25	19.19	3.16E-02
cg21578596	6.32	2.73	2.04E-02	5.73	3.42	9.37E-02	13.64	7.28	6.10E-02	4.06	8.62	6.38E-01	2.89	7.72	7.08E-01
cg26303165	8.03	3.46	2.04E-02	9.03	6.48	1.64E-01	16.66	14.00	2.34E-01	11.02	4.27	9.78E-03	-3.87	7.57	6.09E-01
cg25114183	-11.96	5.16	2.04E-02	-8.48	11.93	4.77E-01	-5.47	13.88	6.94E-01	-14.78	6.66	2.65E-02	-10.03	18.69	5.92E-01
cg25020820	14.96	6.45	2.04E-02	15.51	10.10	1.24E-01	23.45	11.97	5.00E-02	8.75	17.13	6.09E-01	3.53	16.20	8.27E-01
cg08185241	7.89	3.41	2.04E-02	11.22	6.32	7.57E-02	8.77	7.84	2.63E-01	5.72	7.34	4.35E-01	5.72	6.16	3.53E-01
cg17280134	-10.84	4.68	2.05E-02	-5.94	7.68	4.39E-01	-21.65	10.51	3.95E-02	-9.68	9.77	3.22E-01	-10.57	10.42	3.10E-01
cg04934652	20.59	8.88	2.05E-02	29.38	15.84	6.37E-02	9.31	18.61	6.17E-01	13.45	16.92	4.27E-01	30.37	20.83	1.45E-01
cg07673376	18.40	7.94	2.05E-02	5.69	18.76	7.62E-01	-0.01	19.82	1.00E+00	25.78	10.70	1.60E-02	28.92	23.88	2.26E-01
cg11161837	8.31	3.59	2.05E-02	5.08	5.12	3.21E-01	4.95	8.51	5.60E-01	18.55	11.61	1.10E-01	13.39	7.37	6.92E-02
cg20312205	17.66	7.62	2.05E-02	38.40	18.73	4.03E-02	26.46	25.70	3.03E-01	17.26	10.30	9.38E-02	-2.33	17.06	8.91E-01
cg10605137	-16.53	7.13	2.05E-02	-20.69	10.28	4.42E-02	-11.52	15.38	4.54E-01	-4.87	21.58	8.22E-01	-18.31	16.14	2.56E-01
cg13074682	-8.52	3.67	2.05E-02	-9.75	8.49	2.51E-01	8.28	15.73	5.99E-01	-9.06	4.59	4.84E-02	-11.44	10.76	2.88E-01
cg14709449	-13.89	5.99	2.05E-02	-16.13	10.95	1.41E-01	7.52	16.71	6.53E-01	-22.04	9.10	1.54E-02	-3.35	16.15	8.36E-01
cg03966785	18.76	8.09	2.05E-02	35.35	19.91	7.58E-02	48.09	39.54	2.24E-01	13.22	10.30	1.99E-01	15.62	19.34	4.19E-01
cg04679323	18.88	8.14	2.05E-02	29.91	13.49	2.66E-02	33.18	18.67	7.56E-02	5.40	14.52	7.10E-01	-0.25	22.51	9.91E-01
cg24945231	19.11	8.24	2.05E-02	45.90	14.70	1.80E-03	2.02	17.95	9.11E-01	13.28	11.71	2.57E-01	14.94	10.62	1.60E-01
ch.2.4251330R	-45.80	19.76	2.05E-02	-50.02	25.83	5.28E-02	-17.43	101.61	8.64E-01	-90.91	84.04	2.79E-01	-33.70	34.86	3.34E-01
cg21611093	-10.21	4.41	2.05E-02	-18.01	6.84	8.50E-03	-4.19	8.96	6.40E-01	-7.89	12.10	5.14E-01	-3.25	9.59	7.34E-01
cg21011621	13.19	5.69	2.05E-02	18.25	8.15	2.51E-02	14.19	12.55	2.58E-01	13.65	15.86	3.90E-01	-2.18	13.49	8.72E-01
cg17151621	-16.80	7.25	2.05E-02	-23.02	11.45	4.44E-02	5.83	31.56	8.54E-01	-24.16	19.44	2.14E-01	-11.10	11.36	3.29E-01
cg10745682	28.50	12.30	2.05E-02	47.82	17.26	5.59E-03	-7.66	34.35	8.23E-01	34.66	33.89	3.06E-01	9.08	22.95	6.92E-01

cg18335535	18.00	7.77	2.05E-02	32.19	15.92	4.32E-02	42.35	28.45	1.37E-01	8.36	10.00	4.03E-01	25.35	26.76	3.43E-01
cg22012476	-15.65	6.75	2.05E-02	-21.93	10.53	3.72E-02	-8.68	15.21	5.68E-01	-2.81	15.21	8.53E-01	-22.47	15.35	1.43E-01
cg05642923	6.66	2.87	2.05E-02	9.57	4.02	1.73E-02	9.61	6.68	1.51E-01	-1.53	6.77	8.21E-01	2.18	8.16	7.89E-01
cg01159097	37.02	15.98	2.05E-02	46.48	18.43	1.17E-02	50.72	21.89	2.05E-02	5.06	9.69	6.01E-01	67.36	28.00	1.61E-02
cg13515774	-35.64	15.38	2.05E-02	-28.93	18.53	1.18E-01	47.97	117.72	6.84E-01	-43.17	37.72	2.52E-01	-73.11	43.00	8.91E-02
cg25388738	-14.60	6.30	2.05E-02	-8.39	12.36	4.97E-01	3.52	18.47	8.49E-01	-15.64	7.62	4.00E-02	-41.09	18.57	2.69E-02
cg05970721	16.71	7.21	2.05E-02	22.84	9.75	1.92E-02	25.99	18.27	1.55E-01	7.67	25.91	7.67E-01	-1.94	15.40	9.00E-01
cg14341551	10.15	4.38	2.05E-02	16.12	10.73	1.33E-01	10.60	12.50	3.96E-01	7.86	5.41	1.46E-01	18.38	18.68	3.25E-01
cg25579085	10.74	4.64	2.05E-02	4.34	11.32	7.02E-01	-10.61	19.39	5.84E-01	10.16	8.86	2.51E-01	15.65	6.55	1.70E-02
cg11826295	-12.13	5.24	2.05E-02	-2.73	14.77	8.54E-01	-31.42	24.23	1.95E-01	-12.20	5.93	3.96E-02	-16.91	24.10	4.83E-01
cg10768996	22.11	9.54	2.05E-02	7.24	17.19	6.73E-01	21.50	18.50	2.45E-01	42.49	19.41	2.86E-02	21.10	22.27	3.43E-01
cg19011001	7.81	3.37	2.05E-02	5.63	4.24	1.85E-01	14.57	8.33	8.02E-02	15.15	10.98	1.68E-01	3.99	10.08	6.92E-01
cg00988548	-13.65	5.89	2.06E-02	-24.95	11.58	3.11E-02	-25.06	18.07	1.65E-01	-4.89	6.23	4.33E-01	-22.44	15.22	1.40E-01
cg05774787	27.94	12.06	2.06E-02	6.47	32.34	8.41E-01	6.27	160.09	9.69E-01	30.80	13.52	2.27E-02	41.92	49.69	3.99E-01
cg02002217	-3.57	1.54	2.06E-02	-3.60	2.65	1.74E-01	-2.29	3.73	5.40E-01	-4.05	2.56	1.14E-01	-3.82	4.29	3.73E-01
cg00002591	22.31	9.64	2.06E-02	6.64	24.89	7.90E-01	17.98	30.28	5.53E-01	21.46	12.03	7.46E-02	53.25	29.34	6.95E-02
cg11975397	3.81	1.64	2.06E-02	3.77	2.36	1.10E-01	3.56	3.56	3.17E-01	10.19	4.70	3.03E-02	-0.18	3.91	9.63E-01
cg25204186	19.48	8.41	2.06E-02	12.23	15.86	4.41E-01	14.10	18.86	4.55E-01	19.06	13.43	1.56E-01	45.00	23.51	5.56E-02
cg22536211	-16.86	7.28	2.06E-02	-2.55	16.15	8.74E-01	1.02	27.38	9.70E-01	-18.74	9.28	4.35E-02	-44.12	21.89	4.39E-02
cg10592454	-12.21	5.27	2.06E-02	-3.60	5.87	5.40E-01	-17.62	9.23	5.63E-02	-31.00	13.71	2.37E-02	-11.84	9.48	2.12E-01
cg03898832	17.56	7.58	2.06E-02	34.14	19.45	7.93E-02	32.57	23.02	1.57E-01	10.19	9.31	2.74E-01	27.28	27.48	3.21E-01
cg05045130	35.62	15.38	2.06E-02	45.28	21.21	3.28E-02	9.37	37.78	8.04E-01	76.27	32.64	1.95E-02	3.75	28.54	8.95E-01
cg26386826	-14.11	6.09	2.06E-02	-20.92	9.55	2.85E-02	-5.22	12.81	6.84E-01	7.46	17.22	6.65E-01	-22.11	12.39	7.44E-02
cg17200690	12.38	5.35	2.06E-02	14.83	7.53	4.89E-02	18.12	11.45	1.14E-01	-2.49	16.98	8.83E-01	6.73	12.69	5.96E-01
cg08378932	-8.71	3.76	2.06E-02	-22.47	8.75	1.02E-02	-9.75	13.36	4.66E-01	-5.56	4.25	1.91E-01	-4.37	10.98	6.90E-01
cg05824174	-47.94	20.70	2.06E-02	-48.93	27.21	7.21E-02	28.73	120.40	8.11E-01	-41.06	49.96	4.11E-01	-61.00	44.17	1.67E-01
cg02786368	15.32	6.62	2.06E-02	11.52	9.34	2.17E-01	25.93	14.46	7.30E-02	11.12	22.01	6.13E-01	15.67	14.88	2.92E-01
cg06452129	-13.35	5.77	2.06E-02	-28.13	14.89	5.88E-02	-9.90	22.28	6.57E-01	-12.40	6.87	7.10E-02	3.47	20.60	8.66E-01
cg13282294	15.84	6.84	2.06E-02	17.94	20.32	3.77E-01	56.35	32.78	8.56E-02	16.03	7.78	3.94E-02	-14.91	25.89	5.65E-01
cg01273336	-11.13	4.81	2.06E-02	-19.59	11.07	7.67E-02	-15.03	17.88	4.00E-01	-7.64	6.06	2.08E-01	-14.07	14.53	3.33E-01
cg10520924	19.76	8.54	2.06E-02	28.87	22.01	1.90E-01	41.14	27.92	1.41E-01	10.82	10.36	2.97E-01	54.65	30.67	7.47E-02
cg02446647	13.68	5.91	2.06E-02	24.11	10.57	2.26E-02	11.05	14.54	4.47E-01	13.28	11.02	2.28E-01	2.16	12.19	8.59E-01
cg13419087	15.83	6.84	2.06E-02	17.24	9.35	6.51E-02	21.74	18.97	2.52E-01	16.39	13.24	2.16E-01	-8.79	26.26	7.38E-01
cg23181170	-10.03	4.33	2.06E-02	2.61	12.29	8.32E-01	-6.68	23.18	7.73E-01	-13.09	5.03	9.18E-03	-3.99	13.90	7.74E-01
cg27171280	35.30	15.25	2.06E-02	4.79	35.77	8.93E-01	-12.01	125.29	9.24E-01	40.78	17.97	2.33E-02	62.67	52.61	2.34E-01

cg11756777	-12.55	5.42	2.06E-02	-12.41	8.93	1.64E-01	0.04	14.00	9.98E-01	-8.78	6.06	1.47E-01	-33.20	12.33	7.07E-03
cg01435464	24.26	10.48	2.06E-02	23.28	18.35	2.05E-01	26.81	22.36	2.31E-01	25.25	18.23	1.66E-01	19.65	29.78	5.09E-01
cg12074329	29.89	12.91	2.06E-02	36.18	14.14	1.05E-02	-40.79	102.20	6.90E-01	-25.11	58.66	6.69E-01	15.52	40.56	7.02E-01
cg01211424	-11.48	4.96	2.06E-02	-7.31	14.93	6.25E-01	-29.50	20.36	1.47E-01	-10.13	5.70	7.57E-02	-17.07	18.25	3.50E-01
cg25008346	24.30	10.50	2.07E-02	44.83	20.64	2.99E-02	43.71	34.05	1.99E-01	25.03	11.38	2.79E-02	-9.52	22.05	6.66E-01
cg22061832	8.89	3.84	2.07E-02	14.76	5.38	6.07E-03	9.64	8.49	2.56E-01	6.02	10.72	5.75E-01	-1.96	7.85	8.03E-01
cg26199445	16.86	7.29	2.07E-02	21.13	9.68	2.90E-02	35.90	18.74	5.55E-02	-3.01	23.69	8.99E-01	-0.17	16.33	9.92E-01
cg16393715	15.83	6.84	2.07E-02	13.18	10.51	2.10E-01	20.15	13.95	1.49E-01	16.55	19.21	3.89E-01	15.76	14.94	2.91E-01
cg09456782	10.31	4.46	2.07E-02	13.86	7.17	5.32E-02	11.90	8.75	1.74E-01	19.90	10.67	6.21E-02	-2.18	8.46	7.97E-01
cg00099766	10.05	4.34	2.07E-02	10.71	5.74	6.19E-02	17.04	10.26	9.68E-02	4.99	18.05	7.82E-01	3.00	9.96	7.63E-01
cg21926451	-18.15	7.84	2.07E-02	-19.17	15.63	2.20E-01	-15.08	33.01	6.48E-01	-14.00	11.26	2.14E-01	-27.47	17.24	1.11E-01
cg21771660	-45.10	19.49	2.07E-02	-26.97	21.11	2.01E-01	30.77	73.02	6.73E-01	-99.63	45.47	2.84E-02	-67.02	34.29	5.07E-02
cg12494774	10.60	4.58	2.07E-02	6.35	6.76	3.48E-01	21.12	9.75	3.03E-02	13.11	11.32	2.47E-01	5.61	11.57	6.27E-01
cg15456082	-11.59	5.01	2.07E-02	-18.50	8.11	2.25E-02	2.58	11.54	8.23E-01	-8.08	11.36	4.77E-01	-14.62	10.32	1.57E-01
cg13550642	20.99	9.07	2.07E-02	38.19	18.06	3.44E-02	17.78	17.76	3.17E-01	5.67	15.12	7.07E-01	36.93	25.53	1.48E-01
cg26250143	60.19	26.01	2.07E-02	102.08	69.35	1.41E-01	-155.33	281.35	5.81E-01	51.59	29.66	8.20E-02	91.61	91.06	3.14E-01
cg06602333	27.97	12.09	2.07E-02	41.79	16.98	1.38E-02	-8.69	35.03	8.04E-01	53.92	39.56	1.73E-01	9.92	22.82	6.64E-01
cg07598034	13.42	5.80	2.07E-02	17.82	13.15	1.75E-01	-87.04	95.88	3.64E-01	22.74	25.82	3.78E-01	12.14	6.69	6.97E-02
cg02691819	31.63	13.67	2.07E-02	91.53	48.03	5.67E-02	14.54	63.36	8.18E-01	26.89	15.39	8.06E-02	27.82	47.38	5.57E-01
cg14372472	-9.38	4.05	2.07E-02	-15.35	8.48	7.04E-02	-15.27	12.50	2.22E-01	-5.67	5.58	3.10E-01	-9.20	10.88	3.98E-01
cg18289141	18.17	7.85	2.07E-02	20.33	10.98	6.42E-02	40.84	24.89	1.01E-01	17.72	21.66	4.13E-01	5.35	15.47	7.29E-01
cg12305265	-9.26	4.00	2.07E-02	-3.87	7.10	5.86E-01	-7.27	9.19	4.29E-01	-12.72	10.95	2.45E-01	-13.79	6.68	3.90E-02
cg00344920	-16.67	7.21	2.07E-02	-44.31	17.13	9.69E-03	-14.48	33.54	6.66E-01	-10.67	5.83	6.72E-02	-16.37	21.10	4.38E-01
cg10511357	-11.45	4.95	2.07E-02	-22.93	9.18	1.26E-02	-6.63	11.78	5.73E-01	-4.94	8.72	5.71E-01	-9.62	10.79	3.73E-01
cg20099005	-9.37	4.05	2.07E-02	-11.19	5.62	4.65E-02	-0.42	10.57	9.68E-01	-9.51	15.70	5.44E-01	-10.71	7.83	1.71E-01
cg25916404	20.69	8.94	2.07E-02	20.86	16.95	2.19E-01	13.38	19.30	4.88E-01	23.76	14.12	9.25E-02	23.42	27.45	3.94E-01
cg06035374	-35.90	15.52	2.07E-02	-45.08	18.57	1.52E-02	-4.14	48.03	9.31E-01	2.98	69.63	9.66E-01	-27.98	40.47	4.89E-01
cg27143204	14.80	6.40	2.07E-02	8.70	7.09	2.20E-01	36.30	11.93	2.34E-03	8.15	15.06	5.88E-01	10.12	11.05	3.60E-01
cg21690489	-25.72	11.12	2.07E-02	-28.67	15.11	5.78E-02	-1.68	30.65	9.56E-01	-37.00	34.00	2.76E-01	-27.37	23.71	2.48E-01
cg21901156	12.76	5.52	2.07E-02	7.14	7.31	3.29E-01	27.76	9.46	3.35E-03	6.43	17.27	7.09E-01	6.66	11.44	5.60E-01
cg15323528	-36.81	15.91	2.07E-02	-39.37	21.98	7.33E-02	-99.77	46.47	3.18E-02	-30.66	45.32	4.99E-01	-2.94	32.81	9.29E-01
cg02681733	28.87	12.48	2.07E-02	-8.82	39.27	8.22E-01	32.90	93.91	7.26E-01	32.98	14.25	2.06E-02	34.00	37.00	3.58E-01
cg09969043	45.52	19.68	2.07E-02	11.90	11.77	3.12E-01	81.09	46.46	8.09E-02	113.99	32.11	3.86E-04	27.63	14.74	6.08E-02
cg21711862	-13.23	5.72	2.07E-02	-15.28	8.32	6.63E-02	-13.73	16.21	3.97E-01	-15.26	17.74	3.90E-01	-9.07	10.46	3.86E-01
cg21584862	-12.22	5.29	2.07E-02	-27.57	12.80	3.12E-02	-29.56	27.03	2.74E-01	-6.79	6.44	2.91E-01	-15.50	15.47	3.16E-01

cg00122406	10.68	4.62	2.07E-02	17.69	6.33	5.17E-03	-0.04	13.18	9.97E-01	2.79	10.88	7.98E-01	4.59	11.40	6.87E-01
cg26739951	-35.22	15.23	2.07E-02	-4.97	52.55	9.25E-01	332.12	270.02	2.19E-01	-42.24	16.46	1.03E-02	5.28	63.97	9.34E-01
cg02512352	25.53	11.04	2.07E-02	16.82	34.59	6.27E-01	78.36	115.25	4.97E-01	30.77	12.56	1.43E-02	-5.65	32.33	8.61E-01
cg10211744	-14.23	6.15	2.07E-02	-21.32	8.96	1.74E-02	-14.88	14.61	3.08E-01	0.07	18.29	9.97E-01	-6.51	12.60	6.06E-01
cg11441533	-32.62	14.11	2.08E-02	-68.73	21.10	1.12E-03	-37.28	34.83	2.84E-01	-15.94	9.11	8.01E-02	-20.60	29.37	4.83E-01
cg15484899	17.70	7.66	2.08E-02	7.67	13.60	5.73E-01	33.15	20.49	1.06E-01	22.05	12.97	8.93E-02	15.19	17.32	3.80E-01
cg02757488	-11.71	5.06	2.08E-02	-17.12	7.36	2.01E-02	-10.85	9.97	2.76E-01	7.24	18.14	6.90E-01	-7.20	11.57	5.34E-01
cg23424003	10.88	4.70	2.08E-02	8.72	9.93	3.80E-01	21.83	16.12	1.76E-01	11.02	6.67	9.83E-02	8.18	10.74	4.46E-01
cg03459415	-8.12	3.51	2.08E-02	-4.63	6.13	4.50E-01	-3.03	8.43	7.19E-01	-14.19	6.10	1.99E-02	-8.19	8.59	3.40E-01
cg11983942	-12.67	5.48	2.08E-02	-13.57	8.28	1.01E-01	-18.58	13.48	1.68E-01	-6.72	12.54	5.92E-01	-11.50	12.07	3.41E-01
cg02335441	-33.42	14.45	2.08E-02	-16.43	21.64	4.48E-01	-32.09	36.28	3.77E-01	-35.15	38.29	3.59E-01	-63.24	28.75	2.78E-02
cg04005707	13.34	5.77	2.08E-02	10.26	10.43	3.26E-01	12.27	16.41	4.55E-01	26.74	11.41	1.91E-02	5.86	10.28	5.69E-01
cg16588500	-18.43	7.97	2.08E-02	1.40	22.90	9.51E-01	-17.28	77.28	8.23E-01	-20.59	8.81	1.94E-02	-31.71	36.08	3.79E-01
cg24907075	-12.30	5.32	2.08E-02	-25.49	14.18	7.21E-02	15.36	28.24	5.87E-01	-10.15	6.21	1.02E-01	-20.20	17.85	2.58E-01
cg06549863	-11.33	4.90	2.08E-02	-16.37	11.94	1.70E-01	4.80	15.67	7.59E-01	-11.39	6.20	6.65E-02	-17.66	14.80	2.33E-01
cg03122282	-16.63	7.19	2.08E-02	-18.02	15.40	2.42E-01	-20.83	16.39	2.04E-01	-10.47	10.95	3.39E-01	-26.46	18.13	1.44E-01
cg18591013	18.83	8.15	2.08E-02	24.18	18.00	1.79E-01	-5.25	22.39	8.15E-01	23.07	10.68	3.08E-02	14.28	28.59	6.17E-01
cg07277869	-23.72	10.26	2.08E-02	-17.38	12.76	1.73E-01	-39.84	30.51	1.92E-01	16.78	42.19	6.91E-01	-49.46	24.10	4.01E-02
cg08928189	-17.58	7.61	2.08E-02	-17.08	11.63	1.42E-01	-10.13	17.89	5.71E-01	-4.94	14.78	7.38E-01	-44.20	17.81	1.31E-02
cg22680761	-9.88	4.27	2.08E-02	-11.38	8.44	1.77E-01	-2.49	13.53	8.54E-01	-10.03	5.97	9.32E-02	-11.98	11.78	3.09E-01
cg06183469	16.51	7.14	2.08E-02	-6.52	18.30	7.22E-01	-0.51	25.09	9.84E-01	22.07	8.49	9.32E-03	32.59	29.41	2.68E-01
cg09567642	-28.12	12.17	2.08E-02	1.59	48.88	9.74E-01	-42.07	205.88	8.38E-01	-30.41	12.85	1.79E-02	-21.12	62.79	7.37E-01
cg11645556	14.31	6.19	2.08E-02	12.64	7.63	9.78E-02	22.82	14.18	1.08E-01	40.52	19.35	3.62E-02	1.81	11.30	8.73E-01
cg27382409	-10.74	4.65	2.08E-02	-8.63	6.56	1.88E-01	-15.48	12.28	2.07E-01	-6.96	12.02	5.63E-01	-15.34	10.25	1.34E-01
cg11688578	24.10	10.43	2.08E-02	32.41	21.87	1.38E-01	58.69	44.31	1.85E-01	15.75	13.45	2.42E-01	34.56	30.61	2.59E-01
cg15015892	18.98	8.21	2.08E-02	-14.56	27.38	5.95E-01	-65.17	96.24	4.98E-01	23.04	8.89	9.54E-03	22.44	37.23	5.47E-01
cg21081971	7.80	3.38	2.08E-02	10.70	5.00	3.25E-02	-0.18	7.95	9.82E-01	11.80	7.76	1.28E-01	4.16	8.06	6.06E-01
cg15436385	-12.60	5.45	2.08E-02	-9.17	7.21	2.03E-01	-26.78	13.54	4.80E-02	-1.46	15.90	9.27E-01	-19.13	14.14	1.76E-01
cg12034678	12.43	5.38	2.08E-02	26.84	19.69	1.73E-01	25.57	38.30	5.04E-01	13.49	6.27	3.14E-02	-0.08	13.07	9.95E-01
cg02127980	8.69	3.76	2.08E-02	6.16	4.67	1.87E-01	13.39	10.10	1.85E-01	27.56	12.66	2.95E-02	3.27	10.65	7.59E-01
cg02764250	-9.53	4.13	2.08E-02	-16.46	9.62	8.70E-02	-8.58	20.99	6.83E-01	-9.56	5.09	6.05E-02	0.86	11.88	9.42E-01
cg19701879	-10.53	4.55	2.08E-02	-12.93	9.61	1.79E-01	-8.46	20.22	6.76E-01	-8.85	5.92	1.35E-01	-14.75	12.54	2.40E-01
cg03489020	-8.67	3.75	2.08E-02	-18.41	7.88	1.95E-02	-3.70	11.46	7.47E-01	-6.02	5.22	2.49E-01	-6.62	9.72	4.96E-01
cg26705765	-64.94	28.10	2.08E-02	-89.33	44.63	4.53E-02	16.75	161.71	9.18E-01	-96.51	57.55	9.36E-02	-20.94	48.57	6.66E-01
cg04636841	28.24	12.22	2.08E-02	28.15	16.70	9.19E-02	-85.30	75.25	2.57E-01	67.03	44.99	1.36E-01	28.73	19.83	1.47E-01

cg27203090	-48.19	20.85	2.09E-02	-11.55	41.64	7.82E-01	-50.98	209.52	8.08E-01	-69.53	27.13	1.04E-02	-24.96	54.14	6.45E-01
cg03960699	44.00	19.04	2.09E-02	47.62	23.01	3.85E-02	-5.99	73.17	9.35E-01	-5.33	64.24	9.34E-01	76.88	47.70	1.07E-01
cg17200564	12.37	5.35	2.09E-02	22.48	14.30	1.16E-01	13.81	25.78	5.92E-01	13.36	6.22	3.15E-02	-17.14	19.52	3.80E-01
cg01680667	-48.42	20.96	2.09E-02	-45.95	26.29	8.05E-02	-176.17	86.21	4.10E-02	-8.14	83.93	9.23E-01	-34.18	42.50	4.21E-01
cg03769939	-7.44	3.22	2.09E-02	-8.38	4.30	5.13E-02	-8.68	8.23	2.92E-01	0.09	9.41	9.92E-01	-8.44	7.86	2.83E-01
cg08564601	9.74	4.22	2.09E-02	11.86	5.58	3.36E-02	18.34	10.25	7.34E-02	12.48	21.01	5.53E-01	-2.86	8.98	7.50E-01
cg16715127	18.06	7.82	2.09E-02	17.97	15.11	2.34E-01	12.52	16.69	4.53E-01	11.78	13.26	3.74E-01	38.73	19.22	4.39E-02
cg07333223	38.33	16.59	2.09E-02	39.67	29.10	1.73E-01	-118.75	187.69	5.27E-01	47.76	21.98	2.98E-02	-8.59	53.10	8.71E-01
cg05358758	-11.66	5.05	2.09E-02	-1.26	9.50	8.95E-01	-19.21	17.94	2.84E-01	-10.44	6.04	8.36E-02	-28.99	12.43	1.97E-02
cg23326901	-19.48	8.43	2.09E-02	-33.08	23.20	1.54E-01	55.96	68.25	4.12E-01	-17.09	9.61	7.55E-02	-33.87	29.23	2.47E-01
cg01269913	-16.13	6.98	2.09E-02	-24.37	12.85	5.80E-02	5.16	27.05	8.49E-01	-13.18	9.57	1.68E-01	-21.34	21.44	3.20E-01
cg01422009	12.85	5.56	2.09E-02	11.02	7.72	1.53E-01	23.17	12.30	5.96E-02	11.56	16.43	4.82E-01	6.57	13.83	6.35E-01
cg06352873	-9.63	4.17	2.09E-02	-22.34	9.69	2.12E-02	-6.66	11.92	5.76E-01	-4.65	5.52	4.00E-01	-16.61	11.93	1.64E-01
cg20183802	14.26	6.17	2.09E-02	16.46	10.60	1.21E-01	24.19	14.23	8.92E-02	18.76	12.25	1.26E-01	-2.87	13.18	8.27E-01
cg04608722	-19.84	8.59	2.09E-02	-4.19	23.76	8.60E-01	-54.45	39.63	1.69E-01	-21.57	9.85	2.86E-02	-5.61	34.33	8.70E-01
cg26239051	33.42	14.47	2.09E-02	37.35	16.81	2.63E-02	18.09	92.30	8.45E-01	-1.98	84.81	9.81E-01	26.09	31.94	4.14E-01
cg03543495	26.80	11.60	2.09E-02	49.34	25.54	5.34E-02	10.66	77.31	8.90E-01	18.53	13.81	1.80E-01	50.40	45.36	2.66E-01
cg04522792	-15.25	6.60	2.09E-02	-11.00	16.16	4.96E-01	-25.58	19.14	1.81E-01	-14.96	8.37	7.37E-02	-11.54	21.88	5.98E-01
cg27101899	14.87	6.44	2.09E-02	19.54	16.75	2.43E-01	-13.07	20.42	5.22E-01	16.48	7.77	3.38E-02	29.57	24.99	2.37E-01
cg26538140	5.51	2.39	2.09E-02	5.02	2.98	9.23E-02	11.13	5.88	5.81E-02	6.21	8.68	4.74E-01	-0.06	6.89	9.93E-01
cg13915277	-9.59	4.15	2.09E-02	-12.70	7.76	1.02E-01	-8.68	9.82	3.77E-01	-10.71	6.87	1.19E-01	-2.89	10.10	7.74E-01
cg07427211	13.43	5.81	2.09E-02	17.16	8.13	3.48E-02	14.52	11.52	2.08E-01	1.94	18.36	9.16E-01	5.67	15.90	7.21E-01
cg24960291	10.12	4.38	2.09E-02	13.42	6.73	4.61E-02	15.44	10.36	1.36E-01	6.15	9.05	4.97E-01	1.37	10.88	8.99E-01
cg15376097	10.35	4.48	2.09E-02	13.99	5.95	1.87E-02	5.40	10.08	5.92E-01	-6.89	16.33	6.73E-01	11.70	11.22	2.97E-01
cg19100464	21.76	9.42	2.09E-02	13.78	19.21	4.73E-01	24.48	20.47	2.32E-01	25.66	14.20	7.07E-02	18.25	28.75	5.26E-01
cg05903710	-17.94	7.77	2.09E-02	-19.61	19.78	3.21E-01	28.17	28.13	3.17E-01	-23.17	8.19	4.64E-03	-10.74	28.88	7.10E-01
cg03639864	-15.41	6.67	2.09E-02	-16.24	13.77	2.38E-01	-1.98	27.19	9.42E-01	-17.95	8.57	3.61E-02	-5.94	21.25	7.80E-01
cg06523618	-10.83	4.69	2.09E-02	-12.15	13.57	3.70E-01	-16.75	14.36	2.43E-01	-10.68	5.66	5.93E-02	-3.02	15.81	8.48E-01
cg20710902	-11.42	4.95	2.09E-02	-12.81	9.33	1.70E-01	-1.16	12.20	9.24E-01	-12.51	9.14	1.71E-01	-15.16	9.67	1.17E-01
cg03147461	12.71	5.50	2.09E-02	-4.65	15.83	7.69E-01	36.03	19.62	6.63E-02	14.00	6.44	2.98E-02	3.08	20.67	8.82E-01
cg16079364	-8.80	3.81	2.09E-02	-14.28	5.85	1.46E-02	-0.63	8.76	9.42E-01	-12.94	9.15	1.57E-01	-1.75	8.28	8.33E-01
cg07638105	25.89	11.21	2.09E-02	13.25	26.98	6.23E-01	-6.04	63.10	9.24E-01	29.50	13.20	2.54E-02	33.83	41.17	4.11E-01
cg24134020	-15.76	6.83	2.09E-02	-7.69	21.89	7.25E-01	-47.87	52.17	3.59E-01	-16.74	7.45	2.46E-02	-2.81	32.01	9.30E-01
cg26346167	-8.12	3.52	2.10E-02	-12.64	6.64	5.68E-02	7.57	10.69	4.78E-01	-7.95	5.22	1.28E-01	-11.36	8.88	2.00E-01
cg22540067	-9.27	4.02	2.10E-02	-5.28	5.77	3.60E-01	-12.94	8.96	1.49E-01	-15.46	10.71	1.49E-01	-11.16	9.63	2.47E-01

cg10888644	19.89	8.62	2.10E-02	5.72	17.25	7.40E-01	21.03	22.80	3.56E-01	25.87	12.16	3.34E-02	23.45	26.50	3.76E-01
cg27602263	19.65	8.51	2.10E-02	21.15	31.19	4.98E-01	-29.12	78.21	7.10E-01	17.71	9.25	5.56E-02	50.82	32.75	1.21E-01
cg09659400	-10.14	4.39	2.10E-02	-8.99	8.02	2.62E-01	-13.80	10.25	1.78E-01	-12.01	7.90	1.29E-01	-5.79	9.62	5.48E-01
cg00492083	20.96	9.08	2.10E-02	39.96	20.21	4.80E-02	35.79	19.80	7.07E-02	5.93	11.53	6.07E-01	30.92	26.52	2.44E-01
cg10805736	23.60	10.22	2.10E-02	5.16	28.05	8.54E-01	-14.45	151.71	9.24E-01	29.47	11.41	9.81E-03	-11.18	41.70	7.89E-01
cg25497530	19.26	8.34	2.10E-02	5.55	9.95	5.77E-01	31.69	13.71	2.09E-02	46.02	20.96	2.81E-02	13.01	13.46	3.34E-01
cg22638105	-19.65	8.51	2.10E-02	-18.30	17.78	3.03E-01	3.02	19.77	8.79E-01	-25.98	12.51	3.78E-02	-32.68	24.39	1.80E-01
cg15722533	-10.74	4.65	2.10E-02	-17.69	11.89	1.37E-01	-3.96	22.99	8.63E-01	-7.92	5.50	1.50E-01	-24.21	15.39	1.16E-01
cg01226265	24.42	10.58	2.10E-02	5.16	20.63	8.02E-01	18.29	31.86	5.66E-01	30.21	16.22	6.25E-02	40.70	23.57	8.42E-02
cg10824972	-10.44	4.52	2.10E-02	-18.45	11.26	1.01E-01	-17.84	23.88	4.55E-01	-8.43	5.26	1.09E-01	-9.36	18.00	6.03E-01
cg26782169	15.37	6.66	2.10E-02	12.15	18.17	5.04E-01	28.00	24.95	2.62E-01	15.49	7.81	4.72E-02	7.03	25.73	7.85E-01
cg26571618	-34.34	14.88	2.10E-02	-48.93	19.89	1.39E-02	-17.93	45.03	6.91E-01	-2.47	52.66	9.63E-01	-19.11	29.67	5.19E-01
cg24957476	43.73	18.95	2.10E-02	39.17	21.61	6.99E-02	14.99	108.45	8.90E-01	55.97	66.51	4.00E-01	72.05	54.78	1.88E-01
cg01962510	-40.09	17.37	2.10E-02	-45.74	50.94	3.69E-01	107.35	212.72	6.14E-01	-41.26	19.57	3.50E-02	-33.42	58.09	5.65E-01
cg17187595	36.22	15.69	2.10E-02	18.56	20.85	3.73E-01	36.73	81.76	6.53E-01	88.81	43.94	4.33E-02	48.40	30.25	1.10E-01
cg16699326	-33.21	14.39	2.10E-02	-40.09	23.80	9.22E-02	-44.49	73.12	5.43E-01	-25.01	23.96	2.97E-01	-33.23	29.65	2.62E-01
cg05585556	-17.03	7.38	2.10E-02	-34.68	12.85	6.97E-03	-7.87	20.21	6.97E-01	-14.32	10.10	1.56E-01	3.68	19.53	8.51E-01
cg07527467	17.39	7.53	2.10E-02	28.03	22.62	2.15E-01	35.86	26.54	1.77E-01	14.55	8.68	9.37E-02	7.80	31.93	8.07E-01
cg08175640	-10.36	4.49	2.10E-02	-23.42	11.46	4.10E-02	-6.46	17.53	7.12E-01	-6.89	5.52	2.12E-01	-14.91	12.97	2.50E-01
cg01348055	-15.89	6.89	2.10E-02	-22.22	8.80	1.16E-02	-5.07	13.89	7.15E-01	9.14	18.23	6.16E-01	-26.38	11.58	2.28E-02
cg23034827	14.50	6.28	2.10E-02	16.95	14.71	2.49E-01	-6.18	15.89	6.97E-01	16.60	8.41	4.84E-02	30.00	19.49	1.24E-01
cg05959277	22.27	9.65	2.10E-02	16.31	17.67	3.56E-01	17.02	36.82	6.44E-01	34.30	13.48	1.09E-02	-11.08	27.76	6.90E-01
cg23241878	30.45	13.20	2.10E-02	38.17	17.84	3.24E-02	47.77	43.83	2.76E-01	-28.72	44.75	5.21E-01	28.11	25.15	2.64E-01
cg24737618	-78.57	34.04	2.10E-02	-103.81	51.53	4.39E-02	-89.15	270.81	7.42E-01	-33.92	21.97	1.23E-01	-169.34	67.38	1.20E-02
cg23189692	5.96	2.58	2.10E-02	4.31	3.52	2.20E-01	11.14	5.62	4.73E-02	6.90	9.21	4.53E-01	4.30	6.23	4.89E-01
cg23752086	21.25	9.21	2.10E-02	34.19	13.16	9.39E-03	-0.05	21.55	9.98E-01	12.91	33.33	6.98E-01	14.07	18.36	4.43E-01
cg19512903	-9.38	4.07	2.10E-02	-12.71	9.67	1.89E-01	-0.48	12.23	9.68E-01	-7.50	5.19	1.48E-01	-24.90	12.88	5.32E-02
cg23550757	-36.88	15.98	2.10E-02	-36.05	18.30	4.88E-02	-96.67	105.25	3.58E-01	-21.54	51.71	6.77E-01	-42.93	46.43	3.55E-01
cg00838829	-18.81	8.15	2.10E-02	-20.21	18.44	2.73E-01	56.48	50.11	2.60E-01	-23.24	9.83	1.81E-02	-4.17	27.03	8.77E-01
cg07197230	7.23	3.13	2.10E-02	4.79	4.12	2.44E-01	11.51	7.58	1.29E-01	15.45	11.95	1.96E-01	7.87	7.37	2.85E-01
cg20222052	30.72	13.31	2.10E-02	11.10	36.98	7.64E-01	96.49	152.22	5.26E-01	38.80	15.20	1.07E-02	-13.07	43.16	7.62E-01
cg01432120	12.51	5.42	2.10E-02	3.07	7.43	6.79E-01	25.59	13.01	4.92E-02	17.26	15.07	2.52E-01	19.81	10.94	7.01E-02
cg20359349	-7.10	3.08	2.11E-02	-6.38	4.72	1.76E-01	-3.54	6.59	5.91E-01	-10.64	7.64	1.64E-01	-9.70	6.98	1.65E-01
cg12509665	8.66	3.75	2.11E-02	8.69	5.17	9.31E-02	13.24	8.78	1.32E-01	6.82	9.99	4.95E-01	4.71	9.69	6.26E-01
cg04520420	22.01	9.54	2.11E-02	42.78	32.39	1.87E-01	-9.93	102.82	9.23E-01	20.93	10.41	4.44E-02	12.43	37.49	7.40E-01

cg05398769	-15.17	6.58	2.11E-02	-26.16	11.86	2.74E-02	-4.68	12.02	6.97E-01	-30.77	16.68	6.51E-02	-5.38	12.53	6.67E-01
cg04707519	6.42	2.78	2.11E-02	6.56	4.98	1.88E-01	7.60	5.82	1.92E-01	0.27	5.87	9.63E-01	10.99	5.75	5.61E-02
cg07062336	9.35	4.05	2.11E-02	11.19	5.39	3.79E-02	14.21	9.55	1.37E-01	13.57	15.53	3.82E-01	-2.43	9.36	7.95E-01
cg24074594	-10.64	4.61	2.11E-02	-9.90	10.70	3.55E-01	-16.76	25.75	5.15E-01	-12.50	5.57	2.49E-02	3.04	14.78	8.37E-01
cg24985060	27.01	11.71	2.11E-02	7.43	25.44	7.70E-01	-1.88	62.07	9.76E-01	38.91	14.57	7.58E-03	3.57	35.78	9.21E-01
cg15651204	-26.04	11.29	2.11E-02	-32.90	16.19	4.21E-02	-4.73	35.42	8.94E-01	-26.01	24.60	2.90E-01	-20.28	25.18	4.21E-01
cg14779404	-9.90	4.29	2.11E-02	-6.89	8.28	4.06E-01	-12.03	11.89	3.12E-01	-13.02	6.44	4.31E-02	-4.43	10.84	6.82E-01
cg04469059	22.17	9.61	2.11E-02	33.99	13.89	1.44E-02	21.49	40.65	5.97E-01	13.22	27.66	6.33E-01	8.99	16.38	5.83E-01
cg13692972	4.95	2.15	2.11E-02	2.96	2.82	2.94E-01	8.72	5.04	8.38E-02	9.12	5.51	9.80E-02	3.13	7.35	6.70E-01
cg19888344	-19.09	8.28	2.11E-02	-4.74	19.39	8.07E-01	-32.74	42.40	4.40E-01	-23.77	10.02	1.77E-02	-7.85	26.45	7.67E-01
cg08015388	-48.62	21.08	2.11E-02	-51.93	19.53	7.83E-03	39.99	52.02	4.42E-01	-103.55	28.64	3.00E-04	-34.81	23.31	1.35E-01
cg15202627	19.54	8.47	2.11E-02	-3.32	22.21	8.81E-01	0.94	69.60	9.89E-01	21.11	9.93	3.36E-02	41.39	25.27	1.01E-01
cg02553070	-19.82	8.59	2.11E-02	-21.67	11.70	6.40E-02	-9.53	21.55	6.58E-01	-43.90	34.79	2.07E-01	-16.36	17.52	3.50E-01
cg20629551	-11.61	5.03	2.11E-02	-19.54	13.56	1.50E-01	-2.33	19.75	9.06E-01	-10.30	6.04	8.83E-02	-15.68	15.69	3.18E-01
cg06575288	16.74	7.26	2.11E-02	10.10	10.60	3.41E-01	18.97	34.18	5.79E-01	34.41	22.53	1.27E-01	19.84	11.75	9.13E-02
cg27426340	19.43	8.43	2.11E-02	28.29	17.00	9.59E-02	21.77	13.91	1.18E-01	8.58	18.37	6.40E-01	15.17	20.03	4.49E-01
cg08130265	6.53	2.83	2.11E-02	4.33	3.77	2.52E-01	14.94	6.79	2.78E-02	9.76	7.79	2.10E-01	1.55	7.86	8.44E-01
cg17345741	-12.65	5.49	2.11E-02	-15.12	8.59	7.83E-02	2.74	13.78	8.42E-01	-14.73	11.10	1.85E-01	-17.55	12.62	1.64E-01
cg11110643	26.13	11.33	2.11E-02	29.35	20.19	1.46E-01	23.04	31.37	4.63E-01	32.04	18.80	8.84E-02	11.72	25.93	6.51E-01
cg11510891	18.38	7.97	2.11E-02	21.56	13.27	1.04E-01	31.74	15.17	3.64E-02	17.32	20.81	4.05E-01	-3.24	17.13	8.50E-01
cg22935609	-12.51	5.43	2.11E-02	-28.65	10.50	6.39E-03	-5.68	14.28	6.91E-01	-6.61	7.11	3.52E-01	-11.39	13.99	4.16E-01
cg03313126	-13.29	5.77	2.11E-02	-9.46	9.63	3.26E-01	-1.76	13.81	8.99E-01	-20.83	10.48	4.70E-02	-20.01	14.21	1.59E-01
cg10185885	41.19	17.87	2.12E-02	30.91	20.17	1.25E-01	-95.15	128.63	4.60E-01	125.21	67.01	6.17E-02	55.61	34.88	1.11E-01
cg19103219	11.24	4.88	2.12E-02	4.74	6.33	4.54E-01	18.12	9.43	5.48E-02	23.63	9.44	1.23E-02	3.28	9.56	7.32E-01
cg11158239	23.33	10.12	2.12E-02	21.96	12.95	8.99E-02	20.80	41.00	6.12E-01	38.65	52.46	4.61E-01	24.78	18.77	1.87E-01
cg26789698	-13.28	5.76	2.12E-02	-22.33	9.23	1.56E-02	-6.79	12.65	5.92E-01	-10.52	12.04	3.82E-01	-4.42	13.80	7.49E-01
cg21025494	17.41	7.55	2.12E-02	22.62	11.25	4.44E-02	17.46	19.33	3.66E-01	40.24	21.89	6.60E-02	-0.87	14.33	9.52E-01
cg12844324	8.20	3.56	2.12E-02	8.63	5.15	9.41E-02	4.96	6.87	4.70E-01	13.97	11.22	2.13E-01	8.74	9.05	3.35E-01
cg16846645	10.31	4.48	2.12E-02	19.77	8.81	2.49E-02	1.83	11.04	8.68E-01	15.25	8.64	7.75E-02	2.65	8.05	7.42E-01
cg14014604	-36.95	16.03	2.12E-02	-18.88	39.94	6.36E-01	-6.36	158.67	9.68E-01	-46.89	18.50	1.13E-02	17.61	57.54	7.60E-01
cg00339769	14.19	6.16	2.12E-02	16.30	8.53	5.60E-02	27.47	15.23	7.12E-02	13.43	16.45	4.14E-01	-3.91	14.73	7.91E-01
cg16905586	26.75	11.61	2.12E-02	9.13	19.11	6.33E-01	27.67	31.90	3.86E-01	46.40	20.36	2.27E-02	26.69	27.83	3.37E-01
cg20196129	21.06	9.14	2.12E-02	11.50	24.10	6.33E-01	-77.75	79.00	3.25E-01	22.76	10.35	2.78E-02	43.10	36.59	2.39E-01
cg26888225	-48.40	21.00	2.12E-02	4.72	52.66	9.29E-01	-114.63	240.80	6.34E-01	-57.05	24.39	1.93E-02	-65.14	69.31	3.47E-01
cg24011531	37.82	16.41	2.12E-02	61.56	44.52	1.67E-01	-111.53	176.11	5.27E-01	29.76	18.45	1.07E-01	106.38	64.50	9.90E-02

cg08358907	8.32	3.61	2.12E-02	5.36	4.67	2.50E-01	19.14	8.50	2.43E-02	5.22	12.66	6.80E-01	8.84	9.69	3.62E-01
cg24133591	25.16	10.92	2.12E-02	5.22	31.27	8.68E-01	23.65	100.30	8.14E-01	30.26	12.83	1.84E-02	16.41	28.97	5.71E-01
cg07303548	11.97	5.20	2.12E-02	4.83	7.44	5.16E-01	23.58	11.18	3.49E-02	7.05	15.77	6.55E-01	19.99	11.98	9.53E-02
cg14712186	29.28	12.71	2.12E-02	38.86	35.67	2.76E-01	35.96	54.63	5.10E-01	16.52	8.70	5.76E-02	70.50	27.83	1.13E-02
cg16783517	51.39	22.30	2.12E-02	56.77	28.08	4.32E-02	52.31	59.27	3.78E-01	-41.45	58.88	4.81E-01	89.88	41.48	3.03E-02
cg27262735	15.53	6.74	2.12E-02	26.02	13.05	4.62E-02	49.17	32.79	1.34E-01	7.68	9.06	3.97E-01	16.44	18.16	3.65E-01
cg20430841	10.08	4.37	2.12E-02	8.57	5.61	1.27E-01	20.11	11.63	8.38E-02	-4.86	19.93	8.07E-01	11.12	9.70	2.52E-01
cg08007465	22.31	9.68	2.12E-02	31.70	14.44	2.82E-02	10.60	30.08	7.25E-01	24.54	28.29	3.86E-01	12.39	16.87	4.63E-01
cg22331862	11.78	5.11	2.12E-02	13.50	7.87	8.65E-02	8.40	10.96	4.44E-01	13.22	14.26	3.54E-01	11.02	10.60	2.98E-01
cg10287137	8.35	3.62	2.12E-02	7.48	4.63	1.06E-01	11.89	9.54	2.13E-01	16.69	9.68	8.45E-02	-2.97	11.39	7.94E-01
cg17125477	31.32	13.59	2.12E-02	44.75	36.94	2.26E-01	18.69	97.96	8.49E-01	28.28	16.15	8.00E-02	35.52	36.72	3.33E-01
cg26227242	-45.32	19.67	2.12E-02	-52.72	24.55	3.18E-02	39.96	70.26	5.70E-01	-153.07	114.53	1.81E-01	-40.35	39.35	3.05E-01
cg16118803	-5.29	2.30	2.12E-02	-7.58	3.15	1.60E-02	-3.92	5.90	5.07E-01	-1.41	6.88	8.38E-01	-2.47	5.09	6.28E-01
cg02598564	-13.24	5.75	2.12E-02	-11.83	9.54	2.15E-01	-10.98	12.94	3.96E-01	-16.20	11.96	1.76E-01	-14.58	12.59	2.47E-01
cg04453552	-38.17	16.57	2.12E-02	-27.37	27.79	3.25E-01	114.29	117.93	3.32E-01	-73.97	37.52	4.87E-02	-37.86	25.28	1.34E-01
cg18388559	19.83	8.61	2.13E-02	8.92	12.25	4.67E-01	41.29	14.40	4.14E-03	21.91	22.91	3.39E-01	7.06	18.61	7.04E-01
cg15247645	-29.02	12.60	2.13E-02	-85.25	36.41	1.92E-02	-12.63	215.77	9.53E-01	-22.01	13.91	1.13E-01	-12.46	53.28	8.15E-01
cg26688936	-75.30	32.69	2.13E-02	-39.19	41.07	3.40E-01	38.45	116.79	7.42E-01	-96.42	49.32	5.06E-02	-173.05	73.35	1.83E-02
cg01687558	-40.53	17.60	2.13E-02	-41.20	22.21	6.36E-02	85.57	110.79	4.40E-01	-74.44	43.84	8.96E-02	-26.00	40.79	5.24E-01
cg23158754	12.18	5.29	2.13E-02	12.95	7.56	8.65E-02	19.68	13.18	1.35E-01	14.16	19.41	4.66E-01	5.88	10.10	5.60E-01
cg05422957	27.80	12.07	2.13E-02	38.88	16.31	1.72E-02	-14.35	42.49	7.35E-01	28.62	30.78	3.52E-01	15.02	25.84	5.61E-01
cg21086066	-34.88	15.14	2.13E-02	-24.38	47.40	6.07E-01	183.12	180.58	3.11E-01	-34.20	16.94	4.35E-02	-68.98	49.87	1.67E-01
cg18931014	-16.04	6.96	2.13E-02	-10.34	13.42	4.41E-01	5.83	41.85	8.89E-01	-18.47	8.88	3.75E-02	-23.35	23.46	3.20E-01
cg05757448	-18.91	8.21	2.13E-02	-26.99	10.72	1.18E-02	9.97	21.14	6.37E-01	-33.69	15.14	2.60E-02	-8.71	14.01	5.34E-01
cg05034374	-25.09	10.89	2.13E-02	-45.64	20.79	2.81E-02	15.53	36.37	6.69E-01	-31.34	10.47	2.75E-03	3.29	25.24	8.96E-01
cg24211006	-6.39	2.77	2.13E-02	-5.45	4.71	2.47E-01	-6.10	5.03	2.25E-01	-1.72	7.70	8.23E-01	-11.02	5.92	6.27E-02
cg25790365	7.38	3.20	2.13E-02	7.39	5.85	2.07E-01	8.33	9.11	3.60E-01	7.37	4.60	1.09E-01	6.09	10.62	5.66E-01
cg00744791	-9.49	4.12	2.13E-02	-11.05	5.96	6.37E-02	-2.02	9.89	8.38E-01	9.65	16.22	5.52E-01	-15.75	7.72	4.14E-02
cg24492694	-30.51	13.25	2.13E-02	-27.56	20.52	1.79E-01	3.65	58.15	9.50E-01	-48.35	29.04	9.59E-02	-28.32	23.31	2.24E-01
cg11855842	-16.49	7.16	2.13E-02	-15.54	10.13	1.25E-01	-13.24	18.50	4.74E-01	-30.81	17.94	8.60E-02	-9.61	16.38	5.57E-01
cg00750402	-44.83	19.47	2.13E-02	-35.57	24.67	1.49E-01	-107.20	64.81	9.81E-02	14.76	74.23	8.42E-01	-64.29	41.68	1.23E-01
cg11374368	9.37	4.07	2.13E-02	16.72	6.62	1.15E-02	1.13	10.08	9.11E-01	2.33	8.83	7.92E-01	9.64	8.20	2.40E-01
cg24860819	-31.91	13.85	2.13E-02	-23.29	19.40	2.30E-01	-3.02	37.09	9.35E-01	-27.83	41.70	5.05E-01	-68.90	28.29	1.49E-02
cg18758987	-14.13	6.14	2.13E-02	-23.23	11.69	4.69E-02	-9.31	15.23	5.41E-01	-8.52	9.65	3.77E-01	-17.63	15.47	2.55E-01
cg14838248	6.70	2.91	2.13E-02	4.52	6.06	4.56E-01	-1.78	9.84	8.56E-01	9.32	3.76	1.31E-02	2.69	10.20	7.92E-01

cg27145409	39.85	17.30	2.13E-02	11.51	13.05	3.78E-01	76.92	45.13	8.83E-02	89.65	36.83	1.49E-02	38.81	20.33	5.63E-02
cg02088166	24.31	10.56	2.13E-02	18.15	17.58	3.02E-01	39.33	22.90	8.59E-02	26.05	22.15	2.40E-01	17.47	23.60	4.59E-01
cg14948448	-16.64	7.23	2.13E-02	3.67	21.18	8.63E-01	-52.28	27.59	5.81E-02	-17.39	8.27	3.54E-02	-3.78	31.96	9.06E-01
cg19570596	27.55	11.97	2.13E-02	19.87	32.23	5.38E-01	-76.11	144.92	5.99E-01	25.71	13.63	5.94E-02	65.08	41.05	1.13E-01
cg03900241	-11.28	4.90	2.13E-02	-24.69	10.51	1.88E-02	14.59	20.63	4.79E-01	-10.19	5.03	4.30E-02	-7.13	13.52	5.98E-01
cg03031868	27.43	11.91	2.13E-02	29.42	13.64	3.10E-02	79.32	46.17	8.58E-02	11.45	70.48	8.71E-01	-4.32	31.57	8.91E-01
cg11400761	26.12	11.34	2.13E-02	52.35	16.07	1.13E-03	-6.56	24.19	7.86E-01	17.92	6.70	7.51E-03	40.61	26.13	1.20E-01
cg13134950	-18.20	7.91	2.13E-02	-17.94	12.28	1.44E-01	1.93	17.28	9.11E-01	-34.60	18.14	5.65E-02	-24.70	18.33	1.78E-01
cg01075559	18.78	8.16	2.13E-02	16.52	20.06	4.10E-01	15.91	17.79	3.71E-01	13.60	11.13	2.22E-01	61.52	27.51	2.53E-02
cg05269432	-15.08	6.55	2.13E-02	-21.90	19.38	2.58E-01	5.73	26.83	8.31E-01	-14.13	7.43	5.73E-02	-39.95	29.72	1.79E-01
cg24631735	-10.26	4.46	2.13E-02	-13.95	11.55	2.27E-01	-8.64	20.83	6.78E-01	-9.14	5.33	8.61E-02	-13.20	13.74	3.37E-01
cg27300494	-9.90	4.30	2.13E-02	-16.97	8.16	3.76E-02	1.44	9.72	8.82E-01	-13.20	8.24	1.09E-01	-7.36	8.51	3.87E-01
cg16024904	12.94	5.62	2.13E-02	-6.88	16.67	6.80E-01	36.83	34.03	2.79E-01	15.51	6.90	2.46E-02	12.43	12.73	3.29E-01
cg14781281	13.81	6.00	2.13E-02	0.05	15.95	9.97E-01	24.67	24.44	3.13E-01	15.61	7.85	4.67E-02	14.92	12.95	2.49E-01
cg15598120	-19.49	8.46	2.13E-02	-18.70	10.70	8.03E-02	-37.64	19.17	4.96E-02	14.78	22.07	5.03E-01	-26.87	15.70	8.70E-02
cg16295030	-8.36	3.63	2.13E-02	-13.26	8.04	9.92E-02	-11.78	13.47	3.82E-01	-7.98	4.75	9.28E-02	-0.94	9.79	9.24E-01
cg02167986	-11.31	4.91	2.13E-02	-17.08	13.96	2.21E-01	-21.75	30.25	4.72E-01	-10.92	5.70	5.51E-02	-4.68	15.07	7.56E-01
cg09241243	-40.22	17.47	2.13E-02	-8.34	51.29	8.71E-01	-85.26	203.43	6.75E-01	-45.18	19.61	2.12E-02	-33.31	60.77	5.84E-01
cg00116463	29.23	12.70	2.13E-02	38.86	22.89	8.95E-02	-26.14	73.92	7.24E-01	18.94	17.75	2.86E-01	55.36	32.71	9.05E-02
cg25905674	57.29	24.89	2.13E-02	60.63	38.68	1.17E-01	-42.01	145.78	7.73E-01	30.65	15.16	4.33E-02	127.24	42.68	2.87E-03
cg08322205	20.49	8.90	2.14E-02	15.19	24.82	5.40E-01	36.83	121.34	7.61E-01	24.16	10.38	1.99E-02	4.35	24.66	8.60E-01
cg10975391	-11.63	5.05	2.14E-02	-16.11	8.97	7.25E-02	-8.60	12.37	4.87E-01	-11.35	9.02	2.08E-01	-7.54	11.25	5.03E-01
cg19671648	-14.09	6.12	2.14E-02	-23.28	14.74	1.14E-01	15.10	22.57	5.04E-01	-17.01	6.15	5.67E-03	9.43	24.27	6.98E-01
cg22147539	27.51	11.95	2.14E-02	45.81	27.17	9.18E-02	-25.08	119.78	8.34E-01	29.96	14.12	3.39E-02	-31.93	42.18	4.49E-01
cg26346104	-38.35	16.66	2.14E-02	-49.42	21.35	2.06E-02	-49.99	99.33	6.15E-01	-59.84	76.80	4.36E-01	-12.75	29.66	6.67E-01
cg00760938	-14.82	6.44	2.14E-02	-23.87	10.56	2.39E-02	-8.36	19.12	6.62E-01	-13.45	10.50	2.00E-01	0.42	17.30	9.81E-01
cg06551493	9.18	3.99	2.14E-02	11.72	7.63	1.24E-01	-19.95	20.42	3.29E-01	7.87	5.37	1.43E-01	17.61	10.83	1.04E-01
cg00019275	-9.85	4.28	2.14E-02	-16.31	8.80	6.38E-02	-8.62	12.94	5.06E-01	-5.55	6.03	3.57E-01	-15.00	11.05	1.75E-01
cg22272492	21.77	9.46	2.14E-02	29.64	17.84	9.65E-02	16.34	19.22	3.95E-01	18.01	15.92	2.58E-01	25.22	26.90	3.49E-01
cg02239453	-8.45	3.67	2.14E-02	-15.67	7.23	3.03E-02	-4.51	14.81	7.61E-01	-3.11	5.07	5.40E-01	-15.97	9.27	8.49E-02
cg18117228	19.30	8.39	2.14E-02	5.81	20.55	7.77E-01	-19.08	63.02	7.62E-01	22.23	9.78	2.30E-02	28.96	29.61	3.28E-01
cg25104397	8.16	3.55	2.14E-02	9.75	5.08	5.48E-02	14.08	8.97	1.16E-01	5.67	8.05	4.82E-01	0.65	8.80	9.41E-01
cg01124575	-8.77	3.81	2.14E-02	-16.40	6.45	1.10E-02	-3.62	9.34	6.98E-01	-7.52	6.40	2.40E-01	1.84	10.65	8.62E-01
cg13407464	-21.12	9.18	2.14E-02	-15.91	13.52	2.39E-01	-0.45	26.34	9.87E-01	-27.30	24.31	2.61E-01	-35.78	17.50	4.09E-02
cg14737571	-18.70	8.13	2.14E-02	-38.73	18.63	3.76E-02	-28.64	40.06	4.75E-01	-16.59	9.92	9.43E-02	10.16	26.06	6.97E-01

cg18378453	18.50	8.04	2.14E-02	52.87	29.73	7.54E-02	-36.64	70.05	6.01E-01	17.28	8.69	4.67E-02	5.57	33.59	8.68E-01
cg06365535	12.27	5.33	2.14E-02	12.21	9.60	2.03E-01	21.18	12.72	9.58E-02	12.10	12.90	3.48E-01	7.86	9.08	3.87E-01
cg07211050	-27.39	11.90	2.14E-02	-26.77	14.95	7.33E-02	56.14	212.48	7.92E-01	-44.36	22.91	5.28E-02	14.93	39.06	7.02E-01
cg00630958	15.29	6.65	2.14E-02	29.62	9.39	1.60E-03	14.28	14.20	3.15E-01	17.31	10.15	8.82E-02	1.45	8.38	8.63E-01
cg04637506	-10.82	4.70	2.14E-02	-15.96	11.71	1.73E-01	-8.62	9.73	3.75E-01	-6.48	7.18	3.67E-01	-19.51	11.17	8.08E-02
cg04872541	-8.31	3.61	2.14E-02	-8.43	5.38	1.17E-01	-18.09	8.28	2.89E-02	-4.24	9.80	6.66E-01	-2.22	7.62	7.71E-01
cg06757357	22.83	9.92	2.14E-02	25.91	16.61	1.19E-01	19.60	24.70	4.28E-01	19.05	18.64	3.07E-01	25.31	22.26	2.56E-01
cg17239008	4.04	1.75	2.14E-02	3.65	2.42	1.32E-01	4.47	3.32	1.79E-01	2.47	9.16	7.88E-01	4.92	4.39	2.62E-01
cg05726764	5.37	2.34	2.14E-02	3.82	3.28	2.44E-01	8.00	4.34	6.50E-02	16.66	9.50	7.94E-02	0.78	6.15	8.99E-01
cg25384556	-9.83	4.27	2.14E-02	-7.36	6.88	2.85E-01	-5.04	10.39	6.28E-01	-11.98	9.82	2.22E-01	-15.13	8.45	7.34E-02
cg11692852	19.32	8.40	2.14E-02	13.71	14.49	3.44E-01	54.21	25.00	3.02E-02	22.54	16.48	1.71E-01	9.42	15.55	5.45E-01
cg10982275	22.33	9.71	2.14E-02	6.70	17.49	7.02E-01	11.15	19.83	5.74E-01	38.03	17.66	3.13E-02	40.65	25.05	1.05E-01
cg15660684	-44.50	19.34	2.14E-02	-34.93	27.17	1.98E-01	-7.26	76.96	9.25E-01	-65.15	43.85	1.37E-01	-58.06	39.89	1.46E-01
cg06245989	10.48	4.55	2.14E-02	13.82	6.48	3.28E-02	17.77	11.97	1.38E-01	9.24	10.89	3.96E-01	-2.95	10.57	7.80E-01
cg11805463	-9.65	4.19	2.14E-02	-3.23	9.65	7.38E-01	-4.36	12.44	7.26E-01	-11.40	5.48	3.75E-02	-16.68	12.54	1.83E-01
cg03508063	14.05	6.11	2.14E-02	11.60	7.71	1.32E-01	32.91	12.56	8.81E-03	-0.88	13.38	9.48E-01	13.85	13.55	3.07E-01
cg08575249	16.89	7.34	2.14E-02	34.16	12.09	4.74E-03	9.20	12.86	4.74E-01	4.19	12.87	7.45E-01	19.62	20.59	3.41E-01
cg00785192	17.75	7.72	2.14E-02	17.22	15.26	2.59E-01	40.18	47.98	4.02E-01	19.01	9.72	5.06E-02	3.75	25.93	8.85E-01
cg20122925	21.93	9.53	2.14E-02	10.74	28.97	7.11E-01	17.84	58.00	7.58E-01	22.87	10.97	3.71E-02	27.47	28.76	3.39E-01
cg26885400	8.27	3.60	2.14E-02	5.81	4.98	2.43E-01	16.52	8.26	4.55E-02	16.32	9.87	9.82E-02	-0.33	9.06	9.71E-01
cg17566541	8.64	3.76	2.14E-02	9.46	4.84	5.03E-02	6.56	7.91	4.06E-01	16.66	12.58	1.85E-01	-0.47	13.15	9.72E-01
cg06080169	-12.67	5.51	2.14E-02	-14.50	10.04	1.49E-01	-0.97	13.82	9.44E-01	-15.37	9.14	9.28E-02	-14.54	13.10	2.67E-01
cg14338548	-20.22	8.79	2.14E-02	-46.65	22.19	3.55E-02	10.61	47.38	8.23E-01	-18.02	10.22	7.78E-02	1.24	33.80	9.71E-01
cg26696685	38.36	16.68	2.14E-02	37.05	19.70	6.00E-02	77.09	75.18	3.05E-01	47.89	49.59	3.34E-01	21.41	47.95	6.55E-01
cg09729166	19.35	8.41	2.14E-02	9.52	15.67	5.43E-01	22.59	16.85	1.80E-01	16.91	15.71	2.82E-01	34.93	20.10	8.23E-02
cg07375836	5.36	2.33	2.15E-02	4.05	3.36	2.28E-01	8.18	6.99	2.42E-01	9.02	4.48	4.40E-02	0.41	6.33	9.48E-01
cg02296931	10.62	4.62	2.15E-02	15.25	10.19	1.35E-01	31.77	55.22	5.65E-01	10.44	5.49	5.70E-02	-1.69	16.48	9.18E-01
cg01877565	18.14	7.89	2.15E-02	2.73	12.99	8.34E-01	29.99	13.43	2.56E-02	12.08	15.08	4.23E-01	38.93	20.75	6.06E-02
cg26282655	41.88	18.21	2.15E-02	35.47	25.73	1.68E-01	82.04	75.67	2.78E-01	79.54	51.88	1.25E-01	30.07	32.30	3.52E-01
cg13942782	-11.66	5.07	2.15E-02	-13.09	10.27	2.03E-01	-5.61	13.53	6.78E-01	-11.46	7.76	1.40E-01	-14.74	11.65	2.06E-01
cg12741645	-31.62	13.75	2.15E-02	-17.87	12.02	1.37E-01	-78.09	37.98	3.98E-02	2.68	45.88	9.53E-01	-51.94	23.68	2.83E-02
cg14932313	9.03	3.92	2.15E-02	11.87	5.24	2.35E-02	12.86	10.02	1.99E-01	-0.96	11.90	9.36E-01	2.80	9.35	7.65E-01
cg18098806	-18.29	7.95	2.15E-02	-33.23	12.93	1.02E-02	2.46	19.62	9.00E-01	-21.07	21.77	3.33E-01	-10.21	13.98	4.65E-01
cg24970879	-10.42	4.53	2.15E-02	-12.11	7.56	1.09E-01	-6.74	12.63	5.93E-01	-12.37	7.38	9.36E-02	-3.96	12.37	7.49E-01
cg00660400	-31.74	13.80	2.15E-02	-30.45	15.66	5.19E-02	-30.04	61.33	6.24E-01	-75.19	64.70	2.45E-01	-24.78	38.68	5.22E-01

cg24151926	-20.28	8.82	2.15E-02	-36.84	18.29	4.40E-02	-1.59	32.77	9.61E-01	-18.51	14.16	1.91E-01	-14.37	15.91	3.66E-01
cg12209693	9.79	4.26	2.15E-02	8.56	5.37	1.11E-01	18.77	8.20	2.20E-02	12.33	11.99	3.04E-01	-8.08	12.88	5.30E-01
cg21644856	14.36	6.25	2.15E-02	23.50	11.80	4.64E-02	-9.32	18.61	6.16E-01	25.33	16.56	1.26E-01	11.24	9.16	2.20E-01
cg05526832	-11.97	5.21	2.15E-02	-14.97	7.36	4.20E-02	-7.60	13.12	5.62E-01	-12.69	13.40	3.44E-01	-7.17	11.93	5.48E-01
cg07337250	-9.55	4.15	2.15E-02	-12.40	8.04	1.23E-01	-22.39	13.34	9.32E-02	-4.61	6.04	4.45E-01	-11.48	10.23	2.62E-01
cg13477101	9.50	4.13	2.15E-02	9.06	5.32	8.87E-02	9.28	10.69	3.85E-01	22.80	13.64	9.45E-02	3.60	10.44	7.30E-01
cg00838919	13.69	5.95	2.15E-02	8.33	9.66	3.89E-01	0.13	13.72	9.92E-01	17.89	19.72	3.64E-01	26.02	10.20	1.07E-02
cg23488607	11.66	5.07	2.15E-02	10.69	6.75	1.13E-01	22.34	11.22	4.65E-02	-12.50	17.51	4.75E-01	14.39	13.27	2.78E-01
cg09062038	-21.25	9.24	2.15E-02	-11.76	18.74	5.30E-01	-39.37	35.03	2.61E-01	-15.34	8.63	7.54E-02	-66.71	28.40	1.88E-02
cg13924807	-10.73	4.67	2.15E-02	-15.06	7.45	4.34E-02	-19.83	11.54	8.57E-02	1.37	9.46	8.85E-01	-9.51	10.39	3.60E-01
cg07583490	16.84	7.32	2.15E-02	26.83	12.09	2.65E-02	22.83	14.14	1.06E-01	8.25	15.70	5.99E-01	-6.32	19.09	7.41E-01
cg02349739	30.16	13.12	2.15E-02	46.54	18.04	9.89E-03	59.12	57.67	3.05E-01	8.80	9.72	3.65E-01	46.56	22.09	3.50E-02
cg08662533	32.74	14.24	2.15E-02	49.14	19.60	1.22E-02	24.56	31.98	4.42E-01	-35.88	50.98	4.82E-01	24.14	32.22	4.54E-01
cg07957953	-49.58	21.57	2.15E-02	-33.76	26.10	1.96E-01	-157.41	122.18	1.98E-01	-135.60	80.76	9.32E-02	-55.71	46.57	2.32E-01
cg10320160	-13.70	5.96	2.15E-02	-15.56	9.41	9.84E-02	-6.82	11.43	5.51E-01	-14.10	16.47	3.92E-01	-19.20	13.46	1.54E-01
cg07196637	-15.29	6.65	2.15E-02	-20.96	18.65	2.61E-01	-12.18	20.57	5.54E-01	-14.52	8.08	7.24E-02	-16.69	22.07	4.50E-01
cg26807301	37.89	16.48	2.15E-02	68.70	18.47	2.00E-04	57.65	26.99	3.27E-02	13.39	9.69	1.67E-01	18.15	30.32	5.49E-01
cg09120934	-12.61	5.49	2.15E-02	-16.80	7.73	2.97E-02	-8.14	13.21	5.38E-01	9.27	15.23	5.43E-01	-20.32	12.45	1.03E-01
cg08368934	7.88	3.43	2.15E-02	6.89	4.20	1.01E-01	13.28	9.06	1.43E-01	18.41	13.43	1.70E-01	1.55	9.62	8.72E-01
cg05701791	17.88	7.78	2.15E-02	37.60	14.09	7.65E-03	4.90	21.14	8.17E-01	17.15	12.27	1.62E-01	3.18	15.28	8.35E-01
cg05308819	16.11	7.01	2.15E-02	11.58	9.58	2.27E-01	16.69	15.35	2.77E-01	29.27	22.30	1.89E-01	22.41	17.61	2.03E-01
cg25463399	12.88	5.60	2.15E-02	3.28	10.32	7.50E-01	22.72	14.68	1.22E-01	17.12	8.14	3.56E-02	5.81	19.02	7.60E-01
cg17020726	-8.78	3.82	2.15E-02	-7.09	4.62	1.25E-01	-1.81	6.60	7.84E-01	-26.23	10.10	9.38E-03	-9.48	6.31	1.33E-01
cg17395064	20.91	9.10	2.15E-02	17.17	23.72	4.69E-01	-27.68	120.12	8.18E-01	25.09	10.23	1.42E-02	-22.96	38.26	5.48E-01
cg04292718	26.44	11.50	2.15E-02	42.66	31.86	1.81E-01	7.31	50.32	8.84E-01	23.72	13.89	8.79E-02	32.16	31.68	3.10E-01
cg14071014	-12.62	5.49	2.15E-02	-22.31	13.97	1.10E-01	-19.12	30.89	5.36E-01	-9.56	6.65	1.50E-01	-15.49	15.08	3.04E-01
cg22297934	19.62	8.54	2.15E-02	29.94	10.94	6.19E-03	3.79	19.66	8.47E-01	2.06	30.15	9.45E-01	4.11	24.43	8.66E-01
cg01244124	19.50	8.49	2.15E-02	1.23	29.16	9.66E-01	4.65	77.57	9.52E-01	22.71	9.24	1.40E-02	3.08	34.69	9.29E-01
cg10416593	8.52	3.71	2.15E-02	12.09	5.05	1.66E-02	10.51	8.26	2.03E-01	-0.51	11.17	9.63E-01	-0.38	9.61	9.68E-01
cg08930741	25.05	10.90	2.15E-02	28.11	21.06	1.82E-01	14.09	92.80	8.79E-01	28.20	15.29	6.51E-02	14.25	23.76	5.49E-01
cg00693599	17.49	7.61	2.15E-02	36.63	15.69	1.96E-02	1.81	16.10	9.11E-01	12.12	9.45	1.99E-01	36.59	24.43	1.34E-01
cg14662119	-49.08	21.35	2.15E-02	-26.60	34.69	4.43E-01	-78.44	138.25	5.70E-01	-57.94	30.45	5.70E-02	-81.85	65.77	2.13E-01
cg05332308	-5.73	2.49	2.15E-02	-7.33	3.72	4.90E-02	-5.48	5.94	3.55E-01	0.60	6.97	9.32E-01	-6.29	5.03	2.11E-01
cg18628787	-51.36	22.35	2.15E-02	-67.21	26.45	1.11E-02	3.32	150.61	9.82E-01	18.10	82.72	8.27E-01	-25.01	51.12	6.25E-01
cg25790850	-46.71	20.32	2.15E-02	-43.54	24.48	7.53E-02	-5.49	67.08	9.35E-01	27.46	70.93	6.99E-01	-101.66	43.27	1.88E-02

cg21804148	-23.76	10.34	2.15E-02	-11.15	26.21	6.70E-01	19.20	85.66	8.23E-01	-25.30	11.82	3.24E-02	-45.35	40.46	2.62E-01
cg10079318	-33.17	14.43	2.15E-02	-35.07	19.50	7.21E-02	-58.92	40.47	1.45E-01	-6.96	42.80	8.71E-01	-26.84	31.40	3.93E-01
cg09777416	-18.08	7.87	2.15E-02	-12.68	14.72	3.89E-01	21.37	27.50	4.37E-01	-31.85	40.19	4.28E-01	-25.22	10.20	1.35E-02
cg25469406	-11.90	5.18	2.16E-02	-12.14	10.01	2.25E-01	-12.45	13.31	3.49E-01	-18.63	8.17	2.25E-02	4.06	12.24	7.40E-01
cg24251218	-10.58	4.60	2.16E-02	-19.23	8.24	1.96E-02	-0.65	10.71	9.52E-01	-6.39	8.45	4.50E-01	-12.40	10.11	2.20E-01
cg26673436	31.01	13.49	2.16E-02	42.33	36.65	2.48E-01	168.94	175.89	3.37E-01	24.42	15.38	1.12E-01	61.81	45.36	1.73E-01
cg00078857	16.05	6.99	2.16E-02	11.00	10.73	3.05E-01	25.71	15.01	8.68E-02	25.60	15.26	9.34E-02	3.04	18.06	8.66E-01
cg20284239	8.68	3.78	2.16E-02	6.19	4.88	2.05E-01	17.25	8.90	5.27E-02	17.80	12.74	1.62E-01	2.26	10.34	8.27E-01
cg19360482	6.54	2.85	2.16E-02	4.46	4.15	2.83E-01	5.10	6.00	3.96E-01	14.44	10.68	1.76E-01	9.72	5.89	9.89E-02
cg06079106	17.66	7.69	2.16E-02	21.42	10.76	4.65E-02	29.03	20.96	1.66E-01	7.24	24.48	7.67E-01	8.24	15.18	5.87E-01
cg01557951	13.51	5.88	2.16E-02	8.72	9.14	3.40E-01	14.86	15.32	3.32E-01	13.06	13.04	3.17E-01	21.42	12.10	7.67E-02
cg10483525	15.52	6.76	2.16E-02	13.35	9.83	1.75E-01	23.00	15.01	1.25E-01	18.34	19.42	3.45E-01	11.45	14.95	4.44E-01
cg16730908	12.81	5.57	2.16E-02	19.01	8.88	3.23E-02	15.56	14.21	2.74E-01	5.60	11.18	6.17E-01	7.53	12.34	5.41E-01
cg12736501	7.80	3.39	2.16E-02	7.58	4.83	1.16E-01	15.11	8.61	7.92E-02	11.67	9.59	2.23E-01	1.08	7.14	8.80E-01
cg21042456	14.84	6.46	2.16E-02	36.25	20.30	7.42E-02	58.18	78.21	4.57E-01	13.91	7.22	5.42E-02	-3.69	21.18	8.62E-01
cg06758497	19.37	8.43	2.16E-02	-6.30	18.60	7.35E-01	39.83	45.08	3.77E-01	22.90	10.31	2.63E-02	43.63	28.04	1.20E-01
cg10118002	34.89	15.19	2.16E-02	-18.33	49.01	7.08E-01	4.75	170.85	9.78E-01	39.61	16.71	1.77E-02	55.53	57.44	3.34E-01
cg26246947	10.60	4.61	2.16E-02	11.26	5.99	6.03E-02	22.82	12.29	6.33E-02	9.45	13.51	4.84E-01	-2.62	11.92	8.26E-01
cg17279085	-27.45	11.95	2.16E-02	-8.92	21.86	6.83E-01	-57.73	54.63	2.91E-01	-28.70	16.96	9.07E-02	-49.44	30.11	1.01E-01
cg00582941	18.96	8.25	2.16E-02	27.74	15.20	6.81E-02	-8.57	21.45	6.90E-01	18.47	12.28	1.33E-01	35.21	25.42	1.66E-01
cg18269826	20.01	8.71	2.16E-02	6.32	19.18	7.42E-01	25.98	26.59	3.28E-01	19.44	12.03	1.06E-01	35.31	21.62	1.02E-01
cg10251594	11.15	4.85	2.16E-02	9.13	6.83	1.81E-01	23.95	11.40	3.57E-02	19.03	15.76	2.27E-01	1.80	10.38	8.62E-01
cg13560901	12.38	5.39	2.16E-02	4.42	8.01	5.81E-01	25.26	12.82	4.88E-02	7.30	13.50	5.89E-01	22.50	11.73	5.51E-02
cg12352601	20.43	8.89	2.16E-02	21.46	19.30	2.66E-01	39.20	23.90	1.01E-01	12.80	11.93	2.83E-01	35.55	29.06	2.21E-01
cg17332369	-36.58	15.93	2.16E-02	-52.31	24.41	3.21E-02	-23.84	49.48	6.30E-01	-31.76	32.77	3.32E-01	-18.50	32.90	5.74E-01
cg04904318	-54.75	23.84	2.16E-02	-52.05	29.74	8.01E-02	-1.22	121.64	9.92E-01	-43.16	80.57	5.92E-01	-75.51	49.53	1.27E-01
cg25189201	13.99	6.09	2.16E-02	25.51	14.30	7.43E-02	14.18	24.85	5.68E-01	9.06	7.37	2.19E-01	30.83	22.24	1.66E-01
cg07058998	9.96	4.33	2.16E-02	9.13	10.62	3.90E-01	-0.88	15.26	9.54E-01	10.74	5.24	4.03E-02	17.02	16.69	3.08E-01
cg24557917	-13.46	5.86	2.16E-02	-2.30	11.36	8.39E-01	-18.65	11.93	1.18E-01	-14.47	10.06	1.50E-01	-22.47	14.99	1.34E-01
cg09716921	-8.21	3.57	2.16E-02	-14.16	7.20	4.92E-02	-11.94	12.03	3.21E-01	-6.25	5.06	2.16E-01	-3.28	8.76	7.08E-01
cg13442606	-11.04	4.81	2.16E-02	-14.28	7.34	5.19E-02	-8.66	10.69	4.18E-01	-9.51	13.65	4.86E-01	-8.13	9.73	4.03E-01
cg00793181	18.19	7.92	2.16E-02	14.44	12.21	2.37E-01	24.16	15.78	1.26E-01	20.36	19.38	2.93E-01	16.40	19.81	4.08E-01
cg25558099	28.06	12.22	2.16E-02	28.82	14.35	4.47E-02	63.04	52.45	2.29E-01	-15.56	40.12	6.98E-01	40.47	34.08	2.35E-01
cg19881928	10.55	4.59	2.16E-02	10.93	9.93	2.71E-01	6.96	30.06	8.17E-01	13.57	5.90	2.13E-02	-1.19	11.61	9.18E-01
cg23892568	-12.10	5.27	2.16E-02	-18.37	9.34	4.93E-02	-9.32	14.17	5.11E-01	-15.77	6.02	8.86E-03	10.94	13.78	4.27E-01

cg18514820	-30.29	13.19	2.16E-02	-63.55	25.77	1.36E-02	-26.66	73.12	7.15E-01	-27.32	14.64	6.20E-02	13.29	34.69	7.02E-01
cg09070378	13.03	5.67	2.16E-02	11.54	9.16	2.08E-01	26.49	11.44	2.05E-02	8.60	11.20	4.42E-01	-1.11	16.84	9.47E-01
cg20398362	15.04	6.55	2.17E-02	17.34	9.41	6.52E-02	13.89	13.42	3.00E-01	20.72	20.19	3.05E-01	6.64	15.80	6.74E-01
cg12688240	7.08	3.08	2.17E-02	8.14	4.46	6.77E-02	8.11	6.69	2.25E-01	5.19	7.11	4.65E-01	4.01	8.88	6.51E-01
cg04578777	11.49	5.01	2.17E-02	20.37	12.71	1.09E-01	-12.03	23.18	6.04E-01	9.79	5.88	9.57E-02	24.46	18.47	1.85E-01
cg04155600	21.28	9.27	2.17E-02	23.04	17.44	1.87E-01	1.75	39.54	9.65E-01	20.10	12.73	1.14E-01	30.26	25.37	2.33E-01
cg20417351	8.92	3.89	2.17E-02	10.24	5.47	6.11E-02	7.47	9.43	4.28E-01	16.60	9.75	8.86E-02	-0.91	9.51	9.24E-01
cg03158666	-35.60	15.50	2.17E-02	-81.48	44.92	6.97E-02	-157.20	241.90	5.16E-01	-25.59	17.51	1.44E-01	-55.89	50.91	2.72E-01
cg23013850	9.19	4.00	2.17E-02	12.11	5.63	3.16E-02	10.65	9.02	2.38E-01	7.06	11.96	5.55E-01	0.97	9.31	9.17E-01
cg08871356	-17.64	7.68	2.17E-02	3.30	18.50	8.58E-01	11.13	35.12	7.51E-01	-25.38	9.22	5.91E-03	-13.13	26.42	6.19E-01
cg18988435	-9.83	4.28	2.17E-02	-14.69	6.41	2.19E-02	-1.14	9.32	9.03E-01	-22.40	11.58	5.31E-02	-3.68	7.49	6.24E-01
cg05091519	26.02	11.33	2.17E-02	33.72	18.99	7.57E-02	30.36	85.83	7.24E-01	23.62	16.20	1.45E-01	14.01	30.57	6.47E-01
cg25936876	14.19	6.18	2.17E-02	16.97	15.10	2.61E-01	20.29	24.01	3.98E-01	14.09	7.38	5.64E-02	1.97	24.27	9.35E-01
cg19126471	-39.74	17.31	2.17E-02	-49.53	24.69	4.48E-02	-12.47	35.97	7.29E-01	-29.09	50.04	5.61E-01	-57.34	43.62	1.89E-01
cg01477133	-12.78	5.57	2.17E-02	-20.20	8.75	2.09E-02	-11.71	12.93	3.65E-01	-19.59	15.25	1.99E-01	0.68	10.60	9.49E-01
cg15181393	-15.01	6.54	2.17E-02	-10.04	15.31	5.12E-01	1.30	25.63	9.59E-01	-18.62	8.02	2.03E-02	-10.16	21.94	6.43E-01
cg15734454	-10.61	4.62	2.17E-02	-5.15	12.54	6.81E-01	-16.63	23.17	4.73E-01	-12.88	5.43	1.76E-02	0.95	14.67	9.49E-01
cg12168275	-24.24	10.56	2.17E-02	-14.73	16.06	3.59E-01	-22.44	24.78	3.65E-01	-23.92	23.39	3.06E-01	-48.87	24.70	4.79E-02
cg03618113	8.26	3.60	2.17E-02	6.93	6.02	2.50E-01	8.19	7.11	2.49E-01	15.87	7.52	3.48E-02	0.32	9.07	9.72E-01
cg20676580	15.72	6.85	2.17E-02	3.25	11.07	7.69E-01	27.29	11.79	2.07E-02	7.38	18.00	6.82E-01	29.27	17.59	9.60E-02
cg04212081	20.74	9.04	2.17E-02	34.69	18.49	6.07E-02	25.32	18.16	1.63E-01	3.79	14.58	7.95E-01	36.61	25.12	1.45E-01
cg10236247	-11.59	5.05	2.17E-02	-23.32	12.71	6.64E-02	-17.84	21.11	3.98E-01	-6.42	5.97	2.82E-01	-32.95	19.14	8.52E-02
cg10717074	31.81	13.86	2.17E-02	29.86	17.37	8.55E-02	-33.31	78.91	6.73E-01	94.38	46.62	4.29E-02	22.46	28.05	4.23E-01
cg06585307	14.11	6.15	2.17E-02	12.70	14.80	3.91E-01	32.03	21.09	1.29E-01	14.13	7.45	5.80E-02	-6.61	24.57	7.88E-01
cg13455623	-40.80	17.78	2.17E-02	-20.96	25.37	4.09E-01	53.04	121.84	6.63E-01	-84.31	36.40	2.06E-02	-46.30	35.61	1.94E-01
cg11700959	-10.02	4.36	2.17E-02	-8.50	5.67	1.34E-01	-16.96	11.07	1.26E-01	-4.29	12.67	7.35E-01	-13.75	11.95	2.50E-01
cg19223782	8.08	3.52	2.17E-02	8.95	4.41	4.23E-02	14.86	9.80	1.29E-01	0.80	10.25	9.38E-01	3.12	10.37	7.63E-01
cg27292870	17.08	7.44	2.17E-02	38.23	15.68	1.48E-02	18.76	29.69	5.28E-01	12.02	9.60	2.11E-01	0.62	22.33	9.78E-01
cg06961122	17.31	7.54	2.17E-02	15.51	20.64	4.52E-01	12.66	29.86	6.71E-01	17.65	9.23	5.60E-02	19.60	20.48	3.38E-01
cg05893300	33.29	14.50	2.17E-02	33.75	18.08	6.19E-02	37.93	55.32	4.93E-01	73.92	69.15	2.85E-01	23.42	29.39	4.26E-01
cg17036419	-11.31	4.93	2.17E-02	-11.53	8.41	1.70E-01	-6.49	18.75	7.29E-01	-10.45	7.28	1.51E-01	-16.34	13.69	2.33E-01
cg03788239	-10.83	4.72	2.17E-02	-15.32	6.66	2.15E-02	-8.56	11.10	4.40E-01	1.39	14.19	9.22E-01	-8.47	10.37	4.14E-01
cg26014796	21.78	9.49	2.17E-02	27.65	13.62	4.24E-02	34.51	28.19	2.21E-01	13.44	23.34	5.65E-01	9.45	19.52	6.28E-01
cg18078170	14.15	6.16	2.17E-02	23.10	14.93	1.22E-01	15.27	15.92	3.38E-01	10.27	7.89	1.93E-01	23.95	23.49	3.08E-01
cg00633473	29.13	12.69	2.17E-02	40.13	15.58	1.00E-02	17.92	58.76	7.60E-01	-15.29	66.62	8.18E-01	8.73	25.23	7.29E-01

cg17514528	7.31	3.19	2.18E-02	11.05	4.58	1.59E-02	5.13	6.26	4.12E-01	5.63	11.72	6.31E-01	1.22	7.43	8.70E-01
cg25182206	-16.54	7.21	2.18E-02	-15.88	11.00	1.49E-01	-6.81	19.05	7.21E-01	-32.90	16.25	4.28E-02	-9.84	15.02	5.12E-01
cg16509531	-10.65	4.64	2.18E-02	-13.15	10.61	2.15E-01	-13.66	16.21	3.99E-01	-8.30	5.87	1.57E-01	-18.09	14.63	2.16E-01
cg01620165	8.69	3.79	2.18E-02	9.91	5.28	6.05E-02	13.05	9.39	1.65E-01	2.23	10.14	8.26E-01	6.33	8.87	4.75E-01
cg04309849	7.03	3.06	2.18E-02	2.99	4.39	4.95E-01	7.14	7.23	3.23E-01	17.84	8.40	3.36E-02	9.57	6.85	1.62E-01
cg02853355	6.21	2.71	2.18E-02	5.63	3.80	1.38E-01	10.62	6.82	1.19E-01	7.78	5.84	1.83E-01	0.10	7.78	9.90E-01
cg00163365	18.80	8.19	2.18E-02	18.15	10.43	8.19E-02	26.13	23.12	2.58E-01	37.80	38.30	3.24E-01	12.24	17.79	4.92E-01
cg19203065	-17.65	7.69	2.18E-02	-15.64	17.65	3.76E-01	5.32	27.23	8.45E-01	-20.37	9.75	3.68E-02	-22.46	23.35	3.36E-01
cg06986158	22.02	9.60	2.18E-02	36.38	20.46	7.53E-02	-2.85	39.53	9.43E-01	20.07	12.08	9.66E-02	16.84	32.11	6.00E-01
cg07618101	20.58	8.97	2.18E-02	14.33	16.60	3.88E-01	9.66	25.71	7.07E-01	24.03	13.26	6.99E-02	32.77	25.01	1.90E-01
cg13547889	-10.71	4.67	2.18E-02	-9.25	6.77	1.72E-01	-7.53	13.18	5.68E-01	-19.28	9.40	4.04E-02	-4.05	11.92	7.34E-01
cg17366563	12.43	5.42	2.18E-02	15.93	9.43	9.12E-02	17.70	12.74	1.65E-01	11.02	8.58	1.99E-01	-4.70	18.05	7.95E-01
cg26939277	-5.41	2.36	2.18E-02	-3.06	3.57	3.91E-01	-3.44	5.12	5.02E-01	-12.37	5.87	3.51E-02	-7.07	5.40	1.90E-01
cg11049863	20.96	9.14	2.18E-02	19.88	18.23	2.76E-01	1.96	18.23	9.15E-01	30.57	15.49	4.84E-02	32.29	23.61	1.71E-01
cg26432350	10.01	4.36	2.18E-02	12.80	5.63	2.29E-02	18.70	12.12	1.23E-01	4.18	13.00	7.48E-01	-3.73	11.02	7.35E-01
cg06839449	-11.54	5.03	2.18E-02	-0.81	13.27	9.51E-01	-12.30	20.48	5.48E-01	-14.63	5.85	1.23E-02	2.64	21.22	9.01E-01
cg09391949	24.08	10.50	2.18E-02	42.19	16.06	8.63E-03	11.94	19.26	5.35E-01	26.47	31.10	3.95E-01	-3.07	26.06	9.06E-01
cg23834919	-10.05	4.38	2.18E-02	-22.25	11.48	5.27E-02	-8.85	17.69	6.17E-01	-6.99	5.28	1.86E-01	-13.85	13.46	3.03E-01
cg24876960	17.32	7.55	2.18E-02	25.55	25.00	3.07E-01	53.83	64.24	4.02E-01	19.95	8.53	1.93E-02	-12.69	22.71	5.76E-01
cg19539182	20.45	8.92	2.18E-02	48.09	20.24	1.75E-02	31.47	23.95	1.89E-01	12.78	10.40	2.19E-01	-2.75	30.60	9.28E-01
cg00587466	-39.50	17.22	2.18E-02	-33.69	22.19	1.29E-01	39.45	62.79	5.30E-01	-47.28	75.45	5.31E-01	-72.89	33.11	2.77E-02
cg08468082	-15.06	6.56	2.18E-02	-11.33	12.14	3.51E-01	-16.03	13.51	2.36E-01	-16.45	12.26	1.80E-01	-17.56	15.26	2.50E-01
cg08443019	21.94	9.57	2.18E-02	26.79	19.03	1.59E-01	28.72	19.36	1.38E-01	7.96	16.25	6.24E-01	34.51	24.18	1.54E-01
cg03055065	9.87	4.30	2.18E-02	5.37	6.68	4.21E-01	18.73	9.47	4.80E-02	13.85	12.18	2.55E-01	8.06	8.54	3.45E-01
cg15820432	-10.34	4.51	2.18E-02	-10.67	10.95	3.30E-01	1.62	16.49	9.22E-01	-11.90	5.45	2.92E-02	-7.20	16.66	6.65E-01
cg05813650	19.67	8.58	2.18E-02	32.02	16.30	4.94E-02	17.72	18.94	3.50E-01	11.43	13.32	3.91E-01	23.53	26.71	3.78E-01
cg02102075	5.71	2.49	2.18E-02	3.96	3.27	2.26E-01	12.12	5.68	3.30E-02	5.80	7.30	4.27E-01	3.70	7.43	6.18E-01
cg02490718	16.43	7.17	2.18E-02	33.43	18.79	7.52E-02	-23.06	40.96	5.73E-01	15.44	8.29	6.25E-02	9.61	25.89	7.10E-01
cg09141303	-5.91	2.58	2.18E-02	-2.00	4.33	6.44E-01	-4.02	5.96	4.99E-01	-8.29	4.47	6.37E-02	-13.44	7.26	6.43E-02
cg03167413	27.67	12.07	2.18E-02	34.81	14.50	1.63E-02	-9.01	59.68	8.80E-01	38.46	33.75	2.54E-01	-7.20	32.43	8.24E-01
cg02006915	-11.33	4.94	2.18E-02	-12.44	9.43	1.87E-01	-2.54	12.48	8.39E-01	-14.19	8.02	7.66E-02	-11.27	11.40	3.23E-01
cg17608706	20.01	8.73	2.18E-02	40.67	16.99	1.67E-02	2.68	16.85	8.74E-01	14.60	14.80	3.24E-01	28.97	25.15	2.49E-01
cg26308042	-14.97	6.53	2.18E-02	-35.21	16.11	2.89E-02	-9.84	21.25	6.43E-01	-8.90	8.21	2.79E-01	-24.18	19.77	2.21E-01
cg15475101	12.72	5.55	2.19E-02	3.28	12.49	7.93E-01	-15.50	25.22	5.39E-01	17.15	6.71	1.06E-02	15.46	20.80	4.57E-01
cg02656891	11.42	4.98	2.19E-02	12.83	11.03	2.45E-01	15.04	11.95	2.08E-01	14.36	8.86	1.05E-01	5.38	9.01	5.50E-01

cg05165940	16.26	7.09	2.19E-02	24.47	13.89	7.81E-02	11.91	14.59	4.14E-01	18.52	13.79	1.79E-01	9.10	14.51	5.31E-01
cg06160973	9.62	4.20	2.19E-02	12.96	6.03	3.15E-02	9.78	9.76	3.16E-01	6.39	10.86	5.56E-01	3.17	9.86	7.48E-01
cg13412066	17.33	7.56	2.19E-02	-14.60	31.90	6.47E-01	-67.34	130.11	6.05E-01	20.99	7.99	8.60E-03	-9.47	35.81	7.91E-01
cg18792381	12.43	5.42	2.19E-02	12.08	7.84	1.23E-01	16.67	14.91	2.64E-01	0.89	20.04	9.65E-01	13.84	9.63	1.51E-01
cg14349763	-11.90	5.19	2.19E-02	-18.44	7.58	1.50E-02	-8.01	14.55	5.82E-01	1.34	11.91	9.10E-01	-11.64	11.24	3.00E-01
cg12570134	-10.79	4.71	2.19E-02	-17.47	11.48	1.28E-01	5.04	16.04	7.53E-01	-11.95	5.78	3.88E-02	-4.47	16.27	7.83E-01
cg19653212	13.93	6.08	2.19E-02	19.88	12.91	1.24E-01	3.64	23.15	8.75E-01	11.19	8.22	1.73E-01	19.41	15.06	1.98E-01
cg14857193	-12.88	5.62	2.19E-02	-17.12	8.08	3.42E-02	-5.95	13.59	6.62E-01	-23.81	16.18	1.41E-01	-3.22	11.82	7.85E-01
cg02007217	-13.62	5.94	2.19E-02	-16.31	9.97	1.02E-01	0.99	12.78	9.38E-01	-9.40	6.01	1.18E-01	-33.92	12.36	6.07E-03
cg27160537	24.88	10.85	2.19E-02	32.56	16.53	4.88E-02	51.21	21.24	1.59E-02	0.19	18.66	9.92E-01	15.86	24.08	5.10E-01
cg02246876	10.09	4.40	2.19E-02	10.56	6.63	1.11E-01	18.36	9.16	4.51E-02	10.47	10.17	3.03E-01	-5.36	11.70	6.47E-01
cg18374914	-14.49	6.32	2.19E-02	-4.08	12.91	7.52E-01	-11.32	16.24	4.86E-01	-12.14	8.02	1.30E-01	-39.33	15.50	1.12E-02
cg21493633	19.72	8.60	2.19E-02	35.17	13.99	1.19E-02	10.65	26.73	6.90E-01	23.02	22.30	3.02E-01	5.11	14.16	7.18E-01
cg14957089	-7.02	3.06	2.19E-02	-5.62	3.98	1.58E-01	-7.93	7.57	2.95E-01	-10.20	8.98	2.56E-01	-9.40	8.53	2.70E-01
cg18101022	15.22	6.64	2.19E-02	14.73	13.26	2.67E-01	13.57	20.80	5.14E-01	13.88	10.35	1.80E-01	18.82	13.69	1.69E-01
cg06092948	14.02	6.11	2.19E-02	11.85	7.71	1.24E-01	8.49	27.46	7.57E-01	-13.76	64.99	8.32E-01	20.01	10.92	6.70E-02
cg17306401	-13.60	5.93	2.19E-02	-17.44	8.93	5.07E-02	-9.85	13.93	4.80E-01	-22.49	17.63	2.02E-01	-5.92	11.55	6.08E-01
cg09599130	17.05	7.44	2.19E-02	14.87	14.41	3.02E-01	22.89	18.82	2.24E-01	23.65	13.83	8.73E-02	9.27	13.87	5.04E-01
cg08137040	6.63	2.89	2.19E-02	8.48	4.09	3.83E-02	5.99	6.14	3.29E-01	-4.43	9.84	6.52E-01	7.52	6.59	2.53E-01
cg15556990	-10.11	4.41	2.19E-02	-23.87	11.35	3.54E-02	5.38	13.38	6.87E-01	-10.17	5.45	6.20E-02	-4.99	15.07	7.40E-01
cg20013688	-13.78	6.01	2.19E-02	-25.63	9.60	7.59E-03	-2.53	13.73	8.54E-01	-2.12	11.73	8.56E-01	-16.96	12.16	1.63E-01
cg16173280	18.38	8.02	2.19E-02	16.51	18.51	3.73E-01	10.39	21.22	6.24E-01	21.52	10.65	4.33E-02	15.57	25.11	5.35E-01
cg26213224	-13.44	5.87	2.19E-02	-14.01	9.31	1.32E-01	-11.33	13.64	4.06E-01	-12.72	13.21	3.35E-01	-14.84	12.50	2.35E-01
cg18345826	31.42	13.71	2.19E-02	40.06	19.80	4.30E-02	8.96	108.68	9.34E-01	27.30	34.85	4.33E-01	22.41	23.19	3.34E-01
cg11325716	-15.13	6.60	2.19E-02	-16.88	12.62	1.81E-01	-28.97	16.33	7.60E-02	-9.05	10.37	3.83E-01	-13.35	16.64	4.22E-01
cg00153106	26.78	11.68	2.19E-02	26.24	16.64	1.15E-01	2.76	31.30	9.30E-01	60.60	30.02	4.35E-02	19.77	25.13	4.32E-01
cg06315217	10.81	4.72	2.19E-02	11.98	6.63	7.07E-02	16.40	11.19	1.43E-01	8.95	11.33	4.30E-01	1.90	12.55	8.80E-01
cg25736517	-19.38	8.46	2.19E-02	-39.31	22.60	8.20E-02	28.40	36.45	4.36E-01	-20.93	9.84	3.33E-02	0.98	32.60	9.76E-01
cg24166018	-12.07	5.27	2.19E-02	-15.21	9.20	9.82E-02	-26.64	11.62	2.19E-02	-4.64	9.66	6.31E-01	-1.39	12.77	9.13E-01
cg06215457	16.90	7.38	2.20E-02	25.60	11.57	2.70E-02	1.75	21.22	9.34E-01	25.25	19.31	1.91E-01	7.98	12.90	5.36E-01
cg18596794	-13.74	6.00	2.20E-02	-9.98	17.37	5.66E-01	27.94	32.24	3.86E-01	-16.44	6.77	1.52E-02	-9.99	24.14	6.79E-01
cg17307348	23.98	10.47	2.20E-02	-4.51	19.24	8.15E-01	89.32	76.60	2.44E-01	28.30	8.99	1.65E-03	43.41	25.53	8.91E-02
cg25298725	-13.54	5.91	2.20E-02	-18.96	9.23	4.00E-02	-6.38	13.80	6.44E-01	-0.73	14.58	9.60E-01	-18.45	11.99	1.24E-01
cg12813768	-6.49	2.83	2.20E-02	-9.56	4.21	2.32E-02	-1.70	5.19	7.43E-01	9.14	13.92	5.11E-01	-9.75	6.19	1.16E-01
cg24644605	-11.24	4.90	2.20E-02	-12.25	9.27	1.86E-01	-10.21	13.37	4.45E-01	-9.02	7.99	2.59E-01	-14.54	10.73	1.75E-01

cg03209412	5.39	2.35	2.20E-02	1.34	3.17	6.72E-01	10.23	5.48	6.16E-02	7.66	6.76	2.57E-01	9.35	4.94	5.82E-02
cg22651416	18.93	8.26	2.20E-02	12.28	18.74	5.12E-01	-0.82	28.47	9.77E-01	20.10	10.49	5.54E-02	41.16	26.05	1.14E-01
cg09985344	-8.61	3.76	2.20E-02	-7.68	5.92	1.95E-01	-13.13	8.15	1.07E-01	-6.42	9.64	5.05E-01	-7.50	7.78	3.35E-01
cg13944175	11.67	5.10	2.20E-02	22.99	9.22	1.27E-02	14.17	10.19	1.64E-01	14.90	7.76	5.48E-02	0.85	6.17	8.91E-01
cg13863204	-19.30	8.43	2.20E-02	-20.61	24.27	3.96E-01	4.68	28.87	8.71E-01	-23.19	9.85	1.85E-02	-3.80	33.84	9.11E-01
cg00199549	-11.53	5.03	2.20E-02	-13.12	14.93	3.80E-01	-3.49	22.27	8.75E-01	-12.90	5.79	2.58E-02	-1.35	17.89	9.40E-01
cg25857090	14.03	6.12	2.20E-02	7.61	13.26	5.66E-01	-0.89	46.44	9.85E-01	16.60	7.33	2.35E-02	11.62	22.98	6.13E-01
cg12665059	-11.63	5.08	2.20E-02	-23.24	14.20	1.02E-01	-10.41	18.40	5.72E-01	-8.82	6.07	1.46E-01	-17.64	16.38	2.82E-01
cg01617211	-12.43	5.42	2.20E-02	-14.77	12.03	2.19E-01	-10.01	17.26	5.62E-01	-10.50	7.31	1.51E-01	-17.98	14.12	2.03E-01
cg10313879	-49.33	21.54	2.20E-02	-37.22	26.73	1.64E-01	67.81	155.56	6.63E-01	-118.12	58.39	4.31E-02	-53.14	48.68	2.75E-01
cg10897223	8.54	3.73	2.20E-02	9.88	5.06	5.09E-02	8.90	9.55	3.52E-01	11.11	9.92	2.63E-01	1.55	9.22	8.67E-01
cg01565508	6.56	2.86	2.20E-02	5.82	3.62	1.08E-01	12.69	7.15	7.58E-02	2.02	9.13	8.25E-01	5.88	8.39	4.83E-01
cg16809304	-19.63	8.57	2.20E-02	-24.47	16.10	1.28E-01	10.40	19.92	6.02E-01	-22.21	17.06	1.93E-01	-32.30	16.22	4.65E-02
cg06006907	-13.20	5.76	2.20E-02	-23.57	7.26	1.17E-03	-10.59	11.95	3.76E-01	3.88	14.33	7.86E-01	-7.70	10.35	4.57E-01
cg09254142	21.62	9.44	2.20E-02	-21.70	27.18	4.25E-01	27.66	80.80	7.32E-01	28.18	11.64	1.55E-02	25.62	20.68	2.15E-01
cg05254518	25.81	11.27	2.20E-02	36.70	14.84	1.34E-02	32.07	51.05	5.30E-01	58.88	47.91	2.19E-01	-0.55	19.94	9.78E-01
cg12272104	16.37	7.15	2.20E-02	16.64	12.23	1.74E-01	22.07	14.48	1.28E-01	9.90	13.20	4.53E-01	19.75	20.44	3.34E-01
cg17948846	-23.67	10.33	2.20E-02	-21.63	12.80	9.11E-02	-24.36	29.22	4.04E-01	-18.68	35.44	5.98E-01	-35.75	27.81	1.99E-01
cg05928873	-11.15	4.87	2.20E-02	-23.61	7.18	1.00E-03	-6.24	14.75	6.73E-01	-5.83	4.11	1.56E-01	-8.09	10.22	4.29E-01
cg01501406	19.60	8.56	2.20E-02	8.00	14.79	5.89E-01	21.27	14.62	1.46E-01	30.31	23.18	1.91E-01	29.54	19.82	1.36E-01
cg00118229	-15.46	6.75	2.20E-02	-30.13	11.05	6.40E-03	-5.61	15.68	7.21E-01	-3.71	17.67	8.34E-01	-8.92	12.42	4.73E-01
cg16124935	-20.86	9.11	2.20E-02	-12.76	13.70	3.52E-01	2.50	32.38	9.38E-01	-32.68	27.47	2.34E-01	-32.05	14.99	3.26E-02
cg16458196	16.34	7.14	2.20E-02	16.21	14.01	2.47E-01	12.66	17.41	4.67E-01	14.51	14.11	3.04E-01	19.88	12.68	1.17E-01
cg02021919	4.73	2.06	2.20E-02	4.50	3.02	1.36E-01	6.70	5.36	2.11E-01	3.98	3.83	2.99E-01	5.11	6.77	4.51E-01
cg16735986	15.43	6.74	2.20E-02	11.97	11.31	2.90E-01	22.38	18.79	2.34E-01	21.72	14.79	1.42E-01	12.31	12.13	3.10E-01
cg09526022	15.29	6.68	2.20E-02	43.58	18.23	1.68E-02	1.89	23.33	9.36E-01	11.89	7.82	1.28E-01	11.42	28.62	6.90E-01
cg13390630	24.75	10.81	2.20E-02	49.70	25.90	5.50E-02	-4.92	47.31	9.17E-01	27.32	13.38	4.12E-02	-11.97	30.96	6.99E-01
cg24191821	-46.90	20.48	2.20E-02	-76.58	37.17	3.94E-02	109.36	131.21	4.05E-01	-47.15	32.61	1.48E-01	-27.79	38.86	4.74E-01
cg19546057	9.76	4.26	2.20E-02	10.30	6.13	9.30E-02	15.01	11.00	1.72E-01	11.51	9.48	2.25E-01	1.26	10.49	9.05E-01
cg24681895	27.77	12.13	2.20E-02	24.94	15.22	1.01E-01	19.06	35.10	5.87E-01	-19.91	76.35	7.94E-01	46.10	25.83	7.42E-02
cg23805884	-31.65	13.82	2.20E-02	-27.06	15.25	7.61E-02	-45.25	70.63	5.22E-01	9.82	63.08	8.76E-01	-87.90	45.29	5.23E-02
cg08660395	19.59	8.56	2.20E-02	46.41	16.91	6.07E-03	9.52	18.29	6.03E-01	11.11	11.16	3.20E-01	17.60	23.73	4.58E-01
cg26263239	-13.83	6.04	2.20E-02	-19.90	9.37	3.37E-02	-8.88	14.04	5.27E-01	-9.64	18.58	6.04E-01	-9.88	11.15	3.75E-01
cg06044004	-6.85	2.99	2.20E-02	-10.02	5.26	5.69E-02	-6.38	7.85	4.17E-01	-4.93	4.76	3.00E-01	-5.42	8.11	5.04E-01
cg00150882	15.42	6.73	2.20E-02	24.92	8.39	2.98E-03	18.85	13.67	1.68E-01	22.56	15.09	1.35E-01	0.07	8.78	9.94E-01

cg15352185	-47.21	20.62	2.20E-02	-69.46	39.06	7.54E-02	-32.93	115.11	7.75E-01	-21.95	20.31	2.80E-01	-113.76	49.87	2.25E-02
cg02388500	17.35	7.58	2.20E-02	9.64	23.36	6.80E-01	-23.60	38.27	5.37E-01	19.88	8.57	2.04E-02	23.37	27.85	4.01E-01
cg17921080	-8.60	3.76	2.20E-02	-9.74	9.32	2.96E-01	3.99	16.02	8.04E-01	-8.58	4.49	5.62E-02	-14.91	13.03	2.52E-01
cg00901878	-11.52	5.03	2.20E-02	-4.43	12.54	7.24E-01	-1.27	13.15	9.23E-01	-13.44	6.45	3.71E-02	-29.11	17.39	9.41E-02
cg00715109	11.52	5.03	2.21E-02	12.54	5.90	3.35E-02	7.24	20.41	7.23E-01	-21.51	28.59	4.52E-01	14.49	11.82	2.20E-01
cg12895304	29.30	12.80	2.21E-02	15.49	21.85	4.78E-01	57.00	51.77	2.71E-01	19.32	13.02	1.38E-01	78.47	30.98	1.13E-02
cg26607031	5.96	2.60	2.21E-02	4.52	3.89	2.46E-01	9.89	5.81	8.89E-02	8.20	5.45	1.32E-01	0.67	7.38	9.28E-01
cg00959431	18.31	8.00	2.21E-02	31.00	13.58	2.25E-02	22.52	22.21	3.10E-01	8.77	17.86	6.24E-01	8.91	14.07	5.27E-01
cg05170183	-8.92	3.89	2.21E-02	-16.75	9.98	9.32E-02	-26.78	18.10	1.39E-01	-6.30	4.73	1.83E-01	-6.92	11.04	5.30E-01
cg00293940	10.11	4.42	2.21E-02	11.88	6.90	8.55E-02	7.18	9.30	4.40E-01	6.29	9.16	4.92E-01	16.33	12.11	1.78E-01
cg27226618	10.71	4.68	2.21E-02	6.27	9.82	5.23E-01	19.88	19.84	3.16E-01	10.61	6.02	7.82E-02	15.53	13.80	2.60E-01
cg24713490	20.62	9.01	2.21E-02	12.94	12.96	3.18E-01	23.06	24.91	3.55E-01	26.84	22.65	2.36E-01	31.19	18.86	9.82E-02
cg10959773	-31.44	13.73	2.21E-02	-27.20	18.04	1.32E-01	-82.31	41.86	4.93E-02	-26.44	39.50	5.03E-01	-18.87	31.33	5.47E-01
cg19522262	-9.10	3.97	2.21E-02	-13.51	8.72	1.21E-01	-8.32	7.21	2.49E-01	-10.26	6.49	1.14E-01	0.70	11.78	9.53E-01
cg18922874	17.40	7.60	2.21E-02	18.06	22.25	4.17E-01	31.75	26.46	2.30E-01	14.93	8.84	9.13E-02	26.70	30.74	3.85E-01
cg01819512	13.54	5.91	2.21E-02	18.36	9.77	6.02E-02	23.58	17.99	1.90E-01	6.82	10.58	5.19E-01	9.98	12.81	4.36E-01
cg16272786	-45.58	19.91	2.21E-02	-23.30	38.05	5.40E-01	-172.69	210.57	4.12E-01	-53.42	27.27	5.01E-02	-49.87	46.44	2.83E-01
cg20793087	-48.73	21.29	2.21E-02	-66.94	27.14	1.36E-02	-42.56	64.09	5.07E-01	17.17	87.70	8.45E-01	-17.90	45.89	6.97E-01
cg10441070	-8.51	3.72	2.21E-02	-13.53	10.44	1.95E-01	-10.24	18.01	5.69E-01	-6.12	4.45	1.69E-01	-15.54	10.13	1.25E-01
cg11143486	-14.36	6.27	2.21E-02	-9.73	8.37	2.45E-01	-31.26	12.32	1.11E-02	6.89	21.29	7.46E-01	-14.16	14.32	3.23E-01
cg01046673	18.13	7.92	2.21E-02	54.01	28.78	6.05E-02	26.29	100.96	7.95E-01	15.33	8.48	7.06E-02	10.89	36.93	7.68E-01
cg15617537	-5.77	2.52	2.21E-02	-8.01	3.98	4.40E-02	2.55	8.60	7.66E-01	-7.65	4.16	6.62E-02	0.26	6.62	9.69E-01
cg24432245	8.44	3.69	2.21E-02	9.31	5.42	8.55E-02	8.52	9.12	3.50E-01	7.84	9.31	4.00E-01	6.96	7.95	3.82E-01
cg20447730	-8.05	3.52	2.21E-02	-8.27	4.60	7.24E-02	-11.65	9.20	2.05E-01	-14.69	9.25	1.12E-01	4.90	9.97	6.23E-01
cg04720596	16.48	7.20	2.21E-02	20.78	12.73	1.03E-01	13.15	12.24	2.82E-01	23.68	16.96	1.63E-01	6.58	18.40	7.20E-01
cg01799015	8.50	3.71	2.21E-02	9.79	4.88	4.47E-02	11.08	8.57	1.96E-01	-0.30	12.26	9.80E-01	5.46	9.87	5.80E-01
cg13722700	9.06	3.96	2.21E-02	15.27	7.50	4.18E-02	2.36	8.91	7.91E-01	4.69	6.92	4.98E-01	14.25	8.91	1.10E-01
cg13188795	-10.16	4.44	2.21E-02	-14.69	7.97	6.54E-02	5.96	17.57	7.34E-01	-5.91	6.14	3.35E-01	-25.18	12.76	4.84E-02
cg17631429	16.91	7.39	2.21E-02	12.74	17.83	4.75E-01	-3.30	38.36	9.31E-01	14.90	8.78	8.98E-02	51.95	25.71	4.33E-02
cg08630080	-15.26	6.67	2.21E-02	-20.89	21.08	3.22E-01	-4.44	39.11	9.10E-01	-16.83	7.53	2.55E-02	1.76	22.62	9.38E-01
cg23502253	11.29	4.93	2.21E-02	6.11	9.74	5.30E-01	10.11	11.93	3.97E-01	14.64	7.07	3.84E-02	10.12	16.86	5.48E-01
cg12285565	9.32	4.07	2.21E-02	8.60	6.62	1.94E-01	1.67	9.32	8.58E-01	9.06	9.06	3.18E-01	17.14	8.53	4.44E-02
cg06798483	-10.46	4.57	2.21E-02	-13.37	6.53	4.07E-02	-8.80	12.06	4.66E-01	-0.01	14.74	9.99E-01	-9.79	8.79	2.65E-01
cg15790037	33.79	14.77	2.21E-02	50.46	39.68	2.03E-01	-33.57	90.87	7.12E-01	29.94	17.04	7.89E-02	61.91	50.95	2.24E-01
cg07632488	18.78	8.21	2.21E-02	33.11	21.92	1.31E-01	43.33	37.32	2.46E-01	17.59	9.50	6.42E-02	-16.49	32.11	6.08E-01

cg14544831	-9.08	3.97	2.21E-02	-11.63	5.37	3.05E-02	-14.16	9.83	1.50E-01	1.95	13.52	8.85E-01	-2.92	8.74	7.38E-01
cg25122395	15.18	6.63	2.21E-02	6.47	6.00	2.81E-01	5.71	11.53	6.21E-01	37.56	11.51	1.10E-03	17.00	8.86	5.49E-02
cg04323069	-42.05	18.38	2.21E-02	-94.09	45.55	3.88E-02	-109.92	155.55	4.80E-01	-37.74	21.46	7.86E-02	27.75	61.36	6.51E-01
cg02580045	-13.37	5.85	2.21E-02	-19.22	9.52	4.35E-02	-15.75	13.11	2.30E-01	-9.33	14.17	5.11E-01	-5.55	11.60	6.32E-01
cg12548634	15.52	6.78	2.21E-02	16.87	9.21	6.69E-02	26.43	37.05	4.76E-01	26.69	28.74	3.53E-01	10.84	11.18	3.32E-01
cg04096150	-8.27	3.61	2.21E-02	-14.48	9.29	1.19E-01	-21.29	14.41	1.39E-01	-5.04	4.50	2.63E-01	-10.51	9.60	2.74E-01
cg26586118	14.51	6.34	2.21E-02	19.06	12.51	1.27E-01	0.72	23.08	9.75E-01	10.35	6.42	1.07E-01	51.22	23.34	2.82E-02
cg05844625	-13.84	6.05	2.22E-02	-21.63	8.96	1.58E-02	1.78	11.69	8.79E-01	-3.08	17.26	8.58E-01	-21.26	10.73	4.75E-02
cg11642127	17.86	7.81	2.22E-02	7.96	13.66	5.60E-01	29.63	14.22	3.72E-02	24.89	20.13	2.16E-01	11.66	16.57	4.82E-01
cg19906694	23.06	10.08	2.22E-02	26.66	25.27	2.91E-01	79.51	99.44	4.24E-01	16.90	10.87	1.20E-01	88.98	42.69	3.71E-02
cg03186339	16.52	7.22	2.22E-02	5.89	11.71	6.15E-01	18.42	16.67	2.69E-01	25.09	14.51	8.37E-02	24.99	16.82	1.37E-01
cg16296290	-32.53	14.22	2.22E-02	-7.37	41.04	8.57E-01	172.88	181.92	3.42E-01	-43.03	16.38	8.61E-03	-2.26	41.02	9.56E-01
cg08908960	21.00	9.18	2.22E-02	22.64	16.76	1.77E-01	18.08	15.85	2.54E-01	23.97	22.45	2.86E-01	20.92	20.65	3.11E-01
cg00513012	19.93	8.71	2.22E-02	12.59	16.02	4.32E-01	21.72	16.24	1.81E-01	12.26	16.47	4.57E-01	47.74	23.57	4.28E-02
cg05681076	17.74	7.76	2.22E-02	19.24	10.39	6.39E-02	29.27	18.82	1.20E-01	33.21	31.72	2.95E-01	0.23	16.83	9.89E-01
cg20742366	26.35	11.52	2.22E-02	26.21	21.05	2.13E-01	-12.60	32.38	6.97E-01	28.03	17.82	1.16E-01	53.78	29.19	6.54E-02
cg26116485	14.45	6.32	2.22E-02	19.18	12.26	1.18E-01	0.53	16.81	9.75E-01	23.47	10.58	2.65E-02	3.84	13.01	7.68E-01
cg25903261	11.55	5.05	2.22E-02	11.79	7.81	1.31E-01	9.06	8.80	3.03E-01	4.61	16.41	7.79E-01	20.24	12.70	1.11E-01
cg14511782	5.82	2.54	2.22E-02	5.44	3.49	1.19E-01	5.89	6.38	3.55E-01	10.22	6.14	9.63E-02	1.73	6.85	8.00E-01
cg10511988	16.37	7.16	2.22E-02	13.43	11.10	2.26E-01	39.97	26.54	1.32E-01	34.85	25.48	1.71E-01	11.85	10.88	2.76E-01
cg19668476	-12.63	5.52	2.22E-02	-7.41	9.49	4.35E-01	-14.63	14.40	3.10E-01	-17.31	9.85	7.88E-02	-12.65	12.36	3.06E-01
cg11200917	7.75	3.39	2.22E-02	6.31	5.71	2.69E-01	11.34	11.21	3.12E-01	5.61	5.79	3.32E-01	12.00	7.32	1.01E-01
cg02541444	6.53	2.85	2.22E-02	9.70	3.95	1.40E-02	5.92	6.07	3.29E-01	-0.06	8.26	9.94E-01	1.10	7.73	8.87E-01
cg04178787	11.61	5.08	2.22E-02	22.90	11.06	3.83E-02	11.50	12.93	3.74E-01	12.14	8.54	1.55E-01	2.55	9.56	7.90E-01
cg11358840	14.78	6.46	2.22E-02	13.59	16.99	4.24E-01	0.24	32.40	9.94E-01	14.37	7.57	5.76E-02	27.09	22.09	2.20E-01
cg16338365	-3.64	1.59	2.22E-02	-3.58	2.36	1.29E-01	-3.22	3.44	3.48E-01	-3.09	4.84	5.23E-01	-4.44	3.38	1.89E-01
cg00670060	-13.42	5.87	2.22E-02	-28.89	16.42	7.85E-02	-0.62	23.40	9.79E-01	-10.43	6.93	1.32E-01	-24.03	19.37	2.15E-01
cg19931596	-13.60	5.95	2.22E-02	-37.23	16.82	2.69E-02	8.01	30.94	7.96E-01	-9.89	6.76	1.43E-01	-24.74	23.47	2.92E-01
cg14952949	-9.82	4.29	2.22E-02	-11.73	5.68	3.88E-02	-19.43	11.03	7.81E-02	-6.99	17.01	6.81E-01	1.34	9.32	8.86E-01
cg11550426	-41.50	18.15	2.22E-02	-54.17	22.95	1.83E-02	59.28	81.91	4.69E-01	3.21	48.34	9.47E-01	-59.48	42.18	1.59E-01
cg03120555	7.66	3.35	2.22E-02	4.01	5.29	4.49E-01	12.19	6.92	7.80E-02	5.81	7.84	4.59E-01	11.68	7.83	1.36E-01
cg09411999	11.07	4.84	2.22E-02	16.91	7.02	1.60E-02	7.67	12.50	5.40E-01	9.25	10.51	3.79E-01	-0.52	12.02	9.66E-01
cg00318865	33.04	14.45	2.22E-02	50.65	22.28	2.30E-02	47.14	46.53	3.11E-01	37.97	33.91	2.63E-01	0.98	26.32	9.70E-01
cg07116163	-22.80	9.97	2.22E-02	-21.07	11.15	5.89E-02	19.08	49.44	7.00E-01	-62.96	59.12	2.87E-01	-37.56	27.48	1.72E-01
cg25773246	31.58	13.81	2.22E-02	36.95	21.37	8.39E-02	49.88	36.81	1.75E-01	12.87	33.11	6.98E-01	25.76	26.70	3.35E-01

cg18422875	27.86	12.18	2.22E-02	30.50	19.34	1.15E-01	-20.57	68.22	7.63E-01	90.42	50.35	7.25E-02	21.68	17.02	2.03E-01
cg22833175	-10.25	4.48	2.22E-02	-7.38	10.83	4.95E-01	-18.58	15.20	2.22E-01	-6.89	5.67	2.24E-01	-26.41	13.18	4.52E-02
cg17967292	-7.46	3.26	2.22E-02	-10.80	5.10	3.42E-02	-7.10	6.14	2.47E-01	0.00	9.37	1.00E+00	-5.52	7.51	4.63E-01
cg03792964	-9.85	4.31	2.22E-02	-16.16	8.88	6.89E-02	1.39	10.90	8.99E-01	-10.22	6.71	1.28E-01	-10.46	9.72	2.82E-01
cg15520042	-20.32	8.89	2.22E-02	15.70	27.70	5.71E-01	62.96	131.87	6.33E-01	-25.11	9.70	9.63E-03	-21.59	38.70	5.77E-01
cg11547355	-38.71	16.93	2.22E-02	-44.38	28.01	1.13E-01	-54.59	111.56	6.25E-01	-35.50	24.90	1.54E-01	-32.32	43.87	4.61E-01
cg01165997	-10.36	4.53	2.22E-02	-13.68	8.58	1.11E-01	-7.36	10.86	4.98E-01	-8.64	7.50	2.50E-01	-11.57	10.59	2.75E-01
cg22732812	22.95	10.04	2.22E-02	66.56	44.95	1.39E-01	29.96	166.40	8.57E-01	17.97	10.64	9.12E-02	62.04	42.08	1.40E-01
cg09956907	19.06	8.34	2.22E-02	10.78	11.32	3.41E-01	8.61	21.42	6.88E-01	46.61	32.29	1.49E-01	36.79	17.05	3.09E-02
cg07232711	-13.40	5.86	2.22E-02	-20.15	12.13	9.67E-02	-4.69	18.69	8.02E-01	-12.65	7.66	9.86E-02	-9.97	20.32	6.24E-01
cg19016652	14.79	6.47	2.22E-02	24.55	11.54	3.33E-02	12.73	18.13	4.82E-01	21.14	13.95	1.30E-01	2.64	11.04	8.11E-01
cg07150506	27.48	12.02	2.22E-02	30.13	28.45	2.90E-01	-55.74	159.26	7.26E-01	27.04	13.72	4.88E-02	34.59	54.78	5.28E-01
cg11126205	-10.98	4.80	2.22E-02	-9.64	15.06	5.22E-01	-12.33	25.32	6.26E-01	-11.20	5.37	3.70E-02	-9.52	19.20	6.20E-01
cg10118925	-53.95	23.60	2.22E-02	-56.62	29.33	5.36E-02	-3.03	87.78	9.72E-01	-0.21	96.85	9.98E-01	-77.28	50.23	1.24E-01
cg19625388	-31.02	13.57	2.23E-02	-22.49	19.72	2.54E-01	-7.82	63.39	9.02E-01	-21.10	37.38	5.72E-01	-49.42	22.98	3.15E-02
cg21819604	25.28	11.06	2.23E-02	27.26	14.26	5.59E-02	4.91	45.40	9.14E-01	40.24	67.40	5.50E-01	24.06	19.80	2.24E-01
cg17001101	27.65	12.10	2.23E-02	13.31	28.15	6.36E-01	14.32	67.39	8.32E-01	34.70	14.74	1.85E-02	12.37	36.62	7.36E-01
cg11152463	11.46	5.01	2.23E-02	10.08	8.29	2.24E-01	6.40	10.27	5.33E-01	16.51	13.24	2.12E-01	15.38	9.99	1.24E-01
cg05936283	20.57	9.00	2.23E-02	23.74	16.12	1.41E-01	15.27	15.57	3.27E-01	16.44	19.31	3.95E-01	32.82	24.29	1.77E-01
cg03092447	22.80	9.97	2.23E-02	23.80	16.94	1.60E-01	16.90	19.06	3.75E-01	13.66	20.78	5.11E-01	45.42	25.82	7.85E-02
cg22350910	14.89	6.52	2.23E-02	5.76	10.39	5.80E-01	16.00	13.72	2.44E-01	10.44	11.50	3.64E-01	38.14	14.91	1.05E-02
cg02019444	7.05	3.08	2.23E-02	3.56	4.06	3.81E-01	14.29	7.38	5.29E-02	17.35	10.95	1.13E-01	6.62	7.47	3.75E-01
cg00354884	-19.38	8.48	2.23E-02	-23.28	14.35	1.05E-01	-16.50	17.01	3.32E-01	-2.59	19.86	8.96E-01	-30.34	18.06	9.29E-02
cg02268354	26.06	11.40	2.23E-02	52.13	37.03	1.59E-01	-2.49	126.81	9.84E-01	19.85	12.42	1.10E-01	80.42	48.65	9.83E-02
cg27497928	-10.15	4.44	2.23E-02	-13.91	6.25	2.61E-02	-10.17	11.03	3.57E-01	0.87	13.57	9.49E-01	-6.97	9.34	4.55E-01
cg21029964	6.96	3.04	2.23E-02	10.31	6.40	1.07E-01	8.69	7.56	2.50E-01	5.11	4.36	2.41E-01	5.83	8.62	4.99E-01
cg12868707	-11.91	5.21	2.23E-02	-24.11	8.55	4.78E-03	4.47	15.23	7.69E-01	-7.36	5.85	2.08E-01	-14.66	12.03	2.23E-01
ch.16.785175F	-44.03	19.27	2.23E-02	-24.59	26.16	3.47E-01	38.70	64.15	5.46E-01	-72.76	33.99	3.23E-02	-72.17	34.74	3.77E-02
cg24421094	6.23	2.72	2.23E-02	7.25	6.13	2.37E-01	10.91	6.35	8.59E-02	5.53	3.83	1.49E-01	-0.06	8.09	9.94E-01
cg16459364	21.50	9.41	2.23E-02	8.89	19.74	6.52E-01	13.88	23.95	5.62E-01	32.61	14.86	2.82E-02	19.60	20.17	3.31E-01
cg12297625	-13.29	5.81	2.23E-02	-19.42	9.87	4.91E-02	-1.65	10.55	8.76E-01	-3.54	15.88	8.23E-01	-25.88	12.53	3.89E-02
cg03729204	-12.14	5.31	2.23E-02	-11.03	8.11	1.74E-01	-7.86	13.80	5.69E-01	-15.73	13.69	2.50E-01	-14.25	10.20	1.62E-01
cg02207200	20.96	9.17	2.23E-02	29.78	11.40	9.00E-03	15.69	26.65	5.56E-01	51.86	34.13	1.29E-01	-0.70	16.21	9.66E-01
cg19223411	20.96	9.17	2.23E-02	18.59	16.47	2.59E-01	11.99	18.05	5.07E-01	23.91	18.28	1.91E-01	33.78	21.62	1.18E-01
cg06501113	-20.38	8.92	2.23E-02	-9.28	10.70	3.86E-01	-50.94	25.31	4.41E-02	-40.37	25.63	1.15E-01	-23.62	21.20	2.65E-01

cg04043455	2.33	1.02	2.23E-02	2.43	1.47	9.83E-02	3.97	1.99	4.60E-02	-1.69	3.72	6.49E-01	1.36	2.39	5.71E-01
cg21619825	18.78	8.22	2.23E-02	24.90	20.15	2.17E-01	29.98	23.45	2.01E-01	12.92	10.12	2.02E-01	47.50	36.29	1.91E-01
cg03735888	25.05	10.96	2.23E-02	-9.05	33.72	7.88E-01	39.73	150.53	7.92E-01	32.93	12.16	6.79E-03	-12.14	39.43	7.58E-01
cg13286116	5.45	2.39	2.23E-02	5.54	3.13	7.63E-02	11.06	6.60	9.37E-02	-3.71	8.87	6.75E-01	4.89	5.16	3.43E-01
cg05761564	14.45	6.32	2.23E-02	31.95	15.54	3.98E-02	-6.39	37.71	8.65E-01	11.43	7.46	1.26E-01	12.88	21.31	5.46E-01
cg17067190	20.81	9.11	2.23E-02	31.90	30.40	2.94E-01	-72.21	152.95	6.37E-01	20.33	9.94	4.07E-02	16.90	35.38	6.33E-01
cg14238959	15.07	6.60	2.23E-02	18.42	10.37	7.58E-02	6.51	30.98	8.34E-01	13.02	10.73	2.25E-01	13.98	15.89	3.79E-01
cg14955617	23.10	10.11	2.23E-02	16.86	18.76	3.69E-01	35.71	20.32	7.88E-02	20.07	19.33	2.99E-01	20.54	23.29	3.78E-01
cg23244463	-14.59	6.39	2.23E-02	-8.36	12.71	5.11E-01	15.00	27.07	5.80E-01	-19.95	8.35	1.68E-02	-15.38	19.58	4.32E-01
cg04999691	14.71	6.44	2.23E-02	14.49	9.43	1.24E-01	19.18	13.61	1.59E-01	23.50	18.14	1.95E-01	3.83	14.99	7.98E-01
cg09259004	37.78	16.53	2.23E-02	-4.68	28.88	8.71E-01	-26.56	122.12	8.28E-01	50.24	12.58	6.48E-05	73.46	48.07	1.26E-01
cg24188163	7.92	3.47	2.23E-02	7.68	7.25	2.89E-01	19.36	8.38	2.09E-02	5.94	4.79	2.15E-01	-3.40	12.56	7.87E-01
cg06138468	-11.08	4.85	2.23E-02	-12.89	13.67	3.46E-01	20.49	21.40	3.38E-01	-13.89	5.61	1.33E-02	-1.67	17.67	9.25E-01
cg08457011	13.74	6.01	2.23E-02	26.33	22.33	2.38E-01	17.54	17.37	3.13E-01	18.57	7.86	1.82E-02	-5.05	12.73	6.92E-01
cg03711717	-15.22	6.66	2.23E-02	-28.00	16.41	8.79E-02	-18.09	28.52	5.26E-01	-11.29	7.99	1.58E-01	-20.72	22.81	3.64E-01
cg23181482	-15.12	6.62	2.23E-02	-25.14	18.19	1.67E-01	-37.19	38.81	3.38E-01	-13.45	7.62	7.74E-02	-6.66	22.89	7.71E-01
cg17047829	-8.99	3.93	2.23E-02	-18.38	10.23	7.23E-02	8.97	14.20	5.28E-01	-8.25	4.68	7.78E-02	-16.54	15.10	2.73E-01
cg17130032	-16.93	7.41	2.23E-02	-33.47	15.76	3.36E-02	29.40	52.99	5.79E-01	-9.14	9.79	3.50E-01	-26.19	17.20	1.28E-01
cg09304006	-37.03	16.21	2.23E-02	-35.90	18.97	5.84E-02	-113.35	114.14	3.21E-01	6.48	67.74	9.24E-01	-46.23	36.91	2.10E-01
cg17533563	-10.62	4.65	2.23E-02	-15.41	11.79	1.91E-01	-12.60	22.05	5.68E-01	-8.01	5.52	1.46E-01	-21.98	15.51	1.56E-01
cg11254700	7.35	3.22	2.23E-02	5.69	5.03	2.58E-01	14.33	6.63	3.08E-02	11.30	8.46	1.82E-01	0.11	6.98	9.87E-01
cg06211550	-3.47	1.52	2.23E-02	-4.11	2.19	6.10E-02	-2.95	3.23	3.62E-01	-3.55	4.51	4.30E-01	-2.39	3.54	4.99E-01
cg15096829	-33.87	14.83	2.23E-02	-27.56	21.51	2.00E-01	20.09	63.96	7.53E-01	-41.27	27.46	1.33E-01	-54.73	35.00	1.18E-01
cg12402129	-9.23	4.04	2.23E-02	-8.37	7.28	2.50E-01	-18.88	8.75	3.10E-02	-11.79	10.90	2.79E-01	-2.94	6.92	6.71E-01
cg06786349	-17.18	7.52	2.23E-02	-23.95	17.49	1.71E-01	-23.51	39.24	5.49E-01	-16.89	9.17	6.56E-02	-5.06	23.10	8.27E-01
cg24077501	25.58	11.20	2.23E-02	40.14	18.04	2.61E-02	11.96	40.78	7.69E-01	25.86	24.35	2.88E-01	11.41	19.57	5.60E-01
cg16615211	8.66	3.79	2.24E-02	9.89	5.27	6.06E-02	14.56	8.31	7.99E-02	6.35	14.58	6.63E-01	0.41	8.33	9.60E-01
cg14805890	7.79	3.41	2.24E-02	8.40	6.34	1.85E-01	-2.11	7.55	7.80E-01	10.94	5.64	5.23E-02	12.76	9.13	1.62E-01
cg14443182	-14.64	6.41	2.24E-02	-25.22	9.64	8.92E-03	-7.61	13.15	5.63E-01	-3.15	18.00	8.61E-01	-6.67	14.53	6.46E-01
cg22199658	19.24	8.42	2.24E-02	70.59	32.26	2.86E-02	4.27	82.75	9.59E-01	16.06	10.65	1.31E-01	14.66	15.49	3.44E-01
cg22940372	-8.88	3.89	2.24E-02	-17.53	7.38	1.75E-02	-7.71	12.85	5.48E-01	-4.76	5.50	3.87E-01	-7.07	10.72	5.09E-01
cg25943821	14.57	6.38	2.24E-02	17.24	17.46	3.23E-01	41.58	27.56	1.31E-01	11.09	7.35	1.31E-01	28.48	26.30	2.79E-01
cg22561757	-14.67	6.42	2.24E-02	-21.80	12.54	8.22E-02	-30.01	15.45	5.21E-02	-9.99	9.75	3.06E-01	4.26	17.71	8.10E-01
cg14537247	-8.08	3.54	2.24E-02	-10.29	8.36	2.18E-01	-4.77	12.72	7.08E-01	-6.79	4.49	1.31E-01	-13.45	10.08	1.82E-01
cg01315916	11.35	4.97	2.24E-02	18.14	8.78	3.89E-02	7.16	13.82	6.04E-01	10.75	9.64	2.65E-01	6.18	9.31	5.07E-01

cg23273465	-23.17	10.14	2.24E-02	4.55	26.36	8.63E-01	-59.15	62.66	3.45E-01	-30.93	11.71	8.24E-03	12.39	37.04	7.38E-01
cg11552853	-15.79	6.91	2.24E-02	-24.90	20.50	2.24E-01	-17.69	25.85	4.94E-01	-12.17	7.98	1.27E-01	-39.57	27.15	1.45E-01
cg21874404	-9.63	4.22	2.24E-02	-1.63	8.68	8.51E-01	-4.77	17.49	7.85E-01	-11.62	5.55	3.61E-02	-17.64	11.82	1.35E-01
cg08388004	31.84	13.94	2.24E-02	60.00	24.16	1.30E-02	41.77	52.71	4.28E-01	9.33	16.94	5.82E-01	49.50	34.84	1.55E-01
cg17341174	6.44	2.82	2.24E-02	8.46	4.19	4.34E-02	8.86	7.37	2.30E-01	7.64	5.48	1.63E-01	-5.21	7.64	4.95E-01
cg26874229	-4.18	1.83	2.24E-02	-3.80	2.47	1.24E-01	-1.44	4.56	7.52E-01	-2.85	5.80	6.23E-01	-8.38	4.22	4.72E-02
cg24931346	-14.59	6.39	2.24E-02	-15.83	9.36	9.08E-02	-24.08	14.38	9.39E-02	-10.27	16.32	5.29E-01	-4.84	14.91	7.45E-01
cg20362529	-27.06	11.85	2.24E-02	-14.78	16.94	3.83E-01	-5.84	30.89	8.50E-01	-60.51	27.98	3.06E-02	-44.07	27.61	1.10E-01
cg19075614	31.20	13.66	2.24E-02	31.22	19.29	1.06E-01	56.22	39.42	1.54E-01	-20.13	38.87	6.04E-01	44.24	27.06	1.02E-01
cg03877364	31.87	13.96	2.24E-02	43.85	17.85	1.40E-02	8.75	80.75	9.14E-01	28.82	59.12	6.26E-01	10.56	25.35	6.77E-01
cg04931090	28.34	12.41	2.24E-02	34.95	16.66	3.59E-02	33.08	33.01	3.16E-01	-11.30	61.06	8.53E-01	18.03	24.25	4.57E-01
cg06661482	38.35	16.80	2.24E-02	43.79	45.62	3.37E-01	-44.67	213.31	8.34E-01	33.83	19.35	8.04E-02	68.61	51.81	1.85E-01
cg05245430	-37.78	16.55	2.24E-02	-44.81	23.74	5.91E-02	54.50	94.78	5.65E-01	-46.33	31.20	1.38E-01	-22.94	36.76	5.33E-01
cg19340889	19.21	8.41	2.24E-02	32.73	14.14	2.06E-02	42.52	23.03	6.48E-02	13.44	15.57	3.88E-01	3.76	12.60	7.65E-01
cg10411510	-20.51	8.98	2.24E-02	-11.54	16.36	4.81E-01	-83.37	37.00	2.42E-02	-20.34	9.91	4.03E-02	-9.47	27.02	7.26E-01
cg18484958	-10.08	4.42	2.24E-02	-16.07	9.00	7.43E-02	-5.86	12.53	6.40E-01	-7.92	6.35	2.12E-01	-10.98	11.38	3.35E-01
cg07689907	20.46	8.96	2.24E-02	28.02	12.98	3.09E-02	-1.54	28.40	9.57E-01	37.69	30.00	2.09E-01	11.64	15.49	4.52E-01
cg06370855	19.74	8.64	2.24E-02	32.51	18.79	8.37E-02	38.11	24.55	1.21E-01	7.78	10.27	4.49E-01	45.46	28.47	1.10E-01
cg08782674	12.79	5.60	2.24E-02	-2.99	16.01	8.52E-01	34.54	29.95	2.49E-01	14.99	6.28	1.70E-02	0.71	25.57	9.78E-01
cg12332902	-14.05	6.16	2.24E-02	-7.89	7.87	3.16E-01	-37.87	15.33	1.35E-02	-6.75	12.06	5.76E-01	-18.89	13.54	1.63E-01
cg01188191	-16.73	7.33	2.24E-02	-13.23	11.12	2.34E-01	-14.91	20.46	4.66E-01	-16.90	16.27	2.99E-01	-24.05	15.12	1.12E-01
cg14342823	16.11	7.05	2.24E-02	27.34	16.94	1.07E-01	-6.61	34.79	8.49E-01	12.63	8.33	1.29E-01	37.72	26.99	1.62E-01
cg07079314	15.93	6.98	2.24E-02	15.77	21.52	4.64E-01	-5.40	33.14	8.71E-01	14.55	7.78	6.15E-02	60.19	32.27	6.22E-02
cg02668729	-38.90	17.04	2.24E-02	-46.60	22.41	3.76E-02	11.40	66.88	8.65E-01	0.94	60.92	9.88E-01	-45.84	32.29	1.56E-01
cg09915519	18.55	8.12	2.24E-02	33.91	21.50	1.15E-01	59.77	38.04	1.16E-01	14.68	9.53	1.23E-01	3.64	27.93	8.96E-01
cg15010213	-53.05	23.24	2.24E-02	-46.57	31.96	1.45E-01	18.77	147.39	8.99E-01	-104.39	51.28	4.18E-02	-30.93	47.33	5.13E-01
cg12858300	-74.51	32.64	2.24E-02	-185.86	72.68	1.06E-02	-160.61	258.02	5.34E-01	-63.44	28.02	2.36E-02	-5.14	67.06	9.39E-01
cg12085570	13.55	5.94	2.24E-02	13.40	14.00	3.39E-01	33.09	17.97	6.56E-02	18.48	9.86	6.08E-02	2.38	10.06	8.13E-01
cg21196581	-14.87	6.51	2.25E-02	-21.21	9.26	2.19E-02	2.87	24.86	9.08E-01	-7.42	11.77	5.28E-01	-17.62	18.05	3.29E-01
cg05441768	11.53	5.05	2.25E-02	14.38	7.34	5.00E-02	21.08	13.72	1.25E-01	10.21	15.40	5.08E-01	2.69	9.49	7.77E-01
cg17003212	-36.00	15.77	2.25E-02	-44.08	19.06	2.07E-02	35.57	54.75	5.16E-01	18.52	67.59	7.84E-01	-54.97	37.40	1.42E-01
cg00398268	-9.81	4.30	2.25E-02	-21.41	11.60	6.49E-02	-0.56	22.41	9.80E-01	-6.51	5.07	1.99E-01	-20.30	13.16	1.23E-01
cg26475911	7.54	3.30	2.25E-02	8.42	4.45	5.83E-02	13.20	8.14	1.05E-01	2.11	10.73	8.44E-01	2.75	7.60	7.18E-01
cg11042561	6.13	2.68	2.25E-02	7.90	3.88	4.18E-02	3.05	7.87	6.98E-01	11.85	6.85	8.38E-02	0.70	5.34	8.95E-01
cg27504369	13.46	5.90	2.25E-02	7.22	8.21	3.79E-01	28.00	12.93	3.03E-02	15.19	18.47	4.11E-01	13.58	14.15	3.37E-01

cg25050723	16.58	7.26	2.25E-02	31.31	13.43	1.97E-02	0.15	16.30	9.93E-01	12.60	11.60	2.78E-01	20.92	21.21	3.24E-01
cg19636302	7.14	3.13	2.25E-02	6.24	4.23	1.40E-01	10.92	7.24	1.31E-01	15.13	11.06	1.71E-01	2.54	7.26	7.26E-01
cg02476628	21.32	9.34	2.25E-02	29.80	16.15	6.51E-02	-1.79	18.55	9.23E-01	22.53	19.08	2.37E-01	37.26	22.54	9.83E-02
cg14728024	-7.75	3.40	2.25E-02	-7.64	4.92	1.21E-01	1.63	9.46	8.63E-01	-14.94	6.60	2.35E-02	-2.80	9.39	7.66E-01
cg00462007	13.18	5.77	2.25E-02	14.22	11.88	2.32E-01	13.53	35.66	7.04E-01	18.24	7.47	1.46E-02	-10.24	15.43	5.07E-01
cg04118119	6.07	2.66	2.25E-02	7.20	3.39	3.36E-02	10.46	7.82	1.81E-01	2.36	7.38	7.49E-01	0.82	7.18	9.09E-01
cg11939496	5.73	2.51	2.25E-02	4.23	3.19	1.84E-01	12.85	6.15	3.67E-02	4.40	7.28	5.46E-01	4.66	8.13	5.66E-01
cg05736642	13.28	5.82	2.25E-02	26.40	13.82	5.61E-02	12.54	17.14	4.64E-01	12.14	7.38	9.98E-02	-4.55	19.81	8.18E-01
cg08627352	15.34	6.72	2.25E-02	18.37	9.53	5.40E-02	4.60	27.84	8.69E-01	11.26	29.63	7.04E-01	13.65	10.73	2.03E-01
cg27260684	5.98	2.62	2.25E-02	5.98	3.41	7.89E-02	12.51	6.78	6.50E-02	2.13	7.85	7.86E-01	2.25	6.86	7.43E-01
cg21597692	-14.05	6.16	2.25E-02	-23.29	10.30	2.38E-02	-6.67	16.06	6.78E-01	-8.18	10.99	4.56E-01	-12.02	14.45	4.06E-01
cg01058602	-15.65	6.86	2.25E-02	-27.66	19.22	1.50E-01	-53.71	31.13	8.45E-02	-12.84	7.97	1.07E-01	-0.18	23.66	9.94E-01
cg16182375	10.22	4.48	2.25E-02	16.22	9.08	7.42E-02	9.55	13.68	4.85E-01	6.33	5.91	2.84E-01	21.38	16.28	1.89E-01
cg20289346	-26.11	11.44	2.25E-02	-16.23	24.39	5.06E-01	-276.02	151.63	6.87E-02	-25.35	13.56	6.15E-02	-40.53	38.80	2.96E-01
cg07180460	-38.53	16.88	2.25E-02	-47.48	22.69	3.64E-02	9.05	95.50	9.24E-01	-41.14	75.17	5.84E-01	-28.65	27.96	3.06E-01
cg11696200	-18.28	8.01	2.25E-02	-20.36	15.16	1.79E-01	-1.54	28.67	9.57E-01	-21.39	11.00	5.18E-02	-10.04	23.93	6.75E-01
cg09077319	9.87	4.32	2.25E-02	5.62	6.20	3.65E-01	11.87	9.14	1.94E-01	7.73	12.90	5.49E-01	20.39	10.28	4.73E-02
cg06176471	-19.05	8.35	2.25E-02	-42.42	16.70	1.11E-02	-10.38	19.42	5.93E-01	-8.35	9.71	3.89E-01	-29.97	20.60	1.46E-01
cg00901982	5.76	2.52	2.25E-02	5.05	3.09	1.02E-01	11.04	5.95	6.36E-02	5.57	11.37	6.24E-01	1.24	7.83	8.74E-01
cg09491948	-29.76	13.04	2.25E-02	-10.93	25.21	6.65E-01	-94.67	143.93	5.11E-01	-41.30	16.80	1.40E-02	-9.64	37.43	7.97E-01
cg12417609	-6.58	2.88	2.25E-02	-9.65	5.24	6.54E-02	-6.14	8.48	4.69E-01	-5.93	4.53	1.91E-01	-3.08	6.87	6.54E-01
cg14618996	21.04	9.22	2.25E-02	22.29	14.00	1.11E-01	43.12	25.26	8.78E-02	34.39	25.76	1.82E-01	3.99	16.70	8.11E-01
cg26256901	-7.90	3.46	2.25E-02	-4.83	4.62	2.95E-01	-17.55	8.06	2.95E-02	-3.52	10.72	7.43E-01	-10.56	8.96	2.38E-01
cg18473137	-12.68	5.56	2.25E-02	-7.22	8.77	4.10E-01	-6.94	22.77	7.61E-01	-13.09	11.99	2.75E-01	-20.24	9.76	3.82E-02
cg12200716	-11.72	5.14	2.25E-02	-19.31	7.74	1.26E-02	-8.35	11.36	4.62E-01	-1.22	17.49	9.44E-01	-5.19	9.91	6.00E-01
cg23460961	10.50	4.60	2.25E-02	15.83	5.97	8.05E-03	4.58	10.40	6.60E-01	14.97	22.34	5.03E-01	-2.59	11.25	8.18E-01
cg21999229	-5.84	2.56	2.25E-02	-4.48	3.22	1.63E-01	-7.89	6.66	2.36E-01	-12.44	10.69	2.44E-01	-6.92	6.35	2.76E-01
cg13336665	7.86	3.45	2.25E-02	1.10	3.82	7.74E-01	9.08	5.32	8.78E-02	15.85	10.63	1.36E-01	13.29	5.13	9.54E-03
cg06081716	-33.20	14.55	2.25E-02	-41.41	17.31	1.67E-02	-34.25	78.45	6.62E-01	-30.97	67.10	6.44E-01	-6.06	31.64	8.48E-01
cg26768816	45.82	20.08	2.25E-02	-27.66	63.67	6.64E-01	-70.86	268.06	7.92E-01	51.80	22.17	1.95E-02	86.93	73.72	2.38E-01
cg15975574	22.59	9.90	2.25E-02	36.00	15.18	1.77E-02	-3.00	24.51	9.03E-01	55.50	33.40	9.66E-02	12.92	14.61	3.76E-01
cg20277670	17.30	7.58	2.25E-02	12.65	21.33	5.53E-01	3.53	31.31	9.10E-01	21.22	8.75	1.53E-02	-7.18	30.14	8.12E-01
cg08288383	-22.11	9.69	2.25E-02	-26.63	13.00	4.05E-02	-18.91	22.98	4.11E-01	26.82	39.54	4.98E-01	-26.91	21.33	2.07E-01
cg03972466	12.10	5.31	2.25E-02	17.17	8.22	3.68E-02	14.02	13.74	3.08E-01	13.64	10.75	2.04E-01	-2.40	12.14	8.43E-01
cg17130251	-13.61	5.97	2.25E-02	-30.09	9.58	1.69E-03	-0.36	13.87	9.79E-01	-8.04	6.23	1.96E-01	-13.80	11.06	2.12E-01

cg27168573	15.04	6.59	2.25E-02	28.17	10.46	7.08E-03	5.06	13.02	6.97E-01	10.62	14.81	4.73E-01	3.00	17.13	8.61E-01
cg05636177	28.71	12.59	2.25E-02	50.10	36.55	1.70E-01	10.51	134.32	9.38E-01	27.61	14.00	4.86E-02	5.68	49.42	9.08E-01
cg26595828	15.33	6.72	2.26E-02	26.38	15.35	8.57E-02	6.37	30.04	8.32E-01	8.57	9.04	3.43E-01	25.42	14.84	8.67E-02
cg07053162	-19.37	8.49	2.26E-02	-4.26	15.08	7.78E-01	-25.03	22.11	2.58E-01	-18.80	13.74	1.71E-01	-46.70	21.71	3.15E-02
cg02923047	21.40	9.38	2.26E-02	35.26	19.13	6.53E-02	6.60	16.36	6.87E-01	19.15	18.89	3.11E-01	32.77	21.89	1.34E-01
cg07030277	25.45	11.16	2.26E-02	31.99	15.85	4.35E-02	33.90	30.39	2.65E-01	34.15	38.82	3.79E-01	7.68	20.82	7.12E-01
cg04281216	-42.34	18.56	2.26E-02	-20.75	26.05	4.26E-01	-127.50	77.11	9.82E-02	-21.91	52.24	6.75E-01	-70.34	33.47	3.56E-02
cg07745562	21.42	9.39	2.26E-02	44.22	19.56	2.38E-02	-4.18	34.76	9.04E-01	12.58	12.22	3.03E-01	38.77	28.87	1.79E-01
cg06829760	-15.87	6.96	2.26E-02	-22.26	7.34	2.43E-03	-9.23	13.44	4.92E-01	3.70	12.16	7.61E-01	-30.01	12.02	1.25E-02
cg03540175	7.70	3.38	2.26E-02	2.19	4.03	5.87E-01	11.91	5.67	3.57E-02	17.80	7.54	1.82E-02	4.98	6.19	4.22E-01
cg25750469	-39.69	17.40	2.26E-02	-79.20	34.69	2.24E-02	-47.46	86.25	5.82E-01	-25.08	22.91	2.74E-01	-25.67	48.15	5.94E-01
cg23326675	-21.54	9.44	2.26E-02	-32.29	32.86	3.26E-01	-51.05	140.71	7.17E-01	-18.99	10.23	6.35E-02	-40.23	38.10	2.91E-01
cg01201399	11.62	5.10	2.26E-02	13.14	6.69	4.97E-02	10.22	8.26	2.16E-01	20.60	6.86	2.66E-03	-10.14	12.57	4.20E-01
cg17295910	-18.65	8.18	2.26E-02	-39.48	17.51	2.41E-02	-9.04	20.51	6.59E-01	-10.78	7.65	1.59E-01	-47.83	28.95	9.85E-02
cg27365991	-11.06	4.85	2.26E-02	-16.23	8.46	5.49E-02	-11.97	10.59	2.59E-01	-1.58	9.11	8.63E-01	-15.49	11.47	1.77E-01
cg16738453	-14.94	6.55	2.26E-02	13.39	20.88	5.21E-01	-16.78	26.44	5.26E-01	-18.30	7.39	1.32E-02	-15.59	28.39	5.83E-01
cg03205103	19.17	8.41	2.26E-02	33.00	34.23	3.35E-01	-45.97	122.47	7.07E-01	17.02	9.06	6.02E-02	37.07	30.92	2.31E-01
cg04987827	53.62	23.51	2.26E-02	15.36	47.47	7.46E-01	-9.87	178.17	9.56E-01	81.52	20.41	6.48E-05	-11.67	59.87	8.45E-01
cg14651082	-18.22	7.99	2.26E-02	-14.85	12.48	2.34E-01	-14.95	15.05	3.21E-01	-48.41	18.91	1.05E-02	-5.26	15.75	7.38E-01
cg03278114	23.76	10.42	2.26E-02	18.59	20.30	3.60E-01	21.38	18.57	2.50E-01	23.92	22.48	2.87E-01	33.81	22.91	1.40E-01
cg08160862	-57.48	25.21	2.26E-02	-68.35	33.61	4.20E-02	63.63	125.18	6.11E-01	-67.18	63.41	2.89E-01	-46.02	51.57	3.72E-01
cg04112100	-9.14	4.01	2.26E-02	-5.58	8.17	4.94E-01	0.39	17.81	9.82E-01	-10.64	5.22	4.14E-02	-12.99	11.66	2.66E-01
cg07068570	-12.26	5.37	2.26E-02	-2.85	12.15	8.14E-01	-13.66	16.14	3.97E-01	-16.41	6.94	1.81E-02	-3.65	17.55	8.35E-01
cg12377641	-17.22	7.55	2.26E-02	-21.68	15.60	1.65E-01	-3.26	26.19	9.01E-01	-16.82	10.04	9.38E-02	-20.15	22.15	3.63E-01
cg18032430	-12.46	5.46	2.26E-02	-3.26	13.86	8.14E-01	11.49	20.64	5.78E-01	-16.60	6.54	1.12E-02	-15.38	19.72	4.36E-01
cg00153919	6.49	2.84	2.26E-02	5.96	4.01	1.37E-01	10.27	7.30	1.59E-01	7.52	6.62	2.56E-01	3.36	7.11	6.37E-01
cg26341102	49.01	21.50	2.26E-02	28.91	29.65	3.30E-01	89.54	82.66	2.79E-01	106.36	63.07	9.17E-02	53.03	39.89	1.84E-01
cg20725716	-24.83	10.89	2.26E-02	-53.96	18.09	2.86E-03	-0.82	16.89	9.61E-01	-29.22	13.21	2.69E-02	-13.96	20.48	4.96E-01
cg24363393	17.09	7.50	2.26E-02	8.48	14.35	5.55E-01	-4.74	24.45	8.46E-01	26.13	10.60	1.37E-02	16.19	20.58	4.32E-01
cg18787420	-10.25	4.49	2.26E-02	-17.43	7.44	1.91E-02	-15.06	10.60	1.55E-01	-2.40	9.14	7.93E-01	-2.79	9.73	7.74E-01
cg17897025	31.62	13.87	2.26E-02	29.29	16.22	7.10E-02	109.32	66.82	1.02E-01	62.09	63.35	3.27E-01	14.20	32.85	6.66E-01
cg17303833	12.84	5.63	2.26E-02	20.99	9.49	2.69E-02	7.10	14.57	6.26E-01	11.67	11.21	2.98E-01	5.84	11.35	6.07E-01
cg03477240	-12.00	5.26	2.26E-02	-8.33	14.43	5.64E-01	1.12	25.87	9.65E-01	-11.80	6.10	5.28E-02	-26.49	18.51	1.52E-01
cg08452898	40.86	17.92	2.26E-02	32.28	48.93	5.09E-01	-221.07	222.25	3.20E-01	41.37	20.23	4.09E-02	73.59	65.49	2.61E-01
cg14327359	-18.08	7.93	2.26E-02	-46.18	23.98	5.41E-02	7.21	28.58	8.01E-01	-17.34	9.19	5.90E-02	-9.66	30.24	7.49E-01

cg02615117	27.11	11.89	2.26E-02	32.50	14.84	2.85E-02	75.93	48.54	1.18E-01	-52.19	68.01	4.43E-01	12.29	22.97	5.92E-01
cg07260806	-9.59	4.20	2.26E-02	-14.48	8.37	8.36E-02	-3.00	14.10	8.32E-01	-8.10	5.66	1.52E-01	-11.19	12.88	3.85E-01
cg03880611	17.30	7.59	2.26E-02	46.57	20.60	2.38E-02	32.30	25.89	2.12E-01	11.01	8.86	2.14E-01	4.76	32.16	8.82E-01
cg13615998	9.21	4.04	2.26E-02	5.86	6.27	3.50E-01	8.26	10.56	4.34E-01	8.85	8.75	3.12E-01	16.32	8.51	5.50E-02
cg17801864	8.60	3.77	2.26E-02	8.92	5.02	7.57E-02	17.83	11.12	1.09E-01	3.88	8.91	6.63E-01	5.78	10.03	5.64E-01
cg16419136	-56.60	24.83	2.26E-02	-71.39	33.68	3.40E-02	-127.07	92.74	1.71E-01	39.65	77.17	6.07E-01	-45.47	46.83	3.32E-01
cg11804833	24.09	10.57	2.26E-02	50.16	18.98	8.21E-03	-19.38	34.99	5.80E-01	23.69	13.76	8.51E-02	14.23	18.18	4.34E-01
cg06796944	14.14	6.20	2.27E-02	0.33	15.84	9.83E-01	78.84	53.71	1.42E-01	17.34	6.78	1.06E-02	-4.57	24.00	8.49E-01
cg21390624	18.00	7.90	2.27E-02	20.97	11.66	7.21E-02	13.75	19.62	4.83E-01	23.38	20.54	2.55E-01	11.64	16.40	4.78E-01
cg02582587	-15.41	6.76	2.27E-02	-34.66	20.23	8.66E-02	-10.82	38.26	7.77E-01	-11.12	7.58	1.42E-01	-38.92	27.59	1.58E-01
cg21123913	23.54	10.33	2.27E-02	26.01	18.02	1.49E-01	-6.89	30.30	8.20E-01	31.31	17.22	6.90E-02	23.18	23.37	3.21E-01
cg25230043	92.20	40.46	2.27E-02	39.19	54.03	4.68E-01	364.64	204.94	7.52E-02	147.16	87.94	9.43E-02	109.39	75.78	1.49E-01
cg19126169	52.11	22.86	2.27E-02	27.62	21.16	1.92E-01	140.94	53.18	8.04E-03	53.44	56.60	3.45E-01	45.96	47.78	3.36E-01
cg27107970	36.67	16.09	2.27E-02	46.63	22.26	3.62E-02	71.03	45.68	1.20E-01	35.93	43.62	4.10E-01	-6.43	34.51	8.52E-01
cg15032897	-13.77	6.04	2.27E-02	-17.97	8.91	4.37E-02	-6.90	15.45	6.55E-01	-36.76	22.74	1.06E-01	-5.86	10.74	5.85E-01
cg06982190	16.55	7.26	2.27E-02	25.84	9.63	7.29E-03	17.52	15.97	2.73E-01	31.98	20.66	1.22E-01	0.51	10.48	9.61E-01
cg04329870	-11.98	5.25	2.27E-02	-16.62	8.70	5.60E-02	-18.45	13.83	1.82E-01	-12.01	11.84	3.10E-01	-3.00	9.70	7.57E-01
cg18207099	45.76	20.08	2.27E-02	43.24	26.87	1.08E-01	29.46	56.48	6.02E-01	44.45	61.11	4.67E-01	63.15	44.10	1.52E-01
cg24984818	-53.69	23.56	2.27E-02	-62.18	30.61	4.22E-02	-110.63	99.46	2.66E-01	3.27	62.72	9.58E-01	-52.81	51.36	3.04E-01
cg13503476	-11.62	5.10	2.27E-02	-15.21	8.65	7.88E-02	-7.40	11.66	5.26E-01	-13.50	9.23	1.44E-01	-5.15	12.85	6.89E-01
cg07141824	28.40	12.46	2.27E-02	-6.49	25.49	7.99E-01	7.69	170.83	9.64E-01	43.92	13.26	9.24E-04	17.95	28.12	5.23E-01
cg06540747	-17.05	7.48	2.27E-02	-25.19	10.09	1.26E-02	-8.88	16.98	6.01E-01	32.63	36.81	3.75E-01	-13.18	16.15	4.14E-01
cg19078520	-34.32	15.06	2.27E-02	-22.69	43.07	5.98E-01	147.44	150.25	3.26E-01	-44.47	17.51	1.11E-02	-1.06	42.13	9.80E-01
cg11325267	12.75	5.60	2.27E-02	18.35	9.06	4.28E-02	2.01	12.48	8.72E-01	21.66	15.24	1.55E-01	8.59	10.54	4.15E-01
cg22970003	5.64	2.47	2.27E-02	3.88	3.81	3.09E-01	10.71	6.29	8.90E-02	6.00	5.16	2.45E-01	4.97	5.59	3.74E-01
cg13938881	-38.82	17.03	2.27E-02	-30.54	23.40	1.92E-01	-175.34	126.30	1.65E-01	-25.17	68.71	7.14E-01	-45.83	27.26	9.27E-02
cg21635203	12.84	5.63	2.27E-02	13.97	6.43	2.98E-02	11.26	32.66	7.30E-01	4.13	46.26	9.29E-01	9.14	12.99	4.82E-01
cg04878851	10.76	4.72	2.27E-02	16.34	8.70	6.03E-02	-11.62	14.44	4.21E-01	13.79	6.95	4.73E-02	7.55	11.27	5.03E-01
cg21466913	-13.04	5.72	2.27E-02	-24.00	16.48	1.45E-01	-14.74	22.19	5.07E-01	-9.87	6.64	1.37E-01	-26.44	21.79	2.25E-01
cg06262098	14.86	6.52	2.27E-02	16.99	9.63	7.76E-02	19.15	13.36	1.52E-01	23.93	17.60	1.74E-01	-4.74	16.03	7.67E-01
cg20169766	-18.38	8.07	2.27E-02	-27.26	19.48	1.62E-01	22.26	34.30	5.16E-01	-15.69	9.71	1.06E-01	-49.32	27.92	7.74E-02
cg16182055	24.76	10.87	2.27E-02	39.87	31.12	2.00E-01	61.25	119.50	6.08E-01	21.84	12.21	7.38E-02	26.93	38.90	4.89E-01
cg21076935	19.60	8.60	2.27E-02	39.28	17.45	2.43E-02	20.78	28.10	4.60E-01	8.30	12.15	4.94E-01	24.32	21.37	2.55E-01
cg12485652	-12.24	5.37	2.27E-02	-11.52	8.25	1.62E-01	-6.71	12.95	6.04E-01	-7.73	18.11	6.70E-01	-17.49	9.57	6.76E-02
cg19767249	-16.54	7.26	2.27E-02	-7.17	11.18	5.21E-01	-34.68	26.36	1.88E-01	-39.22	43.97	3.72E-01	-20.65	10.53	4.98E-02

cg12831208	-13.98	6.14	2.27E-02	-27.67	10.46	8.15E-03	-8.49	14.50	5.58E-01	-4.22	13.38	7.53E-01	-7.68	11.90	5.19E-01
cg04883742	-38.88	17.07	2.27E-02	-30.70	45.09	4.96E-01	36.57	183.62	8.42E-01	-43.84	19.54	2.48E-02	-15.78	58.60	7.88E-01
cg18048949	23.98	10.53	2.27E-02	9.66	28.58	7.35E-01	26.52	115.17	8.18E-01	27.02	11.90	2.32E-02	17.73	38.71	6.47E-01
cg15766408	-19.92	8.74	2.27E-02	-9.59	21.91	6.61E-01	-21.25	41.23	6.06E-01	-20.23	10.26	4.87E-02	-39.43	33.11	2.34E-01
cg22397854	30.73	13.49	2.27E-02	34.82	15.82	2.77E-02	35.39	68.04	6.03E-01	44.15	61.66	4.74E-01	10.27	31.28	7.43E-01
cg19165942	23.01	10.10	2.27E-02	31.31	20.05	1.18E-01	48.14	20.82	2.08E-02	5.51	13.31	6.79E-01	29.08	29.15	3.18E-01
cg21569635	-9.70	4.26	2.27E-02	-13.56	6.36	3.31E-02	-6.32	11.41	5.80E-01	3.72	15.99	8.16E-01	-8.80	7.28	2.27E-01
cg09183388	33.59	14.75	2.27E-02	16.07	16.30	3.24E-01	39.85	63.80	5.32E-01	5.12	78.22	9.48E-01	67.54	23.84	4.61E-03
cg18081881	35.90	15.76	2.27E-02	26.89	18.83	1.53E-01	65.54	72.16	3.64E-01	24.32	54.55	6.56E-01	70.76	38.42	6.55E-02
cg07636846	27.91	12.25	2.27E-02	44.25	19.93	2.64E-02	15.22	35.00	6.64E-01	17.01	28.87	5.56E-01	19.59	21.67	3.66E-01
cg10787000	65.24	28.64	2.27E-02	90.05	37.62	1.67E-02	268.92	201.65	1.82E-01	-9.15	67.89	8.93E-01	41.61	60.74	4.93E-01
cg00472292	-43.36	19.04	2.27E-02	-54.21	23.64	2.19E-02	-23.52	106.46	8.25E-01	62.39	84.13	4.58E-01	-39.69	36.74	2.80E-01
cg04781626	-22.98	10.09	2.27E-02	-25.24	17.14	1.41E-01	-19.14	20.29	3.46E-01	-30.54	19.41	1.16E-01	-9.23	27.31	7.35E-01
cg09006592	-26.58	11.67	2.28E-02	-4.93	40.65	9.03E-01	-36.72	208.67	8.60E-01	-27.31	12.54	2.95E-02	-49.53	52.78	3.48E-01
cg07277923	-9.09	3.99	2.28E-02	-22.80	10.07	2.35E-02	-2.13	17.24	9.02E-01	-8.18	4.87	9.26E-02	0.99	11.70	9.33E-01
cg25765325	35.96	15.79	2.28E-02	34.18	41.74	4.13E-01	123.20	205.69	5.49E-01	38.25	17.93	3.29E-02	8.91	57.56	8.77E-01
cg02770351	15.54	6.82	2.28E-02	12.36	13.81	3.71E-01	-5.71	17.98	7.51E-01	18.74	9.55	4.96E-02	37.38	21.48	8.19E-02
cg05152717	-48.49	21.29	2.28E-02	-72.23	25.94	5.36E-03	2.75	81.37	9.73E-01	5.22	74.16	9.44E-01	-2.57	50.81	9.60E-01
cg19230787	13.72	6.03	2.28E-02	15.73	20.10	4.34E-01	1.95	27.32	9.43E-01	11.04	7.67	1.50E-01	22.09	12.18	6.96E-02
cg11685391	12.88	5.66	2.28E-02	17.16	7.81	2.80E-02	9.63	15.53	5.35E-01	10.74	15.84	4.98E-01	5.72	12.19	6.39E-01
cg14483771	13.17	5.78	2.28E-02	12.57	17.25	4.66E-01	12.44	18.59	5.03E-01	16.05	6.69	1.65E-02	-31.90	27.34	2.43E-01
cg00001854	-11.20	4.92	2.28E-02	-22.76	14.80	1.24E-01	-46.99	39.16	2.30E-01	-9.91	5.50	7.14E-02	-0.13	18.24	9.94E-01
cg25069102	18.50	8.13	2.28E-02	18.25	14.46	2.07E-01	27.59	19.64	1.60E-01	27.61	14.08	4.99E-02	-6.48	19.13	7.35E-01
cg14852505	12.27	5.39	2.28E-02	11.14	11.82	3.46E-01	0.77	15.09	9.59E-01	16.77	7.07	1.77E-02	1.51	18.50	9.35E-01
cg03942922	13.92	6.11	2.28E-02	17.28	10.17	8.93E-02	14.11	50.99	7.82E-01	10.88	7.92	1.70E-01	34.09	35.69	3.40E-01
cg05004506	-11.97	5.26	2.28E-02	-15.02	8.14	6.51E-02	1.89	12.06	8.76E-01	-13.55	13.30	3.08E-01	-16.70	10.81	1.22E-01
cg25049486	-8.86	3.89	2.28E-02	-16.93	7.90	3.21E-02	-21.99	14.41	1.27E-01	-5.57	5.23	2.87E-01	-0.50	10.76	9.63E-01
cg09319202	-23.21	10.19	2.28E-02	-37.66	29.64	2.04E-01	2.31	133.86	9.86E-01	-22.96	11.28	4.18E-02	-0.30	41.89	9.94E-01
cg19981568	14.98	6.58	2.28E-02	21.68	19.99	2.78E-01	50.79	85.87	5.54E-01	13.71	7.26	5.89E-02	16.66	25.94	5.21E-01
cg01750654	-10.95	4.81	2.28E-02	-13.33	9.97	1.81E-01	-1.49	13.36	9.11E-01	-11.63	6.57	7.67E-02	-13.98	15.07	3.53E-01
cg16330786	-13.05	5.73	2.28E-02	-19.73	9.23	3.26E-02	-5.58	11.78	6.36E-01	-11.35	16.45	4.90E-01	-10.71	11.31	3.43E-01
cg21466107	-18.45	8.10	2.28E-02	-24.36	12.36	4.88E-02	9.92	17.33	5.67E-01	-24.02	12.77	5.99E-02	-31.64	22.19	1.54E-01
cg03144922	12.97	5.70	2.28E-02	20.47	11.29	6.97E-02	-2.07	13.85	8.81E-01	12.70	11.00	2.48E-01	15.26	10.27	1.37E-01
cg19408398	5.69	2.50	2.28E-02	5.43	4.32	2.09E-01	4.56	5.91	4.40E-01	7.66	4.32	7.63E-02	3.27	6.43	6.11E-01
cg03343571	3.42	1.50	2.28E-02	2.37	2.13	2.67E-01	5.42	3.07	7.77E-02	5.13	4.74	2.79E-01	2.65	3.72	4.76E-01

cg24069602	-9.08	3.99	2.28E-02	-8.67	5.89	1.41E-01	-7.96	11.19	4.77E-01	-6.17	8.07	4.45E-01	-15.26	9.70	1.15E-01
cg00343906	5.19	2.28	2.28E-02	4.36	2.83	1.23E-01	7.86	6.00	1.91E-01	8.20	7.12	2.50E-01	3.69	7.08	6.02E-01
cg08707123	22.07	9.69	2.28E-02	13.69	18.40	4.57E-01	17.63	21.70	4.17E-01	23.43	15.36	1.27E-01	43.46	27.45	1.13E-01
cg01234420	8.93	3.92	2.28E-02	9.91	5.46	6.96E-02	13.77	8.42	1.02E-01	5.67	11.41	6.20E-01	1.06	10.19	9.17E-01
cg09575421	15.04	6.61	2.28E-02	7.77	11.74	5.08E-01	13.26	12.38	2.84E-01	16.91	12.63	1.81E-01	33.53	18.74	7.35E-02
cg21147203	-10.82	4.75	2.28E-02	-11.38	8.00	1.55E-01	-5.51	10.43	5.97E-01	-18.22	9.53	5.59E-02	-5.91	10.89	5.87E-01
cg25643208	-8.51	3.74	2.28E-02	-7.13	7.78	3.59E-01	-10.58	12.73	4.06E-01	-10.40	5.05	3.94E-02	-1.86	10.16	8.54E-01
cg00017639	-6.77	2.98	2.28E-02	-7.24	5.18	1.62E-01	-8.65	7.06	2.20E-01	-3.27	5.12	5.23E-01	-11.26	7.56	1.37E-01
cg08541880	22.65	9.95	2.28E-02	22.94	23.16	3.22E-01	-40.31	45.47	3.75E-01	26.45	12.50	3.44E-02	26.82	27.23	3.25E-01
cg03085524	6.70	2.94	2.28E-02	10.30	4.12	1.25E-02	2.65	7.16	7.12E-01	9.62	8.06	2.32E-01	-1.46	6.77	8.30E-01
cg13401893	2.82	1.24	2.28E-02	1.70	1.69	3.15E-01	4.09	2.49	1.01E-01	5.76	4.73	2.24E-01	3.47	3.27	2.88E-01
cg24765602	-8.96	3.94	2.28E-02	-11.08	5.74	5.35E-02	-12.24	8.87	1.67E-01	-2.06	11.25	8.55E-01	-5.14	8.60	5.50E-01
cg25005422	-36.03	15.83	2.29E-02	-39.81	41.26	3.35E-01	-157.32	175.17	3.69E-01	-35.91	18.07	4.69E-02	-17.23	56.98	7.62E-01
cg11670211	25.58	11.24	2.29E-02	42.91	17.61	1.48E-02	-8.73	28.94	7.63E-01	23.22	32.72	4.78E-01	20.62	19.74	2.96E-01
cg23275644	6.28	2.76	2.29E-02	5.63	3.87	1.45E-01	6.46	7.42	3.84E-01	9.58	5.84	1.01E-01	2.94	7.72	7.04E-01
cg01992832	-16.24	7.14	2.29E-02	-9.16	18.82	6.26E-01	-27.97	25.71	2.77E-01	-14.96	8.41	7.52E-02	-33.58	29.27	2.51E-01
cg22200118	14.68	6.45	2.29E-02	4.45	14.48	7.58E-01	-17.55	27.80	5.28E-01	20.00	8.87	2.41E-02	19.04	13.80	1.68E-01
cg20197123	-14.51	6.38	2.29E-02	-10.23	18.23	5.75E-01	15.84	32.02	6.21E-01	-17.61	7.22	1.47E-02	-2.50	26.64	9.25E-01
cg24606701	-13.82	6.07	2.29E-02	-19.21	13.12	1.43E-01	-3.09	19.04	8.71E-01	-13.49	8.13	9.70E-02	-14.74	17.10	3.89E-01
cg22484822	14.72	6.47	2.29E-02	20.71	13.20	1.17E-01	14.95	10.10	1.39E-01	21.70	14.39	1.32E-01	-5.28	16.85	7.54E-01
cg04078728	22.64	9.95	2.29E-02	15.06	19.12	4.31E-01	17.00	27.69	5.39E-01	24.94	15.14	9.96E-02	33.24	24.24	1.70E-01
cg01832757	-15.09	6.63	2.29E-02	-9.24	12.24	4.50E-01	-16.12	15.61	3.02E-01	-17.02	11.26	1.31E-01	-19.89	15.66	2.04E-01
cg06910084	14.31	6.29	2.29E-02	5.88	8.58	4.93E-01	20.60	14.43	1.53E-01	48.95	28.05	8.10E-02	21.43	13.32	1.08E-01
cg07108944	-16.06	7.06	2.29E-02	-45.01	20.27	2.64E-02	13.73	47.48	7.72E-01	-11.60	7.92	1.43E-01	-26.98	28.19	3.38E-01
cg24634577	14.84	6.52	2.29E-02	17.41	20.61	3.98E-01	26.07	25.33	3.03E-01	11.57	7.40	1.18E-01	41.99	27.40	1.25E-01
cg15823502	9.68	4.25	2.29E-02	4.55	6.62	4.92E-01	11.99	8.40	1.54E-01	21.38	11.33	5.93E-02	9.02	9.74	3.55E-01
cg05908480	12.47	5.48	2.29E-02	29.00	12.08	1.64E-02	22.07	31.70	4.86E-01	9.83	5.20	5.90E-02	-8.01	19.94	6.88E-01
cg20654074	16.57	7.28	2.29E-02	20.16	10.03	4.44E-02	33.84	20.28	9.52E-02	15.17	18.58	4.14E-01	-4.00	16.70	8.11E-01
cg00731062	-8.78	3.86	2.29E-02	-10.04	7.54	1.83E-01	-1.20	13.30	9.28E-01	-11.57	5.23	2.70E-02	2.18	11.59	8.51E-01
cg22544563	24.73	10.87	2.29E-02	34.23	13.74	1.27E-02	-36.38	45.15	4.20E-01	35.41	47.44	4.55E-01	13.50	21.18	5.24E-01
cg19821682	-12.60	5.54	2.29E-02	-17.15	9.03	5.75E-02	-11.95	13.13	3.63E-01	-4.26	11.34	7.07E-01	-14.52	12.19	2.33E-01
cg26159990	15.50	6.81	2.29E-02	22.14	11.70	5.85E-02	2.02	16.15	9.00E-01	14.95	12.72	2.40E-01	17.06	15.39	2.68E-01
cg17900854	13.43	5.91	2.29E-02	34.94	27.48	2.03E-01	56.46	56.52	3.18E-01	13.08	6.38	4.03E-02	-0.19	20.25	9.92E-01
cg13947528	-17.84	7.84	2.29E-02	-17.11	12.98	1.87E-01	-4.70	16.17	7.71E-01	-21.39	19.79	2.80E-01	-29.37	15.92	6.51E-02
cg03776432	-25.34	11.14	2.29E-02	-26.73	16.55	1.06E-01	-22.21	26.59	4.04E-01	-51.74	35.03	1.40E-01	-15.16	21.41	4.79E-01

cg07137416	-14.89	6.55	2.29E-02	-13.26	11.61	2.53E-01	11.58	24.67	6.39E-01	-19.92	9.45	3.51E-02	-14.68	18.04	4.16E-01
cg26992949	-9.76	4.29	2.29E-02	-20.67	9.59	3.11E-02	-17.40	18.54	3.48E-01	-7.33	5.42	1.76E-01	-0.75	12.47	9.52E-01
cg19172447	-17.26	7.59	2.29E-02	-25.81	12.40	3.73E-02	-24.14	22.77	2.89E-01	-1.16	14.80	9.37E-01	-18.33	15.15	2.26E-01
cg05869491	-9.86	4.34	2.29E-02	-18.31	7.72	1.78E-02	-2.50	10.82	8.17E-01	-6.21	7.44	4.04E-01	-8.56	10.11	3.97E-01
cg21118486	-11.53	5.07	2.30E-02	-20.48	10.31	4.70E-02	-14.95	15.52	3.36E-01	-8.57	7.00	2.21E-01	-3.87	14.18	7.85E-01
cg18131537	35.91	15.79	2.30E-02	63.69	41.71	1.27E-01	7.52	154.15	9.61E-01	37.31	18.13	3.96E-02	-18.35	53.42	7.31E-01
cg15384972	25.83	11.36	2.30E-02	32.40	18.49	7.97E-02	17.76	22.73	4.35E-01	6.95	17.79	6.96E-01	67.98	28.97	1.89E-02
cg14435720	-18.77	8.25	2.30E-02	-32.38	16.15	4.50E-02	-21.94	28.45	4.41E-01	-16.18	19.81	4.14E-01	-11.76	11.90	3.23E-01
cg02268202	-20.08	8.83	2.30E-02	-29.42	15.68	6.06E-02	-23.65	19.79	2.32E-01	1.83	17.32	9.16E-01	-29.13	18.68	1.19E-01
cg00534163	9.72	4.27	2.30E-02	13.58	6.49	3.62E-02	14.08	9.70	1.47E-01	6.13	8.77	4.85E-01	-2.73	11.66	8.15E-01
cg25556225	-5.32	2.34	2.30E-02	-6.43	3.21	4.54E-02	-2.44	4.93	6.21E-01	-4.37	9.02	6.28E-01	-6.03	5.56	2.78E-01
cg02732358	-29.20	12.84	2.30E-02	-2.25	43.17	9.58E-01	-191.60	186.67	3.05E-01	-27.09	13.92	5.16E-02	-91.17	54.69	9.55E-02
cg27078652	22.04	9.69	2.30E-02	41.86	35.12	2.33E-01	20.03	162.79	9.02E-01	23.56	10.37	2.31E-02	-38.60	44.90	3.90E-01
cg00241274	-15.03	6.61	2.30E-02	-10.04	18.68	5.91E-01	11.82	41.24	7.74E-01	-19.88	7.50	8.04E-03	18.55	24.45	4.48E-01
cg19452248	-9.93	4.37	2.30E-02	-6.31	11.01	5.67E-01	1.29	18.42	9.44E-01	-8.87	5.23	8.98E-02	-32.03	14.72	2.96E-02
cg04995826	-16.81	7.39	2.30E-02	-28.75	11.16	9.99E-03	-17.32	20.19	3.91E-01	-9.45	15.52	5.43E-01	1.34	16.51	9.35E-01
cg15235922	-12.28	5.40	2.30E-02	-28.21	11.55	1.46E-02	-8.98	16.78	5.93E-01	-7.32	7.16	3.07E-01	-9.38	16.47	5.69E-01
cg15100426	7.16	3.15	2.30E-02	6.61	4.02	9.99E-02	11.72	7.02	9.51E-02	10.48	12.94	4.18E-01	0.95	8.92	9.15E-01
cg06103086	-6.67	2.93	2.30E-02	-3.72	4.05	3.58E-01	-16.70	7.28	2.18E-02	-3.32	8.66	7.02E-01	-8.17	6.57	2.14E-01
cg00512279	19.03	8.37	2.30E-02	24.08	13.42	7.29E-02	-0.37	23.08	9.87E-01	11.27	21.48	6.00E-01	24.43	14.62	9.47E-02
cg26848724	-55.04	24.21	2.30E-02	-36.87	29.16	2.06E-01	60.31	96.83	5.33E-01	-67.94	43.76	1.21E-01	-120.49	52.14	2.09E-02
cg00349607	13.20	5.80	2.30E-02	9.22	13.48	4.94E-01	-9.86	41.94	8.14E-01	17.88	7.13	1.22E-02	-1.31	15.94	9.34E-01
cg00463562	-20.16	8.87	2.30E-02	-31.90	12.27	9.32E-03	7.13	22.87	7.55E-01	-1.49	29.12	9.59E-01	-18.88	18.29	3.02E-01
cg13339112	-38.12	16.77	2.30E-02	-31.06	39.36	4.30E-01	-79.13	148.95	5.95E-01	-42.21	19.53	3.07E-02	-5.27	64.04	9.34E-01
cg18117347	11.82	5.20	2.30E-02	7.47	6.76	2.69E-01	23.77	12.82	6.37E-02	43.31	26.11	9.72E-02	8.68	11.49	4.50E-01
cg07405810	-8.17	3.59	2.30E-02	-7.59	5.30	1.53E-01	-12.74	9.91	1.99E-01	-8.27	8.30	3.19E-01	-6.60	7.64	3.88E-01
cg02705958	5.76	2.54	2.30E-02	6.65	3.59	6.38E-02	6.19	5.65	2.73E-01	7.31	5.68	1.98E-01	-2.59	8.02	7.47E-01
cg15130433	-28.20	12.40	2.30E-02	6.93	26.20	7.92E-01	25.66	81.49	7.53E-01	-41.21	15.85	9.31E-03	-36.44	33.17	2.72E-01
cg19337593	42.64	18.76	2.30E-02	64.83	42.87	1.30E-01	-107.58	181.23	5.53E-01	39.21	22.79	8.53E-02	40.02	54.02	4.59E-01
cg19960616	-7.53	3.31	2.30E-02	-4.46	6.00	4.57E-01	-13.30	12.07	2.71E-01	-10.58	4.75	2.58E-02	-0.14	9.07	9.88E-01
cg10220544	5.38	2.37	2.30E-02	6.45	3.33	5.24E-02	6.14	6.70	3.59E-01	7.33	4.84	1.30E-01	-3.18	6.61	6.30E-01
cg18660064	8.09	3.56	2.30E-02	8.81	4.58	5.47E-02	15.20	9.72	1.18E-01	10.41	14.25	4.65E-01	0.46	7.95	9.54E-01
cg00552600	34.04	14.97	2.30E-02	1.67	16.14	9.18E-01	60.88	18.98	1.34E-03	26.07	22.16	2.39E-01	54.07	23.06	1.90E-02
cg13793048	22.41	9.86	2.30E-02	13.08	15.70	4.05E-01	34.26	19.60	8.04E-02	30.51	21.88	1.63E-01	15.96	25.50	5.31E-01
cg04857033	10.70	4.71	2.30E-02	16.18	6.86	1.83E-02	5.45	10.35	5.99E-01	9.65	14.23	4.97E-01	4.19	10.22	6.82E-01

cg18416950	10.09	4.44	2.30E-02	13.14	7.16	6.65E-02	6.70	17.12	6.95E-01	9.64	6.81	1.57E-01	4.00	12.60	7.51E-01
cg07455975	38.06	16.74	2.30E-02	47.13	22.17	3.35E-02	24.99	51.51	6.28E-01	-1.77	44.95	9.69E-01	47.42	38.89	2.23E-01
cg04983276	-8.10	3.56	2.30E-02	-6.01	5.27	2.53E-01	-0.25	8.77	9.77E-01	-13.57	10.18	1.83E-01	-14.34	7.07	4.26E-02
cg18678763	17.35	7.63	2.30E-02	14.21	10.41	1.72E-01	30.57	19.57	1.18E-01	-4.91	24.06	8.38E-01	26.52	16.68	1.12E-01
cg05424422	-11.54	5.08	2.30E-02	-16.21	8.69	6.20E-02	-2.14	10.86	8.44E-01	-7.13	10.62	5.02E-01	-18.52	11.07	9.43E-02
cg11252555	-11.56	5.09	2.31E-02	-18.93	7.77	1.48E-02	-3.50	15.57	8.22E-01	-11.70	12.59	3.53E-01	-3.85	9.27	6.78E-01
cg12363722	8.56	3.77	2.31E-02	14.41	6.83	3.48E-02	6.76	8.96	4.51E-01	8.62	7.67	2.61E-01	3.25	7.14	6.48E-01
cg12126243	-7.91	3.48	2.31E-02	-14.55	8.37	8.19E-02	-12.14	16.89	4.72E-01	-6.95	4.30	1.06E-01	-2.47	9.71	7.99E-01
cg08276755	8.06	3.55	2.31E-02	6.79	5.25	1.96E-01	8.77	9.64	3.63E-01	12.72	6.90	6.52E-02	2.83	9.38	7.63E-01
cg23511169	24.13	10.62	2.31E-02	51.76	33.67	1.24E-01	4.85	149.14	9.74E-01	21.00	11.58	6.97E-02	23.84	45.67	6.02E-01
cg17664182	-13.90	6.12	2.31E-02	-22.60	9.45	1.68E-02	-2.92	13.97	8.34E-01	0.81	16.61	9.61E-01	-15.69	12.15	1.97E-01
cg17148976	12.13	5.34	2.31E-02	8.14	8.21	3.22E-01	28.74	12.40	2.04E-02	17.06	10.60	1.07E-01	1.26	10.46	9.04E-01
cg03944810	32.79	14.43	2.31E-02	39.07	16.19	1.58E-02	-5.61	92.87	9.52E-01	45.07	50.73	3.74E-01	-17.56	45.55	7.00E-01
cg13820205	-12.21	5.38	2.31E-02	-2.23	9.79	8.20E-01	-13.23	12.52	2.91E-01	-19.05	9.47	4.43E-02	-15.45	12.26	2.08E-01
cg05371993	27.54	12.12	2.31E-02	20.05	14.69	1.72E-01	-15.07	64.12	8.14E-01	73.07	31.62	2.08E-02	27.02	32.84	4.11E-01
cg08586228	-37.06	16.31	2.31E-02	-28.01	20.25	1.67E-01	-40.08	97.48	6.81E-01	-101.44	66.41	1.27E-01	-44.31	31.82	1.64E-01
cg05879498	16.60	7.31	2.31E-02	14.60	10.62	1.69E-01	54.85	26.90	4.14E-02	31.61	25.41	2.13E-01	8.19	12.00	4.95E-01
cg23611220	-12.58	5.54	2.31E-02	-24.92	14.00	7.51E-02	-14.61	18.88	4.39E-01	-9.06	6.81	1.84E-01	-14.91	17.83	4.03E-01
cg12149299	10.89	4.79	2.31E-02	13.77	7.05	5.07E-02	18.00	11.81	1.28E-01	8.83	10.65	4.07E-01	-1.35	11.60	9.07E-01
cg22744555	12.96	5.70	2.31E-02	15.82	16.51	3.38E-01	17.82	21.22	4.01E-01	10.63	6.55	1.05E-01	34.09	25.35	1.79E-01
cg01073765	9.77	4.30	2.31E-02	7.55	8.45	3.72E-01	19.39	10.20	5.73E-02	6.49	6.50	3.18E-01	12.13	12.10	3.16E-01
cg21908960	-10.47	4.61	2.31E-02	-12.64	10.03	2.08E-01	-15.96	18.65	3.92E-01	-11.39	5.99	5.73E-02	-0.63	12.51	9.60E-01
cg14179383	-12.94	5.70	2.31E-02	-23.62	9.32	1.12E-02	-7.51	19.43	6.99E-01	-3.53	9.53	7.11E-01	-12.07	13.33	3.65E-01
cg02568541	-69.49	30.59	2.31E-02	-36.76	41.63	3.77E-01	22.38	138.55	8.72E-01	-159.53	67.97	1.89E-02	-88.19	66.92	1.88E-01
cg11299459	9.89	4.35	2.31E-02	16.79	6.19	6.69E-03	4.29	13.87	7.57E-01	1.59	9.12	8.61E-01	4.49	10.29	6.63E-01
cg24338091	15.49	6.82	2.31E-02	15.64	10.04	1.19E-01	21.42	13.51	1.13E-01	-6.04	18.80	7.48E-01	23.71	17.48	1.75E-01
cg26306329	8.87	3.90	2.31E-02	7.49	5.07	1.40E-01	17.04	10.20	9.50E-02	21.71	12.74	8.83E-02	-0.65	9.57	9.46E-01
cg14972827	-9.74	4.29	2.31E-02	-20.89	9.18	2.29E-02	4.59	16.63	7.83E-01	-10.09	5.01	4.39E-02	1.47	11.30	8.96E-01
cg19750643	-13.76	6.06	2.31E-02	-24.78	10.01	1.33E-02	-5.54	12.79	6.65E-01	-7.82	13.24	5.55E-01	-8.98	13.54	5.07E-01
cg23275998	40.66	17.90	2.31E-02	22.39	23.17	3.34E-01	58.08	70.16	4.08E-01	83.59	58.71	1.54E-01	64.29	36.19	7.56E-02
cg10531104	-10.65	4.69	2.31E-02	-25.07	9.03	5.50E-03	-10.06	12.77	4.31E-01	-5.93	5.38	2.71E-01	-4.93	11.57	6.70E-01
cg02683759	16.30	7.18	2.31E-02	28.60	15.08	5.79E-02	16.13	19.24	4.02E-01	14.29	13.28	2.82E-01	9.95	12.27	4.17E-01
cg23722592	-12.36	5.44	2.31E-02	-3.03	14.14	8.31E-01	-11.03	24.15	6.48E-01	-12.45	6.43	5.29E-02	-28.33	18.52	1.26E-01
cg16392213	20.94	9.22	2.31E-02	48.95	30.70	1.11E-01	21.79	48.07	6.50E-01	21.38	10.93	5.04E-02	3.09	22.99	8.93E-01
cg20742981	16.78	7.39	2.31E-02	28.02	9.56	3.38E-03	9.12	19.97	6.48E-01	25.07	13.67	6.68E-02	0.27	11.02	9.81E-01

cg21277995	39.32	17.31	2.31E-02	63.36	42.21	1.33E-01	236.29	218.29	2.79E-01	33.92	20.23	9.36E-02	25.04	56.68	6.59E-01
cg21865157	70.91	31.22	2.31E-02	81.25	44.54	6.81E-02	271.93	154.73	7.88E-02	24.12	88.91	7.86E-01	49.15	53.19	3.55E-01
cg10552523	-8.58	3.78	2.31E-02	-3.80	5.05	4.52E-01	-12.30	9.11	1.77E-01	-21.08	15.46	1.73E-01	-14.67	8.24	7.48E-02
cg00377681	-12.10	5.33	2.32E-02	-12.66	7.78	1.04E-01	-14.01	13.27	2.91E-01	-7.44	12.89	5.64E-01	-13.24	11.96	2.68E-01
cg07926644	20.17	8.88	2.32E-02	45.05	18.55	1.52E-02	21.64	18.85	2.51E-01	7.59	13.17	5.65E-01	16.85	28.89	5.60E-01
cg03111404	12.12	5.34	2.32E-02	10.67	7.95	1.79E-01	13.25	12.41	2.86E-01	17.39	17.99	3.34E-01	12.05	10.16	2.36E-01
cg08332148	17.69	7.79	2.32E-02	20.57	12.77	1.07E-01	15.35	13.55	2.57E-01	7.35	17.53	6.75E-01	35.21	24.68	1.54E-01
cg07127945	18.74	8.25	2.32E-02	29.76	18.58	1.09E-01	64.47	89.66	4.72E-01	14.12	10.69	1.86E-01	19.71	18.57	2.89E-01
cg10043090	10.32	4.54	2.32E-02	13.58	6.96	5.09E-02	13.95	9.61	1.47E-01	5.44	11.26	6.29E-01	2.79	10.49	7.91E-01
cg27666123	-10.22	4.50	2.32E-02	-10.38	12.32	3.99E-01	4.55	21.84	8.35E-01	-12.43	5.28	1.85E-02	0.12	14.47	9.93E-01
cg02606728	-34.78	15.32	2.32E-02	-2.21	27.56	9.36E-01	-66.13	37.03	7.41E-02	-50.24	26.61	5.91E-02	-32.55	35.28	3.56E-01
cg02185248	8.09	3.56	2.32E-02	9.32	5.17	7.16E-02	18.43	9.29	4.72E-02	-3.10	10.76	7.73E-01	4.82	6.87	4.83E-01
cg15061682	-11.69	5.15	2.32E-02	-14.79	10.37	1.54E-01	-11.30	13.61	4.06E-01	-11.96	7.65	1.18E-01	-6.40	12.98	6.22E-01
cg18396987	-6.07	2.67	2.32E-02	-8.65	4.24	4.14E-02	-7.05	5.45	1.96E-01	0.01	7.66	9.99E-01	-3.90	5.47	4.76E-01
cg04141610	27.43	12.08	2.32E-02	36.88	35.37	2.97E-01	-216.85	157.12	1.68E-01	27.23	13.59	4.52E-02	33.19	40.91	4.17E-01
cg13628971	20.33	8.96	2.32E-02	42.16	28.38	1.37E-01	15.32	110.32	8.90E-01	19.37	9.80	4.80E-02	-2.60	37.01	9.44E-01
cg26558485	9.50	4.19	2.32E-02	17.83	7.36	1.54E-02	8.48	9.57	3.76E-01	6.08	9.68	5.30E-01	3.28	7.66	6.69E-01
cg24667575	7.03	3.10	2.32E-02	7.39	4.19	7.77E-02	8.52	6.22	1.71E-01	0.48	12.16	9.69E-01	6.04	8.27	4.65E-01
cg17229388	10.02	4.41	2.32E-02	15.29	5.38	4.50E-03	-6.15	12.47	6.22E-01	4.68	6.24	4.53E-01	18.13	9.30	5.12E-02
cg26581504	-17.72	7.80	2.32E-02	-27.64	20.72	1.82E-01	20.73	27.93	4.58E-01	-17.39	9.27	6.05E-02	-43.47	29.34	1.38E-01
cg24126567	11.86	5.23	2.32E-02	11.99	10.43	2.50E-01	18.47	8.68	3.33E-02	-2.83	11.13	7.99E-01	16.74	12.82	1.92E-01
cg01808739	14.46	6.37	2.32E-02	15.55	7.58	4.03E-02	8.84	20.77	6.70E-01	21.09	31.12	4.98E-01	11.17	15.99	4.85E-01
cg15034464	-49.74	21.91	2.32E-02	-110.87	47.37	1.93E-02	-245.34	209.36	2.41E-01	-33.65	23.63	1.54E-01	-22.71	57.15	6.91E-01
cg09040752	18.41	8.11	2.32E-02	17.62	12.13	1.47E-01	35.08	15.53	2.39E-02	-4.99	21.55	8.17E-01	12.07	21.76	5.79E-01
cg03668470	-22.89	10.09	2.32E-02	-32.03	14.75	2.99E-02	21.45	37.47	5.67E-01	-32.39	25.36	2.02E-01	-14.42	18.36	4.32E-01
cg16572315	-50.87	22.41	2.32E-02	-36.15	28.48	2.04E-01	-145.61	106.57	1.72E-01	35.34	85.21	6.78E-01	-91.53	43.33	3.46E-02
cg04330597	-15.47	6.82	2.32E-02	-11.06	11.85	3.51E-01	-2.16	18.43	9.07E-01	-23.85	13.85	8.52E-02	-19.79	12.65	1.18E-01
cg12185873	11.15	4.91	2.32E-02	8.06	6.49	2.15E-01	16.74	11.14	1.33E-01	8.93	25.20	7.23E-01	15.15	11.16	1.75E-01
cg21208734	-14.36	6.33	2.32E-02	-9.09	9.67	3.47E-01	-18.60	15.64	2.34E-01	-36.73	16.34	2.46E-02	-7.42	12.46	5.52E-01
cg27493117	21.58	9.51	2.32E-02	46.25	22.41	3.90E-02	26.64	23.75	2.62E-01	10.30	12.64	4.15E-01	33.66	31.05	2.78E-01
cg13821914	30.90	13.62	2.32E-02	40.84	21.73	6.01E-02	30.65	27.13	2.59E-01	-6.99	34.60	8.40E-01	41.00	30.41	1.77E-01
cg08097359	13.68	6.03	2.32E-02	14.35	7.93	7.04E-02	38.59	17.98	3.19E-02	17.35	17.68	3.27E-01	1.69	10.73	8.75E-01
cg23460430	11.92	5.25	2.32E-02	10.35	7.42	1.63E-01	7.34	11.15	5.10E-01	31.47	17.64	7.44E-02	12.28	12.12	3.11E-01
cg06581409	9.76	4.30	2.32E-02	13.41	9.96	1.78E-01	17.91	15.64	2.52E-01	5.76	5.47	2.92E-01	19.70	12.48	1.14E-01
cg24947064	-16.19	7.13	2.33E-02	-3.40	21.31	8.73E-01	13.31	34.74	7.02E-01	-18.39	7.99	2.13E-02	-34.98	32.26	2.78E-01

cg23660123	-10.35	4.56	2.33E-02	-7.98	9.50	4.01E-01	-11.47	8.68	1.86E-01	-11.49	7.15	1.08E-01	-7.71	15.46	6.18E-01
cg06624527	32.44	14.29	2.33E-02	54.87	15.47	3.88E-04	55.38	26.46	3.64E-02	4.00	10.47	7.02E-01	29.69	17.56	9.09E-02
cg19978669	-31.39	13.83	2.33E-02	-29.24	15.81	6.44E-02	52.93	84.88	5.33E-01	37.44	75.64	6.21E-01	-67.01	33.17	4.34E-02
cg19535267	20.49	9.03	2.33E-02	17.20	22.68	4.48E-01	15.92	24.35	5.13E-01	15.47	11.91	1.94E-01	51.73	25.12	3.95E-02
cg18395675	28.41	12.52	2.33E-02	24.84	16.88	1.41E-01	-21.63	37.28	5.62E-01	48.25	31.96	1.31E-01	53.33	29.25	6.82E-02
cg08075469	-55.80	24.59	2.33E-02	-77.83	31.90	1.47E-02	-111.03	104.17	2.86E-01	35.96	64.33	5.76E-01	-42.28	54.46	4.38E-01
cg13428152	25.86	11.40	2.33E-02	28.69	17.13	9.40E-02	3.70	31.19	9.06E-01	51.06	30.67	9.60E-02	19.66	21.30	3.56E-01
cg05590818	7.58	3.34	2.33E-02	9.37	4.80	5.10E-02	11.16	7.82	1.53E-01	9.72	11.10	3.81E-01	0.50	6.80	9.41E-01
cg22395573	-40.09	17.67	2.33E-02	-50.38	22.26	2.36E-02	-95.60	61.77	1.22E-01	34.70	64.74	5.92E-01	-14.59	38.22	7.03E-01
cg21053741	14.51	6.40	2.33E-02	12.65	9.19	1.69E-01	34.27	14.57	1.86E-02	5.35	15.60	7.32E-01	5.67	16.28	7.27E-01
cg07639472	-30.86	13.60	2.33E-02	14.79	45.52	7.45E-01	-247.51	167.32	1.39E-01	-32.43	14.66	2.69E-02	-61.02	65.75	3.53E-01
cg16852115	34.87	15.37	2.33E-02	41.00	18.11	2.36E-02	109.70	90.57	2.26E-01	71.24	92.26	4.40E-01	0.90	32.53	9.78E-01
cg00830435	-38.41	16.93	2.33E-02	-27.05	16.90	1.09E-01	-0.17	40.37	9.97E-01	-94.22	29.50	1.40E-03	-29.19	25.22	2.47E-01
cg10251641	-43.93	19.37	2.33E-02	-51.28	28.24	6.94E-02	-46.69	63.90	4.65E-01	-17.67	43.67	6.86E-01	-49.96	39.42	2.05E-01
cg19108432	34.10	15.03	2.33E-02	8.74	37.52	8.16E-01	-84.73	179.88	6.38E-01	41.16	17.40	1.80E-02	29.72	51.18	5.61E-01
cg21144120	-6.75	2.97	2.33E-02	-10.47	4.33	1.55E-02	-2.47	6.67	7.11E-01	-7.28	8.61	3.98E-01	-2.09	6.51	7.48E-01
cg13973472	15.02	6.62	2.33E-02	8.92	7.55	2.38E-01	37.83	54.20	4.85E-01	34.70	15.90	2.91E-02	36.73	31.88	2.49E-01
cg23214628	9.74	4.29	2.33E-02	5.99	6.23	3.37E-01	10.29	7.84	1.90E-01	9.44	18.22	6.04E-01	19.40	10.43	6.29E-02
cg18109530	-9.29	4.09	2.33E-02	-14.95	10.23	1.44E-01	0.42	13.43	9.75E-01	-7.85	5.03	1.19E-01	-20.62	14.13	1.45E-01
cg01807131	-12.17	5.37	2.33E-02	-17.00	8.27	3.98E-02	-13.02	12.18	2.85E-01	-0.68	12.94	9.58E-01	-11.12	11.64	3.39E-01
cg14592491	30.95	13.65	2.33E-02	21.99	25.87	3.95E-01	-138.95	158.70	3.81E-01	39.54	17.01	2.01E-02	5.86	51.32	9.09E-01
cg05236705	-14.48	6.39	2.33E-02	-24.01	11.15	3.13E-02	-6.91	26.65	7.95E-01	-5.76	9.54	5.46E-01	-21.78	15.63	1.63E-01
cg19899882	31.16	13.74	2.33E-02	41.09	21.74	5.88E-02	-0.40	42.99	9.92E-01	28.34	27.62	3.05E-01	30.99	27.41	2.58E-01
cg06499778	-11.46	5.05	2.33E-02	-18.60	8.06	2.10E-02	-6.98	11.74	5.52E-01	0.62	11.42	9.56E-01	-13.17	10.63	2.15E-01
cg03978514	15.31	6.75	2.33E-02	13.79	9.74	1.57E-01	15.87	12.69	2.11E-01	7.37	21.18	7.28E-01	25.47	18.34	1.65E-01
cg24950817	23.21	10.24	2.33E-02	9.34	17.81	6.00E-01	24.66	20.16	2.21E-01	28.72	19.56	1.42E-01	42.79	27.55	1.20E-01
cg26663590	6.74	2.97	2.33E-02	4.90	3.85	2.03E-01	10.64	6.64	1.09E-01	12.30	8.87	1.65E-01	3.34	9.95	7.37E-01
cg13424393	4.79	2.11	2.34E-02	4.01	3.07	1.92E-01	7.94	4.89	1.04E-01	5.43	4.18	1.94E-01	0.31	7.21	9.66E-01
cg13206920	19.95	8.80	2.34E-02	-9.35	23.23	6.87E-01	61.35	54.00	2.56E-01	18.68	11.86	1.15E-01	33.58	16.65	4.38E-02
cg16542392	-25.81	11.38	2.34E-02	21.10	32.61	5.18E-01	-57.84	147.87	6.96E-01	-34.31	12.74	7.10E-03	-9.02	41.59	8.28E-01
cg20780180	-7.76	3.42	2.34E-02	-9.21	6.68	1.68E-01	-5.29	11.41	6.43E-01	-5.94	4.82	2.18E-01	-13.15	9.08	1.48E-01
cg04476286	19.28	8.50	2.34E-02	36.63	17.10	3.22E-02	10.99	45.35	8.08E-01	17.78	10.94	1.04E-01	-7.90	25.21	7.54E-01
cg25678532	35.42	15.62	2.34E-02	34.34	33.35	3.03E-01	49.86	99.72	6.17E-01	36.86	19.46	5.82E-02	26.06	46.68	5.77E-01
cg20340866	14.98	6.61	2.34E-02	9.52	15.99	5.52E-01	-6.47	19.94	7.45E-01	20.78	9.24	2.45E-02	16.54	14.50	2.54E-01
cg08644276	-6.57	2.90	2.34E-02	-9.29	5.65	1.00E-01	-6.57	9.13	4.72E-01	-5.82	4.06	1.52E-01	-3.95	8.14	6.28E-01

cg08696316	-24.43	10.77	2.34E-02	-28.74	16.54	8.23E-02	1.26	31.41	9.68E-01	-55.68	29.50	5.91E-02	-15.26	18.91	4.20E-01
cg14911393	-9.01	3.97	2.34E-02	-13.28	10.53	2.07E-01	-27.13	20.66	1.89E-01	-6.45	4.66	1.66E-01	-15.27	13.03	2.41E-01
cg26216433	8.29	3.66	2.34E-02	12.42	5.50	2.39E-02	5.30	9.09	5.60E-01	12.65	8.47	1.35E-01	-1.99	7.98	8.03E-01
cg23004758	13.86	6.11	2.34E-02	19.03	9.69	4.95E-02	10.12	14.63	4.89E-01	17.02	21.63	4.31E-01	9.09	10.37	3.81E-01
cg02881374	-15.91	7.02	2.34E-02	-7.92	19.02	6.77E-01	-29.15	27.48	2.89E-01	-16.31	8.18	4.63E-02	-14.76	27.79	5.95E-01
cg07382347	2.96	1.31	2.34E-02	2.08	1.88	2.69E-01	5.25	2.63	4.55E-02	1.46	4.32	7.34E-01	2.95	3.10	3.41E-01
cg06647382	-27.92	12.32	2.34E-02	-5.28	23.29	8.21E-01	-16.80	55.22	7.61E-01	-46.28	15.26	2.43E-03	0.78	33.31	9.81E-01
cg20543426	-9.80	4.32	2.34E-02	-17.70	6.25	4.64E-03	-4.75	11.28	6.74E-01	-0.16	11.09	9.89E-01	-3.66	8.75	6.76E-01
cg07939503	23.42	10.33	2.34E-02	10.23	18.99	5.90E-01	31.79	21.50	1.39E-01	15.70	8.62	6.84E-02	77.81	31.08	1.23E-02
cg01966117	11.93	5.26	2.34E-02	14.79	7.65	5.33E-02	24.42	11.76	3.79E-02	-2.06	14.82	8.90E-01	1.53	11.73	8.96E-01
cg17505469	-7.56	3.34	2.34E-02	-6.82	4.85	1.59E-01	-8.37	7.55	2.68E-01	3.01	9.99	7.63E-01	-13.80	7.12	5.26E-02
cg01153737	21.10	9.31	2.34E-02	21.10	17.93	2.39E-01	15.97	16.87	3.44E-01	19.55	20.80	3.47E-01	29.40	19.60	1.34E-01
cg01394324	21.45	9.46	2.34E-02	49.15	17.50	4.98E-03	12.01	17.63	4.96E-01	9.40	15.21	5.36E-01	17.02	21.33	4.25E-01
cg26935168	13.48	5.95	2.34E-02	7.05	8.87	4.27E-01	18.08	13.37	1.76E-01	4.66	18.42	8.00E-01	25.17	11.93	3.50E-02
cg03715931	30.47	13.44	2.34E-02	49.60	25.78	5.44E-02	0.37	67.48	9.96E-01	31.77	18.61	8.79E-02	2.40	32.90	9.42E-01
cg08310224	19.73	8.70	2.34E-02	9.88	16.75	5.55E-01	16.18	18.37	3.78E-01	36.65	13.75	7.69E-03	-7.00	24.98	7.79E-01
cg10416622	-31.89	14.07	2.34E-02	-44.39	28.59	1.21E-01	-182.29	128.23	1.55E-01	-27.38	17.97	1.28E-01	-16.29	38.57	6.73E-01
cg07081477	11.04	4.87	2.34E-02	12.28	6.25	4.95E-02	8.75	17.47	6.16E-01	4.25	17.50	8.08E-01	10.84	10.01	2.78E-01
cg02708922	45.07	19.89	2.34E-02	51.40	32.76	1.17E-01	32.14	52.01	5.37E-01	30.17	38.43	4.32E-01	61.40	42.64	1.50E-01
cg12054341	14.76	6.51	2.34E-02	18.94	20.41	3.54E-01	58.64	37.78	1.21E-01	10.36	7.64	1.75E-01	25.25	17.36	1.46E-01
cg02774129	-8.00	3.53	2.34E-02	-8.89	5.29	9.32E-02	-12.86	7.53	8.77E-02	-3.99	9.48	6.74E-01	-3.40	7.95	6.69E-01
cg16579555	50.34	22.21	2.34E-02	52.73	62.29	3.97E-01	-3.02	220.53	9.89E-01	63.53	25.94	1.43E-02	-22.67	61.79	7.14E-01
cg19643545	-10.55	4.66	2.34E-02	-7.16	9.11	4.32E-01	-10.52	12.79	4.11E-01	-9.55	6.89	1.66E-01	-19.58	12.04	1.04E-01
cg07147573	20.45	9.02	2.34E-02	-2.79	24.54	9.09E-01	24.77	37.08	5.04E-01	20.62	10.64	5.25E-02	52.57	30.79	8.77E-02
cg24145007	16.11	7.11	2.34E-02	14.34	20.38	4.82E-01	35.31	32.61	2.79E-01	15.31	8.16	6.07E-02	14.80	26.36	5.75E-01
cg21279353	10.89	4.81	2.34E-02	8.41	13.17	5.23E-01	-8.58	23.61	7.16E-01	10.29	5.50	6.13E-02	36.91	19.40	5.71E-02
cg02470736	-60.66	26.77	2.34E-02	-112.35	65.65	8.70E-02	-9.59	319.33	9.76E-01	-68.84	33.91	4.24E-02	4.78	59.30	9.36E-01
cg03759556	-19.60	8.65	2.34E-02	-12.73	20.45	5.34E-01	-31.74	30.94	3.05E-01	-18.31	10.78	8.94E-02	-30.84	27.49	2.62E-01
cg16247184	-8.89	3.92	2.35E-02	-11.65	7.56	1.23E-01	-10.05	8.95	2.62E-01	-4.90	6.31	4.37E-01	-12.67	10.07	2.08E-01
cg00148862	7.42	3.27	2.35E-02	4.94	4.93	3.16E-01	7.38	6.56	2.61E-01	21.62	11.30	5.58E-02	7.03	6.89	3.08E-01
cg05719902	7.12	3.14	2.35E-02	3.71	4.47	4.06E-01	5.55	8.60	5.19E-01	5.94	8.73	4.96E-01	15.47	6.36	1.49E-02
cg05328761	-13.25	5.85	2.35E-02	-24.94	14.75	9.08E-02	-21.97	28.56	4.42E-01	-9.01	6.88	1.90E-01	-24.20	20.88	2.46E-01
cg26804057	-19.23	8.49	2.35E-02	-17.81	22.68	4.32E-01	-30.44	42.37	4.72E-01	-24.06	9.84	1.45E-02	30.99	30.69	3.13E-01
cg04077662	21.11	9.32	2.35E-02	10.76	25.82	6.77E-01	-17.58	60.49	7.71E-01	30.93	11.20	5.75E-03	-8.30	23.75	7.27E-01
cg12501220	11.40	5.03	2.35E-02	8.07	8.20	3.25E-01	20.79	9.56	2.97E-02	8.67	14.26	5.43E-01	6.86	10.66	5.20E-01

cg26944301	32.92	14.53	2.35E-02	19.11	18.37	2.98E-01	13.34	49.13	7.86E-01	55.05	43.17	2.02E-01	77.98	34.83	2.52E-02
cg04813787	-8.76	3.86	2.35E-02	-9.15	5.69	1.08E-01	-8.87	9.55	3.53E-01	-8.14	9.86	4.09E-01	-8.28	8.20	3.13E-01
cg05249836	5.48	2.42	2.35E-02	5.29	3.19	9.72E-02	9.50	5.09	6.17E-02	6.52	8.00	4.15E-01	-2.76	7.31	7.06E-01
cg09730170	-12.00	5.29	2.35E-02	-8.15	11.18	4.66E-01	-10.59	14.76	4.73E-01	-14.47	7.35	4.89E-02	-10.10	14.79	4.94E-01
cg10193091	-16.87	7.45	2.35E-02	-38.52	18.32	3.55E-02	-8.81	35.17	8.02E-01	-12.38	8.83	1.61E-01	-16.60	26.55	5.32E-01
cg26014036	15.27	6.74	2.35E-02	19.71	7.86	1.22E-02	3.05	13.73	8.24E-01	53.39	25.09	3.34E-02	8.24	10.46	4.31E-01
cg09022808	-13.17	5.81	2.35E-02	-16.50	10.05	1.01E-01	-6.69	12.95	6.05E-01	-14.71	11.65	2.07E-01	-12.27	12.53	3.27E-01
cg20151401	-3.76	1.66	2.35E-02	-3.54	2.27	1.19E-01	-1.06	3.73	7.76E-01	-7.37	7.05	2.96E-01	-5.88	3.60	1.02E-01
cg16547579	8.69	3.83	2.35E-02	11.21	5.50	4.14E-02	10.92	10.69	3.07E-01	9.83	13.35	4.62E-01	3.36	6.98	6.30E-01
cg19980199	16.86	7.44	2.35E-02	30.16	12.80	1.85E-02	10.12	15.31	5.08E-01	10.98	14.03	4.34E-01	8.21	19.61	6.75E-01
cg07853992	23.38	10.32	2.35E-02	57.29	21.37	7.33E-03	-4.49	91.98	9.61E-01	15.95	8.78	6.94E-02	9.52	33.11	7.74E-01
cg21751058	27.76	12.25	2.35E-02	22.34	14.65	1.27E-01	-10.00	50.20	8.42E-01	16.42	70.48	8.16E-01	57.97	26.67	2.97E-02
cg12255995	-42.60	18.80	2.35E-02	-39.51	23.85	9.76E-02	-117.67	57.36	4.02E-02	-0.63	65.76	9.92E-01	-28.23	43.23	5.14E-01
cg17499345	-16.79	7.41	2.35E-02	-8.52	16.64	6.09E-01	0.84	16.14	9.58E-01	-27.31	10.60	1.00E-02	-18.96	23.22	4.14E-01
cg20537886	-10.68	4.71	2.35E-02	-10.10	7.10	1.55E-01	-9.90	10.24	3.33E-01	-11.25	12.03	3.50E-01	-12.38	10.70	2.47E-01
cg13709244	38.58	17.03	2.35E-02	94.15	43.75	3.14E-02	-73.54	179.05	6.81E-01	36.13	16.80	3.15E-02	-22.67	57.98	6.96E-01
cg00096307	-11.99	5.30	2.35E-02	-12.55	13.36	3.47E-01	-3.71	29.02	8.98E-01	-10.29	6.26	1.00E-01	-26.94	17.25	1.18E-01
cg12633154	2.86	1.26	2.35E-02	1.65	1.76	3.50E-01	4.05	2.49	1.04E-01	5.18	4.38	2.37E-01	3.74	3.31	2.59E-01
cg01291544	37.93	16.75	2.35E-02	38.50	25.03	1.24E-01	77.09	36.14	3.29E-02	34.48	38.03	3.65E-01	-17.80	44.23	6.87E-01
cg17694130	-6.25	2.76	2.35E-02	-5.06	3.93	1.98E-01	-5.03	7.25	4.87E-01	-10.04	7.73	1.94E-01	-7.45	5.70	1.92E-01
cg04421891	-11.98	5.29	2.35E-02	-23.54	11.20	3.55E-02	-8.68	18.96	6.47E-01	-7.72	6.88	2.62E-01	-13.84	16.12	3.91E-01
cg04927066	-11.63	5.14	2.35E-02	-4.12	13.52	7.61E-01	-19.68	17.82	2.69E-01	-12.27	6.14	4.57E-02	-11.22	19.00	5.55E-01
cg16587725	-5.88	2.60	2.35E-02	-2.66	4.55	5.59E-01	-9.13	6.21	1.42E-01	-9.63	4.63	3.76E-02	-2.11	6.04	7.27E-01
cg21821214	18.60	8.21	2.35E-02	23.99	12.92	6.32E-02	-3.26	21.43	8.79E-01	21.95	19.45	2.59E-01	20.19	15.78	2.01E-01
cg02232377	-7.02	3.10	2.35E-02	-7.93	5.68	1.63E-01	-8.69	7.37	2.38E-01	-10.07	5.21	5.31E-02	2.68	7.52	7.22E-01
cg08244301	7.39	3.26	2.35E-02	8.56	4.12	3.79E-02	10.87	8.06	1.78E-01	4.62	12.00	7.00E-01	-0.67	8.83	9.39E-01
cg20950646	14.45	6.38	2.35E-02	19.77	8.99	2.78E-02	-3.78	15.03	8.01E-01	16.14	18.62	3.86E-01	16.45	14.31	2.50E-01
cg15381609	-16.68	7.36	2.35E-02	-24.80	21.35	2.45E-01	-44.46	28.56	1.20E-01	-13.92	8.48	1.01E-01	-4.46	30.05	8.82E-01
cg08085853	-12.21	5.39	2.35E-02	-27.11	12.87	3.52E-02	-11.90	25.81	6.45E-01	-7.82	6.46	2.27E-01	-17.48	18.40	3.42E-01
cg02743674	-12.77	5.64	2.35E-02	-12.30	7.72	1.11E-01	-13.15	12.79	3.04E-01	-4.01	17.86	8.22E-01	-18.86	13.58	1.65E-01
cg01907688	26.15	11.55	2.35E-02	47.18	31.09	1.29E-01	131.24	151.22	3.85E-01	23.17	13.15	7.81E-02	11.88	39.49	7.64E-01
cg19522185	-17.36	7.67	2.35E-02	-5.00	18.62	7.88E-01	-5.92	42.15	8.88E-01	-21.69	9.06	1.67E-02	-9.65	26.93	7.20E-01
cg21744723	49.45	21.84	2.36E-02	-37.93	56.85	5.05E-01	35.65	218.23	8.70E-01	68.23	23.27	3.37E-03	30.93	74.12	6.76E-01
cg26530275	-10.56	4.66	2.36E-02	-5.69	11.69	6.26E-01	-38.74	23.85	1.04E-01	-11.33	5.55	4.14E-02	-1.89	14.91	8.99E-01
cg12102573	23.24	10.26	2.36E-02	12.59	22.21	5.71E-01	42.62	19.88	3.21E-02	6.42	16.94	7.05E-01	44.74	26.25	8.83E-02

cg05805736	-43.42	19.18	2.36E-02	-37.93	23.23	1.03E-01	-158.81	95.12	9.50E-02	-48.21	70.68	4.95E-01	-37.08	42.45	3.82E-01
cg04136490	-11.65	5.14	2.36E-02	-23.20	10.95	3.41E-02	-22.08	15.15	1.45E-01	-4.75	7.26	5.13E-01	-9.84	12.78	4.41E-01
cg09447217	-16.01	7.07	2.36E-02	2.06	15.48	8.94E-01	-3.65	32.11	9.10E-01	-20.93	8.96	1.95E-02	-26.92	20.42	1.87E-01
cg09455182	-53.96	23.83	2.36E-02	-37.47	46.87	4.24E-01	4.13	231.96	9.86E-01	-79.26	31.05	1.07E-02	16.76	63.31	7.91E-01
cg12171761	-8.72	3.85	2.36E-02	-8.16	5.18	1.15E-01	-4.75	10.14	6.40E-01	-4.95	9.68	6.09E-01	-18.90	10.11	6.16E-02
cg23058177	29.11	12.86	2.36E-02	8.06	32.19	8.02E-01	-8.39	100.88	9.34E-01	36.87	16.98	2.99E-02	27.19	25.68	2.90E-01
cg14041283	23.07	10.19	2.36E-02	36.39	18.08	4.41E-02	17.03	31.27	5.86E-01	10.11	15.92	5.25E-01	33.42	24.99	1.81E-01
cg04272632	22.85	10.10	2.36E-02	44.78	22.38	4.54E-02	15.23	45.86	7.40E-01	16.90	12.96	1.92E-01	19.47	26.89	4.69E-01
cg25272065	30.33	13.40	2.36E-02	22.71	16.52	1.69E-01	29.36	83.96	7.27E-01	80.95	46.19	7.97E-02	33.69	27.77	2.25E-01
cg09322984	47.15	20.83	2.36E-02	14.21	19.99	4.77E-01	50.66	49.44	3.05E-01	93.00	45.64	4.16E-02	81.55	38.73	3.52E-02
cg10142436	9.16	4.05	2.36E-02	8.98	5.78	1.20E-01	14.64	9.24	1.13E-01	16.80	12.10	1.65E-01	0.37	8.89	9.66E-01
cg20255713	-11.54	5.10	2.36E-02	-33.11	14.66	2.39E-02	-20.69	19.99	3.00E-01	-7.28	6.07	2.30E-01	-9.74	15.51	5.30E-01
cg06557630	15.06	6.65	2.36E-02	7.56	12.54	5.47E-01	28.36	43.90	5.18E-01	14.69	9.45	1.20E-01	25.04	14.89	9.27E-02
cg01678172	-22.70	10.03	2.36E-02	-24.13	27.01	3.72E-01	-28.76	77.78	7.12E-01	-19.74	11.57	8.79E-02	-43.03	32.64	1.87E-01
cg16256414	17.51	7.74	2.36E-02	24.55	10.60	2.05E-02	-4.36	23.10	8.50E-01	41.11	33.56	2.21E-01	9.04	14.09	5.21E-01
cg25600256	-5.89	2.60	2.36E-02	-6.70	5.05	1.84E-01	-11.23	6.51	8.44E-02	-4.35	3.99	2.76E-01	-3.12	6.73	6.43E-01
cg22955628	36.09	15.94	2.36E-02	-28.70	50.97	5.73E-01	49.44	215.39	8.18E-01	39.97	17.43	2.19E-02	86.04	64.87	1.85E-01
cg17817564	-31.60	13.96	2.36E-02	-47.93	19.30	1.30E-02	13.67	41.05	7.39E-01	-34.44	36.31	3.43E-01	-14.15	30.20	6.39E-01
cg20937028	-13.96	6.17	2.36E-02	1.22	12.44	9.22E-01	-20.64	15.51	1.83E-01	-13.71	8.50	1.07E-01	-34.22	16.80	4.16E-02
cg25203980	12.99	5.74	2.36E-02	11.95	14.67	4.15E-01	-0.48	14.81	9.74E-01	18.55	7.25	1.05E-02	-5.62	21.73	7.96E-01
cg09279736	3.45	1.52	2.36E-02	2.36	2.08	2.58E-01	6.44	3.09	3.69E-02	2.70	4.97	5.86E-01	2.87	4.27	5.02E-01
cg11181171	22.79	10.07	2.36E-02	21.74	15.25	1.54E-01	37.83	29.69	2.03E-01	43.12	23.44	6.58E-02	3.81	19.57	8.46E-01
cg22632987	21.50	9.50	2.36E-02	27.71	19.48	1.55E-01	6.88	17.54	6.95E-01	14.73	16.75	3.79E-01	55.46	24.77	2.52E-02
cg09888562	24.37	10.77	2.36E-02	-5.97	31.07	8.48E-01	-5.08	112.21	9.64E-01	27.71	12.32	2.45E-02	37.21	33.03	2.60E-01
cg00567703	7.78	3.44	2.36E-02	7.87	4.61	8.80E-02	5.55	7.24	4.43E-01	13.15	12.31	2.86E-01	7.99	9.12	3.81E-01
cg11234098	17.31	7.65	2.36E-02	-13.31	23.14	5.65E-01	15.99	28.10	5.69E-01	19.22	8.83	2.95E-02	47.29	29.55	1.10E-01
cg20655792	8.69	3.84	2.36E-02	6.91	5.55	2.13E-01	17.86	7.41	1.59E-02	0.21	15.56	9.89E-01	2.95	8.79	7.37E-01
cg22856434	-29.45	13.01	2.36E-02	0.18	31.21	9.95E-01	-25.22	55.87	6.52E-01	-36.85	17.09	3.11E-02	-35.14	29.67	2.36E-01
cg11853320	15.06	6.65	2.36E-02	2.79	8.24	7.35E-01	24.60	13.31	6.45E-02	38.31	20.16	5.74E-02	16.97	9.74	8.15E-02
cg26254193	17.85	7.89	2.36E-02	14.69	20.68	4.78E-01	9.81	32.72	7.64E-01	19.19	9.35	4.01E-02	17.55	27.17	5.18E-01
cg03284554	9.06	4.00	2.36E-02	12.28	6.20	4.76E-02	6.18	8.75	4.80E-01	16.09	9.49	9.02E-02	-1.11	9.05	9.02E-01
cg06508445	-21.57	9.53	2.37E-02	-35.13	15.19	2.08E-02	6.65	22.62	7.69E-01	-28.98	20.68	1.61E-01	-12.79	20.48	5.32E-01
cg11692403	-22.20	9.81	2.37E-02	-25.65	19.61	1.91E-01	-21.14	42.71	6.21E-01	-21.13	13.65	1.21E-01	-20.79	23.15	3.69E-01
cg19926144	-11.03	4.88	2.37E-02	-30.10	14.07	3.24E-02	-15.14	23.94	5.27E-01	-7.72	5.65	1.72E-01	-11.05	15.88	4.86E-01
cg13247581	47.02	20.78	2.37E-02	82.13	62.03	1.86E-01	-29.78	228.05	8.96E-01	45.44	23.44	5.26E-02	24.99	68.05	7.13E-01

cg24060473	56.30	24.88	2.37E-02	38.24	52.09	4.63E-01	-160.94	277.16	5.61E-01	41.04	30.68	1.81E-01	141.88	58.93	1.61E-02
cg01688026	31.82	14.06	2.37E-02	27.88	28.29	3.24E-01	5.28	103.37	9.59E-01	29.96	17.99	9.57E-02	52.95	40.11	1.87E-01
cg24269846	-44.35	19.60	2.37E-02	-56.29	24.28	2.04E-02	-1.06	88.51	9.90E-01	45.87	65.31	4.83E-01	-56.19	42.88	1.90E-01
cg10185552	38.93	17.21	2.37E-02	70.81	49.81	1.55E-01	87.60	215.35	6.84E-01	36.56	19.40	5.94E-02	13.11	58.25	8.22E-01
cg26643870	10.53	4.65	2.37E-02	10.31	6.36	1.05E-01	23.32	8.66	7.08E-03	2.64	9.35	7.78E-01	1.03	11.79	9.30E-01
cg03310376	-32.44	14.34	2.37E-02	-22.52	17.19	1.90E-01	-78.80	70.07	2.61E-01	-74.51	65.22	2.53E-01	-46.12	31.01	1.37E-01
cg16794390	19.25	8.51	2.37E-02	26.30	17.16	1.25E-01	29.54	15.75	6.07E-02	12.26	15.17	4.19E-01	2.08	22.12	9.25E-01
cg18099408	-69.77	30.84	2.37E-02	-99.66	42.09	1.79E-02	122.45	191.12	5.22E-01	-97.55	78.09	2.12E-01	-15.04	58.18	7.96E-01
cg11308747	42.06	18.59	2.37E-02	-14.76	46.87	7.53E-01	-60.64	248.13	8.07E-01	59.62	20.04	2.93E-03	-12.41	67.31	8.54E-01
cg00068153	14.57	6.44	2.37E-02	19.11	17.85	2.84E-01	2.55	20.87	9.03E-01	14.87	7.71	5.38E-02	18.95	23.15	4.13E-01
cg06850241	-8.57	3.79	2.37E-02	-9.52	6.10	1.18E-01	-1.64	8.93	8.54E-01	-6.50	7.55	3.90E-01	-16.22	8.86	6.73E-02
cg06869641	-11.42	5.05	2.37E-02	-11.26	7.24	1.20E-01	-13.87	12.51	2.68E-01	-12.72	13.00	3.28E-01	-8.86	11.30	4.33E-01
cg25417281	-41.72	18.45	2.37E-02	-37.06	23.26	1.11E-01	-89.06	108.24	4.11E-01	-21.72	69.95	7.56E-01	-52.52	35.31	1.37E-01
cg27394127	13.15	5.82	2.37E-02	4.62	8.64	5.93E-01	19.74	10.35	5.64E-02	5.03	22.26	8.21E-01	27.56	14.42	5.60E-02
cg26753302	7.54	3.33	2.37E-02	11.18	6.00	6.25E-02	10.46	5.11	4.07E-02	-3.63	9.46	7.01E-01	0.66	8.83	9.40E-01
cg14329680	-11.41	5.05	2.37E-02	-16.46	7.76	3.39E-02	-13.87	12.52	2.68E-01	-9.90	10.66	3.53E-01	0.08	11.55	9.94E-01
cg12731925	26.38	11.66	2.37E-02	8.49	22.41	7.05E-01	-18.67	42.70	6.62E-01	41.13	15.84	9.39E-03	28.16	34.79	4.18E-01
cg03721528	14.65	6.48	2.37E-02	13.59	16.21	4.02E-01	17.67	19.36	3.62E-01	17.71	7.93	2.55E-02	-21.39	26.16	4.13E-01
cg08693600	41.86	18.51	2.37E-02	37.45	48.53	4.40E-01	-105.67	233.50	6.51E-01	44.63	20.99	3.35E-02	33.60	69.41	6.28E-01
cg16294668	6.90	3.05	2.37E-02	6.91	4.10	9.21E-02	13.33	6.28	3.38E-02	-3.04	10.86	7.80E-01	1.30	8.39	8.77E-01
cg14733637	-15.01	6.64	2.37E-02	-11.35	9.80	2.47E-01	-6.86	22.34	7.59E-01	-13.06	16.95	4.41E-01	-24.05	12.14	4.75E-02
cg18255550	-12.43	5.50	2.37E-02	-24.93	12.29	4.26E-02	-41.66	25.09	9.68E-02	-9.14	5.50	9.62E-02	-0.85	14.71	9.54E-01
cg13698224	-7.97	3.53	2.37E-02	-15.21	8.03	5.83E-02	-4.26	13.39	7.50E-01	-6.80	4.49	1.30E-01	-4.60	10.08	6.48E-01
cg04214075	18.46	8.16	2.37E-02	21.46	10.45	4.00E-02	-2.45	36.86	9.47E-01	20.80	40.84	6.10E-01	15.47	14.90	2.99E-01
cg08764162	5.58	2.47	2.37E-02	5.31	3.14	9.10E-02	11.57	6.78	8.80E-02	7.70	7.65	3.14E-01	-0.19	6.44	9.77E-01
cg05354180	-18.03	7.97	2.37E-02	-20.17	20.66	3.29E-01	-26.00	41.14	5.27E-01	-15.05	9.18	1.01E-01	-45.64	32.79	1.64E-01
cg07988979	-13.26	5.87	2.37E-02	-22.22	11.09	4.50E-02	-5.28	11.60	6.49E-01	-9.32	10.96	3.95E-01	-17.02	13.92	2.21E-01
cg20469680	15.57	6.89	2.37E-02	32.51	20.32	1.10E-01	-0.83	33.18	9.80E-01	13.37	8.03	9.60E-02	19.08	21.00	3.64E-01
cg08097817	-16.74	7.40	2.37E-02	-9.96	17.11	5.60E-01	-30.22	28.34	2.86E-01	-20.91	9.46	2.71E-02	-0.02	20.39	9.99E-01
cg23747996	20.32	8.99	2.37E-02	24.21	18.41	1.88E-01	12.04	15.48	4.37E-01	13.44	17.34	4.38E-01	44.13	22.76	5.25E-02
cg27196768	10.42	4.61	2.37E-02	1.85	12.98	8.87E-01	-66.04	50.91	1.95E-01	11.81	5.17	2.23E-02	18.66	17.12	2.76E-01
cg22117188	-11.66	5.16	2.37E-02	-19.14	7.91	1.56E-02	-3.81	10.71	7.22E-01	-7.54	13.14	5.66E-01	-7.85	11.85	5.08E-01
cg07088882	-9.68	4.28	2.38E-02	-14.11	6.08	2.03E-02	-12.08	9.97	2.25E-01	-2.57	14.93	8.64E-01	-1.05	8.78	9.04E-01
cg00806490	17.69	7.82	2.38E-02	29.67	12.29	1.57E-02	6.96	22.97	7.62E-01	17.71	17.31	3.06E-01	4.49	14.95	7.64E-01
cg12812583	15.32	6.78	2.38E-02	29.04	19.43	1.35E-01	33.06	36.12	3.60E-01	12.60	8.15	1.22E-01	12.63	17.43	4.69E-01

cg19907483	-21.41	9.47	2.38E-02	-6.37	14.97	6.70E-01	-1.66	37.96	9.65E-01	-35.93	17.23	3.70E-02	-33.55	19.51	8.55E-02
cg00375608	14.63	6.47	2.38E-02	26.70	16.47	1.05E-01	2.35	34.55	9.46E-01	11.88	7.51	1.14E-01	23.62	24.84	3.42E-01
cg06835425	-52.41	23.18	2.38E-02	-60.19	27.22	2.70E-02	-60.37	146.07	6.79E-01	-7.16	99.41	9.43E-01	-35.10	52.48	5.04E-01
cg12212706	15.06	6.66	2.38E-02	13.89	8.72	1.11E-01	2.02	16.16	9.00E-01	78.00	42.52	6.66E-02	20.60	13.57	1.29E-01
cg06969527	42.87	18.96	2.38E-02	29.07	22.07	1.88E-01	49.08	119.41	6.81E-01	174.41	87.27	4.57E-02	63.02	43.56	1.48E-01
cg26796727	28.13	12.44	2.38E-02	28.09	29.71	3.44E-01	50.10	53.23	3.47E-01	18.27	15.49	2.38E-01	69.59	35.23	4.82E-02
cg00731739	12.40	5.48	2.38E-02	25.63	12.78	4.48E-02	-0.76	12.19	9.51E-01	12.28	7.52	1.03E-01	15.84	19.11	4.07E-01
cg15146859	16.27	7.20	2.38E-02	46.32	31.30	1.39E-01	24.65	48.97	6.15E-01	13.82	7.89	8.00E-02	19.06	23.39	4.15E-01
cg16023600	-12.93	5.72	2.38E-02	-15.09	10.63	1.56E-01	-7.41	13.46	5.82E-01	-13.71	8.93	1.25E-01	-13.43	16.58	4.18E-01
cg02577368	42.80	18.93	2.38E-02	38.54	54.63	4.80E-01	-165.63	198.45	4.04E-01	44.83	21.31	3.54E-02	52.78	66.39	4.27E-01
cg13223918	17.10	7.57	2.38E-02	6.88	19.92	7.30E-01	76.66	43.18	7.59E-02	18.93	8.39	2.41E-02	-9.45	29.31	7.47E-01
cg03098047	30.22	13.37	2.38E-02	26.97	13.88	5.20E-02	159.87	108.62	1.41E-01	113.97	70.49	1.06E-01	16.45	31.11	5.97E-01
cg00583733	11.94	5.28	2.38E-02	11.01	7.49	1.42E-01	20.59	11.98	8.55E-02	17.31	14.07	2.19E-01	0.11	12.93	9.93E-01
cg06180915	19.91	8.81	2.38E-02	14.87	10.68	1.64E-01	49.50	30.04	9.94E-02	-38.76	57.16	4.98E-01	30.80	19.23	1.09E-01
cg01149712	20.38	9.02	2.38E-02	25.93	18.43	1.60E-01	30.17	37.14	4.17E-01	16.08	11.48	1.61E-01	29.22	30.92	3.45E-01
cg13912964	6.42	2.84	2.38E-02	5.26	3.76	1.62E-01	15.09	7.61	4.72E-02	9.22	7.16	1.98E-01	-1.06	7.81	8.92E-01
cg04663870	-18.61	8.23	2.38E-02	-19.77	10.47	5.89E-02	-28.15	26.40	2.86E-01	-20.57	47.84	6.67E-01	-11.92	16.33	4.66E-01
cg26204713	-44.19	19.55	2.38E-02	2.61	61.40	9.66E-01	-190.34	116.57	1.02E-01	-44.33	22.25	4.63E-02	-49.52	62.40	4.27E-01
cg10260999	-43.78	19.37	2.38E-02	-52.34	25.29	3.85E-02	-68.22	76.29	3.71E-01	-57.81	62.12	3.52E-01	-12.17	38.60	7.53E-01
cg16986298	-24.49	10.84	2.38E-02	-70.10	34.32	4.11E-02	-72.25	160.66	6.53E-01	-17.42	11.85	1.42E-01	-43.66	44.32	3.25E-01
cg02957150	-13.09	5.79	2.38E-02	-15.42	7.41	3.73E-02	-15.90	18.12	3.80E-01	-19.11	35.86	5.94E-01	-5.90	11.36	6.03E-01
cg21473142	9.54	4.22	2.38E-02	10.31	7.50	1.69E-01	24.44	12.59	5.23E-02	5.40	6.77	4.26E-01	7.85	9.90	4.28E-01
cg27039237	19.41	8.59	2.38E-02	50.30	24.54	4.04E-02	55.60	53.48	2.99E-01	15.57	9.64	1.06E-01	-9.49	35.74	7.91E-01
cg01177796	-19.50	8.63	2.38E-02	-2.14	16.69	8.98E-01	13.65	65.07	8.34E-01	-27.85	11.14	1.25E-02	-21.40	25.37	3.99E-01
cg18218752	19.72	8.73	2.38E-02	32.06	17.07	6.03E-02	6.52	20.56	7.51E-01	26.41	12.78	3.87E-02	-15.98	26.41	5.45E-01
cg14143887	-14.78	6.54	2.38E-02	-10.02	15.76	5.25E-01	-12.62	27.36	6.45E-01	-17.08	7.96	3.18E-02	-8.37	21.21	6.93E-01
cg03749154	-7.81	3.45	2.38E-02	-14.80	8.47	8.05E-02	-17.16	13.11	1.91E-01	-5.17	4.38	2.38E-01	-6.59	9.14	4.71E-01
cg15650161	-13.75	6.08	2.38E-02	-22.42	9.94	2.41E-02	5.48	13.79	6.91E-01	-17.63	11.94	1.40E-01	-10.74	14.69	4.64E-01
cg15345477	-6.36	2.82	2.38E-02	-7.69	4.33	7.60E-02	-5.23	7.01	4.56E-01	-0.90	6.05	8.82E-01	-10.39	6.29	9.87E-02
cg10143323	-16.40	7.26	2.38E-02	-10.52	19.35	5.87E-01	35.82	32.83	2.75E-01	-19.72	8.34	1.81E-02	-29.71	27.84	2.86E-01
cg00076797	-11.45	5.07	2.38E-02	-16.97	9.47	7.32E-02	-25.72	13.37	5.44E-02	-1.18	8.20	8.85E-01	-12.62	11.06	2.54E-01
cg26802119	21.72	9.61	2.38E-02	6.61	9.83	5.01E-01	40.92	13.97	3.40E-03	40.06	21.13	5.80E-02	11.16	16.11	4.88E-01
cg11292836	-6.78	3.00	2.38E-02	-6.30	5.15	2.21E-01	-6.91	7.11	3.32E-01	-7.79	5.06	1.24E-01	-5.12	8.24	5.34E-01
cg18534491	17.71	7.84	2.38E-02	26.90	13.41	4.49E-02	9.44	22.00	6.68E-01	9.51	14.78	5.20E-01	18.60	15.67	2.35E-01
cg04927931	10.64	4.71	2.38E-02	16.73	10.50	1.11E-01	18.22	30.46	5.50E-01	7.08	5.85	2.26E-01	17.69	13.17	1.79E-01

cg19836145	-8.24	3.65	2.38E-02	-1.35	7.47	8.56E-01	-12.37	12.68	3.29E-01	-8.31	5.01	9.72E-02	-16.60	9.41	7.75E-02
cg09174717	10.84	4.80	2.38E-02	11.27	8.76	1.98E-01	-4.36	11.04	6.93E-01	17.25	7.56	2.24E-02	12.29	14.57	3.99E-01
cg00947782	3.70	1.64	2.39E-02	2.39	2.42	3.22E-01	5.52	3.38	1.02E-01	7.10	4.64	1.26E-01	2.31	3.83	5.47E-01
cg11036338	26.71	11.82	2.39E-02	72.13	37.82	5.65E-02	110.14	195.82	5.74E-01	21.47	12.80	9.34E-02	20.64	55.11	7.08E-01
cg08737770	-14.09	6.23	2.39E-02	-14.40	13.65	2.92E-01	-40.47	26.98	1.34E-01	-10.61	7.78	1.73E-01	-21.95	20.09	2.75E-01
cg11857256	20.78	9.20	2.39E-02	38.12	17.13	2.60E-02	35.66	20.00	7.46E-02	7.89	6.17	2.01E-01	30.04	27.08	2.67E-01
cg11427898	15.13	6.70	2.39E-02	18.89	14.89	2.05E-01	-31.31	39.51	4.28E-01	14.99	8.36	7.28E-02	20.43	18.86	2.79E-01
cg06943141	-6.26	2.77	2.39E-02	-4.94	4.20	2.40E-01	-10.32	5.42	5.70E-02	-7.39	8.68	3.95E-01	-3.30	6.17	5.93E-01
cg11458498	-6.46	2.86	2.39E-02	-3.12	4.73	5.10E-01	-7.86	8.97	3.81E-01	-9.45	4.68	4.36E-02	-6.23	7.12	3.82E-01
cg19363466	19.60	8.68	2.39E-02	-3.88	24.54	8.74E-01	10.41	55.65	8.52E-01	27.73	11.95	2.03E-02	16.12	15.25	2.91E-01
cg07715041	-13.11	5.80	2.39E-02	-24.38	10.42	1.93E-02	-4.45	17.89	8.04E-01	-4.82	9.02	5.93E-01	-18.08	14.05	1.98E-01
cg16201146	-11.45	5.07	2.39E-02	-10.78	8.35	1.97E-01	-8.58	12.02	4.76E-01	-14.39	9.81	1.42E-01	-11.29	11.71	3.35E-01
cg21380181	-17.89	7.92	2.39E-02	-19.80	10.47	5.87E-02	-22.44	30.76	4.66E-01	5.44	26.05	8.35E-01	-20.74	15.27	1.74E-01
cg06652085	11.83	5.24	2.39E-02	14.93	7.02	3.35E-02	13.38	12.37	2.80E-01	12.18	16.11	4.50E-01	-1.02	13.15	9.38E-01
cg11314668	-14.63	6.47	2.39E-02	-26.11	10.63	1.40E-02	-4.23	13.76	7.58E-01	-14.68	15.58	3.46E-01	-6.25	13.36	6.40E-01
cg25173971	23.25	10.29	2.39E-02	22.00	37.14	5.54E-01	-10.25	180.27	9.55E-01	26.21	11.01	1.73E-02	-29.22	48.32	5.45E-01
cg13217590	28.14	12.46	2.39E-02	41.93	19.15	2.86E-02	40.80	37.17	2.72E-01	20.08	30.18	5.06E-01	8.12	22.98	7.24E-01
cg10959664	-13.74	6.08	2.39E-02	0.73	15.35	9.62E-01	10.12	29.36	7.30E-01	-17.59	7.09	1.31E-02	-20.91	24.03	3.84E-01
cg02527030	-13.17	5.83	2.39E-02	-18.04	12.86	1.61E-01	-12.86	18.65	4.90E-01	-13.74	7.49	6.66E-02	1.37	19.40	9.44E-01
cg00399139	-26.13	11.57	2.39E-02	-43.55	22.29	5.07E-02	-1.34	52.03	9.79E-01	-23.80	15.57	1.26E-01	-9.23	32.23	7.74E-01
cg20443501	13.88	6.15	2.39E-02	16.31	8.71	6.11E-02	19.87	13.94	1.54E-01	23.12	17.86	1.95E-01	-4.50	14.16	7.51E-01
cg06495961	11.33	5.02	2.39E-02	19.67	6.97	4.79E-03	8.57	10.40	4.10E-01	17.23	14.34	2.29E-01	-0.09	8.34	9.91E-01
cg19826864	21.98	9.73	2.39E-02	17.72	12.66	1.62E-01	12.13	34.60	7.26E-01	-50.01	63.50	4.31E-01	38.22	17.57	2.96E-02
cg21886851	-54.09	23.95	2.39E-02	-114.26	53.70	3.34E-02	-367.60	243.35	1.31E-01	-40.62	16.71	1.51E-02	-23.93	64.97	7.13E-01
cg23737049	-16.90	7.48	2.39E-02	-14.96	11.39	1.89E-01	-13.88	16.08	3.88E-01	-37.27	22.59	9.90E-02	-13.83	15.20	3.63E-01
cg09384400	19.93	8.82	2.39E-02	21.79	16.42	1.84E-01	19.71	16.36	2.28E-01	7.13	19.89	7.20E-01	29.05	18.65	1.19E-01
cg06713671	13.59	6.02	2.39E-02	12.01	8.14	1.40E-01	16.08	12.90	2.12E-01	14.04	19.07	4.61E-01	15.61	16.33	3.39E-01
cg10451724	18.76	8.31	2.39E-02	16.47	15.18	2.78E-01	22.98	15.36	1.35E-01	8.28	15.42	5.91E-01	39.87	24.18	9.91E-02
cg15481483	22.17	9.82	2.39E-02	20.82	13.61	1.26E-01	6.60	21.71	7.61E-01	57.56	30.77	6.14E-02	23.81	23.58	3.13E-01
cg13954457	9.83	4.35	2.39E-02	20.61	8.90	2.07E-02	5.65	10.15	5.78E-01	3.01	8.78	7.32E-01	9.45	7.57	2.12E-01
cg00643222	-8.88	3.93	2.39E-02	-3.75	8.79	6.69E-01	-5.49	14.25	7.00E-01	-12.33	5.05	1.47E-02	-2.04	11.45	8.59E-01
cg11422695	16.05	7.11	2.39E-02	15.04	16.36	3.58E-01	6.23	37.48	8.68E-01	15.24	8.50	7.31E-02	30.66	25.74	2.34E-01
cg03316359	-15.55	6.89	2.39E-02	-21.25	11.39	6.20E-02	-21.66	14.91	1.46E-01	4.53	15.48	7.70E-01	-18.16	14.57	2.13E-01
cg03550794	24.84	11.00	2.40E-02	4.57	26.83	8.65E-01	7.48	86.35	9.31E-01	28.75	13.06	2.77E-02	33.50	33.84	3.22E-01
cg06547175	24.63	10.91	2.40E-02	28.99	20.65	1.60E-01	7.93	22.40	7.23E-01	16.63	20.31	4.13E-01	50.47	24.68	4.08E-02

cg10156714	-12.97	5.74	2.40E-02	-17.04	16.99	3.16E-01	11.14	32.04	7.28E-01	-13.61	6.50	3.63E-02	-10.33	21.20	6.26E-01
cg27233989	15.81	7.00	2.40E-02	30.09	21.26	1.57E-01	23.58	44.23	5.94E-01	13.31	8.09	1.00E-01	16.91	20.39	4.07E-01
cg25140190	6.52	2.89	2.40E-02	2.01	4.32	6.41E-01	10.03	6.80	1.40E-01	10.54	6.61	1.11E-01	9.98	6.81	1.43E-01
cg08222702	-20.61	9.13	2.40E-02	-13.43	13.37	3.15E-01	-14.26	19.34	4.61E-01	-59.39	30.13	4.87E-02	-26.09	19.50	1.81E-01
cg15553814	-10.04	4.45	2.40E-02	-3.26	8.92	7.15E-01	-4.74	18.48	7.97E-01	-15.23	5.81	8.75E-03	-0.36	13.49	9.79E-01
cg08446038	10.59	4.69	2.40E-02	16.16	6.30	1.04E-02	7.83	11.32	4.89E-01	3.82	19.63	8.46E-01	0.36	10.06	9.72E-01
cg14241836	-8.49	3.76	2.40E-02	-7.73	5.35	1.48E-01	-16.26	10.33	1.16E-01	1.07	8.00	8.93E-01	-18.06	9.63	6.07E-02
cg01406988	8.09	3.59	2.40E-02	2.16	7.44	7.72E-01	5.21	10.35	6.15E-01	12.12	5.07	1.69E-02	6.18	9.31	5.07E-01
cg16397620	17.55	7.77	2.40E-02	33.21	21.21	1.17E-01	8.48	71.52	9.06E-01	15.38	8.82	8.13E-02	13.57	28.01	6.28E-01
cg19929638	6.28	2.78	2.40E-02	3.59	4.04	3.73E-01	3.53	5.96	5.54E-01	2.96	10.50	7.78E-01	14.65	5.55	8.30E-03
cg22949004	19.53	8.65	2.40E-02	0.70	14.38	9.61E-01	31.65	15.88	4.63E-02	28.23	21.20	1.83E-01	29.68	20.69	1.52E-01
cg00489539	-11.85	5.25	2.40E-02	-7.52	14.43	6.02E-01	-5.89	29.91	8.44E-01	-10.55	5.95	7.62E-02	-42.08	21.71	5.26E-02
cg18626346	32.29	14.30	2.40E-02	39.94	34.12	2.42E-01	95.49	117.68	4.17E-01	34.77	16.90	3.96E-02	-11.41	46.96	8.08E-01
cg14395791	14.18	6.28	2.40E-02	25.24	10.59	1.71E-02	6.07	11.42	5.95E-01	7.03	14.27	6.22E-01	13.85	16.13	3.91E-01
cg04564920	17.63	7.81	2.40E-02	4.60	23.89	8.47E-01	-6.87	73.49	9.26E-01	18.93	8.65	2.85E-02	26.90	30.52	3.78E-01
cg24803290	20.25	8.97	2.40E-02	29.27	24.89	2.40E-01	-12.09	129.91	9.26E-01	19.31	9.95	5.22E-02	15.34	39.07	6.95E-01
cg15420785	26.26	11.63	2.40E-02	31.05	24.89	2.12E-01	26.40	51.68	6.09E-01	24.65	14.83	9.65E-02	25.71	34.22	4.53E-01
cg19811337	33.88	15.01	2.40E-02	28.25	19.60	1.50E-01	91.33	70.61	1.96E-01	57.54	41.46	1.65E-01	23.79	30.79	4.40E-01
cg04125710	26.59	11.78	2.40E-02	21.37	17.97	2.34E-01	54.33	25.34	3.20E-02	2.90	32.35	9.29E-01	23.84	25.02	3.41E-01
cg04495270	9.61	4.26	2.40E-02	12.88	6.24	3.91E-02	-1.52	9.26	8.69E-01	23.17	16.92	1.71E-01	9.50	8.35	2.55E-01
cg19317258	-11.49	5.09	2.40E-02	-17.28	8.07	3.21E-02	-17.86	10.37	8.50E-02	-1.84	16.23	9.10E-01	-0.47	9.94	9.62E-01
cg01601476	14.25	6.31	2.40E-02	15.94	9.60	9.66E-02	23.85	18.67	2.01E-01	16.75	12.93	1.95E-01	2.92	13.64	8.31E-01
cg21913652	-9.33	4.13	2.40E-02	-19.98	10.93	6.75E-02	-3.30	15.30	8.29E-01	-6.54	4.94	1.85E-01	-19.91	14.39	1.67E-01
cg06747362	19.42	8.61	2.40E-02	11.84	15.96	4.58E-01	-11.52	33.10	7.28E-01	21.37	11.91	7.28E-02	46.87	24.89	5.97E-02
cg05661533	9.39	4.16	2.40E-02	8.64	5.82	1.38E-01	8.84	9.47	3.50E-01	18.64	10.27	6.94E-02	1.63	11.41	8.86E-01
cg13766589	23.96	10.61	2.40E-02	20.97	16.18	1.95E-01	29.83	23.52	2.05E-01	3.71	22.50	8.69E-01	55.99	28.03	4.58E-02
cg11227621	35.27	15.63	2.40E-02	-1.03	44.47	9.81E-01	-229.23	195.45	2.41E-01	43.12	17.99	1.65E-02	37.39	45.96	4.16E-01
cg23491599	16.00	7.09	2.40E-02	43.29	18.97	2.25E-02	-26.63	57.73	6.45E-01	13.01	8.40	1.22E-01	8.22	19.37	6.71E-01
cg15623249	17.91	7.94	2.40E-02	13.78	22.69	5.44E-01	-34.89	50.70	4.91E-01	21.38	8.98	1.73E-02	5.40	29.46	8.55E-01
cg08881785	-8.05	3.57	2.40E-02	-6.04	7.02	3.90E-01	-1.20	12.66	9.25E-01	-9.83	4.92	4.58E-02	-8.96	9.61	3.51E-01
cg18085807	27.27	12.09	2.40E-02	-2.60	31.01	9.33E-01	-120.74	141.46	3.93E-01	31.95	13.68	1.95E-02	59.76	49.07	2.23E-01
cg11394278	-8.73	3.87	2.40E-02	-8.63	7.54	2.53E-01	-7.55	15.15	6.18E-01	-7.49	5.19	1.49E-01	-15.56	11.36	1.71E-01
cg06005891	14.37	6.37	2.40E-02	9.11	16.79	5.87E-01	33.09	26.90	2.19E-01	16.24	7.85	3.85E-02	3.61	16.94	8.31E-01
cg21958743	-8.16	3.62	2.40E-02	-11.75	8.16	1.50E-01	0.76	13.31	9.54E-01	-7.35	4.71	1.19E-01	-11.25	9.65	2.44E-01
cg24979233	-28.63	12.69	2.40E-02	-45.21	22.57	4.52E-02	-34.39	61.52	5.76E-01	-49.42	27.60	7.33E-02	-5.66	19.34	7.70E-01

cg17506621	4.94	2.19	2.40E-02	5.25	3.01	8.05E-02	0.74	5.37	8.90E-01	6.88	8.11	3.96E-01	6.63	4.56	1.46E-01
cg07701579	-12.91	5.72	2.41E-02	-18.39	8.95	4.00E-02	-11.44	12.76	3.70E-01	-4.97	14.63	7.34E-01	-9.84	11.74	4.02E-01
cg19399370	-11.77	5.22	2.41E-02	-4.93	12.90	7.02E-01	-31.16	28.24	2.70E-01	-12.16	6.18	4.89E-02	-13.75	17.48	4.32E-01
cg08008636	-49.95	22.14	2.41E-02	-39.20	28.15	1.64E-01	30.97	90.04	7.31E-01	-89.00	57.89	1.24E-01	-83.33	52.98	1.16E-01
cg13770399	7.27	3.22	2.41E-02	8.67	4.22	4.00E-02	9.22	8.31	2.67E-01	6.98	11.29	5.36E-01	1.42	7.51	8.50E-01
cg03575765	-8.93	3.96	2.41E-02	-6.65	6.34	2.94E-01	-7.61	7.44	3.06E-01	-27.16	12.08	2.45E-02	-5.77	8.46	4.95E-01
cg00899659	7.07	3.13	2.41E-02	6.33	4.13	1.25E-01	16.26	7.59	3.23E-02	4.06	10.67	7.04E-01	1.84	7.62	8.09E-01
cg27598107	6.33	2.80	2.41E-02	8.32	3.88	3.23E-02	7.93	5.77	1.70E-01	7.71	10.44	4.60E-01	-2.56	6.79	7.07E-01
cg02334987	-7.17	3.18	2.41E-02	-4.21	4.36	3.34E-01	-14.49	8.80	9.97E-02	-12.47	9.85	2.06E-01	-7.46	6.57	2.56E-01
cg06750832	19.39	8.60	2.41E-02	5.12	31.98	8.73E-01	-132.71	115.91	2.52E-01	20.62	9.29	2.64E-02	31.80	33.54	3.43E-01
cg10220895	-6.45	2.86	2.41E-02	-2.82	4.48	5.29E-01	-7.41	6.80	2.76E-01	-11.73	6.83	8.57E-02	-8.04	5.83	1.68E-01
cg10294329	12.81	5.68	2.41E-02	-5.50	13.26	6.78E-01	17.66	20.15	3.81E-01	16.73	6.92	1.56E-02	17.98	22.37	4.22E-01
cg21216258	6.51	2.89	2.41E-02	4.55	3.93	2.47E-01	10.04	7.45	1.78E-01	14.09	7.10	4.72E-02	1.52	7.56	8.40E-01
cg23646360	-7.37	3.27	2.41E-02	-4.32	4.52	3.39E-01	-8.72	6.86	2.04E-01	-15.23	16.86	3.66E-01	-12.06	7.09	8.89E-02
cg01069104	17.34	7.69	2.41E-02	12.15	9.32	1.92E-01	20.37	35.62	5.67E-01	0.59	57.88	9.92E-01	31.78	15.21	3.67E-02
cg23012054	-13.47	5.97	2.41E-02	-20.12	13.50	1.36E-01	-0.86	17.55	9.61E-01	-14.12	7.87	7.28E-02	-11.56	17.78	5.15E-01
cg20171011	-11.47	5.08	2.41E-02	-21.62	8.48	1.07E-02	1.55	12.52	9.02E-01	-9.16	10.64	3.89E-01	-7.50	10.23	4.63E-01
cg17161419	-10.63	4.71	2.41E-02	-13.99	7.27	5.43E-02	-4.66	9.05	6.07E-01	-29.81	16.23	6.62E-02	-4.33	9.97	6.64E-01
cg20385461	-7.64	3.39	2.41E-02	-10.19	6.71	1.29E-01	-17.55	8.53	3.97E-02	-4.72	4.94	3.40E-01	-0.53	9.87	9.57E-01
cg22283754	-18.43	8.17	2.41E-02	-11.21	20.77	5.89E-01	19.51	30.33	5.20E-01	-19.00	8.60	2.71E-02	-47.58	23.57	4.35E-02
cg17321561	-28.21	12.50	2.41E-02	-27.05	22.62	2.32E-01	-58.06	47.68	2.23E-01	-37.08	12.80	3.76E-03	29.48	35.13	4.01E-01
cg20758789	-10.59	4.69	2.41E-02	-20.14	9.39	3.19E-02	-6.16	12.24	6.15E-01	-5.93	7.06	4.00E-01	-12.60	11.72	2.83E-01
cg13618111	-14.41	6.39	2.41E-02	-17.53	11.64	1.32E-01	-10.24	17.80	5.65E-01	-13.38	10.68	2.10E-01	-14.25	13.89	3.05E-01
cg01077808	-34.42	15.26	2.41E-02	-63.71	39.41	1.06E-01	-51.11	191.82	7.90E-01	-30.83	17.68	8.12E-02	-16.02	48.58	7.42E-01
cg20767977	14.67	6.50	2.41E-02	20.50	16.64	2.18E-01	38.66	42.86	3.67E-01	12.36	7.49	9.86E-02	19.00	24.73	4.42E-01
cg17596554	-28.44	12.61	2.41E-02	-21.24	17.18	2.16E-01	-19.47	37.09	6.00E-01	-66.15	54.70	2.27E-01	-38.38	23.30	9.95E-02
cg06526620	7.78	3.45	2.41E-02	6.57	4.34	1.30E-01	14.93	8.80	8.98E-02	3.39	11.96	7.77E-01	8.04	9.52	3.99E-01
cg24398933	22.56	10.00	2.41E-02	28.39	18.65	1.28E-01	6.75	19.74	7.32E-01	29.35	17.56	9.47E-02	23.92	27.59	3.86E-01
cg21117476	17.66	7.83	2.41E-02	49.41	18.36	7.12E-03	20.49	23.83	3.90E-01	12.01	9.66	2.13E-01	9.15	10.98	4.05E-01
cg09414983	-7.21	3.19	2.41E-02	-12.25	5.42	2.37E-02	0.87	6.93	9.00E-01	-10.51	4.56	2.12E-02	-0.35	7.39	9.62E-01
cg11188027	-50.29	22.30	2.41E-02	-67.70	27.79	1.49E-02	-11.65	98.53	9.06E-01	-68.39	92.59	4.60E-01	-8.70	44.86	8.46E-01
cg15677294	16.29	7.22	2.41E-02	7.90	10.85	4.67E-01	20.37	14.30	1.54E-01	14.13	22.19	5.24E-01	31.17	16.34	5.64E-02
cg15690853	18.19	8.06	2.41E-02	28.92	16.58	8.10E-02	3.59	32.15	9.11E-01	13.45	10.56	2.03E-01	27.96	23.57	2.35E-01
cg06707978	12.26	5.44	2.41E-02	23.51	8.89	8.19E-03	5.64	14.09	6.89E-01	1.01	10.23	9.22E-01	12.20	11.96	3.08E-01
cg07821629	20.54	9.11	2.41E-02	17.53	27.65	5.26E-01	48.11	66.45	4.69E-01	20.39	10.47	5.14E-02	19.88	26.85	4.59E-01

cg23342234	19.47	8.63	2.41E-02	36.52	15.05	1.53E-02	11.15	15.40	4.69E-01	12.78	19.37	5.10E-01	8.92	21.73	6.81E-01
cg00065302	15.78	7.00	2.41E-02	18.04	12.79	1.58E-01	10.06	14.81	4.97E-01	11.95	13.78	3.86E-01	22.97	14.92	1.24E-01
cg14479344	6.27	2.78	2.41E-02	8.53	4.25	4.46E-02	8.26	6.57	2.09E-01	4.72	6.32	4.55E-01	1.10	6.26	8.61E-01
cg01036779	-18.62	8.26	2.41E-02	-14.26	12.76	2.64E-01	-24.05	24.82	3.32E-01	-27.29	16.34	9.49E-02	-14.03	17.82	4.31E-01
cg06470424	-24.77	10.99	2.42E-02	-25.70	15.31	9.31E-02	-23.08	23.06	3.17E-01	-32.95	33.92	3.31E-01	-18.54	28.10	5.09E-01
cg10981907	8.53	3.78	2.42E-02	11.59	5.48	3.44E-02	10.15	9.56	2.88E-01	20.40	13.04	1.18E-01	-1.10	7.13	8.78E-01
cg08849875	17.67	7.84	2.42E-02	10.14	13.16	4.41E-01	-2.15	19.61	9.13E-01	16.97	6.02	4.85E-03	51.39	18.79	6.23E-03
cg23147393	12.34	5.47	2.42E-02	12.98	9.52	1.73E-01	3.85	11.96	7.47E-01	13.74	9.82	1.62E-01	19.84	14.12	1.60E-01
cg08322194	-14.16	6.28	2.42E-02	-12.73	9.04	1.59E-01	-0.83	14.30	9.54E-01	-33.34	20.29	1.00E-01	-20.37	13.13	1.21E-01
cg05928980	-9.63	4.27	2.42E-02	-12.06	9.65	2.11E-01	-16.95	11.08	1.26E-01	-9.48	5.98	1.13E-01	0.68	11.24	9.52E-01
cg26329538	13.25	5.88	2.42E-02	12.06	11.10	2.77E-01	34.84	14.55	1.66E-02	10.83	7.70	1.60E-01	-3.77	17.43	8.29E-01
cg06281694	-41.18	18.27	2.42E-02	-52.53	22.89	2.17E-02	-92.31	52.32	7.77E-02	-18.94	63.01	7.64E-01	10.87	39.27	7.82E-01
cg03013070	10.94	4.85	2.42E-02	15.28	7.94	5.44E-02	8.49	11.73	4.69E-01	6.60	8.69	4.47E-01	11.98	12.80	3.49E-01
cg02937233	-43.53	19.31	2.42E-02	-54.73	23.79	2.14E-02	-47.93	94.77	6.13E-01	31.73	63.87	6.19E-01	-40.25	42.34	3.42E-01
cg11001581	8.12	3.60	2.42E-02	9.04	5.76	1.16E-01	13.95	7.01	4.67E-02	-4.15	9.53	6.63E-01	7.42	8.03	3.56E-01
cg14568936	-36.92	16.38	2.42E-02	-59.77	48.61	2.19E-01	-133.41	217.30	5.39E-01	-35.13	18.27	5.45E-02	-14.83	58.96	8.01E-01
cg01273565	33.19	14.73	2.42E-02	42.87	20.48	3.64E-02	38.61	39.00	3.22E-01	40.78	43.80	3.52E-01	4.02	30.89	8.97E-01
cg11201772	38.53	17.10	2.42E-02	29.11	20.96	1.65E-01	9.94	75.25	8.95E-01	81.73	63.63	1.99E-01	60.48	37.24	1.04E-01
cg04560098	-11.52	5.11	2.42E-02	-18.20	7.37	1.35E-02	-10.22	12.71	4.21E-01	0.75	13.80	9.57E-01	-5.51	10.92	6.14E-01
cg26031742	30.87	13.70	2.42E-02	55.92	20.34	5.97E-03	4.86	20.34	8.11E-01	14.51	14.31	3.11E-01	62.36	26.10	1.69E-02
cg21333861	42.44	18.83	2.42E-02	35.67	38.68	3.57E-01	35.19	151.36	8.16E-01	35.00	24.10	1.46E-01	88.05	50.84	8.33E-02
cg04600297	11.58	5.14	2.42E-02	17.90	7.18	1.27E-02	18.76	10.66	7.83E-02	-0.71	13.10	9.57E-01	-0.12	10.74	9.91E-01
cg14328907	27.05	12.00	2.42E-02	31.56	31.54	3.17E-01	53.96	85.25	5.27E-01	26.26	13.91	5.90E-02	20.41	39.78	6.08E-01
cg13767755	21.26	9.43	2.42E-02	31.16	13.70	2.29E-02	19.02	35.25	5.89E-01	42.62	38.31	2.66E-01	6.44	15.04	6.69E-01
cg24033742	-17.02	7.55	2.42E-02	-15.02	21.52	4.85E-01	-46.24	27.27	9.00E-02	-17.33	8.80	4.89E-02	17.88	29.90	5.50E-01
cg20110591	23.63	10.48	2.42E-02	19.30	13.05	1.39E-01	32.91	37.23	3.77E-01	31.37	77.49	6.86E-01	31.06	20.66	1.33E-01
cg14780449	10.39	4.61	2.42E-02	10.59	6.22	8.84E-02	14.43	12.17	2.36E-01	3.36	12.37	7.86E-01	12.08	11.25	2.83E-01
cg11134777	20.15	8.94	2.42E-02	14.95	16.63	3.69E-01	12.58	15.56	4.19E-01	24.38	17.80	1.71E-01	42.97	24.94	8.49E-02
cg17657467	18.49	8.20	2.42E-02	11.33	15.92	4.77E-01	13.22	35.81	7.12E-01	21.19	11.07	5.57E-02	23.74	22.51	2.92E-01
cg03945122	-21.06	9.34	2.42E-02	-43.33	21.78	4.66E-02	12.40	47.15	7.93E-01	-13.22	11.48	2.49E-01	-42.20	27.70	1.28E-01
cg07997104	-53.85	23.90	2.42E-02	-54.35	28.36	5.53E-02	-126.06	168.22	4.54E-01	-15.08	103.67	8.84E-01	-55.00	51.36	2.84E-01
cg01109333	16.72	7.42	2.42E-02	27.34	16.46	9.66E-02	14.67	20.76	4.80E-01	9.47	9.65	3.26E-01	47.77	26.72	7.38E-02
cg04809093	14.58	6.47	2.43E-02	14.98	11.46	1.91E-01	4.42	16.71	7.91E-01	11.98	9.73	2.18E-01	43.19	21.71	4.66E-02
cg01948148	22.36	9.93	2.43E-02	24.20	32.33	4.54E-01	-79.73	101.44	4.32E-01	23.64	10.79	2.84E-02	16.81	44.51	7.06E-01
cg16080643	-14.05	6.24	2.43E-02	-26.20	15.50	9.09E-02	-0.82	31.12	9.79E-01	-13.71	7.43	6.52E-02	-1.29	20.42	9.50E-01

cg15787039	9.30	4.13	2.43E-02	10.40	5.85	7.57E-02	17.88	9.77	6.73E-02	6.58	12.93	6.11E-01	1.22	8.74	8.89E-01
cg09255505	17.65	7.84	2.43E-02	16.53	16.49	3.16E-01	1.55	19.34	9.36E-01	23.20	11.05	3.58E-02	18.69	23.91	4.35E-01
cg00988708	11.73	5.21	2.43E-02	6.00	11.46	6.01E-01	3.84	17.93	8.31E-01	12.10	6.58	6.60E-02	31.42	18.14	8.32E-02
cg10077746	11.42	5.07	2.43E-02	12.58	6.29	4.57E-02	3.73	15.83	8.14E-01	39.62	18.09	2.85E-02	4.92	8.61	5.68E-01
cg04014685	-11.57	5.14	2.43E-02	-19.93	10.15	4.97E-02	-6.81	14.87	6.47E-01	-11.40	7.47	1.27E-01	-1.72	13.22	8.96E-01
cg23972271	10.32	4.58	2.43E-02	13.61	9.72	1.61E-01	11.55	20.41	5.71E-01	11.33	5.85	5.30E-02	-1.92	13.49	8.87E-01
cg13413488	-17.09	7.59	2.43E-02	-26.25	11.59	2.36E-02	2.50	18.71	8.94E-01	-32.24	21.73	1.38E-01	-8.17	14.20	5.65E-01
cg04278197	-9.62	4.27	2.43E-02	-12.02	13.24	3.64E-01	0.03	23.91	9.99E-01	-10.17	4.79	3.38E-02	-4.23	16.02	7.92E-01
cg10861599	16.34	7.25	2.43E-02	9.56	9.12	2.95E-01	35.39	20.77	8.84E-02	26.31	27.33	3.36E-01	23.54	17.32	1.74E-01
cg27548595	19.57	8.69	2.43E-02	13.28	14.90	3.73E-01	12.81	17.25	4.58E-01	33.23	16.48	4.37E-02	19.98	24.22	4.09E-01
cg09282946	11.08	4.92	2.43E-02	8.20	6.97	2.39E-01	11.76	9.49	2.15E-01	33.54	17.58	5.64E-02	7.82	12.53	5.33E-01
cg08160072	6.22	2.76	2.43E-02	4.75	3.53	1.79E-01	10.39	6.92	1.33E-01	9.14	8.65	2.91E-01	5.73	7.75	4.60E-01
cg09415148	14.83	6.58	2.43E-02	16.42	14.23	2.48E-01	-13.56	24.02	5.72E-01	17.54	8.63	4.21E-02	16.45	18.32	3.69E-01
cg19714940	26.50	11.77	2.43E-02	50.03	31.44	1.12E-01	157.83	201.52	4.34E-01	20.61	13.16	1.17E-01	43.54	49.31	3.77E-01
cg04628008	-27.84	12.36	2.43E-02	-29.48	40.88	4.71E-01	-163.36	250.31	5.14E-01	-26.90	13.47	4.57E-02	-32.78	49.13	5.05E-01
cg16179952	13.64	6.06	2.43E-02	24.44	8.99	6.55E-03	13.88	15.68	3.76E-01	16.40	7.90	3.80E-02	-3.72	10.47	7.22E-01
cg06710785	-9.04	4.01	2.43E-02	-8.62	8.64	3.19E-01	-3.82	12.23	7.55E-01	-9.27	5.32	8.12E-02	-13.93	12.28	2.57E-01
cg11609001	18.25	8.10	2.43E-02	18.71	20.19	3.54E-01	37.81	36.21	2.96E-01	13.08	10.03	1.92E-01	35.25	21.93	1.08E-01
cg13540312	-26.13	11.60	2.43E-02	-38.80	21.44	7.04E-02	-20.33	54.85	7.11E-01	-26.11	15.66	9.55E-02	4.08	34.38	9.06E-01
cg22789976	-9.65	4.28	2.43E-02	-11.25	5.69	4.80E-02	-11.51	10.71	2.82E-01	15.33	15.33	3.17E-01	-13.45	9.70	1.65E-01
cg09670128	14.24	6.32	2.43E-02	41.25	25.28	1.03E-01	81.82	62.89	1.93E-01	11.11	6.91	1.08E-01	16.97	21.02	4.20E-01
cg02223962	10.28	4.57	2.43E-02	-2.69	15.38	8.61E-01	1.19	22.30	9.58E-01	13.33	5.06	8.40E-03	-7.05	19.41	7.16E-01
cg01118730	18.88	8.38	2.43E-02	10.88	18.30	5.52E-01	55.78	38.68	1.49E-01	20.73	10.51	4.86E-02	7.36	25.65	7.74E-01
cg21308322	-16.51	7.33	2.43E-02	-28.14	20.40	1.68E-01	-0.68	27.86	9.81E-01	-15.54	8.56	6.95E-02	-20.97	28.07	4.55E-01
cg12131510	12.86	5.71	2.43E-02	18.70	12.73	1.42E-01	-3.96	13.08	7.62E-01	15.49	7.53	3.97E-02	28.52	31.38	3.63E-01
cg24787335	9.44	4.19	2.43E-02	8.24	6.36	1.95E-01	17.87	8.12	2.78E-02	-1.22	13.70	9.29E-01	5.92	9.26	5.23E-01
cg08299783	-8.55	3.80	2.44E-02	-10.45	6.27	9.55E-02	-16.58	8.26	4.48E-02	-2.51	7.58	7.40E-01	-3.42	9.17	7.10E-01
cg16555505	-10.61	4.71	2.44E-02	-13.83	8.28	9.49E-02	-3.73	14.22	7.93E-01	-15.36	7.75	4.74E-02	-0.22	10.61	9.83E-01
cg11884093	-82.20	36.51	2.44E-02	-149.68	54.67	6.18E-03	-382.40	270.11	1.57E-01	-67.02	28.54	1.89E-02	6.40	73.61	9.31E-01
cg25656096	-20.89	9.28	2.44E-02	-2.90	26.02	9.11E-01	-11.00	55.21	8.42E-01	-27.42	10.42	8.50E-03	29.44	40.70	4.70E-01
cg13696132	32.92	14.62	2.44E-02	51.20	35.65	1.51E-01	25.24	56.75	6.57E-01	31.37	18.82	9.55E-02	22.83	36.33	5.30E-01
cg05889881	15.37	6.83	2.44E-02	15.22	11.54	1.87E-01	20.83	11.56	7.16E-02	0.89	17.65	9.60E-01	17.48	17.55	3.19E-01
cg03078672	16.48	7.32	2.44E-02	33.96	15.97	3.35E-02	18.66	15.15	2.18E-01	3.99	10.95	7.16E-01	29.40	22.18	1.85E-01
cg13096433	-12.32	5.47	2.44E-02	-13.09	15.43	3.96E-01	15.27	25.01	5.42E-01	-12.77	6.25	4.10E-02	-27.06	22.44	2.28E-01
cg18990588	-13.14	5.84	2.44E-02	-25.18	9.23	6.36E-03	-4.05	12.63	7.48E-01	-10.97	16.79	5.13E-01	-3.29	11.34	7.72E-01

cg26671848	-13.15	5.84	2.44E-02	-24.58	9.45	9.27E-03	4.95	13.70	7.18E-01	-9.15	7.70	2.35E-01	-20.16	13.74	1.42E-01
cg22954687	9.41	4.18	2.44E-02	11.89	5.35	2.63E-02	13.38	10.46	2.01E-01	-8.07	18.35	6.60E-01	2.48	9.87	8.02E-01
cg11957798	-44.54	19.78	2.44E-02	-44.72	27.44	1.03E-01	60.21	79.58	4.49E-01	-50.15	43.49	2.49E-01	-69.23	43.03	1.08E-01
cg20600053	-31.21	13.87	2.44E-02	-8.86	22.16	6.89E-01	-109.26	50.58	3.08E-02	-35.47	21.01	9.14E-02	-31.62	25.89	2.22E-01
cg13934406	-11.68	5.19	2.44E-02	-14.30	6.82	3.61E-02	-4.15	10.41	6.90E-01	-35.35	15.50	2.26E-02	-4.69	8.82	5.95E-01
cg00388605	-20.23	8.99	2.44E-02	-24.58	14.92	9.96E-02	18.62	33.24	5.75E-01	-14.68	14.93	3.26E-01	-36.52	20.03	6.82E-02
cg24324179	21.47	9.54	2.44E-02	47.59	22.47	3.41E-02	-10.77	80.33	8.93E-01	15.59	11.25	1.66E-01	21.20	32.37	5.13E-01
cg21638454	-11.88	5.28	2.44E-02	-8.75	15.52	5.73E-01	-26.72	20.26	1.87E-01	-10.21	6.10	9.38E-02	-21.07	20.53	3.05E-01
cg00545469	-12.00	5.33	2.44E-02	3.35	15.40	8.28E-01	20.81	39.78	6.01E-01	-15.45	6.03	1.04E-02	-8.70	18.85	6.44E-01
cg10860578	-17.73	7.88	2.44E-02	-17.01	13.43	2.05E-01	-4.69	19.67	8.12E-01	-20.78	14.99	1.66E-01	-24.56	16.82	1.44E-01
cg06852305	13.30	5.91	2.44E-02	7.65	17.61	6.64E-01	33.51	26.23	2.01E-01	14.05	6.63	3.42E-02	-9.78	28.64	7.33E-01
cg03476195	11.22	4.99	2.44E-02	15.45	7.59	4.17E-02	2.33	10.47	8.24E-01	32.91	19.87	9.78E-02	7.00	9.45	4.59E-01
cg19516647	26.83	11.92	2.44E-02	-7.47	37.92	8.44E-01	15.31	157.00	9.22E-01	34.07	13.35	1.07E-02	3.04	38.20	9.37E-01
cg10496619	-15.42	6.85	2.44E-02	-15.03	21.16	4.78E-01	-60.44	29.16	3.82E-02	-11.71	7.69	1.28E-01	-25.94	31.53	4.11E-01
cg14224196	-59.93	26.63	2.44E-02	3.26	47.72	9.46E-01	24.33	177.07	8.91E-01	-108.44	41.16	8.42E-03	-65.77	40.36	1.03E-01
cg18026235	-15.92	7.08	2.44E-02	-25.62	12.82	4.56E-02	-15.69	15.58	3.14E-01	-6.15	12.63	6.26E-01	-16.83	16.90	3.19E-01
cg26406924	-8.62	3.83	2.44E-02	-15.73	9.30	9.09E-02	1.78	14.03	8.99E-01	-9.93	4.91	4.29E-02	-0.20	10.03	9.84E-01
cg04374719	-7.89	3.51	2.44E-02	-13.28	7.40	7.27E-02	-10.11	13.93	4.68E-01	-5.31	4.48	2.36E-01	-10.16	11.08	3.59E-01
cg02891314	-2.02	0.90	2.44E-02	-3.00	1.20	1.27E-02	-1.00	1.96	6.09E-01	-3.83	2.46	1.20E-01	0.59	1.98	7.67E-01
cg23172080	-11.90	5.29	2.44E-02	-16.03	11.65	1.69E-01	1.92	22.69	9.33E-01	-7.63	5.65	1.77E-01	-33.86	14.76	2.18E-02
cg07123890	15.77	7.01	2.44E-02	1.51	20.76	9.42E-01	-13.15	28.07	6.39E-01	18.86	8.09	1.97E-02	30.85	25.87	2.33E-01
cg10091408	-8.34	3.71	2.44E-02	-13.30	5.46	1.48E-02	-1.68	8.17	8.37E-01	-11.47	10.41	2.71E-01	-1.97	8.16	8.09E-01
cg07482373	14.27	6.34	2.44E-02	15.70	16.89	3.53E-01	80.43	66.31	2.25E-01	16.24	7.21	2.42E-02	-16.59	23.06	4.72E-01
cg26608667	8.75	3.89	2.44E-02	7.70	5.55	1.65E-01	21.81	9.18	1.75E-02	3.41	8.40	6.85E-01	2.81	11.50	8.07E-01
cg16503618	24.62	10.94	2.44E-02	26.25	23.48	2.64E-01	-15.89	107.16	8.82E-01	24.81	13.42	6.44E-02	24.05	33.37	4.71E-01
cg09577455	9.78	4.35	2.44E-02	12.88	5.63	2.23E-02	12.11	10.21	2.35E-01	1.58	16.50	9.24E-01	-1.26	11.09	9.10E-01
cg24122751	19.37	8.61	2.44E-02	47.45	20.54	2.09E-02	10.45	20.63	6.13E-01	10.48	11.55	3.64E-01	35.68	27.89	2.01E-01
cg10575219	9.42	4.19	2.44E-02	10.97	5.69	5.37E-02	21.33	11.67	6.76E-02	1.26	11.54	9.13E-01	2.86	9.39	7.61E-01
cg08783317	-7.39	3.28	2.44E-02	-5.60	5.84	3.38E-01	-15.76	7.61	3.83E-02	-8.81	5.09	8.36E-02	4.53	9.06	6.17E-01
cg21227784	-12.21	5.42	2.44E-02	-9.54	13.32	4.74E-01	-5.91	21.58	7.84E-01	-11.73	6.68	7.89E-02	-22.58	16.29	1.66E-01
cg24851370	32.70	14.53	2.44E-02	28.28	20.29	1.63E-01	71.53	61.76	2.47E-01	22.63	45.62	6.20E-01	36.16	25.30	1.53E-01
cg17932631	-32.69	14.53	2.45E-02	-33.56	19.70	8.84E-02	-28.93	36.36	4.26E-01	-42.18	52.05	4.18E-01	-29.89	31.10	3.37E-01
cg06212607	43.81	19.47	2.45E-02	27.66	16.95	1.03E-01	174.40	64.14	6.55E-03	8.15	78.65	9.17E-01	40.55	18.92	3.20E-02
cg25361447	49.90	22.18	2.45E-02	23.44	19.19	2.22E-01	175.64	66.52	8.28E-03	48.10	43.44	2.68E-01	49.25	30.23	1.03E-01
cg22054362	8.89	3.95	2.45E-02	19.77	16.62	2.34E-01	6.80	58.07	9.07E-01	10.04	4.39	2.22E-02	-3.06	11.02	7.81E-01

cg22801149	7.90	3.51	2.45E-02	12.46	4.91	1.12E-02	6.20	7.95	4.36E-01	-1.86	8.43	8.26E-01	5.38	10.08	5.94E-01
cg13185218	8.74	3.88	2.45E-02	7.21	8.73	4.09E-01	13.10	12.63	3.00E-01	9.86	4.93	4.56E-02	-0.48	13.13	9.71E-01
cg03572260	7.14	3.18	2.45E-02	9.22	4.92	6.07E-02	9.30	7.90	2.39E-01	5.99	5.66	2.90E-01	-0.89	9.75	9.27E-01
cg23875758	18.89	8.40	2.45E-02	15.82	13.62	2.46E-01	37.14	15.63	1.75E-02	-4.56	18.76	8.08E-01	23.28	22.70	3.05E-01
cg22787468	10.45	4.65	2.45E-02	18.11	7.84	2.09E-02	15.99	10.84	1.40E-01	1.78	7.86	8.21E-01	4.59	13.67	7.37E-01
cg13185413	3.20	1.42	2.45E-02	2.41	2.08	2.46E-01	4.79	2.85	9.37E-02	4.51	4.13	2.75E-01	2.12	3.54	5.49E-01
cg13639602	10.54	4.69	2.45E-02	11.58	6.48	7.38E-02	10.03	11.48	3.83E-01	11.62	11.53	3.13E-01	6.15	12.30	6.17E-01
cg12974388	19.07	8.48	2.45E-02	59.99	36.51	1.00E-01	104.53	109.76	3.41E-01	18.55	9.13	4.21E-02	-10.34	30.51	7.35E-01
cg08645257	13.10	5.82	2.45E-02	19.39	8.69	2.56E-02	14.03	29.32	6.32E-01	26.00	22.92	2.56E-01	4.83	8.71	5.79E-01
cg26837399	6.00	2.67	2.45E-02	3.80	3.33	2.54E-01	15.27	7.33	3.71E-02	9.86	8.69	2.56E-01	4.63	7.35	5.29E-01
cg24974217	-14.52	6.46	2.45E-02	-18.59	10.25	6.97E-02	-22.07	15.05	1.43E-01	4.01	15.82	8.00E-01	-14.86	12.85	2.48E-01
cg18705067	67.93	30.20	2.45E-02	41.54	41.87	3.21E-01	25.58	147.45	8.62E-01	59.25	72.73	4.15E-01	131.96	58.61	2.44E-02
cg12013685	-8.64	3.84	2.45E-02	-15.18	5.73	8.11E-03	-6.42	8.26	4.37E-01	-1.27	12.99	9.22E-01	-1.32	7.72	8.64E-01
cg27498197	-10.82	4.81	2.45E-02	-16.87	13.10	1.98E-01	0.65	18.10	9.72E-01	-12.01	5.66	3.39E-02	0.99	17.73	9.56E-01
cg25341925	-10.76	4.78	2.45E-02	-2.76	12.04	8.19E-01	-10.41	17.68	5.56E-01	-11.81	5.87	4.42E-02	-16.39	14.79	2.68E-01
cg11843333	-13.45	5.98	2.45E-02	-20.50	16.83	2.23E-01	10.95	30.74	7.22E-01	-11.71	6.89	8.90E-02	-29.81	20.87	1.53E-01
cg01882930	-12.42	5.52	2.45E-02	-18.87	9.53	4.76E-02	-14.47	12.10	2.32E-01	5.41	14.32	7.05E-01	-12.62	9.97	2.06E-01
cg26391564	-6.14	2.73	2.45E-02	-5.65	3.91	1.49E-01	-12.23	5.37	2.27E-02	-2.59	13.22	8.45E-01	-0.57	5.90	9.23E-01
cg22388347	-10.43	4.64	2.45E-02	-11.79	9.08	1.94E-01	-10.22	13.17	4.38E-01	-4.20	6.92	5.44E-01	-25.37	11.40	2.60E-02
cg13096128	13.36	5.94	2.45E-02	26.34	13.55	5.19E-02	35.06	27.23	1.98E-01	9.67	7.05	1.70E-01	-4.75	26.52	8.58E-01
cg25554998	17.13	7.62	2.45E-02	4.62	15.41	7.64E-01	22.61	15.46	1.44E-01	17.44	12.39	1.59E-01	29.00	20.71	1.61E-01
cg14436426	11.83	5.26	2.45E-02	17.33	7.95	2.92E-02	18.22	10.57	8.48E-02	-4.68	12.53	7.09E-01	3.98	14.19	7.79E-01
cg09308801	19.61	8.72	2.45E-02	27.91	17.65	1.14E-01	23.44	18.37	2.02E-01	20.79	13.79	1.32E-01	-6.10	24.13	8.00E-01
cg11121252	21.15	9.41	2.45E-02	19.70	13.97	1.58E-01	7.72	25.29	7.60E-01	42.60	24.62	8.36E-02	18.81	18.37	3.06E-01
cg08475042	-11.03	4.90	2.45E-02	-18.95	13.41	1.58E-01	8.80	21.47	6.82E-01	-10.46	5.67	6.52E-02	-16.91	18.88	3.71E-01
cg09039685	-5.06	2.25	2.45E-02	-6.92	3.80	6.82E-02	-0.39	5.22	9.40E-01	-7.17	4.34	9.86E-02	-3.24	5.12	5.26E-01
cg26681097	-9.28	4.13	2.45E-02	-15.98	10.06	1.12E-01	-18.46	18.81	3.26E-01	-5.43	5.08	2.85E-01	-17.23	11.77	1.43E-01
cg03557391	32.46	14.43	2.45E-02	27.22	17.47	1.19E-01	-3.04	52.79	9.54E-01	100.85	45.07	2.53E-02	26.85	38.59	4.87E-01
cg12800270	25.71	11.44	2.45E-02	51.70	18.06	4.20E-03	17.03	18.17	3.49E-01	3.95	16.46	8.10E-01	38.46	26.40	1.45E-01
cg10782297	48.15	21.41	2.45E-02	16.50	54.56	7.62E-01	-174.24	267.20	5.14E-01	50.81	25.42	4.56E-02	82.16	59.42	1.67E-01
cg13931134	-11.32	5.04	2.45E-02	-10.58	11.49	3.57E-01	-24.30	14.35	9.05E-02	-8.74	6.85	2.02E-01	-10.93	13.24	4.09E-01
cg23043389	9.38	4.17	2.45E-02	1.40	6.38	8.26E-01	14.83	8.63	8.57E-02	16.10	14.49	2.67E-01	15.52	8.24	5.95E-02
cg23716690	-9.16	4.07	2.46E-02	-11.12	7.59	1.43E-01	-17.66	11.01	1.09E-01	-4.30	6.06	4.78E-01	-12.94	11.59	2.64E-01
cg04667664	22.43	9.98	2.46E-02	18.48	16.01	2.49E-01	1.97	16.62	9.06E-01	26.93	17.50	1.24E-01	54.72	21.99	1.28E-02
cg24321030	-40.78	18.14	2.46E-02	-94.57	54.60	8.32E-02	-243.00	163.59	1.37E-01	-32.95	20.66	1.11E-01	-18.30	55.68	7.42E-01

cg06177555	7.85	3.49	2.46E-02	6.86	4.76	1.49E-01	11.53	7.31	1.14E-01	20.72	11.06	6.09E-02	-2.61	8.91	7.69E-01
cg04174777	-11.20	4.98	2.46E-02	-0.95	12.96	9.42E-01	4.10	17.27	8.12E-01	-13.09	6.07	3.11E-02	-26.80	16.00	9.39E-02
cg11854259	21.62	9.62	2.46E-02	38.45	14.17	6.64E-03	2.50	26.95	9.26E-01	2.02	27.79	9.42E-01	11.43	17.81	5.21E-01
cg07211972	5.00	2.23	2.46E-02	2.76	2.71	3.09E-01	10.53	4.59	2.17E-02	9.12	5.46	9.53E-02	0.15	5.78	9.79E-01
cg27220062	-8.11	3.61	2.46E-02	-5.30	5.27	3.15E-01	-16.48	8.80	6.12E-02	-2.93	8.60	7.33E-01	-12.52	8.35	1.34E-01
cg08477744	20.50	9.12	2.46E-02	26.50	20.47	1.95E-01	13.95	18.92	4.61E-01	22.00	13.56	1.05E-01	17.52	26.64	5.11E-01
cg26183708	-17.79	7.92	2.46E-02	-25.92	11.34	2.23E-02	-0.38	32.08	9.91E-01	5.38	22.29	8.09E-01	-17.87	13.86	1.98E-01
cg18081338	14.61	6.50	2.46E-02	6.14	13.68	6.54E-01	17.12	24.55	4.86E-01	11.76	8.56	1.70E-01	40.84	18.12	2.42E-02
cg25832824	15.67	6.97	2.46E-02	0.00	29.27	1.00E+00	74.51	114.61	5.16E-01	16.04	7.41	3.05E-02	21.94	29.64	4.59E-01
cg22106273	-8.90	3.96	2.46E-02	0.74	5.15	8.86E-01	-12.60	7.54	9.48E-02	-12.58	5.38	1.94E-02	-15.00	7.52	4.60E-02
cg22374742	-8.19	3.64	2.46E-02	-11.59	5.17	2.49E-02	2.09	10.84	8.47E-01	-6.01	12.08	6.19E-01	-7.07	6.66	2.88E-01
cg02138331	5.70	2.54	2.46E-02	6.40	4.09	1.17E-01	8.68	7.03	2.17E-01	5.66	4.13	1.71E-01	-0.33	7.80	9.66E-01
cg12723425	12.17	5.41	2.46E-02	11.63	7.53	1.23E-01	14.97	11.24	1.83E-01	21.24	14.92	1.55E-01	-0.91	15.63	9.54E-01
cg16589135	-19.89	8.85	2.46E-02	-8.78	30.90	7.76E-01	-135.15	102.90	1.89E-01	-22.07	9.71	2.30E-02	1.94	31.25	9.50E-01
cg01157404	17.78	7.91	2.46E-02	24.08	11.97	4.42E-02	11.08	15.82	4.84E-01	31.81	31.73	3.16E-01	10.01	15.78	5.26E-01
cg00421541	-6.69	2.98	2.46E-02	-8.45	5.39	1.17E-01	-5.93	7.12	4.05E-01	-9.69	4.81	4.40E-02	4.66	8.06	5.63E-01
cg21286402	19.86	8.84	2.46E-02	18.15	20.67	3.80E-01	31.66	29.93	2.90E-01	24.72	12.53	4.85E-02	6.39	18.32	7.27E-01
cg05664833	-12.01	5.35	2.46E-02	-13.03	11.36	2.51E-01	-6.06	12.71	6.33E-01	-12.45	8.09	1.24E-01	-15.86	13.13	2.27E-01
cg13298167	-11.12	4.95	2.46E-02	-17.01	9.37	6.95E-02	-12.22	14.81	4.09E-01	-7.76	7.27	2.86E-01	-9.69	12.92	4.53E-01
cg13972054	27.80	12.37	2.46E-02	26.11	15.81	9.87E-02	-26.92	72.12	7.09E-01	122.04	64.45	5.83E-02	25.23	21.80	2.47E-01
cg22967329	44.06	19.60	2.46E-02	81.60	54.90	1.37E-01	-203.80	205.59	3.22E-01	43.55	22.69	5.49E-02	25.57	57.38	6.56E-01
cg09155881	-13.38	5.96	2.46E-02	-23.17	9.69	1.67E-02	-7.31	13.66	5.93E-01	-1.52	13.58	9.11E-01	-12.27	12.16	3.13E-01
cg27081230	-11.43	5.09	2.46E-02	-12.21	7.69	1.12E-01	2.52	13.09	8.47E-01	-4.97	12.45	6.90E-01	-23.02	10.27	2.51E-02
cg01317029	13.64	6.07	2.46E-02	9.46	8.51	2.67E-01	31.95	14.04	2.29E-02	1.61	18.09	9.29E-01	13.95	13.84	3.13E-01
cg00972603	25.69	11.43	2.46E-02	68.80	32.51	3.43E-02	8.79	100.54	9.30E-01	25.27	14.70	8.57E-02	6.95	22.46	7.57E-01
cg13497903	9.57	4.26	2.46E-02	8.36	6.48	1.97E-01	7.43	8.15	3.62E-01	3.46	13.95	8.04E-01	17.90	9.49	5.92E-02
cg25311807	16.50	7.34	2.46E-02	36.05	19.24	6.09E-02	22.38	22.44	3.18E-01	8.58	8.99	3.40E-01	38.88	25.89	1.33E-01
cg19702397	16.56	7.37	2.46E-02	16.43	15.99	3.04E-01	-4.54	27.99	8.71E-01	22.02	9.30	1.79E-02	-4.94	24.56	8.40E-01
cg12396999	-49.15	21.87	2.46E-02	-41.01	27.90	1.42E-01	-47.58	75.98	5.31E-01	-46.12	86.18	5.93E-01	-71.55	44.82	1.10E-01
cg08955754	-38.78	17.26	2.46E-02	-39.27	21.82	7.19E-02	-56.18	47.73	2.39E-01	-10.08	80.48	9.00E-01	-32.41	38.80	4.04E-01
cg07187280	27.07	12.05	2.46E-02	24.25	21.47	2.59E-01	-5.39	76.58	9.44E-01	28.43	16.09	7.72E-02	36.41	38.20	3.40E-01
cg09313745	-44.21	19.67	2.46E-02	-50.60	24.74	4.08E-02	110.00	91.12	2.27E-01	-68.67	48.89	1.60E-01	-43.63	37.30	2.42E-01
cg02675896	-37.14	16.53	2.46E-02	-32.37	56.33	5.66E-01	54.11	238.49	8.21E-01	-41.71	18.09	2.11E-02	2.72	60.63	9.64E-01
cg18303410	-12.38	5.51	2.46E-02	-21.03	8.84	1.74E-02	-5.71	11.37	6.15E-01	-3.34	6.34	5.99E-01	-24.40	10.64	2.19E-02
cg05332152	16.05	7.14	2.47E-02	18.28	12.64	1.48E-01	21.84	17.00	1.99E-01	6.32	11.56	5.85E-01	32.26	20.43	1.14E-01

cg26499363	19.76	8.79	2.47E-02	17.26	12.34	1.62E-01	18.29	24.84	4.62E-01	49.18	26.00	5.85E-02	12.16	17.51	4.87E-01
cg05815920	27.26	12.13	2.47E-02	30.48	36.40	4.02E-01	-68.87	130.82	5.99E-01	33.12	13.11	1.15E-02	-49.02	50.67	3.33E-01
cg05424782	19.83	8.83	2.47E-02	30.09	13.14	2.21E-02	42.39	22.54	6.00E-02	8.00	52.94	8.80E-01	4.53	11.94	7.04E-01
cg24626312	13.10	5.83	2.47E-02	25.33	13.98	7.01E-02	17.67	46.34	7.03E-01	11.53	6.77	8.84E-02	-2.09	22.33	9.26E-01
cg07460919	12.57	5.60	2.47E-02	15.42	8.64	7.42E-02	19.82	16.54	2.31E-01	13.56	10.45	1.95E-01	-0.33	13.23	9.80E-01
cg04583232	15.67	6.98	2.47E-02	26.30	10.19	9.86E-03	13.82	18.86	4.64E-01	18.64	11.59	1.08E-01	-6.30	14.35	6.61E-01
cg20313392	27.19	12.10	2.47E-02	18.54	42.06	6.59E-01	7.70	175.66	9.65E-01	30.20	13.17	2.19E-02	1.76	46.35	9.70E-01
cg01823120	-11.35	5.05	2.47E-02	-26.96	10.91	1.35E-02	-5.11	11.47	6.56E-01	-9.59	7.27	1.87E-01	0.55	15.40	9.71E-01
cg08872494	-24.94	11.10	2.47E-02	-20.96	14.28	1.42E-01	-178.68	149.45	2.32E-01	-40.77	23.92	8.83E-02	-14.34	26.53	5.89E-01
cg13222081	-24.83	11.05	2.47E-02	-33.18	13.42	1.34E-02	-42.32	66.22	5.23E-01	22.36	59.05	7.05E-01	-7.42	21.75	7.33E-01
cg08101375	21.71	9.67	2.47E-02	47.39	25.79	6.61E-02	28.45	51.62	5.82E-01	22.58	12.99	8.20E-02	5.72	18.59	7.58E-01
cg16298867	4.14	1.84	2.47E-02	1.39	2.50	5.79E-01	8.22	3.65	2.45E-02	7.72	8.65	3.72E-01	6.05	4.67	1.95E-01
cg27645498	19.63	8.74	2.47E-02	34.92	13.43	9.32E-03	-1.63	15.73	9.18E-01	15.54	17.09	3.63E-01	29.19	23.24	2.09E-01
cg12470092	-10.97	4.88	2.47E-02	-17.12	7.35	1.99E-02	-4.40	11.81	7.09E-01	-4.59	14.91	7.58E-01	-7.75	9.23	4.01E-01
cg03381996	-53.86	23.98	2.47E-02	-75.63	57.32	1.87E-01	-238.01	284.47	4.03E-01	-52.24	28.51	6.69E-02	-17.84	72.24	8.05E-01
cg05901765	7.73	3.44	2.47E-02	13.42	6.66	4.39E-02	10.27	6.99	1.42E-01	1.51	7.46	8.40E-01	4.82	6.53	4.61E-01
cg26854234	33.23	14.79	2.47E-02	27.13	24.21	2.62E-01	79.58	72.14	2.70E-01	41.87	21.84	5.53E-02	4.32	41.72	9.17E-01
cg09232269	-9.66	4.30	2.47E-02	-9.81	4.94	4.70E-02	0.43	7.15	9.52E-01	-6.46	4.03	1.09E-01	-25.04	7.30	5.98E-04
cg05129597	-8.78	3.91	2.47E-02	-10.58	6.54	1.06E-01	-16.67	7.97	3.66E-02	0.83	7.81	9.15E-01	-7.92	10.02	4.29E-01
cg10764541	32.29	14.38	2.47E-02	75.34	45.63	9.87E-02	-165.09	165.39	3.18E-01	26.76	15.78	8.99E-02	61.29	57.43	2.86E-01
cg10003262	7.30	3.25	2.47E-02	6.56	5.05	1.94E-01	9.80	7.14	1.70E-01	8.28	8.92	3.53E-01	5.91	6.56	3.68E-01
cg16630482	11.15	4.97	2.47E-02	16.05	9.88	1.04E-01	15.15	11.60	1.92E-01	10.19	8.77	2.45E-01	4.34	10.05	6.66E-01
cg06565220	21.82	9.71	2.47E-02	19.51	12.06	1.06E-01	-4.25	44.65	9.24E-01	-47.84	62.26	4.42E-01	37.65	18.37	4.04E-02
cg13253199	18.28	8.14	2.47E-02	29.73	22.67	1.90E-01	27.94	30.40	3.58E-01	14.32	9.51	1.32E-01	29.11	31.40	3.54E-01
cg06482019	26.44	11.77	2.47E-02	20.89	27.45	4.47E-01	74.67	66.76	2.63E-01	19.92	14.48	1.69E-01	57.36	33.46	8.65E-02
cg06474646	29.87	13.30	2.47E-02	-1.28	32.11	9.68E-01	-43.18	115.24	7.08E-01	40.67	15.75	9.80E-03	16.23	41.67	6.97E-01
cg14183865	-8.63	3.84	2.47E-02	-8.56	5.76	1.37E-01	-4.35	8.28	6.00E-01	-18.31	10.05	6.84E-02	-6.24	8.76	4.77E-01
cg06422947	9.51	4.23	2.47E-02	13.46	7.21	6.20E-02	8.75	10.54	4.07E-01	6.74	6.61	3.08E-01	8.28	14.63	5.71E-01
cg15228694	5.28	2.35	2.47E-02	7.43	3.07	1.55E-02	2.89	5.84	6.21E-01	2.87	6.84	6.75E-01	0.88	6.41	8.91E-01
cg23209976	13.08	5.82	2.47E-02	17.10	8.96	5.64E-02	23.34	13.71	8.86E-02	20.11	16.59	2.25E-01	-2.80	11.04	8.00E-01
cg03958004	30.92	13.77	2.47E-02	32.53	37.07	3.80E-01	60.58	151.85	6.90E-01	34.43	15.99	3.13E-02	3.48	41.14	9.33E-01
cg26820636	-7.86	3.50	2.47E-02	-8.29	5.29	1.17E-01	-7.02	8.69	4.19E-01	-17.58	8.93	4.90E-02	-1.60	7.05	8.21E-01
cg20358492	11.87	5.29	2.47E-02	23.49	11.38	3.90E-02	26.42	13.92	5.78E-02	5.51	5.85	3.46E-01	6.49	15.32	6.72E-01
cg13065683	-10.70	4.77	2.47E-02	-12.35	6.52	5.84E-02	-22.96	16.95	1.75E-01	-0.37	14.27	9.80E-01	-8.19	9.08	3.67E-01
cg20586611	15.13	6.74	2.47E-02	8.48	22.09	7.01E-01	22.98	34.91	5.10E-01	17.78	7.41	1.65E-02	-27.44	32.22	3.95E-01

cg16580759	35.30	15.72	2.47E-02	57.53	18.02	1.41E-03	-19.32	31.30	5.37E-01	41.72	35.47	2.40E-01	38.64	23.26	9.66E-02
cg13551141	16.29	7.25	2.47E-02	12.49	10.95	2.54E-01	21.13	14.82	1.54E-01	24.89	16.30	1.27E-01	6.61	20.62	7.49E-01
cg18771855	26.83	11.95	2.47E-02	28.95	29.89	3.33E-01	-187.29	141.30	1.85E-01	24.07	11.65	3.89E-02	61.27	38.05	1.07E-01
cg11518550	22.48	10.01	2.47E-02	23.58	17.29	1.73E-01	58.91	20.26	3.64E-03	10.63	7.18	1.39E-01	15.30	20.91	4.65E-01
cg14129328	-10.96	4.88	2.47E-02	-18.57	9.40	4.82E-02	-5.00	10.76	6.42E-01	-14.16	7.65	6.42E-02	7.31	14.32	6.10E-01
cg14159971	22.42	9.98	2.48E-02	38.55	31.96	2.28E-01	95.07	114.82	4.08E-01	18.84	11.02	8.74E-02	33.22	36.55	3.63E-01
cg19284211	19.19	8.55	2.48E-02	38.67	11.78	1.03E-03	18.16	17.01	2.86E-01	9.99	17.53	5.69E-01	5.11	12.15	6.74E-01
cg03162810	13.90	6.19	2.48E-02	8.36	17.07	6.25E-01	-8.13	23.55	7.30E-01	15.10	7.33	3.93E-02	30.41	21.25	1.52E-01
cg22381248	-10.27	4.57	2.48E-02	-22.80	10.74	3.38E-02	-3.55	18.32	8.46E-01	-6.38	5.56	2.51E-01	-20.02	16.21	2.17E-01
cg19770550	35.06	15.61	2.48E-02	61.36	42.21	1.46E-01	-164.19	157.11	2.96E-01	35.07	17.76	4.84E-02	14.73	54.99	7.89E-01
cg21028142	-4.74	2.11	2.48E-02	-6.89	3.28	3.57E-02	-2.99	4.77	5.30E-01	-3.80	4.89	4.37E-01	-2.91	4.71	5.36E-01
cg12612137	-15.26	6.80	2.48E-02	-10.49	11.11	3.45E-01	-5.69	21.61	7.92E-01	-26.56	12.43	3.27E-02	-12.42	14.23	3.83E-01
cg01875764	20.48	9.12	2.48E-02	25.33	15.31	9.79E-02	18.75	19.90	3.46E-01	25.64	18.01	1.55E-01	5.40	21.62	8.03E-01
cg07223090	29.50	13.14	2.48E-02	19.00	15.53	2.21E-01	11.44	15.69	4.66E-01	23.62	19.25	2.20E-01	79.38	23.28	6.51E-04
cg16745091	39.08	17.41	2.48E-02	47.71	21.74	2.82E-02	78.44	63.34	2.16E-01	4.24	50.44	9.33E-01	12.56	42.98	7.70E-01
cg00281600	29.17	12.99	2.48E-02	20.70	29.15	4.78E-01	17.82	89.95	8.43E-01	27.08	15.66	8.37E-02	65.88	42.94	1.25E-01
cg19673881	18.75	8.35	2.48E-02	30.88	14.22	2.99E-02	25.63	18.21	1.59E-01	2.28	14.46	8.75E-01	17.49	25.01	4.84E-01
cg27272235	13.92	6.20	2.48E-02	10.99	8.39	1.90E-01	32.84	19.57	9.33E-02	27.76	20.98	1.86E-01	8.24	12.03	4.93E-01
cg03510310	20.71	9.22	2.48E-02	20.05	18.48	2.78E-01	43.34	23.26	6.24E-02	12.07	13.36	3.66E-01	26.94	27.04	3.19E-01
cg24396686	-10.88	4.85	2.48E-02	-4.09	14.05	7.71E-01	-1.55	14.87	9.17E-01	-12.94	5.76	2.47E-02	-16.08	18.84	3.93E-01
cg23238790	12.15	5.41	2.48E-02	23.54	13.37	7.83E-02	9.24	26.76	7.30E-01	9.90	6.31	1.17E-01	10.61	22.21	6.33E-01
cg14380939	-9.06	4.03	2.48E-02	-8.59	6.26	1.70E-01	-1.26	8.55	8.82E-01	-12.48	10.30	2.25E-01	-15.81	8.84	7.39E-02
cg25971314	42.22	18.81	2.48E-02	30.23	22.33	1.76E-01	73.54	64.10	2.51E-01	49.18	75.83	5.17E-01	79.96	49.80	1.08E-01
cg19277182	49.38	22.00	2.48E-02	43.89	30.26	1.47E-01	104.27	77.13	1.76E-01	22.56	55.67	6.85E-01	60.58	45.49	1.83E-01
cg07818646	-35.57	15.85	2.48E-02	-26.25	40.55	5.17E-01	-116.22	125.51	3.54E-01	-29.63	18.38	1.07E-01	-87.52	53.52	1.02E-01
cg16024377	-9.96	4.44	2.48E-02	-21.06	9.42	2.54E-02	-23.54	20.41	2.49E-01	-5.90	5.59	2.91E-01	-4.52	13.94	7.46E-01
cg25261328	12.90	5.75	2.48E-02	15.44	8.25	6.12E-02	5.28	13.30	6.91E-01	48.06	25.75	6.20E-02	7.27	10.91	5.05E-01
cg03157862	-13.47	6.00	2.48E-02	-16.61	9.43	7.81E-02	-18.89	15.24	2.15E-01	-4.10	12.03	7.33E-01	-14.64	13.76	2.87E-01
cg10750959	9.83	4.38	2.48E-02	12.94	5.95	2.97E-02	13.45	9.89	1.74E-01	-2.79	14.37	8.46E-01	2.62	10.64	8.05E-01
cg13440265	-36.98	16.48	2.48E-02	-40.46	21.39	5.86E-02	-2.39	53.17	9.64E-01	-23.68	63.90	7.11E-01	-45.77	33.35	1.70E-01
cg03944089	13.86	6.18	2.48E-02	13.01	8.57	1.29E-01	11.83	15.07	4.33E-01	17.92	23.23	4.41E-01	15.92	12.54	2.04E-01
cg17454592	16.18	7.21	2.48E-02	16.00	10.14	1.14E-01	23.66	16.78	1.59E-01	22.22	25.16	3.77E-01	8.32	15.10	5.82E-01
cg14231845	-9.09	4.05	2.48E-02	-21.64	9.98	3.01E-02	-1.87	17.57	9.15E-01	-6.16	4.85	2.04E-01	-13.20	13.84	3.40E-01
cg05208249	-11.35	5.06	2.48E-02	-23.43	11.72	4.56E-02	-18.39	14.23	1.96E-01	-6.19	6.93	3.72E-01	-8.84	12.86	4.92E-01
cg07575680	13.08	5.83	2.48E-02	22.69	9.47	1.66E-02	4.86	12.06	6.87E-01	6.54	11.94	5.84E-01	12.02	15.10	4.26E-01

cg23007087	5.33	2.37	2.48E-02	4.09	3.51	2.44E-01	5.00	5.23	3.39E-01	10.68	4.87	2.84E-02	-1.10	7.53	8.84E-01
cg23940999	21.84	9.73	2.48E-02	27.14	12.22	2.63E-02	-38.04	65.54	5.62E-01	-4.14	41.24	9.20E-01	19.78	18.15	2.76E-01
cg06979669	11.29	5.03	2.48E-02	10.53	6.59	1.10E-01	4.65	12.74	7.15E-01	18.14	22.38	4.18E-01	16.63	10.93	1.28E-01
cg24398450	-29.96	13.35	2.48E-02	-23.15	15.14	1.26E-01	-57.44	64.83	3.76E-01	-77.84	62.18	2.11E-01	-44.38	36.53	2.24E-01
cg00733328	9.57	4.26	2.48E-02	10.19	5.98	8.83E-02	14.42	9.62	1.34E-01	5.94	12.57	6.36E-01	4.84	10.03	6.30E-01
cg02097152	6.39	2.85	2.48E-02	5.69	3.88	1.42E-01	10.53	7.85	1.79E-01	11.21	6.89	1.04E-01	0.12	7.16	9.87E-01
cg17886420	-4.46	1.99	2.48E-02	-5.09	3.11	1.02E-01	-2.23	4.33	6.07E-01	-4.40	4.03	2.74E-01	-6.08	5.34	2.54E-01
cg27442164	14.93	6.65	2.48E-02	16.43	10.40	1.14E-01	21.99	15.32	1.51E-01	0.02	13.99	9.99E-01	23.07	15.87	1.46E-01
cg13910860	28.01	12.48	2.48E-02	40.88	21.28	5.47E-02	-40.80	46.99	3.85E-01	42.01	28.35	1.38E-01	22.16	19.94	2.66E-01
cg04302290	30.56	13.62	2.48E-02	17.93	14.08	2.03E-01	47.64	60.95	4.34E-01	162.11	89.06	6.87E-02	43.32	22.32	5.23E-02
cg16181718	4.90	2.18	2.49E-02	2.86	3.97	4.72E-01	8.11	5.32	1.27E-01	6.29	3.44	6.75E-02	1.08	6.12	8.60E-01
cg18155632	-42.58	18.98	2.49E-02	-41.29	24.11	8.68E-02	-68.36	69.41	3.25E-01	-65.44	75.52	3.86E-01	-31.97	38.51	4.06E-01
cg24642523	-13.70	6.10	2.49E-02	-11.78	8.95	1.88E-01	-3.86	15.58	8.04E-01	-18.45	15.06	2.21E-01	-21.16	13.11	1.06E-01
cg27305383	-26.12	11.64	2.49E-02	-23.81	16.34	1.45E-01	-44.08	50.17	3.80E-01	30.23	41.57	4.67E-01	-38.96	19.40	4.46E-02
cg14859854	39.15	17.45	2.49E-02	23.29	22.37	2.98E-01	60.98	55.42	2.71E-01	47.72	50.56	3.45E-01	76.44	41.92	6.82E-02
cg17080697	5.42	2.42	2.49E-02	3.47	3.18	2.75E-01	12.89	6.14	3.56E-02	6.81	6.79	3.15E-01	3.95	6.44	5.40E-01
cg16080656	26.47	11.80	2.49E-02	30.61	39.43	4.38E-01	52.54	155.78	7.36E-01	28.44	12.82	2.65E-02	-11.84	49.32	8.10E-01
cg09492640	-8.95	3.99	2.49E-02	-16.31	8.64	5.92E-02	-11.22	12.46	3.68E-01	-3.38	5.36	5.28E-01	-18.73	11.01	8.91E-02
cg00169677	18.35	8.18	2.49E-02	28.49	15.85	7.21E-02	15.59	17.39	3.70E-01	8.20	9.58	3.92E-01	57.18	27.47	3.74E-02
cg01066451	20.22	9.01	2.49E-02	30.52	18.99	1.08E-01	1.10	18.20	9.52E-01	15.94	9.41	9.02E-02	59.58	26.66	2.54E-02
cg05232215	8.35	3.72	2.49E-02	5.17	5.25	3.24E-01	18.55	7.21	1.02E-02	-0.94	9.09	9.18E-01	9.79	7.52	1.93E-01
cg02699829	11.28	5.03	2.49E-02	16.87	7.22	1.95E-02	18.21	9.41	5.31E-02	-2.22	13.10	8.65E-01	-0.33	11.14	9.76E-01
cg21044104	-10.14	4.52	2.49E-02	-8.95	13.43	5.05E-01	-8.12	16.25	6.17E-01	-11.33	5.25	3.09E-02	-1.51	17.22	9.30E-01
cg05317600	-9.56	4.26	2.49E-02	-13.06	7.74	9.19E-02	-3.85	16.84	8.19E-01	-4.87	4.77	3.07E-01	-27.05	11.99	2.41E-02
cg25941544	20.60	9.18	2.49E-02	12.15	15.70	4.39E-01	7.36	30.52	8.09E-01	22.24	14.23	1.18E-01	43.12	23.62	6.80E-02
cg17075459	-15.50	6.91	2.49E-02	-24.22	12.68	5.62E-02	-9.32	16.85	5.80E-01	-17.84	12.91	1.67E-01	-6.57	13.86	6.35E-01
cg04707531	-15.04	6.71	2.49E-02	-14.31	15.06	3.42E-01	-12.84	42.62	7.63E-01	-11.75	8.03	1.43E-01	-46.23	23.72	5.13E-02
cg19553869	15.79	7.04	2.49E-02	31.85	11.77	6.82E-03	14.30	23.21	5.38E-01	7.11	12.21	5.60E-01	3.87	14.78	7.93E-01
cg20759600	-14.61	6.51	2.49E-02	-17.65	13.43	1.89E-01	-18.03	17.15	2.93E-01	-13.32	10.55	2.07E-01	-11.61	13.32	3.83E-01
cg24391471	23.47	10.46	2.49E-02	23.44	16.66	1.59E-01	27.01	18.84	1.52E-01	41.29	35.19	2.41E-01	10.75	22.89	6.39E-01
cg18038894	5.98	2.67	2.49E-02	4.69	2.94	1.10E-01	15.73	6.63	1.76E-02	6.97	8.61	4.19E-01	-1.51	8.30	8.56E-01
cg11186850	-32.37	14.43	2.49E-02	-40.26	20.44	4.89E-02	31.97	78.10	6.82E-01	-66.02	34.12	5.30E-02	-5.49	26.85	8.38E-01
cg03095379	-56.00	24.97	2.49E-02	-43.05	38.39	2.62E-01	-176.77	158.73	2.65E-01	-31.96	21.94	1.45E-01	-146.49	58.59	1.24E-02
cg18361420	12.15	5.42	2.49E-02	6.34	7.64	4.07E-01	10.72	14.00	4.44E-01	8.56	27.03	7.51E-01	22.77	9.76	1.96E-02
cg27337124	9.80	4.37	2.49E-02	2.67	8.59	7.56E-01	11.37	11.99	3.43E-01	9.06	6.29	1.50E-01	25.72	12.34	3.71E-02

cg01195276	17.80	7.94	2.49E-02	27.39	16.66	1.00E-01	-39.90	58.09	4.92E-01	16.57	9.80	9.09E-02	14.86	25.34	5.58E-01
cg14661813	35.97	16.04	2.49E-02	16.85	51.63	7.44E-01	-130.16	172.25	4.50E-01	42.16	17.79	1.78E-02	14.68	56.04	7.93E-01
cg09507884	-33.75	15.05	2.49E-02	-38.91	17.77	2.86E-02	-9.70	80.35	9.04E-01	29.70	65.34	6.50E-01	-36.37	34.10	2.86E-01
cg02117924	17.96	8.01	2.49E-02	22.10	21.36	3.01E-01	31.20	37.09	4.00E-01	16.58	9.31	7.48E-02	15.57	29.87	6.02E-01
cg10685612	11.47	5.11	2.49E-02	12.20	8.27	1.40E-01	14.73	12.85	2.52E-01	14.03	13.06	2.83E-01	7.59	9.24	4.12E-01
cg24074477	8.01	3.57	2.49E-02	7.55	4.99	1.31E-01	10.58	9.15	2.48E-01	7.93	8.87	3.72E-01	7.21	8.58	4.01E-01
cg16165370	33.21	14.81	2.49E-02	51.71	20.51	1.17E-02	37.21	39.90	3.51E-01	10.58	9.34	2.58E-01	64.33	31.44	4.07E-02
cg05560873	22.17	9.89	2.49E-02	16.44	22.38	4.62E-01	39.10	56.26	4.87E-01	21.28	11.94	7.48E-02	35.81	33.24	2.81E-01
cg03134083	-20.08	8.96	2.49E-02	-27.62	20.34	1.75E-01	22.00	40.55	5.87E-01	-16.36	11.07	1.40E-01	-49.52	27.91	7.60E-02
cg14360139	12.65	5.64	2.49E-02	15.38	9.83	1.18E-01	7.69	13.45	5.67E-01	16.76	15.31	2.74E-01	11.04	9.42	2.42E-01
cg18264092	-10.16	4.53	2.49E-02	-12.48	7.70	1.05E-01	-3.98	12.58	7.52E-01	-12.19	8.26	1.40E-01	-7.42	9.60	4.40E-01
cg04297067	17.98	8.02	2.49E-02	35.85	18.25	4.95E-02	-6.15	20.11	7.60E-01	18.90	11.50	1.00E-01	17.60	19.97	3.78E-01
cg18271897	8.00	3.57	2.49E-02	6.95	4.75	1.43E-01	16.29	8.71	6.16E-02	6.41	9.97	5.20E-01	3.74	9.58	6.96E-01
cg15947534	14.06	6.27	2.49E-02	19.15	9.61	4.63E-02	7.40	13.10	5.72E-01	20.14	17.73	2.56E-01	7.70	13.37	5.65E-01
cg26599006	24.90	11.11	2.50E-02	12.84	20.54	5.32E-01	13.79	34.92	6.93E-01	16.18	12.44	1.94E-01	61.66	21.58	4.26E-03
cg15646795	19.46	8.68	2.50E-02	37.75	31.82	2.36E-01	-48.64	130.76	7.10E-01	20.23	9.35	3.04E-02	-9.84	35.79	7.83E-01
cg23905542	31.79	14.18	2.50E-02	48.38	35.10	1.68E-01	-10.83	144.50	9.40E-01	33.78	16.34	3.87E-02	-19.26	51.97	7.11E-01
cg06818710	37.02	16.51	2.50E-02	34.84	25.09	1.65E-01	-32.08	79.89	6.88E-01	61.94	30.01	3.90E-02	20.53	35.09	5.59E-01
cg05635169	-10.40	4.64	2.50E-02	-9.94	6.84	1.46E-01	-2.88	10.65	7.86E-01	-9.96	10.80	3.56E-01	-20.81	11.40	6.80E-02
cg01344914	21.53	9.60	2.50E-02	27.28	18.14	1.32E-01	33.61	18.37	6.74E-02	-6.19	20.21	7.59E-01	27.61	20.43	1.76E-01
cg15988010	28.75	12.82	2.50E-02	-12.88	39.80	7.46E-01	132.67	159.45	4.05E-01	37.01	14.46	1.05E-02	1.17	39.91	9.77E-01
cg09919756	-19.61	8.75	2.50E-02	-11.98	18.79	5.24E-01	-36.49	49.24	4.59E-01	-15.23	10.78	1.58E-01	-62.17	28.53	2.93E-02
cg07889938	12.74	5.68	2.50E-02	20.81	7.61	6.27E-03	-4.33	21.82	8.43E-01	9.45	25.08	7.06E-01	2.95	9.99	7.68E-01
cg21533331	-50.30	22.44	2.50E-02	-56.81	28.75	4.82E-02	-63.65	93.54	4.96E-01	39.30	65.46	5.48E-01	-77.09	48.25	1.10E-01
cg25074170	27.83	12.41	2.50E-02	25.63	17.77	1.49E-01	11.08	31.22	7.23E-01	-2.04	39.92	9.59E-01	53.46	24.46	2.88E-02
cg17496990	-12.71	5.67	2.50E-02	-24.58	8.19	2.69E-03	6.02	18.13	7.40E-01	-6.93	4.83	1.51E-01	-18.15	12.07	1.33E-01
cg04747634	13.08	5.84	2.50E-02	12.03	7.45	1.06E-01	13.21	17.10	4.40E-01	33.12	19.36	8.72E-02	6.45	13.77	6.40E-01
cg04989070	-21.89	9.77	2.50E-02	5.23	17.93	7.71E-01	-51.48	30.63	9.28E-02	-28.51	10.86	8.63E-03	-24.75	23.29	2.88E-01
cg10538151	-25.23	11.26	2.50E-02	-20.63	16.80	2.20E-01	-38.14	31.10	2.20E-01	-18.00	29.93	5.48E-01	-30.25	21.32	1.56E-01
cg13614286	20.35	9.08	2.50E-02	34.20	23.47	1.45E-01	21.35	38.77	5.82E-01	15.37	11.01	1.63E-01	31.28	26.73	2.42E-01
cg19402236	12.39	5.53	2.50E-02	13.72	7.98	8.57E-02	9.66	13.27	4.67E-01	23.41	19.73	2.35E-01	8.56	10.67	4.22E-01
cg09715738	33.23	14.83	2.50E-02	66.54	48.72	1.72E-01	72.41	165.74	6.62E-01	25.02	16.34	1.26E-01	77.32	53.73	1.50E-01
cg17838697	10.91	4.87	2.50E-02	5.02	9.48	5.97E-01	9.74	16.66	5.59E-01	12.65	6.86	6.52E-02	16.13	12.64	2.02E-01
cg22067839	19.47	8.69	2.50E-02	30.42	15.65	5.20E-02	13.56	16.50	4.11E-01	5.48	17.47	7.54E-01	29.80	21.24	1.61E-01
cg24013378	57.66	25.73	2.50E-02	68.64	32.87	3.68E-02	181.76	125.32	1.47E-01	13.43	66.14	8.39E-01	30.49	58.47	6.02E-01

cg01278564	18.74	8.36	2.50E-02	17.90	15.45	2.47E-01	12.77	14.21	3.69E-01	16.04	17.20	3.51E-01	42.61	23.75	7.28E-02
cg09287629	7.99	3.56	2.50E-02	11.80	5.35	2.74E-02	6.12	8.51	4.72E-01	11.85	7.87	1.32E-01	-4.28	8.51	6.15E-01
cg26606265	-26.58	11.86	2.50E-02	-32.31	38.76	4.05E-01	0.90	120.39	9.94E-01	-26.78	12.99	3.93E-02	-19.64	47.14	6.77E-01
cg23287404	61.13	27.28	2.50E-02	51.95	31.71	1.01E-01	162.67	64.73	1.20E-02	-5.65	57.63	9.22E-01	65.09	50.54	1.98E-01
cg27438152	-4.43	1.98	2.50E-02	-5.07	2.67	5.78E-02	-2.13	4.07	6.00E-01	-6.02	7.62	4.30E-01	-4.99	5.11	3.28E-01
cg18118660	20.65	9.22	2.50E-02	12.42	17.84	4.86E-01	25.11	22.40	2.62E-01	23.55	13.40	7.89E-02	21.45	30.52	4.82E-01
cg05206633	4.89	2.18	2.50E-02	5.49	2.94	6.18E-02	6.33	5.58	2.57E-01	4.21	5.14	4.13E-01	1.19	6.43	8.53E-01
cg10236857	11.38	5.08	2.50E-02	10.68	6.56	1.03E-01	26.94	12.70	3.38E-02	8.56	16.86	6.11E-01	-0.74	13.11	9.55E-01
cg05584759	18.81	8.39	2.50E-02	18.46	16.06	2.50E-01	9.72	16.52	5.56E-01	23.70	15.42	1.24E-01	24.53	20.20	2.24E-01
cg07007807	-32.55	14.52	2.50E-02	-57.91	40.37	1.51E-01	-49.13	161.33	7.61E-01	-28.71	16.18	7.61E-02	-26.85	60.78	6.59E-01
cg07524456	-7.71	3.44	2.50E-02	-10.05	4.70	3.24E-02	-1.35	7.62	8.59E-01	-12.93	12.77	3.11E-01	-5.91	7.96	4.58E-01
cg09404642	-38.28	17.08	2.50E-02	-48.67	22.40	2.98E-02	-85.96	69.28	2.15E-01	34.22	52.25	5.12E-01	-33.53	34.13	3.26E-01
cg08083335	29.22	13.04	2.50E-02	17.05	26.12	5.14E-01	10.49	82.72	8.99E-01	29.57	16.56	7.43E-02	60.05	39.98	1.33E-01
cg03214420	-10.48	4.68	2.51E-02	-17.17	9.41	6.79E-02	-15.23	13.71	2.66E-01	-5.87	6.81	3.89E-01	-10.29	11.53	3.72E-01
cg02582355	-6.29	2.81	2.51E-02	-8.61	4.10	3.57E-02	-0.71	5.40	8.95E-01	-8.64	9.93	3.84E-01	-7.57	6.61	2.52E-01
cg24836242	-11.84	5.28	2.51E-02	-20.29	8.25	1.39E-02	-7.48	12.87	5.61E-01	-9.25	12.68	4.66E-01	-2.62	10.62	8.05E-01
cg01297349	14.63	6.53	2.51E-02	3.86	12.87	7.64E-01	6.64	22.35	7.66E-01	16.55	8.80	6.02E-02	37.10	19.98	6.33E-02
cg02656609	-11.57	5.16	2.51E-02	-19.52	9.55	4.11E-02	-0.36	12.97	9.78E-01	-12.21	7.79	1.17E-01	-4.06	15.54	7.94E-01
cg16928046	-10.09	4.50	2.51E-02	-9.92	6.75	1.41E-01	-21.78	10.94	4.65E-02	0.54	10.51	9.59E-01	-10.30	10.02	3.04E-01
cg05777316	15.55	6.94	2.51E-02	23.18	12.59	6.56E-02	18.35	12.92	1.56E-01	4.55	15.95	7.75E-01	10.78	14.88	4.69E-01
cg01530154	-9.38	4.19	2.51E-02	-7.92	6.20	2.02E-01	-12.61	10.44	2.27E-01	-8.18	10.78	4.48E-01	-10.81	8.70	2.14E-01
cg14302083	7.50	3.35	2.51E-02	13.20	6.86	5.44E-02	5.07	9.50	5.94E-01	5.27	4.49	2.41E-01	9.71	11.63	4.04E-01
cg00550703	-18.65	8.33	2.51E-02	-9.48	24.03	6.93E-01	0.55	36.67	9.88E-01	-22.13	9.56	2.07E-02	-10.91	31.47	7.29E-01
cg18575863	-32.94	14.70	2.51E-02	-29.43	19.89	1.39E-01	32.95	72.55	6.50E-01	-33.54	33.94	3.23E-01	-53.02	31.02	8.74E-02
cg13654836	11.00	4.91	2.51E-02	13.12	7.19	6.81E-02	14.05	10.47	1.80E-01	14.69	11.86	2.15E-01	-4.99	12.96	7.00E-01
cg26956263	22.83	10.19	2.51E-02	43.28	33.60	1.98E-01	-19.52	131.25	8.82E-01	25.94	11.24	2.10E-02	-29.99	36.21	4.08E-01
cg14046757	40.17	17.93	2.51E-02	114.55	56.08	4.11E-02	-160.57	246.15	5.14E-01	32.91	19.79	9.62E-02	32.03	67.44	6.35E-01
cg22775123	-9.56	4.27	2.51E-02	-11.44	6.04	5.82E-02	-17.59	12.00	1.43E-01	4.97	11.84	6.75E-01	-9.28	8.63	2.82E-01
cg03212952	10.70	4.78	2.51E-02	5.75	11.09	6.04E-01	24.93	39.71	5.30E-01	9.87	5.60	7.81E-02	28.90	17.75	1.03E-01
cg07675656	16.75	7.48	2.51E-02	14.19	11.77	2.28E-01	15.79	15.40	3.05E-01	30.23	15.37	4.92E-02	1.16	21.24	9.56E-01
cg18404811	8.59	3.84	2.51E-02	9.32	5.13	6.93E-02	20.44	11.20	6.81E-02	10.91	11.99	3.63E-01	-0.60	8.16	9.41E-01
cg19117047	-8.37	3.74	2.51E-02	-11.40	5.41	3.50E-02	-14.10	8.75	1.07E-01	4.13	7.84	5.98E-01	-10.23	7.44	1.69E-01
cg06567836	12.94	5.78	2.51E-02	2.14	17.18	9.01E-01	1.90	39.34	9.62E-01	15.41	6.49	1.76E-02	6.16	21.35	7.73E-01
cg14091208	9.64	4.30	2.51E-02	7.39	6.63	2.65E-01	15.42	8.80	7.96E-02	17.27	10.74	1.08E-01	0.35	10.17	9.72E-01
cg27150870	-11.56	5.16	2.51E-02	-11.98	8.05	1.37E-01	-9.31	13.46	4.89E-01	2.10	12.68	8.68E-01	-20.33	9.82	3.84E-02

cg17468459	26.31	11.75	2.51E-02	21.25	20.28	2.95E-01	-11.11	50.40	8.26E-01	24.47	17.89	1.71E-01	51.58	27.76	6.32E-02
cg12010413	14.88	6.65	2.51E-02	11.91	15.89	4.53E-01	34.94	19.33	7.07E-02	14.51	8.38	8.36E-02	-5.65	23.69	8.12E-01
cg03255749	21.03	9.39	2.51E-02	9.86	13.00	4.48E-01	33.35	40.26	4.07E-01	6.15	31.20	8.44E-01	40.59	16.28	1.26E-02
cg23808704	9.12	4.07	2.51E-02	9.34	7.47	2.12E-01	17.21	11.53	1.36E-01	4.55	6.23	4.66E-01	14.92	10.46	1.54E-01
cg16426870	-40.75	18.20	2.51E-02	4.09	42.08	9.23E-01	-123.82	177.22	4.85E-01	-44.04	22.45	4.98E-02	-77.50	47.69	1.04E-01
cg16728934	-14.24	6.36	2.51E-02	-22.68	8.09	5.04E-03	-22.43	14.03	1.10E-01	7.07	14.25	6.20E-01	-9.59	11.04	3.85E-01
cg22155376	17.31	7.73	2.51E-02	19.06	10.66	7.38E-02	24.63	16.77	1.42E-01	-7.97	35.61	8.23E-01	11.33	16.69	4.97E-01
cg27148529	9.35	4.17	2.51E-02	11.08	5.52	4.48E-02	8.11	8.75	3.54E-01	13.89	18.29	4.48E-01	3.00	10.80	7.81E-01
cg23983887	-6.15	2.75	2.51E-02	-5.72	4.01	1.54E-01	-11.45	5.39	3.37E-02	-1.10	8.00	8.90E-01	-2.37	7.04	7.36E-01
cg18482164	20.33	9.08	2.51E-02	36.59	22.05	9.71E-02	32.17	46.89	4.93E-01	16.41	11.39	1.50E-01	15.85	22.91	4.89E-01
cg21963318	14.64	6.54	2.52E-02	11.63	8.58	1.75E-01	13.88	16.35	3.96E-01	17.01	37.93	6.54E-01	22.45	13.64	9.98E-02
cg05933789	-7.94	3.54	2.52E-02	-6.92	7.06	3.27E-01	-1.95	14.87	8.96E-01	-7.66	4.97	1.23E-01	-11.99	8.31	1.49E-01
cg18538668	-10.27	4.59	2.52E-02	-12.80	6.75	5.80E-02	-8.78	10.17	3.88E-01	-7.84	15.11	6.04E-01	-7.63	9.29	4.12E-01
cg15108537	-8.26	3.69	2.52E-02	-12.07	6.85	7.82E-02	-3.68	12.74	7.73E-01	-10.15	5.37	5.88E-02	2.15	9.37	8.18E-01
cg08577727	12.53	5.60	2.52E-02	10.12	8.40	2.28E-01	25.88	11.30	2.21E-02	7.43	15.38	6.29E-01	3.98	13.23	7.64E-01
cg08545416	22.27	9.95	2.52E-02	15.56	17.34	3.70E-01	22.00	18.29	2.29E-01	4.31	17.57	8.06E-01	52.87	19.77	7.50E-03
cg16895464	-27.15	12.13	2.52E-02	-41.51	41.78	3.21E-01	-197.40	128.37	1.24E-01	-25.82	13.14	4.93E-02	2.03	51.79	9.69E-01
cg02362970	17.56	7.84	2.52E-02	-10.56	36.83	7.74E-01	179.26	170.69	2.94E-01	19.87	8.36	1.74E-02	2.13	29.35	9.42E-01
cg25630380	-13.61	6.08	2.52E-02	-16.87	9.04	6.19E-02	-10.59	13.61	4.36E-01	-5.35	16.63	7.47E-01	-14.68	13.14	2.64E-01
cg17851392	17.48	7.81	2.52E-02	33.02	21.10	1.18E-01	5.38	50.92	9.16E-01	14.09	8.92	1.14E-01	27.77	28.82	3.35E-01
cg15711028	17.94	8.02	2.52E-02	36.63	17.67	3.82E-02	16.42	39.35	6.76E-01	10.54	9.79	2.81E-01	32.32	27.97	2.48E-01
cg08545136	41.92	18.73	2.52E-02	51.49	24.19	3.33E-02	8.96	121.92	9.41E-01	55.91	38.93	1.51E-01	-14.30	49.05	7.71E-01
cg10855540	-14.86	6.64	2.52E-02	-20.16	13.75	1.43E-01	-7.08	16.63	6.70E-01	-11.29	9.80	2.49E-01	-25.92	17.21	1.32E-01
cg05655116	-34.24	15.29	2.52E-02	-33.36	18.79	7.59E-02	-70.22	46.59	1.32E-01	-0.08	62.16	9.99E-01	-26.98	37.14	4.68E-01
cg23234726	-7.87	3.51	2.52E-02	-4.98	6.19	4.21E-01	-12.98	10.12	2.00E-01	-6.65	5.46	2.23E-01	-13.67	9.33	1.43E-01
cg14203721	-11.52	5.15	2.52E-02	-21.66	12.59	8.55E-02	-25.13	29.04	3.87E-01	-5.99	6.33	3.44E-01	-22.61	13.80	1.01E-01
cg04521981	10.76	4.81	2.52E-02	6.92	11.26	5.39E-01	1.83	14.13	8.97E-01	15.49	6.16	1.19E-02	-1.60	15.78	9.19E-01
cg22796704	10.16	4.54	2.52E-02	6.70	6.36	2.93E-01	14.01	9.66	1.47E-01	0.69	15.56	9.64E-01	19.45	10.55	6.53E-02
cg26335760	11.19	5.00	2.52E-02	7.05	7.41	3.41E-01	10.95	11.44	3.38E-01	20.44	11.73	8.15E-02	12.61	12.02	2.94E-01
cg20084219	-4.88	2.18	2.52E-02	-1.06	2.45	6.63E-01	-5.34	3.97	1.79E-01	-9.62	5.44	7.71E-02	-9.08	4.20	3.04E-02
cg22321572	8.34	3.72	2.52E-02	8.62	5.77	1.35E-01	9.19	8.11	2.57E-01	6.98	9.99	4.85E-01	7.86	7.71	3.08E-01
cg07980405	-41.30	18.45	2.52E-02	-32.98	23.08	1.53E-01	-44.91	98.12	6.47E-01	-68.13	62.91	2.79E-01	-53.32	37.68	1.57E-01
cg00686926	8.44	3.77	2.52E-02	9.99	5.29	5.91E-02	9.08	7.89	2.50E-01	9.53	10.65	3.71E-01	0.71	10.15	9.44E-01
cg23732629	-4.84	2.16	2.52E-02	-6.77	3.80	7.53E-02	-3.73	5.15	4.68E-01	-4.73	3.50	1.76E-01	-1.58	6.28	8.01E-01
cg27217253	-7.46	3.33	2.52E-02	-6.85	4.75	1.49E-01	-16.58	8.01	3.83E-02	5.84	11.14	6.00E-01	-7.09	6.74	2.93E-01

cg14921757	26.57	11.87	2.52E-02	24.93	15.47	1.07E-01	82.23	53.87	1.27E-01	8.54	28.37	7.63E-01	34.19	27.45	2.13E-01
cg02691091	23.16	10.35	2.52E-02	15.94	23.79	5.03E-01	47.97	33.60	1.53E-01	13.92	10.38	1.80E-01	65.30	29.66	2.77E-02
cg03839074	7.32	3.27	2.52E-02	8.67	4.64	6.17E-02	3.67	6.61	5.79E-01	8.71	10.31	3.98E-01	7.85	8.24	3.41E-01
cg25364469	12.57	5.62	2.52E-02	9.37	13.92	5.01E-01	23.68	18.86	2.09E-01	13.33	6.99	5.66E-02	3.34	17.43	8.48E-01
cg00110545	-7.54	3.37	2.52E-02	-7.22	5.12	1.58E-01	-11.37	6.18	6.59E-02	-4.96	9.17	5.89E-01	-2.72	9.15	7.66E-01
cg23752007	9.82	4.39	2.52E-02	13.67	6.39	3.25E-02	14.18	9.05	1.17E-01	-2.07	11.61	8.58E-01	2.26	11.27	8.41E-01
cg01136942	16.95	7.57	2.52E-02	14.32	14.32	3.17E-01	25.39	16.83	1.31E-01	22.61	11.87	5.68E-02	-12.63	22.75	5.79E-01
cg06344576	24.66	11.02	2.52E-02	25.27	15.85	1.11E-01	45.79	22.85	4.51E-02	16.78	31.52	5.95E-01	-1.56	27.39	9.55E-01
cg14676666	-9.66	4.31	2.52E-02	-15.08	8.00	5.95E-02	-13.97	11.59	2.28E-01	-7.40	6.63	2.65E-01	-1.39	11.25	9.02E-01
cg03343453	14.95	6.68	2.52E-02	14.73	18.59	4.28E-01	13.86	25.59	5.88E-01	11.50	7.84	1.42E-01	48.84	24.11	4.28E-02
cg08332594	25.15	11.24	2.52E-02	34.03	16.93	4.45E-02	39.53	45.34	3.83E-01	24.53	43.07	5.69E-01	14.10	17.14	4.11E-01
cg18245365	58.70	26.24	2.52E-02	33.21	59.73	5.78E-01	-252.79	185.91	1.74E-01	70.30	26.11	7.10E-03	81.03	72.42	2.63E-01
cg24662243	-19.69	8.80	2.53E-02	-13.86	14.13	3.27E-01	-24.84	49.09	6.13E-01	-31.57	13.90	2.32E-02	-4.83	20.78	8.16E-01
cg02151754	16.84	7.52	2.53E-02	28.65	25.33	2.58E-01	46.55	108.88	6.69E-01	17.16	8.22	3.69E-02	-4.18	28.58	8.84E-01
cg06969265	23.92	10.69	2.53E-02	55.28	27.53	4.46E-02	20.22	129.30	8.76E-01	22.07	12.23	7.10E-02	-18.24	38.25	6.33E-01
cg25716149	-10.63	4.75	2.53E-02	-21.10	8.43	1.23E-02	-12.47	12.74	3.28E-01	-2.32	5.88	6.94E-01	-14.50	10.27	1.58E-01
cg25778446	-11.11	4.97	2.53E-02	-12.51	9.51	1.89E-01	-1.14	10.95	9.17E-01	-12.79	8.76	1.44E-01	-16.72	11.08	1.31E-01
cg03874978	-14.44	6.45	2.53E-02	-19.57	10.99	7.50E-02	-6.57	12.37	5.95E-01	-8.45	17.33	6.26E-01	-19.37	13.06	1.38E-01
cg01613817	23.62	10.56	2.53E-02	16.95	13.84	2.21E-01	22.09	27.36	4.19E-01	17.11	34.09	6.16E-01	50.80	25.31	4.47E-02
cg07700680	-18.16	8.12	2.53E-02	-9.58	13.31	4.72E-01	-18.24	38.39	6.35E-01	-27.88	11.53	1.56E-02	0.40	27.41	9.88E-01
cg05744487	10.86	4.85	2.53E-02	9.19	8.04	2.53E-01	-2.00	12.37	8.72E-01	14.25	9.10	1.17E-01	19.12	10.95	8.09E-02
cg13788090	44.73	20.00	2.53E-02	83.86	55.90	1.34E-01	-118.64	224.24	5.97E-01	30.99	22.74	1.73E-01	120.70	66.27	6.85E-02
cg17642201	-24.32	10.87	2.53E-02	-41.20	21.89	5.98E-02	3.79	27.93	8.92E-01	-27.58	15.78	8.04E-02	-12.85	30.50	6.73E-01
cg23173307	19.71	8.81	2.53E-02	13.80	25.44	5.88E-01	2.89	30.56	9.25E-01	22.05	10.44	3.46E-02	24.95	30.43	4.12E-01
cg03673624	-58.42	26.12	2.53E-02	-77.47	35.00	2.69E-02	155.83	136.33	2.53E-01	-71.53	61.62	2.46E-01	-35.95	54.85	5.12E-01
cg07198170	13.71	6.13	2.53E-02	10.76	15.26	4.81E-01	18.50	15.66	2.38E-01	10.80	7.81	1.66E-01	35.75	23.25	1.24E-01
cg07359306	9.81	4.39	2.53E-02	14.63	6.39	2.20E-02	10.80	9.61	2.61E-01	5.12	10.41	6.22E-01	-1.69	11.59	8.84E-01
cg05721773	15.11	6.75	2.53E-02	10.43	8.98	2.46E-01	29.20	16.37	7.44E-02	14.64	20.57	4.77E-01	17.05	17.10	3.19E-01
cg19535394	-43.53	19.46	2.53E-02	-34.44	27.35	2.08E-01	-102.00	53.10	5.47E-02	-52.58	46.65	2.60E-01	-17.49	45.20	6.99E-01
cg07493097	-11.81	5.28	2.53E-02	-23.87	8.01	2.88E-03	-20.73	14.88	1.63E-01	-4.35	4.50	3.34E-01	-7.96	9.07	3.80E-01
cg01293143	13.34	5.97	2.53E-02	13.68	8.79	1.19E-01	17.60	11.95	1.41E-01	19.56	21.47	3.62E-01	5.38	12.92	6.77E-01
cg03978658	8.97	4.01	2.53E-02	9.23	5.04	6.72E-02	13.11	10.65	2.18E-01	3.42	12.29	7.81E-01	7.65	11.67	5.12E-01
cg10479431	7.24	3.24	2.53E-02	8.19	4.05	4.34E-02	10.60	8.24	1.98E-01	-2.15	10.09	8.31E-01	5.76	10.04	5.66E-01
cg17434366	-22.68	10.14	2.53E-02	-26.73	12.09	2.70E-02	39.66	37.94	2.96E-01	-19.86	41.65	6.33E-01	-33.38	24.90	1.80E-01
cg06650206	-13.51	6.04	2.53E-02	-10.44	11.23	3.52E-01	11.07	27.39	6.86E-01	-16.05	8.18	4.99E-02	-19.52	17.66	2.69E-01

cg10597322	11.76	5.26	2.53E-02	11.77	11.03	2.86E-01	17.80	14.12	2.07E-01	14.43	7.18	4.44E-02	-11.66	16.88	4.90E-01
cg22126171	-9.80	4.38	2.53E-02	-9.00	10.14	3.74E-01	-12.98	15.78	4.11E-01	-9.06	5.72	1.13E-01	-12.07	11.38	2.89E-01
cg14794043	12.85	5.75	2.53E-02	11.47	9.94	2.48E-01	5.93	12.08	6.23E-01	28.94	14.41	4.46E-02	10.95	10.86	3.13E-01
cg10013969	-9.47	4.23	2.53E-02	-5.44	6.71	4.17E-01	3.18	10.51	7.62E-01	-12.63	6.50	5.19E-02	-20.53	9.17	2.52E-02
cg08064269	12.12	5.42	2.53E-02	18.83	12.83	1.42E-01	13.88	24.67	5.74E-01	8.99	6.53	1.69E-01	22.38	18.58	2.28E-01
cg23605991	11.57	5.18	2.53E-02	4.33	7.40	5.58E-01	16.04	13.07	2.20E-01	27.88	14.16	4.90E-02	14.58	11.02	1.86E-01
cg08817937	-32.48	14.52	2.54E-02	-87.30	69.71	2.10E-01	-341.55	212.90	1.09E-01	-29.32	15.47	5.80E-02	-17.67	54.89	7.48E-01
cg14324255	-22.16	9.91	2.54E-02	-13.81	15.27	3.66E-01	0.65	36.53	9.86E-01	7.52	58.53	8.98E-01	-34.87	14.36	1.52E-02
cg23855505	-33.64	15.05	2.54E-02	-61.79	33.22	6.29E-02	30.47	165.62	8.54E-01	-19.00	17.43	2.76E-01	-96.93	52.38	6.42E-02
cg07185131	28.12	12.58	2.54E-02	70.31	23.31	2.56E-03	8.47	37.67	8.22E-01	17.22	11.74	1.43E-01	19.83	21.07	3.47E-01
cg11179513	-37.47	16.76	2.54E-02	-41.75	35.65	2.42E-01	172.07	115.35	1.36E-01	-41.02	13.99	3.37E-03	-49.76	55.80	3.73E-01
cg11950292	-12.97	5.80	2.54E-02	-2.82	18.23	8.77E-01	-19.53	43.17	6.51E-01	-12.62	6.44	5.00E-02	-30.00	21.96	1.72E-01
cg14236430	-13.86	6.20	2.54E-02	-22.95	16.01	1.52E-01	-8.84	20.45	6.66E-01	-11.87	7.66	1.21E-01	-17.83	19.44	3.59E-01
cg09512891	-3.47	1.55	2.54E-02	-4.05	2.21	6.65E-02	1.89	3.70	6.09E-01	-5.23	4.38	2.33E-01	-5.69	3.47	1.01E-01
cg10415314	-9.68	4.33	2.54E-02	-15.60	8.86	7.83E-02	-5.01	13.18	7.04E-01	-7.25	6.14	2.37E-01	-11.58	10.98	2.92E-01
cg08250738	-8.48	3.79	2.54E-02	-24.30	10.18	1.70E-02	-6.09	20.61	7.68E-01	-5.81	4.52	1.99E-01	-6.55	10.78	5.43E-01
cg05262382	7.67	3.43	2.54E-02	6.35	4.52	1.60E-01	-2.40	8.98	7.89E-01	7.11	4.78	1.37E-01	20.66	8.12	1.10E-02
cg02696201	-11.51	5.15	2.54E-02	-11.02	12.10	3.62E-01	-21.00	22.81	3.57E-01	-7.92	6.33	2.11E-01	-30.13	15.79	5.63E-02
cg19260329	9.15	4.09	2.54E-02	2.41	6.67	7.17E-01	14.71	6.38	2.11E-02	21.17	13.70	1.22E-01	2.37	11.72	8.40E-01
cg06118122	-14.48	6.48	2.54E-02	-15.86	15.61	3.10E-01	-1.84	21.32	9.31E-01	-13.47	8.31	1.05E-01	-26.63	18.18	1.43E-01
cg06884254	12.34	5.52	2.54E-02	12.18	8.74	1.63E-01	14.82	11.39	1.93E-01	6.05	11.60	6.02E-01	18.82	14.79	2.03E-01
cg12381416	6.06	2.71	2.54E-02	6.80	4.47	1.28E-01	13.52	7.15	5.87E-02	2.18	4.64	6.38E-01	5.91	7.09	4.05E-01
ch.3.1432250F	-26.89	12.03	2.54E-02	-16.22	16.24	3.18E-01	-3.38	39.27	9.32E-01	-95.33	66.36	1.51E-01	-44.81	21.13	3.39E-02
cg03599224	-14.07	6.30	2.54E-02	-21.24	10.54	4.38E-02	-6.39	13.29	6.31E-01	-20.35	15.67	1.94E-01	-6.87	12.43	5.80E-01
cg15164958	25.60	11.45	2.54E-02	14.27	13.88	3.04E-01	4.00	52.00	9.39E-01	69.12	25.62	6.99E-03	21.20	16.15	1.89E-01
cg00293660	8.21	3.67	2.54E-02	8.07	6.49	2.14E-01	3.57	10.41	7.31E-01	10.30	5.81	7.65E-02	6.84	9.29	4.62E-01
cg14209920	12.22	5.47	2.54E-02	14.16	8.64	1.01E-01	6.18	11.58	5.94E-01	23.24	11.04	3.52E-02	-4.02	15.08	7.90E-01
cg03682823	18.47	8.27	2.55E-02	16.43	13.12	2.10E-01	9.78	16.30	5.48E-01	50.44	23.55	3.22E-02	14.46	17.54	4.10E-01
cg23023844	4.14	1.85	2.55E-02	4.74	2.56	6.37E-02	-0.63	3.88	8.71E-01	7.36	7.00	2.94E-01	7.27	4.42	9.99E-02
cg25499746	-19.57	8.76	2.55E-02	-10.39	16.74	5.35E-01	-38.48	36.88	2.97E-01	-21.70	12.09	7.26E-02	-21.81	22.98	3.43E-01
cg07378309	-9.78	4.38	2.55E-02	-9.30	6.74	1.68E-01	0.07	9.42	9.94E-01	-13.18	11.53	2.53E-01	-18.19	9.36	5.20E-02
cg21932513	13.19	5.90	2.55E-02	20.14	9.93	4.25E-02	14.00	13.14	2.87E-01	14.82	10.89	1.73E-01	-7.44	15.24	6.26E-01
cg21878650	7.72	3.46	2.55E-02	6.42	4.53	1.56E-01	6.02	7.66	4.31E-01	7.42	15.59	6.34E-01	14.46	8.49	8.86E-02
cg05988698	-13.57	6.07	2.55E-02	-7.20	15.62	6.45E-01	-0.71	28.56	9.80E-01	-15.63	7.08	2.73E-02	-13.96	23.15	5.47E-01
cg00401091	26.01	11.64	2.55E-02	-10.91	35.46	7.58E-01	5.86	190.73	9.76E-01	30.89	12.83	1.61E-02	26.54	45.39	5.59E-01

cg10318528	-10.06	4.50	2.55E-02	-16.49	7.66	3.13E-02	-4.26	9.82	6.64E-01	-5.40	9.66	5.76E-01	-10.09	9.46	2.86E-01
cg15396800	-11.62	5.20	2.55E-02	-17.34	8.53	4.19E-02	7.03	13.19	5.94E-01	-10.88	9.00	2.27E-01	-19.07	14.03	1.74E-01
cg19738283	15.25	6.83	2.55E-02	34.25	22.63	1.30E-01	88.99	72.66	2.21E-01	12.68	7.50	9.08E-02	11.75	25.63	6.47E-01
cg09006498	37.06	16.59	2.55E-02	39.98	35.71	2.63E-01	-10.05	126.50	9.37E-01	34.56	19.97	8.36E-02	61.60	59.60	3.01E-01
cg14377594	11.31	5.06	2.55E-02	22.52	9.34	1.59E-02	7.62	10.38	4.63E-01	11.78	10.26	2.51E-01	0.05	10.69	9.97E-01
cg25324261	-27.29	12.22	2.55E-02	-24.78	15.29	1.05E-01	-3.69	122.69	9.76E-01	32.42	66.29	6.25E-01	-39.46	21.67	6.86E-02
cg06207728	19.54	8.74	2.55E-02	13.93	16.67	4.03E-01	17.05	15.66	2.76E-01	14.25	18.12	4.31E-01	39.25	20.62	5.69E-02
cg22437276	-6.20	2.77	2.55E-02	-3.63	3.92	3.55E-01	-0.89	4.02	8.26E-01	-15.34	6.65	2.11E-02	-9.48	3.87	1.44E-02
cg19262563	-9.10	4.07	2.55E-02	-6.01	7.13	3.99E-01	1.20	12.98	9.26E-01	-9.29	6.50	1.53E-01	-19.79	9.55	3.82E-02
cg00928226	23.87	10.69	2.55E-02	48.50	20.97	2.07E-02	-2.99	39.64	9.40E-01	17.41	14.43	2.28E-01	16.32	30.94	5.98E-01
cg06873916	11.86	5.31	2.55E-02	20.51	16.37	2.10E-01	1.08	23.75	9.64E-01	7.96	6.87	2.47E-01	19.84	10.70	6.37E-02
cg15211864	19.71	8.83	2.55E-02	26.49	19.81	1.81E-01	13.14	33.77	6.97E-01	16.07	10.96	1.42E-01	37.20	30.42	2.21E-01
cg02914501	-10.17	4.56	2.55E-02	-13.72	5.78	1.75E-02	0.20	13.43	9.88E-01	13.64	28.28	6.30E-01	-8.50	9.36	3.64E-01
cg15701412	-11.27	5.05	2.55E-02	-14.18	12.19	2.45E-01	-34.24	30.99	2.69E-01	-12.36	6.01	3.96E-02	8.23	16.27	6.13E-01
cg10031042	-18.74	8.39	2.55E-02	-12.89	15.22	3.97E-01	-18.93	30.15	5.30E-01	-14.67	12.02	2.22E-01	-47.13	23.11	4.14E-02
cg15561305	-8.04	3.60	2.55E-02	-8.62	5.53	1.19E-01	-15.46	8.36	6.44E-02	-9.60	9.13	2.93E-01	-0.15	7.41	9.84E-01
cg13644262	9.91	4.44	2.55E-02	12.26	6.52	6.00E-02	16.15	9.58	9.17E-02	-6.47	13.35	6.28E-01	6.98	9.63	4.69E-01
cg06630494	29.34	13.14	2.55E-02	14.73	16.46	3.71E-01	36.40	46.68	4.36E-01	70.10	40.01	7.97E-02	54.06	31.30	8.41E-02
cg26511972	-5.95	2.67	2.55E-02	-4.79	4.24	2.59E-01	-3.23	4.30	4.52E-01	-18.74	10.08	6.30E-02	-10.03	6.88	1.45E-01
cg01719100	16.97	7.60	2.55E-02	25.90	15.13	8.69E-02	13.60	16.73	4.16E-01	8.03	11.33	4.79E-01	44.10	25.18	7.99E-02
cg10659138	-9.73	4.36	2.55E-02	-7.32	8.78	4.05E-01	14.34	15.47	3.54E-01	-13.57	6.10	2.62E-02	-13.04	10.73	2.24E-01
cg24507266	7.51	3.36	2.55E-02	9.80	4.77	3.98E-02	8.97	7.32	2.21E-01	16.75	11.95	1.61E-01	-2.72	7.28	7.09E-01
cg03382549	-10.87	4.87	2.55E-02	-16.50	9.97	9.80E-02	-12.93	12.45	2.99E-01	-9.66	7.10	1.73E-01	-3.03	13.04	8.16E-01
cg18947570	-33.54	15.02	2.55E-02	-41.85	20.37	4.00E-02	-5.63	42.16	8.94E-01	19.76	48.60	6.84E-01	-51.10	31.02	9.96E-02
cg16604066	27.93	12.51	2.55E-02	48.67	22.32	2.92E-02	22.41	22.86	3.27E-01	6.52	16.29	6.89E-01	64.35	33.42	5.41E-02
cg25318211	-10.85	4.86	2.55E-02	-4.49	13.89	7.47E-01	-2.09	19.62	9.15E-01	-12.08	5.68	3.33E-02	-15.85	16.86	3.47E-01
cg00763594	-10.08	4.51	2.55E-02	-7.72	10.26	4.52E-01	-4.79	18.45	7.95E-01	-9.78	5.64	8.30E-02	-19.08	13.81	1.67E-01
cg27281690	9.97	4.47	2.55E-02	12.99	6.08	3.27E-02	19.63	11.08	7.66E-02	2.75	11.47	8.10E-01	-4.37	11.68	7.08E-01
cg07224291	-15.81	7.08	2.55E-02	-28.89	9.18	1.65E-03	-29.00	12.17	1.72E-02	-4.93	4.79	3.03E-01	-6.92	11.04	5.30E-01
cg14227486	14.94	6.69	2.55E-02	15.88	9.69	1.01E-01	15.11	17.10	3.77E-01	28.02	18.98	1.40E-01	6.39	13.51	6.36E-01
cg23093404	-18.23	8.16	2.55E-02	-37.95	16.56	2.20E-02	9.94	19.18	6.04E-01	-18.79	9.04	3.77E-02	-19.46	22.57	3.89E-01
cg07784054	17.64	7.90	2.56E-02	19.47	13.92	1.62E-01	-4.02	44.10	9.27E-01	13.55	10.66	2.03E-01	41.90	25.39	9.89E-02
cg01295022	33.74	15.11	2.56E-02	54.33	18.65	3.58E-03	31.42	36.94	3.95E-01	50.29	44.27	2.56E-01	-3.91	25.33	8.77E-01
cg07807497	-10.18	4.56	2.56E-02	-14.46	10.01	1.49E-01	-7.58	18.35	6.80E-01	-7.57	5.82	1.94E-01	-17.49	13.22	1.86E-01
cg11386098	-11.80	5.29	2.56E-02	-14.08	6.82	3.88E-02	-1.77	9.54	8.53E-01	-0.27	13.09	9.83E-01	-25.04	9.56	8.80E-03

cg19739774	-7.11	3.18	2.56E-02	-11.71	6.41	6.77E-02	-16.71	11.87	1.59E-01	-5.81	4.34	1.80E-01	0.77	8.42	9.27E-01
cg01680054	-8.93	4.00	2.56E-02	-1.26	9.11	8.90E-01	-11.17	14.55	4.43E-01	-9.90	5.07	5.09E-02	-15.35	12.07	2.03E-01
cg22097768	14.38	6.44	2.56E-02	15.16	11.92	2.03E-01	12.21	15.54	4.32E-01	5.83	11.78	6.21E-01	25.74	13.21	5.14E-02
cg01710607	-11.76	5.27	2.56E-02	-12.38	10.61	2.43E-01	6.14	26.17	8.14E-01	-12.21	6.91	7.71E-02	-14.05	14.45	3.31E-01
cg22678708	-33.29	14.91	2.56E-02	-34.24	18.04	5.77E-02	-99.07	63.28	1.17E-01	52.89	64.33	4.11E-01	-34.86	32.69	2.86E-01
cg26736341	-9.01	4.04	2.56E-02	-8.67	5.91	1.42E-01	-8.13	8.34	3.30E-01	-23.03	13.05	7.75E-02	-4.20	8.97	6.39E-01
cg23685712	21.10	9.45	2.56E-02	37.68	24.06	1.17E-01	-31.99	36.04	3.75E-01	22.89	12.59	6.90E-02	21.52	20.47	2.93E-01
cg11793413	8.49	3.80	2.56E-02	12.32	6.66	6.44E-02	1.60	11.51	8.90E-01	3.94	5.81	4.97E-01	19.07	10.27	6.34E-02
cg05981785	9.34	4.18	2.56E-02	11.07	5.74	5.36E-02	17.70	9.96	7.55E-02	-5.35	10.74	6.18E-01	8.14	11.20	4.67E-01
cg25661142	18.91	8.47	2.56E-02	39.07	15.45	1.14E-02	9.04	37.14	8.08E-01	13.22	11.41	2.47E-01	-6.30	27.41	8.18E-01
cg16441550	20.58	9.22	2.56E-02	41.51	16.66	1.27E-02	16.03	16.41	3.29E-01	4.21	19.92	8.33E-01	11.63	22.80	6.10E-01
cg04481170	8.17	3.66	2.56E-02	9.10	5.12	7.54E-02	12.74	9.44	1.77E-01	8.23	9.22	3.72E-01	1.69	8.61	8.45E-01
cg18825458	26.18	11.73	2.56E-02	20.37	13.67	1.36E-01	5.65	49.50	9.09E-01	44.92	53.13	3.98E-01	54.56	29.41	6.35E-02
cg23123163	22.50	10.08	2.56E-02	34.88	29.35	2.35E-01	12.48	113.85	9.13E-01	18.32	11.27	1.04E-01	49.00	37.03	1.86E-01
cg04360233	20.58	9.22	2.56E-02	25.88	15.17	8.79E-02	-3.16	16.97	8.52E-01	35.57	20.02	7.57E-02	35.59	26.19	1.74E-01
cg18487246	43.36	19.42	2.56E-02	40.89	22.76	7.24E-02	-7.21	83.82	9.31E-01	-2.47	79.19	9.75E-01	89.46	48.92	6.74E-02
cg14369981	5.94	2.66	2.56E-02	4.63	3.43	1.76E-01	9.16	6.23	1.42E-01	6.32	10.36	5.42E-01	7.16	6.93	3.01E-01
cg24480012	12.68	5.68	2.56E-02	26.87	15.42	8.13E-02	36.78	46.12	4.25E-01	11.27	6.60	8.76E-02	1.13	17.23	9.47E-01
cg03220447	-30.26	13.55	2.56E-02	-33.16	13.83	1.65E-02	-66.01	25.33	9.15E-03	31.62	36.19	3.82E-01	-26.71	17.89	1.35E-01
cg18376817	23.87	10.69	2.56E-02	51.37	20.63	1.28E-02	13.84	23.76	5.60E-01	12.20	7.92	1.23E-01	52.42	33.91	1.22E-01
cg14748160	43.42	19.45	2.56E-02	-6.11	48.43	9.00E-01	-179.27	224.93	4.25E-01	61.27	21.50	4.37E-03	7.42	60.82	9.03E-01
cg06692785	9.40	4.21	2.56E-02	13.20	6.31	3.63E-02	4.20	9.85	6.70E-01	8.92	9.40	3.42E-01	5.59	10.17	5.83E-01
cg02471149	-10.24	4.59	2.56E-02	-12.92	9.54	1.76E-01	-23.84	18.61	2.00E-01	-9.81	6.04	1.04E-01	-1.01	12.74	9.37E-01
cg27405644	45.96	20.59	2.56E-02	54.74	25.02	2.87E-02	143.41	108.80	1.87E-01	41.72	102.01	6.83E-01	8.34	41.49	8.41E-01
cg16121470	-14.27	6.39	2.56E-02	-11.29	8.36	1.77E-01	-29.69	16.73	7.59E-02	9.23	37.18	8.04E-01	-15.04	13.07	2.50E-01
cg14168923	17.69	7.93	2.56E-02	54.31	26.49	4.03E-02	20.91	34.34	5.43E-01	12.32	9.50	1.95E-01	19.44	19.73	3.24E-01
cg09017619	23.02	10.32	2.56E-02	10.76	28.48	7.05E-01	-51.25	93.23	5.82E-01	27.60	11.91	2.05E-02	14.37	31.65	6.50E-01
cg08546707	9.55	4.28	2.56E-02	11.33	6.02	5.98E-02	0.48	8.21	9.53E-01	19.90	15.85	2.09E-01	14.92	11.03	1.76E-01
ch.2.800013F	27.56	12.35	2.56E-02	10.50	17.80	5.56E-01	46.77	78.05	5.49E-01	55.80	23.81	1.91E-02	28.13	26.03	2.80E-01
cg11603443	6.03	2.70	2.56E-02	7.59	3.70	4.05E-02	2.64	6.33	6.76E-01	0.23	10.39	9.83E-01	6.87	5.79	2.36E-01
cg20911168	-32.71	14.66	2.57E-02	-10.99	40.57	7.86E-01	-57.98	38.56	1.33E-01	-37.38	17.74	3.51E-02	62.32	71.35	3.82E-01
cg11516004	-9.51	4.26	2.57E-02	-22.06	12.08	6.79E-02	-20.89	22.94	3.63E-01	-5.84	4.90	2.33E-01	-19.27	14.68	1.89E-01
cg26602100	-45.76	20.51	2.57E-02	-49.77	25.96	5.52E-02	-77.78	73.19	2.88E-01	-37.18	71.85	6.05E-01	-25.76	44.14	5.59E-01
cg02129885	14.49	6.49	2.57E-02	17.77	8.66	4.01E-02	23.41	16.28	1.51E-01	26.36	22.41	2.39E-01	-7.40	14.71	6.15E-01
cg01794802	-8.61	3.86	2.57E-02	-3.85	9.50	6.86E-01	-5.79	17.74	7.44E-01	-9.69	4.68	3.86E-02	-10.35	11.72	3.77E-01

cg27287167	9.43	4.23	2.57E-02	9.35	5.35	8.02E-02	17.25	11.04	1.18E-01	9.03	12.93	4.85E-01	0.77	12.12	9.49E-01
cg15240876	-7.34	3.29	2.57E-02	-13.98	6.51	3.17E-02	-4.75	8.97	5.97E-01	-6.85	4.70	1.45E-01	1.76	9.42	8.51E-01
cg23670794	-17.27	7.74	2.57E-02	-30.64	11.76	9.21E-03	-28.00	19.38	1.48E-01	-4.76	5.51	3.88E-01	-22.22	14.78	1.33E-01
cg03437186	16.28	7.30	2.57E-02	28.68	13.26	3.05E-02	1.60	13.94	9.08E-01	21.09	16.33	1.96E-01	13.16	15.42	3.94E-01
cg14652038	10.50	4.71	2.57E-02	18.67	7.22	9.72E-03	2.70	8.84	7.60E-01	8.92	13.84	5.19E-01	4.39	11.20	6.95E-01
cg11141336	18.58	8.33	2.57E-02	45.27	19.12	1.79E-02	2.30	21.76	9.16E-01	13.74	11.06	2.14E-01	19.22	26.78	4.73E-01
cg03422204	-50.03	22.42	2.57E-02	-46.54	31.67	1.42E-01	-203.93	95.28	3.23E-02	-27.27	60.20	6.50E-01	-38.13	40.64	3.48E-01
cg18594467	-10.05	4.50	2.57E-02	-3.88	8.85	6.61E-01	-1.96	11.85	8.68E-01	-12.28	6.78	7.00E-02	-21.52	11.44	5.99E-02
cg26036806	-40.20	18.02	2.57E-02	-42.58	22.31	5.63E-02	-16.72	77.03	8.28E-01	-76.44	72.69	2.93E-01	-29.42	37.47	4.32E-01
cg11838384	26.91	12.06	2.57E-02	41.81	19.89	3.55E-02	-20.94	31.65	5.08E-01	17.22	25.65	5.02E-01	38.04	19.31	4.89E-02
cg14258935	-64.65	28.98	2.57E-02	-59.85	54.78	2.75E-01	32.27	222.18	8.85E-01	-81.69	41.11	4.69E-02	-38.10	63.83	5.51E-01
cg04527612	-11.58	5.19	2.57E-02	-23.43	10.63	2.75E-02	3.97	16.46	8.09E-01	-9.99	7.37	1.75E-01	-8.62	12.76	4.99E-01
cg17104596	-9.15	4.10	2.57E-02	-20.66	9.16	2.42E-02	-9.59	14.29	5.02E-01	-6.89	5.24	1.88E-01	0.08	12.77	9.95E-01
cg21760363	14.30	6.41	2.57E-02	8.10	19.53	6.78E-01	6.28	41.18	8.79E-01	17.69	7.17	1.36E-02	-12.70	24.49	6.04E-01
cg16654806	22.36	10.03	2.57E-02	22.61	12.35	6.73E-02	-18.80	41.21	6.48E-01	23.85	33.11	4.71E-01	33.59	22.97	1.44E-01
cg06204938	9.12	4.09	2.57E-02	3.91	5.66	4.89E-01	14.67	10.38	1.57E-01	21.02	10.65	4.84E-02	9.74	9.81	3.20E-01
cg20090957	-12.41	5.56	2.57E-02	-3.68	8.74	6.73E-01	-10.49	13.48	4.36E-01	-22.42	13.22	9.00E-02	-20.82	11.17	6.24E-02
cg08468322	19.66	8.82	2.57E-02	20.08	11.74	8.72E-02	1.22	17.61	9.45E-01	61.06	29.19	3.64E-02	22.68	21.60	2.94E-01
cg26161997	9.76	4.38	2.57E-02	10.63	6.02	7.75E-02	11.61	9.62	2.28E-01	13.39	14.31	3.49E-01	2.87	10.58	7.86E-01
cg07707586	-11.28	5.06	2.57E-02	-22.72	9.94	2.22E-02	-11.42	12.81	3.73E-01	-3.11	6.76	6.45E-01	-20.38	13.94	1.44E-01
cg21333338	10.31	4.62	2.57E-02	14.19	6.77	3.62E-02	18.19	10.40	8.04E-02	3.36	11.55	7.71E-01	-2.38	10.96	8.28E-01
cg16731266	-6.33	2.84	2.57E-02	-7.68	4.17	6.53E-02	-13.00	5.85	2.62E-02	-7.04	3.14	2.52E-02	4.50	6.03	4.56E-01
cg12709244	34.68	15.55	2.57E-02	56.82	39.54	1.51E-01	-216.27	185.54	2.44E-01	36.89	18.01	4.05E-02	-1.07	51.12	9.83E-01
cg23917744	-11.74	5.26	2.57E-02	-22.28	8.95	1.28E-02	-22.52	11.85	5.74E-02	-4.64	6.82	4.96E-01	-3.03	10.14	7.65E-01
cg02174359	20.30	9.10	2.57E-02	12.43	11.04	2.60E-01	48.53	17.45	5.43E-03	6.19	32.59	8.49E-01	12.17	18.16	5.03E-01
cg14108380	-9.27	4.16	2.57E-02	-9.53	6.38	1.35E-01	-7.91	11.20	4.80E-01	-10.45	10.52	3.20E-01	-8.91	7.85	2.57E-01
cg18794622	-12.07	5.41	2.57E-02	-18.49	10.49	7.81E-02	7.87	18.59	6.72E-01	-11.79	7.79	1.30E-01	-12.80	13.30	3.36E-01
cg25880537	-11.25	5.05	2.57E-02	-5.25	7.29	4.71E-01	-3.77	8.81	6.69E-01	-11.17	10.92	3.06E-01	-24.47	7.93	2.04E-03
cg26981076	6.22	2.79	2.57E-02	6.32	3.60	7.91E-02	9.97	7.09	1.60E-01	8.16	8.04	3.10E-01	-0.81	7.91	9.19E-01
cg06412759	-17.26	7.74	2.58E-02	-21.16	12.32	8.58E-02	-20.58	30.30	4.97E-01	-5.75	11.21	6.08E-01	-49.62	23.38	3.38E-02
cg03893271	13.53	6.07	2.58E-02	26.63	9.75	6.30E-03	0.72	15.16	9.62E-01	18.34	10.99	9.53E-02	4.12	8.82	6.41E-01
cg11260422	18.61	8.35	2.58E-02	33.79	16.55	4.12E-02	10.49	12.80	4.12E-01	38.00	26.18	1.47E-01	7.74	17.85	6.65E-01
cg03534031	5.86	2.63	2.58E-02	4.97	3.78	1.89E-01	7.08	5.61	2.07E-01	6.26	6.02	2.98E-01	6.62	7.99	4.07E-01
cg26973018	-15.06	6.75	2.58E-02	-21.18	10.97	5.36E-02	-4.27	14.00	7.60E-01	-19.32	16.51	2.42E-01	-12.69	14.36	3.77E-01
cg13628325	20.32	9.11	2.58E-02	8.54	14.48	5.55E-01	19.31	19.17	3.14E-01	26.13	31.87	4.12E-01	35.27	16.75	3.53E-02

cg03820695	14.85	6.66	2.58E-02	37.50	17.39	3.11E-02	16.67	33.69	6.21E-01	9.43	7.76	2.24E-01	22.79	24.07	3.44E-01
cg16629523	6.15	2.76	2.58E-02	1.70	4.28	6.91E-01	8.27	4.90	9.11E-02	10.16	8.34	2.23E-01	10.73	6.91	1.20E-01
cg23425316	15.80	7.09	2.58E-02	11.92	16.64	4.74E-01	42.51	19.11	2.61E-02	11.50	11.88	3.33E-01	11.38	12.44	3.60E-01
cg14602839	18.90	8.48	2.58E-02	3.55	20.34	8.61E-01	9.36	46.63	8.41E-01	21.03	10.19	3.91E-02	33.64	26.52	2.05E-01
cg20223728	18.50	8.30	2.58E-02	5.89	17.53	7.37E-01	-8.84	27.77	7.50E-01	29.24	11.54	1.13E-02	16.82	20.11	4.03E-01
cg18310205	23.27	10.44	2.58E-02	30.58	18.52	9.87E-02	4.99	46.83	9.15E-01	12.47	15.30	4.15E-01	44.84	25.51	7.87E-02
cg04189320	-16.44	7.38	2.58E-02	-3.31	10.38	7.50E-01	-26.65	13.68	5.14E-02	-9.94	17.10	5.61E-01	-33.29	14.88	2.53E-02
cg06463800	-48.98	21.97	2.58E-02	-43.40	27.83	1.19E-01	-108.22	137.34	4.31E-01	-8.14	96.33	9.33E-01	-62.64	40.18	1.19E-01
cg09017434	6.65	2.98	2.58E-02	10.19	4.85	3.56E-02	5.07	6.72	4.50E-01	12.21	8.21	1.37E-01	0.62	5.51	9.10E-01
cg16225405	27.75	12.45	2.58E-02	20.98	24.35	3.89E-01	18.32	56.32	7.45E-01	21.57	16.97	2.04E-01	64.43	31.98	4.39E-02
cg27441114	7.29	3.27	2.58E-02	8.96	4.51	4.71E-02	10.09	7.93	2.03E-01	-0.60	7.48	9.36E-01	8.59	9.65	3.73E-01
cg10692363	7.69	3.45	2.58E-02	8.39	4.59	6.76E-02	8.83	8.02	2.71E-01	13.60	13.41	3.11E-01	2.28	8.04	7.76E-01
cg00920139	13.67	6.13	2.58E-02	26.80	11.69	2.19E-02	18.09	14.75	2.20E-01	12.56	6.23	4.37E-02	-13.39	17.55	4.45E-01
cg24926465	-8.02	3.60	2.58E-02	-11.48	8.27	1.65E-01	-16.03	11.66	1.69E-01	-4.49	4.73	3.43E-01	-12.57	9.71	1.96E-01
cg06623274	-9.69	4.35	2.58E-02	-13.86	7.99	8.30E-02	-3.97	10.96	7.17E-01	-10.13	6.62	1.26E-01	-5.20	12.74	6.84E-01
cg14305028	19.73	8.85	2.58E-02	18.87	17.85	2.90E-01	6.27	16.25	6.99E-01	26.37	17.44	1.31E-01	32.26	19.82	1.04E-01
cg16256643	16.93	7.59	2.58E-02	15.32	10.22	1.34E-01	32.77	19.95	1.00E-01	-5.90	41.09	8.86E-01	14.59	14.65	3.19E-01
cg13299325	-9.53	4.28	2.58E-02	-12.90	5.21	1.32E-02	3.62	8.65	6.76E-01	-20.58	11.04	6.24E-02	-8.41	8.00	2.93E-01
cg08522340	5.99	2.69	2.58E-02	7.41	4.17	7.57E-02	0.59	7.07	9.34E-01	8.51	4.87	8.08E-02	1.78	7.28	8.07E-01
cg24873842	12.73	5.71	2.58E-02	16.69	10.44	1.10E-01	-0.43	13.01	9.73E-01	11.59	9.07	2.01E-01	29.07	17.16	9.03E-02
cg16071219	-6.25	2.80	2.58E-02	-2.50	4.13	5.45E-01	-9.94	5.29	6.04E-02	-11.17	8.48	1.88E-01	-7.26	7.24	3.16E-01
cg14235271	18.09	8.11	2.58E-02	19.29	19.84	3.31E-01	23.29	41.11	5.71E-01	22.34	9.69	2.12E-02	-18.16	26.56	4.94E-01
cg14542646	32.97	14.79	2.58E-02	22.13	18.49	2.31E-01	37.38	22.30	9.37E-02	11.14	11.56	3.35E-01	94.27	30.15	1.77E-03
cg02288301	24.15	10.83	2.58E-02	32.74	17.66	6.37E-02	-9.93	28.53	7.28E-01	51.48	29.73	8.34E-02	18.52	18.41	3.14E-01
cg08041364	37.28	16.72	2.58E-02	-10.27	45.89	8.23E-01	29.37	137.92	8.31E-01	48.96	19.73	1.31E-02	22.70	45.65	6.19E-01
cg05614904	-28.36	12.73	2.58E-02	-25.85	15.08	8.66E-02	-91.51	62.44	1.43E-01	-27.66	60.65	6.48E-01	-24.40	28.27	3.88E-01
cg10090212	20.22	9.07	2.58E-02	19.38	20.68	3.49E-01	65.64	45.25	1.47E-01	16.59	11.12	1.36E-01	27.54	28.39	3.32E-01
cg13048008	9.23	4.14	2.58E-02	9.37	5.17	6.96E-02	16.17	12.96	2.12E-01	13.69	13.91	3.25E-01	2.07	10.13	8.38E-01
cg25021575	21.64	9.71	2.58E-02	22.07	21.33	3.01E-01	-47.53	42.77	2.66E-01	25.66	12.38	3.82E-02	29.60	27.37	2.79E-01
cg25684349	-10.04	4.50	2.58E-02	-6.28	6.06	3.00E-01	-22.21	10.42	3.31E-02	3.56	16.50	8.29E-01	-14.46	10.46	1.67E-01
cg14930065	11.12	4.99	2.58E-02	11.08	7.29	1.29E-01	14.48	9.75	1.37E-01	1.31	15.89	9.34E-01	11.72	12.05	3.31E-01
cg01003815	-8.61	3.86	2.58E-02	-12.37	8.52	1.47E-01	-7.24	14.51	6.18E-01	-7.67	4.81	1.11E-01	-7.77	13.76	5.72E-01
cg15726245	15.01	6.74	2.58E-02	10.61	14.52	4.65E-01	17.72	21.20	4.03E-01	15.17	8.85	8.66E-02	20.53	20.77	3.23E-01
cg16620537	26.96	12.10	2.58E-02	38.91	17.15	2.33E-02	-9.52	39.45	8.09E-01	3.85	33.60	9.09E-01	28.69	22.91	2.11E-01
cg22786472	-9.01	4.04	2.58E-02	-14.14	6.82	3.82E-02	-7.37	11.04	5.05E-01	-1.25	7.10	8.60E-01	-13.90	9.25	1.33E-01

cg19603195	28.00	12.56	2.58E-02	14.67	15.09	3.31E-01	26.68	23.30	2.52E-01	107.12	45.01	1.73E-02	28.90	21.07	1.70E-01
cg03122201	24.17	10.84	2.58E-02	26.71	24.76	2.81E-01	10.46	54.80	8.49E-01	26.62	13.31	4.55E-02	9.12	33.48	7.85E-01
cg17491031	16.15	7.25	2.58E-02	14.43	15.59	3.55E-01	-4.15	25.61	8.71E-01	18.36	9.50	5.32E-02	22.01	20.80	2.90E-01
cg06865992	9.04	4.06	2.58E-02	10.36	5.50	5.97E-02	-3.70	10.36	7.21E-01	26.34	19.31	1.73E-01	10.89	7.99	1.73E-01
cg10155026	-11.08	4.97	2.58E-02	-13.61	7.06	5.38E-02	0.34	12.96	9.79E-01	-17.46	13.30	1.89E-01	-8.94	10.69	4.03E-01
cg10639428	25.42	11.41	2.59E-02	5.36	19.18	7.80E-01	38.91	22.03	7.74E-02	15.69	17.06	3.58E-01	62.21	26.82	2.03E-02
cg02834871	21.29	9.55	2.59E-02	16.75	18.85	3.74E-01	8.76	20.53	6.69E-01	29.25	15.22	5.46E-02	26.90	26.28	3.06E-01
cg14666892	-53.89	24.18	2.59E-02	-37.54	29.38	2.01E-01	-141.61	131.66	2.82E-01	-19.91	75.91	7.93E-01	-115.69	55.90	3.85E-02
cg05289022	10.06	4.51	2.59E-02	6.75	6.13	2.71E-01	23.43	11.52	4.19E-02	10.67	12.68	4.00E-01	8.14	10.68	4.46E-01
cg14016166	-17.80	7.99	2.59E-02	-15.05	17.21	3.82E-01	-26.22	37.24	4.81E-01	-17.15	10.18	9.20E-02	-22.77	22.84	3.19E-01
cg02642958	16.48	7.40	2.59E-02	25.26	9.86	1.04E-02	21.08	31.47	5.03E-01	21.00	27.36	4.43E-01	-1.41	13.31	9.16E-01
cg02334109	10.62	4.77	2.59E-02	13.36	6.80	4.95E-02	9.40	10.56	3.73E-01	-3.04	17.97	8.66E-01	10.05	9.85	3.08E-01
cg01921126	-6.33	2.84	2.59E-02	-6.90	5.31	1.94E-01	-4.39	7.39	5.52E-01	-5.87	4.51	1.93E-01	-8.14	6.90	2.38E-01
cg05037628	18.19	8.16	2.59E-02	-5.34	24.43	8.27E-01	-54.72	119.34	6.47E-01	24.15	8.96	7.05E-03	-18.53	35.22	5.99E-01
cg25918541	21.85	9.80	2.59E-02	10.97	28.30	6.98E-01	-7.97	70.04	9.09E-01	27.45	11.15	1.38E-02	-6.45	33.35	8.47E-01
cg04198914	-5.20	2.33	2.59E-02	-5.99	3.29	6.88E-02	-4.69	5.34	3.79E-01	-7.96	9.65	4.10E-01	-3.34	4.68	4.76E-01
cg12977146	23.16	10.39	2.59E-02	35.32	32.89	2.83E-01	39.69	99.14	6.89E-01	21.11	11.49	6.62E-02	27.16	39.10	4.87E-01
cg18583378	15.84	7.11	2.59E-02	33.66	16.96	4.71E-02	1.61	28.10	9.54E-01	17.49	9.34	6.11E-02	-1.81	16.77	9.14E-01
cg12864853	26.40	11.85	2.59E-02	42.75	18.48	2.07E-02	-9.06	34.08	7.90E-01	36.24	25.80	1.60E-01	8.85	23.39	7.05E-01
cg20249071	-25.96	11.65	2.59E-02	-15.04	14.59	3.03E-01	-17.42	31.31	5.78E-01	-14.47	7.20	4.44E-02	-66.68	19.01	4.53E-04
cg14684088	19.97	8.96	2.59E-02	22.23	14.19	1.17E-01	25.48	16.86	1.31E-01	13.20	21.04	5.30E-01	10.96	24.29	6.52E-01
cg20402747	-41.58	18.66	2.59E-02	-42.17	25.44	9.74E-02	113.15	105.82	2.85E-01	-66.71	46.26	1.49E-01	-43.11	36.06	2.32E-01
cg03502656	6.89	3.09	2.59E-02	7.75	4.36	7.58E-02	2.63	8.64	7.61E-01	9.52	9.36	3.09E-01	6.21	6.04	3.04E-01
cg19551912	-13.49	6.05	2.59E-02	-21.78	11.90	6.72E-02	-13.62	25.40	5.92E-01	-10.50	8.05	1.92E-01	-9.58	17.55	5.85E-01
cg05340094	-9.26	4.16	2.59E-02	-4.33	7.41	5.59E-01	-8.47	10.46	4.18E-01	-17.23	7.04	1.43E-02	-3.05	9.85	7.57E-01
cg15458222	11.17	5.01	2.59E-02	22.52	8.62	8.99E-03	-0.13	14.70	9.93E-01	9.61	6.96	1.67E-01	-1.75	14.31	9.03E-01
cg26937038	15.74	7.07	2.59E-02	22.24	9.55	1.99E-02	26.51	19.10	1.65E-01	-1.83	21.40	9.32E-01	0.69	15.54	9.65E-01
cg24403804	8.63	3.87	2.59E-02	9.49	5.68	9.46E-02	6.13	11.28	5.87E-01	13.06	7.96	1.01E-01	2.20	9.12	8.10E-01
cg06093354	36.28	16.29	2.59E-02	13.34	31.26	6.70E-01	67.63	174.02	6.98E-01	42.14	20.44	3.92E-02	62.71	56.00	2.63E-01
cg01366419	16.11	7.23	2.59E-02	35.26	12.91	6.29E-03	7.81	17.31	6.52E-01	11.42	16.42	4.87E-01	6.62	11.67	5.71E-01
cg08179898	-40.07	17.99	2.59E-02	-19.47	39.90	6.26E-01	-240.71	177.82	1.76E-01	-40.86	22.07	6.41E-02	-53.28	51.45	3.00E-01
cg13000082	24.40	10.95	2.59E-02	30.93	18.34	9.18E-02	20.46	31.04	5.10E-01	16.69	34.67	6.30E-01	21.86	16.92	1.96E-01
cg08412537	8.73	3.92	2.59E-02	10.91	5.69	5.50E-02	13.06	8.18	1.10E-01	-7.48	11.11	5.01E-01	8.66	9.50	3.62E-01
cg07314821	21.83	9.80	2.59E-02	21.45	18.12	2.36E-01	35.11	20.30	8.37E-02	9.48	7.05	1.79E-01	62.69	28.50	2.78E-02
cg17294928	-42.96	19.28	2.59E-02	-49.35	39.21	2.08E-01	-13.65	128.59	9.15E-01	-21.58	25.47	3.97E-01	-112.95	47.85	1.83E-02

cg00983912	-14.99	6.73	2.59E-02	-25.89	19.55	1.85E-01	-20.98	35.68	5.57E-01	-12.19	7.53	1.06E-01	-30.28	30.77	3.25E-01
cg06545019	18.04	8.10	2.59E-02	21.98	11.88	6.44E-02	18.10	20.08	3.67E-01	14.48	22.83	5.26E-01	12.41	16.30	4.47E-01
cg08979737	19.00	8.53	2.59E-02	11.27	17.04	5.08E-01	-0.40	34.35	9.91E-01	27.19	11.63	1.94E-02	10.48	22.04	6.34E-01
cg10328220	24.04	10.79	2.59E-02	31.09	13.85	2.48E-02	-8.76	35.53	8.05E-01	6.73	38.48	8.61E-01	24.50	22.91	2.85E-01
cg10784143	19.39	8.71	2.59E-02	15.34	18.40	4.04E-01	-11.27	35.11	7.48E-01	16.55	11.41	1.47E-01	52.98	23.93	2.68E-02
cg17785786	-16.35	7.34	2.59E-02	-30.87	15.98	5.34E-02	-33.24	49.90	5.05E-01	-14.20	9.09	1.18E-01	1.32	21.70	9.51E-01
cg27198696	9.80	4.40	2.59E-02	12.90	5.90	2.89E-02	10.91	9.99	2.74E-01	11.49	13.13	3.82E-01	-5.59	11.84	6.37E-01
cg21474257	20.68	9.28	2.59E-02	31.89	20.42	1.18E-01	44.18	23.44	5.94E-02	13.28	12.91	3.04E-01	2.41	26.85	9.28E-01
cg14572581	27.56	12.37	2.59E-02	4.09	24.43	8.67E-01	-67.54	85.92	4.32E-01	29.71	9.45	1.66E-03	65.72	30.62	3.18E-02
cg23825522	-19.25	8.64	2.59E-02	-16.48	21.80	4.50E-01	47.25	46.28	3.07E-01	-20.48	10.17	4.39E-02	-41.14	29.63	1.65E-01
cg07072366	18.28	8.21	2.59E-02	32.51	13.97	2.00E-02	16.26	20.02	4.17E-01	20.70	19.33	2.84E-01	1.94	14.82	8.96E-01
cg13166171	-15.80	7.09	2.59E-02	-33.07	11.62	4.42E-03	-9.69	12.57	4.41E-01	-8.62	17.60	6.24E-01	-4.18	13.77	7.62E-01
cg10145533	-6.01	2.70	2.59E-02	-6.46	3.86	9.44E-02	-8.19	8.42	3.31E-01	-6.11	5.80	2.92E-01	-3.61	6.16	5.58E-01
cg14512089	98.39	44.18	2.59E-02	135.09	71.98	6.05E-02	38.08	261.61	8.84E-01	201.20	109.93	6.72E-02	32.12	67.13	6.32E-01
cg03447699	6.52	2.93	2.59E-02	6.99	4.31	1.05E-01	6.00	6.40	3.48E-01	10.42	9.31	2.63E-01	4.39	6.09	4.71E-01
cg22915906	-16.56	7.44	2.59E-02	-6.31	16.13	6.95E-01	6.33	27.16	8.16E-01	-22.19	9.32	1.73E-02	-20.57	26.87	4.44E-01
cg09444036	16.75	7.52	2.60E-02	13.18	14.76	3.72E-01	-23.16	35.71	5.17E-01	23.25	9.96	1.95E-02	8.68	21.25	6.83E-01
cg27356296	-8.61	3.87	2.60E-02	-2.35	8.73	7.88E-01	-23.33	14.48	1.07E-01	-6.29	5.02	2.11E-01	-19.71	10.33	5.63E-02
cg12689425	-18.78	8.43	2.60E-02	-14.68	13.96	2.93E-01	-34.51	35.19	3.27E-01	-29.41	14.40	4.11E-02	-5.76	17.41	7.41E-01
cg24580185	52.36	23.51	2.60E-02	44.06	29.72	1.38E-01	129.17	165.84	4.36E-01	109.93	76.36	1.50E-01	45.41	46.20	3.26E-01
cg12421138	-10.64	4.78	2.60E-02	-9.94	9.94	3.17E-01	-19.59	14.30	1.71E-01	-9.50	6.58	1.49E-01	-8.82	13.21	5.04E-01
cg13922669	13.21	5.93	2.60E-02	22.75	10.09	2.41E-02	9.11	16.85	5.89E-01	15.10	14.25	2.89E-01	4.46	9.94	6.53E-01
cg27062215	9.60	4.31	2.60E-02	4.78	5.25	3.63E-01	13.22	8.16	1.05E-01	30.33	13.09	2.05E-02	6.22	8.16	4.46E-01
cg25310241	36.79	16.52	2.60E-02	46.48	23.13	4.45E-02	44.45	46.28	3.37E-01	51.28	42.99	2.33E-01	-0.75	35.65	9.83E-01
cg13555772	11.25	5.05	2.60E-02	11.17	7.11	1.17E-01	5.68	12.15	6.40E-01	16.86	12.90	1.91E-01	12.10	12.26	3.24E-01
cg08454455	17.39	7.81	2.60E-02	20.49	12.04	8.89E-02	-13.16	19.81	5.06E-01	28.79	15.20	5.82E-02	20.08	18.84	2.86E-01
cg16560847	-13.56	6.09	2.60E-02	-21.88	10.17	3.15E-02	-9.99	14.96	5.04E-01	-5.66	11.80	6.31E-01	-12.19	13.29	3.59E-01
cg01725586	27.23	12.23	2.60E-02	-11.77	29.40	6.89E-01	2.48	65.99	9.70E-01	35.31	14.75	1.67E-02	46.70	37.73	2.16E-01
cg20087221	46.30	20.79	2.60E-02	59.68	26.97	2.69E-02	-26.64	84.78	7.53E-01	39.84	45.95	3.86E-01	30.40	55.41	5.83E-01
cg08626888	18.26	8.20	2.60E-02	29.92	12.24	1.45E-02	53.75	38.65	1.64E-01	7.38	6.24	2.37E-01	27.40	21.84	2.10E-01
cg12321011	16.63	7.47	2.60E-02	29.80	18.63	1.10E-01	-6.46	23.74	7.86E-01	16.63	9.10	6.76E-02	19.21	28.96	5.07E-01
cg21129006	-9.00	4.04	2.60E-02	-10.51	7.34	1.53E-01	-0.84	11.59	9.43E-01	-6.03	6.23	3.33E-01	-20.63	10.31	4.54E-02
cg19716038	-10.68	4.80	2.60E-02	-9.81	9.79	3.16E-01	-7.95	16.63	6.33E-01	-13.50	6.27	3.13E-02	2.55	15.82	8.72E-01
cg16788322	23.65	10.62	2.60E-02	30.07	16.37	6.63E-02	14.31	38.20	7.08E-01	14.22	27.72	6.08E-01	21.97	17.83	2.18E-01
cg16536399	-66.46	29.85	2.60E-02	-41.96	41.56	3.13E-01	-60.39	169.51	7.22E-01	-67.45	59.38	2.56E-01	-129.23	66.68	5.26E-02

cg04851044	27.29	12.26	2.60E-02	58.32	17.06	6.32E-04	33.25	24.17	1.69E-01	14.41	7.12	4.30E-02	4.71	26.47	8.59E-01
cg04027916	-31.22	14.02	2.60E-02	-42.10	18.46	2.26E-02	-54.83	37.03	1.39E-01	-16.28	48.83	7.39E-01	11.52	31.59	7.15E-01
cg00712050	-11.15	5.01	2.60E-02	-9.76	7.49	1.92E-01	-1.11	11.18	9.21E-01	-15.56	15.76	3.23E-01	-19.84	9.98	4.69E-02
cg08903465	-16.88	7.58	2.60E-02	-30.71	12.45	1.36E-02	-10.11	17.72	5.68E-01	-2.46	18.35	8.93E-01	-11.68	14.46	4.19E-01
cg11063170	7.34	3.30	2.60E-02	6.29	7.36	3.92E-01	17.24	11.30	1.27E-01	6.74	4.18	1.07E-01	4.56	10.81	6.73E-01
cg23143313	-7.76	3.49	2.60E-02	-6.90	5.14	1.79E-01	1.64	9.54	8.64E-01	-9.13	7.97	2.52E-01	-14.23	7.52	5.84E-02
cg13487334	17.73	7.97	2.60E-02	11.78	15.71	4.53E-01	16.44	18.01	3.61E-01	17.44	12.17	1.52E-01	33.73	23.07	1.44E-01
cg23451821	14.45	6.49	2.60E-02	29.39	13.20	2.60E-02	6.08	29.22	8.35E-01	9.72	8.16	2.33E-01	11.77	23.67	6.19E-01
cg23803206	8.77	3.94	2.60E-02	4.91	6.31	4.37E-01	9.40	8.62	2.76E-01	13.40	8.45	1.13E-01	10.75	9.17	2.41E-01
cg03483330	-9.28	4.17	2.60E-02	-15.82	8.63	6.69E-02	5.31	11.43	6.42E-01	-9.40	6.36	1.39E-01	-11.08	9.23	2.30E-01
cg12984636	-10.47	4.70	2.60E-02	-9.35	12.02	4.37E-01	19.67	22.21	3.76E-01	-12.32	5.69	3.04E-02	-12.60	13.58	3.54E-01
cg17011300	15.55	6.98	2.60E-02	14.46	13.90	2.98E-01	10.94	22.98	6.34E-01	13.57	9.44	1.51E-01	31.98	21.22	1.32E-01
cg00036137	13.88	6.24	2.60E-02	12.70	12.49	3.09E-01	20.56	15.69	1.90E-01	7.74	6.73	2.50E-01	47.53	21.34	2.59E-02
cg13180678	-32.06	14.40	2.60E-02	-6.19	46.23	8.93E-01	114.65	226.16	6.12E-01	-41.57	15.90	8.92E-03	28.21	51.57	5.84E-01
cg03127898	8.38	3.76	2.60E-02	6.80	5.01	1.75E-01	11.45	8.97	2.01E-01	6.45	10.63	5.44E-01	12.71	10.25	2.15E-01
cg00926926	20.81	9.35	2.60E-02	-0.58	22.32	9.79E-01	24.51	28.08	3.83E-01	20.59	11.78	8.07E-02	62.30	32.24	5.34E-02
cg01863398	21.94	9.86	2.60E-02	49.35	20.26	1.49E-02	4.07	42.65	9.24E-01	10.89	12.09	3.68E-01	36.75	31.23	2.39E-01
cg16801267	21.31	9.57	2.60E-02	12.96	29.10	6.56E-01	89.03	114.01	4.35E-01	23.40	10.71	2.89E-02	6.72	32.77	8.37E-01
cg03909081	15.50	6.96	2.60E-02	15.71	19.19	4.13E-01	-3.20	21.30	8.81E-01	16.56	8.50	5.15E-02	29.34	23.07	2.03E-01
cg04751358	-8.60	3.87	2.60E-02	-11.90	8.90	1.81E-01	-9.36	21.20	6.59E-01	-8.34	4.67	7.41E-02	-3.57	12.74	7.80E-01
cg13620439	-12.24	5.50	2.60E-02	-10.24	10.15	3.13E-01	1.42	15.31	9.26E-01	-14.89	8.59	8.29E-02	-19.83	13.46	1.41E-01
cg02300825	41.93	18.84	2.60E-02	35.09	27.95	2.09E-01	25.01	163.79	8.79E-01	52.53	35.45	1.38E-01	43.30	37.68	2.51E-01
cg14397171	-12.81	5.75	2.60E-02	-13.24	8.65	1.26E-01	-5.57	15.33	7.17E-01	-28.79	15.80	6.84E-02	-8.25	10.81	4.45E-01
cg00545804	21.00	9.44	2.60E-02	-4.10	31.18	8.96E-01	-16.09	52.44	7.59E-01	28.25	10.69	8.23E-03	-1.17	30.30	9.69E-01
cg24856673	5.72	2.57	2.60E-02	5.27	3.70	1.55E-01	5.38	5.28	3.08E-01	14.29	7.36	5.23E-02	1.03	6.44	8.74E-01
cg12313535	15.37	6.91	2.61E-02	11.79	15.76	4.54E-01	39.98	18.77	3.31E-02	9.86	8.91	2.69E-01	24.74	25.85	3.39E-01
cg26146390	18.30	8.22	2.61E-02	11.12	18.78	5.54E-01	-3.54	48.45	9.42E-01	22.52	9.88	2.26E-02	7.60	27.99	7.86E-01
cg09322605	13.37	6.01	2.61E-02	18.85	12.93	1.45E-01	-4.82	18.10	7.90E-01	10.03	8.34	2.29E-01	29.87	15.27	5.04E-02
cg12195695	-27.12	12.19	2.61E-02	-30.83	14.31	3.12E-02	23.43	32.08	4.65E-01	-61.46	44.99	1.72E-01	-36.92	23.40	1.15E-01
cg21670717	-12.97	5.83	2.61E-02	-17.58	10.27	8.69E-02	1.51	19.14	9.37E-01	-6.70	4.76	1.59E-01	-30.00	11.42	8.63E-03
cg19084628	-43.02	19.33	2.61E-02	-56.74	26.50	3.23E-02	33.13	67.75	6.25E-01	-53.39	42.84	2.13E-01	-25.46	45.22	5.73E-01
cg03370752	5.66	2.54	2.61E-02	4.60	3.25	1.57E-01	14.27	6.53	2.89E-02	6.50	7.20	3.67E-01	-1.18	7.60	8.77E-01
cg26873439	-23.71	10.65	2.61E-02	-24.81	16.24	1.27E-01	-30.60	42.30	4.70E-01	-36.11	17.89	4.35E-02	11.37	27.38	6.78E-01
cg27109748	-10.88	4.89	2.61E-02	-9.84	6.85	1.51E-01	1.18	12.97	9.27E-01	-12.80	14.19	3.67E-01	-19.70	10.22	5.39E-02
cg01662334	11.55	5.19	2.61E-02	19.52	10.97	7.52E-02	13.02	13.95	3.51E-01	11.67	6.89	9.03E-02	-17.51	19.51	3.69E-01

cg17462107	-12.39	5.57	2.61E-02	-28.96	12.99	2.58E-02	-2.56	22.75	9.10E-01	-6.93	5.52	2.09E-01	-24.61	15.95	1.23E-01
cg14102811	31.08	13.97	2.61E-02	-3.86	32.56	9.06E-01	-62.15	120.40	6.06E-01	42.62	16.95	1.19E-02	29.85	39.70	4.52E-01
cg04771206	17.31	7.78	2.61E-02	22.56	17.06	1.86E-01	20.47	38.24	5.93E-01	17.05	9.49	7.23E-02	3.95	27.83	8.87E-01
cg17526513	-29.23	13.14	2.61E-02	-41.34	18.21	2.32E-02	-15.20	39.36	6.99E-01	-8.96	31.75	7.78E-01	-22.82	29.59	4.41E-01
cg05626128	13.08	5.88	2.61E-02	10.39	8.14	2.02E-01	19.53	13.19	1.39E-01	17.66	16.74	2.91E-01	10.25	14.82	4.89E-01
cg10330019	27.51	12.36	2.61E-02	35.50	18.44	5.43E-02	56.00	39.81	1.60E-01	5.93	35.76	8.68E-01	16.28	21.37	4.46E-01
cg13284614	36.41	16.36	2.61E-02	41.07	22.19	6.42E-02	51.41	58.21	3.77E-01	-27.52	51.61	5.94E-01	46.19	31.11	1.38E-01
cg00244517	15.51	6.97	2.61E-02	12.79	14.70	3.84E-01	18.03	11.96	1.32E-01	8.55	12.70	5.01E-01	29.32	19.03	1.23E-01
cg25521400	6.33	2.84	2.61E-02	6.01	3.67	1.01E-01	8.63	7.09	2.24E-01	12.78	9.13	1.62E-01	0.60	7.60	9.37E-01
cg08070022	12.82	5.76	2.61E-02	12.71	6.37	4.58E-02	19.25	15.88	2.26E-01	52.39	26.74	5.01E-02	1.82	11.36	8.73E-01
cg09698471	12.67	5.69	2.61E-02	18.60	9.96	6.18E-02	11.89	16.34	4.67E-01	13.78	10.52	1.90E-01	4.28	11.20	7.02E-01
cg02086839	10.28	4.62	2.61E-02	3.44	5.21	5.09E-01	12.37	9.19	1.78E-01	26.30	14.70	7.36E-02	18.52	9.99	6.37E-02
cg15578948	-47.90	21.53	2.61E-02	-50.10	26.42	5.79E-02	170.03	162.92	2.97E-01	-7.22	67.33	9.15E-01	-77.97	46.30	9.22E-02
cg00921097	-11.44	5.14	2.61E-02	-15.83	7.36	3.14E-02	-12.13	12.35	3.26E-01	-13.72	16.86	4.16E-01	-1.34	10.38	8.97E-01
cg07462863	-8.66	3.89	2.61E-02	-15.28	7.05	3.02E-02	-3.81	10.65	7.21E-01	-2.79	5.93	6.38E-01	-17.56	10.78	1.03E-01
cg27246991	29.48	13.25	2.61E-02	28.48	19.99	1.54E-01	27.03	31.71	3.94E-01	0.85	32.49	9.79E-01	55.12	28.28	5.13E-02
cg23709231	-15.54	6.99	2.61E-02	-22.03	10.63	3.82E-02	-7.56	15.26	6.20E-01	-3.00	21.02	8.86E-01	-16.57	14.02	2.37E-01
cg01845501	-9.51	4.28	2.61E-02	-5.65	6.37	3.75E-01	-15.17	10.00	1.29E-01	-2.36	8.86	7.90E-01	-23.29	10.44	2.57E-02
cg08081805	17.42	7.83	2.61E-02	5.63	17.72	7.51E-01	101.32	124.26	4.15E-01	17.59	9.16	5.47E-02	44.11	29.79	1.39E-01
cg23620889	30.98	13.93	2.61E-02	44.67	20.37	2.83E-02	18.19	29.62	5.39E-01	-4.20	42.79	9.22E-01	31.75	30.72	3.01E-01
cg19006127	-32.46	14.59	2.61E-02	-47.45	19.03	1.26E-02	-50.95	46.16	2.70E-01	-27.72	60.34	6.46E-01	8.50	28.98	7.69E-01
cg18041814	-9.25	4.16	2.61E-02	-16.41	7.48	2.83E-02	-8.13	11.16	4.66E-01	-6.54	6.24	2.95E-01	-1.34	12.68	9.16E-01
cg13743500	19.05	8.56	2.61E-02	17.58	11.33	1.21E-01	24.52	22.25	2.71E-01	-10.13	37.24	7.86E-01	25.95	17.94	1.48E-01
cg05036937	-12.37	5.56	2.61E-02	-16.32	14.62	2.64E-01	-40.67	27.43	1.38E-01	-9.51	6.54	1.46E-01	-16.10	18.41	3.82E-01
cg07139165	15.22	6.84	2.61E-02	-3.17	15.32	8.36E-01	33.65	29.36	2.52E-01	19.62	8.65	2.33E-02	14.48	19.67	4.62E-01
cg05495155	44.92	20.19	2.61E-02	36.10	27.18	1.84E-01	57.21	115.10	6.19E-01	4.02	61.18	9.48E-01	73.92	36.36	4.21E-02
cg00370820	12.38	5.56	2.61E-02	12.81	14.63	3.81E-01	24.38	25.12	3.32E-01	13.23	6.47	4.11E-02	-6.52	21.39	7.60E-01
cg03030665	23.08	10.37	2.61E-02	25.00	19.97	2.11E-01	15.45	19.02	4.16E-01	28.89	19.64	1.41E-01	23.92	26.46	3.66E-01
cg01295264	15.63	7.03	2.61E-02	11.15	10.56	2.91E-01	23.92	12.61	5.79E-02	-10.25	22.50	6.49E-01	28.65	18.20	1.15E-01
cg27206903	13.45	6.04	2.61E-02	12.85	10.16	2.06E-01	9.62	10.16	3.43E-01	14.84	12.01	2.17E-01	44.55	30.64	1.46E-01
cg18769353	19.26	8.66	2.61E-02	14.44	10.84	1.83E-01	31.58	26.37	2.31E-01	62.28	38.29	1.04E-01	17.05	19.21	3.75E-01
cg09383816	-35.15	15.80	2.61E-02	-21.82	20.20	2.80E-01	-18.51	59.10	7.54E-01	-40.50	67.52	5.49E-01	-69.70	30.87	2.40E-02
cg12333043	-8.42	3.79	2.61E-02	-8.38	7.32	2.53E-01	-0.33	13.82	9.81E-01	-8.63	5.23	9.92E-02	-12.22	10.33	2.37E-01
cg17183531	16.71	7.51	2.61E-02	13.69	11.92	2.51E-01	7.14	14.04	6.11E-01	26.05	19.98	1.92E-01	31.75	17.99	7.75E-02
ch.19.21460585R	-22.57	10.15	2.61E-02	-26.97	12.97	3.75E-02	-19.21	25.94	4.59E-01	29.90	43.71	4.94E-01	-26.16	23.87	2.73E-01

cg21862081	-8.93	4.01	2.61E-02	-13.49	6.39	3.48E-02	-7.25	9.86	4.62E-01	1.82	9.42	8.47E-01	-10.61	7.91	1.80E-01
cg04613133	-44.52	20.02	2.61E-02	-39.06	25.01	1.18E-01	-70.93	82.97	3.93E-01	2.70	83.09	9.74E-01	-63.83	40.60	1.16E-01
cg10228777	22.34	10.04	2.61E-02	16.42	36.56	6.53E-01	112.66	114.51	3.25E-01	20.91	10.83	5.36E-02	39.37	41.91	3.48E-01
cg24427660	6.38	2.87	2.61E-02	6.04	3.47	8.22E-02	14.36	8.30	8.34E-02	1.26	9.91	8.99E-01	3.83	8.44	6.50E-01
cg00336475	-12.70	5.71	2.61E-02	-27.45	11.89	2.09E-02	3.98	14.68	7.87E-01	-11.96	7.79	1.25E-01	-6.70	20.09	7.39E-01
cg24135112	29.31	13.18	2.62E-02	-19.04	43.49	6.62E-01	21.94	156.06	8.88E-01	31.66	14.35	2.74E-02	72.99	54.95	1.84E-01
cg02647874	7.92	3.56	2.62E-02	8.59	5.37	1.09E-01	9.36	7.41	2.06E-01	2.30	10.94	8.33E-01	7.77	7.55	3.03E-01
cg23104436	-11.51	5.18	2.62E-02	-27.48	13.95	4.89E-02	15.28	22.37	4.95E-01	-9.59	6.13	1.18E-01	-17.89	16.77	2.86E-01
cg06030166	-32.66	14.69	2.62E-02	-0.59	38.23	9.88E-01	203.75	200.27	3.09E-01	-37.78	16.71	2.38E-02	-60.28	53.86	2.63E-01
cg00424286	-8.22	3.70	2.62E-02	-9.57	6.39	1.34E-01	-14.73	10.40	1.56E-01	-5.67	5.76	3.25E-01	-6.48	10.42	5.34E-01
cg06803184	-36.41	16.37	2.62E-02	-36.81	18.23	4.35E-02	-81.23	68.15	2.33E-01	41.20	93.15	6.58E-01	-31.52	50.55	5.33E-01
cg04750421	-9.38	4.22	2.62E-02	-11.60	6.30	6.56E-02	-6.23	10.81	5.64E-01	-12.01	9.44	2.03E-01	-4.15	9.43	6.60E-01
cg11478024	4.88	2.19	2.62E-02	3.40	2.93	2.46E-01	8.90	5.09	8.02E-02	5.58	5.77	3.33E-01	4.71	6.70	4.82E-01
cg17863681	-8.03	3.61	2.62E-02	-12.49	4.91	1.10E-02	-2.93	8.62	7.34E-01	-1.82	17.39	9.17E-01	-2.83	7.36	7.00E-01
cg07575519	-14.78	6.65	2.62E-02	-32.98	10.30	1.37E-03	-6.60	17.83	7.11E-01	-7.35	5.83	2.07E-01	-12.43	12.76	3.30E-01
cg03229538	-16.01	7.20	2.62E-02	-10.27	16.07	5.23E-01	1.77	31.21	9.55E-01	-20.08	8.80	2.25E-02	-7.77	26.03	7.65E-01
cg03407228	-12.77	5.74	2.62E-02	-6.17	8.19	4.52E-01	-15.99	13.00	2.19E-01	-36.00	16.13	2.56E-02	-11.00	13.29	4.08E-01
cg06926146	17.79	8.00	2.62E-02	34.54	14.56	1.77E-02	21.51	26.06	4.09E-01	-1.03	16.43	9.50E-01	15.20	13.21	2.50E-01
cg27312255	-10.83	4.87	2.62E-02	1.61	13.74	9.07E-01	-18.22	23.20	4.32E-01	-14.28	5.59	1.07E-02	8.44	18.21	6.43E-01
cg06438484	-21.67	9.75	2.62E-02	-14.91	15.03	3.21E-01	-28.66	28.03	3.07E-01	-6.03	27.03	8.23E-01	-33.99	17.02	4.59E-02
cg02432528	18.69	8.41	2.62E-02	18.42	9.15	4.41E-02	15.07	54.33	7.81E-01	29.01	47.09	5.38E-01	18.55	26.60	4.86E-01
cg14118850	6.66	2.99	2.62E-02	5.45	3.91	1.63E-01	10.71	6.92	1.22E-01	9.54	9.58	3.19E-01	4.07	8.38	6.27E-01
cg01133655	-41.67	18.75	2.62E-02	-34.71	27.80	2.12E-01	9.93	42.63	8.16E-01	-94.73	42.18	2.47E-02	-52.55	39.06	1.79E-01
cg22904296	11.10	4.99	2.62E-02	8.39	6.85	2.21E-01	20.89	13.72	1.28E-01	25.78	16.36	1.15E-01	6.06	10.11	5.49E-01
cg13648651	13.15	5.91	2.62E-02	2.88	14.77	8.45E-01	27.06	19.77	1.71E-01	14.09	7.11	4.75E-02	8.80	24.39	7.18E-01
cg02705800	18.10	8.14	2.62E-02	-19.48	35.13	5.79E-01	13.31	42.34	7.53E-01	23.18	8.84	8.71E-03	-17.02	33.14	6.07E-01
cg02609127	17.04	7.67	2.62E-02	51.02	24.90	4.05E-02	29.18	29.02	3.15E-01	18.16	9.25	4.95E-02	2.81	12.63	8.24E-01
cg17099569	-9.58	4.31	2.62E-02	-10.55	6.96	1.30E-01	-8.98	11.82	4.47E-01	-3.57	4.42	4.19E-01	-24.29	9.32	9.15E-03
cg23732962	20.88	9.39	2.62E-02	15.40	17.78	3.86E-01	31.95	19.46	1.01E-01	13.46	16.28	4.08E-01	30.07	23.89	2.08E-01
cg16617723	10.18	4.58	2.62E-02	17.92	6.63	6.90E-03	4.69	10.06	6.41E-01	2.24	14.13	8.74E-01	2.05	9.95	8.37E-01
cg02069999	18.43	8.29	2.62E-02	15.16	8.69	8.10E-02	-6.40	67.64	9.25E-01	62.28	67.13	3.54E-01	63.96	34.17	6.13E-02
cg04272355	-10.71	4.82	2.63E-02	0.64	10.27	9.50E-01	-29.43	17.10	8.53E-02	-13.44	6.61	4.20E-02	-8.12	11.75	4.90E-01
cg24851249	-12.69	5.71	2.63E-02	-12.43	8.15	1.28E-01	-10.50	13.39	4.33E-01	-23.75	17.43	1.73E-01	-9.70	12.16	4.25E-01
cg17841267	-8.08	3.64	2.63E-02	-9.75	4.83	4.36E-02	-7.87	9.44	4.04E-01	-4.15	11.01	7.06E-01	-5.32	8.67	5.39E-01
cg25163297	21.73	9.78	2.63E-02	8.69	17.00	6.09E-01	37.42	18.50	4.32E-02	15.37	19.58	4.32E-01	32.60	26.11	2.12E-01

cg18077307	7.76	3.49	2.63E-02	7.20	4.70	1.25E-01	12.04	8.15	1.40E-01	7.01	11.77	5.51E-01	5.42	8.34	5.15E-01
cg02311496	17.49	7.87	2.63E-02	23.22	14.51	1.10E-01	8.89	18.08	6.23E-01	10.24	14.95	4.93E-01	25.70	16.12	1.11E-01
cg01310829	16.83	7.57	2.63E-02	20.34	15.76	1.97E-01	4.54	16.90	7.88E-01	20.79	14.11	1.41E-01	18.66	14.30	1.92E-01
cg16809293	-29.60	13.32	2.63E-02	-35.28	22.04	1.10E-01	20.35	43.78	6.42E-01	-18.35	25.90	4.79E-01	-49.46	25.26	5.03E-02
cg03999067	-7.47	3.36	2.63E-02	-6.14	6.43	3.40E-01	-10.97	11.27	3.30E-01	-9.15	4.70	5.17E-02	-1.09	9.48	9.08E-01
cg10257070	-13.15	5.92	2.63E-02	-16.21	11.63	1.64E-01	-27.75	20.53	1.76E-01	-13.56	7.96	8.86E-02	7.87	18.15	6.65E-01
cg10982364	15.48	6.97	2.63E-02	41.21	16.60	1.30E-02	0.28	22.78	9.90E-01	12.80	8.84	1.48E-01	8.18	14.84	5.81E-01
cg05844798	14.85	6.68	2.63E-02	19.20	10.40	6.49E-02	13.12	13.14	3.18E-01	18.67	17.49	2.86E-01	4.39	15.66	7.79E-01
cg13745852	38.12	17.16	2.63E-02	23.17	21.09	2.72E-01	6.15	113.29	9.57E-01	91.95	53.34	8.47E-02	62.00	37.28	9.62E-02
cg15225657	-32.68	14.71	2.63E-02	-6.18	28.56	8.29E-01	-28.60	135.67	8.33E-01	-45.51	17.89	1.10E-02	0.98	67.59	9.88E-01
cg13546414	12.69	5.71	2.63E-02	20.96	9.43	2.62E-02	1.21	14.09	9.31E-01	20.75	12.69	1.02E-01	2.24	11.08	8.40E-01
cg11581280	-11.31	5.09	2.63E-02	-13.63	9.81	1.65E-01	-8.17	15.35	5.95E-01	-8.64	7.23	2.32E-01	-19.65	14.39	1.72E-01
cg27131953	-38.83	17.48	2.63E-02	-12.56	26.74	6.39E-01	6.26	106.73	9.53E-01	-40.49	34.80	2.45E-01	-79.72	32.25	1.34E-02
cg04975834	21.25	9.56	2.63E-02	19.42	14.96	1.94E-01	23.03	20.95	2.72E-01	4.80	24.32	8.44E-01	34.03	20.01	8.90E-02
cg14572436	18.56	8.36	2.63E-02	15.31	15.11	3.11E-01	-2.98	27.83	9.15E-01	18.69	11.92	1.17E-01	44.01	24.86	7.67E-02
cg24477640	25.47	11.47	2.63E-02	25.78	20.82	2.16E-01	0.04	41.19	9.99E-01	25.81	16.22	1.12E-01	39.77	33.12	2.30E-01
cg13787134	18.12	8.16	2.63E-02	36.52	20.69	7.75E-02	53.88	37.21	1.48E-01	10.26	9.59	2.85E-01	33.50	30.30	2.69E-01
cg22529184	-9.29	4.18	2.63E-02	-11.09	9.55	2.46E-01	-2.33	20.43	9.09E-01	-7.06	5.22	1.76E-01	-20.42	11.87	8.55E-02
cg08992360	25.40	11.43	2.63E-02	26.85	15.79	8.91E-02	23.44	34.06	4.91E-01	54.16	28.76	5.97E-02	0.59	25.26	9.81E-01
cg13948456	20.32	9.14	2.63E-02	15.32	12.52	2.21E-01	16.42	19.52	4.00E-01	25.06	31.94	4.33E-01	39.23	22.48	8.09E-02
cg22932649	-11.72	5.28	2.63E-02	-9.52	11.07	3.90E-01	-24.76	20.88	2.36E-01	-9.65	6.92	1.63E-01	-18.63	14.80	2.08E-01
cg05672912	15.86	7.14	2.63E-02	11.41	9.44	2.27E-01	27.70	16.84	9.99E-02	56.60	30.22	6.11E-02	6.22	16.23	7.01E-01
cg02381898	20.95	9.43	2.63E-02	26.68	15.04	7.62E-02	8.06	23.85	7.35E-01	44.43	26.64	9.54E-02	11.18	16.54	4.99E-01
cg08452327	-25.57	11.51	2.63E-02	-16.80	13.93	2.28E-01	-78.83	41.70	5.87E-02	-49.53	63.30	4.34E-01	-31.05	25.26	2.19E-01
cg19643344	42.78	19.26	2.63E-02	99.26	60.81	1.03E-01	-177.25	223.49	4.28E-01	37.59	21.24	7.68E-02	46.11	72.69	5.26E-01
cg25062812	22.78	10.26	2.63E-02	-27.64	38.71	4.75E-01	-57.15	157.37	7.16E-01	25.60	10.94	1.93E-02	53.25	47.75	2.65E-01
cg13690525	21.92	9.87	2.63E-02	55.27	19.43	4.44E-03	30.07	30.70	3.27E-01	11.44	8.01	1.53E-01	13.58	16.50	4.10E-01
cg05730027	7.36	3.31	2.63E-02	7.37	4.68	1.15E-01	2.61	7.61	7.31E-01	1.09	12.95	9.33E-01	12.74	6.73	5.83E-02
cg23903774	-10.13	4.56	2.63E-02	-9.94	9.44	2.92E-01	-1.21	14.49	9.33E-01	-9.10	6.35	1.52E-01	-19.66	11.67	9.20E-02
cg16364805	-28.37	12.77	2.63E-02	-37.40	15.49	1.58E-02	95.27	79.75	2.32E-01	-28.45	39.61	4.73E-01	-13.63	28.67	6.35E-01
cg00075103	-8.72	3.93	2.63E-02	-15.12	8.16	6.39E-02	-8.16	13.62	5.49E-01	-4.78	5.48	3.83E-01	-12.09	9.44	2.00E-01
cg19978859	-15.78	7.11	2.63E-02	-32.87	18.11	6.95E-02	-13.74	27.98	6.23E-01	-14.46	8.59	9.22E-02	0.66	22.83	9.77E-01
cg07154956	29.90	13.46	2.64E-02	49.13	38.19	1.98E-01	-53.67	181.13	7.67E-01	25.62	14.96	8.67E-02	55.66	55.03	3.12E-01
cg00280895	8.83	3.98	2.64E-02	11.08	6.02	6.55E-02	5.91	10.07	5.57E-01	0.16	10.44	9.88E-01	11.62	7.77	1.35E-01
cg26065919	47.18	21.25	2.64E-02	21.40	18.41	2.45E-01	118.60	65.17	6.88E-02	129.92	60.38	3.14E-02	37.27	26.80	1.64E-01

cg15507449	-13.83	6.23	2.64E-02	-15.24	12.25	2.13E-01	-19.23	16.22	2.36E-01	-12.14	9.19	1.87E-01	-10.98	16.94	5.17E-01
cg02852529	-15.58	7.02	2.64E-02	-10.80	15.90	4.97E-01	-73.56	35.48	3.81E-02	-15.24	8.66	7.86E-02	-5.52	21.13	7.94E-01
cg13679691	14.55	6.55	2.64E-02	18.43	10.45	7.77E-02	47.60	24.57	5.27E-02	9.05	10.07	3.69E-01	0.71	19.63	9.71E-01
cg11814087	5.91	2.66	2.64E-02	4.15	3.52	2.38E-01	12.95	6.40	4.30E-02	6.50	6.98	3.51E-01	3.19	8.00	6.90E-01
cg23456595	-8.53	3.84	2.64E-02	-10.64	4.95	3.15E-02	-7.52	9.95	4.50E-01	-0.50	12.38	9.68E-01	-6.25	9.87	5.26E-01
cg27560132	-12.11	5.45	2.64E-02	-33.57	16.70	4.44E-02	-2.97	28.10	9.16E-01	-9.09	6.16	1.40E-01	-18.14	20.44	3.75E-01
cg05374428	-13.87	6.25	2.64E-02	-20.32	9.45	3.16E-02	-17.23	17.33	3.20E-01	-16.22	12.51	1.95E-01	6.93	14.54	6.34E-01
cg02150262	-10.71	4.82	2.64E-02	-11.46	8.14	1.59E-01	-15.46	12.29	2.08E-01	-4.66	9.50	6.24E-01	-13.08	9.90	1.86E-01
cg01023872	-15.87	7.15	2.64E-02	3.53	15.21	8.17E-01	-22.00	35.61	5.37E-01	-20.02	8.98	2.58E-02	-29.11	21.96	1.85E-01
cg04880940	15.22	6.85	2.64E-02	5.64	16.32	7.30E-01	16.54	24.58	5.01E-01	20.52	8.32	1.37E-02	-14.42	26.31	5.84E-01
cg05042439	-11.58	5.22	2.64E-02	-22.49	14.73	1.27E-01	-28.72	29.03	3.22E-01	-7.68	5.98	1.99E-01	-24.50	18.33	1.81E-01
cg12794432	-36.99	16.66	2.64E-02	-46.68	20.04	1.98E-02	37.45	84.85	6.59E-01	26.59	65.95	6.87E-01	-38.08	36.62	2.98E-01
cg13692476	9.48	4.27	2.64E-02	1.15	7.33	8.75E-01	21.09	9.85	3.22E-02	8.64	8.22	2.94E-01	13.75	9.47	1.47E-01
cg22184996	16.95	7.63	2.64E-02	31.42	17.10	6.61E-02	5.24	17.99	7.71E-01	14.10	14.93	3.45E-01	16.87	12.74	1.86E-01
cg04605667	25.76	11.60	2.64E-02	26.60	13.92	5.60E-02	34.50	45.59	4.49E-01	-17.65	60.21	7.69E-01	27.99	25.69	2.76E-01
cg00560632	5.11	2.30	2.64E-02	7.11	4.43	1.09E-01	-0.32	5.48	9.54E-01	6.70	3.62	6.40E-02	3.60	5.93	5.44E-01
cg27255275	7.00	3.16	2.64E-02	7.91	4.10	5.39E-02	12.86	8.04	1.10E-01	0.04	9.75	9.97E-01	2.29	8.15	7.79E-01
cg02844589	-12.89	5.81	2.64E-02	-25.13	7.87	1.40E-03	-8.64	9.43	3.60E-01	-4.42	16.67	7.91E-01	-2.43	10.86	8.23E-01
cg12542255	4.64	2.09	2.64E-02	3.45	3.45	3.17E-01	7.27	3.74	5.21E-02	3.48	6.11	5.68E-01	3.41	4.61	4.60E-01
cg09740450	-7.72	3.48	2.64E-02	-8.99	5.03	7.41E-02	-12.17	7.86	1.22E-01	-5.97	10.21	5.59E-01	-1.69	7.56	8.23E-01
cg20222562	5.14	2.32	2.64E-02	4.82	3.18	1.29E-01	6.98	6.42	2.77E-01	8.62	5.12	9.19E-02	-0.74	6.36	9.07E-01
cg08732750	9.05	4.08	2.64E-02	12.74	5.75	2.67E-02	2.43	8.76	7.82E-01	16.37	14.27	2.51E-01	3.92	9.15	6.68E-01
cg25012185	10.93	4.93	2.64E-02	18.25	8.03	2.30E-02	13.82	10.95	2.07E-01	3.42	11.35	7.63E-01	2.68	10.20	7.93E-01
cg02610064	-13.43	6.05	2.64E-02	-12.99	9.46	1.70E-01	-22.03	15.11	1.45E-01	-23.69	13.33	7.56E-02	1.31	12.76	9.18E-01
cg19740361	13.52	6.09	2.64E-02	6.36	12.21	6.03E-01	-27.07	32.04	3.98E-01	17.10	7.65	2.54E-02	25.71	21.48	2.31E-01
cg03510732	-13.24	5.97	2.65E-02	-28.06	9.33	2.64E-03	-1.97	13.67	8.86E-01	-10.02	8.70	2.50E-01	-4.98	12.51	6.90E-01
cg10376161	6.37	2.87	2.65E-02	4.76	3.62	1.89E-01	13.37	7.45	7.27E-02	6.89	8.74	4.31E-01	5.67	8.48	5.03E-01
cg22688566	7.19	3.24	2.65E-02	6.18	4.37	1.57E-01	18.51	9.07	4.13E-02	8.02	7.31	2.73E-01	-1.18	9.14	8.98E-01
cg13120771	-40.38	18.20	2.65E-02	-48.60	21.88	2.63E-02	-54.03	63.33	3.94E-01	-23.98	64.30	7.09E-01	-2.62	47.70	9.56E-01
cg14628354	17.46	7.87	2.65E-02	2.01	22.52	9.29E-01	35.08	53.33	5.11E-01	18.59	8.85	3.58E-02	26.68	30.51	3.82E-01
cg09122158	19.79	8.92	2.65E-02	25.07	10.09	1.29E-02	-46.27	73.59	5.29E-01	38.79	62.37	5.34E-01	0.48	20.81	9.82E-01
cg21182715	-12.13	5.47	2.65E-02	-28.64	12.37	2.06E-02	-5.03	14.10	7.21E-01	-8.30	7.71	2.82E-01	-10.64	14.04	4.49E-01
cg16782493	23.75	10.70	2.65E-02	26.75	20.12	1.84E-01	58.04	40.55	1.52E-01	10.54	10.48	3.15E-01	56.74	27.23	3.72E-02
cg02390637	11.85	5.34	2.65E-02	12.78	6.95	6.59E-02	12.00	12.56	3.40E-01	13.15	18.57	4.79E-01	7.17	13.92	6.07E-01
cg09999441	-35.43	15.96	2.65E-02	-19.16	29.79	5.20E-01	75.99	212.57	7.21E-01	-54.51	20.74	8.58E-03	16.85	47.09	7.21E-01

cg15612947	6.83	3.08	2.65E-02	5.62	4.10	1.71E-01	12.12	8.45	1.51E-01	10.96	8.64	2.04E-01	3.77	7.32	6.07E-01
cg25948982	9.95	4.49	2.65E-02	11.02	6.41	8.54E-02	16.60	13.35	2.14E-01	9.25	9.93	3.52E-01	4.10	10.21	6.88E-01
cg21927420	-22.46	10.12	2.65E-02	-32.68	19.39	9.19E-02	-34.60	43.35	4.25E-01	-16.78	13.69	2.20E-01	-19.74	28.44	4.88E-01
cg06087962	-25.75	11.60	2.65E-02	-71.54	41.44	8.43E-02	-167.53	159.11	2.92E-01	-18.44	12.52	1.41E-01	-59.83	48.66	2.19E-01
cg08404225	-8.41	3.79	2.65E-02	-18.25	8.78	3.77E-02	-4.86	12.99	7.08E-01	-3.91	4.36	3.70E-01	-17.42	9.84	7.67E-02
cg24690709	13.06	5.89	2.65E-02	9.90	7.33	1.77E-01	13.61	16.38	4.06E-01	14.10	27.43	6.07E-01	23.73	13.87	8.71E-02
cg17764008	-35.88	16.17	2.65E-02	-85.53	66.21	1.96E-01	-188.70	219.29	3.90E-01	-34.83	17.37	4.49E-02	6.33	61.89	9.19E-01
cg23196831	11.25	5.07	2.65E-02	20.54	8.30	1.34E-02	9.55	15.47	5.37E-01	12.49	8.81	1.56E-01	-2.39	9.88	8.09E-01
cg05475107	-8.49	3.83	2.65E-02	-10.63	8.12	1.91E-01	-9.14	14.46	5.28E-01	-6.80	4.98	1.72E-01	-12.65	11.22	2.60E-01
cg10911054	-16.65	7.50	2.65E-02	-21.97	11.51	5.64E-02	0.90	22.25	9.68E-01	-31.67	20.24	1.18E-01	-9.47	13.18	4.72E-01
cg13382322	20.23	9.12	2.65E-02	52.84	33.68	1.17E-01	23.81	63.15	7.06E-01	14.60	9.90	1.40E-01	60.32	37.93	1.12E-01
cg13424673	18.39	8.29	2.65E-02	27.79	14.76	5.98E-02	0.89	22.09	9.68E-01	33.30	18.59	7.32E-02	8.36	14.11	5.54E-01
cg17519696	-6.50	2.93	2.65E-02	-3.98	4.10	3.31E-01	-10.44	6.00	8.19E-02	-11.82	11.21	2.92E-01	-6.40	6.85	3.50E-01
cg02234281	-9.49	4.28	2.65E-02	-10.09	6.08	9.71E-02	-8.68	10.98	4.29E-01	0.99	12.74	9.38E-01	-13.68	8.72	1.17E-01
cg19165640	9.28	4.18	2.65E-02	10.64	6.00	7.63E-02	9.29	11.31	4.11E-01	5.80	13.40	6.65E-01	8.12	7.89	3.04E-01
cg21339084	18.29	8.24	2.65E-02	17.08	14.22	2.30E-01	10.11	15.43	5.12E-01	41.51	18.23	2.28E-02	6.76	19.77	7.33E-01
cg11076902	-11.86	5.35	2.65E-02	-11.51	9.08	2.05E-01	-25.26	11.50	2.81E-02	-0.67	11.58	9.54E-01	-10.13	11.29	3.70E-01
cg18387659	-9.84	4.43	2.65E-02	-11.44	9.96	2.51E-01	-2.63	13.52	8.46E-01	-8.32	5.84	1.55E-01	-21.13	12.90	1.01E-01
cg23931796	-13.05	5.88	2.65E-02	-21.05	9.11	2.09E-02	-12.60	13.09	3.36E-01	-2.18	16.79	8.97E-01	-5.69	11.56	6.23E-01
cg21395723	15.68	7.07	2.65E-02	32.18	17.22	6.17E-02	-5.24	38.98	8.93E-01	12.97	8.32	1.19E-01	13.84	25.41	5.86E-01
cg21616626	23.31	10.51	2.65E-02	36.35	18.85	5.39E-02	2.42	24.79	9.22E-01	18.49	17.68	2.96E-01	32.26	26.53	2.24E-01
cg14210811	24.02	10.83	2.65E-02	47.40	21.80	2.97E-02	28.03	27.34	3.05E-01	7.55	15.99	6.37E-01	32.43	29.16	2.66E-01
cg17196805	16.65	7.51	2.65E-02	21.33	21.39	3.19E-01	29.90	25.52	2.41E-01	10.51	8.82	2.33E-01	57.75	29.16	4.77E-02
cg24630825	-7.74	3.49	2.65E-02	-17.19	7.28	1.82E-02	1.88	9.02	8.35E-01	-7.24	5.01	1.48E-01	-4.13	9.50	6.64E-01
cg03422757	10.32	4.65	2.65E-02	10.29	8.96	2.51E-01	1.90	17.49	9.14E-01	9.24	6.17	1.34E-01	23.83	15.45	1.23E-01
cg25553665	24.63	11.10	2.65E-02	39.75	16.74	1.76E-02	36.62	40.41	3.65E-01	28.61	30.81	3.53E-01	1.89	18.63	9.19E-01
cg24113950	-8.76	3.95	2.65E-02	-4.23	5.08	4.06E-01	-7.87	9.03	3.84E-01	-3.88	13.82	7.79E-01	-20.53	7.73	7.90E-03
cg18943599	-29.94	13.50	2.65E-02	-18.64	20.34	3.59E-01	-65.66	36.82	7.45E-02	-49.03	41.41	2.36E-01	-24.13	23.90	3.13E-01
cg22544679	18.44	8.31	2.65E-02	39.77	21.30	6.19E-02	-1.67	26.83	9.50E-01	15.29	10.18	1.33E-01	27.68	28.58	3.33E-01
cg21904251	19.91	8.98	2.65E-02	38.45	18.45	3.72E-02	29.77	19.98	1.36E-01	10.61	13.39	4.28E-01	0.33	26.80	9.90E-01
cg12072833	-13.65	6.15	2.65E-02	-19.93	9.54	3.66E-02	-9.76	13.27	4.62E-01	-4.25	16.54	7.97E-01	-11.57	12.84	3.67E-01
cg12626076	-9.09	4.10	2.65E-02	-7.44	5.47	1.74E-01	-22.07	10.35	3.29E-02	2.63	12.13	8.29E-01	-10.46	10.01	2.96E-01
cg00919994	45.15	20.36	2.66E-02	121.51	46.81	9.44E-03	21.01	202.29	9.17E-01	30.70	18.56	9.81E-02	24.98	52.37	6.33E-01
cg13521002	12.36	5.57	2.66E-02	21.00	9.64	2.94E-02	-1.60	10.54	8.79E-01	11.21	10.60	2.91E-01	23.18	15.87	1.44E-01
cg01153660	-15.19	6.85	2.66E-02	-30.04	36.19	4.06E-01	39.89	133.04	7.64E-01	-14.60	7.13	4.05E-02	-19.26	34.99	5.82E-01

cg12809489	20.21	9.11	2.66E-02	17.61	17.57	3.16E-01	24.49	16.45	1.37E-01	14.69	17.70	4.06E-01	25.52	22.84	2.64E-01
cg23221737	-17.72	7.99	2.66E-02	-38.28	16.18	1.80E-02	-8.89	16.46	5.89E-01	1.28	19.33	9.47E-01	-18.60	13.51	1.68E-01
cg11525409	6.13	2.76	2.66E-02	5.21	3.52	1.39E-01	13.62	7.53	7.07E-02	4.39	7.87	5.77E-01	4.34	7.78	5.77E-01
cg00967854	22.84	10.30	2.66E-02	55.15	19.70	5.12E-03	13.31	26.59	6.17E-01	18.43	8.85	3.74E-02	-4.46	28.26	8.75E-01
cg18297538	18.17	8.19	2.66E-02	12.79	26.97	6.35E-01	-4.95	87.72	9.55E-01	21.86	9.29	1.87E-02	0.36	23.49	9.88E-01
cg19725489	9.89	4.46	2.66E-02	10.26	5.75	7.44E-02	20.59	15.70	1.90E-01	20.36	15.78	1.97E-01	1.81	9.15	8.43E-01
cg16520357	5.33	2.41	2.66E-02	5.09	3.65	1.63E-01	12.03	5.93	4.23E-02	2.57	4.43	5.61E-01	3.62	7.39	6.24E-01
cg13940029	-13.73	6.19	2.66E-02	-18.22	12.41	1.42E-01	-7.91	17.89	6.58E-01	-11.32	8.86	2.01E-01	-19.01	16.38	2.46E-01
cg12552033	30.92	13.94	2.66E-02	1.03	36.67	9.77E-01	-38.80	177.35	8.27E-01	40.33	15.88	1.11E-02	-1.26	49.98	9.80E-01
cg14240490	18.80	8.48	2.66E-02	24.96	18.61	1.80E-01	-13.74	25.02	5.83E-01	26.28	11.47	2.19E-02	6.38	23.41	7.85E-01
cg21773162	-12.21	5.51	2.66E-02	-6.06	7.55	4.22E-01	-23.91	14.72	1.04E-01	-11.24	13.47	4.04E-01	-23.26	13.68	8.92E-02
cg12025832	16.69	7.52	2.66E-02	-6.03	21.53	7.80E-01	-3.40	30.86	9.12E-01	19.05	8.94	3.31E-02	37.53	22.70	9.82E-02
cg07017901	29.00	13.08	2.66E-02	28.14	27.02	2.98E-01	28.08	46.05	5.42E-01	22.32	17.48	2.02E-01	60.95	36.90	9.86E-02
cg23851515	-8.80	3.97	2.66E-02	-11.56	8.28	1.63E-01	10.83	19.48	5.78E-01	-9.79	4.85	4.36E-02	-0.76	16.16	9.63E-01
cg09749844	7.71	3.48	2.66E-02	11.57	5.96	5.22E-02	11.66	7.91	1.40E-01	2.76	5.86	6.38E-01	4.79	10.29	6.42E-01
cg10516359	19.01	8.57	2.66E-02	28.39	13.77	3.92E-02	17.41	16.88	3.02E-01	13.27	19.23	4.90E-01	5.61	21.75	7.97E-01
cg11741753	23.31	10.51	2.66E-02	33.14	17.08	5.24E-02	20.84	21.85	3.40E-01	19.60	20.50	3.39E-01	6.15	29.52	8.35E-01
cg08893692	18.27	8.24	2.66E-02	16.91	25.96	5.15E-01	103.32	84.27	2.20E-01	19.74	9.30	3.38E-02	0.82	25.47	9.74E-01
cg05177729	18.53	8.36	2.66E-02	11.95	14.87	4.22E-01	13.45	16.68	4.20E-01	19.78	17.57	2.60E-01	33.33	18.35	6.94E-02
cg08371772	19.34	8.72	2.66E-02	51.75	31.38	9.91E-02	-36.09	135.64	7.90E-01	18.63	9.38	4.69E-02	-11.67	37.63	7.56E-01
cg12549211	-17.45	7.87	2.66E-02	-41.75	24.14	8.37E-02	-9.27	36.06	7.97E-01	-14.58	8.82	9.82E-02	-19.19	35.23	5.86E-01
cg21745307	-20.77	9.37	2.66E-02	-22.02	13.13	9.34E-02	6.51	36.23	8.57E-01	-33.67	25.43	1.85E-01	-18.80	17.45	2.81E-01
cg16781907	-15.61	7.04	2.66E-02	-16.41	11.79	1.64E-01	-28.05	15.21	6.53E-02	-22.55	12.20	6.45E-02	7.18	14.79	6.28E-01
cg04855961	-13.10	5.91	2.66E-02	-4.13	12.84	7.48E-01	-17.04	15.97	2.86E-01	-17.82	8.29	3.15E-02	-5.84	15.61	7.09E-01
cg09595050	6.38	2.88	2.66E-02	4.51	4.30	2.94E-01	3.51	4.76	4.62E-01	17.20	9.51	7.05E-02	15.59	9.26	9.23E-02
cg08895021	-42.95	19.37	2.66E-02	-35.28	24.30	1.47E-01	-109.24	69.18	1.14E-01	24.83	81.64	7.61E-01	-58.16	40.42	1.50E-01
cg00591949	-12.69	5.72	2.66E-02	-12.00	9.45	2.04E-01	-21.85	12.52	8.08E-02	-14.94	14.60	3.06E-01	-5.25	11.01	6.34E-01
cg26186727	12.98	5.86	2.66E-02	26.18	10.60	1.35E-02	4.64	16.84	7.83E-01	8.24	10.77	4.44E-01	7.15	11.11	5.20E-01
cg22304262	-10.59	4.78	2.66E-02	-10.48	7.20	1.45E-01	-11.33	9.60	2.38E-01	-3.24	14.29	8.20E-01	-14.06	10.71	1.90E-01
cg19510075	34.61	15.61	2.66E-02	92.11	42.83	3.15E-02	-21.55	193.02	9.11E-01	29.86	17.71	9.18E-02	-8.17	53.93	8.80E-01
cg08000413	17.15	7.73	2.66E-02	36.64	14.74	1.29E-02	-7.50	22.57	7.40E-01	16.96	8.66	5.02E-02	1.41	22.04	9.49E-01
cg07546293	-10.76	4.86	2.66E-02	-10.90	7.17	1.28E-01	-0.55	10.55	9.59E-01	-8.21	13.30	5.37E-01	-23.19	10.96	3.43E-02
cg13561792	17.09	7.71	2.66E-02	-1.25	22.56	9.56E-01	36.86	20.88	7.75E-02	15.06	9.48	1.12E-01	26.35	26.45	3.19E-01
cg20334627	35.07	15.82	2.66E-02	9.29	38.80	8.11E-01	-10.79	46.20	8.15E-01	42.37	19.68	3.13E-02	105.66	59.75	7.70E-02
cg18993778	8.18	3.69	2.67E-02	15.90	4.77	8.46E-04	7.86	6.00	1.90E-01	1.28	4.69	7.85E-01	6.95	9.74	4.75E-01

cg05693204	-10.74	4.85	2.67E-02	-12.52	6.79	6.54E-02	-9.21	11.65	4.29E-01	-19.12	14.71	1.94E-01	-3.35	10.59	7.51E-01
cg21899992	17.04	7.69	2.67E-02	10.66	18.06	5.55E-01	17.31	17.56	3.24E-01	5.44	17.96	7.62E-01	24.32	11.54	3.51E-02
cg23267759	10.67	4.81	2.67E-02	29.11	12.85	2.35E-02	6.05	14.46	6.76E-01	7.58	7.89	3.37E-01	8.22	7.85	2.95E-01
cg01997606	-6.54	2.95	2.67E-02	-5.85	5.76	3.09E-01	-1.84	9.09	8.39E-01	-5.52	4.41	2.11E-01	-12.76	6.90	6.45E-02
cg22177356	-38.90	17.55	2.67E-02	-97.25	40.01	1.51E-02	-58.67	162.41	7.18E-01	-28.65	21.30	1.79E-01	-0.53	51.24	9.92E-01
cg11838876	-12.54	5.66	2.67E-02	-2.50	9.66	7.96E-01	-20.03	14.30	1.61E-01	-25.68	9.94	9.77E-03	-6.56	9.42	4.86E-01
cg17983064	9.16	4.13	2.67E-02	11.19	5.68	4.89E-02	-0.04	10.03	9.97E-01	16.30	10.65	1.26E-01	5.24	10.63	6.22E-01
cg08822023	-19.50	8.80	2.67E-02	-34.70	15.55	2.57E-02	-11.97	15.94	4.53E-01	-19.19	19.81	3.33E-01	-5.39	20.87	7.96E-01
cg06588802	-7.78	3.51	2.67E-02	-6.54	4.51	1.46E-01	-16.30	9.43	8.37E-02	1.12	12.10	9.26E-01	-9.65	8.52	2.57E-01
cg25711246	-10.03	4.53	2.67E-02	-9.54	6.68	1.53E-01	-16.37	10.20	1.09E-01	-1.87	11.56	8.71E-01	-11.21	10.36	2.79E-01
cg18668679	16.04	7.24	2.67E-02	3.71	19.55	8.49E-01	13.43	17.51	4.43E-01	16.50	9.33	7.72E-02	36.68	24.09	1.28E-01
cg12649238	5.16	2.33	2.67E-02	4.54	2.99	1.29E-01	9.65	5.90	1.02E-01	5.95	6.01	3.23E-01	0.10	7.86	9.90E-01
cg02066331	7.87	3.55	2.67E-02	4.38	5.51	4.27E-01	9.51	7.51	2.05E-01	18.01	8.32	3.04E-02	3.58	8.42	6.71E-01
cg22637538	15.71	7.09	2.67E-02	24.48	15.19	1.07E-01	4.27	31.02	8.90E-01	14.75	10.13	1.45E-01	12.21	14.48	3.99E-01
cg06385202	-8.98	4.05	2.67E-02	-13.81	6.11	2.39E-02	2.04	8.46	8.09E-01	-11.28	11.57	3.29E-01	-9.59	8.89	2.81E-01
cg02763617	-17.16	7.75	2.67E-02	-20.60	11.44	7.16E-02	-18.11	15.89	2.54E-01	9.50	21.92	6.65E-01	-25.69	18.31	1.61E-01
cg13866093	8.70	3.93	2.67E-02	6.06	6.05	3.16E-01	15.14	8.45	7.31E-02	8.60	11.10	4.38E-01	7.57	8.05	3.47E-01
cg18108008	23.08	10.42	2.67E-02	-1.29	19.61	9.47E-01	50.42	43.09	2.42E-01	35.78	14.91	1.64E-02	17.69	25.20	4.83E-01
cg05412028	10.34	4.66	2.67E-02	12.52	10.98	2.54E-01	35.18	23.77	1.39E-01	10.47	5.64	6.33E-02	-4.50	14.94	7.63E-01
cg07099073	-10.57	4.77	2.67E-02	-12.62	11.34	2.66E-01	5.07	24.37	8.35E-01	-11.23	5.96	5.93E-02	-9.24	12.60	4.63E-01
cg08086720	15.42	6.96	2.67E-02	32.68	17.18	5.71E-02	14.96	23.38	5.22E-01	13.22	9.04	1.43E-01	5.78	17.70	7.44E-01
cg08615694	16.44	7.42	2.67E-02	13.45	10.49	2.00E-01	28.03	17.82	1.16E-01	0.39	28.13	9.89E-01	18.78	14.64	1.99E-01
cg23109041	16.24	7.33	2.67E-02	33.87	20.02	9.07E-02	31.15	29.79	2.96E-01	10.14	8.48	2.32E-01	37.84	30.04	2.08E-01
cg23852348	-8.60	3.88	2.67E-02	-7.07	7.00	3.12E-01	-9.77	12.82	4.46E-01	-7.13	5.61	2.04E-01	-17.27	11.08	1.19E-01
cg00487142	5.14	2.32	2.67E-02	5.71	3.80	1.33E-01	2.53	4.68	5.88E-01	2.99	4.46	5.03E-01	14.10	6.92	4.15E-02
cg20425130	27.76	12.53	2.67E-02	29.62	14.10	3.57E-02	-20.17	70.67	7.75E-01	76.79	43.48	7.74E-02	-14.36	40.53	7.23E-01
cg23474890	-6.08	2.75	2.67E-02	-3.80	4.03	3.46E-01	-9.16	7.77	2.38E-01	-6.99	5.58	2.10E-01	-8.81	6.69	1.88E-01
cg13461622	-7.54	3.40	2.67E-02	-7.20	4.08	7.72E-02	-18.90	8.00	1.82E-02	3.10	9.06	7.32E-01	-6.06	7.02	3.88E-01
cg02625925	17.50	7.90	2.67E-02	22.02	12.42	7.62E-02	18.25	13.69	1.83E-01	10.50	22.27	6.37E-01	8.74	21.30	6.81E-01
cg14991487	19.68	8.88	2.67E-02	21.64	14.17	1.27E-01	12.94	25.41	6.11E-01	36.99	23.09	1.09E-01	12.23	15.31	4.24E-01
cg05567646	-8.05	3.63	2.67E-02	-10.23	5.52	6.40E-02	-14.56	7.33	4.71E-02	5.66	9.34	5.45E-01	-6.31	7.57	4.05E-01
cg25677288	12.30	5.55	2.67E-02	13.78	12.59	2.74E-01	21.16	30.70	4.91E-01	7.93	6.95	2.54E-01	28.94	15.20	5.70E-02
cg06621060	-55.65	25.12	2.67E-02	-25.22	35.04	4.72E-01	-80.10	128.82	5.34E-01	-42.20	100.39	6.74E-01	-95.99	40.46	1.77E-02
cg01505109	17.95	8.10	2.67E-02	1.55	16.49	9.25E-01	25.46	14.55	8.01E-02	18.10	14.39	2.09E-01	29.92	22.31	1.80E-01
cg23352424	-14.49	6.54	2.67E-02	-25.47	11.34	2.47E-02	-3.86	14.47	7.89E-01	-3.65	12.71	7.74E-01	-21.50	14.69	1.43E-01

cg15387785	29.70	13.41	2.67E-02	28.11	17.00	9.83E-02	-6.09	50.81	9.05E-01	21.92	35.92	5.42E-01	56.68	32.59	8.20E-02
cg03539765	6.06	2.73	2.67E-02	7.34	4.57	1.08E-01	6.77	5.77	2.40E-01	8.33	6.11	1.73E-01	1.12	5.86	8.49E-01
cg23367076	15.61	7.04	2.67E-02	36.88	14.54	1.12E-02	-0.75	15.04	9.60E-01	14.47	9.79	1.40E-01	11.36	15.82	4.73E-01
cg13148650	-10.28	4.64	2.67E-02	-14.91	10.00	1.36E-01	-25.40	15.08	9.22E-02	-7.03	6.10	2.49E-01	-5.38	13.86	6.98E-01
cg10827479	-7.68	3.47	2.67E-02	-15.26	6.65	2.17E-02	-6.88	6.53	2.92E-01	-3.40	5.96	5.68E-01	-4.07	10.55	7.00E-01
cg01703329	10.91	4.92	2.67E-02	10.61	6.70	1.13E-01	25.29	12.36	4.08E-02	7.31	12.13	5.47E-01	-0.33	13.35	9.80E-01
cg02712165	13.93	6.29	2.67E-02	6.98	10.85	5.20E-01	14.16	11.36	2.13E-01	16.11	15.46	2.97E-01	23.81	14.33	9.66E-02
cg17187521	8.26	3.73	2.68E-02	9.45	4.93	5.52E-02	17.25	9.30	6.37E-02	3.11	12.15	7.98E-01	-1.21	8.96	8.93E-01
cg02659854	8.24	3.72	2.68E-02	7.75	4.81	1.07E-01	16.37	10.56	1.21E-01	5.93	10.44	5.70E-01	5.44	9.56	5.69E-01
cg05051734	-21.60	9.75	2.68E-02	-15.49	18.72	4.08E-01	-47.36	32.97	1.51E-01	-21.31	13.50	1.14E-01	-17.90	28.24	5.26E-01
cg01576260	-12.46	5.63	2.68E-02	-13.72	9.89	1.65E-01	-13.96	11.62	2.30E-01	-8.99	10.60	3.96E-01	-13.84	14.07	3.25E-01
cg25646029	13.92	6.28	2.68E-02	19.16	9.16	3.64E-02	16.65	14.22	2.42E-01	-8.12	19.16	6.72E-01	11.12	13.20	3.99E-01
cg00116092	17.26	7.79	2.68E-02	28.21	12.18	2.05E-02	13.01	18.99	4.93E-01	11.28	26.18	6.66E-01	7.55	13.49	5.76E-01
cg10643578	36.37	16.42	2.68E-02	81.44	52.40	1.20E-01	95.13	230.80	6.80E-01	30.62	18.67	1.01E-01	34.10	46.65	4.65E-01
cg20794855	6.60	2.98	2.68E-02	7.93	3.83	3.83E-02	11.48	8.26	1.65E-01	5.35	8.80	5.43E-01	-2.05	7.68	7.90E-01
cg20319698	9.61	4.34	2.68E-02	15.36	6.40	1.64E-02	-1.32	10.24	8.97E-01	7.29	8.52	3.92E-01	8.82	13.55	5.15E-01
cg01896807	-8.98	4.05	2.68E-02	-11.94	5.62	3.35E-02	5.22	9.56	5.85E-01	-18.56	13.13	1.58E-01	-9.50	8.75	2.77E-01
cg00342313	-18.41	8.31	2.68E-02	-7.10	17.46	6.84E-01	-21.29	25.79	4.09E-01	-15.01	11.23	1.81E-01	-52.39	23.85	2.80E-02
cg16026088	20.55	9.28	2.68E-02	26.20	11.53	2.30E-02	-8.13	30.22	7.88E-01	-63.19	64.38	3.26E-01	23.85	19.06	2.11E-01
cg24202817	16.96	7.66	2.68E-02	18.64	10.54	7.70E-02	15.37	19.17	4.23E-01	16.40	23.73	4.90E-01	14.22	16.78	3.97E-01
cg15984516	17.91	8.09	2.68E-02	19.66	11.32	8.25E-02	5.43	17.47	7.56E-01	46.19	30.67	1.32E-01	17.01	17.81	3.39E-01
cg10284006	-12.18	5.50	2.68E-02	-10.08	9.44	2.86E-01	-8.46	12.29	4.91E-01	-17.93	12.77	1.60E-01	-13.59	10.48	1.95E-01
cg26950624	25.51	11.52	2.68E-02	59.33	17.62	7.59E-04	13.04	22.37	5.60E-01	15.30	7.91	5.32E-02	14.48	28.21	6.08E-01
cg24263283	-11.33	5.12	2.68E-02	-18.25	10.89	9.38E-02	-9.74	14.56	5.03E-01	-9.06	7.45	2.24E-01	-9.93	11.94	4.06E-01
cg15644413	7.05	3.18	2.68E-02	6.67	4.38	1.28E-01	15.80	8.49	6.27E-02	9.64	7.20	1.81E-01	-4.33	8.67	6.17E-01
cg03352975	-25.10	11.33	2.68E-02	-27.31	17.78	1.25E-01	-25.81	56.86	6.50E-01	-46.61	53.70	3.85E-01	-21.41	15.88	1.78E-01
cg22081879	-7.64	3.45	2.68E-02	-3.32	6.06	5.83E-01	-2.54	8.00	7.51E-01	-11.73	5.99	5.01E-02	-13.99	8.72	1.09E-01
cg10503473	-48.89	22.08	2.68E-02	-63.12	34.64	6.85E-02	96.40	98.29	3.27E-01	-36.78	40.92	3.69E-01	-68.99	43.94	1.16E-01
cg00704780	9.63	4.35	2.68E-02	10.04	6.72	1.35E-01	16.44	11.05	1.37E-01	13.53	7.63	7.61E-02	-8.97	11.94	4.52E-01
cg02258112	19.00	8.58	2.68E-02	1.23	18.87	9.48E-01	36.60	48.91	4.54E-01	22.63	10.43	3.00E-02	26.91	29.38	3.60E-01
cg12209042	37.42	16.90	2.68E-02	35.25	24.32	1.47E-01	39.09	67.35	5.62E-01	15.79	39.60	6.90E-01	55.34	32.39	8.75E-02
cg02574484	16.91	7.64	2.68E-02	-0.39	16.27	9.81E-01	19.60	17.42	2.61E-01	20.63	11.50	7.28E-02	28.28	19.99	1.57E-01
cg02358477	-29.90	13.50	2.68E-02	-40.58	15.40	8.43E-03	-9.26	66.24	8.89E-01	28.96	59.79	6.28E-01	1.38	36.22	9.70E-01
cg10416668	-13.45	6.08	2.68E-02	-14.41	10.72	1.79E-01	-8.33	14.53	5.67E-01	-13.47	12.29	2.73E-01	-15.71	11.94	1.88E-01
cg17106073	8.90	4.02	2.68E-02	13.83	8.84	1.18E-01	-3.02	18.46	8.70E-01	8.56	4.87	7.85E-02	5.36	15.94	7.37E-01

cg27450668	-18.37	8.30	2.68E-02	-31.75	15.31	3.82E-02	0.54	33.49	9.87E-01	-20.17	15.61	1.96E-01	-9.33	13.78	4.98E-01
cg23222057	-14.74	6.66	2.68E-02	-21.83	10.63	4.01E-02	-13.84	18.38	4.51E-01	-17.83	15.99	2.65E-01	-4.20	12.09	7.28E-01
cg18118033	42.26	19.09	2.68E-02	58.59	23.82	1.39E-02	38.29	65.17	5.57E-01	31.99	59.70	5.92E-01	-11.36	46.32	8.06E-01
cg08464402	-13.46	6.08	2.68E-02	-19.67	11.33	8.25E-02	-5.68	14.59	6.97E-01	-6.31	5.36	2.40E-01	-33.60	13.43	1.23E-02
cg23133255	-10.66	4.82	2.68E-02	-8.99	7.23	2.14E-01	-17.90	14.49	2.17E-01	-6.03	10.20	5.54E-01	-15.07	10.22	1.40E-01
cg21536585	37.19	16.80	2.68E-02	31.39	21.29	1.40E-01	3.46	57.39	9.52E-01	-11.81	58.80	8.41E-01	87.10	36.63	1.74E-02
cg00249032	17.02	7.69	2.68E-02	29.73	18.21	1.03E-01	55.19	38.28	1.49E-01	9.80	9.21	2.87E-01	31.56	26.47	2.33E-01
cg07283795	-10.65	4.81	2.68E-02	-17.50	10.19	8.59E-02	-2.55	14.18	8.57E-01	-8.62	7.10	2.25E-01	-12.36	10.71	2.49E-01
cg19371339	-6.95	3.14	2.68E-02	-9.02	6.49	1.65E-01	-17.97	9.20	5.08E-02	-2.96	4.49	5.10E-01	-8.10	7.83	3.01E-01
cg07169834	21.44	9.69	2.69E-02	24.18	10.94	2.71E-02	19.73	39.04	6.13E-01	-36.99	59.43	5.34E-01	17.60	27.11	5.16E-01
cg02674384	11.15	5.04	2.69E-02	7.34	9.35	4.32E-01	29.03	20.96	1.66E-01	11.34	8.58	1.86E-01	11.19	9.09	2.18E-01
cg24051487	-25.42	11.49	2.69E-02	-42.97	14.13	2.36E-03	-40.31	27.54	1.43E-01	-6.68	5.94	2.61E-01	-30.39	19.15	1.13E-01
cg02053477	-10.14	4.58	2.69E-02	-12.18	7.86	1.21E-01	1.27	10.35	9.03E-01	-18.18	9.02	4.38E-02	-7.56	10.08	4.53E-01
cg06131338	26.18	11.83	2.69E-02	11.73	22.25	5.98E-01	68.88	52.08	1.86E-01	28.63	15.75	6.91E-02	31.02	36.96	4.01E-01
cg12476455	-8.10	3.66	2.69E-02	-6.52	6.18	2.92E-01	-9.43	9.91	3.42E-01	-4.67	6.26	4.55E-01	-17.14	8.85	5.27E-02
cg21975834	6.90	3.12	2.69E-02	9.28	4.24	2.85E-02	5.01	7.07	4.79E-01	7.38	8.95	4.10E-01	0.02	8.28	9.98E-01
cg20522483	16.88	7.63	2.69E-02	22.96	14.96	1.25E-01	-0.81	27.55	9.77E-01	12.20	10.23	2.33E-01	39.10	23.28	9.31E-02
cg14797990	-9.53	4.30	2.69E-02	-8.29	8.02	3.01E-01	-10.28	8.81	2.43E-01	-5.32	7.69	4.89E-01	-18.86	10.76	7.97E-02
cg05475667	22.41	10.12	2.69E-02	37.93	16.57	2.20E-02	23.96	28.00	3.92E-01	16.42	31.66	6.04E-01	8.72	16.13	5.89E-01
cg03376481	-18.38	8.30	2.69E-02	-41.06	13.08	1.70E-03	2.38	23.66	9.20E-01	-12.36	6.73	6.62E-02	-13.52	18.85	4.73E-01
cg02628801	-34.28	15.49	2.69E-02	-19.22	24.16	4.26E-01	-93.15	218.12	6.69E-01	-54.56	22.29	1.44E-02	4.21	48.68	9.31E-01
cg13003513	15.80	7.14	2.69E-02	8.63	13.76	5.30E-01	17.75	17.20	3.02E-01	17.63	10.52	9.39E-02	23.52	22.82	3.03E-01
cg11789534	6.44	2.91	2.69E-02	4.63	3.79	2.22E-01	11.61	6.60	7.83E-02	13.07	9.61	1.74E-01	2.05	8.26	8.04E-01
cg16548254	18.49	8.35	2.69E-02	13.52	12.66	2.86E-01	21.77	16.01	1.74E-01	1.40	21.46	9.48E-01	45.91	22.26	3.92E-02
cg15270790	-12.77	5.77	2.69E-02	-24.18	10.61	2.27E-02	-7.52	13.29	5.71E-01	-7.50	8.99	4.04E-01	-10.75	18.00	5.50E-01
cg26864036	-19.75	8.92	2.69E-02	-25.67	12.76	4.42E-02	-24.13	26.32	3.59E-01	-15.92	18.80	3.97E-01	-4.85	21.62	8.22E-01
cg03253303	-9.67	4.37	2.69E-02	-12.10	6.78	7.44E-02	-9.53	10.94	3.84E-01	-9.56	9.97	3.37E-01	-5.53	9.04	5.40E-01
cg13718827	11.11	5.02	2.69E-02	7.72	5.14	1.33E-01	22.64	9.65	1.90E-02	21.57	12.45	8.31E-02	-2.11	11.00	8.48E-01
cg04565473	8.05	3.64	2.69E-02	9.50	5.27	7.18E-02	8.98	9.18	3.28E-01	5.46	8.91	5.40E-01	6.04	8.11	4.57E-01
cg23666831	-11.00	4.97	2.69E-02	-12.97	7.63	8.94E-02	-0.46	11.65	9.69E-01	-10.04	14.80	4.98E-01	-15.25	9.38	1.04E-01
cg24997231	5.72	2.59	2.69E-02	6.12	5.05	2.26E-01	8.81	6.92	2.03E-01	5.08	3.66	1.66E-01	3.56	8.17	6.63E-01
cg23614125	5.80	2.62	2.69E-02	2.34	4.86	6.30E-01	5.87	5.35	2.72E-01	9.20	4.51	4.15E-02	4.59	7.20	5.24E-01
cg15713391	14.39	6.50	2.69E-02	38.92	19.29	4.36E-02	0.46	24.92	9.85E-01	10.50	7.37	1.54E-01	42.77	31.88	1.80E-01
cg15986555	-7.74	3.50	2.69E-02	-7.75	6.73	2.49E-01	-0.56	9.20	9.52E-01	-12.49	5.31	1.86E-02	-0.91	9.03	9.19E-01
cg21792737	-8.05	3.64	2.69E-02	-7.88	5.19	1.29E-01	-13.96	8.32	9.31E-02	-3.11	8.79	7.23E-01	-6.65	9.47	4.82E-01

cg21152690	11.10	5.02	2.69E-02	15.77	13.09	2.28E-01	52.97	43.25	2.21E-01	11.32	5.80	5.09E-02	-4.46	16.65	7.89E-01
cg14508508	13.31	6.02	2.69E-02	10.66	12.27	3.85E-01	16.17	13.98	2.47E-01	16.15	10.57	1.27E-01	10.07	12.01	4.02E-01
cg14398946	31.08	14.05	2.69E-02	28.42	30.83	3.57E-01	82.24	86.09	3.39E-01	38.22	17.84	3.22E-02	-4.78	36.77	8.97E-01
cg13357714	-10.03	4.53	2.69E-02	-12.20	6.75	7.06E-02	6.29	12.45	6.14E-01	-5.81	10.36	5.75E-01	-18.94	9.58	4.80E-02
cg23224666	-9.26	4.19	2.69E-02	-5.87	9.19	5.23E-01	-26.04	18.06	1.49E-01	-7.67	5.21	1.41E-01	-18.22	13.70	1.84E-01
cg15629134	22.82	10.31	2.69E-02	8.31	31.98	7.95E-01	19.82	99.15	8.42E-01	23.51	11.35	3.82E-02	39.14	42.38	3.56E-01
cg26020069	-10.79	4.88	2.69E-02	-13.94	9.10	1.25E-01	-1.25	13.15	9.24E-01	-8.13	7.85	3.00E-01	-18.42	11.23	1.01E-01
cg04957757	14.45	6.53	2.69E-02	21.69	9.90	2.84E-02	19.66	12.70	1.22E-01	-8.60	16.15	5.94E-01	8.91	17.67	6.14E-01
cg02832322	42.86	19.37	2.69E-02	45.07	26.49	8.89E-02	-4.91	61.07	9.36E-01	57.78	45.15	2.01E-01	47.74	45.61	2.95E-01
cg26097364	22.33	10.09	2.69E-02	20.34	20.27	3.16E-01	-19.27	43.39	6.57E-01	22.20	13.43	9.83E-02	43.62	27.71	1.16E-01
cg19717640	-6.62	2.99	2.69E-02	-12.21	6.03	4.30E-02	-8.39	9.76	3.90E-01	-2.32	4.16	5.78E-01	-11.49	7.95	1.49E-01
cg01080357	22.34	10.10	2.69E-02	38.76	19.96	5.22E-02	12.22	19.81	5.37E-01	5.90	17.72	7.39E-01	45.85	25.24	6.93E-02
cg10373733	25.66	11.60	2.70E-02	10.69	15.56	4.92E-01	61.55	54.01	2.54E-01	55.25	53.47	3.01E-01	40.68	19.57	3.77E-02
cg15799791	-37.38	16.90	2.70E-02	-20.18	23.86	3.98E-01	29.31	59.64	6.23E-01	-64.07	40.59	1.14E-01	-66.51	30.09	2.71E-02
cg03103770	20.68	9.35	2.70E-02	27.40	32.12	3.94E-01	72.36	109.95	5.10E-01	17.94	10.11	7.59E-02	47.22	40.68	2.46E-01
cg24728435	-40.14	18.14	2.70E-02	-64.72	54.21	2.32E-01	-201.03	190.61	2.92E-01	-37.89	20.34	6.24E-02	-10.87	63.06	8.63E-01
cg27262702	11.31	5.11	2.70E-02	9.83	8.78	2.63E-01	-23.23	29.83	4.36E-01	11.09	7.02	1.14E-01	27.72	16.19	8.68E-02
cg21031917	-10.70	4.83	2.70E-02	-22.32	6.80	1.04E-03	-2.30	9.58	8.10E-01	-4.96	5.08	3.29E-01	-13.09	10.18	1.98E-01
cg03947979	16.49	7.45	2.70E-02	20.94	10.25	4.11E-02	15.30	26.48	5.63E-01	23.06	30.51	4.50E-01	8.52	12.92	5.10E-01
cg25287482	-11.65	5.27	2.70E-02	-9.35	7.42	2.08E-01	-22.38	11.95	6.10E-02	-3.84	18.26	8.34E-01	-10.39	11.26	3.56E-01
cg25673241	-11.27	5.09	2.70E-02	-12.88	7.76	9.71E-02	-11.69	11.37	3.04E-01	-5.62	12.53	6.54E-01	-12.05	11.30	2.86E-01
cg09547427	22.41	10.13	2.70E-02	-2.97	16.52	8.58E-01	29.97	15.70	5.62E-02	42.81	17.30	1.33E-02	19.61	21.80	3.68E-01
cg20880234	-58.12	26.28	2.70E-02	-51.67	27.18	5.73E-02	-200.74	79.37	1.14E-02	-10.01	75.70	8.95E-01	-39.15	39.89	3.26E-01
cg03806261	-13.31	6.02	2.70E-02	-14.06	8.94	1.16E-01	-11.76	17.22	4.95E-01	-13.12	13.87	3.44E-01	-12.81	12.35	3.00E-01
cg13934560	10.69	4.83	2.70E-02	17.05	9.64	7.71E-02	-0.88	9.93	9.29E-01	12.40	7.57	1.01E-01	14.88	14.90	3.18E-01
cg05844789	-7.92	3.58	2.70E-02	-8.54	4.84	7.77E-02	-7.08	8.80	4.21E-01	-14.34	13.05	2.72E-01	-4.70	7.79	5.47E-01
cg02724647	-15.25	6.90	2.70E-02	-17.51	12.47	1.60E-01	-19.97	14.45	1.67E-01	-10.06	13.56	4.58E-01	-13.22	15.13	3.82E-01
cg20379725	-9.06	4.09	2.70E-02	-12.04	5.56	3.03E-02	-8.13	9.42	3.88E-01	-24.77	23.50	2.92E-01	-0.99	8.39	9.06E-01
cg05557991	7.50	3.39	2.70E-02	5.09	4.90	2.99E-01	14.52	6.84	3.37E-02	7.90	9.62	4.12E-01	3.37	8.73	6.99E-01
cg16872028	15.61	7.06	2.70E-02	15.69	14.01	2.63E-01	43.57	42.26	3.02E-01	18.28	8.93	4.07E-02	-10.79	23.10	6.40E-01
cg06760830	13.24	5.99	2.70E-02	15.99	8.72	6.68E-02	9.18	15.42	5.52E-01	29.79	21.45	1.65E-01	6.67	10.92	5.41E-01
cg13543254	-13.90	6.29	2.70E-02	-17.44	9.62	6.98E-02	-8.57	14.07	5.42E-01	4.91	18.87	7.95E-01	-20.13	12.27	1.01E-01
cg23248424	-1.93	0.87	2.70E-02	-2.71	1.25	3.09E-02	-1.06	2.04	6.05E-01	-3.42	2.17	1.15E-01	0.79	2.14	7.11E-01
cg01882113	-18.98	8.58	2.70E-02	-28.54	13.05	2.87E-02	-34.71	20.59	9.18E-02	4.87	16.89	7.73E-01	-15.76	19.60	4.21E-01
cg25244008	-45.46	20.56	2.70E-02	-45.26	22.77	4.68E-02	-41.42	71.69	5.63E-01	80.92	79.02	3.06E-01	-77.68	36.85	3.50E-02

cg08901901	18.75	8.48	2.70E-02	25.92	14.08	6.55E-02	-27.12	39.93	4.97E-01	34.34	42.63	4.21E-01	16.67	11.41	1.44E-01
cg23200506	-12.99	5.87	2.70E-02	-23.03	8.82	9.02E-03	0.56	12.94	9.65E-01	-12.78	18.88	4.99E-01	-6.53	11.67	5.76E-01
cg27608981	15.86	7.17	2.70E-02	0.49	16.99	9.77E-01	1.12	23.08	9.61E-01	23.52	8.96	8.67E-03	7.05	24.75	7.76E-01
cg00255925	22.48	10.17	2.70E-02	6.06	30.65	8.43E-01	-3.90	143.71	9.78E-01	23.32	11.26	3.83E-02	40.66	38.64	2.93E-01
cg16373023	-49.05	22.18	2.70E-02	-43.61	28.75	1.29E-01	-37.30	95.45	6.96E-01	-84.41	65.43	1.97E-01	-48.25	45.71	2.91E-01
cg25942572	-13.65	6.17	2.70E-02	-28.41	13.75	3.88E-02	-10.25	15.27	5.02E-01	-10.28	8.29	2.15E-01	-6.82	21.79	7.54E-01
cg23242489	10.63	4.81	2.70E-02	12.06	7.48	1.07E-01	9.93	8.89	2.64E-01	6.77	12.02	5.73E-01	12.39	13.15	3.46E-01
cg24939196	-10.80	4.89	2.70E-02	-14.54	6.89	3.48E-02	-2.87	11.46	8.02E-01	-4.66	15.86	7.69E-01	-11.45	10.40	2.71E-01
cg18586891	13.23	5.98	2.70E-02	11.74	8.39	1.61E-01	14.14	11.83	2.32E-01	13.95	18.53	4.52E-01	16.63	16.50	3.14E-01
cg14316305	20.06	9.07	2.70E-02	8.86	16.78	5.98E-01	23.12	20.59	2.62E-01	13.38	15.14	3.77E-01	53.03	23.11	2.17E-02
cg10641986	42.19	19.08	2.70E-02	47.85	55.69	3.90E-01	123.49	203.93	5.45E-01	35.32	21.60	1.02E-01	84.82	62.41	1.74E-01
cg08989932	-43.80	19.81	2.70E-02	-24.56	59.06	6.78E-01	-163.16	228.29	4.75E-01	-46.69	22.15	3.51E-02	-30.82	69.78	6.59E-01
cg14926717	33.96	15.36	2.70E-02	24.05	29.91	4.21E-01	105.77	88.82	2.34E-01	17.61	9.89	7.50E-02	70.85	25.71	5.86E-03
cg12905574	-29.86	13.50	2.70E-02	-8.04	49.32	8.71E-01	8.25	225.14	9.71E-01	-33.41	14.45	2.08E-02	-2.53	61.29	9.67E-01
cg17181167	33.36	15.09	2.70E-02	6.26	11.72	5.93E-01	56.50	24.65	2.19E-02	56.17	44.19	2.04E-01	46.71	19.91	1.90E-02
cg23374863	-7.20	3.26	2.70E-02	-6.26	5.08	2.18E-01	-12.99	6.44	4.36E-02	-0.52	9.48	9.56E-01	-5.79	7.01	4.09E-01
cg11165391	-13.28	6.01	2.70E-02	-18.01	15.87	2.56E-01	-15.46	30.91	6.17E-01	-11.72	7.00	9.43E-02	-17.98	20.84	3.88E-01
cg11581627	30.04	13.59	2.70E-02	25.84	18.72	1.67E-01	72.56	39.56	6.66E-02	30.61	35.77	3.92E-01	16.33	29.61	5.81E-01
cg14210726	6.38	2.88	2.70E-02	4.80	3.45	1.64E-01	15.09	7.90	5.62E-02	4.41	11.57	7.03E-01	6.99	8.93	4.34E-01
cg11609366	-41.18	18.63	2.71E-02	-46.70	23.17	4.39E-02	-136.08	92.47	1.41E-01	42.39	68.92	5.39E-01	-35.70	38.01	3.48E-01
cg15398152	10.88	4.92	2.71E-02	6.21	6.84	3.64E-01	17.77	10.33	8.54E-02	15.18	17.66	3.90E-01	13.75	11.64	2.38E-01
cg25345722	-11.05	5.00	2.71E-02	-8.60	7.06	2.23E-01	-22.41	10.52	3.31E-02	4.09	14.53	7.78E-01	-13.99	12.72	2.72E-01
cg03739877	19.41	8.78	2.71E-02	22.02	14.87	1.39E-01	15.59	16.29	3.39E-01	19.89	19.27	3.02E-01	20.04	22.39	3.71E-01
cg02794788	12.25	5.54	2.71E-02	3.59	16.76	8.31E-01	27.34	26.71	3.06E-01	12.09	6.20	5.11E-02	21.42	25.53	4.02E-01
cg21192185	15.30	6.92	2.71E-02	21.40	20.31	2.92E-01	13.60	21.67	5.30E-01	13.06	8.17	1.10E-01	32.11	27.45	2.42E-01
cg15689513	19.79	8.95	2.71E-02	32.03	17.13	6.15E-02	9.51	15.44	5.38E-01	6.94	18.82	7.12E-01	38.26	22.12	8.36E-02
cg07018260	7.11	3.22	2.71E-02	5.32	7.96	5.04E-01	17.34	11.06	1.17E-01	7.33	3.91	6.04E-02	-2.60	11.76	8.25E-01
cg26311932	-14.51	6.56	2.71E-02	-11.45	8.72	1.89E-01	-19.94	17.21	2.47E-01	-13.75	23.45	5.58E-01	-19.31	14.34	1.78E-01
cg22034203	4.68	2.12	2.71E-02	8.14	3.56	2.24E-02	-0.46	4.45	9.18E-01	5.79	3.81	1.29E-01	1.11	6.32	8.61E-01
cg02273944	15.93	7.21	2.71E-02	22.20	10.91	4.19E-02	5.89	14.19	6.78E-01	9.23	18.11	6.10E-01	22.21	18.83	2.38E-01
cg11420192	38.34	17.34	2.71E-02	52.46	25.09	3.66E-02	23.51	41.71	5.73E-01	-16.77	43.73	7.01E-01	61.68	39.58	1.19E-01
cg03895593	9.39	4.25	2.71E-02	10.35	7.14	1.47E-01	16.33	9.58	8.82E-02	10.76	8.87	2.25E-01	0.23	9.05	9.80E-01
cg05985134	31.69	14.34	2.71E-02	51.59	35.99	1.52E-01	-111.31	103.40	2.82E-01	31.84	16.56	5.45E-02	24.51	53.17	6.45E-01
cg23486142	26.56	12.02	2.71E-02	28.14	15.22	6.44E-02	34.27	46.23	4.58E-01	91.34	55.10	9.74E-02	8.99	23.52	7.02E-01
cg10437839	7.65	3.46	2.71E-02	7.64	4.59	9.60E-02	15.34	8.17	6.04E-02	10.63	10.12	2.93E-01	-5.06	9.39	5.90E-01

cg02514318	-8.29	3.75	2.71E-02	-13.78	8.80	1.18E-01	-15.19	15.02	3.12E-01	-7.14	4.56	1.17E-01	-0.07	13.32	9.96E-01
cg07939836	24.94	11.28	2.71E-02	58.24	29.32	4.70E-02	-15.32	89.64	8.64E-01	21.76	13.25	1.00E-01	6.96	33.95	8.38E-01
cg16531924	-28.70	12.98	2.71E-02	-20.73	15.93	1.93E-01	-81.93	69.62	2.39E-01	-58.11	47.63	2.22E-01	-34.27	27.30	2.09E-01
cg12471986	15.29	6.92	2.71E-02	21.12	9.28	2.28E-02	16.74	23.08	4.68E-01	23.97	21.09	2.56E-01	-2.19	13.95	8.75E-01
cg25486145	22.99	10.40	2.71E-02	14.46	23.41	5.37E-01	9.34	29.79	7.54E-01	20.95	13.43	1.19E-01	79.95	36.71	2.94E-02
cg14850660	-10.54	4.77	2.71E-02	-24.47	12.48	4.99E-02	-19.21	17.01	2.59E-01	-8.25	5.88	1.61E-01	-0.22	13.96	9.87E-01
cg03386480	25.96	11.75	2.71E-02	20.66	14.30	1.49E-01	38.96	74.68	6.02E-01	91.38	62.34	1.43E-01	29.51	22.83	1.96E-01
cg07111554	15.56	7.04	2.71E-02	33.44	15.39	2.98E-02	4.71	17.57	7.89E-01	15.91	9.65	9.92E-02	-6.80	22.51	7.63E-01
cg26804891	9.30	4.21	2.71E-02	12.70	6.12	3.82E-02	9.86	9.42	2.95E-01	1.41	9.38	8.80E-01	8.30	11.83	4.83E-01
cg16437211	-8.00	3.62	2.71E-02	-14.07	5.54	1.10E-02	-4.71	8.25	5.68E-01	-6.28	10.65	5.55E-01	-1.33	7.03	8.50E-01
cg14991769	6.78	3.07	2.71E-02	6.37	4.24	1.33E-01	8.56	6.23	1.69E-01	7.46	10.34	4.71E-01	4.90	8.06	5.43E-01
cg09257735	10.80	4.89	2.71E-02	7.13	8.68	4.12E-01	13.45	12.57	2.84E-01	18.64	8.55	2.94E-02	2.07	10.77	8.48E-01
cg01852186	-7.12	3.22	2.71E-02	-11.35	5.99	5.82E-02	-3.73	10.60	7.25E-01	-5.73	4.70	2.22E-01	-5.37	8.37	5.21E-01
cg15962336	18.31	8.29	2.71E-02	35.21	13.47	8.92E-03	11.76	18.35	5.22E-01	17.45	14.99	2.44E-01	-4.82	18.76	7.97E-01
cg10834721	-50.23	22.73	2.71E-02	-44.24	30.13	1.42E-01	-33.19	102.84	7.47E-01	-175.91	85.69	4.01E-02	-35.46	40.71	3.84E-01
cg19894747	32.97	14.92	2.71E-02	54.47	32.97	9.85E-02	-149.58	163.78	3.61E-01	20.32	10.81	6.01E-02	66.33	31.76	3.67E-02
cg19472770	14.98	6.78	2.71E-02	16.77	9.82	8.78E-02	4.67	16.42	7.76E-01	21.06	16.97	2.15E-01	14.66	15.42	3.42E-01
cg11762213	11.59	5.24	2.71E-02	9.97	7.24	1.68E-01	16.68	11.56	1.49E-01	15.45	17.44	3.75E-01	8.52	12.38	4.91E-01
cg25654677	-17.15	7.76	2.71E-02	-13.94	12.14	2.51E-01	-25.64	15.84	1.05E-01	-9.73	17.36	5.75E-01	-22.17	19.98	2.67E-01
cg01757168	5.12	2.32	2.71E-02	7.51	3.32	2.39E-02	-1.82	5.08	7.20E-01	10.65	7.65	1.64E-01	4.08	5.01	4.16E-01
cg17819489	-15.31	6.93	2.71E-02	-18.58	14.97	2.15E-01	13.05	36.65	7.22E-01	-19.04	8.67	2.81E-02	3.25	20.73	8.76E-01
cg00634652	-19.59	8.87	2.72E-02	-26.42	12.61	3.62E-02	6.92	25.70	7.88E-01	-20.12	34.83	5.64E-01	-18.81	15.65	2.29E-01
cg04765483	11.57	5.24	2.72E-02	10.72	8.37	2.00E-01	5.33	12.18	6.62E-01	14.37	10.79	1.83E-01	15.99	12.10	1.86E-01
cg07459650	27.29	12.35	2.72E-02	27.53	18.08	1.28E-01	27.20	25.21	2.81E-01	46.80	37.69	2.14E-01	15.51	28.69	5.89E-01
cg07772781	8.24	3.73	2.72E-02	13.62	5.63	1.56E-02	5.61	7.49	4.54E-01	0.89	5.21	8.65E-01	16.68	8.47	4.90E-02
cg06910299	18.87	8.54	2.72E-02	18.38	17.56	2.95E-01	61.21	78.18	4.34E-01	18.78	10.41	7.13E-02	14.68	30.42	6.29E-01
cg21272996	6.82	3.09	2.72E-02	6.17	4.10	1.33E-01	12.09	7.01	8.47E-02	16.55	10.80	1.25E-01	-2.36	7.77	7.62E-01
cg02434007	-11.55	5.23	2.72E-02	-10.91	7.16	1.28E-01	-17.98	12.20	1.41E-01	0.35	21.71	9.87E-01	-10.89	11.01	3.23E-01
cg24372325	44.27	20.04	2.72E-02	5.43	49.90	9.13E-01	324.58	269.58	2.29E-01	53.56	23.44	2.23E-02	23.96	62.66	7.02E-01
cg22270027	6.68	3.02	2.72E-02	3.00	4.50	5.05E-01	6.91	5.63	2.20E-01	13.36	9.11	1.42E-01	12.38	7.80	1.13E-01
cg13543355	-19.25	8.72	2.72E-02	-13.32	11.62	2.52E-01	-43.47	25.15	8.40E-02	-5.68	27.70	8.38E-01	-27.36	18.64	1.42E-01
cg01878435	21.33	9.66	2.72E-02	24.36	14.02	8.24E-02	44.14	34.18	1.97E-01	31.94	26.60	2.30E-01	6.52	17.22	7.05E-01
cg12351068	-15.97	7.23	2.72E-02	-24.63	18.48	1.83E-01	1.09	37.89	9.77E-01	-12.35	8.53	1.48E-01	-36.46	23.79	1.25E-01
cg07567612	-21.80	9.87	2.72E-02	-19.30	20.93	3.57E-01	-25.31	39.14	5.18E-01	-21.10	12.83	1.00E-01	-27.87	28.18	3.23E-01
cg02568619	26.83	12.15	2.72E-02	37.20	18.84	4.83E-02	56.06	19.95	4.95E-03	8.93	13.35	5.03E-01	3.95	30.10	8.96E-01

cg06754565	-58.59	26.53	2.72E-02	-64.78	35.59	6.87E-02	-64.80	108.79	5.51E-01	-4.52	79.21	9.54E-01	-66.88	50.83	1.88E-01
cg20631628	77.10	34.91	2.72E-02	24.65	30.64	4.21E-01	108.87	138.95	4.33E-01	89.63	69.81	1.99E-01	146.02	49.79	3.36E-03
cg08378342	15.80	7.15	2.72E-02	17.18	12.27	1.61E-01	3.76	19.15	8.44E-01	28.05	14.09	4.64E-02	8.36	13.96	5.49E-01
cg24237081	22.04	9.98	2.72E-02	20.97	15.71	1.82E-01	16.05	25.84	5.35E-01	54.46	31.03	7.92E-02	16.14	17.01	3.43E-01
cg25608078	46.62	21.11	2.72E-02	57.83	26.54	2.93E-02	-52.96	89.34	5.53E-01	45.31	70.56	5.21E-01	40.24	44.80	3.69E-01
cg06376558	-14.87	6.73	2.72E-02	-24.39	11.14	2.85E-02	-3.04	14.17	8.30E-01	-11.68	15.37	4.47E-01	-13.95	14.45	3.34E-01
cg19932079	-10.86	4.92	2.72E-02	-9.43	8.83	2.86E-01	-12.76	10.47	2.23E-01	-8.38	9.13	3.58E-01	-15.05	11.65	1.97E-01
cg23104592	-15.54	7.04	2.72E-02	-11.53	9.45	2.22E-01	-20.62	16.92	2.23E-01	9.64	18.57	6.04E-01	-29.29	11.48	1.08E-02
cg06867478	14.18	6.42	2.72E-02	18.45	9.74	5.82E-02	14.88	13.79	2.81E-01	5.91	17.07	7.29E-01	10.14	14.13	4.73E-01
cg08849294	13.10	5.94	2.72E-02	16.34	8.98	6.87E-02	18.53	12.13	1.27E-01	9.78	17.72	5.81E-01	2.04	12.91	8.74E-01
cg04741094	-11.61	5.26	2.72E-02	-12.72	11.62	2.74E-01	1.04	19.99	9.58E-01	-13.48	6.74	4.55E-02	-7.44	15.31	6.27E-01
cg23421970	-57.98	26.26	2.72E-02	-25.23	48.29	6.01E-01	-132.05	318.47	6.78E-01	-78.41	34.47	2.29E-02	-35.05	76.77	6.48E-01
cg14753094	-15.10	6.84	2.73E-02	-17.56	14.47	2.25E-01	-1.30	17.93	9.42E-01	-17.62	9.39	6.06E-02	-16.25	21.50	4.50E-01
cg12934461	16.72	7.57	2.73E-02	9.80	27.76	7.24E-01	28.78	23.68	2.24E-01	11.39	9.60	2.35E-01	29.71	16.92	7.90E-02
cg17888554	11.55	5.23	2.73E-02	10.54	7.73	1.73E-01	13.48	11.89	2.57E-01	0.08	15.77	9.96E-01	17.17	10.69	1.08E-01
cg11118615	13.27	6.01	2.73E-02	16.34	10.29	1.12E-01	17.16	10.67	1.08E-01	8.16	13.91	5.57E-01	4.71	15.25	7.58E-01
cg23032421	-10.28	4.65	2.73E-02	-5.28	7.42	4.77E-01	-14.40	10.15	1.56E-01	-3.26	10.31	7.52E-01	-23.36	10.59	2.74E-02
cg00471159	13.31	6.03	2.73E-02	13.19	9.13	1.49E-01	0.63	12.96	9.61E-01	23.23	21.98	2.91E-01	20.84	11.55	7.12E-02
cg07014308	7.20	3.26	2.73E-02	10.18	4.39	2.04E-02	-3.08	8.00	7.00E-01	15.27	9.06	9.20E-02	3.73	6.56	5.70E-01
cg02560973	24.87	11.26	2.73E-02	37.25	13.79	6.90E-03	-3.80	44.59	9.32E-01	66.56	44.75	1.37E-01	6.13	16.63	7.12E-01
cg09454997	-21.66	9.81	2.73E-02	-5.62	22.99	8.07E-01	9.37	50.06	8.52E-01	-27.42	12.13	2.38E-02	-24.40	27.74	3.79E-01
cg12128149	-42.84	19.41	2.73E-02	-46.54	29.39	1.13E-01	-29.53	49.41	5.50E-01	-49.20	36.96	1.83E-01	-33.07	52.98	5.32E-01
cg19439043	6.10	2.76	2.73E-02	6.70	3.79	7.74E-02	9.39	6.27	1.35E-01	3.43	8.07	6.71E-01	2.05	6.92	7.67E-01
cg14182820	6.85	3.10	2.73E-02	4.40	6.38	4.91E-01	-0.68	9.75	9.44E-01	10.78	4.17	9.79E-03	-0.78	9.43	9.34E-01
cg07340651	-11.66	5.28	2.73E-02	-16.54	8.98	6.54E-02	-7.76	12.02	5.19E-01	-11.15	9.65	2.48E-01	-6.81	13.14	6.04E-01
cg27576259	10.12	4.58	2.73E-02	7.58	5.99	2.06E-01	23.68	11.20	3.44E-02	10.41	15.31	4.97E-01	4.95	11.55	6.68E-01
cg21158528	-10.24	4.64	2.73E-02	-13.42	7.45	7.18E-02	-6.48	10.64	5.42E-01	-13.40	10.09	1.84E-01	-4.63	10.11	6.47E-01
cg13411122	24.61	11.15	2.73E-02	37.38	14.73	1.12E-02	11.46	29.62	6.99E-01	30.25	39.09	4.39E-01	-4.24	24.63	8.63E-01
cg10009497	7.86	3.56	2.73E-02	6.53	5.07	1.98E-01	19.65	9.63	4.13E-02	4.52	8.27	5.85E-01	6.04	8.28	4.65E-01
cg18828619	11.28	5.11	2.73E-02	15.10	7.35	3.99E-02	14.83	11.80	2.09E-01	6.27	11.94	5.99E-01	0.37	13.35	9.78E-01
cg18723442	7.68	3.48	2.73E-02	14.21	5.42	8.71E-03	8.83	6.55	1.78E-01	4.33	7.79	5.78E-01	-1.71	7.26	8.14E-01
cg12492380	17.58	7.96	2.73E-02	23.67	14.50	1.03E-01	22.36	17.25	1.95E-01	10.29	12.73	4.19E-01	17.56	26.00	4.99E-01
cg07168939	12.37	5.60	2.73E-02	10.70	7.68	1.63E-01	16.54	12.99	2.03E-01	-12.03	20.35	5.54E-01	21.86	12.34	7.64E-02
cg00055603	-8.36	3.79	2.73E-02	-1.27	7.76	8.70E-01	3.13	10.34	7.62E-01	-13.57	4.29	1.55E-03	-9.53	9.21	3.01E-01
cg22257099	23.53	10.66	2.73E-02	33.83	14.23	1.75E-02	33.11	33.35	3.21E-01	12.28	32.28	7.04E-01	-0.74	22.34	9.74E-01

cg17701942	-13.92	6.30	2.73E-02	-8.06	12.57	5.22E-01	-17.85	16.43	2.77E-01	-11.10	8.60	1.97E-01	-52.17	25.11	3.77E-02
cg27638196	10.45	4.73	2.73E-02	16.89	6.72	1.19E-02	0.53	11.24	9.62E-01	19.74	15.01	1.89E-01	1.13	9.50	9.05E-01
cg07691624	9.65	4.37	2.73E-02	16.72	8.34	4.50E-02	21.65	11.59	6.19E-02	4.23	6.16	4.93E-01	0.13	13.80	9.93E-01
cg01844866	-34.31	15.54	2.73E-02	-39.93	21.34	6.14E-02	-17.56	51.60	7.34E-01	-27.02	47.78	5.72E-01	-31.79	29.76	2.85E-01
cg14000467	9.37	4.25	2.73E-02	4.79	5.66	3.97E-01	14.74	8.08	6.82E-02	19.93	23.48	3.96E-01	15.29	11.87	1.98E-01
cg24570303	29.08	13.17	2.73E-02	32.15	20.87	1.23E-01	13.38	52.36	7.98E-01	23.75	37.47	5.26E-01	30.11	20.46	1.41E-01
cg13277940	-32.95	14.93	2.73E-02	-35.88	16.33	2.80E-02	-90.34	124.02	4.66E-01	109.62	92.32	2.35E-01	-36.64	42.59	3.90E-01
cg22688535	-12.02	5.45	2.73E-02	-16.84	15.02	2.62E-01	9.29	20.56	6.51E-01	-14.76	6.38	2.07E-02	4.29	20.64	8.35E-01
cg24311251	17.63	7.99	2.73E-02	23.53	13.50	8.14E-02	2.19	28.15	9.38E-01	16.08	11.95	1.78E-01	16.58	22.84	4.68E-01
cg13954297	-30.00	13.59	2.73E-02	-38.31	27.85	1.69E-01	-37.25	184.30	8.40E-01	-24.21	16.46	1.41E-01	-55.98	49.89	2.62E-01
cg24980158	-9.49	4.30	2.73E-02	-15.67	8.09	5.29E-02	1.33	15.89	9.33E-01	-8.24	5.84	1.58E-01	-6.79	13.37	6.12E-01
cg05671636	11.61	5.26	2.73E-02	16.27	7.94	4.06E-02	5.39	11.47	6.39E-01	13.52	11.65	2.46E-01	3.97	13.74	7.73E-01
cg00855901	8.41	3.81	2.73E-02	11.72	5.16	2.31E-02	13.34	9.64	1.66E-01	0.00	12.53	1.00E+00	-0.35	8.41	9.67E-01
cg22411165	-13.70	6.21	2.73E-02	-22.09	16.80	1.89E-01	-6.61	25.20	7.93E-01	-9.20	7.98	2.49E-01	-23.84	13.96	8.78E-02
cg26134665	13.89	6.30	2.73E-02	12.96	8.98	1.49E-01	28.94	13.32	2.98E-02	6.44	24.34	7.91E-01	2.87	13.48	8.32E-01
cg05426485	18.12	8.21	2.73E-02	14.92	12.42	2.30E-01	12.61	20.53	5.39E-01	42.75	22.52	5.77E-02	14.44	15.79	3.60E-01
cg02339122	-11.52	5.22	2.73E-02	-16.91	8.32	4.20E-02	-10.39	12.62	4.10E-01	-7.00	11.20	5.32E-01	-7.16	11.21	5.23E-01
cg12480903	10.98	4.98	2.73E-02	14.35	7.70	6.25E-02	4.79	12.66	7.05E-01	21.04	14.69	1.52E-01	5.86	8.89	5.10E-01
cg19380675	-7.58	3.44	2.73E-02	-13.16	5.97	2.75E-02	-7.29	6.64	2.72E-01	-7.28	6.54	2.65E-01	5.94	9.72	5.41E-01
cg11786338	-8.22	3.72	2.73E-02	-11.67	7.56	1.23E-01	-16.62	12.94	1.99E-01	-7.80	5.03	1.21E-01	2.03	10.44	8.46E-01
cg24650940	10.40	4.71	2.73E-02	13.95	6.70	3.75E-02	20.85	11.84	7.81E-02	5.43	11.76	6.44E-01	-3.59	10.91	7.42E-01
cg11623293	21.84	9.90	2.74E-02	33.53	15.34	2.88E-02	24.66	19.97	2.17E-01	9.39	21.76	6.66E-01	-0.93	27.37	9.73E-01
cg10146663	-39.06	17.70	2.74E-02	-18.72	23.41	4.24E-01	-64.62	46.56	1.65E-01	-63.04	75.94	4.06E-01	-67.99	36.98	6.60E-02
cg12231584	16.44	7.45	2.74E-02	24.67	18.85	1.91E-01	29.84	21.82	1.71E-01	10.15	9.22	2.71E-01	33.58	27.43	2.21E-01
cg01739167	-14.93	6.76	2.74E-02	-18.68	9.66	5.30E-02	-7.97	17.42	6.47E-01	-8.89	16.20	5.83E-01	-16.33	15.77	3.00E-01
cg03048354	-54.36	24.64	2.74E-02	-68.80	31.73	3.01E-02	-76.31	116.08	5.11E-01	-113.12	73.82	1.25E-01	13.18	50.23	7.93E-01
cg13149147	8.93	4.05	2.74E-02	9.01	5.53	1.03E-01	16.91	10.65	1.12E-01	9.91	10.12	3.28E-01	0.46	10.14	9.64E-01
cg19589396	-9.55	4.33	2.74E-02	-14.60	6.64	2.79E-02	-1.27	9.44	8.93E-01	-5.90	10.29	5.66E-01	-10.85	10.00	2.78E-01
cg19526908	-29.76	13.49	2.74E-02	-24.05	36.12	5.06E-01	-14.49	182.77	9.37E-01	-29.68	15.42	5.43E-02	-40.21	44.97	3.71E-01
cg23832822	-20.94	9.49	2.74E-02	-13.54	14.68	3.56E-01	-58.83	24.14	1.48E-02	-12.56	22.50	5.77E-01	-15.85	19.01	4.04E-01
cg27558693	17.41	7.89	2.74E-02	18.26	10.96	9.57E-02	13.80	20.52	5.01E-01	48.91	24.39	4.49E-02	3.41	16.50	8.36E-01
cg24632873	-13.37	6.06	2.74E-02	-20.31	9.76	3.74E-02	-16.83	14.70	2.52E-01	-8.78	14.61	5.48E-01	-4.27	11.62	7.13E-01
cg08637514	-20.24	9.18	2.74E-02	-14.07	13.68	3.04E-01	-4.41	18.62	8.13E-01	-58.05	39.87	1.45E-01	-38.42	18.20	3.48E-02
cg23271318	-34.56	15.66	2.74E-02	-40.32	18.81	3.21E-02	-24.77	61.49	6.87E-01	-11.83	74.00	8.73E-01	-22.67	35.31	5.21E-01
cg23362687	-20.44	9.27	2.74E-02	-27.41	13.71	4.56E-02	-23.24	34.47	5.00E-01	-16.21	15.74	3.03E-01	-5.04	26.24	8.48E-01

cg03849279	13.87	6.29	2.74E-02	7.96	11.93	5.05E-01	16.51	18.53	3.73E-01	15.47	8.98	8.50E-02	18.63	18.39	3.11E-01
cg05984290	-36.82	16.69	2.74E-02	-44.27	22.20	4.62E-02	-88.45	45.73	5.31E-02	-21.18	72.01	7.69E-01	4.48	33.51	8.94E-01
cg19744122	59.69	27.06	2.74E-02	81.41	51.86	1.16E-01	-372.30	234.37	1.12E-01	59.15	24.57	1.61E-02	67.50	65.26	3.01E-01
cg20932768	37.10	16.82	2.74E-02	35.47	22.68	1.18E-01	38.24	72.00	5.95E-01	10.62	60.05	8.60E-01	46.26	29.86	1.21E-01
cg07908868	-12.84	5.82	2.74E-02	-19.15	9.03	3.39E-02	-8.24	12.84	5.21E-01	-13.69	17.22	4.27E-01	-6.13	11.32	5.88E-01
cg07458500	-15.11	6.85	2.74E-02	-8.83	12.21	4.69E-01	9.72	25.37	7.02E-01	-19.18	9.77	4.96E-02	-29.89	19.72	1.30E-01
cg14260530	13.47	6.11	2.74E-02	23.89	8.93	7.47E-03	9.97	12.34	4.19E-01	-6.97	18.14	7.01E-01	5.43	13.74	6.93E-01
cg05817443	13.50	6.12	2.74E-02	11.52	12.64	3.62E-01	13.74	14.27	3.36E-01	20.43	8.90	2.17E-02	-12.65	18.52	4.94E-01
cg24829430	20.62	9.35	2.74E-02	39.84	16.21	1.40E-02	15.62	20.30	4.42E-01	0.79	18.18	9.65E-01	20.18	21.38	3.45E-01
cg09276451	11.18	5.07	2.74E-02	6.75	7.68	3.79E-01	18.31	9.98	6.65E-02	7.29	15.19	6.31E-01	13.83	11.46	2.27E-01
cg05670472	-6.18	2.80	2.74E-02	-4.53	4.13	2.72E-01	-7.91	5.87	1.78E-01	-10.54	10.17	3.00E-01	-6.35	5.78	2.72E-01
cg23192824	-15.35	6.96	2.74E-02	-11.93	20.07	5.52E-01	-77.24	40.97	5.94E-02	-14.53	7.98	6.86E-02	-7.01	23.26	7.63E-01
cg12588208	15.81	7.17	2.74E-02	39.82	20.14	4.80E-02	30.36	21.81	1.64E-01	10.68	8.50	2.09E-01	-2.00	30.69	9.48E-01
cg02370877	16.42	7.44	2.74E-02	30.54	14.30	3.27E-02	-4.51	22.30	8.40E-01	16.30	12.37	1.88E-01	10.75	14.71	4.65E-01
cg01229943	-25.07	11.36	2.74E-02	-37.88	16.23	1.96E-02	-16.40	27.34	5.49E-01	-8.30	7.78	2.86E-01	-54.03	22.24	1.51E-02
cg14786757	20.32	9.21	2.74E-02	25.64	13.49	5.75E-02	26.62	19.04	1.62E-01	27.52	23.77	2.47E-01	-13.26	23.79	5.77E-01
cg26827394	-31.83	14.43	2.74E-02	-23.81	29.27	4.16E-01	-29.43	115.98	8.00E-01	-24.62	19.14	1.98E-01	-66.90	34.66	5.36E-02
cg17522953	-10.98	4.98	2.74E-02	-16.84	10.43	1.06E-01	-15.04	14.79	3.09E-01	-6.37	6.76	3.45E-01	-17.13	14.67	2.43E-01
cg10713839	-7.37	3.34	2.74E-02	-6.59	4.59	1.51E-01	-5.79	8.05	4.72E-01	-17.55	6.92	1.12E-02	0.09	6.94	9.90E-01
cg02362020	13.29	6.03	2.74E-02	18.55	9.75	5.71E-02	3.08	12.44	8.04E-01	7.38	13.04	5.71E-01	23.07	14.66	1.16E-01
cg04350202	7.06	3.20	2.74E-02	7.62	4.22	7.13E-02	15.29	9.16	9.50E-02	9.41	8.09	2.45E-01	-4.43	8.33	5.95E-01
cg05713486	11.20	5.08	2.74E-02	12.86	7.71	9.56E-02	6.80	9.93	4.93E-01	-0.45	17.09	9.79E-01	17.90	10.89	1.00E-01
cg11966583	-51.62	23.41	2.74E-02	-61.79	67.84	3.62E-01	243.67	275.73	3.77E-01	-42.82	27.10	1.14E-01	-110.15	65.47	9.25E-02
cg13338454	-7.59	3.44	2.74E-02	-15.28	9.00	8.96E-02	-0.51	11.71	9.65E-01	-6.90	4.16	9.74E-02	-7.10	11.89	5.50E-01
cg24478696	41.89	19.00	2.74E-02	30.78	24.09	2.01E-01	94.04	88.35	2.87E-01	19.93	77.52	7.97E-01	63.26	36.41	8.23E-02
cg21695485	19.71	8.94	2.74E-02	30.36	29.16	2.98E-01	61.29	82.07	4.55E-01	17.77	9.69	6.67E-02	23.35	42.98	5.87E-01
cg06550986	-8.67	3.93	2.74E-02	-7.17	8.55	4.02E-01	0.42	14.61	9.77E-01	-8.83	5.14	8.62E-02	-15.40	10.84	1.55E-01
cg10024501	-21.54	9.77	2.74E-02	-20.85	21.38	3.29E-01	-6.72	43.93	8.78E-01	-24.83	11.99	3.83E-02	-4.64	35.10	8.95E-01
cg03758467	9.14	4.15	2.74E-02	9.83	6.30	1.19E-01	13.68	9.69	1.58E-01	10.05	9.79	3.04E-01	2.85	9.15	7.55E-01
cg07669223	18.06	8.19	2.74E-02	9.58	15.60	5.39E-01	12.37	14.32	3.88E-01	29.71	17.10	8.23E-02	27.20	20.02	1.74E-01
cg26726529	-19.96	9.05	2.74E-02	-21.61	20.96	3.03E-01	-12.50	47.78	7.94E-01	-26.64	11.27	1.81E-02	12.84	24.87	6.06E-01
cg01431908	24.26	11.00	2.74E-02	43.70	19.85	2.77E-02	13.71	34.38	6.90E-01	17.52	23.69	4.60E-01	15.08	17.98	4.02E-01
cg24617741	11.92	5.40	2.74E-02	9.14	12.35	4.59E-01	3.33	17.74	8.51E-01	13.88	6.83	4.20E-02	13.00	18.12	4.73E-01
cg05504729	-8.91	4.04	2.75E-02	-10.05	6.17	1.03E-01	-13.36	8.62	1.21E-01	4.78	10.26	6.42E-01	-12.23	9.12	1.80E-01
cg14095316	-16.31	7.40	2.75E-02	-2.52	21.00	9.04E-01	-47.84	44.90	2.87E-01	-18.12	8.35	3.01E-02	-7.57	29.24	7.96E-01

cg18893025	-13.56	6.15	2.75E-02	-9.95	18.54	5.91E-01	-11.23	30.01	7.08E-01	-14.51	6.97	3.74E-02	-10.04	23.29	6.66E-01
cg00869668	6.73	3.05	2.75E-02	4.83	3.74	1.97E-01	13.95	6.54	3.28E-02	14.67	14.87	3.24E-01	-1.87	11.15	8.67E-01
cg10145246	15.70	7.12	2.75E-02	18.85	11.16	9.14E-02	22.19	19.58	2.57E-01	31.84	21.08	1.31E-01	4.22	12.10	7.27E-01
cg20789700	5.74	2.61	2.75E-02	3.83	3.43	2.64E-01	10.98	6.12	7.30E-02	11.41	7.67	1.37E-01	1.84	7.30	8.01E-01
cg17280106	6.41	2.91	2.75E-02	7.94	4.06	5.05E-02	4.20	7.06	5.53E-01	4.99	7.47	5.04E-01	5.26	7.16	4.62E-01
cg23029851	14.37	6.52	2.75E-02	13.87	9.89	1.61E-01	3.31	16.97	8.45E-01	16.73	15.37	2.76E-01	20.32	13.34	1.28E-01
cg04707332	10.81	4.90	2.75E-02	18.88	9.45	4.58E-02	1.28	12.65	9.19E-01	6.12	11.11	5.82E-01	11.26	7.89	1.54E-01
cg20058043	22.15	10.05	2.75E-02	40.55	22.33	6.93E-02	6.58	32.40	8.39E-01	22.29	13.71	1.04E-01	8.09	24.82	7.44E-01
cg18616862	32.46	14.72	2.75E-02	44.46	20.53	3.04E-02	16.80	39.28	6.69E-01	26.20	50.58	6.04E-01	19.27	28.84	5.04E-01
cg22357679	-12.52	5.68	2.75E-02	-17.22	8.46	4.18E-02	-4.80	14.01	7.32E-01	-8.65	14.21	5.43E-01	-11.48	11.95	3.37E-01
cg15658577	-67.24	30.50	2.75E-02	-64.28	26.79	1.64E-02	-64.06	84.71	4.50E-01	-155.85	50.80	2.15E-03	-2.30	41.38	9.56E-01
cg20034100	-10.17	4.61	2.75E-02	-10.98	11.69	3.47E-01	-1.75	22.41	9.38E-01	-9.91	5.53	7.30E-02	-14.02	14.17	3.23E-01
cg24319902	15.06	6.83	2.75E-02	31.70	12.01	8.28E-03	7.33	14.43	6.12E-01	19.62	10.42	5.98E-02	1.15	10.70	9.14E-01
cg17959962	10.77	4.89	2.75E-02	17.88	7.85	2.27E-02	-6.34	17.06	7.10E-01	7.48	7.44	3.15E-01	11.46	15.51	4.60E-01
cg15945999	-8.90	4.04	2.75E-02	-11.79	5.56	3.41E-02	-6.88	8.77	4.33E-01	-12.05	12.93	3.51E-01	-0.35	9.98	9.72E-01
cg07306190	9.67	4.39	2.75E-02	10.03	6.82	1.41E-01	12.36	10.72	2.49E-01	16.13	13.39	2.28E-01	5.52	7.87	4.83E-01
cg01223793	21.68	9.84	2.75E-02	17.43	20.37	3.92E-01	-4.92	53.38	9.27E-01	26.66	12.44	3.21E-02	10.32	30.01	7.31E-01
cg25649188	-11.84	5.37	2.75E-02	-11.48	8.04	1.53E-01	-18.34	11.29	1.04E-01	-4.35	16.60	7.93E-01	-9.49	11.40	4.05E-01
cg12018809	-9.50	4.31	2.75E-02	-11.31	5.64	4.51E-02	-6.84	10.84	5.28E-01	2.80	12.69	8.25E-01	-14.98	11.39	1.89E-01
cg15906675	16.04	7.28	2.75E-02	12.12	13.22	3.59E-01	3.12	19.51	8.73E-01	21.18	11.52	6.58E-02	21.89	18.28	2.31E-01
cg17262025	-34.07	15.46	2.75E-02	-31.30	40.86	4.44E-01	147.63	192.59	4.43E-01	-37.57	17.53	3.21E-02	-18.26	57.30	7.50E-01
cg26859042	14.40	6.53	2.75E-02	7.62	11.40	5.04E-01	11.51	16.43	4.84E-01	27.03	11.47	1.84E-02	6.88	15.05	6.48E-01
cg14854112	-10.54	4.78	2.75E-02	-21.30	10.42	4.08E-02	-4.79	12.71	7.06E-01	-9.15	6.95	1.88E-01	-5.98	11.43	6.01E-01
cg07284273	-9.03	4.10	2.75E-02	-6.53	6.09	2.83E-01	-11.71	8.27	1.57E-01	0.54	12.01	9.64E-01	-17.54	9.50	6.49E-02
cg11885098	-11.27	5.11	2.75E-02	-5.50	7.65	4.72E-01	-14.10	14.40	3.27E-01	-10.48	10.90	3.37E-01	-22.88	11.25	4.20E-02
cg12894449	13.88	6.30	2.75E-02	9.71	9.79	3.21E-01	29.38	15.05	5.09E-02	34.57	30.91	2.63E-01	8.87	10.36	3.92E-01
cg12211557	16.63	7.55	2.75E-02	34.14	15.34	2.60E-02	-8.83	22.33	6.92E-01	17.73	9.56	6.37E-02	0.29	22.51	9.90E-01
cg16362480	10.03	4.55	2.75E-02	11.55	7.61	1.29E-01	6.35	9.89	5.21E-01	11.26	9.59	2.40E-01	9.83	10.05	3.28E-01
cg09662823	16.24	7.37	2.75E-02	24.84	16.08	1.22E-01	2.52	19.15	8.95E-01	11.48	9.86	2.44E-01	50.62	25.46	4.68E-02
cg14121971	-23.19	10.53	2.76E-02	-12.93	13.71	3.46E-01	-19.19	26.82	4.74E-01	-14.28	47.81	7.65E-01	-57.28	23.07	1.30E-02
cg13229972	8.14	3.69	2.76E-02	7.76	4.59	9.14E-02	17.94	9.93	7.08E-02	4.39	11.52	7.03E-01	1.74	10.96	8.74E-01
cg24208206	-29.83	13.54	2.76E-02	-42.28	17.79	1.75E-02	19.37	65.45	7.67E-01	31.10	57.87	5.91E-01	-24.35	23.80	3.06E-01
cg12568669	6.26	2.84	2.76E-02	5.57	3.90	1.54E-01	-2.08	6.48	7.49E-01	14.06	6.21	2.36E-02	7.72	6.21	2.14E-01
cg02513485	18.07	8.20	2.76E-02	25.99	11.93	2.93E-02	22.92	20.71	2.69E-01	32.01	25.60	2.11E-01	-4.08	15.85	7.97E-01
cg00622010	16.15	7.33	2.76E-02	9.54	10.92	3.82E-01	30.32	15.10	4.47E-02	-0.13	22.50	9.95E-01	22.75	16.08	1.57E-01

cg22361291	23.71	10.76	2.76E-02	22.55	41.35	5.86E-01	-24.72	160.65	8.78E-01	25.64	11.48	2.55E-02	-5.24	49.01	9.15E-01
cg25162473	17.77	8.07	2.76E-02	25.49	14.97	8.86E-02	0.30	23.55	9.90E-01	13.08	12.09	2.79E-01	30.63	21.01	1.45E-01
cg27134645	-14.48	6.57	2.76E-02	-22.56	11.14	4.28E-02	-1.38	18.73	9.41E-01	-8.71	10.15	3.91E-01	-25.49	19.81	1.98E-01
cg09182435	-13.29	6.03	2.76E-02	-12.07	9.73	2.15E-01	-15.56	13.74	2.57E-01	-13.33	13.24	3.14E-01	-13.38	12.98	3.03E-01
cg08797423	-14.00	6.36	2.76E-02	-20.81	9.97	3.68E-02	3.30	15.99	8.37E-01	-15.89	14.78	2.82E-01	-12.47	12.71	3.26E-01
cg01466011	-7.31	3.32	2.76E-02	-10.70	5.06	3.43E-02	-2.39	6.77	7.24E-01	-2.28	7.65	7.65E-01	-12.05	8.84	1.73E-01
cg12777730	-12.38	5.62	2.76E-02	-6.06	13.73	6.59E-01	-2.87	23.35	9.02E-01	-13.83	6.77	4.10E-02	-19.54	19.26	3.10E-01
cg25256661	-15.90	7.22	2.76E-02	-17.73	10.71	9.78E-02	-4.82	23.07	8.35E-01	60.17	46.15	1.92E-01	-20.43	9.69	3.49E-02
cg08764037	10.50	4.77	2.76E-02	10.19	7.21	1.58E-01	17.71	11.57	1.26E-01	-1.06	10.33	9.18E-01	18.14	11.23	1.06E-01
cg17546247	10.22	4.64	2.76E-02	8.72	7.65	2.55E-01	18.56	11.77	1.15E-01	10.21	10.08	3.11E-01	7.42	9.02	4.10E-01
cg01249187	-8.54	3.88	2.76E-02	-11.82	5.17	2.22E-02	-1.66	9.70	8.64E-01	-9.31	14.35	5.16E-01	-4.63	8.57	5.89E-01
cg03440944	-11.29	5.12	2.76E-02	-9.13	9.58	3.41E-01	-19.81	11.76	9.22E-02	-1.60	8.72	8.55E-01	-24.41	12.11	4.39E-02
cg19328764	-49.65	22.54	2.76E-02	-59.38	28.06	3.43E-02	-3.61	94.84	9.70E-01	-3.22	78.10	9.67E-01	-50.52	48.59	2.98E-01
cg24876404	-7.61	3.45	2.76E-02	-6.09	7.31	4.05E-01	-20.05	9.99	4.48E-02	-5.56	4.67	2.34E-01	-7.34	10.34	4.78E-01
cg19980771	4.91	2.23	2.76E-02	6.26	4.00	1.18E-01	9.26	6.48	1.53E-01	5.07	4.05	2.11E-01	1.25	4.30	7.72E-01
cg17018896	16.75	7.61	2.76E-02	4.97	14.89	7.39E-01	34.44	16.74	3.97E-02	20.32	16.57	2.20E-01	12.63	13.40	3.46E-01
cg03027244	13.09	5.94	2.76E-02	13.21	8.16	1.06E-01	15.00	21.15	4.78E-01	21.84	22.72	3.36E-01	10.57	10.46	3.12E-01
cg00620449	-27.94	12.68	2.76E-02	-35.63	32.92	2.79E-01	51.28	98.90	6.04E-01	-26.97	14.72	6.70E-02	-37.43	41.63	3.69E-01
cg22253838	23.89	10.84	2.76E-02	47.96	40.58	2.37E-01	7.85	59.07	8.94E-01	20.43	12.28	9.61E-02	37.12	32.02	2.46E-01
cg14018153	8.67	3.94	2.76E-02	13.13	5.99	2.83E-02	8.04	10.56	4.46E-01	5.58	8.32	5.03E-01	3.08	8.69	7.23E-01
cg00587628	20.22	9.18	2.76E-02	55.29	25.49	3.01E-02	0.73	36.60	9.84E-01	15.23	10.85	1.60E-01	22.89	30.29	4.50E-01
cg12911476	51.00	23.16	2.76E-02	52.25	29.71	7.87E-02	-32.70	108.82	7.64E-01	33.48	65.19	6.08E-01	74.72	49.24	1.29E-01
cg15893127	19.72	8.95	2.76E-02	32.35	22.92	1.58E-01	-45.49	89.85	6.13E-01	15.56	10.17	1.26E-01	50.33	35.59	1.57E-01
cg11534596	-10.68	4.85	2.76E-02	-15.27	6.79	2.45E-02	-2.79	10.69	7.94E-01	-25.29	15.97	1.13E-01	0.07	11.05	9.95E-01
cg08923379	-42.77	19.42	2.76E-02	-115.46	60.77	5.75E-02	19.19	231.91	9.34E-01	-29.76	21.54	1.67E-01	-88.48	69.37	2.02E-01
cg05083033	11.97	5.43	2.76E-02	26.03	12.88	4.34E-02	50.77	38.18	1.84E-01	7.56	6.72	2.61E-01	9.24	14.12	5.13E-01
cg16696727	15.03	6.82	2.76E-02	18.85	13.02	1.48E-01	7.76	16.51	6.38E-01	11.75	10.04	2.42E-01	33.50	22.44	1.35E-01
cg10067723	8.59	3.90	2.76E-02	10.52	8.09	1.93E-01	17.91	7.94	2.41E-02	0.07	8.13	9.93E-01	6.11	7.16	3.94E-01
cg00417421	21.10	9.58	2.76E-02	39.04	17.70	2.74E-02	6.25	25.35	8.05E-01	13.46	16.08	4.02E-01	19.08	20.96	3.63E-01
cg00734436	-13.75	6.25	2.76E-02	-28.13	13.84	4.22E-02	4.01	19.90	8.40E-01	-11.23	8.23	1.72E-01	-16.06	17.91	3.70E-01
cg01612140	8.51	3.86	2.76E-02	10.06	5.33	5.91E-02	13.05	8.72	1.35E-01	3.42	13.20	7.95E-01	1.93	8.79	8.27E-01
cg19505767	11.78	5.35	2.76E-02	-2.53	8.83	7.74E-01	12.77	11.91	2.83E-01	18.53	6.93	7.48E-03	18.37	11.68	1.16E-01
cg06480736	21.56	9.79	2.76E-02	7.63	26.45	7.73E-01	-49.98	72.76	4.92E-01	23.22	11.21	3.82E-02	45.44	34.35	1.86E-01
cg21295467	-22.43	10.19	2.76E-02	-27.97	13.51	3.84E-02	1.96	44.80	9.65E-01	89.31	82.03	2.76E-01	-21.98	16.87	1.93E-01
cg26119740	-19.83	9.01	2.76E-02	-8.24	15.84	6.03E-01	-45.79	20.17	2.32E-02	-25.01	14.52	8.50E-02	0.84	22.17	9.70E-01

cg06470727	-10.77	4.89	2.77E-02	-7.83	7.45	2.93E-01	-15.18	11.37	1.82E-01	-11.73	11.93	3.26E-01	-12.11	10.52	2.50E-01
cg06028247	8.81	4.00	2.77E-02	4.87	6.73	4.69E-01	10.24	10.71	3.39E-01	7.11	7.36	3.33E-01	16.85	8.71	5.31E-02
cg24720038	-15.47	7.03	2.77E-02	-3.30	9.16	7.19E-01	-23.59	15.78	1.35E-01	-15.40	19.48	4.29E-01	-30.84	12.82	1.62E-02
cg19148201	7.19	3.27	2.77E-02	7.03	4.24	9.71E-02	5.06	6.96	4.67E-01	12.96	13.09	3.22E-01	8.85	9.29	3.40E-01
cg12350196	7.20	3.27	2.77E-02	7.00	5.16	1.75E-01	7.32	5.65	1.95E-01	7.22	11.33	5.24E-01	7.43	7.71	3.35E-01
cg25013443	-8.39	3.81	2.77E-02	-9.26	7.99	2.46E-01	19.25	19.96	3.35E-01	-7.93	5.08	1.19E-01	-14.53	9.14	1.12E-01
cg25996061	15.53	7.05	2.77E-02	1.06	20.31	9.58E-01	6.25	23.43	7.90E-01	16.31	8.38	5.17E-02	40.50	24.73	1.01E-01
cg10862399	-9.17	4.17	2.77E-02	-14.07	8.53	9.89E-02	-16.49	13.38	2.18E-01	-4.09	5.73	4.75E-01	-15.16	11.32	1.81E-01
cg19902173	-13.72	6.23	2.77E-02	-27.04	13.36	4.29E-02	-35.72	17.55	4.17E-02	-8.14	5.87	1.66E-01	-3.85	12.55	7.59E-01
cg15665646	28.02	12.73	2.77E-02	40.25	18.43	2.90E-02	-20.34	29.16	4.85E-01	44.79	28.61	1.17E-01	30.78	21.46	1.52E-01
cg11998932	-6.19	2.81	2.77E-02	-5.94	4.46	1.83E-01	0.28	5.47	9.60E-01	-8.85	5.54	1.10E-01	-16.45	8.56	5.48E-02
cg07275207	17.99	8.17	2.77E-02	27.55	13.97	4.87E-02	3.87	17.28	8.23E-01	30.77	15.20	4.30E-02	-3.94	19.82	8.43E-01
cg00536939	7.78	3.53	2.77E-02	7.41	4.57	1.05E-01	16.11	8.16	4.82E-02	16.52	16.98	3.31E-01	-2.17	8.48	7.98E-01
cg12023589	11.72	5.32	2.77E-02	5.87	13.18	6.56E-01	-36.78	35.63	3.02E-01	13.21	6.24	3.45E-02	22.70	18.00	2.07E-01
cg00554993	7.98	3.62	2.77E-02	4.08	3.49	2.42E-01	13.00	7.15	6.89E-02	23.63	10.82	2.90E-02	4.62	8.07	5.67E-01
cg08173915	5.32	2.41	2.77E-02	4.60	3.07	1.34E-01	11.34	6.11	6.35E-02	4.07	6.77	5.47E-01	1.82	7.74	8.14E-01
cg06552160	-11.89	5.40	2.77E-02	-5.75	9.62	5.50E-01	-16.92	12.44	1.74E-01	-12.25	10.22	2.31E-01	-15.95	11.58	1.68E-01
cg02025157	46.21	20.99	2.77E-02	46.26	26.55	8.15E-02	23.72	69.08	7.31E-01	73.06	61.87	2.38E-01	39.99	51.31	4.36E-01
cg22245615	19.93	9.05	2.77E-02	43.36	17.12	1.13E-02	6.82	19.10	7.21E-01	10.20	14.85	4.92E-01	19.94	25.70	4.38E-01
cg09934782	16.32	7.41	2.77E-02	14.17	15.55	3.62E-01	39.21	31.10	2.07E-01	14.82	9.13	1.04E-01	19.35	31.09	5.34E-01
cg07029862	28.64	13.01	2.77E-02	36.51	45.60	4.23E-01	-44.55	156.06	7.75E-01	31.22	14.02	2.59E-02	-18.69	58.40	7.49E-01
cg20712820	-10.87	4.94	2.77E-02	-16.04	9.32	8.53E-02	-25.73	17.95	1.52E-01	-7.07	6.80	2.99E-01	-5.98	14.53	6.81E-01
cg01811559	23.87	10.85	2.77E-02	32.76	21.66	1.30E-01	-24.10	49.42	6.26E-01	22.86	14.38	1.12E-01	28.85	29.84	3.34E-01
cg05361459	-7.71	3.50	2.77E-02	-7.18	7.28	3.24E-01	-15.42	11.92	1.96E-01	-9.68	4.83	4.52E-02	2.31	8.82	7.94E-01
cg21172497	-7.87	3.57	2.77E-02	-10.21	8.14	2.09E-01	-13.51	15.66	3.88E-01	-7.19	4.42	1.04E-01	-4.83	11.30	6.69E-01
cg13880779	16.03	7.28	2.77E-02	14.57	17.16	3.96E-01	-17.18	74.84	8.18E-01	15.80	8.40	6.00E-02	29.04	30.22	3.37E-01
cg17907057	11.77	5.35	2.77E-02	22.03	13.14	9.36E-02	-2.91	10.69	7.86E-01	13.36	7.41	7.14E-02	24.49	17.90	1.71E-01
cg11100465	-12.39	5.63	2.77E-02	-13.80	8.24	9.41E-02	5.37	14.22	7.06E-01	-13.97	15.07	3.54E-01	-20.44	11.57	7.72E-02
cg19928195	-14.43	6.56	2.78E-02	-24.62	8.01	2.10E-03	0.97	13.33	9.42E-01	0.91	14.57	9.50E-01	-20.15	10.04	4.47E-02
cg10935720	27.75	12.61	2.78E-02	43.56	39.78	2.74E-01	91.47	195.32	6.40E-01	29.69	13.94	3.32E-02	-16.93	45.47	7.10E-01
cg21341821	5.44	2.47	2.78E-02	6.68	3.35	4.63E-02	7.70	4.80	1.08E-01	-1.79	11.56	8.77E-01	-1.03	6.46	8.74E-01
cg09554951	9.43	4.29	2.78E-02	10.27	8.99	2.53E-01	6.75	9.55	4.80E-01	11.77	8.57	1.70E-01	8.70	7.56	2.50E-01
cg06070002	-8.14	3.70	2.78E-02	-3.28	7.04	6.41E-01	-6.63	9.67	4.93E-01	-11.66	5.42	3.14E-02	-7.42	11.09	5.03E-01
cg26159077	-29.40	13.36	2.78E-02	-91.50	44.37	3.92E-02	-16.54	191.26	9.31E-01	-21.33	14.88	1.52E-01	-38.92	42.58	3.61E-01
cg10683503	31.90	14.50	2.78E-02	37.04	20.75	7.43E-02	30.04	41.77	4.72E-01	9.30	15.34	5.44E-01	78.26	30.57	1.05E-02

cg07531056	-21.00	9.54	2.78E-02	-15.03	15.44	3.30E-01	-11.45	22.42	6.09E-01	-37.70	22.66	9.61E-02	-25.08	18.76	1.81E-01
cg12832726	9.49	4.31	2.78E-02	9.74	6.30	1.23E-01	19.56	8.94	2.88E-02	4.98	13.58	7.14E-01	-0.57	9.66	9.53E-01
cg04857420	13.95	6.34	2.78E-02	14.81	12.90	2.51E-01	4.36	24.06	8.56E-01	16.10	9.58	9.28E-02	12.01	12.64	3.42E-01
cg19483465	-13.62	6.19	2.78E-02	-12.84	8.98	1.53E-01	15.89	24.63	5.19E-01	-32.71	15.08	3.01E-02	-10.81	10.40	2.98E-01
cg16489468	-11.36	5.16	2.78E-02	5.76	13.82	6.77E-01	2.75	20.24	8.92E-01	-14.64	6.03	1.51E-02	-26.03	20.83	2.12E-01
cg18456933	6.37	2.89	2.78E-02	7.37	5.03	1.42E-01	8.82	7.40	2.33E-01	6.32	4.55	1.64E-01	0.11	8.72	9.90E-01
cg14477581	-9.28	4.22	2.78E-02	-12.57	7.58	9.73E-02	-18.23	14.11	1.96E-01	-6.86	6.20	2.69E-01	-4.27	11.28	7.05E-01
cg11801629	-32.86	14.93	2.78E-02	-48.90	46.02	2.88E-01	76.33	221.99	7.31E-01	-28.76	16.57	8.26E-02	-60.44	53.61	2.60E-01
cg16643558	20.61	9.37	2.78E-02	17.50	16.77	2.97E-01	9.87	17.52	5.73E-01	27.60	19.24	1.51E-01	35.09	23.07	1.28E-01
cg13701159	32.39	14.72	2.78E-02	40.41	18.78	3.14E-02	25.86	47.94	5.90E-01	42.72	62.15	4.92E-01	11.56	30.37	7.04E-01
cg23934071	-13.72	6.24	2.78E-02	-13.60	8.84	1.24E-01	-27.08	14.18	5.62E-02	-6.02	19.74	7.61E-01	-5.35	13.63	6.95E-01
cg17416748	35.79	16.26	2.78E-02	18.83	22.77	4.08E-01	37.21	54.95	4.98E-01	63.36	42.42	1.35E-01	53.35	32.21	9.77E-02
cg16366843	15.34	6.97	2.78E-02	41.10	29.43	1.63E-01	41.81	47.89	3.83E-01	12.98	8.07	1.08E-01	13.92	16.54	4.00E-01
cg16290335	27.44	12.47	2.78E-02	13.41	18.11	4.59E-01	71.65	41.07	8.11E-02	54.77	29.24	6.10E-02	17.91	24.84	4.71E-01
cg23685759	20.87	9.49	2.78E-02	-24.38	29.66	4.11E-01	78.73	79.59	3.23E-01	25.22	10.34	1.47E-02	24.40	26.86	3.64E-01
cg17795540	20.28	9.22	2.78E-02	5.27	17.25	7.60E-01	10.02	18.64	5.91E-01	37.41	17.16	2.93E-02	30.45	21.62	1.59E-01
cg19165216	-11.55	5.25	2.78E-02	-5.53	8.42	5.12E-01	-15.92	12.81	2.14E-01	-8.86	14.07	5.29E-01	-18.05	9.51	5.78E-02
cg23803223	-8.46	3.85	2.78E-02	-11.18	6.39	8.01E-02	-4.26	7.56	5.73E-01	0.26	8.98	9.77E-01	-17.17	8.70	4.84E-02
cg16570129	-12.73	5.79	2.78E-02	-18.46	8.22	2.48E-02	-18.64	16.04	2.45E-01	-7.52	13.26	5.71E-01	1.49	13.51	9.12E-01
cg16706427	30.70	13.95	2.78E-02	33.40	32.67	3.07E-01	-33.75	129.42	7.94E-01	34.62	16.27	3.34E-02	-6.63	52.60	9.00E-01
cg02536065	10.01	4.55	2.78E-02	10.06	8.13	2.16E-01	23.37	10.43	2.50E-02	9.17	5.38	8.85E-02	-9.72	14.32	4.98E-01
cg07566080	15.32	6.96	2.78E-02	45.76	20.16	2.32E-02	15.77	27.99	5.73E-01	12.76	8.59	1.37E-01	3.04	17.35	8.61E-01
cg27297576	12.72	5.78	2.78E-02	25.71	11.33	2.32E-02	11.42	29.00	6.94E-01	8.02	7.62	2.92E-01	7.65	16.40	6.41E-01
cg25284762	17.53	7.97	2.78E-02	4.29	13.91	7.58E-01	19.81	15.06	1.89E-01	32.35	15.77	4.03E-02	16.98	21.56	4.31E-01
cg17279458	5.39	2.45	2.78E-02	2.93	3.96	4.59E-01	5.81	6.03	3.36E-01	10.72	4.33	1.33E-02	-0.95	6.75	8.88E-01
cg04932082	6.57	2.99	2.78E-02	6.81	3.86	7.78E-02	16.23	8.89	6.80E-02	2.88	8.16	7.24E-01	1.80	7.57	8.12E-01
cg20269160	-10.11	4.60	2.78E-02	-27.07	12.10	2.53E-02	-17.15	18.03	3.41E-01	-6.23	5.40	2.48E-01	-8.77	18.06	6.27E-01
cg01272627	20.47	9.31	2.78E-02	30.97	20.77	1.36E-01	25.76	20.81	2.16E-01	17.57	13.37	1.89E-01	5.13	27.47	8.52E-01
cg15000071	-13.34	6.07	2.78E-02	-23.06	9.09	1.12E-02	0.53	17.52	9.76E-01	-5.44	14.56	7.09E-01	-8.41	11.87	4.79E-01
cg24025721	6.47	2.94	2.78E-02	7.75	3.64	3.32E-02	10.62	7.89	1.78E-01	-1.02	9.84	9.18E-01	0.14	8.60	9.87E-01
cg09881253	48.00	21.83	2.79E-02	48.99	30.09	1.03E-01	153.71	104.23	1.40E-01	-23.91	64.37	7.10E-01	57.88	38.89	1.37E-01
cg18646186	18.67	8.49	2.79E-02	43.65	19.82	2.76E-02	2.69	22.84	9.06E-01	11.93	10.06	2.36E-01	42.12	29.85	1.58E-01
cg23917868	11.53	5.24	2.79E-02	9.41	7.44	2.06E-01	24.80	12.18	4.18E-02	17.98	12.93	1.65E-01	-4.44	13.35	7.39E-01
cg22574586	16.12	7.33	2.79E-02	17.89	12.41	1.49E-01	5.14	14.71	7.27E-01	10.21	7.48	1.72E-01	49.89	18.97	8.55E-03
cg06386482	2.72	1.24	2.79E-02	2.41	1.72	1.60E-01	0.38	2.80	8.93E-01	5.47	3.44	1.13E-01	4.32	3.08	1.61E-01

cg08759041	-4.67	2.13	2.79E-02	-7.15	3.17	2.41E-02	-7.41	4.96	1.35E-01	1.18	7.41	8.73E-01	-0.69	3.98	8.63E-01
cg13069237	17.10	7.78	2.79E-02	28.49	16.76	8.91E-02	21.21	19.96	2.88E-01	6.70	11.10	5.46E-01	31.42	20.64	1.28E-01
cg16545773	18.39	8.36	2.79E-02	41.59	21.99	5.86E-02	10.08	21.78	6.44E-01	11.64	10.60	2.72E-01	42.61	28.61	1.36E-01
cg10757679	18.76	8.53	2.79E-02	23.05	14.95	1.23E-01	21.51	17.95	2.31E-01	4.80	15.35	7.55E-01	35.20	22.83	1.23E-01
cg05359130	17.16	7.81	2.79E-02	24.42	10.41	1.90E-02	18.51	29.87	5.35E-01	-25.94	35.70	4.67E-01	10.61	13.77	4.41E-01
cg26230516	19.09	8.68	2.79E-02	7.62	27.47	7.81E-01	25.04	52.62	6.34E-01	18.02	9.76	6.49E-02	41.33	30.28	1.72E-01
cg16705578	21.38	9.72	2.79E-02	2.35	38.21	9.51E-01	114.79	136.50	4.00E-01	25.47	10.37	1.40E-02	-34.23	43.04	4.26E-01
cg24476449	-9.01	4.10	2.79E-02	-14.44	5.61	1.01E-02	-1.58	9.72	8.71E-01	-13.52	14.15	3.39E-01	0.50	9.05	9.56E-01
cg01344171	9.91	4.51	2.79E-02	9.56	6.22	1.24E-01	10.90	10.04	2.77E-01	12.36	13.20	3.49E-01	7.99	11.38	4.83E-01
cg23189044	9.53	4.34	2.79E-02	8.34	6.35	1.90E-01	19.79	9.79	4.33E-02	10.64	12.17	3.82E-01	1.99	9.43	8.33E-01
cg06867755	5.47	2.49	2.79E-02	4.64	3.27	1.56E-01	14.65	7.17	4.10E-02	7.29	6.61	2.70E-01	-0.09	6.25	9.89E-01
cg02089348	-16.03	7.29	2.79E-02	-16.64	10.25	1.05E-01	1.20	14.98	9.36E-01	-43.13	19.28	2.53E-02	-15.18	14.70	3.02E-01
cg07613391	-6.94	3.15	2.79E-02	-5.34	4.26	2.10E-01	-17.26	8.02	3.14E-02	2.92	10.69	7.85E-01	-7.58	6.88	2.70E-01
cg01437135	-12.32	5.60	2.79E-02	-21.80	9.77	2.57E-02	-6.27	13.57	6.44E-01	-2.45	10.32	8.12E-01	-16.32	12.35	1.86E-01
cg13715798	13.18	6.00	2.79E-02	9.48	9.20	3.03E-01	3.57	13.05	7.85E-01	21.70	14.76	1.41E-01	24.25	13.45	7.15E-02
cg15010903	5.04	2.29	2.79E-02	3.10	2.94	2.91E-01	11.67	5.28	2.71E-02	7.53	4.64	1.05E-01	-1.59	6.58	8.10E-01
cg00638075	38.05	17.31	2.79E-02	55.34	42.72	1.95E-01	-158.67	173.26	3.60E-01	34.27	19.95	8.58E-02	64.76	63.81	3.10E-01
cg14786024	18.75	8.53	2.79E-02	35.44	31.90	2.67E-01	29.38	34.77	3.98E-01	19.96	9.89	4.35E-02	-3.25	24.21	8.93E-01
cg01805215	12.08	5.49	2.79E-02	12.32	8.55	1.50E-01	23.04	12.92	7.45E-02	4.67	17.60	7.91E-01	7.67	9.89	4.38E-01
cg08439890	-9.65	4.39	2.79E-02	-16.78	9.02	6.27E-02	-4.67	12.12	7.00E-01	-6.14	6.06	3.11E-01	-17.25	13.45	2.00E-01
cg26920627	-12.00	5.46	2.79E-02	-21.64	10.83	4.58E-02	-13.08	14.02	3.51E-01	-8.26	8.17	3.11E-01	-5.63	14.19	6.92E-01
cg13539030	-42.04	19.12	2.79E-02	-62.43	52.95	2.38E-01	17.31	217.63	9.37E-01	-40.81	21.90	6.24E-02	-29.33	60.55	6.28E-01
cg04217140	-19.10	8.69	2.79E-02	-13.52	23.47	5.64E-01	108.96	88.18	2.17E-01	-19.99	9.91	4.37E-02	-34.70	29.84	2.45E-01
cg24968692	13.09	5.95	2.79E-02	10.51	10.40	3.13E-01	-2.41	15.11	8.73E-01	15.27	9.13	9.46E-02	38.18	19.56	5.10E-02
cg17550566	8.83	4.02	2.79E-02	6.80	5.02	1.75E-01	23.22	9.14	1.11E-02	7.18	10.36	4.89E-01	1.38	9.23	8.81E-01
cg14265868	41.58	18.92	2.79E-02	55.04	23.25	1.79E-02	-8.80	96.30	9.27E-01	47.40	56.79	4.04E-01	1.27	43.55	9.77E-01
cg06097077	-9.60	4.37	2.79E-02	-4.60	8.38	5.83E-01	-10.13	19.85	6.10E-01	-10.75	5.93	7.00E-02	-14.79	11.78	2.09E-01
cg26909564	13.36	6.08	2.79E-02	-9.26	17.33	5.93E-01	14.02	26.15	5.92E-01	14.88	6.98	3.30E-02	38.40	24.07	1.11E-01
cg11987068	-15.69	7.14	2.79E-02	-20.34	12.10	9.26E-02	-4.97	17.05	7.71E-01	-15.34	15.00	3.06E-01	-17.05	14.29	2.33E-01
cg23343066	-12.83	5.84	2.79E-02	-11.34	9.88	2.51E-01	-20.13	12.76	1.15E-01	-6.63	15.48	6.68E-01	-12.40	10.66	2.45E-01
cg17356964	-35.57	16.18	2.80E-02	-47.81	20.82	2.17E-02	-54.87	69.24	4.28E-01	-29.11	49.01	5.53E-01	-2.24	33.58	9.47E-01
cg07794981	-8.10	3.69	2.80E-02	-4.46	8.21	5.87E-01	6.73	14.15	6.34E-01	-8.65	4.79	7.09E-02	-18.31	9.90	6.45E-02
cg05053818	13.27	6.04	2.80E-02	19.55	9.95	4.93E-02	1.79	15.66	9.09E-01	12.84	10.61	2.26E-01	10.31	15.14	4.96E-01
cg17331566	16.65	7.58	2.80E-02	6.86	11.32	5.44E-01	24.66	14.70	9.35E-02	15.24	21.62	4.81E-01	31.47	18.71	9.25E-02
cg26057380	-62.17	28.29	2.80E-02	-133.94	55.94	1.67E-02	-36.30	265.21	8.91E-01	-35.98	36.33	3.22E-01	-44.93	79.42	5.72E-01

cg27457191	-8.68	3.95	2.80E-02	-10.85	5.74	5.88E-02	-5.01	9.68	6.05E-01	-7.95	9.64	4.10E-01	-7.17	9.01	4.26E-01
cg19392831	16.42	7.47	2.80E-02	18.01	11.77	1.26E-01	12.09	19.57	5.37E-01	31.74	16.08	4.84E-02	2.32	15.42	8.81E-01
cg06298740	5.99	2.72	2.80E-02	5.54	3.54	1.17E-01	14.08	7.75	6.93E-02	4.06	7.51	5.89E-01	2.82	6.99	6.87E-01
cg26578983	6.67	3.03	2.80E-02	7.05	4.15	8.94E-02	14.84	6.85	3.04E-02	-1.43	10.19	8.89E-01	0.67	7.11	9.25E-01
cg16080125	8.73	3.97	2.80E-02	9.60	5.43	7.72E-02	12.76	8.15	1.17E-01	18.32	15.22	2.29E-01	-4.36	9.98	6.62E-01
cg23878577	-13.54	6.16	2.80E-02	-1.60	13.49	9.05E-01	-10.03	18.00	5.77E-01	-20.55	8.11	1.12E-02	-1.64	19.85	9.34E-01
cg22578843	25.40	11.56	2.80E-02	52.46	49.34	2.88E-01	-53.50	165.44	7.46E-01	20.78	12.57	9.83E-02	54.96	37.52	1.43E-01
cg27166993	20.81	9.47	2.80E-02	58.53	23.36	1.22E-02	38.65	46.23	4.03E-01	13.41	9.65	1.65E-01	12.27	17.69	4.88E-01
cg08450280	33.06	15.04	2.80E-02	32.42	19.11	8.98E-02	8.26	45.54	8.56E-01	113.36	56.51	4.48E-02	20.13	33.62	5.49E-01
cg08212913	19.07	8.68	2.80E-02	23.98	12.85	6.20E-02	18.11	16.51	2.73E-01	15.91	24.00	5.07E-01	7.64	23.50	7.45E-01
cg03317463	-37.35	16.99	2.80E-02	-39.02	22.25	7.95E-02	-42.39	50.85	4.05E-01	-51.50	62.56	4.10E-01	-26.17	35.34	4.59E-01
cg21151963	-10.57	4.81	2.80E-02	-34.54	15.84	2.92E-02	-9.83	27.83	7.24E-01	-9.49	5.54	8.69E-02	0.40	13.57	9.77E-01
cg02369052	-10.72	4.88	2.80E-02	-21.05	8.79	1.66E-02	-10.33	11.59	3.73E-01	-5.62	8.64	5.15E-01	-3.12	11.03	7.78E-01
cg01293740	-9.44	4.30	2.80E-02	-5.54	6.14	3.67E-01	-21.45	11.01	5.13E-02	-16.47	11.35	1.47E-01	-5.12	9.27	5.80E-01
cg10446356	22.12	10.07	2.80E-02	38.95	19.01	4.05E-02	47.97	54.17	3.76E-01	15.07	13.66	2.70E-01	9.57	26.70	7.20E-01
cg01479911	29.31	13.34	2.80E-02	33.07	38.61	3.92E-01	59.95	153.04	6.95E-01	30.97	14.84	3.69E-02	-1.84	52.31	9.72E-01
cg22127848	6.07	2.76	2.80E-02	7.00	4.44	1.15E-01	5.38	7.55	4.77E-01	7.54	4.49	9.29E-02	-2.22	8.77	8.00E-01
cg00371568	-32.97	15.00	2.80E-02	-29.79	17.65	9.14E-02	-182.25	97.56	6.18E-02	12.15	72.75	8.67E-01	-36.19	32.64	2.68E-01
cg20729460	-9.93	4.52	2.80E-02	-11.94	7.88	1.30E-01	-8.83	13.83	5.23E-01	-8.22	6.95	2.37E-01	-11.22	12.04	3.51E-01
cg19357849	-12.45	5.67	2.80E-02	-14.65	8.03	6.83E-02	-22.03	12.15	6.99E-02	14.39	25.17	5.68E-01	-4.71	11.70	6.88E-01
cg00311133	20.98	9.55	2.80E-02	8.40	19.19	6.62E-01	2.57	18.85	8.91E-01	35.87	15.54	2.10E-02	39.75	27.78	1.52E-01
cg02814805	8.33	3.79	2.80E-02	10.83	5.70	5.75E-02	-2.13	10.33	8.37E-01	2.27	8.93	7.99E-01	14.10	7.70	6.71E-02
cg17352975	25.14	11.44	2.80E-02	24.91	16.03	1.20E-01	24.47	30.02	4.15E-01	11.78	31.74	7.11E-01	34.21	24.68	1.66E-01
cg08469215	7.15	3.25	2.80E-02	8.68	4.02	3.09E-02	6.92	8.11	3.93E-01	21.44	14.23	1.32E-01	-4.49	8.52	5.99E-01
cg13872688	16.69	7.60	2.80E-02	46.58	27.54	9.08E-02	-107.15	103.28	3.00E-01	13.84	8.25	9.36E-02	28.32	28.61	3.22E-01
cg08170583	-12.88	5.86	2.80E-02	-26.39	10.04	8.57E-03	2.49	13.81	8.57E-01	-7.59	9.14	4.06E-01	-14.92	12.45	2.31E-01
cg05651688	-20.70	9.42	2.80E-02	-15.51	27.91	5.79E-01	-226.28	152.64	1.38E-01	-20.53	10.39	4.82E-02	-19.82	38.32	6.05E-01
cg23109129	24.35	11.08	2.80E-02	29.89	13.39	2.56E-02	-58.58	57.27	3.06E-01	21.32	58.93	7.17E-01	21.95	22.52	3.30E-01
cg27250841	14.43	6.57	2.80E-02	14.26	7.35	5.25E-02	47.31	30.42	1.20E-01	-43.49	57.82	4.52E-01	9.86	17.38	5.70E-01
cg05146544	9.35	4.26	2.80E-02	16.56	7.75	3.27E-02	1.73	8.92	8.46E-01	14.81	8.14	6.88E-02	0.07	9.35	9.94E-01
cg02058949	16.82	7.65	2.80E-02	29.94	23.66	2.06E-01	-70.25	74.05	3.43E-01	18.43	8.41	2.85E-02	-14.57	32.16	6.51E-01
cg00257659	-38.32	17.44	2.80E-02	-30.45	23.93	2.03E-01	27.26	63.64	6.68E-01	-60.33	42.31	1.54E-01	-62.30	36.88	9.12E-02
cg14059339	4.40	2.00	2.80E-02	4.78	2.71	7.79E-02	8.50	4.51	5.96E-02	3.38	4.98	4.97E-01	-4.47	6.46	4.89E-01
cg01630925	12.18	5.54	2.80E-02	4.89	14.77	7.41E-01	-1.94	31.65	9.51E-01	13.92	6.37	2.87E-02	14.10	20.94	5.01E-01
cg00880290	-32.34	14.72	2.80E-02	2.30	40.02	9.54E-01	-43.77	198.36	8.25E-01	-43.91	16.58	8.10E-03	30.96	55.26	5.75E-01

cg26966828	9.44	4.30	2.80E-02	13.02	7.23	7.17E-02	12.49	9.83	2.04E-01	12.88	9.46	1.74E-01	-0.83	8.62	9.23E-01
cg14355941	-11.72	5.34	2.81E-02	-4.53	14.72	7.58E-01	16.91	29.52	5.67E-01	-16.00	6.10	8.68E-03	8.10	20.08	6.87E-01
cg10281996	-28.59	13.02	2.81E-02	-34.34	16.47	3.70E-02	-30.65	45.88	5.04E-01	-1.07	43.27	9.80E-01	-22.39	28.81	4.37E-01
cg03115530	20.40	9.29	2.81E-02	8.19	15.68	6.02E-01	32.54	17.48	6.27E-02	29.89	19.74	1.30E-01	11.87	24.33	6.26E-01
cg02808075	-27.28	12.42	2.81E-02	-50.09	28.03	7.39E-02	-135.74	206.41	5.11E-01	-21.33	14.58	1.43E-01	-19.83	45.59	6.64E-01
cg24519867	-11.87	5.41	2.81E-02	-27.74	12.75	2.95E-02	-11.88	18.84	5.28E-01	-7.11	6.67	2.86E-01	-15.24	19.00	4.23E-01
cg02116251	-43.58	19.84	2.81E-02	-32.23	27.56	2.42E-01	-85.13	93.24	3.61E-01	-68.35	36.27	5.95E-02	-18.71	53.57	7.27E-01
cg05715828	-7.62	3.47	2.81E-02	-6.67	4.87	1.71E-01	-11.00	8.26	1.83E-01	-5.58	10.14	5.82E-01	-8.23	7.75	2.88E-01
cg01157070	14.74	6.71	2.81E-02	29.74	22.42	1.85E-01	6.83	77.13	9.29E-01	16.05	7.33	2.85E-02	-22.72	26.64	3.94E-01
cg24770856	-40.71	18.54	2.81E-02	-48.99	21.63	2.35E-02	-121.53	140.37	3.87E-01	78.45	86.85	3.66E-01	-30.55	41.19	4.58E-01
cg12858895	-9.21	4.19	2.81E-02	-11.73	11.34	3.01E-01	11.02	24.60	6.54E-01	-8.96	4.81	6.26E-02	-14.91	15.27	3.29E-01
cg21599324	-14.19	6.46	2.81E-02	-13.85	9.31	1.37E-01	-20.01	14.44	1.66E-01	-13.59	20.31	5.03E-01	-9.88	13.88	4.77E-01
cg12376402	36.50	16.62	2.81E-02	42.04	19.62	3.21E-02	-30.04	74.61	6.87E-01	130.18	70.28	6.40E-02	15.13	32.16	6.38E-01
cg22626169	5.75	2.62	2.81E-02	4.43	3.42	1.95E-01	12.24	6.52	6.05E-02	8.17	7.90	3.01E-01	1.96	7.01	7.80E-01
cg18269219	19.14	8.72	2.81E-02	3.69	15.73	8.15E-01	23.61	19.43	2.24E-01	18.43	14.42	2.01E-01	51.69	24.53	3.51E-02
cg13176012	13.50	6.15	2.81E-02	21.61	10.68	4.30E-02	4.65	16.64	7.80E-01	17.86	10.66	9.38E-02	-1.18	13.75	9.32E-01
cg09234995	-33.28	15.15	2.81E-02	-36.57	18.98	5.41E-02	-84.10	67.89	2.15E-01	63.96	73.51	3.84E-01	-31.43	29.13	2.81E-01
cg26559209	12.49	5.69	2.81E-02	19.48	8.28	1.86E-02	3.66	15.33	8.11E-01	25.00	16.97	1.41E-01	-0.07	10.78	9.95E-01
cg16707405	24.44	11.13	2.81E-02	18.76	25.40	4.60E-01	40.52	49.68	4.15E-01	32.40	14.04	2.10E-02	-11.96	30.92	6.99E-01
cg08907926	-16.12	7.34	2.81E-02	-16.56	11.33	1.44E-01	-38.25	19.96	5.53E-02	-5.10	5.16	3.23E-01	-30.18	14.41	3.63E-02
cg05717677	-31.77	14.47	2.81E-02	5.14	26.64	8.47E-01	-9.35	66.32	8.88E-01	-50.95	16.59	2.14E-03	-31.30	37.03	3.98E-01
cg26168772	13.10	5.97	2.81E-02	6.82	8.28	4.10E-01	21.09	16.31	1.96E-01	24.95	9.11	6.19E-03	-1.16	13.38	9.31E-01
cg21747496	-6.91	3.15	2.81E-02	-9.80	4.23	2.05E-02	-6.45	7.35	3.80E-01	12.36	15.53	4.26E-01	-3.65	6.69	5.86E-01
cg23271556	19.32	8.80	2.81E-02	31.49	15.94	4.82E-02	17.00	26.68	5.24E-01	15.10	15.67	3.35E-01	11.49	16.89	4.96E-01
cg13710553	8.05	3.67	2.81E-02	8.36	4.84	8.42E-02	15.52	9.54	1.04E-01	9.34	10.03	3.52E-01	-1.99	9.64	8.36E-01
cg09869811	26.68	12.15	2.81E-02	15.54	22.88	4.97E-01	39.42	52.38	4.52E-01	24.71	16.95	1.45E-01	49.71	31.32	1.12E-01
cg13186559	8.84	4.03	2.81E-02	9.72	5.63	8.44E-02	10.92	9.69	2.60E-01	8.61	10.15	3.96E-01	3.97	10.07	6.93E-01
cg05765605	3.51	1.60	2.81E-02	2.42	2.05	2.38E-01	5.06	3.29	1.25E-01	10.98	7.27	1.31E-01	2.92	4.86	5.48E-01
cg07398614	15.76	7.18	2.81E-02	-11.04	25.09	6.60E-01	18.72	44.94	6.77E-01	19.29	8.00	1.59E-02	7.56	24.11	7.54E-01
cg00975226	-12.35	5.62	2.81E-02	-8.71	10.69	4.15E-01	-3.53	15.82	8.23E-01	-11.37	8.26	1.69E-01	-31.70	15.42	3.98E-02
cg06027807	22.75	10.37	2.81E-02	24.48	14.36	8.83E-02	5.68	33.09	8.64E-01	43.71	32.29	1.76E-01	17.78	19.66	3.66E-01
cg09069593	19.70	8.97	2.81E-02	18.42	13.00	1.56E-01	28.15	24.09	2.43E-01	20.96	22.09	3.43E-01	16.18	19.13	3.98E-01
cg02972788	-24.22	11.03	2.81E-02	-64.64	44.57	1.47E-01	145.07	158.89	3.61E-01	-20.04	11.77	8.87E-02	-60.05	46.64	1.98E-01
cg00355784	7.87	3.58	2.81E-02	7.28	7.03	3.00E-01	-3.85	10.66	7.18E-01	8.35	4.89	8.76E-02	21.47	11.98	7.30E-02
cg22203776	19.11	8.70	2.81E-02	31.46	14.49	2.99E-02	1.14	22.97	9.60E-01	34.29	19.79	8.31E-02	3.17	15.84	8.41E-01

cg19981475	-12.42	5.66	2.81E-02	-14.62	11.00	1.84E-01	0.98	13.44	9.42E-01	-16.63	8.22	4.30E-02	-10.05	19.57	6.08E-01
cg06505423	-13.99	6.38	2.82E-02	-4.92	17.75	7.82E-01	-22.79	34.81	5.13E-01	-14.77	7.37	4.52E-02	-17.32	21.27	4.15E-01
cg15651650	15.83	7.21	2.82E-02	14.95	21.35	4.84E-01	113.41	102.07	2.67E-01	13.60	7.99	8.87E-02	37.29	27.96	1.82E-01
cg14468692	6.51	2.97	2.82E-02	6.56	3.88	9.10E-02	11.01	6.75	1.03E-01	7.91	11.44	4.89E-01	0.13	7.54	9.86E-01
cg06332339	102.84	46.85	2.82E-02	106.64	71.06	1.33E-01	171.73	147.13	2.43E-01	120.56	110.42	2.75E-01	61.17	87.93	4.87E-01
cg03922095	17.30	7.88	2.82E-02	9.25	20.19	6.47E-01	32.42	22.63	1.52E-01	16.35	9.65	9.04E-02	17.74	32.24	5.82E-01
cg23983783	8.59	3.91	2.82E-02	9.26	5.68	1.03E-01	15.34	10.01	1.26E-01	8.08	8.47	3.40E-01	0.76	9.84	9.39E-01
cg15814923	30.12	13.72	2.82E-02	44.36	43.81	3.11E-01	4.76	218.65	9.83E-01	32.15	14.94	3.14E-02	-24.97	58.73	6.71E-01
cg15475323	9.73	4.43	2.82E-02	10.12	8.56	2.37E-01	3.13	9.73	7.48E-01	7.70	7.37	2.96E-01	21.94	10.97	4.54E-02
cg11561737	-18.62	8.48	2.82E-02	12.95	40.13	7.47E-01	-114.25	164.79	4.88E-01	-19.34	8.98	3.13E-02	-27.20	34.53	4.31E-01
cg15571353	11.47	5.23	2.82E-02	9.27	8.73	2.88E-01	21.02	12.45	9.15E-02	13.22	9.64	1.70E-01	3.25	12.63	7.97E-01
cg16824319	12.44	5.67	2.82E-02	9.40	7.90	2.35E-01	12.31	12.59	3.28E-01	26.00	18.78	1.66E-01	14.30	12.94	2.69E-01
cg06773873	-16.76	7.64	2.82E-02	-20.30	15.35	1.86E-01	-4.55	15.55	7.70E-01	-13.67	12.52	2.75E-01	-39.85	20.47	5.15E-02
cg10347418	-18.38	8.38	2.82E-02	-6.97	21.10	7.41E-01	9.22	30.12	7.60E-01	-25.15	10.13	1.30E-02	-9.76	29.43	7.40E-01
cg21422623	-8.08	3.68	2.82E-02	-3.16	5.32	5.53E-01	-9.26	8.18	2.58E-01	-12.20	9.18	1.84E-01	-17.35	9.29	6.18E-02
cg07978099	6.69	3.05	2.82E-02	9.15	4.66	4.96E-02	9.94	6.99	1.55E-01	4.69	7.33	5.22E-01	0.36	6.66	9.57E-01
cg03933646	38.20	17.41	2.82E-02	59.27	22.19	7.55E-03	46.70	25.33	6.52E-02	4.98	10.50	6.35E-01	64.47	30.46	3.43E-02
cg18823499	-10.13	4.62	2.82E-02	-17.60	6.99	1.18E-02	-8.13	11.24	4.69E-01	-5.01	15.03	7.39E-01	-2.00	8.42	8.12E-01
cg05526498	9.27	4.22	2.82E-02	9.54	5.49	8.24E-02	14.64	9.45	1.22E-01	4.77	14.81	7.47E-01	2.49	11.80	8.33E-01
cg17873910	-29.91	13.63	2.82E-02	-33.87	18.88	7.29E-02	-48.58	42.69	2.55E-01	0.30	40.11	9.94E-01	-28.09	26.65	2.92E-01
cg16362378	-18.45	8.41	2.82E-02	-22.09	18.78	2.39E-01	24.04	49.86	6.30E-01	-20.32	10.16	4.56E-02	-9.23	28.57	7.47E-01
cg03889540	6.27	2.86	2.82E-02	4.46	4.03	2.69E-01	1.95	7.29	7.89E-01	12.49	8.35	1.35E-01	9.93	5.97	9.63E-02
cg06785701	-5.65	2.58	2.82E-02	-0.59	4.19	8.88E-01	-4.13	6.32	5.14E-01	-9.02	3.93	2.17E-02	-13.86	8.76	1.14E-01
cg23690528	11.72	5.34	2.82E-02	9.52	12.81	4.57E-01	29.89	14.82	4.36E-02	9.82	7.62	1.98E-01	6.62	11.81	5.75E-01
cg04215564	-16.20	7.38	2.82E-02	-30.22	14.50	3.72E-02	6.06	16.54	7.14E-01	-20.08	11.41	7.85E-02	-9.53	21.10	6.52E-01
cg07017437	-5.54	2.52	2.82E-02	-8.90	4.77	6.24E-02	-10.26	7.12	1.50E-01	-3.58	3.73	3.37E-01	-0.93	6.79	8.91E-01
cg23189194	6.00	2.73	2.82E-02	7.89	5.33	1.39E-01	1.43	5.75	8.04E-01	3.92	4.47	3.80E-01	15.66	7.41	3.45E-02
cg20864389	34.35	15.66	2.82E-02	44.52	21.81	4.13E-02	39.70	89.27	6.57E-01	68.12	58.74	2.46E-01	13.99	25.30	5.80E-01
cg18556676	11.68	5.32	2.82E-02	15.42	9.34	9.87E-02	11.57	14.72	4.32E-01	17.35	9.98	8.22E-02	0.86	10.43	9.34E-01
cg08462030	-10.60	4.83	2.82E-02	-21.63	9.70	2.58E-02	2.24	13.37	8.67E-01	-7.86	7.30	2.82E-01	-11.36	11.26	3.13E-01
cg24669856	-41.77	19.04	2.82E-02	-53.43	22.98	2.00E-02	-13.35	108.22	9.02E-01	-0.63	83.79	9.94E-01	-20.09	39.62	6.12E-01
cg12742209	19.86	9.05	2.82E-02	37.86	18.44	4.01E-02	21.07	20.88	3.13E-01	11.16	13.15	3.96E-01	15.27	28.97	5.98E-01
cg01035160	26.41	12.04	2.82E-02	18.52	18.53	3.18E-01	-11.34	54.38	8.35E-01	21.28	28.15	4.50E-01	44.06	20.45	3.12E-02
cg06759265	13.78	6.28	2.82E-02	18.56	9.51	5.10E-02	11.18	17.47	5.22E-01	13.44	15.37	3.82E-01	7.46	12.15	5.39E-01
cg14633456	20.22	9.22	2.82E-02	36.81	31.45	2.42E-01	-48.79	132.29	7.12E-01	19.47	10.02	5.21E-02	13.05	36.38	7.20E-01

cg06848073	19.73	8.99	2.82E-02	22.63	15.96	1.56E-01	29.12	16.60	7.95E-02	16.17	18.44	3.81E-01	1.06	23.14	9.64E-01
cg08493590	8.60	3.92	2.82E-02	6.31	5.16	2.21E-01	7.66	9.72	4.30E-01	14.98	16.67	3.69E-01	14.12	8.69	1.04E-01
cg00022688	-39.22	17.88	2.82E-02	-45.46	22.02	3.90E-02	-159.15	100.61	1.14E-01	10.67	49.05	8.28E-01	-32.05	42.58	4.52E-01
cg23435671	6.85	3.12	2.82E-02	4.05	4.64	3.83E-01	11.79	6.75	8.07E-02	8.04	8.36	3.37E-01	7.10	7.10	3.17E-01
cg03414898	8.44	3.85	2.82E-02	10.57	6.89	1.25E-01	2.28	11.13	8.37E-01	8.47	5.40	1.17E-01	9.32	15.36	5.44E-01
cg12576688	17.01	7.75	2.82E-02	12.38	17.46	4.78E-01	22.22	41.31	5.91E-01	13.89	9.36	1.38E-01	52.53	27.26	5.39E-02
cg01802453	18.55	8.46	2.83E-02	35.69	16.25	2.80E-02	1.56	21.89	9.43E-01	26.34	14.69	7.30E-02	1.84	16.05	9.09E-01
cg16551349	28.14	12.83	2.83E-02	27.52	15.19	7.01E-02	39.45	53.72	4.63E-01	11.08	12.66	3.81E-01	82.21	32.07	1.04E-02
cg16143997	-13.10	5.97	2.83E-02	-23.03	10.07	2.21E-02	-14.43	13.66	2.91E-01	-5.70	12.31	6.43E-01	-4.04	12.68	7.50E-01
cg01195564	-7.24	3.30	2.83E-02	-8.11	5.34	1.29E-01	-4.29	7.89	5.86E-01	-6.87	5.45	2.08E-01	-11.46	11.97	3.39E-01
cg09966309	-8.34	3.80	2.83E-02	-8.02	5.22	1.24E-01	-6.20	9.45	5.12E-01	-2.64	10.30	7.98E-01	-15.95	9.21	8.34E-02
cg14610633	37.68	17.18	2.83E-02	49.51	23.66	3.64E-02	37.40	55.00	4.97E-01	18.11	62.84	7.73E-01	21.89	31.34	4.85E-01
cg09434667	-10.42	4.75	2.83E-02	-10.35	7.67	1.77E-01	-10.66	11.01	3.33E-01	-8.64	10.29	4.01E-01	-12.07	10.19	2.36E-01
cg06851827	-57.43	26.18	2.83E-02	-15.60	24.29	5.21E-01	-131.81	48.14	6.18E-03	-42.75	51.08	4.03E-01	-76.04	42.12	7.11E-02
cg23457166	-7.92	3.61	2.83E-02	-11.90	5.90	4.37E-02	-7.83	6.95	2.60E-01	-1.01	8.14	9.01E-01	-7.24	9.05	4.24E-01
cg27141474	6.99	3.19	2.83E-02	10.20	4.24	1.61E-02	2.53	6.81	7.10E-01	4.97	10.62	6.40E-01	1.78	8.96	8.42E-01
cg05603367	14.26	6.50	2.83E-02	8.43	7.17	2.40E-01	32.02	10.70	2.77E-03	-10.10	25.79	6.95E-01	11.81	9.86	2.31E-01
cg02743878	8.96	4.09	2.83E-02	10.77	5.78	6.23E-02	13.27	9.84	1.78E-01	7.79	10.67	4.65E-01	0.81	9.61	9.32E-01
cg13047308	5.94	2.71	2.83E-02	3.24	3.52	3.57E-01	16.36	7.05	2.03E-02	7.27	7.40	3.26E-01	5.04	7.67	5.11E-01
cg03220751	-29.65	13.52	2.83E-02	-49.92	41.06	2.24E-01	88.58	156.20	5.71E-01	-30.82	15.03	4.04E-02	0.20	49.18	9.97E-01
cg19197652	-14.40	6.57	2.83E-02	-35.68	17.43	4.06E-02	8.66	34.33	8.01E-01	-11.01	7.55	1.45E-01	-20.48	25.86	4.28E-01
cg17985972	-7.41	3.38	2.83E-02	-7.66	5.15	1.37E-01	-7.10	8.16	3.84E-01	-11.84	7.75	1.26E-01	-3.10	7.42	6.76E-01
cg22537474	19.20	8.75	2.83E-02	20.42	17.77	2.51E-01	28.10	32.29	3.84E-01	22.33	13.57	9.97E-02	10.76	16.92	5.25E-01
cg12222244	-13.07	5.96	2.83E-02	-10.77	13.65	4.30E-01	3.91	24.77	8.75E-01	-15.28	7.47	4.07E-02	-13.18	17.60	4.54E-01
cg00660167	4.44	2.02	2.83E-02	4.60	2.76	9.53E-02	5.01	3.85	1.92E-01	5.95	8.24	4.70E-01	1.72	5.78	7.66E-01
cg16718260	31.03	14.15	2.83E-02	13.86	24.57	5.73E-01	-36.13	89.28	6.86E-01	35.74	20.16	7.62E-02	64.65	36.47	7.63E-02
cg09033857	-73.57	33.55	2.83E-02	-168.57	82.72	4.16E-02	-462.30	341.07	1.75E-01	-51.71	41.82	2.16E-01	-46.09	73.50	5.31E-01
cg19429405	5.35	2.44	2.83E-02	5.08	3.47	1.43E-01	8.06	6.10	1.86E-01	7.34	5.04	1.45E-01	-1.56	7.33	8.31E-01
cg20764817	18.27	8.33	2.83E-02	50.57	21.45	1.84E-02	-3.78	30.83	9.02E-01	13.33	8.44	1.14E-01	26.55	32.90	4.20E-01
cg12263377	-13.23	6.03	2.83E-02	-24.74	9.28	7.65E-03	-5.50	13.50	6.84E-01	-6.41	17.38	7.12E-01	-3.48	11.91	7.70E-01
cg03879160	-17.37	7.92	2.83E-02	-25.69	12.76	4.40E-02	-0.86	18.64	9.63E-01	-4.95	15.41	7.48E-01	-35.30	19.21	6.61E-02
cg11415512	-43.84	19.99	2.83E-02	-54.29	30.11	7.14E-02	-73.21	123.11	5.52E-01	-57.15	34.68	9.94E-02	5.03	44.63	9.10E-01
cg19853927	16.41	7.49	2.83E-02	20.32	9.61	3.45E-02	8.79	22.32	6.94E-01	-0.56	32.03	9.86E-01	13.83	15.72	3.79E-01
cg05874549	17.23	7.86	2.83E-02	13.66	12.95	2.92E-01	5.65	15.83	7.21E-01	18.30	19.02	3.36E-01	35.83	16.97	3.48E-02
cg02521750	8.08	3.69	2.83E-02	7.15	5.70	2.10E-01	9.25	8.02	2.48E-01	-0.29	10.28	9.78E-01	13.15	7.51	7.99E-02

cg23679992	-11.59	5.28	2.83E-02	-18.01	8.27	2.95E-02	-11.10	11.80	3.47E-01	-7.39	15.45	6.32E-01	-4.18	10.09	6.78E-01
cg07622521	15.69	7.15	2.83E-02	19.30	10.74	7.24E-02	18.15	17.70	3.05E-01	10.08	18.88	5.93E-01	10.87	14.33	4.48E-01
cg23442187	11.32	5.16	2.83E-02	17.64	8.81	4.52E-02	5.07	11.21	6.51E-01	6.63	8.82	4.52E-01	18.84	16.20	2.45E-01
cg14905577	-11.39	5.20	2.83E-02	7.65	14.33	5.94E-01	-11.63	25.31	6.46E-01	-15.75	5.99	8.53E-03	-0.62	19.22	9.74E-01
cg02029478	26.60	12.13	2.83E-02	-28.02	38.32	4.65E-01	15.60	198.33	9.37E-01	33.23	13.28	1.23E-02	26.22	48.72	5.90E-01
cg26782451	17.95	8.19	2.83E-02	24.92	13.39	6.26E-02	16.12	14.40	2.63E-01	10.38	19.11	5.87E-01	12.68	23.74	5.93E-01
cg12099423	3.05	1.39	2.83E-02	3.69	1.86	4.73E-02	3.71	2.97	2.12E-01	-0.78	5.43	8.86E-01	1.44	3.53	6.83E-01
cg07689503	-24.20	11.04	2.83E-02	-9.57	32.73	7.70E-01	-109.14	189.28	5.64E-01	-25.57	12.64	4.30E-02	-26.96	31.91	3.98E-01
cg22988346	-11.76	5.36	2.83E-02	-15.29	8.44	7.01E-02	1.40	14.93	9.25E-01	-16.21	12.44	1.92E-01	-9.79	10.12	3.34E-01
cg17926816	15.60	7.12	2.83E-02	20.19	10.66	5.83E-02	20.45	14.60	1.61E-01	11.55	16.21	4.76E-01	-3.81	20.19	8.50E-01
cg00376816	-6.19	2.82	2.83E-02	-6.97	4.94	1.58E-01	-18.78	13.43	1.62E-01	-2.76	4.04	4.95E-01	-12.22	7.49	1.03E-01
cg12587904	25.01	11.41	2.84E-02	23.64	29.93	4.30E-01	-64.43	111.85	5.65E-01	27.76	13.01	3.28E-02	11.89	41.70	7.76E-01
cg14236443	9.06	4.13	2.84E-02	8.78	5.54	1.13E-01	22.23	10.02	2.66E-02	5.16	11.83	6.62E-01	-1.45	10.55	8.91E-01
cg21809711	40.12	18.30	2.84E-02	14.02	34.07	6.81E-01	-50.40	193.10	7.94E-01	47.76	23.59	4.29E-02	77.10	57.55	1.80E-01
cg07780517	5.19	2.37	2.84E-02	7.43	3.47	3.21E-02	1.27	5.11	8.04E-01	6.95	9.38	4.59E-01	3.96	4.70	3.99E-01
cg09729866	11.67	5.33	2.84E-02	2.89	6.59	6.61E-01	17.95	9.42	5.67E-02	3.79	14.67	7.96E-01	22.96	9.14	1.20E-02
cg00319655	9.42	4.30	2.84E-02	9.66	6.59	1.43E-01	1.84	11.69	8.75E-01	8.89	8.47	2.94E-01	15.22	10.06	1.30E-01
cg05165087	-10.47	4.77	2.84E-02	-9.11	12.43	4.64E-01	5.05	29.50	8.64E-01	-11.35	5.52	3.97E-02	-9.78	17.14	5.68E-01
cg00636946	-6.48	2.96	2.84E-02	-4.94	4.53	2.75E-01	-7.04	5.97	2.38E-01	-8.46	6.81	2.14E-01	-7.56	7.94	3.42E-01
cg16558348	-36.78	16.78	2.84E-02	-49.02	20.78	1.84E-02	-22.25	57.89	7.01E-01	5.08	69.28	9.41E-01	-15.88	37.01	6.68E-01
cg13396830	-85.35	38.94	2.84E-02	-43.83	24.99	7.94E-02	-83.59	115.47	4.69E-01	-183.13	44.09	3.27E-05	-39.77	48.60	4.13E-01
cg19890277	25.89	11.81	2.84E-02	44.33	40.79	2.77E-01	54.82	148.39	7.12E-01	22.62	12.93	8.02E-02	39.21	43.07	3.63E-01
cg19898526	-32.08	14.64	2.84E-02	-29.65	18.35	1.06E-01	35.42	51.16	4.89E-01	-86.34	44.87	5.43E-02	-35.70	25.45	1.61E-01
cg09221478	-12.19	5.56	2.84E-02	-14.95	8.94	9.47E-02	-11.57	12.15	3.41E-01	-8.62	13.77	5.31E-01	-10.72	11.33	3.44E-01
cg11119275	-14.10	6.43	2.84E-02	-8.19	14.45	5.71E-01	2.98	25.38	9.07E-01	-15.68	8.23	5.68E-02	-24.36	18.05	1.77E-01
cg24504349	5.10	2.33	2.84E-02	4.76	3.21	1.39E-01	5.09	6.63	4.43E-01	9.50	4.99	5.72E-02	-0.63	6.35	9.21E-01
cg02898883	-39.28	17.92	2.84E-02	-44.14	23.88	6.46E-02	-62.78	48.90	1.99E-01	-40.95	56.22	4.66E-01	-9.13	39.98	8.19E-01
cg24617504	19.08	8.71	2.84E-02	32.02	10.19	1.69E-03	37.10	18.19	4.14E-02	0.03	12.89	9.98E-01	10.01	13.10	4.45E-01
cg02086310	13.07	5.96	2.84E-02	17.28	8.81	4.97E-02	25.41	20.58	2.17E-01	15.91	21.08	4.51E-01	4.61	9.70	6.35E-01
cg14707392	38.00	17.34	2.84E-02	38.64	19.91	5.23E-02	44.82	117.38	7.03E-01	72.13	92.02	4.33E-01	28.00	40.36	4.88E-01
cg05583014	-21.00	9.58	2.84E-02	-21.94	21.01	2.96E-01	-17.35	42.87	6.86E-01	-15.21	12.12	2.09E-01	-51.75	28.00	6.46E-02
cg26283496	-7.02	3.20	2.84E-02	-7.51	5.11	1.41E-01	-17.11	10.20	9.35E-02	-2.29	5.43	6.73E-01	-9.85	7.99	2.18E-01
cg24592546	-9.25	4.22	2.84E-02	-13.84	9.41	1.42E-01	-18.72	14.31	1.91E-01	-4.07	5.43	4.54E-01	-22.14	12.87	8.55E-02
cg02655711	6.76	3.09	2.84E-02	5.77	4.95	2.44E-01	8.31	4.95	9.32E-02	11.30	13.27	3.94E-01	4.04	7.52	5.92E-01
cg05361811	-45.55	20.79	2.84E-02	-51.04	26.65	5.54E-02	-82.21	55.15	1.36E-01	62.89	80.82	4.36E-01	-38.06	48.53	4.33E-01

cg24432768	-9.64	4.40	2.84E-02	-8.04	6.75	2.34E-01	2.20	9.17	8.10E-01	-10.11	6.82	1.38E-01	-22.66	8.90	1.09E-02
cg15512528	-9.76	4.45	2.84E-02	-12.13	9.00	1.78E-01	-1.45	11.88	9.03E-01	-9.55	6.32	1.31E-01	-15.58	12.95	2.29E-01
cg17123676	11.89	5.43	2.84E-02	17.69	9.13	5.27E-02	0.21	13.05	9.87E-01	15.63	12.60	2.15E-01	9.39	10.10	3.53E-01
cg17967813	-10.64	4.86	2.84E-02	-14.38	9.03	1.11E-01	-1.53	14.04	9.13E-01	-10.28	7.27	1.58E-01	-11.83	12.75	3.53E-01
cg17051318	-26.80	12.23	2.84E-02	-24.70	17.04	1.47E-01	22.27	40.90	5.86E-01	-37.33	28.52	1.90E-01	-43.48	26.58	1.02E-01
cg01374580	-10.04	4.58	2.84E-02	-18.24	8.09	2.41E-02	-12.65	10.18	2.14E-01	1.44	8.02	8.57E-01	-12.55	9.72	1.97E-01
cg19409229	-30.63	13.98	2.84E-02	-38.71	18.08	3.23E-02	-30.25	46.23	5.13E-01	-31.22	37.09	4.00E-01	-1.82	33.97	9.57E-01
cg06510563	19.79	9.03	2.84E-02	8.77	26.17	7.37E-01	-40.80	91.94	6.57E-01	21.27	10.10	3.51E-02	29.79	33.84	3.79E-01
cg17809353	9.15	4.17	2.84E-02	11.50	6.08	5.84E-02	14.01	9.34	1.33E-01	6.74	11.36	5.53E-01	0.05	9.50	9.96E-01
cg02361306	11.09	5.06	2.84E-02	-1.67	8.58	8.45E-01	16.42	12.37	1.84E-01	15.67	7.11	2.75E-02	21.41	14.90	1.51E-01
cg27607338	21.51	9.82	2.84E-02	13.92	25.15	5.80E-01	-5.93	95.05	9.50E-01	20.25	11.25	7.19E-02	53.33	35.66	1.35E-01
cg23729020	28.62	13.06	2.84E-02	19.04	16.19	2.39E-01	-42.76	72.84	5.57E-01	67.18	37.17	7.07E-02	48.10	29.69	1.05E-01
cg18877699	11.80	5.39	2.84E-02	8.70	8.62	3.13E-01	26.27	10.84	1.54E-02	9.00	16.37	5.83E-01	3.72	10.68	7.28E-01
cg05408284	20.55	9.38	2.84E-02	19.47	12.50	1.19E-01	33.38	23.76	1.60E-01	52.50	33.22	1.14E-01	1.03	20.90	9.61E-01
cg25820479	-10.07	4.60	2.84E-02	-5.71	6.44	3.75E-01	-15.40	11.90	1.96E-01	-8.36	16.21	6.06E-01	-16.08	8.99	7.39E-02
cg23898951	-14.01	6.39	2.84E-02	-9.59	15.61	5.39E-01	-5.42	19.85	7.85E-01	-16.80	7.85	3.23E-02	-10.67	25.05	6.70E-01
cg05599825	-37.03	16.90	2.84E-02	-38.99	21.91	7.51E-02	58.82	92.50	5.25E-01	-9.15	46.76	8.45E-01	-60.55	34.41	7.85E-02
cg11793978	-24.15	11.02	2.84E-02	-31.00	16.60	6.18E-02	11.25	30.37	7.11E-01	-39.62	22.17	7.40E-02	-12.08	25.94	6.41E-01
cg23838245	12.69	5.79	2.84E-02	12.32	8.01	1.24E-01	4.64	14.46	7.48E-01	27.28	20.21	1.77E-01	13.90	11.93	2.44E-01
cg02153490	-7.09	3.23	2.84E-02	-3.04	4.63	5.11E-01	-10.46	9.26	2.58E-01	-6.77	9.04	4.54E-01	-13.21	6.32	3.66E-02
cg10444491	-6.87	3.14	2.84E-02	-7.51	4.20	7.34E-02	-5.41	7.45	4.67E-01	-24.58	12.61	5.13E-02	-0.95	6.99	8.92E-01
cg07404961	-15.75	7.19	2.84E-02	-19.01	14.14	1.79E-01	-39.35	16.05	1.42E-02	-11.49	9.73	2.38E-01	0.46	15.12	9.76E-01
cg00460795	20.67	9.43	2.84E-02	16.34	18.27	3.71E-01	24.40	19.52	2.11E-01	8.45	15.04	5.74E-01	61.31	26.85	2.24E-02
cg25126678	18.68	8.53	2.84E-02	46.71	27.10	8.48E-02	12.77	89.37	8.86E-01	14.15	9.24	1.26E-01	46.94	42.40	2.68E-01
cg14474898	-30.54	13.94	2.84E-02	-49.01	34.31	1.53E-01	-129.28	123.27	2.94E-01	-19.06	16.45	2.47E-01	-68.40	43.22	1.13E-01
cg03937687	21.26	9.70	2.84E-02	9.95	15.98	5.33E-01	11.77	22.62	6.03E-01	24.93	18.61	1.80E-01	49.28	23.16	3.34E-02
cg07402396	-17.43	7.96	2.84E-02	-34.38	14.94	2.14E-02	-10.60	18.11	5.59E-01	-7.94	13.24	5.49E-01	-17.07	19.72	3.87E-01
cg07710335	-9.21	4.21	2.85E-02	-18.99	8.33	2.26E-02	-1.78	12.39	8.86E-01	-6.45	6.15	2.95E-01	-7.10	10.43	4.96E-01
cg00227490	33.81	15.43	2.85E-02	45.33	18.24	1.30E-02	34.91	58.59	5.51E-01	-11.36	72.07	8.75E-01	-3.13	37.51	9.34E-01
cg08766812	-11.10	5.07	2.85E-02	-16.57	7.29	2.30E-02	-8.34	10.48	4.26E-01	-16.86	11.08	1.28E-01	8.40	12.90	5.15E-01
cg14966716	21.36	9.75	2.85E-02	1.16	18.91	9.51E-01	20.23	18.90	2.84E-01	38.25	17.27	2.68E-02	23.32	25.24	3.55E-01
cg25888881	6.42	2.93	2.85E-02	5.83	3.81	1.26E-01	15.39	7.85	4.99E-02	8.42	8.55	3.25E-01	-1.14	7.56	8.80E-01
cg07126163	-31.70	14.47	2.85E-02	-20.73	18.15	2.53E-01	-34.51	41.84	4.10E-01	4.29	72.97	9.53E-01	-70.96	31.94	2.63E-02
cg26197220	-35.20	16.07	2.85E-02	-40.39	56.17	4.72E-01	121.35	188.98	5.21E-01	-39.70	17.64	2.44E-02	2.16	56.39	9.69E-01
cg26842089	-28.54	13.03	2.85E-02	-35.00	16.06	2.93E-02	3.32	48.00	9.45E-01	47.38	73.03	5.16E-01	-30.68	26.80	2.52E-01

cg08274234	16.24	7.41	2.85E-02	17.96	10.83	9.74E-02	36.24	25.79	1.60E-01	45.01	30.47	1.40E-01	5.57	11.87	6.39E-01
cg12502785	33.43	15.26	2.85E-02	23.34	28.82	4.18E-01	-36.84	72.56	6.12E-01	37.78	21.10	7.33E-02	57.44	39.10	1.42E-01
cg04006565	16.61	7.58	2.85E-02	24.14	14.63	9.89E-02	-3.06	24.41	9.00E-01	7.60	11.42	5.06E-01	36.47	17.23	3.43E-02
cg13046608	13.67	6.24	2.85E-02	21.29	12.17	8.04E-02	36.78	17.22	3.27E-02	8.55	6.88	2.14E-01	-1.93	17.99	9.14E-01
cg11522145	-17.64	8.05	2.85E-02	-22.28	13.98	1.11E-01	-26.66	16.36	1.03E-01	2.30	16.84	8.91E-01	-21.88	18.13	2.27E-01
cg04362907	30.83	14.08	2.85E-02	-18.66	34.15	5.85E-01	12.95	137.30	9.25E-01	38.76	16.63	1.98E-02	59.07	43.78	1.77E-01
cg24914848	-9.88	4.51	2.85E-02	-10.51	8.52	2.17E-01	3.30	11.99	7.83E-01	-15.70	6.81	2.12E-02	-3.72	12.10	7.58E-01
cg21649258	11.25	5.14	2.85E-02	14.31	12.51	2.53E-01	9.91	21.20	6.40E-01	11.85	6.94	8.76E-02	7.84	10.83	4.69E-01
cg07133445	-29.76	13.58	2.85E-02	-21.51	17.89	2.29E-01	-3.09	37.61	9.34E-01	-88.32	39.90	2.68E-02	-37.93	32.30	2.40E-01
cg14706739	17.96	8.20	2.85E-02	39.16	20.41	5.51E-02	33.36	22.48	1.38E-01	10.54	10.49	3.15E-01	8.04	26.75	7.64E-01
cg22514863	-42.45	19.38	2.85E-02	-39.35	23.31	9.14E-02	-107.26	93.00	2.49E-01	33.85	61.84	5.84E-01	-83.32	47.43	7.90E-02
cg15931168	14.11	6.44	2.85E-02	22.26	10.26	3.01E-02	3.13	20.11	8.77E-01	16.21	15.56	2.98E-01	6.75	11.19	5.47E-01
cg24943066	-9.38	4.28	2.85E-02	-12.41	7.04	7.81E-02	-3.62	10.12	7.20E-01	-6.00	8.31	4.71E-01	-13.75	9.95	1.67E-01
cg03316474	23.68	10.81	2.85E-02	3.01	30.35	9.21E-01	-26.01	108.63	8.11E-01	28.73	12.18	1.83E-02	12.06	39.44	7.60E-01
cg11224946	-11.92	5.44	2.85E-02	-5.60	8.84	5.26E-01	-18.95	10.42	6.91E-02	-11.10	13.48	4.10E-01	-15.28	12.68	2.28E-01
cg03511282	-15.66	7.15	2.85E-02	-6.97	21.24	7.43E-01	2.59	27.71	9.26E-01	-21.60	8.27	9.01E-03	15.10	26.49	5.69E-01
cg01468579	-7.32	3.34	2.85E-02	-9.99	5.58	7.34E-02	-10.97	8.06	1.73E-01	-2.76	6.00	6.45E-01	-6.25	8.40	4.57E-01
cg25995460	13.78	6.29	2.85E-02	12.61	9.59	1.89E-01	37.63	13.10	4.07E-03	4.41	5.67	4.36E-01	13.63	10.54	1.96E-01
cg26406074	13.02	5.95	2.85E-02	13.82	8.34	9.77E-02	-0.19	13.31	9.89E-01	31.59	24.26	1.93E-01	17.80	12.32	1.49E-01
cg27513684	8.32	3.80	2.85E-02	6.40	5.10	2.10E-01	17.41	8.83	4.86E-02	15.43	11.01	1.61E-01	-1.99	10.07	8.44E-01
cg12491659	8.00	3.65	2.85E-02	7.60	4.95	1.25E-01	17.23	9.39	6.66E-02	7.92	11.37	4.86E-01	2.18	8.16	7.89E-01
cg10560079	-5.97	2.73	2.85E-02	-9.84	4.09	1.63E-02	-3.48	11.07	7.53E-01	-3.51	4.42	4.28E-01	-0.56	7.99	9.44E-01
cg05431334	-26.72	12.20	2.86E-02	-21.12	17.28	2.22E-01	2.02	40.91	9.61E-01	-47.14	24.18	5.13E-02	-27.68	30.74	3.68E-01
cg22458831	19.06	8.70	2.86E-02	36.62	18.52	4.80E-02	-5.09	20.44	8.04E-01	21.28	12.66	9.28E-02	14.65	24.65	5.52E-01
cg04486886	17.59	8.03	2.86E-02	23.20	10.99	3.48E-02	23.81	21.89	2.77E-01	24.48	26.13	3.49E-01	-1.39	16.53	9.33E-01
cg06841783	-18.50	8.45	2.86E-02	-24.64	15.23	1.06E-01	-1.20	13.83	9.31E-01	-32.56	10.54	2.01E-03	-0.84	22.53	9.70E-01
cg20456243	-10.68	4.88	2.86E-02	-13.62	7.54	7.08E-02	-2.99	12.58	8.12E-01	-10.66	9.79	2.76E-01	-10.31	11.44	3.67E-01
cg11801727	-4.37	2.00	2.86E-02	-2.28	2.77	4.12E-01	-6.58	3.97	9.74E-02	-6.77	9.38	4.71E-01	-6.62	4.64	1.54E-01
cg02347989	20.52	9.37	2.86E-02	29.61	13.44	2.76E-02	-3.71	16.10	8.18E-01	20.27	19.50	2.99E-01	41.15	21.75	5.86E-02
cg00661523	11.38	5.20	2.86E-02	14.65	6.88	3.33E-02	4.44	13.63	7.45E-01	1.13	19.43	9.53E-01	10.79	11.24	3.37E-01
cg11596397	-31.93	14.58	2.86E-02	-37.18	18.64	4.62E-02	2.43	52.77	9.63E-01	-19.92	64.68	7.58E-01	-32.02	28.54	2.62E-01
cg13272644	16.17	7.39	2.86E-02	30.08	12.94	2.00E-02	2.64	25.30	9.17E-01	11.09	12.11	3.60E-01	9.28	15.87	5.59E-01
cg14478368	-9.37	4.28	2.86E-02	-11.60	8.68	1.82E-01	-16.75	12.46	1.79E-01	-2.05	6.32	7.45E-01	-20.10	10.07	4.59E-02
cg06822193	-10.70	4.89	2.86E-02	-11.70	8.79	1.83E-01	1.95	11.83	8.69E-01	-13.15	8.66	1.29E-01	-15.96	10.87	1.42E-01
cg27312530	-13.66	6.24	2.86E-02	-15.30	20.32	4.51E-01	-1.97	26.77	9.41E-01	-14.67	6.97	3.52E-02	-6.96	27.93	8.03E-01

cg21980394	-8.41	3.84	2.86E-02	-5.99	5.51	2.77E-01	-10.34	9.00	2.50E-01	-5.62	12.08	6.42E-01	-13.23	8.01	9.88E-02
cg00578032	13.88	6.34	2.86E-02	27.79	11.69	1.74E-02	3.06	20.77	8.83E-01	13.87	10.81	1.99E-01	2.41	12.22	8.44E-01
cg14235723	-33.50	15.30	2.86E-02	-18.49	37.86	6.25E-01	-49.28	222.27	8.25E-01	-32.71	17.80	6.61E-02	-65.36	50.19	1.93E-01
cg27615432	32.70	14.94	2.86E-02	32.19	30.78	2.96E-01	41.42	126.02	7.42E-01	26.23	18.19	1.49E-01	90.46	54.33	9.59E-02
cg10100318	17.36	7.93	2.86E-02	17.46	20.02	3.83E-01	6.69	52.63	8.99E-01	16.55	9.17	7.12E-02	28.72	29.33	3.28E-01
cg26669876	-59.31	27.09	2.86E-02	-82.21	33.27	1.35E-02	-37.07	173.11	8.30E-01	7.98	100.48	9.37E-01	-18.68	55.33	7.36E-01
cg13780614	20.60	9.41	2.86E-02	31.46	15.21	3.86E-02	21.81	53.24	6.82E-01	5.74	13.48	6.70E-01	51.56	29.97	8.54E-02
cg08349108	4.57	2.09	2.86E-02	3.50	3.26	2.82E-01	3.82	4.63	4.10E-01	4.63	4.08	2.57E-01	9.25	5.95	1.20E-01
cg18947016	17.15	7.83	2.86E-02	14.71	14.87	3.22E-01	27.66	17.79	1.20E-01	14.18	13.13	2.80E-01	15.37	18.85	4.15E-01
cg25053484	49.06	22.41	2.86E-02	52.13	28.83	7.06E-02	114.70	110.28	2.98E-01	61.24	59.61	3.04E-01	19.54	48.58	6.87E-01
cg10016358	16.04	7.33	2.86E-02	18.44	10.56	8.09E-02	26.42	17.01	1.20E-01	8.16	17.30	6.37E-01	5.19	18.70	7.81E-01
cg07052737	5.80	2.65	2.86E-02	4.21	3.41	2.18E-01	11.08	5.95	6.27E-02	9.02	8.43	2.85E-01	1.77	8.29	8.31E-01
cg10705570	-6.96	3.18	2.86E-02	-7.85	5.27	1.37E-01	6.85	9.53	4.73E-01	-8.59	4.81	7.37E-02	-12.55	10.69	2.40E-01
cg12790424	-11.00	5.02	2.86E-02	-15.55	9.48	1.01E-01	-1.04	11.57	9.28E-01	-11.18	8.67	1.97E-01	-13.74	11.36	2.27E-01
cg16273979	-10.18	4.65	2.86E-02	-16.28	8.69	6.11E-02	-8.44	9.31	3.65E-01	-8.98	9.08	3.23E-01	-5.24	10.35	6.13E-01
cg01402631	-11.13	5.09	2.86E-02	-10.46	9.51	2.72E-01	-1.12	12.68	9.30E-01	-14.25	7.42	5.48E-02	-15.26	17.67	3.88E-01
cg00559054	-42.50	19.42	2.86E-02	-104.83	46.81	2.51E-02	-220.34	196.53	2.62E-01	-29.65	18.67	1.12E-01	-16.99	65.50	7.95E-01
cg10838469	20.52	9.37	2.86E-02	30.18	16.91	7.42E-02	14.57	17.43	4.03E-01	24.79	19.00	1.92E-01	6.20	23.43	7.91E-01
cg12152540	-8.25	3.77	2.86E-02	-2.77	6.90	6.88E-01	-8.51	12.48	4.95E-01	-11.21	5.45	3.97E-02	-9.76	10.39	3.48E-01
cg14456800	13.33	6.09	2.86E-02	6.71	10.68	5.30E-01	11.31	11.93	3.43E-01	9.86	7.59	1.94E-01	43.19	16.73	9.82E-03
cg17423597	7.42	3.39	2.86E-02	11.34	7.44	1.28E-01	-12.29	12.78	3.36E-01	9.11	4.33	3.53E-02	3.17	10.29	7.58E-01
cg04686354	5.66	2.58	2.86E-02	4.84	3.01	1.08E-01	12.79	6.93	6.50E-02	2.74	11.05	8.04E-01	2.32	9.79	8.13E-01
cg19350747	19.79	9.04	2.86E-02	23.69	15.85	1.35E-01	28.52	18.73	1.28E-01	13.90	18.03	4.41E-01	10.21	20.78	6.23E-01
cg22147660	11.61	5.31	2.86E-02	21.43	10.18	3.53E-02	3.70	16.51	8.23E-01	12.08	8.96	1.77E-01	4.26	10.13	6.74E-01
cg20924825	-12.53	5.73	2.87E-02	-14.06	9.45	1.37E-01	-9.59	13.09	4.64E-01	-14.61	14.15	3.02E-01	-11.31	10.88	2.98E-01
cg12786945	23.64	10.80	2.87E-02	66.53	41.50	1.09E-01	-137.74	169.45	4.16E-01	18.46	11.69	1.15E-01	52.75	39.52	1.82E-01
cg11922563	6.19	2.83	2.87E-02	1.51	2.97	6.13E-01	11.42	5.37	3.35E-02	10.87	4.17	9.19E-03	2.89	6.36	6.49E-01
cg14101687	-12.88	5.88	2.87E-02	-4.52	13.71	7.42E-01	-21.46	32.03	5.03E-01	-12.83	6.99	6.67E-02	-30.22	21.62	1.62E-01
cg18607418	-28.72	13.13	2.87E-02	-40.11	15.58	1.01E-02	-44.43	81.59	5.86E-01	0.98	47.80	9.84E-01	4.29	30.17	8.87E-01
cg15854847	16.08	7.35	2.87E-02	24.39	15.16	1.08E-01	3.34	22.25	8.81E-01	16.77	10.81	1.21E-01	11.55	16.69	4.89E-01
cg26169845	23.16	10.59	2.87E-02	20.34	13.88	1.43E-01	44.09	24.11	6.75E-02	16.45	44.27	7.10E-01	11.23	25.77	6.63E-01
cg03438742	32.55	14.88	2.87E-02	31.22	18.75	9.60E-02	5.50	42.76	8.98E-01	71.15	54.78	1.94E-01	39.72	35.47	2.63E-01
cg22053945	8.72	3.99	2.87E-02	11.95	6.84	8.06E-02	19.25	12.95	1.37E-01	9.50	4.95	5.47E-02	-7.62	10.33	4.61E-01
cg20185718	19.54	8.93	2.87E-02	14.55	12.12	2.30E-01	38.45	14.49	7.94E-03	25.91	16.04	1.06E-01	-9.22	19.66	6.39E-01
cg20893031	13.81	6.31	2.87E-02	14.56	8.69	9.39E-02	4.94	19.30	7.98E-01	2.97	41.01	9.42E-01	16.20	10.81	1.34E-01

cg07651048	18.61	8.51	2.87E-02	10.24	14.32	4.75E-01	34.54	17.62	4.99E-02	3.99	17.43	8.19E-01	34.17	20.31	9.25E-02
cg09563325	-10.33	4.72	2.87E-02	-15.90	7.80	4.16E-02	-3.57	11.00	7.46E-01	-2.19	9.11	8.10E-01	-18.01	11.09	1.05E-01
cg16644764	21.42	9.79	2.87E-02	-12.27	32.86	7.09E-01	98.97	116.97	3.97E-01	26.03	10.63	1.43E-02	-4.89	41.48	9.06E-01
cg20449648	15.69	7.17	2.87E-02	0.68	18.66	9.71E-01	55.45	32.08	8.39E-02	17.38	8.54	4.17E-02	5.68	23.14	8.06E-01
cg18406000	-4.98	2.28	2.87E-02	-5.40	3.62	1.35E-01	0.50	5.68	9.30E-01	-3.35	4.48	4.55E-01	-11.16	5.30	3.55E-02
cg01027532	-20.57	9.40	2.87E-02	-43.06	16.64	9.65E-03	-12.28	19.70	5.33E-01	-2.15	17.15	9.00E-01	-21.96	20.57	2.86E-01
cg10851715	-14.99	6.85	2.87E-02	-22.30	12.80	8.16E-02	-5.75	17.84	7.47E-01	-13.94	10.08	1.67E-01	-12.65	21.22	5.51E-01
cg21450784	-7.05	3.22	2.87E-02	-7.82	4.36	7.27E-02	-0.24	7.15	9.74E-01	-13.07	9.73	1.79E-01	-9.22	8.61	2.84E-01
cg04298953	13.15	6.01	2.87E-02	19.42	9.77	4.69E-02	26.50	17.12	1.22E-01	10.43	12.78	4.14E-01	0.80	11.43	9.44E-01
cg03716590	-11.83	5.41	2.87E-02	-3.96	11.15	7.23E-01	-11.08	15.56	4.76E-01	-12.91	7.53	8.63E-02	-22.73	15.16	1.34E-01
cg18614510	8.31	3.80	2.87E-02	9.84	5.14	5.53E-02	12.13	10.11	2.30E-01	7.78	9.34	4.05E-01	-0.48	9.91	9.62E-01
cg10567706	-7.57	3.46	2.87E-02	-9.59	4.97	5.37E-02	-6.43	8.71	4.60E-01	-20.16	14.53	1.65E-01	-2.54	6.31	6.88E-01
cg00926657	9.98	4.56	2.87E-02	9.24	6.08	1.29E-01	17.48	8.14	3.17E-02	18.78	13.31	1.58E-01	-4.86	10.22	6.35E-01
cg11259591	15.34	7.01	2.87E-02	17.51	14.46	2.26E-01	6.02	21.62	7.81E-01	18.35	9.61	5.63E-02	6.48	19.61	7.41E-01
cg16018972	53.61	24.51	2.87E-02	33.03	34.04	3.32E-01	88.49	112.03	4.30E-01	70.68	62.68	2.59E-01	76.36	46.25	9.87E-02
cg00335124	6.53	2.99	2.87E-02	4.97	3.92	2.05E-01	7.35	7.02	2.95E-01	15.66	10.95	1.53E-01	7.06	7.41	3.41E-01
cg16619935	18.78	8.59	2.87E-02	33.61	18.92	7.56E-02	25.22	20.84	2.26E-01	6.66	9.57	4.86E-01	46.01	25.23	6.82E-02
cg01400750	5.06	2.31	2.87E-02	6.27	3.14	4.59E-02	11.39	6.41	7.57E-02	2.30	5.29	6.64E-01	-1.92	6.26	7.59E-01
cg08906442	26.73	12.22	2.87E-02	35.50	17.49	4.24E-02	-5.98	27.13	8.25E-01	41.65	39.52	2.92E-01	31.08	26.47	2.40E-01
cg01109256	-8.90	4.07	2.87E-02	-10.11	5.74	7.84E-02	-8.71	9.55	3.62E-01	-16.14	18.13	3.73E-01	-5.39	7.91	4.96E-01
cg25805739	-28.23	12.91	2.87E-02	-74.34	46.76	1.12E-01	157.12	164.48	3.39E-01	-26.08	14.29	6.81E-02	-22.21	40.48	5.83E-01
cg26104143	13.19	6.03	2.87E-02	10.17	19.62	6.04E-01	-45.20	99.92	6.51E-01	14.74	6.51	2.36E-02	-5.58	28.67	8.46E-01
cg07415388	19.61	8.97	2.87E-02	17.72	15.50	2.53E-01	22.59	18.06	2.11E-01	17.00	16.64	3.07E-01	24.70	25.00	3.23E-01
cg19889626	11.69	5.35	2.87E-02	4.60	12.08	7.03E-01	10.24	16.54	5.36E-01	12.89	7.87	1.01E-01	15.82	10.94	1.48E-01
cg25303462	15.50	7.09	2.87E-02	26.70	9.45	4.73E-03	27.56	19.75	1.63E-01	1.31	8.53	8.78E-01	16.34	13.15	2.14E-01
cg00980649	7.66	3.50	2.87E-02	10.26	4.77	3.15E-02	11.96	7.69	1.20E-01	-4.68	11.86	6.93E-01	0.31	8.61	9.71E-01
cg12573871	-7.51	3.44	2.87E-02	-2.16	8.09	7.90E-01	-10.50	14.55	4.71E-01	-9.15	4.29	3.31E-02	-5.50	9.79	5.74E-01
cg14929757	-60.89	27.84	2.87E-02	-79.14	44.13	7.30E-02	-172.52	181.38	3.42E-01	-24.29	13.13	6.44E-02	-119.15	48.46	1.39E-02
cg09938408	-42.05	19.23	2.87E-02	-10.33	32.46	7.50E-01	-83.54	51.13	1.02E-01	-39.83	33.63	2.36E-01	-75.21	45.24	9.65E-02
cg20463894	14.12	6.45	2.88E-02	12.80	9.53	1.79E-01	25.86	14.72	7.90E-02	2.90	16.46	8.60E-01	14.48	14.60	3.22E-01
cg03182218	-45.97	21.02	2.88E-02	-66.58	33.51	4.70E-02	6.95	53.11	8.96E-01	-63.46	50.05	2.05E-01	-35.36	40.20	3.79E-01
cg09029538	13.02	5.95	2.88E-02	21.33	11.08	5.42E-02	17.22	13.60	2.06E-01	0.67	9.20	9.42E-01	25.59	15.63	1.02E-01
cg24339597	-22.03	10.07	2.88E-02	-45.68	17.81	1.03E-02	-44.54	28.41	1.17E-01	-9.81	7.34	1.81E-01	-13.62	22.39	5.43E-01
cg11351475	33.71	15.41	2.88E-02	75.08	38.29	4.99E-02	-80.73	119.30	4.99E-01	27.41	18.13	1.31E-01	31.18	49.10	5.25E-01
cg24996270	28.24	12.92	2.88E-02	45.05	27.78	1.05E-01	220.08	117.07	6.01E-02	24.51	10.44	1.90E-02	7.52	32.48	8.17E-01

cg24704476	12.09	5.53	2.88E-02	15.43	8.40	6.64E-02	6.06	14.21	6.70E-01	17.60	14.25	2.17E-01	6.95	10.74	5.17E-01
cg12595026	33.51	15.32	2.88E-02	32.69	20.99	1.19E-01	50.53	40.52	2.12E-01	48.76	43.69	2.64E-01	14.23	34.19	6.77E-01
cg09636163	-10.86	4.96	2.88E-02	-15.88	7.43	3.24E-02	-7.87	11.32	4.87E-01	10.93	14.32	4.45E-01	-14.80	10.12	1.44E-01
cg12763978	-4.16	1.90	2.88E-02	-5.24	3.18	9.95E-02	-3.31	4.75	4.86E-01	-5.33	3.36	1.13E-01	-0.29	4.75	9.52E-01
cg10740660	10.83	4.95	2.88E-02	10.78	6.52	9.80E-02	9.02	12.86	4.83E-01	17.59	15.84	2.67E-01	8.74	11.78	4.58E-01
cg04611159	22.91	10.48	2.88E-02	10.75	17.28	5.34E-01	5.97	15.65	7.03E-01	26.04	14.60	7.45E-02	58.70	20.85	4.88E-03
cg12811871	-5.97	2.73	2.88E-02	-5.96	3.41	8.04E-02	-11.55	7.55	1.26E-01	3.27	10.26	7.50E-01	-5.51	6.88	4.23E-01
cg05166871	8.40	3.84	2.88E-02	10.85	5.30	4.08E-02	11.98	9.29	1.97E-01	6.78	9.82	4.90E-01	-2.52	9.88	7.99E-01
cg16983627	21.23	9.71	2.88E-02	27.99	19.23	1.46E-01	16.01	43.33	7.12E-01	14.54	12.76	2.54E-01	42.13	28.56	1.40E-01
cg07109551	-11.70	5.35	2.88E-02	-17.25	7.95	3.00E-02	-11.26	11.64	3.33E-01	2.82	16.11	8.61E-01	-8.07	11.29	4.75E-01
cg16353615	6.71	3.07	2.88E-02	6.96	4.91	1.56E-01	7.76	6.90	2.61E-01	5.85	5.66	3.02E-01	6.30	9.00	4.84E-01
cg12393318	17.07	7.81	2.88E-02	29.54	17.02	8.27E-02	21.68	20.17	2.82E-01	22.39	13.16	8.89E-02	-0.94	14.55	9.48E-01
cg02989080	-11.86	5.43	2.88E-02	-26.14	11.09	1.84E-02	-10.98	16.21	4.98E-01	-8.61	7.47	2.49E-01	1.36	15.59	9.30E-01
cg09904358	-8.59	3.93	2.88E-02	-3.98	10.33	7.00E-01	1.22	16.64	9.42E-01	-9.92	4.67	3.35E-02	-11.55	13.04	3.76E-01
cg20691428	-45.94	21.01	2.88E-02	-67.62	29.36	2.13E-02	-27.99	64.00	6.62E-01	-30.13	44.17	4.95E-01	-9.51	53.63	8.59E-01
cg16606638	12.63	5.78	2.88E-02	16.06	9.11	7.78E-02	20.64	14.81	1.63E-01	15.84	14.40	2.71E-01	1.66	10.83	8.78E-01
cg20759486	-13.73	6.28	2.88E-02	-6.03	9.80	5.38E-01	-28.29	13.06	3.04E-02	-13.93	20.48	4.97E-01	-12.88	12.21	2.91E-01
cg21607172	10.74	4.91	2.88E-02	11.29	6.39	7.75E-02	22.71	9.78	2.02E-02	-5.81	12.61	6.45E-01	6.85	11.83	5.63E-01
cg21425003	-48.48	22.17	2.88E-02	-58.80	29.94	4.95E-02	-20.07	91.36	8.26E-01	-80.33	66.32	2.26E-01	-21.57	41.85	6.06E-01
cg02664157	27.58	12.61	2.88E-02	64.33	35.40	6.92E-02	-63.56	149.41	6.71E-01	22.80	14.04	1.04E-01	24.79	52.18	6.35E-01
cg13826890	18.62	8.52	2.88E-02	30.68	15.98	5.49E-02	32.23	45.35	4.77E-01	22.26	8.48	8.70E-03	-14.17	19.73	4.73E-01
cg03532013	-34.14	15.62	2.88E-02	-41.96	29.75	1.58E-01	82.82	115.18	4.72E-01	-34.16	21.58	1.14E-01	-34.08	36.59	3.52E-01
cg00491404	-12.81	5.86	2.88E-02	-13.12	8.41	1.19E-01	-11.90	13.43	3.76E-01	-1.63	18.02	9.28E-01	-18.31	12.53	1.44E-01
cg08259506	21.84	9.99	2.88E-02	7.67	11.52	5.05E-01	57.07	30.05	5.75E-02	24.60	32.81	4.53E-01	34.86	17.47	4.59E-02
cg20061010	-21.08	9.64	2.88E-02	-29.03	14.61	4.69E-02	-37.21	12.07	2.05E-03	0.31	11.57	9.79E-01	-20.21	21.71	3.52E-01
cg19329644	18.82	8.61	2.88E-02	19.91	16.72	2.34E-01	25.72	15.39	9.45E-02	1.06	14.67	9.42E-01	48.88	25.08	5.13E-02
cg18601426	10.30	4.71	2.88E-02	11.35	6.84	9.69E-02	11.96	14.87	4.21E-01	-2.47	14.67	8.66E-01	12.33	8.32	1.38E-01
cg14098681	19.70	9.01	2.88E-02	-13.29	39.66	7.38E-01	57.64	67.73	3.95E-01	18.52	9.81	5.90E-02	43.21	30.69	1.59E-01
cg08891110	11.38	5.21	2.88E-02	12.16	6.89	7.75E-02	12.57	13.73	3.60E-01	17.47	19.32	3.66E-01	6.39	11.29	5.71E-01
cg27287245	-60.46	27.66	2.88E-02	-75.35	67.56	2.65E-01	-128.13	256.29	6.17E-01	-73.06	32.96	2.67E-02	43.91	81.04	5.88E-01
cg03607054	-10.45	4.78	2.88E-02	-14.59	7.82	6.21E-02	-2.71	11.21	8.09E-01	-13.28	8.38	1.13E-01	-1.56	13.82	9.10E-01
cg14652717	-23.27	10.65	2.88E-02	-28.58	16.60	8.50E-02	-29.49	32.40	3.63E-01	-39.61	22.47	7.80E-02	2.21	21.03	9.16E-01
cg23210148	-62.35	28.53	2.89E-02	-38.41	38.58	3.19E-01	22.33	148.69	8.81E-01	-86.34	83.40	3.01E-01	-107.16	52.16	3.99E-02
cg09390350	-7.63	3.49	2.89E-02	-12.58	4.75	8.16E-03	-1.89	8.75	8.29E-01	-4.51	13.13	7.31E-01	-1.01	7.24	8.89E-01
cg12422551	11.07	5.07	2.89E-02	-1.28	10.83	9.06E-01	-3.90	21.88	8.58E-01	17.33	6.11	4.57E-03	2.45	18.46	8.94E-01

cg17959327	26.16	11.97	2.89E-02	47.96	41.34	2.46E-01	-37.88	109.84	7.30E-01	33.43	12.68	8.37E-03	-19.08	30.95	5.38E-01
cg07268332	6.42	2.94	2.89E-02	4.14	3.66	2.59E-01	17.83	7.62	1.93E-02	8.33	6.34	1.89E-01	0.78	7.58	9.18E-01
cg09444744	-17.08	7.81	2.89E-02	-9.12	16.38	5.78E-01	-14.49	32.82	6.59E-01	-24.10	10.14	1.74E-02	1.16	22.40	9.59E-01
cg08773955	-8.81	4.03	2.89E-02	-15.18	7.84	5.29E-02	-16.60	11.45	1.47E-01	-5.09	5.88	3.87E-01	-2.45	10.75	8.20E-01
cg25075504	-9.90	4.53	2.89E-02	-9.83	9.66	3.09E-01	-20.33	13.19	1.23E-01	-8.67	6.27	1.67E-01	-5.83	12.09	6.30E-01
cg01763890	13.57	6.21	2.89E-02	15.96	10.45	1.27E-01	12.18	17.08	4.76E-01	5.38	11.94	6.53E-01	19.91	12.55	1.13E-01
cg04794887	-18.33	8.39	2.89E-02	-13.49	12.21	2.69E-01	-29.97	17.00	7.78E-02	-29.67	31.08	3.40E-01	-11.81	18.24	5.17E-01
cg21515003	-13.70	6.27	2.89E-02	-23.85	15.10	1.14E-01	0.52	19.00	9.78E-01	-12.05	7.81	1.23E-01	-25.34	23.02	2.71E-01
cg08833577	11.33	5.19	2.89E-02	21.65	10.15	3.29E-02	1.85	12.37	8.81E-01	14.10	9.00	1.17E-01	2.90	10.79	7.88E-01
cg26094599	10.55	4.83	2.89E-02	9.90	7.19	1.69E-01	19.90	12.02	9.79E-02	5.70	10.28	5.79E-01	9.66	11.79	4.13E-01
cg11123440	4.72	2.16	2.89E-02	1.03	2.36	6.61E-01	6.65	3.04	2.87E-02	10.59	3.57	3.03E-03	2.14	3.42	5.31E-01
cg26492847	-14.05	6.43	2.89E-02	-24.90	14.78	9.21E-02	-5.53	25.64	8.29E-01	-13.72	7.89	8.22E-02	1.34	22.14	9.52E-01
cg19254119	10.83	4.96	2.89E-02	22.93	10.54	2.95E-02	11.27	15.72	4.73E-01	6.19	7.17	3.89E-01	8.33	11.05	4.51E-01
cg02407762	17.03	7.79	2.89E-02	14.59	14.25	3.06E-01	3.48	18.36	8.50E-01	26.82	12.42	3.08E-02	11.64	21.89	5.95E-01
cg00431050	11.70	5.36	2.89E-02	8.97	7.33	2.21E-01	20.41	11.06	6.48E-02	4.93	18.39	7.89E-01	11.62	13.97	4.05E-01
cg20445034	-49.19	22.51	2.89E-02	-30.82	30.21	3.08E-01	94.38	130.94	4.71E-01	-84.32	47.23	7.42E-02	-83.55	51.94	1.08E-01
cg10203522	41.24	18.88	2.89E-02	62.22	52.71	2.38E-01	-51.23	207.15	8.05E-01	45.91	21.12	2.98E-02	-45.75	74.06	5.37E-01
cg23047134	13.93	6.38	2.89E-02	19.67	9.49	3.82E-02	0.13	12.50	9.92E-01	23.01	18.28	2.08E-01	13.30	15.62	3.94E-01
cg07121547	20.99	9.61	2.89E-02	26.05	13.07	4.62E-02	24.95	26.25	3.42E-01	50.96	29.51	8.42E-02	-5.40	19.47	7.81E-01
cg05295919	43.28	19.81	2.89E-02	49.15	25.15	5.07E-02	-66.05	86.84	4.47E-01	53.06	75.38	4.81E-01	48.62	38.99	2.12E-01
cg07252778	16.08	7.36	2.89E-02	-9.01	22.48	6.88E-01	9.00	38.28	8.14E-01	19.46	8.24	1.82E-02	20.50	30.56	5.02E-01
cg12076953	31.77	14.54	2.89E-02	12.04	19.68	5.41E-01	57.68	44.90	1.99E-01	77.47	47.02	9.94E-02	46.28	28.89	1.09E-01
cg00558215	8.25	3.78	2.89E-02	5.16	6.92	4.56E-01	23.40	11.64	4.43E-02	6.38	5.89	2.78E-01	8.72	8.74	3.18E-01
cg07625774	-14.53	6.65	2.89E-02	-24.07	10.90	2.72E-02	4.73	19.03	8.04E-01	-8.50	14.20	5.49E-01	-14.95	12.43	2.29E-01
cg15514496	18.02	8.25	2.89E-02	30.29	14.72	3.97E-02	-2.31	22.21	9.17E-01	22.97	16.57	1.66E-01	10.44	15.06	4.88E-01
cg05584078	-11.55	5.29	2.89E-02	-16.37	13.22	2.16E-01	-14.01	31.02	6.51E-01	-14.51	6.40	2.34E-02	10.91	14.82	4.62E-01
cg08832603	11.81	5.41	2.89E-02	16.30	9.11	7.34E-02	17.66	28.63	5.37E-01	2.66	8.04	7.40E-01	26.46	13.53	5.04E-02
cg24851600	-14.27	6.53	2.90E-02	-17.99	15.94	2.59E-01	-19.73	26.23	4.52E-01	-11.83	7.85	1.32E-01	-23.58	23.48	3.15E-01
cg26270975	-8.77	4.02	2.90E-02	-11.66	7.12	1.01E-01	-4.21	7.94	5.96E-01	-11.44	7.50	1.27E-01	-5.04	10.79	6.40E-01
cg10547893	-25.77	11.80	2.90E-02	-24.34	15.85	1.25E-01	27.64	45.01	5.39E-01	-52.20	30.34	8.54E-02	-27.83	24.81	2.62E-01
cg08553524	-14.53	6.65	2.90E-02	-31.36	12.29	1.07E-02	4.85	19.70	8.05E-01	-13.87	8.18	9.01E-02	-2.19	16.41	8.94E-01
cg01408932	-22.21	10.17	2.90E-02	-34.50	13.06	8.25E-03	-9.52	38.54	8.05E-01	-12.86	23.90	5.91E-01	11.95	26.93	6.57E-01
cg24938830	10.09	4.62	2.90E-02	21.61	11.70	6.47E-02	5.73	13.13	6.62E-01	12.63	7.07	7.43E-02	2.14	8.51	8.02E-01
cg14913644	15.75	7.21	2.90E-02	15.97	21.88	4.65E-01	-56.13	77.34	4.68E-01	15.59	8.09	5.39E-02	23.96	24.29	3.24E-01
cg06786630	8.15	3.73	2.90E-02	8.47	8.28	3.06E-01	14.73	12.76	2.48E-01	8.16	4.79	8.84E-02	2.08	11.54	8.57E-01

cg08363114	6.36	2.91	2.90E-02	5.85	3.82	1.26E-01	10.49	6.25	9.31E-02	-2.12	10.40	8.39E-01	6.84	8.24	4.06E-01
cg03012370	8.69	3.98	2.90E-02	8.01	7.01	2.53E-01	21.58	9.54	2.37E-02	1.12	7.18	8.76E-01	10.18	8.95	2.55E-01
cg08443038	-17.85	8.17	2.90E-02	-20.06	13.95	1.50E-01	-30.14	25.29	2.33E-01	-9.74	13.64	4.75E-01	-22.39	18.61	2.29E-01
cg02525637	-15.84	7.25	2.90E-02	-26.66	11.89	2.49E-02	-9.47	13.67	4.89E-01	3.04	16.60	8.55E-01	-24.57	18.36	1.81E-01
cg09996288	39.99	18.31	2.90E-02	87.09	53.57	1.04E-01	-42.07	206.19	8.38E-01	36.93	20.74	7.49E-02	14.14	59.20	8.11E-01
cg22152728	17.38	7.96	2.90E-02	38.81	16.24	1.69E-02	24.48	22.39	2.74E-01	4.21	12.37	7.34E-01	14.71	16.99	3.87E-01
cg00705661	21.68	9.93	2.90E-02	15.58	15.42	3.12E-01	3.68	52.61	9.44E-01	28.32	27.46	3.02E-01	27.16	15.33	7.65E-02
cg03421926	17.35	7.95	2.90E-02	20.70	13.53	1.26E-01	4.83	16.30	7.67E-01	16.84	16.28	3.01E-01	28.21	18.78	1.33E-01
cg19266910	21.60	9.89	2.90E-02	11.62	25.43	6.48E-01	10.60	42.26	8.02E-01	14.21	13.02	2.75E-01	51.03	21.25	1.63E-02
cg23343073	13.72	6.28	2.90E-02	21.39	9.39	2.27E-02	13.42	13.73	3.28E-01	-1.97	14.06	8.89E-01	12.03	16.59	4.68E-01
cg27657473	24.90	11.40	2.90E-02	43.48	23.55	6.49E-02	-112.08	96.20	2.44E-01	22.31	14.06	1.12E-01	17.11	37.35	6.47E-01
cg23306295	42.87	19.63	2.90E-02	85.77	42.26	4.24E-02	-27.25	134.32	8.39E-01	21.48	12.42	8.38E-02	80.29	38.85	3.88E-02
cg21307484	-9.24	4.23	2.90E-02	-7.16	5.87	2.23E-01	-13.55	10.63	2.03E-01	-6.84	11.03	5.35E-01	-13.55	10.13	1.81E-01
cg20279561	23.21	10.63	2.90E-02	11.06	14.38	4.42E-01	61.06	23.43	9.17E-03	20.51	29.23	4.83E-01	17.41	19.28	3.67E-01
cg03150463	-35.68	16.34	2.90E-02	-80.49	40.54	4.71E-02	177.94	169.04	2.92E-01	-27.43	16.16	8.96E-02	-51.69	55.99	3.56E-01
cg09989886	-49.31	22.58	2.90E-02	-49.92	27.10	6.55E-02	35.96	115.95	7.56E-01	50.47	92.73	5.86E-01	-91.22	49.49	6.53E-02
cg19089314	23.85	10.92	2.90E-02	16.65	28.89	5.64E-01	-50.34	105.34	6.33E-01	21.97	12.44	7.75E-02	67.10	39.70	9.10E-02
cg19825483	-9.34	4.28	2.90E-02	-7.70	7.56	3.08E-01	6.67	16.21	6.81E-01	-13.86	6.22	2.58E-02	-5.68	11.62	6.25E-01
cg03804136	16.65	7.63	2.90E-02	-5.44	25.07	8.28E-01	-46.43	92.61	6.16E-01	17.86	8.55	3.66E-02	31.15	23.64	1.88E-01
cg24795297	22.03	10.09	2.90E-02	9.91	19.33	6.08E-01	23.67	37.69	5.30E-01	23.70	13.87	8.75E-02	40.33	28.42	1.56E-01
cg10574783	-18.65	8.54	2.90E-02	-18.93	16.81	2.60E-01	-5.65	26.93	8.34E-01	-21.49	11.71	6.66E-02	-16.11	25.82	5.33E-01
cg12882392	19.84	9.09	2.90E-02	19.97	16.40	2.23E-01	25.99	16.98	1.26E-01	12.00	18.26	5.11E-01	20.72	22.82	3.64E-01
cg03120829	-39.24	17.98	2.90E-02	-42.42	23.24	6.80E-02	18.60	74.42	8.03E-01	-77.08	85.31	3.66E-01	-38.56	32.88	2.41E-01
cg22412680	-13.12	6.01	2.90E-02	-20.23	9.34	3.02E-02	-3.00	13.61	8.25E-01	-2.02	16.19	9.01E-01	-15.30	11.94	2.00E-01
cg20122727	14.19	6.50	2.90E-02	23.02	9.65	1.70E-02	-11.15	22.02	6.13E-01	1.79	16.17	9.12E-01	14.89	11.93	2.12E-01
cg10536898	9.16	4.20	2.90E-02	11.34	5.94	5.60E-02	10.66	9.61	2.67E-01	5.70	12.99	6.61E-01	4.20	9.28	6.50E-01
cg16658579	14.90	6.82	2.90E-02	15.52	13.16	2.38E-01	8.36	11.20	4.56E-01	20.05	13.26	1.30E-01	24.24	22.08	2.72E-01
cg06372475	10.98	5.03	2.91E-02	12.21	6.43	5.77E-02	16.32	13.85	2.39E-01	7.04	14.38	6.24E-01	3.71	13.75	7.87E-01
cg03187073	5.60	2.57	2.91E-02	3.62	3.52	3.04E-01	11.53	6.45	7.37E-02	10.33	6.26	9.88E-02	0.82	6.77	9.03E-01
cg06860998	7.88	3.61	2.91E-02	7.55	5.18	1.45E-01	6.85	9.10	4.51E-01	-3.46	12.98	7.90E-01	12.17	6.82	7.45E-02
cg23129573	7.88	3.61	2.91E-02	7.20	7.08	3.09E-01	3.98	7.75	6.07E-01	10.26	6.63	1.22E-01	9.26	7.58	2.21E-01
cg11848928	19.30	8.84	2.91E-02	26.54	12.76	3.75E-02	25.68	20.00	1.99E-01	2.70	27.18	9.21E-01	5.72	18.92	7.63E-01
cg21747617	18.71	8.57	2.91E-02	-4.57	33.05	8.90E-01	6.83	114.03	9.52E-01	21.28	9.13	1.97E-02	4.71	40.29	9.07E-01
cg13427055	-41.61	19.07	2.91E-02	-53.02	24.41	2.98E-02	-76.01	65.79	2.48E-01	33.73	54.53	5.36E-01	-38.11	44.51	3.92E-01
cg21696012	6.88	3.15	2.91E-02	5.55	2.95	5.95E-02	13.79	6.13	2.45E-02	13.89	8.05	8.42E-02	-2.30	6.58	7.26E-01

cg16015276	18.50	8.47	2.91E-02	28.86	24.88	2.46E-01	-17.70	61.64	7.74E-01	15.74	10.44	1.31E-01	24.79	18.68	1.84E-01
cg18267471	-43.28	19.83	2.91E-02	-48.17	24.79	5.20E-02	-87.99	92.83	3.43E-01	-20.69	77.65	7.90E-01	-28.44	39.72	4.74E-01
cg02433979	-13.22	6.06	2.91E-02	-11.15	11.57	3.35E-01	-7.39	15.59	6.35E-01	-13.15	9.87	1.83E-01	-20.72	13.64	1.29E-01
cg02780849	18.47	8.46	2.91E-02	22.36	9.20	1.50E-02	-37.73	34.92	2.80E-01	8.55	12.36	4.89E-01	32.88	11.48	4.18E-03
cg24943198	-12.81	5.87	2.91E-02	-19.72	12.47	1.14E-01	-7.44	16.19	6.46E-01	-14.24	8.40	9.02E-02	-3.25	14.73	8.26E-01
cg05686990	-14.43	6.61	2.91E-02	-17.07	15.08	2.58E-01	-31.35	36.46	3.90E-01	-14.42	8.13	7.63E-02	-5.19	19.54	7.91E-01
cg19262958	18.17	8.32	2.91E-02	5.38	16.72	7.48E-01	14.01	15.56	3.68E-01	18.19	14.09	1.97E-01	55.25	24.30	2.30E-02
cg19773296	14.26	6.53	2.91E-02	7.72	12.76	5.45E-01	21.83	22.92	3.41E-01	14.23	8.95	1.12E-01	23.30	18.61	2.10E-01
cg18334345	-11.35	5.20	2.91E-02	-22.04	8.98	1.41E-02	-9.58	13.82	4.88E-01	-2.77	5.96	6.42E-01	-18.79	11.36	9.82E-02
cg05521792	16.11	7.38	2.91E-02	39.38	25.01	1.15E-01	34.73	29.30	2.36E-01	13.15	8.86	1.38E-01	8.65	18.75	6.44E-01
cg04042828	-14.25	6.53	2.91E-02	-16.03	9.56	9.38E-02	-8.13	15.70	6.05E-01	-6.10	27.77	8.26E-01	-16.48	11.82	1.63E-01
cg05111110	18.39	8.43	2.91E-02	18.56	17.09	2.78E-01	21.80	16.54	1.87E-01	14.11	13.62	3.00E-01	24.58	24.90	3.24E-01
cg06105555	-8.74	4.00	2.91E-02	-14.74	8.47	8.19E-02	1.65	17.30	9.24E-01	-5.43	5.18	2.94E-01	-18.34	11.36	1.06E-01
cg15999188	22.12	10.14	2.91E-02	15.41	14.90	3.01E-01	4.37	30.84	8.87E-01	20.36	25.25	4.20E-01	41.95	19.59	3.23E-02
cg21187920	-7.60	3.48	2.91E-02	-6.42	6.82	3.46E-01	1.63	12.68	8.98E-01	-9.37	4.89	5.54E-02	-8.25	8.79	3.47E-01
cg24505713	8.04	3.68	2.91E-02	8.19	4.76	8.53E-02	15.08	10.18	1.39E-01	12.81	10.52	2.23E-01	-2.76	9.57	7.73E-01
cg12746908	14.19	6.50	2.91E-02	15.08	9.25	1.03E-01	-8.51	22.89	7.10E-01	6.54	24.32	7.88E-01	19.66	10.93	7.21E-02
cg14908307	-11.76	5.39	2.91E-02	-16.70	8.66	5.39E-02	-12.00	13.19	3.63E-01	-14.92	11.63	2.00E-01	-0.41	11.19	9.71E-01
cg03716125	-6.02	2.76	2.91E-02	-1.55	4.48	7.29E-01	-7.61	7.97	3.40E-01	-10.28	4.53	2.32E-02	-5.45	7.68	4.78E-01
cg23711059	-13.30	6.09	2.91E-02	-15.27	9.75	1.17E-01	-0.08	12.71	9.95E-01	-31.94	16.69	5.56E-02	-12.42	12.29	3.12E-01
cg07281879	18.88	8.66	2.91E-02	17.54	19.40	3.66E-01	48.55	41.00	2.36E-01	13.35	11.30	2.37E-01	31.79	21.02	1.30E-01
cg02632542	24.12	11.06	2.91E-02	33.75	17.29	5.10E-02	31.86	22.46	1.56E-01	-15.70	31.26	6.16E-01	20.40	23.36	3.82E-01
cg26196912	-44.03	20.18	2.91E-02	-23.46	48.68	6.30E-01	-9.02	230.44	9.69E-01	-55.53	23.66	1.89E-02	5.10	66.25	9.39E-01
cg04360334	-14.02	6.43	2.91E-02	-14.78	14.77	3.17E-01	30.24	32.10	3.46E-01	-14.35	8.05	7.46E-02	-24.67	17.61	1.61E-01
cg27020941	47.91	21.96	2.91E-02	64.45	56.52	2.54E-01	-179.97	240.76	4.55E-01	42.23	24.97	9.07E-02	104.56	84.89	2.18E-01
cg05245379	-9.34	4.28	2.91E-02	-18.40	10.66	8.45E-02	5.67	17.44	7.45E-01	-7.12	5.10	1.63E-01	-22.67	15.61	1.46E-01
cg10514793	20.50	9.40	2.91E-02	8.19	18.01	6.49E-01	29.35	18.56	1.14E-01	11.16	17.49	5.23E-01	41.17	21.98	6.11E-02
cg18669406	26.27	12.04	2.92E-02	17.04	16.54	3.03E-01	29.56	30.92	3.39E-01	100.70	45.01	2.53E-02	22.05	22.03	3.17E-01
cg20300135	11.83	5.42	2.92E-02	18.57	15.20	2.22E-01	-27.11	31.28	3.86E-01	11.10	6.08	6.82E-02	30.15	24.55	2.19E-01
cg24843413	35.47	16.26	2.92E-02	49.58	22.86	3.01E-02	24.44	101.53	8.10E-01	36.06	38.54	3.50E-01	11.49	30.18	7.03E-01
cg13433612	15.05	6.90	2.92E-02	23.51	15.15	1.21E-01	0.21	21.22	9.92E-01	14.17	9.19	1.23E-01	17.52	19.62	3.72E-01
cg06555246	17.71	8.12	2.92E-02	13.15	13.04	3.13E-01	18.49	14.78	2.11E-01	20.26	21.78	3.52E-01	24.57	19.59	2.10E-01
cg04050463	8.63	3.96	2.92E-02	10.25	5.11	4.48E-02	14.79	11.42	1.95E-01	11.02	21.62	6.10E-01	1.37	7.96	8.64E-01
cg04901622	-17.37	7.96	2.92E-02	-13.30	23.05	5.64E-01	-17.01	36.66	6.43E-01	-19.14	9.12	3.58E-02	-5.36	29.88	8.58E-01
cg11397135	17.28	7.92	2.92E-02	25.30	13.75	6.58E-02	20.05	13.23	1.30E-01	0.25	19.48	9.90E-01	11.45	20.90	5.84E-01

cg03415518	12.31	5.65	2.92E-02	4.40	12.57	7.26E-01	18.86	11.44	9.91E-02	9.39	8.08	2.45E-01	33.78	21.87	1.23E-01
cg08981228	21.43	9.83	2.92E-02	30.48	13.80	2.72E-02	-6.95	29.99	8.17E-01	45.98	28.97	1.13E-01	5.30	18.89	7.79E-01
cg02810660	-19.16	8.79	2.92E-02	-25.23	18.21	1.66E-01	-22.15	44.84	6.21E-01	-20.25	11.00	6.56E-02	5.25	29.14	8.57E-01
cg07148197	23.91	10.96	2.92E-02	20.44	24.70	4.08E-01	-25.24	69.72	7.17E-01	29.24	13.35	2.86E-02	7.68	33.95	8.21E-01
cg22590761	10.07	4.62	2.92E-02	14.71	6.46	2.27E-02	8.65	11.23	4.41E-01	4.35	11.43	7.04E-01	2.40	11.68	8.37E-01
cg02804087	43.50	19.95	2.92E-02	40.24	50.63	4.27E-01	225.46	158.95	1.56E-01	27.60	16.18	8.80E-02	99.60	47.68	3.67E-02
cg18155267	-7.86	3.61	2.92E-02	-9.90	4.82	3.99E-02	-5.99	7.81	4.43E-01	-2.24	18.31	9.03E-01	-5.07	8.31	5.42E-01
cg04421583	-8.38	3.84	2.92E-02	-12.71	8.40	1.30E-01	3.06	14.45	8.32E-01	-8.96	4.98	7.19E-02	-4.81	10.84	6.57E-01
cg24532400	-27.87	12.78	2.92E-02	-20.74	42.78	6.28E-01	-29.65	157.29	8.50E-01	-32.54	13.82	1.86E-02	40.02	57.40	4.86E-01
cg19730691	12.62	5.78	2.92E-02	14.25	8.75	1.03E-01	-1.41	11.45	9.02E-01	32.52	14.74	2.73E-02	11.64	10.63	2.73E-01
cg01950844	16.50	7.57	2.92E-02	7.99	12.46	5.21E-01	25.35	42.95	5.55E-01	19.32	11.83	1.02E-01	25.47	17.32	1.41E-01
cg00028929	-11.33	5.19	2.92E-02	-7.40	9.22	4.22E-01	-8.95	10.77	4.06E-01	-20.36	10.28	4.75E-02	-8.71	11.77	4.59E-01
cg00227225	28.68	13.15	2.92E-02	51.12	20.41	1.22E-02	3.38	36.76	9.27E-01	28.69	33.48	3.91E-01	8.57	23.92	7.20E-01
cg13844463	-8.99	4.12	2.92E-02	-12.64	6.18	4.07E-02	0.29	7.83	9.70E-01	-18.56	15.57	2.33E-01	-10.27	9.03	2.55E-01
cg00169354	3.62	1.66	2.92E-02	3.05	2.06	1.39E-01	5.19	3.30	1.15E-01	8.25	3.53	1.95E-02	-0.80	3.22	8.03E-01
cg21638533	6.02	2.76	2.92E-02	5.81	3.40	8.73E-02	12.68	7.41	8.68E-02	-1.96	9.96	8.44E-01	4.61	7.84	5.56E-01
cg23063070	-10.46	4.80	2.92E-02	-6.21	13.56	6.47E-01	-23.74	24.63	3.35E-01	-8.85	5.59	1.13E-01	-22.54	15.15	1.37E-01
cg14527439	8.56	3.93	2.92E-02	5.48	5.76	3.41E-01	17.85	9.12	5.02E-02	16.56	11.77	1.60E-01	3.60	8.05	6.55E-01
cg23985995	6.48	2.97	2.92E-02	9.84	4.30	2.19E-02	7.06	6.88	3.04E-01	-4.24	8.12	6.01E-01	5.09	6.66	4.45E-01
cg07823755	-12.94	5.94	2.92E-02	-9.65	15.62	5.37E-01	-19.62	30.25	5.16E-01	-13.43	7.10	5.86E-02	-11.90	17.23	4.90E-01
cg24628866	19.46	8.92	2.92E-02	40.68	19.97	4.17E-02	4.55	20.72	8.26E-01	11.32	12.68	3.72E-01	40.76	25.76	1.14E-01
cg15407257	-11.41	5.23	2.92E-02	-19.26	7.59	1.12E-02	-0.58	11.85	9.61E-01	3.72	18.28	8.39E-01	-9.87	10.51	3.48E-01
cg00655184	6.58	3.02	2.92E-02	8.37	4.36	5.49E-02	5.59	7.72	4.69E-01	9.80	8.23	2.34E-01	1.71	6.23	7.84E-01
cg21193484	-27.03	12.40	2.92E-02	-29.80	15.75	5.84E-02	-30.07	39.15	4.42E-01	-90.60	47.14	5.46E-02	0.97	25.90	9.70E-01
cg12817782	11.97	5.49	2.92E-02	3.03	12.62	8.10E-01	7.36	13.91	5.97E-01	17.25	7.74	2.58E-02	10.33	14.12	4.65E-01
cg10925729	-7.84	3.60	2.92E-02	-8.95	6.14	1.45E-01	-7.02	6.43	2.75E-01	-15.32	9.32	1.00E-01	-1.48	8.15	8.56E-01
cg04283815	10.94	5.02	2.92E-02	11.36	7.00	1.05E-01	12.21	12.25	3.19E-01	17.05	15.54	2.73E-01	5.98	10.85	5.82E-01
cg13515269	-8.98	4.12	2.92E-02	-18.44	7.60	1.52E-02	-6.88	9.50	4.69E-01	-5.89	6.68	3.78E-01	-0.24	11.05	9.83E-01
cg03793804	17.80	8.16	2.92E-02	2.79	16.25	8.63E-01	-2.67	39.37	9.46E-01	26.61	10.70	1.29E-02	13.97	23.37	5.50E-01
cg11884704	9.73	4.46	2.93E-02	10.22	5.93	8.51E-02	8.07	9.27	3.84E-01	22.24	17.55	2.05E-01	4.66	12.00	6.98E-01
cg26582706	-13.66	6.27	2.93E-02	-9.46	13.80	4.93E-01	12.57	27.65	6.49E-01	-18.17	7.71	1.85E-02	-4.43	21.81	8.39E-01
cg27217350	-9.61	4.41	2.93E-02	-7.53	5.86	1.99E-01	-7.98	11.77	4.98E-01	-6.83	11.74	5.61E-01	-21.30	11.25	5.84E-02
cg22732644	-9.54	4.38	2.93E-02	-16.46	6.95	1.78E-02	-0.89	9.16	9.23E-01	-2.13	7.86	7.87E-01	-17.12	9.43	6.94E-02
cg07270021	-15.21	6.98	2.93E-02	-28.14	10.48	7.23E-03	-24.41	18.51	1.87E-01	-4.65	5.27	3.77E-01	-18.67	15.90	2.40E-01
cg27196467	7.09	3.25	2.93E-02	6.48	3.65	7.59E-02	9.40	5.85	1.08E-01	26.54	12.01	2.71E-02	1.11	5.34	8.36E-01

cg12292531	41.57	19.07	2.93E-02	20.72	22.78	3.63E-01	0.39	77.44	9.96E-01	83.41	42.79	5.13E-02	93.23	50.28	6.37E-02
cg27067760	13.89	6.37	2.93E-02	13.61	8.36	1.03E-01	33.14	15.43	3.17E-02	1.25	24.46	9.59E-01	1.33	15.00	9.29E-01
cg06245962	-28.75	13.19	2.93E-02	-41.22	23.76	8.28E-02	99.67	180.06	5.80E-01	-34.07	16.09	3.42E-02	32.44	41.18	4.31E-01
cg06516865	-10.60	4.86	2.93E-02	-7.94	6.66	2.33E-01	-17.90	13.99	2.01E-01	-3.22	15.18	8.32E-01	-15.89	9.85	1.07E-01
cg15264811	33.53	15.38	2.93E-02	56.16	21.31	8.40E-03	22.83	44.56	6.08E-01	-8.76	53.22	8.69E-01	8.24	29.27	7.78E-01
cg02973469	12.67	5.81	2.93E-02	19.74	14.58	1.76E-01	-7.72	34.46	8.23E-01	18.65	8.04	2.04E-02	0.04	10.79	9.97E-01
cg00301601	-46.58	21.37	2.93E-02	-77.52	40.84	5.77E-02	-345.77	244.51	1.57E-01	-24.34	28.72	3.97E-01	-56.07	52.66	2.87E-01
cg00056985	24.34	11.16	2.93E-02	21.60	19.13	2.59E-01	1.64	33.92	9.61E-01	43.62	23.61	6.46E-02	21.52	19.51	2.70E-01
cg03105603	30.50	13.99	2.93E-02	21.31	27.24	4.34E-01	-51.09	74.15	4.91E-01	36.35	18.58	5.04E-02	45.54	38.27	2.34E-01
cg26203839	12.18	5.59	2.93E-02	12.63	7.72	1.02E-01	14.69	11.49	2.01E-01	26.54	19.91	1.83E-01	0.00	13.94	1.00E+00
cg27527736	36.44	16.72	2.93E-02	-17.51	51.41	7.33E-01	31.02	160.76	8.47E-01	39.39	18.60	3.42E-02	81.23	60.87	1.82E-01
cg08303283	10.33	4.74	2.93E-02	9.32	8.97	2.99E-01	6.48	10.79	5.48E-01	13.97	7.37	5.81E-02	6.15	13.93	6.59E-01
cg05854601	-7.96	3.65	2.93E-02	-7.62	8.26	3.56E-01	-6.31	16.24	6.98E-01	-9.63	4.66	3.87E-02	-1.66	9.78	8.65E-01
cg07617339	-31.78	14.58	2.93E-02	-25.26	17.05	1.39E-01	-50.61	66.33	4.45E-01	-116.11	69.38	9.42E-02	-32.52	34.69	3.48E-01
cg17073859	19.68	9.03	2.93E-02	26.82	12.38	3.02E-02	19.84	20.20	3.26E-01	-2.69	27.56	9.22E-01	10.77	22.55	6.33E-01
cg01503881	-25.12	11.53	2.93E-02	0.57	36.09	9.87E-01	-12.23	151.80	9.36E-01	-32.27	12.98	1.29E-02	3.42	35.87	9.24E-01
cg09234567	24.02	11.02	2.93E-02	35.89	38.37	3.50E-01	129.76	131.90	3.25E-01	21.24	12.73	9.53E-02	26.28	27.43	3.38E-01
cg13026773	8.60	3.94	2.93E-02	9.97	8.78	2.56E-01	25.32	12.03	3.53E-02	6.39	5.13	2.13E-01	0.91	12.45	9.42E-01
cg24498031	29.31	13.45	2.93E-02	52.75	45.63	2.48E-01	-94.43	154.76	5.42E-01	31.23	14.62	3.27E-02	-16.69	55.28	7.63E-01
cg21816456	-15.00	6.88	2.93E-02	-16.71	11.49	1.46E-01	-69.38	31.02	2.53E-02	-9.70	6.09	1.11E-01	-16.12	21.78	4.59E-01
cg10774018	35.15	16.13	2.93E-02	72.75	38.63	5.96E-02	-57.33	190.31	7.63E-01	34.35	18.96	7.00E-02	-20.77	52.29	6.91E-01
cg03549227	11.85	5.44	2.93E-02	5.71	10.33	5.80E-01	-4.63	15.72	7.69E-01	19.10	7.72	1.33E-02	12.56	16.66	4.51E-01
cg12136950	-13.06	5.99	2.93E-02	-16.78	9.10	6.52E-02	1.18	10.82	9.13E-01	-12.15	12.44	3.29E-01	-30.97	14.95	3.83E-02
cg14117166	26.82	12.31	2.93E-02	63.59	40.27	1.14E-01	66.38	132.50	6.16E-01	22.14	13.89	1.11E-01	25.96	36.71	4.79E-01
cg16416806	19.53	8.96	2.93E-02	1.60	27.14	9.53E-01	-6.87	159.95	9.66E-01	23.32	9.81	1.74E-02	-1.68	38.91	9.66E-01
cg23822732	-18.20	8.35	2.93E-02	-19.84	17.12	2.47E-01	-29.80	37.32	4.25E-01	-15.29	11.18	1.71E-01	-22.47	21.31	2.92E-01
cg11063167	25.63	11.76	2.93E-02	49.65	30.60	1.05E-01	-12.07	113.13	9.15E-01	21.82	13.38	1.03E-01	22.72	44.73	6.12E-01
cg25294502	-36.57	16.78	2.93E-02	-22.56	21.59	2.96E-01	-66.67	49.65	1.79E-01	-55.81	63.39	3.79E-01	-53.99	36.52	1.39E-01
cg24686074	23.69	10.87	2.93E-02	16.16	28.22	5.67E-01	3.28	98.36	9.73E-01	21.56	12.32	8.01E-02	73.68	44.20	9.55E-02
cg25391143	-14.48	6.65	2.93E-02	-33.77	19.81	8.81E-02	-15.19	35.62	6.70E-01	-13.52	7.40	6.77E-02	16.28	30.88	5.98E-01
cg17399362	12.92	5.93	2.93E-02	12.35	13.22	3.50E-01	-35.72	42.70	4.03E-01	16.17	7.26	2.59E-02	2.90	17.78	8.70E-01
cg15612053	20.69	9.50	2.93E-02	23.75	14.51	1.02E-01	24.97	19.07	1.90E-01	1.53	28.77	9.57E-01	19.38	20.50	3.45E-01
cg11377088	50.83	23.33	2.93E-02	66.26	33.14	4.56E-02	26.96	78.26	7.31E-01	67.45	43.71	1.23E-01	-27.64	64.52	6.68E-01
cg24167606	22.89	10.51	2.94E-02	22.72	13.87	1.02E-01	61.69	33.73	6.74E-02	36.23	34.55	2.94E-01	2.21	21.60	9.19E-01
cg22391205	-12.99	5.96	2.94E-02	-18.74	9.09	3.92E-02	17.40	18.47	3.46E-01	-16.82	13.14	2.01E-01	-13.32	10.18	1.91E-01

cg22950904	-12.36	5.68	2.94E-02	-15.26	8.60	7.61E-02	-2.57	15.98	8.72E-01	-21.07	13.50	1.19E-01	-6.40	11.09	5.64E-01
cg25503410	-14.96	6.87	2.94E-02	-9.20	16.64	5.81E-01	-12.48	31.55	6.92E-01	-13.23	8.40	1.15E-01	-34.91	20.42	8.74E-02
cg02759765	32.75	15.03	2.94E-02	17.04	34.44	6.21E-01	38.79	102.26	7.04E-01	33.29	17.97	6.39E-02	61.18	50.80	2.28E-01
cg08188486	17.75	8.15	2.94E-02	20.64	14.82	1.64E-01	27.78	15.88	8.01E-02	6.46	14.97	6.66E-01	16.54	21.94	4.51E-01
cg17786659	12.66	5.81	2.94E-02	10.97	7.76	1.58E-01	23.33	12.28	5.74E-02	7.10	25.83	7.83E-01	5.61	14.31	6.95E-01
cg14637631	28.14	12.92	2.94E-02	-0.75	24.61	9.76E-01	14.26	52.76	7.87E-01	49.08	18.11	6.73E-03	16.22	32.75	6.21E-01
cg11085282	10.90	5.00	2.94E-02	15.09	11.89	2.04E-01	20.41	12.72	1.09E-01	7.84	6.80	2.49E-01	6.50	14.03	6.43E-01
cg19198791	34.27	15.73	2.94E-02	72.38	36.64	4.82E-02	-36.37	146.93	8.04E-01	32.79	18.61	7.81E-02	-23.12	52.46	6.59E-01
cg01447902	29.73	13.65	2.94E-02	20.93	20.18	3.00E-01	47.00	26.66	7.79E-02	-21.29	50.08	6.71E-01	45.62	30.03	1.29E-01
cg21049397	-10.99	5.05	2.94E-02	-22.65	11.38	4.66E-02	-1.56	15.19	9.18E-01	-9.15	6.53	1.61E-01	-9.43	16.29	5.63E-01
cg23182674	-23.33	10.71	2.94E-02	-22.94	24.31	3.45E-01	-14.44	51.60	7.80E-01	-31.57	13.20	1.68E-02	24.20	33.14	4.65E-01
cg07803856	-8.16	3.75	2.94E-02	-4.90	5.82	4.00E-01	-8.63	9.58	3.68E-01	-5.61	8.40	5.04E-01	-15.78	7.74	4.14E-02
cg00020052	37.76	17.34	2.94E-02	70.94	18.79	1.60E-04	-21.60	55.70	6.98E-01	40.33	26.01	1.21E-01	14.58	21.17	4.91E-01
cg09844976	8.75	4.02	2.94E-02	10.98	5.66	5.25E-02	7.54	8.04	3.48E-01	3.99	16.80	8.12E-01	5.86	9.21	5.24E-01
cg16006841	2.48	1.14	2.94E-02	0.36	1.03	7.23E-01	3.63	1.68	3.04E-02	4.34	2.92	1.37E-01	4.04	2.00	4.34E-02
cg26975587	-18.21	8.36	2.94E-02	-34.87	22.22	1.16E-01	12.88	47.87	7.88E-01	-16.84	9.67	8.16E-02	-13.40	29.48	6.49E-01
cg07546334	-8.27	3.80	2.94E-02	-10.06	8.22	2.21E-01	0.97	12.64	9.39E-01	-8.62	5.03	8.71E-02	-10.27	10.62	3.33E-01
cg03588966	19.08	8.76	2.94E-02	20.39	17.75	2.51E-01	30.17	24.60	2.20E-01	17.94	11.97	1.34E-01	7.24	28.52	8.00E-01
cg10665892	-8.95	4.11	2.94E-02	-7.24	5.88	2.18E-01	-13.19	8.32	1.13E-01	-3.41	15.29	8.23E-01	-10.00	9.32	2.83E-01
cg05673079	-8.02	3.68	2.94E-02	-12.39	9.50	1.92E-01	-5.23	11.83	6.59E-01	-7.04	4.53	1.20E-01	-10.78	12.12	3.74E-01
cg03302822	-9.97	4.58	2.94E-02	-10.97	5.83	5.97E-02	-22.35	11.96	6.16E-02	-0.38	14.91	9.80E-01	0.80	12.15	9.48E-01
cg10346111	10.33	4.74	2.94E-02	-5.14	11.09	6.43E-01	26.02	20.26	1.99E-01	13.67	5.57	1.41E-02	3.81	19.06	8.42E-01
cg05189570	-10.08	4.63	2.94E-02	-23.82	12.06	4.82E-02	-3.47	25.20	8.90E-01	-5.79	5.43	2.86E-01	-24.53	15.33	1.10E-01
cg21938845	-80.91	37.15	2.94E-02	-56.39	54.28	2.99E-01	46.23	207.47	8.24E-01	-195.61	78.52	1.27E-02	-44.21	70.75	5.32E-01
cg23363911	27.11	12.45	2.94E-02	74.32	33.98	2.87E-02	146.83	152.80	3.37E-01	18.79	14.08	1.82E-01	19.04	44.91	6.72E-01
cg15721475	22.32	10.25	2.94E-02	35.43	20.58	8.51E-02	11.73	23.68	6.20E-01	12.80	15.94	4.22E-01	39.92	26.34	1.30E-01
cg15676226	32.60	14.97	2.94E-02	70.11	26.24	7.54E-03	23.47	90.68	7.96E-01	12.66	17.99	4.82E-01	34.41	38.43	3.71E-01
cg20490015	15.90	7.30	2.94E-02	7.48	16.74	6.55E-01	0.87	32.11	9.78E-01	17.67	8.93	4.80E-02	29.28	24.37	2.29E-01
cg17687282	-12.11	5.56	2.94E-02	-7.31	10.87	5.01E-01	-22.49	14.20	1.13E-01	-9.21	8.34	2.70E-01	-18.92	14.86	2.03E-01
cg15564098	10.75	4.94	2.94E-02	9.81	8.86	2.68E-01	14.25	17.55	4.17E-01	11.24	7.52	1.35E-01	9.66	11.70	4.09E-01
cg27215185	8.80	4.04	2.94E-02	9.17	5.64	1.04E-01	11.71	10.44	2.62E-01	9.76	8.85	2.70E-01	2.36	11.27	8.34E-01
cg00502926	7.33	3.37	2.94E-02	10.10	4.64	2.95E-02	10.75	7.95	1.76E-01	2.02	8.97	8.22E-01	-1.31	8.62	8.79E-01
cg15853125	26.04	11.96	2.94E-02	22.16	16.06	1.68E-01	52.00	41.48	2.10E-01	-8.02	44.82	8.58E-01	34.32	22.14	1.21E-01
cg12411309	33.49	15.38	2.94E-02	37.13	18.56	4.54E-02	-17.14	66.56	7.97E-01	-53.10	80.12	5.07E-01	48.73	32.59	1.35E-01
cg02187231	9.11	4.19	2.94E-02	14.03	6.14	2.23E-02	8.75	8.62	3.10E-01	1.79	11.23	8.73E-01	1.75	10.44	8.67E-01

cg06217312	20.19	9.27	2.94E-02	14.70	16.37	3.69E-01	7.01	39.88	8.61E-01	24.58	13.19	6.24E-02	22.54	25.64	3.79E-01
cg15504459	-41.80	19.20	2.95E-02	-53.62	24.29	2.73E-02	7.30	65.12	9.11E-01	17.44	70.73	8.05E-01	-47.60	41.38	2.50E-01
cg16145071	-61.39	28.20	2.95E-02	-75.03	37.10	4.32E-02	-13.19	151.72	9.31E-01	-82.20	65.27	2.08E-01	-11.25	62.84	8.58E-01
cg11814235	15.71	7.21	2.95E-02	16.12	11.09	1.46E-01	20.19	16.17	2.12E-01	16.02	21.54	4.57E-01	11.57	14.00	4.09E-01
cg17962756	6.42	2.95	2.95E-02	8.37	4.32	5.26E-02	6.90	6.66	3.00E-01	5.08	6.71	4.49E-01	1.28	7.75	8.69E-01
cg05255275	18.20	8.36	2.95E-02	16.26	12.33	1.87E-01	32.50	30.16	2.81E-01	54.29	37.44	1.47E-01	13.35	13.01	3.05E-01
cg24710020	20.03	9.20	2.95E-02	37.27	15.62	1.70E-02	18.18	15.04	2.27E-01	-2.13	15.24	8.89E-01	33.61	21.87	1.24E-01
cg11315544	15.57	7.15	2.95E-02	9.50	18.12	6.00E-01	19.87	20.88	3.41E-01	14.07	8.77	1.09E-01	38.47	28.57	1.78E-01
cg03736467	39.65	18.21	2.95E-02	48.15	22.65	3.35E-02	25.97	100.14	7.95E-01	15.43	94.71	8.71E-01	25.03	34.20	4.64E-01
cg00804354	9.57	4.39	2.95E-02	13.87	6.34	2.88E-02	15.72	13.53	2.45E-01	4.02	9.40	6.69E-01	1.93	9.91	8.46E-01
cg00014806	25.63	11.77	2.95E-02	23.69	31.88	4.57E-01	-152.20	127.68	2.33E-01	26.70	13.44	4.70E-02	36.48	39.70	3.58E-01
cg15134649	32.05	14.72	2.95E-02	31.11	19.84	1.17E-01	33.61	58.15	5.63E-01	16.08	32.42	6.20E-01	52.78	34.79	1.29E-01
cg20312012	4.62	2.12	2.95E-02	3.40	2.90	2.41E-01	8.82	4.74	6.28E-02	6.47	5.20	2.13E-01	-0.53	6.84	9.39E-01
cg04289208	-8.84	4.06	2.95E-02	-9.16	5.99	1.26E-01	-9.92	10.07	3.25E-01	-7.85	9.90	4.28E-01	-8.11	8.89	3.62E-01
cg06880612	12.25	5.63	2.95E-02	22.70	8.20	5.65E-03	12.34	13.58	3.64E-01	12.12	9.42	1.98E-01	-3.23	10.62	7.61E-01
cg03918756	23.86	10.96	2.95E-02	29.33	21.03	1.63E-01	76.27	33.65	2.34E-02	17.85	11.21	1.11E-01	-4.79	32.62	8.83E-01
cg21356630	-15.52	7.13	2.95E-02	-14.96	10.96	1.72E-01	-25.71	15.45	9.61E-02	-6.42	18.54	7.29E-01	-12.81	15.36	4.04E-01
cg14469684	8.28	3.81	2.95E-02	6.85	4.98	1.69E-01	16.14	9.03	7.38E-02	7.65	14.70	6.03E-01	5.27	9.16	5.65E-01
cg12274077	35.78	16.44	2.95E-02	43.08	22.16	5.19E-02	11.46	52.37	8.27E-01	4.05	48.45	9.33E-01	44.38	33.82	1.90E-01
cg04563775	54.91	25.23	2.95E-02	38.87	38.23	3.09E-01	110.96	82.12	1.77E-01	59.40	66.04	3.68E-01	58.12	44.30	1.90E-01
cg02390447	12.08	5.55	2.95E-02	7.23	7.72	3.49E-01	6.29	12.32	6.10E-01	28.56	22.85	2.11E-01	24.30	11.79	3.93E-02
cg05090695	8.36	3.84	2.95E-02	9.00	5.69	1.13E-01	11.75	8.15	1.50E-01	5.61	10.28	5.85E-01	4.74	8.98	5.98E-01
cg03148457	-7.93	3.64	2.95E-02	-9.89	4.99	4.76E-02	-8.46	8.34	3.10E-01	14.19	15.45	3.58E-01	-8.31	7.75	2.83E-01
cg16844053	5.76	2.64	2.95E-02	5.55	3.36	9.86E-02	9.30	7.15	1.93E-01	7.23	8.15	3.75E-01	2.04	7.13	7.75E-01
cg14747498	18.35	8.43	2.95E-02	4.11	11.64	7.24E-01	22.46	14.51	1.22E-01	10.07	18.32	5.82E-01	41.31	15.37	7.19E-03
cg23703079	7.48	3.44	2.95E-02	8.42	5.73	1.42E-01	7.08	7.50	3.45E-01	5.53	8.37	5.09E-01	7.79	6.73	2.47E-01
cg10884174	36.56	16.80	2.95E-02	32.13	19.53	9.99E-02	126.78	68.14	6.28E-02	37.47	63.20	5.53E-01	18.93	46.74	6.86E-01
cg26962618	8.37	3.85	2.95E-02	8.65	5.44	1.12E-01	8.15	7.44	2.74E-01	8.98	12.59	4.76E-01	7.43	10.28	4.70E-01
cg25597535	-10.27	4.72	2.95E-02	-13.35	6.33	3.49E-02	-0.24	9.46	9.80E-01	-19.11	5.97	1.36E-03	0.36	9.27	9.69E-01
cg25219939	-15.35	7.05	2.95E-02	0.11	20.04	9.95E-01	-2.27	37.99	9.52E-01	-15.54	9.51	1.02E-01	-23.09	13.05	7.69E-02
cg13880167	-17.58	8.08	2.95E-02	-27.59	17.18	1.08E-01	-6.81	44.68	8.79E-01	-18.93	9.89	5.57E-02	17.31	28.72	5.47E-01
cg00213745	-17.35	7.97	2.95E-02	-12.95	20.37	5.25E-01	-10.60	27.88	7.04E-01	-15.19	9.62	1.15E-01	-51.94	28.47	6.80E-02
cg24508143	42.80	19.67	2.95E-02	10.38	15.26	4.97E-01	34.58	60.12	5.65E-01	91.76	41.14	2.57E-02	60.69	20.46	3.01E-03
cg05097455	-41.25	18.95	2.95E-02	-51.72	24.53	3.50E-02	64.57	76.86	4.01E-01	-48.98	73.94	5.08E-01	-40.08	36.07	2.66E-01
cg09240763	30.13	13.84	2.95E-02	8.02	12.27	5.14E-01	31.92	28.04	2.55E-01	79.89	33.65	1.76E-02	38.49	19.73	5.11E-02

cg27169858	19.32	8.88	2.95E-02	6.44	16.20	6.91E-01	-35.82	66.97	5.93E-01	27.69	11.54	1.64E-02	18.04	29.57	5.42E-01
cg14601050	-36.63	16.83	2.95E-02	-36.51	22.56	1.06E-01	61.17	68.50	3.72E-01	-78.73	50.61	1.20E-01	-41.44	32.23	1.99E-01
cg02031712	-8.15	3.74	2.95E-02	-11.58	6.05	5.57E-02	6.95	13.12	5.96E-01	-10.76	6.02	7.41E-02	-0.86	9.65	9.29E-01
cg05400741	-36.96	16.99	2.95E-02	-36.76	20.52	7.32E-02	-58.40	72.65	4.21E-01	-7.70	61.47	9.00E-01	-43.53	39.67	2.73E-01
cg06495501	45.01	20.68	2.95E-02	47.61	18.32	9.34E-03	57.36	62.91	3.62E-01	122.81	51.41	1.69E-02	1.93	29.62	9.48E-01
cg16457196	23.73	10.90	2.96E-02	17.37	16.39	2.89E-01	-40.32	45.15	3.72E-01	26.99	14.27	5.86E-02	53.84	27.20	4.78E-02
cg05526376	-44.26	20.34	2.96E-02	-82.47	53.21	1.21E-01	115.88	209.37	5.80E-01	-35.94	23.58	1.27E-01	-65.49	64.31	3.09E-01
cg08039322	20.15	9.26	2.96E-02	3.76	21.96	8.64E-01	52.84	92.57	5.68E-01	24.42	11.04	2.70E-02	16.31	28.00	5.60E-01
cg24472965	-10.29	4.73	2.96E-02	-11.86	8.62	1.69E-01	-6.04	11.16	5.88E-01	-10.38	7.10	1.44E-01	-13.62	17.19	4.28E-01
cg06000951	19.42	8.93	2.96E-02	48.00	17.96	7.54E-03	17.45	23.83	4.64E-01	18.85	10.29	6.69E-02	1.69	13.77	9.02E-01
cg16232126	10.82	4.97	2.96E-02	16.66	9.87	9.14E-02	5.79	12.25	6.36E-01	12.05	8.61	1.62E-01	6.52	9.99	5.14E-01
cg02440214	19.96	9.17	2.96E-02	23.01	19.12	2.29E-01	26.03	38.76	5.02E-01	20.96	12.11	8.36E-02	8.47	24.47	7.29E-01
cg03045648	16.34	7.51	2.96E-02	23.95	12.14	4.85E-02	22.42	15.33	1.44E-01	-4.17	15.40	7.87E-01	19.97	20.11	3.21E-01
cg22754866	-10.50	4.82	2.96E-02	-13.04	12.18	2.84E-01	-10.36	26.46	6.95E-01	-12.17	5.82	3.65E-02	2.13	13.78	8.77E-01
cg26275242	18.18	8.36	2.96E-02	16.63	22.55	4.61E-01	0.78	59.12	9.89E-01	18.56	9.55	5.20E-02	21.72	30.12	4.71E-01
cg11118171	15.36	7.06	2.96E-02	14.69	9.17	1.09E-01	-3.86	20.51	8.51E-01	17.53	23.51	4.56E-01	27.85	15.84	7.86E-02
cg22061831	11.89	5.47	2.96E-02	16.23	19.46	4.04E-01	-0.34	29.41	9.91E-01	11.61	6.34	6.70E-02	13.90	14.44	3.36E-01
cg10530680	-12.51	5.75	2.96E-02	-20.56	14.22	1.48E-01	-6.52	16.93	7.00E-01	-7.90	7.49	2.91E-01	-28.55	15.90	7.26E-02
cg14183693	-13.96	6.42	2.96E-02	-11.14	17.31	5.20E-01	-26.65	20.96	2.04E-01	-12.15	8.32	1.44E-01	-15.54	15.37	3.12E-01
cg23548686	-40.50	18.62	2.96E-02	-44.56	22.67	4.93E-02	-93.66	79.43	2.38E-01	43.50	61.66	4.80E-01	-51.61	43.96	2.40E-01
cg24527461	11.75	5.40	2.96E-02	9.26	8.82	2.94E-01	10.89	9.57	2.55E-01	12.12	12.18	3.20E-01	22.22	16.38	1.75E-01
cg26946015	-9.88	4.54	2.96E-02	-16.99	8.86	5.51E-02	6.42	12.10	5.96E-01	-9.92	6.87	1.49E-01	-12.43	11.37	2.74E-01
cg00561903	8.54	3.93	2.96E-02	14.28	7.68	6.28E-02	-5.47	15.25	7.20E-01	4.99	5.28	3.45E-01	20.28	11.40	7.53E-02
cg15459165	-8.70	4.00	2.96E-02	-5.15	5.34	3.35E-01	-15.06	11.89	2.05E-01	-2.06	10.85	8.49E-01	-20.20	9.20	2.80E-02
cg23580287	16.27	7.48	2.96E-02	17.80	19.53	3.62E-01	1.57	24.39	9.49E-01	16.63	9.12	6.81E-02	26.99	25.55	2.91E-01
cg14468090	12.34	5.67	2.96E-02	15.60	8.38	6.28E-02	16.09	11.47	1.61E-01	10.27	15.81	5.16E-01	-0.37	13.83	9.79E-01
cg10979364	-10.76	4.95	2.96E-02	-23.46	10.85	3.06E-02	-5.30	21.92	8.09E-01	-6.47	6.17	2.94E-01	-14.91	15.89	3.48E-01
cg27431587	18.48	8.50	2.96E-02	18.53	12.75	1.46E-01	19.06	19.35	3.24E-01	15.94	17.17	3.53E-01	22.62	24.68	3.60E-01
cg05304979	-11.63	5.34	2.96E-02	-34.42	18.39	6.12E-02	2.79	81.71	9.73E-01	-7.64	5.95	2.00E-01	-24.41	16.44	1.38E-01
cg00543196	-47.93	22.04	2.96E-02	-12.80	37.29	7.31E-01	-344.51	249.80	1.68E-01	-68.88	27.06	1.09E-02	-12.12	74.68	8.71E-01
cg00359635	12.44	5.72	2.96E-02	18.84	17.15	2.72E-01	25.13	22.90	2.72E-01	9.42	6.49	1.47E-01	28.53	25.17	2.57E-01
cg13320585	-8.35	3.84	2.96E-02	-2.68	7.27	7.12E-01	1.67	10.86	8.78E-01	-14.01	5.64	1.30E-02	-9.96	10.54	3.44E-01
cg22740796	-7.61	3.50	2.96E-02	-8.55	5.21	1.01E-01	0.16	6.06	9.79E-01	-20.72	12.68	1.02E-01	-12.51	7.40	9.08E-02
cg10633931	-42.97	19.76	2.96E-02	-109.72	51.31	3.25E-02	83.89	177.63	6.37E-01	-28.63	23.09	2.15E-01	-63.22	60.32	2.95E-01
cg09699369	-31.47	14.47	2.96E-02	-69.00	50.71	1.74E-01	-244.84	235.10	2.98E-01	-27.73	15.91	8.13E-02	-22.69	48.95	6.43E-01

cg14294250	18.97	8.72	2.96E-02	4.26	32.60	8.96E-01	15.11	106.62	8.87E-01	18.99	9.30	4.11E-02	44.00	42.41	2.99E-01
cg01546873	-9.45	4.35	2.96E-02	-14.35	11.64	2.17E-01	-31.01	18.47	9.32E-02	-5.80	5.11	2.56E-01	-18.79	15.20	2.16E-01
cg11839815	5.14	2.36	2.96E-02	5.64	3.38	9.53E-02	7.83	5.50	1.54E-01	4.09	5.13	4.26E-01	0.60	6.99	9.31E-01
cg19655510	16.40	7.54	2.96E-02	40.51	18.85	3.16E-02	18.43	19.73	3.50E-01	10.32	9.57	2.81E-01	11.15	27.99	6.90E-01
cg09916577	18.96	8.72	2.96E-02	24.56	17.25	1.55E-01	37.60	18.67	4.40E-02	5.05	14.42	7.26E-01	16.39	21.69	4.50E-01
cg22531183	20.37	9.36	2.96E-02	-7.23	24.88	7.71E-01	-26.92	64.62	6.77E-01	30.26	11.44	8.17E-03	10.05	22.88	6.61E-01
cg15010184	16.62	7.64	2.96E-02	7.35	17.37	6.72E-01	-30.89	62.96	6.24E-01	16.52	9.11	6.98E-02	45.48	25.66	7.64E-02
cg14183184	33.62	15.46	2.96E-02	36.09	20.75	8.20E-02	-21.86	44.00	6.19E-01	76.81	49.11	1.18E-01	38.99	32.76	2.34E-01
cg12277532	24.71	11.36	2.96E-02	16.14	14.12	2.53E-01	-13.68	61.95	8.25E-01	-4.12	66.92	9.51E-01	51.15	21.10	1.53E-02
cg12965599	9.79	4.50	2.97E-02	9.87	7.59	1.94E-01	-2.74	13.05	8.34E-01	18.13	9.19	4.85E-02	7.92	8.37	3.44E-01
cg22568673	72.38	33.28	2.97E-02	61.89	35.70	8.29E-02	226.73	84.12	7.03E-03	71.65	75.52	3.43E-01	23.91	43.75	5.85E-01
cg08768904	17.07	7.85	2.97E-02	28.41	16.28	8.09E-02	31.03	15.62	4.69E-02	6.80	12.43	5.84E-01	-0.75	23.02	9.74E-01
cg24141036	-30.12	13.85	2.97E-02	-58.99	43.09	1.71E-01	54.44	144.93	7.07E-01	-25.87	15.42	9.34E-02	-45.26	48.75	3.53E-01
cg07003920	33.76	15.52	2.97E-02	40.28	20.66	5.13E-02	35.76	61.26	5.59E-01	85.08	61.62	1.67E-01	10.82	27.98	6.99E-01
cg00462559	-12.63	5.81	2.97E-02	-25.10	16.06	1.18E-01	-37.57	28.01	1.80E-01	-7.91	6.62	2.32E-01	-29.23	24.51	2.33E-01
cg13151006	-9.71	4.47	2.97E-02	-19.01	11.31	9.29E-02	-13.40	17.35	4.40E-01	-8.76	5.32	9.98E-02	4.22	16.51	7.98E-01
cg21775463	-11.11	5.11	2.97E-02	-10.66	7.94	1.79E-01	-13.26	11.51	2.49E-01	0.59	13.15	9.64E-01	-17.51	10.46	9.43E-02
cg06543101	-15.62	7.18	2.97E-02	-23.61	10.54	2.51E-02	1.24	16.50	9.40E-01	-18.94	19.01	3.19E-01	-10.75	15.95	5.00E-01
cg12592635	24.71	11.36	2.97E-02	38.56	17.90	3.12E-02	6.50	38.43	8.66E-01	56.13	36.43	1.23E-01	7.62	17.70	6.67E-01
cg12615150	17.89	8.23	2.97E-02	22.87	20.03	2.54E-01	41.32	33.03	2.11E-01	18.80	9.92	5.81E-02	-18.05	28.90	5.32E-01
cg06521597	40.21	18.50	2.97E-02	23.28	41.00	5.70E-01	6.18	105.41	9.53E-01	47.04	23.01	4.09E-02	40.92	53.54	4.45E-01
cg03464573	19.05	8.76	2.97E-02	26.73	11.48	1.99E-02	32.89	32.64	3.14E-01	-48.01	48.71	3.24E-01	8.52	15.64	5.86E-01
cg00405043	21.08	9.69	2.97E-02	30.31	21.57	1.60E-01	24.81	19.26	1.98E-01	7.01	12.67	5.80E-01	61.26	29.59	3.85E-02
cg20633317	21.18	9.74	2.97E-02	14.11	19.34	4.66E-01	45.44	29.43	1.23E-01	23.06	15.81	1.45E-01	15.03	19.21	4.34E-01
cg07888967	14.52	6.68	2.97E-02	21.00	10.70	4.97E-02	5.75	16.32	7.24E-01	8.98	14.41	5.33E-01	15.09	13.96	2.80E-01
cg12675906	5.28	2.43	2.97E-02	2.56	4.25	5.46E-01	0.06	5.72	9.91E-01	10.12	4.17	1.52E-02	6.48	6.20	2.96E-01
cg00038298	-14.48	6.66	2.97E-02	-18.24	13.06	1.63E-01	-17.34	22.07	4.32E-01	-17.05	9.29	6.66E-02	4.45	18.14	8.06E-01
cg18413900	10.56	4.86	2.97E-02	6.03	6.64	3.64E-01	24.75	12.26	4.36E-02	16.76	12.23	1.70E-01	5.37	12.53	6.68E-01
cg07880727	7.06	3.25	2.97E-02	7.32	4.32	9.00E-02	16.86	9.41	7.31E-02	4.57	9.04	6.13E-01	1.72	7.53	8.19E-01
cg06146607	24.76	11.39	2.97E-02	32.46	16.38	4.75E-02	27.95	29.81	3.48E-01	5.77	35.03	8.69E-01	16.51	22.12	4.55E-01
cg17196051	-10.66	4.90	2.97E-02	-17.37	10.42	9.56E-02	-1.05	20.10	9.58E-01	-6.50	6.27	3.00E-01	-25.97	15.00	8.35E-02
cg13782937	-7.96	3.66	2.97E-02	-13.91	8.73	1.11E-01	-5.69	14.16	6.88E-01	-4.01	4.59	3.82E-01	-21.34	10.53	4.28E-02
cg11334644	9.11	4.19	2.97E-02	2.28	5.86	6.97E-01	15.66	10.39	1.32E-01	18.83	19.30	3.29E-01	16.18	7.94	4.16E-02
cg21167532	-77.99	35.88	2.97E-02	-62.07	48.46	2.00E-01	167.62	160.90	2.98E-01	-128.53	61.91	3.79E-02	-102.83	86.57	2.35E-01
cg02453146	19.18	8.82	2.97E-02	10.11	14.06	4.72E-01	5.63	35.88	8.75E-01	21.25	19.76	2.82E-01	30.66	14.99	4.08E-02

cg00779056	-25.42	11.70	2.97E-02	-21.62	16.94	2.02E-01	-37.83	47.11	4.22E-01	-34.31	23.34	1.42E-01	-19.80	25.49	4.37E-01
cg24627517	46.44	21.37	2.97E-02	42.30	31.42	1.78E-01	133.48	88.73	1.33E-01	7.32	51.75	8.88E-01	57.88	38.43	1.32E-01
cg19405718	39.66	18.24	2.97E-02	35.31	23.55	1.34E-01	52.39	83.70	5.31E-01	20.51	47.26	6.64E-01	63.55	40.46	1.16E-01
cg08570521	15.09	6.94	2.97E-02	9.26	6.32	1.43E-01	50.83	86.09	5.55E-01	102.57	53.90	5.70E-02	19.22	8.64	2.60E-02
cg07872373	-5.99	2.76	2.97E-02	-3.86	3.72	2.99E-01	-5.78	5.70	3.11E-01	1.43	15.93	9.28E-01	-13.69	6.37	3.16E-02
cg03393754	-16.47	7.58	2.97E-02	-24.91	22.49	2.68E-01	-11.28	32.66	7.30E-01	-16.35	8.67	5.95E-02	-8.04	28.85	7.81E-01
cg25962829	-13.88	6.39	2.97E-02	-24.06	10.01	1.62E-02	-7.71	12.38	5.33E-01	-20.40	21.01	3.31E-01	-0.61	13.21	9.63E-01
cg15536401	-8.60	3.96	2.97E-02	-8.51	10.40	4.13E-01	-2.11	19.90	9.15E-01	-8.68	4.63	6.08E-02	-11.07	13.53	4.14E-01
cg05624376	5.22	2.40	2.97E-02	4.13	3.35	2.19E-01	7.22	4.80	1.32E-01	10.03	6.72	1.35E-01	0.15	7.25	9.83E-01
cg19632236	-32.46	14.94	2.98E-02	-17.86	19.52	3.60E-01	-18.72	61.90	7.62E-01	-6.23	55.28	9.10E-01	-72.23	28.06	1.01E-02
cg26665246	28.33	13.04	2.98E-02	39.57	39.29	3.14E-01	-36.69	118.59	7.57E-01	24.48	14.51	9.17E-02	65.98	49.02	1.78E-01
cg23709430	-12.97	5.97	2.98E-02	-7.71	10.95	4.82E-01	-14.62	13.48	2.78E-01	-17.43	10.31	9.10E-02	-11.50	14.39	4.24E-01
cg08544192	-9.61	4.42	2.98E-02	-15.66	7.56	3.84E-02	10.35	11.75	3.78E-01	-9.14	4.68	5.07E-02	-16.46	9.74	9.09E-02
cg12898275	-24.58	11.31	2.98E-02	-63.65	45.19	1.59E-01	-53.64	160.38	7.38E-01	-22.77	11.95	5.66E-02	2.38	59.52	9.68E-01
cg18186672	12.80	5.89	2.98E-02	14.99	7.96	5.95E-02	12.59	14.72	3.92E-01	3.67	25.93	8.87E-01	9.89	11.99	4.10E-01
cg00935108	7.84	3.61	2.98E-02	7.25	4.56	1.12E-01	18.47	7.44	1.30E-02	1.79	10.21	8.61E-01	-0.35	8.85	9.68E-01
cg12581592	17.42	8.01	2.98E-02	22.59	11.37	4.71E-02	-6.36	22.38	7.76E-01	22.98	19.14	2.30E-01	14.95	17.93	4.04E-01
cg20672477	-8.01	3.68	2.98E-02	-7.72	5.31	1.46E-01	-5.40	8.80	5.39E-01	-16.09	15.29	2.93E-01	-8.43	6.89	2.21E-01
cg19135706	26.28	12.10	2.98E-02	28.55	16.30	7.99E-02	22.15	66.35	7.38E-01	97.36	52.82	6.53E-02	13.00	20.05	5.17E-01
cg22924015	19.39	8.92	2.98E-02	1.39	22.99	9.52E-01	51.41	49.22	2.96E-01	20.64	10.42	4.76E-02	28.38	31.01	3.60E-01
cg07681935	10.67	4.91	2.98E-02	6.89	13.28	6.04E-01	13.57	26.84	6.13E-01	9.46	5.80	1.03E-01	22.14	14.62	1.30E-01
cg14621254	7.53	3.46	2.98E-02	8.10	4.89	9.74E-02	14.38	7.77	6.44E-02	-6.79	13.01	6.02E-01	4.75	7.26	5.12E-01
cg26673073	49.63	22.84	2.98E-02	64.80	32.47	4.59E-02	1.86	65.75	9.77E-01	-5.32	50.62	9.16E-01	101.89	53.72	5.79E-02
cg20348746	6.09	2.80	2.98E-02	9.15	3.58	1.06E-02	-1.41	5.05	7.79E-01	3.82	6.31	5.45E-01	11.17	5.79	5.37E-02
cg14241370	8.26	3.80	2.98E-02	9.46	5.76	1.00E-01	19.90	9.71	4.05E-02	2.00	8.31	8.09E-01	3.31	8.48	6.97E-01
cg06432479	31.42	14.46	2.98E-02	46.26	19.15	1.57E-02	41.55	49.12	3.98E-01	46.56	28.32	1.00E-01	-10.76	26.58	6.86E-01
cg04709931	-60.89	28.03	2.98E-02	-44.50	29.91	1.37E-01	-91.43	105.52	3.86E-01	13.96	77.31	8.57E-01	-144.51	58.72	1.39E-02
cg03660603	-10.35	4.76	2.98E-02	-10.87	11.22	3.33E-01	0.40	20.90	9.85E-01	-11.02	5.95	6.40E-02	-10.59	13.34	4.28E-01
cg14181409	6.53	3.01	2.98E-02	4.78	3.87	2.16E-01	14.05	5.29	7.86E-03	-4.20	10.36	6.85E-01	4.30	6.67	5.19E-01
cg11364645	46.82	21.55	2.98E-02	37.55	25.80	1.46E-01	44.86	121.12	7.11E-01	32.69	63.60	6.07E-01	99.14	54.60	6.94E-02
cg10705503	-21.86	10.06	2.98E-02	-28.81	19.72	1.44E-01	7.25	35.70	8.39E-01	-26.94	13.86	5.19E-02	-5.56	27.53	8.40E-01
cg17480641	-15.75	7.25	2.98E-02	-33.81	13.50	1.23E-02	19.52	25.46	4.43E-01	-15.48	6.57	1.84E-02	-8.33	16.26	6.09E-01
cg17795258	16.32	7.51	2.98E-02	8.18	17.08	6.32E-01	10.62	35.21	7.63E-01	20.53	9.35	2.82E-02	8.75	22.08	6.92E-01
cg27223543	20.60	9.48	2.98E-02	23.79	10.91	2.93E-02	2.30	44.17	9.59E-01	31.36	71.00	6.59E-01	10.92	22.25	6.24E-01
cg05387519	17.41	8.01	2.98E-02	37.83	18.88	4.51E-02	-6.13	24.66	8.04E-01	23.39	11.06	3.44E-02	3.93	14.47	7.86E-01

cg09913171	-28.68	13.20	2.98E-02	-31.10	21.01	1.39E-01	6.19	52.43	9.06E-01	-48.09	23.09	3.73E-02	-5.02	28.47	8.60E-01
cg19561908	10.81	4.98	2.98E-02	12.18	7.01	8.22E-02	4.22	10.19	6.79E-01	11.14	15.51	4.72E-01	16.33	12.69	1.98E-01
cg07219227	8.85	4.08	2.98E-02	4.46	5.97	4.55E-01	17.59	10.09	8.12E-02	18.28	16.03	2.54E-01	8.88	7.36	2.28E-01
cg10690277	-18.56	8.54	2.98E-02	-37.87	17.22	2.79E-02	-46.62	37.77	2.17E-01	-7.63	9.30	4.12E-01	-27.56	24.71	2.65E-01
cg09745873	22.00	10.13	2.98E-02	23.18	17.98	1.97E-01	-3.23	21.85	8.83E-01	16.70	12.62	1.86E-01	54.72	20.83	8.63E-03
cg22532907	-13.81	6.36	2.98E-02	-3.10	18.81	8.69E-01	-6.20	28.36	8.27E-01	-14.37	7.19	4.55E-02	-36.23	27.83	1.93E-01
cg15670706	21.01	9.67	2.99E-02	20.68	18.81	2.72E-01	44.93	19.96	2.44E-02	12.65	16.21	4.35E-01	3.41	25.39	8.93E-01
cg07397612	-11.17	5.14	2.99E-02	-12.56	11.08	2.57E-01	-3.82	24.78	8.77E-01	-11.78	6.13	5.48E-02	-0.63	26.05	9.81E-01
cg16988970	-8.61	3.96	2.99E-02	-6.08	5.43	2.63E-01	-5.01	8.44	5.53E-01	-12.97	14.28	3.64E-01	-19.28	9.63	4.53E-02
cg23541422	-21.26	9.79	2.99E-02	-33.04	15.92	3.80E-02	-4.07	25.60	8.74E-01	-24.50	18.39	1.83E-01	-6.45	22.27	7.72E-01
cg07143418	20.94	9.64	2.99E-02	39.99	21.51	6.29E-02	-0.71	30.54	9.81E-01	14.94	13.52	2.69E-01	28.19	22.07	2.01E-01
cg08543015	-8.17	3.76	2.99E-02	-7.91	5.67	1.63E-01	-15.94	8.08	4.86E-02	-6.40	10.91	5.57E-01	-2.13	7.94	7.89E-01
cg17739428	25.38	11.68	2.99E-02	38.21	17.15	2.59E-02	-15.67	32.89	6.34E-01	1.49	27.39	9.57E-01	38.01	19.71	5.38E-02
cg07210454	-10.04	4.62	2.99E-02	-10.22	10.10	3.12E-01	-12.28	15.15	4.18E-01	-12.59	6.35	4.73E-02	-0.44	11.33	9.69E-01
cg08594651	6.55	3.02	2.99E-02	5.36	3.93	1.73E-01	12.25	6.97	7.87E-02	3.17	10.11	7.54E-01	6.06	8.20	4.60E-01
cg16465012	19.47	8.97	2.99E-02	23.31	14.75	1.14E-01	15.98	16.63	3.37E-01	-3.90	19.89	8.44E-01	48.09	23.00	3.66E-02
cg10074813	15.94	7.34	2.99E-02	7.37	11.68	5.28E-01	21.80	13.46	1.05E-01	13.78	17.79	4.39E-01	30.48	19.75	1.23E-01
cg01283096	13.80	6.35	2.99E-02	19.63	7.93	1.33E-02	14.30	11.87	2.28E-01	34.35	19.27	7.46E-02	-1.21	9.84	9.02E-01
cg18315831	-8.92	4.11	2.99E-02	-22.04	9.90	2.60E-02	-0.63	17.35	9.71E-01	-5.18	5.07	3.07E-01	-14.59	12.05	2.26E-01
cg18447727	20.41	9.40	2.99E-02	30.10	15.73	5.57E-02	0.56	27.98	9.84E-01	30.75	23.79	1.96E-01	12.84	15.37	4.03E-01
cg11510999	6.24	2.88	2.99E-02	4.50	3.54	2.03E-01	12.81	6.40	4.53E-02	5.89	13.32	6.58E-01	4.49	9.59	6.39E-01
cg27507681	-8.60	3.96	2.99E-02	-5.32	5.38	3.22E-01	-10.04	9.87	3.09E-01	-30.09	14.22	3.44E-02	-8.05	8.46	3.41E-01
cg16226962	13.72	6.32	2.99E-02	19.12	9.70	4.87E-02	9.60	15.94	5.47E-01	8.98	16.37	5.83E-01	10.23	12.15	4.00E-01
cg09754110	-58.57	26.97	2.99E-02	-31.58	24.00	1.88E-01	49.03	79.66	5.38E-01	-104.63	40.49	9.76E-03	-98.87	42.95	2.14E-02
cg19267259	33.10	15.24	2.99E-02	55.59	41.58	1.81E-01	1.88	159.09	9.91E-01	34.16	17.50	5.09E-02	-3.05	48.67	9.50E-01
cg14984246	-28.98	13.35	2.99E-02	-24.30	17.05	1.54E-01	-42.99	45.75	3.47E-01	30.73	51.29	5.49E-01	-53.37	27.56	5.28E-02
cg05158197	18.25	8.40	2.99E-02	28.91	13.22	2.87E-02	6.43	32.76	8.44E-01	34.08	22.58	1.31E-01	3.63	13.43	7.87E-01
cg10766103	33.11	15.25	2.99E-02	64.82	46.27	1.61E-01	93.78	188.07	6.18E-01	33.93	17.08	4.70E-02	-17.74	51.25	7.29E-01
cg06032735	-8.89	4.09	2.99E-02	-3.94	10.77	7.14E-01	-7.30	14.57	6.16E-01	-10.00	4.99	4.50E-02	-9.79	12.78	4.43E-01
cg05045969	17.56	8.09	2.99E-02	14.81	10.05	1.41E-01	49.08	33.97	1.49E-01	-0.52	36.18	9.88E-01	21.19	16.30	1.94E-01
cg18331412	8.16	3.76	2.99E-02	7.99	5.46	1.43E-01	17.35	8.50	4.12E-02	13.24	8.51	1.20E-01	-1.25	6.83	8.55E-01
cg23281012	-29.27	13.48	2.99E-02	-23.08	17.77	1.94E-01	-44.10	39.50	2.64E-01	-81.80	71.39	2.52E-01	-29.13	25.85	2.60E-01
cg18137704	12.46	5.74	2.99E-02	21.14	8.90	1.76E-02	2.54	14.52	8.61E-01	6.88	12.55	5.83E-01	8.40	12.26	4.93E-01
cg07054668	-11.29	5.20	2.99E-02	-10.54	14.24	4.59E-01	23.91	38.43	5.34E-01	-12.35	5.90	3.65E-02	-10.21	19.25	5.96E-01
cg05958702	21.38	9.85	2.99E-02	30.86	13.25	1.98E-02	21.32	30.80	4.89E-01	41.91	35.71	2.41E-01	-3.86	18.97	8.39E-01

cg02167203	3.51	1.61	2.99E-02	2.00	2.55	4.32E-01	4.64	4.22	2.72E-01	2.92	2.67	2.74E-01	11.11	5.51	4.36E-02
cg06346667	-14.72	6.78	2.99E-02	-8.69	10.14	3.91E-01	-24.36	12.10	4.40E-02	27.66	35.37	4.34E-01	-20.82	15.11	1.68E-01
cg11392776	18.63	8.58	2.99E-02	33.95	14.47	1.90E-02	29.47	15.53	5.76E-02	3.77	15.54	8.08E-01	0.11	18.80	9.95E-01
cg03034864	-38.96	17.95	2.99E-02	-37.42	23.51	1.11E-01	-76.18	82.29	3.55E-01	64.09	71.67	3.71E-01	-57.16	32.39	7.76E-02
cg19079000	44.56	20.53	2.99E-02	20.80	46.01	6.51E-01	180.81	210.26	3.90E-01	53.01	25.19	3.54E-02	27.52	57.44	6.32E-01
cg26382697	-8.45	3.89	2.99E-02	-10.51	5.15	4.14E-02	-11.86	8.89	1.82E-01	-0.37	15.00	9.80E-01	-0.88	9.45	9.25E-01
cg04844714	27.58	12.70	2.99E-02	36.82	18.20	4.31E-02	31.21	43.99	4.78E-01	31.28	33.67	3.53E-01	9.02	23.70	7.03E-01
cg12594793	-9.82	4.53	2.99E-02	-10.82	6.17	7.98E-02	-4.38	13.45	7.44E-01	-2.10	20.70	9.19E-01	-11.32	8.24	1.69E-01
cg15108590	15.98	7.36	3.00E-02	15.95	13.11	2.24E-01	-8.08	21.30	7.04E-01	19.95	12.76	1.18E-01	22.69	15.27	1.37E-01
cg01382671	-15.13	6.97	3.00E-02	-15.80	7.52	3.56E-02	-6.98	38.29	8.55E-01	-54.86	60.71	3.66E-01	-6.34	22.79	7.81E-01
cg20103758	23.23	10.70	3.00E-02	31.46	15.68	4.48E-02	-7.52	30.22	8.03E-01	62.71	37.61	9.55E-02	13.54	18.71	4.69E-01
cg17173896	7.72	3.56	3.00E-02	7.06	4.83	1.44E-01	15.31	8.28	6.44E-02	1.29	10.43	9.01E-01	5.84	9.01	5.17E-01
cg00403579	-18.81	8.67	3.00E-02	-35.25	14.09	1.24E-02	-9.10	20.22	6.53E-01	-5.82	20.09	7.72E-01	-10.82	17.27	5.31E-01
cg23909633	-9.88	4.55	3.00E-02	0.47	13.72	9.73E-01	-15.13	26.33	5.66E-01	-11.35	5.24	3.04E-02	-8.73	14.06	5.35E-01
cg22421859	23.62	10.88	3.00E-02	20.27	13.44	1.31E-01	20.19	61.98	7.45E-01	114.39	55.80	4.04E-02	19.44	20.76	3.49E-01
cg26349048	-7.40	3.41	3.00E-02	-4.94	5.87	4.00E-01	-5.51	9.08	5.44E-01	-8.77	5.43	1.07E-01	-11.69	9.49	2.18E-01
cg01651364	51.23	23.60	3.00E-02	20.07	17.74	2.58E-01	55.33	79.49	4.86E-01	132.01	66.13	4.59E-02	78.27	35.78	2.87E-02
cg06800849	-11.07	5.10	3.00E-02	-12.03	8.22	1.43E-01	-10.94	17.83	5.39E-01	-13.89	11.33	2.20E-01	-8.26	8.87	3.52E-01
cg19489885	-16.00	7.37	3.00E-02	-22.81	12.08	5.89E-02	-2.86	15.30	8.51E-01	-17.00	16.55	3.04E-01	-17.58	16.61	2.90E-01
cg24987622	-11.76	5.42	3.00E-02	-21.00	8.21	1.05E-02	-2.24	12.14	8.53E-01	-2.59	17.06	8.79E-01	-7.20	10.53	4.94E-01
cg08230648	-9.88	4.55	3.00E-02	-21.77	10.26	3.39E-02	-3.10	11.19	7.82E-01	-8.97	6.08	1.40E-01	-0.69	16.42	9.67E-01
cg15924691	-11.32	5.21	3.00E-02	-23.71	7.65	1.93E-03	-7.72	10.32	4.55E-01	-2.68	6.50	6.80E-01	-11.99	10.80	2.67E-01
cg10089801	9.19	4.24	3.00E-02	16.66	7.33	2.30E-02	5.57	9.10	5.41E-01	11.87	9.96	2.33E-01	1.03	8.17	8.99E-01
cg06236559	10.43	4.80	3.00E-02	15.49	6.19	1.23E-02	3.66	14.81	8.05E-01	2.59	22.35	9.08E-01	2.41	9.68	8.04E-01
cg06385152	-27.10	12.49	3.00E-02	-25.60	15.54	9.96E-02	-53.16	37.51	1.56E-01	-6.70	52.25	8.98E-01	-23.07	28.91	4.25E-01
cg01981445	22.02	10.15	3.00E-02	33.09	20.38	1.05E-01	7.73	17.55	6.60E-01	21.28	19.43	2.73E-01	37.40	26.62	1.60E-01
cg14104327	-10.74	4.95	3.00E-02	-11.17	10.58	2.91E-01	5.87	15.78	7.10E-01	-10.24	6.78	1.31E-01	-22.76	12.78	7.49E-02
cg14772118	19.18	8.84	3.00E-02	18.99	15.36	2.16E-01	-8.01	32.62	8.06E-01	25.29	12.65	4.56E-02	10.54	26.95	6.96E-01
cg10358981	13.72	6.32	3.00E-02	13.88	10.74	1.96E-01	16.67	17.97	3.54E-01	15.72	16.85	3.51E-01	11.91	10.14	2.40E-01
cg02372613	23.13	10.66	3.00E-02	25.06	18.99	1.87E-01	35.29	21.93	1.07E-01	23.98	20.48	2.42E-01	2.29	25.24	9.28E-01
cg01558785	-14.89	6.86	3.00E-02	-32.16	14.56	2.72E-02	-19.71	14.97	1.88E-01	-6.47	12.08	5.92E-01	-6.20	13.87	6.55E-01
cg13880467	-12.53	5.77	3.00E-02	-12.12	17.79	4.96E-01	-10.63	27.35	6.98E-01	-11.82	6.54	7.06E-02	-22.26	21.81	3.07E-01
cg06673273	-9.84	4.54	3.00E-02	-8.42	6.52	1.97E-01	-4.06	10.58	7.01E-01	-22.03	20.38	2.80E-01	-13.91	8.54	1.03E-01
cg23184252	-24.32	11.21	3.00E-02	-10.18	14.94	4.96E-01	-24.24	43.61	5.78E-01	-48.18	29.16	9.85E-02	-44.10	23.68	6.25E-02
cg12941312	12.19	5.62	3.00E-02	2.90	13.56	8.31E-01	3.57	16.63	8.30E-01	15.03	7.01	3.20E-02	22.55	20.87	2.80E-01

cg26831566	14.63	6.74	3.00E-02	-6.73	14.96	6.53E-01	6.54	25.72	7.99E-01	22.71	8.79	9.75E-03	15.62	18.07	3.87E-01
cg02328545	-26.27	12.11	3.00E-02	-20.66	19.35	2.86E-01	-0.50	80.08	9.95E-01	-49.96	25.01	4.57E-02	-18.39	20.43	3.68E-01
cg15693299	-7.71	3.55	3.00E-02	-6.47	5.23	2.16E-01	-13.30	8.68	1.26E-01	-1.94	8.39	8.18E-01	-11.24	8.14	1.67E-01
cg00057240	-6.63	3.05	3.00E-02	-9.93	4.70	3.48E-02	-4.78	7.15	5.04E-01	-5.07	6.89	4.62E-01	-2.87	6.85	6.75E-01
cg14575656	-10.02	4.62	3.00E-02	-12.21	6.41	5.67E-02	-7.45	11.89	5.31E-01	-6.59	12.51	5.99E-01	-8.56	10.50	4.15E-01
cg02753203	12.12	5.59	3.00E-02	13.52	14.93	3.65E-01	15.76	13.72	2.51E-01	10.07	7.11	1.57E-01	18.25	20.18	3.66E-01
cg04845185	12.82	5.91	3.00E-02	29.12	16.43	7.63E-02	17.03	15.50	2.72E-01	9.18	7.35	2.12E-01	8.17	21.15	6.99E-01
cg15906733	-7.26	3.35	3.00E-02	-11.62	4.57	1.10E-02	-1.95	7.64	7.98E-01	-10.53	10.87	3.33E-01	1.91	7.93	8.09E-01
cg05159279	15.16	6.99	3.00E-02	-0.35	18.62	9.85E-01	26.16	22.86	2.52E-01	14.45	8.33	8.28E-02	41.57	27.94	1.37E-01
cg22528556	18.50	8.53	3.00E-02	16.61	12.17	1.72E-01	36.60	15.86	2.10E-02	-7.20	28.01	7.97E-01	3.40	23.91	8.87E-01
cg22226568	20.39	9.40	3.01E-02	28.99	21.37	1.75E-01	8.88	24.65	7.19E-01	14.53	12.75	2.54E-01	47.46	27.38	8.30E-02
cg25449924	15.02	6.93	3.01E-02	14.31	11.22	2.02E-01	21.09	12.77	9.85E-02	-1.07	15.88	9.46E-01	26.50	18.86	1.60E-01
cg06543087	-23.37	10.77	3.01E-02	-5.54	17.70	7.54E-01	-58.52	192.00	7.61E-01	-37.77	15.01	1.18E-02	-15.01	32.30	6.42E-01
cg21898299	-12.31	5.67	3.01E-02	-20.57	7.87	8.99E-03	-10.99	13.65	4.21E-01	-8.49	18.96	6.54E-01	3.96	11.91	7.40E-01
cg25345520	-11.07	5.10	3.01E-02	-11.31	6.73	9.28E-02	-15.33	13.44	2.54E-01	-3.83	18.07	8.32E-01	-10.20	11.38	3.70E-01
cg11652496	22.83	10.52	3.01E-02	11.39	14.06	4.18E-01	55.54	20.95	8.01E-03	9.98	28.84	7.29E-01	19.37	23.14	4.03E-01
cg14700820	26.10	12.03	3.01E-02	4.60	38.43	9.05E-01	-71.22	170.10	6.75E-01	27.72	13.15	3.51E-02	46.74	49.08	3.41E-01
cg24303806	-14.73	6.79	3.01E-02	-23.35	15.30	1.27E-01	8.19	29.49	7.81E-01	-14.75	8.82	9.44E-02	-11.60	17.16	4.99E-01
cg03489427	-9.17	4.23	3.01E-02	-7.82	10.13	4.40E-01	-6.02	18.70	7.48E-01	-7.92	5.19	1.27E-01	-20.19	12.68	1.11E-01
cg05136452	19.48	8.98	3.01E-02	31.82	22.37	1.55E-01	86.00	79.25	2.78E-01	17.46	10.48	9.56E-02	4.54	29.76	8.79E-01
cg27492860	24.29	11.20	3.01E-02	21.32	13.02	1.02E-01	-31.55	55.48	5.70E-01	75.04	72.47	3.00E-01	40.89	25.29	1.06E-01
cg00941010	-13.63	6.28	3.01E-02	-15.42	13.24	2.44E-01	-9.16	17.18	5.94E-01	-12.97	8.64	1.33E-01	-18.53	18.82	3.25E-01
cg12785983	14.79	6.82	3.01E-02	13.52	11.59	2.43E-01	-0.87	19.80	9.65E-01	23.89	10.74	2.61E-02	4.38	18.80	8.16E-01
cg16269532	-16.10	7.42	3.01E-02	-23.92	17.95	1.83E-01	-51.97	35.70	1.45E-01	-13.03	8.86	1.41E-01	-7.34	25.64	7.75E-01
cg21968169	22.49	10.37	3.01E-02	26.42	12.94	4.13E-02	17.79	33.45	5.95E-01	-57.99	66.09	3.80E-01	22.11	21.26	2.99E-01
cg24409649	-30.51	14.07	3.01E-02	-38.67	47.67	4.17E-01	-50.81	201.64	8.01E-01	-30.94	15.55	4.65E-02	-17.46	47.10	7.11E-01
cg26932614	-11.64	5.37	3.01E-02	-16.24	7.76	3.64E-02	-13.62	11.00	2.15E-01	17.04	19.60	3.85E-01	-9.14	11.76	4.37E-01
cg15676015	27.15	12.52	3.01E-02	49.81	32.60	1.27E-01	-126.47	148.19	3.93E-01	25.70	14.25	7.12E-02	11.78	46.31	7.99E-01
cg05976325	24.72	11.40	3.01E-02	44.99	22.82	4.87E-02	49.28	42.56	2.47E-01	9.79	10.72	3.61E-01	51.36	31.75	1.06E-01
cg00650006	32.24	14.87	3.01E-02	32.56	19.36	9.26E-02	52.50	47.84	2.72E-01	89.49	59.33	1.32E-01	9.39	29.65	7.51E-01
cg03575292	15.59	7.19	3.01E-02	14.17	11.59	2.21E-01	9.67	13.98	4.89E-01	19.54	16.11	2.25E-01	24.35	18.47	1.88E-01
cg11313468	4.64	2.14	3.01E-02	3.59	2.70	1.84E-01	12.23	5.24	1.96E-02	4.44	6.90	5.20E-01	-0.64	6.39	9.20E-01
cg00397871	-6.36	2.93	3.01E-02	-7.03	4.81	1.44E-01	4.07	7.21	5.73E-01	-9.06	4.89	6.41E-02	-11.33	9.17	2.17E-01
cg02068989	28.89	13.32	3.01E-02	31.02	30.09	3.03E-01	-10.00	41.06	8.07E-01	32.39	17.14	5.88E-02	45.34	43.30	2.95E-01
cg09614932	-7.92	3.65	3.01E-02	-12.56	7.11	7.71E-02	0.81	10.15	9.36E-01	-9.49	5.28	7.21E-02	-1.26	10.23	9.02E-01

cg01358740	38.93	17.95	3.01E-02	44.53	52.12	3.93E-01	-150.63	277.72	5.88E-01	34.96	20.25	8.42E-02	74.55	59.47	2.10E-01
cg24114708	-10.93	5.04	3.01E-02	0.30	13.72	9.82E-01	-8.74	23.32	7.08E-01	-13.07	5.93	2.76E-02	-11.65	16.20	4.72E-01
cg26296364	21.01	9.69	3.01E-02	44.81	17.46	1.03E-02	14.29	31.69	6.52E-01	20.75	16.73	2.15E-01	2.31	16.23	8.87E-01
cg17995967	30.17	13.91	3.01E-02	40.31	22.42	7.22E-02	28.70	47.21	5.43E-01	9.52	13.60	4.84E-01	70.01	29.12	1.62E-02
cg11045198	-11.23	5.18	3.01E-02	-21.28	13.33	1.10E-01	-13.45	18.94	4.78E-01	-8.49	6.29	1.77E-01	-12.96	16.61	4.35E-01
cg00864530	-8.15	3.76	3.01E-02	-3.90	8.00	6.26E-01	-1.65	13.12	9.00E-01	-9.50	4.93	5.40E-02	-14.22	11.12	2.01E-01
cg06448961	12.56	5.80	3.01E-02	5.34	14.49	7.13E-01	34.55	54.61	5.27E-01	13.04	6.66	5.03E-02	20.20	21.60	3.50E-01
cg06323049	-9.38	4.33	3.01E-02	-20.73	9.87	3.56E-02	-7.50	16.08	6.41E-01	-7.85	5.35	1.42E-01	3.59	15.26	8.14E-01
cg10754002	-12.13	5.59	3.02E-02	-20.11	8.21	1.43E-02	-1.03	13.48	9.39E-01	-1.91	17.14	9.11E-01	-9.37	11.05	3.96E-01
cg00196407	11.67	5.38	3.02E-02	14.23	7.40	5.45E-02	27.66	13.51	4.06E-02	11.11	10.20	2.76E-01	-3.82	11.07	7.30E-01
cg02603192	-56.09	25.87	3.02E-02	-93.18	61.00	1.27E-01	-158.96	316.77	6.16E-01	-39.86	31.06	1.99E-01	-88.58	74.71	2.36E-01
cg01062930	20.96	9.67	3.02E-02	2.75	19.48	8.88E-01	0.57	26.77	9.83E-01	33.77	15.26	2.69E-02	29.97	20.51	1.44E-01
cg04807025	14.77	6.81	3.02E-02	-13.39	21.86	5.40E-01	32.47	39.59	4.12E-01	17.42	7.59	2.18E-02	15.92	26.11	5.42E-01
cg17786516	-7.63	3.52	3.02E-02	-16.06	6.08	8.23E-03	2.60	7.33	7.22E-01	-6.52	4.38	1.36E-01	-9.05	7.86	2.49E-01
cg22417420	-42.92	19.80	3.02E-02	-60.19	68.60	3.80E-01	-167.28	118.56	1.58E-01	-40.34	21.79	6.41E-02	1.19	79.02	9.88E-01
cg00162046	21.86	10.08	3.02E-02	18.21	16.77	2.78E-01	19.18	20.10	3.40E-01	24.04	21.15	2.56E-01	31.23	25.23	2.16E-01
cg13726218	13.89	6.41	3.02E-02	7.70	9.21	4.03E-01	20.63	16.82	2.20E-01	-16.04	32.71	6.24E-01	23.40	11.11	3.51E-02
cg19940065	21.03	9.70	3.02E-02	-3.90	18.44	8.32E-01	18.46	47.54	6.98E-01	31.00	12.99	1.70E-02	32.75	27.57	2.35E-01
cg25349990	6.57	3.03	3.02E-02	6.03	3.96	1.28E-01	9.46	6.76	1.62E-01	3.04	12.49	8.07E-01	6.22	7.73	4.21E-01
cg02534450	8.77	4.04	3.02E-02	0.83	7.91	9.17E-01	12.92	10.64	2.25E-01	7.37	6.10	2.27E-01	22.29	10.29	3.02E-02
cg12070987	7.63	3.52	3.02E-02	8.00	4.11	5.16E-02	7.49	9.61	4.36E-01	21.33	15.12	1.58E-01	-5.22	12.67	6.80E-01
cg20704450	-4.43	2.04	3.02E-02	-3.88	2.83	1.70E-01	-1.54	5.10	7.63E-01	-9.24	5.93	1.20E-01	-5.35	4.59	2.44E-01
cg06760467	-12.15	5.60	3.02E-02	-13.10	9.19	1.54E-01	-10.89	12.38	3.79E-01	-12.24	14.92	4.12E-01	-11.77	10.56	2.65E-01
cg08471972	-12.86	5.93	3.02E-02	-25.96	16.42	1.14E-01	-0.50	24.78	9.84E-01	-12.60	6.86	6.63E-02	-0.28	23.35	9.90E-01
cg03338754	9.52	4.39	3.02E-02	10.12	6.61	1.26E-01	16.29	10.93	1.36E-01	24.53	13.92	7.80E-02	-0.06	8.04	9.94E-01
cg23655039	10.82	4.99	3.02E-02	14.04	7.11	4.82E-02	14.26	12.51	2.54E-01	13.22	15.22	3.85E-01	0.83	10.20	9.35E-01
cg10109111	7.59	3.50	3.02E-02	1.94	4.11	6.36E-01	12.10	5.18	1.95E-02	18.10	16.74	2.80E-01	14.89	10.45	1.54E-01
cg25253677	18.03	8.32	3.02E-02	62.63	30.68	4.12E-02	-16.91	85.41	8.43E-01	12.66	9.28	1.72E-01	30.07	24.72	2.24E-01
cg16579770	-15.29	7.06	3.02E-02	-8.27	11.95	4.89E-01	6.36	27.06	8.14E-01	-12.13	7.80	1.20E-01	-38.37	14.07	6.39E-03
cg04850211	-4.13	1.91	3.02E-02	-4.36	2.58	9.15E-02	-5.96	4.59	1.94E-01	-3.07	5.98	6.08E-01	-2.31	4.48	6.07E-01
cg11065154	7.18	3.31	3.02E-02	5.72	4.85	2.38E-01	1.36	7.60	8.58E-01	20.40	10.15	4.45E-02	8.74	6.79	1.98E-01
cg14769589	18.31	8.45	3.02E-02	11.75	11.41	3.03E-01	18.52	22.75	4.16E-01	6.41	30.78	8.35E-01	37.11	17.33	3.23E-02
cg04303509	6.30	2.91	3.02E-02	5.82	4.29	1.75E-01	9.07	11.06	4.12E-01	5.33	5.25	3.10E-01	8.33	7.18	2.46E-01
cg02600625	-12.99	6.00	3.02E-02	-24.44	9.59	1.08E-02	-18.69	13.64	1.71E-01	-1.98	14.12	8.89E-01	-2.33	10.51	8.24E-01
cg17222941	-26.21	12.10	3.02E-02	-27.20	34.62	4.32E-01	55.40	158.78	7.27E-01	-19.91	13.83	1.50E-01	-74.74	37.02	4.35E-02

cg14419581	-22.36	10.32	3.02E-02	-29.98	12.40	1.56E-02	4.57	89.02	9.59E-01	-16.44	23.98	4.93E-01	12.80	31.29	6.82E-01
cg16235962	-8.78	4.05	3.02E-02	-6.12	5.92	3.02E-01	-13.52	9.93	1.74E-01	-11.10	10.58	2.94E-01	-9.32	8.66	2.82E-01
cg01006102	26.10	12.05	3.03E-02	57.28	18.85	2.37E-03	17.61	20.33	3.86E-01	17.34	18.74	3.55E-01	0.26	27.12	9.92E-01
cg06851207	-3.70	1.71	3.03E-02	-1.51	2.35	5.20E-01	-6.09	3.72	1.02E-01	-2.52	7.61	7.40E-01	-7.11	3.73	5.64E-02
cg02250431	8.67	4.00	3.03E-02	2.42	8.33	7.72E-01	17.78	13.00	1.71E-01	9.80	5.19	5.90E-02	7.50	14.12	5.96E-01
cg18430314	-35.88	16.56	3.03E-02	-39.38	22.27	7.70E-02	-83.53	47.41	7.81E-02	-26.72	41.43	5.19E-01	2.18	40.75	9.57E-01
cg01538166	13.20	6.09	3.03E-02	12.77	7.89	1.06E-01	29.75	11.51	9.72E-03	9.36	12.87	4.67E-01	-3.83	13.76	7.81E-01
cg26530485	10.27	4.74	3.03E-02	15.17	6.48	1.93E-02	8.30	13.43	5.36E-01	3.15	16.30	8.47E-01	3.34	9.37	7.21E-01
cg04702527	9.63	4.44	3.03E-02	7.62	6.88	2.68E-01	8.91	9.66	3.56E-01	17.92	9.70	6.46E-02	4.98	11.08	6.53E-01
cg06178299	-12.39	5.72	3.03E-02	-15.28	7.65	4.58E-02	-8.16	17.30	6.37E-01	-3.21	25.73	9.01E-01	-9.91	10.76	3.57E-01
cg27594176	22.73	10.49	3.03E-02	4.36	29.09	8.81E-01	-56.84	148.72	7.02E-01	28.00	11.77	1.74E-02	2.98	39.32	9.40E-01
cg08891605	9.43	4.35	3.03E-02	8.32	6.04	1.68E-01	13.58	8.81	1.23E-01	12.42	16.51	4.52E-01	5.56	10.64	6.01E-01
cg00020405	10.52	4.85	3.03E-02	10.16	6.00	9.04E-02	27.09	16.31	9.67E-02	8.32	16.80	6.20E-01	4.46	11.64	7.01E-01
cg11908155	-11.52	5.32	3.03E-02	-10.90	8.25	1.87E-01	-2.60	11.99	8.29E-01	-17.92	11.16	1.08E-01	-14.94	13.21	2.58E-01
cg15237047	-50.38	23.26	3.03E-02	-52.31	28.51	6.65E-02	-62.02	82.08	4.50E-01	66.75	81.57	4.13E-01	-92.61	55.93	9.77E-02
cg02957270	8.79	4.06	3.03E-02	9.79	6.75	1.47E-01	4.86	9.29	6.00E-01	16.29	8.02	4.24E-02	0.86	9.23	9.26E-01
cg24513522	-11.04	5.10	3.03E-02	-18.58	7.54	1.38E-02	0.80	10.52	9.40E-01	-14.37	17.91	4.23E-01	-6.95	10.68	5.15E-01
cg24338483	-40.27	18.59	3.03E-02	-48.17	24.51	4.94E-02	-32.32	87.08	7.10E-01	33.46	63.55	5.99E-01	-47.49	34.27	1.66E-01
cg15907819	-10.37	4.79	3.03E-02	-4.80	9.65	6.19E-01	-10.93	17.99	5.44E-01	-15.10	6.31	1.67E-02	2.51	14.57	8.63E-01
cg16325174	-10.18	4.70	3.03E-02	-21.46	10.53	4.16E-02	1.22	12.28	9.21E-01	-7.14	6.49	2.71E-01	-18.02	13.03	1.67E-01
cg26396370	4.21	1.94	3.03E-02	3.71	2.85	1.94E-01	6.59	4.40	1.34E-01	3.85	3.73	3.02E-01	2.29	7.42	7.58E-01
cg07549208	-19.77	9.12	3.03E-02	-13.96	11.11	2.09E-01	0.72	16.53	9.65E-01	-42.00	22.30	5.97E-02	-38.60	17.68	2.90E-02
cg01432087	-13.76	6.35	3.03E-02	-19.93	13.13	1.29E-01	-27.40	27.95	3.27E-01	-12.67	8.13	1.19E-01	0.48	19.66	9.81E-01
cg13528513	-5.90	2.73	3.03E-02	-10.22	5.03	4.23E-02	-5.42	7.30	4.58E-01	-4.12	4.26	3.34E-01	-2.95	6.84	6.66E-01
cg04710402	23.33	10.77	3.03E-02	71.50	30.86	2.05E-02	14.10	63.80	8.25E-01	23.34	9.35	1.26E-02	2.10	19.69	9.15E-01
cg18123048	44.48	20.53	3.03E-02	66.84	61.65	2.78E-01	62.84	214.78	7.70E-01	46.96	23.03	4.15E-02	-9.73	70.37	8.90E-01
cg17588400	-13.70	6.33	3.03E-02	0.23	15.72	9.88E-01	-10.75	20.39	5.98E-01	-18.52	7.69	1.61E-02	-2.75	24.78	9.12E-01
cg17658854	10.39	4.79	3.03E-02	15.59	13.64	2.53E-01	25.84	20.96	2.18E-01	7.22	5.43	1.83E-01	33.68	22.94	1.42E-01
cg13123826	-14.30	6.60	3.03E-02	-13.32	15.04	3.76E-01	-9.54	21.90	6.63E-01	-17.51	8.59	4.15E-02	-4.21	18.54	8.20E-01
cg02776448	15.22	7.03	3.03E-02	0.75	8.11	9.26E-01	21.13	11.80	7.33E-02	27.15	17.08	1.12E-01	24.94	11.70	3.30E-02
cg27177296	-8.19	3.78	3.03E-02	-16.61	8.84	6.04E-02	-7.09	18.67	7.04E-01	-6.90	4.65	1.38E-01	-2.67	11.13	8.11E-01
cg17216080	9.76	4.50	3.03E-02	3.88	7.35	5.98E-01	17.56	9.42	6.24E-02	19.41	15.93	2.23E-01	8.66	8.02	2.80E-01
cg16576575	9.11	4.21	3.03E-02	6.56	6.53	3.15E-01	10.60	8.44	2.09E-01	27.33	13.35	4.07E-02	4.39	8.62	6.10E-01
cg15786205	13.17	6.08	3.03E-02	22.04	9.30	1.78E-02	6.48	16.83	7.00E-01	24.42	16.30	1.34E-01	0.31	10.32	9.76E-01
cg23295629	-9.12	4.21	3.03E-02	-9.42	5.53	8.87E-02	-7.02	10.71	5.12E-01	3.38	14.49	8.15E-01	-15.81	9.90	1.10E-01

cg18200827	39.29	18.14	3.03E-02	47.28	21.25	2.60E-02	35.78	91.10	6.94E-01	31.66	61.82	6.09E-01	4.65	47.60	9.22E-01
cg23694261	-25.66	11.85	3.03E-02	-47.06	25.82	6.84E-02	-4.83	166.54	9.77E-01	-17.70	13.97	2.05E-01	-46.23	46.60	3.21E-01
cg18431951	9.36	4.32	3.03E-02	7.78	5.80	1.80E-01	14.55	9.22	1.15E-01	12.29	15.42	4.25E-01	6.02	11.31	5.95E-01
cg18477666	-13.66	6.31	3.03E-02	-15.73	9.98	1.15E-01	-12.04	14.59	4.09E-01	-21.56	14.98	1.50E-01	-5.51	12.96	6.70E-01
cg17992056	11.99	5.53	3.03E-02	10.60	7.31	1.47E-01	17.42	15.26	2.54E-01	41.80	34.63	2.27E-01	9.46	10.66	3.75E-01
cg05956058	-11.23	5.19	3.03E-02	-9.67	13.58	4.76E-01	17.29	23.49	4.62E-01	-11.71	6.12	5.56E-02	-25.96	17.63	1.41E-01
cg07554408	10.49	4.84	3.03E-02	1.29	10.45	9.02E-01	18.44	15.11	2.22E-01	14.14	6.33	2.55E-02	0.40	15.56	9.80E-01
cg24001556	-23.92	11.05	3.03E-02	-23.14	13.99	9.82E-02	-17.89	25.77	4.88E-01	-18.41	45.73	6.87E-01	-38.08	30.06	2.05E-01
cg13232118	22.26	10.28	3.03E-02	22.38	15.73	1.55E-01	23.95	26.03	3.58E-01	13.86	27.95	6.20E-01	25.18	19.36	1.93E-01
cg13396068	-11.48	5.30	3.04E-02	-23.26	12.75	6.82E-02	-16.07	16.11	3.19E-01	-7.90	6.79	2.45E-01	-8.28	15.98	6.04E-01
cg11523191	-28.69	13.25	3.04E-02	-43.74	28.65	1.27E-01	20.00	60.69	7.42E-01	-27.29	18.62	1.43E-01	-27.88	27.49	3.10E-01
cg14821245	-8.40	3.88	3.04E-02	-9.78	6.91	1.57E-01	-12.64	10.27	2.18E-01	-6.33	6.25	3.11E-01	-6.87	9.81	4.84E-01
cg25643595	-14.64	6.76	3.04E-02	-22.28	18.31	2.24E-01	-90.43	78.43	2.49E-01	-12.45	7.67	1.05E-01	-15.95	24.16	5.09E-01
cg15780870	-61.02	28.18	3.04E-02	-78.21	36.52	3.22E-02	-23.78	202.23	9.06E-01	32.89	82.91	6.92E-01	-65.98	54.28	2.24E-01
cg20948740	10.28	4.75	3.04E-02	13.88	7.42	6.16E-02	21.13	12.34	8.69E-02	5.06	9.70	6.02E-01	1.32	10.51	9.00E-01
cg00227781	21.27	9.83	3.04E-02	2.14	34.91	9.51E-01	97.91	102.54	3.40E-01	22.57	10.84	3.73E-02	18.45	32.79	5.74E-01
cg03945895	16.50	7.62	3.04E-02	27.27	21.54	2.06E-01	26.09	31.28	4.04E-01	13.49	8.85	1.28E-01	20.82	28.03	4.58E-01
cg01653132	14.30	6.61	3.04E-02	28.43	20.16	1.58E-01	25.50	22.73	2.62E-01	11.05	7.67	1.50E-01	13.62	25.72	5.97E-01
cg26222247	16.77	7.74	3.04E-02	16.74	12.21	1.70E-01	28.18	23.53	2.31E-01	17.90	17.12	2.96E-01	11.64	14.51	4.23E-01
cg14564616	20.85	9.63	3.04E-02	43.46	40.81	2.87E-01	-153.53	178.96	3.91E-01	21.33	10.27	3.77E-02	1.67	38.85	9.66E-01
cg25836232	6.36	2.94	3.04E-02	2.50	4.23	5.55E-01	10.19	6.94	1.42E-01	10.59	6.80	1.19E-01	8.90	7.52	2.37E-01
cg26312191	-7.66	3.54	3.04E-02	-7.35	4.59	1.09E-01	-10.56	8.38	2.07E-01	-5.49	11.61	6.36E-01	-6.64	9.61	4.89E-01
cg21616142	-18.56	8.57	3.04E-02	-30.96	14.33	3.08E-02	-6.22	19.04	7.44E-01	-13.91	18.09	4.42E-01	-14.40	18.46	4.35E-01
cg18809377	19.08	8.81	3.04E-02	28.40	30.85	3.57E-01	104.25	141.68	4.62E-01	16.41	9.56	8.61E-02	37.22	34.57	2.82E-01
cg00348222	-12.29	5.68	3.04E-02	-19.22	9.40	4.09E-02	-7.56	11.27	5.02E-01	-8.19	13.49	5.44E-01	-9.36	12.57	4.56E-01
cg16279544	19.05	8.80	3.04E-02	28.13	21.21	1.85E-01	25.99	36.45	4.76E-01	10.88	10.71	3.10E-01	56.95	28.73	4.74E-02
cg01218206	-9.14	4.22	3.04E-02	-7.34	4.75	1.22E-01	-15.03	8.43	7.47E-02	8.63	11.92	4.69E-01	-17.09	8.41	4.21E-02
cg26003360	-14.26	6.59	3.04E-02	-20.94	15.04	1.64E-01	9.14	30.31	7.63E-01	-13.25	8.01	9.81E-02	-20.08	22.48	3.72E-01
cg09719124	17.23	7.96	3.04E-02	9.38	15.37	5.42E-01	14.89	33.93	6.61E-01	21.37	10.59	4.36E-02	16.34	23.82	4.93E-01
cg16061528	-16.21	7.49	3.04E-02	-26.35	12.67	3.76E-02	-1.63	15.06	9.14E-01	1.34	17.74	9.40E-01	-27.30	13.52	4.34E-02
cg04340918	-8.41	3.88	3.04E-02	-11.35	8.92	2.03E-01	1.28	13.28	9.23E-01	-7.00	5.09	1.69E-01	-16.15	10.33	1.18E-01
cg23311258	7.78	3.59	3.04E-02	11.14	5.48	4.21E-02	16.65	9.26	7.21E-02	3.27	6.98	6.39E-01	-2.50	9.15	7.85E-01
cg03505501	10.01	4.62	3.04E-02	11.13	6.94	1.09E-01	14.94	14.26	2.95E-01	17.89	10.83	9.85E-02	0.87	8.93	9.23E-01
cg18591727	-10.16	4.69	3.04E-02	-8.34	10.90	4.44E-01	-30.32	13.59	2.57E-02	-6.64	6.14	2.79E-01	-10.07	14.09	4.75E-01
cg04216051	-18.42	8.51	3.04E-02	-13.32	13.45	3.22E-01	8.11	21.96	7.12E-01	-30.91	15.52	4.65E-02	-33.63	22.03	1.27E-01

cg07007080	27.87	12.87	3.04E-02	33.47	29.59	2.58E-01	-77.38	63.69	2.24E-01	28.13	11.72	1.64E-02	57.80	38.97	1.38E-01
cg01159980	12.74	5.88	3.04E-02	6.93	9.03	4.43E-01	25.83	11.66	2.68E-02	15.38	15.94	3.35E-01	6.10	13.68	6.56E-01
cg13434396	10.01	4.62	3.04E-02	12.22	6.91	7.71E-02	14.58	12.05	2.26E-01	2.75	10.15	7.86E-01	9.21	10.40	3.75E-01
cg11902728	12.23	5.65	3.04E-02	10.18	6.29	1.05E-01	19.10	8.99	3.37E-02	27.23	14.10	5.35E-02	-7.28	12.98	5.75E-01
cg18127410	-16.59	7.66	3.04E-02	-9.98	14.09	4.79E-01	-40.16	54.76	4.63E-01	-20.08	9.79	4.02E-02	-7.53	28.67	7.93E-01
cg25887236	18.62	8.60	3.04E-02	5.81	14.74	6.94E-01	-8.53	36.57	8.16E-01	26.92	12.66	3.35E-02	32.82	22.75	1.49E-01
cg26407930	9.50	4.39	3.04E-02	5.51	10.34	5.94E-01	8.70	13.76	5.27E-01	10.78	5.48	4.93E-02	9.30	15.88	5.58E-01
cg15434211	11.05	5.10	3.04E-02	8.50	6.91	2.19E-01	16.23	11.68	1.65E-01	8.17	17.31	6.37E-01	14.69	12.11	2.25E-01
cg03110368	15.83	7.31	3.04E-02	19.37	9.71	4.61E-02	15.19	29.80	6.10E-01	-20.10	33.41	5.47E-01	15.06	12.83	2.40E-01
cg23256951	7.05	3.26	3.04E-02	3.53	4.75	4.57E-01	14.75	6.47	2.26E-02	6.61	9.70	4.95E-01	5.49	8.01	4.93E-01
cg17599586	-38.97	18.01	3.04E-02	-32.76	23.49	1.63E-01	-49.74	70.35	4.80E-01	-15.26	55.25	7.82E-01	-61.66	36.71	9.30E-02
cg25911482	15.53	7.17	3.04E-02	22.59	15.06	1.34E-01	1.61	14.24	9.10E-01	19.22	11.49	9.44E-02	19.31	19.95	3.33E-01
cg21534423	14.32	6.62	3.04E-02	8.40	18.56	6.51E-01	50.93	35.50	1.51E-01	15.19	8.02	5.81E-02	7.24	16.73	6.65E-01
cg18557185	-7.65	3.53	3.04E-02	-4.70	5.25	3.71E-01	-8.52	8.60	3.22E-01	4.59	12.63	7.16E-01	-14.79	6.45	2.18E-02
cg17210004	8.01	3.70	3.05E-02	5.68	5.53	3.04E-01	3.12	7.76	6.88E-01	14.52	11.86	2.21E-01	14.65	7.74	5.85E-02
cg00601368	-8.64	3.99	3.05E-02	-9.46	5.24	7.10E-02	-13.85	9.40	1.41E-01	-4.27	14.73	7.72E-01	-2.02	9.82	8.37E-01
cg05349077	-62.79	29.01	3.05E-02	-49.83	38.93	2.01E-01	17.64	126.53	8.89E-01	-96.53	60.68	1.12E-01	-85.51	71.77	2.33E-01
cg20091384	-9.47	4.38	3.05E-02	-8.11	6.15	1.87E-01	-5.93	9.58	5.36E-01	-28.46	13.62	3.66E-02	-6.54	10.29	5.25E-01
cg01232748	7.87	3.64	3.05E-02	6.27	3.77	9.64E-02	17.99	7.02	1.04E-02	10.59	8.43	2.09E-01	-2.32	7.82	7.67E-01
cg15581944	-9.55	4.41	3.05E-02	0.13	13.61	9.92E-01	-9.54	10.21	3.50E-01	-10.88	5.53	4.92E-02	-12.04	16.59	4.68E-01
cg24647108	15.65	7.23	3.05E-02	25.32	15.86	1.10E-01	4.12	18.58	8.24E-01	17.60	9.72	7.02E-02	0.24	24.45	9.92E-01
cg07547403	-14.51	6.70	3.05E-02	1.30	19.65	9.47E-01	-18.25	25.39	4.72E-01	-16.51	7.66	3.11E-02	-15.44	30.67	6.15E-01
cg22016716	-16.25	7.51	3.05E-02	-24.74	13.43	6.55E-02	-3.04	23.07	8.95E-01	-16.77	11.16	1.33E-01	-4.74	20.86	8.20E-01
cg18759693	-58.15	26.87	3.05E-02	-54.77	24.41	2.48E-02	-73.60	155.97	6.37E-01	61.75	86.72	4.76E-01	-128.35	59.36	3.06E-02
cg12438044	9.17	4.24	3.05E-02	4.28	7.90	5.88E-01	5.73	7.90	4.68E-01	16.08	7.91	4.21E-02	12.15	11.38	2.86E-01
cg01297721	10.72	4.96	3.05E-02	2.83	8.49	7.39E-01	19.92	12.00	9.70E-02	5.76	10.27	5.74E-01	19.62	9.80	4.52E-02
cg15832405	24.03	11.11	3.05E-02	32.86	14.26	2.12E-02	-8.55	43.62	8.45E-01	-20.76	66.62	7.55E-01	17.37	20.25	3.91E-01
cg09175289	15.32	7.08	3.05E-02	14.84	10.16	1.44E-01	15.53	15.45	3.15E-01	0.55	21.52	9.80E-01	24.46	15.99	1.26E-01
cg12667732	-13.51	6.24	3.05E-02	-16.57	12.44	1.83E-01	-21.40	16.94	2.06E-01	-13.92	9.57	1.46E-01	-2.71	14.44	8.51E-01
cg14081270	7.25	3.35	3.05E-02	6.72	4.50	1.35E-01	12.50	8.37	1.35E-01	13.53	9.57	1.57E-01	-0.89	8.34	9.15E-01
cg13885451	36.40	16.82	3.05E-02	32.43	20.84	1.20E-01	84.44	54.22	1.19E-01	92.59	69.75	1.84E-01	9.05	38.22	8.13E-01
cg17007896	6.94	3.21	3.05E-02	6.75	4.12	1.01E-01	18.46	9.72	5.74E-02	5.55	8.62	5.20E-01	0.50	8.34	9.52E-01
cg27359566	15.56	7.19	3.05E-02	10.23	23.62	6.65E-01	-30.99	110.44	7.79E-01	20.02	7.91	1.14E-02	-23.51	25.96	3.65E-01
cg04826772	42.29	19.55	3.05E-02	59.25	23.86	1.30E-02	-25.09	77.24	7.45E-01	23.01	58.92	6.96E-01	10.38	49.64	8.34E-01
cg21604856	-10.71	4.95	3.05E-02	-10.81	7.93	1.73E-01	-6.51	10.59	5.39E-01	-17.23	11.22	1.25E-01	-8.74	11.14	4.33E-01

cg22846388	-14.86	6.87	3.05E-02	-4.60	19.88	8.17E-01	17.31	31.61	5.84E-01	-19.08	7.87	1.53E-02	-8.26	25.57	7.47E-01
cg14543152	12.76	5.90	3.05E-02	17.49	10.94	1.10E-01	6.45	14.01	6.45E-01	15.63	8.92	7.98E-02	-3.06	19.09	8.73E-01
cg03918306	-33.44	15.46	3.05E-02	-12.12	27.94	6.64E-01	-388.85	234.16	9.68E-02	-41.24	19.28	3.24E-02	-34.09	51.33	5.07E-01
cg15470750	-44.75	20.69	3.05E-02	-50.23	24.26	3.84E-02	-80.02	133.14	5.48E-01	30.86	67.96	6.50E-01	-58.69	52.39	2.63E-01
cg10714639	-10.88	5.03	3.05E-02	-11.73	8.23	1.54E-01	-9.06	10.77	4.00E-01	-13.05	9.43	1.66E-01	-6.54	14.32	6.48E-01
cg09823095	21.65	10.01	3.05E-02	32.80	35.45	3.55E-01	-128.87	152.77	3.99E-01	21.22	10.75	4.84E-02	24.16	44.82	5.90E-01
cg03225002	32.74	15.14	3.05E-02	42.82	18.46	2.04E-02	-67.16	73.87	3.63E-01	8.45	47.51	8.59E-01	32.13	35.22	3.62E-01
cg17960629	-9.33	4.31	3.05E-02	-11.05	6.22	7.54E-02	-15.42	10.54	1.43E-01	-1.31	10.81	9.03E-01	-6.34	9.82	5.18E-01
cg24208375	23.42	10.83	3.05E-02	12.54	20.48	5.40E-01	-57.23	78.62	4.67E-01	35.76	13.98	1.05E-02	-4.30	33.91	8.99E-01
cg14326354	-11.57	5.35	3.05E-02	-19.78	8.14	1.51E-02	-6.15	12.13	6.12E-01	-3.51	16.77	8.34E-01	-5.42	10.25	5.97E-01
cg14561276	16.61	7.68	3.05E-02	29.33	19.27	1.28E-01	19.46	23.80	4.14E-01	13.97	9.39	1.37E-01	8.60	29.35	7.69E-01
cg26122963	-12.75	5.89	3.05E-02	-7.54	9.65	4.35E-01	-12.45	15.03	4.08E-01	-20.21	11.73	8.49E-02	-13.24	12.55	2.91E-01
cg26757711	13.39	6.19	3.05E-02	15.80	9.41	9.32E-02	22.89	17.93	2.02E-01	5.01	13.47	7.10E-01	11.67	12.69	3.58E-01
cg10400362	-30.28	14.00	3.05E-02	-37.64	21.44	7.91E-02	-14.44	35.96	6.88E-01	-9.78	35.13	7.81E-01	-39.85	27.28	1.44E-01
cg21515023	-41.86	19.35	3.05E-02	-16.18	27.49	5.56E-01	-21.30	64.08	7.40E-01	-78.80	42.36	6.29E-02	-75.54	42.77	7.74E-02
cg07908000	-17.83	8.24	3.05E-02	-25.22	16.62	1.29E-01	1.16	26.78	9.66E-01	-20.30	11.57	7.95E-02	-9.48	21.13	6.54E-01
cg17803175	11.48	5.31	3.05E-02	11.34	7.82	1.47E-01	15.95	14.89	2.84E-01	14.53	11.34	2.00E-01	5.41	12.08	6.54E-01
cg07068756	15.17	7.01	3.05E-02	44.75	23.45	5.63E-02	12.52	38.09	7.42E-01	13.89	8.52	1.03E-01	6.70	15.70	6.69E-01
cg05065948	6.86	3.17	3.05E-02	7.40	4.15	7.44E-02	12.41	8.46	1.42E-01	3.72	8.75	6.70E-01	2.10	8.37	8.02E-01
cg06013494	8.46	3.91	3.05E-02	7.93	5.41	1.42E-01	12.98	8.73	1.37E-01	16.35	14.58	2.62E-01	2.60	8.64	7.63E-01
cg14906638	17.37	8.03	3.05E-02	17.67	11.09	1.11E-01	-11.18	37.97	7.68E-01	16.79	29.01	5.63E-01	20.67	13.50	1.26E-01
cg09998861	7.82	3.62	3.05E-02	8.40	5.59	1.33E-01	11.68	8.13	1.51E-01	4.49	10.03	6.54E-01	5.56	7.17	4.38E-01
cg08659047	22.96	10.62	3.05E-02	19.84	13.17	1.32E-01	-3.99	30.70	8.97E-01	78.27	40.90	5.56E-02	32.33	26.29	2.19E-01
cg22212250	-7.30	3.38	3.05E-02	-13.57	6.24	2.95E-02	-15.01	11.25	1.82E-01	-1.35	4.76	7.77E-01	-9.39	8.27	2.56E-01
cg13527921	7.98	3.69	3.06E-02	12.04	5.27	2.24E-02	13.41	8.70	1.23E-01	7.02	13.56	6.05E-01	-1.91	6.86	7.81E-01
cg21141445	16.33	7.55	3.06E-02	8.40	19.71	6.70E-01	4.69	29.94	8.75E-01	15.95	9.04	7.76E-02	39.94	24.91	1.09E-01
cg06120235	7.71	3.56	3.06E-02	6.23	5.14	2.26E-01	14.75	6.99	3.48E-02	-6.27	13.78	6.49E-01	6.75	8.14	4.07E-01
cg27300887	23.11	10.68	3.06E-02	22.50	16.10	1.62E-01	19.43	20.76	3.49E-01	6.07	38.85	8.76E-01	34.65	22.83	1.29E-01
cg13856126	-15.62	7.22	3.06E-02	-12.94	11.81	2.73E-01	-16.23	14.77	2.72E-01	-12.41	19.28	5.20E-01	-20.94	14.56	1.50E-01
cg09501687	15.48	7.16	3.06E-02	22.77	8.95	1.10E-02	-0.48	22.30	9.83E-01	-17.18	36.51	6.38E-01	7.47	15.28	6.25E-01
cg22765178	-8.69	4.02	3.06E-02	-12.44	9.60	1.95E-01	-29.85	24.71	2.27E-01	-5.85	4.82	2.25E-01	-16.08	12.53	1.99E-01
cg22217449	-7.94	3.67	3.06E-02	-7.76	4.45	8.13E-02	-17.98	7.73	1.99E-02	7.57	12.93	5.58E-01	-4.29	7.68	5.76E-01
cg00928751	-12.14	5.61	3.06E-02	-21.08	11.91	7.66E-02	-13.87	16.19	3.92E-01	-10.85	7.60	1.54E-01	1.13	16.74	9.46E-01
cg09138267	-33.70	15.58	3.06E-02	-22.77	17.35	1.89E-01	28.81	49.48	5.60E-01	-94.31	56.14	9.30E-02	-50.77	20.64	1.39E-02
cg19300502	6.52	3.01	3.06E-02	3.26	4.11	4.28E-01	9.22	6.33	1.45E-01	1.46	11.20	8.96E-01	15.64	7.43	3.52E-02

cg00463011	22.98	10.62	3.06E-02	16.37	23.23	4.81E-01	62.25	45.80	1.74E-01	22.61	13.50	9.41E-02	18.72	30.93	5.45E-01
cg00435576	-9.23	4.27	3.06E-02	-22.69	11.93	5.72E-02	4.20	14.81	7.77E-01	-7.64	5.04	1.29E-01	-16.61	15.94	2.97E-01
cg07672688	7.12	3.29	3.06E-02	4.44	6.97	5.24E-01	4.30	10.82	6.91E-01	10.15	4.30	1.83E-02	-2.25	10.55	8.31E-01
cg00083790	8.05	3.72	3.06E-02	12.00	5.31	2.37E-02	5.68	7.14	4.27E-01	-2.83	16.34	8.62E-01	4.06	8.69	6.40E-01
cg16589260	5.77	2.67	3.06E-02	6.85	4.51	1.29E-01	2.85	5.28	5.89E-01	11.16	5.80	5.43E-02	1.54	6.24	8.05E-01
cg22944461	22.32	10.32	3.06E-02	-4.04	21.83	8.53E-01	-15.30	44.86	7.33E-01	33.29	13.16	1.14E-02	32.79	31.37	2.96E-01
cg13678070	-33.46	15.47	3.06E-02	-29.21	25.52	2.52E-01	-40.52	123.29	7.42E-01	-33.56	20.85	1.08E-01	-54.67	60.25	3.64E-01
cg13500072	11.63	5.38	3.06E-02	15.03	11.23	1.81E-01	17.01	14.05	2.26E-01	12.89	8.25	1.18E-01	1.03	12.05	9.32E-01
cg18017926	16.32	7.55	3.06E-02	22.52	11.08	4.22E-02	14.52	20.61	4.81E-01	36.14	24.91	1.47E-01	1.99	13.54	8.83E-01
cg27440002	17.22	7.97	3.06E-02	4.99	16.69	7.65E-01	33.52	34.69	3.34E-01	22.63	10.13	2.56E-02	3.33	24.98	8.94E-01
cg01621145	20.19	9.34	3.06E-02	33.69	22.81	1.40E-01	-2.09	38.03	9.56E-01	14.61	11.24	1.94E-01	56.11	32.68	8.60E-02
cg22114568	-11.39	5.27	3.06E-02	-17.44	11.39	1.26E-01	-19.44	23.64	4.11E-01	-10.95	6.63	9.85E-02	2.06	16.22	8.99E-01
cg25707669	32.59	15.07	3.06E-02	25.58	20.62	2.15E-01	76.88	48.29	1.11E-01	54.03	36.77	1.42E-01	11.75	33.68	7.27E-01
cg23801544	14.30	6.61	3.06E-02	16.45	16.65	3.23E-01	-4.21	29.46	8.86E-01	13.67	7.82	8.06E-02	27.84	23.81	2.42E-01
cg18872881	21.56	9.97	3.06E-02	17.93	21.69	4.09E-01	-17.91	47.53	7.06E-01	22.44	12.25	6.70E-02	44.85	34.74	1.97E-01
cg15791815	-7.07	3.27	3.06E-02	-8.27	6.45	2.00E-01	-1.36	10.48	8.97E-01	-7.15	4.50	1.12E-01	-8.74	9.45	3.55E-01
cg18098065	-13.33	6.16	3.06E-02	-17.94	8.09	2.67E-02	-5.94	16.88	7.25E-01	-4.59	26.36	8.62E-01	-8.10	12.79	5.27E-01
cg11804725	-10.88	5.03	3.06E-02	-11.10	11.65	3.41E-01	-12.67	9.92	2.02E-01	-10.97	8.37	1.90E-01	-8.13	11.41	4.76E-01
cg00508855	23.03	10.65	3.06E-02	43.17	19.02	2.33E-02	-0.32	33.91	9.93E-01	9.44	13.75	4.92E-01	46.77	28.15	9.67E-02
cg24869601	8.97	4.15	3.06E-02	16.72	6.99	1.68E-02	5.59	10.73	6.03E-01	13.24	8.01	9.83E-02	-0.05	6.76	9.95E-01
cg04865715	-34.19	15.81	3.06E-02	-75.05	61.04	2.19E-01	-289.67	176.82	1.01E-01	-26.69	17.14	1.19E-01	-55.71	58.19	3.38E-01
cg03062196	-8.34	3.86	3.06E-02	-8.95	7.48	2.32E-01	-1.11	14.03	9.37E-01	-7.56	5.65	1.80E-01	-12.22	8.80	1.65E-01
cg12210255	9.29	4.30	3.06E-02	9.73	6.47	1.33E-01	12.40	10.37	2.32E-01	8.33	9.53	3.82E-01	6.40	10.07	5.25E-01
cg24864413	-17.04	7.88	3.06E-02	-26.74	22.40	2.33E-01	23.31	29.32	4.27E-01	-19.24	9.13	3.51E-02	-18.22	32.31	5.73E-01
cg17987968	-11.62	5.37	3.06E-02	-15.36	9.01	8.84E-02	-9.60	12.13	4.29E-01	-19.30	11.41	9.06E-02	0.05	11.30	9.96E-01
cg00871358	18.20	8.42	3.06E-02	33.56	13.59	1.35E-02	12.12	23.61	6.08E-01	12.60	23.39	5.90E-01	5.97	14.05	6.71E-01
cg21146184	12.74	5.90	3.06E-02	2.77	11.09	8.03E-01	23.32	16.98	1.70E-01	18.86	9.79	5.40E-02	9.87	12.19	4.18E-01
cg26521402	-12.37	5.72	3.06E-02	-8.77	14.97	5.58E-01	-10.97	22.07	6.19E-01	-10.12	6.80	1.37E-01	-40.32	20.34	4.74E-02
cg00985040	-7.01	3.24	3.06E-02	-5.50	4.68	2.41E-01	-4.85	7.54	5.20E-01	5.34	14.59	7.14E-01	-13.05	6.06	3.11E-02
cg26721193	15.28	7.07	3.07E-02	29.56	21.44	1.68E-01	39.64	47.45	4.04E-01	14.02	8.47	9.80E-02	8.26	16.99	6.27E-01
cg19315854	-12.69	5.87	3.07E-02	-2.52	13.81	8.55E-01	-34.54	19.45	7.58E-02	-11.07	7.46	1.38E-01	-20.41	17.72	2.50E-01
cg26361041	-8.75	4.05	3.07E-02	-10.84	9.92	2.74E-01	-6.42	20.84	7.58E-01	-5.84	4.96	2.38E-01	-21.62	11.23	5.42E-02
cg01641432	-18.58	8.59	3.07E-02	-22.66	20.26	2.63E-01	23.83	40.68	5.58E-01	-21.13	10.57	4.55E-02	-13.91	25.47	5.85E-01
cg11537479	-12.26	5.67	3.07E-02	-27.10	14.77	6.65E-02	-18.54	17.25	2.82E-01	-7.07	7.03	3.15E-01	-17.76	18.60	3.40E-01
cg20765716	5.26	2.43	3.07E-02	4.78	3.18	1.32E-01	10.97	5.18	3.43E-02	2.35	9.86	8.12E-01	-0.82	6.70	9.03E-01

cg09997082	6.50	3.01	3.07E-02	4.00	3.97	3.13E-01	15.81	6.50	1.50E-02	6.83	10.87	5.30E-01	2.17	8.20	7.91E-01
cg00191178	9.69	4.48	3.07E-02	14.02	6.44	2.96E-02	13.77	12.35	2.65E-01	0.36	18.67	9.84E-01	3.28	7.85	6.76E-01
cg09652533	-13.33	6.17	3.07E-02	-33.50	12.29	6.41E-03	-3.10	9.72	7.50E-01	-11.07	7.87	1.59E-01	-11.23	17.32	5.17E-01
cg21951459	-13.02	6.02	3.07E-02	-14.00	12.15	2.49E-01	-24.23	16.61	1.45E-01	-7.79	8.35	3.51E-01	-22.90	18.86	2.25E-01
cg13591352	12.85	5.95	3.07E-02	12.50	8.66	1.49E-01	19.30	14.39	1.80E-01	26.72	23.64	2.59E-01	6.69	10.96	5.42E-01
cg12701768	15.35	7.10	3.07E-02	16.50	18.39	3.70E-01	18.06	20.77	3.84E-01	11.07	8.75	2.06E-01	46.39	25.93	7.36E-02
cg00003091	-38.69	17.90	3.07E-02	-30.55	21.86	1.62E-01	-109.29	88.06	2.15E-01	-81.86	57.00	1.51E-01	-29.63	41.10	4.71E-01
cg02046881	20.20	9.35	3.07E-02	21.87	19.80	2.69E-01	57.10	22.42	1.09E-02	10.89	7.59	1.52E-01	16.75	28.41	5.56E-01
cg13986536	-14.37	6.65	3.07E-02	-6.99	16.13	6.65E-01	35.15	37.92	3.54E-01	-18.98	7.78	1.47E-02	-5.72	25.30	8.21E-01
cg23881299	26.09	12.07	3.07E-02	41.78	20.40	4.06E-02	54.32	21.80	1.27E-02	7.12	11.36	5.31E-01	15.58	26.52	5.57E-01
cg21821231	-33.73	15.61	3.07E-02	-46.48	20.42	2.28E-02	1.77	64.87	9.78E-01	-57.17	42.14	1.75E-01	5.30	33.23	8.73E-01
cg10356187	20.87	9.66	3.07E-02	31.07	14.76	3.53E-02	19.84	23.73	4.03E-01	-4.47	26.29	8.65E-01	17.99	18.53	3.32E-01
cg01300405	26.38	12.21	3.07E-02	20.04	20.17	3.20E-01	-55.62	89.34	5.34E-01	39.18	17.46	2.48E-02	7.32	34.35	8.31E-01
cg05113898	17.09	7.91	3.07E-02	21.56	17.09	2.07E-01	-8.14	27.79	7.70E-01	20.02	9.98	4.48E-02	7.27	28.61	7.99E-01
cg00001582	-27.28	12.62	3.07E-02	-28.46	14.31	4.68E-02	-52.66	50.42	2.96E-01	7.20	41.41	8.62E-01	-37.64	48.87	4.41E-01
cg02012771	17.68	8.18	3.07E-02	25.30	21.45	2.38E-01	22.36	28.92	4.39E-01	15.68	9.74	1.07E-01	16.68	31.24	5.93E-01
cg02316713	10.19	4.71	3.07E-02	9.77	7.27	1.79E-01	10.33	8.57	2.28E-01	15.92	16.04	3.21E-01	8.29	10.81	4.43E-01
cg25116789	21.17	9.80	3.07E-02	32.11	20.87	1.24E-01	35.59	46.15	4.41E-01	19.05	12.35	1.23E-01	4.73	30.28	8.76E-01
cg26621127	22.13	10.24	3.07E-02	36.11	19.34	6.19E-02	35.05	37.97	3.56E-01	10.91	14.01	4.36E-01	32.24	30.54	2.91E-01
cg06926703	-6.05	2.80	3.07E-02	-5.72	4.71	2.24E-01	-4.96	8.75	5.71E-01	-7.23	4.51	1.09E-01	-4.60	7.01	5.12E-01
cg22155039	8.29	3.83	3.07E-02	7.86	5.22	1.33E-01	9.00	8.17	2.70E-01	9.86	10.23	3.35E-01	6.80	12.10	5.74E-01
cg02385153	-14.09	6.52	3.07E-02	-11.80	10.60	2.66E-01	-12.94	17.01	4.47E-01	-18.70	15.47	2.27E-01	-14.81	11.96	2.16E-01
cg20123649	20.27	9.38	3.07E-02	0.05	15.34	9.98E-01	33.27	15.58	3.27E-02	16.52	22.31	4.59E-01	40.00	22.76	7.89E-02
cg08954863	19.88	9.20	3.07E-02	28.39	15.72	7.09E-02	-3.72	46.77	9.37E-01	21.57	14.35	1.33E-01	6.88	20.22	7.34E-01
cg04471045	18.74	8.67	3.07E-02	10.46	13.21	4.28E-01	3.68	18.23	8.40E-01	30.63	17.30	7.66E-02	48.78	24.18	4.37E-02
cg09586183	14.44	6.68	3.07E-02	4.12	5.71	4.71E-01	27.82	9.32	2.84E-03	28.22	12.60	2.51E-02	5.81	9.22	5.28E-01
cg14193097	17.78	8.23	3.07E-02	0.08	20.18	9.97E-01	1.18	24.66	9.62E-01	24.43	10.27	1.73E-02	24.22	29.05	4.04E-01
cg12350762	27.87	12.90	3.07E-02	47.31	18.53	1.07E-02	-18.00	42.53	6.72E-01	40.73	26.40	1.23E-01	7.53	21.32	7.24E-01
cg10631530	-24.31	11.25	3.07E-02	-16.01	15.14	2.90E-01	0.52	38.39	9.89E-01	-61.97	34.87	7.55E-02	-35.18	22.18	1.13E-01
cg25278343	21.44	9.92	3.07E-02	14.65	18.74	4.35E-01	32.94	18.10	6.87E-02	13.10	18.49	4.79E-01	27.87	27.40	3.09E-01
cg19653594	-7.08	3.28	3.07E-02	-6.99	4.34	1.07E-01	-11.81	6.89	8.67E-02	-7.48	20.99	7.22E-01	-1.37	7.73	8.60E-01
cg14062119	10.30	4.77	3.08E-02	9.48	9.08	2.96E-01	23.31	11.77	4.76E-02	12.28	10.17	2.27E-01	3.41	8.17	6.76E-01
cg22458693	-13.17	6.10	3.08E-02	-23.98	13.32	7.18E-02	-26.29	17.38	1.30E-01	-9.46	8.27	2.53E-01	1.85	17.32	9.15E-01
cg19032532	12.93	5.99	3.08E-02	13.59	9.83	1.67E-01	-2.33	11.29	8.37E-01	23.19	18.31	2.05E-01	24.07	11.67	3.92E-02
cg14319235	11.84	5.48	3.08E-02	14.30	8.96	1.11E-01	-7.54	14.92	6.13E-01	21.97	11.18	4.95E-02	8.90	10.93	4.16E-01

cg12981843	16.46	7.62	3.08E-02	3.17	13.17	8.10E-01	35.46	14.01	1.14E-02	16.33	16.56	3.24E-01	9.22	19.15	6.30E-01
cg26389950	5.58	2.58	3.08E-02	5.73	4.24	1.77E-01	0.44	5.32	9.35E-01	10.31	5.40	5.62E-02	6.01	6.35	3.44E-01
cg16056449	13.72	6.35	3.08E-02	15.59	12.84	2.25E-01	-1.87	17.63	9.16E-01	17.60	9.11	5.35E-02	11.42	16.96	5.01E-01
cg17510385	-14.11	6.53	3.08E-02	-15.80	8.25	5.54E-02	1.80	35.59	9.60E-01	-38.98	22.95	8.94E-02	-4.27	12.87	7.40E-01
cg04945753	-9.34	4.32	3.08E-02	-12.75	7.59	9.32E-02	-21.97	12.04	6.80E-02	-1.92	7.25	7.91E-01	-8.82	9.88	3.72E-01
cg21871151	-43.50	20.14	3.08E-02	-50.96	28.34	7.22E-02	1.34	59.70	9.82E-01	-76.94	47.02	1.02E-01	-19.24	45.30	6.71E-01
cg25778304	-26.84	12.42	3.08E-02	-26.69	18.08	1.40E-01	-23.39	30.58	4.44E-01	-33.76	34.09	3.22E-01	-25.61	25.93	3.23E-01
cg00797651	11.63	5.38	3.08E-02	9.68	7.97	2.25E-01	21.43	19.59	2.74E-01	22.07	15.33	1.50E-01	8.32	9.16	3.64E-01
cg07230786	-20.27	9.39	3.08E-02	-13.29	16.78	4.28E-01	-28.62	20.16	1.56E-01	-31.16	18.86	9.84E-02	-9.83	19.90	6.21E-01
cg06353425	15.91	7.37	3.08E-02	9.12	16.74	5.86E-01	75.97	51.24	1.38E-01	17.25	9.02	5.58E-02	9.00	21.34	6.73E-01
ch.7.246556R	-10.58	4.90	3.08E-02	-12.00	6.94	8.35E-02	-8.85	9.65	3.59E-01	-9.10	16.06	5.71E-01	-9.73	12.61	4.41E-01
cg13695746	-13.87	6.42	3.08E-02	-15.60	11.98	1.93E-01	-27.38	16.98	1.07E-01	-6.24	9.60	5.16E-01	-21.95	18.39	2.33E-01
cg14509153	11.45	5.30	3.08E-02	15.18	7.20	3.51E-02	10.72	13.48	4.26E-01	15.71	16.32	3.36E-01	-0.47	11.93	9.68E-01
cg13640626	25.56	11.83	3.08E-02	42.56	18.69	2.28E-02	-18.99	36.08	5.99E-01	13.22	18.08	4.65E-01	44.16	27.47	1.08E-01
cg17853575	54.08	25.04	3.08E-02	130.32	50.77	1.03E-02	-104.17	240.43	6.65E-01	40.40	22.93	7.80E-02	9.67	70.44	8.91E-01
cg09024124	22.56	10.44	3.08E-02	25.38	13.10	5.26E-02	77.60	37.50	3.85E-02	30.21	32.65	3.55E-01	5.70	15.37	7.11E-01
cg06398881	-15.57	7.21	3.08E-02	-22.66	13.33	8.93E-02	-14.83	17.56	3.98E-01	-5.12	12.20	6.75E-01	-24.61	16.58	1.38E-01
cg03289906	9.78	4.53	3.08E-02	7.30	7.59	3.36E-01	9.89	9.77	3.11E-01	18.02	9.31	5.30E-02	4.13	10.30	6.88E-01
cg14286320	16.21	7.51	3.08E-02	3.92	19.57	8.41E-01	-21.98	36.85	5.51E-01	19.48	8.66	2.45E-02	31.71	30.62	3.00E-01
cg26897717	-49.17	22.77	3.08E-02	-33.82	39.50	3.92E-01	138.42	197.66	4.84E-01	-44.76	32.99	1.75E-01	-103.52	53.94	5.49E-02
cg16813864	18.58	8.61	3.08E-02	14.41	13.92	3.01E-01	33.63	15.90	3.45E-02	16.57	20.09	4.09E-01	1.33	22.87	9.53E-01
cg12830119	-18.59	8.61	3.08E-02	-19.29	10.85	7.52E-02	5.16	29.33	8.60E-01	-14.05	45.64	7.58E-01	-25.68	17.28	1.37E-01
cg11062079	8.72	4.04	3.08E-02	8.99	5.83	1.23E-01	13.73	9.05	1.29E-01	14.83	15.21	3.30E-01	2.48	8.10	7.59E-01
cg17029690	27.11	12.56	3.08E-02	44.64	37.89	2.39E-01	59.16	181.28	7.44E-01	29.53	13.80	3.24E-02	-43.03	52.02	4.08E-01
cg05202389	-12.11	5.61	3.08E-02	-10.78	11.35	3.43E-01	-13.55	13.35	3.10E-01	-10.43	8.34	2.11E-01	-18.59	15.70	2.36E-01
cg21887097	-7.56	3.50	3.09E-02	-13.34	5.93	2.44E-02	-6.98	9.30	4.53E-01	-4.89	5.75	3.96E-01	-0.76	9.38	9.35E-01
cg12141662	18.02	8.35	3.09E-02	29.26	20.88	1.61E-01	6.95	26.79	7.95E-01	15.89	10.30	1.23E-01	25.76	28.31	3.63E-01
cg04263316	-16.69	7.73	3.09E-02	-20.71	12.16	8.85E-02	-10.94	15.80	4.89E-01	-12.94	21.89	5.55E-01	-17.62	16.05	2.72E-01
cg12299795	-15.18	7.03	3.09E-02	-27.21	15.39	7.71E-02	-7.23	15.40	6.39E-01	-15.11	10.35	1.45E-01	-8.44	20.15	6.75E-01
cg05464534	32.00	14.82	3.09E-02	28.37	18.75	1.30E-01	12.62	50.19	8.02E-01	61.28	53.44	2.51E-01	40.07	32.27	2.14E-01
ch.2.4817431F	-12.69	5.88	3.09E-02	-18.04	11.91	1.30E-01	5.04	14.55	7.29E-01	-23.70	11.40	3.77E-02	-8.61	10.28	4.02E-01
cg16829924	-20.14	9.33	3.09E-02	-12.61	10.66	2.37E-01	-70.51	27.71	1.09E-02	-21.39	20.10	2.87E-01	-12.97	14.42	3.69E-01
cg01034411	-11.20	5.19	3.09E-02	-26.67	8.64	2.02E-03	-8.55	11.36	4.51E-01	-5.49	5.02	2.74E-01	-6.53	10.51	5.34E-01
cg26401541	-8.04	3.72	3.09E-02	-17.67	6.50	6.60E-03	-10.19	14.45	4.81E-01	-3.16	4.02	4.31E-01	-7.15	8.67	4.10E-01
cg14609520	-49.52	22.94	3.09E-02	-54.55	25.99	3.59E-02	-127.37	69.88	6.84E-02	42.69	64.54	5.08E-01	-50.18	48.07	2.97E-01

cg02524941	-19.78	9.17	3.09E-02	-11.92	16.20	4.62E-01	-19.71	23.86	4.09E-01	-24.90	14.85	9.37E-02	-23.61	23.54	3.16E-01
cg17879299	6.93	3.21	3.09E-02	3.19	4.55	4.84E-01	13.58	7.21	5.97E-02	2.84	8.57	7.40E-01	13.91	7.98	8.16E-02
cg24119225	-8.41	3.90	3.09E-02	-3.51	4.33	4.17E-01	-4.67	6.66	4.84E-01	-21.90	7.09	2.01E-03	-7.55	6.25	2.27E-01
cg02396865	-46.12	21.37	3.09E-02	-41.78	27.92	1.35E-01	-104.98	67.98	1.23E-01	-29.86	64.58	6.44E-01	-38.89	47.06	4.09E-01
cg12441126	9.78	4.53	3.09E-02	6.28	6.61	3.42E-01	12.03	12.51	3.36E-01	21.12	22.85	3.55E-01	12.28	7.55	1.04E-01
cg01058360	5.14	2.38	3.09E-02	3.77	3.08	2.20E-01	10.66	5.65	5.91E-02	5.94	7.14	4.05E-01	2.91	7.11	6.82E-01
cg09965668	-28.38	13.15	3.09E-02	-20.07	17.86	2.61E-01	-55.02	41.55	1.86E-01	9.92	36.68	7.87E-01	-57.93	27.48	3.50E-02
cg14040131	-12.58	5.83	3.09E-02	-14.88	7.06	3.50E-02	-28.33	18.86	1.33E-01	-28.21	16.08	7.94E-02	-1.23	7.85	8.75E-01
cg23859313	-52.75	24.44	3.09E-02	-76.39	20.27	1.65E-04	43.29	39.75	2.76E-01	-89.63	37.17	1.59E-02	-65.79	26.84	1.42E-02
cg03210827	21.24	9.84	3.09E-02	-6.50	22.75	7.75E-01	20.95	48.13	6.63E-01	29.96	11.94	1.21E-02	13.33	32.54	6.82E-01
cg03253314	21.10	9.77	3.09E-02	1.48	21.53	9.45E-01	-93.77	90.48	3.00E-01	28.05	11.67	1.62E-02	27.34	34.33	4.26E-01
cg22700148	-15.43	7.15	3.09E-02	-17.81	11.72	1.29E-01	-21.92	15.10	1.46E-01	-21.30	18.83	2.58E-01	-3.17	14.03	8.21E-01
cg25735572	-11.05	5.12	3.09E-02	-16.84	7.46	2.40E-02	6.62	15.26	6.64E-01	-7.19	12.21	5.56E-01	-10.81	10.45	3.01E-01
cg20626645	-20.04	9.28	3.09E-02	-4.77	33.42	8.86E-01	-111.85	171.47	5.14E-01	-20.17	10.14	4.68E-02	-29.79	32.42	3.58E-01
cg04208928	7.81	3.62	3.09E-02	8.69	8.04	2.80E-01	22.55	9.60	1.88E-02	4.04	4.92	4.11E-01	5.80	10.75	5.89E-01
cg13855998	15.86	7.35	3.09E-02	16.23	15.58	2.97E-01	29.83	19.55	1.27E-01	18.02	10.02	7.20E-02	-17.12	23.53	4.67E-01
cg15718066	19.19	8.89	3.09E-02	26.76	16.27	1.00E-01	3.35	27.29	9.02E-01	20.91	15.40	1.75E-01	14.79	17.37	3.94E-01
cg13704271	-30.02	13.91	3.09E-02	-27.83	17.88	1.20E-01	-55.79	47.96	2.45E-01	-22.04	73.95	7.66E-01	-28.00	26.51	2.91E-01
cg08956101	-9.83	4.55	3.09E-02	-13.01	10.45	2.13E-01	-7.16	16.78	6.70E-01	-6.59	5.82	2.57E-01	-22.56	12.94	8.12E-02
cg02711886	8.88	4.11	3.09E-02	7.92	5.50	1.50E-01	17.80	11.69	1.28E-01	11.56	9.36	2.17E-01	0.09	11.69	9.94E-01
cg23142035	-27.79	12.88	3.09E-02	-29.18	15.23	5.53E-02	-21.70	63.53	7.33E-01	-35.28	83.18	6.71E-01	-23.59	27.46	3.90E-01
cg17174275	-16.65	7.72	3.09E-02	-28.39	10.41	6.38E-03	3.61	15.84	8.20E-01	-4.94	18.07	7.84E-01	-22.76	16.10	1.58E-01
cg10792302	6.49	3.01	3.09E-02	4.17	4.90	3.94E-01	3.80	6.01	5.27E-01	9.00	6.81	1.86E-01	12.42	7.13	8.15E-02
cg13449257	-10.52	4.87	3.09E-02	-16.09	7.79	3.89E-02	-3.17	13.35	8.12E-01	-7.91	11.44	4.89E-01	-8.04	8.99	3.71E-01
cg04251368	9.54	4.42	3.09E-02	7.54	8.87	3.95E-01	35.09	19.12	6.64E-02	11.32	6.46	7.99E-02	2.14	9.22	8.17E-01
cg00078025	-7.71	3.57	3.09E-02	-12.30	4.63	7.85E-03	2.61	9.07	7.73E-01	2.28	14.90	8.79E-01	-4.77	8.15	5.58E-01
cg20874031	-6.16	2.86	3.09E-02	-7.05	4.66	1.30E-01	-7.72	7.61	3.10E-01	-4.66	4.76	3.28E-01	-6.06	8.09	4.54E-01
cg11703745	12.23	5.67	3.09E-02	12.26	7.99	1.25E-01	18.34	12.80	1.52E-01	-0.78	19.05	9.67E-01	11.95	12.30	3.31E-01
cg05023099	25.22	11.69	3.09E-02	48.12	20.79	2.06E-02	3.99	24.68	8.71E-01	11.50	15.34	4.53E-01	53.27	30.05	7.62E-02
cg01601573	-7.29	3.38	3.09E-02	-4.99	4.22	2.38E-01	0.42	7.36	9.54E-01	-17.44	9.92	7.86E-02	-13.95	6.79	4.01E-02
cg01789501	-9.34	4.33	3.10E-02	-8.87	8.81	3.14E-01	-3.64	12.99	7.79E-01	-12.72	6.15	3.85E-02	-3.22	11.08	7.71E-01
cg15114651	-13.92	6.45	3.10E-02	-12.65	9.96	2.04E-01	-14.66	15.52	3.45E-01	-25.02	17.62	1.56E-01	-9.95	12.33	4.19E-01
cg19814280	9.77	4.53	3.10E-02	10.39	9.98	2.98E-01	-0.29	17.61	9.87E-01	10.79	5.76	6.11E-02	8.95	13.63	5.12E-01
cg20500551	10.42	4.83	3.10E-02	28.19	11.43	1.36E-02	8.14	13.91	5.59E-01	5.78	6.27	3.57E-01	9.00	14.64	5.39E-01
cg26348243	-4.43	2.06	3.10E-02	-4.19	2.48	9.05E-02	-4.33	4.82	3.68E-01	-8.88	9.24	3.37E-01	-4.00	7.28	5.82E-01

cg05886574	-44.63	20.69	3.10E-02	-36.37	24.16	1.32E-01	-48.23	126.24	7.02E-01	10.56	93.72	9.10E-01	-89.89	47.32	5.75E-02
cg11787791	14.80	6.86	3.10E-02	7.14	10.28	4.87E-01	14.87	13.36	2.66E-01	17.03	18.68	3.62E-01	34.77	17.41	4.59E-02
cg11619216	13.02	6.04	3.10E-02	10.48	8.60	2.23E-01	16.26	12.79	2.04E-01	10.96	18.22	5.47E-01	17.35	14.45	2.30E-01
cg25747427	-10.90	5.05	3.10E-02	-14.00	7.03	4.63E-02	-11.99	11.13	2.81E-01	7.26	18.68	6.98E-01	-8.44	11.21	4.51E-01
cg23318804	-38.99	18.07	3.10E-02	-86.00	54.35	1.14E-01	-330.50	238.07	1.65E-01	-32.85	20.22	1.04E-01	-15.72	62.12	8.00E-01
cg24673221	-12.21	5.66	3.10E-02	-12.69	13.47	3.46E-01	17.09	17.97	3.42E-01	-16.58	9.49	8.06E-02	-15.66	9.29	9.19E-02
cg07697597	10.60	4.91	3.10E-02	16.38	7.99	4.04E-02	13.89	11.90	2.43E-01	7.28	11.45	5.25E-01	2.62	9.49	7.82E-01
cg12594001	21.42	9.93	3.10E-02	14.53	16.46	3.77E-01	46.43	27.49	9.13E-02	29.43	18.95	1.20E-01	8.63	20.65	6.76E-01
cg08951638	5.76	2.67	3.10E-02	3.77	3.69	3.06E-01	12.84	6.29	4.13E-02	5.82	6.98	4.04E-01	4.13	6.90	5.50E-01
cg24553417	5.58	2.59	3.10E-02	5.35	3.26	1.01E-01	9.58	6.63	1.49E-01	4.66	7.65	5.42E-01	2.12	8.07	7.93E-01
cg12627695	11.56	5.36	3.10E-02	18.23	9.19	4.72E-02	7.38	10.93	5.00E-01	14.36	13.40	2.84E-01	4.95	10.54	6.39E-01
cg02417427	8.61	3.99	3.10E-02	8.27	5.30	1.19E-01	22.21	11.24	4.82E-02	9.72	10.38	3.49E-01	-1.99	10.01	8.42E-01
cg11592640	-9.45	4.38	3.10E-02	-7.61	11.09	4.93E-01	3.09	23.35	8.95E-01	-9.10	5.25	8.28E-02	-18.30	13.19	1.65E-01
cg25153233	-8.23	3.82	3.10E-02	-12.77	5.74	2.62E-02	-6.90	8.25	4.03E-01	-5.40	10.76	6.16E-01	-2.00	8.16	8.06E-01
cg24339704	4.94	2.29	3.10E-02	4.14	2.72	1.28E-01	14.27	6.20	2.14E-02	-0.60	9.23	9.49E-01	1.03	7.46	8.90E-01
cg09441223	-23.68	10.98	3.10E-02	-30.73	14.32	3.19E-02	28.90	45.69	5.27E-01	-41.19	30.66	1.79E-01	-8.85	23.09	7.01E-01
cg16576935	-16.69	7.74	3.10E-02	-38.35	17.12	2.51E-02	-15.30	33.65	6.49E-01	-9.11	9.74	3.49E-01	-20.60	23.23	3.75E-01
cg16375358	-10.83	5.02	3.10E-02	-8.15	7.98	3.07E-01	-6.28	11.06	5.70E-01	-18.46	12.28	1.33E-01	-13.98	10.48	1.82E-01
cg07840318	6.98	3.24	3.10E-02	6.23	4.31	1.48E-01	-2.39	7.85	7.61E-01	18.10	8.74	3.85E-02	9.11	6.99	1.93E-01
cg17626301	-6.20	2.87	3.11E-02	-3.89	4.24	3.59E-01	-13.47	7.28	6.44E-02	-5.64	6.18	3.61E-01	-6.50	7.03	3.55E-01
cg18733967	-17.02	7.89	3.11E-02	-19.62	13.38	1.43E-01	8.56	18.55	6.45E-01	-20.94	16.59	2.07E-01	-28.56	15.93	7.31E-02
cg21913528	-8.26	3.83	3.11E-02	-5.06	6.74	4.53E-01	-16.09	14.07	2.53E-01	-8.91	5.59	1.11E-01	-9.43	10.53	3.71E-01
cg09967877	35.96	16.68	3.11E-02	-11.40	44.90	8.00E-01	69.03	272.80	8.00E-01	48.05	19.13	1.20E-02	7.60	53.24	8.86E-01
cg04317051	15.20	7.05	3.11E-02	3.59	15.53	8.17E-01	42.43	28.05	1.30E-01	17.51	8.72	4.46E-02	4.34	25.36	8.64E-01
cg05176131	34.06	15.80	3.11E-02	41.01	23.27	7.80E-02	-27.84	50.23	5.79E-01	20.89	16.67	2.10E-01	78.98	30.59	9.82E-03
cg03503046	-9.47	4.39	3.11E-02	-20.60	5.78	3.63E-04	-3.44	8.10	6.71E-01	-6.07	5.64	2.82E-01	-3.93	8.23	6.33E-01
cg08393660	14.38	6.67	3.11E-02	3.36	11.11	7.62E-01	18.92	21.57	3.81E-01	19.30	10.42	6.41E-02	25.64	18.16	1.58E-01
cg02232840	-12.55	5.82	3.11E-02	-16.82	18.37	3.60E-01	-11.91	32.90	7.17E-01	-12.14	6.41	5.83E-02	-11.00	27.90	6.93E-01
cg06920324	18.33	8.50	3.11E-02	14.28	16.96	4.00E-01	0.56	28.05	9.84E-01	18.58	11.66	1.11E-01	38.43	24.05	1.10E-01
cg00812438	10.38	4.82	3.11E-02	11.33	9.66	2.41E-01	4.96	13.00	7.03E-01	20.28	8.87	2.22E-02	2.85	8.52	7.38E-01
cg25535999	-15.46	7.17	3.11E-02	1.82	15.11	9.04E-01	-18.76	20.18	3.53E-01	-18.05	9.76	6.44E-02	-34.68	21.78	1.11E-01
cg05190718	-10.93	5.07	3.11E-02	-18.67	7.98	1.93E-02	-2.57	10.44	8.06E-01	0.12	10.33	9.91E-01	-18.89	11.19	9.14E-02
cg20013286	27.74	12.87	3.11E-02	42.06	38.08	2.69E-01	41.14	180.86	8.20E-01	27.83	14.35	5.24E-02	4.56	46.52	9.22E-01
cg05685056	-10.69	4.96	3.11E-02	-17.57	7.06	1.28E-02	-10.14	12.55	4.19E-01	3.21	16.52	8.46E-01	-2.80	9.70	7.73E-01
cg22888418	19.59	9.09	3.11E-02	30.85	17.91	8.50E-02	37.05	25.43	1.45E-01	9.31	13.53	4.91E-01	16.61	22.44	4.59E-01

cg14538146	22.01	10.21	3.11E-02	-0.17	21.02	9.94E-01	38.70	123.71	7.54E-01	29.95	12.65	1.79E-02	21.52	31.41	4.93E-01
cg04642105	15.00	6.96	3.11E-02	19.03	18.89	3.14E-01	8.85	31.22	7.77E-01	13.53	8.01	9.13E-02	29.48	28.38	2.99E-01
cg18013830	-42.79	19.85	3.11E-02	-38.29	24.66	1.20E-01	-33.42	82.02	6.84E-01	-39.22	74.48	5.98E-01	-59.50	42.08	1.57E-01
cg10274453	5.44	2.52	3.11E-02	5.39	3.93	1.70E-01	5.01	6.08	4.10E-01	7.22	4.46	1.06E-01	0.46	8.15	9.55E-01
cg00988247	22.61	10.49	3.11E-02	27.22	15.10	7.15E-02	9.46	31.21	7.62E-01	26.61	23.42	2.56E-01	15.07	23.18	5.16E-01
cg10124993	9.13	4.24	3.11E-02	9.01	7.20	2.11E-01	13.05	9.74	1.81E-01	7.23	7.26	3.20E-01	8.72	11.98	4.67E-01
cg18813390	-60.29	27.97	3.11E-02	-59.69	37.78	1.14E-01	92.90	149.92	5.35E-01	-36.26	60.48	5.49E-01	-113.43	62.03	6.75E-02
cg18133966	-11.99	5.56	3.11E-02	-11.81	9.79	2.27E-01	-10.44	11.69	3.72E-01	-13.13	11.98	2.73E-01	-12.70	11.49	2.69E-01
cg12173535	-15.57	7.22	3.11E-02	-8.18	10.95	4.55E-01	11.34	37.97	7.65E-01	-30.70	12.44	1.36E-02	-10.82	16.51	5.12E-01
cg07692853	13.28	6.16	3.11E-02	18.71	8.38	2.56E-02	15.95	15.90	3.16E-01	15.35	24.55	5.32E-01	-0.78	12.41	9.50E-01
cg10785340	8.30	3.85	3.11E-02	12.23	5.26	2.02E-02	9.96	10.92	3.62E-01	4.44	9.70	6.47E-01	-0.96	8.97	9.15E-01
cg26182037	-38.70	17.95	3.11E-02	-92.70	52.63	7.82E-02	-93.48	139.37	5.02E-01	-33.76	20.01	9.16E-02	12.92	71.92	8.57E-01
cg22803223	-8.38	3.89	3.11E-02	-16.10	8.01	4.44E-02	-14.08	14.62	3.36E-01	-2.12	4.93	6.67E-01	-14.75	8.75	9.20E-02
cg25051111	11.63	5.40	3.11E-02	11.95	6.02	4.70E-02	0.42	27.26	9.88E-01	8.90	15.60	5.68E-01	25.69	28.24	3.63E-01
cg09549591	-13.23	6.14	3.11E-02	-24.19	9.31	9.37E-03	6.74	15.34	6.61E-01	-10.77	12.70	3.96E-01	-10.18	12.49	4.15E-01
cg07202413	23.67	10.98	3.11E-02	41.31	15.44	7.45E-03	29.13	26.03	2.63E-01	32.19	27.04	2.34E-01	-0.25	15.63	9.87E-01
cg17240133	-7.84	3.64	3.11E-02	-11.80	6.55	7.18E-02	-3.90	10.65	7.14E-01	-6.11	5.32	2.51E-01	-8.28	11.06	4.54E-01
cg00906147	27.16	12.60	3.11E-02	16.77	14.19	2.37E-01	46.18	41.75	2.69E-01	-16.62	58.69	7.77E-01	65.14	28.16	2.07E-02
cg11732619	15.83	7.35	3.11E-02	10.87	21.16	6.08E-01	-11.92	40.22	7.67E-01	17.17	8.74	4.94E-02	19.99	19.67	3.10E-01
cg05299774	-14.66	6.80	3.11E-02	-42.07	17.00	1.33E-02	-8.25	17.62	6.40E-01	-10.06	8.57	2.40E-01	-9.06	18.83	6.30E-01
cg07976328	-12.29	5.70	3.11E-02	-15.62	13.19	2.36E-01	-5.16	10.67	6.29E-01	-13.18	8.95	1.41E-01	-20.96	16.37	2.00E-01
cg08472398	36.52	16.94	3.11E-02	33.88	27.92	2.25E-01	-135.37	94.26	1.51E-01	38.93	14.18	6.05E-03	68.68	40.62	9.08E-02
cg18417201	18.64	8.65	3.12E-02	17.63	20.01	3.78E-01	25.20	39.79	5.26E-01	18.83	10.59	7.54E-02	16.11	27.50	5.58E-01
cg00199846	-21.69	10.06	3.12E-02	-27.29	37.26	4.64E-01	17.11	159.78	9.15E-01	-19.40	10.73	7.07E-02	-61.42	47.87	1.99E-01
cg23372852	-11.44	5.31	3.12E-02	-1.59	10.41	8.79E-01	-7.48	19.16	6.96E-01	-16.66	7.22	2.10E-02	-11.83	15.20	4.36E-01
cg06605558	16.80	7.80	3.12E-02	28.37	17.66	1.08E-01	31.58	34.46	3.59E-01	14.33	9.39	1.27E-01	-3.58	30.78	9.07E-01
cg21574349	-12.65	5.87	3.12E-02	-31.26	15.91	4.94E-02	4.40	44.69	9.22E-01	-9.97	6.77	1.41E-01	-10.28	18.97	5.88E-01
cg13683667	-34.15	15.85	3.12E-02	-48.25	20.32	1.76E-02	99.52	76.74	1.95E-01	-42.58	30.76	1.66E-01	-27.20	24.41	2.65E-01
cg20636794	19.33	8.97	3.12E-02	30.06	16.96	7.63E-02	5.13	18.07	7.76E-01	19.25	14.99	1.99E-01	23.88	26.40	3.66E-01
cg27203924	-8.53	3.96	3.12E-02	-12.27	9.17	1.81E-01	0.31	15.98	9.84E-01	-7.39	4.90	1.31E-01	-14.37	12.52	2.51E-01
cg10175910	14.91	6.92	3.12E-02	0.08	16.52	9.96E-01	32.22	20.16	1.10E-01	13.35	8.65	1.23E-01	38.29	26.77	1.53E-01
cg15488009	7.39	3.43	3.12E-02	7.05	5.64	2.11E-01	9.34	6.99	1.82E-01	7.95	8.98	3.76E-01	5.64	6.94	4.16E-01
cg08936706	18.81	8.73	3.12E-02	7.50	14.32	6.01E-01	28.70	15.37	6.19E-02	28.09	20.80	1.77E-01	14.03	24.25	5.63E-01
cg10734665	-10.18	4.73	3.12E-02	-10.55	6.78	1.20E-01	-23.75	12.88	6.52E-02	4.38	11.05	6.92E-01	-13.55	10.68	2.05E-01
cg15032166	11.10	5.15	3.12E-02	16.43	8.21	4.53E-02	6.28	14.68	6.69E-01	15.64	10.82	1.48E-01	1.20	10.18	9.06E-01

cg05824973	-10.84	5.03	3.12E-02	-14.72	7.55	5.11E-02	-7.38	10.51	4.83E-01	7.87	14.46	5.86E-01	-17.36	11.11	1.18E-01
cg14893576	9.75	4.53	3.12E-02	10.89	5.73	5.73E-02	11.69	11.76	3.20E-01	12.91	19.32	5.04E-01	2.96	10.92	7.86E-01
cg13672103	22.55	10.47	3.12E-02	19.12	21.69	3.78E-01	-44.41	50.03	3.75E-01	28.09	13.21	3.34E-02	25.20	33.94	4.58E-01
cg06663648	-44.43	20.62	3.12E-02	-29.44	22.94	1.99E-01	-75.83	143.20	5.96E-01	-113.87	81.45	1.62E-01	-109.55	62.91	8.16E-02
cg20795139	17.00	7.89	3.12E-02	-5.71	25.27	8.21E-01	-27.85	86.19	7.47E-01	19.31	8.57	2.42E-02	31.03	37.08	4.03E-01
cg05949203	14.78	6.86	3.12E-02	19.53	11.15	7.99E-02	24.14	18.11	1.83E-01	0.95	11.73	9.35E-01	26.42	18.60	1.55E-01
cg01569709	15.30	7.10	3.12E-02	17.68	10.32	8.68E-02	22.70	16.61	1.72E-01	14.70	22.63	5.16E-01	5.43	14.35	7.05E-01
cg15023038	10.46	4.86	3.12E-02	15.50	7.25	3.25E-02	3.48	10.05	7.30E-01	-2.24	17.83	9.00E-01	11.73	9.84	2.33E-01
cg06820243	-30.81	14.30	3.12E-02	-23.48	18.00	1.92E-01	-24.47	88.25	7.82E-01	-72.65	41.50	8.00E-02	-30.02	30.22	3.20E-01
cg22573528	-32.38	15.03	3.12E-02	-32.49	21.94	1.39E-01	76.94	68.33	2.60E-01	-46.60	30.62	1.28E-01	-39.88	30.60	1.92E-01
cg21243919	22.07	10.24	3.12E-02	22.76	20.17	2.59E-01	28.42	54.70	6.03E-01	13.96	14.50	3.35E-01	39.62	22.48	7.79E-02
cg06309788	10.53	4.89	3.12E-02	13.27	6.49	4.11E-02	11.34	11.60	3.28E-01	-0.55	17.37	9.75E-01	5.90	11.63	6.12E-01
cg27516100	7.81	3.63	3.12E-02	6.82	4.45	1.26E-01	11.99	9.62	2.13E-01	15.21	13.39	2.56E-01	3.90	10.45	7.09E-01
cg13844341	6.33	2.94	3.12E-02	3.48	3.57	3.30E-01	14.88	7.35	4.29E-02	7.05	11.95	5.55E-01	11.45	9.22	2.14E-01
cg25697357	-10.49	4.87	3.12E-02	-15.69	10.59	1.38E-01	-7.13	14.33	6.19E-01	-7.56	6.76	2.64E-01	-15.79	12.43	2.04E-01
cg12103152	19.93	9.25	3.12E-02	31.85	15.40	3.86E-02	13.15	23.41	5.74E-01	20.14	25.02	4.21E-01	10.47	15.73	5.06E-01
cg26826957	-7.95	3.69	3.13E-02	-10.54	9.20	2.52E-01	-2.70	11.72	8.18E-01	-7.73	4.70	1.00E-01	-9.86	10.48	3.47E-01
cg12572625	9.91	4.60	3.13E-02	14.04	7.38	5.72E-02	18.05	10.97	9.97E-02	-2.14	9.38	8.20E-01	9.19	10.42	3.78E-01
cg16733419	-8.96	4.16	3.13E-02	-6.05	9.25	5.13E-01	-3.49	13.03	7.89E-01	-12.22	5.39	2.34E-02	-1.00	13.16	9.40E-01
cg13861749	-9.02	4.19	3.13E-02	-16.33	9.07	7.18E-02	11.08	20.26	5.85E-01	-7.55	5.13	1.41E-01	-12.73	15.14	4.00E-01
cg02978140	-5.19	2.41	3.13E-02	-6.41	3.47	6.47E-02	-5.29	4.94	2.85E-01	3.13	7.65	6.83E-01	-6.39	5.68	2.61E-01
cg27592794	8.59	3.99	3.13E-02	11.88	5.26	2.40E-02	9.85	9.13	2.81E-01	-5.96	17.00	7.26E-01	1.17	9.42	9.01E-01
cg12975597	-8.27	3.84	3.13E-02	-8.48	8.70	3.29E-01	16.52	19.80	4.04E-01	-10.08	4.82	3.65E-02	-6.32	10.51	5.48E-01
cg11557901	4.45	2.07	3.13E-02	4.23	2.82	1.35E-01	8.89	5.28	9.25E-02	2.93	4.61	5.25E-01	2.16	6.20	7.28E-01
cg15977773	-8.19	3.80	3.13E-02	-12.63	6.91	6.75E-02	-3.91	8.80	6.56E-01	-9.21	6.25	1.41E-01	-1.58	10.16	8.76E-01
cg01923089	7.40	3.44	3.13E-02	7.34	4.60	1.11E-01	12.04	7.89	1.27E-01	10.33	9.85	2.94E-01	-1.81	9.51	8.49E-01
cg03537386	9.07	4.21	3.13E-02	9.62	5.70	9.13E-02	15.62	10.22	1.26E-01	2.86	10.97	7.94E-01	5.42	11.42	6.35E-01
cg09856709	24.14	11.21	3.13E-02	21.90	17.05	1.99E-01	36.10	29.36	2.19E-01	35.17	28.51	2.17E-01	14.86	21.68	4.93E-01
cg20304988	-15.68	7.28	3.13E-02	-18.98	19.65	3.34E-01	-13.77	24.41	5.73E-01	-15.30	8.66	7.72E-02	-15.45	28.23	5.84E-01
cg15006864	13.13	6.10	3.13E-02	6.35	8.67	4.64E-01	19.41	12.74	1.28E-01	28.85	16.64	8.30E-02	11.75	16.17	4.67E-01
cg09629675	-11.07	5.14	3.13E-02	-14.80	7.96	6.29E-02	-7.13	11.43	5.33E-01	-11.92	13.72	3.85E-01	-7.40	10.48	4.80E-01
cg23228450	-15.03	6.98	3.13E-02	-18.96	10.39	6.80E-02	-17.78	14.82	2.30E-01	-10.95	20.51	5.94E-01	-5.99	15.20	6.94E-01
cg05516012	-26.97	12.53	3.13E-02	-42.89	22.37	5.52E-02	-19.29	27.98	4.91E-01	-10.30	22.44	6.46E-01	-36.98	29.98	2.17E-01
cg00203089	33.38	15.50	3.13E-02	26.63	58.94	6.51E-01	-12.74	223.17	9.54E-01	34.37	16.77	4.04E-02	31.12	57.98	5.91E-01
cg00374263	-4.85	2.25	3.13E-02	-4.75	3.52	1.77E-01	-4.51	4.33	2.98E-01	-7.21	5.84	2.17E-01	-3.58	5.43	5.09E-01

cg01792749	-6.80	3.16	3.13E-02	-15.93	7.27	2.85E-02	5.76	13.23	6.63E-01	-4.82	3.93	2.19E-01	-9.25	9.54	3.32E-01
cg17121140	-6.22	2.89	3.13E-02	-2.75	3.76	4.64E-01	-9.40	4.69	4.50E-02	2.50	8.55	7.70E-01	-13.14	5.95	2.72E-02
cg17112426	9.63	4.47	3.13E-02	6.82	6.28	2.78E-01	16.47	10.30	1.10E-01	9.68	11.24	3.89E-01	10.48	11.66	3.69E-01
cg01557791	-21.48	9.97	3.13E-02	-58.91	37.74	1.19E-01	79.08	147.93	5.93E-01	-17.89	10.73	9.55E-02	-36.63	40.07	3.61E-01
cg07765813	39.08	18.15	3.13E-02	50.16	31.84	1.15E-01	-175.43	129.11	1.74E-01	32.63	14.60	2.54E-02	80.01	46.80	8.74E-02
cg03577837	-15.08	7.01	3.13E-02	-30.54	13.22	2.08E-02	-10.71	18.05	5.53E-01	-12.71	11.18	2.55E-01	0.59	16.72	9.72E-01
cg05813194	20.68	9.61	3.13E-02	49.82	25.92	5.46E-02	2.24	102.72	9.83E-01	20.25	9.57	3.43E-02	-28.03	36.25	4.39E-01
cg22156456	14.96	6.95	3.13E-02	14.18	9.66	1.42E-01	19.62	17.09	2.51E-01	34.00	18.61	6.76E-02	-2.06	16.48	9.01E-01
cg22252999	4.79	2.23	3.13E-02	2.92	3.36	3.85E-01	5.29	4.31	2.19E-01	7.09	4.88	1.47E-01	7.20	7.53	3.39E-01
cg21048700	-7.73	3.59	3.13E-02	-10.61	7.14	1.37E-01	-0.94	8.05	9.07E-01	-7.45	5.46	1.72E-01	-14.24	10.59	1.79E-01
cg02023973	5.82	2.70	3.13E-02	4.57	3.28	1.63E-01	10.66	6.40	9.61E-02	5.62	10.48	5.91E-01	5.82	9.80	5.52E-01
cg18516365	21.76	10.11	3.13E-02	13.98	24.29	5.65E-01	106.88	48.50	2.76E-02	19.12	10.59	7.09E-02	17.30	25.11	4.91E-01
cg01039752	11.92	5.54	3.13E-02	19.72	10.36	5.70E-02	16.17	10.10	1.09E-01	1.15	11.22	9.18E-01	6.75	13.42	6.15E-01
cg24749470	-7.62	3.54	3.13E-02	-14.19	6.46	2.81E-02	-5.32	7.75	4.93E-01	-5.70	6.28	3.64E-01	-2.54	8.53	7.65E-01
cg24023097	31.07	14.43	3.13E-02	56.18	44.31	2.05E-01	-96.91	130.11	4.56E-01	30.77	16.23	5.79E-02	21.68	48.08	6.52E-01
cg01752322	16.85	7.83	3.13E-02	26.89	22.98	2.42E-01	43.47	32.92	1.87E-01	14.12	8.99	1.16E-01	8.26	29.59	7.80E-01
cg16692735	-5.02	2.33	3.13E-02	-6.24	3.21	5.17E-02	-1.53	5.62	7.86E-01	5.49	8.41	5.14E-01	-8.47	4.95	8.72E-02
cg02630207	-10.25	4.76	3.13E-02	-20.44	12.68	1.07E-01	-0.12	21.44	9.95E-01	-9.85	5.50	7.30E-02	0.60	19.59	9.75E-01
cg05134013	-72.89	33.86	3.14E-02	-73.60	45.73	1.08E-01	214.26	187.39	2.53E-01	-97.68	65.27	1.34E-01	-88.34	87.48	3.13E-01
cg19342782	4.79	2.23	3.14E-02	6.33	3.16	4.51E-02	4.70	5.85	4.22E-01	3.69	4.63	4.25E-01	0.91	6.23	8.84E-01
cg07298177	6.06	2.81	3.14E-02	5.23	3.57	1.43E-01	14.06	7.29	5.37E-02	0.74	8.58	9.31E-01	5.19	8.03	5.18E-01
cg02937763	-12.37	5.75	3.14E-02	-8.56	10.98	4.36E-01	-1.71	17.28	9.21E-01	-15.23	8.38	6.93E-02	-18.45	15.09	2.21E-01
cg04102208	-28.32	13.16	3.14E-02	-16.44	18.84	3.83E-01	-76.87	38.47	4.57E-02	-28.67	38.49	4.56E-01	-28.59	24.94	2.52E-01
cg26479028	-8.83	4.10	3.14E-02	-4.61	8.75	5.98E-01	6.09	15.12	6.87E-01	-10.68	5.28	4.31E-02	-17.65	12.79	1.67E-01
cg17565113	-12.14	5.64	3.14E-02	-1.55	12.02	8.98E-01	-38.13	20.61	6.43E-02	-12.28	7.10	8.39E-02	-16.23	20.75	4.34E-01
cg05332854	6.92	3.22	3.14E-02	3.73	4.57	4.14E-01	8.17	6.50	2.09E-01	18.57	11.86	1.18E-01	9.19	7.47	2.18E-01
cg10359143	-7.90	3.67	3.14E-02	-9.18	7.80	2.39E-01	-8.96	12.10	4.59E-01	-6.19	5.12	2.27E-01	-10.73	8.79	2.22E-01
cg09849405	14.28	6.64	3.14E-02	-8.92	21.80	6.82E-01	20.14	48.95	6.81E-01	17.91	7.31	1.44E-02	0.00	25.86	1.00E+00
cg06081580	-33.77	15.69	3.14E-02	-29.15	19.90	1.43E-01	33.70	105.45	7.49E-01	-67.44	44.97	1.34E-01	-34.89	32.37	2.81E-01
cg14425564	10.57	4.91	3.14E-02	13.91	9.96	1.62E-01	12.65	9.32	1.75E-01	13.79	9.81	1.60E-01	0.94	10.29	9.27E-01
cg04747036	-28.12	13.06	3.14E-02	-28.89	16.92	8.77E-02	-2.24	37.65	9.53E-01	-65.94	28.67	2.15E-02	3.31	32.56	9.19E-01
cg14032195	-16.75	7.78	3.14E-02	-33.22	22.26	1.36E-01	-13.60	30.69	6.58E-01	-13.88	9.05	1.25E-01	-21.00	28.58	4.62E-01
cg08047482	13.26	6.16	3.14E-02	19.55	8.55	2.23E-02	16.37	18.31	3.71E-01	12.97	18.57	4.85E-01	-0.63	12.13	9.58E-01
cg12576153	-39.50	18.35	3.14E-02	-20.05	37.11	5.89E-01	-133.14	236.30	5.73E-01	-39.51	22.79	8.29E-02	-81.05	57.83	1.61E-01
cg14387743	8.85	4.11	3.14E-02	8.70	5.66	1.24E-01	16.94	6.43	8.47E-03	6.94	9.49	4.64E-01	-5.61	10.04	5.76E-01

cg11363197	43.40	20.16	3.14E-02	14.28	17.45	4.13E-01	9.28	51.45	8.57E-01	71.43	39.16	6.81E-02	79.21	25.47	1.87E-03
cg09447182	29.43	13.67	3.14E-02	8.60	17.24	6.18E-01	69.92	35.62	4.97E-02	51.94	38.41	1.76E-01	40.48	28.22	1.51E-01
cg08895928	13.48	6.26	3.14E-02	1.58	14.08	9.10E-01	9.36	12.56	4.56E-01	9.37	16.88	5.79E-01	22.97	9.71	1.80E-02
cg19434164	13.35	6.21	3.14E-02	14.43	8.04	7.28E-02	12.30	14.00	3.80E-01	22.48	24.81	3.65E-01	6.47	16.26	6.91E-01
cg06378107	16.90	7.85	3.14E-02	8.77	17.23	6.11E-01	68.21	41.65	1.01E-01	13.09	9.63	1.74E-01	42.86	25.84	9.72E-02
cg11784191	20.24	9.40	3.14E-02	41.79	17.40	1.63E-02	-2.94	18.54	8.74E-01	19.51	16.55	2.38E-01	19.83	23.25	3.94E-01
cg27327334	20.95	9.73	3.14E-02	30.25	16.48	6.64E-02	-22.05	39.87	5.80E-01	15.43	15.18	3.09E-01	29.69	22.90	1.95E-01
cg04508884	-44.50	20.68	3.14E-02	-69.05	25.22	6.18E-03	-12.92	76.87	8.67E-01	35.96	60.58	5.53E-01	-24.16	47.18	6.09E-01
cg09009536	16.37	7.61	3.14E-02	37.36	18.57	4.42E-02	14.48	25.40	5.69E-01	18.93	9.39	4.38E-02	-5.58	16.11	7.29E-01
cg24282845	17.79	8.26	3.14E-02	18.93	21.42	3.77E-01	37.99	30.60	2.14E-01	16.60	9.82	9.11E-02	6.34	31.22	8.39E-01
cg25707945	25.79	11.98	3.14E-02	26.49	15.27	8.27E-02	61.34	50.87	2.28E-01	2.76	42.04	9.48E-01	23.62	24.10	3.27E-01
cg21946195	10.77	5.01	3.14E-02	11.39	7.12	1.10E-01	9.92	11.55	3.91E-01	22.26	12.35	7.16E-02	-2.44	12.77	8.48E-01
cg11217934	-13.65	6.34	3.14E-02	-18.03	16.34	2.70E-01	-59.91	35.64	9.28E-02	-9.44	7.43	2.04E-01	-24.17	21.23	2.55E-01
cg13983319	12.24	5.69	3.14E-02	35.19	17.98	5.03E-02	20.71	22.11	3.49E-01	9.89	7.57	1.92E-01	6.55	10.97	5.50E-01
cg01423335	19.31	8.97	3.14E-02	22.48	10.22	2.78E-02	13.38	60.73	8.26E-01	-22.28	53.98	6.80E-01	12.82	21.17	5.45E-01
cg20690488	9.41	4.37	3.14E-02	-3.60	10.43	7.30E-01	7.89	11.57	4.95E-01	15.17	5.83	9.24E-03	3.14	12.73	8.05E-01
cg26705720	24.27	11.28	3.14E-02	56.59	32.22	7.90E-02	-41.10	133.87	7.59E-01	17.26	12.59	1.70E-01	55.45	43.22	2.00E-01
cg09372063	18.84	8.75	3.14E-02	11.09	15.00	4.60E-01	26.63	16.12	9.86E-02	8.97	21.20	6.72E-01	29.24	19.87	1.41E-01
cg03344820	25.67	11.93	3.14E-02	51.49	39.03	1.87E-01	51.89	117.74	6.59E-01	23.93	13.23	7.06E-02	10.48	41.30	8.00E-01
cg15301489	26.54	12.33	3.14E-02	30.93	31.51	3.26E-01	19.95	69.03	7.73E-01	19.71	14.76	1.82E-01	63.58	36.17	7.88E-02
ch.1.3178922R	-25.77	11.98	3.14E-02	-23.01	13.21	8.14E-02	-28.61	53.78	5.95E-01	-57.22	56.66	3.13E-01	-34.46	41.55	4.07E-01
cg24564603	5.55	2.58	3.14E-02	8.67	4.37	4.73E-02	0.98	5.46	8.58E-01	4.19	4.60	3.62E-01	8.68	7.62	2.55E-01
cg16261857	-9.07	4.21	3.14E-02	-11.07	8.41	1.88E-01	-2.55	11.22	8.20E-01	-8.31	6.15	1.77E-01	-14.58	11.28	1.96E-01
cg08336604	-12.38	5.75	3.14E-02	-2.19	10.38	8.33E-01	-17.32	16.43	2.92E-01	-9.79	5.17	5.84E-02	-36.82	14.77	1.27E-02
cg20256403	25.11	11.67	3.14E-02	17.34	36.71	6.37E-01	150.84	172.61	3.82E-01	22.31	12.90	8.36E-02	58.60	42.64	1.69E-01
cg17306814	-10.18	4.73	3.14E-02	-4.61	11.89	6.98E-01	-27.73	19.92	1.64E-01	-11.04	5.58	4.79E-02	0.80	18.43	9.65E-01
cg02614016	-13.38	6.22	3.14E-02	-1.01	13.33	9.40E-01	18.58	26.04	4.76E-01	-20.33	6.72	2.51E-03	-14.24	14.32	3.20E-01
cg00460049	24.29	11.29	3.14E-02	40.46	17.56	2.12E-02	0.27	45.20	9.95E-01	30.41	28.90	2.93E-01	7.83	18.52	6.73E-01
cg19621271	20.23	9.41	3.14E-02	19.45	13.12	1.38E-01	23.50	21.51	2.75E-01	9.32	35.86	7.95E-01	22.57	19.78	2.54E-01
cg01432520	-8.46	3.93	3.15E-02	-10.08	7.39	1.72E-01	0.03	13.55	9.98E-01	-7.06	5.52	2.01E-01	-16.22	11.13	1.45E-01
cg04177287	-8.34	3.87	3.15E-02	-14.55	7.41	4.98E-02	-9.30	9.93	3.49E-01	-1.44	5.32	7.87E-01	-17.23	9.49	6.95E-02
cg09209002	-16.55	7.70	3.15E-02	-6.71	22.41	7.65E-01	40.26	125.63	7.49E-01	-20.92	8.40	1.28E-02	41.73	38.80	2.82E-01
cg21645554	10.55	4.90	3.15E-02	6.97	7.80	3.71E-01	18.21	9.86	6.46E-02	9.31	13.05	4.76E-01	9.12	10.55	3.87E-01
cg23713724	12.68	5.89	3.15E-02	19.79	13.40	1.40E-01	11.52	18.51	5.34E-01	18.24	7.95	2.17E-02	-6.76	12.72	5.95E-01
cg20761290	34.55	16.06	3.15E-02	28.47	17.26	9.91E-02	-52.64	73.78	4.76E-01	95.42	78.44	2.24E-01	73.37	39.82	6.54E-02

cg16918050	-8.68	4.03	3.15E-02	-7.70	7.61	3.12E-01	-6.14	8.40	4.64E-01	-11.83	7.30	1.05E-01	-8.11	9.44	3.91E-01
cg04984709	-13.76	6.39	3.15E-02	-19.23	18.30	2.93E-01	7.05	26.97	7.94E-01	-14.29	7.39	5.30E-02	-15.11	23.78	5.25E-01
cg06094482	-6.87	3.20	3.15E-02	-6.97	4.62	1.32E-01	-2.75	7.25	7.04E-01	-4.77	8.74	5.85E-01	-12.18	7.24	9.26E-02
cg04919263	21.25	9.88	3.15E-02	60.99	38.46	1.13E-01	157.56	146.34	2.82E-01	19.57	11.21	8.09E-02	8.59	25.24	7.33E-01
cg14566103	11.87	5.52	3.15E-02	18.83	9.41	4.54E-02	-4.74	11.58	6.82E-01	18.67	12.23	1.27E-01	11.86	11.62	3.08E-01
cg14523238	-10.29	4.79	3.15E-02	-11.11	7.28	1.27E-01	5.07	13.03	6.97E-01	-17.66	10.08	7.97E-02	-10.56	10.51	3.15E-01
cg12798118	26.05	12.11	3.15E-02	40.53	41.70	3.31E-01	-165.33	140.36	2.39E-01	27.82	13.27	3.61E-02	9.19	44.16	8.35E-01
cg07446438	-29.01	13.49	3.15E-02	-40.57	32.52	2.12E-01	111.56	156.03	4.75E-01	-27.64	15.62	7.67E-02	-30.10	49.48	5.43E-01
cg16744314	-10.49	4.88	3.15E-02	-10.44	6.90	1.30E-01	-27.14	13.73	4.81E-02	1.33	10.79	9.02E-01	-12.49	11.85	2.92E-01
cg21142349	-7.91	3.68	3.15E-02	-5.56	8.26	5.01E-01	-24.19	15.66	1.22E-01	-9.45	4.55	3.79E-02	7.23	11.92	5.44E-01
cg09805925	-17.18	7.99	3.15E-02	1.68	17.91	9.25E-01	-35.94	41.39	3.85E-01	-18.22	10.01	6.88E-02	-35.88	22.36	1.09E-01
cg14469826	-5.07	2.36	3.15E-02	-6.65	4.71	1.57E-01	-3.58	5.41	5.09E-01	-5.53	3.70	1.35E-01	-3.09	6.03	6.08E-01
cg01266375	-7.75	3.61	3.15E-02	-3.65	6.64	5.82E-01	-10.10	11.54	3.81E-01	-11.15	5.35	3.70E-02	-4.04	9.23	6.61E-01
cg14138132	19.90	9.25	3.15E-02	21.26	20.39	2.97E-01	-92.28	92.41	3.18E-01	19.15	10.92	7.94E-02	40.89	36.06	2.57E-01
cg01805856	21.48	9.99	3.15E-02	29.46	19.54	1.32E-01	53.50	39.21	1.72E-01	11.66	13.62	3.92E-01	29.69	27.08	2.73E-01
cg23554497	10.52	4.89	3.15E-02	9.68	6.97	1.65E-01	4.94	11.54	6.69E-01	2.03	17.90	9.10E-01	18.64	9.73	5.55E-02
cg09453870	26.02	12.10	3.15E-02	36.25	19.42	6.20E-02	96.56	41.61	2.03E-02	15.45	13.27	2.44E-01	10.62	20.75	6.09E-01
cg12506013	-5.64	2.62	3.15E-02	-4.05	3.76	2.82E-01	-12.13	5.64	3.17E-02	2.21	7.98	7.82E-01	-6.79	6.01	2.59E-01
cg25023752	-33.30	15.49	3.15E-02	-20.88	18.31	2.54E-01	-93.69	63.08	1.38E-01	-48.25	73.02	5.09E-01	-58.77	36.55	1.08E-01
cg25102726	5.25	2.44	3.15E-02	2.65	3.76	4.81E-01	6.57	5.43	2.26E-01	7.78	6.85	2.57E-01	7.28	4.89	1.36E-01
cg02771142	16.24	7.55	3.15E-02	16.31	12.00	1.74E-01	13.16	20.87	5.28E-01	25.28	17.81	1.56E-01	11.97	13.95	3.91E-01
cg27069188	11.73	5.46	3.15E-02	13.86	7.02	4.83E-02	-5.85	17.57	7.39E-01	23.73	23.83	3.19E-01	10.84	10.98	3.24E-01
cg00277384	18.23	8.48	3.15E-02	-0.33	14.67	9.82E-01	33.34	16.43	4.24E-02	16.77	15.89	2.91E-01	35.95	22.48	1.10E-01
cg05016425	-35.42	16.48	3.15E-02	-14.50	21.52	5.00E-01	-56.54	53.15	2.87E-01	-113.59	65.58	8.32E-02	-56.29	32.67	8.48E-02
cg21746884	15.51	7.21	3.15E-02	22.19	20.03	2.68E-01	8.92	21.32	6.76E-01	12.45	8.75	1.55E-01	41.42	26.17	1.13E-01
cg07236562	-7.83	3.64	3.15E-02	-12.99	7.15	6.90E-02	-1.85	7.25	7.98E-01	-7.31	6.17	2.36E-01	-10.34	9.76	2.89E-01
cg10792982	-12.07	5.61	3.15E-02	-27.54	15.74	8.03E-02	-16.75	15.42	2.77E-01	-8.21	6.88	2.33E-01	-11.96	20.74	5.64E-01
cg27223805	13.45	6.26	3.16E-02	15.44	10.62	1.46E-01	21.38	17.84	2.31E-01	12.79	11.06	2.48E-01	6.53	13.65	6.32E-01
cg12683929	11.79	5.48	3.16E-02	15.21	7.27	3.65E-02	5.92	15.45	7.02E-01	4.94	24.61	8.41E-01	8.41	10.82	4.37E-01
cg02914087	28.01	13.03	3.16E-02	30.30	17.16	7.74E-02	17.92	59.76	7.64E-01	19.94	48.52	6.81E-01	27.14	23.61	2.50E-01
cg17824173	-21.56	10.03	3.16E-02	-35.63	23.52	1.30E-01	-133.57	78.79	9.00E-02	-13.46	11.14	2.27E-01	-34.82	30.49	2.53E-01
cg10308749	-7.30	3.39	3.16E-02	-6.37	5.87	2.78E-01	4.11	9.58	6.68E-01	-9.79	5.40	7.01E-02	-12.51	8.89	1.59E-01
cg13624833	9.16	4.26	3.16E-02	15.35	6.60	2.00E-02	7.35	9.90	4.58E-01	5.77	9.45	5.41E-01	1.13	9.69	9.07E-01
cg06022179	12.43	5.78	3.16E-02	14.17	10.34	1.70E-01	15.11	15.42	3.27E-01	15.19	11.18	1.74E-01	6.48	10.93	5.53E-01
cg01751245	-5.71	2.66	3.16E-02	-8.82	4.38	4.40E-02	-0.87	6.03	8.86E-01	-4.25	4.78	3.74E-01	-7.59	7.38	3.03E-01

cg05992395	-14.56	6.77	3.16E-02	-27.72	10.34	7.33E-03	2.34	11.47	8.38E-01	-10.40	8.61	2.27E-01	-25.70	14.77	8.18E-02
cg13255398	9.72	4.52	3.16E-02	12.93	6.09	3.36E-02	12.95	11.89	2.76E-01	8.06	17.43	6.44E-01	0.72	9.30	9.38E-01
cg06981279	-9.94	4.62	3.16E-02	-5.82	6.45	3.68E-01	-9.81	10.69	3.59E-01	-21.25	25.31	4.01E-01	-16.54	8.95	6.47E-02
cg05147143	-11.40	5.30	3.16E-02	-5.67	13.86	6.82E-01	-33.18	28.43	2.43E-01	-11.18	6.24	7.33E-02	-13.89	17.04	4.15E-01
cg08541649	-9.52	4.43	3.16E-02	-14.45	11.38	2.04E-01	-8.61	23.01	7.08E-01	-10.33	5.18	4.62E-02	6.54	15.54	6.74E-01
cg08711063	13.32	6.20	3.16E-02	26.83	14.66	6.73E-02	27.73	18.85	1.41E-01	5.62	8.33	5.00E-01	15.13	15.50	3.29E-01
cg14251777	24.04	11.18	3.16E-02	32.48	15.65	3.79E-02	9.61	38.72	8.04E-01	41.09	29.87	1.69E-01	3.34	21.69	8.78E-01
cg24048517	12.75	5.93	3.16E-02	20.41	9.58	3.31E-02	21.25	12.85	9.84E-02	-2.26	12.47	8.56E-01	5.15	14.07	7.14E-01
cg08447934	13.13	6.11	3.16E-02	18.43	8.68	3.37E-02	14.46	13.19	2.73E-01	15.95	18.93	4.00E-01	-4.07	14.15	7.74E-01
cg08229018	15.25	7.09	3.16E-02	8.79	13.02	5.00E-01	15.96	22.10	4.70E-01	12.01	11.02	2.76E-01	32.38	16.46	4.91E-02
cg16770400	-9.65	4.49	3.16E-02	-14.16	11.39	2.13E-01	-9.93	21.11	6.38E-01	-8.14	5.27	1.23E-01	-14.96	16.64	3.69E-01
cg07671752	9.31	4.33	3.16E-02	8.81	5.45	1.06E-01	6.47	10.66	5.44E-01	6.92	16.15	6.68E-01	16.55	11.93	1.65E-01
cg27645955	17.54	8.16	3.16E-02	33.95	35.53	3.39E-01	92.46	149.37	5.36E-01	16.36	8.78	6.25E-02	16.65	28.68	5.62E-01
cg23836594	9.33	4.34	3.16E-02	11.29	6.62	8.81E-02	15.42	8.84	8.12E-02	1.88	12.99	8.85E-01	2.52	9.32	7.86E-01
cg14192299	-4.38	2.04	3.16E-02	-5.38	2.77	5.23E-02	-4.53	4.47	3.12E-01	1.50	8.22	8.55E-01	-3.27	4.64	4.81E-01
cg10008589	12.91	6.01	3.16E-02	23.16	13.12	7.75E-02	23.25	16.93	1.70E-01	3.20	10.12	7.52E-01	12.83	10.76	2.33E-01
cg14059988	-26.38	12.27	3.16E-02	-26.41	23.34	2.58E-01	-97.91	59.55	1.00E-01	-20.07	16.73	2.30E-01	-28.78	32.50	3.76E-01
cg19305511	-35.43	16.49	3.16E-02	-47.58	19.12	1.28E-02	-10.87	72.43	8.81E-01	22.30	74.41	7.64E-01	-3.70	41.82	9.29E-01
cg10414946	-11.98	5.58	3.16E-02	-22.15	13.98	1.13E-01	4.07	16.59	8.06E-01	-11.84	6.98	8.97E-02	-15.17	18.63	4.15E-01
cg01655958	-9.26	4.31	3.16E-02	-4.37	9.02	6.29E-01	-18.20	16.93	2.82E-01	-10.54	5.77	6.77E-02	-8.09	11.17	4.69E-01
cg01395127	6.18	2.88	3.16E-02	6.15	4.93	2.13E-01	5.02	6.41	4.34E-01	6.04	5.13	2.39E-01	8.21	7.60	2.80E-01
cg15563070	-11.50	5.35	3.16E-02	-19.97	7.79	1.03E-02	-4.05	13.29	7.60E-01	-4.78	15.35	7.55E-01	-3.37	10.86	7.57E-01
cg12620942	23.93	11.13	3.16E-02	66.38	34.24	5.25E-02	-25.02	128.74	8.46E-01	21.68	12.34	7.88E-02	-7.91	41.49	8.49E-01
cg02451774	-4.45	2.07	3.16E-02	-5.98	2.94	4.24E-02	-4.51	5.15	3.81E-01	-2.10	5.34	6.94E-01	-2.32	4.73	6.24E-01
cg13888662	-18.80	8.75	3.17E-02	-24.71	21.55	2.52E-01	19.93	34.73	5.66E-01	-21.75	10.36	3.58E-02	-8.20	36.31	8.21E-01
cg02862467	5.36	2.50	3.17E-02	3.76	3.22	2.43E-01	12.07	6.04	4.59E-02	7.57	7.43	3.09E-01	1.66	7.36	8.22E-01
cg04637454	9.32	4.34	3.17E-02	11.59	6.35	6.81E-02	5.48	10.91	6.15E-01	14.29	10.01	1.54E-01	1.96	10.01	8.45E-01
cg00449756	-24.39	11.35	3.17E-02	-34.94	13.16	7.93E-03	-23.33	50.02	6.41E-01	-19.00	34.74	5.85E-01	20.72	29.24	4.78E-01
cg23707590	-8.76	4.08	3.17E-02	-3.34	6.66	6.16E-01	-5.44	12.40	6.61E-01	-13.53	6.76	4.54E-02	-13.07	10.43	2.10E-01
cg00749220	19.47	9.06	3.17E-02	23.37	12.76	6.70E-02	26.15	19.88	1.88E-01	24.03	36.43	5.10E-01	3.37	19.05	8.60E-01
cg16304950	22.44	10.45	3.17E-02	39.21	32.64	2.30E-01	31.21	29.13	2.84E-01	23.33	13.77	9.02E-02	5.09	23.75	8.30E-01
cg07244927	5.01	2.33	3.17E-02	3.93	3.88	3.11E-01	5.45	4.86	2.62E-01	4.01	4.79	4.03E-01	8.11	5.65	1.51E-01
cg08649099	65.02	30.26	3.17E-02	39.12	63.11	5.35E-01	-119.83	302.52	6.92E-01	45.57	26.69	8.77E-02	172.47	66.64	9.65E-03
cg03039990	8.98	4.18	3.17E-02	6.99	6.01	2.45E-01	17.07	10.36	9.96E-02	1.88	10.58	8.59E-01	12.84	9.42	1.73E-01
cg06680511	-10.86	5.05	3.17E-02	-6.91	6.83	3.12E-01	-12.14	10.86	2.64E-01	-22.13	25.72	3.89E-01	-18.16	11.36	1.10E-01

cg19853143	6.50	3.03	3.17E-02	6.81	5.00	1.73E-01	4.99	5.45	3.59E-01	2.36	7.01	7.37E-01	14.64	8.14	7.19E-02
cg06357908	-8.43	3.92	3.17E-02	-22.84	9.89	2.09E-02	-9.51	11.81	4.20E-01	-4.78	4.98	3.38E-01	-7.34	11.74	5.32E-01
cg22041988	32.20	14.99	3.17E-02	74.79	30.17	1.32E-02	-87.15	115.25	4.50E-01	23.10	11.43	4.32E-02	27.75	44.24	5.31E-01
cg27357232	20.88	9.72	3.17E-02	9.26	26.19	7.24E-01	-8.62	78.04	9.12E-01	25.78	11.12	2.04E-02	0.53	33.81	9.88E-01
cg22983827	-10.92	5.08	3.17E-02	-18.12	10.85	9.47E-02	1.43	20.00	9.43E-01	-12.00	6.85	7.98E-02	-2.57	12.50	8.37E-01
cg04844120	18.49	8.61	3.17E-02	10.08	19.58	6.07E-01	21.08	63.39	7.39E-01	18.06	10.05	7.23E-02	53.26	36.87	1.49E-01
cg04292159	-16.01	7.45	3.17E-02	-26.06	12.80	4.18E-02	3.59	14.65	8.06E-01	-13.46	16.15	4.05E-01	-27.59	17.09	1.07E-01
cg05798125	31.87	14.83	3.17E-02	72.69	36.43	4.60E-02	-74.16	144.11	6.07E-01	21.46	17.14	2.11E-01	60.60	54.26	2.64E-01
cg26283879	-18.22	8.48	3.17E-02	-14.54	9.50	1.26E-01	-17.39	34.11	6.10E-01	-38.55	55.96	4.91E-01	-39.25	24.55	1.10E-01
cg10854819	-3.78	1.76	3.17E-02	-4.49	2.57	8.05E-02	-3.39	3.01	2.60E-01	3.24	7.94	6.83E-01	-4.76	4.66	3.07E-01
cg20796611	-6.97	3.24	3.17E-02	-12.56	4.65	6.94E-03	1.32	6.80	8.46E-01	-1.17	10.57	9.12E-01	-6.69	5.96	2.62E-01
cg01943155	-10.57	4.92	3.17E-02	-1.87	8.12	8.18E-01	-26.52	11.16	1.75E-02	-8.65	9.41	3.58E-01	-12.35	9.69	2.02E-01
cg20523861	6.32	2.94	3.17E-02	6.94	4.07	8.85E-02	1.41	7.24	8.45E-01	-1.61	12.65	8.99E-01	9.83	5.77	8.83E-02
cg27171569	2.85	1.33	3.17E-02	3.36	1.90	7.70E-02	4.34	2.80	1.21E-01	2.34	4.49	6.01E-01	0.18	2.94	9.52E-01
cg14196263	7.32	3.41	3.17E-02	9.04	4.59	4.86E-02	14.39	9.59	1.34E-01	-1.08	8.06	8.94E-01	4.92	9.03	5.86E-01
cg27187555	9.30	4.33	3.17E-02	9.08	5.50	9.90E-02	21.92	10.07	2.94E-02	8.48	11.95	4.78E-01	-4.69	11.21	6.75E-01
cg00898111	-9.63	4.48	3.17E-02	-15.07	7.27	3.81E-02	-0.68	10.44	9.48E-01	-9.91	10.12	3.27E-01	-7.63	9.16	4.05E-01
cg03402351	-5.22	2.43	3.17E-02	-2.71	3.56	4.47E-01	-2.99	6.15	6.27E-01	-5.33	5.85	3.62E-01	-12.51	5.36	1.96E-02
cg09741342	-42.43	19.75	3.17E-02	-60.25	22.85	8.35E-03	-30.03	91.82	7.44E-01	0.72	91.16	9.94E-01	24.95	49.50	6.14E-01
cg11504081	31.57	14.70	3.17E-02	17.51	16.44	2.87E-01	78.45	63.34	2.16E-01	106.44	58.97	7.11E-02	46.65	33.79	1.67E-01
cg00725388	31.07	14.46	3.17E-02	31.37	16.98	6.47E-02	66.38	60.84	2.75E-01	-24.02	71.87	7.38E-01	31.16	34.31	3.64E-01
cg13304825	7.87	3.66	3.17E-02	5.48	3.75	1.44E-01	15.79	7.85	4.43E-02	19.87	11.59	8.64E-02	-2.04	10.00	8.38E-01
cg12808596	11.17	5.20	3.17E-02	14.08	7.23	5.13E-02	-3.08	10.94	7.78E-01	15.10	16.15	3.50E-01	19.77	13.35	1.38E-01
cg19374305	3.64	1.70	3.17E-02	2.52	2.49	3.11E-01	0.89	3.36	7.90E-01	9.23	4.79	5.39E-02	7.03	4.32	1.04E-01
cg02278499	16.47	7.67	3.17E-02	13.17	11.67	2.59E-01	22.74	21.74	2.96E-01	27.68	24.18	2.52E-01	15.06	13.09	2.50E-01
cg07931792	-50.06	23.31	3.17E-02	-47.18	24.72	5.63E-02	83.21	80.24	3.00E-01	-64.24	52.11	2.18E-01	-89.49	42.31	3.44E-02
cg24686551	6.33	2.95	3.17E-02	6.44	3.84	9.33E-02	10.92	7.68	1.55E-01	7.31	9.71	4.52E-01	1.47	7.12	8.36E-01
cg24919768	7.31	3.41	3.17E-02	4.00	5.00	4.24E-01	1.81	6.18	7.70E-01	18.67	9.58	5.12E-02	13.06	6.60	4.77E-02
cg15557144	13.10	6.10	3.17E-02	0.56	12.85	9.65E-01	29.50	14.38	4.03E-02	15.41	8.93	8.44E-02	3.74	17.00	8.26E-01
cg17846016	4.46	2.08	3.17E-02	3.75	2.54	1.40E-01	7.00	5.24	1.82E-01	9.19	7.10	1.96E-01	0.83	6.85	9.03E-01
cg16250812	-9.69	4.51	3.17E-02	-7.21	9.09	4.28E-01	-1.86	11.93	8.76E-01	-8.01	6.57	2.23E-01	-27.76	12.08	2.15E-02
cg15225325	5.38	2.50	3.17E-02	2.93	3.43	3.93E-01	13.54	6.06	2.54E-02	5.15	5.96	3.88E-01	4.94	7.20	4.93E-01
cg10211193	-7.30	3.40	3.17E-02	-9.62	5.91	1.04E-01	-5.66	9.83	5.65E-01	-8.23	5.53	1.37E-01	-1.97	8.15	8.09E-01
cg27343325	-12.19	5.68	3.18E-02	-17.65	8.80	4.49E-02	-4.13	11.90	7.29E-01	-18.47	17.69	2.96E-01	-7.91	11.27	4.83E-01
cg01855636	12.38	5.76	3.18E-02	15.54	11.29	1.69E-01	-10.75	24.16	6.56E-01	12.29	7.63	1.07E-01	17.20	17.23	3.18E-01

cg27171201	15.21	7.08	3.18E-02	18.37	12.14	1.30E-01	15.40	13.92	2.68E-01	1.73	14.05	9.02E-01	30.91	18.49	9.47E-02
cg26923410	-25.52	11.88	3.18E-02	-29.47	18.19	1.05E-01	-19.41	32.85	5.55E-01	-61.31	32.04	5.57E-02	-6.46	21.53	7.64E-01
cg21714557	-8.20	3.82	3.18E-02	-8.16	6.19	1.87E-01	-1.93	7.70	8.02E-01	-9.57	11.08	3.88E-01	-13.66	7.56	7.09E-02
cg00808370	-12.67	5.90	3.18E-02	-25.86	15.22	8.93E-02	-33.29	25.91	1.99E-01	-8.55	6.96	2.19E-01	-11.52	20.96	5.83E-01
cg18764577	12.22	5.69	3.18E-02	33.39	15.37	2.98E-02	8.60	20.69	6.78E-01	12.67	7.96	1.11E-01	1.84	10.85	8.65E-01
cg14752451	18.70	8.71	3.18E-02	20.48	10.92	6.07E-02	42.90	29.74	1.49E-01	-9.05	33.27	7.86E-01	12.46	19.02	5.13E-01
cg00746143	13.50	6.28	3.18E-02	9.43	17.00	5.79E-01	-37.60	34.20	2.72E-01	17.07	7.26	1.87E-02	8.57	22.18	6.99E-01
cg25527090	11.69	5.44	3.18E-02	25.34	11.80	3.17E-02	6.75	12.95	6.02E-01	6.99	8.59	4.16E-01	10.96	11.90	3.57E-01
cg03917521	-26.99	12.57	3.18E-02	-29.82	15.22	5.00E-02	-72.94	34.48	3.44E-02	-27.85	52.65	5.97E-01	0.57	23.02	9.80E-01
cg21935519	9.01	4.20	3.18E-02	15.84	8.13	5.13E-02	3.34	9.52	7.26E-01	9.65	7.06	1.72E-01	3.93	9.74	6.87E-01
cg25947283	-14.80	6.89	3.18E-02	-21.24	10.93	5.20E-02	-20.22	19.73	3.05E-01	-17.19	13.25	1.94E-01	3.67	15.07	8.08E-01
cg16219283	-6.55	3.05	3.18E-02	-6.35	3.93	1.06E-01	-11.40	9.72	2.41E-01	-8.22	10.49	4.33E-01	-4.23	6.56	5.19E-01
cg11293241	6.03	2.81	3.18E-02	6.79	5.25	1.95E-01	5.65	4.30	1.89E-01	11.49	6.80	9.13E-02	-2.39	8.21	7.70E-01
cg13244241	-29.42	13.70	3.18E-02	-46.21	22.53	4.03E-02	12.51	48.27	7.95E-01	-31.39	22.61	1.65E-01	-9.90	32.10	7.58E-01
cg26996703	9.68	4.51	3.18E-02	16.08	7.07	2.29E-02	-4.94	11.09	6.56E-01	7.48	13.17	5.70E-01	9.91	8.09	2.21E-01
cg26562772	6.91	3.22	3.18E-02	7.17	4.93	1.46E-01	8.91	7.78	2.53E-01	6.47	6.17	2.94E-01	4.38	8.87	6.22E-01
cg03722472	18.72	8.72	3.18E-02	11.90	14.43	4.10E-01	26.45	17.37	1.28E-01	16.08	20.82	4.40E-01	23.55	19.12	2.18E-01
cg15008124	13.64	6.36	3.18E-02	19.07	17.74	2.82E-01	41.90	27.50	1.28E-01	13.10	7.26	7.11E-02	-20.71	27.84	4.57E-01
cg04129227	-30.82	14.36	3.18E-02	1.95	40.28	9.61E-01	26.02	244.10	9.15E-01	-35.24	15.94	2.71E-02	-44.14	59.36	4.57E-01
cg26810908	-8.80	4.10	3.18E-02	-11.45	9.18	2.12E-01	11.08	14.07	4.31E-01	-9.54	5.33	7.34E-02	-14.53	11.60	2.10E-01
cg03174933	-13.79	6.42	3.18E-02	-12.35	11.26	2.73E-01	6.12	16.68	7.14E-01	-23.87	7.55	1.56E-03	-2.31	15.98	8.85E-01
cg26912671	15.01	6.99	3.18E-02	24.00	18.46	1.94E-01	-0.22	29.11	9.94E-01	15.55	8.20	5.80E-02	3.92	25.93	8.80E-01
cg20689978	-16.60	7.73	3.18E-02	-13.97	12.96	2.81E-01	-32.81	18.16	7.07E-02	-3.91	13.28	7.68E-01	-35.31	22.04	1.09E-01
cg04229842	21.78	10.15	3.18E-02	19.46	11.89	1.02E-01	29.27	36.60	4.24E-01	48.25	63.73	4.49E-01	24.38	24.63	3.22E-01
cg14805932	15.38	7.17	3.18E-02	5.42	16.93	7.49E-01	32.12	17.66	6.90E-02	20.21	11.89	8.91E-02	6.09	13.24	6.45E-01
cg00043564	10.07	4.69	3.18E-02	11.75	7.38	1.11E-01	17.00	14.06	2.26E-01	5.53	9.98	5.79E-01	8.37	9.14	3.60E-01
cg09977288	-5.41	2.52	3.18E-02	-3.65	4.94	4.60E-01	0.75	7.33	9.18E-01	-7.47	3.75	4.62E-02	-6.89	6.11	2.60E-01
cg05172940	12.43	5.79	3.18E-02	13.46	8.10	9.64E-02	7.85	12.64	5.35E-01	4.87	19.74	8.05E-01	18.09	13.21	1.71E-01
cg23075968	9.14	4.26	3.18E-02	13.22	5.75	2.15E-02	12.60	9.37	1.79E-01	-9.35	11.25	4.06E-01	9.23	8.40	2.71E-01
cg09389557	-14.76	6.88	3.18E-02	-25.60	18.26	1.61E-01	13.33	24.54	5.87E-01	-17.55	8.11	3.04E-02	7.64	28.13	7.86E-01
cg02806156	4.76	2.22	3.18E-02	5.00	2.93	8.78E-02	8.32	5.49	1.29E-01	3.06	5.87	6.03E-01	0.84	6.34	8.94E-01
cg18088056	18.35	8.55	3.18E-02	11.40	13.47	3.97E-01	40.41	16.84	1.64E-02	9.32	18.92	6.22E-01	10.64	23.25	6.47E-01
cg04956382	-9.93	4.63	3.18E-02	-16.65	8.98	6.38E-02	-12.94	13.31	3.31E-01	-5.37	6.51	4.09E-01	-11.37	14.05	4.18E-01
cg01432055	-8.41	3.92	3.18E-02	-8.62	7.99	2.81E-01	-28.20	29.25	3.35E-01	-6.74	4.92	1.71E-01	-14.53	11.95	2.24E-01
cg07918846	37.00	17.24	3.18E-02	37.96	18.94	4.51E-02	25.93	35.61	4.67E-01	9.00	15.53	5.62E-01	94.90	30.45	1.83E-03

cg16818725	5.06	2.36	3.18E-02	4.74	3.35	1.57E-01	3.74	5.24	4.76E-01	11.86	7.44	1.11E-01	3.79	5.23	4.69E-01
cg01663570	-28.85	13.44	3.19E-02	-26.25	19.21	1.72E-01	-72.99	37.72	5.30E-02	-24.43	29.99	4.15E-01	-9.98	31.46	7.51E-01
cg10319904	-41.28	19.23	3.19E-02	-55.39	22.94	1.58E-02	-58.11	89.06	5.14E-01	-4.30	66.57	9.48E-01	4.34	47.07	9.26E-01
cg16378063	4.56	2.12	3.19E-02	4.09	2.84	1.51E-01	9.08	4.62	4.96E-02	5.09	5.99	3.96E-01	-2.58	6.51	6.92E-01
cg01956154	-19.53	9.10	3.19E-02	-3.52	9.63	7.15E-01	-23.64	18.56	2.03E-01	-45.75	20.87	2.84E-02	-26.91	14.87	7.03E-02
cg00415993	7.19	3.35	3.19E-02	6.47	4.46	1.47E-01	15.12	8.29	6.81E-02	13.00	8.75	1.38E-01	-3.55	8.28	6.68E-01
cg02474926	14.68	6.84	3.19E-02	27.59	17.58	1.17E-01	20.59	33.81	5.43E-01	10.67	8.02	1.84E-01	23.51	24.02	3.28E-01
cg22188058	41.05	19.13	3.19E-02	-8.34	53.03	8.75E-01	26.39	200.64	8.95E-01	52.93	21.95	1.59E-02	16.87	59.95	7.78E-01
cg19624299	8.87	4.14	3.19E-02	18.04	7.49	1.60E-02	7.67	6.84	2.62E-01	2.28	8.82	7.96E-01	0.63	12.48	9.60E-01
cg13602242	-28.10	13.09	3.19E-02	-14.90	14.96	3.19E-01	-58.63	38.91	1.32E-01	-101.95	54.51	6.14E-02	-30.17	26.17	2.49E-01
cg10075260	14.11	6.57	3.19E-02	21.87	16.88	1.95E-01	11.38	39.01	7.71E-01	10.62	7.58	1.61E-01	36.75	25.34	1.47E-01
cg25641145	-9.57	4.46	3.19E-02	-6.95	5.76	2.28E-01	-3.13	7.31	6.69E-01	-5.23	13.34	6.95E-01	-23.03	8.03	4.12E-03
cg02851047	16.75	7.80	3.19E-02	4.37	13.74	7.51E-01	17.58	13.41	1.90E-01	31.97	18.55	8.47E-02	23.02	19.40	2.35E-01
cg00565348	15.29	7.13	3.19E-02	2.13	11.30	8.51E-01	18.35	18.70	3.26E-01	16.25	19.54	4.05E-01	29.69	12.52	1.77E-02
cg10446433	-11.55	5.38	3.19E-02	-13.55	14.06	3.35E-01	-40.05	25.80	1.21E-01	-8.29	6.22	1.83E-01	-26.25	21.70	2.26E-01
cg15737554	13.84	6.45	3.19E-02	22.55	17.51	1.98E-01	12.92	22.68	5.69E-01	14.25	7.62	6.14E-02	-6.98	24.83	7.79E-01
cg09714114	-13.13	6.12	3.19E-02	-35.85	16.31	2.79E-02	-8.92	31.06	7.74E-01	-10.27	7.03	1.44E-01	0.73	24.45	9.76E-01
cg23912081	8.16	3.80	3.19E-02	4.75	4.49	2.90E-01	11.95	6.78	7.81E-02	24.77	10.65	2.00E-02	2.38	6.79	7.26E-01
cg00041989	8.21	3.82	3.19E-02	9.32	5.23	7.51E-02	9.01	8.94	3.14E-01	13.28	9.78	1.74E-01	-3.43	10.59	7.46E-01
cg13153394	-40.11	18.69	3.19E-02	-79.72	53.08	1.33E-01	321.57	277.14	2.46E-01	-40.16	20.96	5.54E-02	3.44	67.91	9.60E-01
cg18030453	11.56	5.39	3.19E-02	12.85	7.57	8.96E-02	13.12	13.00	3.13E-01	7.81	12.94	5.46E-01	9.74	13.99	4.86E-01
cg09010323	-20.89	9.74	3.19E-02	-31.75	13.77	2.11E-02	-14.28	25.75	5.79E-01	-2.25	23.81	9.25E-01	-13.68	22.36	5.41E-01
cg05028187	-19.03	8.87	3.19E-02	-34.36	22.09	1.20E-01	-75.95	76.35	3.20E-01	-14.41	10.17	1.57E-01	-23.30	34.81	5.03E-01
cg24029414	33.59	15.65	3.19E-02	25.50	19.34	1.87E-01	-19.34	72.22	7.89E-01	12.15	57.38	8.32E-01	75.55	33.11	2.25E-02
cg07443717	-14.25	6.64	3.19E-02	-27.77	16.14	8.53E-02	-0.31	35.78	9.93E-01	-13.41	7.85	8.74E-02	0.89	23.54	9.70E-01
cg08797242	22.42	10.45	3.19E-02	25.61	12.51	4.06E-02	70.76	35.40	4.56E-02	-5.04	57.19	9.30E-01	3.32	19.51	8.65E-01
cg05098343	-9.21	4.29	3.19E-02	-12.14	7.30	9.63E-02	-9.00	10.19	3.77E-01	-4.18	8.34	6.16E-01	-10.88	9.32	2.43E-01
cg25376491	10.11	4.71	3.19E-02	5.90	8.27	4.76E-01	7.61	10.81	4.81E-01	10.90	7.63	1.53E-01	24.99	14.63	8.76E-02
cg10334885	13.19	6.15	3.19E-02	15.88	8.25	5.41E-02	9.41	9.89	3.41E-01	-2.03	13.25	8.78E-01	37.86	17.14	2.72E-02
cg16932017	30.28	14.11	3.19E-02	18.51	18.93	3.28E-01	4.61	42.78	9.14E-01	30.00	46.77	5.21E-01	68.54	28.54	1.63E-02
cg03655395	8.27	3.85	3.19E-02	8.61	6.26	1.69E-01	3.74	6.37	5.57E-01	25.85	11.65	2.65E-02	5.53	10.13	5.85E-01
cg01698108	-33.72	15.72	3.19E-02	-45.22	51.20	3.77E-01	86.33	203.34	6.71E-01	-29.33	17.37	9.14E-02	-73.60	55.22	1.83E-01
cg14794487	-14.86	6.93	3.19E-02	-26.28	20.30	1.95E-01	25.96	38.74	5.03E-01	-14.82	7.88	5.99E-02	-15.00	24.87	5.46E-01
cg04120407	35.35	16.48	3.19E-02	44.25	19.84	2.57E-02	133.45	87.54	1.27E-01	-9.39	63.30	8.82E-01	3.47	36.24	9.24E-01
cg06631310	12.16	5.67	3.19E-02	8.79	8.49	3.01E-01	30.27	13.10	2.08E-02	1.30	13.21	9.22E-01	12.76	13.25	3.35E-01

cg20659941	-8.33	3.88	3.19E-02	-3.36	7.52	6.55E-01	-9.34	9.36	3.18E-01	-10.88	5.75	5.83E-02	-8.19	11.99	4.94E-01
cg26968203	-38.40	17.90	3.19E-02	-38.27	25.62	1.35E-01	11.95	77.71	8.78E-01	-17.99	81.91	8.26E-01	-47.44	27.93	8.94E-02
cg13891702	-24.00	11.19	3.19E-02	-14.27	10.49	1.74E-01	-61.50	17.63	4.84E-04	-1.09	23.62	9.63E-01	-18.98	13.40	1.57E-01
cg22240717	5.83	2.72	3.19E-02	4.50	3.66	2.18E-01	6.32	6.14	3.03E-01	15.22	11.31	1.78E-01	6.34	6.18	3.06E-01
cg00055341	9.22	4.30	3.19E-02	4.44	6.17	4.72E-01	23.89	10.74	2.61E-02	14.02	9.49	1.40E-01	3.28	10.06	7.45E-01
cg13105904	-4.60	2.15	3.19E-02	-6.37	3.06	3.72E-02	-8.86	5.96	1.37E-01	-0.76	5.26	8.84E-01	-0.91	4.65	8.45E-01
cg15675123	-9.22	4.30	3.19E-02	-16.49	11.54	1.53E-01	-10.07	15.66	5.20E-01	-8.12	5.08	1.10E-01	-5.16	16.24	7.51E-01
cg19855559	-8.40	3.92	3.19E-02	-9.38	8.03	2.43E-01	-7.75	10.50	4.60E-01	-4.89	5.41	3.66E-01	-25.33	12.39	4.09E-02
cg24678869	15.52	7.24	3.19E-02	24.39	10.80	2.39E-02	12.77	13.97	3.60E-01	4.82	21.43	8.22E-01	3.48	17.65	8.44E-01
cg24202916	-9.30	4.34	3.20E-02	-8.89	6.75	1.88E-01	-1.52	10.01	8.79E-01	-3.18	9.36	7.34E-01	-22.47	9.17	1.42E-02
cg15500841	-7.19	3.35	3.20E-02	-13.28	6.46	3.97E-02	4.35	12.16	7.21E-01	-6.10	4.51	1.76E-01	-5.57	10.50	5.96E-01
cg12459028	-10.36	4.83	3.20E-02	-15.37	6.79	2.37E-02	-5.11	11.18	6.48E-01	-5.50	19.82	7.81E-01	-5.27	9.70	5.87E-01
cg23674788	20.61	9.61	3.20E-02	13.77	15.16	3.64E-01	27.40	21.36	2.00E-01	7.36	21.57	7.33E-01	40.84	21.61	5.88E-02
cg05264870	8.84	4.12	3.20E-02	13.36	5.02	7.81E-03	12.97	8.35	1.20E-01	7.35	9.40	4.34E-01	-5.69	9.02	5.28E-01
cg00096419	37.09	17.29	3.20E-02	57.99	21.83	7.90E-03	53.71	44.21	2.24E-01	5.51	14.93	7.12E-01	61.79	33.96	6.88E-02
cg03761885	10.15	4.73	3.20E-02	17.62	9.66	6.82E-02	3.92	17.42	8.22E-01	5.70	6.38	3.71E-01	18.39	12.86	1.53E-01
cg07015511	20.58	9.59	3.20E-02	12.57	16.63	4.50E-01	14.01	19.01	4.61E-01	28.02	19.39	1.48E-01	35.60	23.44	1.29E-01
cg13763952	22.47	10.48	3.20E-02	15.85	16.02	3.22E-01	55.76	24.49	2.28E-02	7.98	22.10	7.18E-01	22.44	25.86	3.86E-01
cg05364326	30.29	14.12	3.20E-02	89.81	40.09	2.51E-02	-36.47	135.93	7.88E-01	21.89	15.89	1.68E-01	29.92	51.63	5.62E-01
cg13679842	-9.08	4.23	3.20E-02	-17.86	8.97	4.64E-02	-10.73	17.87	5.48E-01	-9.25	5.51	9.36E-02	7.19	11.64	5.37E-01
cg12308631	26.90	12.54	3.20E-02	35.74	19.02	6.03E-02	2.46	19.01	8.97E-01	16.69	18.56	3.68E-01	67.59	26.15	9.74E-03
cg20228119	39.14	18.25	3.20E-02	109.61	57.39	5.61E-02	-95.41	225.76	6.73E-01	32.11	20.49	1.17E-01	32.36	58.02	5.77E-01
cg02557755	49.07	22.88	3.20E-02	92.73	48.51	5.59E-02	-92.51	249.29	7.11E-01	37.86	28.12	1.78E-01	38.77	70.11	5.80E-01
cg08901157	-8.90	4.15	3.20E-02	-5.49	5.95	3.56E-01	-14.86	11.17	1.83E-01	-17.32	9.55	6.97E-02	-4.85	9.64	6.15E-01
cg08352115	9.59	4.47	3.20E-02	14.46	6.55	2.72E-02	5.76	9.97	5.63E-01	14.18	9.58	1.39E-01	-7.79	11.92	5.13E-01
cg26378593	23.90	11.15	3.20E-02	25.92	37.35	4.88E-01	-1.47	134.75	9.91E-01	27.67	12.05	2.16E-02	-43.66	50.97	3.92E-01
cg16361947	13.54	6.31	3.20E-02	0.54	16.27	9.74E-01	11.35	22.64	6.16E-01	19.67	6.10	1.26E-03	-14.18	21.40	5.07E-01
cg18594332	-11.14	5.20	3.20E-02	4.26	11.14	7.02E-01	-20.44	17.61	2.46E-01	-13.64	6.76	4.36E-02	-21.40	16.13	1.85E-01
cg23340017	17.35	8.09	3.20E-02	20.65	14.69	1.60E-01	24.07	22.15	2.77E-01	17.24	17.01	3.11E-01	11.80	13.95	3.97E-01
cg12873037	-48.21	22.48	3.20E-02	-39.43	29.84	1.86E-01	99.17	105.70	3.48E-01	-101.31	49.29	3.98E-02	-49.89	41.89	2.34E-01
cg09837648	19.90	9.28	3.20E-02	9.90	19.66	6.15E-01	21.74	15.01	1.48E-01	7.02	16.95	6.79E-01	56.92	24.56	2.05E-02
cg12080675	11.89	5.55	3.20E-02	24.92	15.22	1.02E-01	39.57	30.72	1.98E-01	12.80	7.20	7.57E-02	-1.20	11.28	9.15E-01
cg03293015	15.56	7.26	3.20E-02	31.79	10.67	2.88E-03	3.88	16.14	8.10E-01	17.55	17.06	3.04E-01	5.31	10.11	5.99E-01
cg04340258	6.11	2.85	3.20E-02	3.73	3.46	2.81E-01	14.91	7.99	6.19E-02	8.59	9.10	3.45E-01	8.80	9.25	3.42E-01
cg03432176	6.66	3.10	3.20E-02	7.63	4.34	7.86E-02	5.63	6.49	3.86E-01	17.43	12.81	1.74E-01	2.18	6.93	7.52E-01

cg19503977	18.39	8.58	3.20E-02	27.60	13.63	4.28E-02	19.93	21.06	3.44E-01	32.81	23.84	1.69E-01	-0.31	15.44	9.84E-01
cg14908170	9.15	4.27	3.20E-02	6.78	6.27	2.80E-01	13.66	8.66	1.15E-01	5.17	15.21	7.34E-01	10.63	9.21	2.49E-01
cg22214498	18.38	8.57	3.20E-02	18.79	13.96	1.78E-01	4.23	15.91	7.91E-01	26.02	19.91	1.91E-01	35.62	22.35	1.11E-01
cg27158308	25.75	12.01	3.20E-02	34.93	30.70	2.55E-01	93.60	127.04	4.61E-01	30.59	9.97	2.15E-03	-40.35	40.19	3.15E-01
cg16611584	5.24	2.45	3.20E-02	8.47	3.37	1.21E-02	4.69	5.42	3.87E-01	1.66	7.61	8.27E-01	-1.99	5.98	7.39E-01
cg09284275	6.13	2.86	3.20E-02	6.05	3.57	9.03E-02	15.91	7.97	4.59E-02	-0.47	8.79	9.58E-01	2.03	8.16	8.03E-01
cg08520742	19.60	9.14	3.20E-02	32.06	15.98	4.48E-02	16.31	16.06	3.10E-01	6.81	21.73	7.54E-01	15.22	22.10	4.91E-01
cg01294253	18.52	8.64	3.21E-02	1.92	16.39	9.07E-01	29.22	15.73	6.33E-02	16.71	15.64	2.85E-01	35.26	25.40	1.65E-01
cg02543879	26.88	12.54	3.21E-02	41.32	16.88	1.44E-02	-0.63	29.21	9.83E-01	26.56	36.46	4.66E-01	7.32	32.81	8.23E-01
cg16854917	11.99	5.59	3.21E-02	21.31	16.67	2.01E-01	53.33	68.46	4.36E-01	9.69	6.32	1.25E-01	16.79	17.83	3.46E-01
cg03264729	-12.55	5.85	3.21E-02	-40.95	18.24	2.48E-02	-0.10	21.48	9.96E-01	-10.94	6.00	6.83E-02	0.44	25.10	9.86E-01
cg17221584	-4.25	1.98	3.21E-02	-3.45	2.89	2.33E-01	-5.90	4.05	1.45E-01	4.56	7.28	5.31E-01	-7.16	4.26	9.23E-02
cg12057158	-10.57	4.93	3.21E-02	-25.15	10.77	1.95E-02	-16.01	22.16	4.70E-01	-6.01	6.06	3.21E-01	-6.64	17.55	7.05E-01
cg23109649	-21.10	9.84	3.21E-02	-31.45	10.76	3.47E-03	19.29	25.94	4.57E-01	-24.78	50.88	6.26E-01	-17.92	18.65	3.37E-01
cg02467956	13.73	6.40	3.21E-02	8.79	9.24	3.41E-01	14.20	13.76	3.02E-01	18.20	15.90	2.52E-01	24.73	17.10	1.48E-01
cg07315006	-13.58	6.34	3.21E-02	-5.11	15.80	7.46E-01	0.30	29.63	9.92E-01	-18.52	7.50	1.35E-02	5.85	22.58	7.96E-01
cg22035959	-13.34	6.22	3.21E-02	-3.55	9.59	7.11E-01	-23.99	15.82	1.29E-01	-27.19	16.58	1.01E-01	-15.19	11.70	1.94E-01
cg14040679	36.00	16.80	3.21E-02	62.21	21.39	3.64E-03	-2.11	31.10	9.46E-01	3.60	47.95	9.40E-01	43.27	36.97	2.42E-01
cg05547777	15.49	7.23	3.21E-02	22.54	14.45	1.19E-01	21.95	27.82	4.30E-01	14.92	10.60	1.59E-01	6.58	15.52	6.71E-01
cg07289618	-14.27	6.66	3.21E-02	-19.09	10.50	6.91E-02	-5.67	13.97	6.85E-01	-25.75	16.21	1.12E-01	-4.78	14.80	7.47E-01
cg22579841	11.82	5.51	3.21E-02	16.98	9.33	6.89E-02	13.21	11.82	2.64E-01	-0.22	9.93	9.82E-01	24.66	15.59	1.14E-01
cg25330843	18.34	8.56	3.21E-02	42.38	19.06	2.62E-02	12.62	24.70	6.09E-01	13.42	12.85	2.97E-01	9.93	17.65	5.74E-01
cg18046311	-13.90	6.49	3.21E-02	-6.77	11.92	5.70E-01	11.14	24.36	6.47E-01	-20.61	8.49	1.51E-02	-13.15	29.40	6.55E-01
cg16968662	18.31	8.54	3.21E-02	20.94	15.02	1.63E-01	6.14	15.37	6.89E-01	25.30	20.00	2.06E-01	27.10	19.84	1.72E-01
cg17187287	13.08	6.10	3.21E-02	26.76	15.06	7.56E-02	14.69	18.90	4.37E-01	11.78	8.94	1.88E-01	6.27	11.83	5.96E-01
cg03227128	32.21	15.03	3.21E-02	7.72	16.03	6.30E-01	75.85	32.17	1.84E-02	26.37	29.94	3.78E-01	48.36	26.16	6.45E-02
cg01372811	-10.05	4.69	3.21E-02	-11.93	6.67	7.36E-02	-9.99	10.77	3.54E-01	8.19	14.37	5.69E-01	-14.95	10.25	1.45E-01
cg04989278	13.00	6.06	3.21E-02	14.67	8.43	8.18E-02	15.40	14.76	2.97E-01	17.41	23.01	4.49E-01	6.54	12.28	5.94E-01
cg05459517	-8.67	4.05	3.21E-02	-6.98	7.39	3.45E-01	-11.14	11.57	3.36E-01	-6.68	6.48	3.03E-01	-13.94	9.35	1.36E-01
cg02188185	5.32	2.48	3.21E-02	4.12	3.28	2.09E-01	11.86	4.89	1.52E-02	-2.53	6.83	7.11E-01	5.40	6.13	3.78E-01
cg26687119	13.51	6.30	3.21E-02	5.86	6.23	3.47E-01	5.84	10.18	5.66E-01	42.16	15.39	6.17E-03	16.58	8.64	5.50E-02
cg05381287	-10.20	4.76	3.21E-02	-17.84	10.40	8.62E-02	4.41	18.11	8.08E-01	-9.31	6.14	1.30E-01	-9.73	13.66	4.76E-01
cg17174893	15.39	7.18	3.21E-02	21.46	11.93	7.19E-02	16.45	14.55	2.58E-01	16.41	14.28	2.51E-01	-3.91	19.15	8.38E-01
cg17875102	-8.53	3.98	3.21E-02	-9.05	5.60	1.06E-01	-13.18	10.31	2.01E-01	-9.37	10.26	3.61E-01	-2.97	9.02	7.42E-01
cg05273053	15.31	7.15	3.21E-02	46.08	22.73	4.27E-02	35.65	29.49	2.27E-01	9.63	8.01	2.29E-01	21.37	32.98	5.17E-01

cg12405599	4.32	2.02	3.21E-02	4.59	3.05	1.32E-01	2.98	4.43	5.01E-01	5.37	3.88	1.67E-01	2.91	6.85	6.71E-01
cg16177732	17.10	7.98	3.21E-02	20.70	12.75	1.04E-01	6.57	18.22	7.18E-01	34.58	23.46	1.40E-01	12.40	14.57	3.95E-01
cg09328979	-10.16	4.74	3.21E-02	-21.41	8.26	9.51E-03	1.02	13.20	9.38E-01	-3.45	8.17	6.73E-01	-10.38	8.67	2.31E-01
cg02061130	8.03	3.75	3.21E-02	11.25	5.31	3.40E-02	4.32	7.90	5.85E-01	-3.34	12.12	7.83E-01	9.75	8.81	2.68E-01
cg03437540	11.83	5.52	3.21E-02	11.93	7.29	1.02E-01	30.78	12.80	1.62E-02	2.31	13.19	8.61E-01	2.63	12.10	8.28E-01
cg19279257	7.99	3.73	3.21E-02	12.31	5.10	1.58E-02	12.25	7.01	8.04E-02	1.53	10.26	8.82E-01	-3.17	8.15	6.98E-01
cg25289457	-16.22	7.57	3.21E-02	-0.71	20.77	9.73E-01	-58.90	40.75	1.48E-01	-19.47	8.66	2.46E-02	11.38	28.91	6.94E-01
cg10190898	-22.64	10.57	3.21E-02	-39.60	14.88	7.80E-03	-32.59	18.89	8.45E-02	-3.64	8.44	6.67E-01	-30.56	23.12	1.86E-01
cg13375589	-2.65	1.24	3.21E-02	-1.99	1.76	2.58E-01	-2.96	2.39	2.17E-01	-1.20	5.02	8.11E-01	-4.44	2.90	1.26E-01
cg02637843	-9.21	4.30	3.22E-02	-14.75	11.18	1.87E-01	-0.40	15.04	9.79E-01	-11.40	5.26	3.02E-02	6.06	13.42	6.52E-01
cg12721804	17.70	8.26	3.22E-02	26.24	14.32	6.69E-02	8.07	15.20	5.96E-01	20.58	17.68	2.44E-01	13.63	21.12	5.19E-01
cg26644395	-6.54	3.05	3.22E-02	-8.38	5.60	1.34E-01	-2.03	6.38	7.50E-01	-9.94	5.20	5.57E-02	-1.15	8.53	8.93E-01
cg22340475	-12.53	5.85	3.22E-02	-22.62	8.71	9.39E-03	-1.01	14.27	9.43E-01	-4.71	4.81	3.27E-01	-23.55	10.61	2.65E-02
cg18434712	16.07	7.50	3.22E-02	10.34	12.09	3.93E-01	30.05	21.00	1.52E-01	22.11	13.71	1.07E-01	8.72	17.25	6.13E-01
cg07547549	11.34	5.29	3.22E-02	6.44	7.82	4.11E-01	3.21	13.57	8.13E-01	25.55	13.04	5.01E-02	16.36	11.13	1.42E-01
cg19949797	-13.79	6.44	3.22E-02	-5.53	15.25	7.17E-01	-12.78	33.66	7.04E-01	-15.00	7.79	5.43E-02	-20.52	20.13	3.08E-01
cg01488300	10.17	4.75	3.22E-02	10.39	6.82	1.28E-01	22.58	12.95	8.12E-02	2.90	10.31	7.79E-01	8.79	11.55	4.47E-01
cg09356442	21.89	10.22	3.22E-02	33.35	16.81	4.73E-02	29.73	41.20	4.71E-01	19.07	15.72	2.25E-01	-2.12	26.67	9.37E-01
cg03411270	-25.69	11.99	3.22E-02	-63.93	51.62	2.16E-01	-124.77	164.65	4.49E-01	-26.20	12.77	4.01E-02	26.30	49.54	5.96E-01
cg18055557	-10.60	4.95	3.22E-02	-5.92	10.42	5.70E-01	-14.29	13.89	3.04E-01	-8.33	7.05	2.37E-01	-21.52	12.54	8.61E-02
cg14494204	5.51	2.57	3.22E-02	7.59	3.65	3.75E-02	4.22	6.02	4.83E-01	1.52	9.62	8.75E-01	3.45	5.15	5.03E-01
cg01961547	-11.51	5.37	3.22E-02	-25.54	11.01	2.03E-02	-18.78	18.02	2.97E-01	-8.48	5.53	1.25E-01	9.30	18.42	6.14E-01
cg11262093	32.67	15.25	3.22E-02	31.33	21.36	1.42E-01	25.25	51.54	6.24E-01	23.99	32.33	4.58E-01	50.85	35.97	1.58E-01
cg25074426	15.21	7.10	3.22E-02	22.99	13.27	8.32E-02	17.99	16.73	2.82E-01	14.04	13.52	2.99E-01	5.89	13.98	6.74E-01
cg18395355	16.76	7.82	3.22E-02	16.79	12.99	1.96E-01	49.37	26.64	6.39E-02	6.75	14.38	6.39E-01	17.32	15.50	2.64E-01
cg25245082	20.64	9.64	3.22E-02	25.72	12.54	4.02E-02	13.99	26.68	6.00E-01	57.89	34.08	8.94E-02	-5.04	21.60	8.15E-01
cg23000734	5.32	2.48	3.22E-02	3.71	3.65	3.10E-01	5.19	5.97	3.85E-01	7.89	5.62	1.60E-01	6.86	6.02	2.54E-01
cg21290313	-11.47	5.36	3.22E-02	-21.99	12.51	7.88E-02	-4.99	19.57	7.99E-01	-6.27	6.81	3.57E-01	-25.97	15.29	8.93E-02
cg09237846	25.73	12.01	3.22E-02	31.56	19.25	1.01E-01	16.62	45.14	7.13E-01	58.56	33.38	7.94E-02	11.40	18.75	5.43E-01
cg03633004	18.87	8.81	3.22E-02	26.57	18.80	1.58E-01	28.81	21.07	1.72E-01	12.70	12.49	3.09E-01	15.57	26.82	5.62E-01
cg22853542	-12.00	5.60	3.22E-02	-11.73	16.33	4.73E-01	-16.10	33.82	6.34E-01	-11.77	6.27	6.04E-02	-13.89	23.77	5.59E-01
cg26381210	6.65	3.10	3.22E-02	8.04	4.06	4.77E-02	8.87	7.32	2.25E-01	0.53	11.98	9.65E-01	1.87	7.59	8.05E-01
cg06577876	-9.89	4.62	3.22E-02	-2.60	9.15	7.76E-01	-16.10	16.65	3.33E-01	-11.61	6.35	6.72E-02	-13.23	12.38	2.85E-01
cg16910340	26.78	12.50	3.22E-02	-2.34	28.47	9.35E-01	-51.20	74.36	4.91E-01	39.65	14.85	7.58E-03	12.02	43.90	7.84E-01
cg12655190	15.46	7.22	3.22E-02	5.09	20.91	8.08E-01	26.50	33.82	4.33E-01	17.39	8.66	4.48E-02	11.19	19.24	5.61E-01

cg19529273	12.25	5.72	3.22E-02	14.60	8.98	1.04E-01	10.77	13.01	4.08E-01	8.74	10.59	4.09E-01	15.50	17.30	3.70E-01
cg25925006	13.37	6.24	3.22E-02	10.51	8.39	2.10E-01	13.22	23.51	5.74E-01	66.42	41.77	1.12E-01	14.53	10.50	1.66E-01
cg11183935	-8.92	4.16	3.22E-02	-13.86	5.96	2.00E-02	-11.16	11.57	3.35E-01	-2.67	11.19	8.11E-01	-1.35	8.44	8.73E-01
cg25188395	14.44	6.74	3.22E-02	17.70	14.63	2.26E-01	27.75	16.85	9.96E-02	12.61	11.71	2.81E-01	6.94	12.40	5.76E-01
cg13876878	-9.75	4.55	3.22E-02	-7.66	8.39	3.61E-01	-7.04	10.22	4.91E-01	-6.26	7.58	4.09E-01	-26.23	11.90	2.75E-02
cg04157865	6.39	2.98	3.22E-02	6.33	3.87	1.02E-01	11.13	7.77	1.52E-01	6.22	8.34	4.56E-01	1.48	8.25	8.58E-01
cg01575930	-5.87	2.74	3.23E-02	-8.93	4.28	3.68E-02	-6.20	7.25	3.92E-01	-3.63	5.26	4.90E-01	-1.88	6.55	7.74E-01
cg11585670	-9.45	4.41	3.23E-02	-9.10	9.76	3.51E-01	4.10	13.52	7.62E-01	-10.86	5.79	6.05E-02	-15.91	13.47	2.38E-01
cg16919771	34.97	16.33	3.23E-02	50.11	21.64	2.06E-02	-10.16	38.54	7.92E-01	85.59	50.45	8.98E-02	15.80	31.45	6.15E-01
cg02367316	5.16	2.41	3.23E-02	4.65	3.54	1.88E-01	9.12	4.95	6.56E-02	3.88	8.15	6.34E-01	2.37	5.24	6.51E-01
cg27094856	23.00	10.74	3.23E-02	20.67	25.47	4.17E-01	122.37	99.44	2.18E-01	25.46	13.03	5.07E-02	4.53	29.69	8.79E-01
cg26625319	40.37	18.86	3.23E-02	28.88	23.67	2.22E-01	95.45	77.14	2.16E-01	69.03	47.04	1.42E-01	36.21	49.50	4.64E-01
cg16850192	-15.01	7.01	3.23E-02	-21.74	17.91	2.25E-01	0.74	32.77	9.82E-01	-9.77	8.43	2.47E-01	-44.84	21.06	3.32E-02
cg13455759	-10.34	4.83	3.23E-02	-6.24	11.69	5.94E-01	-1.06	16.96	9.50E-01	-10.02	6.32	1.13E-01	-20.27	11.90	8.86E-02
cg25003047	-8.85	4.13	3.23E-02	-14.03	5.88	1.71E-02	3.97	10.26	6.99E-01	-10.04	14.23	4.80E-01	-6.63	8.11	4.13E-01
cg12461092	-9.49	4.43	3.23E-02	-7.20	8.11	3.75E-01	-4.61	11.02	6.76E-01	-12.32	7.30	9.15E-02	-12.00	10.71	2.63E-01
cg24458896	27.37	12.78	3.23E-02	30.02	16.72	7.26E-02	46.54	54.42	3.92E-01	-43.72	72.49	5.46E-01	26.16	22.27	2.40E-01
cg24561661	12.60	5.88	3.23E-02	12.85	12.28	2.96E-01	16.50	18.85	3.81E-01	9.33	8.10	2.49E-01	21.48	15.45	1.64E-01
cg05519658	14.49	6.77	3.23E-02	16.54	14.26	2.46E-01	6.86	25.22	7.86E-01	12.99	8.74	1.37E-01	23.99	21.06	2.55E-01
cg03925821	-24.51	11.45	3.23E-02	-10.99	17.01	5.18E-01	-2.24	32.26	9.45E-01	-20.05	9.15	2.85E-02	-68.43	22.79	2.68E-03
cg21356631	6.12	2.86	3.23E-02	4.50	3.93	2.53E-01	13.35	6.05	2.73E-02	2.96	9.88	7.64E-01	3.12	7.07	6.59E-01
cg07597892	-13.76	6.43	3.23E-02	-26.71	16.35	1.02E-01	2.73	27.58	9.21E-01	-12.67	7.78	1.03E-01	-10.45	19.57	5.93E-01
cg25721366	-45.08	21.06	3.23E-02	-62.04	35.08	7.70E-02	36.88	142.69	7.96E-01	-40.43	31.29	1.96E-01	-31.60	51.83	5.42E-01
cg17499324	16.69	7.80	3.23E-02	4.63	10.36	6.55E-01	36.80	14.49	1.11E-02	24.60	21.36	2.50E-01	12.61	16.46	4.44E-01
cg18641423	19.16	8.95	3.23E-02	13.01	16.19	4.22E-01	8.62	22.11	6.97E-01	14.02	7.59	6.47E-02	62.35	23.15	7.08E-03
cg25717464	10.53	4.92	3.23E-02	14.16	6.71	3.48E-02	7.21	12.04	5.49E-01	21.82	17.46	2.11E-01	-0.09	10.58	9.93E-01
cg27502457	10.56	4.93	3.23E-02	9.54	7.62	2.11E-01	16.40	11.52	1.55E-01	10.44	15.23	4.93E-01	8.41	9.11	3.56E-01
cg02888677	-43.63	20.38	3.23E-02	-90.76	38.50	1.84E-02	-54.54	184.23	7.67E-01	-25.68	26.64	3.35E-01	-20.44	58.25	7.26E-01
cg12836959	12.61	5.89	3.23E-02	16.73	10.80	1.22E-01	8.69	20.40	6.70E-01	16.80	9.20	6.79E-02	0.12	12.87	9.92E-01
cg25857569	-7.44	3.48	3.23E-02	-11.09	7.49	1.39E-01	-9.74	13.67	4.76E-01	-6.34	4.39	1.49E-01	-4.81	11.31	6.70E-01
cg24813588	16.18	7.56	3.23E-02	19.78	11.61	8.86E-02	17.09	14.65	2.43E-01	8.77	18.06	6.27E-01	12.73	20.60	5.36E-01
cg25957043	9.93	4.64	3.23E-02	17.69	9.74	6.93E-02	12.45	12.09	3.03E-01	7.11	6.24	2.55E-01	2.09	17.20	9.03E-01
cg08734527	-9.86	4.61	3.23E-02	-2.44	9.89	8.05E-01	-8.27	11.03	4.53E-01	-13.93	6.90	4.37E-02	-10.36	11.42	3.64E-01
cg25283284	23.18	10.83	3.23E-02	22.99	15.39	1.35E-01	14.50	28.83	6.15E-01	24.97	30.17	4.08E-01	27.78	22.33	2.13E-01
cg04705084	21.14	9.88	3.23E-02	31.11	20.75	1.34E-01	4.75	20.93	8.20E-01	15.51	15.97	3.32E-01	42.27	24.10	7.94E-02

cg07535628	13.47	6.29	3.23E-02	5.14	7.19	4.75E-01	32.28	11.95	6.90E-03	8.66	17.57	6.22E-01	14.08	12.07	2.43E-01
cg12557254	13.35	6.24	3.23E-02	32.26	17.40	6.38E-02	19.50	22.21	3.80E-01	11.44	7.25	1.15E-01	-14.64	26.95	5.87E-01
cg13223682	6.92	3.23	3.23E-02	4.03	4.69	3.91E-01	11.15	7.65	1.45E-01	15.78	7.27	3.00E-02	-0.69	8.39	9.34E-01
cg13514037	19.07	8.91	3.23E-02	10.65	17.18	5.35E-01	26.63	21.80	2.22E-01	17.79	12.88	1.67E-01	37.78	30.40	2.14E-01
cg14548380	17.12	8.00	3.23E-02	13.66	17.70	4.40E-01	1.80	30.87	9.54E-01	17.22	9.99	8.48E-02	36.18	27.00	1.80E-01
cg09629053	-12.22	5.71	3.23E-02	-23.73	20.00	2.35E-01	-29.21	67.95	6.67E-01	-10.18	6.15	9.80E-02	-26.10	25.46	3.05E-01
cg27420236	18.77	8.77	3.23E-02	30.06	14.38	3.66E-02	-17.64	26.58	5.07E-01	33.25	25.17	1.86E-01	13.76	13.91	3.23E-01
cg25416334	6.94	3.24	3.23E-02	6.28	4.67	1.78E-01	10.63	8.28	1.99E-01	2.46	7.92	7.56E-01	9.49	7.32	1.95E-01
cg15407517	23.93	11.18	3.23E-02	6.07	47.56	8.98E-01	19.24	66.59	7.73E-01	27.62	11.99	2.12E-02	-21.28	51.93	6.82E-01
cg10279055	23.70	11.07	3.23E-02	20.95	15.42	1.74E-01	-4.25	38.47	9.12E-01	4.37	27.47	8.74E-01	52.47	22.65	2.05E-02
cg10764907	30.26	14.14	3.23E-02	4.18	16.83	8.04E-01	44.12	19.95	2.70E-02	15.24	18.24	4.04E-01	70.99	24.34	3.54E-03
cg11130317	13.72	6.41	3.23E-02	22.87	18.05	2.05E-01	17.93	21.24	3.99E-01	12.44	9.09	1.71E-01	10.55	11.99	3.79E-01
cg04633683	-6.76	3.16	3.23E-02	-8.47	3.93	3.13E-02	-14.15	6.86	3.93E-02	2.55	9.22	7.82E-01	0.10	7.06	9.89E-01
cg07162060	-26.10	12.19	3.23E-02	-23.97	32.58	4.62E-01	-197.65	152.98	1.96E-01	-22.97	13.68	9.32E-02	-54.79	50.17	2.75E-01
cg07931785	-9.07	4.24	3.23E-02	-12.44	6.27	4.73E-02	-10.59	14.29	4.59E-01	-2.09	7.35	7.76E-01	-14.28	12.05	2.36E-01
cg10777851	-27.47	12.83	3.23E-02	-37.70	16.06	1.89E-02	-27.03	57.71	6.40E-01	-27.35	67.71	6.86E-01	-3.87	24.43	8.74E-01
cg27066155	22.46	10.49	3.23E-02	63.75	35.17	6.99E-02	-45.95	141.52	7.45E-01	21.66	11.36	5.66E-02	-27.89	46.03	5.45E-01
cg20140394	-5.22	2.44	3.23E-02	-3.21	3.94	4.15E-01	-5.62	5.52	3.08E-01	-8.48	5.07	9.47E-02	-4.91	5.61	3.82E-01
cg01578017	15.20	7.10	3.23E-02	23.04	10.61	2.98E-02	10.06	25.30	6.91E-01	32.14	26.05	2.17E-01	4.24	11.24	7.06E-01
cg03700218	-10.41	4.86	3.23E-02	-2.96	14.29	8.36E-01	-2.13	8.88	8.11E-01	4.39	44.82	9.22E-01	-16.56	6.43	1.00E-02
cg02193925	-8.43	3.94	3.23E-02	-6.44	6.96	3.55E-01	2.76	8.74	7.52E-01	-13.24	6.53	4.25E-02	-18.40	11.53	1.11E-01
cg12387859	56.09	26.21	3.24E-02	72.58	35.53	4.11E-02	125.72	109.61	2.51E-01	45.05	82.88	5.87E-01	16.41	47.95	7.32E-01
cg06602871	-12.46	5.82	3.24E-02	-22.75	9.90	2.16E-02	-14.55	12.70	2.52E-01	-1.60	11.57	8.90E-01	-5.94	13.31	6.55E-01
cg15929181	-11.11	5.19	3.24E-02	-14.91	9.41	1.13E-01	-19.11	19.49	3.27E-01	-7.28	7.13	3.07E-01	-14.46	16.97	3.94E-01
cg00296894	-42.54	19.88	3.24E-02	-21.57	26.39	4.14E-01	-27.35	112.09	8.07E-01	-110.84	50.39	2.78E-02	-49.65	40.11	2.16E-01
cg11199869	19.07	8.91	3.24E-02	3.09	23.25	8.94E-01	7.07	89.02	9.37E-01	23.26	11.72	4.72E-02	19.25	17.32	2.66E-01
cg10124287	-33.39	15.60	3.24E-02	-91.25	35.75	1.07E-02	-0.34	177.01	9.98E-01	-21.60	13.76	1.16E-01	-22.76	42.86	5.95E-01
cg12907379	20.28	9.48	3.24E-02	-14.45	29.31	6.22E-01	-7.80	54.14	8.85E-01	27.92	10.82	9.91E-03	6.41	30.27	8.32E-01
cg01108392	23.14	10.82	3.24E-02	28.66	33.74	3.96E-01	56.36	65.26	3.88E-01	22.90	12.56	6.83E-02	13.03	30.19	6.66E-01
cg26192309	-13.66	6.38	3.24E-02	-13.22	9.59	1.68E-01	-2.89	16.18	8.58E-01	-35.91	18.92	5.77E-02	-11.37	11.91	3.40E-01
cg19355263	-33.03	15.43	3.24E-02	-47.34	19.31	1.42E-02	-34.94	50.18	4.86E-01	5.47	58.84	9.26E-01	0.75	34.72	9.83E-01
cg05338969	22.64	10.58	3.24E-02	39.85	18.24	2.89E-02	13.95	28.86	6.29E-01	8.30	11.01	4.51E-01	54.66	28.96	5.91E-02
cg23957126	15.91	7.43	3.24E-02	31.82	21.06	1.31E-01	-14.31	25.47	5.74E-01	16.53	8.64	5.57E-02	18.61	33.42	5.78E-01
cg02049979	-14.04	6.56	3.24E-02	-18.66	13.37	1.63E-01	-23.49	16.55	1.56E-01	-16.42	10.81	1.29E-01	0.84	13.58	9.51E-01
cg27239280	8.07	3.77	3.24E-02	10.30	5.10	4.36E-02	9.18	8.97	3.06E-01	8.38	9.97	4.01E-01	-2.83	10.31	7.84E-01

cg02635932	6.38	2.98	3.24E-02	6.96	4.26	1.02E-01	5.47	7.32	4.54E-01	10.35	9.80	2.91E-01	4.39	5.94	4.60E-01
cg18614734	18.10	8.46	3.24E-02	31.06	9.68	1.34E-03	-6.77	14.09	6.31E-01	25.91	21.56	2.30E-01	18.96	11.29	9.32E-02
cg05967487	8.81	4.12	3.24E-02	12.29	7.63	1.07E-01	16.18	9.33	8.29E-02	2.00	6.45	7.56E-01	11.97	12.73	3.47E-01
cg17842918	4.95	2.31	3.24E-02	3.70	2.46	1.33E-01	10.71	3.92	6.24E-03	7.15	6.60	2.79E-01	-0.89	4.66	8.49E-01
cg14091497	13.55	6.33	3.24E-02	16.35	9.52	8.59E-02	6.07	11.51	5.98E-01	11.47	19.76	5.62E-01	21.74	16.27	1.82E-01
cg20857228	35.24	16.47	3.24E-02	-6.93	50.69	8.91E-01	-207.19	172.26	2.29E-01	44.52	17.50	1.09E-02	17.09	68.28	8.02E-01
cg03536474	15.00	7.01	3.24E-02	35.25	21.59	1.02E-01	53.78	32.87	1.02E-01	13.36	7.50	7.49E-02	0.13	15.93	9.94E-01
cg24378421	-6.34	2.96	3.24E-02	-10.64	4.20	1.13E-02	-1.64	6.99	8.14E-01	5.21	9.62	5.88E-01	-5.46	6.22	3.80E-01
cg03939485	34.22	16.00	3.24E-02	35.33	18.68	5.86E-02	71.38	72.85	3.27E-01	37.11	77.16	6.31E-01	18.69	38.20	6.25E-01
cg17747171	-44.50	20.80	3.24E-02	-49.23	26.79	6.61E-02	-131.55	75.57	8.17E-02	-14.71	58.90	8.03E-01	-15.39	46.89	7.43E-01
cg11719323	8.90	4.16	3.24E-02	1.41	11.47	9.02E-01	18.20	15.31	2.34E-01	11.39	4.93	2.10E-02	-8.86	14.47	5.40E-01
cg13043862	24.33	11.37	3.24E-02	42.68	13.29	1.32E-03	29.29	42.55	4.91E-01	12.53	32.72	7.02E-01	3.84	15.83	8.08E-01
cg08274380	14.70	6.87	3.24E-02	13.95	9.03	1.22E-01	-34.97	92.15	7.04E-01	15.36	12.82	2.31E-01	18.77	19.17	3.28E-01
cg01756381	14.32	6.70	3.24E-02	29.24	17.40	9.28E-02	15.47	25.75	5.48E-01	10.30	9.76	2.91E-01	13.07	11.96	2.74E-01
cg13449535	-7.42	3.47	3.24E-02	-3.81	4.88	4.36E-01	-9.84	8.93	2.71E-01	-10.12	10.39	3.30E-01	-12.41	7.19	8.45E-02
cg03700131	22.69	10.61	3.24E-02	21.24	13.05	1.04E-01	18.24	39.61	6.45E-01	71.10	57.82	2.19E-01	21.18	21.90	3.33E-01
cg01813335	9.25	4.33	3.24E-02	14.19	7.37	5.41E-02	4.04	10.38	6.97E-01	13.18	9.75	1.76E-01	3.74	8.11	6.44E-01
cg11497952	-24.63	11.51	3.24E-02	-1.86	27.51	9.46E-01	-170.69	105.97	1.07E-01	-29.82	14.50	3.97E-02	-19.11	26.95	4.78E-01
cg06793581	-22.92	10.71	3.24E-02	-9.10	15.13	5.48E-01	-24.61	24.74	3.20E-01	-23.96	28.51	4.01E-01	-61.01	26.01	1.90E-02
cg09539720	-11.25	5.26	3.24E-02	-8.91	7.35	2.26E-01	-18.32	13.70	1.81E-01	-12.82	12.60	3.09E-01	-10.58	12.91	4.13E-01
cg25950325	15.74	7.36	3.24E-02	23.88	24.09	3.22E-01	70.39	64.53	2.75E-01	15.94	8.14	5.03E-02	-5.61	26.64	8.33E-01
cg02847897	-5.87	2.74	3.24E-02	-4.75	3.21	1.39E-01	-0.18	5.13	9.72E-01	-6.97	10.79	5.18E-01	-13.74	5.22	8.51E-03
cg22545121	12.14	5.68	3.24E-02	10.29	8.39	2.20E-01	20.12	21.47	3.49E-01	21.69	11.24	5.37E-02	2.31	12.16	8.49E-01
cg04880804	-12.67	5.93	3.24E-02	-26.71	17.29	1.23E-01	8.55	21.52	6.91E-01	-13.05	6.79	5.47E-02	-5.62	27.63	8.39E-01
cg19420930	-13.57	6.34	3.25E-02	-24.90	7.93	1.68E-03	0.47	11.66	9.68E-01	-6.16	18.15	7.34E-01	-10.59	12.37	3.92E-01
cg23440520	18.18	8.50	3.25E-02	13.22	15.89	4.05E-01	25.50	18.45	1.67E-01	15.37	16.88	3.63E-01	20.51	17.07	2.29E-01
cg10901806	-10.59	4.95	3.25E-02	-15.82	7.51	3.52E-02	-6.03	11.15	5.88E-01	-6.60	14.20	6.42E-01	-6.98	9.95	4.83E-01
cg27640908	-25.59	11.97	3.25E-02	-11.11	17.53	5.26E-01	34.21	122.80	7.81E-01	-34.32	18.84	6.84E-02	-57.06	34.45	9.77E-02
cg13932794	-10.64	4.98	3.25E-02	-2.67	7.05	7.05E-01	-21.07	11.28	6.18E-02	-8.01	20.18	6.91E-01	-19.23	10.04	5.54E-02
cg02199826	-9.18	4.29	3.25E-02	-15.38	9.08	9.03E-02	-38.05	20.05	5.77E-02	-5.46	5.16	2.90E-01	-6.34	11.74	5.89E-01
cg03333330	18.38	8.60	3.25E-02	22.62	12.87	7.89E-02	-1.52	23.65	9.49E-01	41.97	22.11	5.77E-02	7.95	16.52	6.30E-01
cg03389046	18.53	8.66	3.25E-02	14.02	14.30	3.27E-01	10.17	16.68	5.42E-01	16.18	20.46	4.29E-01	42.05	20.20	3.73E-02
cg01811815	8.40	3.93	3.25E-02	9.56	5.66	9.10E-02	11.36	7.68	1.39E-01	7.15	12.21	5.58E-01	0.51	10.09	9.60E-01
cg08189968	22.97	10.74	3.25E-02	9.28	13.92	5.05E-01	35.24	28.08	2.10E-01	9.65	37.39	7.96E-01	52.66	22.04	1.69E-02
cg14487131	15.89	7.43	3.25E-02	47.02	22.09	3.33E-02	30.81	40.08	4.42E-01	15.98	5.75	5.46E-03	-2.18	14.68	8.82E-01

cg10645855	31.56	14.76	3.25E-02	30.70	21.63	1.56E-01	1.83	40.12	9.64E-01	46.00	46.21	3.19E-01	41.46	27.06	1.26E-01
cg18384794	-15.83	7.40	3.25E-02	-16.64	14.48	2.50E-01	10.32	29.11	7.23E-01	-16.33	10.45	1.18E-01	-22.91	17.81	1.98E-01
cg08359697	20.86	9.75	3.25E-02	6.19	19.49	7.51E-01	-30.24	47.51	5.24E-01	31.15	12.72	1.43E-02	18.97	28.20	5.01E-01
cg06480998	-12.38	5.79	3.25E-02	-12.65	14.00	3.66E-01	25.15	27.52	3.61E-01	-14.74	7.02	3.58E-02	-12.51	17.93	4.85E-01
cg16892046	43.72	20.45	3.25E-02	13.36	50.96	7.93E-01	78.73	84.29	3.50E-01	61.37	25.21	1.49E-02	-28.03	58.46	6.32E-01
cg23727321	17.85	8.35	3.25E-02	37.23	28.30	1.88E-01	-58.00	116.36	6.18E-01	16.25	8.97	6.99E-02	19.97	41.16	6.28E-01
cg21981270	24.75	11.57	3.25E-02	34.92	16.70	3.66E-02	27.49	57.57	6.33E-01	-3.41	56.47	9.52E-01	16.03	17.50	3.60E-01
cg09460563	15.81	7.39	3.25E-02	13.95	13.41	2.98E-01	52.21	32.27	1.06E-01	13.01	9.76	1.83E-01	19.50	27.95	4.85E-01
cg19411729	-9.36	4.38	3.25E-02	-17.07	7.76	2.79E-02	-8.81	10.61	4.07E-01	-1.48	8.49	8.61E-01	-8.29	8.82	3.47E-01
cg04265951	12.36	5.78	3.25E-02	3.72	15.22	8.07E-01	-0.75	15.77	9.62E-01	17.47	7.22	1.55E-02	9.01	20.31	6.58E-01
cg01182690	13.11	6.13	3.25E-02	5.16	17.54	7.69E-01	22.19	59.47	7.09E-01	16.16	6.86	1.85E-02	-10.15	23.67	6.68E-01
cg11819402	11.40	5.33	3.25E-02	6.64	8.94	4.58E-01	12.39	12.60	3.25E-01	9.36	10.45	3.71E-01	21.43	11.79	6.92E-02
cg24671202	15.23	7.12	3.25E-02	19.96	9.39	3.36E-02	17.15	22.74	4.51E-01	21.55	32.00	5.01E-01	3.60	13.54	7.90E-01
cg13752005	29.43	13.77	3.25E-02	44.78	44.29	3.12E-01	-145.77	183.65	4.27E-01	26.87	15.11	7.53E-02	53.69	53.13	3.12E-01
cg11890080	32.21	15.06	3.25E-02	34.14	16.82	4.24E-02	138.68	133.20	2.98E-01	47.17	70.82	5.05E-01	6.49	40.34	8.72E-01
cg26441291	-16.40	7.67	3.25E-02	-8.21	20.63	6.91E-01	-7.48	53.86	8.90E-01	-22.69	8.88	1.06E-02	18.96	24.82	4.45E-01
cg01106338	15.67	7.33	3.25E-02	21.52	10.29	3.65E-02	16.82	17.59	3.39E-01	25.51	9.68	8.41E-03	-7.23	12.95	5.77E-01
cg10900526	17.56	8.21	3.25E-02	11.64	17.99	5.18E-01	-2.60	32.04	9.35E-01	21.51	10.40	3.87E-02	18.48	25.64	4.71E-01
cg02790691	6.06	2.84	3.25E-02	6.43	4.71	1.73E-01	12.69	6.55	5.28E-02	4.75	4.79	3.21E-01	-3.10	8.98	7.30E-01
cg10870587	-8.39	3.92	3.25E-02	-11.23	6.19	6.94E-02	-1.36	22.72	9.52E-01	-6.32	6.36	3.20E-01	-7.58	9.06	4.03E-01
cg16256106	26.33	12.32	3.26E-02	24.45	14.34	8.83E-02	27.36	56.42	6.28E-01	66.76	81.94	4.15E-01	28.50	28.07	3.10E-01
cg01920536	15.65	7.32	3.26E-02	12.75	10.56	2.27E-01	15.30	15.14	3.12E-01	12.25	26.79	6.48E-01	23.87	15.96	1.35E-01
cg02165743	-8.46	3.96	3.26E-02	-12.45	5.69	2.85E-02	-10.21	7.95	1.99E-01	-9.93	23.79	6.76E-01	1.57	8.07	8.46E-01
cg06771021	16.47	7.71	3.26E-02	19.95	20.32	3.26E-01	38.19	30.20	2.06E-01	14.66	9.03	1.04E-01	6.90	30.89	8.23E-01
cg21184369	5.06	2.37	3.26E-02	4.34	3.37	1.97E-01	6.53	5.60	2.43E-01	5.94	5.08	2.42E-01	4.14	7.11	5.61E-01
cg13823666	-12.72	5.95	3.26E-02	-23.73	15.60	1.28E-01	0.37	20.22	9.85E-01	-12.97	7.23	7.29E-02	-5.65	19.85	7.76E-01
cg21241902	8.98	4.20	3.26E-02	12.58	4.83	9.21E-03	18.24	8.89	4.02E-02	-5.12	9.02	5.70E-01	6.33	7.15	3.76E-01
cg26307355	-36.68	17.16	3.26E-02	-44.64	25.78	8.34E-02	22.91	74.16	7.57E-01	-40.22	28.39	1.57E-01	-24.84	46.26	5.91E-01
cg23451129	-7.89	3.69	3.26E-02	-7.90	5.74	1.69E-01	-13.78	11.61	2.35E-01	-3.23	6.10	5.96E-01	-17.10	10.67	1.09E-01
cg27445265	-37.01	17.32	3.26E-02	-32.39	20.68	1.17E-01	-138.47	78.25	7.68E-02	-22.90	86.45	7.91E-01	-31.45	37.80	4.05E-01
cg13544294	15.94	7.46	3.26E-02	12.59	13.43	3.49E-01	21.70	19.77	2.73E-01	19.68	11.26	8.07E-02	3.08	22.44	8.91E-01
cg24351452	-12.71	5.95	3.26E-02	-2.39	16.94	8.88E-01	-10.54	23.20	6.50E-01	-14.01	6.83	4.02E-02	-20.78	25.91	4.23E-01
cg13480898	9.14	4.28	3.26E-02	8.23	8.61	3.39E-01	24.54	11.57	3.40E-02	8.48	5.76	1.41E-01	-6.00	13.45	6.56E-01
cg19967421	-39.73	18.59	3.26E-02	-37.88	28.70	1.87E-01	8.76	68.36	8.98E-01	-91.23	40.20	2.32E-02	-16.99	34.38	6.21E-01
cg00043324	-11.88	5.56	3.26E-02	-16.29	7.78	3.64E-02	-8.11	7.73	2.94E-01	-71.40	31.80	2.47E-02	-6.67	6.40	2.97E-01

cg06566994	10.71	5.01	3.26E-02	7.60	13.34	5.69E-01	28.53	17.55	1.04E-01	9.52	5.92	1.08E-01	8.04	20.40	6.93E-01
cg14414154	4.79	2.24	3.26E-02	4.81	3.14	1.25E-01	7.83	5.85	1.81E-01	7.66	4.96	1.22E-01	-2.72	6.01	6.50E-01
cg27549619	28.70	13.43	3.26E-02	56.34	13.94	5.28E-05	28.37	29.04	3.28E-01	22.10	19.45	2.56E-01	4.84	15.13	7.49E-01
cg23856967	-15.32	7.17	3.26E-02	-24.17	10.03	1.60E-02	-21.53	15.94	1.77E-01	-1.41	7.36	8.48E-01	-26.93	15.30	7.83E-02
cg00006459	13.51	6.32	3.26E-02	16.04	15.14	2.89E-01	11.25	30.42	7.11E-01	15.30	7.62	4.48E-02	-3.08	20.52	8.81E-01
cg26986731	33.18	15.53	3.26E-02	45.67	24.81	6.57E-02	83.05	45.60	6.85E-02	-1.49	29.66	9.60E-01	27.82	33.24	4.03E-01
cg05481445	-32.46	15.19	3.26E-02	-39.03	17.56	2.62E-02	-68.71	64.38	2.86E-01	14.09	55.23	7.99E-01	-4.09	43.76	9.25E-01
cg19881263	30.27	14.17	3.26E-02	35.99	17.08	3.50E-02	28.83	15.63	6.52E-02	1.65	10.75	8.78E-01	76.92	25.46	2.52E-03
cg25262044	-27.31	12.78	3.26E-02	-49.16	43.44	2.58E-01	-302.15	198.14	1.27E-01	-24.81	14.32	8.32E-02	-18.03	38.03	6.35E-01
cg18120576	-20.08	9.40	3.26E-02	-24.00	16.37	1.43E-01	7.32	25.98	7.78E-01	-23.55	16.74	1.60E-01	-25.44	19.83	2.00E-01
cg09009788	8.76	4.10	3.26E-02	7.31	5.63	1.94E-01	23.96	10.70	2.51E-02	8.01	10.03	4.25E-01	0.18	10.37	9.86E-01
cg09201327	28.05	13.13	3.26E-02	46.44	16.55	5.03E-03	22.24	41.82	5.95E-01	-51.92	72.23	4.72E-01	10.04	20.71	6.28E-01
cg13547551	-38.68	18.11	3.26E-02	-30.01	23.53	2.02E-01	-38.64	54.97	4.82E-01	-40.30	52.13	4.40E-01	-66.37	42.83	1.21E-01
cg00673191	6.96	3.26	3.26E-02	8.76	4.56	5.45E-02	14.99	8.82	8.93E-02	5.00	7.52	5.06E-01	-3.02	8.01	7.06E-01
cg03064080	-27.02	12.65	3.26E-02	26.19	38.55	4.97E-01	56.22	141.98	6.92E-01	-34.13	13.97	1.46E-02	-35.52	49.37	4.72E-01
cg00824156	17.04	7.98	3.27E-02	16.76	17.74	3.45E-01	19.54	25.29	4.40E-01	11.36	10.39	2.74E-01	46.05	24.18	5.68E-02
cg23361009	-47.16	22.08	3.27E-02	-32.65	28.30	2.49E-01	118.45	134.83	3.80E-01	-86.23	59.55	1.48E-01	-82.01	46.35	7.68E-02
cg06742044	-22.73	10.64	3.27E-02	-29.24	18.52	1.14E-01	-82.18	108.46	4.49E-01	-20.54	13.95	1.41E-01	-4.20	38.08	9.12E-01
cg03436461	28.65	13.41	3.27E-02	47.92	22.07	2.99E-02	-8.30	37.29	8.24E-01	18.02	31.20	5.63E-01	27.49	23.84	2.49E-01
cg24903497	-7.37	3.45	3.27E-02	-15.62	7.45	3.61E-02	3.05	11.73	7.95E-01	-7.06	4.56	1.21E-01	-1.87	9.75	8.48E-01
cg26197117	35.09	16.43	3.27E-02	41.51	24.28	8.73E-02	31.91	73.25	6.63E-01	20.12	50.64	6.91E-01	31.97	26.43	2.26E-01
cg23603376	19.87	9.30	3.27E-02	20.10	13.04	1.23E-01	21.09	23.94	3.78E-01	6.13	29.29	8.34E-01	24.40	19.02	1.99E-01
cg21745248	-14.18	6.64	3.27E-02	4.86	31.67	8.78E-01	-38.13	154.96	8.06E-01	-13.23	6.89	5.49E-02	-78.45	41.10	5.63E-02
cg24427376	8.94	4.18	3.27E-02	11.18	5.73	5.13E-02	4.67	9.21	6.12E-01	14.72	12.37	2.34E-01	2.30	10.93	8.34E-01
cg00894352	-13.23	6.19	3.27E-02	-16.50	11.25	1.42E-01	0.67	13.34	9.60E-01	-21.73	10.51	3.86E-02	-6.10	16.97	7.20E-01
cg12184293	8.94	4.19	3.27E-02	5.90	11.38	6.04E-01	10.86	19.16	5.71E-01	10.16	4.76	3.27E-02	-5.93	20.47	7.72E-01
cg17959631	-12.43	5.82	3.27E-02	-17.73	8.91	4.65E-02	-11.41	13.51	3.98E-01	-17.93	15.44	2.45E-01	-0.78	11.76	9.47E-01
cg17861618	-13.28	6.22	3.27E-02	-20.50	9.99	4.02E-02	0.10	14.59	9.95E-01	-16.53	13.09	2.06E-01	-7.91	13.71	5.64E-01
cg14170756	-41.48	19.42	3.27E-02	-25.38	34.44	4.61E-01	-48.81	187.00	7.94E-01	-44.72	26.83	9.55E-02	-64.12	50.59	2.05E-01
cg09154639	16.51	7.73	3.27E-02	12.56	10.81	2.45E-01	13.83	19.55	4.79E-01	48.76	33.06	1.40E-01	18.95	14.68	1.97E-01
cg08484506	-10.27	4.81	3.27E-02	-10.96	7.84	1.62E-01	-11.34	10.76	2.92E-01	-5.25	11.29	6.42E-01	-12.08	9.77	2.16E-01
cg01432692	-12.34	5.78	3.27E-02	-18.24	12.50	1.45E-01	-0.23	15.89	9.89E-01	-8.65	7.85	2.70E-01	-33.02	17.19	5.47E-02
cg18500988	11.09	5.19	3.27E-02	22.25	7.56	3.25E-03	2.28	12.03	8.50E-01	9.74	9.73	3.17E-01	1.98	9.67	8.38E-01
cg21105019	-11.59	5.43	3.27E-02	-20.67	11.16	6.40E-02	-6.11	17.41	7.25E-01	-9.25	7.09	1.92E-01	-8.52	19.13	6.56E-01
cg17206604	-13.51	6.33	3.27E-02	-17.82	15.86	2.61E-01	-36.95	25.59	1.49E-01	-9.26	7.50	2.17E-01	-26.85	24.28	2.69E-01

cg05127440	30.13	14.11	3.27E-02	60.01	25.56	1.89E-02	82.50	65.72	2.09E-01	20.26	16.82	2.28E-01	-5.08	36.00	8.88E-01
cg23993559	11.52	5.39	3.27E-02	13.44	9.42	1.54E-01	-3.53	11.38	7.56E-01	19.20	9.14	3.57E-02	12.27	17.09	4.73E-01
cg07464571	13.07	6.12	3.27E-02	3.54	6.88	6.08E-01	30.85	11.32	6.41E-03	17.23	11.27	1.26E-01	8.36	11.31	4.60E-01
cg23957311	7.96	3.73	3.27E-02	3.23	5.88	5.82E-01	3.85	8.29	6.43E-01	17.52	7.39	1.77E-02	10.05	9.90	3.10E-01
cg00289094	-13.14	6.15	3.27E-02	-19.88	10.22	5.18E-02	-3.05	14.08	8.28E-01	1.19	13.92	9.32E-01	-22.22	12.27	7.02E-02
cg08449748	14.42	6.75	3.27E-02	9.74	9.74	3.18E-01	13.68	13.23	3.01E-01	17.61	19.64	3.70E-01	29.16	18.02	1.06E-01
cg22988581	-9.08	4.25	3.27E-02	-8.81	11.27	4.34E-01	-6.61	13.62	6.28E-01	-7.32	5.21	1.61E-01	-24.30	13.77	7.77E-02
cg26417346	-38.72	18.13	3.27E-02	-47.10	26.82	7.91E-02	13.27	55.78	8.12E-01	-72.68	56.47	1.98E-01	-33.23	31.37	2.90E-01
cg06641415	13.51	6.33	3.27E-02	16.29	18.61	3.81E-01	66.83	67.39	3.21E-01	15.32	7.14	3.19E-02	-10.95	21.02	6.02E-01
cg10839291	15.83	7.41	3.27E-02	15.91	9.44	9.18E-02	25.04	21.22	2.38E-01	-1.99	29.76	9.47E-01	15.48	16.62	3.52E-01
cg13531562	20.30	9.51	3.27E-02	36.60	18.77	5.12E-02	8.98	18.69	6.31E-01	8.00	16.66	6.31E-01	37.56	23.82	1.15E-01
cg24173182	9.63	4.51	3.27E-02	4.98	4.05	2.18E-01	7.10	6.75	2.92E-01	33.69	13.36	1.17E-02	12.75	10.10	2.07E-01
cg26320080	17.63	8.26	3.27E-02	1.84	12.10	8.79E-01	38.34	19.48	4.90E-02	30.90	18.44	9.39E-02	18.48	15.25	2.26E-01
cg12811552	39.06	18.29	3.27E-02	11.64	45.68	7.99E-01	7.11	184.89	9.69E-01	48.24	21.21	2.29E-02	14.41	62.44	8.17E-01
cg13504318	5.71	2.67	3.27E-02	5.72	3.41	9.29E-02	12.30	5.11	1.60E-02	0.77	9.98	9.39E-01	-1.30	5.98	8.28E-01
cg03517146	20.89	9.78	3.27E-02	7.33	19.07	7.01E-01	31.23	16.79	6.29E-02	17.38	18.73	3.53E-01	29.05	27.72	2.95E-01
cg20819482	4.76	2.23	3.27E-02	6.02	3.20	6.01E-02	8.33	5.21	1.10E-01	1.21	4.64	7.95E-01	0.41	6.93	9.53E-01
cg04131010	13.45	6.30	3.28E-02	38.97	20.24	5.42E-02	32.32	23.18	1.63E-01	8.09	7.13	2.56E-01	20.26	28.89	4.83E-01
cg03957095	9.66	4.53	3.28E-02	9.11	9.58	3.42E-01	3.27	11.52	7.77E-01	10.61	6.96	1.28E-01	13.22	10.13	1.92E-01
cg19381766	12.54	5.87	3.28E-02	9.64	8.09	2.34E-01	20.83	14.10	1.40E-01	30.85	19.65	1.16E-01	5.20	12.78	6.84E-01
cg21234244	9.08	4.25	3.28E-02	6.47	9.31	4.87E-01	17.38	19.68	3.77E-01	10.17	5.28	5.38E-02	3.22	13.88	8.17E-01
cg23415489	-23.84	11.17	3.28E-02	-27.06	16.27	9.63E-02	-10.45	30.17	7.29E-01	-28.80	26.63	2.80E-01	-21.27	24.01	3.76E-01
cg22694813	-15.11	7.08	3.28E-02	-3.63	16.91	8.30E-01	-20.86	36.39	5.66E-01	-17.99	8.35	3.13E-02	-11.11	26.93	6.80E-01
cg06834005	-7.53	3.53	3.28E-02	-14.70	8.64	8.89E-02	-4.02	16.97	8.13E-01	-6.08	4.32	1.59E-01	-6.93	10.04	4.90E-01
cg00454734	13.36	6.26	3.28E-02	1.37	17.07	9.36E-01	15.70	13.58	2.48E-01	13.78	8.13	9.03E-02	27.67	25.38	2.76E-01
cg23256579	-5.51	2.58	3.28E-02	-4.10	3.81	2.81E-01	-5.06	5.18	3.28E-01	-9.52	6.00	1.13E-01	-5.70	7.93	4.72E-01
cg27543385	-10.47	4.90	3.28E-02	-12.79	6.67	5.51E-02	-19.81	8.34	1.75E-02	-11.48	13.01	3.77E-01	3.07	8.55	7.20E-01
cg14069049	14.61	6.84	3.28E-02	24.91	17.31	1.50E-01	32.75	26.95	2.24E-01	10.56	9.05	2.43E-01	12.39	15.04	4.10E-01
cg05358729	21.19	9.92	3.28E-02	24.29	13.33	6.85E-02	-29.10	32.43	3.70E-01	19.74	30.81	5.22E-01	33.80	19.90	8.94E-02
cg18036653	16.82	7.88	3.28E-02	20.82	12.94	1.07E-01	3.66	15.73	8.16E-01	14.21	17.54	4.18E-01	30.09	18.76	1.09E-01
cg02500392	-8.22	3.85	3.28E-02	-16.26	8.54	5.69E-02	-7.37	11.66	5.27E-01	-4.82	5.17	3.51E-01	-10.80	10.58	3.07E-01
cg26888063	11.93	5.59	3.28E-02	-5.88	14.97	6.95E-01	33.77	25.64	1.88E-01	14.20	6.56	3.03E-02	9.50	18.99	6.17E-01
cg17385414	17.69	8.29	3.28E-02	15.15	16.28	3.52E-01	13.26	19.70	5.01E-01	23.68	12.00	4.84E-02	1.34	28.10	9.62E-01
cg16267376	35.90	16.82	3.28E-02	30.32	20.69	1.43E-01	135.70	78.98	8.58E-02	-25.58	83.50	7.59E-01	42.46	33.45	2.04E-01
cg22535057	-14.75	6.91	3.28E-02	-1.61	11.26	8.86E-01	-4.81	20.59	8.15E-01	-29.09	12.63	2.12E-02	-21.42	13.53	1.14E-01

cg16042259	15.97	7.48	3.28E-02	10.00	10.16	3.25E-01	23.13	21.12	2.73E-01	-0.34	29.45	9.91E-01	28.64	14.46	4.77E-02
cg24662698	12.58	5.90	3.28E-02	15.74	11.86	1.84E-01	16.59	14.69	2.59E-01	14.67	11.32	1.95E-01	6.37	10.42	5.41E-01
cg04556393	10.03	4.70	3.28E-02	0.88	13.71	9.49E-01	39.80	28.26	1.59E-01	10.38	5.21	4.63E-02	9.24	23.28	6.91E-01
cg26004221	-10.21	4.78	3.28E-02	-16.19	7.30	2.65E-02	-7.88	11.96	5.10E-01	-6.28	13.04	6.30E-01	-4.17	9.09	6.47E-01
cg11147878	-6.70	3.14	3.28E-02	-5.00	6.11	4.13E-01	-8.04	8.18	3.26E-01	-5.37	4.87	2.71E-01	-11.30	7.52	1.33E-01
cg07843120	15.28	7.16	3.28E-02	14.60	9.25	1.15E-01	18.47	22.80	4.18E-01	12.88	23.51	5.84E-01	16.78	15.63	2.83E-01
cg20521198	-10.43	4.88	3.28E-02	-17.05	7.87	3.02E-02	0.86	11.58	9.41E-01	-10.85	12.16	3.72E-01	-8.19	9.31	3.79E-01
cg18118503	-11.28	5.28	3.28E-02	-9.76	9.84	3.21E-01	4.23	18.18	8.16E-01	-15.43	7.21	3.24E-02	-5.93	17.57	7.36E-01
cg05081033	-5.83	2.73	3.28E-02	-3.27	4.56	4.73E-01	-11.18	6.35	7.85E-02	-9.05	4.78	5.85E-02	2.82	7.59	7.10E-01
cg18482098	39.16	18.35	3.28E-02	31.39	42.25	4.57E-01	-222.36	150.16	1.39E-01	42.60	16.49	9.77E-03	64.70	54.00	2.31E-01
cg00865541	15.10	7.07	3.28E-02	10.26	11.80	3.85E-01	16.27	14.31	2.55E-01	29.69	14.01	3.40E-02	-1.05	18.86	9.56E-01
cg11352369	16.25	7.62	3.28E-02	19.78	13.81	1.52E-01	3.25	19.00	8.64E-01	13.58	11.64	2.43E-01	36.53	23.34	1.17E-01
cg26511108	8.63	4.05	3.28E-02	9.34	5.55	9.26E-02	3.68	9.98	7.12E-01	8.54	13.58	5.30E-01	10.68	8.69	2.19E-01
cg02240936	24.40	11.44	3.29E-02	17.40	21.96	4.28E-01	36.87	52.98	4.86E-01	16.95	15.24	2.66E-01	70.59	33.11	3.30E-02
cg04231636	10.75	5.04	3.29E-02	18.64	7.75	1.61E-02	9.03	11.51	4.33E-01	9.63	11.78	4.14E-01	-3.02	11.16	7.87E-01
cg14281591	4.65	2.18	3.29E-02	3.81	3.18	2.32E-01	7.16	4.81	1.36E-01	6.44	4.63	1.64E-01	-0.30	6.73	9.64E-01
cg06669759	21.98	10.30	3.29E-02	27.84	16.71	9.57E-02	36.42	64.62	5.73E-01	58.42	27.84	3.59E-02	6.06	14.78	6.82E-01
cg23484899	8.38	3.93	3.29E-02	5.78	6.77	3.93E-01	7.50	8.87	3.98E-01	13.32	7.05	5.88E-02	5.28	9.93	5.95E-01
cg27008363	11.43	5.36	3.29E-02	16.69	12.48	1.81E-01	10.63	17.72	5.49E-01	7.72	6.86	2.61E-01	23.49	15.88	1.39E-01
cg11808658	12.36	5.79	3.29E-02	7.46	10.40	4.73E-01	29.79	13.29	2.50E-02	5.03	7.51	5.03E-01	24.61	15.72	1.18E-01
cg17642941	10.16	4.76	3.29E-02	10.37	6.88	1.32E-01	16.00	10.54	1.29E-01	1.85	14.20	8.96E-01	8.42	10.55	4.25E-01
cg11105830	18.88	8.85	3.29E-02	8.67	14.53	5.51E-01	9.84	17.73	5.79E-01	31.63	19.23	1.00E-01	38.67	21.56	7.28E-02
cg25164237	10.89	5.10	3.29E-02	16.91	7.42	2.27E-02	3.62	12.72	7.76E-01	16.78	12.84	1.91E-01	-1.60	11.16	8.86E-01
cg02050028	-12.16	5.70	3.29E-02	-12.81	9.28	1.67E-01	7.73	12.12	5.24E-01	-16.55	8.01	3.89E-02	-23.42	12.60	6.30E-02
cg26345037	36.11	16.93	3.29E-02	8.34	26.36	7.52E-01	16.36	104.65	8.76E-01	65.59	27.77	1.82E-02	41.45	38.81	2.85E-01
cg08685733	-8.62	4.04	3.29E-02	-5.88	5.76	3.07E-01	-8.39	8.92	3.47E-01	-5.45	11.61	6.39E-01	-18.36	9.46	5.24E-02
cg09682311	-36.19	16.97	3.29E-02	-46.04	21.61	3.31E-02	-100.37	93.26	2.82E-01	68.02	77.32	3.79E-01	-25.69	30.86	4.05E-01
cg17270843	11.08	5.20	3.29E-02	12.95	9.31	1.64E-01	4.86	9.44	6.06E-01	17.36	13.04	1.83E-01	12.44	10.91	2.54E-01
cg06763054	11.31	5.30	3.29E-02	26.84	11.44	1.89E-02	7.79	12.55	5.35E-01	12.85	8.93	1.50E-01	0.93	9.39	9.21E-01
cg02023548	-11.82	5.54	3.29E-02	-17.29	8.90	5.20E-02	0.34	12.88	9.79E-01	-10.51	14.02	4.53E-01	-13.05	10.65	2.20E-01
cg21673240	15.88	7.44	3.29E-02	37.71	19.82	5.71E-02	-18.19	30.98	5.57E-01	15.44	8.79	7.87E-02	6.25	25.82	8.09E-01
cg25587233	11.44	5.36	3.29E-02	7.48	6.77	2.69E-01	29.05	13.67	3.36E-02	13.38	19.98	5.03E-01	8.93	14.00	5.24E-01
cg02129122	16.75	7.85	3.29E-02	4.08	17.73	8.18E-01	15.94	26.99	5.55E-01	16.89	9.92	8.86E-02	43.34	25.80	9.30E-02
cg09096787	7.00	3.28	3.29E-02	6.68	4.56	1.43E-01	13.49	7.87	8.65E-02	10.30	12.18	3.98E-01	1.92	6.74	7.76E-01
cg02812207	14.14	6.63	3.29E-02	26.13	10.84	1.60E-02	3.30	22.38	8.83E-01	11.57	13.49	3.91E-01	4.36	12.15	7.20E-01

cg24002839	52.13	24.44	3.29E-02	97.58	64.59	1.31E-01	-206.94	251.19	4.10E-01	47.28	28.40	9.60E-02	47.82	74.81	5.23E-01
cg19069360	5.68	2.66	3.29E-02	3.79	3.49	2.78E-01	8.67	5.84	1.38E-01	12.29	9.44	1.93E-01	5.33	7.37	4.70E-01
cg15749157	11.46	5.37	3.29E-02	14.74	9.94	1.38E-01	13.97	10.60	1.88E-01	11.77	9.98	2.38E-01	0.94	13.40	9.44E-01
cg18256205	-6.55	3.07	3.29E-02	-5.50	4.57	2.28E-01	-1.82	6.47	7.79E-01	-16.12	8.17	4.86E-02	-7.61	7.22	2.92E-01
cg17663235	30.12	14.12	3.29E-02	1.21	38.99	9.75E-01	168.26	211.82	4.27E-01	36.69	16.04	2.21E-02	8.63	47.25	8.55E-01
cg24830559	15.05	7.06	3.29E-02	3.00	16.47	8.55E-01	21.02	41.96	6.16E-01	17.47	8.40	3.75E-02	19.21	24.74	4.37E-01
cg05457923	-18.52	8.68	3.29E-02	-39.74	35.29	2.60E-01	-52.41	114.43	6.47E-01	-16.18	9.28	8.14E-02	-28.04	35.64	4.31E-01
cg22775860	13.51	6.33	3.29E-02	8.24	12.86	5.21E-01	19.02	29.25	5.15E-01	16.80	8.05	3.69E-02	2.36	20.95	9.10E-01
cg15748470	12.23	5.74	3.29E-02	18.79	15.54	2.27E-01	31.66	55.70	5.70E-01	8.84	6.81	1.94E-01	21.39	15.17	1.58E-01
cg14549078	14.27	6.69	3.29E-02	35.53	16.27	2.89E-02	4.48	19.79	8.21E-01	10.22	8.45	2.26E-01	14.93	22.37	5.04E-01
cg25729560	-13.19	6.18	3.29E-02	-18.16	14.93	2.24E-01	-40.25	28.29	1.55E-01	-7.45	6.62	2.61E-01	-38.90	22.01	7.72E-02
cg03945663	21.17	9.93	3.30E-02	8.88	19.68	6.52E-01	22.43	20.91	2.83E-01	30.64	16.61	6.50E-02	17.86	24.60	4.68E-01
cg13923018	21.48	10.07	3.30E-02	24.11	14.23	9.03E-02	15.11	28.26	5.93E-01	34.36	34.31	3.17E-01	15.82	18.83	4.01E-01
cg26426938	13.58	6.37	3.30E-02	13.02	9.47	1.69E-01	17.75	17.22	3.03E-01	27.57	18.46	1.35E-01	6.81	11.79	5.64E-01
cg08418670	5.07	2.38	3.30E-02	3.60	2.81	2.00E-01	11.67	5.88	4.71E-02	13.01	11.33	2.51E-01	0.18	8.55	9.84E-01
cg06965590	18.45	8.65	3.30E-02	20.23	19.25	2.93E-01	44.14	32.02	1.68E-01	9.84	8.24	2.32E-01	53.78	26.63	4.34E-02
cg17067226	-8.07	3.78	3.30E-02	-7.73	7.80	3.22E-01	-12.34	11.40	2.79E-01	-4.43	5.28	4.01E-01	-18.44	10.04	6.63E-02
cg22531668	13.36	6.27	3.30E-02	13.27	8.92	1.37E-01	-1.47	17.50	9.33E-01	10.18	20.51	6.20E-01	21.26	11.75	7.04E-02
cg25967146	18.09	8.48	3.30E-02	17.54	17.39	3.13E-01	-2.23	30.93	9.43E-01	19.39	11.36	8.78E-02	25.32	23.60	2.83E-01
cg01254707	-10.53	4.94	3.30E-02	-11.15	11.80	3.45E-01	-26.32	19.98	1.88E-01	-9.16	6.13	1.35E-01	-8.92	14.65	5.42E-01
cg07603056	18.22	8.54	3.30E-02	21.84	25.29	3.88E-01	30.90	97.67	7.52E-01	20.65	9.60	3.14E-02	-10.34	29.25	7.24E-01
cg03867871	-38.93	18.26	3.30E-02	-36.86	22.58	1.03E-01	-78.35	91.69	3.93E-01	35.69	78.04	6.47E-01	-54.33	36.40	1.36E-01
cg25189764	-8.50	3.99	3.30E-02	-13.85	9.33	1.38E-01	-3.10	16.40	8.50E-01	-6.28	4.91	2.01E-01	-16.61	12.65	1.89E-01
cg22499797	13.46	6.31	3.30E-02	15.52	9.81	1.13E-01	14.74	12.67	2.45E-01	-7.57	17.08	6.58E-01	21.89	14.07	1.20E-01
cg01214063	-8.23	3.86	3.30E-02	-14.21	8.16	8.16E-02	12.63	15.20	4.06E-01	-7.81	5.06	1.22E-01	-10.12	10.69	3.44E-01
cg19739596	6.01	2.82	3.30E-02	5.82	3.57	1.03E-01	9.05	7.52	2.29E-01	8.23	8.39	3.27E-01	1.52	7.95	8.48E-01
cg00945663	19.67	9.23	3.30E-02	25.68	17.47	1.42E-01	23.90	18.11	1.87E-01	2.38	16.64	8.86E-01	36.11	23.47	1.24E-01
cg15092561	13.19	6.19	3.30E-02	25.94	12.25	3.42E-02	9.39	13.81	4.96E-01	16.98	10.16	9.46E-02	-4.83	13.39	7.18E-01
cg11254053	4.86	2.28	3.30E-02	3.74	2.98	2.09E-01	10.58	5.23	4.29E-02	5.50	7.30	4.51E-01	0.91	6.45	8.87E-01
cg20180050	-13.07	6.13	3.30E-02	-31.36	12.96	1.55E-02	-21.46	26.30	4.15E-01	-7.04	7.83	3.68E-01	-5.19	18.59	7.80E-01
cg23955334	-8.98	4.21	3.30E-02	-14.61	7.60	5.45E-02	7.18	13.76	6.02E-01	-7.10	5.71	2.14E-01	-23.32	17.82	1.91E-01
cg05992875	-97.97	45.95	3.30E-02	-147.48	61.69	1.68E-02	241.83	256.82	3.46E-01	-85.81	109.49	4.33E-01	-37.00	94.37	6.95E-01
cg10488031	13.93	6.53	3.30E-02	12.88	9.41	1.71E-01	13.47	15.45	3.83E-01	3.57	19.29	8.53E-01	21.82	13.78	1.13E-01
cg08561732	15.30	7.18	3.30E-02	12.24	9.18	1.83E-01	31.69	30.74	3.03E-01	-10.50	27.68	7.04E-01	25.47	13.89	6.66E-02
cg19384379	15.27	7.16	3.30E-02	11.46	10.72	2.85E-01	20.32	15.22	1.82E-01	26.05	20.79	2.10E-01	12.02	15.49	4.38E-01

cg11482719	14.93	7.00	3.30E-02	24.27	11.16	2.97E-02	12.83	20.82	5.38E-01	25.08	16.84	1.36E-01	-1.26	12.37	9.19E-01
cg14284211	10.59	4.97	3.30E-02	9.50	6.21	1.26E-01	27.64	13.99	4.82E-02	3.54	17.35	8.38E-01	4.83	12.75	7.04E-01
cg13378643	-15.61	7.32	3.30E-02	-23.70	10.97	3.07E-02	-12.18	17.18	4.78E-01	-16.83	16.88	3.19E-01	1.82	17.06	9.15E-01
cg12320621	17.57	8.24	3.30E-02	26.81	18.18	1.40E-01	31.78	23.51	1.76E-01	7.84	10.89	4.71E-01	37.14	26.25	1.57E-01
cg21028182	-14.92	7.00	3.30E-02	-10.26	19.34	5.96E-01	-28.45	42.29	5.01E-01	-14.03	8.05	8.15E-02	-25.67	23.95	2.84E-01
cg05683049	16.28	7.64	3.30E-02	14.09	10.04	1.61E-01	8.50	19.19	6.58E-01	54.69	48.48	2.59E-01	22.80	15.66	1.45E-01
cg06886736	-28.40	13.32	3.30E-02	-19.22	35.73	5.91E-01	-244.71	154.91	1.14E-01	-29.95	15.41	5.19E-02	-14.36	40.95	7.26E-01
cg26717786	-22.55	10.58	3.30E-02	-15.12	13.57	2.65E-01	-62.01	25.75	1.60E-02	-13.41	44.58	7.64E-01	-12.82	25.84	6.20E-01
cg01520853	19.31	9.06	3.30E-02	35.69	17.88	4.60E-02	25.87	18.10	1.53E-01	11.49	15.24	4.51E-01	-2.76	24.22	9.09E-01
cg02449461	10.03	4.71	3.30E-02	12.27	7.05	8.20E-02	17.49	10.66	1.01E-01	13.77	11.38	2.26E-01	-6.35	10.84	5.58E-01
cg15367000	-13.77	6.46	3.30E-02	-27.27	13.53	4.39E-02	14.84	20.32	4.65E-01	-12.82	8.12	1.14E-01	-19.46	23.59	4.09E-01
cg20334079	-5.48	2.57	3.30E-02	-4.46	3.88	2.51E-01	-7.52	5.86	2.00E-01	-6.21	5.58	2.66E-01	-4.84	6.46	4.54E-01
cg18906002	12.08	5.67	3.30E-02	18.16	9.18	4.80E-02	11.49	10.69	2.82E-01	10.65	19.74	5.90E-01	4.13	11.19	7.12E-01
cg27027631	9.93	4.66	3.31E-02	10.05	6.01	9.47E-02	9.88	13.42	4.62E-01	25.00	16.73	1.35E-01	3.83	10.34	7.11E-01
cg06741096	-11.17	5.24	3.31E-02	-6.51	13.08	6.19E-01	-25.77	26.01	3.22E-01	-9.71	6.15	1.14E-01	-28.03	19.53	1.51E-01
cg17433211	-19.08	8.95	3.31E-02	-10.64	13.21	4.20E-01	-28.92	23.98	2.28E-01	-31.91	30.31	2.93E-01	-23.48	15.96	1.41E-01
cg16670446	14.29	6.70	3.31E-02	7.03	10.23	4.92E-01	5.68	13.90	6.83E-01	28.41	15.66	6.97E-02	30.64	17.03	7.20E-02
cg27380429	12.79	6.00	3.31E-02	-0.77	14.74	9.58E-01	-3.79	29.83	8.99E-01	13.88	7.37	5.96E-02	29.48	16.56	7.51E-02
cg17736259	17.83	8.37	3.31E-02	25.82	16.67	1.21E-01	13.30	25.51	6.02E-01	8.72	8.49	3.04E-01	53.49	24.03	2.60E-02
cg26715883	-21.17	9.93	3.31E-02	-20.78	21.94	3.44E-01	-70.63	50.73	1.64E-01	-18.02	12.35	1.45E-01	-23.17	29.94	4.39E-01
cg15377871	5.19	2.43	3.31E-02	3.47	2.98	2.43E-01	11.06	5.89	6.05E-02	3.06	8.93	7.32E-01	8.77	8.35	2.94E-01
cg01975495	9.03	4.24	3.31E-02	8.50	5.96	1.54E-01	10.32	10.86	3.42E-01	1.19	9.62	9.01E-01	19.73	10.99	7.27E-02
cg05257275	8.90	4.18	3.31E-02	7.56	5.62	1.79E-01	10.88	9.82	2.68E-01	24.28	14.99	1.05E-01	4.62	9.61	6.30E-01
cg01462668	-8.02	3.76	3.31E-02	-16.37	6.50	1.18E-02	0.10	6.76	9.88E-01	-10.18	5.92	8.56E-02	-2.39	8.97	7.90E-01
cg08361126	16.54	7.76	3.31E-02	30.57	12.02	1.10E-02	1.60	18.83	9.32E-01	26.27	21.27	2.17E-01	3.65	13.15	7.81E-01
cg17168636	28.78	13.51	3.31E-02	9.95	37.18	7.89E-01	-6.13	148.47	9.67E-01	29.96	15.17	4.82E-02	56.41	52.35	2.81E-01
cg00414898	45.87	21.52	3.31E-02	62.52	30.62	4.12E-02	86.48	78.96	2.73E-01	33.42	57.86	5.64E-01	13.40	39.75	7.36E-01
cg00702030	-37.77	17.72	3.31E-02	-85.66	54.84	1.18E-01	-182.04	205.85	3.77E-01	-37.43	19.78	5.85E-02	30.20	60.67	6.19E-01
cg07534331	30.13	14.14	3.31E-02	29.71	17.27	8.54E-02	43.19	67.10	5.20E-01	22.77	69.77	7.44E-01	30.13	28.59	2.92E-01
cg04001941	-10.57	4.96	3.31E-02	-13.26	14.48	3.60E-01	-14.81	17.85	4.07E-01	-7.13	5.75	2.15E-01	-42.04	20.10	3.64E-02
cg13685680	18.36	8.62	3.31E-02	10.43	17.68	5.55E-01	13.10	14.57	3.68E-01	18.95	16.46	2.49E-01	44.13	23.17	5.68E-02
cg27521562	9.93	4.66	3.31E-02	11.59	6.89	9.29E-02	4.82	9.78	6.22E-01	16.20	13.65	2.35E-01	8.28	10.42	4.27E-01
cg20702205	-14.14	6.64	3.31E-02	-14.43	16.36	3.78E-01	-23.98	30.09	4.25E-01	-15.53	8.03	5.33E-02	-0.06	20.52	9.98E-01
cg15360181	-7.07	3.32	3.31E-02	-9.22	4.68	4.88E-02	-4.75	7.49	5.26E-01	-2.24	8.55	7.94E-01	-7.75	8.55	3.65E-01
cg05193538	8.96	4.21	3.31E-02	12.61	6.28	4.46E-02	4.63	11.28	6.81E-01	8.81	8.72	3.12E-01	3.40	9.91	7.32E-01

cg08473764	13.78	6.47	3.31E-02	7.35	6.25	2.40E-01	31.89	8.93	3.55E-04	18.24	6.83	7.57E-03	-2.04	9.28	8.26E-01
cg16806041	-6.02	2.83	3.31E-02	-6.39	4.28	1.35E-01	-3.39	5.96	5.69E-01	-3.81	7.26	6.00E-01	-10.12	6.54	1.22E-01
cg05981335	10.81	5.08	3.31E-02	18.02	8.63	3.68E-02	5.73	13.53	6.72E-01	17.72	11.98	1.39E-01	1.78	8.78	8.39E-01
cg03065090	-9.64	4.52	3.31E-02	-13.77	10.20	1.77E-01	6.18	12.62	6.25E-01	-9.45	6.13	1.23E-01	-19.72	12.50	1.15E-01
cg23760165	33.35	15.65	3.31E-02	24.22	37.34	5.16E-01	-45.46	238.27	8.49E-01	39.23	18.40	3.30E-02	9.30	50.44	8.54E-01
cg09885086	-46.31	21.74	3.31E-02	-29.82	29.33	3.09E-01	-125.44	76.32	1.00E-01	-28.05	61.36	6.48E-01	-66.47	43.98	1.31E-01
cg14750277	19.66	9.23	3.31E-02	29.56	15.97	6.43E-02	15.25	25.51	5.50E-01	24.71	22.03	2.62E-01	9.63	15.37	5.31E-01
cg04016660	6.83	3.21	3.31E-02	6.31	4.80	1.88E-01	15.30	6.78	2.39E-02	2.79	7.14	6.96E-01	0.17	8.98	9.85E-01
cg10739095	23.13	10.86	3.31E-02	31.20	26.13	2.32E-01	34.41	45.65	4.51E-01	23.81	13.74	8.30E-02	6.31	28.42	8.24E-01
cg02289682	-33.69	15.81	3.31E-02	-17.53	19.91	3.79E-01	-84.21	73.87	2.54E-01	-64.73	62.12	2.97E-01	-56.37	31.09	6.98E-02
cg10483660	-5.53	2.60	3.31E-02	-0.02	5.61	9.97E-01	-13.40	8.25	1.05E-01	-4.66	3.57	1.92E-01	-10.93	6.51	9.29E-02
cg27641317	-8.29	3.89	3.31E-02	-12.52	5.68	2.76E-02	-2.72	9.04	7.64E-01	-4.68	11.08	6.73E-01	-6.03	8.27	4.66E-01
cg15901550	25.54	11.99	3.32E-02	28.14	17.62	1.10E-01	38.10	25.42	1.34E-01	-27.40	34.31	4.24E-01	38.39	27.34	1.60E-01
cg15442444	23.41	10.99	3.32E-02	32.08	12.80	1.22E-02	-0.76	56.02	9.89E-01	-39.20	53.18	4.61E-01	8.12	25.74	7.53E-01
cg01226806	-51.34	24.10	3.32E-02	-130.63	65.29	4.54E-02	-131.73	248.43	5.96E-01	-25.05	27.35	3.60E-01	-96.88	60.47	1.09E-01
cg08219773	5.15	2.42	3.32E-02	6.35	4.27	1.37E-01	-2.92	5.47	5.94E-01	6.54	3.94	9.72E-02	10.79	6.98	1.22E-01
cg05517697	17.50	8.22	3.32E-02	14.04	13.87	3.11E-01	0.29	17.04	9.86E-01	16.25	16.61	3.28E-01	42.65	17.59	1.53E-02
cg04800490	12.39	5.82	3.32E-02	9.85	9.96	3.22E-01	12.25	11.33	2.79E-01	14.99	11.71	2.01E-01	14.15	15.09	3.48E-01
cg11621667	25.50	11.97	3.32E-02	9.97	23.90	6.77E-01	-60.01	70.42	3.94E-01	29.89	15.79	5.83E-02	51.88	31.37	9.82E-02
cg12889538	31.45	14.76	3.32E-02	47.16	21.24	2.64E-02	-47.16	95.41	6.21E-01	67.85	52.11	1.93E-01	10.52	22.98	6.47E-01
cg04554195	-8.00	3.76	3.32E-02	-12.13	8.03	1.31E-01	-8.19	13.81	5.53E-01	-3.40	5.08	5.03E-01	-17.96	9.38	5.56E-02
cg17277199	-5.79	2.72	3.32E-02	-4.96	3.79	1.91E-01	-3.43	6.45	5.95E-01	-4.86	8.49	5.67E-01	-10.39	6.00	8.31E-02
cg03553308	-9.59	4.50	3.32E-02	-8.81	5.17	8.79E-02	-16.73	8.15	4.00E-02	7.12	9.64	4.60E-01	-16.04	7.51	3.27E-02
cg07721852	12.03	5.65	3.32E-02	18.34	7.52	1.48E-02	12.15	17.11	4.78E-01	2.70	22.63	9.05E-01	0.76	10.98	9.45E-01
cg24854010	12.11	5.69	3.32E-02	12.22	8.22	1.37E-01	25.04	12.02	3.72E-02	10.62	15.09	4.82E-01	-5.42	14.39	7.07E-01
cg18110444	30.22	14.19	3.32E-02	29.96	35.54	3.99E-01	177.91	150.57	2.37E-01	35.74	16.71	3.24E-02	-17.24	42.63	6.86E-01
cg01042641	-7.58	3.56	3.32E-02	-6.16	4.41	1.62E-01	-20.86	9.79	3.31E-02	1.99	12.57	8.74E-01	-7.14	9.66	4.60E-01
cg09110148	21.23	9.97	3.32E-02	21.22	14.01	1.30E-01	11.15	22.96	6.27E-01	32.58	35.53	3.59E-01	25.69	20.92	2.19E-01
cg00090648	-27.74	13.02	3.32E-02	8.35	33.27	8.02E-01	-21.79	134.54	8.71E-01	-27.32	15.40	7.61E-02	-75.90	37.24	4.16E-02
cg15838320	-11.24	5.28	3.32E-02	-18.15	9.28	5.05E-02	-14.93	13.10	2.54E-01	-4.37	13.17	7.40E-01	-6.35	8.86	4.73E-01
cg15125891	-10.32	4.85	3.32E-02	-28.50	13.07	2.92E-02	-4.60	20.31	8.21E-01	-6.52	5.74	2.55E-01	-16.25	16.05	3.11E-01
cg12044828	17.87	8.39	3.32E-02	11.52	13.50	3.94E-01	36.65	26.98	1.74E-01	50.23	23.97	3.61E-02	9.44	13.36	4.80E-01
cg12699321	-7.36	3.46	3.32E-02	-6.12	7.63	4.23E-01	-8.92	13.11	4.96E-01	-7.10	4.49	1.14E-01	-9.64	9.48	3.09E-01
cg05133340	11.38	5.34	3.32E-02	15.12	7.85	5.40E-02	15.08	11.91	2.05E-01	15.84	14.38	2.71E-01	-4.33	12.04	7.19E-01
cg15773539	-17.24	8.09	3.32E-02	-20.88	11.87	7.87E-02	5.27	17.18	7.59E-01	-14.62	20.35	4.72E-01	-38.60	19.36	4.62E-02

cg15785898	7.55	3.55	3.32E-02	10.62	5.11	3.78E-02	8.60	7.71	2.65E-01	5.15	11.52	6.55E-01	0.66	7.67	9.31E-01
cg17285709	15.87	7.45	3.32E-02	3.91	12.97	7.63E-01	10.46	14.09	4.58E-01	29.22	18.85	1.21E-01	30.32	15.42	4.92E-02
cg24834740	29.84	14.01	3.32E-02	36.93	16.83	2.82E-02	-51.05	53.34	3.38E-01	31.76	61.48	6.05E-01	32.93	32.52	3.11E-01
cg20980653	-8.17	3.84	3.32E-02	-7.85	6.48	2.26E-01	-2.85	7.43	7.01E-01	-20.10	11.26	7.42E-02	-8.71	7.42	2.40E-01
cg17939295	10.92	5.13	3.32E-02	1.61	7.32	8.26E-01	-2.75	14.77	8.52E-01	20.95	6.71	1.80E-03	13.88	6.85	4.27E-02
cg18721838	-8.30	3.90	3.32E-02	-3.01	9.36	7.47E-01	2.20	13.46	8.70E-01	-10.17	5.10	4.61E-02	-12.77	9.81	1.93E-01
cg22004151	-50.85	23.88	3.32E-02	-47.21	28.58	9.86E-02	-185.63	113.80	1.03E-01	-30.55	84.81	7.19E-01	-40.87	56.51	4.70E-01
cg11978048	-38.22	17.95	3.32E-02	-44.58	48.02	3.53E-01	-124.61	212.88	5.58E-01	-29.05	20.28	1.52E-01	-119.37	67.87	7.86E-02
cg07532965	-17.93	8.42	3.32E-02	-50.35	20.97	1.63E-02	-20.79	43.23	6.31E-01	-12.83	9.51	1.78E-01	-1.30	26.86	9.62E-01
cg11193201	-9.99	4.69	3.32E-02	-18.68	10.11	6.47E-02	8.42	20.36	6.79E-01	-8.43	5.82	1.48E-01	-11.52	16.39	4.82E-01
cg26322137	-10.40	4.88	3.32E-02	-6.60	14.85	6.57E-01	3.94	24.04	8.70E-01	-10.56	5.63	6.06E-02	-19.51	15.67	2.13E-01
cg25533154	10.30	4.84	3.32E-02	10.25	8.20	2.11E-01	5.03	16.87	7.66E-01	11.97	7.55	1.13E-01	8.83	12.10	4.65E-01
cg17207736	6.37	2.99	3.32E-02	6.23	4.14	1.32E-01	12.09	7.11	8.92E-02	2.89	7.37	6.95E-01	3.69	8.12	6.49E-01
cg18528593	13.69	6.43	3.32E-02	6.22	20.45	7.61E-01	-14.78	28.61	6.05E-01	15.72	7.25	3.00E-02	22.71	25.46	3.72E-01
cg03072035	6.44	3.02	3.32E-02	6.32	3.91	1.06E-01	15.93	8.20	5.20E-02	1.44	8.62	8.67E-01	2.24	7.95	7.78E-01
cg08682866	-16.18	7.60	3.32E-02	-14.66	21.33	4.92E-01	-4.97	38.15	8.96E-01	-13.46	8.60	1.17E-01	-69.52	33.39	3.74E-02
cg23535137	-22.95	10.78	3.32E-02	-17.43	13.15	1.85E-01	2.09	37.53	9.56E-01	17.28	63.42	7.85E-01	-55.12	23.20	1.75E-02
cg24989962	13.35	6.27	3.32E-02	28.50	13.83	3.93E-02	9.55	17.63	5.88E-01	5.54	10.09	5.83E-01	14.69	11.80	2.13E-01
cg02983043	-13.94	6.55	3.32E-02	-18.48	16.77	2.70E-01	-49.63	37.96	1.91E-01	-9.57	7.55	2.05E-01	-37.33	25.56	1.44E-01
cg07783458	17.64	8.29	3.32E-02	-0.60	12.74	9.62E-01	0.52	31.33	9.87E-01	25.75	7.89	1.10E-03	34.53	20.63	9.41E-02
cg10689404	10.81	5.08	3.32E-02	21.73	7.67	4.60E-03	10.77	10.35	2.98E-01	0.41	11.91	9.72E-01	3.30	8.82	7.09E-01
cg03798217	-10.97	5.15	3.32E-02	-5.15	15.49	7.39E-01	-5.21	22.35	8.16E-01	-12.03	5.81	3.85E-02	-13.31	22.92	5.62E-01
cg04332513	-30.54	14.34	3.32E-02	-17.58	38.32	6.46E-01	-322.90	179.24	7.16E-02	-30.81	16.12	5.60E-02	-26.23	57.67	6.49E-01
cg00121876	5.85	2.75	3.32E-02	3.68	3.70	3.20E-01	12.72	6.55	5.23E-02	10.84	13.06	4.07E-01	4.82	5.73	4.00E-01
cg04798314	1.41	0.66	3.33E-02	0.98	0.95	3.00E-01	1.56	1.57	3.20E-01	1.68	1.71	3.25E-01	2.13	1.52	1.61E-01
cg21546300	19.66	9.24	3.33E-02	35.87	16.18	2.66E-02	17.21	16.41	2.94E-01	-2.65	18.81	8.88E-01	27.16	27.09	3.16E-01
cg14709766	-29.69	13.94	3.33E-02	-12.07	29.01	6.77E-01	-172.14	187.30	3.58E-01	-32.52	16.74	5.20E-02	-48.54	52.85	3.58E-01
cg18116971	14.98	7.04	3.33E-02	37.93	13.43	4.73E-03	9.25	16.29	5.70E-01	9.37	10.29	3.63E-01	6.98	11.35	5.39E-01
cg21462732	33.20	15.59	3.33E-02	38.14	21.95	8.23E-02	58.26	43.99	1.85E-01	87.49	53.48	1.02E-01	-2.83	29.22	9.23E-01
cg24186506	4.95	2.33	3.33E-02	3.80	3.77	3.14E-01	9.43	5.10	6.48E-02	4.94	4.31	2.52E-01	0.91	6.73	8.93E-01
cg17895496	16.86	7.92	3.33E-02	43.05	21.22	4.25E-02	3.49	38.66	9.28E-01	12.63	9.15	1.67E-01	18.12	30.19	5.48E-01
cg03730114	-22.68	10.65	3.33E-02	-31.79	19.87	1.10E-01	-16.69	27.39	5.42E-01	-19.36	17.17	2.59E-01	-20.24	25.39	4.25E-01
cg13934625	-13.80	6.48	3.33E-02	-10.64	7.14	1.36E-01	0.22	13.78	9.87E-01	-43.85	18.34	1.68E-02	-16.25	11.05	1.41E-01
cg00250500	15.14	7.11	3.33E-02	36.06	14.87	1.53E-02	25.54	25.88	3.24E-01	6.71	10.37	5.18E-01	8.23	14.58	5.72E-01
cg08145231	-23.27	10.93	3.33E-02	-23.65	15.28	1.22E-01	-19.45	18.12	2.83E-01	-35.68	72.94	6.25E-01	-32.29	34.26	3.46E-01

cg14239983	-9.75	4.58	3.33E-02	-2.85	7.14	6.90E-01	-11.91	10.92	2.76E-01	-16.02	10.99	1.45E-01	-15.48	9.37	9.85E-02
cg03650342	24.13	11.34	3.33E-02	20.74	33.61	5.37E-01	33.49	134.77	8.04E-01	29.93	12.93	2.06E-02	-13.28	34.09	6.97E-01
cg03885271	16.20	7.61	3.33E-02	30.51	20.60	1.39E-01	21.63	35.92	5.47E-01	15.42	9.53	1.06E-01	6.82	17.87	7.03E-01
cg12008350	16.87	7.92	3.33E-02	22.45	11.90	5.93E-02	24.52	18.95	1.96E-01	3.60	14.96	8.10E-01	15.98	24.91	5.21E-01
cg01714749	10.94	5.14	3.33E-02	11.59	9.90	2.42E-01	3.76	10.73	7.26E-01	9.73	8.66	2.61E-01	23.67	13.31	7.54E-02
cg00000236	9.92	4.66	3.33E-02	11.20	8.41	1.83E-01	25.67	11.15	2.13E-02	7.78	5.38	1.48E-01	-6.57	13.86	6.36E-01
cg05286653	10.42	4.89	3.33E-02	5.80	6.58	3.78E-01	18.63	10.53	7.70E-02	2.06	25.70	9.36E-01	16.00	11.10	1.49E-01
cg26476925	6.79	3.19	3.33E-02	5.56	3.81	1.45E-01	17.96	8.74	3.99E-02	-1.01	12.34	9.35E-01	5.78	10.12	5.68E-01
cg13795120	-37.97	17.84	3.33E-02	-31.91	23.42	1.73E-01	-62.41	71.12	3.80E-01	-32.49	57.59	5.73E-01	-47.57	34.92	1.73E-01
cg06059056	17.95	8.43	3.33E-02	53.91	29.15	6.44E-02	13.83	54.40	7.99E-01	16.18	9.55	9.02E-02	4.35	25.14	8.63E-01
cg15615645	8.45	3.97	3.33E-02	9.98	6.28	1.12E-01	18.64	8.99	3.81E-02	3.04	7.42	6.81E-01	-0.39	11.52	9.73E-01
cg08641514	-15.94	7.49	3.33E-02	-22.67	20.94	2.79E-01	-22.66	39.49	5.66E-01	-18.14	8.64	3.59E-02	16.48	25.72	5.22E-01
cg19313373	-10.77	5.06	3.33E-02	-8.01	14.60	5.83E-01	-17.44	21.97	4.27E-01	-9.15	5.88	1.20E-01	-24.15	17.07	1.57E-01
cg08767627	-7.79	3.66	3.33E-02	-15.48	7.21	3.17E-02	-5.11	8.78	5.60E-01	-2.49	5.54	6.53E-01	-13.81	10.07	1.70E-01
cg07893968	16.46	7.73	3.33E-02	28.32	19.81	1.53E-01	-12.72	30.03	6.72E-01	14.24	9.16	1.20E-01	41.26	29.49	1.62E-01
cg26256916	-20.96	9.85	3.33E-02	-22.17	12.25	7.03E-02	-11.78	26.91	6.61E-01	-48.73	54.71	3.73E-01	-18.55	22.75	4.15E-01
cg04392266	-4.97	2.33	3.33E-02	-4.22	3.06	1.68E-01	-6.79	5.34	2.04E-01	-10.10	7.15	1.58E-01	-1.17	6.69	8.61E-01
cg02128417	-15.14	7.11	3.33E-02	-2.12	19.34	9.13E-01	-11.49	28.46	6.86E-01	-15.78	8.33	5.81E-02	-36.00	26.35	1.72E-01
cg15432249	21.54	10.12	3.33E-02	-12.02	29.72	6.86E-01	-92.22	106.89	3.88E-01	26.21	11.12	1.84E-02	44.05	47.15	3.50E-01
cg04780380	-8.09	3.80	3.33E-02	-7.40	5.63	1.89E-01	-15.62	10.06	1.21E-01	0.63	8.01	9.37E-01	-14.94	9.05	9.88E-02
cg01408194	-44.55	20.93	3.33E-02	-49.11	30.48	1.07E-01	-40.99	84.68	6.28E-01	-76.73	35.99	3.30E-02	33.34	52.06	5.22E-01
cg23490246	-10.27	4.82	3.33E-02	-10.42	7.36	1.57E-01	-13.49	11.15	2.26E-01	-7.53	12.23	5.38E-01	-9.20	10.13	3.64E-01
cg05991009	5.64	2.65	3.33E-02	4.15	3.34	2.14E-01	11.40	6.86	9.66E-02	12.47	8.43	1.39E-01	0.78	7.53	9.17E-01
cg13676726	15.55	7.30	3.33E-02	12.01	16.78	4.74E-01	35.14	19.32	6.89E-02	14.16	10.67	1.85E-01	8.10	16.38	6.21E-01
cg01458686	37.37	17.56	3.33E-02	57.51	27.45	3.62E-02	21.19	51.27	6.79E-01	13.32	36.14	7.13E-01	34.58	36.03	3.37E-01
cg19096825	24.54	11.53	3.33E-02	12.74	10.71	2.34E-01	14.06	21.07	5.04E-01	20.37	31.40	5.17E-01	60.96	20.55	3.02E-03
cg11996860	7.04	3.31	3.33E-02	7.20	4.43	1.04E-01	6.75	8.09	4.04E-01	13.80	10.09	1.72E-01	2.43	8.12	7.65E-01
cg23344257	23.24	10.92	3.33E-02	13.03	16.39	4.26E-01	-20.10	50.33	6.90E-01	86.25	49.18	7.95E-02	30.79	16.11	5.60E-02
cg22043381	9.62	4.52	3.33E-02	12.17	6.23	5.07E-02	16.09	10.75	1.34E-01	12.87	8.22	1.17E-01	-7.89	10.54	4.54E-01
cg16479102	-5.59	2.63	3.33E-02	-2.29	3.33	4.92E-01	-2.50	6.50	7.00E-01	-10.10	6.31	1.09E-01	-12.94	5.83	2.64E-02
cg01601016	-33.24	15.62	3.33E-02	-36.35	20.19	7.17E-02	-159.26	90.84	7.96E-02	1.35	81.62	9.87E-01	-20.33	26.98	4.51E-01
cg23180941	21.77	10.23	3.34E-02	34.80	19.27	7.09E-02	43.30	31.00	1.62E-01	3.90	14.76	7.92E-01	41.70	28.51	1.44E-01
cg24850445	30.80	14.48	3.34E-02	4.47	19.61	8.20E-01	29.55	20.80	1.55E-01	19.95	11.01	6.99E-02	89.03	26.22	6.85E-04
cg01822573	-22.61	10.63	3.34E-02	-46.79	27.71	9.13E-02	-80.84	98.70	4.13E-01	-16.06	12.11	1.85E-01	-34.00	39.79	3.93E-01
cg05753589	13.20	6.20	3.34E-02	12.48	12.14	3.04E-01	-0.66	21.51	9.75E-01	14.52	8.97	1.05E-01	17.20	14.73	2.43E-01

cg06342430	-8.23	3.87	3.34E-02	-3.00	9.43	7.51E-01	-20.48	14.00	1.44E-01	-7.51	4.77	1.15E-01	-12.69	12.49	3.10E-01
cg18042593	-19.04	8.95	3.34E-02	-22.47	19.57	2.51E-01	-11.73	42.99	7.85E-01	-11.72	7.99	1.42E-01	-61.91	26.36	1.89E-02
cg07742017	-12.45	5.85	3.34E-02	-25.70	14.31	7.25E-02	-33.99	37.05	3.59E-01	-7.85	7.03	2.64E-01	-16.29	17.35	3.48E-01
cg26764200	39.49	18.56	3.34E-02	62.92	21.35	3.21E-03	-2.87	51.74	9.56E-01	62.38	54.68	2.54E-01	-2.35	35.57	9.47E-01
cg06057656	9.83	4.62	3.34E-02	10.68	7.12	1.34E-01	12.25	10.22	2.30E-01	6.81	10.50	5.17E-01	8.37	10.87	4.41E-01
cg26711230	-15.76	7.41	3.34E-02	-28.92	13.03	2.65E-02	-1.87	24.11	9.38E-01	-9.88	11.30	3.82E-01	-13.07	18.94	4.90E-01
cg07667469	-10.22	4.81	3.34E-02	-13.97	10.03	1.63E-01	-6.12	23.18	7.92E-01	-10.87	6.18	7.83E-02	-1.38	13.77	9.20E-01
cg08340737	11.53	5.42	3.34E-02	20.85	9.97	3.64E-02	-0.34	14.48	9.81E-01	15.76	11.63	1.75E-01	5.73	9.20	5.34E-01
cg22576950	-8.79	4.13	3.34E-02	-10.20	8.26	2.17E-01	-3.30	10.61	7.56E-01	-8.45	6.67	2.05E-01	-11.66	8.93	1.92E-01
cg26740109	-5.79	2.72	3.34E-02	-7.21	4.92	1.42E-01	-3.40	6.72	6.13E-01	-6.19	4.22	1.42E-01	-3.91	8.05	6.27E-01
cg15827387	22.57	10.61	3.34E-02	52.22	29.18	7.35E-02	23.84	96.88	8.06E-01	20.94	11.94	7.94E-02	-17.67	41.37	6.69E-01
cg08239041	27.34	12.85	3.34E-02	15.91	20.12	4.29E-01	67.98	26.21	9.51E-03	11.01	9.43	2.43E-01	48.85	29.43	9.69E-02
cg12325588	-43.25	20.33	3.34E-02	-76.93	66.17	2.45E-01	-362.66	261.80	1.66E-01	-36.48	22.42	1.04E-01	-49.23	73.04	5.00E-01
cg20760920	-11.83	5.56	3.34E-02	-24.04	9.78	1.39E-02	-5.44	12.43	6.62E-01	-4.76	11.20	6.71E-01	-7.78	11.58	5.02E-01
cg19702779	19.43	9.13	3.34E-02	24.27	10.90	2.60E-02	21.91	36.52	5.49E-01	-28.22	49.69	5.70E-01	9.81	20.32	6.29E-01
cg14909730	14.37	6.76	3.34E-02	26.19	11.43	2.20E-02	-1.51	16.70	9.28E-01	3.42	10.77	7.51E-01	23.68	12.05	4.95E-02
cg27241559	-32.14	15.11	3.34E-02	-71.76	38.50	6.24E-02	-26.75	115.64	8.17E-01	-22.02	17.48	2.08E-01	-51.38	53.02	3.32E-01
cg21789008	-7.64	3.59	3.34E-02	-13.39	4.98	7.23E-03	-5.71	9.01	5.26E-01	4.80	16.97	7.77E-01	-0.65	6.60	9.21E-01
cg27116787	26.66	12.53	3.34E-02	38.90	45.19	3.89E-01	-146.62	192.29	4.46E-01	26.16	13.57	5.39E-02	29.99	48.75	5.38E-01
cg08670658	5.75	2.70	3.34E-02	4.24	3.33	2.03E-01	12.15	7.42	1.01E-01	8.01	8.99	3.73E-01	5.29	7.92	5.04E-01
cg17827477	-11.36	5.34	3.34E-02	-9.60	7.92	2.25E-01	-14.65	13.77	2.87E-01	-17.16	15.66	2.73E-01	-10.02	10.12	3.22E-01
cg25140795	17.33	8.15	3.34E-02	-1.45	16.78	9.31E-01	29.73	16.61	7.35E-02	22.10	12.56	7.85E-02	11.89	25.39	6.40E-01
cg18321976	19.84	9.33	3.34E-02	32.57	19.03	8.70E-02	4.24	37.53	9.10E-01	22.41	13.77	1.04E-01	6.12	19.09	7.48E-01
cg08792703	8.34	3.92	3.34E-02	11.93	5.58	3.26E-02	10.38	8.98	2.48E-01	-2.72	10.36	7.93E-01	5.02	9.44	5.95E-01
cg19582265	8.48	3.98	3.34E-02	14.11	7.81	7.06E-02	3.88	23.10	8.67E-01	7.14	5.10	1.61E-01	3.24	12.70	7.99E-01
cg14604066	-7.68	3.61	3.34E-02	-9.39	4.91	5.56E-02	2.81	8.83	7.50E-01	-8.13	10.19	4.25E-01	-12.32	8.86	1.64E-01
cg26298855	-18.88	8.88	3.34E-02	-15.79	16.09	3.26E-01	-16.65	20.99	4.28E-01	-15.21	15.03	3.12E-01	-34.46	21.66	1.12E-01
cg15917625	16.77	7.89	3.34E-02	17.87	11.88	1.33E-01	21.57	23.53	3.59E-01	21.76	20.01	2.77E-01	10.62	14.61	4.67E-01
cg15632936	-6.85	3.22	3.34E-02	-7.17	4.66	1.24E-01	-5.74	8.19	4.84E-01	-10.96	9.14	2.31E-01	-4.83	6.51	4.58E-01
cg14197071	6.29	2.96	3.34E-02	4.38	4.53	3.33E-01	1.44	4.09	7.24E-01	9.94	4.52	2.77E-02	20.01	9.96	4.45E-02
cg07660838	17.07	8.03	3.34E-02	19.64	11.71	9.35E-02	12.82	21.11	5.43E-01	9.60	20.29	6.36E-01	19.57	16.76	2.43E-01
cg02383008	-26.30	12.37	3.34E-02	-20.76	14.61	1.55E-01	-85.25	50.11	8.89E-02	57.39	75.48	4.47E-01	-39.70	27.94	1.55E-01
cg06819865	67.15	31.58	3.35E-02	112.58	47.82	1.86E-02	-26.13	158.21	8.69E-01	60.61	67.50	3.69E-01	19.13	57.14	7.38E-01
cg10989763	16.04	7.54	3.35E-02	27.58	12.53	2.78E-02	16.92	18.88	3.70E-01	30.22	25.98	2.45E-01	2.05	12.02	8.65E-01
cg06696963	18.91	8.89	3.35E-02	13.73	16.46	4.04E-01	15.98	16.04	3.19E-01	16.65	19.01	3.81E-01	34.84	20.83	9.44E-02

cg26271776	-8.85	4.16	3.35E-02	-15.15	6.61	2.20E-02	-1.28	9.64	8.95E-01	-11.63	8.85	1.89E-01	-0.20	9.40	9.83E-01
cg17739181	15.14	7.12	3.35E-02	14.91	19.14	4.36E-01	35.68	20.45	8.10E-02	10.93	8.60	2.04E-01	22.73	30.07	4.50E-01
cg07675031	6.99	3.29	3.35E-02	9.47	4.23	2.51E-02	7.60	6.97	2.75E-01	5.29	13.43	6.93E-01	-6.62	9.80	4.99E-01
cg07262585	22.16	10.42	3.35E-02	17.85	24.03	4.58E-01	25.97	52.42	6.20E-01	16.13	12.73	2.05E-01	67.96	32.54	3.67E-02
cg24206362	38.81	18.25	3.35E-02	61.49	36.37	9.09E-02	-81.73	164.91	6.20E-01	29.99	23.30	1.98E-01	48.46	52.19	3.53E-01
cg07532183	39.89	18.76	3.35E-02	42.75	23.52	6.91E-02	61.90	70.03	3.77E-01	-5.49	61.19	9.29E-01	44.22	42.15	2.94E-01
cg02585702	32.79	15.42	3.35E-02	53.14	21.61	1.39E-02	-27.71	43.93	5.28E-01	61.40	34.63	7.62E-02	19.43	19.98	3.31E-01
cg06271850	14.44	6.79	3.35E-02	22.06	10.29	3.20E-02	16.66	13.28	2.09E-01	-13.98	20.18	4.89E-01	10.81	15.59	4.88E-01
cg08962087	16.41	7.72	3.35E-02	22.01	10.31	3.28E-02	9.82	27.22	7.18E-01	15.56	21.46	4.69E-01	5.56	16.09	7.30E-01
cg10640731	39.95	18.79	3.35E-02	36.30	26.39	1.69E-01	41.04	73.78	5.78E-01	88.47	72.64	2.23E-01	35.89	31.26	2.51E-01
cg20369763	18.93	8.90	3.35E-02	38.95	19.89	5.01E-02	-96.46	93.72	3.03E-01	17.57	10.92	1.08E-01	2.51	25.10	9.20E-01
cg17640744	8.08	3.80	3.35E-02	8.50	5.00	8.92E-02	5.23	7.12	4.63E-01	29.50	13.13	2.46E-02	2.37	7.88	7.64E-01
cg26512635	-8.56	4.03	3.35E-02	-14.02	10.17	1.68E-01	-0.60	13.48	9.65E-01	-6.10	4.95	2.17E-01	-24.81	13.33	6.27E-02
cg11185188	19.37	9.11	3.35E-02	22.68	19.25	2.39E-01	38.06	21.29	7.38E-02	7.65	13.14	5.60E-01	32.54	27.24	2.32E-01
cg10933457	-3.64	1.71	3.35E-02	-7.03	4.42	1.11E-01	-5.59	2.47	2.34E-02	-6.11	11.81	6.05E-01	-0.05	2.62	9.84E-01
cg10629350	-13.49	6.35	3.35E-02	-16.04	12.38	1.95E-01	-13.16	28.47	6.44E-01	-14.57	8.23	7.69E-02	0.35	20.77	9.86E-01
cg23190093	16.93	7.96	3.35E-02	38.32	19.66	5.12E-02	17.80	34.58	6.07E-01	9.76	9.43	3.01E-01	39.19	30.14	1.93E-01
cg07851675	17.72	8.34	3.35E-02	13.77	10.11	1.73E-01	3.59	20.23	8.59E-01	49.30	17.64	5.18E-03	10.57	12.57	4.00E-01
cg01956472	16.71	7.86	3.35E-02	16.33	15.35	2.87E-01	-2.13	18.82	9.10E-01	26.65	11.74	2.32E-02	7.46	23.13	7.47E-01
cg14662210	-7.71	3.63	3.35E-02	-9.73	7.34	1.85E-01	-17.62	13.32	1.86E-01	-3.11	4.94	5.29E-01	-16.36	9.56	8.69E-02
cg20083730	-31.32	14.73	3.35E-02	-39.89	23.85	9.45E-02	-56.26	50.92	2.69E-01	-7.64	27.02	7.77E-01	-38.43	30.24	2.04E-01
cg01153946	-8.83	4.15	3.35E-02	-8.16	7.23	2.59E-01	-4.93	10.03	6.23E-01	-11.61	7.94	1.43E-01	-9.37	8.75	2.84E-01
cg24039778	43.49	20.46	3.35E-02	42.54	26.85	1.13E-01	218.70	147.25	1.37E-01	72.86	73.80	3.23E-01	27.76	35.98	4.40E-01
cg19577074	-8.75	4.12	3.35E-02	-11.89	7.51	1.13E-01	-7.64	9.89	4.40E-01	-2.94	7.30	6.87E-01	-14.06	9.05	1.20E-01
cg12600843	-10.40	4.89	3.35E-02	-13.98	9.45	1.39E-01	2.06	11.25	8.55E-01	-13.27	8.22	1.07E-01	-12.38	11.24	2.71E-01
cg22310279	27.25	12.82	3.35E-02	15.93	20.49	4.37E-01	-24.85	71.95	7.30E-01	41.80	19.29	3.02E-02	24.72	34.86	4.78E-01
cg22462856	18.83	8.86	3.35E-02	7.75	21.37	7.17E-01	47.93	46.45	3.02E-01	20.01	10.37	5.36E-02	18.56	35.69	6.03E-01
cg22008075	21.49	10.11	3.35E-02	29.94	21.93	1.72E-01	22.79	25.65	3.74E-01	9.51	13.68	4.87E-01	69.24	32.99	3.58E-02
cg10045321	14.74	6.93	3.35E-02	16.24	17.40	3.51E-01	30.25	21.66	1.63E-01	14.35	8.79	1.02E-01	1.06	20.36	9.58E-01
cg00045118	-11.41	5.37	3.35E-02	-14.65	7.89	6.33E-02	1.84	13.06	8.88E-01	-16.06	13.19	2.23E-01	-11.27	11.94	3.45E-01
cg25984249	20.66	9.72	3.35E-02	26.66	16.89	1.15E-01	15.92	26.18	5.43E-01	2.04	17.37	9.06E-01	41.32	20.83	4.73E-02
cg23141914	-15.02	7.07	3.35E-02	-38.35	19.73	5.19E-02	-11.07	28.19	6.94E-01	-12.75	8.27	1.23E-01	-1.30	25.00	9.58E-01
cg02539793	12.71	5.98	3.35E-02	16.02	8.01	4.56E-02	33.86	15.18	2.57E-02	9.27	11.75	4.30E-01	0.00	10.13	1.00E+00
cg07100000	18.15	8.54	3.35E-02	17.51	12.32	1.55E-01	12.99	22.60	5.65E-01	53.18	28.75	6.44E-02	11.05	15.90	4.87E-01
cg23424933	14.49	6.82	3.35E-02	3.78	12.66	7.65E-01	19.17	21.22	3.66E-01	18.99	10.39	6.78E-02	18.37	16.20	2.57E-01

cg03355327	19.08	8.98	3.35E-02	32.49	18.78	8.37E-02	-2.10	19.02	9.12E-01	16.87	13.57	2.14E-01	42.61	26.91	1.13E-01
cg18087953	-14.78	6.95	3.36E-02	-24.41	8.47	3.95E-03	7.60	13.15	5.64E-01	-17.56	15.28	2.50E-01	-17.31	11.82	1.43E-01
cg23852354	-10.75	5.06	3.36E-02	-11.05	9.24	2.32E-01	-6.57	24.09	7.85E-01	-14.01	6.94	4.35E-02	2.33	14.30	8.70E-01
cg14783987	13.54	6.37	3.36E-02	-5.78	17.66	7.44E-01	30.05	26.48	2.56E-01	16.19	7.34	2.75E-02	6.17	26.30	8.14E-01
cg22286184	-44.06	20.73	3.36E-02	-47.36	25.17	5.99E-02	58.41	140.84	6.78E-01	-45.87	75.64	5.44E-01	-43.35	43.71	3.21E-01
cg26110130	-11.91	5.60	3.36E-02	-6.57	8.33	4.31E-01	-16.09	14.73	2.75E-01	-25.75	19.13	1.78E-01	-13.87	9.95	1.63E-01
cg14064503	-8.30	3.91	3.36E-02	-7.64	5.93	1.98E-01	-5.29	11.00	6.31E-01	-18.85	8.57	2.79E-02	-1.74	8.12	8.31E-01
cg11688376	-10.03	4.72	3.36E-02	-5.92	5.92	3.18E-01	4.18	12.32	7.34E-01	-11.71	8.35	1.61E-01	-22.06	8.17	6.93E-03
cg10959711	21.01	9.89	3.36E-02	26.02	20.45	2.03E-01	62.20	29.27	3.36E-02	10.69	13.62	4.33E-01	17.55	27.92	5.30E-01
cg01690029	-50.43	23.73	3.36E-02	-81.76	66.22	2.17E-01	-330.05	265.74	2.14E-01	-36.81	27.61	1.82E-01	-80.91	67.13	2.28E-01
cg15526094	-10.45	4.92	3.36E-02	-12.23	7.77	1.16E-01	-10.10	9.05	2.65E-01	-6.87	12.27	5.75E-01	-10.24	12.99	4.30E-01
cg23280720	-5.93	2.79	3.36E-02	-4.84	3.64	1.83E-01	-15.02	7.30	3.96E-02	-4.31	8.56	6.14E-01	-2.67	7.04	7.04E-01
cg18109389	-12.33	5.80	3.36E-02	-14.37	14.74	3.30E-01	-3.34	18.39	8.56E-01	-14.08	7.15	4.89E-02	-5.70	19.69	7.72E-01
cg25364273	14.90	7.01	3.36E-02	11.56	10.75	2.82E-01	4.70	11.67	6.87E-01	20.19	14.48	1.63E-01	47.77	21.63	2.72E-02
cg02322400	7.21	3.39	3.36E-02	7.15	5.20	1.69E-01	16.11	9.35	8.49E-02	3.64	6.49	5.76E-01	6.19	8.22	4.52E-01
cg10750945	-18.60	8.75	3.36E-02	-9.39	21.65	6.64E-01	45.38	81.57	5.78E-01	-18.91	10.00	5.88E-02	-52.56	35.97	1.44E-01
cg01759437	15.19	7.15	3.36E-02	12.78	18.68	4.94E-01	-32.25	53.15	5.44E-01	17.67	8.31	3.35E-02	8.69	23.02	7.06E-01
cg11697474	15.77	7.42	3.36E-02	7.57	12.97	5.60E-01	18.91	13.92	1.74E-01	5.61	16.16	7.28E-01	37.82	17.58	3.14E-02
cg00373279	-24.29	11.43	3.36E-02	-25.48	13.96	6.79E-02	-2.30	38.42	9.52E-01	21.67	71.55	7.62E-01	-35.09	24.64	1.54E-01
cg08691235	6.99	3.29	3.36E-02	5.11	4.37	2.42E-01	18.17	9.49	5.57E-02	8.92	8.41	2.89E-01	3.41	8.23	6.78E-01
cg25779483	7.98	3.76	3.36E-02	9.74	5.35	6.88E-02	11.95	8.56	1.62E-01	8.90	12.77	4.86E-01	0.49	7.86	9.50E-01
cg15015109	6.38	3.00	3.36E-02	4.67	3.69	2.06E-01	19.75	8.75	2.40E-02	7.27	9.88	4.61E-01	2.32	8.35	7.81E-01
cg13787356	16.01	7.54	3.36E-02	16.14	21.04	4.43E-01	53.24	30.09	7.68E-02	13.02	8.74	1.36E-01	14.07	29.51	6.34E-01
cg10332704	22.86	10.76	3.36E-02	20.51	18.26	2.61E-01	23.28	24.36	3.39E-01	16.03	18.90	3.96E-01	44.92	29.43	1.27E-01
cg20475322	19.99	9.41	3.36E-02	28.45	14.60	5.13E-02	12.10	29.53	6.82E-01	38.54	35.87	2.83E-01	10.36	14.61	4.78E-01
cg09891129	26.94	12.68	3.36E-02	54.12	41.37	1.91E-01	21.87	79.75	7.84E-01	18.30	15.05	2.24E-01	48.56	30.64	1.13E-01
cg05545454	-15.64	7.36	3.36E-02	-11.08	15.18	4.66E-01	5.55	28.73	8.47E-01	-20.21	9.76	3.83E-02	-14.60	20.43	4.75E-01
cg02202742	20.11	9.47	3.36E-02	4.92	12.67	6.98E-01	40.30	19.47	3.85E-02	8.06	28.06	7.74E-01	35.23	18.02	5.06E-02
cg04445512	13.79	6.49	3.36E-02	-2.80	15.63	8.58E-01	4.17	23.98	8.62E-01	20.02	8.09	1.33E-02	9.74	19.57	6.19E-01
cg04077417	-10.85	5.11	3.36E-02	-12.58	10.62	2.36E-01	-9.55	15.84	5.47E-01	-5.49	7.22	4.47E-01	-25.60	12.61	4.24E-02
cg03550773	8.70	4.10	3.36E-02	4.38	5.47	4.24E-01	14.60	8.28	7.77E-02	21.24	15.20	1.62E-01	9.27	11.75	4.30E-01
cg03629948	18.45	8.68	3.36E-02	32.69	15.04	2.98E-02	-0.94	14.83	9.49E-01	30.76	20.80	1.39E-01	16.81	22.45	4.54E-01
cg02560638	-37.91	17.85	3.36E-02	-41.47	19.82	3.64E-02	60.66	111.08	5.85E-01	2.47	85.04	9.77E-01	-49.95	51.64	3.33E-01
cg15068318	8.49	4.00	3.36E-02	8.79	5.19	9.02E-02	12.84	10.56	2.24E-01	14.48	11.70	2.16E-01	-1.82	10.49	8.63E-01
cg14597739	-5.74	2.70	3.36E-02	-4.21	3.43	2.20E-01	-8.61	8.19	2.93E-01	-15.14	10.33	1.43E-01	-5.72	6.03	3.42E-01

cg20676418	-28.37	13.36	3.36E-02	-29.02	16.81	8.42E-02	-7.24	41.91	8.63E-01	-18.63	49.77	7.08E-01	-40.89	30.27	1.77E-01
cg23521140	-25.91	12.20	3.36E-02	8.99	36.25	8.04E-01	-168.07	151.87	2.68E-01	-28.91	13.37	3.06E-02	-37.34	55.92	5.04E-01
cg23606922	24.14	11.37	3.37E-02	27.01	29.65	3.62E-01	69.22	138.63	6.18E-01	27.37	13.05	3.59E-02	-12.23	38.47	7.50E-01
cg07841173	11.79	5.55	3.37E-02	17.27	8.13	3.37E-02	3.79	15.01	8.01E-01	18.20	13.58	1.80E-01	0.82	11.55	9.43E-01
cg01803844	-14.04	6.61	3.37E-02	-12.41	13.02	3.41E-01	-2.50	17.52	8.86E-01	-15.31	9.94	1.23E-01	-23.51	16.62	1.57E-01
cg12494373	17.08	8.04	3.37E-02	16.76	18.84	3.74E-01	1.41	46.50	9.76E-01	18.74	9.74	5.44E-02	11.45	24.64	6.42E-01
cg23951816	-13.25	6.24	3.37E-02	-24.28	18.02	1.78E-01	-4.36	34.05	8.98E-01	-13.27	7.05	5.99E-02	2.85	24.52	9.08E-01
cg09172766	-34.52	16.25	3.37E-02	-39.46	45.45	3.85E-01	78.98	135.75	5.61E-01	-35.99	18.92	5.71E-02	-33.80	46.96	4.72E-01
cg27490875	-6.28	2.96	3.37E-02	-8.24	5.17	1.11E-01	-2.63	6.04	6.64E-01	-8.89	5.70	1.19E-01	-3.43	7.32	6.39E-01
cg22040039	13.86	6.52	3.37E-02	12.23	9.07	1.78E-01	25.07	13.90	7.12E-02	1.29	19.23	9.46E-01	12.63	17.00	4.57E-01
cg22344254	-16.71	7.87	3.37E-02	-28.54	13.90	4.01E-02	-30.44	17.09	7.49E-02	-2.84	14.60	8.46E-01	-1.70	18.65	9.28E-01
cg12940558	25.92	12.20	3.37E-02	60.46	31.45	5.46E-02	-33.76	140.84	8.11E-01	20.65	14.04	1.41E-01	16.97	41.58	6.83E-01
cg17315426	12.25	5.77	3.37E-02	7.09	7.10	3.18E-01	33.72	17.07	4.82E-02	-0.12	24.84	9.96E-01	21.65	13.89	1.19E-01
cg09773586	15.59	7.34	3.37E-02	22.95	8.86	9.61E-03	-18.24	29.96	5.43E-01	19.41	38.62	6.15E-01	1.11	15.72	9.44E-01
cg06019865	-32.18	15.15	3.37E-02	-36.88	18.37	4.47E-02	-23.92	50.71	6.37E-01	-27.05	57.20	6.36E-01	-19.12	37.80	6.13E-01
cg20809881	16.22	7.64	3.37E-02	22.66	11.05	4.03E-02	20.41	19.16	2.87E-01	-12.36	30.09	6.81E-01	9.86	13.98	4.80E-01
cg26982892	-17.11	8.06	3.37E-02	-21.59	20.16	2.84E-01	-14.37	27.79	6.05E-01	-16.70	9.88	9.11E-02	-14.78	26.57	5.78E-01
cg27557048	31.29	14.73	3.37E-02	37.92	17.62	3.14E-02	-13.80	81.62	8.66E-01	38.54	66.06	5.60E-01	15.15	31.50	6.31E-01
cg04336164	26.57	12.51	3.37E-02	30.68	24.38	2.08E-01	144.26	97.33	1.38E-01	19.95	15.89	2.09E-01	37.33	39.52	3.45E-01
cg02086690	-7.76	3.65	3.37E-02	-11.72	7.44	1.15E-01	-3.30	14.41	8.19E-01	-5.79	4.95	2.42E-01	-10.47	9.43	2.67E-01
cg02674639	-11.73	5.53	3.37E-02	-20.19	8.84	2.24E-02	-2.15	11.90	8.57E-01	0.43	14.52	9.76E-01	-13.85	11.08	2.11E-01
cg07991621	7.03	3.31	3.37E-02	7.66	4.33	7.68E-02	2.78	7.70	7.18E-01	-10.15	13.03	4.36E-01	14.16	7.06	4.50E-02
cg26015539	-7.72	3.64	3.37E-02	-3.82	7.55	6.13E-01	-1.42	15.33	9.26E-01	-10.01	4.64	3.08E-02	-6.18	11.66	5.96E-01
cg08325021	31.54	14.85	3.37E-02	50.06	40.37	2.15E-01	-115.72	169.45	4.95E-01	33.53	16.78	4.56E-02	-8.50	54.82	8.77E-01
cg14704758	22.83	10.75	3.37E-02	51.51	17.64	3.50E-03	18.51	27.42	5.00E-01	6.95	11.39	5.42E-01	22.27	19.13	2.44E-01
cg10885417	15.61	7.35	3.37E-02	0.42	19.60	9.83E-01	52.24	35.78	1.44E-01	16.34	8.53	5.55E-02	16.30	26.92	5.45E-01
cg26891210	4.56	2.15	3.37E-02	2.93	3.08	3.42E-01	11.08	5.17	3.19E-02	1.85	4.26	6.64E-01	8.60	7.25	2.36E-01
cg10164686	-50.78	23.91	3.37E-02	-63.44	32.58	5.15E-02	-71.03	117.74	5.46E-01	33.11	55.34	5.50E-01	-85.06	49.49	8.56E-02
cg15084499	21.55	10.15	3.37E-02	1.99	28.58	9.45E-01	-24.90	46.21	5.90E-01	28.30	11.84	1.68E-02	18.70	33.63	5.78E-01
cg08503913	-86.33	40.66	3.37E-02	-69.67	47.92	1.46E-01	194.33	187.86	3.01E-01	-170.97	65.04	8.57E-03	-71.77	64.99	2.69E-01
cg00405060	-38.22	18.00	3.37E-02	-58.93	23.10	1.07E-02	50.31	86.88	5.63E-01	-46.06	60.01	4.43E-01	-1.72	35.31	9.61E-01
cg03585912	11.85	5.58	3.37E-02	11.73	10.30	2.55E-01	8.18	13.65	5.49E-01	20.80	10.77	5.34E-02	5.38	10.71	6.16E-01
cg14819229	-12.51	5.89	3.37E-02	-11.23	11.39	3.24E-01	-21.29	13.94	1.27E-01	-13.12	8.78	1.35E-01	2.05	18.37	9.11E-01
cg02847384	-51.63	24.32	3.37E-02	-22.13	23.45	3.45E-01	-115.56	85.48	1.76E-01	-64.27	65.06	3.23E-01	-115.87	53.40	3.00E-02
cg07414984	19.73	9.29	3.37E-02	20.78	30.09	4.90E-01	-9.08	138.53	9.48E-01	18.43	10.12	6.84E-02	39.61	39.09	3.11E-01

cg23957643	13.78	6.49	3.37E-02	10.13	10.44	3.32E-01	28.53	13.69	3.72E-02	3.98	16.03	8.04E-01	12.46	13.70	3.63E-01
cg14308647	-10.30	4.85	3.37E-02	-0.34	10.50	9.75E-01	-32.77	21.51	1.28E-01	-10.50	6.51	1.07E-01	-15.13	11.40	1.85E-01
cg17627427	17.85	8.41	3.37E-02	-5.12	19.38	7.92E-01	33.37	42.33	4.30E-01	23.76	10.06	1.82E-02	12.30	30.74	6.89E-01
cg12583659	25.22	11.88	3.37E-02	20.30	43.05	6.37E-01	48.43	180.62	7.89E-01	26.50	12.66	3.64E-02	3.77	59.47	9.50E-01
cg27539233	8.69	4.09	3.37E-02	6.22	6.27	3.21E-01	12.59	8.41	1.35E-01	13.74	8.84	1.20E-01	0.94	11.69	9.36E-01
cg19591908	-7.47	3.52	3.37E-02	-12.89	6.64	5.22E-02	0.55	7.78	9.43E-01	-9.25	5.56	9.62E-02	-2.30	10.35	8.24E-01
cg19479086	-7.85	3.70	3.38E-02	-0.29	7.92	9.71E-01	-14.49	16.61	3.83E-01	-11.11	4.63	1.64E-02	0.20	12.01	9.87E-01
cg23676829	14.67	6.91	3.38E-02	4.72	14.67	7.48E-01	2.80	28.00	9.20E-01	16.97	8.77	5.30E-02	30.28	22.27	1.74E-01
cg17194668	14.89	7.01	3.38E-02	25.04	15.11	9.76E-02	29.38	18.87	1.19E-01	9.51	9.45	3.14E-01	2.08	22.69	9.27E-01
cg02980000	40.76	19.20	3.38E-02	8.14	24.46	7.39E-01	81.94	89.42	3.59E-01	65.03	61.99	2.94E-01	71.72	28.50	1.18E-02
cg00259046	5.88	2.77	3.38E-02	2.61	4.04	5.19E-01	7.84	5.59	1.60E-01	19.16	9.70	4.81E-02	5.74	6.15	3.51E-01
cg07185695	21.40	10.08	3.38E-02	29.55	15.68	5.94E-02	49.66	27.27	6.86E-02	11.35	32.62	7.28E-01	3.69	16.94	8.28E-01
cg26985203	6.20	2.92	3.38E-02	9.18	3.97	2.08E-02	7.43	6.35	2.42E-01	10.53	8.22	2.00E-01	-2.70	5.73	6.37E-01
cg02458062	-13.31	6.27	3.38E-02	-3.56	19.44	8.55E-01	8.91	26.90	7.41E-01	-15.60	7.06	2.71E-02	-21.27	27.47	4.39E-01
cg17882959	-31.35	14.77	3.38E-02	9.13	34.71	7.92E-01	-93.48	155.46	5.48E-01	-42.49	17.47	1.50E-02	-18.90	47.88	6.93E-01
cg00345025	-12.02	5.66	3.38E-02	-9.15	7.69	2.35E-01	-24.95	13.32	6.10E-02	11.65	21.60	5.90E-01	-16.08	12.39	1.95E-01
cg27211284	11.28	5.31	3.38E-02	12.21	8.38	1.45E-01	-5.72	15.38	7.10E-01	13.66	9.92	1.68E-01	16.33	12.13	1.78E-01
cg21400296	-7.86	3.70	3.38E-02	-13.10	8.79	1.36E-01	1.35	13.59	9.21E-01	-5.80	4.59	2.06E-01	-19.19	11.90	1.07E-01
cg03517913	-11.30	5.32	3.38E-02	-15.86	13.37	2.35E-01	15.19	19.85	4.44E-01	-11.93	6.53	6.76E-02	-18.51	16.42	2.60E-01
cg14076258	-8.11	3.82	3.38E-02	-6.36	6.58	3.34E-01	2.74	11.47	8.11E-01	-10.20	6.04	9.13E-02	-14.53	9.86	1.41E-01
cg17171407	8.44	3.98	3.38E-02	6.80	5.21	1.92E-01	6.91	9.40	4.62E-01	36.49	22.86	1.10E-01	10.31	8.73	2.38E-01
cg02032282	-13.31	6.27	3.38E-02	-22.80	10.92	3.68E-02	-6.17	14.65	6.73E-01	-3.66	14.50	8.01E-01	-13.25	11.44	2.47E-01
cg06676088	14.24	6.71	3.38E-02	8.64	15.09	5.67E-01	7.53	18.22	6.79E-01	18.39	8.75	3.56E-02	8.89	23.92	7.10E-01
cg11996390	17.93	8.44	3.38E-02	14.98	22.67	5.09E-01	-25.21	43.53	5.63E-01	17.83	9.80	6.88E-02	43.94	29.70	1.39E-01
cg25391820	15.14	7.13	3.38E-02	14.97	10.90	1.70E-01	22.93	16.71	1.70E-01	30.62	19.13	1.10E-01	1.19	14.24	9.33E-01
cg10582639	18.50	8.72	3.38E-02	18.11	12.39	1.44E-01	-6.18	18.96	7.45E-01	15.07	11.87	2.04E-01	45.65	17.05	7.40E-03
cg20315445	10.91	5.14	3.38E-02	10.97	9.95	2.70E-01	-1.97	12.73	8.77E-01	11.18	7.56	1.39E-01	28.94	15.61	6.38E-02
cg22763649	10.97	5.17	3.38E-02	16.08	7.59	3.42E-02	7.53	13.74	5.83E-01	11.04	12.85	3.90E-01	2.86	10.70	7.89E-01
cg12510999	9.56	4.51	3.38E-02	10.96	9.11	2.29E-01	23.90	13.87	8.49E-02	6.89	6.00	2.51E-01	5.54	15.33	7.18E-01
cg02499768	-10.21	4.81	3.38E-02	-15.17	6.50	1.97E-02	-11.16	11.36	3.26E-01	16.87	28.76	5.57E-01	-1.56	9.70	8.72E-01
cg04772947	-10.28	4.84	3.38E-02	-24.83	10.59	1.91E-02	-5.72	15.47	7.11E-01	-8.29	6.54	2.04E-01	0.21	12.79	9.87E-01
cg22387323	9.22	4.35	3.38E-02	6.89	5.44	2.05E-01	24.22	9.82	1.37E-02	-0.30	12.72	9.81E-01	6.17	11.40	5.89E-01
cg15706657	9.40	4.43	3.38E-02	4.94	6.12	4.19E-01	18.95	8.67	2.89E-02	2.82	15.57	8.56E-01	12.28	12.13	3.11E-01
cg05106699	-10.99	5.18	3.38E-02	-19.13	11.88	1.07E-01	9.16	23.43	6.96E-01	-7.92	6.66	2.35E-01	-19.30	13.11	1.41E-01
cg07414239	-30.71	14.47	3.38E-02	-18.84	44.68	6.73E-01	80.49	167.06	6.30E-01	-35.86	15.99	2.49E-02	0.72	55.50	9.90E-01

cg10135708	13.91	6.55	3.38E-02	5.25	14.56	7.18E-01	9.85	16.16	5.42E-01	18.62	8.85	3.54E-02	12.01	22.64	5.96E-01
cg11328875	-51.75	24.39	3.38E-02	-51.12	28.21	6.99E-02	-19.24	183.07	9.16E-01	46.22	92.17	6.16E-01	-99.84	60.12	9.68E-02
cg17752015	-18.67	8.80	3.38E-02	-24.54	26.43	3.53E-01	6.00	108.05	9.56E-01	-20.32	9.71	3.63E-02	11.66	35.72	7.44E-01
cg13996155	21.18	9.98	3.38E-02	44.29	33.58	1.87E-01	58.20	48.42	2.29E-01	21.27	12.06	7.78E-02	1.22	23.25	9.58E-01
cg03421440	-7.22	3.40	3.38E-02	-10.82	4.92	2.79E-02	-6.22	8.82	4.81E-01	3.22	9.62	7.38E-01	-6.14	6.83	3.69E-01
cg02495767	15.46	7.29	3.39E-02	15.04	18.20	4.09E-01	25.30	38.55	5.12E-01	12.54	8.55	1.42E-01	39.14	26.16	1.35E-01
cg24107988	-32.45	15.29	3.39E-02	-5.84	27.84	8.34E-01	-75.19	167.51	6.54E-01	-43.44	19.79	2.81E-02	-44.36	50.27	3.78E-01
cg09357934	5.79	2.73	3.39E-02	6.58	3.88	8.95E-02	9.49	6.82	1.64E-01	1.49	7.77	8.48E-01	3.72	5.79	5.21E-01
cg11923631	8.19	3.86	3.39E-02	7.10	5.24	1.76E-01	7.98	11.39	4.84E-01	4.56	9.41	6.28E-01	15.19	9.23	9.97E-02
cg26216243	-7.91	3.73	3.39E-02	-10.93	6.73	1.04E-01	-16.49	10.79	1.26E-01	-3.84	5.63	4.95E-01	-6.69	10.18	5.11E-01
cg04586126	5.73	2.70	3.39E-02	6.40	3.48	6.56E-02	10.47	6.45	1.04E-01	3.86	8.11	6.34E-01	-3.58	8.10	6.59E-01
cg21697512	6.12	2.88	3.39E-02	6.11	3.88	1.15E-01	8.42	7.52	2.63E-01	9.99	7.28	1.70E-01	-0.46	7.62	9.52E-01
cg13568334	-39.57	18.65	3.39E-02	-52.64	22.84	2.12E-02	-130.29	108.33	2.29E-01	14.44	59.26	8.07E-01	-9.98	41.25	8.09E-01
cg22694191	12.22	5.76	3.39E-02	11.35	9.99	2.56E-01	28.53	14.87	5.49E-02	4.11	10.71	7.01E-01	13.04	12.07	2.80E-01
cg02604290	20.86	9.83	3.39E-02	19.18	13.93	1.69E-01	2.97	29.66	9.20E-01	37.83	26.99	1.61E-01	22.99	19.32	2.34E-01
cg20686347	37.53	17.69	3.39E-02	42.86	24.19	7.64E-02	27.31	48.82	5.76E-01	19.37	53.05	7.15E-01	39.81	37.48	2.88E-01
cg00208504	16.68	7.86	3.39E-02	18.40	11.36	1.05E-01	41.43	25.73	1.07E-01	25.85	22.09	2.42E-01	2.41	14.32	8.67E-01
cg05320607	-45.87	21.62	3.39E-02	-48.38	26.58	6.88E-02	-86.80	105.31	4.10E-01	-31.21	78.13	6.90E-01	-35.55	46.11	4.41E-01
cg09133192	48.55	22.89	3.39E-02	50.07	30.89	1.05E-01	87.56	111.45	4.32E-01	73.64	50.06	1.41E-01	9.87	51.21	8.47E-01
cg01763709	18.31	8.63	3.39E-02	12.83	19.88	5.19E-01	-11.38	48.42	8.14E-01	19.43	10.41	6.21E-02	31.36	28.36	2.69E-01
cg11606261	6.49	3.06	3.39E-02	3.66	3.16	2.47E-01	12.42	6.76	6.61E-02	16.04	7.63	3.55E-02	0.88	7.06	9.01E-01
cg26375660	-16.25	7.66	3.39E-02	-10.33	16.04	5.20E-01	-26.44	29.63	3.72E-01	-20.32	9.88	3.97E-02	0.79	23.69	9.73E-01
cg02458483	17.38	8.19	3.39E-02	0.02	9.99	9.98E-01	29.98	11.76	1.08E-02	14.85	16.13	3.57E-01	30.22	14.70	3.97E-02
cg11311280	-8.65	4.08	3.39E-02	-21.05	9.29	2.35E-02	8.02	22.31	7.19E-01	-6.22	5.08	2.21E-01	-6.59	11.36	5.62E-01
cg07698804	-7.14	3.37	3.39E-02	-4.69	4.50	2.98E-01	-17.68	10.05	7.84E-02	-14.58	10.74	1.74E-01	-4.79	7.02	4.95E-01
cg04615850	19.43	9.16	3.39E-02	14.70	19.54	4.52E-01	30.71	17.42	7.80E-02	9.94	15.21	5.13E-01	29.14	24.42	2.33E-01
cg05939647	16.44	7.75	3.39E-02	10.82	15.61	4.88E-01	2.87	22.01	8.96E-01	18.25	10.51	8.23E-02	40.99	26.61	1.23E-01
cg05348321	16.79	7.92	3.39E-02	14.56	15.76	3.55E-01	13.88	15.61	3.74E-01	11.20	12.59	3.73E-01	53.78	25.67	3.61E-02
cg12385165	-8.53	4.02	3.39E-02	-5.69	5.88	3.33E-01	-18.98	9.24	3.99E-02	-6.41	11.82	5.88E-01	-6.74	8.45	4.25E-01
cg26476156	-8.79	4.14	3.39E-02	-5.06	8.18	5.36E-01	-14.36	14.57	3.25E-01	-9.29	5.59	9.62E-02	-10.83	12.36	3.81E-01
cg02839351	10.27	4.84	3.39E-02	5.04	6.21	4.17E-01	25.05	11.33	2.71E-02	9.61	17.80	5.89E-01	14.16	13.15	2.82E-01
cg25307641	15.13	7.14	3.39E-02	4.57	12.04	7.04E-01	22.01	12.73	8.39E-02	20.16	16.97	2.35E-01	19.33	17.96	2.82E-01
cg17566936	49.00	23.10	3.39E-02	55.89	19.30	3.78E-03	-46.56	80.35	5.62E-01	115.55	50.29	2.16E-02	10.19	44.94	8.21E-01
cg00446046	4.63	2.18	3.39E-02	3.13	3.18	3.26E-01	9.85	4.66	3.43E-02	1.85	4.64	6.90E-01	6.63	7.36	3.68E-01
cg09540102	-5.27	2.48	3.39E-02	-5.17	3.35	1.23E-01	-3.69	6.30	5.58E-01	-11.44	7.47	1.25E-01	-3.18	5.82	5.85E-01

cg21788624	-14.41	6.80	3.39E-02	-12.35	20.21	5.41E-01	-3.16	53.45	9.53E-01	-14.50	7.65	5.81E-02	-18.68	23.77	4.32E-01
cg17537935	-13.71	6.46	3.39E-02	-20.14	11.65	8.39E-02	-0.01	14.33	1.00E+00	-13.27	12.56	2.91E-01	-17.82	13.66	1.92E-01
cg23497644	-6.95	3.28	3.39E-02	-8.88	5.65	1.16E-01	-1.06	7.93	8.94E-01	-12.27	5.93	3.87E-02	-0.24	7.54	9.74E-01
cg06251978	-7.50	3.54	3.39E-02	-1.55	5.07	7.60E-01	-10.28	8.69	2.37E-01	-14.15	8.74	1.05E-01	-14.83	8.25	7.21E-02
cg02162286	11.24	5.30	3.39E-02	12.98	8.64	1.33E-01	0.83	13.55	9.51E-01	9.47	11.21	3.98E-01	16.66	10.67	1.19E-01
cg03402794	-34.98	16.50	3.39E-02	-29.72	20.89	1.55E-01	-32.85	74.57	6.60E-01	-65.99	80.25	4.11E-01	-42.25	30.87	1.71E-01
cg16083044	-7.45	3.51	3.40E-02	-11.00	4.60	1.68E-02	-2.21	8.14	7.86E-01	-7.94	14.80	5.91E-01	-1.03	8.40	9.02E-01
cg09225861	6.65	3.14	3.40E-02	7.67	4.26	7.15E-02	6.39	6.34	3.14E-01	-2.80	14.33	8.45E-01	6.44	7.74	4.06E-01
cg26287741	18.98	8.95	3.40E-02	4.73	16.33	7.72E-01	15.19	17.69	3.91E-01	24.61	18.50	1.83E-01	37.73	19.55	5.36E-02
cg26001287	12.82	6.05	3.40E-02	14.77	7.29	4.27E-02	35.91	23.72	1.30E-01	7.32	21.95	7.39E-01	-1.34	14.61	9.27E-01
cg24642439	-25.64	12.09	3.40E-02	-54.94	43.26	2.04E-01	-245.77	164.43	1.35E-01	-23.70	13.18	7.22E-02	-1.05	44.10	9.81E-01
cg02449166	15.51	7.31	3.40E-02	12.94	10.42	2.14E-01	24.14	16.82	1.51E-01	4.56	23.31	8.45E-01	18.74	15.60	2.30E-01
cg22019177	29.91	14.11	3.40E-02	28.62	18.77	1.27E-01	7.10	56.48	9.00E-01	34.41	37.26	3.56E-01	36.50	29.47	2.16E-01
cg20985067	-11.81	5.57	3.40E-02	2.91	13.17	8.25E-01	-11.66	16.23	4.73E-01	-18.32	7.20	1.10E-02	-0.07	17.09	9.97E-01
cg05304393	8.89	4.19	3.40E-02	11.58	6.28	6.52E-02	8.80	8.96	3.26E-01	-9.38	13.43	4.85E-01	11.45	8.61	1.84E-01
cg04520793	-11.81	5.57	3.40E-02	-8.59	7.42	2.47E-01	3.63	14.01	7.96E-01	-12.92	10.20	2.05E-01	-29.41	12.11	1.52E-02
cg15498134	-6.59	3.11	3.40E-02	-8.74	4.40	4.68E-02	-6.65	7.53	3.77E-01	-9.85	11.29	3.83E-01	-1.36	6.15	8.25E-01
cg10082354	-9.45	4.46	3.40E-02	-14.83	9.90	1.34E-01	-4.27	12.20	7.26E-01	-7.91	6.15	1.98E-01	-12.48	12.04	3.00E-01
cg22791453	9.88	4.66	3.40E-02	14.85	7.29	4.17E-02	-1.35	10.34	8.96E-01	17.28	11.53	1.34E-01	5.63	9.82	5.67E-01
cg13700250	-7.13	3.36	3.40E-02	-11.45	7.21	1.12E-01	3.80	10.93	7.28E-01	-7.26	4.55	1.10E-01	-7.24	8.90	4.16E-01
cg23265210	-9.98	4.71	3.40E-02	-24.45	8.35	3.41E-03	-5.11	11.23	6.49E-01	-5.38	4.75	2.58E-01	-6.70	10.37	5.18E-01
cg10624161	24.23	11.43	3.40E-02	19.23	17.46	2.71E-01	-3.64	30.27	9.04E-01	18.11	15.13	2.31E-01	64.69	24.47	8.19E-03
cg15083158	40.52	19.11	3.40E-02	100.30	50.92	4.89E-02	39.30	246.72	8.73E-01	36.73	21.66	9.00E-02	-32.52	69.86	6.42E-01
cg16696043	21.86	10.31	3.40E-02	4.09	13.83	7.67E-01	27.69	16.28	8.91E-02	13.28	18.55	4.74E-01	54.06	20.57	8.58E-03
cg24157814	-12.80	6.04	3.40E-02	-22.36	10.81	3.87E-02	-0.52	14.42	9.71E-01	-9.54	10.23	3.51E-01	-14.69	14.90	3.24E-01
cg02092807	22.10	10.42	3.40E-02	-1.71	19.39	9.30E-01	40.01	22.24	7.20E-02	18.72	15.46	2.26E-01	50.23	27.23	6.51E-02
cg17448192	-5.18	2.44	3.40E-02	-4.67	3.51	1.83E-01	-3.09	5.46	5.72E-01	-9.33	6.50	1.51E-01	-5.64	5.90	3.38E-01
cg26461695	16.83	7.94	3.40E-02	33.03	13.51	1.45E-02	6.17	20.48	7.63E-01	24.24	19.23	2.07E-01	2.29	13.21	8.63E-01
cg26413691	-9.78	4.61	3.40E-02	-11.90	6.55	6.92E-02	-12.19	11.38	2.84E-01	2.77	11.12	8.03E-01	-14.00	11.25	2.13E-01
cg10625096	-10.74	5.07	3.40E-02	-11.84	11.32	2.95E-01	-9.27	20.28	6.48E-01	-12.11	6.35	5.64E-02	-0.69	16.06	9.66E-01
cg22711218	20.59	9.71	3.40E-02	23.98	23.02	2.98E-01	61.76	43.23	1.53E-01	11.92	11.81	3.13E-01	54.40	31.62	8.53E-02
cg17154542	13.19	6.22	3.40E-02	20.36	8.46	1.61E-02	15.79	15.33	3.03E-01	3.18	22.81	8.89E-01	-3.04	13.28	8.19E-01
cg23205936	-10.45	4.93	3.40E-02	-19.45	8.78	2.67E-02	-2.94	11.80	8.03E-01	-2.68	9.18	7.70E-01	-13.63	10.45	1.92E-01
cg16738180	18.81	8.88	3.40E-02	10.61	13.64	4.37E-01	13.30	18.77	4.79E-01	18.33	22.65	4.18E-01	42.80	19.88	3.13E-02
cg16035777	-10.17	4.80	3.40E-02	-17.19	7.41	2.03E-02	-19.97	11.75	8.91E-02	0.11	7.25	9.88E-01	-8.63	10.18	3.96E-01

cg09355697	24.57	11.59	3.40E-02	17.05	18.45	3.55E-01	32.84	24.56	1.81E-01	23.03	23.73	3.32E-01	34.93	30.56	2.53E-01
cg07540326	-8.13	3.84	3.40E-02	-12.54	11.83	2.89E-01	-14.92	20.17	4.60E-01	-7.47	4.38	8.79E-02	-5.91	12.71	6.42E-01
cg15809352	-25.04	11.81	3.40E-02	-17.72	23.38	4.48E-01	-0.89	33.86	9.79E-01	-31.00	16.24	5.63E-02	-42.51	38.46	2.69E-01
cg02309725	9.14	4.31	3.40E-02	5.24	8.10	5.17E-01	7.35	8.98	4.13E-01	12.31	6.93	7.58E-02	12.06	13.69	3.78E-01
cg13655242	-7.45	3.52	3.40E-02	-4.41	8.18	5.90E-01	-11.12	12.70	3.81E-01	-6.47	4.38	1.40E-01	-17.04	11.40	1.35E-01
cg17497419	-29.05	13.71	3.40E-02	-30.78	39.87	4.40E-01	210.52	205.65	3.06E-01	-30.73	15.35	4.52E-02	-23.03	48.55	6.35E-01
cg23160016	7.56	3.57	3.40E-02	11.42	5.21	2.84E-02	4.38	12.35	7.23E-01	12.92	10.07	2.00E-01	0.70	6.27	9.11E-01
cg24656529	6.93	3.27	3.40E-02	3.55	6.22	5.68E-01	14.47	9.54	1.29E-01	9.34	4.46	3.65E-02	-8.73	11.66	4.54E-01
cg18014983	-5.16	2.43	3.41E-02	-2.90	3.61	4.22E-01	-7.63	5.09	1.34E-01	-7.22	8.00	3.67E-01	-6.34	5.11	2.15E-01
cg11581706	15.28	7.21	3.41E-02	18.89	15.20	2.14E-01	-4.75	20.37	8.16E-01	15.84	10.58	1.34E-01	23.04	16.75	1.69E-01
cg14948804	-6.93	3.27	3.41E-02	-10.32	5.82	7.62E-02	-8.15	13.87	5.57E-01	-4.51	4.56	3.23E-01	-7.88	9.68	4.16E-01
cg24374013	-10.81	5.10	3.41E-02	-12.44	7.50	9.72E-02	-10.30	12.26	4.01E-01	-9.32	15.03	5.35E-01	-8.83	10.22	3.87E-01
cg03426888	10.90	5.14	3.41E-02	17.20	6.20	5.56E-03	21.18	14.06	1.32E-01	1.24	12.59	9.22E-01	-0.15	9.34	9.87E-01
cg06727269	19.10	9.01	3.41E-02	14.26	10.84	1.88E-01	22.07	34.70	5.25E-01	13.04	50.29	7.95E-01	35.07	19.71	7.52E-02
cg06912814	-14.89	7.03	3.41E-02	-26.04	14.51	7.27E-02	6.66	16.97	6.95E-01	-19.27	10.12	5.69E-02	-5.65	21.05	7.88E-01
cg25306991	16.81	7.93	3.41E-02	21.62	9.39	2.13E-02	6.26	36.43	8.64E-01	-3.76	58.07	9.48E-01	5.26	16.88	7.56E-01
cg25360535	15.34	7.24	3.41E-02	42.50	22.37	5.74E-02	-3.01	22.97	8.96E-01	14.19	8.55	9.71E-02	12.90	25.84	6.17E-01
cg25216196	10.70	5.05	3.41E-02	14.80	6.81	2.97E-02	22.62	12.53	7.12E-02	5.94	11.76	6.13E-01	-3.93	10.91	7.18E-01
cg17431888	12.80	6.04	3.41E-02	17.74	10.39	8.78E-02	7.18	12.45	5.64E-01	-1.23	16.00	9.39E-01	18.63	11.34	1.00E-01
cg25692290	17.40	8.21	3.41E-02	13.90	33.55	6.79E-01	-69.37	106.25	5.14E-01	21.05	8.70	1.56E-02	-39.57	39.04	3.11E-01
cg25500948	-13.61	6.42	3.41E-02	-26.19	11.93	2.82E-02	-9.91	13.39	4.59E-01	-4.21	5.21	4.19E-01	-27.36	13.20	3.82E-02
cg04722215	-6.34	2.99	3.41E-02	-5.04	3.84	1.89E-01	-13.54	8.00	9.06E-02	-6.61	10.23	5.19E-01	-4.87	7.29	5.04E-01
cg22266211	10.85	5.12	3.41E-02	22.50	7.02	1.36E-03	1.25	9.88	8.99E-01	10.71	7.33	1.44E-01	1.74	10.25	8.65E-01
cg11578064	12.32	5.81	3.41E-02	5.80	12.40	6.40E-01	32.65	19.10	8.73E-02	9.69	7.34	1.87E-01	32.41	23.78	1.73E-01
cg03970319	23.63	11.15	3.41E-02	59.78	35.00	8.76E-02	-15.82	138.34	9.09E-01	18.90	12.36	1.26E-01	29.18	39.79	4.63E-01
cg12399700	13.38	6.31	3.41E-02	23.17	13.44	8.46E-02	22.53	18.86	2.32E-01	12.56	9.56	1.89E-01	1.14	13.12	9.31E-01
cg14138540	7.62	3.60	3.41E-02	5.81	4.66	2.13E-01	15.33	8.97	8.72E-02	6.88	10.60	5.16E-01	7.01	9.99	4.83E-01
cg25685934	20.63	9.74	3.41E-02	25.18	20.71	2.24E-01	11.61	20.93	5.79E-01	24.14	14.41	9.39E-02	14.43	29.98	6.30E-01
cg25987744	9.44	4.46	3.41E-02	10.35	6.96	1.37E-01	19.36	10.97	7.76E-02	-2.23	9.63	8.17E-01	11.76	9.70	2.25E-01
cg27304666	18.67	8.81	3.41E-02	11.01	16.27	4.99E-01	8.41	17.66	6.34E-01	24.62	15.21	1.06E-01	41.67	25.23	9.86E-02
cg08301965	20.52	9.68	3.41E-02	25.09	17.88	1.61E-01	-4.64	18.77	8.05E-01	30.14	17.45	8.41E-02	38.57	26.61	1.47E-01
cg11475454	12.20	5.76	3.41E-02	11.66	8.95	1.93E-01	2.21	12.56	8.60E-01	34.99	15.96	2.84E-02	9.59	11.62	4.09E-01
cg01106649	17.47	8.25	3.41E-02	31.45	20.72	1.29E-01	19.54	39.41	6.20E-01	15.03	9.89	1.29E-01	11.57	25.76	6.53E-01
cg04305082	18.78	8.86	3.41E-02	45.19	18.81	1.63E-02	14.19	19.06	4.57E-01	14.10	12.92	2.75E-01	-6.72	27.34	8.06E-01
cg14969094	-9.42	4.45	3.41E-02	-4.69	6.37	4.62E-01	-5.89	12.47	6.37E-01	-26.42	11.57	2.24E-02	-10.43	9.12	2.52E-01

cg09605818	20.74	9.79	3.41E-02	29.81	18.52	1.07E-01	2.66	23.95	9.12E-01	11.76	13.26	3.76E-01	59.37	27.50	3.09E-02
cg12691330	-12.32	5.82	3.41E-02	-19.79	6.35	1.84E-03	-3.66	10.25	7.21E-01	1.00	8.78	9.09E-01	-22.85	8.85	9.81E-03
cg26757722	5.18	2.44	3.41E-02	3.36	4.28	4.33E-01	4.76	7.98	5.51E-01	6.81	3.68	6.44E-02	4.55	6.54	4.87E-01
cg06671962	-8.18	3.86	3.41E-02	-10.65	5.81	6.65E-02	2.05	8.55	8.11E-01	-14.85	11.40	1.93E-01	-9.13	7.90	2.48E-01
cg01410163	27.50	12.98	3.41E-02	47.12	20.53	2.17E-02	9.63	43.10	8.23E-01	16.13	28.02	5.65E-01	14.66	23.92	5.40E-01
cg15219145	16.24	7.67	3.41E-02	25.96	19.88	1.91E-01	-2.82	24.41	9.08E-01	19.91	10.13	4.94E-02	6.97	18.06	7.00E-01
cg06857900	-16.90	7.98	3.42E-02	-25.15	15.73	1.10E-01	3.65	20.17	8.57E-01	-15.07	11.97	2.08E-01	-30.35	21.19	1.52E-01
cg07279214	48.07	22.70	3.42E-02	41.64	40.76	3.07E-01	296.73	152.90	5.23E-02	43.05	30.62	1.60E-01	41.99	65.92	5.24E-01
cg15680020	25.53	12.05	3.42E-02	28.41	15.76	7.14E-02	-19.42	34.65	5.75E-01	86.94	59.74	1.46E-01	30.47	23.94	2.03E-01
cg07138217	20.52	9.69	3.42E-02	26.44	18.58	1.55E-01	29.01	21.23	1.72E-01	9.70	14.75	5.11E-01	35.07	32.51	2.81E-01
cg09615056	5.89	2.78	3.42E-02	7.20	4.11	7.99E-02	1.84	5.83	7.52E-01	11.29	8.76	1.97E-01	4.85	6.02	4.20E-01
cg17614506	-6.72	3.17	3.42E-02	-8.04	5.23	1.24E-01	0.16	8.21	9.84E-01	-5.71	5.75	3.20E-01	-11.47	7.52	1.27E-01
cg21506565	5.96	2.81	3.42E-02	9.24	3.93	1.87E-02	-0.83	7.63	9.14E-01	11.16	7.76	1.51E-01	-0.10	5.80	9.86E-01
cg27266176	10.01	4.73	3.42E-02	10.59	7.75	1.72E-01	13.31	13.20	3.13E-01	0.37	8.21	9.64E-01	23.98	11.09	3.06E-02
ch.19.1079710R	-14.89	7.03	3.42E-02	-11.58	8.75	1.86E-01	-0.63	23.27	9.78E-01	-6.24	26.62	8.15E-01	-35.89	16.01	2.50E-02
cg04445427	6.33	2.99	3.42E-02	7.33	4.08	7.23E-02	13.60	6.64	4.06E-02	5.02	5.51	3.62E-01	-5.62	8.37	5.02E-01
cg06674731	10.04	4.74	3.42E-02	14.81	9.52	1.20E-01	3.64	10.58	7.31E-01	20.63	10.52	4.99E-02	4.16	8.03	6.05E-01
cg26444528	8.38	3.96	3.42E-02	15.58	7.24	3.14E-02	7.57	8.52	3.74E-01	5.46	9.25	5.55E-01	3.61	7.21	6.17E-01
cg06168404	-11.09	5.23	3.42E-02	-13.22	11.71	2.59E-01	3.67	22.12	8.68E-01	-9.85	6.56	1.33E-01	-22.17	15.97	1.65E-01
cg05882878	-23.44	11.07	3.42E-02	-5.47	10.64	6.07E-01	-48.75	41.03	2.35E-01	-37.97	21.38	7.57E-02	-39.84	19.00	3.60E-02
cg01722297	15.20	7.18	3.42E-02	11.23	9.20	2.22E-01	13.96	18.67	4.55E-01	7.62	39.45	8.47E-01	28.75	15.64	6.61E-02
cg04549460	14.96	7.06	3.42E-02	23.39	10.34	2.37E-02	11.00	23.09	6.34E-01	22.76	20.65	2.70E-01	1.11	12.43	9.29E-01
cg12306086	5.90	2.79	3.42E-02	6.12	3.82	1.09E-01	8.88	6.41	1.66E-01	2.09	9.22	8.21E-01	4.12	6.45	5.23E-01
cg14773523	20.27	9.57	3.42E-02	27.85	19.88	1.61E-01	-18.65	36.09	6.05E-01	19.51	12.62	1.22E-01	31.78	27.30	2.44E-01
cg14523841	20.97	9.90	3.42E-02	37.36	22.20	9.24E-02	-24.05	27.51	3.82E-01	22.31	9.44	1.81E-02	36.89	29.31	2.08E-01
cg06132694	24.57	11.60	3.42E-02	26.08	16.49	1.14E-01	-23.25	51.81	6.54E-01	66.71	35.78	6.23E-02	16.63	19.61	3.97E-01
cg17716358	23.89	11.28	3.42E-02	14.09	19.82	4.77E-01	31.98	21.07	1.29E-01	8.40	14.29	5.57E-01	65.11	26.07	1.25E-02
cg13051970	-11.85	5.60	3.42E-02	-20.36	8.03	1.12E-02	-4.31	13.71	7.53E-01	0.80	16.59	9.62E-01	-5.70	11.57	6.22E-01
cg01216623	39.08	18.45	3.42E-02	45.94	21.06	2.92E-02	51.79	95.41	5.87E-01	-73.97	85.71	3.88E-01	35.68	47.83	4.56E-01
cg09510531	9.09	4.29	3.42E-02	5.07	5.83	3.85E-01	4.09	14.25	7.74E-01	13.99	10.13	1.67E-01	18.39	9.89	6.30E-02
cg11703179	-14.68	6.93	3.42E-02	-2.32	18.74	9.01E-01	-4.02	36.50	9.12E-01	-19.36	7.91	1.44E-02	10.89	28.46	7.02E-01
cg15526387	37.67	17.79	3.42E-02	54.18	22.62	1.66E-02	-63.09	78.14	4.19E-01	-8.10	92.65	9.30E-01	26.41	32.85	4.21E-01
cg25342568	13.68	6.46	3.42E-02	4.26	11.96	7.21E-01	16.80	12.35	1.74E-01	16.65	11.85	1.60E-01	21.05	17.44	2.27E-01
cg02121104	-10.74	5.07	3.42E-02	-17.82	7.16	1.28E-02	2.47	12.21	8.40E-01	-3.08	16.04	8.48E-01	-8.47	10.70	4.29E-01
cg20569593	33.74	15.93	3.42E-02	48.86	44.85	2.76E-01	-24.76	212.80	9.07E-01	33.21	17.74	6.11E-02	14.84	64.31	8.17E-01

cg24938761	4.87	2.30	3.42E-02	4.92	2.81	8.01E-02	9.48	6.00	1.14E-01	-0.18	8.03	9.83E-01	1.95	7.21	7.87E-01
cg13187349	-16.10	7.60	3.42E-02	-13.39	11.50	2.44E-01	11.23	24.92	6.52E-01	-15.07	13.69	2.71E-01	-40.96	18.85	2.98E-02
cg14023558	-40.48	19.12	3.42E-02	-24.95	23.40	2.86E-01	-108.19	97.71	2.68E-01	-81.20	79.82	3.09E-01	-63.42	39.28	1.06E-01
cg16986748	10.96	5.18	3.42E-02	13.34	6.90	5.33E-02	8.15	15.87	6.07E-01	17.72	11.05	1.09E-01	-11.56	15.46	4.54E-01
cg13852284	5.33	2.52	3.42E-02	5.60	3.33	9.27E-02	13.57	7.22	6.01E-02	3.07	6.16	6.19E-01	-0.24	6.75	9.71E-01
cg20263444	31.08	14.68	3.42E-02	11.13	45.03	8.05E-01	-115.73	175.04	5.09E-01	39.65	16.35	1.53E-02	-15.27	51.62	7.67E-01
cg13001012	-12.25	5.79	3.42E-02	-22.37	9.74	2.16E-02	-4.13	13.68	7.62E-01	-6.55	11.61	5.72E-01	-9.05	12.34	4.63E-01
cg04582843	-42.30	19.98	3.42E-02	-21.58	28.64	4.51E-01	-87.25	48.03	6.93E-02	-74.72	54.75	1.72E-01	-32.58	43.87	4.58E-01
cg12820122	-31.81	15.03	3.42E-02	-41.77	47.08	3.75E-01	-215.06	217.30	3.22E-01	-33.63	16.48	4.13E-02	22.75	60.21	7.06E-01
cg17238065	4.75	2.24	3.42E-02	4.92	2.73	7.10E-02	10.22	6.21	9.98E-02	4.39	7.34	5.49E-01	-3.25	7.10	6.47E-01
cg14360448	-16.77	7.92	3.42E-02	-9.26	11.25	4.11E-01	-53.70	29.40	6.77E-02	-19.63	17.32	2.57E-01	-18.74	16.77	2.64E-01
cg11738198	-7.38	3.49	3.42E-02	-8.04	6.69	2.29E-01	-11.96	10.22	2.42E-01	-6.32	5.20	2.24E-01	-5.95	8.68	4.93E-01
cg23983449	13.99	6.61	3.43E-02	13.98	9.53	1.42E-01	9.01	15.14	5.52E-01	30.03	19.58	1.25E-01	9.91	14.26	4.87E-01
cg24078363	-39.10	18.47	3.43E-02	-89.13	44.69	4.61E-02	-161.63	200.33	4.20E-01	-21.07	21.55	3.28E-01	-81.22	62.73	1.95E-01
cg20704560	6.89	3.25	3.43E-02	8.10	4.72	8.61E-02	7.15	7.09	3.13E-01	2.33	10.56	8.25E-01	5.99	6.96	3.90E-01
cg00181669	-8.15	3.85	3.43E-02	-20.80	10.21	4.16E-02	-13.32	20.06	5.07E-01	-4.29	4.49	3.40E-01	-17.83	13.03	1.71E-01
cg08297985	9.37	4.43	3.43E-02	12.24	6.70	6.79E-02	10.19	9.90	3.03E-01	4.20	9.40	6.55E-01	7.46	11.69	5.23E-01
cg07024017	-44.96	21.24	3.43E-02	-65.98	27.56	1.66E-02	12.21	67.91	8.57E-01	-58.11	82.07	4.79E-01	-12.71	43.26	7.69E-01
cg15891757	-29.61	13.99	3.43E-02	-40.44	22.63	7.39E-02	-92.89	40.81	2.28E-02	-10.83	9.82	2.70E-01	-30.12	26.62	2.58E-01
cg02415470	17.16	8.11	3.43E-02	6.96	12.53	5.78E-01	30.79	17.79	8.36E-02	19.32	19.16	3.13E-01	22.62	18.40	2.19E-01
cg12400702	-12.24	5.78	3.43E-02	-20.87	12.59	9.75E-02	-5.85	31.88	8.54E-01	-13.30	7.29	6.82E-02	5.49	16.11	7.34E-01
cg27368025	11.85	5.60	3.43E-02	18.74	6.81	5.90E-03	19.53	12.04	1.05E-01	-5.59	11.57	6.29E-01	7.41	11.15	5.06E-01
cg00396158	16.36	7.73	3.43E-02	26.00	10.53	1.36E-02	5.34	22.68	8.14E-01	16.99	19.33	3.80E-01	-5.28	17.96	7.69E-01
cg09082852	18.18	8.59	3.43E-02	29.85	21.24	1.60E-01	2.93	24.30	9.04E-01	14.00	10.81	1.95E-01	51.19	30.40	9.22E-02
cg26348348	10.58	5.00	3.43E-02	3.58	8.05	6.56E-01	16.28	14.03	2.46E-01	13.41	8.73	1.24E-01	17.07	12.48	1.71E-01
cg24089887	10.77	5.09	3.43E-02	5.67	9.99	5.70E-01	-2.04	12.41	8.69E-01	18.16	7.50	1.55E-02	11.42	15.13	4.50E-01
cg16083838	23.46	11.08	3.43E-02	15.05	15.09	3.19E-01	57.10	44.05	1.95E-01	56.06	36.38	1.23E-01	21.44	20.10	2.86E-01
cg03988107	14.83	7.00	3.43E-02	25.70	10.32	1.27E-02	6.16	15.63	6.94E-01	-10.79	20.90	6.06E-01	13.07	14.74	3.75E-01
cg17221813	2.23	1.05	3.43E-02	2.58	1.40	6.55E-02	3.12	2.22	1.59E-01	-2.68	3.72	4.71E-01	2.19	2.95	4.59E-01
cg05208605	15.02	7.10	3.43E-02	21.80	9.19	1.76E-02	32.09	13.72	1.93E-02	1.25	13.47	9.26E-01	1.95	12.71	8.78E-01
cg08940669	51.01	24.10	3.43E-02	44.84	31.03	1.48E-01	-0.72	165.83	9.97E-01	102.00	81.73	2.12E-01	52.32	44.84	2.43E-01
cg16172549	6.06	2.86	3.43E-02	7.06	4.42	1.11E-01	0.13	5.86	9.83E-01	16.22	7.71	3.53E-02	4.08	6.33	5.19E-01
cg05638165	14.11	6.67	3.43E-02	25.18	20.11	2.10E-01	20.12	27.90	4.71E-01	12.89	7.54	8.73E-02	2.19	29.64	9.41E-01
cg16696648	-11.17	5.28	3.43E-02	-17.90	10.85	9.88E-02	11.39	17.67	5.19E-01	-14.81	5.82	1.09E-02	3.72	15.41	8.09E-01
cg13726563	-13.71	6.48	3.43E-02	-9.72	9.99	3.31E-01	1.66	15.57	9.15E-01	-32.29	15.12	3.27E-02	-17.84	13.70	1.93E-01

cg14154186	15.41	7.28	3.43E-02	17.19	18.59	3.55E-01	-12.31	42.27	7.71E-01	16.54	8.45	5.03E-02	11.51	26.64	6.66E-01
cg13801475	-16.26	7.68	3.43E-02	-10.77	20.38	5.97E-01	-24.79	23.14	2.84E-01	-14.04	9.63	1.45E-01	-27.45	23.01	2.33E-01
cg19693503	-21.17	10.00	3.43E-02	-17.82	10.85	1.00E-01	-25.92	90.47	7.75E-01	-47.45	73.00	5.16E-01	-40.47	29.03	1.63E-01
cg20959480	-17.94	8.48	3.43E-02	-29.67	13.10	2.35E-02	-9.48	35.50	7.89E-01	3.84	18.73	8.38E-01	-18.02	14.99	2.29E-01
cg08156349	32.82	15.51	3.43E-02	18.46	57.56	7.48E-01	-185.72	200.43	3.54E-01	29.43	16.97	8.29E-02	92.65	52.72	7.88E-02
cg13424029	7.17	3.39	3.43E-02	6.36	4.64	1.70E-01	8.65	7.81	2.68E-01	16.81	10.43	1.07E-01	2.16	8.18	7.92E-01
cg26451415	8.96	4.23	3.43E-02	5.83	6.15	3.43E-01	8.09	10.49	4.41E-01	-3.01	14.21	8.32E-01	18.75	8.08	2.04E-02
cg15076384	-45.48	21.49	3.43E-02	-27.60	22.04	2.11E-01	22.56	85.95	7.93E-01	-133.93	53.84	1.29E-02	-47.24	31.59	1.35E-01
cg15812322	-6.67	3.15	3.43E-02	-9.37	4.29	2.88E-02	-0.93	7.40	9.01E-01	-7.14	10.28	4.88E-01	-4.14	7.33	5.72E-01
cg14510926	-11.14	5.27	3.43E-02	-12.48	7.63	1.02E-01	7.82	14.43	5.88E-01	-10.43	12.50	4.04E-01	-20.60	11.40	7.08E-02
cg18375960	25.69	12.14	3.43E-02	22.37	24.43	3.60E-01	46.57	55.89	4.05E-01	27.74	16.18	8.66E-02	16.49	32.04	6.07E-01
cg16724800	-6.20	2.93	3.43E-02	-12.28	7.24	8.97E-02	6.86	8.37	4.13E-01	-6.88	3.47	4.72E-02	-7.59	7.53	3.14E-01
cg04251571	-27.82	13.15	3.43E-02	-21.90	29.55	4.59E-01	5.28	81.98	9.49E-01	-31.24	16.17	5.33E-02	-25.73	38.73	5.06E-01
cg08867707	8.05	3.80	3.43E-02	11.65	8.40	1.66E-01	14.91	10.57	1.58E-01	6.11	4.91	2.13E-01	0.95	14.91	9.49E-01
cg10221896	-12.99	6.14	3.43E-02	-18.88	15.36	2.19E-01	-4.83	15.13	7.50E-01	-14.80	8.16	6.98E-02	-7.35	18.47	6.91E-01
cg05584919	16.66	7.87	3.43E-02	7.57	12.17	5.34E-01	36.62	15.57	1.87E-02	9.93	21.02	6.37E-01	14.71	18.26	4.20E-01
cg20750832	9.94	4.70	3.44E-02	11.74	9.93	2.37E-01	3.53	11.94	7.67E-01	18.97	7.94	1.70E-02	0.47	9.03	9.58E-01
cg16280132	-5.18	2.45	3.44E-02	-4.23	2.94	1.50E-01	-7.00	6.07	2.48E-01	-11.56	8.31	1.64E-01	-1.69	10.54	8.73E-01
cg13908968	-12.81	6.05	3.44E-02	-28.66	15.04	5.68E-02	-5.30	21.88	8.09E-01	-9.86	7.31	1.77E-01	-13.11	21.86	5.49E-01
cg14707231	6.31	2.98	3.44E-02	3.26	4.06	4.21E-01	10.35	6.72	1.24E-01	8.46	8.68	3.30E-01	10.50	7.87	1.82E-01
cg18004847	5.88	2.78	3.44E-02	5.42	3.25	9.51E-02	10.39	7.04	1.40E-01	5.84	13.09	6.55E-01	0.45	10.71	9.66E-01
cg04887494	15.09	7.13	3.44E-02	15.62	10.04	1.20E-01	0.55	19.29	9.77E-01	31.93	23.41	1.72E-01	15.69	13.83	2.57E-01
cg12415917	-13.61	6.43	3.44E-02	-25.56	14.74	8.29E-02	2.19	16.51	8.95E-01	-10.99	8.81	2.12E-01	-25.80	18.21	1.57E-01
cg10004882	-10.67	5.04	3.44E-02	-8.80	8.22	2.85E-01	2.38	11.94	8.42E-01	-19.88	12.47	1.11E-01	-16.08	9.50	9.06E-02
cg08359958	-53.96	25.51	3.44E-02	-29.76	58.34	6.10E-01	-144.02	268.28	5.91E-01	-76.29	31.60	1.58E-02	18.44	66.24	7.81E-01
cg23948240	12.43	5.88	3.44E-02	20.37	8.76	2.00E-02	-1.12	17.75	9.50E-01	23.07	29.45	4.33E-01	6.15	9.29	5.08E-01
cg15946224	-10.69	5.05	3.44E-02	-10.29	12.17	3.98E-01	-16.30	16.15	3.13E-01	-6.78	6.46	2.94E-01	-26.72	14.66	6.83E-02
cg01342572	4.64	2.19	3.44E-02	4.23	2.89	1.42E-01	8.39	5.07	9.79E-02	9.51	7.63	2.13E-01	-1.01	5.58	8.56E-01
cg21139076	-15.20	7.19	3.44E-02	-10.40	17.19	5.45E-01	-4.37	45.58	9.24E-01	-14.58	8.42	8.32E-02	-37.29	26.98	1.67E-01
cg00372692	-14.15	6.69	3.44E-02	-11.08	14.52	4.45E-01	1.36	19.31	9.44E-01	-18.87	9.04	3.69E-02	-13.58	19.31	4.82E-01
cg11827086	17.89	8.46	3.44E-02	17.09	12.51	1.72E-01	33.22	23.34	1.55E-01	24.82	19.17	1.96E-01	4.12	18.14	8.20E-01
cg01611223	-11.87	5.61	3.44E-02	-3.86	10.84	7.22E-01	-12.19	16.91	4.71E-01	-13.92	8.04	8.35E-02	-20.13	15.28	1.88E-01
cg14161438	38.19	18.05	3.44E-02	40.83	21.85	6.16E-02	65.01	114.50	5.70E-01	33.19	80.48	6.80E-01	29.02	36.68	4.29E-01
cg02232704	16.51	7.81	3.44E-02	25.18	17.91	1.60E-01	41.14	26.04	1.14E-01	18.26	11.13	1.01E-01	-4.17	16.34	7.99E-01
cg16998327	16.88	7.98	3.44E-02	18.02	21.52	4.02E-01	-15.48	26.95	5.66E-01	17.09	10.18	9.33E-02	32.71	19.89	1.00E-01

cg08284233	-8.40	3.97	3.44E-02	-7.46	5.74	1.94E-01	-8.74	8.79	3.20E-01	-7.93	12.41	5.23E-01	-10.42	8.59	2.25E-01
cg22388653	37.77	17.85	3.44E-02	58.00	53.31	2.77E-01	-272.90	206.22	1.86E-01	36.60	19.65	6.25E-02	56.59	76.48	4.59E-01
cg16097772	5.74	2.72	3.44E-02	6.13	3.45	7.58E-02	7.12	6.40	2.66E-01	8.56	10.20	4.01E-01	0.50	7.49	9.46E-01
cg01003211	12.11	5.72	3.44E-02	3.77	9.35	6.86E-01	2.99	14.81	8.40E-01	19.63	15.38	2.02E-01	22.32	9.86	2.35E-02
cg20867746	-6.84	3.24	3.44E-02	-14.09	5.59	1.16E-02	-3.51	9.20	7.03E-01	-2.99	5.17	5.63E-01	-3.43	8.40	6.83E-01
cg16905265	-31.07	14.69	3.44E-02	-34.20	15.57	2.81E-02	-2.23	32.27	9.45E-01	62.53	66.34	3.46E-01	-54.40	20.17	7.01E-03
cg14501119	18.82	8.90	3.44E-02	24.66	14.78	9.52E-02	18.33	18.32	3.17E-01	0.47	17.19	9.78E-01	40.69	24.36	9.49E-02
cg11834473	9.65	4.56	3.44E-02	12.51	6.34	4.86E-02	8.41	11.34	4.58E-01	13.67	12.71	2.82E-01	0.28	10.42	9.79E-01
cg07568203	-11.60	5.49	3.44E-02	-21.56	11.98	7.18E-02	-24.47	21.28	2.50E-01	-8.46	6.98	2.26E-01	-2.20	16.79	8.96E-01
cg12932306	-11.19	5.29	3.44E-02	-13.97	8.41	9.66E-02	-12.02	12.06	3.19E-01	-8.09	14.18	5.69E-01	-8.14	10.13	4.21E-01
cg11454415	30.24	14.30	3.44E-02	23.06	18.33	2.08E-01	54.99	50.69	2.78E-01	95.38	60.98	1.18E-01	25.64	28.19	3.63E-01
cg09037777	10.17	4.81	3.44E-02	9.01	7.05	2.02E-01	-1.84	11.28	8.71E-01	25.94	13.64	5.72E-02	13.48	10.04	1.79E-01
cg05205664	-13.85	6.55	3.44E-02	-17.99	9.15	4.94E-02	5.38	16.38	7.43E-01	30.16	45.59	5.08E-01	-19.91	11.81	9.18E-02
cg07960067	18.91	8.94	3.44E-02	25.53	25.00	3.07E-01	-50.69	73.91	4.93E-01	18.88	10.29	6.65E-02	20.82	28.00	4.57E-01
cg10724054	-13.01	6.15	3.44E-02	-24.68	10.19	1.54E-02	4.40	12.62	7.27E-01	-9.98	13.26	4.52E-01	-15.52	11.37	1.72E-01
cg01389445	17.40	8.23	3.44E-02	41.30	22.03	6.08E-02	-2.18	41.40	9.58E-01	15.02	9.83	1.27E-01	9.99	23.65	6.73E-01
cg25432300	9.04	4.27	3.44E-02	5.08	6.08	4.03E-01	9.97	10.09	3.23E-01	20.14	11.04	6.82E-02	9.76	10.19	3.38E-01
cg10835770	11.84	5.60	3.45E-02	9.54	8.53	2.63E-01	15.17	15.73	3.35E-01	16.16	11.96	1.77E-01	10.15	11.86	3.92E-01
cg14286373	-15.46	7.31	3.45E-02	-9.07	15.17	5.50E-01	-36.38	31.80	2.53E-01	-19.22	9.50	4.31E-02	-0.41	20.84	9.84E-01
cg24142735	-19.30	9.13	3.45E-02	-30.04	15.45	5.18E-02	-9.80	48.15	8.39E-01	-15.01	12.87	2.43E-01	-8.14	27.21	7.65E-01
cg20390500	-14.36	6.79	3.45E-02	-31.07	16.10	5.36E-02	-47.89	32.15	1.36E-01	-9.63	8.16	2.38E-01	-0.22	23.46	9.92E-01
cg22338567	42.48	20.09	3.45E-02	109.80	36.10	2.36E-03	59.45	113.57	6.01E-01	24.92	9.96	1.24E-02	21.93	30.76	4.76E-01
cg00588920	52.02	24.60	3.45E-02	31.35	18.39	8.83E-02	-11.86	104.48	9.10E-01	136.49	37.36	2.59E-04	28.09	22.27	2.07E-01
cg21265783	-56.35	26.65	3.45E-02	-28.91	27.40	2.91E-01	-158.02	57.32	5.84E-03	-25.39	65.11	6.97E-01	-52.14	43.92	2.35E-01
cg14327505	-6.56	3.10	3.45E-02	-1.77	5.70	7.56E-01	-3.99	9.92	6.87E-01	-10.41	4.70	2.68E-02	-6.53	7.53	3.86E-01
cg17359198	11.75	5.56	3.45E-02	21.94	10.29	3.30E-02	-0.64	14.32	9.64E-01	13.12	7.72	8.90E-02	-11.45	20.31	5.73E-01
cg19774846	-8.51	4.02	3.45E-02	-9.36	5.55	9.19E-02	-9.60	11.10	3.87E-01	1.34	10.76	9.01E-01	-12.37	8.91	1.65E-01
cg00171084	33.42	15.81	3.45E-02	21.69	19.28	2.61E-01	70.73	66.66	2.89E-01	-11.51	75.71	8.79E-01	67.41	33.11	4.17E-02
cg05700681	-18.68	8.83	3.45E-02	-16.61	15.50	2.84E-01	-9.87	19.83	6.19E-01	-14.59	16.69	3.82E-01	-36.80	19.92	6.47E-02
cg07413941	31.95	15.11	3.45E-02	26.57	17.68	1.33E-01	21.32	100.41	8.32E-01	69.82	82.22	3.96E-01	45.57	32.76	1.64E-01
cg21477907	12.43	5.88	3.45E-02	19.71	13.15	1.34E-01	33.74	42.30	4.25E-01	8.28	7.05	2.40E-01	24.44	20.16	2.25E-01
cg08815583	20.46	9.68	3.45E-02	31.22	18.93	9.91E-02	35.53	19.80	7.27E-02	-0.70	15.16	9.63E-01	34.03	25.53	1.83E-01
cg07266910	5.58	2.64	3.45E-02	6.19	3.75	9.86E-02	1.48	6.10	8.09E-01	2.97	6.93	6.68E-01	10.43	6.34	1.00E-01
cg10898212	14.05	6.65	3.45E-02	0.90	10.43	9.32E-01	17.90	10.91	1.01E-01	25.31	7.02	3.14E-04	-0.10	16.28	9.95E-01
cg06354193	-16.73	7.91	3.45E-02	-23.91	15.87	1.32E-01	-9.27	19.12	6.28E-01	-22.62	11.68	5.27E-02	9.78	22.72	6.67E-01

cg04737356	-20.30	9.60	3.45E-02	-15.53	15.60	3.20E-01	-38.43	28.38	1.76E-01	-39.63	20.84	5.72E-02	-5.45	17.69	7.58E-01
cg15082257	-8.55	4.05	3.45E-02	-5.38	7.16	4.52E-01	-14.32	9.49	1.31E-01	-3.89	4.52	3.89E-01	-24.39	10.55	2.07E-02
cg18258916	25.94	12.27	3.45E-02	22.33	20.53	2.77E-01	64.96	24.42	7.81E-03	7.76	16.14	6.31E-01	25.09	30.65	4.13E-01
cg03025880	-12.14	5.74	3.45E-02	-14.39	8.70	9.81E-02	-8.34	13.19	5.27E-01	-7.45	17.41	6.69E-01	-13.07	11.13	2.40E-01
cg18032164	9.28	4.39	3.45E-02	9.62	6.26	1.24E-01	17.27	10.59	1.03E-01	10.62	9.25	2.51E-01	-7.38	13.19	5.76E-01
cg20971147	-8.13	3.85	3.45E-02	-13.06	5.41	1.58E-02	-4.76	7.88	5.45E-01	7.41	10.65	4.87E-01	-10.67	7.99	1.81E-01
cg06776976	12.63	5.97	3.45E-02	13.66	7.83	8.10E-02	28.53	16.75	8.85E-02	1.17	22.02	9.58E-01	4.42	12.83	7.30E-01
cg02611998	17.94	8.49	3.45E-02	24.62	17.37	1.56E-01	-8.64	34.46	8.02E-01	15.84	11.10	1.54E-01	28.74	24.93	2.49E-01
cg21578207	7.99	3.78	3.45E-02	7.45	4.81	1.22E-01	14.80	10.62	1.63E-01	1.02	11.29	9.28E-01	9.71	9.93	3.28E-01
cg01055281	16.90	8.00	3.45E-02	17.66	13.65	1.96E-01	16.64	14.49	2.51E-01	11.64	17.55	5.07E-01	23.25	21.04	2.69E-01
cg00747944	17.33	8.20	3.45E-02	22.58	14.25	1.13E-01	12.49	21.43	5.60E-01	31.79	17.10	6.30E-02	2.49	15.13	8.69E-01
cg00388391	13.06	6.18	3.45E-02	13.86	8.96	1.22E-01	23.61	17.18	1.69E-01	17.50	19.20	3.62E-01	5.50	11.44	6.31E-01
cg24594504	25.25	11.95	3.45E-02	22.56	17.12	1.88E-01	24.55	40.16	5.41E-01	34.91	35.01	3.19E-01	26.05	21.52	2.26E-01
cg00054890	21.20	10.03	3.45E-02	5.27	23.16	8.20E-01	15.20	24.65	5.38E-01	24.56	13.27	6.43E-02	48.15	36.30	1.85E-01
cg03091590	11.97	5.66	3.45E-02	10.94	13.59	4.21E-01	19.51	18.09	2.81E-01	12.01	6.84	7.93E-02	-1.37	26.98	9.59E-01
cg14783639	12.68	6.00	3.45E-02	25.23	13.93	7.01E-02	10.53	23.08	6.48E-01	8.73	7.25	2.29E-01	21.15	24.09	3.80E-01
cg20524972	16.75	7.93	3.45E-02	19.28	20.57	3.48E-01	63.96	85.80	4.56E-01	13.91	8.98	1.22E-01	39.08	31.25	2.11E-01
cg15231749	18.16	8.59	3.46E-02	16.44	14.17	2.46E-01	30.81	15.06	4.07E-02	21.80	21.17	3.03E-01	-10.63	22.79	6.41E-01
cg17338299	-10.74	5.08	3.46E-02	-13.71	12.60	2.77E-01	-12.86	14.16	3.64E-01	-10.90	6.46	9.15E-02	-1.21	16.95	9.43E-01
cg05071770	-15.04	7.12	3.46E-02	-3.85	18.27	8.33E-01	12.34	29.72	6.78E-01	-18.62	8.35	2.57E-02	-25.32	28.04	3.67E-01
cg08798862	-8.14	3.85	3.46E-02	-8.55	5.11	9.46E-02	-12.71	8.96	1.56E-01	12.27	13.25	3.54E-01	-12.16	9.56	2.03E-01
cg23837799	24.74	11.70	3.46E-02	26.96	32.17	4.02E-01	24.45	136.68	8.58E-01	27.90	13.36	3.67E-02	-4.75	38.52	9.02E-01
cg10729325	-26.64	12.61	3.46E-02	-49.06	30.35	1.06E-01	-104.02	151.90	4.93E-01	-24.58	14.44	8.87E-02	21.81	52.20	6.76E-01
cg15418980	16.18	7.66	3.46E-02	18.59	14.18	1.90E-01	17.87	15.95	2.63E-01	12.02	12.50	3.36E-01	20.79	23.88	3.84E-01
cg11229267	-14.15	6.70	3.46E-02	-17.11	12.60	1.74E-01	-13.25	18.90	4.83E-01	-7.00	5.80	2.27E-01	-44.16	18.57	1.74E-02
cg25685262	13.30	6.29	3.46E-02	9.47	9.60	3.24E-01	12.68	17.12	4.59E-01	24.13	20.87	2.47E-01	15.45	10.72	1.50E-01
cg13236166	-9.27	4.38	3.46E-02	-17.94	7.82	2.19E-02	-3.41	12.39	7.83E-01	-7.32	6.71	2.75E-01	-0.62	11.97	9.59E-01
cg07791011	9.42	4.46	3.46E-02	5.26	6.65	4.29E-01	15.35	10.77	1.54E-01	27.00	16.42	1.00E-01	7.98	8.07	3.23E-01
cg05855116	30.38	14.37	3.46E-02	22.54	19.84	2.56E-01	20.30	44.07	6.45E-01	26.96	41.66	5.18E-01	52.75	28.75	6.66E-02
cg21759953	-20.82	9.85	3.46E-02	-28.81	16.17	7.47E-02	-3.02	21.61	8.89E-01	-27.81	20.71	1.79E-01	-16.47	22.36	4.61E-01
cg12593634	-10.03	4.75	3.46E-02	-16.14	8.15	4.77E-02	-5.63	11.65	6.29E-01	-8.04	8.68	3.54E-01	-6.21	10.73	5.63E-01
cg26189021	-9.60	4.55	3.46E-02	-14.38	6.77	3.36E-02	-14.69	10.54	1.63E-01	5.42	14.30	7.05E-01	-3.57	8.88	6.88E-01
cg13865891	15.51	7.34	3.46E-02	17.38	14.22	2.22E-01	-0.53	36.22	9.88E-01	19.42	9.49	4.07E-02	-7.68	23.95	7.49E-01
cg00739353	18.89	8.94	3.46E-02	25.32	12.82	4.83E-02	22.29	18.05	2.17E-01	0.83	26.44	9.75E-01	6.62	22.76	7.71E-01
cg12666279	16.55	7.83	3.46E-02	28.57	9.40	2.37E-03	1.53	27.70	9.56E-01	22.36	14.68	1.28E-01	0.05	11.75	9.97E-01

cg09078100	12.98	6.14	3.46E-02	5.02	15.74	7.50E-01	28.34	24.03	2.38E-01	7.00	5.78	2.26E-01	29.97	12.05	1.29E-02
cg19422460	18.65	8.83	3.46E-02	27.59	13.53	4.14E-02	43.83	21.41	4.06E-02	4.85	8.14	5.51E-01	23.18	24.28	3.40E-01
cg04738656	28.08	13.29	3.46E-02	-25.58	38.28	5.04E-01	25.80	136.25	8.50E-01	37.27	14.87	1.22E-02	15.97	49.88	7.49E-01
cg11754995	30.13	14.26	3.46E-02	56.78	36.57	1.20E-01	-17.09	85.50	8.42E-01	31.99	16.69	5.53E-02	-15.17	47.45	7.49E-01
cg03091551	14.87	7.04	3.46E-02	18.27	11.92	1.25E-01	17.45	19.76	3.77E-01	15.52	16.78	3.55E-01	10.19	11.92	3.92E-01
cg10699731	26.44	12.52	3.46E-02	37.49	22.30	9.28E-02	105.77	72.77	1.46E-01	29.40	16.56	7.58E-02	-10.36	27.97	7.11E-01
cg03116837	-2.77	1.31	3.46E-02	-4.09	1.96	3.67E-02	-4.02	3.15	2.02E-01	-0.91	2.80	7.46E-01	-0.24	3.31	9.41E-01
cg19279342	7.59	3.59	3.46E-02	0.63	6.26	9.19E-01	3.08	6.94	6.58E-01	15.75	6.08	9.55E-03	10.23	7.06	1.47E-01
cg04928930	6.00	2.84	3.46E-02	5.80	3.64	1.11E-01	14.83	7.85	5.89E-02	2.85	7.92	7.19E-01	1.23	7.83	8.75E-01
cg11721566	-15.78	7.47	3.46E-02	-19.55	11.60	9.18E-02	0.53	18.75	9.78E-01	-25.62	16.61	1.23E-01	-11.48	15.78	4.67E-01
cg03776042	17.82	8.43	3.46E-02	19.16	17.35	2.70E-01	18.21	14.62	2.13E-01	11.06	16.99	5.15E-01	24.39	19.61	2.14E-01
cg23404101	-14.63	6.93	3.46E-02	-7.11	13.53	5.99E-01	-46.47	38.81	2.31E-01	-12.81	8.89	1.49E-01	-35.48	22.01	1.07E-01
cg03936436	20.82	9.86	3.46E-02	14.16	19.76	4.74E-01	8.40	20.27	6.78E-01	35.91	15.70	2.22E-02	9.65	28.36	7.34E-01
cg24149904	11.98	5.67	3.46E-02	15.98	8.53	6.11E-02	7.67	14.16	5.88E-01	13.74	12.64	2.77E-01	4.73	12.75	7.11E-01
cg06230736	24.59	11.64	3.46E-02	21.90	17.53	2.12E-01	29.02	40.18	4.70E-01	29.08	27.21	2.85E-01	24.55	21.51	2.54E-01
cg13580008	-36.97	17.50	3.46E-02	-42.19	21.18	4.64E-02	-33.99	60.87	5.77E-01	-24.22	63.02	7.01E-01	-22.18	44.06	6.15E-01
cg11659501	27.50	13.02	3.46E-02	42.95	11.76	2.59E-04	4.59	18.29	8.02E-01	60.67	20.86	3.64E-03	5.99	13.25	6.51E-01
cg14345154	4.20	1.99	3.46E-02	4.20	2.87	1.43E-01	4.60	4.68	3.25E-01	4.38	4.02	2.76E-01	2.94	6.47	6.49E-01
cg06651508	22.71	10.75	3.46E-02	57.09	21.28	7.29E-03	17.89	25.29	4.79E-01	11.11	9.53	2.44E-01	23.96	31.78	4.51E-01
cg14584085	-15.50	7.34	3.46E-02	-7.46	15.86	6.38E-01	-22.47	28.15	4.25E-01	-17.91	9.35	5.54E-02	-13.20	23.03	5.67E-01
cg09901342	15.62	7.40	3.47E-02	20.16	9.83	4.02E-02	20.26	20.02	3.12E-01	17.80	31.43	5.71E-01	1.86	15.05	9.02E-01
cg05398773	26.02	12.32	3.47E-02	38.18	30.68	2.13E-01	12.14	88.50	8.91E-01	23.63	14.36	9.98E-02	26.78	42.56	5.29E-01
cg05035508	14.46	6.85	3.47E-02	16.66	17.43	3.39E-01	17.00	18.90	3.68E-01	14.60	8.64	9.09E-02	5.61	23.36	8.10E-01
cg16554447	-7.47	3.54	3.47E-02	-8.25	7.10	2.45E-01	3.24	11.84	7.85E-01	-7.93	4.93	1.08E-01	-11.08	9.24	2.30E-01
cg25010771	-41.63	19.71	3.47E-02	-14.52	28.93	6.16E-01	-11.21	69.31	8.72E-01	-79.13	38.92	4.20E-02	-68.99	44.25	1.19E-01
cg13911501	13.18	6.24	3.47E-02	18.88	9.98	5.86E-02	-0.45	8.52	9.58E-01	20.03	14.02	1.53E-01	24.83	13.35	6.28E-02
cg20165459	39.14	18.53	3.47E-02	52.74	24.36	3.04E-02	67.49	32.47	3.77E-02	5.52	10.23	5.90E-01	62.19	32.86	5.84E-02
cg04700283	17.92	8.48	3.47E-02	16.87	27.03	5.33E-01	-101.85	88.00	2.47E-01	20.84	9.29	2.48E-02	-3.21	35.24	9.27E-01
cg13997278	-19.52	9.24	3.47E-02	-22.27	12.25	6.90E-02	4.71	57.93	9.35E-01	-30.31	16.67	6.90E-02	24.25	29.61	4.13E-01
cg04680150	16.38	7.76	3.47E-02	11.75	17.89	5.11E-01	-6.94	21.29	7.44E-01	21.75	10.55	3.92E-02	24.05	20.83	2.48E-01
cg18201077	38.49	18.23	3.47E-02	42.28	21.88	5.33E-02	45.33	80.88	5.75E-01	-20.92	69.94	7.65E-01	44.13	42.08	2.94E-01
cg21242663	16.77	7.94	3.47E-02	12.94	22.22	5.60E-01	3.11	25.50	9.03E-01	17.88	9.40	5.71E-02	33.26	31.96	2.98E-01
cg24446009	13.29	6.30	3.47E-02	2.34	16.41	8.86E-01	21.56	23.99	3.69E-01	13.13	7.32	7.29E-02	39.08	29.65	1.87E-01
cg07938869	19.52	9.24	3.47E-02	-1.84	23.31	9.37E-01	9.61	54.26	8.59E-01	22.60	10.95	3.91E-02	33.71	28.90	2.43E-01
cg26726006	8.81	4.17	3.47E-02	12.56	4.75	8.26E-03	1.11	8.32	8.93E-01	24.33	11.88	4.06E-02	2.44	6.71	7.16E-01

cg08233142	30.62	14.50	3.47E-02	22.96	40.69	5.72E-01	183.46	161.54	2.56E-01	30.07	16.38	6.64E-02	32.71	50.82	5.20E-01
cg03668539	15.09	7.15	3.47E-02	11.20	18.52	5.45E-01	-7.61	25.67	7.67E-01	18.75	8.50	2.75E-02	11.49	27.62	6.77E-01
cg07159875	13.00	6.16	3.47E-02	14.17	9.03	1.17E-01	-2.03	13.53	8.81E-01	17.47	14.61	2.32E-01	24.74	15.83	1.18E-01
cg12004641	-10.77	5.10	3.47E-02	-18.47	7.52	1.40E-02	2.12	11.83	8.58E-01	-8.90	12.86	4.89E-01	-6.42	11.48	5.76E-01
cg02072875	-7.51	3.56	3.47E-02	-8.50	7.23	2.40E-01	13.19	19.81	5.06E-01	-6.37	4.56	1.62E-01	-17.30	10.47	9.84E-02
cg02709432	-19.28	9.13	3.47E-02	-21.87	11.89	6.59E-02	24.79	31.21	4.27E-01	-15.09	41.50	7.16E-01	-28.09	17.34	1.05E-01
cg21663916	-8.74	4.14	3.47E-02	-20.16	8.57	1.86E-02	-2.87	8.33	7.31E-01	-7.39	7.12	3.00E-01	-4.57	9.69	6.37E-01
cg26072930	12.06	5.71	3.47E-02	8.90	14.86	5.49E-01	30.65	17.43	7.87E-02	12.80	7.40	8.35E-02	-1.16	14.78	9.37E-01
cg14838970	8.37	3.97	3.47E-02	15.11	5.65	7.51E-03	2.22	8.61	7.96E-01	9.28	9.83	3.45E-01	-1.34	8.79	8.79E-01
cg14906103	7.14	3.38	3.47E-02	5.06	5.45	3.53E-01	10.04	8.04	2.12E-01	7.98	6.04	1.86E-01	7.28	9.52	4.44E-01
cg04250847	21.78	10.31	3.47E-02	22.57	14.44	1.18E-01	10.59	29.58	7.20E-01	67.66	30.43	2.62E-02	6.30	19.43	7.46E-01
cg03169242	-16.34	7.74	3.47E-02	-20.91	11.56	7.04E-02	-15.16	16.45	3.57E-01	-6.69	20.19	7.40E-01	-14.31	18.05	4.28E-01
cg00334274	-9.27	4.39	3.47E-02	-10.00	6.83	1.43E-01	-9.66	11.32	3.94E-01	-16.35	9.46	8.39E-02	-0.73	9.34	9.37E-01
cg27421105	8.68	4.11	3.47E-02	5.15	6.32	4.16E-01	5.78	8.03	4.71E-01	22.26	12.14	6.67E-02	12.13	9.17	1.86E-01
cg17591343	-24.31	11.51	3.47E-02	-22.39	13.18	8.95E-02	-57.83	42.59	1.75E-01	-47.97	66.73	4.72E-01	-11.77	31.37	7.08E-01
cg08989558	17.78	8.42	3.47E-02	19.58	20.73	3.45E-01	19.27	17.35	2.67E-01	21.51	12.08	7.49E-02	-3.93	24.98	8.75E-01
cg18806378	17.19	8.14	3.47E-02	8.51	20.39	6.76E-01	-9.10	65.62	8.90E-01	21.12	9.85	3.20E-02	10.87	21.62	6.15E-01
cg18107006	5.66	2.68	3.47E-02	3.63	3.61	3.15E-01	10.33	6.60	1.17E-01	14.17	7.34	5.34E-02	0.41	6.92	9.53E-01
cg06431105	-35.62	16.87	3.47E-02	-26.89	21.48	2.11E-01	31.61	54.42	5.61E-01	-86.80	43.08	4.39E-02	-48.15	31.72	1.29E-01
cg23496571	-13.66	6.47	3.47E-02	-16.98	11.81	1.50E-01	-5.69	15.59	7.15E-01	-13.96	11.66	2.31E-01	-14.97	13.82	2.79E-01
cg27364780	12.33	5.84	3.47E-02	14.28	14.37	3.20E-01	11.75	22.44	6.01E-01	14.12	7.04	4.50E-02	-6.61	20.67	7.49E-01
cg26157802	18.93	8.97	3.48E-02	33.51	29.84	2.61E-01	-72.51	95.44	4.47E-01	19.52	9.74	4.51E-02	0.06	38.90	9.99E-01
cg10315334	-9.44	4.47	3.48E-02	-9.76	6.61	1.40E-01	-16.75	10.96	1.27E-01	0.28	10.95	9.79E-01	-10.65	9.76	2.75E-01
cg19092343	-11.49	5.44	3.48E-02	-15.97	9.33	8.71E-02	-15.44	16.68	3.55E-01	-2.44	6.29	6.98E-01	-25.81	11.80	2.87E-02
cg24186249	-15.85	7.51	3.48E-02	-14.15	20.65	4.93E-01	-7.82	36.36	8.30E-01	-14.88	8.63	8.47E-02	-34.60	28.56	2.26E-01
cg18864857	-8.94	4.24	3.48E-02	-9.55	6.56	1.46E-01	-17.82	17.26	3.02E-01	-13.38	6.83	5.00E-02	7.85	11.02	4.76E-01
cg23106045	-4.21	2.00	3.48E-02	-3.05	2.91	2.95E-01	-8.37	5.01	9.52E-02	-4.52	5.62	4.21E-01	-3.61	4.03	3.71E-01
cg03763616	10.10	4.78	3.48E-02	12.40	6.74	6.57E-02	20.15	11.02	6.75E-02	4.10	13.00	7.52E-01	-2.94	11.53	7.99E-01
cg17079497	-11.51	5.45	3.48E-02	-3.95	11.80	7.38E-01	-22.87	16.73	1.72E-01	-9.47	7.44	2.03E-01	-21.99	14.40	1.27E-01
cg18328190	22.30	10.56	3.48E-02	29.54	16.47	7.28E-02	51.42	22.09	1.99E-02	5.73	7.20	4.26E-01	28.14	22.65	2.14E-01
cg13413955	12.15	5.75	3.48E-02	14.23	9.92	1.52E-01	18.39	12.90	1.54E-01	5.99	13.91	6.67E-01	9.12	10.62	3.90E-01
cg26208644	-20.10	9.52	3.48E-02	2.69	25.84	9.17E-01	-67.90	39.94	8.91E-02	-22.39	11.16	4.47E-02	-3.69	33.95	9.13E-01
cg05249479	17.77	8.42	3.48E-02	15.01	10.59	1.56E-01	40.39	27.64	1.44E-01	50.97	35.57	1.52E-01	7.66	17.99	6.70E-01
cg14201324	12.22	5.79	3.48E-02	2.49	14.50	8.63E-01	47.82	27.04	7.70E-02	14.07	7.36	5.58E-02	5.24	13.81	7.04E-01
cg14829606	26.22	12.42	3.48E-02	35.91	18.16	4.80E-02	38.73	29.05	1.83E-01	-19.46	41.74	6.41E-01	15.58	24.33	5.22E-01

cg23893460	15.63	7.41	3.48E-02	12.22	10.66	2.52E-01	23.82	14.37	9.75E-02	-0.06	22.07	9.98E-01	24.50	19.81	2.16E-01
cg23300897	-29.84	14.14	3.48E-02	-38.25	16.52	2.06E-02	-21.66	46.93	6.44E-01	-27.51	52.75	6.02E-01	20.15	43.64	6.44E-01
cg19687230	9.39	4.45	3.48E-02	10.23	6.13	9.53E-02	15.63	10.42	1.33E-01	-2.26	14.63	8.77E-01	6.86	9.98	4.92E-01
cg26365742	17.89	8.48	3.48E-02	18.34	11.05	9.69E-02	-6.75	35.21	8.48E-01	47.47	36.60	1.95E-01	16.48	15.48	2.87E-01
cg10089865	11.71	5.55	3.48E-02	16.91	16.77	3.13E-01	-23.88	29.76	4.22E-01	12.27	6.29	5.12E-02	14.68	19.83	4.59E-01
cg24435669	-9.44	4.47	3.48E-02	-11.32	6.72	9.21E-02	-7.30	8.99	4.16E-01	-2.10	13.74	8.79E-01	-11.76	9.91	2.35E-01
cg09999984	-12.01	5.69	3.48E-02	-19.27	12.36	1.19E-01	0.35	16.72	9.83E-01	-17.96	7.54	1.72E-02	4.74	13.03	7.16E-01
cg22022881	-3.48	1.65	3.48E-02	-3.14	2.28	1.69E-01	-2.12	3.86	5.82E-01	-0.22	4.54	9.61E-01	-8.84	4.11	3.17E-02
cg07011231	-17.89	8.48	3.48E-02	-27.82	18.96	1.42E-01	-56.29	39.32	1.52E-01	-14.32	10.46	1.71E-01	-3.25	27.16	9.05E-01
cg09240793	-7.18	3.40	3.48E-02	-9.77	5.46	7.36E-02	-6.66	7.70	3.87E-01	-7.33	6.40	2.52E-01	-0.09	9.32	9.93E-01
cg22151113	26.72	12.66	3.48E-02	21.43	18.84	2.55E-01	11.82	25.54	6.43E-01	58.92	35.47	9.67E-02	37.86	30.27	2.11E-01
cg05174975	-10.35	4.90	3.48E-02	-10.42	7.31	1.54E-01	-3.65	13.82	7.91E-01	-4.67	10.93	6.69E-01	-19.08	10.37	6.59E-02
cg14899164	-69.70	33.03	3.48E-02	-52.19	42.94	2.24E-01	41.32	149.08	7.82E-01	-145.67	85.30	8.77E-02	-90.81	72.19	2.08E-01
cg26852159	16.32	7.73	3.48E-02	27.25	16.74	1.03E-01	-0.56	32.01	9.86E-01	11.10	9.41	2.38E-01	57.93	33.79	8.64E-02
cg03340303	-11.65	5.52	3.48E-02	18.07	20.98	3.89E-01	-14.56	7.23	4.40E-02	-12.89	9.76	1.87E-01	-10.37	33.00	7.53E-01
cg16688582	-10.38	4.92	3.48E-02	-4.58	13.64	7.37E-01	12.13	23.22	6.01E-01	-10.71	5.63	5.71E-02	-34.85	19.79	7.83E-02
cg16885235	-9.59	4.55	3.48E-02	-9.33	10.84	3.89E-01	-5.64	20.06	7.79E-01	-10.44	5.48	5.67E-02	-5.64	15.62	7.18E-01
cg16803791	23.09	10.94	3.48E-02	34.77	18.63	6.19E-02	-1.72	37.24	9.63E-01	31.65	20.16	1.16E-01	7.01	20.88	7.37E-01
cg16643542	5.61	2.66	3.48E-02	4.53	3.10	1.44E-01	10.02	7.03	1.54E-01	6.62	11.18	5.54E-01	7.29	10.47	4.86E-01
cg24654264	17.34	8.22	3.48E-02	32.60	11.21	3.65E-03	19.77	19.46	3.10E-01	15.88	24.43	5.16E-01	2.16	10.85	8.42E-01
cg26889552	14.42	6.83	3.48E-02	16.97	16.73	3.10E-01	24.10	17.42	1.67E-01	10.18	8.73	2.43E-01	24.81	26.60	3.51E-01
cg27389255	9.99	4.73	3.48E-02	8.63	7.41	2.44E-01	14.52	11.11	1.91E-01	12.64	8.75	1.49E-01	1.16	13.78	9.33E-01
cg08713474	13.04	6.18	3.48E-02	17.95	14.72	2.23E-01	8.87	21.27	6.76E-01	8.55	7.77	2.71E-01	34.57	18.82	6.62E-02
cg23401756	9.73	4.61	3.48E-02	11.64	7.84	1.38E-01	9.22	9.18	3.15E-01	16.30	10.93	1.36E-01	2.15	9.72	8.25E-01
cg04428139	9.62	4.56	3.48E-02	12.99	6.71	5.27E-02	16.60	10.94	1.29E-01	18.02	17.04	2.90E-01	-1.89	8.42	8.22E-01
cg20864608	29.33	13.90	3.48E-02	59.46	44.49	1.81E-01	61.27	169.02	7.17E-01	23.82	15.31	1.20E-01	48.62	51.93	3.49E-01
cg26267678	23.52	11.15	3.48E-02	39.56	17.26	2.19E-02	53.84	35.37	1.28E-01	7.15	26.58	7.88E-01	3.52	19.12	8.54E-01
cg09694921	14.67	6.95	3.48E-02	11.96	10.84	2.70E-01	12.97	13.70	3.44E-01	10.19	17.72	5.65E-01	27.42	16.56	9.77E-02
cg08277287	11.36	5.38	3.49E-02	3.91	14.48	7.87E-01	35.81	18.45	5.23E-02	10.01	6.46	1.21E-01	9.92	18.69	5.96E-01
cg00968021	18.13	8.59	3.49E-02	33.16	18.32	7.03E-02	117.26	78.03	1.33E-01	14.04	11.61	2.26E-01	7.81	18.34	6.70E-01
cg16655795	10.48	4.97	3.49E-02	6.20	6.44	3.36E-01	27.61	10.96	1.17E-02	5.23	16.18	7.46E-01	6.73	12.57	5.93E-01
cg24934063	18.67	8.85	3.49E-02	5.98	26.94	8.24E-01	22.72	40.35	5.73E-01	25.37	11.00	2.11E-02	2.65	19.94	8.94E-01
cg27505440	-9.79	4.64	3.49E-02	-15.16	9.22	1.00E-01	-8.03	10.75	4.55E-01	-11.75	6.79	8.35E-02	11.04	15.17	4.67E-01
cg16340767	11.14	5.28	3.49E-02	6.34	6.83	3.53E-01	24.30	14.57	9.54E-02	18.30	15.74	2.45E-01	13.24	13.26	3.18E-01
cg10217202	7.72	3.66	3.49E-02	10.29	7.19	1.53E-01	5.10	8.47	5.47E-01	6.54	5.91	2.68E-01	9.33	8.85	2.92E-01

cg25948831	13.50	6.40	3.49E-02	12.31	12.11	3.09E-01	-3.85	15.47	8.04E-01	20.79	9.51	2.88E-02	13.53	20.58	5.11E-01
cg17165284	-41.56	19.70	3.49E-02	-57.20	49.66	2.49E-01	-184.71	210.29	3.80E-01	-31.03	22.72	1.72E-01	-92.88	68.84	1.77E-01
cg05448404	6.16	2.92	3.49E-02	3.80	4.09	3.53E-01	11.26	6.19	6.88E-02	-3.13	15.28	8.38E-01	7.92	6.07	1.92E-01
cg12511310	-13.54	6.42	3.49E-02	-6.92	16.59	6.77E-01	-0.03	23.82	9.99E-01	-13.16	7.58	8.25E-02	-50.19	25.96	5.32E-02
cg19925035	18.99	9.00	3.49E-02	10.83	17.84	5.44E-01	-8.14	43.46	8.51E-01	24.27	11.79	3.96E-02	20.37	25.99	4.33E-01
cg01574673	16.93	8.02	3.49E-02	8.61	11.76	4.64E-01	42.01	21.47	5.04E-02	24.71	24.48	3.13E-01	15.30	14.96	3.06E-01
cg16026501	18.38	8.71	3.49E-02	23.58	18.09	1.92E-01	6.56	19.53	7.37E-01	13.05	13.08	3.18E-01	46.39	24.62	5.95E-02
cg01901434	-8.58	4.07	3.49E-02	-7.57	8.08	3.49E-01	-2.56	13.33	8.48E-01	-9.67	5.56	8.21E-02	-10.52	11.76	3.71E-01
cg16250145	-13.82	6.55	3.49E-02	-25.30	11.79	3.19E-02	-26.28	21.45	2.21E-01	-8.58	9.68	3.76E-01	2.60	17.47	8.82E-01
cg06193043	-5.83	2.76	3.49E-02	-3.28	3.95	4.07E-01	-3.00	5.97	6.16E-01	-16.65	8.56	5.17E-02	-9.61	6.30	1.27E-01
cg11590282	10.85	5.15	3.49E-02	13.83	6.34	2.91E-02	0.84	8.55	9.21E-01	-2.06	17.48	9.06E-01	24.13	10.75	2.48E-02
cg04714088	-45.25	21.45	3.49E-02	-7.77	52.41	8.82E-01	-0.15	181.88	9.99E-01	-54.22	25.16	3.11E-02	-49.44	70.88	4.86E-01
cg06853894	8.68	4.11	3.49E-02	8.22	5.20	1.14E-01	20.98	9.65	2.97E-02	-2.21	16.42	8.93E-01	-1.09	11.44	9.24E-01
cg26353296	23.01	10.91	3.49E-02	22.09	14.13	1.18E-01	32.70	57.89	5.72E-01	86.02	66.13	1.93E-01	18.58	18.68	3.20E-01
cg04316231	22.69	10.76	3.49E-02	4.99	19.09	7.94E-01	22.04	19.37	2.55E-01	35.75	24.05	1.37E-01	41.19	25.81	1.11E-01
cg23610053	10.70	5.07	3.49E-02	14.12	12.53	2.60E-01	-1.16	39.70	9.77E-01	12.67	5.96	3.37E-02	-7.84	16.33	6.31E-01
cg15221831	99.67	47.25	3.49E-02	120.02	68.91	8.16E-02	251.24	227.02	2.68E-01	-60.48	117.85	6.08E-01	129.16	82.78	1.19E-01
cg09459719	11.86	5.62	3.49E-02	12.34	14.01	3.78E-01	25.08	42.52	5.55E-01	11.91	6.70	7.54E-02	8.91	16.43	5.87E-01
cg03849707	17.92	8.50	3.49E-02	16.06	14.69	2.74E-01	16.32	17.34	3.47E-01	13.37	16.95	4.30E-01	30.26	20.36	1.37E-01
cg05041351	17.32	8.21	3.49E-02	47.96	20.97	2.22E-02	0.24	33.45	9.94E-01	16.26	10.01	1.04E-01	1.75	18.66	9.25E-01
cg02698886	7.63	3.62	3.49E-02	5.24	5.34	3.27E-01	7.78	9.22	3.99E-01	15.58	7.08	2.78E-02	-0.34	10.21	9.74E-01
cg14321708	18.35	8.70	3.49E-02	11.65	14.63	4.26E-01	26.42	16.57	1.11E-01	7.03	18.78	7.08E-01	34.91	22.05	1.13E-01
cg04953958	11.56	5.48	3.49E-02	9.37	7.61	2.18E-01	6.15	10.98	5.76E-01	26.18	18.77	1.63E-01	20.06	14.35	1.62E-01
cg06799756	15.83	7.51	3.49E-02	17.39	17.30	3.15E-01	18.61	19.60	3.42E-01	15.48	9.74	1.12E-01	8.91	28.30	7.53E-01
cg08224208	15.10	7.16	3.49E-02	7.81	13.48	5.62E-01	8.68	28.36	7.60E-01	16.53	10.29	1.08E-01	25.53	17.37	1.42E-01
cg07596209	34.40	16.31	3.49E-02	16.66	52.96	7.53E-01	-128.24	222.97	5.65E-01	42.68	17.98	1.76E-02	-21.24	58.86	7.18E-01
cg17364411	15.64	7.42	3.50E-02	25.85	14.12	6.72E-02	0.16	17.35	9.93E-01	18.81	11.41	9.93E-02	4.51	21.50	8.34E-01
cg21380560	21.33	10.11	3.50E-02	26.99	14.10	5.56E-02	16.77	34.23	6.24E-01	33.02	35.33	3.50E-01	10.34	17.98	5.65E-01
cg21547649	7.25	3.44	3.50E-02	6.87	4.61	1.36E-01	10.38	7.48	1.65E-01	16.16	12.24	1.87E-01	-0.26	8.79	9.76E-01
cg24306982	16.83	7.98	3.50E-02	25.97	15.83	1.01E-01	7.80	18.51	6.74E-01	13.01	17.49	4.57E-01	17.25	13.45	2.00E-01
cg21784940	8.54	4.05	3.50E-02	9.96	7.99	2.13E-01	17.73	9.92	7.39E-02	7.87	5.77	1.73E-01	-10.23	14.03	4.66E-01
cg27320734	-22.53	10.69	3.50E-02	-30.00	18.06	9.67E-02	20.92	30.62	4.94E-01	-34.47	20.41	9.13E-02	-20.20	21.19	3.41E-01
cg22218588	14.77	7.00	3.50E-02	6.52	19.43	7.37E-01	26.51	31.11	3.94E-01	16.85	8.22	4.05E-02	3.78	22.85	8.69E-01
cg20185945	-10.76	5.10	3.50E-02	-15.60	13.53	2.49E-01	-29.11	17.16	8.98E-02	-5.90	6.27	3.47E-01	-18.99	15.46	2.19E-01
cg03056614	19.65	9.32	3.50E-02	32.67	16.80	5.18E-02	26.34	23.25	2.57E-01	5.90	14.87	6.92E-01	22.02	25.06	3.80E-01

cg14435092	37.19	17.64	3.50E-02	-31.53	44.30	4.77E-01	16.11	228.01	9.44E-01	45.03	17.07	8.34E-03	75.58	49.47	1.27E-01
cg18275620	-13.41	6.36	3.50E-02	-13.13	13.58	3.33E-01	-0.08	17.29	9.96E-01	-15.62	8.70	7.26E-02	-19.58	19.10	3.05E-01
cg15727564	47.10	22.34	3.50E-02	27.19	57.05	6.34E-01	-8.27	270.33	9.76E-01	57.47	25.81	2.60E-02	-0.64	74.09	9.93E-01
cg21917866	12.12	5.75	3.50E-02	11.81	10.76	2.73E-01	4.05	12.71	7.50E-01	12.69	11.76	2.80E-01	18.04	11.05	1.02E-01
cg14673319	18.11	8.59	3.50E-02	44.93	22.47	4.56E-02	36.48	42.59	3.92E-01	9.50	9.47	3.16E-01	43.13	31.73	1.74E-01
cg23731836	31.06	14.73	3.50E-02	34.95	17.99	5.20E-02	99.27	105.59	3.47E-01	72.21	49.78	1.47E-01	-2.90	31.26	9.26E-01
cg06079509	-12.95	6.14	3.50E-02	-21.90	10.52	3.74E-02	-1.80	12.64	8.87E-01	-13.44	12.99	3.01E-01	-10.30	13.73	4.53E-01
cg25220537	-8.37	3.97	3.50E-02	-14.58	7.50	5.18E-02	-1.28	8.20	8.76E-01	-9.73	6.50	1.34E-01	-3.15	11.88	7.91E-01
cg24702451	21.61	10.25	3.50E-02	32.95	18.87	8.07E-02	10.93	20.92	6.01E-01	21.49	18.86	2.55E-01	17.18	24.87	4.90E-01
cg24084606	-11.37	5.39	3.50E-02	-13.22	10.03	1.88E-01	3.43	11.55	7.67E-01	-14.86	9.00	9.89E-02	-22.12	14.73	1.33E-01
cg23519155	-12.57	5.96	3.50E-02	-16.63	9.60	8.30E-02	-8.36	12.83	5.15E-01	5.05	13.91	7.17E-01	-24.66	12.90	5.60E-02
cg12998599	13.95	6.62	3.50E-02	27.04	15.40	7.90E-02	26.21	28.99	3.66E-01	12.69	7.95	1.10E-01	-17.24	25.00	4.90E-01
cg16326902	17.32	8.22	3.50E-02	3.69	12.90	7.75E-01	37.82	14.14	7.47E-03	8.96	8.84	3.11E-01	33.98	21.10	1.07E-01
cg24025255	8.32	3.95	3.50E-02	10.58	5.96	7.59E-02	7.10	10.04	4.79E-01	14.82	8.60	8.48E-02	-2.74	8.91	7.58E-01
cg26926138	13.89	6.59	3.50E-02	25.47	13.19	5.36E-02	29.43	15.94	6.48E-02	7.36	12.35	5.51E-01	1.79	11.91	8.81E-01
cg07737644	-15.18	7.20	3.50E-02	-32.09	10.53	2.30E-03	-1.01	12.91	9.37E-01	-6.70	7.22	3.53E-01	-23.45	13.53	8.30E-02
cg24094513	12.72	6.03	3.50E-02	9.81	11.08	3.76E-01	19.77	17.34	2.54E-01	14.09	9.84	1.52E-01	10.27	13.30	4.40E-01
cg04919592	5.99	2.84	3.50E-02	5.95	3.48	8.71E-02	14.02	8.21	8.75E-02	2.25	11.37	8.43E-01	1.30	7.35	8.59E-01
cg21150901	17.50	8.30	3.50E-02	22.31	11.59	5.43E-02	3.08	45.70	9.46E-01	49.25	27.21	7.03E-02	3.79	13.83	7.84E-01
cg08082142	-37.56	17.82	3.50E-02	-36.40	21.05	8.38E-02	-31.14	132.73	8.15E-01	25.43	65.09	6.96E-01	-67.26	40.80	9.92E-02
cg09612534	18.10	8.59	3.50E-02	4.23	18.12	8.15E-01	25.70	16.92	1.29E-01	6.80	16.48	6.80E-01	35.29	17.31	4.15E-02
cg13147822	26.78	12.70	3.50E-02	63.06	36.71	8.58E-02	34.05	100.25	7.34E-01	22.56	14.23	1.13E-01	10.38	49.09	8.33E-01
cg01350293	10.47	4.97	3.50E-02	23.27	15.26	1.27E-01	27.36	21.15	1.96E-01	6.48	5.64	2.51E-01	23.21	19.73	2.39E-01
cg04302628	-8.95	4.25	3.50E-02	-9.41	7.76	2.25E-01	-1.58	12.73	9.01E-01	-11.94	6.21	5.47E-02	-3.11	12.12	7.97E-01
cg07269125	13.13	6.23	3.50E-02	1.83	16.37	9.11E-01	-0.56	32.65	9.86E-01	13.86	7.11	5.12E-02	43.72	27.48	1.12E-01
cg00940560	7.79	3.70	3.50E-02	6.09	5.79	2.93E-01	7.72	6.72	2.51E-01	18.17	7.60	1.67E-02	-2.75	9.11	7.62E-01
cg10829120	-30.37	14.41	3.50E-02	-78.98	54.48	1.47E-01	-205.54	187.78	2.74E-01	-27.91	15.46	7.11E-02	10.69	61.00	8.61E-01
cg24158452	25.46	12.08	3.50E-02	-9.27	28.27	7.43E-01	-16.21	85.72	8.50E-01	34.01	14.28	1.73E-02	38.25	42.05	3.63E-01
cg27631794	26.47	12.56	3.50E-02	26.40	35.95	4.63E-01	17.56	150.70	9.07E-01	25.72	14.08	6.77E-02	35.25	45.68	4.40E-01
cg20037575	13.68	6.49	3.50E-02	22.06	14.56	1.30E-01	13.27	24.51	5.88E-01	10.59	8.13	1.93E-01	17.18	21.17	4.17E-01
cg20550154	-3.57	1.69	3.51E-02	-3.27	2.32	1.59E-01	-6.76	3.25	3.75E-02	-3.81	5.97	5.23E-01	2.69	4.97	5.89E-01
cg08594606	-56.82	26.96	3.51E-02	-16.63	34.96	6.34E-01	18.83	158.97	9.06E-01	-126.05	53.10	1.76E-02	-74.06	47.29	1.17E-01
cg02281854	-12.03	5.71	3.51E-02	-19.53	8.72	2.51E-02	-5.32	12.82	6.78E-01	-5.05	16.81	7.64E-01	-7.84	11.22	4.85E-01
cg02285725	-8.93	4.24	3.51E-02	-12.97	9.64	1.78E-01	-17.14	15.41	2.66E-01	-3.96	5.39	4.63E-01	-23.64	12.57	6.01E-02
cg01385157	44.07	20.91	3.51E-02	40.27	28.97	1.65E-01	68.05	58.11	2.42E-01	-11.07	52.47	8.33E-01	84.04	47.85	7.91E-02

cg12845432	21.39	10.15	3.51E-02	29.60	19.06	1.20E-01	24.06	24.67	3.29E-01	8.30	16.25	6.10E-01	36.15	25.58	1.58E-01
cg14443558	31.63	15.01	3.51E-02	37.64	18.13	3.79E-02	21.23	62.90	7.36E-01	54.41	44.24	2.19E-01	-11.31	39.66	7.76E-01
cg09267217	-16.77	7.96	3.51E-02	-14.30	23.43	5.42E-01	-62.71	94.89	5.09E-01	-14.70	8.90	9.84E-02	-37.29	28.41	1.89E-01
cg13333374	14.07	6.68	3.51E-02	13.92	11.32	2.19E-01	2.78	13.51	8.37E-01	21.24	13.51	1.16E-01	20.60	16.54	2.13E-01
cg19020434	-8.95	4.24	3.51E-02	-11.95	8.25	1.48E-01	-19.22	24.00	4.23E-01	-11.97	5.97	4.49E-02	4.42	9.54	6.43E-01
cg24329717	19.76	9.38	3.51E-02	44.90	17.88	1.21E-02	11.07	20.19	5.84E-01	13.36	15.28	3.82E-01	-0.01	25.76	1.00E+00
cg26467949	12.85	6.10	3.51E-02	11.54	8.11	1.55E-01	10.74	12.34	3.84E-01	6.40	29.96	8.31E-01	23.00	15.77	1.45E-01
cg19875852	9.96	4.73	3.51E-02	12.04	9.94	2.26E-01	17.85	14.27	2.11E-01	6.90	6.19	2.64E-01	15.51	16.65	3.52E-01
cg15822394	7.17	3.40	3.51E-02	3.95	4.86	4.16E-01	15.71	7.76	4.27E-02	3.48	8.44	6.80E-01	10.63	8.66	2.19E-01
cg02659338	10.10	4.79	3.51E-02	9.54	10.08	3.44E-01	4.16	12.20	7.33E-01	11.14	6.48	8.56E-02	16.78	17.92	3.49E-01
cg13492803	9.13	4.33	3.51E-02	11.89	6.71	7.62E-02	12.60	10.09	2.12E-01	10.89	11.34	3.37E-01	1.01	8.63	9.07E-01
cg16452651	4.99	2.37	3.51E-02	4.48	2.94	1.28E-01	10.58	6.39	9.81E-02	6.02	7.74	4.37E-01	0.66	6.76	9.22E-01
cg06226150	-13.35	6.34	3.51E-02	-13.16	9.71	1.75E-01	0.21	16.48	9.90E-01	-18.75	12.66	1.39E-01	-17.51	15.12	2.47E-01
cg03566787	-10.53	5.00	3.51E-02	-8.41	11.94	4.81E-01	-1.58	18.15	9.31E-01	-11.69	6.10	5.54E-02	-14.15	18.01	4.32E-01
cg18464991	-10.70	5.08	3.51E-02	-16.01	7.14	2.50E-02	-3.02	11.96	8.01E-01	2.92	18.13	8.72E-01	-9.71	10.44	3.52E-01
cg24720604	-11.86	5.63	3.51E-02	5.23	14.72	7.22E-01	-16.71	33.02	6.13E-01	-16.71	6.46	9.73E-03	7.92	21.76	7.16E-01
cg23517752	18.89	8.97	3.51E-02	16.41	18.72	3.81E-01	37.85	19.09	4.74E-02	13.40	13.65	3.26E-01	8.43	26.07	7.46E-01
cg25379649	22.73	10.79	3.51E-02	34.45	13.24	9.25E-03	3.74	36.13	9.18E-01	-20.17	59.22	7.33E-01	0.89	23.34	9.70E-01
cg13647601	33.85	16.06	3.51E-02	22.90	19.19	2.33E-01	26.80	70.92	7.05E-01	115.28	82.65	1.63E-01	57.47	35.06	1.01E-01
cg13142152	-9.53	4.52	3.51E-02	-13.61	8.85	1.24E-01	-5.48	15.78	7.29E-01	-7.36	6.29	2.42E-01	-12.40	12.15	3.08E-01
cg02166450	28.11	13.34	3.51E-02	56.68	27.25	3.75E-02	7.28	43.24	8.66E-01	23.73	19.25	2.18E-01	13.15	31.04	6.72E-01
cg07468397	-11.23	5.33	3.51E-02	-15.07	9.15	9.95E-02	-7.44	11.82	5.29E-01	-13.92	10.54	1.86E-01	-5.16	11.89	6.64E-01
cg18129755	11.79	5.59	3.51E-02	20.64	10.52	4.97E-02	6.24	15.24	6.82E-01	10.66	9.67	2.70E-01	6.21	11.25	5.81E-01
cg06890761	15.00	7.12	3.51E-02	13.73	13.79	3.19E-01	23.71	21.21	2.63E-01	16.02	10.00	1.09E-01	4.75	21.17	8.23E-01
cg22566787	20.31	9.64	3.51E-02	39.77	18.42	3.08E-02	10.67	19.42	5.83E-01	3.14	17.09	8.54E-01	35.74	23.95	1.36E-01
cg13462082	11.44	5.43	3.51E-02	8.72	6.15	1.56E-01	21.85	11.37	5.47E-02	27.05	14.56	6.32E-02	-2.52	11.53	8.27E-01
cg12496363	21.53	10.22	3.51E-02	14.13	17.86	4.29E-01	19.50	19.68	3.22E-01	30.71	20.80	1.40E-01	26.17	25.38	3.02E-01
cg27474149	-19.30	9.16	3.51E-02	-24.71	12.31	4.48E-02	-1.52	22.63	9.46E-01	-16.80	27.84	5.46E-01	-20.36	21.91	3.53E-01
cg09515767	24.43	11.60	3.51E-02	44.31	36.97	2.31E-01	-17.04	172.65	9.21E-01	22.67	12.66	7.32E-02	19.33	48.23	6.89E-01
cg19787366	-38.87	18.45	3.51E-02	-114.43	60.47	5.85E-02	-43.08	150.06	7.74E-01	-31.38	20.89	1.33E-01	-27.69	55.32	6.17E-01
cg08574758	15.87	7.53	3.51E-02	13.44	19.74	4.96E-01	43.34	20.15	3.15E-02	13.28	9.45	1.60E-01	-7.54	26.87	7.79E-01
cg01804500	14.71	6.98	3.51E-02	15.05	17.90	4.01E-01	1.39	18.21	9.39E-01	17.45	8.79	4.72E-02	17.16	26.22	5.13E-01
cg23307264	-8.34	3.96	3.51E-02	-3.45	8.74	6.93E-01	-10.21	13.30	4.43E-01	14.33	62.97	8.20E-01	-9.66	4.72	4.09E-02
cg00870449	-8.33	3.95	3.52E-02	-14.81	8.13	6.84E-02	-12.71	14.87	3.93E-01	-4.62	5.18	3.72E-01	-11.28	11.97	3.46E-01
cg07272654	8.67	4.11	3.52E-02	11.08	5.56	4.64E-02	8.94	9.68	3.56E-01	13.93	11.31	2.18E-01	-6.06	10.98	5.81E-01

cg13946191	19.46	9.24	3.52E-02	10.27	18.44	5.77E-01	4.94	21.73	8.20E-01	31.28	13.69	2.23E-02	15.42	27.49	5.75E-01
cg05717794	9.97	4.73	3.52E-02	11.91	6.33	6.01E-02	22.01	10.47	3.56E-02	-3.97	11.25	7.24E-01	4.09	11.74	7.28E-01
cg06355830	-26.39	12.53	3.52E-02	6.75	36.92	8.55E-01	-115.60	233.91	6.21E-01	-27.26	13.92	5.02E-02	-66.03	46.70	1.57E-01
cg13172696	13.96	6.63	3.52E-02	24.36	11.69	3.72E-02	11.19	25.35	6.59E-01	4.49	9.30	6.29E-01	30.13	20.68	1.45E-01
cg21777553	16.15	7.67	3.52E-02	21.41	10.29	3.75E-02	-2.03	20.97	9.23E-01	19.14	27.26	4.82E-01	13.03	15.91	4.13E-01
cg06968181	13.24	6.28	3.52E-02	27.74	17.57	1.14E-01	-36.22	77.96	6.42E-01	13.94	7.06	4.85E-02	-14.91	23.08	5.18E-01
cg02381965	76.69	36.41	3.52E-02	29.27	49.31	5.53E-01	23.17	187.50	9.02E-01	185.64	89.65	3.84E-02	115.89	72.48	1.10E-01
cg12712771	-21.16	10.04	3.52E-02	-35.20	17.88	4.89E-02	-44.58	29.88	1.36E-01	-10.32	15.81	5.14E-01	-5.01	24.52	8.38E-01
cg26322591	-10.68	5.07	3.52E-02	-4.42	7.07	5.31E-01	-21.86	12.67	8.45E-02	-16.37	14.09	2.46E-01	-14.27	11.49	2.14E-01
cg19666687	-13.25	6.29	3.52E-02	-0.13	13.84	9.92E-01	16.58	34.61	6.32E-01	-16.77	9.37	7.34E-02	-20.10	11.33	7.59E-02
cg03469682	-16.60	7.88	3.52E-02	-37.47	11.84	1.55E-03	5.51	15.27	7.18E-01	-13.83	7.17	5.36E-02	-15.91	15.48	3.04E-01
cg26705583	-25.35	12.04	3.52E-02	-4.03	20.55	8.44E-01	-18.88	29.28	5.19E-01	-49.97	20.60	1.53E-02	-25.36	31.43	4.20E-01
cg25424937	19.95	9.47	3.52E-02	25.08	20.01	2.10E-01	-7.25	41.89	8.63E-01	28.14	12.27	2.19E-02	-15.88	26.34	5.47E-01
cg00965133	17.84	8.47	3.52E-02	14.30	13.99	3.07E-01	27.72	15.43	7.25E-02	1.15	19.78	9.54E-01	27.10	21.94	2.17E-01
cg23371746	20.51	9.74	3.52E-02	13.29	30.87	6.67E-01	-150.37	114.69	1.90E-01	23.83	12.66	5.98E-02	20.47	17.74	2.48E-01
cg10207553	8.99	4.27	3.52E-02	13.14	6.52	4.39E-02	13.95	9.17	1.28E-01	5.22	11.16	6.40E-01	-2.10	9.38	8.23E-01
cg24082880	12.49	5.93	3.52E-02	14.51	13.26	2.74E-01	-3.54	19.01	8.52E-01	14.80	7.63	5.26E-02	10.06	18.81	5.93E-01
cg06423949	-16.30	7.74	3.52E-02	-7.27	18.75	6.98E-01	-0.19	30.79	9.95E-01	-21.90	9.30	1.85E-02	1.77	28.64	9.51E-01
cg08915603	8.86	4.21	3.52E-02	19.32	10.43	6.40E-02	1.78	11.92	8.81E-01	10.82	6.63	1.03E-01	3.67	7.55	6.27E-01
cg13139848	17.26	8.20	3.52E-02	7.24	13.13	5.81E-01	29.10	17.13	8.94E-02	29.38	16.86	8.14E-02	5.77	21.51	7.89E-01
cg16798537	-11.93	5.67	3.52E-02	-17.69	8.69	4.18E-02	-8.94	15.76	5.70E-01	-14.80	11.79	2.09E-01	0.74	12.23	9.52E-01
cg08390172	21.62	10.27	3.52E-02	37.60	15.11	1.29E-02	35.45	31.21	2.56E-01	15.35	32.86	6.40E-01	-0.51	16.86	9.76E-01
cg03209472	19.98	9.49	3.52E-02	34.56	17.34	4.63E-02	14.15	18.84	4.52E-01	25.28	17.44	1.47E-01	-9.52	24.41	6.97E-01
cg14073219	-35.01	16.63	3.52E-02	-54.40	45.39	2.31E-01	-126.84	226.59	5.76E-01	-30.02	19.11	1.16E-01	-41.61	51.74	4.21E-01
cg00360072	-49.25	23.39	3.52E-02	-113.92	60.71	6.06E-02	-277.92	282.07	3.24E-01	-40.70	27.08	1.33E-01	-0.66	74.53	9.93E-01
cg07001072	12.17	5.78	3.52E-02	5.73	11.30	6.12E-01	-17.03	22.74	4.54E-01	15.09	7.92	5.69E-02	26.35	15.33	8.56E-02
cg06902698	8.20	3.89	3.52E-02	4.98	4.63	2.82E-01	18.78	7.04	7.69E-03	12.34	10.15	2.24E-01	0.65	7.32	9.30E-01
cg17681516	6.15	2.92	3.52E-02	3.00	3.91	4.43E-01	7.80	6.71	2.45E-01	0.75	12.93	9.54E-01	14.74	6.51	2.37E-02
cg05926943	22.84	10.85	3.52E-02	31.73	40.80	4.37E-01	-37.65	151.62	8.04E-01	24.53	11.64	3.51E-02	-9.03	45.75	8.44E-01
cg00471371	5.50	2.61	3.52E-02	4.52	3.42	1.86E-01	8.12	6.49	2.11E-01	8.64	7.57	2.53E-01	3.83	7.15	5.93E-01
cg04635364	-8.05	3.82	3.52E-02	-9.83	5.43	7.02E-02	-12.88	8.16	1.14E-01	-11.17	5.33	3.60E-02	7.84	8.82	3.74E-01
cg02616368	-13.67	6.49	3.52E-02	-16.00	9.66	9.76E-02	-0.31	14.92	9.84E-01	-23.72	17.86	1.84E-01	-14.30	13.61	2.93E-01
cg22241838	-7.06	3.35	3.52E-02	-4.08	4.08	3.17E-01	-19.15	8.47	2.37E-02	-1.05	9.12	9.08E-01	-10.81	7.99	1.76E-01
cg26449586	10.34	4.91	3.52E-02	9.69	6.14	1.14E-01	26.91	9.91	6.62E-03	4.04	9.44	6.69E-01	1.97	9.85	8.42E-01
cg17490089	-6.60	3.14	3.52E-02	-10.34	4.37	1.80E-02	-4.46	7.68	5.61E-01	-0.61	10.96	9.56E-01	-2.05	6.44	7.50E-01

cg19255084	-15.90	7.55	3.52E-02	-34.84	16.48	3.45E-02	-15.83	29.87	5.96E-01	-11.45	9.31	2.19E-01	-0.43	29.12	9.88E-01
cg25098585	-20.55	9.76	3.52E-02	-26.05	15.30	8.86E-02	-25.42	25.87	3.26E-01	-20.66	21.22	3.30E-01	-8.19	19.96	6.82E-01
cg24215635	16.15	7.67	3.52E-02	30.06	17.01	7.72E-02	4.88	37.43	8.96E-01	11.49	9.57	2.30E-01	21.90	22.95	3.40E-01
cg02847140	19.00	9.02	3.52E-02	40.10	25.01	1.09E-01	-7.08	59.13	9.05E-01	14.89	10.45	1.54E-01	28.05	28.32	3.22E-01
cg15854394	-12.12	5.76	3.53E-02	-29.32	18.51	1.13E-01	14.35	24.63	5.60E-01	-9.25	7.14	1.95E-01	-20.45	12.92	1.14E-01
cg09468767	-24.85	11.80	3.53E-02	-44.38	40.20	2.70E-01	-86.01	154.60	5.78E-01	-23.57	13.49	8.07E-02	-17.44	31.19	5.76E-01
cg23480730	23.43	11.13	3.53E-02	31.84	16.09	4.78E-02	43.71	37.88	2.49E-01	52.56	37.11	1.57E-01	-0.88	18.93	9.63E-01
cg04606367	17.35	8.24	3.53E-02	15.63	13.79	2.57E-01	17.40	23.91	4.67E-01	8.40	18.16	6.44E-01	25.05	14.61	8.64E-02
cg21053051	-24.02	11.41	3.53E-02	-13.73	37.72	7.16E-01	-154.96	154.74	3.17E-01	-20.96	12.38	9.04E-02	-77.58	49.59	1.18E-01
cg02995731	-16.40	7.79	3.53E-02	-28.06	12.52	2.50E-02	-24.11	17.82	1.76E-01	4.35	18.41	8.13E-01	-7.04	15.80	6.56E-01
cg23337501	-18.09	8.59	3.53E-02	-12.82	17.92	4.74E-01	-74.92	35.73	3.60E-02	-11.84	8.54	1.65E-01	-31.32	22.80	1.70E-01
cg12418947	14.86	7.06	3.53E-02	26.26	13.65	5.45E-02	20.56	17.93	2.51E-01	9.30	13.60	4.94E-01	6.97	12.72	5.84E-01
cg26326208	11.79	5.60	3.53E-02	20.16	7.15	4.82E-03	17.57	13.36	1.88E-01	-4.66	16.77	7.81E-01	2.48	9.04	7.84E-01
cg06673130	7.37	3.50	3.53E-02	6.33	5.21	2.25E-01	14.57	8.59	9.00E-02	-2.66	9.93	7.89E-01	9.39	6.89	1.73E-01
cg04128880	12.62	5.99	3.53E-02	5.95	14.91	6.90E-01	-13.09	31.20	6.75E-01	15.93	7.04	2.38E-02	7.85	21.49	7.15E-01
cg19102271	33.00	15.68	3.53E-02	21.36	22.66	3.46E-01	1.16	42.33	9.78E-01	59.00	69.80	3.98E-01	58.83	27.13	3.01E-02
cg02844051	15.14	7.19	3.53E-02	17.58	16.61	2.90E-01	-25.11	42.07	5.51E-01	14.72	8.66	8.92E-02	25.87	23.48	2.71E-01
cg06171830	-8.76	4.16	3.53E-02	-11.74	9.06	1.95E-01	19.52	18.28	2.86E-01	-9.99	5.31	5.99E-02	-9.38	11.81	4.27E-01
cg00370173	-9.94	4.72	3.53E-02	-14.34	8.93	1.08E-01	-6.11	13.64	6.54E-01	-10.00	6.76	1.39E-01	-2.82	14.05	8.41E-01
cg21273856	43.47	20.65	3.53E-02	46.79	31.84	1.42E-01	-46.66	79.93	5.59E-01	29.40	14.84	4.76E-02	117.15	45.16	9.49E-03
cg13594465	-12.44	5.91	3.53E-02	-11.14	10.43	2.86E-01	-11.83	13.63	3.85E-01	-7.14	10.59	5.00E-01	-24.67	13.97	7.75E-02
cg14301000	-61.00	28.98	3.53E-02	-96.84	43.21	2.50E-02	69.38	167.68	6.79E-01	-47.21	57.07	4.08E-01	-27.91	56.61	6.22E-01
cg16977596	20.40	9.69	3.53E-02	7.26	14.76	6.23E-01	29.13	22.30	1.91E-01	25.64	20.83	2.18E-01	38.12	24.01	1.12E-01
cg04317651	18.61	8.84	3.53E-02	19.47	17.25	2.59E-01	14.88	17.16	3.86E-01	9.02	10.51	3.91E-01	60.07	24.39	1.38E-02
cg00983803	-12.97	6.16	3.53E-02	-23.70	10.44	2.32E-02	-5.19	13.84	7.08E-01	-2.41	12.69	8.50E-01	-14.36	13.23	2.78E-01
cg03951833	-5.82	2.76	3.53E-02	-10.06	2.85	4.23E-04	1.12	4.12	7.86E-01	-6.38	6.42	3.21E-01	-6.48	4.86	1.82E-01
cg02670929	10.47	4.97	3.53E-02	12.13	9.18	1.86E-01	3.08	11.64	7.91E-01	13.68	8.28	9.85E-02	8.64	12.34	4.84E-01
cg09608652	9.95	4.73	3.53E-02	14.09	6.35	2.66E-02	9.47	14.99	5.28E-01	9.24	12.98	4.77E-01	-0.07	10.21	9.95E-01
cg08780166	-10.63	5.05	3.53E-02	-20.87	13.06	1.10E-01	-4.45	22.11	8.41E-01	-9.30	5.89	1.14E-01	-6.89	20.17	7.32E-01
cg26927516	-13.84	6.58	3.53E-02	-24.82	16.93	1.43E-01	-2.99	31.13	9.23E-01	-12.29	7.76	1.13E-01	-13.18	22.54	5.59E-01
cg23219720	29.90	14.21	3.53E-02	38.06	19.97	5.67E-02	34.59	50.65	4.95E-01	44.59	45.74	3.30E-01	11.33	25.18	6.53E-01
cg09851981	-10.62	5.05	3.53E-02	-21.18	9.47	2.53E-02	-4.90	13.12	7.09E-01	-4.54	7.30	5.34E-01	-18.83	16.71	2.60E-01
cg08832851	-6.28	2.98	3.53E-02	-3.46	4.32	4.23E-01	-12.74	5.60	2.30E-02	-4.50	9.57	6.38E-01	-4.07	7.88	6.06E-01
cg25632672	-51.78	24.61	3.53E-02	-50.84	69.54	4.65E-01	-140.55	200.03	4.82E-01	-61.32	28.64	3.23E-02	16.17	70.56	8.19E-01
cg07918453	17.39	8.26	3.53E-02	7.27	17.99	6.86E-01	30.80	36.48	3.99E-01	21.45	10.43	3.98E-02	7.42	24.88	7.65E-01

cg14913676	14.35	6.82	3.53E-02	22.27	14.41	1.22E-01	7.04	54.85	8.98E-01	13.83	8.19	9.11E-02	-5.13	26.41	8.46E-01
cg04764907	-17.17	8.16	3.53E-02	-0.90	5.69	8.75E-01	-18.09	8.62	3.59E-02	-46.21	22.05	3.61E-02	-25.54	8.84	3.88E-03
cg06742159	-46.97	22.32	3.53E-02	-41.13	29.10	1.57E-01	-39.07	86.91	6.53E-01	-21.46	76.14	7.78E-01	-70.64	43.80	1.07E-01
cg07025678	-22.51	10.70	3.53E-02	-14.69	21.33	4.91E-01	1.14	52.65	9.83E-01	-27.39	13.68	4.53E-02	-22.13	34.61	5.22E-01
cg14747225	-32.53	15.46	3.54E-02	-41.97	18.18	2.10E-02	-49.00	96.71	6.12E-01	16.89	93.99	8.57E-01	-6.16	32.66	8.50E-01
cg02079645	-11.64	5.53	3.54E-02	-13.36	11.08	2.28E-01	-1.62	16.38	9.21E-01	-12.48	7.59	1.00E-01	-14.20	17.07	4.06E-01
cg02515233	22.22	10.56	3.54E-02	28.18	17.53	1.08E-01	3.87	34.64	9.11E-01	26.80	18.73	1.52E-01	13.75	22.18	5.35E-01
cg21826784	6.88	3.27	3.54E-02	5.62	4.37	1.99E-01	15.69	7.57	3.83E-02	6.54	10.35	5.28E-01	1.05	8.33	9.00E-01
cg12749636	22.78	10.83	3.54E-02	14.28	16.47	3.86E-01	49.85	39.01	2.01E-01	22.76	28.03	4.17E-01	27.43	18.51	1.39E-01
cg12300018	-10.56	5.02	3.54E-02	-17.13	7.85	2.90E-02	-10.49	12.38	3.97E-01	0.81	12.10	9.46E-01	-7.73	9.95	4.37E-01
cg18011999	24.08	11.44	3.54E-02	45.02	31.13	1.48E-01	-16.54	148.17	9.11E-01	24.25	12.91	6.03E-02	-13.10	42.27	7.57E-01
cg11582369	-9.68	4.60	3.54E-02	-23.36	15.53	1.33E-01	-2.90	12.83	8.21E-01	-9.95	5.46	6.82E-02	-2.51	17.04	8.83E-01
cg13002493	12.54	5.96	3.54E-02	28.59	15.55	6.60E-02	-6.09	63.56	9.24E-01	9.78	6.85	1.53E-01	11.42	20.33	5.74E-01
cg15399131	-7.57	3.60	3.54E-02	-15.49	8.17	5.79E-02	-23.73	19.67	2.28E-01	-6.69	4.67	1.52E-01	1.11	8.50	8.96E-01
cg18074726	-10.03	4.77	3.54E-02	-19.59	14.26	1.70E-01	-2.09	32.95	9.49E-01	-7.11	5.57	2.02E-01	-19.20	12.98	1.39E-01
cg22401883	17.37	8.26	3.54E-02	6.15	20.66	7.66E-01	-14.21	53.22	7.89E-01	22.49	9.65	1.98E-02	3.22	28.41	9.10E-01
cg15839752	-12.53	5.96	3.54E-02	-15.21	10.81	1.59E-01	-18.57	11.44	1.05E-01	-7.66	13.49	5.70E-01	-6.04	12.39	6.26E-01
cg03857198	5.78	2.75	3.54E-02	5.38	3.52	1.26E-01	9.28	7.34	2.06E-01	7.19	8.31	3.87E-01	2.94	7.32	6.88E-01
cg26877678	6.53	3.11	3.54E-02	7.73	4.19	6.49E-02	7.98	6.45	2.16E-01	3.84	10.58	7.17E-01	0.78	8.54	9.27E-01
cg10613381	11.10	5.28	3.54E-02	7.95	7.07	2.60E-01	-2.29	10.64	8.30E-01	23.70	13.54	8.01E-02	20.50	9.60	3.27E-02
cg14553881	5.87	2.79	3.54E-02	1.03	4.48	8.18E-01	6.75	5.48	2.18E-01	7.78	7.09	2.72E-01	12.66	6.26	4.29E-02
cg24428040	-10.54	5.01	3.54E-02	-3.52	7.79	6.51E-01	-22.50	11.80	5.67E-02	-6.92	11.45	5.46E-01	-17.23	10.80	1.11E-01
cg00953211	18.03	8.57	3.54E-02	32.15	16.54	5.19E-02	19.22	18.48	2.98E-01	12.97	14.02	3.55E-01	2.93	22.69	8.97E-01
cg12659114	11.42	5.43	3.54E-02	11.55	13.31	3.85E-01	11.30	21.46	5.98E-01	11.60	6.68	8.26E-02	10.21	16.40	5.34E-01
cg17356328	-32.33	15.37	3.54E-02	-27.08	39.90	4.97E-01	-170.99	202.96	4.00E-01	-33.85	17.51	5.31E-02	-16.54	56.06	7.68E-01
cg24430322	21.59	10.26	3.54E-02	30.24	13.42	2.43E-02	14.26	31.92	6.55E-01	15.21	41.96	7.17E-01	6.06	20.44	7.67E-01
cg15866367	7.78	3.70	3.54E-02	9.77	6.35	1.24E-01	16.77	9.18	6.79E-02	2.43	5.57	6.63E-01	11.71	15.39	4.47E-01
cg02121447	10.86	5.16	3.54E-02	15.79	8.22	5.46E-02	6.56	11.59	5.71E-01	14.24	10.26	1.65E-01	-1.83	13.17	8.90E-01
cg20871277	18.49	8.79	3.54E-02	36.41	17.60	3.86E-02	13.36	16.76	4.26E-01	0.63	15.60	9.68E-01	34.95	22.07	1.13E-01
cg00308792	23.59	11.21	3.54E-02	42.91	20.43	3.57E-02	43.62	23.05	5.85E-02	5.00	11.26	6.57E-01	27.84	28.85	3.35E-01
cg06526326	-12.63	6.00	3.54E-02	-5.51	12.97	6.71E-01	-18.12	33.88	5.93E-01	-15.79	7.28	3.00E-02	-1.82	22.07	9.34E-01
cg24079727	5.02	2.39	3.54E-02	5.58	3.23	8.45E-02	8.35	6.11	1.72E-01	6.74	5.71	2.38E-01	-3.72	6.70	5.79E-01
cg17357285	18.44	8.76	3.54E-02	19.14	11.75	1.03E-01	14.95	55.49	7.88E-01	32.91	30.21	2.76E-01	13.88	15.16	3.60E-01
cg25772039	-39.58	18.81	3.54E-02	-43.96	28.09	1.18E-01	51.59	56.88	3.64E-01	-55.96	36.75	1.28E-01	-59.94	43.99	1.73E-01
cg26778754	34.19	16.25	3.54E-02	24.01	20.42	2.40E-01	57.52	103.13	5.77E-01	93.40	55.33	9.14E-02	37.16	32.16	2.48E-01

cg27075786	18.45	8.77	3.54E-02	36.86	21.13	8.10E-02	-12.33	34.32	7.19E-01	13.19	8.42	1.17E-01	54.82	31.43	8.11E-02
cg10009737	18.23	8.67	3.54E-02	19.90	14.41	1.67E-01	4.68	20.58	8.20E-01	11.04	9.33	2.37E-01	57.86	23.03	1.20E-02
cg22792552	16.32	7.76	3.54E-02	18.64	20.99	3.74E-01	17.81	31.19	5.68E-01	13.32	9.05	1.41E-01	43.40	30.12	1.50E-01
cg09012708	24.49	11.64	3.54E-02	37.26	37.38	3.19E-01	-138.71	170.10	4.15E-01	23.34	12.75	6.70E-02	32.08	46.09	4.86E-01
cg19046959	25.45	12.10	3.54E-02	37.44	17.12	2.87E-02	28.24	33.40	3.98E-01	14.78	32.69	6.51E-01	4.36	25.12	8.62E-01
cg17788013	-8.46	4.02	3.54E-02	-11.43	9.60	2.34E-01	-10.97	14.41	4.46E-01	-5.11	5.08	3.15E-01	-19.97	11.61	8.53E-02
cg13919369	-17.89	8.50	3.54E-02	-29.96	25.72	2.44E-01	-31.33	31.02	3.13E-01	-16.47	9.85	9.45E-02	0.35	32.23	9.91E-01
cg04208434	9.96	4.73	3.54E-02	13.75	6.94	4.75E-02	8.24	10.74	4.43E-01	15.09	14.18	2.87E-01	1.18	9.91	9.05E-01
cg09797463	8.59	4.08	3.54E-02	7.05	5.36	1.88E-01	15.62	9.81	1.11E-01	11.08	12.20	3.64E-01	4.05	11.15	7.17E-01
cg06573787	5.59	2.66	3.55E-02	8.72	4.50	5.29E-02	4.48	5.40	4.07E-01	7.72	6.13	2.08E-01	0.08	5.64	9.88E-01
cg03163819	8.52	4.05	3.55E-02	11.28	7.10	1.12E-01	13.47	10.44	1.97E-01	5.28	6.14	3.90E-01	5.92	13.65	6.64E-01
cg22572454	19.49	9.27	3.55E-02	16.34	22.11	4.60E-01	16.70	22.79	4.64E-01	18.30	12.47	1.42E-01	35.08	28.31	2.15E-01
cg01345517	36.35	17.29	3.55E-02	49.57	23.12	3.20E-02	-7.51	58.88	8.99E-01	65.23	79.50	4.12E-01	20.19	31.17	5.17E-01
cg21149811	-17.69	8.41	3.55E-02	-29.38	12.42	1.80E-02	-15.19	19.45	4.35E-01	-3.01	21.25	8.87E-01	-4.59	18.89	8.08E-01
cg01558877	-8.43	4.01	3.55E-02	-7.82	7.39	2.90E-01	-9.65	12.19	4.28E-01	-7.68	5.96	1.97E-01	-11.11	10.55	2.92E-01
cg07338715	11.05	5.25	3.55E-02	17.76	7.14	1.29E-02	24.14	15.01	1.08E-01	5.36	9.34	5.66E-01	-2.07	10.78	8.48E-01
cg09356141	14.95	7.11	3.55E-02	12.52	13.74	3.62E-01	-14.68	26.93	5.86E-01	20.00	9.57	3.67E-02	14.29	21.29	5.02E-01
cg18279467	42.47	20.20	3.55E-02	7.15	41.44	8.63E-01	1.90	136.45	9.89E-01	68.19	21.04	1.19E-03	-11.10	51.29	8.29E-01
cg20736321	-13.36	6.35	3.55E-02	-14.82	9.75	1.29E-01	8.76	16.94	6.05E-01	-24.62	14.86	9.75E-02	-15.08	12.66	2.34E-01
cg15852446	7.38	3.51	3.55E-02	8.60	5.27	1.03E-01	9.14	7.89	2.47E-01	8.14	8.41	3.33E-01	1.87	8.15	8.19E-01
cg23449764	-13.80	6.56	3.55E-02	-15.94	12.24	1.93E-01	-2.31	14.71	8.75E-01	-18.57	11.07	9.35E-02	-13.79	16.29	3.97E-01
cg11129609	11.77	5.60	3.55E-02	13.99	7.97	7.91E-02	18.74	10.56	7.59E-02	0.19	17.79	9.92E-01	-3.31	15.73	8.33E-01
cg23502565	28.59	13.60	3.55E-02	27.27	16.48	9.79E-02	74.78	51.55	1.47E-01	63.69	45.91	1.65E-01	-4.69	33.78	8.90E-01
cg05905184	15.44	7.34	3.55E-02	13.13	7.95	9.85E-02	28.12	30.97	3.64E-01	4.66	5.92	4.31E-01	38.72	13.35	3.72E-03
cg02264082	21.98	10.45	3.55E-02	27.61	22.95	2.29E-01	28.05	32.46	3.88E-01	12.87	14.56	3.77E-01	38.62	25.06	1.23E-01
cg27341860	-16.60	7.89	3.55E-02	-30.69	16.07	5.62E-02	0.49	17.33	9.77E-01	-12.41	12.06	3.03E-01	-32.36	22.50	1.50E-01
cg20583432	8.38	3.98	3.55E-02	2.73	5.50	6.20E-01	11.37	7.51	1.30E-01	20.24	8.54	1.78E-02	3.15	10.41	7.62E-01
cg10504150	17.66	8.40	3.55E-02	15.11	17.36	3.84E-01	9.65	17.66	5.85E-01	16.04	13.20	2.24E-01	40.32	22.84	7.75E-02
cg04239994	15.72	7.47	3.55E-02	21.84	18.46	2.37E-01	12.05	29.88	6.87E-01	13.90	9.54	1.45E-01	17.86	18.75	3.41E-01
cg12832492	-28.78	13.69	3.55E-02	-40.79	43.64	3.50E-01	-128.29	172.30	4.57E-01	-26.14	15.14	8.43E-02	-33.31	49.01	4.97E-01
cg04592144	-9.14	4.35	3.55E-02	-5.49	8.22	5.04E-01	-12.16	11.46	2.89E-01	-8.88	6.77	1.90E-01	-13.36	10.72	2.13E-01
cg16545105	9.95	4.73	3.55E-02	14.49	6.81	3.35E-02	0.05	12.85	9.97E-01	9.39	10.52	3.72E-01	5.87	11.17	5.99E-01
cg05444816	-21.75	10.35	3.55E-02	-21.14	13.74	1.24E-01	11.78	27.14	6.64E-01	-1.78	30.28	9.53E-01	-41.18	15.18	6.66E-03
cg02755455	11.49	5.47	3.55E-02	11.99	12.11	3.22E-01	11.54	23.39	6.22E-01	14.18	7.15	4.73E-02	0.80	13.81	9.54E-01
cg25148705	-7.95	3.78	3.55E-02	-13.78	6.31	2.89E-02	-9.75	8.33	2.42E-01	-1.90	6.51	7.70E-01	-3.59	12.18	7.68E-01

cg16372648	-11.15	5.30	3.55E-02	-5.88	11.56	6.11E-01	-3.64	24.21	8.81E-01	-11.19	6.88	1.04E-01	-20.81	13.74	1.30E-01
cg08345575	18.58	8.84	3.55E-02	27.04	18.10	1.35E-01	22.18	43.17	6.07E-01	19.00	11.38	9.48E-02	-2.25	25.89	9.31E-01
cg09607363	39.15	18.62	3.55E-02	51.64	22.44	2.14E-02	81.05	36.52	2.65E-02	4.03	13.11	7.59E-01	53.41	32.78	1.03E-01
cg25236277	-10.15	4.83	3.55E-02	-8.30	10.93	4.48E-01	-24.59	21.69	2.57E-01	-9.40	5.87	1.09E-01	-12.16	17.29	4.82E-01
cg13465946	23.38	11.12	3.55E-02	12.38	15.44	4.22E-01	38.90	70.80	5.83E-01	95.35	43.69	2.91E-02	23.01	14.47	1.12E-01
cg18382389	16.33	7.77	3.55E-02	31.78	20.02	1.12E-01	23.35	41.09	5.70E-01	13.03	8.91	1.44E-01	14.92	33.10	6.52E-01
cg11876705	-7.74	3.68	3.55E-02	-8.63	7.93	2.77E-01	-2.43	14.16	8.64E-01	-6.96	4.84	1.51E-01	-12.17	9.87	2.17E-01
cg13458596	19.36	9.21	3.55E-02	-2.56	27.42	9.25E-01	28.74	122.05	8.14E-01	19.94	10.17	5.00E-02	50.80	37.02	1.70E-01
cg01813393	18.63	8.86	3.55E-02	14.09	17.36	4.17E-01	-0.28	38.42	9.94E-01	21.80	12.01	6.95E-02	21.91	23.56	3.52E-01
cg26409978	-45.16	21.48	3.55E-02	-50.02	28.27	7.69E-02	7.31	81.83	9.29E-01	-22.24	56.16	6.92E-01	-65.28	47.20	1.67E-01
cg21721681	8.83	4.20	3.55E-02	7.68	5.30	1.47E-01	25.79	11.01	1.92E-02	0.18	10.36	9.86E-01	6.37	10.26	5.35E-01
cg02232206	18.00	8.56	3.56E-02	41.12	20.16	4.14E-02	25.03	29.44	3.95E-01	15.96	9.31	8.64E-02	-21.74	29.99	4.68E-01
cg07261015	16.87	8.03	3.56E-02	31.22	15.91	4.98E-02	26.63	24.40	2.75E-01	9.25	10.87	3.95E-01	10.93	26.55	6.81E-01
cg14028856	7.22	3.43	3.56E-02	8.71	4.43	4.92E-02	16.01	9.56	9.40E-02	5.20	10.89	6.33E-01	-3.54	8.32	6.71E-01
cg25679766	-6.80	3.23	3.56E-02	-5.76	4.67	2.17E-01	-9.39	8.09	2.46E-01	-3.89	9.80	6.92E-01	-8.39	6.45	1.93E-01
cg27400216	-8.16	3.88	3.56E-02	-19.01	9.14	3.76E-02	1.32	16.48	9.36E-01	-6.72	4.83	1.64E-01	-3.94	11.18	7.25E-01
cg17397955	31.23	14.86	3.56E-02	26.29	35.82	4.63E-01	-49.93	135.64	7.13E-01	30.28	17.00	7.48E-02	80.53	65.31	2.18E-01
cg06531870	-9.05	4.31	3.56E-02	-5.74	7.38	4.37E-01	-5.79	9.03	5.21E-01	-14.18	8.68	1.02E-01	-12.32	9.98	2.17E-01
cg15459537	-9.24	4.40	3.56E-02	-7.05	8.09	3.84E-01	-21.04	15.41	1.72E-01	-6.65	5.96	2.65E-01	-23.07	15.61	1.39E-01
cg14161953	-44.04	20.95	3.56E-02	-37.86	24.59	1.24E-01	-150.07	98.12	1.26E-01	8.74	69.99	9.01E-01	-75.70	56.32	1.79E-01
cg06515159	37.21	17.71	3.56E-02	35.75	18.54	5.37E-02	-18.89	35.06	5.90E-01	94.87	29.79	1.45E-03	31.01	18.83	9.96E-02
cg07432969	29.64	14.10	3.56E-02	28.92	16.92	8.75E-02	50.60	23.55	3.17E-02	3.28	10.06	7.44E-01	64.09	27.79	2.11E-02
cg24838764	-12.53	5.96	3.56E-02	-29.74	11.47	9.49E-03	-22.65	19.77	2.52E-01	-6.06	4.54	1.82E-01	-7.94	13.27	5.50E-01
cg24650748	-19.63	9.34	3.56E-02	-4.69	23.21	8.40E-01	-0.52	34.01	9.88E-01	-22.95	11.40	4.41E-02	-37.51	30.92	2.25E-01
cg26364205	15.43	7.34	3.56E-02	29.07	12.38	1.88E-02	0.74	17.61	9.66E-01	24.46	19.42	2.08E-01	4.75	12.77	7.10E-01
cg16068833	-7.10	3.38	3.56E-02	-5.02	4.71	2.86E-01	-3.71	8.31	6.56E-01	-14.08	9.57	1.41E-01	-11.04	7.67	1.50E-01
cg16599143	-6.32	3.01	3.56E-02	-7.14	5.83	2.21E-01	-10.31	7.31	1.58E-01	-5.53	4.75	2.45E-01	-2.81	7.40	7.04E-01
cg19033943	-52.08	24.78	3.56E-02	-50.22	30.11	9.53E-02	45.09	150.48	7.64E-01	-115.43	70.16	9.99E-02	-28.58	59.98	6.34E-01
cg13152070	-17.89	8.51	3.56E-02	-3.82	17.52	8.28E-01	-19.64	26.39	4.57E-01	-19.57	11.52	8.94E-02	-37.49	25.27	1.38E-01
cg21880476	17.43	8.30	3.56E-02	19.57	10.47	6.15E-02	20.13	34.08	5.55E-01	-19.71	35.62	5.80E-01	19.42	16.33	2.34E-01
cg09922452	5.06	2.41	3.56E-02	4.73	3.30	1.51E-01	2.20	5.42	6.85E-01	0.49	7.69	9.49E-01	12.03	5.83	3.90E-02
cg22053452	43.39	20.65	3.56E-02	54.28	27.11	4.53E-02	-38.52	97.30	6.92E-01	86.19	82.13	2.94E-01	26.28	36.97	4.77E-01
cg21533997	16.60	7.90	3.56E-02	19.78	13.75	1.50E-01	31.74	20.30	1.18E-01	10.09	14.42	4.84E-01	10.25	16.89	5.44E-01
cg25127214	-12.08	5.75	3.56E-02	-21.38	17.46	2.21E-01	11.25	20.70	5.87E-01	-12.45	6.66	6.16E-02	-19.58	21.85	3.70E-01
cg18014042	-35.45	16.87	3.56E-02	-40.37	22.64	7.46E-02	-19.13	80.61	8.12E-01	-43.79	39.25	2.65E-01	-19.01	36.28	6.00E-01

cg04131431	20.19	9.61	3.56E-02	9.75	25.00	6.96E-01	38.45	61.53	5.32E-01	26.21	11.74	2.56E-02	1.62	24.15	9.47E-01
cg04959480	10.83	5.15	3.56E-02	10.62	7.92	1.80E-01	19.04	12.46	1.27E-01	9.15	11.70	4.35E-01	6.16	11.20	5.82E-01
cg20369204	11.41	5.43	3.56E-02	16.99	7.28	1.96E-02	13.84	19.08	4.68E-01	-3.35	12.60	7.91E-01	8.23	12.91	5.24E-01
cg09994114	16.24	7.73	3.56E-02	6.60	15.71	6.74E-01	43.27	20.49	3.47E-02	8.87	9.18	3.33E-01	32.48	19.85	1.02E-01
cg19639367	20.64	9.82	3.56E-02	33.65	20.15	9.49E-02	25.93	20.26	2.01E-01	4.25	14.89	7.75E-01	50.78	32.28	1.16E-01
cg15732657	11.54	5.49	3.56E-02	14.17	9.46	1.34E-01	1.82	15.46	9.06E-01	6.48	8.47	4.44E-01	32.85	16.14	4.19E-02
cg23983671	86.50	41.17	3.56E-02	42.16	34.10	2.16E-01	200.41	78.65	1.08E-02	150.77	48.06	1.71E-03	0.00	52.20	1.00E+00
cg18049164	-6.96	3.31	3.56E-02	-9.81	6.59	1.36E-01	-1.98	9.57	8.36E-01	-6.08	4.81	2.06E-01	-8.88	8.46	2.94E-01
cg04582365	-7.60	3.62	3.56E-02	-6.46	7.42	3.84E-01	3.03	12.27	8.05E-01	-9.67	4.74	4.13E-02	-7.50	11.90	5.28E-01
cg05189127	7.00	3.33	3.56E-02	2.51	3.80	5.10E-01	4.58	5.75	4.26E-01	19.38	7.55	1.02E-02	9.03	6.38	1.57E-01
cg10016610	-10.27	4.89	3.56E-02	-6.82	43.42	8.75E-01	0.64	145.67	9.96E-01	-4.77	6.77	4.81E-01	-16.54	7.16	2.10E-02
cg10930502	20.98	9.98	3.56E-02	35.05	14.95	1.91E-02	20.73	16.25	2.02E-01	-9.79	19.09	6.08E-01	34.16	21.70	1.15E-01
cg08848774	17.57	8.36	3.56E-02	39.97	32.93	2.25E-01	-3.39	178.22	9.85E-01	15.22	8.91	8.76E-02	30.62	36.74	4.05E-01
cg15961219	-7.19	3.42	3.56E-02	-7.09	6.15	2.49E-01	0.50	11.73	9.66E-01	-10.55	5.02	3.55E-02	-0.93	9.17	9.19E-01
ch.8.120063967R	-37.86	18.02	3.56E-02	-36.73	22.12	9.67E-02	4.78	78.88	9.52E-01	-69.97	81.29	3.89E-01	-43.80	37.19	2.39E-01
cg13150171	14.79	7.04	3.56E-02	27.12	12.53	3.04E-02	10.87	35.05	7.56E-01	7.09	10.44	4.97E-01	13.54	16.15	4.02E-01
cg26474717	-37.06	17.64	3.56E-02	-21.98	25.72	3.93E-01	-26.18	48.57	5.90E-01	-69.19	38.02	6.87E-02	-45.84	41.26	2.67E-01
cg21098005	15.86	7.55	3.56E-02	8.21	13.31	5.38E-01	30.11	16.35	6.55E-02	23.13	11.51	4.45E-02	-12.17	21.31	5.68E-01
cg03438773	8.49	4.04	3.56E-02	9.58	5.56	8.49E-02	15.71	10.78	1.45E-01	9.98	9.88	3.12E-01	-2.67	9.94	7.88E-01
cg26850503	44.44	21.15	3.56E-02	56.59	26.32	3.16E-02	-0.76	115.97	9.95E-01	121.10	74.23	1.03E-01	-7.99	43.21	8.53E-01
cg23553119	-33.49	15.94	3.56E-02	-5.98	56.97	9.16E-01	-183.68	219.57	4.03E-01	-35.91	17.72	4.27E-02	-27.92	48.65	5.66E-01
cg16341266	-7.70	3.66	3.57E-02	-16.94	8.69	5.12E-02	2.73	13.65	8.42E-01	-4.87	4.58	2.88E-01	-16.03	11.04	1.47E-01
cg25430849	30.92	14.72	3.57E-02	-7.87	46.24	8.65E-01	-17.09	158.51	9.14E-01	34.81	16.39	3.37E-02	45.24	50.83	3.73E-01
cg15053937	21.16	10.07	3.57E-02	32.72	21.71	1.32E-01	23.49	36.75	5.23E-01	12.43	13.10	3.43E-01	42.25	29.27	1.49E-01
cg17751569	15.76	7.50	3.57E-02	30.38	14.30	3.36E-02	-4.83	16.20	7.66E-01	20.01	10.67	6.07E-02	1.62	23.16	9.44E-01
cg00217795	5.35	2.55	3.57E-02	5.24	3.69	1.55E-01	7.63	6.67	2.53E-01	1.22	6.65	8.54E-01	6.77	5.32	2.03E-01
cg20285128	17.50	8.33	3.57E-02	47.37	24.95	5.76E-02	-11.81	41.97	7.78E-01	11.74	10.23	2.51E-01	26.25	19.27	1.73E-01
cg09717261	36.15	17.21	3.57E-02	27.33	20.54	1.83E-01	103.83	88.92	2.43E-01	109.06	75.08	1.46E-01	35.32	37.75	3.50E-01
cg09179347	20.31	9.67	3.57E-02	17.04	20.09	3.96E-01	10.79	20.86	6.05E-01	22.46	14.79	1.29E-01	35.29	27.22	1.95E-01
cg24310786	-15.45	7.35	3.57E-02	-28.67	10.68	7.28E-03	4.80	15.50	7.57E-01	-8.82	14.00	5.29E-01	-19.04	15.71	2.26E-01
cg26671183	26.26	12.50	3.57E-02	36.13	23.29	1.21E-01	32.85	80.86	6.85E-01	17.90	16.45	2.76E-01	42.86	37.67	2.55E-01
cg16205375	-11.46	5.46	3.57E-02	-17.38	10.24	8.98E-02	-9.46	8.55	2.69E-01	-11.15	10.70	2.97E-01	4.54	24.70	8.54E-01
cg06967016	15.49	7.37	3.57E-02	14.57	18.65	4.35E-01	-10.93	39.73	7.83E-01	13.89	8.73	1.11E-01	38.44	23.86	1.07E-01
cg07544189	-8.06	3.84	3.57E-02	-11.86	7.39	1.08E-01	-2.41	9.31	7.96E-01	-9.41	6.08	1.22E-01	-4.33	9.52	6.49E-01
cg06054026	19.29	9.19	3.57E-02	10.25	9.55	2.83E-01	7.76	22.73	7.33E-01	61.53	22.60	6.46E-03	17.90	12.18	1.42E-01

cg24532083	-8.98	4.28	3.57E-02	-7.28	6.05	2.29E-01	-23.28	11.19	3.75E-02	0.92	11.30	9.35E-01	-9.84	9.30	2.90E-01
cg11885433	15.69	7.47	3.57E-02	10.12	11.10	3.62E-01	23.87	14.88	1.09E-01	-2.15	24.98	9.31E-01	25.72	16.48	1.19E-01
cg16474722	17.12	8.15	3.57E-02	23.69	13.53	8.00E-02	17.30	16.23	2.86E-01	-5.32	16.69	7.50E-01	37.17	21.33	8.14E-02
cg05985576	12.69	6.04	3.57E-02	12.19	11.93	3.07E-01	7.26	14.95	6.27E-01	12.53	8.95	1.62E-01	21.44	17.13	2.11E-01
cg15720500	-6.70	3.19	3.57E-02	-9.92	6.02	9.95E-02	-6.54	7.41	3.77E-01	-4.17	5.10	4.13E-01	-7.56	8.49	3.73E-01
cg12142062	-16.95	8.07	3.57E-02	-27.60	14.54	5.76E-02	-3.12	14.73	8.32E-01	-10.57	17.43	5.44E-01	-29.48	19.13	1.23E-01
cg10613252	21.80	10.38	3.57E-02	8.88	29.36	7.62E-01	-10.26	150.28	9.46E-01	29.19	11.62	1.20E-02	-35.59	38.71	3.58E-01
cg11793562	-25.45	12.12	3.57E-02	-32.94	14.98	2.79E-02	-6.60	42.68	8.77E-01	13.50	79.50	8.65E-01	-15.25	24.63	5.36E-01
cg11785832	-54.91	26.14	3.57E-02	-45.12	33.55	1.79E-01	-47.86	87.52	5.84E-01	-19.14	73.37	7.94E-01	-117.78	62.19	5.82E-02
cg07159230	10.41	4.96	3.57E-02	8.53	7.05	2.27E-01	15.59	12.16	2.00E-01	15.96	16.49	3.33E-01	8.67	9.91	3.82E-01
cg17410431	-19.77	9.41	3.57E-02	-24.13	16.18	1.36E-01	-71.29	37.88	5.98E-02	-19.58	10.63	6.56E-02	10.51	24.21	6.64E-01
cg13177195	13.05	6.21	3.57E-02	10.98	12.86	3.93E-01	-3.51	19.01	8.54E-01	16.08	8.19	4.97E-02	19.08	21.39	3.72E-01
cg01662455	11.44	5.45	3.57E-02	7.96	7.44	2.85E-01	18.82	13.15	1.52E-01	25.63	16.27	1.15E-01	5.94	12.84	6.44E-01
cg13414212	16.99	8.09	3.57E-02	24.64	15.40	1.10E-01	18.16	22.00	4.09E-01	16.31	17.24	3.44E-01	11.23	13.31	3.99E-01
cg09944325	28.33	13.49	3.57E-02	82.06	33.51	1.43E-02	13.58	155.35	9.30E-01	20.02	13.19	1.29E-01	8.39	42.99	8.45E-01
cg16054278	-20.38	9.70	3.57E-02	-7.56	24.32	7.56E-01	-19.59	47.28	6.79E-01	-24.41	11.37	3.18E-02	-8.17	36.53	8.23E-01
cg23641305	-7.87	3.75	3.57E-02	-10.34	6.54	1.14E-01	4.33	10.50	6.80E-01	-10.64	6.34	9.34E-02	-6.71	8.49	4.29E-01
cg16394290	7.61	3.62	3.57E-02	10.74	4.81	2.57E-02	11.63	9.33	2.13E-01	-2.20	10.84	8.39E-01	0.07	8.80	9.93E-01
cg14431547	11.80	5.62	3.57E-02	16.42	7.77	3.47E-02	13.72	14.06	3.29E-01	-4.21	27.60	8.79E-01	4.35	10.70	6.84E-01
cg26803289	-11.90	5.67	3.57E-02	-14.41	11.85	2.24E-01	-8.84	16.21	5.85E-01	-9.82	7.86	2.12E-01	-18.69	15.73	2.35E-01
cg00379648	13.77	6.56	3.57E-02	20.44	13.63	1.34E-01	-9.32	26.17	7.22E-01	15.72	8.90	7.75E-02	6.70	16.27	6.80E-01
cg26668272	16.94	8.07	3.57E-02	15.72	15.81	3.20E-01	29.88	13.46	2.64E-02	-5.67	18.19	7.55E-01	17.56	18.80	3.50E-01
cg16136338	22.22	10.58	3.57E-02	-11.16	28.29	6.93E-01	40.21	116.41	7.30E-01	28.09	12.14	2.07E-02	22.95	34.91	5.11E-01
cg11155016	15.57	7.41	3.57E-02	16.46	15.96	3.02E-01	-21.55	35.73	5.46E-01	15.17	9.13	9.65E-02	36.02	25.97	1.65E-01
cg22933058	-4.95	2.36	3.57E-02	-4.53	3.19	1.55E-01	-10.11	5.04	4.50E-02	-0.28	8.74	9.75E-01	-1.48	5.88	8.01E-01
cg06670911	20.94	9.97	3.57E-02	41.01	23.35	7.90E-02	11.51	81.40	8.88E-01	14.25	12.39	2.50E-01	26.15	25.31	3.02E-01
cg14948434	-13.87	6.61	3.57E-02	-13.55	10.65	2.03E-01	4.45	17.55	8.00E-01	-22.07	13.32	9.76E-02	-16.95	13.84	2.21E-01
cg04233201	-10.40	4.95	3.57E-02	-10.61	9.44	2.61E-01	15.15	17.52	3.87E-01	-15.46	7.41	3.71E-02	-9.06	11.13	4.16E-01
cg20967585	6.11	2.91	3.57E-02	7.28	4.21	8.39E-02	-1.24	7.06	8.60E-01	6.52	12.49	6.01E-01	8.35	5.32	1.17E-01
cg21960303	-15.51	7.38	3.58E-02	-17.38	12.19	1.54E-01	-2.67	15.48	8.63E-01	-7.47	15.84	6.37E-01	-36.65	17.02	3.13E-02
cg00833352	27.81	13.25	3.58E-02	34.96	19.05	6.65E-02	40.03	49.88	4.22E-01	68.43	52.34	1.91E-01	9.71	21.43	6.50E-01
cg25550425	-12.88	6.14	3.58E-02	-25.53	10.62	1.62E-02	-3.35	13.54	8.04E-01	-10.64	13.51	4.31E-01	-5.81	12.16	6.33E-01
cg20451636	21.22	10.10	3.58E-02	46.86	21.00	2.56E-02	11.07	21.11	6.00E-01	10.13	15.70	5.19E-01	29.04	28.58	3.09E-01
cg04744624	11.77	5.60	3.58E-02	13.02	8.79	1.39E-01	13.22	14.20	3.52E-01	21.53	12.25	7.90E-02	-0.38	11.72	9.74E-01
cg10269439	-10.49	5.00	3.58E-02	-16.72	11.03	1.30E-01	4.87	22.95	8.32E-01	-11.23	6.10	6.57E-02	3.03	17.96	8.66E-01

cg26585899	5.74	2.73	3.58E-02	3.08	4.44	4.88E-01	9.93	6.80	1.44E-01	7.26	4.54	1.10E-01	3.51	8.79	6.90E-01
cg12489736	-34.41	16.39	3.58E-02	-39.45	20.64	5.59E-02	-34.41	49.70	4.89E-01	-36.91	71.73	6.07E-01	-18.52	35.90	6.06E-01
cg10078540	-6.44	3.07	3.58E-02	-7.40	4.26	8.25E-02	-1.96	6.51	7.63E-01	-1.89	13.59	8.90E-01	-9.90	6.70	1.39E-01
cg26216323	7.12	3.39	3.58E-02	8.37	5.36	1.18E-01	7.72	7.35	2.93E-01	10.88	6.08	7.37E-02	-9.91	10.52	3.46E-01
cg23335304	21.17	10.08	3.58E-02	24.96	25.20	3.22E-01	23.09	30.50	4.49E-01	15.07	12.41	2.25E-01	66.60	37.95	7.92E-02
cg25504443	26.74	12.74	3.58E-02	31.95	17.20	6.33E-02	-7.78	52.21	8.81E-01	38.16	44.15	3.87E-01	21.08	22.92	3.58E-01
cg15686615	9.07	4.32	3.58E-02	6.99	5.80	2.29E-01	22.02	11.55	5.65E-02	6.51	10.31	5.28E-01	7.47	11.96	5.32E-01
cg13943355	-17.33	8.26	3.58E-02	-14.80	11.59	2.02E-01	2.59	23.42	9.12E-01	-28.59	14.75	5.25E-02	-21.59	35.16	5.39E-01
cg02388788	-11.47	5.46	3.58E-02	-8.26	12.06	4.93E-01	-17.64	21.52	4.12E-01	-6.76	7.19	3.48E-01	-30.74	13.91	2.71E-02
cg23524048	-15.84	7.55	3.58E-02	-33.34	15.34	2.97E-02	-0.74	15.66	9.62E-01	-12.89	11.70	2.71E-01	-20.43	22.79	3.70E-01
cg13978542	18.13	8.64	3.58E-02	25.62	15.76	1.04E-01	14.90	15.00	3.20E-01	14.48	18.09	4.23E-01	15.63	23.05	4.98E-01
cg26001902	9.50	4.52	3.58E-02	13.92	13.09	2.88E-01	9.67	24.67	6.95E-01	11.62	5.77	4.40E-02	1.58	9.38	8.66E-01
cg02261408	20.35	9.70	3.58E-02	20.04	19.25	2.98E-01	16.93	18.17	3.51E-01	14.05	16.99	4.08E-01	43.20	26.30	1.00E-01
cg10176667	51.89	24.72	3.58E-02	74.40	33.85	2.79E-02	24.27	76.70	7.52E-01	-0.89	52.76	9.87E-01	69.03	65.33	2.91E-01
cg22145318	-11.06	5.27	3.58E-02	-1.22	8.02	8.79E-01	-2.21	13.35	8.69E-01	-20.06	5.65	3.84E-04	-11.99	11.04	2.78E-01
cg20267897	-14.28	6.80	3.58E-02	-23.30	20.49	2.55E-01	33.45	39.56	3.98E-01	-17.63	7.75	2.29E-02	10.20	22.90	6.56E-01
cg24085426	17.80	8.48	3.58E-02	21.51	17.92	2.30E-01	-22.58	34.14	5.08E-01	25.09	11.06	2.33E-02	-3.02	23.90	8.99E-01
cg02620769	35.23	16.78	3.58E-02	49.60	18.36	6.91E-03	48.84	74.86	5.14E-01	52.61	36.32	1.47E-01	-18.88	33.41	5.72E-01
cg01599714	7.01	3.34	3.58E-02	7.56	4.41	8.67E-02	12.46	6.36	4.99E-02	-3.17	17.25	8.54E-01	-5.79	9.98	5.62E-01
cg08219213	10.99	5.23	3.58E-02	21.03	13.69	1.24E-01	37.69	41.98	3.69E-01	8.36	6.01	1.64E-01	12.42	18.64	5.05E-01
cg19288027	-34.80	16.58	3.58E-02	-42.26	21.52	4.96E-02	-0.68	45.98	9.88E-01	9.33	70.24	8.94E-01	-46.00	35.28	1.92E-01
cg02349638	32.85	15.65	3.58E-02	3.68	45.90	9.36E-01	55.00	162.40	7.35E-01	38.17	17.40	2.82E-02	15.64	61.47	7.99E-01
cg09430391	25.00	11.91	3.58E-02	17.03	10.66	1.10E-01	77.35	44.17	7.99E-02	82.87	44.10	6.03E-02	15.00	18.24	4.11E-01
cg09329936	-11.11	5.29	3.58E-02	-25.69	10.09	1.09E-02	-4.92	13.67	7.19E-01	-5.18	8.59	5.46E-01	-6.85	11.99	5.68E-01
cg17398227	8.26	3.94	3.58E-02	5.41	5.33	3.11E-01	12.86	8.45	1.28E-01	13.11	13.39	3.27E-01	9.16	10.10	3.65E-01
cg21308760	-15.30	7.29	3.58E-02	-28.72	21.87	1.89E-01	-11.59	48.52	8.11E-01	-15.79	8.15	5.25E-02	12.07	28.37	6.70E-01
cg19595244	5.84	2.78	3.58E-02	6.28	3.63	8.34E-02	8.26	7.37	2.62E-01	7.43	8.08	3.58E-01	0.59	7.16	9.34E-01
cg01263222	23.32	11.11	3.58E-02	8.91	13.99	5.24E-01	55.38	32.09	8.44E-02	29.42	47.95	5.40E-01	47.13	24.63	5.57E-02
cg20085268	7.30	3.48	3.58E-02	9.68	5.27	6.63E-02	0.47	10.75	9.65E-01	4.73	6.76	4.84E-01	9.20	7.91	2.45E-01
cg03778788	27.18	12.95	3.58E-02	47.29	35.55	1.83E-01	84.70	103.68	4.14E-01	20.53	14.74	1.64E-01	46.91	45.96	3.07E-01
cg27565277	-10.37	4.94	3.58E-02	-17.07	8.11	3.52E-02	-3.24	11.89	7.85E-01	-12.29	10.35	2.35E-01	-2.93	10.35	7.77E-01
cg10613546	-10.31	4.91	3.59E-02	-13.06	7.01	6.24E-02	2.41	11.43	8.33E-01	-4.26	16.63	7.98E-01	-16.73	10.09	9.71E-02
cg27547283	39.68	18.91	3.59E-02	-28.47	60.60	6.38E-01	77.48	262.60	7.68E-01	52.07	21.02	1.33E-02	-0.86	63.65	9.89E-01
cg09118048	19.11	9.11	3.59E-02	26.60	16.73	1.12E-01	14.23	19.54	4.67E-01	10.47	14.79	4.79E-01	38.79	27.77	1.62E-01
cg23402769	24.97	11.90	3.59E-02	15.55	15.73	3.23E-01	26.89	31.50	3.93E-01	51.47	59.67	3.88E-01	41.53	24.02	8.38E-02

cg02053566	38.37	18.28	3.59E-02	35.38	22.19	1.11E-01	-113.68	111.11	3.06E-01	101.33	85.96	2.38E-01	51.62	36.67	1.59E-01
cg09284106	18.22	8.68	3.59E-02	45.81	21.97	3.70E-02	19.85	24.95	4.26E-01	10.62	10.92	3.31E-01	21.55	28.95	4.57E-01
cg03698009	25.95	12.37	3.59E-02	18.53	21.91	3.98E-01	9.02	45.46	8.43E-01	71.90	30.60	1.88E-02	17.19	18.57	3.55E-01
cg08203210	8.23	3.92	3.59E-02	-2.88	8.22	7.26E-01	10.27	13.74	4.55E-01	11.81	4.95	1.71E-02	10.13	15.60	5.16E-01
cg25427871	7.72	3.68	3.59E-02	7.59	5.76	1.88E-01	10.78	6.76	1.11E-01	15.97	10.35	1.23E-01	-3.39	8.90	7.03E-01
cg21218059	-29.68	14.15	3.59E-02	-61.22	43.48	1.59E-01	137.67	176.04	4.34E-01	-30.68	15.78	5.19E-02	6.62	48.73	8.92E-01
cg15769726	29.84	14.22	3.59E-02	51.30	19.62	8.93E-03	-5.15	25.78	8.42E-01	13.34	34.92	7.02E-01	45.11	30.62	1.41E-01
cg08644980	17.83	8.50	3.59E-02	15.90	14.94	2.87E-01	30.54	16.79	6.89E-02	-1.42	17.32	9.35E-01	28.92	20.03	1.49E-01
cg21667367	8.65	4.12	3.59E-02	5.01	5.80	3.87E-01	18.39	8.53	3.11E-02	15.71	9.73	1.07E-01	-0.87	9.36	9.26E-01
cg03410223	-22.50	10.73	3.59E-02	-28.50	11.62	1.42E-02	-24.48	56.08	6.63E-01	53.00	54.50	3.31E-01	8.58	39.79	8.29E-01
cg07222900	20.67	9.85	3.59E-02	40.45	18.30	2.71E-02	-22.71	36.29	5.31E-01	20.17	14.05	1.51E-01	4.88	25.85	8.50E-01
cg17317859	-8.34	3.97	3.59E-02	-2.05	9.13	8.23E-01	-7.25	13.35	5.87E-01	-9.83	5.05	5.15E-02	-11.94	12.48	3.39E-01
cg25876319	22.65	10.79	3.59E-02	18.51	19.30	3.38E-01	46.14	21.33	3.05E-02	1.60	16.91	9.25E-01	42.79	27.02	1.13E-01
cg00366237	54.39	25.93	3.59E-02	38.96	29.31	1.84E-01	-33.55	76.65	6.62E-01	150.44	83.10	7.02E-02	87.67	46.17	5.76E-02
cg12323063	6.43	3.07	3.59E-02	7.81	4.29	6.85E-02	7.03	8.39	4.03E-01	6.93	6.67	2.99E-01	0.26	8.09	9.75E-01
cg04787750	21.38	10.19	3.59E-02	14.92	18.98	4.32E-01	25.05	19.80	2.06E-01	23.98	18.47	1.94E-01	22.06	26.99	4.14E-01
cg01675229	-29.96	14.28	3.59E-02	-24.68	18.63	1.85E-01	-48.29	80.05	5.46E-01	-53.34	62.41	3.93E-01	-33.94	24.95	1.74E-01
cg00685723	41.28	19.68	3.59E-02	26.80	25.32	2.90E-01	110.23	63.02	8.03E-02	-27.83	81.22	7.32E-01	66.65	40.20	9.73E-02
cg13356540	-16.01	7.63	3.59E-02	-27.06	12.57	3.13E-02	5.28	14.93	7.24E-01	-26.16	13.90	5.98E-02	-10.35	15.94	5.16E-01
cg00382185	18.96	9.04	3.59E-02	33.41	14.90	2.49E-02	8.57	19.41	6.59E-01	1.48	15.25	9.23E-01	38.09	22.78	9.45E-02
cg19172278	-6.76	3.22	3.59E-02	-10.53	4.44	1.77E-02	-0.33	8.16	9.67E-01	-4.36	10.26	6.71E-01	-3.34	6.92	6.30E-01
cg10510558	-14.68	7.00	3.59E-02	3.00	19.95	8.80E-01	-11.54	26.38	6.62E-01	-16.90	8.08	3.64E-02	-27.92	29.71	3.47E-01
cg17767567	14.07	6.71	3.59E-02	35.29	19.08	6.43E-02	36.72	24.62	1.36E-01	9.42	7.86	2.31E-01	1.63	24.67	9.47E-01
cg16695483	28.39	13.53	3.59E-02	42.38	30.38	1.63E-01	30.66	67.72	6.51E-01	18.59	17.12	2.78E-01	52.11	36.56	1.54E-01
cg05744184	7.35	3.50	3.59E-02	7.97	4.18	5.64E-02	13.98	10.21	1.71E-01	-3.24	12.14	7.89E-01	3.85	11.33	7.34E-01
cg21844291	-12.17	5.80	3.59E-02	-19.02	12.02	1.14E-01	-5.25	13.55	6.98E-01	-12.36	8.94	1.67E-01	-9.64	14.40	5.03E-01
cg23275958	9.44	4.50	3.59E-02	13.27	8.08	1.00E-01	19.58	9.22	3.37E-02	3.01	9.05	7.39E-01	-0.47	9.98	9.62E-01
cg21432954	15.97	7.61	3.59E-02	18.34	12.16	1.31E-01	33.80	22.76	1.37E-01	29.33	22.46	1.92E-01	4.27	12.33	7.29E-01
cg00585558	16.13	7.69	3.59E-02	25.32	10.68	1.77E-02	-12.36	32.62	7.05E-01	35.92	43.00	4.03E-01	6.44	12.26	6.00E-01
cg13982220	16.90	8.06	3.59E-02	18.52	13.49	1.70E-01	20.39	14.82	1.69E-01	13.73	17.66	4.37E-01	10.09	21.54	6.39E-01
cg07310259	19.38	9.24	3.59E-02	22.81	11.96	5.65E-02	60.34	58.85	3.05E-01	4.09	48.88	9.33E-01	12.06	15.78	4.45E-01
cg01377459	-15.00	7.15	3.60E-02	-29.87	17.75	9.24E-02	-14.57	27.83	6.01E-01	-14.04	8.63	1.04E-01	5.39	24.58	8.26E-01
cg27348370	-9.07	4.33	3.60E-02	5.97	10.23	5.60E-01	-14.03	18.53	4.49E-01	-11.42	5.32	3.20E-02	-17.24	13.24	1.93E-01
cg24032156	16.37	7.80	3.60E-02	15.71	12.09	1.94E-01	12.23	15.19	4.20E-01	18.89	19.37	3.29E-01	22.47	19.72	2.54E-01
cg02206323	-12.14	5.79	3.60E-02	-5.35	10.44	6.08E-01	-12.33	13.07	3.45E-01	-21.30	12.38	8.53E-02	-12.32	10.99	2.62E-01

cg25791868	-14.78	7.04	3.60E-02	-10.03	13.55	4.59E-01	-17.45	19.36	3.67E-01	-12.81	9.94	1.97E-01	-34.98	22.88	1.26E-01
cg11907323	-8.23	3.92	3.60E-02	-11.92	10.17	2.41E-01	6.84	13.52	6.13E-01	-7.63	4.77	1.10E-01	-20.56	12.99	1.13E-01
cg27539721	50.22	23.94	3.60E-02	70.00	36.18	5.30E-02	48.62	98.36	6.21E-01	98.69	47.06	3.60E-02	-2.60	37.26	9.44E-01
cg11559250	29.77	14.19	3.60E-02	28.12	19.32	1.46E-01	-0.93	54.14	9.86E-01	114.82	53.87	3.30E-02	20.76	25.01	4.06E-01
cg20497635	5.40	2.57	3.60E-02	4.45	3.22	1.68E-01	11.38	6.32	7.18E-02	4.75	8.28	5.66E-01	2.15	8.20	7.93E-01
cg27056145	18.72	8.93	3.60E-02	22.85	20.86	2.73E-01	23.16	40.42	5.67E-01	11.65	10.84	2.83E-01	61.53	29.84	3.92E-02
cg01640684	-3.31	1.58	3.60E-02	-3.75	2.21	8.95E-02	-3.72	2.92	2.03E-01	-0.22	7.95	9.78E-01	-1.90	3.99	6.34E-01
cg07841312	28.29	13.49	3.60E-02	14.65	47.93	7.60E-01	236.93	203.04	2.43E-01	24.33	14.64	9.66E-02	80.62	51.98	1.21E-01
cg09321029	14.53	6.93	3.60E-02	16.41	10.16	1.06E-01	18.81	13.75	1.71E-01	11.25	22.97	6.24E-01	5.81	15.84	7.14E-01
cg22878148	-10.52	5.02	3.60E-02	-20.42	11.48	7.53E-02	16.90	18.45	3.60E-01	-12.27	4.61	7.74E-03	-1.50	15.36	9.22E-01
cg21088542	-10.02	4.78	3.60E-02	-22.43	11.18	4.49E-02	3.26	20.78	8.75E-01	-6.23	5.90	2.91E-01	-18.40	14.44	2.03E-01
cg00264419	-12.64	6.03	3.60E-02	-25.21	13.77	6.71E-02	-16.93	15.96	2.89E-01	-6.28	8.09	4.37E-01	-17.26	18.13	3.41E-01
cg20826165	-10.71	5.11	3.60E-02	-20.52	13.72	1.35E-01	-22.48	23.50	3.39E-01	-7.53	5.91	2.02E-01	-17.36	19.56	3.75E-01
cg08130912	8.72	4.16	3.60E-02	9.76	5.95	1.01E-01	15.86	10.70	1.38E-01	12.90	9.40	1.70E-01	-5.79	10.21	5.70E-01
cg20564969	19.67	9.38	3.60E-02	24.38	12.36	4.85E-02	18.46	30.89	5.50E-01	48.14	31.28	1.24E-01	-1.71	19.09	9.29E-01
cg19502936	15.83	7.55	3.60E-02	31.81	19.60	1.05E-01	4.32	22.49	8.48E-01	13.23	9.23	1.52E-01	25.15	28.39	3.76E-01
cg20974724	18.43	8.79	3.60E-02	34.76	10.46	8.91E-04	19.48	14.24	1.71E-01	23.90	11.30	3.44E-02	-1.60	8.93	8.58E-01
cg24866784	19.80	9.44	3.60E-02	30.95	19.57	1.14E-01	18.56	19.76	3.48E-01	14.24	14.71	3.33E-01	19.62	26.48	4.59E-01
cg13111733	36.31	17.31	3.60E-02	43.81	21.91	4.55E-02	9.25	110.37	9.33E-01	19.34	81.34	8.12E-01	25.65	31.34	4.13E-01
cg16842187	4.09	1.95	3.60E-02	4.98	3.27	1.27E-01	3.44	4.18	4.10E-01	5.40	3.61	1.35E-01	-0.04	5.30	9.94E-01
cg10792238	16.90	8.06	3.60E-02	36.35	22.72	1.10E-01	19.43	24.60	4.30E-01	9.98	9.70	3.03E-01	43.85	29.18	1.33E-01
cg07672051	10.88	5.19	3.60E-02	7.80	7.12	2.73E-01	18.95	11.60	1.03E-01	21.20	18.27	2.46E-01	6.57	11.99	5.84E-01
cg00746083	31.89	15.21	3.60E-02	33.16	18.17	6.79E-02	-63.54	77.63	4.13E-01	-16.21	80.78	8.41E-01	51.77	32.06	1.06E-01
cg00091760	-8.37	3.99	3.60E-02	-9.06	6.44	1.59E-01	-3.50	10.06	7.28E-01	-9.44	10.32	3.60E-01	-9.49	7.20	1.87E-01
cg08481075	7.52	3.59	3.60E-02	7.49	6.45	2.46E-01	12.76	8.16	1.18E-01	8.26	6.77	2.22E-01	1.93	7.70	8.02E-01
cg00138101	13.62	6.50	3.60E-02	14.31	9.11	1.16E-01	2.93	20.90	8.88E-01	26.59	13.80	5.39E-02	0.93	15.63	9.53E-01
cg26219977	15.72	7.50	3.60E-02	14.35	18.28	4.32E-01	35.66	42.52	4.02E-01	15.71	8.78	7.34E-02	10.27	28.30	7.17E-01
cg17401435	18.66	8.90	3.60E-02	14.13	17.05	4.07E-01	7.22	17.47	6.79E-01	23.68	15.13	1.18E-01	38.96	25.53	1.27E-01
cg16080512	33.17	15.82	3.60E-02	35.84	17.50	4.06E-02	18.39	73.12	8.01E-01	140.00	70.79	4.80E-02	4.23	32.71	8.97E-01
cg12187358	-18.70	8.92	3.60E-02	-28.20	15.90	7.60E-02	-12.43	18.42	5.00E-01	-3.80	15.93	8.12E-01	-41.84	24.15	8.32E-02
cg06155414	-14.89	7.10	3.61E-02	-10.95	15.03	4.66E-01	-5.91	25.81	8.19E-01	-14.13	9.12	1.21E-01	-36.38	23.14	1.16E-01
cg08504812	-7.07	3.37	3.61E-02	-7.26	5.34	1.73E-01	1.13	8.72	8.97E-01	-8.53	6.70	2.03E-01	-11.03	7.59	1.46E-01
cg07672908	-5.84	2.79	3.61E-02	-7.24	4.20	8.45E-02	-4.93	5.67	3.85E-01	-1.72	7.87	8.27E-01	-6.47	6.36	3.09E-01
cg21190595	-11.56	5.52	3.61E-02	-17.41	8.45	3.94E-02	-2.46	12.35	8.42E-01	-5.43	15.11	7.19E-01	-12.14	11.22	2.79E-01
cg23330212	-8.45	4.03	3.61E-02	-16.26	8.22	4.79E-02	0.05	11.59	9.97E-01	-6.32	5.82	2.77E-01	-9.55	10.15	3.46E-01

cg03104704	-35.65	17.01	3.61E-02	-48.38	21.94	2.74E-02	-40.48	53.41	4.49E-01	13.78	45.48	7.62E-01	-27.84	42.80	5.15E-01
cg03225235	21.44	10.23	3.61E-02	20.96	18.89	2.67E-01	26.31	21.29	2.17E-01	11.90	17.63	5.00E-01	37.45	27.40	1.72E-01
cg26418434	9.06	4.32	3.61E-02	8.29	4.48	6.43E-02	7.72	7.97	3.33E-01	28.53	10.88	8.72E-03	1.03	7.26	8.87E-01
cg02322048	-38.51	18.37	3.61E-02	-37.12	23.71	1.17E-01	4.68	67.53	9.45E-01	-59.29	49.33	2.29E-01	-44.66	42.51	2.94E-01
cg25769590	5.39	2.57	3.61E-02	1.06	2.92	7.15E-01	5.70	4.36	1.91E-01	7.06	7.70	3.59E-01	11.69	4.42	8.18E-03
cg20000940	46.17	22.03	3.61E-02	44.59	29.32	1.28E-01	50.95	117.03	6.63E-01	59.32	49.78	2.33E-01	37.13	48.72	4.46E-01
cg06022561	-5.23	2.49	3.61E-02	-5.07	3.66	1.66E-01	-5.99	5.18	2.47E-01	0.52	8.18	9.50E-01	-7.25	5.42	1.81E-01
cg21069005	10.44	4.98	3.61E-02	9.31	7.28	2.01E-01	13.60	11.50	2.37E-01	24.52	13.97	7.92E-02	1.90	10.68	8.59E-01
cg07970948	-16.42	7.84	3.61E-02	-25.14	12.60	4.59E-02	-21.06	18.57	2.57E-01	3.51	18.79	8.52E-01	-13.61	15.33	3.75E-01
cg24887140	27.22	12.99	3.61E-02	16.65	16.37	3.09E-01	40.69	74.40	5.84E-01	85.36	60.24	1.57E-01	39.32	23.99	1.01E-01
cg12995004	-8.76	4.18	3.61E-02	-9.27	10.16	3.62E-01	-11.49	17.39	5.09E-01	-8.53	5.12	9.58E-02	-7.95	12.85	5.36E-01
cg20405742	-9.52	4.54	3.61E-02	-13.96	9.98	1.62E-01	-5.43	13.50	6.87E-01	-5.03	6.10	4.09E-01	-25.64	12.80	4.52E-02
cg09528275	17.74	8.46	3.61E-02	19.02	13.85	1.70E-01	5.45	23.19	8.14E-01	48.24	30.73	1.16E-01	14.97	13.09	2.53E-01
cg21139587	12.01	5.73	3.61E-02	17.39	7.96	2.88E-02	12.54	14.34	3.82E-01	17.98	17.79	3.12E-01	-4.08	12.29	7.40E-01
cg06529600	-6.49	3.10	3.61E-02	-11.04	4.93	2.50E-02	0.50	6.30	9.37E-01	-9.24	6.50	1.55E-01	-1.09	8.41	8.97E-01
cg19851242	-20.79	9.92	3.61E-02	28.86	36.96	4.35E-01	122.20	152.17	4.22E-01	-25.75	10.63	1.55E-02	-18.39	43.00	6.69E-01
cg23757825	14.88	7.10	3.61E-02	8.62	15.45	5.77E-01	-14.72	24.58	5.49E-01	22.65	9.13	1.31E-02	5.88	22.35	7.93E-01
cg10223233	8.24	3.93	3.61E-02	5.66	5.08	2.66E-01	20.92	13.67	1.26E-01	7.67	9.51	4.20E-01	12.21	10.18	2.30E-01
cg19447156	-29.27	13.97	3.61E-02	-90.58	49.27	6.60E-02	53.11	200.59	7.91E-01	-25.26	15.25	9.76E-02	-13.96	50.64	7.83E-01
cg22430463	12.90	6.16	3.61E-02	8.96	8.91	3.15E-01	16.20	12.77	2.05E-01	31.96	14.97	3.28E-02	-4.47	17.68	8.00E-01
cg13720108	15.51	7.40	3.61E-02	18.35	9.91	6.42E-02	18.52	21.45	3.88E-01	17.40	26.81	5.16E-01	7.08	14.89	6.35E-01
cg01049916	-19.00	9.07	3.61E-02	-19.70	12.59	1.18E-01	-4.70	26.58	8.60E-01	-43.76	24.99	8.00E-02	-10.62	18.75	5.71E-01
cg10765397	-14.49	6.92	3.61E-02	-20.58	12.10	8.91E-02	-10.06	16.09	5.32E-01	-10.94	18.30	5.50E-01	-12.58	11.76	2.85E-01
cg00741986	-8.36	3.99	3.61E-02	-10.66	5.27	4.29E-02	-11.48	8.56	1.80E-01	4.04	17.21	8.15E-01	0.26	10.16	9.79E-01
cg05926722	9.51	4.54	3.61E-02	12.36	6.83	7.03E-02	7.56	11.49	5.10E-01	16.50	10.72	1.24E-01	-0.37	9.60	9.69E-01
cg21759570	7.81	3.73	3.61E-02	7.46	5.63	1.85E-01	16.61	9.18	7.03E-02	10.97	10.45	2.94E-01	1.51	7.18	8.34E-01
cg09818502	22.74	10.85	3.61E-02	10.87	29.47	7.12E-01	-35.39	100.81	7.26E-01	23.61	12.44	5.77E-02	40.49	35.87	2.59E-01
cg07551022	19.95	9.52	3.61E-02	32.15	21.03	1.26E-01	11.03	51.03	8.29E-01	17.94	11.57	1.21E-01	10.00	33.13	7.63E-01
cg05522349	29.10	13.89	3.61E-02	47.32	20.00	1.80E-02	3.08	33.32	9.26E-01	13.91	7.05	4.85E-02	76.35	33.90	2.43E-02
cg09366312	14.72	7.02	3.61E-02	20.92	13.22	1.13E-01	22.06	24.28	3.64E-01	14.19	12.44	2.54E-01	7.74	12.51	5.36E-01
cg19972590	-14.04	6.70	3.61E-02	-13.61	16.85	4.19E-01	-28.07	28.04	3.17E-01	-15.46	8.15	5.78E-02	1.54	20.35	9.40E-01
cg25585712	13.86	6.62	3.61E-02	17.01	10.38	1.01E-01	14.34	17.51	4.13E-01	26.41	14.44	6.73E-02	-2.68	13.48	8.42E-01
cg23721425	14.51	6.92	3.61E-02	9.20	14.00	5.11E-01	3.48	18.50	8.51E-01	21.04	10.02	3.57E-02	12.51	18.65	5.02E-01
cg15262728	-42.19	20.13	3.61E-02	-37.54	28.65	1.90E-01	92.43	114.10	4.18E-01	-58.02	36.56	1.12E-01	-52.01	48.61	2.85E-01
cg07763737	11.43	5.45	3.61E-02	19.82	12.56	1.15E-01	26.55	25.30	2.94E-01	9.90	6.65	1.37E-01	-2.04	17.85	9.09E-01

cg10538486	-8.92	4.26	3.61E-02	-11.44	11.95	3.38E-01	-22.85	21.84	2.95E-01	-6.28	4.87	1.97E-01	-25.36	15.99	1.13E-01
cg02958515	-7.09	3.39	3.62E-02	-5.03	6.09	4.08E-01	-5.30	6.12	3.87E-01	-10.17	6.48	1.16E-01	-10.20	10.15	3.15E-01
cg00100703	-14.72	7.03	3.62E-02	-6.48	17.03	7.04E-01	24.40	35.45	4.91E-01	-20.09	8.30	1.55E-02	-2.54	25.73	9.21E-01
cg15199350	-14.92	7.12	3.62E-02	-40.73	17.46	1.97E-02	-8.45	30.37	7.81E-01	-8.26	7.72	2.85E-01	-24.59	23.37	2.93E-01
cg11974710	36.01	17.19	3.62E-02	27.68	21.77	2.04E-01	94.27	84.49	2.65E-01	-31.05	94.17	7.42E-01	52.63	31.28	9.24E-02
cg26140512	8.36	3.99	3.62E-02	6.77	7.18	3.46E-01	8.22	6.75	2.23E-01	2.05	10.36	8.43E-01	15.93	9.05	7.84E-02
cg26935304	-10.69	5.10	3.62E-02	-12.14	10.54	2.50E-01	19.24	17.59	2.74E-01	-12.55	5.30	1.79E-02	-19.69	14.96	1.88E-01
cg17278062	13.09	6.25	3.62E-02	21.40	8.10	8.24E-03	14.11	12.65	2.65E-01	16.01	17.13	3.50E-01	-6.14	12.41	6.21E-01
cg21029108	12.81	6.11	3.62E-02	10.37	9.93	2.97E-01	17.11	14.03	2.23E-01	8.73	10.24	3.94E-01	33.77	22.38	1.31E-01
cg16798252	13.64	6.51	3.62E-02	12.69	11.51	2.70E-01	17.48	15.37	2.55E-01	13.41	13.84	3.32E-01	12.44	12.33	3.13E-01
cg00686761	-21.29	10.16	3.62E-02	-9.63	24.93	6.99E-01	-111.90	112.50	3.20E-01	-27.02	11.83	2.24E-02	13.27	34.34	6.99E-01
cg14208664	14.99	7.16	3.62E-02	29.27	19.37	1.31E-01	10.84	21.54	6.15E-01	9.20	8.71	2.91E-01	46.06	25.64	7.25E-02
cg20574381	-11.94	5.70	3.62E-02	-15.03	16.97	3.76E-01	-5.67	30.67	8.53E-01	-10.11	6.40	1.14E-01	-34.15	23.39	1.44E-01
cg23129930	6.06	2.89	3.62E-02	6.15	3.93	1.18E-01	9.68	6.13	1.14E-01	1.55	11.48	8.92E-01	2.77	6.97	6.90E-01
cg04698114	15.44	7.37	3.62E-02	54.88	25.29	3.00E-02	25.38	33.00	4.42E-01	9.61	8.27	2.45E-01	18.84	19.99	3.46E-01
cg08681409	9.46	4.52	3.62E-02	10.85	7.36	1.41E-01	-0.04	8.81	9.96E-01	18.94	13.47	1.60E-01	13.11	9.05	1.48E-01
cg08013270	19.87	9.48	3.62E-02	44.48	14.49	2.15E-03	11.72	26.44	6.58E-01	10.07	12.62	4.25E-01	8.03	16.61	6.29E-01
cg22454053	10.94	5.22	3.62E-02	8.16	8.36	3.29E-01	-5.07	10.27	6.22E-01	16.42	5.94	5.70E-03	23.14	12.76	6.97E-02
cg20558646	16.43	7.84	3.62E-02	18.11	15.84	2.53E-01	5.83	33.65	8.62E-01	16.12	10.20	1.14E-01	19.64	23.76	4.08E-01
cg13678205	-12.88	6.15	3.62E-02	-9.62	9.76	3.24E-01	-9.33	12.80	4.66E-01	-12.00	14.16	3.97E-01	-25.19	14.31	7.83E-02
cg01013890	-30.74	14.68	3.62E-02	-24.83	18.32	1.75E-01	-47.91	41.98	2.54E-01	5.53	62.33	9.29E-01	-51.25	34.52	1.38E-01
cg00037940	16.52	7.89	3.62E-02	49.00	23.04	3.34E-02	-4.40	45.40	9.23E-01	13.39	8.94	1.34E-01	6.65	28.89	8.18E-01
cg07654982	-10.10	4.82	3.62E-02	-4.42	6.86	5.20E-01	-8.17	9.66	3.98E-01	-19.82	18.96	2.96E-01	-23.82	10.97	2.99E-02
cg01437439	15.33	7.32	3.62E-02	12.03	13.98	3.90E-01	28.37	15.89	7.42E-02	11.06	11.66	3.43E-01	13.81	21.10	5.13E-01
cg07402669	12.78	6.10	3.62E-02	25.61	11.80	3.00E-02	3.53	16.57	8.31E-01	14.40	10.87	1.85E-01	3.25	11.48	7.77E-01
cg23775883	-6.65	3.18	3.62E-02	-7.89	4.95	1.11E-01	-5.88	6.46	3.63E-01	6.61	9.95	5.06E-01	-10.86	6.43	9.12E-02
cg04607334	17.83	8.51	3.62E-02	37.28	22.48	9.73E-02	-4.00	41.46	9.23E-01	13.55	9.83	1.68E-01	38.86	33.69	2.49E-01
cg15600488	12.83	6.13	3.62E-02	16.13	10.81	1.35E-01	20.92	19.34	2.79E-01	18.83	11.07	8.91E-02	-0.80	11.75	9.46E-01
cg22855052	14.56	6.95	3.62E-02	16.26	13.81	2.39E-01	-27.32	35.13	4.37E-01	18.08	9.25	5.06E-02	9.08	18.36	6.21E-01
cg00079169	16.16	7.72	3.62E-02	5.88	18.23	7.47E-01	39.44	19.09	3.88E-02	9.73	9.00	2.80E-01	42.27	27.70	1.27E-01
cg09156073	-9.77	4.67	3.62E-02	-6.90	7.65	3.67E-01	-11.28	12.13	3.52E-01	-9.05	9.26	3.28E-01	-14.31	9.82	1.45E-01
cg03617054	6.44	3.08	3.62E-02	3.06	4.70	5.15E-01	13.49	6.55	3.95E-02	11.93	8.79	1.75E-01	3.06	6.42	6.34E-01
cg04661194	13.96	6.67	3.62E-02	28.95	17.87	1.05E-01	23.52	26.32	3.71E-01	12.72	7.77	1.02E-01	-15.74	27.23	5.63E-01
cg06913865	-6.42	3.07	3.62E-02	-9.83	5.58	7.80E-02	3.66	6.79	5.90E-01	-9.29	5.12	6.95E-02	-6.42	8.38	4.44E-01
cg04406863	-9.22	4.40	3.62E-02	-6.14	6.00	3.06E-01	-16.09	11.36	1.57E-01	-19.61	11.59	9.08E-02	-4.01	10.74	7.09E-01

cg16081457	-5.04	2.40	3.62E-02	-6.05	3.64	9.61E-02	-5.27	5.03	2.95E-01	-2.01	6.40	7.54E-01	-4.67	5.46	3.93E-01
cg14561672	31.51	15.04	3.62E-02	40.11	31.08	1.97E-01	-53.95	81.36	5.07E-01	27.89	19.24	1.47E-01	57.52	43.45	1.86E-01
cg04980428	10.31	4.92	3.62E-02	17.53	7.12	1.38E-02	10.27	13.09	4.33E-01	2.37	10.78	8.26E-01	-0.10	11.87	9.93E-01
cg19960778	-19.84	9.48	3.62E-02	-46.40	13.18	4.29E-04	-9.01	16.81	5.92E-01	-10.05	12.15	4.08E-01	-11.19	14.56	4.42E-01
cg08842062	22.69	10.84	3.62E-02	36.33	21.91	9.72E-02	51.35	25.16	4.12E-02	7.09	9.90	4.73E-01	32.86	29.65	2.68E-01
cg18130688	-8.64	4.13	3.62E-02	-9.08	8.15	2.65E-01	-29.31	15.97	6.64E-02	-7.69	5.55	1.66E-01	-0.80	11.74	9.46E-01
cg06962678	-27.24	13.01	3.62E-02	-23.54	18.17	1.95E-01	-105.41	56.85	6.37E-02	-30.86	27.43	2.61E-01	-12.93	28.35	6.48E-01
cg11520366	19.95	9.53	3.63E-02	24.66	26.11	3.45E-01	88.85	133.55	5.06E-01	18.20	10.56	8.48E-02	29.34	43.65	5.01E-01
cg16786703	27.28	13.03	3.63E-02	43.95	22.19	4.76E-02	-0.32	35.25	9.93E-01	7.87	36.20	8.28E-01	28.67	20.89	1.70E-01
cg10499926	-32.96	15.74	3.63E-02	-42.06	17.86	1.85E-02	43.73	91.10	6.31E-01	16.69	70.56	8.13E-01	-16.89	41.56	6.84E-01
cg10307632	-8.98	4.29	3.63E-02	-7.88	9.50	4.07E-01	-16.63	20.98	4.28E-01	-6.64	5.45	2.23E-01	-18.83	11.59	1.04E-01
cg05781996	8.44	4.03	3.63E-02	6.72	5.46	2.19E-01	17.41	9.33	6.21E-02	4.57	15.57	7.69E-01	6.07	8.97	4.99E-01
cg02846329	15.63	7.46	3.63E-02	3.77	18.20	8.36E-01	60.68	48.14	2.08E-01	17.03	8.82	5.34E-02	14.58	24.72	5.55E-01
cg22488719	-8.67	4.14	3.63E-02	-19.02	9.49	4.51E-02	-9.10	12.50	4.66E-01	-2.64	5.65	6.40E-01	-16.16	10.27	1.16E-01
cg20739510	13.65	6.52	3.63E-02	15.80	7.55	3.64E-02	-6.04	22.59	7.89E-01	46.34	32.81	1.58E-01	4.19	17.87	8.14E-01
cg18196374	-11.39	5.44	3.63E-02	-10.38	12.11	3.91E-01	-2.81	18.63	8.80E-01	-12.39	7.23	8.66E-02	-13.94	14.22	3.27E-01
cg00960395	19.20	9.17	3.63E-02	22.13	15.42	1.51E-01	26.88	27.92	3.36E-01	18.49	23.31	4.27E-01	14.62	14.79	3.23E-01
cg07312514	18.59	8.88	3.63E-02	47.57	46.93	3.11E-01	37.65	203.31	8.53E-01	17.30	9.44	6.68E-02	19.47	31.89	5.41E-01
cg07829740	-12.18	5.82	3.63E-02	-25.98	12.64	3.98E-02	-20.33	21.18	3.37E-01	-5.06	6.60	4.43E-01	-29.35	21.33	1.69E-01
cg22745437	42.79	20.44	3.63E-02	37.28	44.21	3.99E-01	-344.03	234.45	1.42E-01	43.10	23.64	6.83E-02	78.26	61.37	2.02E-01
cg01615704	10.87	5.19	3.63E-02	19.57	9.91	4.84E-02	18.55	14.22	1.92E-01	9.74	11.05	3.78E-01	2.37	8.53	7.81E-01
cg17526770	18.90	9.03	3.63E-02	37.47	16.67	2.46E-02	11.92	15.89	4.53E-01	20.46	18.23	2.62E-01	-6.85	24.22	7.77E-01
cg16569014	31.66	15.12	3.63E-02	30.73	17.24	7.47E-02	-24.14	91.81	7.93E-01	73.99	72.74	3.09E-01	34.17	37.75	3.65E-01
cg09294084	7.04	3.36	3.63E-02	7.65	5.07	1.31E-01	11.35	6.66	8.81E-02	7.42	8.90	4.04E-01	-1.76	8.35	8.34E-01
cg01708648	17.75	8.48	3.63E-02	20.41	18.01	2.57E-01	25.07	18.68	1.80E-01	15.87	12.53	2.05E-01	6.96	25.09	7.82E-01
cg23191956	16.69	7.97	3.63E-02	11.98	18.36	5.14E-01	14.16	26.91	5.99E-01	14.50	11.18	1.95E-01	26.98	17.16	1.16E-01
cg03808158	-32.60	15.57	3.63E-02	-40.64	20.11	4.33E-02	-38.50	78.71	6.25E-01	-88.38	70.66	2.11E-01	-7.81	27.83	7.79E-01
cg00830492	-16.41	7.84	3.63E-02	-49.72	20.84	1.71E-02	-7.94	35.34	8.22E-01	-10.66	8.64	2.17E-01	-17.47	30.17	5.63E-01
cg06618968	27.01	12.90	3.63E-02	24.26	15.72	1.23E-01	43.43	46.20	3.47E-01	71.96	63.46	2.57E-01	20.82	28.35	4.63E-01
cg19375539	-7.47	3.57	3.63E-02	-13.10	9.10	1.50E-01	-0.92	15.89	9.54E-01	-5.28	4.29	2.18E-01	-16.82	11.03	1.27E-01
cg15768265	9.69	4.63	3.63E-02	12.34	6.82	7.02E-02	7.35	10.07	4.65E-01	21.94	18.83	2.44E-01	4.19	8.96	6.40E-01
cg09233730	20.71	9.89	3.63E-02	29.38	19.03	1.23E-01	11.44	17.84	5.21E-01	14.63	19.13	4.44E-01	34.53	25.16	1.70E-01
cg14543285	5.09	2.43	3.63E-02	3.73	3.12	2.31E-01	12.13	6.26	5.26E-02	6.48	7.10	3.62E-01	1.85	6.90	7.89E-01
cg13242199	-10.55	5.04	3.63E-02	-6.23	7.63	4.14E-01	-22.62	14.42	1.17E-01	-0.25	12.31	9.84E-01	-18.36	9.63	5.66E-02
cg00753442	-8.37	4.00	3.63E-02	-16.09	9.69	9.69E-02	-7.67	18.17	6.73E-01	-6.79	4.81	1.58E-01	-6.31	13.22	6.33E-01

cg23306526	25.78	12.31	3.63E-02	39.08	19.37	4.36E-02	1.39	39.23	9.72E-01	40.41	26.16	1.22E-01	3.22	23.46	8.91E-01
cg08307963	27.64	13.21	3.63E-02	19.34	18.78	3.03E-01	36.86	36.06	3.07E-01	30.98	34.24	3.66E-01	38.29	27.97	1.71E-01
cg08089031	24.30	11.61	3.63E-02	29.99	19.64	1.27E-01	-8.58	48.52	8.60E-01	24.16	17.42	1.66E-01	24.01	30.04	4.24E-01
cg06516650	41.82	19.98	3.63E-02	35.83	23.40	1.26E-01	-27.20	112.48	8.09E-01	-2.53	64.14	9.69E-01	118.04	52.96	2.58E-02
cg14105952	-38.05	18.18	3.63E-02	-49.52	22.45	2.74E-02	-80.68	88.09	3.60E-01	29.26	70.44	6.78E-01	-17.40	37.50	6.43E-01
cg03366884	-8.81	4.21	3.63E-02	-4.99	5.35	3.51E-01	-14.91	11.56	1.97E-01	-19.53	12.88	1.29E-01	-11.78	11.23	2.94E-01
cg27203560	12.02	5.74	3.63E-02	15.06	6.54	2.13E-02	28.84	13.81	3.67E-02	12.44	11.81	2.92E-01	-3.14	9.78	7.48E-01
cg22942950	37.95	18.13	3.63E-02	25.50	24.36	2.95E-01	150.22	84.92	7.69E-02	-9.19	56.79	8.71E-01	56.73	31.06	6.78E-02
cg13060154	-33.77	16.13	3.63E-02	-24.43	22.67	2.81E-01	-41.60	50.65	4.11E-01	-0.86	45.29	9.85E-01	-64.32	31.32	4.00E-02
cg12540260	21.24	10.15	3.63E-02	50.54	26.13	5.31E-02	-114.01	134.37	3.96E-01	17.15	11.51	1.36E-01	14.17	39.49	7.20E-01
cg04416111	-9.67	4.62	3.63E-02	-9.30	9.41	3.23E-01	-3.26	11.74	7.82E-01	-11.86	6.88	8.48E-02	-10.26	11.76	3.83E-01
cg10526277	13.95	6.66	3.63E-02	23.65	10.29	2.16E-02	3.98	19.04	8.34E-01	17.46	21.48	4.16E-01	5.16	11.07	6.41E-01
cg07023704	20.20	9.65	3.63E-02	16.72	13.93	2.30E-01	22.54	27.19	4.07E-01	48.22	29.53	1.03E-01	14.56	17.99	4.18E-01
cg19581696	-27.57	13.17	3.63E-02	-47.50	31.65	1.33E-01	-191.04	167.38	2.54E-01	-26.39	15.27	8.39E-02	19.33	47.61	6.85E-01
cg07645004	21.15	10.10	3.63E-02	24.14	17.29	1.63E-01	25.51	19.27	1.86E-01	2.27	21.50	9.16E-01	33.10	25.04	1.86E-01
cg24122364	16.26	7.77	3.63E-02	-2.08	19.12	9.13E-01	24.69	25.20	3.27E-01	17.38	9.47	6.65E-02	38.39	30.10	2.02E-01
cg00281152	31.72	15.16	3.64E-02	17.49	29.73	5.56E-01	16.11	131.47	9.02E-01	38.94	19.06	4.11E-02	24.77	49.33	6.16E-01
cg00742738	13.63	6.51	3.64E-02	-8.78	19.43	6.51E-01	31.28	19.79	1.14E-01	12.50	7.66	1.03E-01	38.23	27.10	1.58E-01
cg06335773	52.43	25.05	3.64E-02	79.70	29.91	7.72E-03	33.76	99.60	7.35E-01	59.82	74.84	4.24E-01	-28.73	54.32	5.97E-01
cg16943185	-12.93	6.18	3.64E-02	-21.70	10.51	3.90E-02	-6.48	14.22	6.49E-01	-14.83	12.52	2.36E-01	-2.67	13.13	8.39E-01
cg07405182	-4.65	2.22	3.64E-02	-4.39	3.71	2.36E-01	-8.54	5.38	1.12E-01	-3.49	3.98	3.81E-01	-3.33	5.55	5.49E-01
cg14301348	-7.45	3.56	3.64E-02	-10.72	4.93	2.98E-02	-5.06	9.55	5.96E-01	2.30	9.08	8.00E-01	-8.16	8.26	3.23E-01
cg24385940	-8.60	4.11	3.64E-02	-18.78	7.88	1.71E-02	-5.70	9.52	5.49E-01	-5.66	6.36	3.73E-01	-0.51	11.66	9.65E-01
cg20401101	13.03	6.23	3.64E-02	19.21	12.90	1.36E-01	1.58	13.93	9.10E-01	19.76	9.50	3.75E-02	-1.85	16.82	9.13E-01
cg14520511	34.37	16.42	3.64E-02	27.34	19.94	1.70E-01	129.44	76.64	9.12E-02	86.50	91.34	3.44E-01	29.10	33.26	3.82E-01
cg16108230	11.34	5.42	3.64E-02	20.40	7.23	4.79E-03	8.17	10.58	4.40E-01	15.69	11.83	1.85E-01	-2.60	9.58	7.86E-01
cg06754606	17.00	8.12	3.64E-02	23.85	12.77	6.17E-02	8.48	15.28	5.79E-01	3.65	22.54	8.71E-01	24.45	18.99	1.98E-01
cg04552451	24.26	11.60	3.64E-02	59.50	17.96	9.23E-04	28.39	34.68	4.13E-01	14.66	7.56	5.24E-02	6.44	16.78	7.01E-01
cg19119032	12.86	6.15	3.64E-02	23.24	10.25	2.33E-02	6.40	15.76	6.85E-01	20.12	10.81	6.26E-02	-1.25	10.31	9.03E-01
cg00469771	11.27	5.39	3.64E-02	10.00	7.14	1.61E-01	9.45	12.33	4.43E-01	27.91	20.03	1.63E-01	10.51	13.22	4.26E-01
cg18412834	22.86	10.93	3.64E-02	73.45	48.26	1.28E-01	-7.04	99.89	9.44E-01	20.19	11.80	8.71E-02	23.49	38.65	5.43E-01
cg24737761	4.83	2.31	3.64E-02	4.43	3.05	1.47E-01	10.54	6.02	7.99E-02	2.97	5.67	6.00E-01	2.22	6.78	7.43E-01
cg24548817	5.20	2.48	3.64E-02	3.74	3.05	2.20E-01	9.82	6.03	1.04E-01	6.93	8.62	4.21E-01	5.66	8.58	5.09E-01
cg11380748	-10.73	5.13	3.64E-02	-9.28	15.42	5.47E-01	3.38	22.73	8.82E-01	-13.19	5.80	2.30E-02	7.64	21.56	7.23E-01
cg16036142	34.98	16.72	3.64E-02	22.42	14.47	1.21E-01	105.69	41.58	1.10E-02	76.25	41.27	6.47E-02	10.35	14.91	4.88E-01

cg02940562	11.14	5.32	3.64E-02	2.83	7.82	7.18E-01	15.97	9.43	9.01E-02	2.11	16.45	8.98E-01	25.37	11.46	2.69E-02
cg26186331	-11.36	5.43	3.64E-02	-18.66	8.09	2.11E-02	-1.91	9.36	8.38E-01	-4.01	6.70	5.49E-01	-26.35	11.24	1.91E-02
cg10961753	14.19	6.78	3.64E-02	38.24	17.43	2.83E-02	16.48	26.20	5.29E-01	11.17	8.44	1.85E-01	0.56	18.44	9.76E-01
cg01455456	-9.93	4.75	3.64E-02	-8.77	10.51	4.04E-01	-9.29	20.08	6.44E-01	-9.30	5.90	1.15E-01	-17.42	15.67	2.66E-01
cg11287647	6.02	2.88	3.64E-02	10.31	4.23	1.48E-02	6.30	5.74	2.72E-01	-4.23	8.17	6.04E-01	1.52	6.99	8.28E-01
cg13483882	13.32	6.37	3.65E-02	24.03	7.86	2.24E-03	-6.34	13.66	6.43E-01	14.62	12.98	2.60E-01	9.27	11.37	4.15E-01
cg25722198	29.59	14.14	3.65E-02	44.13	41.23	2.84E-01	-138.71	134.65	3.03E-01	26.05	15.92	1.02E-01	65.10	49.23	1.86E-01
cg17719099	21.52	10.29	3.65E-02	11.91	20.96	5.70E-01	1.93	44.74	9.66E-01	27.95	13.10	3.29E-02	14.68	34.31	6.69E-01
cg14472291	-8.84	4.23	3.65E-02	-16.62	9.47	7.92E-02	-8.47	11.65	4.67E-01	-6.06	5.92	3.06E-01	-8.35	10.64	4.33E-01
cg15025383	-34.26	16.38	3.65E-02	-11.91	31.03	7.01E-01	-107.57	131.98	4.15E-01	-37.05	21.70	8.77E-02	-60.01	44.38	1.76E-01
cg26716103	12.64	6.04	3.65E-02	15.03	14.01	2.84E-01	50.19	30.11	9.55E-02	9.97	7.07	1.58E-01	12.35	28.86	6.69E-01
cg20731384	-11.47	5.48	3.65E-02	-15.30	7.98	5.53E-02	-4.42	11.37	6.97E-01	3.28	18.93	8.62E-01	-16.50	11.90	1.66E-01
cg03954045	-8.53	4.08	3.65E-02	-11.67	7.06	9.86E-02	-6.61	12.28	5.91E-01	-7.55	6.36	2.35E-01	-5.56	10.68	6.02E-01
cg02769781	8.96	4.28	3.65E-02	14.04	7.36	5.64E-02	12.82	8.40	1.27E-01	10.05	9.42	2.86E-01	-5.51	9.49	5.62E-01
cg26227310	-7.71	3.68	3.65E-02	-2.59	6.56	6.93E-01	5.60	13.26	6.73E-01	-13.49	5.18	9.25E-03	-4.94	11.55	6.69E-01
cg02027780	18.93	9.05	3.65E-02	4.05	19.60	8.36E-01	50.75	31.30	1.05E-01	23.51	11.74	4.53E-02	-1.29	27.42	9.62E-01
cg25200453	-11.71	5.60	3.65E-02	-16.08	11.11	1.48E-01	0.09	12.66	9.95E-01	-9.90	8.95	2.69E-01	-23.59	14.00	9.19E-02
cg11032474	-32.62	15.60	3.65E-02	-41.17	20.01	3.97E-02	-82.74	47.94	8.43E-02	-2.81	49.46	9.55E-01	7.60	36.03	8.33E-01
cg26942829	4.91	2.35	3.65E-02	4.99	3.13	1.11E-01	9.62	5.40	7.46E-02	3.34	6.51	6.08E-01	-1.16	6.74	8.63E-01
cg19350679	-4.97	2.38	3.65E-02	-6.62	4.30	1.24E-01	-0.39	5.57	9.44E-01	-5.85	3.85	1.28E-01	-4.95	6.57	4.51E-01
cg25162117	-15.70	7.51	3.65E-02	-35.23	13.11	7.18E-03	-21.35	24.90	3.91E-01	-6.24	7.06	3.77E-01	-13.62	18.25	4.55E-01
cg20981058	36.08	17.25	3.65E-02	50.68	42.32	2.31E-01	86.99	204.48	6.71E-01	25.04	19.97	2.10E-01	103.65	60.74	8.79E-02
cg21605566	29.30	14.01	3.65E-02	29.70	17.46	8.89E-02	2.22	83.90	9.79E-01	45.71	82.07	5.78E-01	29.34	25.60	2.52E-01
cg05817054	19.16	9.16	3.65E-02	19.78	17.16	2.49E-01	14.18	17.11	4.08E-01	16.61	18.45	3.68E-01	29.49	21.49	1.70E-01
cg25247596	14.49	6.93	3.65E-02	5.02	19.37	7.95E-01	0.03	25.09	9.99E-01	17.59	8.05	2.88E-02	14.73	29.79	6.21E-01
cg14861570	-12.30	5.88	3.65E-02	-22.79	8.63	8.25E-03	2.20	13.46	8.70E-01	-4.52	18.84	8.11E-01	-7.05	11.70	5.47E-01
cg04658021	6.28	3.00	3.65E-02	7.17	3.69	5.18E-02	11.30	7.10	1.11E-01	1.80	13.21	8.92E-01	-5.45	9.16	5.52E-01
cg09899220	8.23	3.94	3.65E-02	7.51	5.04	1.37E-01	18.74	9.97	6.01E-02	7.01	14.20	6.22E-01	1.25	9.90	8.99E-01
ch.15.811911R	-26.10	12.48	3.65E-02	-20.42	16.09	2.04E-01	-70.62	46.62	1.30E-01	-38.08	55.48	4.92E-01	-24.74	23.79	2.98E-01
cg02791338	-28.59	13.67	3.65E-02	-37.40	18.86	4.74E-02	-83.16	76.23	2.75E-01	-27.83	30.74	3.65E-01	-3.07	27.66	9.12E-01
cg02973680	20.81	9.95	3.65E-02	34.83	21.06	9.82E-02	48.18	45.75	2.92E-01	14.58	12.81	2.55E-01	15.52	28.09	5.81E-01
cg26228591	19.73	9.43	3.65E-02	23.25	18.49	2.09E-01	21.21	16.44	1.97E-01	8.11	18.82	6.67E-01	29.24	23.65	2.16E-01
cg19082496	4.74	2.27	3.65E-02	5.15	3.33	1.22E-01	6.53	5.32	2.19E-01	3.49	4.43	4.30E-01	2.68	7.44	7.19E-01
cg21117373	-27.17	12.99	3.65E-02	-43.57	40.31	2.80E-01	-121.97	177.94	4.93E-01	-24.17	14.45	9.44E-02	-29.80	45.37	5.11E-01
cg25156443	18.50	8.85	3.65E-02	17.40	14.86	2.42E-01	92.22	53.84	8.67E-02	9.43	8.13	2.46E-01	37.58	19.49	5.38E-02

cg16779811	-10.41	4.98	3.65E-02	-16.46	7.01	1.89E-02	-1.76	11.35	8.77E-01	-17.72	17.35	3.07E-01	-1.44	10.58	8.92E-01
cg01015395	-6.65	3.18	3.65E-02	-13.07	7.30	7.35E-02	-12.83	10.01	2.00E-01	-3.41	4.11	4.07E-01	-7.50	9.53	4.31E-01
cg09313140	-8.29	3.96	3.65E-02	-10.09	7.20	1.61E-01	-5.73	10.22	5.75E-01	-8.45	6.45	1.90E-01	-6.96	9.62	4.70E-01
cg15275313	9.63	4.60	3.65E-02	4.72	6.32	4.55E-01	17.93	10.32	8.24E-02	6.86	18.27	7.07E-01	15.05	10.11	1.37E-01
cg13296238	12.84	6.14	3.66E-02	17.88	8.51	3.56E-02	6.57	14.65	6.54E-01	11.60	17.16	4.99E-01	5.04	14.70	7.32E-01
cg03767333	12.23	5.85	3.66E-02	4.52	11.98	7.06E-01	21.87	18.10	2.27E-01	15.77	7.78	4.26E-02	-0.51	19.27	9.79E-01
cg05225883	6.40	3.06	3.66E-02	7.47	4.41	8.97E-02	9.03	7.98	2.58E-01	-0.85	7.85	9.14E-01	7.33	6.58	2.65E-01
cg19453154	22.08	10.56	3.66E-02	10.36	11.93	3.85E-01	40.45	16.71	1.55E-02	50.22	23.48	3.24E-02	2.36	17.18	8.91E-01
cg17182808	13.85	6.63	3.66E-02	16.79	17.09	3.26E-01	24.68	29.51	4.03E-01	9.71	7.70	2.07E-01	48.78	27.18	7.27E-02
cg02126477	-13.57	6.49	3.66E-02	-1.73	20.81	9.34E-01	-4.50	36.92	9.03E-01	-15.46	7.16	3.10E-02	-11.29	28.75	6.95E-01
cg17813225	-9.28	4.44	3.66E-02	-16.00	10.99	1.45E-01	9.78	21.92	6.55E-01	-7.37	5.32	1.65E-01	-19.54	14.13	1.67E-01
cg05040219	14.89	7.12	3.66E-02	36.53	15.18	1.61E-02	-1.52	30.34	9.60E-01	7.82	8.71	3.70E-01	20.96	21.65	3.33E-01
cg08401990	-28.42	13.60	3.66E-02	-8.64	53.36	8.71E-01	-17.23	210.01	9.35E-01	-31.47	14.84	3.40E-02	-15.04	44.88	7.38E-01
cg07179033	6.32	3.02	3.66E-02	4.35	4.05	2.83E-01	15.89	7.15	2.62E-02	7.81	8.33	3.49E-01	0.22	8.30	9.79E-01
cg17393917	14.12	6.75	3.66E-02	23.26	10.13	2.17E-02	-10.82	18.20	5.52E-01	24.66	17.63	1.62E-01	9.09	10.83	4.01E-01
cg13654525	7.35	3.52	3.66E-02	5.74	4.88	2.39E-01	4.92	7.91	5.34E-01	21.07	14.77	1.54E-01	9.71	7.38	1.89E-01
cg04940764	-7.14	3.41	3.66E-02	-6.42	5.26	2.22E-01	-6.87	7.23	3.42E-01	-9.71	10.92	3.74E-01	-7.56	6.72	2.61E-01
cg05453631	14.57	6.97	3.66E-02	11.69	9.46	2.16E-01	12.73	19.42	5.12E-01	18.54	14.20	1.92E-01	24.25	23.60	3.04E-01
cg25432792	-7.01	3.35	3.66E-02	-10.81	6.18	8.04E-02	3.01	12.33	8.07E-01	-5.46	4.76	2.52E-01	-9.84	9.04	2.77E-01
cg25830696	19.16	9.16	3.66E-02	-5.60	34.69	8.72E-01	17.06	168.02	9.19E-01	22.38	9.72	2.13E-02	-10.81	47.14	8.19E-01
cg07251193	24.00	11.48	3.66E-02	8.87	29.71	7.65E-01	42.72	60.73	4.82E-01	18.75	13.60	1.68E-01	75.83	35.80	3.42E-02
cg05219493	7.04	3.37	3.66E-02	2.43	4.85	6.16E-01	10.13	7.61	1.83E-01	12.39	8.62	1.51E-01	11.71	8.16	1.52E-01
cg22112267	19.39	9.27	3.66E-02	31.45	13.82	2.29E-02	18.91	26.98	4.83E-01	26.90	23.01	2.42E-01	-5.12	17.88	7.75E-01
cg18207699	19.86	9.50	3.66E-02	17.47	16.74	2.97E-01	19.76	17.25	2.52E-01	4.38	21.62	8.40E-01	40.74	22.31	6.78E-02
cg09540612	5.59	2.67	3.66E-02	5.22	3.28	1.11E-01	11.36	6.44	7.76E-02	-0.70	11.45	9.51E-01	1.79	8.19	8.27E-01
cg07243700	26.79	12.82	3.66E-02	30.72	14.82	3.81E-02	16.59	88.73	8.52E-01	23.29	64.37	7.17E-01	13.25	29.34	6.51E-01
cg02770745	4.20	2.01	3.66E-02	4.40	2.78	1.13E-01	7.14	4.66	1.25E-01	0.82	4.45	8.53E-01	4.63	6.86	5.00E-01
cg20436458	-8.87	4.24	3.66E-02	-5.90	10.25	5.65E-01	1.94	17.85	9.13E-01	-9.55	5.13	6.28E-02	-16.25	14.25	2.54E-01
cg21404045	14.53	6.95	3.66E-02	14.39	16.80	3.92E-01	31.99	38.15	4.02E-01	14.74	8.24	7.35E-02	6.09	24.03	8.00E-01
cg26360953	-20.64	9.87	3.66E-02	-15.91	19.12	4.05E-01	-7.79	43.53	8.58E-01	-20.62	12.86	1.09E-01	-41.41	32.41	2.01E-01
cg00852573	23.77	11.38	3.66E-02	13.17	21.87	5.47E-01	27.32	54.95	6.19E-01	27.87	14.45	5.37E-02	26.42	44.07	5.49E-01
cg21646471	15.08	7.22	3.66E-02	2.89	17.23	8.67E-01	44.93	25.61	7.93E-02	17.37	9.20	5.90E-02	2.43	20.06	9.04E-01
cg16586200	-14.92	7.14	3.66E-02	-24.63	17.68	1.64E-01	1.65	22.54	9.42E-01	-11.19	8.95	2.11E-01	-39.70	22.66	7.98E-02
cg18031708	-13.91	6.66	3.66E-02	-10.04	10.78	3.52E-01	-19.14	15.53	2.18E-01	-19.87	17.34	2.52E-01	-12.65	12.40	3.08E-01
cg07703790	-13.02	6.23	3.66E-02	-30.83	12.87	1.66E-02	-13.28	16.91	4.32E-01	-4.36	7.69	5.71E-01	-18.32	16.88	2.78E-01

cg18357736	13.73	6.57	3.66E-02	14.51	8.61	9.17E-02	31.93	16.28	4.98E-02	11.42	21.88	6.02E-01	-5.91	16.25	7.16E-01
cg10698355	19.55	9.35	3.66E-02	17.57	11.86	1.39E-01	-13.33	42.27	7.53E-01	47.20	27.65	8.79E-02	18.04	20.18	3.71E-01
cg01796225	-9.09	4.35	3.66E-02	-19.48	7.45	8.87E-03	7.11	12.96	5.83E-01	-6.24	4.10	1.29E-01	-12.16	10.09	2.28E-01
cg21044572	8.00	3.83	3.66E-02	10.97	6.31	8.23E-02	4.71	6.95	4.98E-01	9.14	9.06	3.13E-01	6.03	9.86	5.41E-01
cg00160818	14.59	6.98	3.66E-02	19.01	13.51	1.59E-01	-3.58	16.20	8.25E-01	19.62	12.11	1.05E-01	17.04	15.09	2.59E-01
cg02569718	-6.46	3.09	3.67E-02	-5.76	3.97	1.47E-01	-6.50	7.44	3.82E-01	0.11	10.80	9.92E-01	-13.24	8.24	1.08E-01
cg13454331	-19.06	9.12	3.67E-02	-21.07	25.26	4.04E-01	-58.85	109.68	5.92E-01	-20.71	10.20	4.24E-02	10.01	36.12	7.82E-01
cg27561099	-8.85	4.23	3.67E-02	-12.88	6.68	5.38E-02	4.85	10.38	6.40E-01	-6.04	5.96	3.11E-01	-20.01	9.86	4.25E-02
cg26428830	-9.03	4.32	3.67E-02	-15.92	7.22	2.73E-02	-12.30	9.38	1.90E-01	-3.69	10.19	7.18E-01	-0.20	8.64	9.82E-01
cg00938658	-6.84	3.27	3.67E-02	-7.71	4.72	1.03E-01	-8.60	7.35	2.42E-01	-12.99	10.93	2.35E-01	-1.17	6.78	8.63E-01
cg15888097	-11.47	5.49	3.67E-02	-7.79	7.86	3.22E-01	-2.36	14.97	8.75E-01	-14.74	13.16	2.63E-01	-23.54	12.17	5.32E-02
cg09427718	-14.16	6.78	3.67E-02	-17.82	13.53	1.88E-01	-13.12	26.78	6.24E-01	-12.21	8.90	1.70E-01	-16.83	20.92	4.21E-01
cg06961354	-11.06	5.29	3.67E-02	-18.10	11.20	1.06E-01	1.57	14.28	9.12E-01	-9.78	7.46	1.90E-01	-17.01	14.37	2.37E-01
cg23724016	17.60	8.42	3.67E-02	19.89	16.47	2.27E-01	22.66	13.96	1.05E-01	27.59	17.99	1.25E-01	-12.15	21.36	5.69E-01
cg19573599	-15.03	7.20	3.67E-02	-19.61	13.99	1.61E-01	-1.42	30.64	9.63E-01	-18.15	9.56	5.76E-02	4.49	21.30	8.33E-01
cg21915970	-23.17	11.09	3.67E-02	1.65	35.69	9.63E-01	43.54	152.01	7.75E-01	-21.94	12.14	7.08E-02	-82.33	43.87	6.06E-02
cg26470719	17.64	8.45	3.67E-02	29.10	16.80	8.33E-02	25.67	22.02	2.44E-01	6.25	12.48	6.17E-01	25.67	22.38	2.51E-01
cg26506790	-11.02	5.28	3.67E-02	-11.15	6.79	1.01E-01	-15.52	11.19	1.65E-01	-2.81	23.66	9.06E-01	-5.66	14.94	7.05E-01
cg19569526	-6.05	2.90	3.67E-02	-6.01	4.31	1.63E-01	-0.79	6.82	9.07E-01	-8.58	7.55	2.56E-01	-8.74	6.16	1.56E-01
cg01337429	-38.88	18.61	3.67E-02	-41.55	23.93	8.25E-02	30.52	62.22	6.24E-01	-98.18	53.35	6.57E-02	-24.60	43.38	5.71E-01
cg09147024	-9.08	4.34	3.67E-02	-6.67	6.37	2.95E-01	-13.87	9.92	1.62E-01	-14.09	11.92	2.37E-01	-6.87	9.46	4.68E-01
cg18323095	-10.42	4.99	3.67E-02	0.02	11.23	9.98E-01	9.16	24.45	7.08E-01	-13.23	6.55	4.33E-02	-17.25	11.71	1.41E-01
cg16303353	7.06	3.38	3.67E-02	6.49	4.38	1.38E-01	15.89	9.53	9.56E-02	7.80	9.18	3.96E-01	0.98	8.92	9.13E-01
cg02927682	5.58	2.67	3.67E-02	4.96	3.54	1.61E-01	10.21	5.42	5.97E-02	-1.57	11.03	8.87E-01	2.86	7.50	7.03E-01
cg10824354	-10.33	4.95	3.67E-02	-16.93	6.66	1.11E-02	-10.19	11.16	3.61E-01	8.53	15.39	5.79E-01	-1.46	11.81	9.02E-01
cg07259660	-14.30	6.84	3.67E-02	-17.71	20.06	3.77E-01	17.35	27.01	5.21E-01	-15.46	7.85	4.90E-02	-26.91	28.02	3.37E-01
cg21663536	20.40	9.77	3.67E-02	19.62	17.76	2.69E-01	5.00	17.75	7.78E-01	29.20	20.63	1.57E-01	37.52	23.62	1.12E-01
cg11029270	-12.83	6.14	3.67E-02	-25.72	18.18	1.57E-01	18.68	38.95	6.32E-01	-12.95	6.80	5.68E-02	4.88	29.18	8.67E-01
cg11617975	34.13	16.34	3.67E-02	28.68	20.70	1.66E-01	-7.08	83.56	9.32E-01	45.64	71.32	5.22E-01	49.43	30.57	1.06E-01
cg05122437	-2.40	1.15	3.67E-02	-1.91	1.80	2.89E-01	-2.62	2.56	3.04E-01	-3.42	2.36	1.48E-01	-1.86	2.97	5.31E-01
cg14630839	-25.67	12.29	3.67E-02	-34.17	40.10	3.94E-01	-103.79	215.93	6.31E-01	-20.33	13.42	1.30E-01	-79.55	48.68	1.02E-01
cg15353603	-7.74	3.71	3.67E-02	-7.41	7.03	2.92E-01	-14.60	10.55	1.67E-01	-10.44	5.91	7.75E-02	1.08	8.18	8.95E-01
cg23481246	9.00	4.31	3.67E-02	9.59	5.96	1.08E-01	23.46	12.18	5.41E-02	5.65	9.22	5.40E-01	-1.35	11.77	9.09E-01
cg19026320	-9.43	4.52	3.67E-02	-12.54	9.85	2.03E-01	-7.41	16.84	6.60E-01	-11.59	5.70	4.19E-02	11.50	15.14	4.47E-01
cg26982447	13.91	6.66	3.67E-02	14.54	14.58	3.19E-01	6.56	30.64	8.30E-01	12.77	8.63	1.39E-01	19.95	17.28	2.48E-01

cg05195050	-11.84	5.67	3.67E-02	-30.04	13.42	2.52E-02	6.14	17.46	7.25E-01	-8.79	6.73	1.92E-01	-15.94	15.08	2.91E-01
cg06652523	14.30	6.85	3.67E-02	5.71	11.21	6.11E-01	8.22	14.41	5.68E-01	24.88	12.79	5.18E-02	27.89	20.27	1.69E-01
cg06299307	11.46	5.49	3.67E-02	14.17	15.74	3.68E-01	16.45	68.18	8.09E-01	10.41	6.31	9.91E-02	15.23	16.14	3.45E-01
cg26546664	13.82	6.61	3.67E-02	25.02	10.15	1.37E-02	2.67	12.28	8.28E-01	20.03	20.26	3.23E-01	1.54	15.67	9.22E-01
cg20540372	-11.59	5.55	3.67E-02	-24.06	15.95	1.31E-01	-17.92	23.66	4.49E-01	-11.90	6.35	6.12E-02	22.29	22.30	3.18E-01
cg13480197	6.18	2.96	3.68E-02	4.54	3.42	1.84E-01	15.72	7.88	4.61E-02	11.50	14.71	4.34E-01	1.40	11.28	9.01E-01
cg26737458	-47.07	22.54	3.68E-02	-33.11	61.83	5.92E-01	-57.85	227.68	7.99E-01	-47.19	25.80	6.73E-02	-64.65	73.48	3.79E-01
cg11795488	-10.47	5.01	3.68E-02	-17.08	7.34	2.00E-02	-9.05	11.96	4.49E-01	6.98	14.55	6.31E-01	-7.29	10.24	4.77E-01
cg09753778	-12.14	5.81	3.68E-02	-6.30	19.03	7.41E-01	-12.09	38.68	7.55E-01	-13.38	6.30	3.37E-02	3.58	32.26	9.12E-01
cg09571972	5.39	2.58	3.68E-02	4.11	3.45	2.34E-01	8.00	5.66	1.57E-01	8.67	8.99	3.35E-01	4.73	6.67	4.78E-01
cg01021488	-16.59	7.95	3.68E-02	-21.28	12.20	8.12E-02	-19.49	18.28	2.86E-01	-25.26	20.00	2.07E-01	0.42	16.59	9.80E-01
cg26823505	-8.85	4.24	3.68E-02	-7.17	6.30	2.55E-01	-12.22	9.30	1.89E-01	-7.90	11.31	4.85E-01	-9.80	9.48	3.01E-01
cg15296767	7.04	3.37	3.68E-02	8.85	4.36	4.24E-02	10.60	7.43	1.54E-01	-0.83	12.49	9.47E-01	-3.04	9.61	7.52E-01
cg05337387	-7.95	3.80	3.68E-02	-16.46	8.26	4.61E-02	-0.60	12.20	9.61E-01	-5.53	5.13	2.81E-01	-9.62	10.16	3.44E-01
cg21960859	27.81	13.32	3.68E-02	42.83	15.68	6.30E-03	-9.70	35.86	7.87E-01	59.25	33.44	7.64E-02	7.69	20.19	7.03E-01
cg14932715	-12.03	5.76	3.68E-02	-16.25	15.26	2.87E-01	-1.05	24.04	9.65E-01	-8.07	6.90	2.42E-01	-39.25	17.99	2.91E-02
cg21588838	-9.38	4.49	3.68E-02	-24.96	10.69	1.96E-02	-15.55	18.62	4.04E-01	-4.50	5.16	3.83E-01	-10.65	12.47	3.93E-01
cg01958008	-8.56	4.10	3.68E-02	1.22	9.53	8.98E-01	-5.01	14.62	7.32E-01	-9.59	5.00	5.51E-02	-30.21	16.16	6.15E-02
cg11309496	14.93	7.15	3.68E-02	38.49	19.65	5.01E-02	34.57	54.88	5.29E-01	9.35	8.38	2.65E-01	19.91	20.43	3.30E-01
cg17091312	-13.52	6.47	3.68E-02	-8.14	17.66	6.45E-01	-21.81	22.64	3.35E-01	-13.51	7.75	8.14E-02	-14.10	22.02	5.22E-01
cg02410887	20.95	10.03	3.68E-02	23.10	13.85	9.53E-02	21.10	32.43	5.15E-01	55.37	23.94	2.08E-02	3.15	14.87	8.32E-01
cg15083775	-17.47	8.37	3.68E-02	3.37	18.30	8.54E-01	-14.63	19.78	4.60E-01	-22.05	12.16	6.99E-02	-37.02	22.50	9.99E-02
cg03193472	8.57	4.10	3.68E-02	7.25	5.98	2.25E-01	9.17	8.46	2.79E-01	13.01	12.49	2.98E-01	8.60	9.55	3.68E-01
cg05314679	27.62	13.23	3.68E-02	21.30	22.47	3.43E-01	10.36	60.39	8.64E-01	40.67	36.07	2.59E-01	30.31	19.28	1.16E-01
cg11159650	29.81	14.28	3.68E-02	55.57	40.02	1.65E-01	-79.22	159.38	6.19E-01	25.85	16.12	1.09E-01	38.57	50.45	4.45E-01
cg04363470	-8.63	4.13	3.68E-02	-10.21	6.01	8.92E-02	-5.03	9.41	5.93E-01	-12.73	15.68	4.17E-01	-7.34	8.03	3.61E-01
cg18440523	-12.20	5.84	3.68E-02	-8.29	11.54	4.72E-01	-25.09	16.70	1.33E-01	-8.53	8.84	3.35E-01	-17.74	13.59	1.92E-01
cg16524818	-8.54	4.09	3.68E-02	-15.04	8.36	7.21E-02	-0.62	15.92	9.69E-01	-5.63	5.53	3.09E-01	-12.34	10.65	2.47E-01
cg14977433	-11.05	5.29	3.68E-02	-15.21	7.37	3.90E-02	-26.74	10.47	1.06E-02	-7.66	5.35	1.53E-01	5.36	11.11	6.30E-01
cg13967908	-5.01	2.40	3.68E-02	-1.97	4.22	6.41E-01	-7.57	5.94	2.02E-01	-5.97	3.71	1.07E-01	-6.80	7.88	3.88E-01
cg02108850	14.64	7.01	3.68E-02	39.37	24.77	1.12E-01	101.85	103.08	3.23E-01	11.80	7.76	1.28E-01	13.99	22.43	5.33E-01
cg20458244	27.26	13.06	3.68E-02	34.63	21.97	1.15E-01	39.78	23.90	9.60E-02	6.89	8.94	4.41E-01	67.22	31.95	3.54E-02
cg09415366	-9.13	4.37	3.68E-02	-11.14	5.74	5.23E-02	-11.96	10.81	2.69E-01	-0.83	18.34	9.64E-01	-3.32	9.78	7.34E-01
cg24603408	15.05	7.21	3.68E-02	8.17	15.12	5.89E-01	50.99	44.11	2.48E-01	17.72	8.70	4.17E-02	-5.53	29.41	8.51E-01
cg11792824	-11.20	5.37	3.68E-02	-21.96	13.51	1.04E-01	-5.53	29.57	8.52E-01	-11.22	6.26	7.30E-02	9.52	19.81	6.31E-01

cg09740554	-16.54	7.92	3.68E-02	-26.30	11.53	2.25E-02	-10.24	23.72	6.66E-01	17.84	34.60	6.06E-01	-10.75	13.14	4.13E-01
cg19009471	-8.37	4.01	3.68E-02	-6.77	5.45	2.14E-01	-4.25	9.24	6.46E-01	-5.08	12.35	6.81E-01	-20.49	9.90	3.84E-02
cg10755085	10.00	4.79	3.68E-02	13.36	6.63	4.40E-02	12.86	11.16	2.49E-01	8.10	12.78	5.26E-01	-3.04	12.18	8.03E-01
cg25700339	13.98	6.69	3.68E-02	14.53	9.63	1.31E-01	9.96	14.48	4.91E-01	7.83	17.42	6.53E-01	23.63	17.00	1.64E-01
cg02023717	-12.16	5.83	3.69E-02	-33.60	18.11	6.34E-02	1.82	36.72	9.61E-01	-8.92	6.52	1.71E-01	-22.13	21.69	3.08E-01
cg25478600	8.09	3.88	3.69E-02	9.63	6.88	1.62E-01	23.52	11.67	4.38E-02	7.98	5.61	1.55E-01	-2.06	8.35	8.05E-01
cg01794563	-34.49	16.52	3.69E-02	-43.70	39.72	2.71E-01	-150.28	110.57	1.74E-01	-25.63	19.42	1.87E-01	-62.11	58.10	2.85E-01
cg06098530	9.68	4.64	3.69E-02	16.42	6.74	1.49E-02	5.55	15.76	7.24E-01	5.32	16.54	7.48E-01	2.80	7.71	7.16E-01
cg21775570	7.75	3.71	3.69E-02	6.71	4.80	1.62E-01	18.83	10.52	7.35E-02	11.35	10.97	3.01E-01	0.54	9.23	9.53E-01
cg01616394	12.77	6.12	3.69E-02	7.18	10.88	5.09E-01	23.48	15.95	1.41E-01	7.97	10.85	4.63E-01	20.60	13.07	1.15E-01
cg12529083	33.23	15.92	3.69E-02	-31.14	49.93	5.33E-01	-46.89	179.03	7.93E-01	38.57	18.08	3.29E-02	59.62	46.88	2.03E-01
cg08970112	9.08	4.35	3.69E-02	14.07	10.23	1.69E-01	18.57	17.24	2.81E-01	7.90	5.42	1.45E-01	2.37	13.07	8.56E-01
cg23626122	9.90	4.75	3.69E-02	12.68	7.08	7.33E-02	8.74	11.93	4.64E-01	19.45	14.82	1.89E-01	2.86	8.82	7.45E-01
cg01852522	-9.21	4.41	3.69E-02	-14.16	7.12	4.67E-02	9.26	12.41	4.56E-01	-13.52	9.46	1.53E-01	-7.33	8.45	3.86E-01
cg12881470	-49.61	23.77	3.69E-02	-15.66	18.69	4.02E-01	-55.07	51.31	2.83E-01	-106.58	72.69	1.43E-01	-95.45	40.11	1.73E-02
cg24308847	12.14	5.82	3.69E-02	5.65	8.27	4.95E-01	13.36	14.09	3.43E-01	36.81	15.83	2.00E-02	10.25	11.97	3.92E-01
cg18362448	7.20	3.45	3.69E-02	7.54	4.72	1.10E-01	-0.40	7.86	9.59E-01	24.13	11.70	3.92E-02	6.28	7.47	4.00E-01
cg15646741	16.48	7.90	3.69E-02	13.35	9.55	1.62E-01	42.85	17.57	1.47E-02	-9.52	28.73	7.40E-01	10.30	16.07	5.21E-01
cg20168554	17.43	8.35	3.69E-02	19.68	16.91	2.45E-01	1.14	32.37	9.72E-01	16.25	11.03	1.41E-01	27.84	24.46	2.55E-01
cg01934781	-8.29	3.97	3.69E-02	-16.92	6.29	7.13E-03	-3.17	7.90	6.89E-01	-0.01	8.41	1.00E+00	-7.72	8.37	3.56E-01
cg01436998	14.97	7.18	3.69E-02	14.16	12.13	2.43E-01	5.19	22.23	8.15E-01	40.28	17.63	2.23E-02	7.37	11.64	5.27E-01
cg05136804	6.15	2.95	3.69E-02	6.19	4.13	1.34E-01	8.05	6.16	1.91E-01	13.86	9.73	1.54E-01	-0.73	7.18	9.19E-01
cg07727358	-74.15	35.53	3.69E-02	-60.74	45.71	1.84E-01	-49.83	178.65	7.80E-01	-170.57	79.97	3.29E-02	-11.34	89.16	8.99E-01
cg00278359	-13.36	6.40	3.69E-02	-4.17	7.38	5.72E-01	-26.10	14.13	6.48E-02	-7.34	12.49	5.57E-01	-29.09	13.11	2.66E-02
cg19083007	-8.18	3.92	3.69E-02	-14.51	8.54	8.94E-02	-12.49	15.13	4.09E-01	-4.68	5.25	3.72E-01	-10.24	9.70	2.91E-01
cg14605274	-11.52	5.52	3.69E-02	-4.06	11.26	7.19E-01	-17.85	28.71	5.34E-01	-13.90	6.89	4.35E-02	-11.83	19.46	5.43E-01
cg21717629	9.03	4.33	3.69E-02	12.10	7.62	1.13E-01	-5.00	9.48	5.98E-01	11.23	7.26	1.22E-01	18.39	12.28	1.34E-01
cg19961043	14.05	6.74	3.69E-02	14.08	15.52	3.64E-01	21.03	15.53	1.76E-01	12.71	9.99	2.03E-01	9.88	16.39	5.46E-01
cg26671050	-12.30	5.90	3.69E-02	-11.22	13.28	3.98E-01	-22.08	23.23	3.42E-01	-14.16	7.34	5.36E-02	5.21	19.41	7.88E-01
cg19272468	7.43	3.56	3.69E-02	6.44	6.03	2.85E-01	8.80	7.11	2.16E-01	8.99	7.03	2.01E-01	4.66	9.37	6.19E-01
cg02770688	-23.04	11.04	3.69E-02	-27.79	10.29	6.90E-03	25.22	28.21	3.71E-01	-49.00	20.96	1.94E-02	-18.40	17.86	3.03E-01
cg26429674	-30.06	14.41	3.69E-02	-43.46	17.15	1.13E-02	36.57	42.08	3.85E-01	-55.35	26.73	3.84E-02	-18.05	20.67	3.83E-01
cg17787710	-23.68	11.35	3.69E-02	-22.66	16.29	1.64E-01	-24.37	30.44	4.23E-01	-38.53	31.77	2.25E-01	-17.65	22.78	4.39E-01
cg24652615	3.17	1.52	3.69E-02	1.53	2.17	4.82E-01	4.23	3.00	1.58E-01	11.60	5.69	4.15E-02	2.80	3.57	4.32E-01
cg09159285	7.97	3.82	3.69E-02	11.45	5.88	5.14E-02	6.71	8.56	4.33E-01	13.52	8.90	1.28E-01	-3.57	8.67	6.80E-01

cg04170999	15.12	7.25	3.69E-02	1.71	22.01	9.38E-01	5.77	67.64	9.32E-01	18.19	8.19	2.62E-02	6.32	23.34	7.87E-01
cg05379597	7.84	3.76	3.69E-02	7.32	4.90	1.36E-01	17.44	8.99	5.25E-02	2.73	11.61	8.14E-01	1.58	10.30	8.78E-01
cg22543026	15.63	7.49	3.69E-02	24.54	9.78	1.21E-02	-7.78	24.24	7.48E-01	3.80	39.47	9.23E-01	6.53	14.12	6.44E-01
cg10166825	-8.50	4.07	3.69E-02	-14.59	8.55	8.80E-02	-3.66	11.81	7.57E-01	-6.67	5.74	2.45E-01	-9.26	10.50	3.77E-01
cg21663666	-18.59	8.91	3.69E-02	-21.39	14.90	1.51E-01	-6.39	65.15	9.22E-01	-41.31	21.12	5.05E-02	-7.78	13.35	5.60E-01
cg07589081	34.61	16.59	3.69E-02	55.57	45.90	2.26E-01	-108.18	171.36	5.28E-01	30.03	18.69	1.08E-01	65.23	61.71	2.90E-01
cg13107669	16.78	8.04	3.69E-02	4.27	15.66	7.85E-01	23.93	17.55	1.73E-01	21.29	13.59	1.17E-01	17.99	19.15	3.48E-01
cg04078658	45.46	21.79	3.69E-02	56.01	29.96	6.16E-02	80.49	70.31	2.52E-01	15.99	44.68	7.20E-01	31.39	58.74	5.93E-01
cg26951875	25.82	12.38	3.69E-02	6.93	35.22	8.44E-01	254.40	185.52	1.70E-01	27.98	13.66	4.05E-02	16.87	54.77	7.58E-01
cg15717598	-34.40	16.49	3.69E-02	-33.14	21.35	1.21E-01	-97.49	69.03	1.58E-01	-26.63	38.83	4.93E-01	-25.69	40.40	5.25E-01
cg18879793	-43.47	20.84	3.69E-02	-41.42	26.32	1.16E-01	-112.40	60.52	6.33E-02	19.45	64.75	7.64E-01	-41.04	53.58	4.44E-01
cg08971667	12.25	5.87	3.70E-02	14.10	8.49	9.68E-02	28.41	14.46	4.95E-02	8.78	15.01	5.58E-01	-2.54	13.00	8.45E-01
cg16000683	-11.63	5.57	3.70E-02	-16.04	9.25	8.28E-02	-10.01	13.13	4.46E-01	-10.29	11.02	3.50E-01	-6.80	12.45	5.85E-01
cg02048651	20.81	9.97	3.70E-02	32.61	18.71	8.12E-02	18.51	18.74	3.23E-01	1.34	18.27	9.42E-01	43.82	27.19	1.07E-01
cg08777490	-27.82	13.33	3.70E-02	-28.06	17.30	1.05E-01	8.21	42.63	8.47E-01	-47.87	75.47	5.26E-01	-37.80	25.36	1.36E-01
cg11586890	17.28	8.28	3.70E-02	19.27	13.10	1.41E-01	20.27	15.79	1.99E-01	5.83	19.36	7.63E-01	20.65	22.01	3.48E-01
cg13451548	14.09	6.75	3.70E-02	17.35	13.59	2.02E-01	22.04	27.56	4.24E-01	16.39	9.94	9.90E-02	3.93	14.05	7.80E-01
cg21195176	33.72	16.16	3.70E-02	39.05	21.21	6.56E-02	68.92	30.83	2.54E-02	-28.90	42.47	4.96E-01	24.67	30.98	4.26E-01
cg23675837	23.20	11.12	3.70E-02	44.67	19.64	2.29E-02	10.13	22.94	6.59E-01	4.20	21.80	8.47E-01	29.39	25.96	2.58E-01
cg10233953	-11.91	5.71	3.70E-02	-19.81	10.72	6.47E-02	-17.82	13.03	1.72E-01	-6.94	8.85	4.33E-01	0.11	17.29	9.95E-01
cg16250355	-29.10	13.95	3.70E-02	-28.04	17.13	1.02E-01	-29.74	48.17	5.37E-01	10.49	48.43	8.29E-01	-52.16	33.79	1.23E-01
cg04669895	-10.99	5.27	3.70E-02	-19.67	15.99	2.19E-01	-2.66	26.94	9.21E-01	-10.93	5.97	6.70E-02	-3.17	19.38	8.70E-01
cg19242459	19.81	9.50	3.70E-02	3.78	20.97	8.57E-01	34.05	21.52	1.14E-01	17.16	13.93	2.18E-01	32.75	25.81	2.05E-01
cg09736083	26.47	12.69	3.70E-02	37.43	17.07	2.83E-02	-14.56	30.11	6.29E-01	6.47	33.61	8.47E-01	46.69	26.11	7.38E-02
cg02504465	-49.16	23.57	3.70E-02	-119.63	55.76	3.19E-02	15.27	206.21	9.41E-01	-46.98	25.54	6.58E-02	30.50	66.57	6.47E-01
cg10389812	-6.49	3.11	3.70E-02	-3.82	6.28	5.44E-01	-5.91	5.37	2.71E-01	-10.28	5.98	8.57E-02	-5.30	8.05	5.11E-01
cg15215077	17.44	8.36	3.70E-02	37.57	16.41	2.20E-02	2.84	22.24	8.99E-01	9.10	8.81	3.02E-01	40.09	26.61	1.32E-01
cg26372254	17.47	8.37	3.70E-02	17.86	15.47	2.48E-01	-10.87	32.95	7.42E-01	16.57	12.06	1.69E-01	30.87	20.92	1.40E-01
cg25268422	-13.00	6.23	3.70E-02	-16.81	9.51	7.70E-02	-15.55	13.82	2.61E-01	-11.30	14.80	4.45E-01	-3.21	14.31	8.23E-01
cg18447402	-15.54	7.45	3.70E-02	-30.31	11.14	6.52E-03	-22.84	26.95	3.97E-01	-4.78	5.03	3.42E-01	-19.91	14.02	1.55E-01
cg23887102	11.96	5.73	3.70E-02	-4.53	12.60	7.19E-01	7.79	15.66	6.19E-01	17.29	7.49	2.10E-02	23.62	21.18	2.65E-01
cg22524061	8.29	3.98	3.70E-02	16.25	8.56	5.77E-02	5.35	13.64	6.95E-01	5.96	5.91	3.13E-01	6.63	7.99	4.07E-01
cg18491744	-11.68	5.60	3.70E-02	0.96	13.88	9.45E-01	-24.21	19.82	2.22E-01	-15.59	5.31	3.31E-03	14.21	20.77	4.94E-01
cg11270042	16.06	7.70	3.70E-02	52.15	24.22	3.13E-02	46.62	67.26	4.88E-01	10.83	8.69	2.13E-01	16.65	24.28	4.93E-01
cg26629730	11.52	5.52	3.70E-02	4.51	8.47	5.95E-01	8.11	11.46	4.79E-01	24.27	16.43	1.40E-01	21.63	11.51	6.03E-02

cg04958658	15.73	7.54	3.70E-02	38.98	19.24	4.27E-02	11.21	21.54	6.03E-01	15.13	9.56	1.14E-01	-10.27	23.64	6.64E-01
cg00408678	14.01	6.72	3.70E-02	22.88	11.57	4.81E-02	29.12	13.28	2.83E-02	3.67	5.02	4.64E-01	14.68	15.90	3.56E-01
cg27160578	-11.39	5.46	3.70E-02	-12.38	7.74	1.10E-01	4.33	14.42	7.64E-01	-14.99	16.78	3.72E-01	-16.87	10.88	1.21E-01
cg09242721	15.60	7.48	3.70E-02	11.79	13.48	3.82E-01	-2.67	18.45	8.85E-01	31.65	12.92	1.43E-02	9.37	17.06	5.83E-01
cg12836863	6.05	2.90	3.70E-02	5.74	4.14	1.66E-01	7.91	6.18	2.01E-01	12.65	7.66	9.88E-02	-2.16	7.57	7.75E-01
cg17895289	-31.52	15.11	3.70E-02	-45.10	41.66	2.79E-01	-119.00	231.97	6.08E-01	-31.41	17.04	6.53E-02	-4.68	54.38	9.31E-01
cg13698996	28.38	13.61	3.70E-02	32.99	52.43	5.29E-01	-116.67	192.03	5.43E-01	25.07	16.18	1.21E-01	40.92	29.00	1.58E-01
cg00259279	22.00	10.55	3.70E-02	53.55	18.37	3.55E-03	0.77	18.66	9.67E-01	14.77	10.34	1.53E-01	25.95	24.31	2.86E-01
cg02687288	-11.67	5.60	3.70E-02	-6.19	9.57	5.18E-01	-23.46	12.91	6.93E-02	-1.85	13.06	8.87E-01	-16.80	10.46	1.08E-01
cg11728578	30.01	14.39	3.70E-02	18.77	22.37	4.01E-01	65.93	92.39	4.75E-01	5.23	47.01	9.11E-01	43.05	21.03	4.07E-02
cg19734536	-18.96	9.09	3.70E-02	-15.09	11.68	1.96E-01	-34.36	20.80	9.85E-02	16.63	42.34	6.94E-01	-25.69	23.01	2.64E-01
cg15552370	-12.26	5.88	3.70E-02	-15.10	11.18	1.77E-01	-20.54	15.98	1.99E-01	-16.85	9.80	8.56E-02	3.29	12.29	7.89E-01
cg16288579	-43.19	20.71	3.70E-02	-52.41	25.14	3.70E-02	-19.19	87.39	8.26E-01	-100.06	46.05	2.98E-02	3.32	35.09	9.25E-01
cg08273672	-13.82	6.63	3.70E-02	-21.40	9.82	2.94E-02	-4.27	15.67	7.85E-01	-4.29	18.65	8.18E-01	-11.57	13.53	3.92E-01
cg21910663	25.16	12.06	3.70E-02	22.45	16.23	1.66E-01	69.60	44.53	1.18E-01	-15.67	44.42	7.24E-01	29.31	22.03	1.83E-01
cg18555289	20.46	9.81	3.70E-02	32.03	19.20	9.54E-02	20.90	17.40	2.30E-01	7.68	18.83	6.83E-01	22.55	25.38	3.74E-01
cg04195807	20.86	10.00	3.70E-02	22.27	20.02	2.66E-01	34.36	22.05	1.19E-01	2.99	15.79	8.50E-01	49.12	26.45	6.33E-02
cg12019717	21.79	10.45	3.71E-02	25.04	10.97	2.25E-02	16.15	115.06	8.88E-01	9.38	66.36	8.88E-01	-21.81	42.84	6.11E-01
cg00797346	26.71	12.81	3.71E-02	31.69	16.52	5.50E-02	-8.67	42.34	8.38E-01	98.91	59.38	9.58E-02	14.74	25.10	5.57E-01
cg06428797	-9.65	4.63	3.71E-02	-9.35	10.41	3.69E-01	1.37	13.16	9.17E-01	-9.92	6.39	1.21E-01	-17.96	11.78	1.27E-01
cg18996274	-12.22	5.86	3.71E-02	-20.64	11.59	7.51E-02	-8.60	13.84	5.34E-01	-11.54	9.05	2.02E-01	-3.87	15.35	8.01E-01
cg08230695	31.94	15.32	3.71E-02	59.24	42.75	1.66E-01	-157.10	209.85	4.54E-01	31.42	17.37	7.05E-02	8.29	51.57	8.72E-01
cg18955704	20.10	9.64	3.71E-02	28.94	18.42	1.16E-01	-48.85	95.78	6.10E-01	20.23	12.47	1.05E-01	4.89	28.05	8.62E-01
cg27207796	21.93	10.52	3.71E-02	38.55	44.07	3.82E-01	-57.07	167.77	7.34E-01	19.64	11.32	8.27E-02	39.72	38.28	3.00E-01
cg20915029	-36.43	17.47	3.71E-02	-30.10	20.95	1.51E-01	26.40	85.31	7.57E-01	-45.80	59.02	4.38E-01	-71.91	41.75	8.50E-02
cg04601137	9.57	4.59	3.71E-02	13.42	6.25	3.18E-02	8.46	12.87	5.11E-01	9.33	11.65	4.23E-01	-1.12	10.90	9.18E-01
cg06561892	18.28	8.77	3.71E-02	40.20	21.19	5.79E-02	5.26	58.90	9.29E-01	13.27	10.26	1.96E-01	20.91	31.80	5.11E-01
cg08410227	-12.62	6.05	3.71E-02	-24.98	8.70	4.11E-03	-2.61	13.28	8.44E-01	-5.77	14.98	7.00E-01	-4.27	11.60	7.12E-01
cg04845144	31.46	15.09	3.71E-02	-31.70	46.31	4.94E-01	-23.84	170.55	8.89E-01	43.53	15.92	6.25E-03	-8.76	56.96	8.78E-01
cg20172563	7.94	3.81	3.71E-02	11.89	8.05	1.40E-01	5.10	10.92	6.41E-01	9.33	5.13	6.87E-02	-4.91	11.96	6.82E-01
cg06418531	-48.22	23.13	3.71E-02	-42.96	28.45	1.31E-01	-120.31	110.94	2.78E-01	39.08	103.06	7.05E-01	-67.53	46.69	1.48E-01
cg04456238	9.39	4.50	3.71E-02	16.93	6.79	1.26E-02	5.52	10.72	6.07E-01	7.23	9.97	4.68E-01	-2.86	10.64	7.88E-01
cg14265033	-8.27	3.97	3.71E-02	-11.16	8.19	1.73E-01	-14.12	16.16	3.82E-01	-5.24	5.08	3.02E-01	-17.01	12.94	1.89E-01
cg09392658	-34.34	16.47	3.71E-02	-32.91	24.78	1.84E-01	2.67	51.25	9.58E-01	-28.82	37.14	4.38E-01	-55.84	32.43	8.51E-02
cg03442425	13.48	6.47	3.71E-02	20.43	11.64	7.94E-02	17.77	15.87	2.63E-01	10.12	12.68	4.25E-01	6.02	12.56	6.32E-01

cg12786907	-19.06	9.15	3.71E-02	-13.13	23.39	5.75E-01	-9.70	29.17	7.39E-01	-23.01	11.12	3.84E-02	-7.32	34.08	8.30E-01
cg13190531	-71.40	34.25	3.71E-02	-33.87	56.29	5.47E-01	-454.67	245.26	6.38E-02	-49.36	21.74	2.32E-02	-142.45	65.98	3.08E-02
cg01308118	9.43	4.52	3.71E-02	3.46	10.11	7.32E-01	10.32	14.91	4.89E-01	11.57	5.89	4.92E-02	8.09	13.19	5.40E-01
cg23730558	-7.38	3.54	3.71E-02	-10.14	5.17	4.99E-02	-2.27	8.10	7.79E-01	-16.39	11.67	1.60E-01	-2.75	7.11	6.99E-01
cg18773129	18.66	8.95	3.71E-02	25.73	14.87	8.36E-02	30.87	41.18	4.54E-01	12.41	13.01	3.40E-01	17.10	26.15	5.13E-01
cg12827530	8.96	4.30	3.71E-02	8.87	6.67	1.84E-01	11.42	9.01	2.05E-01	8.61	10.96	4.32E-01	6.67	9.53	4.84E-01
cg23719209	-9.49	4.55	3.71E-02	-4.61	7.35	5.31E-01	-15.52	9.82	1.14E-01	-5.17	10.97	6.37E-01	-15.21	9.49	1.09E-01
cg02288684	-8.87	4.26	3.71E-02	-10.66	6.93	1.24E-01	-3.95	8.85	6.55E-01	-9.80	8.99	2.76E-01	-10.43	10.42	3.17E-01
cg06495347	14.48	6.95	3.71E-02	11.21	9.55	2.40E-01	28.03	18.75	1.35E-01	27.03	20.39	1.85E-01	7.18	14.92	6.30E-01
cg14754555	5.60	2.68	3.71E-02	5.21	4.07	2.01E-01	5.06	6.41	4.30E-01	9.47	5.31	7.46E-02	0.19	7.32	9.80E-01
cg26425305	15.42	7.40	3.71E-02	26.68	11.73	2.30E-02	12.60	18.14	4.87E-01	10.80	15.49	4.85E-01	1.21	16.23	9.41E-01
cg10758875	-9.24	4.44	3.71E-02	-11.91	11.38	2.95E-01	-16.38	17.01	3.35E-01	-6.56	5.37	2.21E-01	-18.98	14.25	1.83E-01
cg13728650	15.91	7.63	3.71E-02	-7.44	19.87	7.08E-01	29.56	30.34	3.30E-01	17.53	8.93	4.97E-02	39.67	31.49	2.08E-01
cg21440776	4.39	2.10	3.71E-02	3.41	2.77	2.18E-01	7.24	5.24	1.67E-01	8.93	5.54	1.07E-01	-0.33	6.17	9.58E-01
cg08978588	24.72	11.86	3.72E-02	27.11	24.59	2.70E-01	58.16	51.29	2.57E-01	13.53	15.19	3.73E-01	67.50	36.67	6.57E-02
cg04500730	25.95	12.45	3.72E-02	27.71	15.65	7.67E-02	6.68	61.07	9.13E-01	15.26	38.72	6.94E-01	29.52	26.40	2.64E-01
cg12898595	16.96	8.14	3.72E-02	6.80	20.77	7.43E-01	38.47	33.58	2.52E-01	20.78	9.64	3.11E-02	-15.47	29.74	6.03E-01
cg02466440	12.33	5.92	3.72E-02	8.53	9.52	3.70E-01	10.40	11.19	3.53E-01	29.17	16.11	7.03E-02	11.01	13.24	4.06E-01
cg03318469	6.69	3.21	3.72E-02	4.79	4.17	2.51E-01	15.74	7.75	4.23E-02	10.84	12.56	3.88E-01	2.63	7.76	7.35E-01
cg20962444	18.26	8.76	3.72E-02	14.81	17.36	3.94E-01	1.29	39.74	9.74E-01	25.11	11.34	2.69E-02	-5.53	27.66	8.41E-01
cg06444781	16.94	8.13	3.72E-02	22.98	16.41	1.61E-01	-3.25	20.78	8.76E-01	17.33	12.29	1.59E-01	25.66	20.04	2.00E-01
cg23462788	-12.29	5.90	3.72E-02	-26.52	16.09	9.94E-02	4.71	32.91	8.86E-01	-13.31	6.85	5.21E-02	10.54	19.36	5.86E-01
cg00041014	-14.47	6.94	3.72E-02	-16.15	11.44	1.58E-01	-25.96	19.01	1.72E-01	-4.11	5.10	4.20E-01	-31.88	13.55	1.87E-02
cg13858611	6.36	3.05	3.72E-02	2.80	4.39	5.23E-01	11.01	6.95	1.13E-01	20.35	12.52	1.04E-01	6.33	5.93	2.86E-01
cg21275283	18.12	8.70	3.72E-02	24.21	13.56	7.42E-02	18.76	16.97	2.69E-01	3.53	23.10	8.78E-01	14.85	20.27	4.64E-01
cg09162148	11.34	5.44	3.72E-02	5.44	8.98	5.44E-01	23.93	17.26	1.65E-01	14.55	7.77	6.12E-02	-3.78	25.99	8.84E-01
cg11304234	12.31	5.91	3.72E-02	15.00	8.16	6.58E-02	20.24	17.35	2.44E-01	18.03	23.14	4.36E-01	3.11	10.90	7.75E-01
cg25499461	-30.83	14.79	3.72E-02	-5.70	28.20	8.40E-01	-102.23	95.49	2.84E-01	-46.75	19.45	1.62E-02	2.07	42.36	9.61E-01
cg22991943	21.38	10.26	3.72E-02	4.97	19.27	7.97E-01	32.47	17.70	6.65E-02	35.17	21.10	9.56E-02	5.17	27.02	8.48E-01
cg09500317	15.85	7.60	3.72E-02	15.75	11.58	1.74E-01	6.01	30.54	8.44E-01	22.62	12.00	5.95E-02	-3.79	23.42	8.71E-01
cg14485787	19.70	9.45	3.72E-02	30.10	22.19	1.75E-01	13.58	25.96	6.01E-01	10.80	12.31	3.81E-01	63.04	30.47	3.86E-02
cg04757411	-8.87	4.26	3.72E-02	-14.00	9.32	1.33E-01	-16.65	13.02	2.01E-01	-4.74	5.74	4.09E-01	-11.60	11.59	3.17E-01
cg09797433	12.03	5.78	3.72E-02	9.94	9.78	3.10E-01	-0.63	19.18	9.74E-01	17.65	8.59	3.99E-02	5.96	17.54	7.34E-01
cg06387980	39.12	18.78	3.72E-02	24.49	43.85	5.77E-01	171.78	237.87	4.70E-01	52.92	22.33	1.78E-02	-37.16	58.38	5.24E-01
cg01039763	11.75	5.64	3.72E-02	20.97	8.47	1.33E-02	14.49	10.87	1.82E-01	12.91	7.51	8.56E-02	-9.42	12.17	4.39E-01

cg10438589	6.42	3.08	3.72E-02	5.85	4.18	1.61E-01	13.63	7.93	8.55E-02	8.15	8.19	3.20E-01	0.15	7.60	9.85E-01
cg09512322	-29.62	14.22	3.72E-02	-53.29	49.53	2.82E-01	-26.90	252.67	9.15E-01	-31.72	15.39	3.93E-02	31.73	57.64	5.82E-01
cg03942932	7.68	3.69	3.72E-02	12.61	5.96	3.43E-02	6.24	9.11	4.93E-01	3.76	7.40	6.12E-01	4.38	8.12	5.90E-01
cg12938320	-14.38	6.90	3.72E-02	-5.27	18.86	7.80E-01	-12.96	34.80	7.10E-01	-14.83	7.98	6.31E-02	-26.47	24.71	2.84E-01
cg23130849	-7.21	3.46	3.72E-02	-12.83	7.36	8.13E-02	0.96	10.68	9.29E-01	-7.16	4.72	1.29E-01	-4.58	9.33	6.24E-01
cg11406780	16.66	8.00	3.72E-02	8.78	16.60	5.97E-01	8.31	28.64	7.72E-01	14.50	10.46	1.66E-01	52.31	24.67	3.40E-02
cg03261004	49.78	23.90	3.72E-02	29.57	65.00	6.49E-01	98.71	254.43	6.98E-01	43.67	27.61	1.14E-01	114.12	73.08	1.18E-01
cg00086334	17.47	8.39	3.72E-02	7.92	15.78	6.16E-01	12.88	14.08	3.60E-01	28.93	18.23	1.12E-01	30.10	21.60	1.63E-01
cg23290121	6.30	3.02	3.72E-02	6.16	4.28	1.51E-01	2.41	7.03	7.31E-01	12.38	10.03	2.17E-01	7.34	6.35	2.48E-01
cg22834281	-13.13	6.30	3.72E-02	-5.65	13.35	6.72E-01	15.29	25.11	5.43E-01	-15.44	8.06	5.55E-02	-33.18	19.71	9.24E-02
cg14628819	27.41	13.16	3.72E-02	33.65	18.16	6.39E-02	-8.14	30.28	7.88E-01	41.03	45.75	3.70E-01	38.76	29.17	1.84E-01
cg23723194	-14.31	6.87	3.72E-02	-26.10	17.51	1.36E-01	-33.98	34.22	3.21E-01	-14.15	8.10	8.06E-02	14.65	23.42	5.31E-01
cg20841156	-30.80	14.78	3.72E-02	-35.14	17.58	4.56E-02	-10.09	62.45	8.72E-01	-24.18	55.11	6.61E-01	-22.08	36.43	5.44E-01
cg11029346	-35.54	17.06	3.72E-02	0.30	59.39	9.96E-01	-276.46	218.43	2.06E-01	-35.63	18.57	5.50E-02	-56.85	66.09	3.90E-01
cg18184935	16.93	8.13	3.73E-02	33.47	10.41	1.31E-03	12.67	15.04	3.99E-01	1.85	10.78	8.63E-01	18.46	19.72	3.49E-01
cg00249383	6.75	3.24	3.73E-02	4.73	4.38	2.81E-01	15.40	7.32	3.54E-02	13.16	10.31	2.02E-01	-0.94	8.13	9.08E-01
cg24368876	-7.18	3.45	3.73E-02	-11.60	6.22	6.20E-02	-16.14	10.03	1.07E-01	-3.23	5.12	5.29E-01	-2.01	9.87	8.39E-01
cg00102093	12.05	5.78	3.73E-02	1.61	11.90	8.92E-01	7.59	19.97	7.04E-01	13.77	7.68	7.30E-02	28.60	17.24	9.72E-02
cg15277658	17.78	8.53	3.73E-02	12.96	14.19	3.61E-01	-9.09	46.97	8.47E-01	19.96	12.44	1.09E-01	29.73	23.29	2.02E-01
cg18043104	28.65	13.75	3.73E-02	29.97	14.72	4.18E-02	61.20	128.20	6.33E-01	30.37	85.49	7.22E-01	11.13	45.95	8.09E-01
cg02773671	-16.67	8.01	3.73E-02	-18.21	18.61	3.28E-01	-10.02	22.02	6.49E-01	-17.84	10.68	9.48E-02	-16.19	23.07	4.83E-01
cg19713565	13.78	6.62	3.73E-02	34.59	21.01	9.97E-02	2.44	29.66	9.35E-01	12.14	7.41	1.01E-01	10.16	28.78	7.24E-01
cg01181350	22.99	11.04	3.73E-02	27.13	13.18	3.95E-02	40.57	57.32	4.79E-01	12.62	50.49	8.03E-01	8.65	23.89	7.17E-01
cg03613618	12.85	6.17	3.73E-02	10.64	8.67	2.20E-01	27.44	11.53	1.73E-02	16.96	11.89	1.54E-01	-5.32	12.89	6.80E-01
cg14863667	16.86	8.10	3.73E-02	13.06	11.55	2.58E-01	14.55	20.44	4.77E-01	51.29	25.30	4.26E-02	11.67	16.20	4.71E-01
cg12619400	-6.07	2.92	3.73E-02	-5.43	4.80	2.58E-01	-8.33	5.90	1.58E-01	-14.08	7.61	6.45E-02	0.11	5.94	9.86E-01
cg20335179	13.60	6.53	3.73E-02	5.12	7.65	5.04E-01	13.32	14.40	3.55E-01	17.43	20.89	4.04E-01	33.75	13.64	1.33E-02
cg21224380	4.88	2.34	3.73E-02	4.09	3.39	2.28E-01	1.46	5.83	8.02E-01	9.45	4.58	3.90E-02	2.19	7.47	7.70E-01
cg08878263	11.07	5.31	3.73E-02	8.69	7.78	2.64E-01	21.48	12.54	8.67E-02	15.19	14.40	2.91E-01	4.98	11.40	6.62E-01
cg17410660	-13.38	6.42	3.73E-02	-24.79	12.07	4.00E-02	0.41	13.58	9.76E-01	-9.10	13.09	4.87E-01	-16.87	12.79	1.87E-01
cg09060789	7.84	3.77	3.73E-02	5.44	4.70	2.47E-01	13.32	10.40	2.00E-01	5.88	12.47	6.37E-01	15.30	10.24	1.35E-01
cg24432048	-27.46	13.18	3.73E-02	-29.46	16.58	7.55E-02	-61.62	38.76	1.12E-01	-5.13	58.58	9.30E-01	-7.12	29.40	8.09E-01
cg21661815	-19.80	9.51	3.73E-02	-15.96	17.16	3.52E-01	-6.42	20.71	7.57E-01	-20.29	16.98	2.32E-01	-42.64	23.16	6.56E-02
cg23272632	20.22	9.71	3.73E-02	11.69	27.10	6.66E-01	98.40	87.44	2.60E-01	19.49	10.92	7.44E-02	30.44	36.89	4.09E-01
cg21438382	20.22	9.71	3.73E-02	13.70	12.35	2.67E-01	7.83	31.53	8.04E-01	13.49	48.69	7.82E-01	42.29	19.50	3.01E-02

cg10741308	-10.65	5.11	3.73E-02	-8.63	12.09	4.75E-01	-6.54	18.71	7.27E-01	-12.60	6.51	5.30E-02	-6.54	14.19	6.45E-01
cg10474368	-50.64	24.32	3.73E-02	-118.63	81.32	1.45E-01	-509.46	326.78	1.19E-01	-37.24	23.65	1.15E-01	-77.56	79.39	3.29E-01
cg07178968	16.46	7.90	3.73E-02	29.55	13.00	2.30E-02	15.06	13.22	2.54E-01	14.54	21.99	5.08E-01	-12.03	20.86	5.64E-01
cg23699098	14.98	7.19	3.73E-02	14.78	18.29	4.19E-01	37.31	25.27	1.40E-01	13.99	8.50	9.98E-02	-7.21	32.73	8.26E-01
cg03705299	18.28	8.78	3.73E-02	-12.78	23.33	5.84E-01	-1.87	32.10	9.53E-01	24.71	10.38	1.73E-02	37.35	33.49	2.65E-01
cg00465970	6.97	3.35	3.73E-02	5.06	4.46	2.57E-01	12.71	7.32	8.24E-02	5.29	10.11	6.00E-01	7.47	9.72	4.42E-01
cg13271430	-13.63	6.55	3.73E-02	-12.95	12.99	3.19E-01	-22.15	42.89	6.05E-01	-15.13	8.16	6.38E-02	-1.17	23.26	9.60E-01
cg11485154	12.48	5.99	3.73E-02	14.40	8.43	8.76E-02	18.76	12.68	1.39E-01	1.66	22.01	9.40E-01	4.50	13.49	7.39E-01
cg00026141	-9.98	4.79	3.73E-02	-22.70	11.09	4.06E-02	3.47	14.52	8.11E-01	-8.91	6.11	1.45E-01	-7.16	16.05	6.55E-01
cg26150621	16.44	7.89	3.73E-02	6.27	17.53	7.20E-01	9.24	29.59	7.55E-01	20.26	10.05	4.38E-02	18.41	23.93	4.42E-01
cg03885343	-4.70	2.26	3.73E-02	-5.76	3.85	1.35E-01	-6.44	4.73	1.74E-01	-3.91	4.66	4.02E-01	-1.74	5.11	7.34E-01
cg23946364	18.91	9.08	3.73E-02	23.03	16.64	1.67E-01	35.43	16.06	2.74E-02	-7.35	17.99	6.83E-01	20.25	19.59	3.01E-01
cg13153808	20.68	9.93	3.73E-02	9.92	15.46	5.21E-01	31.74	19.66	1.07E-01	-1.81	28.14	9.49E-01	41.96	21.79	5.42E-02
cg00328227	-8.94	4.29	3.73E-02	-3.98	6.32	5.29E-01	-7.45	10.69	4.86E-01	-9.46	9.47	3.18E-01	-23.20	10.41	2.58E-02
cg16034060	-16.22	7.79	3.73E-02	-15.32	13.28	2.49E-01	1.95	35.69	9.56E-01	-20.40	11.39	7.34E-02	-10.68	20.74	6.07E-01
cg19864002	18.62	8.94	3.73E-02	33.59	25.26	1.84E-01	20.53	32.22	5.24E-01	17.80	10.41	8.73E-02	-4.91	36.33	8.92E-01
cg04273661	9.14	4.39	3.73E-02	12.14	4.83	1.20E-02	-1.22	7.65	8.73E-01	27.89	12.97	3.16E-02	6.66	6.77	3.26E-01
cg09502339	12.18	5.85	3.73E-02	16.30	14.14	2.49E-01	2.87	18.43	8.76E-01	11.28	7.44	1.30E-01	19.33	17.61	2.72E-01
cg23444305	20.92	10.05	3.74E-02	17.56	11.84	1.38E-01	39.15	40.21	3.30E-01	-40.72	69.65	5.59E-01	33.97	22.66	1.34E-01
cg03685063	16.38	7.87	3.74E-02	-1.14	15.92	9.43E-01	6.19	24.99	8.04E-01	27.01	10.74	1.19E-02	12.93	22.76	5.70E-01
cg18100008	-11.64	5.59	3.74E-02	-14.84	8.63	8.57E-02	-7.80	13.23	5.56E-01	-6.09	14.40	6.73E-01	-12.36	11.15	2.68E-01
cg16599698	21.13	10.15	3.74E-02	24.67	29.53	4.04E-01	84.33	93.40	3.67E-01	20.99	11.34	6.40E-02	5.63	38.97	8.85E-01
cg01361499	4.60	2.21	3.74E-02	4.19	3.10	1.76E-01	4.76	5.09	3.50E-01	7.57	5.17	1.43E-01	1.56	6.40	8.07E-01
cg00331852	16.33	7.84	3.74E-02	17.83	13.56	1.89E-01	13.96	14.11	3.23E-01	2.47	16.11	8.78E-01	45.77	22.70	4.37E-02
cg00780909	-17.29	8.31	3.74E-02	-28.77	20.77	1.66E-01	-46.08	35.81	1.98E-01	-12.59	9.87	2.02E-01	-16.55	29.68	5.77E-01
cg06704469	-14.91	7.16	3.74E-02	-37.27	16.09	2.05E-02	-0.09	28.65	9.97E-01	-8.35	6.59	2.05E-01	-27.11	21.16	2.00E-01
cg02330706	12.72	6.11	3.74E-02	16.64	12.95	1.99E-01	17.01	13.62	2.12E-01	18.55	9.72	5.64E-02	-9.55	14.35	5.05E-01
cg03742950	26.74	12.85	3.74E-02	26.85	17.90	1.34E-01	5.20	34.30	8.79E-01	46.35	28.86	1.08E-01	20.49	33.59	5.42E-01
cg04280969	15.93	7.65	3.74E-02	8.23	8.83	3.51E-01	24.39	25.84	3.45E-01	89.78	49.46	6.95E-02	21.66	12.37	7.99E-02
cg20311846	5.86	2.81	3.74E-02	7.02	3.72	5.92E-02	6.65	6.19	2.82E-01	-18.22	15.90	2.52E-01	5.46	6.46	3.98E-01
cg10277268	30.90	14.85	3.74E-02	28.05	28.81	3.30E-01	-114.06	131.81	3.87E-01	38.22	19.95	5.54E-02	22.23	36.23	5.40E-01
cg22894329	9.50	4.57	3.74E-02	11.30	6.84	9.86E-02	8.76	10.67	4.12E-01	10.54	9.82	2.83E-01	3.79	11.57	7.43E-01
cg02183025	22.02	10.58	3.74E-02	38.81	17.22	2.43E-02	-16.84	32.91	6.09E-01	28.32	25.13	2.60E-01	11.98	18.08	5.08E-01
cg24457403	-10.58	5.08	3.74E-02	-16.71	7.76	3.14E-02	-1.38	11.65	9.06E-01	-15.17	14.94	3.10E-01	-5.28	9.88	5.93E-01
cg18486698	32.88	15.80	3.74E-02	50.83	22.45	2.36E-02	-13.13	46.23	7.76E-01	49.75	44.41	2.63E-01	11.29	30.90	7.15E-01

cg00078221	11.86	5.70	3.74E-02	26.94	13.56	4.70E-02	14.72	18.44	4.25E-01	8.40	8.21	3.06E-01	6.71	11.51	5.60E-01
cg23676151	11.69	5.62	3.74E-02	12.78	9.06	1.58E-01	-3.52	15.81	8.24E-01	31.94	17.73	7.17E-02	10.33	9.02	2.52E-01
cg14580211	7.71	3.70	3.74E-02	9.09	6.38	1.54E-01	14.98	9.53	1.16E-01	5.48	6.02	3.62E-01	2.26	10.17	8.24E-01
cg07175290	23.12	11.11	3.74E-02	27.78	37.02	4.53E-01	-112.02	165.78	4.99E-01	24.39	12.01	4.22E-02	4.90	50.04	9.22E-01
cg19645639	-7.57	3.64	3.74E-02	-5.47	5.48	3.19E-01	-11.43	8.44	1.76E-01	-10.46	9.47	2.69E-01	-6.60	7.64	3.88E-01
cg18837932	28.69	13.79	3.74E-02	-7.79	45.39	8.64E-01	70.16	204.19	7.31E-01	34.83	15.01	2.03E-02	-4.79	56.49	9.32E-01
cg08355568	40.91	19.66	3.74E-02	82.90	25.67	1.24E-03	-19.70	69.26	7.76E-01	21.20	13.32	1.11E-01	48.22	42.90	2.61E-01
cg17989313	13.42	6.45	3.74E-02	7.36	19.32	7.03E-01	-10.86	27.44	6.92E-01	16.27	7.28	2.55E-02	8.97	29.10	7.58E-01
cg21575295	18.61	8.94	3.74E-02	32.89	14.63	2.45E-02	6.05	23.32	7.95E-01	16.31	23.47	4.87E-01	9.15	15.49	5.54E-01
cg06747907	13.38	6.43	3.74E-02	12.05	10.14	2.35E-01	15.02	11.45	1.90E-01	13.45	16.29	4.09E-01	13.39	18.01	4.57E-01
cg00268009	56.78	27.29	3.74E-02	69.05	34.64	4.62E-02	141.02	131.52	2.84E-01	98.02	83.60	2.41E-01	-11.27	56.93	8.43E-01
cg18273412	10.05	4.83	3.74E-02	14.90	7.49	4.66E-02	9.29	13.46	4.90E-01	5.53	9.65	5.67E-01	6.20	10.68	5.61E-01
cg19599410	-12.78	6.14	3.74E-02	-7.08	8.57	4.09E-01	-20.45	16.65	2.19E-01	-37.46	16.93	2.69E-02	-6.58	13.01	6.13E-01
cg18689253	28.08	13.50	3.74E-02	33.35	15.83	3.51E-02	5.85	52.59	9.11E-01	32.12	51.59	5.34E-01	9.04	36.22	8.03E-01
cg11410882	-13.52	6.50	3.74E-02	-25.50	23.20	2.72E-01	-25.75	45.01	5.67E-01	-12.46	7.04	7.69E-02	-7.69	29.20	7.92E-01
cg02543470	-13.68	6.58	3.74E-02	-10.87	17.44	5.33E-01	-3.73	31.30	9.05E-01	-14.78	7.64	5.31E-02	-14.08	24.32	5.63E-01
cg01850245	-7.74	3.72	3.75E-02	-13.15	7.14	6.56E-02	-15.21	12.40	2.20E-01	-3.34	5.38	5.35E-01	-7.51	9.27	4.18E-01
cg02695677	-8.58	4.12	3.75E-02	1.89	11.41	8.69E-01	-14.79	20.37	4.68E-01	-12.03	4.90	1.40E-02	2.60	11.94	8.28E-01
cg12028816	40.21	19.33	3.75E-02	65.08	47.58	1.71E-01	-69.01	256.06	7.88E-01	37.25	22.53	9.82E-02	26.31	63.21	6.77E-01
cg19610370	-32.35	15.55	3.75E-02	-20.66	63.18	7.44E-01	-3.08	227.93	9.89E-01	-31.28	16.65	6.03E-02	-60.36	61.81	3.29E-01
cg19807716	-7.85	3.77	3.75E-02	-16.57	9.71	8.78E-02	0.04	12.82	9.97E-01	-5.63	4.59	2.21E-01	-17.84	12.79	1.63E-01
cg03013636	-10.54	5.07	3.75E-02	-15.77	7.59	3.78E-02	-7.63	10.15	4.52E-01	-2.25	18.57	9.03E-01	-6.27	10.53	5.51E-01
cg00924484	27.41	13.17	3.75E-02	45.55	29.82	1.27E-01	102.06	122.56	4.05E-01	19.68	15.82	2.14E-01	36.92	41.63	3.75E-01
cg13573928	11.18	5.37	3.75E-02	15.46	7.86	4.92E-02	2.33	12.99	8.57E-01	12.00	11.83	3.10E-01	6.95	13.65	6.10E-01
cg11386757	12.77	6.14	3.75E-02	13.51	18.20	4.58E-01	6.01	38.96	8.77E-01	10.14	6.84	1.38E-01	52.22	25.99	4.45E-02
cg04601143	26.51	12.74	3.75E-02	38.02	34.87	2.76E-01	41.56	94.66	6.61E-01	30.02	14.58	3.94E-02	-26.98	43.99	5.40E-01
cg01441127	5.06	2.43	3.75E-02	7.41	3.71	4.61E-02	5.09	5.07	3.15E-01	0.10	4.67	9.82E-01	9.67	9.12	2.89E-01
cg24939470	41.12	19.76	3.75E-02	26.50	22.90	2.47E-01	139.22	57.90	1.62E-02	19.73	49.32	6.89E-01	41.19	38.72	2.87E-01
cg05359465	42.17	20.27	3.75E-02	65.93	48.28	1.72E-01	-56.84	218.26	7.95E-01	42.04	24.25	8.30E-02	14.28	59.41	8.10E-01
cg09991633	-20.98	10.08	3.75E-02	-51.21	18.52	5.68E-03	-11.82	40.82	7.72E-01	-13.48	9.15	1.41E-01	-4.53	25.76	8.60E-01
cg21573745	23.11	11.11	3.75E-02	43.41	18.77	2.07E-02	18.90	17.32	2.75E-01	0.12	15.15	9.93E-01	45.99	25.06	6.65E-02
cg10578701	-12.72	6.11	3.75E-02	-25.95	15.54	9.48E-02	4.08	27.52	8.82E-01	-11.24	7.35	1.26E-01	-10.79	18.94	5.69E-01
cg13039512	15.79	7.59	3.75E-02	31.93	18.63	8.66E-02	16.73	29.75	5.74E-01	12.45	9.12	1.73E-01	10.26	27.29	7.07E-01
cg20198923	-15.28	7.34	3.75E-02	-19.57	14.14	1.66E-01	-13.02	17.90	4.67E-01	-9.69	10.82	3.71E-01	-32.96	23.05	1.53E-01
cg07298299	13.39	6.44	3.75E-02	12.77	10.72	2.33E-01	17.67	14.45	2.22E-01	15.25	13.14	2.46E-01	8.06	14.36	5.75E-01

cg07221635	32.56	15.65	3.75E-02	21.00	19.39	2.79E-01	-7.03	61.92	9.10E-01	85.89	44.38	5.30E-02	53.94	39.08	1.68E-01
cg24535130	27.00	12.98	3.75E-02	24.14	18.43	1.90E-01	39.42	43.69	3.67E-01	52.86	41.87	2.07E-01	20.23	22.97	3.78E-01
cg25448856	4.30	2.07	3.75E-02	3.41	2.95	2.47E-01	9.55	4.85	4.89E-02	3.01	4.29	4.83E-01	1.98	6.79	7.71E-01
cg27315239	7.40	3.56	3.75E-02	0.35	6.56	9.57E-01	9.50	7.71	2.18E-01	11.48	5.50	3.70E-02	6.42	13.02	6.22E-01
cg25246012	-37.22	17.89	3.75E-02	-44.06	22.27	4.79E-02	52.02	73.90	4.81E-01	33.08	69.65	6.35E-01	-61.01	37.35	1.02E-01
cg12359809	-6.76	3.25	3.75E-02	-8.36	4.84	8.46E-02	-5.19	8.88	5.59E-01	-1.47	7.69	8.49E-01	-8.61	6.67	1.97E-01
cg23296572	18.36	8.83	3.75E-02	9.33	15.18	5.39E-01	6.59	15.69	6.74E-01	37.30	19.70	5.83E-02	38.92	23.19	9.34E-02
cg02743713	13.02	6.26	3.75E-02	12.41	10.10	2.19E-01	6.98	18.38	7.04E-01	28.11	21.12	1.83E-01	12.08	9.75	2.16E-01
cg16815882	7.39	3.56	3.75E-02	6.03	4.51	1.81E-01	17.33	9.31	6.27E-02	2.93	10.02	7.70E-01	7.04	10.90	5.19E-01
cg10892587	-41.10	19.76	3.75E-02	-52.17	25.86	4.36E-02	0.68	72.38	9.93E-01	46.52	71.00	5.12E-01	-54.12	38.46	1.59E-01
cg21966764	-7.00	3.36	3.75E-02	-6.56	6.27	2.95E-01	-18.23	11.14	1.02E-01	-5.05	4.86	2.99E-01	-7.26	8.95	4.17E-01
cg07545037	9.22	4.43	3.75E-02	5.54	5.36	3.02E-01	10.18	7.91	1.98E-01	25.43	9.37	6.63E-03	1.83	7.83	8.15E-01
cg02116942	-15.41	7.41	3.75E-02	-18.05	21.14	3.93E-01	-4.00	32.89	9.03E-01	-13.63	8.48	1.08E-01	-41.04	29.51	1.64E-01
cg10726312	10.50	5.05	3.75E-02	10.34	8.92	2.46E-01	13.49	19.19	4.82E-01	9.69	7.15	1.75E-01	12.69	15.05	3.99E-01
cg02520639	-6.77	3.25	3.75E-02	-7.63	4.41	8.35E-02	-2.58	6.58	6.95E-01	-22.18	13.67	1.05E-01	-4.69	8.30	5.72E-01
cg24884084	-8.73	4.20	3.75E-02	-15.16	6.40	1.79E-02	-4.91	10.28	6.33E-01	0.83	10.22	9.36E-01	-6.54	8.65	4.50E-01
cg03578662	23.28	11.19	3.76E-02	30.27	14.02	3.08E-02	0.49	35.44	9.89E-01	12.95	33.20	6.97E-01	16.50	28.95	5.69E-01
cg14853265	13.88	6.67	3.76E-02	-7.84	16.23	6.29E-01	13.27	43.04	7.58E-01	20.53	7.78	8.35E-03	-2.98	25.01	9.05E-01
cg01687997	16.17	7.78	3.76E-02	27.77	12.92	3.15E-02	22.40	18.15	2.17E-01	4.72	13.54	7.27E-01	3.50	22.10	8.74E-01
cg01432609	7.17	3.45	3.76E-02	8.34	4.30	5.24E-02	13.47	10.13	1.84E-01	-9.51	11.56	4.11E-01	7.21	8.88	4.17E-01
cg12112870	-8.11	3.90	3.76E-02	-0.67	6.81	9.22E-01	-14.35	7.68	6.18E-02	-8.13	7.22	2.60E-01	-14.85	11.14	1.83E-01
cg16687344	-23.31	11.21	3.76E-02	-30.90	13.08	1.82E-02	4.78	19.23	8.04E-01	-51.24	19.17	7.50E-03	-11.38	19.58	5.61E-01
cg06806891	8.25	3.97	3.76E-02	5.80	4.72	2.19E-01	23.22	10.91	3.33E-02	5.27	16.93	7.56E-01	7.46	12.14	5.39E-01
cg04708321	17.30	8.32	3.76E-02	33.32	14.63	2.27E-02	21.48	15.40	1.63E-01	-4.15	16.27	7.99E-01	12.16	21.64	5.74E-01
cg10051414	41.41	19.92	3.76E-02	19.59	19.93	3.26E-01	-6.94	55.45	9.00E-01	82.87	59.98	1.67E-01	78.85	28.75	6.09E-03
cg00281837	7.32	3.52	3.76E-02	9.23	4.09	2.42E-02	1.79	6.97	7.97E-01	25.21	11.82	3.29E-02	2.48	6.33	6.96E-01
cg02299940	17.88	8.60	3.76E-02	20.38	13.54	1.32E-01	54.88	28.58	5.48E-02	16.18	25.34	5.23E-01	7.23	13.76	5.99E-01
cg14991695	-39.93	19.20	3.76E-02	-85.51	53.51	1.10E-01	-228.31	295.11	4.39E-01	-26.38	21.53	2.21E-01	-97.39	71.79	1.75E-01
cg22862961	13.93	6.70	3.76E-02	-8.95	17.37	6.07E-01	24.16	21.67	2.65E-01	18.09	8.14	2.63E-02	8.98	24.01	7.08E-01
cg02083676	7.38	3.55	3.76E-02	4.97	4.51	2.71E-01	10.66	8.22	1.95E-01	14.42	14.21	3.10E-01	10.83	9.82	2.70E-01
cg12804104	-14.28	6.87	3.76E-02	-8.99	18.84	6.33E-01	16.30	25.38	5.21E-01	-15.33	8.11	5.89E-02	-42.62	24.69	8.44E-02
cg26394257	9.24	4.44	3.76E-02	9.46	6.69	1.57E-01	20.14	11.09	6.93E-02	15.02	13.24	2.57E-01	0.51	8.30	9.51E-01
cg18131932	-15.94	7.66	3.76E-02	-24.91	12.79	5.15E-02	-11.46	16.36	4.84E-01	-15.44	16.63	3.53E-01	-5.72	16.77	7.33E-01
cg05607271	29.75	14.31	3.76E-02	30.43	19.11	1.11E-01	42.54	44.44	3.38E-01	34.33	42.91	4.24E-01	19.87	30.20	5.11E-01
cg11257300	-12.43	5.98	3.76E-02	-11.26	12.45	3.66E-01	-13.78	19.37	4.77E-01	-13.52	8.04	9.26E-02	-8.61	17.18	6.16E-01

cg02044161	14.46	6.96	3.76E-02	28.71	21.54	1.83E-01	5.34	25.93	8.37E-01	15.61	7.93	4.90E-02	-17.40	30.01	5.62E-01
cg18297960	-7.49	3.60	3.76E-02	-9.94	5.45	6.79E-02	-8.04	8.45	3.41E-01	-5.80	9.88	5.57E-01	-3.66	7.25	6.14E-01
cg18091465	-9.26	4.45	3.76E-02	-15.27	7.12	3.21E-02	3.30	9.41	7.26E-01	-8.10	13.37	5.45E-01	-11.42	8.51	1.79E-01
cg18987690	-29.67	14.27	3.76E-02	-39.38	21.96	7.29E-02	-67.41	68.18	3.23E-01	-9.21	7.91	2.44E-01	-57.26	23.70	1.57E-02
cg07211239	13.33	6.41	3.76E-02	17.15	8.83	5.21E-02	13.73	13.53	3.10E-01	-1.60	22.49	9.43E-01	8.00	15.72	6.11E-01
cg27502580	-11.71	5.63	3.76E-02	-19.57	13.00	1.32E-01	8.05	23.98	7.37E-01	-9.15	7.30	2.10E-01	-18.74	13.99	1.80E-01
cg03117079	14.36	6.91	3.76E-02	12.72	9.45	1.78E-01	25.25	18.16	1.64E-01	29.40	18.75	1.17E-01	-0.39	16.01	9.81E-01
cg01309081	-13.47	6.48	3.76E-02	-10.96	10.74	3.08E-01	0.11	11.49	9.92E-01	-30.74	14.49	3.39E-02	-21.97	14.81	1.38E-01
cg02149740	9.18	4.42	3.76E-02	6.88	9.08	4.48E-01	-10.38	19.37	5.92E-01	11.13	5.49	4.25E-02	13.83	17.51	4.30E-01
cg05325193	16.60	7.99	3.76E-02	7.47	9.47	4.30E-01	8.80	25.96	7.35E-01	26.62	29.34	3.64E-01	39.05	15.70	1.29E-02
cg10052782	-11.40	5.48	3.76E-02	-22.89	12.50	6.72E-02	-16.63	20.05	4.07E-01	-8.92	7.02	2.04E-01	-2.56	15.61	8.70E-01
cg00223245	-7.84	3.77	3.76E-02	-13.76	6.84	4.42E-02	-14.18	9.92	1.53E-01	-3.13	6.21	6.14E-01	-2.47	8.80	7.79E-01
cg08248461	20.72	9.97	3.76E-02	24.83	18.80	1.87E-01	-2.90	20.21	8.86E-01	16.85	10.51	1.09E-01	60.11	24.78	1.53E-02
cg10673140	43.74	21.04	3.76E-02	16.44	15.24	2.81E-01	7.23	96.94	9.41E-01	98.79	49.09	4.42E-02	70.49	30.11	1.92E-02
cg15312943	5.64	2.71	3.76E-02	5.06	3.55	1.53E-01	9.87	7.59	1.94E-01	5.54	7.21	4.42E-01	4.32	7.09	5.42E-01
cg19621628	19.19	9.23	3.76E-02	39.46	15.67	1.18E-02	15.41	38.39	6.88E-01	17.72	8.41	3.51E-02	-17.40	25.49	4.95E-01
cg11976193	17.06	8.21	3.77E-02	31.94	17.61	6.97E-02	27.91	18.91	1.40E-01	22.66	19.06	2.35E-01	1.62	12.83	9.00E-01
cg06157570	21.76	10.47	3.77E-02	7.23	17.00	6.71E-01	40.28	20.80	5.28E-02	37.27	22.37	9.57E-02	4.40	27.19	8.72E-01
cg00894134	-11.06	5.32	3.77E-02	-17.84	8.94	4.60E-02	-10.14	11.85	3.92E-01	-9.12	12.39	4.62E-01	-3.90	10.46	7.09E-01
cg20936443	13.65	6.57	3.77E-02	12.43	14.62	3.95E-01	-22.04	26.86	4.12E-01	15.87	8.16	5.18E-02	24.04	21.79	2.70E-01
cg05645404	-12.75	6.13	3.77E-02	-15.14	19.82	4.45E-01	6.88	87.52	9.37E-01	-11.91	6.65	7.31E-02	-24.79	27.99	3.76E-01
cg03884132	28.28	13.61	3.77E-02	20.19	16.93	2.33E-01	60.99	46.30	1.88E-01	12.19	71.96	8.65E-01	41.12	28.26	1.46E-01
cg06358608	9.71	4.67	3.77E-02	10.30	5.80	7.56E-02	33.36	23.77	1.60E-01	12.33	22.02	5.76E-01	4.41	9.05	6.26E-01
cg15918259	10.06	4.84	3.77E-02	7.22	7.13	3.11E-01	13.95	12.69	2.71E-01	12.05	10.30	2.42E-01	11.80	11.62	3.10E-01
cg00672332	15.55	7.48	3.77E-02	15.52	11.46	1.76E-01	23.33	14.30	1.03E-01	-9.54	23.43	6.84E-01	17.80	16.83	2.90E-01
cg26059287	14.60	7.02	3.77E-02	7.99	11.05	4.70E-01	21.39	12.23	8.04E-02	20.95	21.75	3.36E-01	13.17	17.45	4.50E-01
cg16182691	-13.53	6.51	3.77E-02	-34.77	18.00	5.34E-02	1.63	20.85	9.38E-01	-12.31	7.78	1.14E-01	-7.32	24.42	7.64E-01
cg03002428	25.92	12.47	3.77E-02	23.75	14.02	9.03E-02	77.57	94.61	4.12E-01	64.60	73.47	3.79E-01	24.13	30.97	4.36E-01
cg02821998	16.47	7.93	3.77E-02	31.83	12.60	1.15E-02	4.19	15.31	7.84E-01	1.09	16.31	9.47E-01	23.02	20.72	2.66E-01
cg17917325	9.99	4.80	3.77E-02	13.08	6.78	5.36E-02	13.72	11.52	2.34E-01	0.24	13.42	9.85E-01	5.09	10.88	6.40E-01
cg19306380	42.82	20.60	3.77E-02	38.55	26.23	1.42E-01	-34.80	81.84	6.71E-01	-23.27	69.33	7.37E-01	83.32	35.12	1.77E-02
cg06635059	-7.02	3.38	3.77E-02	0.31	5.92	9.58E-01	-1.17	9.11	8.98E-01	-11.72	4.31	6.51E-03	-11.84	9.31	2.03E-01
cg02459569	9.88	4.76	3.77E-02	6.32	6.22	3.10E-01	26.67	11.52	2.06E-02	5.60	16.25	7.30E-01	7.31	11.90	5.39E-01
cg14870383	-38.56	18.55	3.77E-02	-40.18	24.05	9.47E-02	2.94	61.86	9.62E-01	-30.87	58.32	5.97E-01	-55.19	40.17	1.69E-01
cg07546943	-8.77	4.22	3.77E-02	-7.83	5.33	1.42E-01	-14.17	12.24	2.47E-01	6.27	12.38	6.13E-01	-21.10	11.37	6.35E-02

cg14334161	9.38	4.51	3.77E-02	7.01	6.65	2.91E-01	13.46	10.23	1.88E-01	11.10	12.68	3.81E-01	9.76	9.69	3.14E-01
cg26835419	-15.51	7.46	3.77E-02	-23.98	14.48	9.77E-02	0.61	18.64	9.74E-01	-14.74	11.83	2.13E-01	-19.14	17.80	2.82E-01
cg14523259	-13.56	6.53	3.77E-02	-29.24	14.60	4.52E-02	-33.61	20.49	1.01E-01	-5.62	7.99	4.82E-01	-10.94	19.23	5.69E-01
cg00663000	-16.45	7.92	3.77E-02	-16.75	12.57	1.83E-01	-58.45	39.80	1.42E-01	-21.38	9.04	1.80E-02	12.42	19.73	5.29E-01
cg23998071	7.21	3.47	3.77E-02	3.97	4.78	4.06E-01	17.17	10.78	1.11E-01	-1.35	20.87	9.49E-01	9.87	5.93	9.61E-02
cg10524582	-6.19	2.98	3.77E-02	-11.49	5.43	3.45E-02	-1.46	6.04	8.10E-01	-5.72	5.78	3.22E-01	-4.52	6.80	5.07E-01
cg14737332	12.76	6.14	3.77E-02	10.60	12.66	4.03E-01	74.45	41.46	7.25E-02	9.32	6.82	1.72E-01	29.28	20.55	1.54E-01
cg16292343	13.13	6.32	3.77E-02	20.65	8.57	1.60E-02	-3.03	14.51	8.34E-01	8.39	18.44	6.49E-01	10.03	16.32	5.39E-01
cg05366024	-10.69	5.15	3.77E-02	-15.39	10.89	1.58E-01	-10.44	14.63	4.75E-01	-9.02	7.13	2.06E-01	-9.60	14.13	4.97E-01
cg08036850	-33.09	15.93	3.77E-02	-79.95	45.41	7.83E-02	165.71	216.55	4.44E-01	-29.40	17.89	1.00E-01	-10.88	56.46	8.47E-01
cg20150017	17.64	8.49	3.77E-02	48.37	21.43	2.40E-02	11.49	38.81	7.67E-01	12.26	11.10	2.69E-01	11.08	18.53	5.50E-01
cg17670477	14.70	7.07	3.77E-02	10.82	8.34	1.94E-01	-7.58	43.33	8.61E-01	29.57	16.09	6.61E-02	23.26	28.94	4.22E-01
cg07704672	56.66	27.27	3.77E-02	57.10	36.71	1.20E-01	-9.86	85.62	9.08E-01	53.67	74.16	4.69E-01	89.29	59.28	1.32E-01
cg03727433	14.70	7.07	3.77E-02	23.76	17.50	1.75E-01	27.31	29.57	3.56E-01	11.62	8.41	1.67E-01	14.38	26.29	5.84E-01
cg06372015	19.18	9.23	3.77E-02	23.11	12.10	5.62E-02	50.98	38.98	1.91E-01	17.22	51.26	7.37E-01	7.04	16.06	6.61E-01
cg13782781	16.53	7.95	3.77E-02	7.93	19.20	6.79E-01	-42.22	71.31	5.54E-01	17.08	9.16	6.20E-02	45.79	32.21	1.55E-01
cg21750233	14.22	6.84	3.77E-02	18.18	15.14	2.30E-01	13.92	20.55	4.98E-01	14.31	9.02	1.13E-01	6.67	20.69	7.47E-01
cg15395461	17.17	8.27	3.78E-02	7.87	19.46	6.86E-01	44.72	34.97	2.01E-01	18.83	10.14	6.31E-02	7.35	26.36	7.80E-01
cg25407528	29.44	14.17	3.78E-02	50.37	22.56	2.56E-02	41.79	26.64	1.17E-01	5.65	9.40	5.48E-01	50.23	30.16	9.58E-02
cg09093212	-16.25	7.82	3.78E-02	-16.54	17.17	3.35E-01	-21.20	37.50	5.72E-01	-16.72	9.90	9.14E-02	-11.72	22.16	5.97E-01
cg20400248	11.26	5.42	3.78E-02	4.78	12.99	7.13E-01	30.01	20.22	1.38E-01	10.10	6.50	1.20E-01	21.21	22.29	3.41E-01
cg11608005	-12.12	5.83	3.78E-02	-6.66	14.06	6.36E-01	31.68	37.39	3.97E-01	-14.86	6.85	2.99E-02	-12.31	21.03	5.58E-01
cg27266250	-47.11	22.68	3.78E-02	-76.31	30.21	1.15E-02	-26.23	80.77	7.45E-01	-13.86	50.95	7.86E-01	4.38	56.78	9.39E-01
cg03177042	10.84	5.22	3.78E-02	8.20	9.03	3.64E-01	9.45	19.44	6.27E-01	12.86	8.74	1.41E-01	11.92	10.70	2.65E-01
cg04087740	6.79	3.27	3.78E-02	4.95	5.08	3.30E-01	12.22	6.81	7.26E-02	9.27	9.07	3.07E-01	3.19	6.87	6.42E-01
cg06522915	14.33	6.90	3.78E-02	0.84	17.75	9.62E-01	11.29	40.21	7.79E-01	18.85	7.95	1.78E-02	-4.75	26.73	8.59E-01
cg07900444	-8.42	4.05	3.78E-02	-13.32	5.41	1.38E-02	-8.08	10.27	4.32E-01	2.70	11.75	8.18E-01	-0.03	10.01	9.98E-01
cg09793883	13.77	6.63	3.78E-02	13.63	9.45	1.49E-01	7.12	16.44	6.65E-01	15.32	19.57	4.34E-01	17.99	13.80	1.92E-01
cg19627034	7.82	3.76	3.78E-02	3.01	4.91	5.40E-01	18.84	10.30	6.75E-02	11.97	11.08	2.80E-01	13.26	9.34	1.56E-01
cg11435441	-7.19	3.46	3.78E-02	-7.20	4.72	1.27E-01	-5.42	8.97	5.46E-01	-16.73	10.27	1.03E-01	-3.04	7.75	6.94E-01
cg19020855	12.70	6.12	3.78E-02	2.62	16.93	8.77E-01	-2.82	27.27	9.18E-01	15.55	7.06	2.76E-02	12.11	23.33	6.04E-01
cg07959747	-17.91	8.62	3.78E-02	-10.30	18.27	5.73E-01	-74.79	36.50	4.05E-02	-15.41	11.08	1.64E-01	-18.20	25.34	4.73E-01
cg00857137	10.66	5.13	3.78E-02	5.46	8.42	5.17E-01	10.15	11.63	3.83E-01	17.22	10.20	9.14E-02	12.70	12.08	2.93E-01
cg02827132	-40.29	19.40	3.78E-02	-30.49	25.69	2.35E-01	-51.05	97.38	6.00E-01	-63.74	42.08	1.30E-01	-41.26	46.01	3.70E-01
cg14738823	10.89	5.24	3.78E-02	8.40	8.50	3.23E-01	19.93	11.50	8.31E-02	-0.60	11.72	9.59E-01	17.41	11.43	1.28E-01

cg04215126	-11.98	5.77	3.78E-02	-14.27	9.20	1.21E-01	-20.95	15.90	1.88E-01	-7.17	12.99	5.81E-01	-7.91	10.96	4.71E-01
cg09219171	25.80	12.42	3.78E-02	29.82	15.64	5.66E-02	3.35	40.74	9.34E-01	7.09	62.21	9.09E-01	27.09	25.58	2.90E-01
cg01393945	6.09	2.93	3.78E-02	8.49	4.07	3.68E-02	6.41	6.27	3.07E-01	2.42	9.20	7.93E-01	0.18	7.32	9.80E-01
cg16792969	15.42	7.42	3.78E-02	13.23	23.09	5.67E-01	37.74	34.61	2.76E-01	11.31	8.53	1.85E-01	40.38	24.37	9.75E-02
cg16029623	-7.41	3.57	3.78E-02	-10.17	6.25	1.04E-01	-2.16	8.91	8.09E-01	-6.24	5.69	2.72E-01	-10.68	10.22	2.96E-01
cg15935791	14.44	6.95	3.78E-02	15.37	10.85	1.57E-01	10.00	16.87	5.54E-01	33.84	21.25	1.11E-01	9.00	12.44	4.70E-01
cg12084124	50.34	24.24	3.78E-02	62.52	31.08	4.43E-02	-9.07	128.48	9.44E-01	104.68	62.08	9.18E-02	-16.30	53.71	7.62E-01
cg17382888	16.70	8.04	3.78E-02	11.37	14.99	4.48E-01	33.42	23.73	1.59E-01	18.35	11.08	9.77E-02	-1.02	30.19	9.73E-01
cg19562707	12.13	5.84	3.78E-02	14.38	9.04	1.12E-01	9.49	11.61	4.14E-01	23.65	18.19	1.94E-01	5.70	12.29	6.42E-01
[truncated: 680,071 more chars]
