# Supplementary material for: DNA methylation is associated with lung function in never smokers
Source: Respir Res. 2019 Dec 2;20:268. doi: 10.1186/s12931-019-1222-8 (PMC6889726; doi:10.1186/s12931-019-1222-8)
Supplement: Supplementary file 2 — Additional file 2: Table S2. Sensitivity analysis of the association of the top 36 CpG-sites with FEV1/FVC in 659 subjects that were not exposed to environmental tobacco smoke. [file 12931_2019_1222_MOESM2_ESM.pdf]

Online supplement Table 2: Sensitivity analysis of the association of the top 36 CpG-sites with FEV_1/FVC in 659 subjects that were not exposed to environmental tobacco smoke.

		LL COPD&C (N=659)		
		**Beta**	**SE**	**P-value**
**cg10012512**	*Intergenic*	-43.08	14.31	2.62E-03
**cg02885771**	LTV1	10.34	10.18	3.10E-01
**cg25105536**	KLHL32	-101.41	53.17	5.65E-02
**cg20102034**	RTKN	53.80	18.17	3.06E-03
**cg03703840**	KIAA1731	72.53	51.21	1.57E-01
**cg21614201**	SYNPO2	-22.65	16.15	1.61E-01
**cg07957088**	PRIC285	36.15	18.31	4.83E-02
**cg05304461**	C1orf127	-105.23	41.73	1.17E-02
**cg11749902**	*Intergenic*	-28.17	8.81	1.39E-03
**cg02207312**	PRPF19	130.16	63.55	4.06E-02
**cg19734370**	NPTX1	10.21	4.81	3.38E-02
**cg03077331**	FN3K	18.20	5.87	1.95E-03
**cg18387671**	ANKRD13B	-118.99	81.41	1.44E-01
**cg03224276**	ZFHX3	40.77	22.78	7.35E-02
**cg02137691**	FGFR3	6.88	15.62	6.60E-01
**cg25884324**	UNC45A	-46.71	23.02	4.25E-02
**cg27158523**	PPIL4	-61.75	26.33	1.90E-02
**cg01157143**	NAV2	-19.16	18.38	2.97E-01
**cg07160694**	DCAF5	102.03	51.94	4.95E-02
**cg22127773**	KDM6B	-55.43	22.74	1.48E-02
**cg20939319**	TEX15	-22.73	9.88	2.15E-02
**cg02206852**	PROCA1	32.06	19.10	9.33E-02
**cg17075019**	*Intergenic*	35.08	15.71	2.56E-02
**cg25556432**	*Intergenic*	29.26	9.72	2.61E-03
**cg22742965**	TMEFF2	-32.19	13.08	1.39E-02
**cg16734845**	CTDSPL2	-49.45	26.87	6.57E-02
**cg09108394**	PRKCB	-18.41	9.90	6.29E-02
**cg10034572**	*Intergenic*	-20.76	16.06	1.96E-01
**cg20066227**	C1QL3	37.09	21.28	8.13E-02
**cg07148038**	TNXB	30.78	19.81	1.20E-01
**cg23396786**	SFXN5	21.79	8.82	1.35E-02
**cg06218079**	TBCD	5.74	3.50	1.01E-01
**cg06982745**	ADAMTS14	-35.28	21.97	1.08E-01
**cg05946118**	*Intergenic*	-13.58	8.23	9.90E-02
**cg08065963**	*Intergenic*	-18.04	6.92	9.13E-03
**cg12064372**	*Intergenic*	33.93	21.78	1.19E-01
