# Supplementary material for: DNA methylation is associated with lung function in never smokers
Source: Respir Res. 2019 Dec 2;20:268. doi: 10.1186/s12931-019-1222-8 (PMC6889726; doi:10.1186/s12931-019-1222-8)
Supplement: Supplementary file 3 — Additional file 3: Table S3. Overview of association between DNA methylation and gene expression. [file 12931_2019_1222_MOESM3_ESM.pdf]

Online supplement Table 3: Overview of association between DNA methylation and gene expression.

Study			Meta			LL			LLS			NTR			RS		
CpG-site	N genes within 2MB	ENSG gene	Beta	SE	P-value	Beta	SE	P-value	Beta	SE	P-value	Beta	SE	P-value	Beta	SE	P-value
cg02137691	31	ENSG00000145217	0.01556	0.00376	3.53E-05	0.01180	0.00936	2.07E-01	0.01818	0.01212	1.34E-01	0.01837	0.00495	2.07E-04	0.00787	0.00929	3.97E-01
cg08065963	12	ENSG00000183044	0.01274	0.00341	1.85E-04	0.00886	0.00805	2.71E-01	0.00865	0.01306	5.07E-01	0.01539	0.00412	1.87E-04	0.00045	0.01304	9.72E-01
cg02206852	52	ENSG00000108256	0.00840	0.00217	1.06E-04	0.01120	0.00340	9.89E-04	-0.00100	0.01014	9.21E-01	0.00508	0.00362	1.60E-01	0.01094	0.00498	2.80E-02
cg22127773	80	ENSG00000167874	0.00105	0.00028	1.82E-04	0.00064	0.00092	4.88E-01	0.00071	0.00073	3.30E-01	0.00115	0.00033	5.78E-04	0.00140	0.00122	2.52E-01
cg25105536	4	ENSG00000186231	-0.00041	0.00015	5.52E-03	-0.00053	0.00037	1.55E-01	-0.00062	0.00047	1.82E-01	-0.00033	0.00018	6.79E-02	-0.00052	0.00049	2.94E-01
cg02206852	52	ENSG00000108262	0.00801	0.00234	6.11E-04	0.01142	0.00592	5.37E-02	0.01125	0.00392	4.07E-03	0.00371	0.00364	3.09E-01	0.00914	0.00848	2.81E-01
cg23396786	18	ENSG00000003137	0.00243	0.00078	1.78E-03	0.00415	0.00185	2.46E-02	-0.00147	0.00307	6.33E-01	0.00224	0.00095	1.86E-02	0.00325	0.00261	2.13E-01
cg07148038	89	ENSG00000250535	0.00354	0.00099	3.77E-04	0.00417	0.00494	3.98E-01	0.00065	0.00253	7.97E-01	0.00294	0.00127	2.10E-02	0.00571	0.00165	5.44E-04
cg20939319	10	ENSG00000133872	-0.00293	0.00100	3.36E-03	-0.00248	0.00130	5.78E-02	-0.00377	0.00233	1.06E-01	-0.00603	0.00358	9.20E-02	-0.00209	0.00257	4.16E-01
cg07148038	89	ENSG00000213760	0.00738	0.00208	3.79E-04	0.01107	0.00686	1.07E-01	0.00491	0.00742	5.08E-01	0.00671	0.00289	2.01E-02	0.00800	0.00370	3.06E-02
cg02885771	11	ENSG00000229036	0.00961	0.00329	3.51E-03	0.00246	0.00526	6.40E-01	0.00751	0.00609	2.17E-01	0.01696	0.00546	1.91E-03	0.01211	0.00632	5.52E-02
cg05946118	12	ENSG00000263244	-0.01444	0.00506	4.28E-03	-0.02285	0.00636	3.26E-04	-0.01575	0.01105	1.54E-01	-0.01640	0.00393	3.04E-05	0.00712	0.01065	5.04E-01
cg21614201	13	ENSG00000245958	-0.00549	0.00195	4.84E-03	-0.00475	0.00433	2.73E-01	-0.01032	0.00307	7.85E-04	-0.00276	0.00196	1.60E-01	-0.00573	0.00492	2.45E-01
cg25884324	20	ENSG00000140553	-0.00187	0.00066	4.54E-03	-0.00187	0.00162	2.49E-01	-0.00260	0.00165	1.15E-01	-0.00237	0.00096	1.30E-02	-0.00009	0.00148	9.52E-01
cg07148038	89	ENSG00000204256	0.00839	0.00257	1.07E-03	0.00399	0.00561	4.77E-01	0.00393	0.00657	5.49E-01	0.01236	0.00363	6.69E-04	0.00569	0.00687	4.08E-01
cg02207312	37	ENSG00000134824	-0.00082	0.00027	2.62E-03	0.00006	0.00072	9.39E-01	-0.00147	0.00062	1.78E-02	-0.00092	0.00039	1.74E-02	-0.00054	0.00068	4.31E-01
cg20102034	37	ENSG00000115317	0.01018	0.00339	2.65E-03	0.00175	0.00769	8.20E-01	0.01641	0.00882	6.27E-02	0.01341	0.00462	3.73E-03	0.00184	0.00972	8.50E-01
cg22742965	4	ENSG00000168497	0.00463	0.00218	3.40E-02	0.00074	0.00550	8.93E-01	0.00621	0.00678	3.60E-01	0.00427	0.00268	1.11E-01	0.01372	0.00796	8.46E-02
cg03077331	45	ENSG00000141580	-0.00445	0.00152	3.53E-03	-0.00753	0.01008	4.55E-01	-0.00589	0.00336	7.99E-02	-0.00395	0.00178	2.63E-02	-0.00440	0.00793	5.79E-01
cg10034572	8	ENSG00000226266	-0.00413	0.00179	2.11E-02	-0.00358	0.00337	2.88E-01	-0.00782	0.00508	1.23E-01	-0.00462	0.00248	6.28E-02	0.00358	0.00666	5.91E-01
cg10034572	8	ENSG00000196151	-0.00520	0.00227	2.21E-02	-0.00402	0.00510	4.31E-01	-0.01164	0.00879	1.86E-01	-0.00590	0.00276	3.28E-02	0.00596	0.00927	5.20E-01
cg25884324	20	ENSG00000185033	-0.00101	0.00039	9.13E-03	-0.00066	0.00118	5.74E-01	-0.00160	0.00127	2.10E-01	-0.00084	0.00046	6.60E-02	-0.00226	0.00134	9.27E-02
cg02206852	52	ENSG00000076604	-0.00391	0.00134	3.58E-03	-0.00333	0.00441	4.49E-01	-0.00372	0.00181	3.95E-02	-0.00364	0.00239	1.28E-01	-0.01027	0.00681	1.31E-01
cg06218079	42	ENSG00000266445	0.00152	0.00054	4.53E-03	0.00006	0.00197	9.74E-01	0.00324	0.00205	1.15E-01	0.00167	0.00061	5.93E-03	-0.00009	0.00193	9.62E-01
cg08065963	12	ENSG00000263244	-0.01377	0.00573	1.63E-02	-0.00663	0.00821	4.19E-01	-0.01599	0.01354	2.38E-01	-0.02274	0.00496	4.50E-06	0.00168	0.01326	8.99E-01
cg05304461	26	ENSG00000120948	0.00058	0.00022	7.77E-03	0.00083	0.00045	6.37E-02	0.00084	0.00063	1.82E-01	0.00046	0.00029	1.10E-01	0.00027	0.00071	7.04E-01
cg07148038	89	ENSG00000196126	-0.00257	0.00084	2.30E-03	-0.00223	0.00112	4.63E-02	-0.00323	0.00170	5.72E-02	-0.00119	0.00070	9.04E-02	-0.00503	0.00146	5.63E-04
cg08065963	12	ENSG00000262944	-0.00906	0.00390	2.01E-02	-0.01110	0.00741	1.34E-01	-0.00892	0.00977	3.61E-01	-0.01237	0.00497	1.29E-02	0.01012	0.01148	3.78E-01
cg20066227	9	ENSG00000148484	-0.00434	0.00197	2.78E-02	-0.00839	0.00520	1.07E-01	-0.00856	0.00644	1.84E-01	-0.00357	0.00242	1.40E-01	0.00045	0.00629	9.44E-01
cg05304461	26	ENSG00000171824	-0.00037	0.00014	1.01E-02	-0.00076	0.00062	2.20E-01	-0.00144	0.00122	2.36E-01	-0.00037	0.00017	2.74E-02	-0.00019	0.00033	5.74E-01
cg06982745	18	ENSG00000166228	0.00164	0.00068	1.53E-02	-0.00039	0.00300	8.96E-01	0.00300	0.00135	2.69E-02	0.00115	0.00084	1.69E-01	0.00346	0.00313	2.70E-01
cg25884324	20	ENSG00000184508	0.00358	0.00146	1.44E-02	0.00435	0.00308	1.58E-01	0.00977	0.00419	1.98E-02	0.00285	0.00180	1.12E-01	0.00010	0.00377	9.80E-01
cg16734845	25	ENSG00000092470	0.00103	0.00041	1.20E-02	0.00103	0.00097	2.88E-01	0.00292	0.00132	2.73E-02	0.00080	0.00051	1.15E-01	0.00062	0.00160	6.99E-01
cg20102034	37	ENSG00000114993	-0.00844	0.00319	8.27E-03	-0.00243	0.00844	7.73E-01	-0.00023	0.01182	9.84E-01	-0.00984	0.00382	9.89E-03	-0.01417	0.01112	2.02E-01
cg06218079	42	ENSG00000268852	-0.00097	0.00036	7.34E-03	-0.00067	0.00055	2.22E-01	-0.00133	0.00053	1.21E-02	-0.00044	0.00113	6.99E-01	-0.00594	0.00686	3.87E-01

cg10034572	8	ENSG00000224152	-0.00273	0.00132	3.87E-02	-0.00133	0.00094	1.56E-01	-0.00466	0.00170	6.01E-03	-0.00009	0.00063	8.89E-01	-0.00667	0.00185	3.03E-04
cg03703840	14	ENSG00000196371	-0.00077	0.00034	2.26E-02	0.00009	0.00078	9.09E-01	-0.00155	0.00079	4.89E-02	-0.00107	0.00050	3.15E-02	-0.00022	0.00073	7.59E-01
cg10034572	8	ENSG00000241399	-0.00362	0.00178	4.19E-02	-0.00514	0.00413	2.13E-01	-0.01112	0.00608	6.75E-02	-0.00235	0.00219	2.83E-01	-0.00238	0.00695	7.32E-01
cg07957088	43	ENSG00000060491	-0.00434	0.00165	8.42E-03	-0.00037	0.00414	9.29E-01	-0.00570	0.00456	2.11E-01	-0.00540	0.00232	1.99E-02	-0.00395	0.00363	2.77E-01
cg25105536	4	ENSG00000146263	0.00037	0.00022	9.26E-02	-0.00007	0.00067	9.19E-01	-0.00013	0.00076	8.61E-01	0.00054	0.00026	3.49E-02	-0.00014	0.00082	8.63E-01
cg07148038	89	ENSG00000204252	0.00418	0.00146	4.19E-03	0.00537	0.00322	9.47E-02	0.00618	0.00372	9.66E-02	0.00425	0.00205	3.78E-02	-0.00037	0.00407	9.27E-01
cg25105536	4	ENSG00000014123	0.00011	0.00007	9.65E-02	0.00027	0.00012	2.08E-02	0.00022	0.00016	1.76E-01	0.00006	0.00006	2.53E-01	-0.00007	0.00014	6.41E-01
cg09108394	15	ENSG00000166501	-0.00735	0.00340	3.08E-02	-0.01109	0.00747	1.38E-01	0.00728	0.01205	5.46E-01	-0.00729	0.00420	8.24E-02	-0.01519	0.01453	2.96E-01
cg07160694	13	ENSG00000100650	0.00010	0.00005	3.64E-02	0.00014	0.00009	1.24E-01	0.00000	0.00018	9.89E-01	0.00007	0.00006	2.13E-01	0.00022	0.00017	1.87E-01
cg22127773	80	ENSG00000166579	0.00079	0.00029	6.14E-03	0.00085	0.00037	2.27E-02	0.00141	0.00120	2.42E-01	0.00074	0.00051	1.46E-01	-0.00153	0.00188	4.17E-01
cg25556432	15	ENSG00000177483	-0.00501	0.00238	3.48E-02	-0.00915	0.00549	9.58E-02	0.00281	0.00627	6.54E-01	-0.00659	0.00301	2.84E-02	0.00143	0.00791	8.56E-01
cg06218079	42	ENSG00000169750	-0.00096	0.00039	1.30E-02	-0.00011	0.00011	2.86E-01	0.00002	0.00011	8.81E-01	-0.00177	0.00026	1.26E-11	-0.00369	0.00086	1.73E-05
cg18387671	42	ENSG00000160602	0.00026	0.00010	1.32E-02	0.00010	0.00040	7.98E-01	0.00051	0.00028	7.17E-02	0.00031	0.00013	2.10E-02	-0.00003	0.00024	9.09E-01
cg07148038	89	ENSG00000241404	0.00250	0.00091	6.21E-03	-0.00529	0.00564	3.48E-01	0.00359	0.00202	7.55E-02	0.00239	0.00107	2.51E-02	0.00403	0.00465	3.86E-01
cg08065963	12	ENSG00000260276	0.00747	0.00375	4.65E-02	0.00173	0.00660	7.93E-01	0.01729	0.00937	6.50E-02	0.00433	0.00326	1.84E-01	0.01984	0.00958	3.85E-02
cg20102034	37	ENSG00000115275	-0.00615	0.00253	1.52E-02	0.00166	0.00547	7.61E-01	-0.01093	0.00672	1.04E-01	-0.00775	0.00346	2.49E-02	-0.00743	0.00778	3.39E-01
cg16734845	25	ENSG00000166734	0.00568	0.00251	2.37E-02	0.00519	0.00536	3.33E-01	0.00868	0.00600	1.48E-01	0.00574	0.00359	1.10E-01	0.00183	0.00736	8.03E-01
cg03077331	45	ENSG00000169750	0.00048	0.00019	1.37E-02	-0.00146	0.00202	4.68E-01	0.00113	0.00078	1.45E-01	0.00050	0.00021	1.56E-02	-0.00026	0.00078	7.41E-01
cg07148038	89	ENSG00000204257	0.00757	0.00281	6.97E-03	0.00399	0.00627	5.24E-01	0.00496	0.00891	5.78E-01	0.01031	0.00371	5.38E-03	0.00288	0.00786	7.14E-01
cg02885771	11	ENSG00000135604	-0.00206	0.00108	5.79E-02	-0.00489	0.00178	5.88E-03	-0.00249	0.00273	3.61E-01	-0.00063	0.00106	5.49E-01	-0.00125	0.00234	5.94E-01
cg06218079	42	ENSG00000265678	-0.00094	0.00039	1.61E-02	-0.00195	0.00126	1.21E-01	0.00002	0.00149	9.88E-01	-0.00087	0.00045	5.23E-02	-0.00135	0.00153	3.79E-01
cg10012512	7	ENSG00000233038	-0.00198	0.00120	9.90E-02	-0.00277	0.00094	3.33E-03	0.00574	0.00513	2.63E-01	-0.00393	0.00255	1.24E-01	-0.00010	0.00228	9.65E-01
cg05304461	26	ENSG00000130939	0.00083	0.00038	2.77E-02	0.00087	0.00077	2.59E-01	0.00053	0.00116	6.51E-01	0.00117	0.00050	1.99E-02	-0.00088	0.00119	4.58E-01
cg02885771	11	ENSG00000001036	0.00116	0.00064	6.87E-02	-0.00045	0.00160	7.80E-01	0.00050	0.00239	8.35E-01	0.00146	0.00079	6.55E-02	0.00195	0.00176	2.68E-01
cg22127773	80	ENSG00000132535	-0.00105	0.00041	9.83E-03	-0.00145	0.00072	4.28E-02	-0.00246	0.00131	5.99E-02	-0.00070	0.00058	2.31E-01	0.00002	0.00142	9.88E-01
cg12064372	10	ENSG00000139160	0.00155	0.00091	8.91E-02	-0.00039	0.00121	7.49E-01	0.00189	0.00227	4.04E-01	0.00204	0.00074	5.95E-03	0.00539	0.00294	6.66E-02
cg09108394	15	ENSG00000260482	-0.00512	0.00274	6.18E-02	-0.00494	0.00768	5.20E-01	0.00139	0.00997	8.89E-01	-0.00732	0.00328	2.57E-02	0.00519	0.00872	5.52E-01
cg07148038	89	ENSG00000204387	0.00247	0.00097	1.12E-02	0.00103	0.00159	5.16E-01	0.00016	0.00220	9.44E-01	0.00390	0.00113	5.34E-04	0.00405	0.00307	1.87E-01
cg02207312	37	ENSG00000156738	-0.00012	0.00005	2.96E-02	-0.00012	0.00006	5.07E-02	0.00009	0.00066	8.98E-01	-0.00002	0.00013	8.53E-01	-0.00028	0.00019	1.35E-01
cg07148038	89	ENSG00000204528	-0.00427	0.00171	1.27E-02	0.00297	0.00505	5.56E-01	-0.00759	0.00493	1.24E-01	-0.00457	0.00243	6.01E-02	-0.00532	0.00331	1.08E-01
cg20066227	9	ENSG00000148488	-0.00063	0.00041	1.28E-01	0.00008	0.00093	9.34E-01	0.00016	0.00123	8.99E-01	-0.00113	0.00055	3.78E-02	-0.00008	0.00124	9.46E-01
cg07160694	13	ENSG00000072110	0.00021	0.00013	9.22E-02	0.00000	0.00027	9.96E-01	0.00007	0.00036	8.53E-01	0.00024	0.00017	1.56E-01	0.00053	0.00034	1.19E-01
cg23396786	18	ENSG00000144034	0.00516	0.00284	6.92E-02	0.00185	0.00604	7.59E-01	0.00736	0.00770	3.39E-01	0.00524	0.00384	1.73E-01	0.00927	0.00920	3.14E-01
cg10012512	7	ENSG00000105982	-0.00238	0.00179	1.85E-01	-0.00320	0.00277	2.48E-01	0.00390	0.00343	2.55E-01	-0.00405	0.00190	3.26E-02	-0.00512	0.00454	2.60E-01
cg19734370	32	ENSG00000157637	0.00396	0.00195	4.23E-02	0.00071	0.00120	5.55E-01	0.00750	0.00246	2.31E-03	0.00120	0.00128	3.49E-01	0.01348	0.00469	4.07E-03
cg10012512	7	ENSG00000105983	-0.00246	0.00191	1.98E-01	0.00058	0.00476	9.03E-01	0.00077	0.00682	9.11E-01	-0.00328	0.00225	1.45E-01	-0.00654	0.00992	5.10E-01
cg20102034	37	ENSG00000204872	0.00097	0.00047	3.78E-02	-0.00256	0.00381	5.01E-01	0.00282	0.00486	5.62E-01	0.00140	0.00217	5.18E-01	0.00099	0.00049	4.16E-02
cg25884324	20	ENSG00000185043	-0.00322	0.00183	7.80E-02	-0.00796	0.00308	9.66E-03	-0.00407	0.00355	2.52E-01	-0.00017	0.00120	8.88E-01	-0.00359	0.00258	1.65E-01
cg02206852	52	ENSG00000160602	-0.00561	0.00259	3.05E-02	-0.01143	0.00713	1.09E-01	0.00641	0.01096	5.59E-01	-0.00533	0.00315	9.12E-02	-0.00633	0.00706	3.70E-01
cg09108394	15	ENSG00000103365	0.00139	0.00086	1.06E-01	0.00112	0.00133	3.97E-01	-0.00231	0.00225	3.05E-01	0.00239	0.00092	9.20E-03	0.00218	0.00249	3.81E-01
cg22742965	4	ENSG00000128641	0.00037	0.00044	4.00E-01	-0.00061	0.00099	5.37E-01	0.00048	0.00146	7.45E-01	0.00089	0.00057	1.18E-01	-0.00088	0.00141	5.32E-01
cg06218079	42	ENSG00000261888	0.00125	0.00060	3.82E-02	0.00005	0.00013	7.04E-01	-0.00021	0.00017	2.31E-01	0.00476	0.00071	1.67E-11	0.00475	0.00340	1.63E-01

cg22742965	4	ENSG00000138378	-0.00163	0.00194	4.02E-01	0.00231	0.00324	4.77E-01	-0.00457	0.00335	1.73E-01	0.00041	0.00223	8.53E-01	-0.00661	0.00368	7.21E-02
cg17075019	4	ENSG00000151208	-0.00237	0.00284	4.04E-01	-0.01202	0.00930	1.96E-01	-0.00762	0.00999	4.46E-01	-0.00182	0.00331	5.82E-01	0.00785	0.00947	4.08E-01
cg05304461	26	ENSG00000175206	0.00030	0.00016	6.27E-02	0.00031	0.00021	1.39E-01	0.00017	0.00032	5.90E-01	0.00078	0.00050	1.16E-01	-0.00055	0.00105	5.98E-01
cg11749902	11	ENSG00000104368	-0.00729	0.00508	1.52E-01	-0.01715	0.01143	1.33E-01	-0.01372	0.00798	8.58E-02	-0.00513	0.00451	2.55E-01	0.02052	0.01700	2.27E-01
cg03703840	14	ENSG00000182919	-0.00037	0.00024	1.19E-01	-0.00041	0.00047	3.79E-01	-0.00096	0.00044	2.78E-02	-0.00023	0.00050	6.39E-01	0.00014	0.00044	7.51E-01
cg25556432	15	ENSG00000186235	0.00357	0.00225	1.14E-01	0.00699	0.00404	8.32E-02	0.00968	0.00459	3.48E-02	0.00190	0.00136	1.63E-01	-0.00351	0.00546	5.21E-01
cg06982745	18	ENSG00000180817	-0.00133	0.00080	9.64E-02	-0.00409	0.00342	2.32E-01	-0.00216	0.00104	3.68E-02	-0.00030	0.00038	4.39E-01	-0.00326	0.00244	1.82E-01
cg09108394	15	ENSG00000166847	-0.00305	0.00194	1.16E-01	-0.00310	0.00385	4.21E-01	-0.00689	0.00479	1.50E-01	-0.00154	0.00276	5.76E-01	-0.00436	0.00671	5.16E-01
cg03224276	13	ENSG00000261337	0.00231	0.00154	1.34E-01	0.00383	0.00358	2.85E-01	0.00488	0.00397	2.19E-01	-0.00032	0.00180	8.60E-01	0.00521	0.00297	7.94E-02
cg27158523	11	ENSG00000186625	-0.00026	0.00018	1.60E-01	-0.00043	0.00040	2.81E-01	-0.00007	0.00054	9.03E-01	-0.00025	0.00023	2.87E-01	-0.00010	0.00076	8.91E-01
cg22127773	80	ENSG00000219200	-0.00097	0.00043	2.21E-02	-0.00109	0.00132	4.09E-01	-0.00163	0.00070	2.05E-02	-0.00010	0.00069	8.85E-01	-0.00148	0.00109	1.73E-01
cg20939319	10	ENSG00000104691	0.00296	0.00220	1.79E-01	0.00649	0.00368	7.74E-02	-0.00488	0.00599	4.15E-01	0.00417	0.00226	6.51E-02	-0.00346	0.00671	6.05E-01
cg11749902	11	ENSG00000147533	-0.00207	0.00149	1.64E-01	-0.00341	0.00209	1.04E-01	0.00049	0.01678	9.77E-01	-0.00321	0.00100	1.41E-03	0.00392	0.00321	2.22E-01
cg07957088	43	ENSG00000092758	-0.00481	0.00237	4.21E-02	0.00522	0.00696	4.54E-01	-0.00895	0.00909	3.25E-01	-0.00590	0.00279	3.43E-02	-0.00573	0.00760	4.51E-01
cg05304461	26	ENSG00000177000	0.00012	0.00007	7.02E-02	0.00028	0.00015	7.01E-02	0.00056	0.00069	4.12E-01	0.00009	0.00008	2.50E-01	-0.00001	0.00022	9.48E-01
cg20939319	10	ENSG00000197265	0.00316	0.00238	1.85E-01	0.00111	0.00370	7.64E-01	0.00519	0.00515	3.14E-01	0.00740	0.00478	1.22E-01	-0.00207	0.00681	7.62E-01
cg07148038	89	ENSG00000258388	0.00320	0.00139	2.12E-02	0.00175	0.00183	3.40E-01	0.00100	0.00580	8.62E-01	0.00630	0.00241	8.89E-03	0.00125	0.00723	8.63E-01
cg03703840	14	ENSG00000042429	0.00026	0.00017	1.34E-01	0.00034	0.00046	4.61E-01	0.00056	0.00042	1.80E-01	0.00015	0.00027	5.72E-01	0.00018	0.00032	5.68E-01
cg08065963	12	ENSG00000187555	0.00671	0.00475	1.57E-01	-0.00286	0.00532	5.91E-01	0.01920	0.00721	7.73E-03	0.01007	0.00368	6.26E-03	-0.00047	0.00925	9.60E-01
cg08065963	12	ENSG00000182831	0.00260	0.00187	1.64E-01	-0.00100	0.00414	8.09E-01	-0.00070	0.00630	9.12E-01	0.00428	0.00235	6.87E-02	0.00208	0.00668	7.55E-01
cg03077331	45	ENSG00000169738	0.00168	0.00084	4.50E-02	0.00101	0.00099	3.09E-01	0.00025	0.00034	4.67E-01	0.00111	0.00052	3.41E-02	0.00755	0.00177	1.99E-05
cg23396786	18	ENSG00000237883	-0.00298	0.00189	1.14E-01	0.00234	0.00578	6.86E-01	-0.00378	0.00849	6.56E-01	-0.00247	0.00224	2.69E-01	-0.00952	0.00513	6.36E-02
cg05304461	26	ENSG00000120942	-0.00065	0.00037	7.93E-02	0.00036	0.00089	6.85E-01	-0.00028	0.00160	8.63E-01	-0.00086	0.00044	5.08E-02	-0.00147	0.00152	3.35E-01
cg03077331	45	ENSG00000169718	-0.00121	0.00061	4.63E-02	-0.00007	0.00292	9.82E-01	-0.00073	0.00121	5.43E-01	-0.00158	0.00075	3.57E-02	0.00020	0.00273	9.42E-01
cg18387671	42	ENSG00000264577	-0.00038	0.00020	5.09E-02	-0.00048	0.00060	4.23E-01	-0.00053	0.00040	1.87E-01	-0.00036	0.00024	1.40E-01	0.00137	0.00144	3.42E-01
cg07148038	89	ENSG00000204301	-0.00327	0.00145	2.41E-02	-0.00234	0.00311	4.52E-01	-0.00681	0.00395	8.45E-02	-0.00229	0.00192	2.32E-01	-0.00689	0.00520	1.85E-01
cg17075019	4	ENSG00000148606	-0.00120	0.00196	5.38E-01	-0.00318	0.00542	5.58E-01	0.00558	0.00491	2.55E-01	-0.00289	0.00274	2.93E-01	-0.00104	0.00434	8.11E-01
cg27158523	11	ENSG00000120253	-0.00027	0.00021	1.97E-01	-0.00059	0.00039	1.32E-01	-0.00104	0.00061	9.05E-02	-0.00006	0.00020	7.50E-01	0.00019	0.00075	7.95E-01
cg07148038	89	ENSG00000204427	-0.00379	0.00169	2.51E-02	-0.00692	0.01056	5.12E-01	-0.00777	0.00351	2.69E-02	-0.00352	0.00232	1.29E-01	0.00027	0.00366	9.41E-01
cg06218079	42	ENSG00000141574	0.00118	0.00061	5.33E-02	0.00094	0.00121	4.35E-01	-0.00042	0.00145	7.70E-01	0.00172	0.00083	3.67E-02	0.00398	0.00463	3.90E-01
cg11749902	11	ENSG00000070718	-0.00617	0.00488	2.06E-01	-0.00418	0.00621	5.01E-01	-0.01964	0.00651	2.54E-03	0.00221	0.00300	4.61E-01	-0.00694	0.00655	2.89E-01
cg01157143	9	ENSG00000179057	0.00468	0.00409	2.53E-01	0.01359	0.00573	1.78E-02	-0.00382	0.00623	5.40E-01	0.00048	0.00303	8.73E-01	0.01286	0.00835	1.24E-01
cg27158523	11	ENSG00000220848	-0.00060	0.00048	2.10E-01	-0.00173	0.00069	1.17E-02	-0.00050	0.00131	7.05E-01	-0.00011	0.00011	3.28E-01	-0.00045	0.00146	7.59E-01
cg20066227	9	ENSG00000165985	0.00225	0.00199	2.59E-01	-0.00433	0.00415	2.97E-01	0.00095	0.00458	8.35E-01	0.00453	0.00189	1.67E-02	0.00398	0.00457	3.83E-01
cg20066227	9	ENSG00000107611	-0.00123	0.00109	2.59E-01	-0.00206	0.00275	4.52E-01	0.00066	0.00255	7.96E-01	-0.00206	0.00160	1.96E-01	-0.00032	0.00246	8.96E-01
cg27158523	11	ENSG00000131013	0.00056	0.00045	2.16E-01	0.00080	0.00049	1.04E-01	0.00170	0.00071	1.59E-02	-0.00021	0.00031	4.97E-01	0.00033	0.00101	7.46E-01
cg20102034	37	ENSG00000144048	-0.00237	0.00128	6.41E-02	-0.00317	0.00611	6.05E-01	-0.00775	0.00944	4.12E-01	-0.00310	0.00153	4.25E-02	0.00040	0.00266	8.80E-01
cg03224276	13	ENSG00000187008	-0.00229	0.00175	1.91E-01	0.00207	0.00284	4.67E-01	-0.00548	0.00275	4.65E-02	-0.00046	0.00116	6.94E-01	-0.00572	0.00221	9.73E-03
cg05304461	26	ENSG00000116685	-0.00046	0.00028	9.62E-02	-0.00023	0.00069	7.38E-01	-0.00037	0.00107	7.29E-01	-0.00056	0.00033	9.31E-02	-0.00014	0.00102	8.90E-01
cg25105536	4	ENSG00000123545	0.00012	0.00024	6.26E-01	0.00083	0.00039	3.13E-02	-0.00021	0.00038	5.71E-01	-0.00021	0.00022	3.28E-01	0.00027	0.00037	4.76E-01
cg03077331	45	ENSG00000169696	-0.00861	0.00450	5.57E-02	-0.01929	0.00771	1.24E-02	-0.00407	0.00304	1.81E-01	0.00034	0.00108	7.54E-01	-0.02625	0.00867	2.46E-03
cg02137691	31	ENSG00000123933	-0.00850	0.00487	8.10E-02	0.00387	0.00932	6.78E-01	0.00167	0.01177	8.88E-01	-0.01489	0.00404	2.28E-04	-0.01261	0.00905	1.63E-01

cg03077331	45	ENSG00000178927	0.00038	0.00020	5.61E-02	0.00209	0.00796	7.93E-01	-0.00203	0.00345	5.57E-01	0.00045	0.00021	2.90E-02	-0.00036	0.00069	6.01E-01
cg02206852	52	ENSG00000244045	-0.00302	0.00153	4.93E-02	-0.00744	0.00897	4.07E-01	-0.00581	0.00385	1.31E-01	-0.00230	0.00171	1.80E-01	-0.00349	0.01605	8.28E-01
cg07160694	13	ENSG00000029364	-0.00011	0.00008	1.97E-01	0.00007	0.00008	4.16E-01	-0.00028	0.00015	7.06E-02	-0.00003	0.00004	5.64E-01	-0.00034	0.00012	5.41E-03
cg23396786	18	ENSG00000144036	0.00881	0.00601	1.43E-01	-0.00747	0.01044	4.74E-01	0.00822	0.01396	5.56E-01	0.01679	0.00664	1.15E-02	0.01274	0.01534	4.06E-01
cg03703840	14	ENSG00000110218	0.00035	0.00026	1.85E-01	-0.00016	0.00079	8.43E-01	-0.00028	0.00083	7.32E-01	0.00053	0.00033	1.06E-01	0.00036	0.00069	6.01E-01
cg08065963	12	ENSG00000140650	0.00314	0.00254	2.17E-01	0.00463	0.00433	2.86E-01	0.01152	0.00518	2.62E-02	0.00102	0.00274	7.10E-01	-0.00250	0.00516	6.29E-01
cg10034572	8	ENSG00000136536	-0.00212	0.00216	3.28E-01	0.00072	0.00469	8.79E-01	0.00801	0.00705	2.56E-01	-0.00422	0.00220	5.48E-02	-0.00344	0.00791	6.63E-01
cg17075019	4	ENSG00000138326	0.00037	0.00084	6.59E-01	0.00306	0.00165	6.46E-02	-0.00212	0.00210	3.12E-01	-0.00008	0.00071	9.15E-01	0.00053	0.00165	7.48E-01
cg03224276	13	ENSG00000182149	-0.00158	0.00125	2.08E-01	-0.00065	0.00232	7.80E-01	-0.00347	0.00219	1.14E-01	0.00063	0.00133	6.33E-01	-0.00418	0.00210	4.69E-02
cg05946118	12	ENSG00000067365	0.00098	0.00084	2.40E-01	0.00267	0.00105	1.11E-02	0.00025	0.00179	8.91E-01	-0.00024	0.00057	6.72E-01	0.00171	0.00156	2.71E-01
cg03077331	45	ENSG00000263731	-0.00270	0.00146	6.44E-02	0.00517	0.00610	3.97E-01	-0.00213	0.00246	3.87E-01	-0.00395	0.00194	4.20E-02	-0.00047	0.00970	9.61E-01
cg02206852	52	ENSG00000258472	-0.00292	0.00153	5.70E-02	-0.00194	0.00171	2.56E-01	0.00154	0.01380	9.11E-01	-0.00319	0.00646	6.22E-01	-0.00932	0.00427	2.92E-02
cg06982745	18	ENSG00000099284	0.00115	0.00083	1.66E-01	-0.00260	0.00296	3.79E-01	0.00318	0.00258	2.18E-01	0.00130	0.00096	1.77E-01	0.00081	0.00298	7.86E-01
cg19734370	32	ENSG00000173894	0.00127	0.00076	9.32E-02	0.00227	0.00101	2.51E-02	-0.00055	0.00232	8.11E-01	0.00023	0.00119	8.48E-01	0.00676	0.00629	2.83E-01
cg19734370	32	ENSG00000141582	0.00177	0.00106	9.47E-02	0.00054	0.00097	5.78E-01	0.00473	0.00193	1.44E-02	0.00087	0.00147	5.54E-01	0.00558	0.00458	2.22E-01
cg11749902	11	ENSG00000158669	-0.00719	0.00663	2.78E-01	0.01199	0.00933	1.99E-01	-0.01354	0.00924	1.43E-01	-0.00444	0.00355	2.10E-01	-0.02873	0.01164	1.36E-02
cg20939319	10	ENSG00000104687	0.00268	0.00264	3.11E-01	-0.00029	0.00634	9.63E-01	-0.00374	0.00963	6.98E-01	0.00436	0.00317	1.70E-01	-0.00020	0.01093	9.86E-01
cg10012512	7	ENSG00000009335	-0.00030	0.00040	4.49E-01	0.00044	0.00085	6.09E-01	-0.00126	0.00144	3.81E-01	-0.00038	0.00049	4.43E-01	-0.00102	0.00175	5.58E-01
cg03703840	14	ENSG00000166012	0.00028	0.00023	2.25E-01	0.00044	0.00057	4.33E-01	0.00083	0.00059	1.62E-01	0.00022	0.00033	5.03E-01	-0.00020	0.00058	7.27E-01
cg03224276	13	ENSG00000102984	-0.00147	0.00126	2.45E-01	-0.00204	0.00192	2.87E-01	-0.00236	0.00152	1.20E-01	0.00135	0.00099	1.72E-01	-0.00328	0.00114	4.04E-03
cg10012512	7	ENSG00000105993	0.00108	0.00146	4.61E-01	-0.00078	0.00271	7.73E-01	0.00701	0.00413	8.97E-02	0.00021	0.00161	8.94E-01	0.00558	0.00592	3.46E-01
cg25556432	15	ENSG00000132330	0.00099	0.00081	2.22E-01	0.00380	0.00187	4.18E-02	0.00088	0.00292	7.64E-01	0.00025	0.00100	8.05E-01	0.00059	0.00297	8.42E-01
cg22742965	4	ENSG00000173559	-0.00030	0.00144	8.33E-01	0.00241	0.00312	4.40E-01	-0.00546	0.00412	1.85E-01	-0.00063	0.00189	7.39E-01	0.00252	0.00490	6.07E-01
cg01157143	9	ENSG00000151116	-0.00150	0.00169	3.74E-01	0.00326	0.00363	3.69E-01	-0.00163	0.00529	7.58E-01	-0.00287	0.00217	1.85E-01	-0.00379	0.00604	5.31E-01
cg11749902	11	ENSG00000083168	0.00325	0.00319	3.08E-01	0.00949	0.01051	3.66E-01	-0.00470	0.01328	7.24E-01	0.00242	0.00357	4.98E-01	0.01385	0.01399	3.22E-01
cg16734845	25	ENSG00000166710	0.00254	0.00171	1.37E-01	0.00377	0.00312	2.27E-01	-0.00137	0.00335	6.83E-01	0.00556	0.00347	1.10E-01	0.00213	0.00384	5.79E-01
cg03224276	13	ENSG00000102967	0.00022	0.00020	2.65E-01	0.00012	0.00058	8.33E-01	0.00096	0.00065	1.37E-01	0.00015	0.00025	5.58E-01	0.00014	0.00045	7.53E-01
cg02207312	37	ENSG00000167985	-0.00052	0.00031	9.31E-02	-0.00007	0.00006	2.11E-01	-0.00163	0.00092	7.55E-02	-0.00010	0.00027	6.99E-01	-0.00144	0.00044	1.07E-03
cg02206852	52	ENSG00000167536	-0.00051	0.00028	6.66E-02	-0.00048	0.00029	9.29E-02	-0.00152	0.00159	3.42E-01	-0.00066	0.00289	8.19E-01	-0.00017	0.00558	9.75E-01
cg20939319	10	ENSG00000177669	0.00231	0.00245	3.47E-01	-0.00293	0.00725	6.85E-01	0.01079	0.01250	3.88E-01	0.00270	0.00271	3.19E-01	0.00051	0.01456	9.72E-01
cg21614201	13	ENSG00000260091	0.00261	0.00236	2.69E-01	0.00907	0.00480	5.89E-02	0.00123	0.00595	8.37E-01	0.00006	0.00170	9.73E-01	0.00837	0.00775	2.80E-01
cg02885771	11	ENSG00000268402	0.00403	0.00405	3.20E-01	-0.00359	0.00908	6.92E-01	0.01147	0.01058	2.78E-01	0.00703	0.00570	2.17E-01	-0.00332	0.01054	7.52E-01
cg07148038	89	ENSG00000204348	-0.00522	0.00254	3.99E-02	-0.00466	0.00666	4.84E-01	0.00597	0.00736	4.17E-01	-0.00766	0.00347	2.73E-02	-0.00574	0.00568	3.12E-01
cg12064372	10	ENSG00000188375	0.00058	0.00062	3.55E-01	-0.00038	0.00125	7.62E-01	-0.00061	0.00242	8.01E-01	0.00104	0.00077	1.79E-01	0.00108	0.00345	7.55E-01
cg03703840	14	ENSG00000134627	0.00025	0.00022	2.54E-01	0.00022	0.00044	6.22E-01	-0.00013	0.00051	7.93E-01	0.00017	0.00041	6.73E-01	0.00062	0.00042	1.39E-01
cg20939319	10	ENSG00000157110	-0.00215	0.00234	3.57E-01	0.00304	0.00458	5.07E-01	-0.00922	0.00688	1.80E-01	-0.00344	0.00313	2.71E-01	0.00065	0.00917	9.43E-01
cg02207312	37	ENSG00000257052	-0.00076	0.00046	9.69E-02	-0.00257	0.00105	1.49E-02	-0.00096	0.00057	9.10E-02	-0.00074	0.00043	8.90E-02	0.00041	0.00063	5.14E-01
cg08065963	12	ENSG00000260349	0.00604	0.00582	2.99E-01	-0.00235	0.00837	7.79E-01	-0.00460	0.01357	7.35E-01	0.00686	0.00430	1.10E-01	0.03317	0.01465	2.36E-02
cg12064372	10	ENSG00000223722	0.00032	0.00035	3.61E-01	-0.00049	0.00063	4.32E-01	0.00113	0.00095	2.35E-01	0.00056	0.00050	2.60E-01	0.00090	0.00160	5.75E-01
cg01157143	9	ENSG00000179119	0.00252	0.00300	4.02E-01	-0.00029	0.00726	9.68E-01	-0.00277	0.00771	7.20E-01	0.00436	0.00400	2.76E-01	0.00463	0.00886	6.01E-01
cg02885771	11	ENSG00000034693	0.00209	0.00216	3.32E-01	0.00209	0.00528	6.92E-01	-0.00096	0.00566	8.66E-01	0.00357	0.00310	2.49E-01	0.00076	0.00480	8.75E-01
cg23396786	18	ENSG00000204872	-0.00373	0.00293	2.03E-01	-0.02372	0.01211	5.02E-02	0.01420	0.01745	4.16E-01	-0.00333	0.00407	4.13E-01	-0.00290	0.00254	2.54E-01

cg21614201	13	ENSG00000260404	0.00293	0.00273	2.82E-01	0.01085	0.00547	4.73E-02	0.00429	0.00235	6.72E-02	-0.00315	0.00224	1.59E-01	0.00412	0.00366	2.60E-01
cg06982745	18	ENSG00000172731	0.00212	0.00167	2.05E-01	0.00341	0.00334	3.07E-01	0.00421	0.00243	8.39E-02	-0.00096	0.00147	5.15E-01	0.00505	0.00357	1.57E-01
cg25556432	15	ENSG00000144488	-0.00345	0.00298	2.47E-01	-0.00508	0.00853	5.52E-01	0.00736	0.01082	4.96E-01	-0.00309	0.00348	3.74E-01	-0.01620	0.01135	1.53E-01
cg05304461	26	ENSG00000162444	-0.00031	0.00021	1.43E-01	-0.00067	0.00043	1.17E-01	-0.00064	0.00071	3.67E-01	-0.00005	0.00027	8.45E-01	-0.00106	0.00092	2.49E-01
cg03703840	14	ENSG00000166004	-0.00014	0.00013	2.68E-01	-0.00005	0.00016	7.65E-01	0.00009	0.00020	6.57E-01	-0.00078	0.00037	3.46E-02	-0.00021	0.00016	1.87E-01
cg05946118	12	ENSG00000262944	-0.00387	0.00387	3.18E-01	-0.01189	0.00591	4.43E-02	-0.00243	0.00816	7.66E-01	-0.00414	0.00403	3.05E-01	0.01158	0.00961	2.28E-01
cg07160694	13	ENSG00000242071	-0.00022	0.00021	3.00E-01	-0.00020	0.00052	7.04E-01	0.00024	0.00038	5.24E-01	-0.00050	0.00031	1.04E-01	-0.00035	0.00075	6.37E-01
cg19734370	32	ENSG00000262580	0.00026	0.00017	1.22E-01	0.00010	0.00028	7.18E-01	0.00024	0.00056	6.60E-01	0.00039	0.00023	9.07E-02	-0.00395	0.00376	2.93E-01
cg17075019	4	ENSG00000156113	-0.00003	0.00191	9.89E-01	-0.00289	0.00521	5.79E-01	0.00580	0.00606	3.38E-01	-0.00030	0.00236	8.98E-01	-0.00013	0.00570	9.81E-01
cg23396786	18	ENSG00000116127	0.00566	0.00463	2.22E-01	0.00189	0.00584	7.47E-01	-0.00919	0.00898	3.06E-01	0.00780	0.00313	1.27E-02	0.02180	0.00907	1.62E-02
cg05304461	26	ENSG00000054523	-0.00041	0.00029	1.55E-01	-0.00002	0.00078	9.77E-01	-0.00030	0.00121	8.05E-01	-0.00062	0.00033	6.36E-02	0.00114	0.00115	3.23E-01
cg05304461	26	ENSG00000198793	0.00052	0.00037	1.55E-01	0.00116	0.00080	1.44E-01	0.00152	0.00127	2.32E-01	0.00047	0.00044	2.86E-01	-0.00088	0.00102	3.87E-01
cg02206852	52	ENSG00000109111	0.00184	0.00104	7.77E-02	0.00143	0.00176	4.17E-01	-0.00313	0.00441	4.78E-01	0.00222	0.00153	1.49E-01	0.00375	0.00289	1.95E-01
cg06982745	18	ENSG00000079332	-0.00099	0.00081	2.25E-01	-0.00044	0.00177	8.03E-01	-0.00305	0.00124	1.43E-02	0.00028	0.00081	7.29E-01	-0.00121	0.00140	3.87E-01
cg23396786	18	ENSG00000116096	0.00731	0.00603	2.25E-01	-0.00940	0.01047	3.69E-01	0.02651	0.01395	5.74E-02	0.00718	0.00443	1.05E-01	0.01439	0.01452	3.22E-01
cg01157143	9	ENSG00000151117	-0.00283	0.00375	4.51E-01	-0.00255	0.00714	7.21E-01	0.01478	0.01108	1.82E-01	-0.00673	0.00379	7.54E-02	0.00024	0.01170	9.84E-01
cg09108394	15	ENSG00000004779	0.00060	0.00055	2.72E-01	0.00053	0.00120	6.61E-01	0.00005	0.00195	9.78E-01	0.00087	0.00069	2.11E-01	-0.00067	0.00190	7.26E-01
cg12064372	10	ENSG00000013573	0.00008	0.00010	4.12E-01	-0.00008	0.00019	6.72E-01	0.00032	0.00039	4.12E-01	0.00016	0.00013	2.13E-01	-0.00032	0.00046	4.78E-01
cg12064372	10	ENSG00000177359	0.00038	0.00047	4.14E-01	-0.00033	0.00097	7.35E-01	0.00207	0.00159	1.94E-01	0.00035	0.00058	5.50E-01	0.00135	0.00227	5.51E-01
cg03703840	14	ENSG00000234106	-0.00025	0.00024	2.96E-01	0.00023	0.00088	7.92E-01	-0.00225	0.00107	3.63E-02	-0.00007	0.00013	5.98E-01	-0.00037	0.00029	2.02E-01
cg21614201	13	ENSG00000145388	-0.00101	0.00103	3.27E-01	-0.00410	0.00283	1.47E-01	0.00056	0.00227	8.05E-01	-0.00055	0.00143	7.01E-01	-0.00207	0.00272	4.46E-01
cg02885771	11	ENSG00000189007	-0.00339	0.00401	3.97E-01	-0.00158	0.01024	8.77E-01	-0.00383	0.01509	8.00E-01	-0.00298	0.00484	5.38E-01	-0.00919	0.01328	4.89E-01
cg08065963	12	ENSG00000260979	-0.00288	0.00317	3.64E-01	-0.00372	0.00426	3.83E-01	-0.00561	0.00540	2.99E-01	-0.00654	0.00285	2.15E-02	0.01135	0.00689	9.97E-02
cg09108394	15	ENSG00000122257	-0.00331	0.00315	2.92E-01	-0.00125	0.00599	8.35E-01	-0.01730	0.00823	3.55E-02	-0.00064	0.00320	8.41E-01	-0.00416	0.00953	6.62E-01
cg20066227	9	ENSG00000165983	0.00106	0.00153	4.90E-01	0.00287	0.00334	3.90E-01	0.00073	0.00362	8.40E-01	0.00186	0.00219	3.96E-01	-0.00490	0.00442	2.68E-01
cg10012512	7	ENSG00000146909	0.00110	0.00236	6.40E-01	0.00291	0.00526	5.81E-01	0.00426	0.00875	6.26E-01	-0.00035	0.00285	9.03E-01	0.01091	0.01165	3.49E-01
cg07148038	89	ENSG00000234745	-0.00214	0.00109	5.04E-02	-0.00071	0.00422	8.67E-01	-0.00655	0.00275	1.74E-02	-0.00145	0.00145	3.16E-01	-0.00124	0.00219	5.73E-01
cg10034572	8	ENSG00000054219	0.00029	0.00050	5.64E-01	-0.00077	0.00083	3.55E-01	0.00230	0.00146	1.17E-01	0.00053	0.00048	2.64E-01	-0.00022	0.00139	8.71E-01
cg03703840	14	ENSG00000168876	0.00028	0.00029	3.23E-01	0.00073	0.00069	2.90E-01	0.00037	0.00074	6.16E-01	0.00043	0.00043	3.11E-01	-0.00041	0.00060	4.96E-01
cg20102034	37	ENSG00000235499	-0.00098	0.00064	1.23E-01	-0.00575	0.00638	3.68E-01	-0.00721	0.00720	3.17E-01	-0.00086	0.00065	1.81E-01	-0.00371	0.00765	6.28E-01
cg02206852	52	ENSG00000132591	0.00225	0.00132	8.79E-02	0.00438	0.00381	2.50E-01	0.00173	0.00168	3.02E-01	0.00028	0.00055	6.10E-01	0.00482	0.00149	1.22E-03
cg05304461	26	ENSG00000130940	0.00056	0.00042	1.76E-01	0.00082	0.00087	3.46E-01	-0.00027	0.00156	8.64E-01	0.00088	0.00052	8.88E-02	-0.00154	0.00138	2.65E-01
cg19734370	32	ENSG00000167291	0.00158	0.00108	1.44E-01	0.00269	0.00155	8.16E-02	-0.00033	0.00358	9.27E-01	0.00100	0.00170	5.57E-01	-0.00459	0.00716	5.22E-01
cg03077331	45	ENSG00000183010	-0.00024	0.00015	1.04E-01	-0.00004	0.00047	9.34E-01	-0.00018	0.00020	3.48E-01	-0.00041	0.00027	1.35E-01	-0.00119	0.00182	5.13E-01
cg01157143	9	ENSG00000177054	0.00088	0.00139	5.27E-01	0.00560	0.00465	2.28E-01	-0.00169	0.00218	4.38E-01	0.00079	0.00344	8.18E-01	0.00269	0.00235	2.53E-01
cg22127773	80	ENSG00000040633	-0.00082	0.00044	5.96E-02	-0.00118	0.00078	1.29E-01	0.00085	0.00116	4.65E-01	-0.00104	0.00060	8.19E-02	-0.00147	0.00405	7.17E-01
cg05304461	26	ENSG00000011021	-0.00027	0.00021	1.84E-01	-0.00045	0.00046	3.24E-01	-0.00016	0.00061	7.90E-01	-0.00041	0.00028	1.45E-01	0.00040	0.00054	4.67E-01
cg23396786	18	ENSG00000235499	0.00318	0.00289	2.71E-01	0.00873	0.00799	2.75E-01	0.00063	0.01240	9.60E-01	0.00394	0.00282	1.62E-01	-0.01534	0.01156	1.85E-01
cg20939319	10	ENSG00000104695	-0.00223	0.00322	4.89E-01	0.00364	0.00704	6.05E-01	0.00148	0.01227	9.04E-01	-0.00517	0.00397	1.93E-01	0.00495	0.01291	7.02E-01
cg19734370	32	ENSG00000167302	-0.00165	0.00115	1.54E-01	-0.00012	0.00078	8.82E-01	-0.00486	0.00143	6.75E-04	-0.00136	0.00112	2.26E-01	0.00053	0.00342	8.77E-01
cg02206852	52	ENSG00000196535	-0.00671	0.00401	9.45E-02	-0.01360	0.00471	3.85E-03	-0.00036	0.00124	7.68E-01	-0.01101	0.00392	5.04E-03	-0.00268	0.00786	7.33E-01
cg07160694	13	ENSG00000100647	0.00019	0.00021	3.79E-01	0.00017	0.00051	7.35E-01	-0.00123	0.00090	1.69E-01	0.00033	0.00025	1.97E-01	-0.00006	0.00085	9.44E-01

cg02206852	52	ENSG00000109107	-0.00264	0.00158	9.50E-02	-0.00226	0.00337	5.03E-01	-0.00530	0.00663	4.24E-01	-0.00258	0.00194	1.85E-01	-0.00225	0.00651	7.30E-01
cg02207312	37	ENSG00000183134	-0.00004	0.00003	1.35E-01	-0.00007	0.00018	7.14E-01	-0.00003	0.00004	4.13E-01	-0.00008	0.00006	1.91E-01	-0.00009	0.00016	5.70E-01
cg08065963	12	ENSG00000184857	0.00094	0.00117	4.21E-01	0.00354	0.00145	1.46E-02	0.00146	0.00249	5.56E-01	-0.00005	0.00094	9.55E-01	-0.00217	0.00261	4.07E-01
cg05304461	26	ENSG00000175262	0.00012	0.00009	1.96E-01	0.00002	0.00008	8.27E-01	-0.00001	0.00012	9.27E-01	0.00030	0.00015	4.17E-02	0.00053	0.00028	5.75E-02
cg01157143	9	ENSG00000109854	-0.00100	0.00175	5.68E-01	-0.00253	0.00375	4.99E-01	-0.00369	0.00436	3.97E-01	0.00007	0.00253	9.77E-01	0.00081	0.00465	8.62E-01
cg06982745	18	ENSG00000138316	-0.00024	0.00022	2.85E-01	0.00019	0.00060	7.49E-01	-0.00065	0.00064	3.11E-01	-0.00011	0.00028	6.97E-01	-0.00118	0.00071	9.69E-02
cg05946118	12	ENSG00000184857	-0.00046	0.00059	4.35E-01	-0.00002	0.00120	9.85E-01	0.00150	0.00206	4.67E-01	-0.00073	0.00076	3.35E-01	-0.00171	0.00207	4.08E-01
cg10034572	8	ENSG00000153250	0.00056	0.00125	6.55E-01	0.00033	0.00265	9.02E-01	0.00024	0.00431	9.56E-01	-0.00008	0.00157	9.60E-01	0.00785	0.00487	1.07E-01
cg02137691	31	ENSG00000185049	-0.00856	0.00623	1.70E-01	-0.01784	0.00992	7.23E-02	-0.01981	0.00953	3.76E-02	-0.01015	0.00428	1.77E-02	0.00876	0.00674	1.94E-01
cg20939319	10	ENSG00000104671	-0.00116	0.00183	5.28E-01	0.00095	0.00372	7.98E-01	-0.00249	0.00498	6.16E-01	-0.00220	0.00262	4.02E-01	0.00020	0.00509	9.68E-01
cg03703840	14	ENSG00000256745	-0.00011	0.00013	3.77E-01	0.00140	0.00089	1.17E-01	-0.00051	0.00093	5.80E-01	-0.00011	0.00034	7.48E-01	-0.00015	0.00012	2.21E-01
cg08065963	12	ENSG00000153048	0.00269	0.00350	4.41E-01	0.00693	0.00746	3.53E-01	0.01030	0.01011	3.09E-01	0.00279	0.00327	3.93E-01	-0.01518	0.01096	1.66E-01
cg21614201	13	ENSG00000269893	0.00160	0.00194	4.11E-01	-0.00135	0.00208	5.16E-01	0.00980	0.00413	1.76E-02	0.00131	0.00147	3.74E-01	-0.00054	0.00621	9.31E-01
cg05946118	12	ENSG00000260349	0.00257	0.00339	4.48E-01	-0.00804	0.00674	2.33E-01	0.00421	0.01143	7.13E-01	0.00552	0.00346	1.11E-01	0.00770	0.01205	5.23E-01
cg07160694	13	ENSG00000081177	0.00020	0.00025	4.15E-01	-0.00050	0.00047	2.87E-01	0.00019	0.00072	7.92E-01	0.00049	0.00021	1.64E-02	0.00008	0.00072	9.17E-01
cg18387671	42	ENSG00000167543	0.00009	0.00006	1.29E-01	0.00036	0.00051	4.76E-01	-0.00018	0.00048	7.13E-01	0.00010	0.00006	9.49E-02	-0.00002	0.00016	8.77E-01
cg01157143	9	ENSG00000129173	-0.00129	0.00253	6.10E-01	-0.00396	0.00640	5.36E-01	0.00246	0.00858	7.75E-01	-0.00131	0.00304	6.67E-01	0.00033	0.01001	9.73E-01
cg07957088	43	ENSG00000101246	-0.00172	0.00114	1.30E-01	0.00085	0.00554	8.78E-01	-0.00631	0.00909	4.87E-01	-0.00160	0.00139	2.49E-01	-0.00219	0.00221	3.22E-01
cg05304461	26	ENSG00000177674	0.00032	0.00026	2.18E-01	0.00009	0.00049	8.57E-01	0.00153	0.00171	3.71E-01	0.00047	0.00035	1.72E-01	-0.00011	0.00081	8.94E-01
cg02206852	52	ENSG00000168792	0.00244	0.00153	1.10E-01	-0.00422	0.00423	3.19E-01	0.00448	0.00239	6.10E-02	0.00351	0.00170	3.91E-02	0.00015	0.00382	9.68E-01
cg02137691	31	ENSG00000063978	-0.00400	0.00302	1.85E-01	-0.00770	0.00493	1.19E-01	-0.01233	0.00456	6.82E-03	0.00000	0.00274	9.99E-01	0.00081	0.00361	8.23E-01
cg05946118	12	ENSG00000140650	0.00180	0.00254	4.79E-01	0.00681	0.00344	4.75E-02	0.00589	0.00422	1.63E-01	-0.00217	0.00220	3.24E-01	-0.00175	0.00420	6.77E-01
cg02137691	31	ENSG00000168884	0.00279	0.00211	1.87E-01	0.00755	0.00240	1.65E-03	0.00217	0.00319	4.96E-01	-0.00150	0.00208	4.70E-01	0.00324	0.00270	2.29E-01
cg03224276	13	ENSG00000140836	-0.00019	0.00025	4.48E-01	0.00047	0.00064	4.61E-01	0.00012	0.00075	8.68E-01	-0.00051	0.00033	1.24E-01	0.00007	0.00060	9.00E-01
cg16734845	25	ENSG00000167004	-0.00311	0.00261	2.33E-01	-0.00630	0.00647	3.30E-01	-0.01272	0.00755	9.21E-02	0.00109	0.00214	6.11E-01	-0.00455	0.00312	1.45E-01
cg07957088	43	ENSG00000197457	0.00106	0.00071	1.36E-01	0.00092	0.00086	2.79E-01	0.00169	0.00143	2.36E-01	0.00077	0.00329	8.16E-01	-0.00400	0.00741	5.89E-01
cg07160694	13	ENSG00000100632	-0.00020	0.00027	4.56E-01	-0.00039	0.00026	1.33E-01	-0.00115	0.00043	7.83E-03	0.00003	0.00015	8.25E-01	0.00061	0.00043	1.63E-01
cg03077331	45	ENSG00000141556	0.00046	0.00030	1.32E-01	-0.00189	0.00200	3.43E-01	0.00096	0.00087	2.70E-01	0.00043	0.00034	1.98E-01	0.00099	0.00180	5.82E-01
cg06982745	18	ENSG00000107731	0.00153	0.00159	3.35E-01	0.00555	0.00326	8.85E-02	0.00041	0.00328	9.02E-01	-0.00054	0.00122	6.60E-01	0.00476	0.00354	1.79E-01
cg08065963	12	ENSG00000067365	-0.00038	0.00058	5.04E-01	-0.00042	0.00133	7.53E-01	0.00077	0.00217	7.24E-01	-0.00059	0.00071	4.04E-01	0.00033	0.00195	8.64E-01
cg16734845	25	ENSG00000242028	-0.00229	0.00196	2.42E-01	-0.00041	0.00339	9.03E-01	-0.00228	0.00359	5.24E-01	-0.00532	0.00338	1.15E-01	0.01216	0.01198	3.10E-01
cg02206852	52	ENSG00000004139	0.00067	0.00043	1.17E-01	-0.00028	0.00110	7.96E-01	0.00180	0.00159	2.60E-01	0.00057	0.00052	2.76E-01	0.00221	0.00143	1.21E-01
cg07160694	13	ENSG00000139990	0.00013	0.00018	4.66E-01	-0.00004	0.00041	9.24E-01	0.00071	0.00051	1.59E-01	-0.00001	0.00023	9.59E-01	0.00064	0.00061	2.91E-01
cg22127773	80	ENSG00000170037	0.00028	0.00016	7.67E-02	0.00030	0.00016	6.57E-02	0.00051	0.00213	8.12E-01	-0.00010	0.00085	9.08E-01	-0.00258	0.00383	5.01E-01
cg10012512	7	ENSG00000244291	-0.00031	0.00209	8.81E-01	0.00373	0.00341	2.74E-01	0.00097	0.00618	8.75E-01	-0.00151	0.00225	5.02E-01	-0.01132	0.00815	1.65E-01
cg03077331	45	ENSG00000169660	-0.00146	0.00098	1.37E-01	0.00232	0.00428	5.88E-01	-0.00300	0.00135	2.66E-02	-0.00056	0.00046	2.20E-01	-0.00466	0.00328	1.55E-01
cg20066227	9	ENSG00000107614	-0.00104	0.00261	6.91E-01	0.00749	0.00467	1.09E-01	-0.00340	0.00500	4.96E-01	-0.00395	0.00189	3.60E-02	-0.00104	0.00539	8.47E-01
cg02207312	37	ENSG00000214787	-0.00017	0.00012	1.68E-01	-0.00102	0.00052	5.25E-02	-0.00007	0.00020	7.19E-01	-0.00017	0.00018	3.56E-01	-0.00008	0.00031	7.92E-01
cg03224276	13	ENSG00000140829	0.00053	0.00075	4.80E-01	0.00224	0.00181	2.16E-01	-0.00201	0.00188	2.86E-01	0.00111	0.00083	1.83E-01	-0.00046	0.00153	7.65E-01
cg21614201	13	ENSG00000225892	-0.00055	0.00079	4.90E-01	0.00110	0.00353	7.56E-01	-0.00059	0.00110	5.93E-01	0.00038	0.00049	4.31E-01	-0.00293	0.00137	3.24E-02
cg22127773	80	ENSG00000072818	-0.00071	0.00040	8.01E-02	-0.00070	0.00075	3.48E-01	-0.00097	0.00141	4.90E-01	-0.00060	0.00051	2.43E-01	-0.00683	0.00465	1.42E-01
cg06982745	18	ENSG00000229097	-0.00090	0.00097	3.57E-01	0.00050	0.00088	5.74E-01	-0.00461	0.00145	1.51E-03	0.00050	0.00078	5.24E-01	-0.00109	0.00108	3.11E-01

cg03224276	13	ENSG00000259901	0.00118	0.00174	4.97E-01	-0.00040	0.00323	9.01E-01	0.00072	0.00293	8.07E-01	-0.00122	0.00179	4.95E-01	0.00593	0.00248	1.66E-02
cg01157143	9	ENSG00000074319	0.00019	0.00052	7.20E-01	0.00014	0.00114	9.03E-01	0.00216	0.00179	2.26E-01	0.00012	0.00065	8.55E-01	-0.00117	0.00178	5.12E-01
cg22127773	80	ENSG00000213876	-0.00111	0.00064	8.21E-02	-0.00150	0.00095	1.17E-01	0.00329	0.00335	3.27E-01	-0.00108	0.00090	2.30E-01	-0.00116	0.00533	8.27E-01
cg03224276	13	ENSG00000257017	0.00047	0.00070	5.05E-01	-0.00058	0.00188	7.57E-01	0.00206	0.00164	2.08E-01	0.00022	0.00101	8.26E-01	0.00032	0.00157	8.40E-01
cg12064372	10	ENSG00000110888	0.00015	0.00034	6.64E-01	-0.00007	0.00064	9.08E-01	0.00060	0.00090	5.02E-01	0.00037	0.00049	4.50E-01	-0.00121	0.00120	3.13E-01
cg03077331	45	ENSG00000141574	-0.00237	0.00164	1.48E-01	-0.00306	0.00579	5.97E-01	-0.00386	0.00190	4.24E-02	-0.00007	0.00101	9.43E-01	-0.00798	0.00463	8.50E-02
cg12064372	10	ENSG00000133704	0.00035	0.00081	6.68E-01	-0.00159	0.00108	1.40E-01	0.00243	0.00179	1.76E-01	0.00076	0.00059	1.96E-01	0.00110	0.00263	6.75E-01
cg27158523	11	ENSG00000178199	-0.00022	0.00044	6.14E-01	0.00002	0.00079	9.76E-01	-0.00112	0.00134	4.06E-01	0.00014	0.00036	7.04E-01	-0.00273	0.00156	8.12E-02
cg22127773	80	ENSG00000161944	0.00108	0.00062	8.45E-02	0.00213	0.00135	1.15E-01	-0.00001	0.00326	9.96E-01	0.00103	0.00074	1.64E-01	-0.00351	0.00348	3.13E-01
cg02885771	11	ENSG00000169976	-0.00341	0.00685	6.19E-01	0.00473	0.00806	5.57E-01	-0.02927	0.00965	2.42E-03	0.00406	0.00369	2.70E-01	0.00128	0.00898	8.87E-01
cg03224276	13	ENSG00000263320	-0.00042	0.00066	5.27E-01	0.00092	0.00136	4.98E-01	-0.00020	0.00121	8.66E-01	0.00008	0.00046	8.62E-01	-0.00251	0.00105	1.73E-02
cg07148038	89	ENSG00000213676	-0.00134	0.00076	7.74E-02	-0.00127	0.00078	1.05E-01	0.00515	0.00824	5.32E-01	-0.00329	0.00349	3.46E-01	-0.00433	0.00798	5.87E-01
cg05304461	26	ENSG00000204624	-0.00013	0.00011	2.66E-01	-0.00141	0.00092	1.28E-01	-0.00010	0.00012	4.03E-01	-0.00031	0.00049	5.22E-01	0.00066	0.00158	6.76E-01
cg07148038	89	ENSG00000204315	0.00116	0.00066	7.78E-02	0.00090	0.00352	7.99E-01	0.00185	0.00395	6.39E-01	0.00052	0.00055	3.40E-01	0.00298	0.00122	1.46E-02
cg16734845	25	ENSG00000137770	0.00143	0.00133	2.81E-01	-0.00022	0.00305	9.44E-01	0.00516	0.00382	1.77E-01	0.00149	0.00167	3.71E-01	-0.00158	0.00546	7.72E-01
cg19734370	32	ENSG00000141527	0.00092	0.00075	2.20E-01	0.00003	0.00079	9.68E-01	0.00328	0.00137	1.63E-02	0.00030	0.00108	7.78E-01	0.00144	0.00256	5.74E-01
cg12064372	10	ENSG00000151743	-0.00005	0.00014	7.04E-01	0.00026	0.00022	2.24E-01	-0.00021	0.00044	6.36E-01	-0.00021	0.00017	2.18E-01	-0.00034	0.00061	5.72E-01
cg23396786	18	ENSG00000135624	-0.00317	0.00371	3.93E-01	-0.00710	0.00749	3.43E-01	0.00922	0.01040	3.75E-01	-0.00438	0.00508	3.89E-01	-0.00280	0.01220	8.19E-01
cg25884324	20	ENSG00000183208	0.00066	0.00071	3.56E-01	0.00005	0.00159	9.76E-01	0.00195	0.00175	2.65E-01	0.00007	0.00101	9.42E-01	0.00210	0.00192	2.73E-01
cg12064372	10	ENSG00000170456	0.00017	0.00046	7.12E-01	-0.00057	0.00078	4.65E-01	0.00122	0.00129	3.43E-01	0.00030	0.00066	6.54E-01	0.00151	0.00216	4.85E-01
cg27158523	11	ENSG00000055211	0.00008	0.00018	6.48E-01	0.00031	0.00040	4.41E-01	-0.00028	0.00050	5.75E-01	0.00009	0.00023	7.01E-01	0.00004	0.00057	9.41E-01
cg02206852	52	ENSG00000265205	-0.00233	0.00157	1.38E-01	-0.00146	0.00328	6.57E-01	-0.00694	0.00754	3.58E-01	-0.00153	0.00192	4.26E-01	-0.01195	0.00664	7.19E-02
cg07160694	13	ENSG00000185650	0.00022	0.00037	5.54E-01	-0.00023	0.00027	3.99E-01	0.00180	0.00095	5.94E-02	-0.00023	0.00027	4.03E-01	0.00165	0.00096	8.56E-02
cg06982745	18	ENSG00000198246	-0.00094	0.00112	4.00E-01	-0.00306	0.00162	5.82E-02	-0.00292	0.00222	1.88E-01	0.00066	0.00089	4.58E-01	0.00054	0.00239	8.22E-01
cg09108394	15	ENSG00000103356	-0.00229	0.00328	4.84E-01	0.00487	0.00735	5.08E-01	-0.00037	0.01157	9.74E-01	-0.00514	0.00406	2.05E-01	0.00206	0.01289	8.73E-01
cg06982745	18	ENSG00000156521	-0.00067	0.00081	4.04E-01	0.00197	0.00266	4.60E-01	-0.00018	0.00235	9.39E-01	-0.00094	0.00097	3.34E-01	-0.00194	0.00263	4.60E-01
cg20102034	37	ENSG00000115318	0.00473	0.00367	1.97E-01	0.00604	0.00854	4.80E-01	0.00751	0.01192	5.29E-01	0.00497	0.00469	2.89E-01	-0.00132	0.01121	9.06E-01
cg10034572	8	ENSG00000123636	-0.00020	0.00192	9.18E-01	0.00156	0.00275	5.70E-01	-0.00549	0.00350	1.17E-01	0.00280	0.00185	1.30E-01	-0.00303	0.00365	4.07E-01
cg16734845	25	ENSG00000171766	-0.00109	0.00104	2.96E-01	-0.00121	0.00156	4.39E-01	-0.00150	0.00211	4.77E-01	-0.00417	0.00343	2.24E-01	0.00088	0.00220	6.88E-01
cg25884324	20	ENSG00000228998	0.00092	0.00102	3.71E-01	0.00161	0.00080	4.52E-02	-0.00491	0.00331	1.38E-01	0.00330	0.00170	5.27E-02	-0.00003	0.00111	9.77E-01
cg20066227	9	ENSG00000026025	0.00007	0.00033	8.41E-01	-0.00092	0.00084	2.76E-01	-0.00040	0.00106	7.04E-01	0.00019	0.00041	6.43E-01	0.00098	0.00093	2.90E-01
cg05946118	12	ENSG00000153048	-0.00153	0.00319	6.31E-01	-0.01061	0.00601	7.75E-02	-0.00487	0.00861	5.72E-01	0.00180	0.00266	4.99E-01	0.00298	0.00899	7.40E-01
cg27158523	11	ENSG00000131023	-0.00017	0.00044	6.90E-01	-0.00004	0.00061	9.47E-01	0.00098	0.00089	2.73E-01	-0.00007	0.00027	8.01E-01	-0.00230	0.00106	3.09E-02
cg20066227	9	ENSG00000148481	-0.00021	0.00107	8.48E-01	0.00242	0.00265	3.62E-01	-0.00094	0.00313	7.64E-01	-0.00080	0.00137	5.61E-01	-0.00002	0.00325	9.95E-01
cg07148038	89	ENSG00000204438	0.00239	0.00139	8.59E-02	0.00298	0.00160	6.20E-02	-0.00254	0.00628	6.86E-01	0.00062	0.00335	8.54E-01	0.00777	0.01016	4.45E-01
cg19734370	32	ENSG00000226137	-0.00013	0.00011	2.39E-01	-0.00017	0.00012	1.48E-01	0.00007	0.00027	7.88E-01	0.00108	0.00169	5.20E-01	-0.00492	0.00756	5.15E-01
cg11749902	11	ENSG00000147536	-0.00114	0.00294	7.00E-01	0.00573	0.00756	4.48E-01	0.00195	0.00323	5.47E-01	-0.00616	0.00404	1.27E-01	-0.01050	0.01164	3.67E-01
cg20939319	10	ENSG00000165392	-0.00085	0.00296	7.73E-01	-0.00646	0.00567	2.55E-01	-0.00501	0.00867	5.63E-01	0.00161	0.00413	6.97E-01	0.00682	0.00951	4.73E-01
cg25884324	20	ENSG00000166965	0.00047	0.00055	3.88E-01	0.00170	0.00055	1.95E-03	0.00025	0.00081	7.59E-01	-0.00042	0.00034	2.19E-01	0.00049	0.00073	5.03E-01
cg22127773	80	ENSG00000181284	0.00052	0.00031	9.88E-02	0.00117	0.00071	1.00E-01	0.00057	0.00047	2.26E-01	0.00021	0.00070	7.69E-01	-0.00002	0.00077	9.84E-01
cg21614201	13	ENSG00000150961	-0.00098	0.00192	6.10E-01	-0.00672	0.00272	1.34E-02	-0.00116	0.00275	6.72E-01	0.00013	0.00121	9.12E-01	0.00456	0.00356	2.00E-01
cg27158523	11	ENSG00000111962	-0.00019	0.00053	7.23E-01	-0.00146	0.00069	3.52E-02	0.00051	0.00101	6.15E-01	0.00042	0.00027	1.25E-01	-0.00051	0.00121	6.72E-01

cg19734370	32	ENSG00000141577	0.00054	0.00047	2.49E-01	-0.00009	0.00026	7.33E-01	0.00178	0.00065	6.47E-03	0.00086	0.00037	1.85E-02	-0.00102	0.00127	4.20E-01
cg23396786	18	ENSG00000144048	0.00115	0.00150	4.46E-01	-0.00052	0.00439	9.05E-01	0.00642	0.00560	2.52E-01	0.00121	0.00173	4.87E-01	-0.00275	0.00622	6.58E-01
cg16734845	25	ENSG00000140259	-0.00210	0.00212	3.21E-01	-0.00054	0.00389	8.89E-01	-0.00890	0.00444	4.50E-02	-0.00105	0.00266	6.92E-01	0.00484	0.00787	5.39E-01
cg20066227	9	ENSG00000229124	0.00056	0.00431	8.97E-01	0.01083	0.00529	4.05E-02	-0.00570	0.00678	4.01E-01	-0.00517	0.00253	4.08E-02	0.00425	0.00706	5.48E-01
cg20939319	10	ENSG00000104660	-0.00013	0.00054	8.10E-01	-0.00005	0.00113	9.66E-01	-0.00120	0.00208	5.63E-01	-0.00020	0.00068	7.64E-01	0.00116	0.00192	5.47E-01
cg03077331	45	ENSG00000176845	0.00053	0.00039	1.81E-01	0.00189	0.00091	3.76E-02	0.00030	0.00036	4.04E-01	-0.00073	0.00169	6.66E-01	0.00205	0.01052	8.45E-01
cg02137691	31	ENSG00000159733	0.00150	0.00134	2.63E-01	0.02118	0.00999	3.39E-02	0.01339	0.01102	2.24E-01	0.00090	0.00069	1.91E-01	0.00105	0.00136	4.41E-01
cg06218079	42	ENSG00000175711	-0.00054	0.00042	1.96E-01	0.00006	0.00066	9.25E-01	0.00037	0.00063	5.55E-01	-0.00054	0.00014	1.37E-04	-0.00214	0.00068	1.62E-03
cg07148038	89	ENSG00000227507	-0.00371	0.00221	9.27E-02	-0.00263	0.00414	5.25E-01	0.00030	0.00213	8.87E-01	-0.00497	0.00115	1.41E-05	-0.01626	0.00724	2.47E-02
cg07160694	13	ENSG00000175985	0.00008	0.00018	6.36E-01	0.00040	0.00053	4.53E-01	-0.00001	0.00065	9.87E-01	0.00012	0.00020	5.46E-01	-0.00065	0.00063	2.99E-01
cg02206852	52	ENSG00000109118	-0.00779	0.00554	1.59E-01	0.00356	0.00162	2.85E-02	-0.00977	0.00758	1.98E-01	-0.00738	0.00353	3.66E-02	-0.02333	0.00712	1.05E-03
cg21614201	13	ENSG00000145390	0.00069	0.00147	6.40E-01	-0.00462	0.00486	3.42E-01	0.00273	0.00454	5.47E-01	0.00093	0.00170	5.85E-01	0.00210	0.00586	7.20E-01
cg25556432	15	ENSG00000132329	0.00509	0.00862	5.55E-01	-0.01241	0.01049	2.37E-01	0.00505	0.01500	7.36E-01	0.00028	0.00439	9.49E-01	0.03961	0.01508	8.64E-03
cg06218079	42	ENSG00000187531	-0.00103	0.00080	1.98E-01	0.00036	0.00072	6.14E-01	0.00063	0.00087	4.73E-01	-0.00171	0.00029	6.01E-09	-0.00356	0.00102	4.78E-04
cg05304461	26	ENSG00000160049	-0.00026	0.00026	3.22E-01	-0.00019	0.00060	7.48E-01	-0.00127	0.00103	2.17E-01	-0.00017	0.00031	5.81E-01	-0.00050	0.00131	7.05E-01
cg02885771	11	ENSG00000135521	-0.00224	0.00749	7.65E-01	-0.00319	0.00990	7.47E-01	0.01674	0.01397	2.31E-01	-0.01545	0.00460	7.83E-04	0.00699	0.01200	5.60E-01
cg02206852	52	ENSG00000132581	0.00153	0.00109	1.62E-01	-0.00002	0.00061	9.79E-01	0.00002	0.00199	9.92E-01	0.00339	0.00104	1.14E-03	0.00305	0.00197	1.21E-01
cg02207312	37	ENSG00000110107	0.00014	0.00012	2.29E-01	0.00006	0.00013	6.57E-01	0.00045	0.00083	5.87E-01	0.00063	0.00032	4.61E-02	0.00000	0.00078	9.95E-01
cg07148038	89	ENSG00000204498	-0.00140	0.00084	9.63E-02	-0.00208	0.00377	5.81E-01	-0.00009	0.00157	9.52E-01	-0.00183	0.00109	9.31E-02	-0.00290	0.00346	4.01E-01
cg27158523	11	ENSG00000120256	0.00008	0.00030	7.83E-01	-0.00024	0.00076	7.51E-01	-0.00039	0.00126	7.59E-01	0.00011	0.00035	7.59E-01	0.00137	0.00140	3.29E-01
cg03224276	13	ENSG00000224470	0.00060	0.00140	6.67E-01	0.00623	0.00365	8.78E-02	-0.00071	0.00412	8.64E-01	-0.00075	0.00154	6.26E-01	0.00164	0.00332	6.21E-01
cg02206852	52	ENSG00000265625	-0.00238	0.00173	1.68E-01	-0.00357	0.00407	3.80E-01	-0.00409	0.00429	3.40E-01	-0.00214	0.00229	3.51E-01	0.00157	0.00579	7.87E-01
cg09108394	15	ENSG00000259806	-0.00055	0.00106	6.08E-01	0.00071	0.00284	8.03E-01	-0.00317	0.00360	3.78E-01	-0.00037	0.00126	7.67E-01	-0.00188	0.00460	6.82E-01
cg27158523	11	ENSG00000055208	-0.00001	0.00005	8.32E-01	-0.00010	0.00012	4.04E-01	0.00002	0.00021	9.28E-01	0.00000	0.00006	9.91E-01	0.00008	0.00021	6.94E-01
cg03224276	13	ENSG00000118557	0.00035	0.00096	7.14E-01	-0.00397	0.00321	2.16E-01	0.00013	0.00308	9.68E-01	0.00064	0.00117	5.83E-01	0.00175	0.00247	4.79E-01
cg07957088	43	ENSG00000101188	-0.00282	0.00228	2.17E-01	-0.00118	0.00620	8.50E-01	0.00674	0.00811	4.06E-01	-0.00291	0.00278	2.95E-01	-0.01091	0.00679	1.08E-01
cg16734845	25	ENSG00000205771	0.00029	0.00032	3.74E-01	0.00055	0.00054	3.02E-01	-0.00052	0.00061	3.92E-01	0.00067	0.00053	2.07E-01	-0.00383	0.00619	5.36E-01
cg09108394	15	ENSG00000103404	0.00146	0.00299	6.27E-01	-0.00118	0.00485	8.08E-01	-0.00150	0.00663	8.21E-01	0.00065	0.00364	8.58E-01	0.01758	0.00902	5.12E-02
cg23396786	18	ENSG00000135631	-0.00234	0.00366	5.22E-01	-0.00316	0.00926	7.32E-01	-0.00278	0.01303	8.31E-01	-0.00012	0.00444	9.78E-01	-0.01833	0.01262	1.46E-01
cg27158523	11	ENSG00000120265	-0.00002	0.00010	8.63E-01	0.00017	0.00014	2.16E-01	-0.00017	0.00024	4.70E-01	0.00001	0.00008	9.43E-01	-0.00045	0.00028	1.08E-01
cg02137691	31	ENSG00000178222	-0.00044	0.00044	3.09E-01	-0.00076	0.00168	6.54E-01	-0.00012	0.00203	9.53E-01	-0.00027	0.00058	6.40E-01	-0.00075	0.00078	3.39E-01
cg16734845	25	ENSG00000166762	0.00068	0.00078	3.83E-01	0.00051	0.00604	9.32E-01	0.00348	0.00810	6.68E-01	0.00108	0.00091	2.34E-01	-0.00063	0.00157	6.90E-01
cg07957088	43	ENSG00000171700	0.00031	0.00025	2.23E-01	0.00016	0.00033	6.34E-01	0.00028	0.00050	5.75E-01	0.00089	0.00064	1.66E-01	0.00274	0.00526	6.03E-01
cg25884324	20	ENSG00000198901	-0.00039	0.00056	4.84E-01	-0.00183	0.00152	2.29E-01	-0.00047	0.00113	6.77E-01	-0.00034	0.00075	6.52E-01	0.00320	0.00248	1.98E-01
cg02885771	11	ENSG00000146416	-0.00048	0.00320	8.81E-01	0.00924	0.00655	1.58E-01	-0.00327	0.00805	6.85E-01	-0.00440	0.00420	2.95E-01	0.00093	0.00861	9.14E-01
cg02885771	11	ENSG00000118495	-0.00031	0.00209	8.81E-01	0.00457	0.00507	3.67E-01	0.00199	0.00699	7.76E-01	-0.00226	0.00263	3.90E-01	0.00141	0.00623	8.21E-01
cg18387671	42	ENSG00000007202	-0.00005	0.00004	2.32E-01	-0.00008	0.00009	3.42E-01	-0.00007	0.00018	6.79E-01	-0.00004	0.00006	5.24E-01	-0.00006	0.00015	6.85E-01
cg25884324	20	ENSG00000140548	0.00027	0.00039	4.88E-01	0.00008	0.00049	8.63E-01	0.00047	0.00071	5.10E-01	-0.00036	0.00025	1.52E-01	0.00125	0.00053	1.70E-02
cg20102034	37	ENSG00000115325	-0.00147	0.00131	2.64E-01	-0.00476	0.00327	1.45E-01	-0.00560	0.00359	1.19E-01	-0.00026	0.00120	8.28E-01	0.00062	0.00394	8.75E-01
cg25556432	15	ENSG00000132326	-0.00102	0.00227	6.53E-01	-0.00423	0.00534	4.28E-01	-0.00405	0.00744	5.86E-01	-0.00138	0.00249	5.79E-01	0.01153	0.00772	1.35E-01
cg11749902	11	ENSG00000029534	0.00009	0.00066	8.95E-01	-0.00100	0.00187	5.94E-01	0.00038	0.00282	8.93E-01	-0.00008	0.00077	9.16E-01	0.00345	0.00245	1.60E-01
cg09108394	15	ENSG00000168434	-0.00345	0.00778	6.57E-01	-0.00304	0.00745	6.83E-01	-0.00888	0.01185	4.54E-01	0.01197	0.00491	1.48E-02	-0.02541	0.01280	4.72E-02

cg21614201	13	ENSG00000138735	0.00016	0.00054	7.61E-01	-0.00098	0.00086	2.56E-01	0.00023	0.00097	8.08E-01	-0.00016	0.00037	6.72E-01	0.00215	0.00100	3.10E-02
cg12064372	10	ENSG00000139146	0.00001	0.00048	9.90E-01	0.00003	0.00062	9.63E-01	-0.00191	0.00111	8.45E-02	0.00027	0.00042	5.23E-01	0.00196	0.00158	2.15E-01
cg20102034	37	ENSG00000204843	0.00024	0.00021	2.68E-01	0.00032	0.00036	3.81E-01	0.00020	0.00050	6.84E-01	0.00026	0.00032	4.18E-01	-0.00314	0.00225	1.63E-01
cg05946118	12	ENSG00000260979	-0.00056	0.00261	8.29E-01	-0.00463	0.00342	1.76E-01	-0.00012	0.00453	9.80E-01	-0.00258	0.00228	2.58E-01	0.01051	0.00544	5.34E-02
cg06218079	42	ENSG00000169660	-0.00195	0.00166	2.39E-01	0.00041	0.00117	7.27E-01	-0.00030	0.00141	8.34E-01	-0.00490	0.00093	1.42E-07	-0.00417	0.00422	3.23E-01
cg18387671	42	ENSG00000132589	-0.00008	0.00007	2.40E-01	-0.00023	0.00059	6.96E-01	0.00144	0.00091	1.13E-01	-0.00010	0.00006	6.82E-02	-0.00001	0.00015	9.38E-01
cg05304461	26	ENSG00000162490	0.00027	0.00031	3.88E-01	-0.00009	0.00023	6.90E-01	-0.00014	0.00041	7.26E-01	0.00001	0.00021	9.78E-01	0.00148	0.00039	1.48E-04
cg05946118	12	ENSG00000260276	0.00057	0.00286	8.42E-01	-0.00793	0.00540	1.42E-01	0.00865	0.00750	2.49E-01	0.00166	0.00265	5.30E-01	0.00269	0.00785	7.32E-01
cg05946118	12	ENSG00000183044	0.00113	0.00572	8.44E-01	-0.00711	0.00654	2.77E-01	-0.00521	0.01067	6.25E-01	0.01101	0.00327	7.66E-04	-0.00098	0.01058	9.26E-01
cg09108394	15	ENSG00000083093	0.00078	0.00186	6.76E-01	0.00072	0.00393	8.55E-01	-0.00233	0.00465	6.16E-01	0.00021	0.00268	9.38E-01	0.00654	0.00504	1.95E-01
cg06218079	42	ENSG00000169738	0.00070	0.00060	2.43E-01	-0.00030	0.00029	2.97E-01	0.00007	0.00029	8.17E-01	0.00252	0.00093	6.77E-03	0.00834	0.00323	9.85E-03
cg19734370	32	ENSG00000171246	-0.00261	0.00262	3.19E-01	0.00117	0.00133	3.79E-01	-0.00752	0.00258	3.61E-03	-0.00555	0.00193	4.12E-03	0.00459	0.00576	4.26E-01
cg25556432	15	ENSG00000222020	0.00029	0.00070	6.82E-01	-0.00849	0.01133	4.54E-01	0.01025	0.01438	4.76E-01	0.00003	0.00074	9.63E-01	0.00261	0.00220	2.35E-01
cg06218079	42	ENSG00000176845	0.00094	0.00082	2.48E-01	0.00013	0.00124	9.15E-01	0.00101	0.00119	3.95E-01	0.00219	0.00081	7.01E-03	-0.00380	0.00306	2.15E-01
cg07148038	89	ENSG00000204394	-0.00151	0.00096	1.17E-01	-0.00614	0.00438	1.61E-01	0.00271	0.00662	6.83E-01	-0.00372	0.00217	8.63E-02	-0.00076	0.00105	4.69E-01
cg25556432	15	ENSG00000065802	0.00040	0.00105	7.04E-01	0.00155	0.00169	3.57E-01	0.00155	0.00252	5.38E-01	-0.00126	0.00075	9.04E-02	0.00256	0.00224	2.52E-01
cg20102034	37	ENSG00000204792	-0.00086	0.00081	2.86E-01	0.00228	0.00613	7.10E-01	0.00125	0.00894	8.89E-01	-0.00447	0.00363	2.17E-01	-0.00074	0.00084	3.75E-01
cg05304461	26	ENSG00000242349	-0.00003	0.00004	4.08E-01	0.00000	0.00007	9.63E-01	0.00052	0.00116	6.57E-01	-0.00007	0.00006	2.65E-01	-0.00002	0.00011	8.23E-01
cg16734845	25	ENSG00000229474	0.00080	0.00101	4.25E-01	-0.00026	0.00107	8.07E-01	0.00272	0.00140	5.24E-02	0.00044	0.00305	8.85E-01	-0.00620	0.00784	4.29E-01
cg25556432	15	ENSG00000132323	0.00218	0.00587	7.10E-01	0.01174	0.00954	2.19E-01	-0.00869	0.01181	4.62E-01	-0.00426	0.00349	2.22E-01	0.01924	0.01260	1.27E-01
cg06218079	42	ENSG00000169710	0.00125	0.00109	2.54E-01	0.00192	0.00079	1.47E-02	-0.00080	0.00094	3.97E-01	0.00023	0.00058	6.85E-01	0.01248	0.00391	1.42E-03
cg20102034	37	ENSG00000159239	0.00175	0.00165	2.90E-01	0.00175	0.00208	4.00E-01	0.00127	0.00247	6.07E-01	0.00684	0.00451	1.30E-01	-0.01037	0.00838	2.16E-01
cg23396786	18	ENSG00000135617	-0.00081	0.00152	5.96E-01	0.00292	0.00208	1.60E-01	-0.00403	0.00357	2.58E-01	-0.00093	0.00127	4.65E-01	-0.00453	0.00342	1.85E-01
cg25556432	15	ENSG00000168427	0.00143	0.00396	7.17E-01	0.01224	0.01084	2.59E-01	-0.00211	0.01588	8.94E-01	0.00020	0.00458	9.65E-01	-0.00394	0.01693	8.16E-01
cg02885771	11	ENSG00000112419	-0.00019	0.00732	9.80E-01	-0.02069	0.00870	1.74E-02	0.01653	0.01102	1.34E-01	0.00646	0.00368	7.91E-02	-0.00312	0.01025	7.61E-01
cg05946118	12	ENSG00000187555	0.00036	0.00309	9.06E-01	-0.00335	0.00427	4.32E-01	0.00522	0.00607	3.89E-01	0.00428	0.00300	1.54E-01	-0.01074	0.00783	1.71E-01
cg11749902	11	ENSG00000165061	-0.00006	0.00399	9.89E-01	0.01571	0.01175	1.81E-01	-0.00044	0.01193	9.70E-01	-0.00185	0.00468	6.93E-01	-0.01029	0.01863	5.81E-01
cg21614201	13	ENSG00000164096	-0.00036	0.00177	8.41E-01	0.00171	0.00098	8.01E-02	-0.00989	0.00562	7.81E-02	-0.00090	0.00214	6.73E-01	-0.00185	0.00689	7.89E-01
cg06218079	42	ENSG00000197063	0.00054	0.00048	2.61E-01	-0.00028	0.00087	7.49E-01	0.00093	0.00132	4.81E-01	0.00077	0.00066	2.46E-01	0.00349	0.00292	2.32E-01
cg22127773	80	ENSG00000185156	-0.00018	0.00012	1.38E-01	0.00094	0.00134	4.83E-01	-0.00061	0.00039	1.20E-01	-0.00014	0.00013	2.72E-01	-0.00016	0.00065	8.05E-01
cg03077331	45	ENSG00000175711	-0.00447	0.00385	2.46E-01	0.00424	0.01030	6.81E-01	-0.00873	0.00508	8.56E-02	0.00040	0.00122	7.40E-01	-0.01219	0.00515	1.79E-02
cg07148038	89	ENSG00000204439	0.00479	0.00312	1.25E-01	0.00549	0.00446	2.18E-01	0.00274	0.00854	7.49E-01	0.01270	0.00467	6.54E-03	0.00023	0.00226	9.19E-01
cg07148038	89	ENSG00000204308	-0.00585	0.00381	1.25E-01	0.00233	0.00155	1.34E-01	0.00028	0.00181	8.77E-01	-0.00893	0.00353	1.13E-02	-0.02415	0.00559	1.56E-05
cg02206852	52	ENSG00000179761	-0.00558	0.00450	2.15E-01	0.00079	0.00075	2.95E-01	-0.00197	0.00692	7.76E-01	0.00258	0.00253	3.09E-01	-0.03428	0.00710	1.38E-06
cg06982745	18	ENSG00000148730	-0.00078	0.00159	6.24E-01	0.00419	0.00216	5.28E-02	-0.00174	0.00187	3.54E-01	-0.00062	0.00110	5.72E-01	-0.00541	0.00246	2.80E-02
cg16734845	25	ENSG00000179523	0.00074	0.00099	4.50E-01	0.00397	0.00430	3.56E-01	-0.01005	0.00635	1.13E-01	0.00152	0.00102	1.36E-01	0.00023	0.00093	8.07E-01
cg03703840	14	ENSG00000020922	0.00001	0.00005	8.05E-01	-0.00003	0.00014	8.37E-01	-0.00003	0.00018	8.70E-01	0.00005	0.00007	5.10E-01	-0.00004	0.00012	7.70E-01
cg03224276	13	ENSG00000140830	0.00018	0.00118	8.76E-01	-0.00358	0.00351	3.08E-01	-0.00359	0.00392	3.60E-01	0.00078	0.00148	5.98E-01	0.00253	0.00291	3.84E-01
cg06982745	18	ENSG00000197604	0.00083	0.00176	6.35E-01	0.00615	0.00223	5.77E-03	-0.00067	0.00312	8.31E-01	0.00008	0.00021	6.92E-01	-0.00379	0.00343	2.69E-01
cg02137691	31	ENSG00000127419	-0.00605	0.00674	3.69E-01	0.01571	0.00952	9.89E-02	-0.01607	0.01211	1.84E-01	-0.01170	0.00421	5.42E-03	-0.01136	0.01017	2.64E-01
cg25884324	20	ENSG00000182768	-0.00071	0.00126	5.72E-01	0.00100	0.00313	7.50E-01	-0.00195	0.00449	6.64E-01	-0.00113	0.00154	4.63E-01	0.00041	0.00414	9.21E-01
cg02137691	31	ENSG00000214367	-0.00165	0.00184	3.69E-01	-0.00115	0.00175	5.10E-01	0.00058	0.00193	7.65E-01	-0.00937	0.00386	1.53E-02	0.00113	0.00528	8.31E-01

cg02206852	52	ENSG00000109113	-0.00118	0.00096	2.23E-01	-0.00876	0.00476	6.57E-02	-0.00131	0.00258	6.13E-01	-0.00059	0.00120	6.23E-01	-0.00146	0.00232	5.29E-01
cg07148038	89	ENSG00000204261	-0.00240	0.00159	1.31E-01	-0.00339	0.00237	1.52E-01	-0.00406	0.00274	1.38E-01	0.00042	0.00094	6.57E-01	-0.00537	0.00280	5.51E-02
cg18387671	42	ENSG00000265205	-0.00008	0.00008	2.78E-01	0.00079	0.00093	3.93E-01	-0.00054	0.00057	3.49E-01	-0.00009	0.00008	2.70E-01	0.00078	0.00103	4.50E-01
cg03703840	14	ENSG00000180773	0.00005	0.00025	8.49E-01	-0.00058	0.00089	5.16E-01	-0.00066	0.00113	5.60E-01	0.00019	0.00028	5.00E-01	-0.00035	0.00093	7.02E-01
cg07148038	89	ENSG00000228727	0.00302	0.00202	1.34E-01	0.00155	0.00135	2.51E-01	0.00661	0.00577	2.52E-01	0.00741	0.00228	1.15E-03	-0.00135	0.00283	6.33E-01
cg06218079	42	ENSG00000178927	0.00035	0.00032	2.84E-01	-0.00017	0.00009	7.55E-02	0.00004	0.00011	6.73E-01	0.00411	0.00089	3.51E-06	-0.00523	0.00477	2.72E-01
cg05946118	12	ENSG00000182831	0.00001	0.00151	9.95E-01	-0.00480	0.00334	1.51E-01	0.00220	0.00517	6.71E-01	0.00140	0.00190	4.63E-01	-0.00103	0.00549	8.52E-01
cg07957088	43	ENSG00000125520	0.00069	0.00063	2.78E-01	0.00128	0.00139	3.59E-01	0.00311	0.00201	1.21E-01	-0.00003	0.00049	9.47E-01	0.00197	0.00185	2.86E-01
cg06982745	18	ENSG00000107719	0.00025	0.00057	6.65E-01	-0.00040	0.00170	8.14E-01	-0.00098	0.00162	5.45E-01	0.00048	0.00070	4.95E-01	0.00097	0.00182	5.95E-01
cg20102034	37	ENSG00000225439	0.00202	0.00206	3.26E-01	0.00514	0.00532	3.34E-01	0.00206	0.00299	4.91E-01	-0.00198	0.00515	7.01E-01	0.00274	0.00441	5.35E-01
cg25556432	15	ENSG00000068024	0.00053	0.00219	8.08E-01	0.00151	0.00563	7.88E-01	-0.00773	0.00586	1.88E-01	0.00206	0.00290	4.79E-01	0.00152	0.00591	7.97E-01
cg02206852	52	ENSG00000087095	-0.00410	0.00345	2.34E-01	0.00524	0.00533	3.25E-01	-0.00835	0.00533	1.17E-01	-0.00289	0.00313	3.55E-01	-0.01397	0.00701	4.62E-02
cg22127773	80	ENSG00000141504	0.00057	0.00040	1.52E-01	0.00099	0.00036	5.55E-03	-0.00037	0.00061	5.38E-01	0.00020	0.00106	8.52E-01	0.00137	0.00097	1.58E-01
cg20102034	37	ENSG00000135637	-0.00185	0.00191	3.32E-01	-0.00359	0.00420	3.93E-01	-0.00663	0.00531	2.12E-01	0.00014	0.00246	9.55E-01	-0.00514	0.00740	4.87E-01
cg07957088	43	ENSG00000101190	0.00199	0.00188	2.89E-01	0.00618	0.00407	1.29E-01	0.00676	0.00482	1.60E-01	0.00002	0.00238	9.93E-01	-0.00150	0.00478	7.54E-01
cg25884324	20	ENSG00000182511	-0.00113	0.00230	6.24E-01	0.00607	0.00241	1.19E-02	-0.00653	0.00297	2.79E-02	-0.00112	0.00116	3.35E-01	-0.00375	0.00269	1.64E-01
cg23396786	18	ENSG00000135632	0.00432	0.01111	6.97E-01	-0.01816	0.01168	1.20E-01	-0.00082	0.01921	9.66E-01	0.00115	0.00586	8.44E-01	0.04984	0.01917	9.32E-03
cg25884324	20	ENSG00000196547	0.00023	0.00048	6.28E-01	0.00254	0.00326	4.35E-01	0.00235	0.00284	4.08E-01	0.00029	0.00108	7.89E-01	0.00007	0.00055	8.96E-01
cg19734370	32	ENSG00000181045	-0.00076	0.00088	3.92E-01	0.00008	0.00036	8.20E-01	-0.00091	0.00072	2.07E-01	-0.00449	0.00190	1.80E-02	0.00669	0.00477	1.60E-01
cg07957088	43	ENSG00000183260	0.00040	0.00038	2.93E-01	0.00228	0.00197	2.45E-01	-0.00128	0.00288	6.57E-01	0.00078	0.00198	6.95E-01	0.00034	0.00040	3.96E-01
cg02207312	37	ENSG00000110077	0.00023	0.00024	3.46E-01	-0.00076	0.00064	2.34E-01	-0.00012	0.00032	7.08E-01	0.00043	0.00021	4.44E-02	0.00068	0.00030	2.33E-02
cg25556432	15	ENSG00000204104	-0.00057	0.00315	8.57E-01	0.00284	0.01093	7.95E-01	0.00821	0.01300	5.28E-01	-0.00161	0.00360	6.55E-01	-0.00059	0.01039	9.55E-01
cg25556432	15	ENSG00000178752	0.00132	0.00736	8.58E-01	0.01338	0.00958	1.63E-01	0.01016	0.01140	3.73E-01	0.00314	0.00533	5.56E-01	-0.02804	0.01275	2.79E-02
cg09108394	15	ENSG00000090905	0.00076	0.00435	8.60E-01	-0.00164	0.00672	8.08E-01	0.01650	0.00917	7.19E-02	-0.00459	0.00318	1.49E-01	0.00447	0.01086	6.81E-01
cg06218079	42	ENSG00000141556	0.00160	0.00157	3.08E-01	0.00108	0.00116	3.53E-01	-0.00046	0.00138	7.41E-01	0.00479	0.00075	1.88E-10	-0.00139	0.00408	7.33E-01
cg03077331	45	ENSG00000184551	0.00145	0.00136	2.88E-01	0.00104	0.00282	7.12E-01	0.00105	0.00114	3.56E-01	-0.00043	0.00084	6.13E-01	0.00971	0.00353	5.96E-03
cg02207312	37	ENSG00000134825	-0.00025	0.00027	3.50E-01	0.00013	0.00027	6.22E-01	-0.00003	0.00084	9.72E-01	-0.00071	0.00040	7.43E-02	-0.00087	0.00078	2.63E-01
cg05304461	26	ENSG00000132879	-0.00008	0.00012	5.00E-01	-0.00060	0.00033	7.00E-02	-0.00003	0.00048	9.42E-01	0.00001	0.00013	9.56E-01	-0.00015	0.00049	7.61E-01
cg02206852	52	ENSG00000132589	0.00111	0.00097	2.51E-01	0.00492	0.00118	2.85E-05	0.00026	0.00074	7.20E-01	-0.00067	0.00042	1.13E-01	0.00084	0.00083	3.11E-01
cg09108394	15	ENSG00000260751	-0.00010	0.00064	8.76E-01	-0.00435	0.00796	5.85E-01	0.00989	0.01093	3.66E-01	-0.00009	0.00068	8.93E-01	-0.00022	0.00188	9.07E-01
cg19734370	32	ENSG00000263069	-0.00078	0.00095	4.11E-01	0.00146	0.00092	1.14E-01	-0.00254	0.00086	2.98E-03	-0.00102	0.00092	2.68E-01	-0.00104	0.00162	5.22E-01
cg05304461	26	ENSG00000116649	0.00020	0.00030	5.08E-01	-0.00056	0.00044	2.03E-01	0.00018	0.00074	8.11E-01	0.00045	0.00024	6.48E-02	0.00085	0.00071	2.31E-01
cg03703840	14	ENSG00000166002	0.00002	0.00031	9.46E-01	-0.00043	0.00085	6.17E-01	-0.00015	0.00105	8.87E-01	-0.00014	0.00009	1.42E-01	0.00146	0.00081	7.25E-02
cg22127773	80	ENSG00000132507	0.00122	0.00089	1.69E-01	0.00189	0.00146	1.96E-01	-0.00257	0.00302	3.95E-01	0.00040	0.00111	7.16E-01	0.00325	0.00168	5.27E-02
cg09108394	15	ENSG00000103353	-0.00022	0.00178	9.03E-01	-0.00120	0.00377	7.50E-01	0.00623	0.00573	2.77E-01	-0.00149	0.00229	5.14E-01	0.00480	0.00658	4.65E-01
cg02206852	52	ENSG00000109079	-0.00341	0.00304	2.62E-01	-0.00595	0.00251	1.77E-02	0.02052	0.00968	3.40E-02	-0.00156	0.00093	9.35E-02	-0.01027	0.00333	2.03E-03
cg23396786	18	ENSG00000114956	-0.00083	0.00270	7.58E-01	0.00386	0.00618	5.32E-01	-0.00004	0.00722	9.95E-01	-0.00220	0.00375	5.57E-01	-0.00275	0.00685	6.89E-01
cg16734845	25	ENSG00000166763	0.00090	0.00149	5.48E-01	0.00006	0.00229	9.80E-01	-0.00180	0.00243	4.60E-01	0.00396	0.00239	9.70E-02	0.00772	0.01030	4.53E-01
cg02207312	37	ENSG00000149485	-0.00018	0.00020	3.73E-01	-0.00087	0.00048	7.17E-02	0.00207	0.00080	9.55E-03	-0.00009	0.00005	1.16E-01	-0.00038	0.00011	8.02E-04
cg16734845	25	ENSG00000104131	-0.00112	0.00190	5.56E-01	-0.00594	0.00316	6.05E-02	-0.00142	0.00312	6.49E-01	-0.00078	0.00195	6.90E-01	0.00565	0.00420	1.78E-01
cg25884324	20	ENSG00000140564	0.00038	0.00098	6.98E-01	0.00018	0.00276	9.48E-01	0.00068	0.00336	8.40E-01	0.00082	0.00117	4.82E-01	-0.00276	0.00311	3.74E-01
cg25556432	15	ENSG00000144485	0.00026	0.00306	9.33E-01	-0.00304	0.00689	6.59E-01	0.00065	0.00863	9.40E-01	0.00296	0.00396	4.55E-01	-0.01253	0.01086	2.48E-01

cg22127773	80	ENSG00000181222	0.00040	0.00030	1.75E-01	0.00008	0.00023	7.29E-01	0.00056	0.00052	2.82E-01	0.00103	0.00044	1.95E-02	-0.00134	0.00172	4.36E-01
cg07957088	43	ENSG00000101216	-0.00109	0.00111	3.26E-01	-0.00376	0.00201	6.12E-02	-0.00142	0.00306	6.43E-01	0.00082	0.00176	6.41E-01	-0.00051	0.00274	8.52E-01
cg23396786	18	ENSG00000144040	-0.00150	0.00537	7.81E-01	-0.00851	0.01173	4.68E-01	-0.01368	0.01872	4.65E-01	0.00080	0.00685	9.07E-01	0.01015	0.01778	5.68E-01
cg07148038	89	ENSG00000137310	0.00058	0.00041	1.59E-01	-0.00033	0.00101	7.45E-01	0.00042	0.00146	7.71E-01	0.00088	0.00052	8.97E-02	0.00037	0.00116	7.47E-01
cg03077331	45	ENSG00000141551	-0.00107	0.00106	3.15E-01	-0.00048	0.00566	9.32E-01	0.00154	0.00221	4.86E-01	-0.00136	0.00083	1.03E-01	-0.00758	0.00469	1.06E-01
cg06982745	18	ENSG00000166224	-0.00023	0.00087	7.90E-01	-0.00060	0.00175	7.31E-01	-0.00362	0.00342	2.90E-01	-0.00048	0.00128	7.10E-01	0.00172	0.00187	3.56E-01
cg05304461	26	ENSG00000009724	0.00007	0.00012	5.52E-01	-0.00019	0.00014	1.67E-01	-0.00008	0.00026	7.48E-01	0.00013	0.00007	8.01E-02	0.00045	0.00020	2.21E-02
cg09108394	15	ENSG00000166851	0.00024	0.00530	9.63E-01	0.00374	0.00672	5.78E-01	0.00779	0.00888	3.80E-01	0.00373	0.00489	4.46E-01	-0.02402	0.01115	3.11E-02
cg25556432	15	ENSG00000184182	0.00019	0.00437	9.66E-01	-0.00140	0.01069	8.96E-01	-0.00676	0.01537	6.60E-01	-0.00107	0.00536	8.41E-01	0.02003	0.01507	1.84E-01
cg23396786	18	ENSG00000163013	-0.00093	0.00389	8.10E-01	-0.00455	0.01179	6.99E-01	0.01910	0.01574	2.25E-01	-0.00320	0.00457	4.84E-01	0.00665	0.01190	5.76E-01
cg19734370	32	ENSG00000175866	-0.00042	0.00056	4.56E-01	-0.00039	0.00074	5.93E-01	-0.00013	0.00157	9.34E-01	-0.00046	0.00104	6.58E-01	-0.00254	0.00434	5.58E-01
cg19734370	32	ENSG00000224877	-0.00070	0.00095	4.58E-01	-0.00176	0.00159	2.68E-01	0.00065	0.00290	8.23E-01	-0.00003	0.00134	9.82E-01	-0.00349	0.00487	4.74E-01
cg18387671	42	ENSG00000264608	0.00040	0.00042	3.50E-01	0.00095	0.00030	1.34E-03	0.00168	0.00112	1.36E-01	-0.00012	0.00062	8.40E-01	-0.00026	0.00035	4.54E-01
cg02137691	31	ENSG00000145214	0.00446	0.00624	4.75E-01	-0.00497	0.00836	5.52E-01	0.01454	0.00883	9.96E-02	0.01414	0.00494	4.20E-03	-0.00855	0.00804	2.87E-01
cg06218079	42	ENSG00000141552	0.00029	0.00031	3.57E-01	0.00019	0.00059	7.43E-01	0.00009	0.00067	8.98E-01	0.00044	0.00045	3.21E-01	-0.00003	0.00214	9.90E-01
cg19734370	32	ENSG00000262877	-0.00014	0.00020	4.74E-01	-0.00142	0.00067	3.40E-02	0.00001	0.00015	9.28E-01	-0.00032	0.00030	2.82E-01	0.00014	0.00028	6.09E-01
cg25884324	20	ENSG00000140577	-0.00035	0.00120	7.67E-01	0.00164	0.00303	5.89E-01	-0.00148	0.00418	7.22E-01	0.00001	0.00147	9.95E-01	-0.00466	0.00368	2.05E-01
cg16734845	25	ENSG00000138606	-0.00025	0.00052	6.23E-01	-0.00055	0.00108	6.09E-01	0.00057	0.00152	7.10E-01	-0.00036	0.00067	5.89E-01	0.00039	0.00211	8.54E-01
cg16734845	25	ENSG00000104133	0.00096	0.00197	6.25E-01	-0.00475	0.00546	3.84E-01	0.00671	0.00612	2.74E-01	0.00088	0.00233	7.05E-01	0.00534	0.00900	5.53E-01
cg25884324	20	ENSG00000182054	-0.00030	0.00110	7.82E-01	0.00265	0.00200	1.86E-01	-0.00247	0.00239	3.02E-01	-0.00137	0.00133	3.06E-01	0.00065	0.00267	8.08E-01
cg03077331	45	ENSG00000169683	-0.00229	0.00245	3.49E-01	0.00943	0.01144	4.10E-01	-0.00330	0.00388	3.95E-01	-0.00056	0.00121	6.40E-01	-0.01236	0.00626	4.85E-02
cg06982745	18	ENSG00000197467	-0.00007	0.00046	8.73E-01	0.00139	0.00354	6.94E-01	-0.00269	0.00283	3.41E-01	0.00012	0.00100	9.03E-01	-0.00007	0.00053	8.97E-01
cg22127773	80	ENSG00000265749	0.00033	0.00026	1.97E-01	0.00023	0.00036	5.24E-01	0.00065	0.00083	4.34E-01	0.00039	0.00043	3.66E-01	0.00039	0.00146	7.91E-01
cg07148038	89	ENSG00000204267	0.00062	0.00046	1.78E-01	-0.00024	0.00666	9.72E-01	-0.00996	0.00829	2.29E-01	0.00061	0.00050	2.29E-01	0.00092	0.00115	4.24E-01
cg06218079	42	ENSG00000169727	-0.00017	0.00020	3.81E-01	-0.00027	0.00030	3.78E-01	-0.00008	0.00037	8.38E-01	-0.00023	0.00040	5.62E-01	0.00083	0.00124	5.06E-01
cg07957088	43	ENSG00000130584	0.00242	0.00272	3.74E-01	0.01146	0.00829	1.67E-01	-0.00476	0.00976	6.26E-01	0.00363	0.00365	3.20E-01	-0.00178	0.00533	7.39E-01
cg16734845	25	ENSG00000171763	0.00064	0.00138	6.43E-01	0.00538	0.00301	7.35E-02	0.00082	0.00358	8.18E-01	-0.00063	0.00116	5.90E-01	-0.00082	0.00815	9.19E-01
cg05304461	26	ENSG00000116663	-0.00004	0.00008	6.20E-01	-0.00010	0.00016	5.19E-01	0.00011	0.00029	7.15E-01	-0.00004	0.00010	6.86E-01	0.00004	0.00027	8.83E-01
cg16734845	25	ENSG00000140264	0.00108	0.00236	6.48E-01	0.00452	0.00616	4.63E-01	-0.00258	0.00825	7.54E-01	0.00219	0.00306	4.75E-01	-0.00375	0.00558	5.02E-01
cg19734370	32	ENSG00000171298	0.00048	0.00073	5.11E-01	-0.00068	0.00056	2.27E-01	0.00258	0.00107	1.64E-02	-0.00025	0.00051	6.26E-01	0.00290	0.00236	2.18E-01
cg20102034	37	ENSG00000115307	0.00275	0.00358	4.43E-01	0.00081	0.00443	8.55E-01	0.00973	0.00482	4.36E-02	0.00671	0.00265	1.12E-02	-0.00931	0.00561	9.72E-02
cg19734370	32	ENSG00000263053	-0.00010	0.00016	5.13E-01	-0.00019	0.00038	6.05E-01	0.00126	0.00142	3.73E-01	-0.00012	0.00013	3.51E-01	0.00368	0.00257	1.52E-01
cg19734370	32	ENSG00000141564	-0.00051	0.00078	5.16E-01	0.00038	0.00076	6.19E-01	0.00072	0.00168	6.68E-01	-0.00148	0.00091	1.04E-01	-0.00491	0.00330	1.38E-01
cg02137691	31	ENSG00000185818	-0.00061	0.00098	5.34E-01	-0.00081	0.00076	2.83E-01	0.00003	0.00093	9.70E-01	-0.00819	0.00475	8.46E-02	0.01373	0.01052	1.92E-01
cg16734845	25	ENSG00000168781	-0.00075	0.00172	6.62E-01	-0.00755	0.00413	6.72E-02	-0.00285	0.00529	5.90E-01	0.00130	0.00147	3.78E-01	-0.00019	0.00258	9.41E-01
cg03077331	45	ENSG00000173762	0.00062	0.00069	3.69E-01	0.00316	0.00627	6.14E-01	-0.00172	0.00204	3.98E-01	0.00039	0.00027	1.54E-01	0.00274	0.00146	6.11E-02
cg18387671	42	ENSG00000108262	-0.00038	0.00045	3.96E-01	0.00016	0.00055	7.69E-01	-0.00162	0.00050	1.25E-03	-0.00021	0.00034	5.40E-01	0.00063	0.00098	5.23E-01
cg25884324	20	ENSG00000259291	0.00008	0.00036	8.32E-01	0.00305	0.00218	1.60E-01	0.00006	0.00088	9.42E-01	0.00019	0.00021	3.55E-01	-0.00110	0.00091	2.26E-01
cg20102034	37	ENSG00000115350	0.00201	0.00267	4.51E-01	0.00568	0.00864	5.11E-01	-0.00419	0.01026	6.83E-01	0.00311	0.00315	3.24E-01	-0.00401	0.00773	6.04E-01
cg19734370	32	ENSG00000171282	-0.00148	0.00231	5.23E-01	0.00279	0.00150	6.31E-02	-0.00340	0.00354	3.36E-01	-0.00173	0.00200	3.85E-01	-0.01281	0.00669	5.54E-02
cg23396786	18	ENSG00000124356	-0.00041	0.00467	9.31E-01	-0.01096	0.01136	3.35E-01	0.00079	0.01821	9.65E-01	0.00058	0.00562	9.18E-01	0.01340	0.01717	4.35E-01
cg22127773	80	ENSG00000072778	-0.00074	0.00059	2.10E-01	-0.00102	0.00132	4.41E-01	-0.00096	0.00230	6.77E-01	-0.00083	0.00072	2.45E-01	0.00150	0.00242	5.34E-01

cg07148038	89	ENSG00000242574	-0.00664	0.00508	1.91E-01	-0.00929	0.00439	3.43E-02	0.00348	0.01034	7.36E-01	0.00080	0.00277	7.72E-01	-0.02051	0.00651	1.63E-03
cg18387671	42	ENSG00000109111	0.00014	0.00017	4.05E-01	0.00017	0.00029	5.69E-01	0.00089	0.00109	4.12E-01	0.00013	0.00023	5.65E-01	-0.00007	0.00052	8.92E-01
cg07957088	43	ENSG00000101193	0.00288	0.00340	3.96E-01	0.00968	0.00773	2.11E-01	0.01026	0.01173	3.82E-01	-0.00195	0.00426	6.48E-01	0.01015	0.00924	2.72E-01
cg22127773	80	ENSG00000132514	-0.00059	0.00047	2.13E-01	-0.00058	0.00151	7.00E-01	0.00095	0.00252	7.06E-01	-0.00043	0.00074	5.65E-01	-0.00086	0.00070	2.22E-01
cg18387671	42	ENSG00000227543	-0.00020	0.00024	4.08E-01	0.00001	0.00006	8.68E-01	-0.00154	0.00075	3.98E-02	-0.00036	0.00028	2.02E-01	0.00051	0.00079	5.19E-01
cg02137691	31	ENSG00000174137	-0.00032	0.00054	5.53E-01	-0.00069	0.00083	4.05E-01	0.00026	0.00090	7.74E-01	-0.00139	0.00143	3.33E-01	0.00093	0.00190	6.22E-01
cg07957088	43	ENSG00000101213	-0.00145	0.00173	4.02E-01	-0.00260	0.00384	4.99E-01	-0.00373	0.00521	4.74E-01	-0.00143	0.00222	5.20E-01	0.00454	0.00619	4.63E-01
cg06982745	18	ENSG00000042286	-0.00001	0.00017	9.61E-01	-0.00018	0.00053	7.30E-01	0.00025	0.00055	6.50E-01	-0.00006	0.00021	7.77E-01	0.00025	0.00053	6.37E-01
cg02207312	37	ENSG00000149534	-0.00016	0.00022	4.68E-01	-0.00029	0.00017	1.00E-01	0.00035	0.00022	1.08E-01	0.00007	0.00016	6.74E-01	-0.00080	0.00023	4.07E-04
cg02137691	31	ENSG00000109685	-0.00105	0.00181	5.62E-01	-0.00103	0.00475	8.28E-01	-0.00160	0.00567	7.78E-01	-0.00080	0.00233	7.33E-01	-0.00171	0.00466	7.13E-01
cg02207312	37	ENSG00000167992	0.00002	0.00002	4.72E-01	0.00000	0.00003	8.91E-01	-0.00044	0.00061	4.76E-01	0.00002	0.00005	6.78E-01	0.00009	0.00006	1.05E-01
cg18387671	42	ENSG00000108582	0.00027	0.00034	4.17E-01	0.00042	0.00044	3.45E-01	0.00108	0.00060	6.95E-02	0.00034	0.00026	1.91E-01	-0.00119	0.00071	9.54E-02
cg25884324	20	ENSG00000197299	0.00014	0.00091	8.78E-01	0.00170	0.00213	4.25E-01	-0.00162	0.00192	3.99E-01	0.00079	0.00094	4.02E-01	-0.00452	0.00361	2.10E-01
cg06982745	18	ENSG00000180644	-0.00005	0.00152	9.76E-01	-0.00169	0.00127	1.84E-01	0.00659	0.00328	4.45E-02	0.00151	0.00149	3.13E-01	-0.00289	0.00126	2.15E-02
cg20102034	37	ENSG00000204822	0.00044	0.00061	4.75E-01	0.00049	0.00157	7.54E-01	0.00157	0.00192	4.13E-01	0.00038	0.00071	5.90E-01	-0.00862	0.00633	1.73E-01
cg22127773	80	ENSG00000174326	0.00116	0.00094	2.20E-01	-0.00094	0.00197	6.32E-01	0.00345	0.00111	1.88E-03	0.00092	0.00069	1.87E-01	-0.00067	0.00213	7.53E-01
cg25884324	20	ENSG00000184056	0.00035	0.00237	8.81E-01	-0.00673	0.00273	1.38E-02	0.00357	0.00321	2.67E-01	0.00251	0.00180	1.64E-01	0.00216	0.00325	5.06E-01
cg02206852	52	ENSG00000087111	0.00176	0.00187	3.47E-01	-0.00011	0.00698	9.88E-01	-0.00445	0.00688	5.18E-01	0.00227	0.00206	2.69E-01	0.00751	0.01090	4.91E-01
cg02137691	31	ENSG00000196810	-0.00024	0.00045	5.86E-01	0.00078	0.00671	9.07E-01	-0.00773	0.00895	3.88E-01	-0.00011	0.00058	8.45E-01	-0.00040	0.00070	5.71E-01
cg22127773	80	ENSG00000178977	0.00053	0.00044	2.27E-01	0.00057	0.00058	3.29E-01	-0.00012	0.00195	9.51E-01	0.00065	0.00075	3.89E-01	-0.00061	0.00299	8.39E-01
cg07957088	43	ENSG00000101189	0.00464	0.00583	4.26E-01	0.01125	0.00794	1.57E-01	0.02125	0.01168	6.90E-02	-0.00436	0.00367	2.35E-01	0.00044	0.01033	9.66E-01
cg03077331	45	ENSG00000169727	-0.00238	0.00289	4.10E-01	0.01320	0.00811	1.03E-01	-0.01176	0.00356	9.57E-04	0.00027	0.00059	6.50E-01	-0.00310	0.00219	1.58E-01
cg07957088	43	ENSG00000130589	0.00063	0.00080	4.30E-01	0.00107	0.00063	9.10E-02	-0.00059	0.00090	5.14E-01	0.00593	0.00400	1.38E-01	0.00428	0.00678	5.27E-01
cg03077331	45	ENSG00000183684	0.00052	0.00063	4.11E-01	0.01303	0.00498	8.85E-03	0.00434	0.00185	1.93E-02	-0.00009	0.00012	4.41E-01	-0.00011	0.00040	7.82E-01
cg18387671	42	ENSG00000141298	0.00027	0.00035	4.42E-01	0.00016	0.00028	5.73E-01	0.00182	0.00068	7.30E-03	-0.00033	0.00021	1.19E-01	0.00026	0.00066	6.97E-01
cg06218079	42	ENSG00000262663	-0.00010	0.00013	4.42E-01	-0.00067	0.00083	4.18E-01	-0.00060	0.00103	5.59E-01	-0.00003	0.00013	8.10E-01	-0.00088	0.00057	1.23E-01
cg02137691	31	ENSG00000178950	0.00034	0.00066	6.01E-01	-0.00043	0.00528	9.35E-01	0.00829	0.00535	1.21E-01	0.00011	0.00075	8.84E-01	0.00072	0.00149	6.28E-01
cg02207312	37	ENSG00000162144	-0.00007	0.00010	5.05E-01	-0.00011	0.00013	4.03E-01	0.00012	0.00017	4.87E-01	-0.00056	0.00038	1.33E-01	-0.00025	0.00059	6.75E-01
cg02206852	52	ENSG00000221995	-0.00165	0.00181	3.61E-01	0.00415	0.00740	5.75E-01	-0.00035	0.00373	9.24E-01	-0.00272	0.00218	2.12E-01	0.00369	0.01455	8.00E-01
cg25884324	20	ENSG00000140575	0.00007	0.00115	9.48E-01	0.00236	0.00155	1.28E-01	-0.00308	0.00208	1.39E-01	0.00125	0.00082	1.28E-01	-0.00205	0.00191	2.84E-01
cg02207312	37	ENSG00000013725	0.00005	0.00008	5.19E-01	0.00086	0.00037	2.04E-02	0.00000	0.00014	9.99E-01	0.00004	0.00006	5.14E-01	-0.00001	0.00012	9.35E-01
cg16734845	25	ENSG00000185880	0.00011	0.00038	7.72E-01	0.00130	0.00145	3.69E-01	0.00112	0.00164	4.95E-01	-0.00002	0.00041	9.55E-01	-0.00040	0.00174	8.19E-01
cg07148038	89	ENSG00000232810	-0.00178	0.00144	2.18E-01	-0.00201	0.00186	2.82E-01	0.00289	0.00746	6.99E-01	-0.00142	0.00252	5.74E-01	-0.00655	0.00791	4.08E-01
cg25884324	20	ENSG00000259704	0.00001	0.00020	9.71E-01	0.00066	0.00075	3.82E-01	0.00017	0.00031	5.99E-01	-0.00019	0.00027	4.78E-01	-0.00014	0.00285	9.59E-01
cg07148038	89	ENSG00000223534	0.00329	0.00268	2.20E-01	-0.00067	0.00583	9.08E-01	-0.00134	0.00875	8.78E-01	0.00165	0.00266	5.35E-01	0.01132	0.00479	1.81E-02
cg03077331	45	ENSG00000141522	-0.00018	0.00023	4.36E-01	-0.00079	0.00181	6.63E-01	-0.00059	0.00076	4.37E-01	-0.00010	0.00025	6.91E-01	-0.00090	0.00135	5.07E-01
cg16734845	25	ENSG00000166963	-0.00065	0.00241	7.89E-01	0.00938	0.00611	1.25E-01	-0.00502	0.00681	4.61E-01	-0.00078	0.00050	1.18E-01	-0.00778	0.00701	2.67E-01
cg07957088	43	ENSG00000229873	-0.00288	0.00391	4.61E-01	0.00460	0.00426	2.80E-01	-0.01229	0.00627	4.99E-02	-0.00135	0.00457	7.67E-01	-0.00929	0.00957	3.32E-01
cg07148038	89	ENSG00000204482	-0.00229	0.00190	2.28E-01	-0.00655	0.00301	2.93E-02	0.00440	0.00598	4.62E-01	-0.00158	0.00233	4.99E-01	0.00015	0.00463	9.74E-01
cg02137691	31	ENSG00000130997	0.00164	0.00384	6.70E-01	-0.00405	0.00486	4.04E-01	0.01136	0.00478	1.75E-02	0.00504	0.00281	7.28E-02	-0.00706	0.00490	1.50E-01
cg07148038	89	ENSG00000204463	-0.00282	0.00237	2.33E-01	-0.00894	0.00412	2.99E-02	0.00022	0.00389	9.55E-01	-0.00048	0.00193	8.02E-01	-0.01074	0.00926	2.46E-01
cg06218079	42	ENSG00000141560	0.00019	0.00028	4.95E-01	0.00068	0.00021	1.14E-03	-0.00051	0.00026	5.44E-02	0.00024	0.00018	1.81E-01	0.00052	0.00082	5.30E-01

cg16734845	25	ENSG00000137857	-0.00100	0.00474	8.33E-01	-0.01184	0.00597	4.74E-02	0.00780	0.00802	3.30E-01	-0.00389	0.00290	1.80E-01	0.01496	0.01046	1.53E-01
cg07957088	43	ENSG00000130590	-0.00034	0.00049	4.86E-01	0.00389	0.00556	4.84E-01	-0.01050	0.00857	2.21E-01	-0.00033	0.00050	5.10E-01	-0.00761	0.01045	4.66E-01
cg07957088	43	ENSG00000026036	-0.00039	0.00057	4.88E-01	-0.00124	0.00124	3.16E-01	0.00223	0.00193	2.48E-01	-0.00013	0.00059	8.31E-01	-0.00171	0.00134	2.03E-01
cg02206852	52	ENSG00000167525	-0.00030	0.00036	4.08E-01	-0.00006	0.00126	9.60E-01	-0.00147	0.00734	8.41E-01	-0.00051	0.00043	2.35E-01	0.00030	0.00076	6.90E-01
cg22127773	80	ENSG00000108839	0.00144	0.00129	2.65E-01	0.00085	0.00147	5.65E-01	0.00769	0.00280	6.07E-03	0.00181	0.00090	4.47E-02	-0.00120	0.00094	2.04E-01
cg18387671	42	ENSG00000108576	0.00004	0.00006	5.05E-01	0.00010	0.00010	3.09E-01	0.00093	0.00104	3.70E-01	0.00002	0.00008	8.14E-01	-0.00010	0.00019	6.19E-01
cg07957088	43	ENSG00000171703	-0.00077	0.00113	4.98E-01	0.00249	0.00345	4.71E-01	-0.00941	0.00471	4.57E-02	-0.00030	0.00029	2.94E-01	-0.00130	0.00182	4.76E-01
cg02137691	31	ENSG00000168924	-0.00050	0.00128	6.95E-01	0.00636	0.00665	3.38E-01	0.00399	0.00939	6.71E-01	-0.00122	0.00164	4.57E-01	-0.00021	0.00220	9.26E-01
cg07957088	43	ENSG00000125534	0.00186	0.00278	5.03E-01	0.01407	0.00720	5.05E-02	-0.01856	0.01281	1.48E-01	-0.00047	0.00286	8.69E-01	0.00242	0.00148	1.02E-01
cg20102034	37	ENSG00000239779	-0.00050	0.00092	5.86E-01	-0.00043	0.00094	6.46E-01	0.00127	0.00837	8.80E-01	-0.00307	0.00704	6.63E-01	-0.00462	0.00888	6.03E-01
cg06218079	42	ENSG00000141551	0.00044	0.00067	5.17E-01	0.00052	0.00107	6.28E-01	0.00206	0.00106	5.26E-02	-0.00027	0.00063	6.68E-01	-0.00315	0.00350	3.69E-01
cg22127773	80	ENSG00000108961	-0.00017	0.00016	2.72E-01	0.00004	0.00027	8.87E-01	0.00004	0.00056	9.39E-01	-0.00032	0.00020	1.20E-01	-0.00153	0.00335	6.48E-01
cg07957088	43	ENSG00000125531	0.00033	0.00049	5.05E-01	0.00605	0.00433	1.62E-01	0.00380	0.00525	4.69E-01	0.00024	0.00063	7.00E-01	0.00018	0.00080	8.18E-01
cg16734845	25	ENSG00000259479	0.00011	0.00069	8.75E-01	0.00542	0.00635	3.94E-01	0.01184	0.00672	7.82E-02	0.00022	0.00041	6.01E-01	-0.00057	0.00082	4.92E-01
cg05304461	26	ENSG00000142657	-0.00004	0.00022	8.42E-01	-0.00043	0.00092	6.43E-01	-0.00354	0.00142	1.26E-02	0.00009	0.00007	1.88E-01	0.00002	0.00020	9.07E-01
cg05304461	26	ENSG00000142655	-0.00015	0.00075	8.42E-01	-0.00246	0.00085	3.95E-03	0.00060	0.00133	6.50E-01	0.00018	0.00035	5.98E-01	0.00128	0.00094	1.72E-01
cg02207312	37	ENSG00000162148	-0.00003	0.00005	5.95E-01	0.00009	0.00040	8.30E-01	-0.00008	0.00006	1.74E-01	0.00012	0.00011	2.95E-01	0.00005	0.00016	7.77E-01
cg02137691	31	ENSG00000218422	0.00014	0.00038	7.18E-01	-0.00026	0.00040	5.16E-01	0.00097	0.00050	5.10E-02	-0.00021	0.00077	7.79E-01	-0.00098	0.00184	5.93E-01
cg07957088	43	ENSG00000125510	0.00032	0.00049	5.20E-01	0.00067	0.00069	3.34E-01	-0.00038	0.00087	6.63E-01	0.00028	0.00121	8.15E-01	0.01012	0.00678	1.35E-01
cg22127773	80	ENSG00000129255	-0.00093	0.00086	2.80E-01	-0.00185	0.00132	1.60E-01	-0.00331	0.00219	1.30E-01	0.00022	0.00109	8.39E-01	0.00275	0.00480	5.66E-01
cg02137691	31	ENSG00000168936	0.00080	0.00224	7.22E-01	0.00013	0.00228	9.55E-01	-0.00224	0.00245	3.59E-01	0.00981	0.00476	3.90E-02	-0.00062	0.00694	9.29E-01
cg02137691	31	ENSG00000013810	-0.00019	0.00055	7.33E-01	-0.00134	0.00148	3.65E-01	0.00105	0.00197	5.95E-01	-0.00016	0.00070	8.17E-01	0.00010	0.00136	9.42E-01
cg02206852	52	ENSG00000263370	-0.00245	0.00316	4.38E-01	0.00316	0.00491	5.20E-01	-0.00019	0.00250	9.39E-01	-0.00838	0.00231	2.77E-04	0.00054	0.01272	9.66E-01
cg19734370	32	ENSG00000141519	-0.00006	0.00017	7.14E-01	0.00006	0.00024	7.94E-01	0.00005	0.00057	9.33E-01	-0.00013	0.00028	6.50E-01	-0.00160	0.00096	9.29E-02
cg06218079	42	ENSG00000265458	0.00043	0.00071	5.46E-01	-0.00007	0.00042	8.60E-01	-0.00056	0.00052	2.89E-01	0.00296	0.00104	4.51E-03	-0.00158	0.00627	8.00E-01
cg16734845	25	ENSG00000140263	-0.00018	0.00193	9.26E-01	-0.00076	0.00481	8.74E-01	-0.00464	0.00561	4.08E-01	0.00029	0.00232	8.99E-01	0.01087	0.01181	3.57E-01
cg02206852	52	ENSG00000076382	-0.00148	0.00195	4.46E-01	0.00100	0.00122	4.12E-01	-0.01865	0.00949	4.94E-02	0.00008	0.00273	9.76E-01	-0.00358	0.00183	5.03E-02
cg19734370	32	ENSG00000185168	0.00009	0.00027	7.28E-01	-0.00056	0.00083	5.01E-01	-0.00051	0.00157	7.43E-01	0.00025	0.00030	4.03E-01	-0.00054	0.00105	6.09E-01
cg06218079	42	ENSG00000141562	0.00042	0.00072	5.56E-01	-0.00069	0.00076	3.63E-01	-0.00008	0.00078	9.21E-01	0.00192	0.00071	7.29E-03	0.00070	0.00296	8.13E-01
cg02137691	31	ENSG00000244459	-0.00063	0.00200	7.54E-01	-0.00236	0.00235	3.17E-01	0.00132	0.00299	6.59E-01	0.00329	0.00233	1.58E-01	-0.00636	0.00356	7.43E-02
cg02137691	31	ENSG00000090316	-0.00121	0.00394	7.58E-01	0.01159	0.00837	1.66E-01	-0.00483	0.00926	6.02E-01	-0.00601	0.00328	6.70E-02	0.00237	0.00768	7.58E-01
cg03077331	45	ENSG00000141542	-0.00075	0.00118	5.23E-01	-0.00369	0.01003	7.13E-01	-0.00636	0.00351	7.02E-02	-0.00001	0.00118	9.92E-01	0.00047	0.00774	9.52E-01
cg02137691	31	ENSG00000179979	0.00082	0.00266	7.59E-01	0.00356	0.00869	6.82E-01	-0.00176	0.01310	8.93E-01	0.00080	0.00338	8.13E-01	0.00025	0.00535	9.63E-01
cg02207312	37	ENSG00000110108	0.00004	0.00010	6.41E-01	0.00064	0.00046	1.64E-01	-0.00018	0.00015	2.43E-01	0.00002	0.00007	7.45E-01	0.00021	0.00015	1.73E-01
cg03077331	45	ENSG00000141568	-0.00215	0.00340	5.27E-01	-0.01107	0.01138	3.31E-01	0.00201	0.00473	6.71E-01	0.00020	0.00154	8.96E-01	-0.01983	0.00990	4.53E-02
cg02207312	37	ENSG00000166928	-0.00005	0.00010	6.44E-01	0.00050	0.00075	5.01E-01	-0.00001	0.00038	9.75E-01	0.00004	0.00006	4.44E-01	-0.00024	0.00013	5.87E-02
cg22127773	80	ENSG00000179029	-0.00059	0.00057	2.98E-01	-0.00067	0.00127	6.00E-01	0.00111	0.00211	5.98E-01	-0.00070	0.00068	3.01E-01	-0.00164	0.00333	6.22E-01
cg06218079	42	ENSG00000263731	0.00029	0.00052	5.69E-01	0.00067	0.00175	7.03E-01	0.00007	0.00206	9.73E-01	0.00020	0.00057	7.34E-01	0.00199	0.00269	4.60E-01
cg22127773	80	ENSG00000141505	0.00018	0.00017	2.99E-01	0.00012	0.00013	3.61E-01	-0.00007	0.00022	7.67E-01	0.00084	0.00031	7.43E-03	-0.00008	0.00041	8.52E-01
cg02207312	37	ENSG00000168496	0.00010	0.00022	6.47E-01	0.00025	0.00031	4.24E-01	-0.00015	0.00014	2.58E-01	0.00067	0.00031	2.79E-02	-0.00038	0.00044	3.97E-01
cg03077331	45	ENSG00000185624	0.00082	0.00133	5.34E-01	-0.00774	0.00693	2.64E-01	-0.00172	0.00281	5.42E-01	0.00072	0.00056	2.01E-01	0.00431	0.00226	5.71E-02
cg07148038	89	ENSG00000213719	-0.00129	0.00118	2.71E-01	0.00063	0.00498	9.00E-01	-0.00389	0.00323	2.28E-01	-0.00352	0.00189	6.21E-02	0.00012	0.00093	8.97E-01

cg02206852	52	ENSG00000198720	0.00037	0.00050	4.65E-01	-0.00149	0.00249	5.48E-01	-0.00037	0.01422	9.79E-01	0.00044	0.00051	3.89E-01	0.00088	0.00630	8.89E-01
cg06218079	42	ENSG00000184551	-0.00008	0.00014	5.78E-01	-0.00102	0.00051	4.42E-02	-0.00052	0.00057	3.58E-01	0.00002	0.00007	7.29E-01	0.00003	0.00018	8.76E-01
cg02207312	37	ENSG00000149483	0.00012	0.00026	6.57E-01	-0.00070	0.00075	3.50E-01	0.00097	0.00071	1.74E-01	-0.00006	0.00027	8.36E-01	0.00052	0.00053	3.30E-01
cg05304461	26	ENSG00000116670	-0.00002	0.00028	9.37E-01	0.00026	0.00056	6.39E-01	-0.00040	0.00086	6.37E-01	-0.00020	0.00038	5.99E-01	0.00075	0.00096	4.35E-01
cg03077331	45	ENSG00000169733	-0.00167	0.00275	5.42E-01	-0.00033	0.00270	9.04E-01	0.00326	0.00093	4.55E-04	-0.00443	0.00173	1.06E-02	-0.01228	0.00691	7.55E-02
cg03077331	45	ENSG00000187531	0.00098	0.00163	5.46E-01	0.00142	0.00918	8.77E-01	0.00055	0.00325	8.65E-01	0.00122	0.00199	5.40E-01	-0.00033	0.00748	9.64E-01
cg03077331	45	ENSG00000265458	-0.00236	0.00398	5.53E-01	0.02596	0.01886	1.69E-01	-0.01272	0.00681	6.19E-02	0.00143	0.00114	2.09E-01	-0.00507	0.00428	2.36E-01
cg19734370	32	ENSG00000173818	0.00034	0.00119	7.78E-01	0.00040	0.00152	7.94E-01	0.00345	0.00297	2.46E-01	-0.00147	0.00133	2.68E-01	0.00538	0.00448	2.30E-01
cg05304461	26	ENSG00000116661	-0.00002	0.00036	9.58E-01	-0.00045	0.00086	6.00E-01	0.00072	0.00151	6.33E-01	-0.00004	0.00042	9.19E-01	0.00070	0.00135	6.02E-01
cg18387671	42	ENSG00000198242	0.00005	0.00009	5.94E-01	0.00025	0.00035	4.67E-01	-0.00035	0.00026	1.89E-01	0.00006	0.00003	3.89E-02	0.00041	0.00038	2.78E-01
cg06218079	42	ENSG00000141580	-0.00038	0.00072	5.94E-01	0.00068	0.00060	2.54E-01	-0.00006	0.00059	9.16E-01	-0.00194	0.00054	3.08E-04	0.00040	0.00218	8.55E-01
cg02207312	37	ENSG00000110104	0.00006	0.00015	6.75E-01	-0.00018	0.00070	7.97E-01	-0.00045	0.00039	2.52E-01	0.00017	0.00018	3.50E-01	0.00016	0.00040	6.90E-01
cg02207312	37	ENSG00000149516	-0.00006	0.00014	6.77E-01	-0.00015	0.00018	4.04E-01	0.00017	0.00056	7.56E-01	0.00001	0.00024	9.80E-01	0.00046	0.00074	5.34E-01
cg22127773	80	ENSG00000125434	0.00080	0.00080	3.13E-01	0.00046	0.00096	6.31E-01	0.00347	0.00161	3.09E-02	-0.00028	0.00082	7.29E-01	0.00242	0.00343	4.82E-01
cg06218079	42	ENSG00000260563	-0.00030	0.00057	5.98E-01	0.00031	0.00096	7.48E-01	0.00005	0.00098	9.59E-01	-0.00167	0.00105	1.12E-01	0.00225	0.00336	5.03E-01
cg22127773	80	ENSG00000178999	-0.00048	0.00047	3.15E-01	-0.00059	0.00128	6.49E-01	-0.00191	0.00130	1.43E-01	-0.00001	0.00058	9.86E-01	-0.00259	0.00206	2.08E-01
cg07957088	43	ENSG00000101187	-0.00126	0.00231	5.86E-01	-0.00176	0.00506	7.28E-01	-0.00423	0.00647	5.13E-01	-0.00064	0.00314	8.40E-01	-0.00007	0.00653	9.92E-01
cg02206852	52	ENSG00000264304	0.00155	0.00221	4.85E-01	-0.00192	0.00268	4.75E-01	0.00868	0.00634	1.71E-01	0.00372	0.00332	2.63E-01	0.00499	0.01172	6.70E-01
cg03077331	45	ENSG00000167363	0.00095	0.00163	5.61E-01	-0.01593	0.01121	1.55E-01	0.00190	0.00462	6.81E-01	0.00134	0.00180	4.58E-01	-0.00159	0.00929	8.64E-01
cg02137691	31	ENSG00000243449	-0.00012	0.00050	8.15E-01	-0.00286	0.00413	4.89E-01	-0.00010	0.00482	9.84E-01	-0.00066	0.00034	5.04E-02	0.00056	0.00040	1.63E-01
cg02137691	31	ENSG00000127418	0.00131	0.00570	8.19E-01	0.02100	0.00722	3.62E-03	-0.00554	0.00846	5.13E-01	-0.00228	0.00327	4.86E-01	-0.00684	0.00668	3.06E-01
cg07148038	89	ENSG00000213722	0.00226	0.00212	2.86E-01	0.00491	0.00289	8.89E-02	-0.00342	0.01011	7.35E-01	0.00059	0.00434	8.93E-01	-0.00218	0.00507	6.67E-01
cg20102034	37	ENSG00000115274	-0.00027	0.00069	6.90E-01	0.00052	0.00153	7.31E-01	-0.00305	0.00221	1.69E-01	-0.00017	0.00088	8.46E-01	0.00017	0.00218	9.39E-01
cg02207312	37	ENSG00000167986	-0.00013	0.00034	6.95E-01	-0.00028	0.00051	5.78E-01	-0.00039	0.00070	5.73E-01	0.00053	0.00034	1.17E-01	-0.00085	0.00056	1.29E-01
cg07148038	89	ENSG00000179344	-0.00461	0.00435	2.89E-01	-0.00850	0.00626	1.75E-01	-0.00410	0.00862	6.35E-01	-0.00985	0.00301	1.05E-03	0.01072	0.00774	1.66E-01
cg19734370	32	ENSG00000173821	-0.00006	0.00026	8.04E-01	0.00051	0.00158	7.47E-01	0.00233	0.00326	4.75E-01	-0.00008	0.00027	7.77E-01	-0.00032	0.00096	7.39E-01
cg03077331	45	ENSG00000262663	0.00130	0.00232	5.74E-01	0.02758	0.01218	2.36E-02	-0.00279	0.00487	5.67E-01	0.00050	0.00035	1.55E-01	0.00211	0.00337	5.31E-01
cg07148038	89	ENSG00000204392	0.00041	0.00039	2.92E-01	-0.00026	0.00069	7.04E-01	-0.00038	0.00110	7.27E-01	0.00096	0.00051	5.68E-02	-0.00335	0.00622	5.91E-01
cg02206852	52	ENSG00000264290	-0.00206	0.00307	5.02E-01	-0.00437	0.00439	3.20E-01	0.01566	0.00932	9.30E-02	-0.00175	0.00110	1.10E-01	-0.01187	0.00790	1.33E-01
cg02206852	52	ENSG00000264608	-0.00168	0.00251	5.03E-01	0.00268	0.00584	6.46E-01	-0.01362	0.00667	4.12E-02	-0.00015	0.00249	9.52E-01	-0.00144	0.00429	7.38E-01
cg18387671	42	ENSG00000198720	0.00007	0.00014	6.23E-01	0.00043	0.00041	2.94E-01	-0.00071	0.00066	2.83E-01	-0.00006	0.00019	7.73E-01	0.00024	0.00024	3.14E-01
cg02137691	31	ENSG00000159692	-0.00054	0.00274	8.44E-01	0.00439	0.00950	6.44E-01	-0.00250	0.01019	8.07E-01	0.00163	0.00338	6.29E-01	-0.00952	0.00630	1.31E-01
cg02207312	37	ENSG00000134780	-0.00006	0.00016	7.09E-01	-0.00003	0.00064	9.62E-01	-0.00022	0.00035	5.25E-01	0.00003	0.00022	8.88E-01	-0.00017	0.00041	6.79E-01
cg20102034	37	ENSG00000144034	0.00063	0.00170	7.10E-01	-0.00200	0.00223	3.69E-01	-0.00232	0.00320	4.67E-01	0.00391	0.00193	4.28E-02	0.00196	0.00334	5.58E-01
cg19734370	32	ENSG00000260005	0.00010	0.00044	8.21E-01	-0.00029	0.00107	7.89E-01	0.00101	0.00259	6.96E-01	0.00017	0.00188	9.28E-01	0.00014	0.00050	7.75E-01
cg02207312	37	ENSG00000110448	0.00012	0.00034	7.11E-01	-0.00117	0.00072	1.05E-01	0.00099	0.00058	8.63E-02	0.00010	0.00026	6.89E-01	0.00026	0.00057	6.48E-01
cg02206852	52	ENSG00000167543	0.00022	0.00033	5.07E-01	0.00135	0.00302	6.55E-01	0.00021	0.00042	6.17E-01	0.00009	0.00056	8.74E-01	0.00150	0.00196	4.45E-01
cg18387671	42	ENSG00000179761	0.00005	0.00011	6.32E-01	0.00016	0.00025	5.05E-01	-0.00003	0.00004	5.47E-01	0.00063	0.00032	4.91E-02	-0.00008	0.00020	6.68E-01
cg18387671	42	ENSG00000167536	0.00002	0.00004	6.33E-01	0.00020	0.00031	5.13E-01	0.00008	0.00018	6.54E-01	0.00003	0.00005	5.88E-01	-0.00003	0.00009	7.26E-01
cg03077331	45	ENSG00000265678	-0.00071	0.00132	5.91E-01	0.00236	0.00528	6.55E-01	-0.00170	0.00181	3.50E-01	-0.00008	0.00210	9.70E-01	0.00971	0.01494	5.16E-01
cg07957088	43	ENSG00000196421	-0.00195	0.00393	6.21E-01	-0.00917	0.00474	5.31E-02	0.01443	0.00708	4.15E-02	0.00061	0.00131	6.40E-01	-0.00883	0.00354	1.27E-02
cg18387671	42	ENSG00000109118	-0.00016	0.00034	6.38E-01	0.00040	0.00056	4.71E-01	-0.00143	0.00078	6.59E-02	-0.00003	0.00041	9.45E-01	-0.00023	0.00100	8.21E-01

cg02206852	52	ENSG00000109083	-0.00200	0.00312	5.21E-01	0.00079	0.00694	9.10E-01	-0.00780	0.00285	6.30E-03	0.00214	0.00248	3.88E-01	-0.00160	0.00669	8.11E-01
cg02137691	31	ENSG00000127415	0.00054	0.00342	8.75E-01	0.00361	0.00612	5.55E-01	-0.01026	0.00671	1.26E-01	0.00542	0.00370	1.44E-01	-0.00196	0.00643	7.61E-01
cg20102034	37	ENSG00000114956	0.00087	0.00260	7.37E-01	0.00105	0.00765	8.91E-01	0.00814	0.00933	3.83E-01	0.00078	0.00306	7.99E-01	-0.00562	0.00923	5.43E-01
cg19734370	32	ENSG00000181409	-0.00002	0.00012	8.53E-01	-0.00011	0.00013	4.16E-01	0.00023	0.00026	3.79E-01	0.00029	0.00057	6.14E-01	0.00037	0.00134	7.86E-01
cg02207312	37	ENSG00000110079	0.00006	0.00019	7.46E-01	0.00001	0.00036	9.88E-01	-0.00012	0.00063	8.52E-01	0.00013	0.00026	6.21E-01	0.00002	0.00066	9.82E-01
cg06218079	42	ENSG00000176155	0.00036	0.00082	6.58E-01	0.00001	0.00108	9.93E-01	0.00055	0.00153	7.19E-01	-0.00059	0.00066	3.67E-01	0.00505	0.00237	3.27E-02
cg02206852	52	ENSG00000160551	-0.00120	0.00192	5.32E-01	0.00547	0.00620	3.77E-01	-0.00394	0.00442	3.72E-01	-0.00147	0.00248	5.53E-01	-0.00087	0.00566	8.78E-01
cg02206852	52	ENSG00000173065	0.00144	0.00232	5.33E-01	-0.01057	0.00504	3.58E-02	0.00674	0.00584	2.49E-01	0.00221	0.00113	4.99E-02	0.00400	0.00237	9.12E-02
cg20102034	37	ENSG00000065911	-0.00058	0.00183	7.51E-01	0.00196	0.00448	6.61E-01	-0.00433	0.00476	3.63E-01	-0.00158	0.00257	5.39E-01	0.00295	0.00431	4.95E-01
cg20102034	37	ENSG00000188687	-0.00012	0.00039	7.54E-01	0.00030	0.00069	6.69E-01	-0.00049	0.00094	6.00E-01	-0.00025	0.00054	6.48E-01	-0.00376	0.01263	7.66E-01
cg02207312	37	ENSG00000187049	0.00001	0.00002	7.55E-01	-0.00017	0.00044	7.08E-01	0.00036	0.00034	2.87E-01	0.00001	0.00003	7.76E-01	0.00000	0.00003	9.47E-01
cg07148038	89	ENSG00000168394	-0.00201	0.00200	3.15E-01	-0.00317	0.00564	5.74E-01	-0.00783	0.00675	2.46E-01	-0.00146	0.00240	5.42E-01	0.00097	0.00659	8.83E-01
cg07148038	89	ENSG00000204310	0.00071	0.00071	3.15E-01	-0.00007	0.00056	9.01E-01	-0.00050	0.00067	4.52E-01	0.00232	0.00104	2.60E-02	0.00283	0.00161	7.92E-02
cg22127773	80	ENSG00000174282	-0.00098	0.00105	3.51E-01	-0.00332	0.00247	1.78E-01	-0.00300	0.00153	5.01E-02	0.00029	0.00051	5.64E-01	0.00047	0.00224	8.34E-01
cg03077331	45	ENSG00000141526	0.00040	0.00083	6.29E-01	0.00013	0.00591	9.82E-01	0.00099	0.00178	5.78E-01	0.00012	0.00098	9.02E-01	0.00177	0.00353	6.15E-01
cg20102034	37	ENSG00000124356	0.00018	0.00060	7.69E-01	-0.00121	0.00899	8.93E-01	-0.00154	0.01121	8.91E-01	0.00058	0.00065	3.74E-01	-0.00227	0.00163	1.64E-01
cg19734370	32	ENSG00000176108	0.00015	0.00113	8.91E-01	-0.00131	0.00106	2.15E-01	0.00666	0.00275	1.54E-02	0.00055	0.00065	3.93E-01	-0.00192	0.00153	2.08E-01
cg18387671	42	ENSG00000196535	-0.00001	0.00003	6.84E-01	-0.00010	0.00014	4.63E-01	0.00032	0.00043	4.54E-01	0.00005	0.00006	4.14E-01	-0.00004	0.00004	3.49E-01
cg20102034	37	ENSG00000005448	-0.00023	0.00082	7.83E-01	-0.00172	0.00137	2.08E-01	-0.00097	0.00195	6.18E-01	0.00111	0.00066	9.23E-02	-0.00113	0.00165	4.96E-01
cg19734370	32	ENSG00000181523	0.00003	0.00030	9.12E-01	0.00004	0.00027	8.83E-01	-0.00090	0.00061	1.37E-01	0.00062	0.00023	6.28E-03	-0.00032	0.00055	5.59E-01
cg02207312	37	ENSG00000221968	0.00003	0.00010	7.93E-01	0.00032	0.00059	5.89E-01	0.00016	0.00051	7.56E-01	0.00010	0.00012	4.19E-01	-0.00021	0.00020	2.77E-01
cg22127773	80	ENSG00000170175	-0.00046	0.00051	3.67E-01	-0.00063	0.00054	2.44E-01	-0.00159	0.00430	7.11E-01	0.00114	0.00165	4.88E-01	0.00232	0.00707	7.43E-01
cg02137691	31	ENSG00000125386	0.00007	0.00111	9.48E-01	0.00042	0.00169	8.05E-01	-0.00465	0.00232	4.55E-02	0.00057	0.00094	5.40E-01	0.00223	0.00180	2.17E-01
cg18387671	42	ENSG00000109113	-0.00009	0.00025	7.02E-01	-0.00014	0.00050	7.82E-01	-0.00012	0.00044	7.89E-01	0.00002	0.00041	9.62E-01	-0.00039	0.00087	6.55E-01
cg22127773	80	ENSG00000213859	-0.00054	0.00061	3.70E-01	-0.00073	0.00162	6.52E-01	0.00006	0.00108	9.53E-01	-0.00118	0.00087	1.77E-01	0.00158	0.00235	5.00E-01
cg18387671	42	ENSG00000173065	0.00003	0.00008	7.05E-01	0.00028	0.00040	4.72E-01	0.00002	0.00008	8.52E-01	0.00013	0.00039	7.46E-01	-0.00015	0.00112	8.97E-01
cg20102034	37	ENSG00000237883	-0.00060	0.00245	8.05E-01	-0.00199	0.00706	7.78E-01	-0.00585	0.01107	5.98E-01	0.00056	0.00281	8.41E-01	-0.00691	0.00905	4.45E-01
cg22127773	80	ENSG00000129244	-0.00066	0.00074	3.74E-01	-0.00075	0.00152	6.24E-01	0.00325	0.00301	2.80E-01	-0.00082	0.00095	3.83E-01	-0.00191	0.00247	4.38E-01
cg20102034	37	ENSG00000163170	0.00064	0.00267	8.10E-01	-0.00847	0.00475	7.47E-02	-0.00277	0.00546	6.13E-01	0.00547	0.00318	8.51E-02	0.00302	0.00179	9.20E-02
cg06218079	42	ENSG00000185813	-0.00019	0.00052	7.15E-01	-0.00037	0.00029	2.10E-01	0.00077	0.00034	2.31E-02	0.00022	0.00045	6.31E-01	-0.00468	0.00162	3.90E-03
cg07957088	43	ENSG00000198276	0.00023	0.00058	6.99E-01	-0.00475	0.00556	3.93E-01	-0.00281	0.00850	7.41E-01	0.00025	0.00059	6.67E-01	0.00704	0.00742	3.43E-01
cg02137691	31	ENSG00000163945	0.00010	0.00247	9.69E-01	0.00672	0.00653	3.04E-01	-0.00380	0.00800	6.34E-01	-0.00067	0.00299	8.22E-01	-0.00056	0.00884	9.49E-01
cg02207312	37	ENSG00000149532	0.00004	0.00016	8.15E-01	-0.00023	0.00051	6.57E-01	0.00001	0.00037	9.68E-01	0.00013	0.00021	5.28E-01	-0.00023	0.00052	6.61E-01
cg02206852	52	ENSG00000227543	-0.00198	0.00360	5.82E-01	0.00552	0.00368	1.34E-01	-0.01431	0.01019	1.60E-01	-0.00264	0.00548	6.30E-01	-0.00524	0.00359	1.45E-01
cg19734370	32	ENSG00000141570	-0.00010	0.00146	9.47E-01	-0.00055	0.00147	7.08E-01	-0.00138	0.00343	6.88E-01	0.00218	0.00162	1.81E-01	-0.01035	0.00642	1.07E-01
cg18387671	42	ENSG00000108587	0.00010	0.00027	7.22E-01	-0.00033	0.00057	5.62E-01	0.00061	0.00075	4.16E-01	0.00016	0.00035	6.45E-01	-0.00007	0.00110	9.49E-01
cg22127773	80	ENSG00000196544	0.00016	0.00018	3.79E-01	0.00018	0.00019	3.27E-01	-0.00019	0.00249	9.40E-01	-0.00034	0.00104	7.43E-01	-0.00216	0.00477	6.51E-01
cg02207312	37	ENSG00000124920	0.00010	0.00043	8.22E-01	-0.00067	0.00037	6.93E-02	-0.00034	0.00085	6.85E-01	0.00041	0.00034	2.22E-01	0.00146	0.00086	8.92E-02
cg02137691	31	ENSG00000163950	0.00004	0.00199	9.84E-01	0.00296	0.00463	5.22E-01	-0.00417	0.00544	4.43E-01	-0.00024	0.00264	9.27E-01	0.00174	0.00605	7.74E-01
cg07957088	43	ENSG00000101210	-0.00133	0.00360	7.11E-01	0.00540	0.00542	3.18E-01	-0.00229	0.00777	7.68E-01	-0.00002	0.00253	9.94E-01	-0.01812	0.00889	4.14E-02
cg19734370	32	ENSG00000141543	-0.00006	0.00111	9.59E-01	0.00116	0.00135	3.90E-01	-0.00208	0.00269	4.40E-01	0.00032	0.00129	8.03E-01	-0.00880	0.00540	1.03E-01
cg20102034	37	ENSG00000135622	0.00050	0.00235	8.32E-01	0.00288	0.00585	6.23E-01	-0.01204	0.00791	1.28E-01	0.00147	0.00280	6.00E-01	0.00131	0.01074	9.03E-01

cg20102034	37	ENSG00000187605	-0.00107	0.00508	8.33E-01	0.00381	0.00898	6.71E-01	0.01572	0.00979	1.08E-01	-0.00710	0.00440	1.07E-01	-0.00847	0.00817	3.00E-01
cg20102034	37	ENSG00000115289	-0.00070	0.00336	8.34E-01	0.00250	0.00868	7.73E-01	0.01231	0.01238	3.20E-01	-0.00291	0.00401	4.69E-01	0.00075	0.01245	9.52E-01
cg02207312	37	ENSG00000110446	-0.00004	0.00021	8.36E-01	-0.00059	0.00057	2.96E-01	0.00041	0.00049	3.98E-01	-0.00015	0.00029	6.21E-01	0.00024	0.00055	6.56E-01
cg02137691	31	ENSG00000159674	0.00001	0.00197	9.98E-01	0.00573	0.00348	1.00E-01	0.00011	0.00362	9.76E-01	0.00029	0.00128	8.22E-01	-0.00610	0.00334	6.80E-02
cg07957088	43	ENSG00000197114	0.00107	0.00302	7.23E-01	0.00372	0.00638	5.59E-01	-0.00174	0.00817	8.32E-01	0.00107	0.00467	8.18E-01	0.00010	0.00643	9.87E-01
cg19734370	32	ENSG00000175911	-0.00001	0.00040	9.73E-01	0.00022	0.00039	5.76E-01	0.00067	0.00092	4.67E-01	-0.00136	0.00083	1.01E-01	0.00043	0.00190	8.23E-01
cg02207312	37	ENSG00000167987	0.00001	0.00006	8.48E-01	-0.00001	0.00013	9.22E-01	0.00002	0.00006	8.11E-01	0.00011	0.00038	7.72E-01	-0.00033	0.00090	7.12E-01
cg07957088	43	ENSG00000101152	-0.00289	0.00846	7.33E-01	0.01930	0.00758	1.09E-02	-0.03407	0.01110	2.15E-03	0.00083	0.00349	8.12E-01	-0.00438	0.00914	6.32E-01
cg22127773	80	ENSG00000161958	-0.00011	0.00013	3.95E-01	0.00039	0.00105	7.08E-01	0.00004	0.00222	9.85E-01	-0.00011	0.00013	4.04E-01	-0.00415	0.00271	1.26E-01
cg06218079	42	ENSG00000266236	0.00014	0.00047	7.57E-01	-0.00002	0.00064	9.79E-01	0.00059	0.00075	4.34E-01	-0.00022	0.00168	8.97E-01	-0.00488	0.00431	2.57E-01
cg22127773	80	ENSG00000132510	0.00038	0.00045	4.02E-01	0.00022	0.00057	6.94E-01	0.00197	0.00087	2.42E-02	-0.00015	0.00028	5.97E-01	0.00096	0.00331	7.72E-01
cg07957088	43	ENSG00000101150	0.00049	0.00158	7.56E-01	0.00437	0.00395	2.68E-01	-0.00338	0.00558	5.45E-01	-0.00003	0.00197	9.87E-01	0.00074	0.00475	8.76E-01
cg03077331	45	ENSG00000141562	0.00038	0.00108	7.24E-01	-0.00298	0.00889	7.38E-01	0.00116	0.00324	7.20E-01	0.00043	0.00117	7.17E-01	-0.00249	0.00672	7.11E-01
cg07148038	89	ENSG00000204472	0.00150	0.00165	3.66E-01	-0.00013	0.00543	9.81E-01	0.00181	0.00699	7.96E-01	0.00102	0.00210	6.26E-01	0.00334	0.00344	3.32E-01
cg18387671	42	ENSG00000160551	-0.00003	0.00010	7.78E-01	0.00000	0.00015	9.94E-01	0.00030	0.00049	5.48E-01	-0.00012	0.00017	4.85E-01	0.00001	0.00029	9.63E-01
cg07148038	89	ENSG00000204366	-0.00043	0.00047	3.69E-01	0.00057	0.00117	6.28E-01	-0.00191	0.00144	1.86E-01	-0.00041	0.00056	4.69E-01	-0.00208	0.00444	6.40E-01
cg07957088	43	ENSG00000149658	-0.00018	0.00059	7.66E-01	-0.00110	0.00144	4.45E-01	0.00161	0.00184	3.81E-01	-0.00289	0.00333	3.85E-01	-0.00009	0.00071	8.95E-01
cg18387671	42	ENSG00000263370	-0.00003	0.00012	7.89E-01	-0.00032	0.00039	4.10E-01	-0.00003	0.00013	8.13E-01	0.00001	0.00045	9.88E-01	0.00097	0.00078	2.12E-01
cg02206852	52	ENSG00000109084	-0.00022	0.00048	6.38E-01	0.00572	0.00726	4.31E-01	-0.00059	0.00835	9.43E-01	-0.00013	0.00051	7.93E-01	-0.00116	0.00142	4.14E-01
cg07957088	43	ENSG00000207554	-0.00098	0.00337	7.72E-01	-0.00475	0.00284	9.37E-02	0.00533	0.00439	2.25E-01	0.00560	0.00641	3.83E-01	-0.00852	0.00590	1.49E-01
cg02206852	52	ENSG00000237575	0.00155	0.00331	6.39E-01	-0.00267	0.00324	4.09E-01	0.02492	0.01426	8.05E-02	0.00554	0.00398	1.63E-01	-0.00153	0.00455	7.36E-01
cg03077331	45	ENSG00000169689	-0.00017	0.00053	7.45E-01	0.01608	0.01184	1.74E-01	-0.00405	0.00421	3.36E-01	-0.00001	0.00025	9.82E-01	-0.00102	0.00135	4.49E-01
cg06218079	42	ENSG00000141526	-0.00004	0.00014	8.03E-01	0.00009	0.00019	6.25E-01	0.00024	0.00023	2.89E-01	-0.00028	0.00013	3.79E-02	-0.00016	0.00061	7.99E-01
cg18387671	42	ENSG00000167525	0.00001	0.00005	8.05E-01	0.00002	0.00010	8.08E-01	-0.00002	0.00008	8.09E-01	-0.00001	0.00012	9.50E-01	0.00023	0.00020	2.52E-01
cg18387671	42	ENSG00000132591	0.00007	0.00027	8.08E-01	-0.00029	0.00038	4.53E-01	0.00183	0.00099	6.56E-02	0.00007	0.00025	7.72E-01	-0.00005	0.00095	9.56E-01
cg06218079	42	ENSG00000183684	0.00001	0.00004	8.09E-01	-0.00005	0.00005	3.01E-01	0.00006	0.00006	2.78E-01	0.00011	0.00015	4.48E-01	0.00070	0.00084	4.00E-01
cg06218079	42	ENSG00000169733	0.00012	0.00051	8.12E-01	-0.00078	0.00084	3.50E-01	-0.00131	0.00110	2.34E-01	0.00055	0.00031	7.96E-02	0.00139	0.00099	1.59E-01
cg03077331	45	ENSG00000225663	0.00025	0.00081	7.59E-01	0.00507	0.00788	5.20E-01	0.00145	0.00338	6.67E-01	-0.00055	0.00025	2.44E-02	0.00178	0.00121	1.41E-01
cg20102034	37	ENSG00000114978	0.00016	0.00175	9.28E-01	-0.00304	0.00433	4.83E-01	0.00243	0.00565	6.67E-01	-0.00011	0.00218	9.61E-01	0.00529	0.00574	3.57E-01
cg02206852	52	ENSG00000109103	0.00085	0.00193	6.60E-01	0.00640	0.00466	1.69E-01	-0.01030	0.00925	2.65E-01	0.00013	0.00220	9.54E-01	0.00271	0.00952	7.76E-01
cg20102034	37	ENSG00000159374	-0.00005	0.00052	9.31E-01	0.00025	0.00076	7.45E-01	-0.00047	0.00091	6.01E-01	-0.00020	0.00134	8.82E-01	0.00050	0.00231	8.28E-01
cg06218079	42	ENSG00000169689	0.00020	0.00088	8.21E-01	-0.00074	0.00119	5.35E-01	-0.00012	0.00120	9.19E-01	-0.00031	0.00065	6.38E-01	0.00692	0.00288	1.61E-02
cg22127773	80	ENSG00000233223	0.00027	0.00035	4.32E-01	0.00003	0.00046	9.53E-01	0.00274	0.00177	1.23E-01	0.00020	0.00035	5.70E-01	0.00392	0.00312	2.08E-01
cg18387671	42	ENSG00000126653	-0.00004	0.00020	8.27E-01	0.00039	0.00021	5.74E-02	-0.00082	0.00107	4.47E-01	-0.00013	0.00011	2.06E-01	-0.00043	0.00035	2.09E-01
cg22127773	80	ENSG00000220205	0.00027	0.00034	4.38E-01	-0.00069	0.00097	4.79E-01	-0.00091	0.00231	6.93E-01	0.00044	0.00038	2.40E-01	0.00045	0.00439	9.18E-01
cg03077331	45	ENSG00000261888	-0.00038	0.00138	7.82E-01	0.00222	0.00407	5.86E-01	-0.00106	0.00175	5.44E-01	0.00050	0.00270	8.52E-01	-0.01074	0.01357	4.29E-01
cg03077331	45	ENSG00000141552	-0.00037	0.00138	7.86E-01	-0.00405	0.01109	7.15E-01	-0.00036	0.00453	9.36E-01	-0.00018	0.00148	9.05E-01	-0.00533	0.00901	5.54E-01
cg06218079	42	ENSG00000167363	-0.00032	0.00161	8.42E-01	0.00126	0.00105	2.29E-01	0.00134	0.00101	1.87E-01	-0.00393	0.00094	3.02E-05	0.00073	0.00361	8.39E-01
cg07148038	89	ENSG00000206337	0.00066	0.00078	3.98E-01	0.00084	0.00081	2.96E-01	-0.00096	0.00856	9.11E-01	-0.00175	0.00343	6.10E-01	-0.00220	0.00700	7.53E-01
cg07957088	43	ENSG00000203880	0.00025	0.00111	8.24E-01	0.01129	0.01180	3.39E-01	0.00272	0.01677	8.71E-01	-0.00304	0.00252	2.28E-01	0.00091	0.00125	4.64E-01
cg20102034	37	ENSG00000115282	0.00018	0.00346	9.60E-01	0.00820	0.00686	2.32E-01	-0.00573	0.00836	4.93E-01	0.00207	0.00306	4.99E-01	-0.01118	0.00812	1.69E-01
cg07957088	43	ENSG00000101197	-0.00092	0.00421	8.26E-01	0.00465	0.00787	5.55E-01	-0.00052	0.00982	9.58E-01	0.00333	0.00286	2.45E-01	-0.01255	0.00612	4.04E-02

cg07148038	89	ENSG00000204469	-0.00130	0.00154	4.00E-01	-0.00063	0.00248	7.98E-01	-0.01127	0.00607	6.35E-02	-0.00096	0.00228	6.73E-01	0.00105	0.00488	8.29E-01
cg22127773	80	ENSG00000161960	-0.00017	0.00022	4.49E-01	-0.00014	0.00027	6.12E-01	-0.00015	0.00047	7.58E-01	-0.00081	0.00087	3.48E-01	0.00016	0.00096	8.65E-01
cg07957088	43	ENSG00000225978	0.00016	0.00081	8.39E-01	-0.00464	0.00845	5.83E-01	-0.01265	0.01222	3.01E-01	0.00032	0.00081	6.97E-01	-0.01465	0.01382	2.89E-01
cg18387671	42	ENSG00000076604	-0.00009	0.00049	8.61E-01	-0.00083	0.00050	9.62E-02	-0.00038	0.00042	3.64E-01	0.00093	0.00027	5.28E-04	-0.00036	0.00083	6.70E-01
cg02207312	37	ENSG00000149476	0.00000	0.00009	9.80E-01	0.00000	0.00073	1.00E+00	0.00031	0.00025	2.16E-01	-0.00008	0.00010	4.46E-01	0.00022	0.00028	4.23E-01
cg02207312	37	ENSG00000166927	0.00000	0.00004	9.81E-01	-0.00088	0.00065	1.75E-01	-0.00002	0.00014	9.14E-01	-0.00001	0.00005	8.06E-01	0.00003	0.00007	6.10E-01
cg03077331	45	ENSG00000268852	0.00042	0.00173	8.08E-01	-0.00807	0.00807	3.17E-01	-0.00353	0.00335	2.93E-01	0.00139	0.00229	5.43E-01	0.00301	0.00259	2.46E-01
cg07957088	43	ENSG00000101199	0.00010	0.00054	8.47E-01	-0.00181	0.00831	8.28E-01	-0.00060	0.01083	9.56E-01	0.00014	0.00059	8.11E-01	-0.00003	0.00136	9.83E-01
cg02206852	52	ENSG00000232859	-0.00027	0.00069	7.00E-01	0.00887	0.01132	4.33E-01	-0.00036	0.00525	9.45E-01	-0.00035	0.00070	6.21E-01	0.00971	0.01031	3.46E-01
cg18387671	42	ENSG00000264304	0.00002	0.00011	8.72E-01	-0.00012	0.00020	5.50E-01	0.00162	0.00159	3.07E-01	0.00001	0.00006	8.18E-01	0.00099	0.00065	1.31E-01
cg20102034	37	ENSG00000159399	0.00000	0.00077	9.95E-01	0.00022	0.00154	8.84E-01	0.00242	0.00204	2.35E-01	-0.00125	0.00053	1.95E-02	0.00076	0.00098	4.41E-01
cg03077331	45	ENSG00000181396	-0.00014	0.00062	8.19E-01	-0.00108	0.00206	5.99E-01	0.00027	0.00075	7.13E-01	-0.00130	0.00140	3.54E-01	0.00158	0.00526	7.63E-01
cg20102034	37	ENSG00000179528	-0.00001	0.00631	9.99E-01	-0.00359	0.00787	6.49E-01	0.03424	0.01292	8.06E-03	-0.00779	0.00395	4.86E-02	-0.00570	0.00641	3.75E-01
cg03077331	45	ENSG00000169710	-0.00019	0.00084	8.24E-01	0.00584	0.00551	2.89E-01	0.00076	0.00208	7.14E-01	-0.00052	0.00094	5.82E-01	-0.00188	0.00612	7.59E-01
cg06218079	42	ENSG00000169696	-0.00005	0.00033	8.86E-01	-0.00198	0.00124	1.12E-01	-0.00041	0.00133	7.60E-01	-0.00007	0.00013	5.78E-01	0.00073	0.00061	2.34E-01
cg22127773	80	ENSG00000170291	0.00015	0.00020	4.70E-01	-0.00112	0.00158	4.79E-01	0.00012	0.00112	9.11E-01	0.00016	0.00021	4.57E-01	0.00113	0.00177	5.24E-01
cg03077331	45	ENSG00000176155	-0.00028	0.00138	8.37E-01	0.00973	0.01175	4.08E-01	0.00079	0.00386	8.39E-01	-0.00087	0.00152	5.66E-01	0.00466	0.00669	4.87E-01
cg07957088	43	ENSG00000101191	0.00055	0.00358	8.78E-01	0.00464	0.00700	5.08E-01	0.00981	0.00866	2.58E-01	-0.00555	0.00419	1.85E-01	0.00376	0.00808	6.41E-01
cg18387671	42	ENSG00000167549	-0.00003	0.00024	9.00E-01	0.00014	0.00059	8.12E-01	-0.00118	0.00117	3.14E-01	-0.00021	0.00028	4.50E-01	0.00071	0.00056	2.05E-01
cg06218079	42	ENSG00000173762	0.00001	0.00009	9.03E-01	0.00015	0.00021	4.61E-01	-0.00002	0.00024	9.36E-01	-0.00001	0.00011	9.06E-01	-0.00010	0.00036	7.76E-01
cg18387671	42	ENSG00000108578	-0.00002	0.00015	9.05E-01	-0.00004	0.00036	9.07E-01	0.00005	0.00020	7.94E-01	-0.00025	0.00030	4.07E-01	0.00046	0.00069	5.03E-01
cg07148038	89	ENSG00000204428	-0.00248	0.00313	4.28E-01	0.00076	0.00296	7.98E-01	-0.02290	0.01233	6.34E-02	-0.00332	0.00430	4.40E-01	-0.00599	0.01281	6.40E-01
cg06218079	42	ENSG00000183010	-0.00002	0.00015	9.10E-01	0.00004	0.00022	8.56E-01	-0.00006	0.00023	7.90E-01	-0.00011	0.00075	8.85E-01	-0.00121	0.00237	6.10E-01
cg18387671	42	ENSG00000168792	0.00000	0.00004	9.11E-01	0.00000	0.00005	9.32E-01	-0.00087	0.00089	3.28E-01	0.00007	0.00035	8.37E-01	0.00060	0.00073	4.14E-01
cg18387671	42	ENSG00000221995	0.00004	0.00039	9.12E-01	0.00030	0.00046	5.14E-01	0.00196	0.00099	4.73E-02	-0.00003	0.00013	7.83E-01	-0.00131	0.00067	5.11E-02
cg22127773	80	ENSG00000181885	0.00061	0.00086	4.79E-01	0.00062	0.00084	4.61E-01	0.00288	0.00135	3.31E-02	-0.00101	0.00101	3.18E-01	0.00013	0.00381	9.74E-01
cg06218079	42	ENSG00000141568	-0.00009	0.00087	9.15E-01	-0.00030	0.00093	7.50E-01	-0.00167	0.00098	8.73E-02	-0.00007	0.00063	9.16E-01	0.00725	0.00307	1.83E-02
cg06218079	42	ENSG00000169718	0.00004	0.00040	9.23E-01	-0.00043	0.00058	4.54E-01	0.00027	0.00063	6.62E-01	-0.00002	0.00051	9.62E-01	0.00494	0.00270	6.72E-02
cg22127773	80	ENSG00000182224	-0.00013	0.00019	4.86E-01	-0.00236	0.00148	1.12E-01	0.00120	0.00219	5.84E-01	-0.00010	0.00019	6.02E-01	-0.00118	0.00289	6.83E-01
cg18387671	42	ENSG00000108256	0.00001	0.00015	9.31E-01	0.00021	0.00028	4.59E-01	-0.00061	0.00083	4.59E-01	-0.00008	0.00019	6.83E-01	0.00026	0.00051	6.03E-01
cg03077331	45	ENSG00000266236	0.00009	0.00053	8.73E-01	0.01066	0.01500	4.77E-01	-0.00522	0.00632	4.09E-01	0.00007	0.00054	8.91E-01	0.00219	0.00411	5.94E-01
cg22127773	80	ENSG00000262251	-0.00033	0.00049	4.91E-01	-0.00150	0.00149	3.13E-01	-0.00200	0.00194	3.03E-01	-0.00022	0.00031	4.78E-01	0.00329	0.00265	2.13E-01
cg22127773	80	ENSG00000215067	-0.00026	0.00037	4.92E-01	-0.00022	0.00040	5.85E-01	0.00350	0.00296	2.37E-01	-0.00123	0.00119	3.01E-01	0.00003	0.00453	9.95E-01
cg18387671	42	ENSG00000265625	0.00002	0.00027	9.39E-01	-0.00021	0.00027	4.44E-01	0.00255	0.00159	1.08E-01	0.00038	0.00044	3.89E-01	-0.00021	0.00046	6.52E-01
cg03077331	45	ENSG00000185813	-0.00018	0.00116	8.77E-01	-0.00675	0.00828	4.15E-01	-0.00165	0.00424	6.97E-01	0.00014	0.00124	9.10E-01	-0.00140	0.00635	8.26E-01
cg07957088	43	ENSG00000196700	0.00029	0.00293	9.20E-01	-0.00383	0.00576	5.06E-01	-0.01842	0.01079	8.80E-02	0.00075	0.00292	7.96E-01	0.00467	0.00233	4.54E-02
cg18387671	42	ENSG00000264290	0.00001	0.00019	9.48E-01	0.00078	0.00084	3.54E-01	0.00027	0.00079	7.33E-01	0.00034	0.00032	2.79E-01	-0.00020	0.00013	1.22E-01
cg06218079	42	ENSG00000141542	-0.00002	0.00032	9.54E-01	0.00024	0.00062	6.99E-01	-0.00017	0.00055	7.55E-01	-0.00011	0.00053	8.29E-01	0.00046	0.00163	7.78E-01
cg03077331	45	ENSG00000197063	-0.00012	0.00088	8.94E-01	0.00021	0.00124	8.63E-01	0.00079	0.00059	1.77E-01	-0.00158	0.00140	2.60E-01	-0.01199	0.00720	9.62E-02
cg06218079	42	ENSG00000169683	0.00001	0.00027	9.61E-01	-0.00034	0.00044	4.36E-01	-0.00022	0.00043	6.16E-01	0.00016	0.00025	5.25E-01	0.00271	0.00152	7.40E-02
cg07148038	89	ENSG00000204390	-0.00368	0.00492	4.54E-01	-0.01222	0.00513	1.73E-02	0.00487	0.00623	4.34E-01	-0.01095	0.00284	1.16E-04	0.00776	0.00641	2.26E-01
cg18387671	42	ENSG00000132581	-0.00001	0.00019	9.68E-01	0.00006	0.00057	9.09E-01	0.00027	0.00033	4.09E-01	-0.00011	0.00028	6.82E-01	-0.00068	0.00068	3.17E-01

cg22127773	80	ENSG00000161956	0.00007	0.00010	5.08E-01	0.00013	0.00012	2.70E-01	-0.00016	0.00023	4.90E-01	-0.00020	0.00104	8.47E-01	0.00006	0.00037	8.80E-01
cg02206852	52	ENSG00000264577	0.00070	0.00254	7.84E-01	-0.00141	0.00593	8.13E-01	0.00277	0.00431	5.20E-01	0.00192	0.00467	6.81E-01	-0.00326	0.00605	5.90E-01
cg18387671	42	ENSG00000176927	0.00000	0.00004	9.73E-01	0.00002	0.00010	8.28E-01	-0.00066	0.00080	4.10E-01	0.00003	0.00005	5.15E-01	-0.00008	0.00008	3.00E-01
cg06218079	42	ENSG00000181396	-0.00001	0.00018	9.75E-01	0.00005	0.00084	9.54E-01	0.00085	0.00103	4.13E-01	-0.00018	0.00011	9.99E-02	0.00040	0.00033	2.22E-01
cg22127773	80	ENSG00000129245	-0.00029	0.00044	5.12E-01	-0.00026	0.00077	7.31E-01	-0.00111	0.00122	3.66E-01	0.00003	0.00062	9.65E-01	-0.00614	0.00414	1.38E-01
cg07957088	43	ENSG00000101194	0.00004	0.00094	9.65E-01	0.00151	0.00294	6.08E-01	-0.00065	0.00355	8.54E-01	-0.00003	0.00108	9.79E-01	-0.00056	0.00330	8.66E-01
cg03077331	45	ENSG00000260563	0.00020	0.00259	9.39E-01	-0.00178	0.01675	9.15E-01	0.00358	0.00660	5.87E-01	0.00187	0.00108	8.26E-02	-0.00969	0.00586	9.79E-02
cg22127773	80	ENSG00000174292	0.00075	0.00119	5.29E-01	0.00088	0.00069	1.99E-01	0.00648	0.00431	1.33E-01	-0.00014	0.00193	9.44E-01	-0.01026	0.00771	1.83E-01
cg07957088	43	ENSG00000232442	-0.00006	0.00350	9.87E-01	0.00467	0.01308	7.21E-01	-0.01746	0.01706	3.06E-01	0.00495	0.00749	5.09E-01	-0.00110	0.00428	7.97E-01
cg22127773	80	ENSG00000170296	0.00041	0.00065	5.32E-01	0.00018	0.00081	8.26E-01	0.00149	0.00313	6.34E-01	0.00081	0.00119	4.94E-01	-0.00088	0.00505	8.61E-01
cg07148038	89	ENSG00000223865	0.00151	0.00213	4.78E-01	0.00011	0.00574	9.85E-01	-0.00198	0.00216	3.59E-01	0.00428	0.00120	3.66E-04	0.00252	0.00604	6.76E-01
cg07957088	43	ENSG00000101161	0.00001	0.00116	9.95E-01	0.00201	0.00139	1.46E-01	-0.00378	0.00213	7.49E-02	-0.00090	0.00080	2.60E-01	0.00217	0.00180	2.28E-01
cg03077331	45	ENSG00000266445	0.00004	0.00090	9.63E-01	0.00126	0.00287	6.60E-01	0.00057	0.00118	6.30E-01	-0.00131	0.00163	4.22E-01	-0.00236	0.01200	8.44E-01
cg07148038	89	ENSG00000204287	-0.00144	0.00207	4.87E-01	-0.00315	0.00432	4.66E-01	0.00845	0.00592	1.53E-01	-0.00227	0.00220	3.01E-01	-0.00525	0.00758	4.88E-01
cg07148038	89	ENSG00000204444	0.00254	0.00366	4.88E-01	0.01488	0.00675	2.74E-02	0.00429	0.00596	4.71E-01	0.00082	0.00249	7.41E-01	-0.00748	0.00643	2.45E-01
cg02206852	52	ENSG00000198242	-0.00034	0.00165	8.37E-01	-0.01251	0.01011	2.16E-01	-0.00997	0.00733	1.74E-01	0.00042	0.00287	8.83E-01	0.00054	0.00134	6.85E-01
cg22127773	80	ENSG00000262943	-0.00043	0.00073	5.52E-01	0.00019	0.00044	6.58E-01	-0.00151	0.00285	5.96E-01	-0.00079	0.00096	4.13E-01	-0.00849	0.00465	6.76E-02
cg03077331	45	ENSG00000141560	-0.00002	0.00115	9.89E-01	-0.00055	0.00722	9.40E-01	-0.00147	0.00256	5.67E-01	0.00018	0.00134	8.91E-01	0.00481	0.00634	4.48E-01
cg02206852	52	ENSG00000264098	0.00091	0.00543	8.67E-01	0.00394	0.00734	5.91E-01	0.00554	0.00697	4.26E-01	0.00721	0.00431	9.45E-02	-0.00995	0.00258	1.14E-04
cg22127773	80	ENSG00000198150	0.00015	0.00026	5.66E-01	0.00176	0.00115	1.25E-01	-0.00006	0.00036	8.58E-01	0.00027	0.00057	6.35E-01	0.00017	0.00057	7.61E-01
cg22127773	80	ENSG00000258315	-0.00028	0.00049	5.68E-01	-0.00224	0.00161	1.64E-01	-0.00013	0.00072	8.54E-01	0.00043	0.00092	6.43E-01	-0.00082	0.00122	5.05E-01
cg22127773	80	ENSG00000175826	-0.00034	0.00060	5.72E-01	0.00176	0.00105	9.47E-02	-0.00099	0.00076	1.92E-01	-0.00023	0.00033	4.95E-01	-0.00235	0.00137	8.66E-02
cg22127773	80	ENSG00000161939	-0.00009	0.00016	5.73E-01	-0.00069	0.00082	4.00E-01	0.00104	0.00132	4.28E-01	-0.00007	0.00016	6.61E-01	-0.00334	0.00276	2.26E-01
cg07148038	89	ENSG00000204264	0.00222	0.00343	5.17E-01	0.01431	0.00640	2.53E-02	-0.00046	0.00756	9.52E-01	0.00177	0.00247	4.74E-01	-0.00514	0.00548	3.48E-01
cg07148038	89	ENSG00000231389	-0.00078	0.00121	5.21E-01	0.00104	0.00453	8.18E-01	0.00111	0.00247	6.55E-01	-0.00153	0.00147	2.99E-01	-0.00496	0.00931	5.94E-01
cg02206852	52	ENSG00000007202	0.00019	0.00159	9.05E-01	0.00107	0.00616	8.62E-01	-0.00247	0.00756	7.44E-01	-0.00013	0.00172	9.39E-01	0.00882	0.00808	2.75E-01
cg07148038	89	ENSG00000204435	-0.00168	0.00274	5.40E-01	0.01091	0.00824	1.86E-01	-0.00295	0.00231	2.02E-01	0.00133	0.00110	2.25E-01	-0.00902	0.00337	7.47E-03
cg07148038	89	ENSG00000204520	0.00064	0.00104	5.40E-01	0.00187	0.00096	5.13E-02	-0.00607	0.00415	1.43E-01	0.00016	0.00148	9.14E-01	0.00010	0.00275	9.72E-01
cg22127773	80	ENSG00000205544	0.00030	0.00058	6.09E-01	0.00076	0.00068	2.61E-01	-0.00202	0.00277	4.65E-01	-0.00054	0.00131	6.81E-01	-0.00184	0.00310	5.52E-01
cg22127773	80	ENSG00000179593	-0.00025	0.00049	6.11E-01	-0.00152	0.00089	8.73E-02	-0.00077	0.00128	5.50E-01	0.00018	0.00033	5.75E-01	0.00147	0.00213	4.90E-01
cg02206852	52	ENSG00000076351	-0.00005	0.00068	9.41E-01	0.00743	0.00669	2.66E-01	-0.00004	0.00467	9.93E-01	-0.00007	0.00069	9.14E-01	-0.01118	0.00975	2.51E-01
cg22127773	80	ENSG00000214999	-0.00032	0.00064	6.14E-01	-0.00209	0.00136	1.23E-01	-0.00006	0.00250	9.81E-01	0.00021	0.00079	7.94E-01	0.00028	0.00292	9.23E-01
cg02206852	52	ENSG00000004142	-0.00014	0.00227	9.51E-01	-0.00460	0.00355	1.95E-01	-0.00427	0.00375	2.55E-01	0.00342	0.00200	8.70E-02	0.00276	0.00383	4.72E-01
cg07148038	89	ENSG00000196735	-0.00231	0.00393	5.56E-01	0.00419	0.00404	2.99E-01	-0.01377	0.00491	5.01E-03	-0.00471	0.00270	8.06E-02	0.00559	0.00544	3.04E-01
cg07148038	89	ENSG00000204525	-0.00132	0.00227	5.62E-01	-0.00557	0.00302	6.55E-02	0.00636	0.00667	3.40E-01	-0.00396	0.00236	9.29E-02	0.00161	0.00125	1.97E-01
cg07148038	89	ENSG00000204305	-0.00150	0.00269	5.78E-01	-0.00383	0.00454	4.00E-01	-0.01883	0.00694	6.64E-03	-0.00009	0.00120	9.40E-01	0.00391	0.00188	3.71E-02
cg22127773	80	ENSG00000266824	-0.00016	0.00034	6.44E-01	-0.00112	0.00098	2.53E-01	-0.00065	0.00074	3.85E-01	0.00023	0.00027	4.00E-01	-0.00122	0.00134	3.65E-01
cg07148038	89	ENSG00000226979	-0.00147	0.00265	5.79E-01	0.00668	0.00563	2.35E-01	-0.01097	0.00285	1.21E-04	-0.00009	0.00125	9.42E-01	0.00119	0.00178	5.05E-01
cg22127773	80	ENSG00000170004	0.00037	0.00080	6.49E-01	-0.00030	0.00122	8.05E-01	0.00305	0.00274	2.65E-01	0.00051	0.00121	6.75E-01	0.00029	0.00389	9.40E-01
cg02206852	52	ENSG00000167549	0.00000	0.00021	9.98E-01	-0.00044	0.00172	7.98E-01	-0.00593	0.00392	1.31E-01	0.00008	0.00025	7.36E-01	-0.00015	0.00043	7.28E-01
cg07148038	89	ENSG00000168477	-0.00069	0.00134	6.03E-01	-0.00480	0.00315	1.27E-01	-0.00146	0.00426	7.32E-01	0.00096	0.00171	5.76E-01	-0.00239	0.00398	5.47E-01
cg07148038	89	ENSG00000198563	0.00129	0.00258	6.15E-01	0.00368	0.00639	5.65E-01	0.01135	0.00911	2.13E-01	-0.00018	0.00314	9.54E-01	-0.00104	0.00880	9.06E-01

cg22127773	80	ENSG00000170043	-0.00005	0.00014	6.91E-01	0.00045	0.00105	6.67E-01	-0.00040	0.00036	2.75E-01	-0.00001	0.00015	9.63E-01	-0.00001	0.00065	9.92E-01
cg07148038	89	ENSG00000198502	-0.00094	0.00192	6.24E-01	0.00344	0.00499	4.90E-01	-0.00547	0.00629	3.84E-01	-0.00129	0.00238	5.89E-01	-0.00098	0.00580	8.66E-01
cg22127773	80	ENSG00000132518	-0.00017	0.00044	6.96E-01	-0.00219	0.00156	1.59E-01	-0.00161	0.00275	5.57E-01	-0.00001	0.00015	9.49E-01	0.00447	0.00462	3.33E-01
cg07148038	89	ENSG00000237541	0.00083	0.00173	6.30E-01	0.00291	0.00369	4.31E-01	-0.00263	0.00178	1.40E-01	0.00108	0.00091	2.35E-01	0.01086	0.00599	7.01E-02
cg22127773	80	ENSG00000178971	-0.00027	0.00076	7.22E-01	0.00034	0.00097	7.26E-01	-0.00160	0.00284	5.74E-01	-0.00139	0.00140	3.20E-01	0.00255	0.00567	6.54E-01
cg22127773	80	ENSG00000161970	-0.00002	0.00005	7.24E-01	-0.00006	0.00006	3.61E-01	0.00011	0.00012	3.81E-01	0.00002	0.00017	8.83E-01	0.00000	0.00021	9.83E-01
cg22127773	80	ENSG00000262089	0.00040	0.00115	7.31E-01	0.00145	0.00105	1.68E-01	0.00526	0.00357	1.41E-01	-0.00068	0.00044	1.24E-01	-0.00686	0.00566	2.25E-01
cg07148038	89	ENSG00000204314	-0.00031	0.00074	6.76E-01	0.00096	0.00111	3.89E-01	0.00121	0.00152	4.23E-01	-0.00176	0.00042	2.23E-05	-0.00024	0.00067	7.26E-01
cg07148038	89	ENSG00000204386	-0.00039	0.00094	6.77E-01	-0.00435	0.00274	1.12E-01	-0.00425	0.00364	2.43E-01	0.00088	0.00171	6.08E-01	0.00019	0.00034	5.82E-01
cg07148038	89	ENSG00000204420	-0.00161	0.00389	6.78E-01	0.00155	0.00679	8.20E-01	0.00168	0.00929	8.57E-01	-0.01006	0.00394	1.06E-02	0.00281	0.00291	3.34E-01
cg07148038	89	ENSG00000204388	-0.00155	0.00374	6.79E-01	0.00171	0.00390	6.61E-01	-0.00185	0.00543	7.34E-01	-0.00946	0.00248	1.35E-04	0.00355	0.00192	6.49E-02
cg22127773	80	ENSG00000141499	0.00019	0.00062	7.63E-01	0.00001	0.00105	9.92E-01	0.00440	0.00230	5.54E-02	-0.00020	0.00036	5.73E-01	0.00066	0.00362	8.54E-01
cg22127773	80	ENSG00000269928	0.00017	0.00057	7.68E-01	0.00112	0.00087	1.98E-01	0.00146	0.00197	4.58E-01	-0.00064	0.00059	2.85E-01	0.00157	0.00365	6.68E-01
cg07148038	89	ENSG00000213654	-0.00035	0.00088	6.90E-01	0.00697	0.00651	2.84E-01	-0.00176	0.00108	1.04E-01	0.00005	0.00043	9.10E-01	0.00719	0.00721	3.18E-01
cg07148038	89	ENSG00000227939	-0.00064	0.00163	6.94E-01	-0.00133	0.00196	4.97E-01	-0.00101	0.01075	9.25E-01	0.00089	0.00377	8.13E-01	0.00131	0.00507	7.96E-01
cg07148038	89	ENSG00000204536	-0.00048	0.00124	6.97E-01	-0.00102	0.00645	8.74E-01	-0.00077	0.00118	5.11E-01	0.00088	0.00064	1.67E-01	-0.00966	0.00471	4.02E-02
cg07148038	89	ENSG00000240053	-0.00087	0.00227	7.01E-01	-0.00452	0.00186	1.53E-02	0.00980	0.00610	1.08E-01	0.00048	0.00200	8.10E-01	-0.00252	0.00423	5.52E-01
cg22127773	80	ENSG00000161955	0.00020	0.00076	7.95E-01	-0.00115	0.00157	4.63E-01	-0.00023	0.00247	9.26E-01	0.00077	0.00094	4.13E-01	-0.00075	0.00543	8.91E-01
cg07148038	89	ENSG00000204371	-0.00007	0.00018	7.19E-01	-0.00008	0.00026	7.49E-01	0.00005	0.00037	9.01E-01	-0.00001	0.00025	9.65E-01	-0.00288	0.00155	6.30E-02
cg07148038	89	ENSG00000221988	-0.00073	0.00225	7.47E-01	-0.00144	0.00411	7.26E-01	0.00779	0.00790	3.24E-01	-0.00165	0.00302	5.85E-01	-0.00020	0.00873	9.82E-01
cg22127773	80	ENSG00000179094	0.00009	0.00045	8.41E-01	0.00004	0.00070	9.51E-01	0.00029	0.00236	9.01E-01	0.00007	0.00063	9.07E-01	0.00092	0.00282	7.43E-01
cg07148038	89	ENSG00000204475	-0.00102	0.00336	7.62E-01	0.00922	0.00535	8.51E-02	-0.00602	0.00388	1.21E-01	0.00165	0.00148	2.65E-01	-0.00979	0.00544	7.21E-02
cg07148038	89	ENSG00000204356	-0.00045	0.00172	7.94E-01	0.00377	0.00448	3.99E-01	-0.01338	0.00669	4.56E-02	-0.00044	0.00287	8.79E-01	0.00000	0.00060	9.98E-01
cg22127773	80	ENSG00000183011	0.00003	0.00019	8.90E-01	0.00006	0.00019	7.61E-01	0.00062	0.00165	7.07E-01	-0.00125	0.00102	2.22E-01	0.00197	0.00419	6.37E-01
cg22127773	80	ENSG00000179859	-0.00002	0.00017	9.05E-01	0.00050	0.00045	2.63E-01	-0.00053	0.00203	7.94E-01	-0.00012	0.00019	5.36E-01	0.00222	0.00290	4.44E-01
cg07148038	89	ENSG00000204516	0.00013	0.00054	8.17E-01	0.00031	0.00188	8.68E-01	-0.00101	0.00144	4.82E-01	0.00026	0.00062	6.76E-01	0.00353	0.00480	4.62E-01
cg22127773	80	ENSG00000129194	-0.00009	0.00080	9.09E-01	-0.00070	0.00117	5.49E-01	-0.00025	0.00207	9.06E-01	-0.00013	0.00071	8.53E-01	0.00993	0.00519	5.57E-02
cg22127773	80	ENSG00000141510	-0.00002	0.00022	9.12E-01	0.00046	0.00026	7.59E-02	-0.00051	0.00050	3.05E-01	-0.00018	0.00013	1.47E-01	-0.00012	0.00077	8.72E-01
cg22127773	80	ENSG00000215041	-0.00003	0.00035	9.27E-01	-0.00078	0.00136	5.63E-01	0.00106	0.00263	6.87E-01	0.00005	0.00037	8.91E-01	-0.00482	0.00363	1.85E-01
cg22127773	80	ENSG00000239697	-0.00003	0.00029	9.28E-01	0.00012	0.00032	7.15E-01	0.00001	0.00099	9.94E-01	-0.00163	0.00113	1.47E-01	-0.00058	0.00172	7.37E-01
cg22127773	80	ENSG00000132522	-0.00004	0.00053	9.36E-01	0.00088	0.00102	3.92E-01	0.00123	0.00198	5.34E-01	-0.00043	0.00066	5.15E-01	-0.00306	0.00312	3.26E-01
cg07148038	89	ENSG00000255552	-0.00041	0.00206	8.42E-01	-0.00280	0.00664	6.73E-01	-0.00905	0.00619	1.44E-01	0.00116	0.00249	6.42E-01	0.00061	0.00609	9.20E-01
cg22127773	80	ENSG00000169992	-0.00004	0.00058	9.51E-01	0.00013	0.00110	9.04E-01	0.00076	0.00183	6.76E-01	-0.00033	0.00075	6.63E-01	0.00143	0.00327	6.62E-01
cg22127773	80	ENSG00000178921	0.00004	0.00077	9.57E-01	0.00107	0.00161	5.06E-01	0.00127	0.00212	5.51E-01	-0.00044	0.00100	6.63E-01	-0.00285	0.00390	4.64E-01
cg22127773	80	ENSG00000170049	0.00003	0.00059	9.57E-01	-0.00206	0.00222	3.52E-01	0.00076	0.00218	7.27E-01	-0.00003	0.00066	9.68E-01	0.00389	0.00308	2.06E-01
cg07148038	89	ENSG00000204389	-0.00097	0.00557	8.61E-01	0.00093	0.00466	8.42E-01	0.00122	0.00829	8.83E-01	0.00888	0.00409	2.98E-02	-0.01579	0.00552	4.20E-03
cg22127773	80	ENSG00000133026	0.00001	0.00032	9.64E-01	0.00000	0.00038	1.00E+00	0.00100	0.00077	1.94E-01	-0.00056	0.00045	2.19E-01	0.00084	0.00115	4.64E-01
cg22127773	80	ENSG00000174327	0.00005	0.00117	9.68E-01	-0.00001	0.00038	9.76E-01	0.00856	0.00402	3.31E-02	-0.00126	0.00128	3.26E-01	-0.00449	0.00625	4.72E-01
cg07148038	89	ENSG00000240065	-0.00038	0.00233	8.72E-01	0.00124	0.00124	3.19E-01	0.00139	0.00758	8.55E-01	0.00371	0.00359	3.01E-01	-0.00574	0.00231	1.30E-02
cg07148038	89	ENSG00000232629	0.00034	0.00222	8.80E-01	-0.00209	0.00633	7.41E-01	0.00829	0.00589	1.59E-01	-0.00237	0.00321	4.61E-01	0.00214	0.00439	6.27E-01
cg22127773	80	ENSG00000004975	0.00000	0.00012	9.88E-01	-0.00008	0.00024	7.43E-01	-0.00026	0.00049	6.04E-01	0.00007	0.00016	6.60E-01	-0.00026	0.00071	7.11E-01
cg07148038	89	ENSG00000244731	0.00052	0.00380	8.91E-01	0.00389	0.00148	8.57E-03	0.01781	0.00894	4.63E-02	-0.00474	0.00351	1.77E-01	-0.00675	0.00438	1.23E-01

cg07148038	89	ENSG00000229391	-0.00043	0.00319	8.92E-01	0.01528	0.00670	2.26E-02	-0.00373	0.00861	6.65E-01	-0.00394	0.00310	2.04E-01	-0.00266	0.00200	1.83E-01
cg07148038	89	ENSG00000204344	-0.00046	0.00367	8.99E-01	0.00278	0.00582	6.33E-01	-0.00383	0.00966	6.92E-01	-0.00656	0.00315	3.73E-02	0.00626	0.00456	1.70E-01
cg07148038	89	ENSG00000204351	-0.00008	0.00065	9.04E-01	0.00028	0.00051	5.79E-01	-0.00013	0.00068	8.44E-01	-0.00074	0.00043	8.28E-02	0.02380	0.00867	6.05E-03
cg07148038	89	ENSG00000224389	-0.00018	0.00150	9.05E-01	0.00048	0.00171	7.76E-01	-0.00516	0.00694	4.58E-01	0.00024	0.00400	9.52E-01	-0.00835	0.00737	2.57E-01
cg07148038	89	ENSG00000204424	0.00028	0.00279	9.20E-01	-0.00041	0.00345	9.06E-01	-0.00648	0.01327	6.26E-01	0.00225	0.00643	7.26E-01	0.00360	0.00828	6.64E-01
cg07148038	89	ENSG00000241106	-0.00013	0.00161	9.36E-01	0.00140	0.00414	7.36E-01	-0.01161	0.00637	6.83E-02	-0.00073	0.00209	7.28E-01	0.00136	0.00157	3.88E-01
cg07148038	89	ENSG00000206344	0.00010	0.00149	9.47E-01	0.00200	0.00564	7.23E-01	-0.01091	0.00743	1.42E-01	0.00404	0.00393	3.03E-01	-0.00017	0.00107	8.73E-01
cg07148038	89	ENSG00000204410	0.00003	0.00066	9.63E-01	-0.00158	0.00228	4.88E-01	0.00208	0.00337	5.36E-01	0.00011	0.00070	8.81E-01	-0.00291	0.01155	8.01E-01
cg07148038	89	ENSG00000204531	-0.00008	0.00221	9.72E-01	-0.00089	0.00361	8.04E-01	-0.00389	0.00827	6.38E-01	0.00023	0.00338	9.46E-01	0.00341	0.00615	5.80E-01
cg07148038	89	ENSG00000204396	-0.00023	0.00742	9.76E-01	-0.01784	0.00940	5.77E-02	0.02695	0.01285	3.59E-02	-0.00902	0.00547	9.93E-02	0.00611	0.00509	2.30E-01
cg07148038	89	ENSG00000204304	0.00002	0.00092	9.80E-01	0.00161	0.00163	3.23E-01	-0.00318	0.00236	1.78E-01	-0.00034	0.00152	8.25E-01	0.00074	0.00233	7.52E-01
cg07148038	89	ENSG00000166278	0.00000	0.00137	9.98E-01	-0.00417	0.00328	2.03E-01	-0.00019	0.00345	9.57E-01	0.00039	0.00201	8.47E-01	0.00288	0.00305	3.45E-01

N genes within 2MB indicates number of genes that are tested within 1MB on both sites of the CpG-sites

CpG-sites are ordered based on the Bonferroni-adjusted p-value for the number of genes within 1MB around the CpG-sites

LL: LifeLines Deep

LLS: Leiden Longevity Study

NTR: Netherlans Twin Register

RS: Rotterdam Study
