# Supplementary material for: DNA methylation is associated with lung function in never smokers
Source: Respir Res. 2019 Dec 2;20:268. doi: 10.1186/s12931-019-1222-8 (PMC6889726; doi:10.1186/s12931-019-1222-8)
Supplement: Supplementary file 4 — Additional file 4: Table S4. Results of the association between 36 top CpG-sites identified from the meta-analysis and A: environmental exposures and B: air pollution measurements. [file 12931_2019_1222_MOESM4_ESM.pdf]

Online supplement table 4A: Results of the association between 36 top CpG-sites identified from the meta-analysis and environmental exposures.

	Gases and fumes		Mineral dust		Biological dust		Pesticides		Solvents		Environmental tobacco smoke		In utero tobacco smoke	
cg10012512	-0.0026	(0.0021)	-0.0009	(0.0020)	-0.0019	(0.0021)	-0.0009	(0.0031)	0.0010	(0.0035)	-0.0034	(0.0016)	-0.0003	(0.0018)
cg02885771	-0.0043	(0.0030)	-0.0042	(0.0028)	-0.0010	(0.0029)	0.0037	(0.0044)	-0.0026	(0.0052)	0.0040	(0.0022)	0.0034	(0.0026)
cg25105536	0.0001	(0.0006)	0.0008	(0.0006)	-0.0012	(0.0006)	0.0008	(0.0009)	0.0017	(0.0010)	-0.0004	(0.0004)	0.0002	(0.0005)
cg20102034	0.0003	(0.0018)	-0.0010	(0.0017)	-0.0008	(0.0017)	0.0023	(0.0026)	0.0047	(0.0030)	-0.0006	(0.0013)	$5.85E^{-6}$	(0.0016)
cg03703840	0.0008	(0.0007)	-0.0002	(0.0006)	0.0002	(0.0006)	-0.0010	(0.0010)	-0.0006	(0.0011)	0.0010	(0.0005)	0.0004	(0.0006)
cg21614201	-0.0004	(0.0019)	-0.0002	(0.0018)	-0.0026	(0.0018)	-0.0011	(0.0028)	-0.0029	(0.0032)	-0.0022	(0.0014)	-0.0017	(0.0016)
cg07957088	0.0023	(0.0017)	0.0044	(0.0015)	0.0009	(0.0016)	0.0003	(0.0024)	0.0005	(0.0028)	0.0009	(0.0012)	0.0013	(0.0015)
cg05304461	0.0003	(0.0008)	0.0007	(0.0007)	0.0004	(0.0007)	$-4.20E^{-5}$	(0.0011)	0.0020	(0.0013)	$-7.11E^{-5}$	(0.0005)	$7.58E^{-5}$	(0.0006)
cg11749902	0.0003	(0.0034)	0.0013	(0.0031)	0.0010	(0.0032)	-0.0007	(0.0048)	-0.0035	(0.0056)	-0.0025	(0.0024)	0.0025	(0.0028)
cg02207312	-0.0008	(0.0005)	-0.0003	(0.0005)	0.0003	(0.0005)	-0.0002	(0.0007)	-0.0008	(0.0008)	$8.95E^{-5}$	(0.0004)	0.0002	(0.0004)
cg19734370	-0.0034	(0.0069)	0.0091	(0.0066)	0.0100	(0.0066)	0.0185	(0.0100)	-0.0096	(0.0116)	0.0058	(0.0050)	-0.0061	(0.0059)
cg03077331	-0.0101	(0.0054)	-0.0032	(0.0052)	-0.0063	(0.0051)	-0.0027	(0.0079)	0.0020	(0.0090)	0.0018	(0.0039)	-0.0039	(0.0046)
cg18387671	0.0002	(0.0004)	-0.0004	(0.0004)	-0.0002	(0.0004)	-0.0010	(0.0006)	-0.0005	(0.0007)	$9.76E^{-5}$	(0.0003)	-0.0006	(0.0003)
cg03224276	-0.0012	(0.0014)	0.0011	(0.0013)	-0.0013	(0.0013)	0.0020	(0.0020)	-0.0017	(0.0023)	0.0005	(0.0010)	-0.0010	(0.0012)
cg02137691	-0.0020	(0.0020)	-0.0032	(0.0019)	-0.0012	(0.0019)	-0.0035	(0.0029)	-0.0024	(0.0034)	0.0026	(0.0015)	-0.0025	(0.0018)
cg25884324	0.0011	(0.0014)	0.0014	(0.0013)	0.0010	(0.0013)	-0.0009	(0.0020)	0.0016	(0.0024)	-0.0007	(0.0010)	-0.0026	(0.0012)
cg27158523	0.0008	(0.0011)	-0.0003	(0.0010)	0.0009	(0.0010)	-0.0014	(0.0016)	-0.0002	(0.0018)	0.0002	(0.0008)	-0.0008	(0.0009)
cg01157143	-0.0002	(0.0017)	0.0016	(0.0017)	-0.0013	(0.0017)	0.0027	(0.0025)	$3.78E^{-5}$	(0.0029)	-0.0003	(0.0013)	-0.0002	(0.0015)
cg07160694	0.0005	(0.0005)	-0.0002	(0.0005)	$4.81E^{-6}$	(0.0005)	-0.0008	(0.0007)	0.0008	(0.0009)	-0.0001	(0.0004)	$5.54E^{-5}$	(0.0004)
cg22127773	0.0021	(0.0014)	0.0014	(0.0013)	0.0012	(0.0013)	0.0009	(0.0020)	-0.0004	(0.0024)	-0.0017	(0.0010)	0.0013	(0.0012)
cg20939319	-0.0008	(0.0031)	-0.0012	(0.0030)	-0.0027	(0.0030)	0.0023	(0.0045)	0.0004	(0.0053)	-0.0043	(0.0023)	0.0011	(0.0027)
cg02206852	-0.0001	(0.0016)	0.0010	(0.0015)	0.0012	(0.0015)	-0.0003	(0.0023)	-0.0009	(0.0027)	-0.0028	(0.0012)	-0.0007	(0.0014)
cg17075019	-0.0016	(0.0021)	0.0010	(0.0020)	0.0023	(0.0020)	$-1.03E^{-5}$	(0.0031)	0.0007	(0.0035)	0.0016	(0.0015)	0.0021	(0.0018)
cg25556432	-0.0030	(0.0029)	-0.0033	(0.0027)	-0.0034	(0.0028)	0.0007	(0.0042)	-0.0034	(0.0049)	0.0024	(0.0021)	-0.0009	(0.0025)
cg22742965	-0.0011	(0.0024)	-0.0024	(0.0023)	$-4.99E^{-5}$	(0.0023)	-0.0027	(0.0035)	-0.0021	(0.0041)	-0.0006	(0.0018)	-0.0010	(0.0021)
cg16734845	-0.0004	(0.0012)	-0.0005	(0.0012)	-0.0008	(0.0012)	-6.4E-5	(0.0018)	0.0002	(0.0020)	-0.0015	(0.0009)	0.0017	(0.0011)
cg09108394	0.0034	(0.0031)	0.0058	(0.0030)	0.0012	(0.0030)	0.0033	(0.0046)	$6.72E^{-5}$	(0.0052)	-0.0032	(0.0023)	0.0024	(0.0027)
cg10034572	0.0039	(0.0020)	0.0010	(0.0019)	-0.0006	(0.0019)	-0.0021	(0.0029)	0.0030	(0.0033)	0.0001	(0.0014)	$5.43E^{-5}$	(0.0017)
cg20066227	0.0001	(0.0015)	0.0003	(0.0015)	$-2.07E^{-6}$	(0.0014)	0.0017	(0.0022)	0.0028	(0.0025)	**0.0036**	**(0.0011)**	0.0020	(0.0013)
cg07148038	-0.0011	(0.0017)	-0.0014	(0.0016)	0.0023	(0.0016)	0.0033	(0.0024)	-0.0010	(0.0029)	$5.52E^{-7}$	(0.0012)	-0.0006	(0.0015)
cg23396786	-0.0017	(0.0036)	0.0009	(0.0034)	0.0006	(0.0033)	0.0062	(0.0051)	0.0054	(0.0060)	0.0016	(0.0025)	-0.0013	(0.0030)
cg06218079	-0.0062	(0.0094)	0.0005	(0.0089)	-0.0133	(0.0089)	0.0067	(0.0136)	0.0155	(0.0159)	0.0047	(0.0068)	0.0005	(0.0079)
cg06982745	-0.0022	(0.0015)	-0.0010	(0.0014)	0.0018	(0.0014)	$7.97E^{-5}$	(0.0022)	-0.0038	(0.0025)	-0.0013	(0.0011)	0.0016	(0.0013)
cg05946118	0.0020	(0.0040)	0.0027	(0.0038)	-0.0040	(0.0038)	-0.0042	(0.0058)	-0.0020	(0.0066)	-0.0038	(0.0028)	0.0009	(0.0034)
cg08065963	0.0031	(0.0047)	0.0023	(0.0045)	-0.0029	(0.0045)	-0.0094	(0.0068)	-0.0030	(0.0079)	-0.0057	(0.0034)	0.0057	(0.0040)
cg12064372	0.0014	(0.0009)	-0.0002	(0.0009)	$3.99E^{-5}$	(0.0008)	-0.0003	(0.0013)	0.0016	(0.0015)	0.0013	(0.0007)	0.0002	(0.0008)

Association between DNA methylation and environmental exposures was tested with a robust linear regression model with exposure as categorical variable indicating no exposure versus high exposure. Model was additionally adjusted for age, gender, technical variation and blood cell composition. For all exposures, beta is presented with standard error between brackets. Significant associations (p-value < 0.05/36 = 0.0014) are indicated in bold. Environmental exposures were estimated as described by de Jong et al (Occup Environ Med 2014; 71: 88-96).

Online supplement table 4B: Results of the association between 36 top CpG-sites identified from the meta-analysis and air pollution measurements.

	**NO_2**		**$PM_{2.5}$**		**PM_{10}**		**$PM_{2.5}$abs**	
cg10012512	-0.0001	(0.0003)	0.0030	(0.0032)	0.0016	(0.0017)	0.0050	(0.0085)
cg02885771	0.0002	(0.0004)	-0.0027	(0.0045)	-0.0004	(0.0024)	-0.0075	(0.0121)
cg25105536	$-3.16E^{-5}$	(0.0001)	0.0011	(0.0009)	0.0005	(0.0005)	0.0022	(0.0025)
cg20102034	0.0005	(0.0002)	-0.0032	(0.0027)	-0.0005	(0.0014)	0.0008	(0.0071)
cg03703840	$-8.76E^{-6}$	(0.0001)	$1.77E^{-5}$	(0.0009)	-0.0002	(0.0005)	-0.0019	(0.0025)
cg21614201	-0.0003	(0.0003)	0.0007	(0.0030)	-0.0002	(0.0015)	-0.0005	(0.0078)
cg07957088	$3.76E^{-5}$	(0.0002)	0.0002	(0.0025)	-0.0001	(0.0013)	0.0014	(0.0067)
cg05304461	-0.0002	(0.0001)	$8.89E^{-5}$	(0.0011)	-0.0003	(0.0006)	-0.0028	(0.0029)
cg11749902	-0.0010	(0.0004)	0.0064	(0.0050)	0.0002	(0.0026)	-0.0059	(0.0131)
cg02207312	$-6.26E^{-5}$	(0.0001)	-0.0010	(0.0007)	-0.0004	(0.0004)	-0.0028	(0.0020)
cg19734370	0.0002	(0.0009)	0.0064	(0.0102)	0.0032	(0.0053)	0.0178	(0.0270)
cg03077331	0.0005	(0.0007)	-0.0127	(0.0080)	-0.0026	(0.0042)	-0.0105	(0.0212)
cg18387671	-0.0001	(0.0001)	0.0007	(0.0006)	-0.0002	(0.0003)	-0.0011	(0.0016)
cg03224276	$-6.43E^{-5}$	(0.0002)	-0.0031	(0.0019)	-0.0013	(0.0010)	-0.0067	(0.0052)
cg02137691	0.0001	(0.0003)	-0.0044	(0.0029)	-0.0025	(0.0015)	-0.0134	(0.0076)
cg25884324	0.0003	(0.0002)	-0.0019	(0.0022)	0.0008	(0.0012)	0.0016	(0.0059)
cg27158523	$-5.94E^{-5}$	(0.0001)	0.0007	(0.0015)	-0.0003	(0.0008)	-0.0006	(0.0041)
cg01157143	0.0003	(0.0002)	0.0012	(0.0027)	0.0007	(0.0014)	0.0071	(0.0070)
cg07160694	$4.89E^{-5}$	(0.0001)	0.0012	(0.0008)	0.0002	(0.0004)	0.0008	(0.0021)
cg22127773	-0.0001	(0.0002)	0.0021	(0.0021)	0.0011	(0.0011)	0.0034	(0.0056)
cg20939319	-0.0005	(0.0004)	0.0067	(0.0048)	0.0029	(0.0025)	0.0083	(0.0127)
cg02206852	$-4.09E^{-5}$	(0.0002)	0.0012	(0.0024)	$9.57E^{-5}$	(0.0012)	-0.0001	(0.0063)
cg17075019	0.0005	(0.0003)	-0.0007	(0.0031)	0.0003	(0.0016)	0.0031	(0.0081)
cg25556432	0.0004	(0.0004)	-0.0071	(0.0042)	-0.0015	(0.0022)	-0.0077	(0.0111)
cg22742965	**-0.0011**	**(0.0003)**	0.0014	(0.0037)	-0.0013	(0.0019)	-0.0128	(0.0097)
cg16734845	$-9.29E^{-5}$	(0.0002)	0.0019	(0.0017)	0.0005	(0.0009)	0.0012	(0.0046)
cg09108394	-0.0008	(0.0004)	0.0089	(0.0048)	0.0016	(0.0025)	-0.0008	(0.0130)
cg10034572	-0.0003	(0.0003)	0.0006	(0.0029)	0.0003	(0.0015)	$-9.99E^{-6}$	(0.0077)
cg20066227	$6.39E^{-5}$	(0.0002)	-0.0004	(0.0022)	-0.0003	(0.0011)	-0.0006	(0.0058)
cg07148038	$4.91E^{-5}$	(0.0002)	-0.0012	(0.0025)	0.0002	(0.0013)	0.0030	(0.0066)
cg23396786	0.0004	(0.0005)	-0.0107	(0.0051)	-0.0023	(0.0027)	-0.0080	(0.0137)
cg06218079	0.0013	(0.0013)	-0.0249	(0.0144)	-0.0060	(0.0075)	-0.0241	(0.0382)
cg06982745	$-2.29E^{-5}$	(0.0002)	0.0010	(0.0021)	-0.0002	(0.0011)	-0.0008	(0.0056)
cg05946118	-0.0012	(0.0005)	0.0060	(0.0059)	-0.0013	(0.0031)	-0.0130	(0.0159)
cg08065963	-0.0007	(0.0006)	0.0065	(0.0072)	0.0004	(0.0037)	-0.0077	(0.0191)
cg12064372	0.0001	(0.0001)	-0.0014	(0.0014)	$5.76E^{-5}$	(0.0007)	0.0012	(0.0037)

Association between DNA methylation and air pollution measurements was tested with a robust linear regression model with exposure to the air pollution measurements as continues variable. Model was additionally adjusted for age, gender, technical variation and blood cell composition. For all exposures, beta is presented with standard error between brackets. Significant associations (p-value < 0.05/36 = 0.0014) are indicated in bold. Air pollution was estimated as described by de F C Lichtenfels et al (Environ Health Perspect 2018; 126: 027004). NO_2: Nitrogen Dioxode; $PM_{2.5}$ and PM_{10}: particles with an aerodynamic diameter less than 2.5 and 10 µm; $PM_{2.5abs}$: indicator of elemental carbon content.
