# Supplementary figures and images for: DNA methylation is associated with lung function in never smokers
Source: Respir Res. 2019 Dec 2;20:268. doi: 10.1186/s12931-019-1222-8 (PMC6889726; doi:10.1186/s12931-019-1222-8)

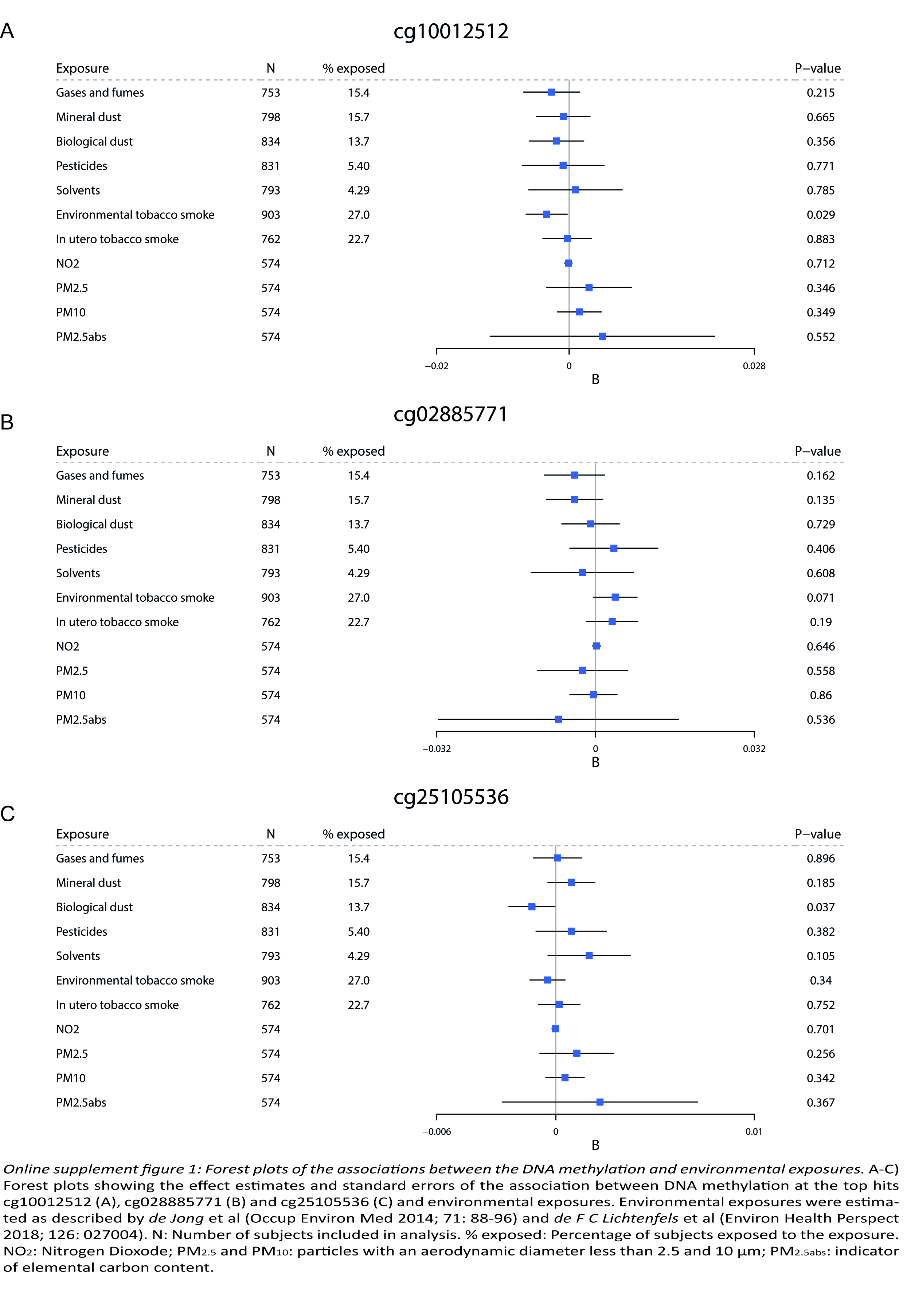

Supplement: Supplementary file 5 — Additional file 5: Figure S1: Forest plots of the associations between DNA methylation and environmental exposures. [file 12931_2019_1222_MOESM5_ESM.tif]
